# Genome-wide association analyses of ovarian cancer patients undergoing primary debulking surgery identify candidate genes for residual disease

**DOI:** 10.1038/s41525-024-00395-y

**Published:** 2024-03-05

**Authors:** Dhanya Ramachandran, Jonathan P. Tyrer, Stefan Kommoss, Anna DeFazio, Marjorie J. Riggan, AOCS Group, David Bowtell, Sian Fereday, Nadia Traficante, Jillian Hung, Penelope M. Webb, Peter A. Fasching, Diether Lambrechts, María J. García, Cristina Rodríguez-Antona, Marc T. Goodman, Francesmary Modugno, Kirsten B. Moysich, Beth Y. Karlan, Jenny Lester, Susanne K. Kjaer, Allan Jensen, Estrid Høgdall, Ellen L. Goode, William A. Cliby, Amanika Kumar, Chen Wang, Julie M. Cunningham, Stacey J. Winham, Alvaro N. Monteiro, Joellen M. Schildkraut, Daniel W. Cramer, Kathryn L. Terry, Linda Titus, Line Bjorge, Liv Cecilie Vestrheim Thomsen, OPAL Study Group, Michael Friedlander, Andreas Obermair, Peter Grant, Vanessa Beesley, Penelope Blomfield, Alison Brand, Alison Davis, Yee Leung, James Nicklin, Michael Quinn, Karen Livingstone, Helen O’Neill, Merran Williams, Tanja Pejovic, Claus K. Høgdall, Iain A. McNeish, Taymaa May, David G. Huntsman, Jacobus Pfisterer, Ulrich Canzler, Tjoung-Won Park-Simon, Willibald Schröder, Antje Belau, Lars Hanker, Philipp Harter, Jalid Sehouli, Rainer Kimmig, Nikolaus de Gregorio, Barbara Schmalfeldt, Klaus Baumann, Felix Hilpert, Alexander Burges, Boris Winterhoff, Peter Schürmann, Lisa-Marie Speith, Peter Hillemanns, Andrew Berchuck, Sharon E. Johnatty, Susan J. Ramus, Georgia Chenevix-Trench, Paul D. P. Pharoah, Thilo Dörk, Florian Heitz

**Affiliations:** 1 Gynaecology Research Unit, Hannover Medical School, Hannover, Germany; 2 Centre for Cancer Genetic Epidemiology, Department of Oncology, University of Cambridge, Cambridge, UK; 3 Department of Women’s Health, Tuebingen University Hospital, Tuebingen, Germany; 4 Centre for Cancer Research, The Westmead Institute for Medical Research, University of Sydney, Sydney, NSW Australia; 5 Discipline of Obstetrics and Gynaecology, The University of Sydney, Sydney, NSW Australia; 6 Department of Gynaecological Oncology, Westmead Hospital, Sydney, NSW Australia; 7 The Daffodil Centre, The University of Sydney, a joint venture with Cancer Council NSW, Sydney, NSW Australia; 8 Department of Obstetrics and Gynecology, Division of Gynecologic Oncology, Duke University Medical Center, Durham, NC USA; 9 Peter MacCallum Cancer Centre, Melbourne, VIC Australia; 10 QIMR Berghofer Medical Research Institute, Brisbane, QLD Australia; 11 Population Health Program, QIMR Berghofer Medical Research Institute, Brisbane, QLD Australia; 12 Department of Gynecology and Obstetrics, Comprehensive Cancer Center Erlangen-EMN, Friedrich-Alexander University Erlangen-Nuremberg, University Hospital Erlangen, Erlangen, Germany; 13 Laboratory for Translational Genetics, Department of Human Genetics, KU Leuven, Leuven, Belgium; 14 VIB Center for Cancer Biology, VIB, Leuven, Belgium; 15 Biochemistry and Molecular Biology area, Department of Basic Health Sciences, Faculty of Health Sciences, Rey Juan Carlos University, Madrid, Spain; 16 Hereditary Endocrine Cancer Group, Spanish National Cancer Research Center (CNIO), Madrid, Spain; 17 Centre for Biomedical Network Research on Rare Diseases (CIBERER), Instituto de Salud Carlos III, Madrid, Spain; 18 Cancer Prevention and Control Program, Cedars-Sinai Cancer, Cedars-Sinai Medical Center, Los Angeles, CA USA; 19 Department of Epidemiology, University of Pittsburgh School of Public Health, Pittsburgh, PA USA; 20 Division of Gynecologic Oncology, Department of Obstetrics, Gynecology and Reproductive Sciences, University of Pittsburgh School of Medicine, Pittsburgh, PA USA; 21 Women’s Cancer Research Center, Magee-Womens Research Institute and Hillman Cancer Center, Pittsburgh, PA USA; 22 Department of Cancer Prevention and Control, Roswell Park Cancer Institute, Buffalo, NY USA; 23 David Geffen School of Medicine, Department of Obstetrics and Gynecology, University of California at Los Angeles, Los Angeles, CA USA; 24 Department of Virus, Lifestyle and Genes, Danish Cancer Institute, Copenhagen, Denmark; 25 Department of Gynaecology, Rigshospitalet, University of Copenhagen, Copenhagen, Denmark; 26 Department of Pathology, Herlev Hospital, University of Copenhagen, Copenhagen, Denmark; 27 Department of Quantitative Health Sciences, Division of Epidemiology, Mayo Clinic, Rochester, MN USA; 28 Department of Obstetrics and Gynecology, Division of Gynecologic Oncology, Mayo Clinic, Rochester, MN USA; 29 Department of Quantitative Health Sciences, Division of Computational Biology, Mayo Clinic, Rochester, MN USA; 30 Department of Laboratory Medicine and Pathology, Mayo Clinic, Rochester, MN USA; 31 Department of Cancer Epidemiology, Moffitt Cancer Center, Tampa, FL USA; 32 Department of Epidemiology, Rollins School of Public Health, Emory University, Atlanta, GA USA; 33 Obstetrics and Gynecology Epidemiology Center, Department of Obstetrics and Gyneclogy, Brigham and Women’s Hospital and Harvard Medical School, Boston, MA USA; 34 Department of Epidemiology, Harvard T.H. Chan School of Public Health, Boston, MA USA; 35 Norris Cotton Cancer Center, Lebanon, NH USA; 36 Department of Obstetrics and Gynecology, Haukeland University Hospital, Bergen, Norway; 37 Centre for Cancer Biomarkers CCBIO, Department of Clinical Science, University of Bergen, Bergen, Norway; 38 School of Clinical Medicine, UNSW Medicine and Health, University of NSW Sydney, Sydney, NSW Australia; 39 Department of ObGyn, Providence Medical Center, Medford, OR USA; 40 Knight Cancer Institute, Oregon Health & Science University, Portland, OR USA; 41 Division of Cancer and Ovarian Cancer Action Research Centre, Department Surgery & Cancer, Imperial College London, London, UK; 42 Institute of Cancer Sciences, University of Glasgow, Glasgow, UK; 43 Division of Gynecologic Oncology, University Health Network, Princess Margaret Hospital, Toronto, ON Canada; 44 Department of Obstetrics and Gynecology, University of British Columbia, Vancouver, BC Canada; 45 Department of Molecular Oncology, BC Cancer Research Centre, Vancouver, BC Canada; 46 Gynecologic Oncology Center, Kiel, Germany; 47 University Hospital Carl Gustav Carus, Technische Universität Dresden, Dresden, Germany; 48 National Center for Tumor Diseases (NCT), Partner Site Dresden, Dresden, Germany; 49 Klinikum Bremen-Mitte, Bremen, Germany; 50 Gynaekologicum Bremen, Bremen, Germany; 51 University Hospital Greifswald, Greifswald, Germany; 52 Frauenarztpraxis Belau, Greifswald, Germany; 53 University Hospital Frankfurt, Frankfurt, Germany; 54 University Hospital Schleswig-Holstein, Campus Lübeck, Lübeck, Germany; 55 Department of Gynecology and Gynecologic Oncology, Evangelische Kliniken Essen-Mitte (KEM), Essen, Germany; 56 Charité - Universitätsmedizin Berlin, Campus Virchow Klinikum, Berlin, Germany; 57 University Hospital Essen, University of Duisburg-Essen, Essen, Germany; 58 University Hospital Ulm, Ulm, Germany; 59 SLK-Kliniken Heilbronn, Klinikum am Gesundbrunnen, Heilbronn, Germany; 60 University Medical Center Hamburg-Eppendorf, Hamburg, Germany; 61 University Hospital Gießen and Marburg, Site Marburg, Marburg, Germany; 62 Klinikum Ludwigshafen, Ludwigshafen, Germany; 63 University Hospital Schleswig-Holstein, Campus Kiel, Kiel, Germany; 64 Krankenhaus Jerusalem, Mammazentrum Hamburg, Hamburg, Germany; 65 University Hospital LMU Munich, Munich, Germany; 66 Department of Obstetrics, Gynecology and Women’s Health, Division of Gynecologic Oncology, University of Minnesota, Minneapolis, MN USA; 67 Cancer Division, QIMR Berghofer Medical Research Institute, Brisbane, QLD Australia; 68 Adult Cancer Program, Lowy Cancer Research Centre, University of NSW Sydney, Sydney, NSW Australia; 69 Department of Computational Biomedicine, Cedars-Sinai Medical Center, West Hollywood, CA USA; 70 Centre for Cancer Genetic Epidemiology, Department of Public Health and Primary Care, University of Cambridge, Cambridge, UK; 71 Department of Gynecology and Gynecological Oncology, HSK, Dr. Horst-Schmidt Klinik, Wiesbaden, Wiesbaden, Germany

**Keywords:** Ovarian cancer, Predictive markers

## Abstract

Survival from ovarian cancer depends on the resection status after primary surgery. We performed genome-wide association analyses for resection status of 7705 ovarian cancer patients, including 4954 with high-grade serous carcinoma (HGSOC), to identify variants associated with residual disease. The most significant association with resection status was observed for rs72845444, upstream of *MGMT*, in HGSOC (*p* = 3.9 × 10^−8^). In gene-based analyses, *PPP2R5C* was the most strongly associated gene in HGSOC after stage adjustment. In an independent set of 378 ovarian tumours from the AGO-OVAR 11 study, variants near *MGMT* and *PPP2R5C* correlated with methylation and transcript levels, and *PPP2R5C* mRNA levels predicted progression-free survival in patients with residual disease. *MGMT* encodes a DNA repair enzyme, and *PPP2R5C* encodes the B56γ subunit of the PP2A tumour suppressor. Our results link heritable variation at these two loci with resection status in HGSOC.

## Introduction

Epithelial ovarian cancer (EOC) is a leading cause of cancer death in women^1^. Most patients with EOC cannot be cured as more than 70% of patients are diagnosed with advanced disease (stage III or IV)^2^ and because tumours develop resistance against systemic therapy^3^. Quality of treatment is an independent prognostic parameter in patients with EOC^4^. Maximal-effort cytoreductive surgery represents a major therapeutic cornerstone and improved surgical techniques have resulted in higher rates of total macroscopic tumour debulking^5,6^. Several analyses have shown that residual disease following primary debulking surgery is strongly associated with survival^7^. For example, the overall survival of patients with FIGO IIIC EOC increases from 34 months in patients with incomplete resection, to 81 months in those with complete resection^8^. The incorporation of extended surgical techniques in the upper abdomen such as diaphragmatic peritoneal stripping or splenectomy has been shown to further increase rates of complete tumour resection^9,10^, and consequently of progression-free and overall survival^11,12^.

Despite this progress, there are several reasons why complete cytoreduction cannot be achieved in all patients with EOC. Even in specialised centres, ~30% of patients have macroscopic residual disease after surgery^13^. The main reason for residual disease is disseminated miliary carcinomatosis scattered over the *viscera* and the *meso* of the small bowel^14^. Such residual disease is apparently due to local tumour spread, which might be influenced by biological features, and some have proposed gene expression variations associated with residual disease^15,16^. While the focus on biological factors influencing residual disease in EOC has been on factors originating from the tumour, there are also interactions between ovarian cancer cells and other cell types such as the connective tissue or the mesothelium^17^. In addition, there is evidence from the Ovarian Cancer Association Consortium (OCAC) that women who were using hormone therapy at the time of diagnosis of EOC less frequently have residual disease after surgery^18^. However, whether and how inherited genetic factors influence residual disease is not known. Compared to transcriptomic or proteomic approaches, a genome-wide association study (GWAS) is independent of tissue-specific differences and may be helpful to identify heritable factors for residual disease.

We hypothesised that residual disease in EOC is partially dependent on inherited factors and therefore performed GWAS analyses for risk of residual disease in a large case series of patients with ovarian cancer. The methylation and expression of candidate genes resulting from these analyses were then tested in an independent series of ovarian tumour samples with known debulking status (residual disease (RD) = 0 cm vs > 0 cm) and patient survival.

## Results

### Genome-wide association study for residual disease identifies rs72845444 at the *MGMT* locus and three further candidate genes

**Fig. 1:**
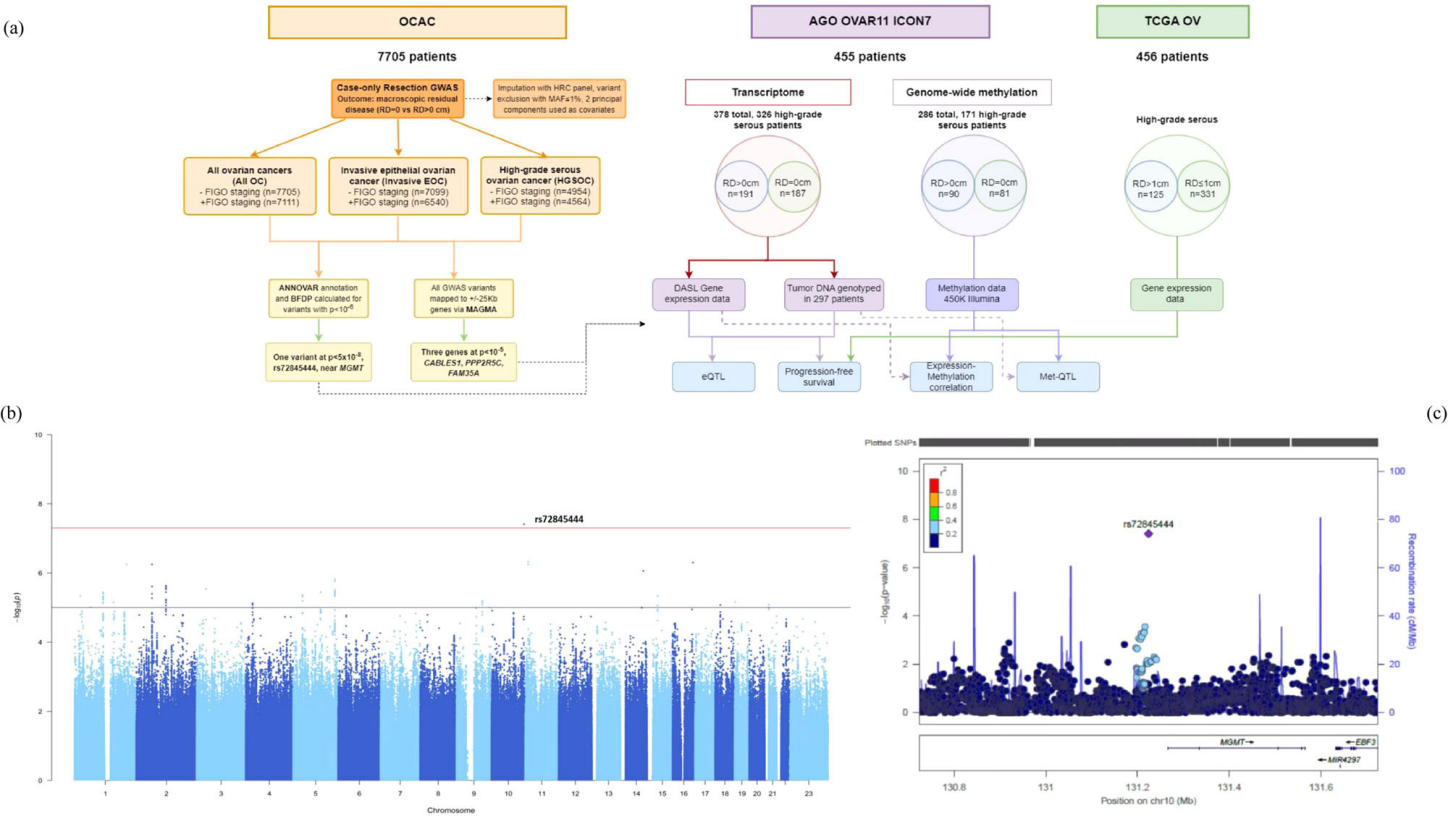
Workflow of the GWAS and follow-up study. **a** Study workflow combining three analyses of OCAC GWAS data for overall, invasive-only and high-grade serous ovarian cancer (left) with AGO-OVAR 11 and TCGA gene expression and clinical datasets. **b** Manhattan plot depicting GWAS results in high-grade serous ovarian cancer (unadjusted for stage) with rs72845444 as the top hit. Blue line: *p* = 1 × 10^−5^, red line: *p* = 5 × 10^−8^. **c** Locus Zoom regional association plot for variant rs72845444, close to *MGMT*.

We applied a two-stage approach to identify potential genetic variants associated with resection status (Fig. 1a). First, we undertook a GWAS of resection status in a large dataset from the Ovarian Cancer Association Consortium using complete resection vs any residual disease. Second, we tested the identified variants for an effect on gene transcript levels and on gene methylation in an independent tumour bank and clinical dataset from the AGO-OVAR 11 study. We also evaluated their correlation with progression-free survival in patients with no macroscopic residual disease (RD = 0) vs patients with residual disease (RD > 0). The validity of these results was additionally tested in the TCGA data set (Fig. 1a).

We extracted the OncoArray and COGS genotyping data from the Ovarian Cancer Association Consortium database^19^ for 7705 patients with information on resection status to perform a GWAS in a case-only design, with macroscopic residual disease (yes/no) as the binary outcome variable. Age-adjusted logistic regression analyses were performed for all OC, invasive EOC, or HGSOCs, with or without adjusting for stage, and the sample numbers for each GWAS analysis are listed in Supplementary Table 1. We identified one variant, rs72845444 (chr10:131224242:A:G; EAF = 0.02; OR = 2.11, 95%CI = 1.61–2.74; p = 3.9 × 10^−8^) that was strongly associated with debulking status in the high-grade serous ovarian cancer group (Fig. 1b, Table 1). This variant is located about 40 kbp upstream of the *MGMT* gene on chromosome 10q26.3 (Fig. 1c). When we repeated the analyses with an adjustment for FIGO stage, this association was only modestly reduced (OR = 2.11, 95% CI = 1.58–2.82, *p* = 4.9 × 10^−7^). Two further variants, rs72859096 and rs12292762 at the *PARVA* locus, were also highly associated (*p* = 6.5 × 10^−8^ and p = 7.4 × 10^−8^, respectively) but this potential association disappeared after adjustment for stage, suggesting that these variants may be linked with tumour stage. No further strong associations were found after adjustment for stage. A list of variants at *p* < 10^−6^ in all GWAS analysis, along with their allele frequencies and additionally calculated Bayesian False Discovery Probability (BFDP) scores are provided in Supplementary Table 2.

**Table 1:** Outcomes from GWAS and MAGMA analyses with and without adjustment for stage

Study Characteristics	Study Name	GIF (lambda)	SNPs above GWS	Genes from MAGMA at *p* < 10^−5^ (25Kb window)
Age-adjusted (invasive and non-invasive)	All OC - FIGO	0.995		
Age-adjusted (invasive)	Invasive EOC - FIGO	1.001		
Age-adjusted (high-grade serous)	HGSOC - FIGO	1.011	rs72845444 (10:131224242:A:G, p = 3.89x10^−8^) near *MGMT*	*CABLES1* (*p* = 2.63 × 10^−6^)
Age and stage adjusted (invasive and non-invasive)	All OC + FIGO	0.981		*FAM35A* (*p* = 6.36 × 10^−6^)
Age and stage-adjusted (invasive)	Invasive EOC + FIGO	0.986		
Age and stage adjusted (high-grade serous)	HGSOC + FIGO	1.004		*PPP2R5C* (*p* = 4.78 × 10^−6^)

Most significant variants and genes for the GWAS and MAGMA analyses in all OC (invasive and non-invasive), invasive OC and high-grade serous-only ovarian cancer (HGSOC) patient groups are noted. The analyses were run just age-adjusted or age and stage adjusted, respectively. Associations at *p* < 5 × 10^−8^ were considered significant, and MAGMA *p* < 10^−5^ was considered noteworthy for follow-up.

**Fig. 2:**
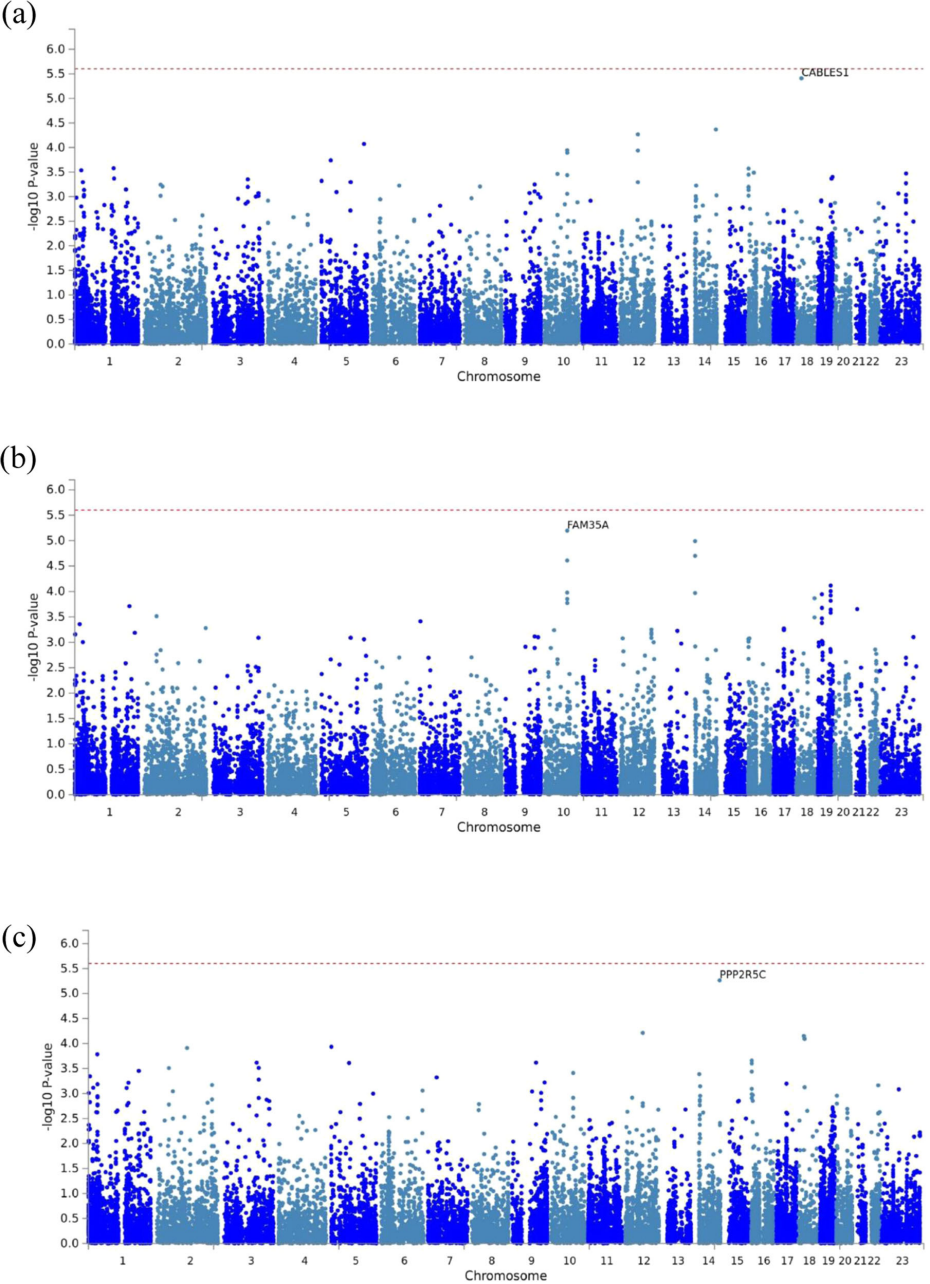
MAGMA gene-based association analyses. Manhattan plots for the MAGMA gene-based association analyses in high-grade serous ovarian cancer without or with adjustment for stage (**a**, **c**) and in overall ovarian cancers after adjustment for stage (**b**). Indicated are the top genes *CABLES1* (**a**), *FAM35A* (**b**), and *PPP2R5C* (**c**), respectively.

We then performed gene-based MAGMA analyses on all the GWAS datasets, to identify cumulative effects of SNPs within and around single genes (up to 25 kbp distance) (Fig. 2a–c, Supplementary Table 3). No gene passed the genome-wide significance threshold of 2.5 × 10^−6^ in these analyses, but three genes were identified at *p* < 10^−5^, with *CABLES1* and *PPP2R5C* in high-grade serous ovarian cancers, without or with adjustment for stage, respectively, and *FAM35A* in all ovarian cancers after adjustment for stage (Table 1). That *PPP2R5C* and *FAM35A* were only associated after adjustment for stage suggested they could represent independent predictors of residual disease. The GWAS summary statistics of the most significant SNPs underlying these MAGMA gene-associations are in Supplementary Table 2.

### Association of *PPP2R5C* risk alleles with mRNA levels in ovarian tumours

We investigated transcript levels in ovarian cancer tissue stratified by debulking status in the genes identified as associated with residual disease. We analysed the log2-fold change in mRNA levels for seven transcripts (four from *PPP2R5C* and one from each of the genes *MGMT, CABLES1*, and *FAM35A*, see Supplementary Table 4 for Illumina Probe IDs) in a series of 378 tumour tissues from the AGO-OVAR 11 study^20,21^. In a comparison between patients undergoing complete resection vs patients with residual disease, the mRNA levels for none of these genes were statistically associated with residual disease, neither in all EOC nor in high-grade serous tumours (Supplementary Table 5). We then investigated whether the GWAS-identified variants may be expression quantitative trait loci (eQTLs) and whether their effect may be dependent on the resection status. We therefore directly genotyped the tumour samples of the AGO-OVAR 11 study for the most strongly associated variants in *CABLES1* (rs77770767, rs28589524, rs6507532, rs4281829), *PPP2R5C* (rs2448233, rs59784377, rs3783362, rs79999043), *FAM35A* (rs11492866) as well as for the *MGMT* variant rs72845444 (Supplementary Table 6). While there was no association between variants near *CABLES1* or *MGMT* with the mRNA levels of their respective genes, rs11492866 showed a borderline association with *FAM35A* levels (*p*_Trend _= 0.04) (Supplementary Fig. 1i). Furthermore, the genotypes for rs2448233, rs3783362, and rs59784377 were associated with *PPP2R5C* mRNA levels in an allelic dose-dependent manner, or when rare allele carriers were combined in a dominant model, respectively (Table 2, further data in Supplementary Fig. 2(viii, xii) and Supplementary Fig. 3ii, iv, xii, xvi). These associations were observed mainly for the major isoform, transcript variant 1, for which the rare alleles were associated with lower *PPP2R5C* mRNA levels. Additional associations were observed with minor isoforms when stratified by resection status (Table 2, Supplementary Fig. 4(vii, xvi) and Supplementary Fig. 5(ii, v, viii, ix, xii).

**Table 2:** Association of *PPP2R5C* variants with mRNA levels of *PPP2R5C* isoforms

Model	Variant ID	ILMN_1780913	ILMN_1789283	ILMN_2364971	ILMN_1795846
		*Isoform 1*	*Isoforms 1,2,3*	*Isoforms 1,2,3*	*Isoform 3*
Overall allelic	rs2448233	0.072	0.200	0.076	0.569
	rs3783362	**0.032**	0.519	0.437	0.150
	rs59784377	**0.003**	0.552	0.529	0.827
	rs79999043	0.890	0.400	0.976	0.113
Overall dominant	rs2448233	**0.041**	0.072	**0.024**	0.728
	rs3783362	0.203	0.858	0.248	0.283
	rs59784377	**0.014**	0.706	0.291	0.564
	rs79999043	0.632	0.187	0.909	**0.037**
No residual disease (RD = 0) dominant	rs2448233	0.448	0.623	0.132	0.810
	rs3783362	0.635	0.304	0.643	**0.025**
	rs59784377	0.352	0.063	0.795	0.435
	rs79999043	0.978	0.471	0.381	0.071
Any residual disease (RD > 0) dominant	rs2448233	0.073	0.057	0.123	0.427
	rs3783362	**0.003**	0.125	0.543	0.899
	rs59784377	**0.007**	0.104	0.053	0.806
	rs79999043	0.544	**0.040**	0.580	**0.038**

Four *PPP2R5C* variant genotypes were tested for association with mRNA levels of *PPP2R5C* isoforms (measured by four illumine probes as indicated) in the AGO-OVAR 11 study under an allelic or dominant model, respectively. Samples were further stratified into complete resection vs residual disease (RD = 0 vs RD > 0). Transcript isoforms indicated represent NM_002719 (1), NM_178586 (2) and NM_178587 (3) in the NCBI Genbank, respectively. Probe ID ILMN_1780913 captured *PPP2R5C* isoform 1, ILMN_1789283 mapped onto isoforms 1, 2, and 3, ILMN_2364971 matched isoforms 1, 2, and 3, and ILMN_1795846 matched isoform 3. Bold values indicate *p* < 0.05.

### Association of risk alleles at *MGMT* and *PPP2R5C* with gene methylation in ovarian tumours

**Fig. 3:**
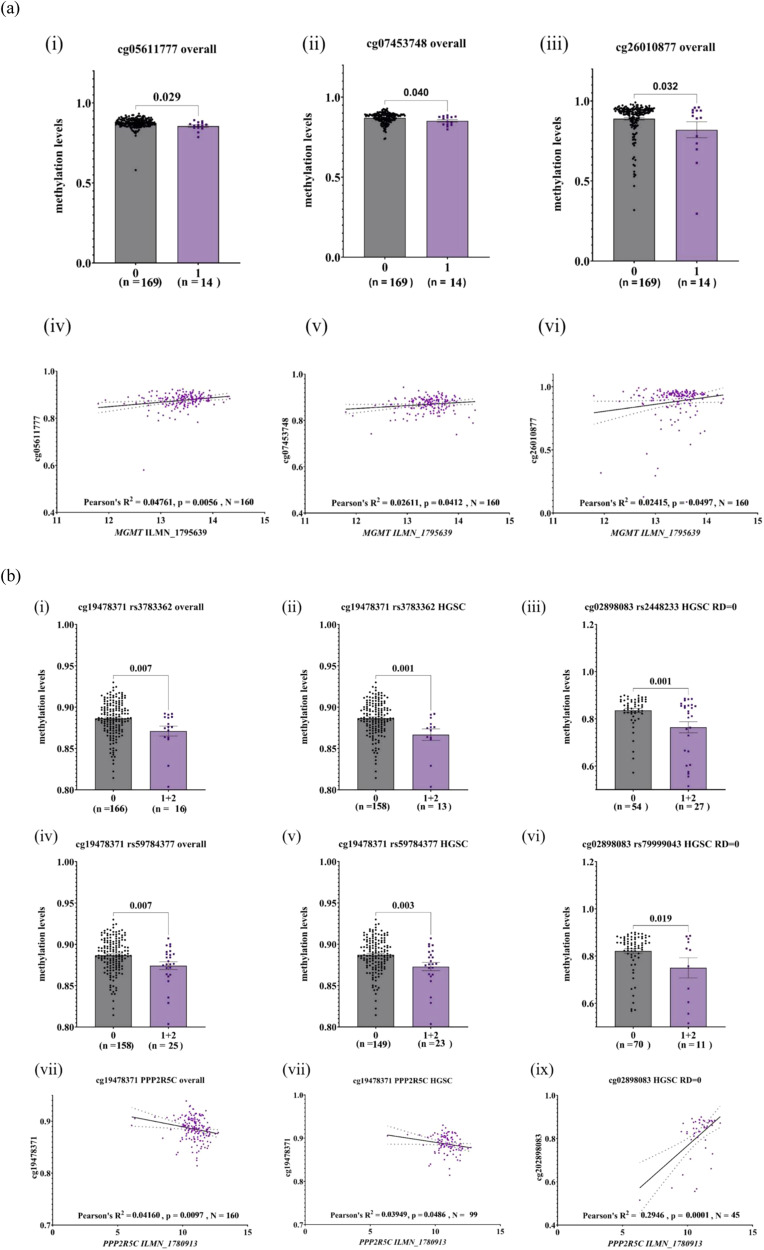
Methylation-QTL and correlation with expression. CpG sites that were nominally significant met-QTLs and also correlated with gene expression at (**a**) *MGMT* and (**b**) *PPP2R5C* in overall, high-grade serous (HGSOC), HGSOC optimal or sub-optimal groups. Plotted are methylation intensity levels (y-axis) vs genotype of the specified SNP (x-axis), or log2 normalised gene expression levels of the corresponding gene transcript (x-axis). The CpG sites are indicated by Illumina cg-Probe IDs, and the Illumina probe per gene is specified by ILMN IDs. *p* values are indicated after unpaired *t* tests between two groups, or following Pearson’s correlation *R*^2^ values and number of samples (*N*).

We then tested for met-QTLs in the vicinity of the genes *MGMT*, *PPP2R5C*, *CABLES1* and *FAM35A* and found multiple potential SNP-methylation and gene transcript-methylation associations (Supplementary Table 7b). For *MGMT*, the risk allele of GWAS variant rs72845444 was associated with hypomethylation at three methylation sites that also correlated with lower *MGMT* mRNA levels (cg05611777 *p* = 0.006; cg07453748 *p* = 0.04; cg26010877 *p* = 0.05; *N* = 160) (Fig. 3a). In HGSOC samples, only cg26010877 correlated with both rs7284544 (*p* = 0.04) and *MGMT* expression (*p* = 0.03; *N* = 99). The detected effects were not due to outliers as their removal generally improved the associations. At the *PPP2R5C* gene, the rare alleles of variants rs3783362 and rs59784377 were associated with hypomethylation of cg19478371 overall and in HGSOC samples (Fig. 3b), and cg19478371 inversely correlated with *PPP2R5C* transcript levels overall (*p* = 0.01; *N* = 160) and weakly in HGSOC (*p* = 0.05; *N* = 99) (Fig. 3b). In HGSOC samples from patients with no residual disease, cg02898083 was associated with rs2448233 and rs7999043 (*p* = 0.001 and *p* = 0.02, respectively (Fig. 3b)), as well as with *PPP2R5C* transcript levels (*p* = 0.0001; *N* = 45) (Supplementary Table 7e and Fig. 3b). Taken together, these analyses supported a functional role for the *MGMT* single variant rs72845444 and suggest a more complex pattern of regulation for *PPP2R5C*.

### Association of *MGMT* and *PPP2R5C* with progression-free survival in patients with residual disease

**Fig. 4:**
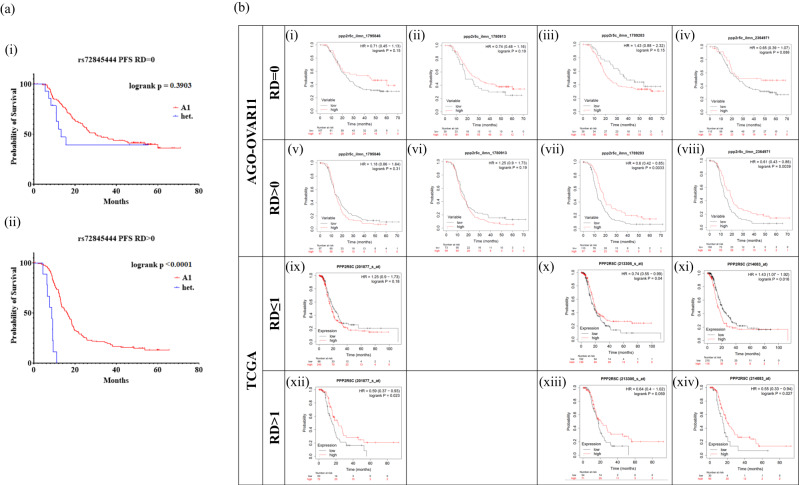
Progression-free survival analysis stratified by debulking status. **a** Kaplan-Meier plots for patients in AGO-OVAR 11 with optimal (RD = 0, top) or suboptimal (RD > 0, bottom) debulking stratified by rs72845444 genotype. **b** Kaplan-Meier plots for high-grade serous patients with optimal (top) or suboptimal (bottom) debulking stratified by *PPP2R5C* mRNA levels in the AGO-OVAR 11 (top panel, RD = 0 vs RD > 0) and the TCGA cohort (bottom panel, RD < = 1 cm vs RD > 1 cm). *PPP2R5C* mRNA levels were measured by four different probes per study as indicated within the figures (Illumina IDs from the AGO-OVAR 11 dataset or specific probe set from the TCGA data accessed via KM-Plotter). For the TCGA dataset, patients were split by auto-selected best cutoff, and high-grade serous patients were chosen, followed by further selection of debulking status. Probe ID ILMN_1780913 captured *PPP2R5C* isoform 1, ILMN_1789283 mapped onto isoforms 1, 2, and 3, ILMN_2364971 matched isoforms 1, 2, and 3, and ILMN_1795846 matched isoform 3. Transcript isoforms indicated represent NM_002719 (1), NM_178586 (2) and NM_178587 (3) in the NCBI Genbank, respectively.

We also determined whether the GWAS-identified variants and candidate gene transcript levels were associated with progression-free survival (PFS) in the 378 patients of the AGO-OVAR 11 trial (number ISRCTN91273375). The rare allele of rs72845444 (*MGMT*) was associated with a worse PFS especially in patients with residual disease (logrank *p* < 0.001), although carrier numbers were small (10/96 with complete resection, 6/115 with residual disease; Fig. 4a(ii)). None of the other single variants were associated with PFS at *p* < 0.05. We then examined the impact of tumour mRNA levels of *CABLES1, FAM35A, MGMT* and *PPP2R5C* on PFS in the AGO-OVAR 11 study, and in the publicly available TCGA datasets. We found no evidence of association for *CABLES1*, *FAM35A or MGMT*. However, *PPP2R5C* mRNA levels positively correlated with PFS in patients with residual disease in the AGO-OVAR 11 dataset with respect to the probes that detect all three major *PPP2R5C* isoforms (HR 0.60, *p* = 0.003, and HR 0.61, *p* = 0.004 (Fig. 4b(vii, viii)), and this was supported by the TCGA data (HR 0.64, *p* = 0.059, and HR 0.55, *p* = 0.027) (Fig. 4b(xiii, xiv)).

## Discussion

Although some evidence has been obtained for modulation of therapeutic response by the genomic background of the patient, our knowledge about the prognostic role of genetic factors remains incomplete^22–24^. We have identified candidate loci associated with residual disease after primary debulking surgery in patients with advanced EOC, providing further insight into the pathophysiology of this devastating condition. Our study indicates that inherited factors are also involved in the complex scenery of residual disease after debulking surgery. All molecular-pathologic studies conducted on residual disease have so far focused on the tumour itself as the object of interest^15,16^, including studies that proposed underlying genetic signatures^25,26^. However, the prediction of resection status after primary debulking surgery in patients with EOC has proven challenging by means of existing gene expression analyses from tumour tissue^20,27,28^. Earlier studies of potential associations between *BRCA1* or *BRCA2* germline mutations and residual disease in advanced EOC revealed conflicting results, as some authors have found significant associations^29^, while others did not^30^. Our present study assumed that genome-wide association analyses could help to pinpoint potential inherited predictors of resection status.

The most significant single variant from our GWAS was rs72845444, located upstream of the *MGMT* gene. *MGMT* encodes O6-methylguanine DNA methyltransferase which repairs the mutagenic DNA lesion O6-methylguanine back to guanine and prevents mismatch and errors during DNA replication and transcription. This role may be consistent with a progressive accumulation of mutations in EOC. In our experiments, we were not able to find a significant association of rs72845444 with *MGMT* mRNA in the AGO-OVAR 11 samples and therefore the direction of effect could not easily be fixed at the transcript level. This could be due to the relatively low minor allele frequency of rs72845444 which limits the power of eQTL analyses. However, we obtained evidence that rs72845444 predicted gene methylation and at least one of the CpG sites near *MGMT* also correlated with *MGMT* mRNA levels. Thus, if rs72845444 exerts its effect through *MGMT*, it may partly occur through an effect on gene methylation to regulate gene expression and, consequently, cellular sensitivity to alkylating agents. Interestingly, promoter methylation of *MGMT* is a known biomarker in predicting the prognosis of patients with *glioblastoma multiforme*^31,32^. Although *MGMT* methylation and gross total resection have been reported as independent prognostic factors^33^, others found *MGMT* methylation to be associated with the extent of resection in this common brain tumour^34^.

We further investigated cumulative effects of variants using a gene-wide approach and identified three candidate genes in different analyses: *CABLES1* and *PPP2R5C* in high-grade serous ovarian cancers without and with adjustment for FIGO stage, respectively, and *FAM35A* in the overall EOC analysis. In our subsequent analysis of the AGO-OVAR 11 tumour samples, the mRNA levels for *PPP2R5C* correlated with resection status, whereas no strong evidence was obtained for *CABLES1* or *FAM35A*. Furthermore, we have shown associations of specific GWAS-derived genetic variants in *PPP2R5C* with the levels of its transcript, and this was partly dependent on the resection status. The genetic variants at *PPP2R5C* exhibited their association independent of each other as they are virtually unlinked (highest r^2^ is 0.24 for rs2448233 and rs59784377). Beyond the prediction of residual disease, we analysed the potential of *PPP2R5C* levels specifically to predict progression-free survival in patients with suboptimal debulking, and this was seen in both the AGO-OVAR 11 and TCGA data sets. Taken together, these results provide convergent evidence for a consistent association between germline variants in *PPP2R5C*, its methylation and mRNA levels, the resection status and progression-free survival.

*PPP2R5C* encodes the serine/threonine-protein phosphatase 2A 56 kDa regulatory subunit gamma isoform, B56γ, that regulates the activity of the PP2A enzyme and can direct it to cancer-specific targets of dephosphorylation, including TP53. The activation of TP53 through PP2A(B56γ) is dependent on DNA damage-induced activation of ATM which then phosphorylates TP53 as well as B56γ^35^. However, *PPP2R5C* was identified after stratification for high-grade serous cancer in our study, and most high-grade serous ovarian tumours harbour a mutation in *TP53*, making this pathway an unlikely explanation. More recently, a complex of PP2A/B56 with BRCA2 has been described to be required for DNA repair by homologous recombination^36^. This connection of PP2A/B56γ with homology-directed repair (HDR) as a positive regulator of BRCA2 function may be important for the results in our study. We found that rare alleles of GWAS variants were associated with lower *PPP2R5C* mRNA levels in patients with high-grade serous tumours and residual disease, suggesting lower levels of PP2A activation. It is conceivable that in such incompletely debulked tumours, impairment of HDR may have contributed to the resection status and to worse survival. PP2A is a druggable tumour suppressor that has been proposed for targeted anticancer therapy, most recently also for ovarian cancer^37–39^. Our data are consistent with recent observations that PP2A genes essential for cellular transformation (B56α, B56γ and PR72) are heterozygously lost in the majority of HGSC and their loss correlates with worse overall patient survival which could be antagonised by stabilisation of PP2A expressed from the remaining allele^39^.

This study used the screening approach of GWAS to identify inherited factors responsible for residual disease in patients undergoing primary debulking surgery. We had a large patient series from the OCAC available to identify genomic variants and genes associated with resection status. A limitation here was that we did not stratify for neoadjuvant chemotherapy due to insufficient data. Furthermore, although *MGMT* and *PPP2R5C* were supported by both genetic association and eQTL/mQTL evidence, the results were below genome-wide significance and therefore will need to be replicated in subsequent studies. We then used a well-described patient cohort from the ICON7 trial (number ISRCTN91273375) to analyse the potential impact of the identified variants on gene methylation, gene expression and progression-free survival. Although this analysis uncovered associations with both methylation and expression, the study size was limited and the role of the identified variants will warrant further investigation. Additionally, we did not have information on copy number variants or specific gene mutations in this patient set, in order to perform an adjusted mQTL analysis. Finally, different definitions of optimal vs suboptimal disease in the AGO-OVAR 11 data vs TCGA data may also have limited the comparability of stratified groups in these data sets. From a clinical point of view, it is important to point out that the results generated here should not be used to minimise surgical resection or to reduce attempts to further increase complete resection rates in each unit. Nevertheless, our study provides evidence that there are biologic reasons for residual disease, despite maximal surgical effort. As we included all stages in the GWAS, some of the genomic variants (such as those in *PARVA*) may act through their effect on stage. However, in the stage-adjusted analyses, the associations with *MGMT* and *PPP2R5C* variants still stood out.

In summary, our GWAS provided strong evidence for candidate genomic loci associated with resection status in patients with EOC undergoing primary debulking surgery and identified a potential role for inherited variants at two genes involved in DNA repair, *MGMT* and *PPP2R5C*, in modulating gene expression, debulking outcome and progression-free survival. Future prospective studies should test genomic markers at these genes as predictive factors for resection status and prognostic factors for survival in patients with epithelial ovarian cancer.

## Methods

### Patients

The studies in the Ovarian Cancer Association Consortium that contributed to the GWAS meta-analyses have been described previously (Supplementary Table 1a)^19^. A total of 7705 female individuals had information on residual disease (RD) after primary surgery and were included in our case-only logistic regression analysis for resection status, comparing macroscopic complete resection vs any RD. Of those, 7111 individuals had information on FIGO stage and could be included in an analysis adjusted for stage. RD was defined as the maximum dimension of disease remaining following primary debulking surgery. The actual size of residual tumour was extracted from surgery reports at each participating site and recorded in centimeters. Samples stratified by country, debulking status, FIGO stage, age, and histotype are shown in Supplementary Table 1a. For the analysis, we defined macroscopic complete resection as no residual disease (RD = 0 cm). Researchers were not blinded to resection status, and randomisation of groups was not necessary for this study. The OCAC study was approved by the Duke University Health System Institutional Review Board (IRB) under two separate protocols, one for the collection of the data (IRB Protocol #: Pro00013555), and a second for the analysis and distribution of the data (IRB Protocol #: Pro00013556). This study was conducted in accordance with the Helsinki Declaration and all participants provided signed consent. All participants were of European descent.

Ovarian tumour tissues were derived from 455 female patients of the AGO-OVAR 11 trial, the German contribution to the ICON7 multicenter phase III trial (Supplementary Table 1b)^40^. The median age at diagnosis for this cohort was 58.5 years (ranging from 19 to 81 years). 425 of the 455 tumour samples had been tested for genome-wide methylation, and transcriptome-wide gene expression data was available for 378 of the 455. Of the latter, 279 tumour DNA samples were available for genotyping in the present study. Patients with gene expression data (*n* = 378) were divided based on RD into 187 patients (49.4%) with complete resection (RD = 0), and 191 (50.6%) patients having had residual disease (RD > 0). 326/378 patients (86.2%) had high-grade serous histology, of whom 154 underwent complete resection and 172 had residual disease (Fig. 1a). PFS was calculated from the date of randomisation to the date of the first indication of disease progression or death, whichever occurred first. Disease progression was defined according to the Response Evaluation Criteria in Solid Tumors (RECIST) guidelines on the basis of radiologic, clinical, or symptomatic indicators of progression^41^.

### GWAS analyses

The dataset from genotyped samples was imputed using the Haplotype Reference Consortium panel. We excluded variants with MAF ≤ 1% and performed age-adjusted logistic regression analyses with residual disease (yes/no) as the binary outcome variable. An initial logistic regression of residual disease by age was significant (log OR = 0.023, SE = 0.019, *z* = 11.93). Therefore, age was included to improve power slightly. Three initial analyses were performed: for all ovarian cancers, all invasive EOC, and limited to high-grade serous ovarian histology.

Being a case-only analysis, we tested how many principal components (PCs) should be included in the GWAS analyses. Our initial logistic regression analysis to test the association between multiple PCs and the outcome variable (residual disease status) showed that only the first two PCs contributed to the outcome (*p* < 0.05), whereas none of the further PCs (3–9) were significantly associated (*p* > 0.05). This suggested that adding any further PCs would not improve the accuracy. Therefore we only included the first two PCs for each panel (Oncoarray and COGS).

Summary statistics were visualised via Manhattan and QQ plots generated using the qqman R package^42^ and the genomic inflation factor (*λ*) was calculated via R 3.6.2. Our study was estimated to have >80% power to detect effect sizes >1.2 for variants with minor allele frequencies larger than 0.14 at a genome-wide significance level *α* = 5 × 10^−8^.

Adding stage information to the logistic regression analysis with age was highly significant (log OR = 0.26, se = 0.08, z-score = 32.8) with the pseudo-R2 going up from 0.0136 (age only) to 0.158 with age and FIGO stage. We therefore performed three further logistic regression analyses with adjustment for FIGO stage.

We also calculated the Bayes False Discovery Probability (BFDP) for all the variants with MAF > 0.01 and *p* < 10^−6^ using the genetic analysis package GAP^43^ in R v3.6.2. Priors of 1:1000, 1:10,000 and 1:100,000 were tested for odds ratios of 1.5 or 2, with similar outcomes (Supplementary Table 2b). A BFDP < 20% was considered strong evidence for an association.

For a gene-wide analysis of cumulative SNP effects, summary statistics were uploaded into FUMA v1.3.61^44^, and MAGMA v1.072^45^ was used to perform gene-based testing. SNPs were mapped within a 25 kb window from the transcription start site (TSS) of genes, and a genome-wide gene-based testing was performed to identify significant genes within each GWAS analysis (i.e. all OC, invasive EOC or just HGSOC, ±FIGO stage). MAGMA genome-wide significance threshold was calculated to be *p* = 2.5 × 10^−6^ after mapping variants to 20,016 protein-coding genes.

### Gene expression and survival analyses

Log2 normalised gene expression data from cDNA-mediated annealing, selection, extension and ligation (DASL) assays was available for 378 patients from the AGO-OVAR 11 trial, as described previously^21^, along with clinical variables. For the top genes from the SNP-based ANNOVAR and MAGMA predictions, gene expression data was converted into Z scores. These data were then stratified based on resection (complete resection (RD = 0, *n* = 187) or residual disease (RD > 0, *n* = 191)) as well as by histology or grade, and t-tests were performed using R 3.6.2. to identify targets with differential expression among the tested cohorts. For in silico annotation of variants in terms of eQTL effects we used SNiPA v.3.4^46^.

For the top genes from the SNP-based ANNOVAR and MAGMA predictions, gene expression data available from the AGO-OVAR 11 trial was used to plot gene-based progression-free survival curves for this patient cohort after stratification based on resection status (complete resection (RD = 0, *n* = 187) or residual disease (RD > 0, *n* = 191)). Gene expression data was converted into Z scores and divided into quartiles, and survival curves were plotted with custom plotting in KM plotter. In parallel, progression-free survival curves were plotted in the TCGA ovarian cancer dataset using KM plotter^47^ using auto-select best cutoff for a total of 456 patients with serous histology and high-grade cancer, with RD ≤ 1 cm (*n* = 331) and RD > 1 cm (*n* = 125) defined as optimal/suboptimal in the available dataset.

### Variant genotyping and eQTL analysis

Tumour DNA was still available for 297 out of 378 patients from the AGO-OVAR 11 trial, from which patients with high-grade serous histology (*n* = 211) and complete resection (*n* = 96) or residual disease (*n* = 115) were selected for variant genotyping via SNPtype assays (Fluidigm). Assays were designed for 10 variants of interest with allele-specific primers (Fluidigm; Supplementary Table 6) and allele-specific PCR products were detected with FAM or HEX-labelled universal probes (Fluidigm). Variant genotype was then tested for association with log2 normalised DASL gene expression data for variant-gene pairs of interest under an allelic model via GraphPad Prism v9.0 using Student’s *t* test to compare two groups or ANOVA between three groups. A linear test for trend was performed after ANOVA as well as after a linear regression analysis to check whether the genotype was associated with the transcript levels under an allelic model. Sequences for the selected Illumina Human HT-12 WG-DASL V4.0 R2 expression bead chip assays are provided in Supplementary Table 4. eQTL analysis was also performed for patients after stratification by debulking. Variant genotypes were further tested as predictors of progression-free survival in survival analysis via GraphPad Prism v9.0.

### Methylation analysis

425 of 455 FFPE tumour samples from the AGO-OVAR 11 study were bisulphite converted and run on the Illumina 450 K Infinium Methylation Beadchip. The Infinium HD FFPE Quality Check assay was performed to remove samples failing (<95% CpG detection) as part of the 450 K ICON7 project. After a rigorous quality check (see Supplementary File 2), methylation data on 286 samples remained. The methylation data, after covariable adjustment, was combined with the available genotype data, as well as the DASL expression data for the corresponding transcripts, and met-QTL and methylation-gene expression correlation analysis (using Pearson’s R) was carried out for 286 patient samples. For methylation-QTL analysis, an association of methylation with SNP genotype was tested using Student’s *t* test for two groups and ANOVA between three groups. From these 286 samples, 172 had high-grade serous histology, from which 1 patient did not undergo surgery, 81 had RD > 0 and 90 had RD = 0. CpG probes ± 25 kbp from the four genes of interest were analysed (189 probes for *MGMT*, 83 probes for *PPP2R5C*, 30 probes for *CABLES1* and 14 probes for *FAM35A*). Tested probe Illumina cg IDs and SNPs per gene are mentioned in Supplementary Table 7a.

### Reporting summary

Further information on research design is available in the Nature Research Reporting Summary linked to this article.

### Supplementary information

Supplementary Information

REPORTING SUMMARY

## Data Availability

Summary statistics from the six GWASs in this study will be available at GWAS Catalogue (accession GCP ID: GCP000727; GCST IDs for each GWAS: All_OC_FIGO GCST90292521, All_OC_no_FIGO GCST90292522, HGSOC_FIGO GCST90292523, HGSOC_no_FIGO GCST90292524, Invasive_EOC_FIGO GCST90292525, Invasive_EOC_no_FIGO GCST90292526). OCAC summary results are available from the combined iCOGS, Oncoarray, GWAS meta-analyses and can be looked up at the OCAC website https://ocac.ccge.medschl.cam.ac.uk/data-projects/results-lookup-by-region/. Individual-level genotyping data generated in this study are not publicly available due to patient privacy requirements but can be applied for through established OCAC procedures. Derived data supporting the findings of this study are available from the corresponding author upon reasonable request.
