# Supplementary material for: Genome-wide association analyses of ovarian cancer patients undergoing primary debulking surgery identify candidate genes for residual disease
Source: NPJ Genom Med. 2024 Mar 5;9:19. doi: 10.1038/s41525-024-00395-y (PMC10915171; doi:10.1038/s41525-024-00395-y)
Supplement: Supplementary file 1 — Supplementary Information [file 41525_2024_395_MOESM1_ESM.pdf]

Supplemental File 1: Members of the OPAL Study Group and the AOCS Management Group

OPAL Study Group Investigators were as follows (https://opalstudy.qimrberghofer.edu.au/page/OPAL_Study_Group/Investigators/):

Professor Penelope Webb, QIMR Berghofer Medical Research Institute, QLD – already named

Professor Anna De Fazio, NSW – already named

Professor Michael Friedlander, Department of Medical Oncology, Prince of Wales Hospital, Sydney, New South Wales, Australia.

Professor Andreas Obermair, Queensland Centre for Gynaecological Cancer Research, University of Queensland, Brisbane, Queensland, Australia.

Professor Peter Grant, Gynecological Oncology Unit, Mercy Hospital for Women, Melbourne, Victoria, Australia.

Dr Vanessa Beesley, QIMR Berghofer Medical Research Institute, QLD

Professor Georgia Chevenix-Trench, QIMR Berghofer Medical Research Institute, QLD – already named

Professor David Bowtell Peter MacCallum Cancer Centre, VIC

Associate Professor Penelope Blomfield, Royal Hobart Hospital, TAS

Associate Professor Alison Brand, Department of Gynaecological Oncology, Westmead Hospital, Westmead, New South Waled and University of Sydney, Sydney, New South Wales, Australia.

Dr Alison Davis, Department of Medical Oncology, The Canberra Hospital and ANU Medical School, Australian National University, Canberra, Australian Capital Territory, Australia

Professor Yee Leung, Division of Obstetrics and Gynaecology, Faculty of Health and Medical Sciences, University of Western Australia, Crawley, Western Australia, Australia.

Associate Professor James Nicklin, Queensland Centre for Gynaecological Cancer, The Wesley Hospital, University of Qld, Brisbane, Queensland, Australia

Professor Michael Quinn, Royal Women's Hospital, Melbourne, VIC

Ms Karen Livingstone, Consumer

Ms Helen O'Neill, Consumer, ANZGOG

Ms Merran Williams, Consumer, QLD

The *AOCS Management Group* of the Australian Ovarian Cancer Study (AOCS, http://www.aocstudy.org/) lists as follows:

Professor David Bowtell, Peter MacCallum Cancer Centre, VIC

Professor Georgia Chevenix-Trench, QIMR Berghofer Medical Research Institute, QLD – already named

Professor Anna De Fazio, NSW – already named

Professor Penelope Webb, QIMR Berghofer Medical Research Institute, QLD – already named

Ms Sian Fereday, Peter MacCallum Cancer, VIC

Ms Nadia Traficante, Peter MacCallum Cancer, VIC

Ms Jillian Hung, QIMR Berghofer Medical Research Institute, QLD

Supplementary File 2

QC Report for the DNA Methylation ICON 7 Data – OVCA – 450K

Information from Illumina: Summary of the Samples Processing

425 samples (424 processed):
- 1 empty tube (#25)
- 104 failed assay (all at 250ng input) with a % CpG sites detected cut off of 95%
  - 11 failed but rehybridized to another chip to improve signal (all but 1 sample on chip failed due to chip failure)
  - 7 failed assay and QC assay
- 340 gave good data with 250ng input
- 84 gave good data by repeating with 1ug input
  - these were chosen from those that failed assay and QC assay
  - (those that had too high a CT score from qCPR QC were not repeated at 1ug input)

Samples were processed by Bisulfite conversion (250ng, or 1ug for repeats).
All material for sample from conversion goes into Infinium FFPE Restoration protocol.
All material for sample from restoration goes into Infinium Methylation protocol.

Samples were analyzed and it was determined that 104 failed the assay (24.5%) with % CpG detected cut off of 95%.
Infinium HD FFPE QC assay (qPCR) was done on the 104 failed samples.
11 samples failed due to chip failure - these were rehybridized to a new chip.

Of the 104 tested for FFPE QC, 101 (97.1%) failed qPCR.
Concentrations were verified using Picogreen assay. If anything, samples were more concentrated that those values provided.

The samples had failed QC qPCR, but had sufficient concentrations for the assay. The samples may have been too degraded.
To see how degraded the samples were, a small selection were run on a gel.
The samples traces were difficult to see. This could have been because they may have been very sheared and the concentration spread over gel lane.

It was decided to repeat the failed samples from the beginning of the Infinium FFPE Restoration protocol.
1ug of sample was used for bisulfite conversion (4x the amount originally used and recommended in the protocol).
Only 95 of the 104 samples were redone to make up a 96 well plate (+1 process control). The 9 samples left out were those that failed QC qPCR with the highest CT scores.

Repeating the samples improved their results.
Of the 104 failed samples: 1st pass data - only 3 samples were above the % detected CpG site cut-off of 95%, giving a failure rate of 25.5% (104/424)
Repeat data - 59 of the 104 are above cut-off, giving a failure rate of 10.6% (45/424)

**Methylation probes**	**N probes**
**Initial set of probes**	**485,577**
**QC exclusions**	
**a.** remove probes at the same location as a marker	36,659
**b.** remove probes with a rsid for probe name	65
**c.** remove probes detected in <70% of the samples	6,020
**Total # probes excluded due to QC**	**42,744**
**Analysis set of probes**	**442,833**

**Sample Exclusion Criteria**	**N Samples**
**Initial set of ovarian samples**	**425**
**QC exclusions**	
a. Empty tube	1
b. samples without covariates available **(not ovarian cancer)**	29
c. Bisulfite conversion green plot	107
d. samples with <92% detected probes	2
**Total # samples excluded due to QC**	**139**
**Analysis set of samples**	**286**

S. Fig. 1

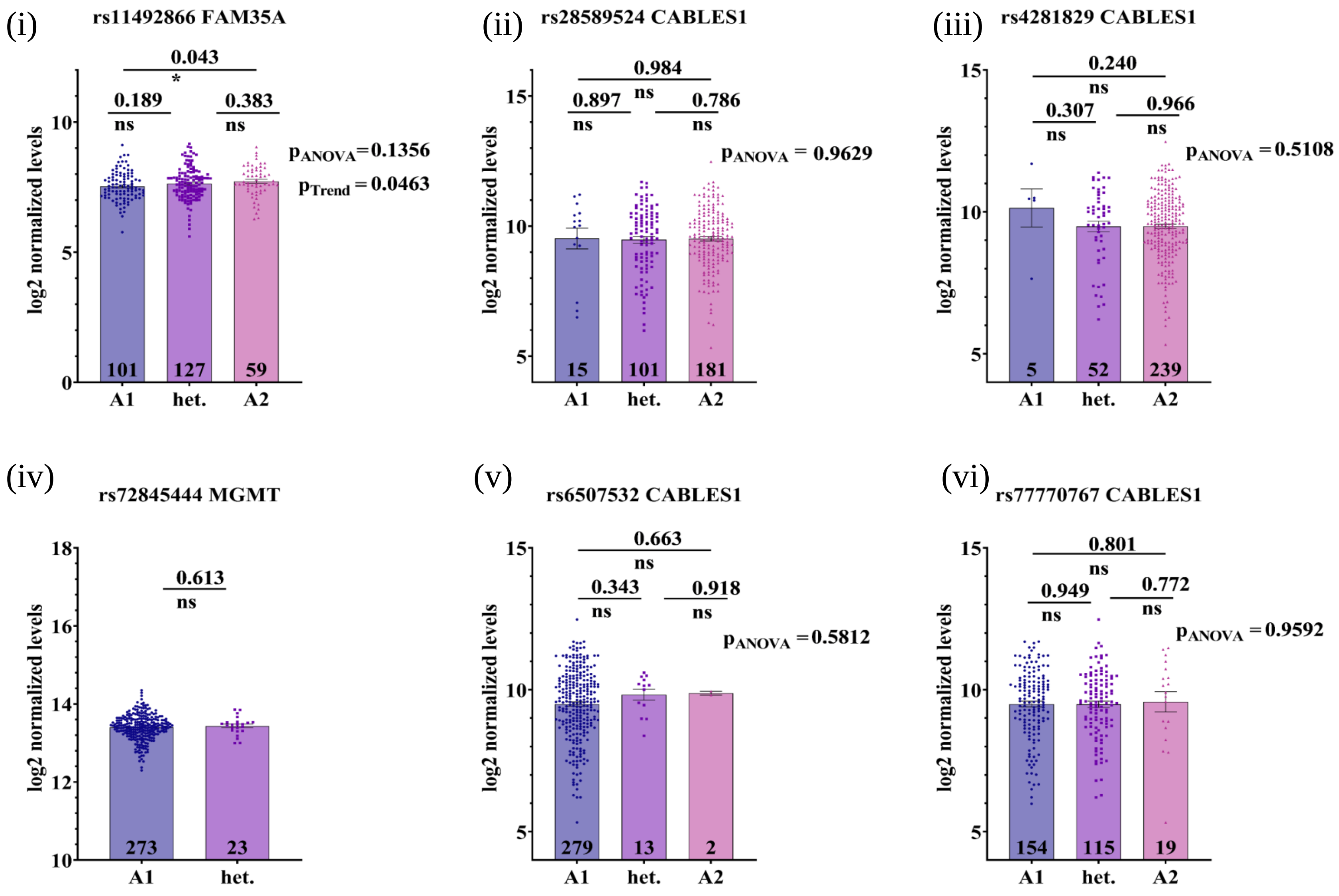

Supplementary Figure 1: eQTL analysis of specific variants for *MGMT*, *FAM35A* and *CABLES1* in ovarian tumours of the AGO-OVAR 11 study. Genotype results were compared under an allelic model with previously assessed log2 normalised gene expression levels using student´s t-tests and one-way ANOVA followed by a linear trend test as indicated.

S. Fig. 2

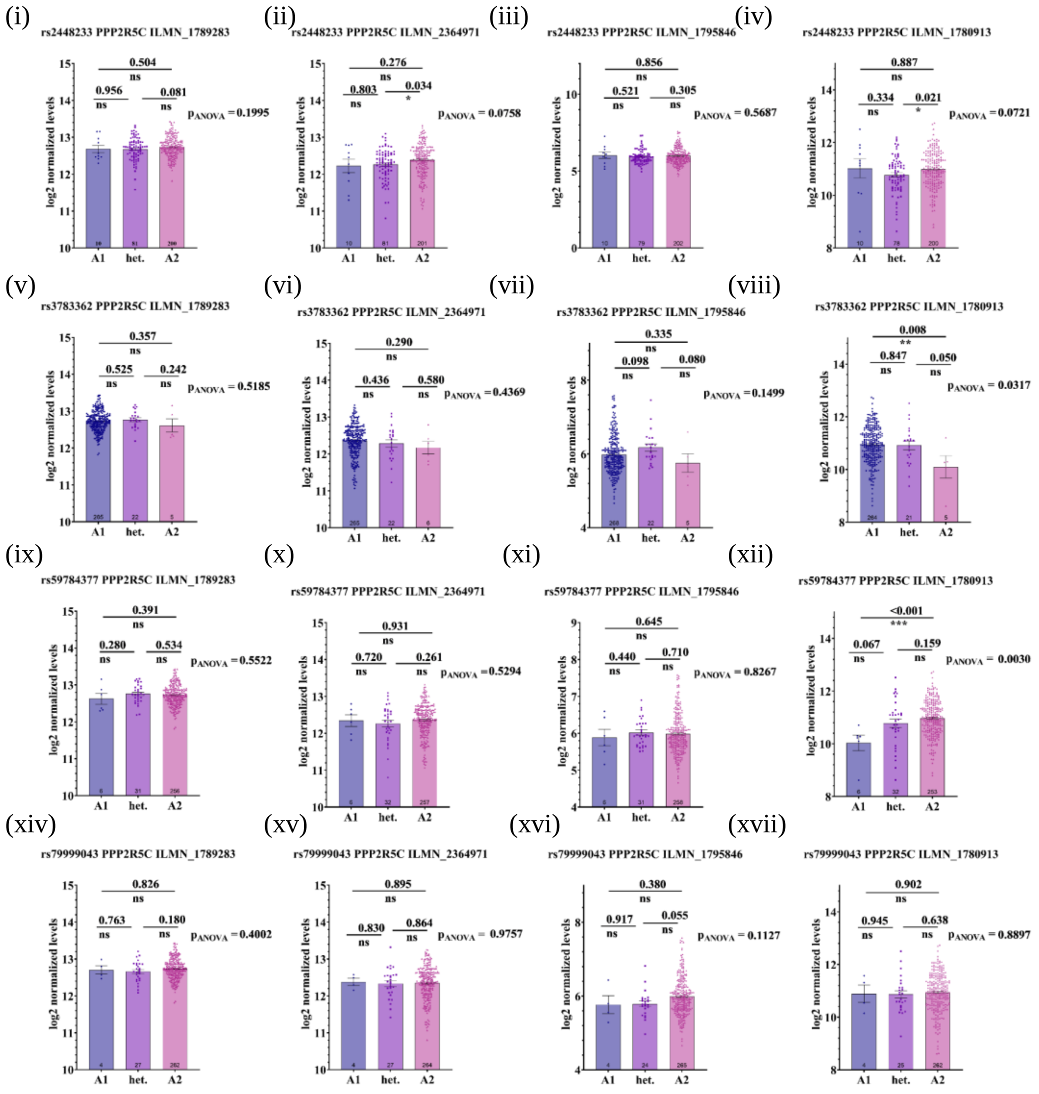

Supplementary Figure 2: eQTL analysis of four specific variants for *PPP2R5C* in ovarian tumours of the AGO-OVAR 11 study. Genotype results were compared under an allelic model with previously assessed log2 normalised gene expression levels for four different probes using student´s t-tests and one-way ANOVA as indicated. Probe ID ILMN_1780913 captured *PPP2R5C* isoform 1, ILMN_1789283 mapped onto isoforms 1, 2, and 3, ILMN_2364971 matched isoforms 1, 2, and 3, and ILMN_1795846 matched isoform 3. Transcript isoforms indicated represent NM_002719 (1), NM_178586 (2) and NM_178587 (3) in the NCBI Genbank, respectively.

S. Fig. 3

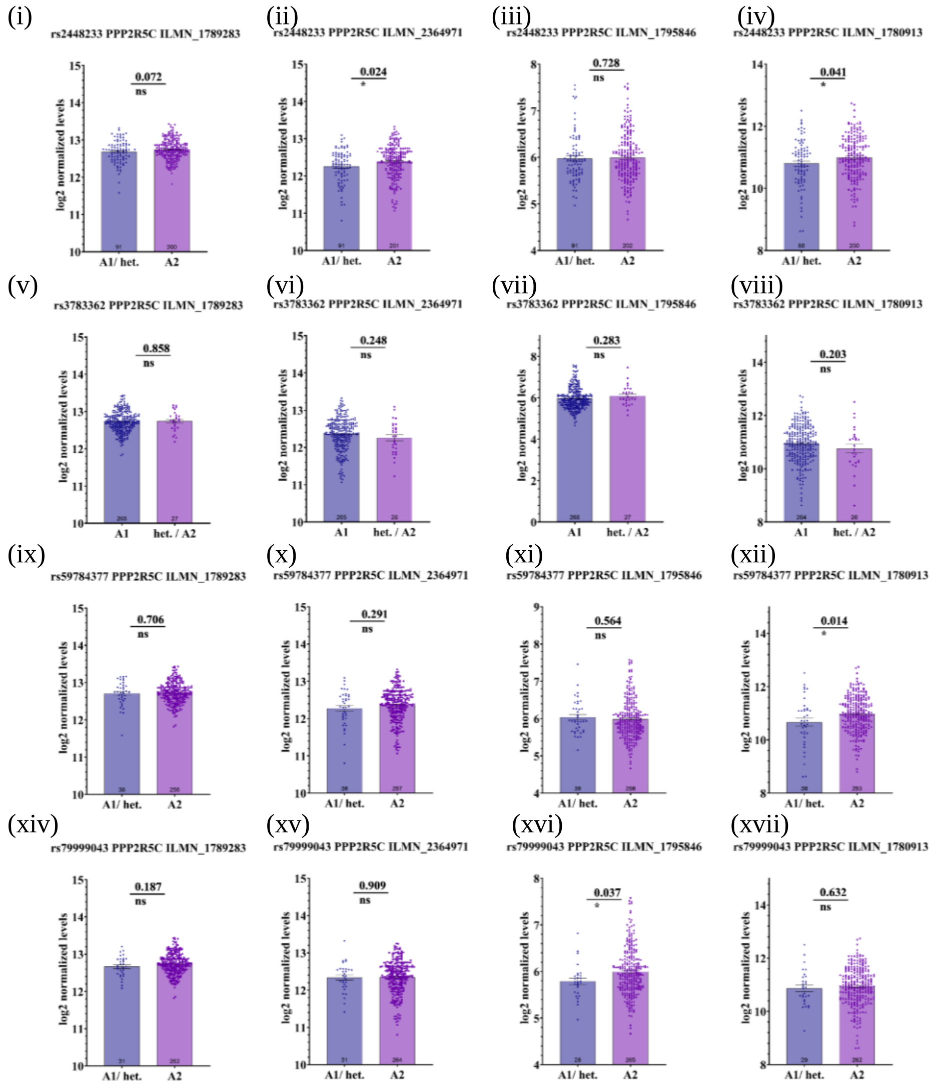

Supplementary Figure 3: eQTL analysis of four specific variants for *PPP2R5C* in ovarian tumours of the AGO-OVAR 11 study. Genotype results were compared under a dominant model with previously assessed log2 normalised gene expression levels for four different probes using student´s t-tests. Probe ID ILMN_1780913 captured *PPP2R5C* isoform 1, ILMN_1789283 mapped onto isoforms 1, 2, and 3, ILMN_2364971 matched isoforms 1, 2, and 3, and ILMN_1795846 matched isoform 3. Transcript isoforms indicated represent NM_002719 (1), NM_178586 (2) and NM_178587 (3) in the NCBI Genbank, respectively.

S. Fig. 4

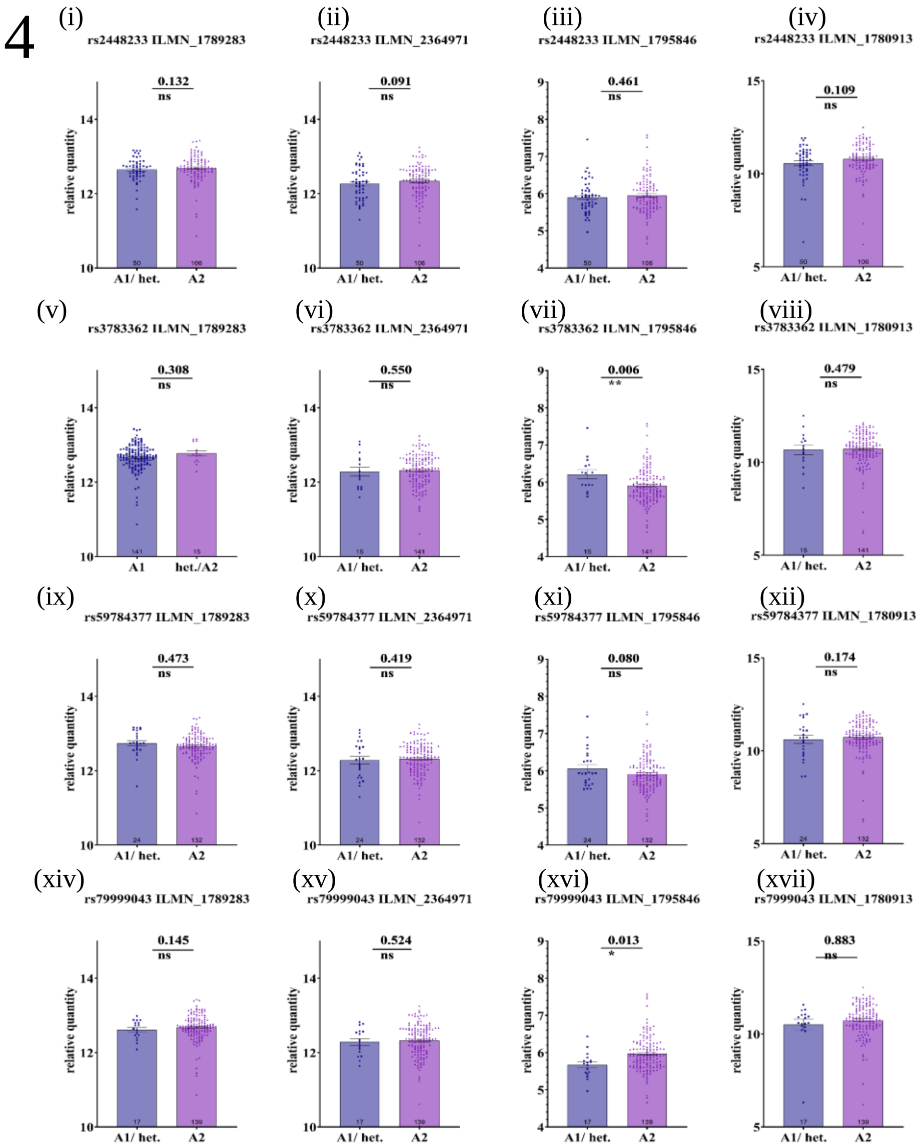

S. Fig. 5

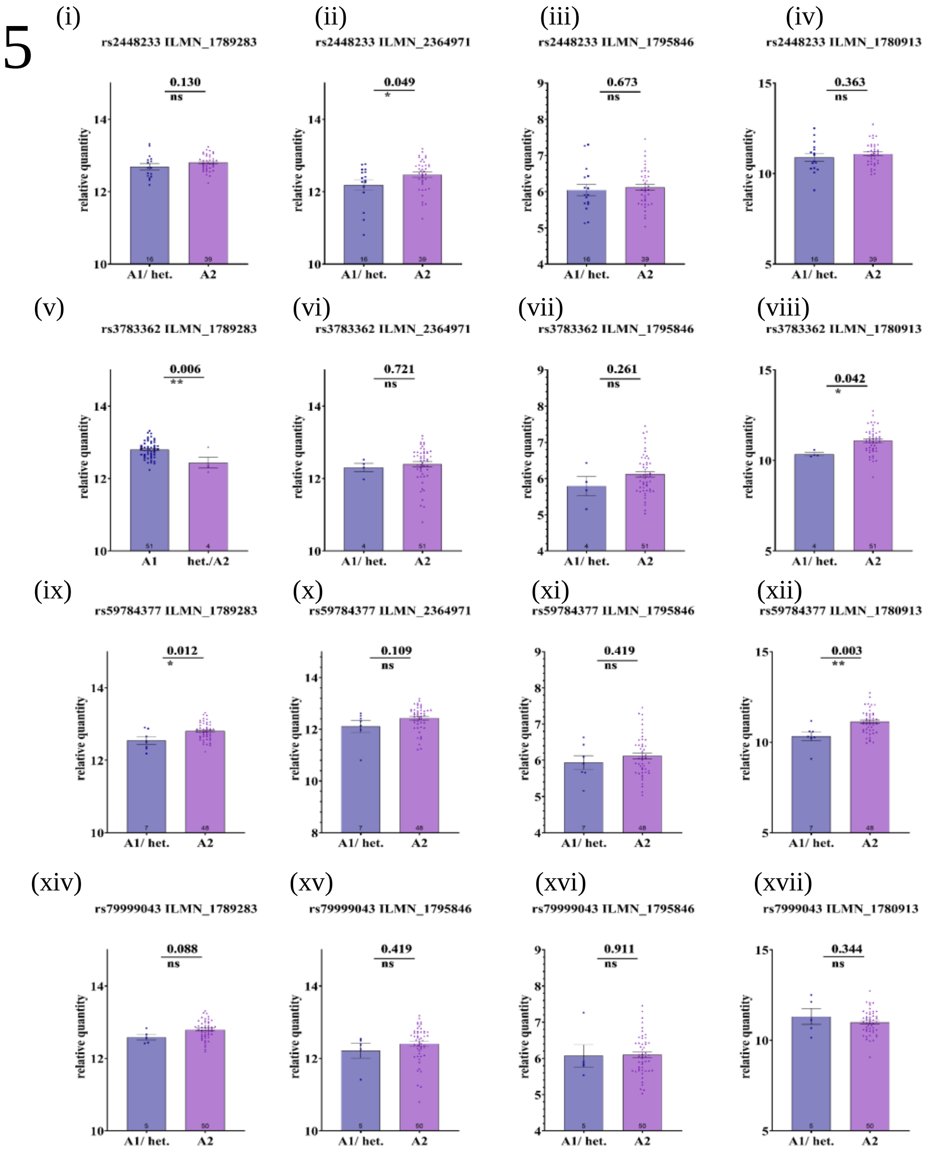

Supplementary Figure 4: eQTL analysis of four specific variants for *PPP2R5C* in ovarian tumours of the AGO-OVAR 11 study with optimal debulking (RD=0). Genotype results were compared under a dominant model with previously assessed log2 normalised gene expression levels for four different probes using student´s t-tests. Probe ID ILMN_1780913 captured *PPP2R5C* isoform 1, ILMN_1789283 mapped onto isoforms 1, 2, and 3, ILMN_2364971 matched isoforms 1, 2, and 3, and ILMN_1795846 matched isoform 3. Transcript isoforms indicated represent NM_002719 (1), NM_178586 (2) and NM_178587 (3) in the NCBI Genbank, respectively.

Supplementary Figure 5: eQTL analysis of four specific variants for *PPP2R5C* in ovarian tumours of the AGO-OVAR 11 study with suboptimal debulking (RD>0). Genotype results were compared under a dominant model with previously assessed log2 normalised gene expression levels for four different probes using student´s t-tests. Probe ID ILMN_1780913 captured *PPP2R5C* isoform 1, ILMN_1789283 mapped onto isoforms 1, 2, and 3, ILMN_2364971 matched isoforms 1, 2, and 3, and ILMN_1795846 matched isoform 3. Transcript isoforms indicated represent NM_002719 (1), NM_178586 (2) and NM_178587 (3) in the NCBI Genbank, respectively.

Supplementary Table 1a: Contributing studies in the OCAC study

Overall numbers

Analysis		Optimal (RD=0)	Sub-optimal (RD>0)	TOTAL N
Age-adjusted (invasive and non-invasive)	All OC - FIGO	3602	4103	7705
Age-adjusted (invasive)	Invasive EOC - FIGO	3195	3904	7099
Age-adjusted (high grade serous)	HGSOC - FIGO	1824	3130	4954
Age and stage adjusted (invasive and non-invasive)	All OC + FIGO	3406	3705	7111
Age and stage adjusted (invasive)	Invasive EOC + FIGO	3010	3530	6540
Age and stage adjusted (high grade serous)	HGSOC + FIGO	1705	2859	4564

Country-specific sample numbers

COHORT	Analysis	Age-adjusted (invasive and non-invasive)			Age-adjusted (invasive)			Age-adjusted (high grade serous)			Age and stage adjusted (invasive and non-invasive)			Age and stage adjusted (invasive)			Age and stage adjusted (high grade serous)		
	Country	Optimal (RD=0)	Sub-optimal (RD>0)	Sum	Optimal (RD=0)	Sub-optimal (RD>0)	Sum	Optimal (RD=0)	Sub-optimal (RD>0)	Sum	Optimal (RD=0)	Sub-optimal (RD>0)	Sum	Optimal (RD=0)	Sub-optimal (RD>0)	Sum	Optimal (RD=0)	Sub-optimal (RD>0)	Sum
Oncoarray	Australia	751	898	1649	734	892	1626	395	742	1137	739	878	1617	725	872	1597	389	727	1116
	Belgium	547	245	792	401	201	602	288	169	457	545	233	778	400	195	595	287	163	450
	Canada	169	159	328	169	159	328	118	141	259	153	136	289	153	136	289	106	121	227
	Denmark	200	377	577	200	377	577	79	267	346	200	377	577	200	377	577	79	267	346
	Germany	70	34	104	64	32	96	26	21	47	70	33	103	64	31	95	26	21	47
	Norway	81	104	185	73	104	177	33	81	114	80	104	184	72	104	176	32	81	113
	UK	141	240	381	141	240	381	86	182	268	0	0	0	0	0	0	0	0	0
	USA	1349	1664	3013	1157	1524	2681	665	1233	1898	1329	1568	2897	1142	1446	2588	654	1186	1840
	**Sum**	**3308**	**3721**	**7029**	**2939**	**3529**	**6468**	**1690**	**2836**	**4526**	**3116**	**3329**	**6445**	**2756**	**3161**	**5917**	**1573**	**2566**	**4139**
COGS	Australia	11	20	31	11	20	31	7	16	23	11	20	31	11	20	31	7	16	23
	Belgium	10	2	12	10	2	12	6	2	8	10	2	12	10	2	12	6	2	8
	Denmark	32	28	60	32	28	60	6	12	18	32	28	60	32	28	60	6	12	18
	Germany	19	12	31	16	9	25	8	9	17	19	12	31	16	9	25	8	9	17
	Norway	21	18	39	18	18	36	9	12	21	21	18	39	18	18	36	9	12	21
	UK	26	107	133	26	107	133	13	81	94	26	107	133	26	107	133	13	81	94
	USA	175	195	370	143	191	334	85	162	247	171	189	360	141	185	326	83	161	244
	**Sum**	**294**	**382**	**676**	**256**	**375**	**631**	**134**	**294**	**428**	**290**	**376**	**666**	**254**	**369**	**623**	**132**	**293**	**425**
**TOTAL**		**3602**	**4103**	**7705**	**3195**	**3904**	**7099**	**1824**	**3130**	**4954**	**3406**	**3705**	**7111**	**3010**	**3530**	**6540**	**1705**	**2859**	**4564**

Sample numbers classified by FIGO stage

COHORT	FIGO stage / Country	IA	IB	IC	I(NOS)	IIA	IIB	IIC	II(NOS)	IIIA	IIIB	IIIC	III(NOS)	IV	Sum
Oncoarray	Australia	139	11	134	10	19	32	83	8	40	66	861	32	182	1617
	Belgium	159	10	90	2	6	24	18	3	13	53	274	7	119	778
	Canada	19	2	32	0	9	9	17	1	11	24	141	3	21	289
	Denmark	57	9	49	0	10	12	29	0	17	52	265	0	77	577
	Germany	18	0	11	1	1	7	7	3	3	12	22	3	15	103
	Norway	19	3	9	0	7	5	5	0	7	8	91	1	29	184
	USA	346	28	260	14	36	59	118	10	73	126	1428	38	361	2897
	**Sum**	**757**	**63**	**585**	**27**	**88**	**148**	**277**	**25**	**164**	**341**	**3082**	**84**	**804**	**6445**
COGS	Australia	1	0	3	0	0	2	0	0	0	1	17	0	7	31
	Belgium	1	0	2	0	0	1	0	0	1	0	6	0	1	12
	Denmark	7	2	9	0	3	0	5	0	4	14	13	0	3	60
	Germany	7	0	6	0	0	2	0	0	0	3	8	1	4	31
	Norway	8	0	2	0	0	2	1	0	1	1	17	0	7	39
	UK	0	0	0	4	0	0	0	7	0	1	0	96	25	133
	USA	33	6	31	8	7	9	7	9	8	17	129	67	29	360
	**Sum**	**57**	**8**	**53**	**12**	**10**	**16**	**13**	**16**	**14**	**37**	**190**	**164**	**76**	**666**
**TOTAL**		**814**	**71**	**638**	**39**	**98**	**164**	**290**	**41**	**178**	**378**	**3272**	**248**	**880**	**7111**

Sample numbers classified by age group

COHORT	Age group / Country	[10,20)	[20,30)	[30,40)	[40,50)	[50,60)	[60,70)	[70,80)	[80,90)	[90,100)	Sum
Oncoarray	Australia	0	13	57	222	517	547	289	4	0	1649
	Belgium	21	53	57	124	217	194	100	25	1	792
	Canada	0	0	9	38	88	103	75	14	1	328
	Denmark	1	0	10	92	152	168	132	22	0	577
	Germany	0	3	10	20	21	28	20	2	0	104
	Norway	0	1	7	25	39	60	35	18	0	185
	UK	0	4	7	66	144	121	39	0	0	381
	USA	0	38	121	457	853	838	550	152	4	3013
	Sum	22	112	278	1044	2031	2059	1240	237	6	7029
COGS	Australia	0	0	0	3	9	11	8	0	0	31
	Belgium	0	1	1	2	4	2	2	0	0	12
	Denmark	0	0	4	9	21	11	15	0	0	60
	Germany	0	0	4	10	4	9	2	2	0	31
	Norway	0	0	0	4	16	7	11	1	0	39
	UK	0	0	3	25	40	50	14	1	0	133
	USA	0	1	21	54	123	102	49	20	0	370
	Sum	0	2	33	107	217	192	101	24	0	676
**TOTAL**		**22**	**114**	**311**	**1151**	**2248**	**2251**	**1341**	**261**	**6**	**7705**

Sample numbers classified by histotype

COHORT	Histotype / Country	High-grade serous	Low-grade serous and borderline	Mucinous	Endometrioid	Clear cell	Other	Sum
**Oncoarray**	Australia	1137	74	71	142	92	133	1649
	Belgium	457	94	88	45	25	83	792
	Canada	259	9	7	25	18	10	328
	Denmark	346	54	48	67	38	24	577
	Germany	47	19	10	15	7	6	104
	Norway	114	12	15	20	8	16	185
	UK	268	14	3	29	36	31	381
	USA	1898	275	170	296	161	213	3013
	Sum	4526	551	412	639	385	516	7029
**COGS**	Australia	23	0	2	3	0	3	31
	Belgium	8	0	1	0	3	0	12
	Denmark	18	30	5	3	4	0	60
	Germany	17	3	3	3	0	5	31
	Norway	21	0	2	5	3	8	39
	UK	94	2	3	14	8	12	133
	USA	247	27	23	30	11	32	370
	Sum	428	62	39	58	29	60	676
**TOTAL**		**4954**	**613**	**451**	**697**	**414**	**576**	**7705**

Supplementary Table 1b: Patient characteristics of the ICON7 study

Classification group	Numbers (%)	FIGO stage				Grade		Debulking Status		
		I	II	III	IV	Low	High	optimal (RD=0)	sub-optimal (RD>0)	no surgery
**Histology**										
Clear cell carcinoma	24 (5%)	11	3	8	2	22	2	14	10	0
Endometrioid	12 (3%)	1	5	5	1	10	2	9	3	0
High grade serous (HGSC)	374 (82%)	18	32	261	63	248	126	182	190	2
Low grade serous (LGSC)	7 (2%)	0	1	6	0	6	1	5	2	0
Metastasis	16 (4%)	1	0	8	7	6	10	5	11	0
Mucinous	6 (1%)	1	0	5	0	5	1	4	2	0
SBOT (Serous borderline ovarian tumors)	15 (3%)	0	2	12	1	12	3	8	7	0
Unknown	1	0	0	0	1	0	1	1	0	0
**SUM**		32	43	305	75	309	146	228	225	2
**TOTAL**	**455**	**455**				**455**		**455**		

Supplementary Table 2

(A)	Contents
(B)	GWAS summary statistics, all variants at p < 10E-6
(C)	MAGMA SNPs summary statistics

GWAS name	SNP	Chromosome	Position	Effect	Baseline	logOR	SE	EAF	OR	OR_L	OR_U	P value	dbSNP	Overlapped Gene	Nearest Upstream Gene	Nearest Downstream Gene	BFDP_0.0001ORhi_2	BFDP_0.00001ORhi_2	BFDP_0.000001ORhi_2	BFDP_0.0001ORhi_1_5	BFDP_0.00001ORhi_1.5	BFDP_0.000001ORhi_1.5
All OC - FIGO	11:12427831_A_G	11	12427831	G	A	-0.23	0.04	0.23	0.79	0.73	0.86	6.51E-08	rs72859096	*PARVA*			0.04	0.32	0.82	0.04	0.29	0.80
All OC - FIGO	11:12426303_T_C	11	12426303	C	T	-0.23	0.04	0.23	0.79	0.73	0.86	7.39E-08	rs12292762	*PARVA*			0.05	0.35	0.84	0.04	0.32	0.82
All OC - FIGO	16:81842873_G_A	16	81842873	A	G	0.19	0.04	0.65	1.21	1.12	1.30	2.77E-07	rs11150413	*PLCG2*			0.17	0.67	0.95	0.14	0.61	0.94
All OC - FIGO	6:156915939_G_A	6	156915939	A	G	-1.05	0.21	0.01	0.35	0.23	0.53	5.82E-07	rs149003716			*ARID1B*	0.66	0.95	0.99	0.97	1.00	1.00
All OC - FIGO	1:115923368_A_G	1	115923368	G	A	0.23	0.05	0.18	1.25	1.15	1.37	8.52E-07	rs35746138		*RP4-663N10.1*	*RP4-663N10.2*	0.34	0.84	0.98	0.31	0.82	0.98
All OC - FIGO	2:12017617_C_T	2	12017617	T	C	0.20	0.04	0.20	1.23	1.13	1.33	8.56E-07	rs75422962	*AC096559.1*			0.36	0.85	0.98	0.31	0.82	0.98
All OC - FIGO	1:115928196_T_C	1	115928196	C	T	0.23	0.05	0.18	1.25	1.15	1.37	8.62E-07	rs11486082		*RP4-663N10.1*	*RP4-663N10.2*	0.34	0.84	0.98	0.31	0.82	0.98
All OC - FIGO	1:115924308_C_G	1	115924308	G	C	0.23	0.05	0.18	1.25	1.14	1.37	9.39E-07	rs34313841		*RP4-663N10.1*	*RP4-663N10.2*	0.36	0.85	0.98	0.33	0.83	0.98
Invasive EOC - FIGO	11:12427831_A_G	11	12427831	G	A	-0.23	0.04	0.23	0.79	0.73	0.87	2.48E-07	rs72859096	*PARVA*			0.14	0.62	0.94	0.12	0.59	0.93
Invasive EOC - FIGO	11:12426303_T_C	11	12426303	C	T	-0.23	0.04	0.23	0.79	0.73	0.87	2.61E-07	rs12292762	*PARVA*			0.15	0.63	0.94	0.13	0.60	0.94
Invasive EOC - FIGO	6:156915939_G_A	6	156915939	A	G	-1.14	0.22	0.01	0.32	0.21	0.50	2.92E-07	rs149003716		*RP11-518I13.1*	*RP11-230C9.1*	0.60	0.94	0.99	0.97	1.00	1.00
Invasive EOC - FIGO	2:12017617_C_T	2	12017617	T	C	0.21	0.04	0.20	1.24	1.14	1.35	7.37E-07	rs75422962	*AC096559.1*			0.32	0.82	0.98	0.28	0.79	0.97
Invasive EOC - FIGO	10:131224242_G_A	10	131224242	A	G	0.52	0.11	0.03	1.68	1.37	2.07	8.41E-07	rs72845444		*RP11-168C9.1*	*MGMT*	0.34	0.84	0.98	0.59	0.94	0.99
Invasive EOC - FIGO	16:81842873_G_A	16	81842873	A	G	0.19	0.04	0.65	1.21	1.12	1.30	9.81E-07	rs11150413	*PLCG2*			0.40	0.87	0.99	0.34	0.84	0.98
HGSOC - FIGO	10:131224242_G_A	10	131224242	A	G	0.74	0.14	0.02	2.11	1.61	2.75	3.89E-08	rs72845444		*RP11-168C9.1*	*MGMT*	0.05	0.35	0.84	0.32	0.82	0.98
HGSOC - FIGO	11:12427831_A_G	11	12427831	G	A	-0.28	0.06	0.23	0.76	0.68	0.84	4.71E-07	rs72859096	*PARVA*			0.21	0.73	0.96	0.22	0.73	0.97
HGSOC - FIGO	16:81842873_G_A	16	81842873	A	G	0.24	0.05	0.64	1.27	1.16	1.39	5.00E-07	rs11150413	*PLCG2*			0.24	0.75	0.97	0.22	0.73	0.96
HGSOC - FIGO	1:210787564_A_C	1	210787564	C	A	-1.24	0.25	0.02	0.29	0.18	0.47	5.61E-07	rs192656906	*HHAT*			0.80	0.98	1.00	0.99	1.00	1.00
HGSOC - FIGO	2:63334101_C_T	2	63334101	T	C	-0.23	0.05	0.35	0.80	0.73	0.87	5.63E-07	rs11125947		*RP11-511I11.2*	*RP11-511I11.1*	0.26	0.78	0.97	0.23	0.75	0.97
HGSOC - FIGO	11:12426303_T_C	11	12426303	C	T	-0.28	0.06	0.23	0.76	0.68	0.85	5.67E-07	rs12292762	*PARVA*			0.24	0.76	0.97	0.25	0.77	0.97
HGSOC - FIGO	14:91905916_C_T	14	91905916	T	C	-0.78	0.16	0.03	0.46	0.34	0.63	8.73E-07	rs113939293		*RP11-895M11.3*	*SMEK1*	0.51	0.91	0.99	0.89	0.99	1.00
All OC + FIGO	2:12017617_C_T	2	12017617	T	C	0.25	0.05	0.20	1.28	1.17	1.41	2.18E-07	rs75422962	*AC096559.1*			0.12	0.58	0.93	0.11	0.56	0.93
All OC + FIGO	3:23741038_A_G	3	23741038	G	A	-0.20	0.04	0.38	0.82	0.76	0.89	7.67E-07	rs9833833		*RNU6-788P*	*AC020626.1*	0.34	0.84	0.98	0.29	0.80	0.98
Invasive EOC + FIGO	7:78169035_G_A	7	78169035	A	G	-0.22	0.04	0.45	0.80	0.73	0.87	2.75E-07	rs12533993	*MAGI2*			0.15	0.65	0.95	0.13	0.61	0.94
Invasive EOC + FIGO	5:162106086_C_T	5	162106086	T	C	0.81	0.16	0.02	2.26	1.64	3.11	5.93E-07	rs143120408		*RP11-167P20.1*	*ARL2BPP5*	0.45	0.89	0.99	0.88	0.99	1.00
Invasive EOC + FIGO	18:5360720_C_T	18	5360720	T	C	-0.40	0.08	0.13	0.67	0.57	0.79	7.04E-07	rs72865035		*CTD-3096M3.1*	*CTD-3096M3.2*	0.27	0.79	0.97	0.39	0.86	0.98
Invasive EOC + FIGO	18:5361910_T_C	18	5361910	C	T	-0.39	0.08	0.13	0.68	0.58	0.79	7.20E-07	rs28637484		*CTD-3096M3.1*	*CTD-3096M3.2*	0.28	0.79	0.97	0.38	0.86	0.98
Invasive EOC + FIGO	7:78170371_G_A	7	78170371	A	G	-0.22	0.04	0.44	0.81	0.74	0.88	9.10E-07	rs2362632	*MAGI2*			0.36	0.85	0.98	0.32	0.82	0.98
HGSOC + FIGO	2:119594136_G_A	2	119594136	A	G	0.39	0.07	0.10	1.48	1.28	1.72	1.37E-07	rs60713612		*RP11-19E11.1*	*EN1*	0.08	0.45	0.89	0.12	0.58	0.93
HGSOC + FIGO	2:119584924_A_G	2	119584924	G	A	0.40	0.08	0.10	1.49	1.28	1.72	1.51E-07	rs67800871		*AC093901.1*	*RP11-19E11.1*	0.08	0.47	0.90	0.13	0.60	0.94
HGSOC + FIGO	2:119594348_G_A	2	119594348	A	G	0.39	0.07	0.10	1.48	1.28	1.71	1.63E-07	rs56245258		*RP11-19E11.1*	*EN1*	0.09	0.49	0.91	0.14	0.61	0.94
HGSOC + FIGO	2:119590125_T_C	2	119590125	C	T	0.39	0.07	0.10	1.48	1.28	1.71	1.74E-07	rs56358111		*AC093901.1*	*RP11-19E11.1*	0.09	0.51	0.91	0.14	0.63	0.94
HGSOC + FIGO	2:119607470_C_A	2	119607470	A	C	0.39	0.07	0.10	1.47	1.27	1.71	1.97E-07	rs893572		*EN1*	*MARCO*	0.10	0.53	0.92	0.16	0.65	0.95
HGSOC + FIGO	2:119634199_C_G	2	119634199	G	C	0.39	0.08	0.11	1.48	1.28	1.72	2.28E-07	rs66498268		*EN1*	*MARCO*	0.12	0.57	0.93	0.18	0.68	0.96
HGSOC + FIGO	2:119621724_C_G	2	119621724	G	C	0.39	0.08	0.10	1.47	1.27	1.71	2.75E-07	rs17007470		*EN1*	*MARCO*	0.14	0.61	0.94	0.20	0.72	0.96
HGSOC + FIGO	2:119622302_G_A	2	119622302	A	G	0.39	0.08	0.10	1.47	1.27	1.71	2.78E-07	rs66677656		*EN1*	*MARCO*	0.14	0.61	0.94	0.20	0.72	0.96
HGSOC + FIGO	2:119623887_A_C	2	119623887	C	A	0.39	0.08	0.10	1.47	1.27	1.71	2.96E-07	rs67731576		*EN1*	*MARCO*	0.14	0.63	0.94	0.21	0.73	0.96
HGSOC + FIGO	2:119615001_T_G	2	119615001	G	T	0.38	0.08	0.10	1.47	1.27	1.70	3.31E-07	rs17007326		*EN1*	*MARCO*	0.16	0.65	0.95	0.23	0.75	0.97
HGSOC + FIGO	2:119614415_C_T	2	119614415	T	C	0.38	0.07	0.10	1.47	1.27	1.70	3.37E-07	rs58063687		*EN1*	*MARCO*	0.16	0.65	0.95	0.23	0.75	0.97
HGSOC + FIGO	2:119610043_G_T	2	119610043	T	G	0.38	0.07	0.10	1.46	1.26	1.69	3.41E-07	rs6719822		*EN1*	*MARCO*	0.16	0.66	0.95	0.23	0.75	0.97
HGSOC + FIGO	10:131224242_G_A	10	131224242	A	G	0.75	0.15	0.02	2.11	1.58	2.83	4.86E-07	rs72845444		*RP11-168C9.1*	*MGMT*	0.35	0.85	0.98	0.80	0.98	1.00
HGSOC + FIGO	18:5361910_T_C	18	5361910	C	T	-0.46	0.09	0.14	0.63	0.53	0.76	5.00E-07	rs28637484		*CTD-3096M3.1*	*CTD-3096M3.2*	0.22	0.74	0.97	0.39	0.86	0.98
HGSOC + FIGO	18:5360720_C_T	18	5360720	T	C	-0.47	0.09	0.13	0.63	0.52	0.75	5.21E-07	rs72865035		*CTD-3096M3.1*	*CTD-3096M3.2*	0.23	0.75	0.97	0.41	0.87	0.99
HGSOC + FIGO	2:119631220_C_T	2	119631220	T	C	0.38	0.08	0.10	1.47	1.26	1.71	5.46E-07	rs56266357		*EN1*	*MARCO*	0.23	0.75	0.97	0.32	0.82	0.98
HGSOC + FIGO	2:119631959_C_T	2	119631959	T	C	0.38	0.08	0.10	1.47	1.26	1.71	5.73E-07	rs12618885		*EN1*	*MARCO*	0.24	0.76	0.97	0.33	0.83	0.98
HGSOC + FIGO	21:15599766_G_A	21	15599766	A	G	-0.45	0.09	0.09	0.64	0.54	0.76	6.14E-07	rs3761316	*RBM11*			0.26	0.77	0.97	0.42	0.88	0.99
HGSOC + FIGO	21:15606959_G_A	21	15606959	A	G	-0.44	0.09	0.09	0.64	0.54	0.76	6.21E-07	rs17305899		*RBM11*	*ABCC13*	0.26	0.78	0.97	0.41	0.88	0.99
HGSOC + FIGO	2:119633000_C_T	2	119633000	T	C	0.38	0.08	0.10	1.47	1.26	1.71	6.43E-07	rs56061010		*EN1*	*MARCO*	0.26	0.77	0.97	0.35	0.84	0.98
HGSOC + FIGO	21:15609493_G_C	21	15609493	C	G	-0.45	0.09	0.09	0.64	0.54	0.76	6.64E-07	rs12482047	*ABCC13*			0.27	0.79	0.97	0.43	0.88	0.99
HGSOC + FIGO	21:15609494_G_T	21	15609494	T	G	-0.44	0.09	0.09	0.64	0.54	0.77	6.73E-07	rs12482048	*ABCC13*			0.27	0.79	0.97	0.43	0.88	0.99
HGSOC + FIGO	2:119634182_C_T	2	119634182	T	C	0.38	0.08	0.10	1.47	1.26	1.71	7.33E-07	rs67606735		*EN1*	*MARCO*	0.28	0.79	0.97	0.38	0.86	0.98
HGSOC + FIGO	18:5357386_A_G	18	5357386	G	A	-0.46	0.09	0.14	0.63	0.53	0.76	7.94E-07	rs9951791		*CTD-3096M3.1*	*CTD-3096M3.2*	0.31	0.81	0.98	0.49	0.90	0.99
HGSOC + FIGO	7:78169035_G_A	7	78169035	A	G	-0.25	0.05	0.46	0.78	0.70	0.86	8.19E-07	rs12533993	*MAGI2*			0.32	0.82	0.98	0.31	0.82	0.98

SNP	MAGMA gene	GWAS analysis	SNP	Chromosome	Position	Effect	Baseline	EAF	logOR	SE	P value
rs77770767	*CABLES1*	HGSOC - FIGO	18:20798091_G_C	18	20798091	C	G	0.29	-0.22	0.05	8.39E-06
rs28589524	*CABLES1*	HGSOC - FIGO	18:20746829_G_A	18	20746829	A	G	0.82	-0.20	0.06	4.12E-04
rs6507532	*CABLES1*	HGSOC - FIGO	18:20781280_A_G	18	20781280	G	A	0.97	-0.43	0.13	1.28E-03
rs4281829	*CABLES1*	HGSOC - FIGO	18:20705310_G_A	18	20705310	A	G	0.92	-0.22	0.08	3.63E-03
rs2448233	*PPP2R5C*	HGSOC + FIGO	14:102358687_G_T	14	102358687	T	G	0.19	-0.24	0.06	1.50E-04
rs59784377	*PPP2R5C*	HGSOC + FIGO	14:102391181_C_T	14	102391181	T	C	0.09	-0.27	0.08	1.13E-03
rs3783362	*PPP2R5C*	HGSOC + FIGO	14:102345752_G_A	14	102345752	A	G	0.05	-0.33	0.11	3.12E-03
rs79999043	*PPP2R5C*	HGSOC + FIGO	14:102327574_A_G	14	102327574	G	A	0.04	-0.35	0.12	5.62E-03
rs11492866	*FAM35A*	All OC + FIGO	10:88936927_T_G	10	88936927	G	T	0.42	-0.12	0.04	3.40E-03

Supplementary Table 2: Summary statistics for variants at $p< 10^{-6}$, and MAGMA gene variants selected for eQTL analysis. (a) Contents. (b) Variants at $p<10^{-6}$ are listed for each GWAS analysis (indicated "GWAS name") and annotated with nearest genes. BFDP values are listed for priors of 10^{-4}, 10^{-5}, and 10^{-6} and for odds ratios of 1.5 or 2, respectively. (c) Variants selected for genotyping and eQTL testing after MAGMA analysis are indicated with corresponding summary statistics. EAF: effect allele frequency, OR/ORhi: odds ratio, logOR: log odds ratio (natural logarithm), SE: standard error, P: p-value.

Supplementary Table 3: MAGMA results for all genes for each of the six GWAS analyses.

Chromosomal regions, number of variants (NSNPS) and z-statistics are given for a MAGMA window of 25 kb around each gene as derived from the FUMA platform. GENE: ENSG gene ID, CHR: chromosome, START: gene start + window, STOP: gene stop + window, NSNPS: number of SNPs annotated to the gene, NPARAM: number of parameters, N: sample size per SNP, ZSTAT: Z-value for each gene, P: p-value for each gene after association testing, SYMBOL: gene symbol or name, job window: specified as 25kb around each gene.

A) MAGMA analysis All OC + FIGO

GENE	CHR	START	STOP	NSNPS	NPARAM	N	ZSTAT	P	SYMBOL	job	window
ENSG00000269308	1	793043	844983	67	12	120000	-0.81092	0.7913	AL645608.2	76353	25kb
ENSG00000187634	1	835260	904955	203	29	120000	0.15747	0.43744	SAMD11	76353	25kb
ENSG00000268179	1	836264	891445	162	24	120000	-0.22673	0.58968	AL645608.1	76353	25kb
ENSG00000188976	1	854584	919689	184	31	120000	0.7497	0.22672	NOC2L	76353	25kb
ENSG00000187961	1	870967	926095	159	26	120000	0.95563	0.16963	KLHL17	76353	25kb
ENSG00000187583	1	876877	936245	191	27	120000	1.0691	0.14252	PLEKHN1	76353	25kb
ENSG00000187642	1	885579	942497	191	27	120000	1.097	0.13632	C1orf170	76353	25kb
ENSG00000188290	1	909342	960552	170	18	120000	0.62418	0.26625	HES4	76353	25kb
ENSG00000187608	1	923803	974920	151	16	120000	0.25395	0.39977	ISG15	76353	25kb
ENSG00000188157	1	930503	1016496	216	23	120000	0.037579	0.48501	AGRN	76353	25kb
ENSG00000237330	1	981346	1034687	134	20	120000	-0.58883	0.72201	RNF223	76353	25kb
ENSG00000131591	1	992198	1076741	258	22	120000	0.11643	0.45366	C1orf159	76353	25kb
ENSG00000162571	1	1084264	1158315	336	37	120000	-1.0763	0.85909	TTLL10	76353	25kb
ENSG00000186891	1	1113888	1167071	256	18	120000	-1.0213	0.84644	TNFRSF18	76353	25kb
ENSG00000186827	1	1121706	1174518	245	19	120000	-1.066	0.85679	TNFRSF4	76353	25kb
ENSG00000078808	1	1127288	1192411	265	15	120000	-0.86088	0.80535	SDF4	76353	25kb
ENSG00000176022	1	1142629	1195421	216	16	120000	-0.57248	0.7165	B3GALT6	76353	25kb
ENSG00000184163	1	1152826	1207102	205	17	120000	-0.71268	0.76198	FAM132A	76353	25kb
ENSG00000160087	1	1164289	1234265	231	30	120000	-1.7054	0.95594	UBE2J2	76353	25kb
ENSG00000162572	1	1190816	1252409	171	23	120000	-2.1999	0.98609	SCNN1D	76353	25kb
ENSG00000131584	1	1202756	1269989	186	26	120000	-1.8402	0.96713	ACAP3	76353	25kb
ENSG00000169972	1	1218947	1272057	130	21	120000	-1.3324	0.90863	PUSL1	76353	25kb
ENSG00000127054	1	1221965	1285071	151	20	120000	-1.1981	0.88456	CPSF3L	76353	25kb
ENSG00000224051	1	1235136	1289277	128	14	120000	-1.0046	0.84245	GLTPD1	76353	25kb
ENSG00000169962	1	1241694	1295686	130	13	120000	-0.877	0.80976	TAS1R3	76353	25kb
ENSG00000107404	1	1245656	1309730	196	19	120000	-1.0092	0.84356	DVL1	76353	25kb
ENSG00000162576	1	1263069	1322157	215	19	120000	-1.0852	0.86107	MXRA8	76353	25kb
ENSG00000175756	1	1284110	1335875	172	17	120000	-1.21	0.88686	AURKAIP1	76353	25kb
ENSG00000221978	1	1296091	1359708	214	19	120000	-1.3912	0.91792	CCNL2	76353	25kb
ENSG00000224870	1	1309902	1362426	157	15	120000	-1.4295	0.92358	RP4-758J18.2	76353	25kb
ENSG00000242485	1	1312288	1367693	147	15	120000	-1.4767	0.93013	MRPL20	76353	25kb
ENSG00000235098	1	1328800	1382149	143	18	120000	-1.6772	0.95325	ANKRD65	76353	25kb
ENSG00000205116	1	1336508	1388167	141	18	120000	-1.6031	0.94555	TMEM88B	76353	25kb
ENSG00000179403	1	1345241	1403262	133	18	120000	-1.3372	0.90943	VWA1	76353	25kb
ENSG00000215915	1	1360069	1430538	132	21	120000	-0.82712	0.79592	ATAD3C	76353	25kb
ENSG00000160072	1	1382143	1458228	123	21	120000	-0.59213	0.72312	ATAD3B	76353	25kb
ENSG00000197785	1	1422531	1495067	156	22	120000	-0.7683	0.77885	ATAD3A	76353	25kb
ENSG00000205090	1	1445554	1500833	139	19	120000	-0.9073	0.81788	TMEM240	76353	25kb
ENSG00000160075	1	1452053	1535249	238	29	120000	-0.98718	0.83822	SSU72	76353	25kb
ENSG00000215014	1	1485355	1536373	156	22	120000	-1.0598	0.85539	AL645728.1	76353	25kb
ENSG00000228594	1	1508392	1560476	152	23	120000	-0.80685	0.79012	C1orf233	76353	25kb
ENSG00000197530	1	1525795	1590990	118	16	120000	-0.34221	0.6339	MIB2	76353	25kb
ENSG00000189409	1	1542474	1595639	83	14	120000	-0.32915	0.62898	MMP23B	76353	25kb
ENSG00000248333	1	1545603	1615473	110	20	120000	-0.24494	0.59675	CDK11B	76353	25kb
ENSG00000189339	1	1567939	1649167	100	19	120000	0.29818	0.38278	SLC35E2B	76353	25kb
ENSG00000008128	1	1609169	1680766	111	16	120000	1.3832	0.0833	CDK11A	76353	25kb
ENSG00000215790	1	1631277	1702431	119	15	120000	2.4595	0.006956	SLC35E2	76353	25kb
ENSG00000008130	1	1657671	1736896	134	16	120000	3.1952	0.00069864	NADK	76353	25kb
ENSG00000078369	1	1691729	1847495	249	26	120000	2.4823	0.0065278	GNB1	76353	25kb
ENSG00000169885	1	1821266	1873735	131	12	120000	2.4855	0.0064691	CALML6	76353	25kb
ENSG00000178821	1	1824029	1875712	142	13	120000	2.5419	0.0055121	TMEM52	76353	25kb
ENSG00000142609	1	1828396	1960276	438	35	120000	1.7469	0.040325	C1orf222	76353	25kb
ENSG00000187730	1	1925780	1987192	173	30	120000	1.0382	0.14959	GABRD	76353	25kb
ENSG00000067606	1	1956909	2141834	497	44	120000	1.2392	0.10763	PRKCZ	76353	25kb
ENSG00000162585	1	2090903	2169159	157	28	120000	1.0691	0.14252	C1orf86	76353	25kb
ENSG00000269554	1	2096237	2148179	101	18	120000	0.96107	0.16826	AL590822.2	76353	25kb
ENSG00000203301	1	2118360	2170620	107	25	120000	0.9881	0.16155	AL590822.1	76353	25kb
ENSG00000157933	1	2135134	2266558	376	62	120000	0.56217	0.287	SKI	76353	25kb
ENSG00000116151	1	2227692	2348146	440	64	120000	-0.63389	0.73692	MORN1	76353	25kb
ENSG00000269753	1	2233581	2284042	215	42	120000	-0.84477	0.80088	AL589739.1	76353	25kb
ENSG00000157916	1	2298267	2361883	215	52	120000	-0.20421	0.58091	RER1	76353	25kb
ENSG00000157911	1	2311236	2370236	197	51	120000	0.093308	0.46283	PEX10	76353	25kb
ENSG00000149527	1	2332419	2461969	391	58	120000	-0.48054	0.68458	PLCH2	76353	25kb
ENSG00000157881	1	2414972	2483039	171	24	120000	0.60179	0.27366	PANK4	76353	25kb
ENSG00000197921	1	2435184	2486684	130	20	120000	0.30774	0.37914	HES5	76353	25kb
ENSG00000157873	1	2462078	2521821	157	18	120000	0.49924	0.30881	TNFRSF14	76353	25kb
ENSG00000157870	1	2492930	2547908	175	13	120000	0.14705	0.44154	FAM213B	76353	25kb
ENSG00000142606	1	2497078	2589481	256	13	120000	0.12672	0.44958	MMEL1	76353	25kb
ENSG00000215912	1	2542415	2743286	266	18	120000	0.29065	0.38566	TTC34	76353	25kb
ENSG00000169717	1	2913046	2964465	196	8	120000	-0.4026	0.65638	ACTRT2	76353	25kb
ENSG00000142611	1	2960732	3380185	1546	186	120000	-1.4334	0.92413	PRDM16	76353	25kb
ENSG00000130762	1	3345990	3422677	281	24	120000	0.15122	0.4399	ARHGEF16	76353	25kb
ENSG00000162591	1	3381484	3553059	671	95	120000	-0.11257	0.54481	MEGF6	76353	25kb
ENSG00000158109	1	3516566	3571691	183	29	120000	1.0004	0.15857	TPRG1L	76353	25kb
ENSG00000116213	1	3522331	3594325	244	44	120000	0.54228	0.29381	WRAP73	76353	25kb
ENSG00000078900	1	3544084	3677765	421	70	120000	-0.30663	0.62044	TP73	76353	25kb
ENSG00000162592	1	3643962	3713209	290	38	120000	0.055841	0.47773	CCDC27	76353	25kb
ENSG00000235169	1	3664352	3717546	230	34	120000	0.29383	0.38444	SMIM1	76353	25kb
ENSG00000130764	1	3671784	3738068	321	28	120000	0.10408	0.45855	LRRC47	76353	25kb
ENSG00000116198	1	3703645	3798778	566	28	120000	-0.42186	0.66343	CEP104	76353	25kb
ENSG00000169598	1	3748845	3826993	428	42	120000	-0.81764	0.79322	DFFB	76353	25kb
ENSG00000198912	1	3780689	3841857	283	28	120000	-2.1736	0.98513	C1orf174	76353	25kb
ENSG00000196581	1	4689792	4877594	825	97	120000	0.34447	0.36525	AJAP1	76353	25kb
ENSG00000131697	1	5897871	6077533	582	35	120000	2.2879	0.011073	NPHP4	76353	25kb
ENSG00000069424	1	6026526	6186253	497	69	120000	2.6084	0.0045479	KCNAB2	76353	25kb
ENSG00000268332	1	6075630	6126399	142	28	120000	2.5068	0.0060919	AL035406.1	76353	25kb
ENSG00000116254	1	6136853	6265183	420	47	120000	-0.57369	0.71691	CHD5	76353	25kb
ENSG00000116251	1	6216329	6294449	193	28	120000	0.25342	0.39997	RPL22	76353	25kb
ENSG00000158286	1	6240535	6306359	141	24	120000	0.19083	0.42433	RNF207	76353	25kb
ENSG00000116237	1	6256253	6321032	149	21	120000	0.66451	0.25318	ICMT	76353	25kb
ENSG00000173673	1	6279252	6330638	119	18	120000	1.1614	0.12274	HES3	76353	25kb
ENSG00000158292	1	6282406	6346035	151	27	120000	1.1459	0.12591	GPR153	76353	25kb
ENSG00000097021	1	6299329	6479451	585	32	120000	0.92877	0.1765	ACOT7	76353	25kb
ENSG00000069812	1	6447478	6509730	215	19	120000	0.96003	0.16852	HES2	76353	25kb

ENSG00000187017	1	6459848	6546430	249	25	120000	0.75088	0.22636	ESPN	76353	25kb
ENSG00000215788	1	6496211	6551255	128	20	120000	0.72812	0.23327	TNFRSF25	76353	25kb
ENSG00000171680	1	6501152	6605121	268	29	120000	0.36242	0.35852	PLEKHG5	76353	25kb
ENSG00000162408	1	6556407	6639595	248	16	120000	-0.14744	0.55861	NOL9	76353	25kb
ENSG00000173662	1	6590241	6664817	198	16	120000	-0.30637	0.62034	TAS1R1	76353	25kb
ENSG00000204859	1	6615061	6674340	115	24	120000	-0.29503	0.61601	ZBTB48	76353	25kb
ENSG00000162413	1	6625784	6699667	145	24	120000	-0.23546	0.59308	KLHL21	76353	25kb
ENSG00000116273	1	6648745	6709093	122	17	120000	-0.0778	0.53101	PHF13	76353	25kb
ENSG00000041988	1	6659926	6720646	124	15	120000	-0.38869	0.65125	THAP3	76353	25kb
ENSG00000007923	1	6669228	6786984	242	21	120000	-0.32785	0.62849	DNAJC11	76353	25kb
ENSG00000171735	1	6820384	7854766	2851	217	120000	-0.16651	0.56612	CAMTA1	76353	25kb
ENSG00000049245	1	7806329	7866492	171	28	120000	0.80029	0.21177	VAMP3	76353	25kb
ENSG00000049246	1	7819380	7930237	406	35	120000	0.1355	0.44611	PER3	76353	25kb
ENSG00000049247	1	7878143	7938572	259	27	120000	-0.10881	0.54332	UTS2	76353	25kb
ENSG00000049249	1	7954907	8025926	175	21	120000	-0.74739	0.77259	TNFRSF9	76353	25kb
ENSG00000116288	1	7989351	8070565	144	17	120000	0.35081	0.36287	PARK7	76353	25kb
ENSG00000116285	1	8039464	8111368	113	11	120000	0.6692	0.25168	ERRFI1	76353	25kb
ENSG00000162426	1	8352886	8429227	203	29	120000	1.7027	0.04431	SLC45A1	76353	25kb
ENSG00000142599	1	8387457	8902702	807	32	120000	1.3899	0.082286	RERE	76353	25kb
ENSG00000074800	1	8896061	8964308	132	18	120000	0.14301	0.44314	ENO1	76353	25kb
ENSG00000131686	1	8980926	9060151	291	54	120000	-1.5276	0.93669	CA6	76353	25kb
ENSG00000197241	1	9038359	9111404	220	43	120000	-0.7815	0.78275	SLC2A7	76353	25kb
ENSG00000142583	1	9070166	9173537	345	34	120000	0.31798	0.37525	SLC2A5	76353	25kb
ENSG00000180758	1	9135364	9214229	212	25	120000	1.0353	0.15027	GPR157	76353	25kb
ENSG00000049239	1	9269834	9356396	330	43	120000	-0.80462	0.78948	H6PD	76353	25kb
ENSG00000171621	1	9327939	9454591	533	77	120000	-1.0546	0.85419	SPSB1	76353	25kb
ENSG00000171612	1	9574541	9670237	136	16	120000	-0.37477	0.64608	SLC25A33	76353	25kb
ENSG00000188807	1	9623932	9699935	117	16	120000	-0.49195	0.68862	TMEM201	76353	25kb
ENSG00000171608	1	9686790	9814172	220	41	120000	-0.63248	0.73646	PIK3CD	76353	25kb
ENSG00000179840	1	9687668	9739644	112	21	120000	-0.77492	0.78081	C1orf200	76353	25kb
ENSG00000171603	1	9764084	9909584	208	24	120000	0.90063	0.18389	CLSTN1	76353	25kb
ENSG00000178585	1	9883334	9995394	170	32	120000	0.34899	0.36355	CTNNBIP1	76353	25kb
ENSG00000162441	1	9957173	10028465	123	20	120000	-0.12499	0.54973	LZIC	76353	25kb
ENSG00000173614	1	9978486	10070559	185	20	120000	0.48158	0.31505	NMNAT1	76353	25kb
ENSG00000162444	1	10032264	10101078	123	14	120000	0.85854	0.1953	RBP7	76353	25kb
ENSG00000130939	1	10067890	10266297	413	23	120000	0.96171	0.1681	UBE4B	76353	25kb
ENSG00000054523	1	10245863	10466661	698	20	120000	0.30818	0.37897	KIF1B	76353	25kb
ENSG00000142657	1	10433649	10505201	191	23	120000	-0.90423	0.81706	PGD	76353	25kb
ENSG00000251503	1	10465159	10537210	147	18	120000	-0.95207	0.82947	APITD1-CORT	76353	25kb
ENSG00000175279	1	10465159	10537210	147	18	120000	-0.95207	0.82947	APITD1	76353	25kb
ENSG00000241563	1	10484776	10536959	101	10	120000	-0.70099	0.75835	CORT	76353	25kb
ENSG00000160049	1	10491579	10557583	118	13	120000	-0.49301	0.689	DFFA	76353	25kb
ENSG00000142655	1	10507345	10715815	496	41	120000	-0.21751	0.58609	PEX14	76353	25kb
ENSG00000130940	1	10671661	10881707	577	100	120000	0.65994	0.25464	CASZ1	76353	25kb
ENSG00000175262	1	10981528	11067094	288	38	120000	0.11301	0.45501	C1orf127	76353	25kb
ENSG00000120948	1	11047414	11110796	148	21	120000	1.7604	0.03917	TARDBP	76353	25kb
ENSG00000009724	1	11061580	11132290	162	16	120000	1.7683	0.038508	MASP2	76353	25kb
ENSG00000116649	1	11089641	11145081	154	12	120000	1.145	0.12611	SRM	76353	25kb
ENSG00000171824	1	11101675	11184938	213	10	120000	0.63641	0.26226	EXOSC10	76353	25kb
ENSG00000198793	1	11141592	11347564	441	14	120000	0.54919	0.29144	MTOR	76353	25kb
ENSG00000171819	1	11224398	11281038	118	7	120000	0.48025	0.31552	ANGPTL7	76353	25kb
ENSG00000120942	1	11308263	11381106	135	15	120000	0.029707	0.48815	UBIAD1	76353	25kb
ENSG00000204624	1	11514223	11622641	321	23	120000	0.92975	0.17625	PTCHD2	76353	25kb
ENSG00000116661	1	11683424	11740842	178	38	120000	0.81584	0.2073	FBXO2	76353	25kb
ENSG00000132879	1	11689432	11748384	183	40	120000	1.0081	0.1567	FBXO44	76353	25kb
ENSG00000116663	1	11699181	11759411	182	41	120000	0.90084	0.18384	FBXO6	76353	25kb
ENSG00000116670	1	11709537	11776707	217	45	120000	0.49696	0.30961	MAD2L2	76353	25kb
ENSG00000162490	1	11726786	11810914	248	55	120000	0.59769	0.27502	DRAXIN	76353	25kb
ENSG00000177674	1	11771141	11839859	242	38	120000	-0.48356	0.68565	AGTRAP	76353	25kb
ENSG00000215910	1	11796844	11874642	355	25	120000	-0.70474	0.75951	C1orf167	76353	25kb
ENSG00000177000	1	11820780	11891977	358	19	120000	-0.84389	0.80063	MTHFR	76353	25kb
ENSG00000011021	1	11841207	11928201	385	24	120000	-0.76328	0.77735	CLCN6	76353	25kb
ENSG00000175206	1	11880766	11933402	221	27	120000	-0.57188	0.7163	NPPA	76353	25kb
ENSG00000120937	1	11892521	11943988	217	31	120000	-0.45097	0.67399	NPPB	76353	25kb
ENSG00000116685	1	11954648	12011485	227	29	120000	-0.82854	0.79632	KIAA2013	76353	25kb
ENSG00000083444	1	11969262	12060595	265	22	120000	-1.2467	0.89374	PLOD1	76353	25kb
ENSG00000116688	1	12015238	12098571	175	21	120000	-1.835	0.96675	MFN2	76353	25kb
ENSG00000116691	1	12054523	12117102	138	25	120000	-0.73497	0.76882	MIIP	76353	25kb
ENSG00000120949	1	12098434	12229264	343	62	120000	1.0137	0.15536	TNFRSF8	76353	25kb
ENSG00000028137	1	12202060	12294285	274	37	120000	-0.16305	0.56476	TNFRSF1B	76353	25kb
ENSG00000048707	1	12265124	12597099	689	27	120000	0.80081	0.21162	VPS13D	76353	25kb
ENSG00000162496	1	12602939	12702737	426	55	120000	0.11294	0.45504	DHRS3	76353	25kb
ENSG00000204518	1	12679566	12752097	222	23	120000	-0.68026	0.75183	AADACL4	76353	25kb
ENSG00000188984	1	12751118	12813726	188	23	120000	-0.45014	0.6737	AADACL3	76353	25kb
ENSG00000157330	1	12781163	12848847	205	19	120000	-0.20615	0.58166	C1orf158	76353	25kb
ENSG00000116726	1	12809984	12863049	146	26	120000	0.33612	0.36839	PRAMEF12	76353	25kb
ENSG00000116721	1	12826546	12881777	220	22	120000	-0.0088892	0.50355	PRAMEF1	76353	25kb
ENSG00000204513	1	12859468	12916264	272	19	120000	-0.68157	0.75225	PRAMEF11	76353	25kb
ENSG00000179172	1	12882261	12933578	139	8	120000	-0.6854	0.75345	HNRNPCL1	76353	25kb
ENSG00000120952	1	12891941	12946764	126	6	120000	-0.8964	0.81498	PRAMEF2	76353	25kb
ENSG00000243073	1	12914045	12971025	26	4	120000	-0.79808	0.78759	PRAMEF4	76353	25kb
ENSG00000187545	1	12927727	12983101	3	2	120000	-0.92728	0.82311	PRAMEF10	76353	25kb
ENSG00000204510	1	12951450	13005569	2	1	120000	-1.1502	0.87496	PRAMEF7	76353	25kb
ENSG00000232423	1	12973181	13142751	7	3	120000	-1.8593	0.96851	PRAMEF6	76353	25kb
ENSG00000204508	1	13010543	13063381	1	1	120000	-1.2593	0.89604	PRAMEF22	76353	25kb
ENSG00000229571	1	13191356	13244581	1	1	120000	-0.34982	0.63676	PRAMEF26	76353	25kb
ENSG00000204502	1	13334819	13394057	6	3	120000	-1.4428	0.92546	PRAMEF5	76353	25kb
ENSG00000182330	1	13361646	13415765	6	3	120000	-1.4428	0.92546	PRAMEF8	76353	25kb
ENSG00000204495	1	13422414	13477656	1	1	120000	0.16301	0.43526	PRAMEF13	76353	25kb
ENSG00000204491	1	13449689	13502522	3	2	120000	0.17144	0.43194	PRAMEF18	76353	25kb
ENSG00000204488	1	13470254	13523260	3	2	120000	0.17144	0.43194	PRAMEF16	76353	25kb
ENSG00000204486	1	13491066	13551943	1	1	120000	-0.80838	0.79056	PRAMEF21	76353	25kb
ENSG00000204480	1	13669889	13723405	1	1	120000	-0.063799	0.52543	PRAMEF19	76353	25kb
ENSG00000204479	1	13691092	13744089	3	2	120000	0.11151	0.4556	PRAMEF17	76353	25kb
ENSG00000204478	1	13711907	13772803	2	1	120000	0.34522	0.36496	PRAMEF20	76353	25kb
ENSG00000162494	1	13776445	13865543	391	58	120000	-1.1769	0.88038	LRRC38	76353	25kb

ENSG00000162493	1	13884960	13969452	323	48	120000	0.75731	0.22443	PDPN	76353	25kb
ENSG00000116731	1	14001693	14176574	558	34	120000	-0.60804	0.72842	PRDM2	76353	25kb
ENSG00000189337	1	14900200	15469539	2050	215	120000	1.4036	0.080226	KAZN	76353	25kb
ENSG00000171729	1	15454028	15571976	628	40	120000	0.95899	0.16878	TMEM51	76353	25kb
ENSG00000204464	1	15465840	15522813	300	24	120000	0.16191	0.43569	C1orf195	76353	25kb
ENSG00000142621	1	15548768	15751779	721	78	120000	2.0976	0.017969	FHAD1	76353	25kb
ENSG00000142634	1	15711391	15781839	185	37	120000	-0.27789	0.60945	EFHD2	76353	25kb
ENSG00000162438	1	15739935	15800737	172	34	120000	-0.45893	0.67686	CTRC	76353	25kb
ENSG00000142615	1	15758223	15823586	187	31	120000	-0.98776	0.83836	CELA2A	76353	25kb
ENSG00000215704	1	15767404	15842895	243	29	120000	-1.1139	0.86735	CELA2B	76353	25kb
ENSG00000132906	1	15792327	15878029	267	16	120000	-1.1782	0.88064	CASP9	76353	25kb
ENSG00000116138	1	15828308	15943874	384	15	120000	-0.17347	0.56886	DNAJC16	76353	25kb
ENSG00000116771	1	15873848	15936605	223	13	120000	0.2183	0.4136	AGMAT	76353	25kb
ENSG00000197312	1	15918995	16020539	259	9	120000	0.96358	0.16763	DDI2	76353	25kb
ENSG00000215695	1	15961364	16013217	106	7	120000	0.87246	0.19148	RSC1A1	76353	25kb
ENSG00000116786	1	15985827	16086264	253	18	120000	0.52039	0.30139	PLEKHM2	76353	25kb
ENSG00000162461	1	16037900	16092891	144	21	120000	0.11802	0.45302	SLC25A34	76353	25kb
ENSG00000162460	1	16043917	16099477	129	23	120000	0.15079	0.44007	TMEM82	76353	25kb
ENSG00000162458	1	16058102	16138089	211	37	120000	-0.10431	0.54154	FBLIM1	76353	25kb
ENSG00000065526	1	16149359	16291955	250	26	120000	1.0667	0.14305	SPEN	76353	25kb
ENSG00000116809	1	16243364	16327627	203	21	120000	1.5134	0.065093	ZBTB17	76353	25kb
ENSG00000183888	1	16305731	16360302	297	20	120000	1.4015	0.080536	C1orf64	76353	25kb
ENSG00000173641	1	16315523	16371089	314	22	120000	1.0394	0.14931	HSPB7	76353	25kb
ENSG00000186510	1	16320370	16385545	392	29	120000	0.54505	0.29286	CLCNKA	76353	25kb
ENSG00000184908	1	16345272	16408803	305	29	120000	0.71748	0.23654	CLCNKB	76353	25kb
ENSG00000185519	1	16359264	16425127	287	26	120000	0.87876	0.18977	FAM131C	76353	25kb
ENSG00000142627	1	16425832	16507582	216	26	120000	1.3011	0.096614	EPHA2	76353	25kb
ENSG00000142632	1	16499349	16564104	187	17	120000	0.56437	0.28625	ARHGEF19	76353	25kb
ENSG00000204377	1	16530368	16581038	107	13	120000	-0.35845	0.64	C1orf134	76353	25kb
ENSG00000132881	1	16533195	16588657	118	13	120000	-0.39039	0.65187	RSG1	76353	25kb
ENSG00000037637	1	16548334	16703949	327	14	120000	0.22922	0.40935	FBXO42	76353	25kb
ENSG00000055070	1	16654070	16749640	231	24	120000	0.32612	0.37217	SZRD1	76353	25kb
ENSG00000187144	1	16688612	16788919	229	33	120000	0.3901	0.34823	SPATA21	76353	25kb
ENSG00000157191	1	16742167	16811573	182	25	120000	-0.035738	0.51425	NECAP2	76353	25kb
ENSG00000268674	1	16840561	16891070	44	8	120000	0.20352	0.41936	FAM231B	76353	25kb
ENSG00000219481	1	16863814	16965057	12	7	120000	-1.5752	0.9424	NBPF1	76353	25kb
ENSG00000237847	1	16974498	17025007	29	9	120000	-0.22848	0.59036	FAM231A	76353	25kb
ENSG00000268991	1	17035447	17085956	8	5	120000	0.024672	0.49016	FAM231C	76353	25kb
ENSG00000058453	1	17041768	17324474	255	25	120000	-1.4626	0.92821	CROCC	76353	25kb
ENSG00000117122	1	17275997	17332330	130	22	120000	-1.6522	0.95075	MFAP2	76353	25kb
ENSG00000159363	1	17287453	17363423	176	27	120000	-2.2927	0.98907	ATP13A2	76353	25kb
ENSG00000117118	1	17320217	17405665	315	17	120000	-1.9012	0.97136	SDHB	76353	25kb
ENSG00000117115	1	17368256	17470948	440	29	120000	-2.1528	0.98433	PADI2	76353	25kb
ENSG00000142623	1	17506621	17597500	415	54	120000	0.22281	0.41184	PADI1	76353	25kb
ENSG00000142619	1	17550593	17635728	403	38	120000	-0.32783	0.62848	PADI3	76353	25kb
ENSG00000159339	1	17609690	17715499	533	43	120000	0.62902	0.26467	PADI4	76353	25kb
ENSG00000269468	1	17630507	17701070	375	34	120000	0.98582	0.16211	AC004824.2	76353	25kb
ENSG00000179051	1	17708256	17791220	299	18	120000	-0.30705	0.6206	RCC2	76353	25kb
ENSG00000074964	1	17841330	18049369	522	50	120000	-0.18662	0.57402	ARHGEF10L	76353	25kb
ENSG00000117148	1	18056808	18178558	341	34	120000	0.19684	0.42198	ACTL8	76353	25kb
ENSG00000117154	1	18409240	18729977	1167	151	120000	-2.3828	0.99141	IGSF21	76353	25kb
ENSG00000179023	1	18782424	18837478	244	38	120000	-0.049262	0.51964	KLHDC7A	76353	25kb
ENSG00000009709	1	18932500	19100360	511	74	120000	-1.1271	0.87016	PAX7	76353	25kb
ENSG00000179002	1	19141093	19211176	405	46	120000	-0.40868	0.65861	TAS1R2	76353	25kb
ENSG00000255275	1	19150767	19272615	531	62	120000	0.18795	0.42546	RP13-279N23.2	76353	25kb
ENSG00000159423	1	19172926	19254275	377	48	120000	-0.059487	0.52372	ALDH4A1	76353	25kb
ENSG00000169991	1	19205775	19308180	354	36	120000	3.3263	0.00044006	IFFO2	76353	25kb
ENSG00000127481	1	19376000	19561770	397	25	120000	0.48002	0.31561	UBR4	76353	25kb
ENSG00000127463	1	19517158	19603046	287	21	120000	-0.83388	0.79783	EMC1	76353	25kb
ENSG00000053372	1	19553033	19611622	274	17	120000	-0.78489	0.78374	MRTO4	76353	25kb
ENSG00000162482	1	19584052	19640744	233	21	120000	-0.42519	0.66465	AKR7A3	76353	25kb
ENSG00000053371	1	19605459	19663640	224	33	120000	-0.82868	0.79636	AKR7A2	76353	25kb
ENSG00000040487	1	19613820	19680794	232	30	120000	-0.9174	0.82053	PQLC2	76353	25kb
ENSG00000077549	1	19640267	19837066	707	35	120000	-0.31883	0.62507	CAPZB	76353	25kb
ENSG00000270136	1	19898473	20009549	309	33	120000	-1.1836	0.88171	MINOS1-NBL1	76353	25kb
ENSG00000173436	1	19898477	19981314	234	29	120000	-1.1714	0.87928	MINOS1	76353	25kb
ENSG00000158747	1	19942048	20009945	193	34	120000	-0.97633	0.83555	NBL1	76353	25kb
ENSG00000158748	1	19966780	20031055	232	29	120000	-0.73447	0.76867	HTR6	76353	25kb
ENSG00000162542	1	19983706	20151438	591	69	120000	0.36027	0.35932	TMCO4	76353	25kb
ENSG00000178828	1	20115523	20166771	227	42	120000	-0.86529	0.80656	RNF186	76353	25kb
ENSG00000169914	1	20184006	20264438	256	28	120000	1.1469	0.12571	OTUD3	76353	25kb
ENSG00000188784	1	20221502	20275110	159	24	120000	-0.48956	0.68778	PLA2G2E	76353	25kb
ENSG00000188257	1	20276925	20331932	194	34	120000	-0.55257	0.70972	PLA2G2A	76353	25kb
ENSG00000127472	1	20329672	20442683	359	33	120000	-0.0083278	0.50332	PLA2G5	76353	25kb
ENSG00000117215	1	20413432	20471050	157	44	120000	1.6531	0.049158	PLA2G2D	76353	25kb
ENSG00000158786	1	20440819	20501879	152	36	120000	2.3361	0.0097426	PLA2G2F	76353	25kb
ENSG00000187980	1	20462746	20528917	148	31	120000	1.0278	0.15203	PLA2G2C	76353	25kb
ENSG00000162543	1	20487578	20547541	135	23	120000	0.34608	0.36464	UBXN10	76353	25kb
ENSG00000158816	1	20592412	20706387	412	38	120000	-0.80999	0.79103	VWA5B1	76353	25kb
ENSG00000162545	1	20783884	20837713	106	21	120000	-1.1426	0.87341	CAMK2N1	76353	25kb
ENSG00000090432	1	20800943	20859654	129	20	120000	-0.90379	0.81695	MUL1	76353	25kb
ENSG00000183114	1	20853932	20906512	250	34	120000	-1.5075	0.93416	FAM43B	76353	25kb
ENSG00000158825	1	20890441	20970401	378	31	120000	0.1154	0.45407	CDA	76353	25kb
ENSG00000158828	1	20934948	21003004	277	20	120000	0.8228	0.20531	PINK1	76353	25kb
ENSG00000244038	1	20953270	21013000	202	28	120000	0.89896	0.18434	DDOST	76353	25kb
ENSG00000117245	1	20965507	21069510	375	56	120000	0.39504	0.34641	KIF17	76353	25kb
ENSG00000189410	1	21021225	21084330	215	33	120000	-0.67272	0.74944	SH2D5	76353	25kb
ENSG00000127483	1	21044154	21138816	202	25	120000	-0.46325	0.67841	HP1BP3	76353	25kb
ENSG00000075151	1	21107963	21528377	767	27	120000	0.5099	0.30506	EIF4G3	76353	25kb
ENSG00000117298	1	21518740	21696997	494	49	120000	-0.16206	0.56437	ECE1	76353	25kb
ENSG00000142794	1	21741621	21836498	301	25	120000	-0.92282	0.82195	NBPF3	76353	25kb
ENSG00000162551	1	21810858	21929905	480	51	120000	-0.32249	0.62646	ALPL	76353	25kb
ENSG00000076864	1	21897708	22020801	476	67	120000	-1.4137	0.92128	RAP1GAP	76353	25kb
ENSG00000090686	1	21979791	22135099	536	41	120000	-1.0017	0.84175	USP48	76353	25kb
ENSG00000187942	1	22113758	22176714	230	45	120000	-0.16079	0.56387	LDLRAD2	76353	25kb
ENSG00000142798	1	22123738	22288790	554	47	120000	0.035274	0.48593	HSPG2	76353	25kb

ENSG00000219073	1	22278514	22350135	306	28	120000	0.71679	0.23675	CELA3B	76353	25kb
ENSG00000142789	1	22303149	22364032	286	33	120000	0.3978	0.34539	CELA3A	76353	25kb
ENSG00000070831	1	22354120	22444437	280	21	120000	-0.025467	0.51016	CDC42	76353	25kb
ENSG00000162552	1	22418798	22495462	252	33	120000	-0.44075	0.6703	WNT4	76353	25kb
ENSG00000184677	1	22753344	22882650	230	34	120000	1.2796	0.10034	ZBTB40	76353	25kb
ENSG00000070886	1	22865057	22955087	288	39	120000	-0.027537	0.51098	EPHA8	76353	25kb
ENSG00000173372	1	22937999	22991101	166	21	120000	-0.080967	0.53227	C1QA	76353	25kb
ENSG00000159189	1	22945123	22999603	157	18	120000	-0.10015	0.53989	C1QC	76353	25kb
ENSG00000173369	1	22954255	23013031	163	20	120000	-0.11494	0.54576	C1QB	76353	25kb
ENSG00000133216	1	23012332	23266818	599	76	120000	0.68779	0.24579	EPHB2	76353	25kb
ENSG00000215906	1	23254536	23316831	165	20	120000	0.083759	0.46662	LACTBL1	76353	25kb
ENSG00000227868	1	23312327	23367343	77	11	120000	-0.28301	0.61141	C1orf234	76353	25kb
ENSG00000004487	1	23320941	23435182	155	14	120000	-0.62017	0.73243	KDM1A	76353	25kb
ENSG00000169641	1	23385516	23529301	285	20	120000	-1.2465	0.89371	LUZP1	76353	25kb
ENSG00000179546	1	23491993	23546222	182	14	120000	-0.97136	0.83432	HTR1D	76353	25kb
ENSG00000125944	1	23605264	23695829	116	14	120000	-0.30743	0.62074	HNRNPR	76353	25kb
ENSG00000125945	1	23660941	23720935	126	17	120000	0.34055	0.36672	ZNF436	76353	25kb
ENSG00000249087	1	23670490	23723332	122	17	120000	0.28452	0.38801	C1orf213	76353	25kb
ENSG00000204219	1	23682554	23776233	223	31	120000	-0.98459	0.83759	TCEA3	76353	25kb
ENSG00000088280	1	23730056	23836061	261	17	120000	-2.2474	0.98769	ASAP3	76353	25kb
ENSG00000007968	1	23807922	23882712	181	25	120000	-2.1226	0.98311	E2F2	76353	25kb
ENSG00000117318	1	23859409	23911285	108	20	120000	-0.10335	0.54116	ID3	76353	25kb
ENSG00000197880	1	23882985	23992058	242	50	120000	0.72572	0.23401	MDS2	76353	25kb
ENSG00000142676	1	23993269	24047915	118	21	120000	-0.58428	0.72049	RPL11	76353	25kb
ENSG00000011007	1	24044645	24113549	60	13	120000	0.76151	0.22318	TCEB3	76353	25kb
ENSG00000057757	1	24079895	24139722	42	9	120000	-0.57692	0.718	PITHD1	76353	25kb
ENSG00000011009	1	24092460	24147029	37	7	120000	-0.91981	0.82116	LYPLA2	76353	25kb
ENSG00000117308	1	24097089	24152271	42	6	120000	-0.711	0.76146	GALE	76353	25kb
ENSG00000117305	1	24103375	24190110	88	9	120000	-0.73932	0.77014	HMGCL	76353	25kb
ENSG00000179163	1	24146567	24219784	228	15	120000	-1.7246	0.9577	FUCA1	76353	25kb
ENSG00000188822	1	24172016	24310549	320	23	120000	-1.029	0.84826	CNR2	76353	25kb
ENSG00000189266	1	24260599	24314952	14	5	120000	0.60233	0.27348	PNRC2	76353	25kb
ENSG00000188529	1	24266294	24332417	15	5	120000	0.69818	0.24253	SRSF10	76353	25kb
ENSG00000142661	1	24357525	24463665	340	35	120000	-1.4429	0.92547	MYOM3	76353	25kb
ENSG00000142677	1	24421261	24494611	246	36	120000	-0.33565	0.63143	IL22RA1	76353	25kb
ENSG00000185436	1	24455647	24539449	322	46	120000	-0.30775	0.62086	IFNLR1	76353	25kb
ENSG00000158055	1	24620812	24715972	262	32	120000	-0.35112	0.63725	GRHL3	76353	25kb
ENSG00000001460	1	24658489	24768424	303	21	120000	-1.2324	0.89111	STPG1	76353	25kb
ENSG00000001461	1	24717284	24824466	262	22	120000	-1.8449	0.96747	NIPAL3	76353	25kb
ENSG00000117602	1	24804387	24892530	293	28	120000	0.0045882	0.49817	RCAN3	76353	25kb
ENSG00000184454	1	24857602	24960819	399	41	120000	0.12785	0.44913	NCMAP	76353	25kb
ENSG00000133226	1	24933207	25024758	166	20	120000	-1.0907	0.8623	SRRM1	76353	25kb
ENSG00000169504	1	25046848	25195815	252	21	120000	0.67445	0.25001	CLIC4	76353	25kb
ENSG00000020633	1	25201002	25316612	304	37	120000	0.98715	0.16178	RUNX3	76353	25kb
ENSG00000117614	1	25524170	25583993	47	21	120000	1.2067	0.11377	SYF2	76353	25kb
ENSG00000117616	1	25543728	25689704	103	26	120000	1.2637	0.10317	C1orf63	76353	25kb
ENSG00000187010	1	25573884	25681936	68	17	120000	1.6203	0.052582	RHD	76353	25kb
ENSG00000183726	1	25639408	25713852	55	12	120000	0.81964	0.20621	TMEM50A	76353	25kb
ENSG00000188672	1	25663740	25781683	148	9	120000	0.15815	0.43717	RHCE	76353	25kb
ENSG00000204178	1	25732388	25851700	174	7	120000	0.16836	0.43315	TMEM57	76353	25kb
ENSG00000157978	1	25845071	25920377	158	27	120000	-0.3148	0.62354	LDLRAP1	76353	25kb
ENSG00000117643	1	25918959	26137698	358	36	120000	1.1255	0.1302	MAN1C1	76353	25kb
ENSG00000162430	1	26101667	26169713	163	13	120000	-0.37635	0.64667	SEPN1	76353	25kb
ENSG00000255054	1	26112961	26175235	153	9	120000	-0.49493	0.68967	RP1-317E23.6	76353	25kb
ENSG00000117640	1	26120131	26184432	159	7	120000	-0.4931	0.68903	MTFR1L	76353	25kb
ENSG00000223474	1	26120212	26172288	133	8	120000	-0.45972	0.67714	AL020996.1	76353	25kb
ENSG00000127423	1	26133414	26210903	152	9	120000	-0.41566	0.66117	AUNIP	76353	25kb
ENSG00000182749	1	26162701	26222744	107	9	120000	-0.23217	0.5918	PAQR7	76353	25kb
ENSG00000117632	1	26185672	26258482	88	11	120000	-0.033423	0.51333	STMN1	76353	25kb
ENSG00000158006	1	26261258	26349648	219	20	120000	-0.92117	0.82152	PAFAH2	76353	25kb
ENSG00000158008	1	26321375	26387955	131	16	120000	-0.97016	0.83402	EXTL1	76353	25kb
ENSG00000158014	1	26338743	26397624	123	14	120000	-0.8701	0.80788	SLC30A2	76353	25kb
ENSG00000158022	1	26352795	26419927	144	13	120000	-1.1643	0.87786	TRIM63	76353	25kb
ENSG00000175087	1	26412664	26477034	118	4	120000	-0.69981	0.75798	PDIK1L	76353	25kb
ENSG00000197245	1	26460511	26514119	127	19	120000	-0.50371	0.69277	FAM110D	76353	25kb
ENSG00000142684	1	26471362	26523551	151	21	120000	-0.65127	0.74256	ZNF593	76353	25kb
ENSG00000142675	1	26478894	26541377	193	26	120000	-0.85005	0.80235	CNKSR1	76353	25kb
ENSG00000188782	1	26492052	26554459	214	27	120000	-0.88921	0.81305	CATSPER4	76353	25kb
ENSG00000130695	1	26535691	26630299	323	15	120000	-0.43861	0.66953	CEP85	76353	25kb
ENSG00000142669	1	26580667	26633007	165	14	120000	-0.35342	0.63811	SH3BGRL3	76353	25kb
ENSG00000158062	1	26582819	26669854	268	15	120000	-0.63389	0.73692	UBXN11	76353	25kb
ENSG00000169442	1	26619448	26672014	158	17	120000	-1.2276	0.8902	CD52	76353	25kb
ENSG00000176092	1	26623350	26705621	272	23	120000	-2.0329	0.97897	AIM1L	76353	25kb
ENSG00000176083	1	26663125	26726013	220	32	120000	-2.7561	0.99708	ZNF683	76353	25kb
ENSG00000131914	1	26712269	26781213	155	28	120000	-0.91413	0.81968	LIN28A	76353	25kb
ENSG00000117682	1	26733773	26822785	162	27	120000	0.27761	0.39066	DHDDS	76353	25kb
ENSG00000198830	1	26773941	26827463	99	17	120000	0.67635	0.24941	HMGN2	76353	25kb
ENSG00000117676	1	26831252	26926521	196	28	120000	0.67568	0.24962	RPS6KA1	76353	25kb
ENSG00000117713	1	26997524	27133595	176	12	120000	0.12786	0.44913	ARID1A	76353	25kb
ENSG00000060642	1	27088963	27149889	60	9	120000	0.7453	0.22805	PIGV	76353	25kb
ENSG00000204160	1	27128201	27209093	100	11	120000	0.91342	0.18051	ZDHHC18	76353	25kb
ENSG00000175793	1	27164633	27215947	71	8	120000	0.62344	0.2665	SFN	76353	25kb
ENSG00000142751	1	27177624	27241788	90	12	120000	0.55094	0.29084	GPN2	76353	25kb
ENSG00000198746	1	27191979	27251957	88	11	120000	0.58108	0.28059	GPATCH3	76353	25kb
ENSG00000090273	1	27201729	27298353	133	15	120000	0.36091	0.35908	NUDC	76353	25kb
ENSG00000131910	1	27212980	27265457	78	11	120000	0.26116	0.39698	NR0B2	76353	25kb
ENSG00000175707	1	27251053	27311897	70	10	120000	-0.0032222	0.50129	C1orf172	76353	25kb
ENSG00000253368	1	27295198	27352389	55	10	120000	-0.51702	0.69743	TRNP1	76353	25kb
ENSG00000158246	1	27306511	27364327	73	12	120000	0.91933	0.17896	FAM46B	76353	25kb
ENSG00000090020	1	27400306	27518472	192	10	120000	0.11456	0.4544	SLC9A1	76353	25kb
ENSG00000142784	1	27536007	27660110	190	20	120000	0.12749	0.44928	WDTC1	76353	25kb
ENSG00000186501	1	27623651	27687891	122	21	120000	0.0031399	0.49875	TMEM222	76353	25kb
ENSG00000142765	1	27643513	27705421	122	25	120000	0.35597	0.36093	SYTL1	76353	25kb
ENSG00000142733	1	27656675	27718383	118	25	120000	0.67947	0.24842	MAP3K6	76353	25kb
ENSG00000142748	1	27670603	27726315	91	21	120000	0.90959	0.18152	FCN3	76353	25kb
ENSG00000174950	1	27680666	27734870	84	19	120000	1.4306	0.076266	CD164L2	76353	25kb

ENSG00000181773	1	27694148	27747318	64	11	120000	1.5751	0.057616	GPR3	76353	25kb
ENSG00000158195	1	27705730	27841669	198	11	120000	1.4242	0.077192	WASF2	76353	25kb
ENSG00000126705	1	27835546	27955942	149	21	120000	0.44578	0.32788	AHDC1	76353	25kb
ENSG00000000938	1	27913575	27986788	120	20	120000	1.0279	0.15199	FGR	76353	25kb
ENSG00000126709	1	27967572	28023729	121	14	120000	1.4937	0.067621	IFI6	76353	25kb
ENSG00000009780	1	28027490	28114633	145	8	120000	-0.15616	0.56205	FAM76A	76353	25kb
ENSG00000117758	1	28074694	28175963	120	9	120000	-0.71305	0.76209	STX12	76353	25kb
ENSG00000117751	1	28132289	28203187	94	14	120000	-1.0078	0.84324	PPP1R8	76353	25kb
ENSG00000268552	1	28152085	28202255	76	13	120000	-1.0269	0.84776	AL109927.1	76353	25kb
ENSG00000130775	1	28174055	28238196	144	17	120000	-0.40885	0.65868	THEMIS2	76353	25kb
ENSG00000117748	1	28193035	28266257	237	20	120000	-0.046593	0.51858	RPA2	76353	25kb
ENSG00000130768	1	28236504	28310668	208	21	120000	0.11761	0.45319	SMPDL3B	76353	25kb
ENSG00000158156	1	28260973	28319607	135	25	120000	-0.093579	0.53728	XKR8	76353	25kb
ENSG00000158161	1	28271855	28440207	327	30	120000	0.31318	0.37707	EYA3	76353	25kb
ENSG00000169403	1	28448677	28545447	149	23	120000	-1.357	0.91261	PTAFR	76353	25kb
ENSG00000126698	1	28500967	28584536	216	15	120000	-1.2301	0.89066	DNAJC8	76353	25kb
ENSG00000215902	1	28502013	28552152	113	12	120000	-1.2258	0.88985	AL353354.1	76353	25kb
ENSG00000269804	1	28502070	28552419	113	12	120000	-1.2258	0.88985	AL353354.2	76353	25kb
ENSG00000130770	1	28537620	28598417	178	14	120000	-1.2258	0.88986	ATPIF1	76353	25kb
ENSG00000130766	1	28561038	28634002	174	17	120000	-0.81564	0.79265	SESN2	76353	25kb
ENSG00000130772	1	28630513	28687476	109	14	120000	-0.46946	0.68063	MED18	76353	25kb
ENSG00000204138	1	28671114	28851881	502	13	120000	-0.32762	0.6284	PHACTR4	76353	25kb
ENSG00000180198	1	28807455	28890812	183	14	120000	-0.18598	0.57377	RCC1	76353	25kb
ENSG00000180098	1	28854597	28930051	113	15	120000	-0.70589	0.75987	TRNAU1AP	76353	25kb
ENSG00000120656	1	28890835	28994597	130	17	120000	0.11487	0.45427	TAF12	76353	25kb
ENSG00000188060	1	28893712	28946955	59	12	120000	-0.10551	0.54201	RAB42	76353	25kb
ENSG00000162419	1	28970244	29070865	149	17	120000	2.1947	0.014091	GMEB1	76353	25kb
ENSG00000198492	1	29038133	29121287	126	16	120000	2.3381	0.0096908	YTHDF2	76353	25kb
ENSG00000116329	1	29113654	29215208	300	32	120000	-0.29627	0.61649	OPRD1	76353	25kb
ENSG00000159023	1	29188603	29471553	584	31	120000	-0.45009	0.67368	EPB41	76353	25kb
ENSG00000253304	1	29420940	29475447	121	19	120000	-0.48108	0.68477	TMEM200B	76353	25kb
ENSG00000116350	1	29449255	29533499	185	25	120000	0.012407	0.49505	SRSF4	76353	25kb
ENSG00000116353	1	29494385	29582454	224	26	120000	0.42481	0.33549	MECR	76353	25kb
ENSG00000060656	1	29538028	29678325	348	39	120000	0.86979	0.19221	PTPRU	76353	25kb
ENSG00000162510	1	31159124	31221434	219	24	120000	0.4622	0.32197	MATN1	76353	25kb
ENSG00000162511	1	31180316	31255667	230	35	120000	-0.021777	0.50869	LAPTM5	76353	25kb
ENSG00000162512	1	31317314	31406608	244	58	120000	-1.7368	0.95879	SDC3	76353	25kb
ENSG00000134644	1	31379353	31563838	377	22	120000	0.19512	0.42265	PUM1	76353	25kb
ENSG00000084628	1	31627592	31737401	339	22	120000	3.0919	0.00099435	NKAIN1	76353	25kb
ENSG00000060688	1	31707417	31794662	228	13	120000	2.015	0.021955	SNRNP40	76353	25kb
ENSG00000121766	1	31744842	31862783	297	12	120000	2.1435	0.016036	ZCCHC17	76353	25kb
ENSG00000121769	1	31813472	31874697	159	11	120000	2.3491	0.0094085	FABP3	76353	25kb
ENSG00000168528	1	31857412	31932525	257	15	120000	2.2466	0.012331	SERINC2	76353	25kb
ENSG00000268950	1	31877192	31927614	176	13	120000	1.57	0.058209	AC114494.1	76353	25kb
ENSG00000142910	1	32017116	32078288	125	27	120000	-0.70035	0.75815	TINAGL1	76353	25kb
ENSG00000121764	1	32058287	32123119	118	11	120000	-0.96636	0.83307	HCRTR1	76353	25kb
ENSG00000162517	1	32070463	32135497	136	12	120000	-0.97531	0.8353	PEF1	76353	25kb
ENSG00000084636	1	32092848	32194920	171	25	120000	0.20519	0.41871	COL16A1	76353	25kb
ENSG00000121753	1	32167718	32255494	100	17	120000	1.4307	0.076258	BAI2	76353	25kb
ENSG00000134668	1	32231023	32306652	131	22	120000	0.9993	0.15883	SPOCD1	76353	25kb
ENSG00000184007	1	32347022	32435457	142	24	120000	0.26858	0.39413	PTP4A2	76353	25kb
ENSG00000212673	1	32354174	32405745	71	14	120000	0.63594	0.26241	AL136115.1	76353	25kb
ENSG00000121774	1	32454430	32551451	86	15	120000	-0.74919	0.77313	KHDRBS1	76353	25kb
ENSG00000121775	1	32512632	32593467	89	13	120000	-0.45678	0.67608	TMEM39B	76353	25kb
ENSG00000025800	1	32548639	32667169	136	12	120000	-0.24058	0.59506	KPNA6	76353	25kb
ENSG00000084652	1	32620287	32688886	83	9	120000	-0.74119	0.77071	TXLNA	76353	25kb
ENSG00000160050	1	32640987	32695988	71	10	120000	-0.64966	0.74204	CCDC28B	76353	25kb
ENSG00000160051	1	32646236	32699288	72	10	120000	-0.76483	0.77781	IQCC	76353	25kb
ENSG00000222046	1	32649695	32706797	84	9	120000	-0.78197	0.78288	DCDC2B	76353	25kb
ENSG00000160055	1	32655073	32712972	89	9	120000	-0.6353	0.73738	TMEM234	76353	25kb
ENSG00000084623	1	32662529	32722205	90	10	120000	-0.64247	0.73972	EIF3I	76353	25kb
ENSG00000183615	1	32687834	32739457	75	12	120000	-0.08412	0.53352	FAM167B	76353	25kb
ENSG00000182866	1	32691840	32776766	99	20	120000	-0.036129	0.51441	LCK	76353	25kb
ENSG00000116478	1	32732687	32824236	57	29	120000	1.0968	0.13637	HDAC1	76353	25kb
ENSG00000175130	1	32774433	32826980	23	16	120000	1.6581	0.048652	MARCKSL1	76353	25kb
ENSG00000162526	1	32792122	32854913	34	20	120000	1.2565	0.10447	TSSK3	76353	25kb
ENSG00000225828	1	32801871	32854879	31	19	120000	1.0572	0.14522	FAM229A	76353	25kb
ENSG00000160058	1	32805704	32885332	51	24	120000	0.73884	0.23	BSDC1	76353	25kb
ENSG00000254553	1	32905658	33091393	379	20	120000	-0.5331	0.70302	RP1-27O5.3	76353	25kb
ENSG00000273274	1	32905670	32987287	142	15	120000	-0.19645	0.57787	ZBTB8B	76353	25kb
ENSG00000160062	1	32980028	33096540	270	15	120000	-0.55582	0.71083	ZBTB8A	76353	25kb
ENSG00000176261	1	33040773	33141504	219	13	120000	-0.23663	0.59353	ZBTB8OS	76353	25kb
ENSG00000162521	1	33091743	33176812	185	7	120000	0.006165	0.49754	RBBP4	76353	25kb
ENSG00000162520	1	33120507	33194197	160	9	120000	-0.046747	0.51864	SYNC	76353	25kb
ENSG00000162522	1	33182486	33265571	141	22	120000	0.28382	0.38827	KIAA1522	76353	25kb
ENSG00000134684	1	33215840	33308754	141	15	120000	-0.27665	0.60897	YARS	76353	25kb
ENSG00000116497	1	33257368	33349476	132	16	120000	-0.51354	0.69621	S100PBP	76353	25kb
ENSG00000160097	1	33302869	33363083	86	17	120000	-0.75398	0.77457	FNDC5	76353	25kb
ENSG00000121905	1	33326595	33389042	77	16	120000	-0.44059	0.67024	HPCA	76353	25kb
ENSG00000121900	1	33335195	33392039	73	15	120000	-0.37809	0.64732	TMEM54	76353	25kb
ENSG00000116514	1	33377046	33455286	129	14	120000	-0.64437	0.74033	RNF19B	76353	25kb
ENSG00000267885	1	33414268	33464642	84	13	120000	-0.88309	0.81141	FKSG48	76353	25kb
ENSG00000004455	1	33448585	33571597	188	17	120000	-0.055391	0.52209	AK2	76353	25kb
ENSG00000142920	1	33521705	33611131	129	15	120000	-0.071653	0.52856	ADC	76353	25kb
ENSG00000116525	1	33586003	33672660	150	10	120000	-0.23115	0.5914	TRIM62	76353	25kb
ENSG00000160094	1	33697146	33791320	170	24	120000	0.549	0.2915	ZNF362	76353	25kb
ENSG00000184389	1	33747367	33811699	147	21	120000	1.0922	0.13736	A3GALT2	76353	25kb
ENSG00000134686	1	33764224	33921653	455	25	120000	1.312	0.09476	PHC2	76353	25kb
ENSG00000121903	1	33913246	33987107	158	20	120000	-0.47738	0.68345	ZSCAN20	76353	25kb
ENSG00000121904	1	33954609	34656443	1865	175	120000	-0.94395	0.8274	CSMD2	76353	25kb
ENSG00000176256	1	34301076	34355392	154	20	120000	0.72297	0.23485	HMGB4	76353	25kb
ENSG00000142698	1	34607484	34709732	328	45	120000	0.37985	0.35203	C1orf94	76353	25kb
ENSG00000163866	1	35153338	35350417	702	79	120000	0.88407	0.18833	SMIM12	76353	25kb
ENSG00000189280	1	35195648	35249113	190	40	120000	0.8243	0.20489	GJB5	76353	25kb
ENSG00000189433	1	35200342	35254325	192	39	120000	1.0068	0.15702	GJB4	76353	25kb
ENSG00000188910	1	35221790	35276970	201	33	120000	1.346	0.089156	GJB3	76353	25kb

ENSG00000187513	1	35233599	35286348	189	31	120000	1.4231	0.077357	GJA4	76353	25kb
ENSG00000116544	1	35306037	35420186	200	53	120000	1.2763	0.10092	DLGAP3	76353	25kb
ENSG00000271741	1	35422134	35522342	115	7	120000	1.9005	0.028681	RP11-244H3.4	76353	25kb
ENSG00000243749	1	35422136	35475954	61	5	120000	1.8563	0.031707	ZMYM6NB	76353	25kb
ENSG00000163867	1	35424523	35522569	115	7	120000	1.9005	0.028681	ZMYM6	76353	25kb
ENSG00000197056	1	35500387	35606460	121	12	120000	2.5319	0.0056724	ZMYM1	76353	25kb
ENSG00000116560	1	35616979	35683749	131	15	120000	2.6442	0.004094	SFPQ	76353	25kb
ENSG00000146463	1	35709568	35912659	234	17	120000	-0.5026	0.69238	ZMYM4	76353	25kb
ENSG00000142687	1	35874091	36048551	185	20	120000	0.078716	0.46863	KIAA0319L	76353	25kb
ENSG00000020129	1	35998074	36057875	64	11	120000	0.35607	0.36089	NCDN	76353	25kb
ENSG00000116819	1	36013971	36085929	63	11	120000	0.39953	0.34475	TFAP2E	76353	25kb
ENSG00000126067	1	36042185	36132445	84	16	120000	-0.16204	0.56436	PSMB2	76353	25kb
ENSG00000142686	1	36154476	36210073	90	12	120000	1.2063	0.11385	C1orf216	76353	25kb
ENSG00000092853	1	36160819	36260568	158	17	120000	0.99246	0.16049	CLSPN	76353	25kb
ENSG00000134698	1	36248773	36348491	132	18	120000	1.0209	0.15364	AGO4	76353	25kb
ENSG00000092847	1	36310409	36420211	131	17	120000	1.8052	0.035521	AGO1	76353	25kb
ENSG00000126070	1	36371319	36563101	205	21	120000	1.9762	0.024067	AGO3	76353	25kb
ENSG00000092850	1	36524676	36578876	79	14	120000	1.7316	0.041676	TEKT2	76353	25kb
ENSG00000116863	1	36529476	36584533	89	14	120000	1.6363	0.050892	ADPRHL2	76353	25kb
ENSG00000171812	1	36535837	36615821	132	18	120000	1.623	0.052298	COL8A2	76353	25kb
ENSG00000054116	1	36577173	36640098	100	14	120000	1.0366	0.14996	TRAPPC3	76353	25kb
ENSG00000116871	1	36596180	36671450	110	18	120000	0.40004	0.34457	MAP7D1	76353	25kb
ENSG00000054118	1	36665017	36795958	161	16	120000	0.97263	0.16537	THRAP3	76353	25kb
ENSG00000214193	1	36746988	36815484	86	12	120000	0.9382	0.17407	SH3D21	76353	25kb
ENSG00000142694	1	36762632	36814755	69	11	120000	0.96696	0.16678	EVA1B	76353	25kb
ENSG00000116883	1	36764335	36819822	76	11	120000	0.87013	0.19211	RP11-268J15.5	76353	25kb
ENSG00000196182	1	36780225	36876497	205	25	120000	0.88405	0.18834	STK40	76353	25kb
ENSG00000181817	1	36831839	36888493	186	19	120000	0.70252	0.24118	LSM10	76353	25kb
ENSG00000116885	1	36856428	36941086	353	15	120000	0.20336	0.41943	OSCP1	76353	25kb
ENSG00000116898	1	36896319	36955038	195	17	120000	0.10211	0.45933	MRPS15	76353	25kb
ENSG00000119535	1	36906644	36973879	219	29	120000	-0.096713	0.53852	CSF3R	76353	25kb
ENSG00000163873	1	37236128	37524730	611	60	120000	-0.07759	0.53092	GRIK3	76353	25kb
ENSG00000163874	1	37915153	37974980	144	24	120000	-0.51067	0.69521	ZC3H12A	76353	25kb
ENSG00000163875	1	37933176	38005375	156	24	120000	-0.37263	0.64529	MEAF6	76353	25kb
ENSG00000163877	1	37975050	38044903	166	21	120000	-0.31911	0.62518	SNIP1	76353	25kb
ENSG00000163879	1	37997520	38057458	144	19	120000	-0.25833	0.60192	DNALI1	76353	25kb
ENSG00000134697	1	38007417	38086536	184	22	120000	-0.4018	0.65608	GNL2	76353	25kb
ENSG00000169218	1	38051951	38125595	179	31	120000	0.60503	0.27258	RSPO1	76353	25kb
ENSG00000116922	1	38122242	38182914	165	25	120000	1.2966	0.097389	C1orf109	76353	25kb
ENSG00000134690	1	38133090	38200391	212	24	120000	0.84201	0.19989	CDCA8	76353	25kb
ENSG00000183317	1	38154552	38255805	338	32	120000	-0.28116	0.61071	EPHA10	76353	25kb
ENSG00000185090	1	38234474	38291809	124	20	120000	0.24637	0.4027	MANEAL	76353	25kb
ENSG00000196449	1	38243616	38298857	107	18	120000	0.098893	0.46061	YRDC	76353	25kb
ENSG00000197982	1	38247651	38300126	97	17	120000	0.097139	0.46131	C1orf122	76353	25kb
ENSG00000188786	1	38250239	38350292	173	23	120000	-0.18824	0.57466	MTF1	76353	25kb
ENSG00000230610	1	38267232	38318886	89	17	120000	0.04072	0.48376	AL929472.1	76353	25kb
ENSG00000204084	1	38301369	38437729	256	21	120000	-0.36129	0.64106	INPP5B	76353	25kb
ENSG00000183431	1	38397647	38481593	196	24	120000	0.21437	0.41513	SF3A3	76353	25kb
ENSG00000183386	1	38437442	38496278	119	27	120000	0.82767	0.20393	FHL3	76353	25kb
ENSG00000183520	1	38449930	38515496	119	31	120000	1.4091	0.079407	UTP11L	76353	25kb
ENSG00000185668	1	38484523	38537450	111	17	120000	1.432	0.076076	POU3F1	76353	25kb
ENSG00000116954	1	39278870	39350495	180	29	120000	-1.7653	0.96124	RRAGC	76353	25kb
ENSG00000214114	1	39303636	39372289	181	19	120000	-1.3227	0.90703	MYCBP	76353	25kb
ENSG00000131233	1	39305175	39372289	177	19	120000	-1.2996	0.90313	GJA9	76353	25kb
ENSG00000158315	1	39326479	39432471	314	33	120000	0.17904	0.42896	RHBDL2	76353	25kb
ENSG00000174574	1	39431895	39496731	202	20	120000	0.47788	0.31637	AKIRIN1	76353	25kb
ENSG00000168653	1	39466990	39525308	191	15	120000	0.62381	0.26638	NDUFS5	76353	25kb
ENSG00000127603	1	39521988	39977849	901	44	120000	-0.74252	0.77112	MACF1	76353	25kb
ENSG00000255103	1	39851151	39907154	78	14	120000	-1.0504	0.85324	KIAA0754	76353	25kb
ENSG00000183682	1	39932318	40016607	180	28	120000	-2.1214	0.98306	BMP8A	76353	25kb
ENSG00000090621	1	40001488	40067462	153	20	120000	-1.7964	0.96378	PABPC4	76353	25kb
ENSG00000163909	1	40064825	40130617	182	36	120000	-1.0445	0.85187	HEYL	76353	25kb
ENSG00000116981	1	40099793	40162710	193	31	120000	-0.32588	0.62774	NT5C1A	76353	25kb
ENSG00000116983	1	40119320	40182361	192	35	120000	-0.25998	0.60256	HPCAL4	76353	25kb
ENSG00000084072	1	40132854	40254586	383	54	120000	-0.3739	0.64576	PPIE	76353	25kb
ENSG00000116985	1	40197854	40279533	273	25	120000	0.69984	0.24201	BMP8B	76353	25kb
ENSG00000198754	1	40210195	40262020	180	23	120000	0.76603	0.22183	OXCT2	76353	25kb
ENSG00000043514	1	40281723	40374183	263	22	120000	1.152	0.12466	TRIT1	76353	25kb
ENSG00000116990	1	40336098	40392928	148	24	120000	1.6834	0.046148	MYCL	76353	25kb
ENSG00000168389	1	40395802	40460638	131	27	120000	0.97467	0.16486	MFSD2A	76353	25kb
ENSG00000131236	1	40480905	40563321	264	13	120000	-0.49038	0.68807	CAP1	76353	25kb
ENSG00000131238	1	40513379	40588375	227	19	120000	-0.25622	0.60111	PPT1	76353	25kb
ENSG00000117000	1	40602045	40731593	156	26	120000	0.26044	0.39726	RLF	76353	25kb
ENSG00000188800	1	40686619	40742363	75	18	120000	-0.28003	0.61027	TMCO2	76353	25kb
ENSG00000084073	1	40698779	40784856	153	35	120000	1.1416	0.12681	ZMPSTE24	76353	25kb
ENSG00000049089	1	40741159	40808488	189	22	120000	0.34387	0.36547	COL9A2	76353	25kb
ENSG00000084070	1	40785522	40913998	363	46	120000	-1.3271	0.90777	SMAP2	76353	25kb
ENSG00000187801	1	40890774	40954390	220	38	120000	-0.30139	0.61844	ZFP69B	76353	25kb
ENSG00000187815	1	40917887	40987015	207	32	120000	-0.81335	0.79199	ZFP69	76353	25kb
ENSG00000164002	1	40949413	41007228	165	17	120000	-0.56288	0.71324	EXO5	76353	25kb
ENSG00000117010	1	40972233	41038841	250	19	120000	-0.71543	0.76283	ZNF684	76353	25kb
ENSG00000117016	1	41061351	41156329	247	34	120000	-0.69943	0.75786	RIMS3	76353	25kb
ENSG00000066136	1	41132320	41262275	313	24	120000	0.24717	0.40239	NFYC	76353	25kb
ENSG00000117013	1	41224684	41331124	364	69	120000	0.25705	0.39857	KCNQ4	76353	25kb
ENSG00000179862	1	41301729	41353018	208	39	120000	0.63751	0.2619	CITED4	76353	25kb
ENSG00000171793	1	41420007	41503235	271	29	120000	-0.18555	0.5736	CTPS1	76353	25kb
ENSG00000171790	1	41456269	41513909	164	21	120000	0.30606	0.37978	SLFNL1	76353	25kb
ENSG00000010803	1	41467872	41732826	635	25	120000	0.6844	0.24686	SCMH1	76353	25kb
ENSG00000204060	1	41802594	41874262	217	41	120000	-0.23825	0.59415	FOXO6	76353	25kb
ENSG00000127129	1	41919446	41975344	197	32	120000	0.79208	0.21416	EDN2	76353	25kb
ENSG00000127124	1	41947036	42526596	1504	78	120000	0.86054	0.19475	HIVEP3	76353	25kb
ENSG00000044012	1	42594092	42646495	96	17	120000	1.2513	0.10541	GUCA2B	76353	25kb
ENSG00000197273	1	42603362	42655389	106	18	120000	1.1353	0.12813	GUCA2A	76353	25kb
ENSG00000198815	1	42617210	42826548	375	20	120000	0.52624	0.29936	FOXJ3	76353	25kb
ENSG00000177181	1	42821466	42913249	266	18	120000	-0.17689	0.5702	RIMKLA	76353	25kb
ENSG00000066185	1	42871000	42946938	206	18	120000	1.2372	0.10802	ZMYND12	76353	25kb

ENSG00000127125	1	42896788	42964056	171	13	120000	1.7381	0.041098	PPCS	76353	25kb
ENSG00000186409	1	42904001	43145335	689	24	120000	1.3987	0.080954	CCDC30	76353	25kb
ENSG00000171960	1	43099096	43167429	141	20	120000	0.99318	0.16031	PPIH	76353	25kb
ENSG00000065978	1	43123098	43193020	122	18	120000	0.7263	0.23383	YBX1	76353	25kb
ENSG00000164007	1	43173764	43230925	110	20	120000	0.052608	0.47902	CLDN19	76353	25kb
ENSG00000117385	1	43187006	43257755	128	19	120000	0.078039	0.4689	LEPRE1	76353	25kb
ENSG00000164008	1	43207940	43288968	160	22	120000	0.45062	0.32613	C1orf50	76353	25kb
ENSG00000177868	1	43247723	43307954	116	18	120000	0.77538	0.21906	CCDC23	76353	25kb
ENSG00000164010	1	43257795	43335660	147	26	120000	0.9941	0.16009	ERMAP	76353	25kb
ENSG00000164011	1	43287280	43343148	93	20	120000	0.60721	0.27186	ZNF691	76353	25kb
ENSG00000117394	1	43366052	43449530	275	25	120000	2.0309	0.021133	SLC2A1	76353	25kb
ENSG00000186973	1	43585824	43647067	269	19	120000	0.12967	0.44841	FAM183A	76353	25kb
ENSG00000117395	1	43604846	43761607	551	38	120000	-0.48933	0.68769	EBNA1BP2	76353	25kb
ENSG00000243710	1	43612820	43745029	475	38	120000	-0.42251	0.66367	WDR65	76353	25kb
ENSG00000179178	1	43710665	43764673	125	23	120000	-0.75347	0.77442	TMEM125	76353	25kb
ENSG00000253313	1	43722554	43776288	124	25	120000	-0.95699	0.83071	C1orf210	76353	25kb
ENSG00000066056	1	43741664	43813779	103	17	120000	-0.68444	0.75315	TIE1	76353	25kb
ENSG00000117400	1	43778478	43843443	70	10	120000	0.11118	0.45574	MPL	76353	25kb
ENSG00000117399	1	43799626	43853874	69	11	120000	0.10011	0.46013	CDC20	76353	25kb
ENSG00000066322	1	43804068	43858696	73	11	120000	-0.11554	0.54599	ELOVL1	76353	25kb
ENSG00000159479	1	43824588	43880479	92	13	120000	-0.11017	0.54386	MED8	76353	25kb
ENSG00000198198	1	43830553	43943321	166	16	120000	-0.21597	0.58549	SZT2	76353	25kb
ENSG00000178922	1	43891824	43944660	67	10	120000	-0.29413	0.61567	HYI	76353	25kb
ENSG00000142949	1	43965858	44114343	362	20	120000	1.2466	0.10628	PTPRF	76353	25kb
ENSG00000066135	1	44090829	44196186	189	19	120000	1.1082	0.13388	KDM4A	76353	25kb
ENSG00000126091	1	44146495	44421831	578	26	120000	0.81265	0.20821	ST3GAL3	76353	25kb
ENSG00000117407	1	44373992	44427913	113	14	120000	0.46755	0.32005	ARTN	76353	25kb
ENSG00000117408	1	44387611	44458694	130	14	120000	0.36828	0.35633	IPO13	76353	25kb
ENSG00000132768	1	44410672	44464041	84	12	120000	0.27689	0.39093	DPH2	76353	25kb
ENSG00000117410	1	44415159	44468967	87	14	120000	0.19477	0.42279	ATP6V0B	76353	25kb
ENSG00000117411	1	44419615	44481840	105	16	120000	-0.012127	0.50484	B4GALT2	76353	25kb
ENSG00000159214	1	44432031	44487200	93	16	120000	0.025675	0.48976	CCDC24	76353	25kb
ENSG00000196517	1	44432172	44522139	175	35	120000	-0.62411	0.73372	SLC6A9	76353	25kb
ENSG00000171872	1	44559522	44625812	221	26	120000	0.57738	0.28184	KLF17	76353	25kb
ENSG00000178028	1	44654127	44711353	78	18	120000	1.1372	0.12773	DMAP1	76353	25kb
ENSG00000117419	1	44661742	44845932	285	20	120000	0.8971	0.18483	ERI3	76353	25kb
ENSG00000187147	1	44845866	45142396	779	89	120000	-1.8231	0.96586	RNF220	76353	25kb
ENSG00000126106	1	45075910	45165227	161	16	120000	-0.62569	0.73424	TMEM53	76353	25kb
ENSG00000198520	1	45115364	45216263	194	36	120000	0.24534	0.4031	C1orf228	76353	25kb
ENSG00000142945	1	45180490	45258439	161	19	120000	0.38572	0.34985	KIF2C	76353	25kb
ENSG00000142937	1	45215923	45269451	92	11	120000	0.44804	0.32706	RPS8	76353	25kb
ENSG00000142959	1	45224257	45278377	93	12	120000	0.48398	0.3142	BEST4	76353	25kb
ENSG00000173846	1	45240897	45296662	92	11	120000	0.82963	0.20337	PLK3	76353	25kb
ENSG00000188396	1	45246585	45297957	83	10	120000	0.93183	0.17571	TCTEX1D4	76353	25kb
ENSG00000222009	1	45249154	45306257	104	11	120000	0.95114	0.17077	BTBD19	76353	25kb
ENSG00000117425	1	45260516	45333735	148	12	120000	0.87051	0.19201	PTCH2	76353	25kb
ENSG00000070785	1	45291450	45477282	453	18	120000	0.94271	0.17292	EIF2B3	76353	25kb
ENSG00000126107	1	45443212	45502001	142	10	120000	0.28482	0.38789	HECTD3	76353	25kb
ENSG00000126088	1	45452819	45506247	124	12	120000	0.23105	0.40864	UROD	76353	25kb
ENSG00000162415	1	45457071	45796881	616	28	120000	0.78755	0.21548	ZSWIM5	76353	25kb
ENSG00000186603	1	45767545	45819347	58	16	120000	1.2671	0.10256	HPDL	76353	25kb
ENSG00000132781	1	45769835	45831142	73	15	120000	1.4918	0.067876	MUTYH	76353	25kb
ENSG00000132773	1	45780342	45834647	65	12	120000	1.6496	0.049509	TOE1	76353	25kb
ENSG00000070759	1	45784555	45981872	305	19	120000	1.5282	0.063225	TESK2	76353	25kb
ENSG00000236624	1	45934538	45990751	113	10	120000	0.49457	0.31045	CCDC163P	76353	25kb
ENSG00000132763	1	45940725	46001739	127	10	120000	0.2164	0.41434	MMACHC	76353	25kb
ENSG00000117450	1	45951708	46013719	134	10	120000	0.49238	0.31123	PRDX1	76353	25kb
ENSG00000117448	1	45991215	46060721	125	14	120000	0.24417	0.40355	AKR1A1	76353	25kb
ENSG00000132780	1	46024518	46109566	131	9	120000	-0.39588	0.6539	NASP	76353	25kb
ENSG00000159588	1	46060716	46114729	99	6	120000	-0.48531	0.68627	CCDC17	76353	25kb
ENSG00000159592	1	46067976	46178785	213	9	120000	-0.481	0.68474	GPBP1L1	76353	25kb
ENSG00000159596	1	46127886	46185115	103	6	120000	-0.41607	0.66132	TMEM69	76353	25kb
ENSG00000197429	1	46134996	46241322	188	8	120000	-0.49543	0.68985	IPP	76353	25kb
ENSG00000086015	1	46227659	46526796	586	13	120000	-0.73823	0.76981	MAST2	76353	25kb
ENSG00000117461	1	46480812	46667160	293	20	120000	-1.0822	0.86042	PIK3R3	76353	25kb
ENSG00000117472	1	46615745	46676630	77	14	120000	-0.97863	0.83612	TSPAN1	76353	25kb
ENSG00000085998	1	46629354	46710977	100	18	120000	-0.605	0.72741	POMGNT1	76353	25kb
ENSG00000171357	1	46644006	46711933	76	13	120000	-0.2381	0.5941	LURAP1	76353	25kb
ENSG00000085999	1	46688360	46769145	81	16	120000	0.022601	0.49098	RAD54L	76353	25kb
ENSG00000132128	1	46701868	46794280	99	19	120000	0.038957	0.48446	LRRC41	76353	25kb
ENSG00000173660	1	46744303	46807448	84	18	120000	-0.28682	0.61288	UQCRH	76353	25kb
ENSG00000117481	1	46780849	46855824	270	7	120000	-1.0073	0.8431	NSUN4	76353	25kb
ENSG00000117480	1	46834937	46904520	166	18	120000	-1.0445	0.85187	FAAH	76353	25kb
ENSG00000197587	1	46947668	47004898	151	23	120000	-0.082811	0.533	DMBX1	76353	25kb
ENSG00000162456	1	46986316	47042199	136	21	120000	0.25648	0.39879	KNCN	76353	25kb
ENSG00000079277	1	46998090	47107515	250	32	120000	-0.43346	0.66766	MKNK1	76353	25kb
ENSG00000142961	1	47048387	47107563	136	18	120000	-0.8357	0.79834	MOB3C	76353	25kb
ENSG00000123472	1	47073409	47164539	176	13	120000	-0.49966	0.69134	ATPAF1	76353	25kb
ENSG00000159658	1	47099366	47209824	239	12	120000	-0.29358	0.61546	EFCAB14	76353	25kb
ENSG00000186118	1	47109527	47164266	93	6	120000	-0.18033	0.57155	TEX38	76353	25kb
ENSG00000142973	1	47198510	47310085	280	39	120000	-1.1118	0.86688	CYP4B1	76353	25kb
ENSG00000187048	1	47369849	47432156	133	13	120000	-0.40071	0.65568	CYP4A11	76353	25kb
ENSG00000186377	1	47402036	47541423	257	20	120000	1.3646	0.08619	CYP4X1	76353	25kb
ENSG00000186160	1	47508160	47608991	231	11	120000	1.6461	0.049866	CYP4Z1	76353	25kb
ENSG00000162365	1	47578107	47640413	164	13	120000	1.569	0.058319	CYP4A22	76353	25kb
ENSG00000162366	1	47624265	47681716	140	24	120000	-0.014184	0.50566	PDZK1IP1	76353	25kb
ENSG00000162367	1	47656962	47722892	183	23	120000	-0.30322	0.61914	TAL1	76353	25kb
ENSG00000123473	1	47690811	47804819	276	16	120000	-0.70464	0.75948	STIL	76353	25kb
ENSG00000162368	1	47774469	47869511	235	20	120000	-0.087902	0.53502	CMPK1	76353	25kb
ENSG00000186790	1	47856744	47908723	107	14	120000	-0.55082	0.70912	FOXE3	76353	25kb
ENSG00000186564	1	47876689	47931363	117	14	120000	-0.1645	0.56533	FOXD2	76353	25kb
ENSG00000269113	1	48201200	48487567	779	44	120000	0.58371	0.27971	TRABD2B	76353	25kb
ENSG00000273395	1	48201979	48252290	122	26	120000	0.08031	0.468	FLJ00388	76353	25kb
ENSG00000269709	1	48544306	48594878	125	28	120000	-0.5565	0.71106	AL109659.1	76353	25kb
ENSG00000117834	1	48663357	48739316	210	24	120000	-0.11834	0.5471	SLC5A9	76353	25kb
ENSG00000132122	1	48736044	48962845	415	34	120000	-0.5242	0.69993	SPATA6	76353	25kb

ENSG00000186094	1	48973527	50514585	2235	66	120000	0.40867	0.34139	AGBL4	76353	25kb
ENSG00000162373	1	49168195	49267641	200	36	120000	-0.17292	0.56864	BEND5	76353	25kb
ENSG00000227839	1	50434990	50486874	54	13	120000	0.81593	0.20727	AL645730.2	76353	25kb
ENSG00000162374	1	50488686	50694458	261	31	120000	0.0816	0.46748	ELAVL4	76353	25kb
ENSG00000142700	1	50858222	50914172	124	10	120000	-0.42139	0.66327	DMRTA2	76353	25kb
ENSG00000185104	1	50880150	51450935	732	27	120000	-0.87392	0.80892	FAF1	76353	25kb
ENSG00000123080	1	51401417	51465305	78	11	120000	0.077575	0.46908	CDKN2C	76353	25kb
ENSG00000204006	1	51542906	51638752	141	12	120000	1.4742	0.070207	C1orf185	76353	25kb
ENSG00000123091	1	51676943	51764127	98	15	120000	2.2409	0.012518	RNF11	76353	25kb
ENSG00000085831	1	51727930	51835788	200	28	120000	1.465	0.071465	TTC39A	76353	25kb
ENSG00000085832	1	51794935	52010000	612	21	120000	1.071	0.14209	EPS15	76353	25kb
ENSG00000117859	1	52017851	52279889	436	35	120000	-0.56433	0.71374	OSBPL9	76353	25kb
ENSG00000078618	1	52229863	52369477	259	26	120000	-0.27049	0.60661	NRD1	76353	25kb
ENSG00000169213	1	52348628	52481436	268	21	120000	-0.82848	0.7963	RAB3B	76353	25kb
ENSG00000117862	1	52460803	52546843	149	14	120000	-0.50455	0.69306	TXNDC12	76353	25kb
ENSG00000198841	1	52472775	52524488	85	9	120000	-0.5122	0.69574	KTI12	76353	25kb
ENSG00000134717	1	52496797	52581388	130	17	120000	-0.36691	0.64316	BTF3L4	76353	25kb
ENSG00000157077	1	52583046	52837358	269	15	120000	0.60571	0.27235	ZFYVE9	76353	25kb
ENSG00000154222	1	52786395	52856865	71	10	120000	0.61051	0.27076	CC2D1B	76353	25kb
ENSG00000085840	1	52813501	52895131	80	15	120000	0.62521	0.26592	ORC1	76353	25kb
ENSG00000134748	1	52845236	52911511	71	12	120000	0.80257	0.21111	PRPF38A	76353	25kb
ENSG00000134744	1	52848954	53044159	294	18	120000	0.41397	0.33945	ZCCHC11	76353	25kb
ENSG00000116157	1	53043044	53099723	142	22	120000	-0.29741	0.61692	GPX7	76353	25kb
ENSG00000182183	1	53074016	53160355	224	34	120000	-0.71066	0.76135	FAM159A	76353	25kb
ENSG00000162377	1	53127508	53189038	170	15	120000	0.1316	0.44765	COA7	76353	25kb
ENSG00000162378	1	53167126	53318014	393	18	120000	0.91137	0.18105	ZYG11B	76353	25kb
ENSG00000203995	1	53283183	53385670	178	14	120000	0.89257	0.18604	ZYG11A	76353	25kb
ENSG00000121310	1	53336656	53417884	116	15	120000	0.71697	0.2367	ECHDC2	76353	25kb
ENSG00000116171	1	53367901	53542375	576	22	120000	0.044531	0.48224	SCP2	76353	25kb
ENSG00000174348	1	53502854	53576174	386	23	120000	1.1448	0.12615	PODN	76353	25kb
ENSG00000162383	1	53527855	53633289	525	39	120000	0.92422	0.17768	SLC1A7	76353	25kb
ENSG00000157184	1	53637101	53704869	125	20	120000	0.6127	0.27004	CPT2	76353	25kb
ENSG00000162384	1	53654771	53711289	89	12	120000	0.13138	0.44774	C1orf123	76353	25kb
ENSG00000162385	1	53667564	53729282	89	13	120000	0.23008	0.40902	MAGOH	76353	25kb
ENSG00000157193	1	53686217	53818742	306	38	120000	-0.027817	0.5111	LRP8	76353	25kb
ENSG00000143006	1	53900072	53958161	164	19	120000	-1.6615	0.95169	DMRTB1	76353	25kb
ENSG00000174332	1	53946910	54224877	788	53	120000	-0.80741	0.79029	GLIS1	76353	25kb
ENSG00000058804	1	54206133	54329533	230	28	120000	-1.1722	0.87945	NDC1	76353	25kb
ENSG00000058799	1	54292392	54381407	267	20	120000	-0.80281	0.78896	YIPF1	76353	25kb
ENSG00000211452	1	54331912	54401759	216	22	120000	-0.83921	0.79932	DIO1	76353	25kb
ENSG00000081870	1	54357247	54436602	181	21	120000	-1.4088	0.92055	HSPB11	76353	25kb
ENSG00000116212	1	54386750	54458841	150	19	120000	-1.48	0.93056	LRRC42	76353	25kb
ENSG00000203985	1	54449412	54508856	166	17	120000	-0.47925	0.68412	LDLRAD1	76353	25kb
ENSG00000116209	1	54472347	54544177	147	20	120000	0.056962	0.47729	TMEM59	76353	25kb
ENSG00000116205	1	54494260	54603192	235	26	120000	0.12931	0.44856	TCEANC2	76353	25kb
ENSG00000212670	1	54544968	54596750	124	16	120000	0.026138	0.48957	AL161915.1	76353	25kb
ENSG00000157211	1	54573747	54644443	228	39	120000	0.67088	0.25115	CDCP2	76353	25kb
ENSG00000256407	1	54581897	54690746	308	43	120000	-0.057319	0.52285	RP11-446E24.4	76353	25kb
ENSG00000248835	1	54610333	54662997	135	25	120000	1.151	0.12486	AL357673.1	76353	25kb
ENSG00000215883	1	54613009	54690709	199	39	120000	0.37654	0.35326	CYB5RL	76353	25kb
ENSG00000116221	1	54624714	54716137	208	43	120000	-0.4632	0.67839	MRPL37	76353	25kb
ENSG00000157216	1	54666105	54904152	661	92	120000	-0.31577	0.62391	SSBP3	76353	25kb
ENSG00000198711	1	54678710	54729736	139	23	120000	-0.58023	0.71912	SSBP3-AS1	76353	25kb
ENSG00000162390	1	54982930	55129865	527	49	120000	1.2613	0.10361	ACOT11	76353	25kb
ENSG00000162391	1	55049855	55114229	247	33	120000	0.45971	0.32286	FAM151A	76353	25kb
ENSG00000271723	1	55082459	55232981	583	41	120000	0.96237	0.16793	MROH7-TTC4	76353	25kb
ENSG00000184313	1	55082463	55200939	453	44	120000	0.5823	0.28018	MROH7	76353	25kb
ENSG00000243725	1	55156495	55233330	315	15	120000	1.6314	0.051407	TTC4	76353	25kb
ENSG00000162396	1	55197571	55255187	217	24	120000	1.7353	0.041345	PARS2	76353	25kb
ENSG00000006555	1	55220385	55291940	243	34	120000	0.06063	0.47583	TTC22	76353	25kb
ENSG00000162398	1	55246736	55332925	330	20	120000	-0.043016	0.51716	C1orf177	76353	25kb
ENSG00000116133	1	55290306	55377891	319	20	120000	0.58685	0.27865	DHCR24	76353	25kb
ENSG00000143001	1	55421465	55482966	231	27	120000	0.94741	0.17171	TMEM61	76353	25kb
ENSG00000162399	1	55439606	55501556	236	35	120000	0.87071	0.19196	BSND	76353	25kb
ENSG00000169174	1	55480221	55555525	266	46	120000	0.22034	0.4128	PCSK9	76353	25kb
ENSG00000162402	1	55507032	55705786	448	27	120000	-0.25529	0.60075	USP24	76353	25kb
ENSG00000162407	1	56935419	57135974	656	47	120000	-0.33763	0.63218	PPAP2B	76353	25kb
ENSG00000162409	1	57085995	57206008	413	37	120000	0.37071	0.35543	PRKAA2	76353	25kb
ENSG00000187889	1	57159477	57310369	495	82	120000	-0.43883	0.66961	C1orf168	76353	25kb
ENSG00000157131	1	57295479	57408894	394	40	120000	-1.1917	0.8833	C8A	76353	25kb
ENSG00000021852	1	57369883	57456813	382	33	120000	-0.85493	0.8037	C8B	76353	25kb
ENSG00000173406	1	57435451	59037406	4882	284	120000	-0.89508	0.81463	DAB1	76353	25kb
ENSG00000162600	1	58856056	59037474	740	48	120000	0.88371	0.18843	OMA1	76353	25kb
ENSG00000184292	1	59016099	59068166	291	21	120000	0.52576	0.29953	TACSTD2	76353	25kb
ENSG00000162601	1	59095411	59190764	352	17	120000	-0.25084	0.59903	MYSM1	76353	25kb
ENSG00000177606	1	59221465	59274785	96	18	120000	-0.14661	0.55828	JUN	76353	25kb
ENSG00000172456	1	59737310	60258347	1095	92	120000	0.17124	0.43202	FGGY	76353	25kb
ENSG00000134709	1	60255458	60367050	227	24	120000	-0.4377	0.6692	HOOK1	76353	25kb
ENSG00000134716	1	60333980	60417462	169	19	120000	0.19678	0.422	CYP2J2	76353	25kb
ENSG00000162598	1	60427941	60564442	298	37	120000	1.1677	0.12147	C1orf87	76353	25kb
ENSG00000162599	1	61305931	61953465	1596	185	120000	0.098593	0.46073	NFIA	76353	25kb
ENSG00000162604	1	62121718	62216095	224	28	120000	-0.38543	0.65004	TM2D1	76353	25kb
ENSG00000132849	1	62183149	62654592	1436	107	120000	-0.50234	0.69229	INADL	76353	25kb
ENSG00000240563	1	62635503	62703000	388	34	120000	0.23507	0.40708	L1TD1	76353	25kb
ENSG00000132854	1	62677651	62810085	589	37	120000	-0.82824	0.79623	KANK4	76353	25kb
ENSG00000162607	1	62876968	62942475	106	11	120000	-1.0165	0.8453	USP1	76353	25kb
ENSG00000116641	1	62895399	63178969	371	13	120000	-1.1527	0.87548	DOCK7	76353	25kb
ENSG00000132855	1	63038158	63096830	63	6	120000	-0.61129	0.7295	ANGPTL3	76353	25kb
ENSG00000269624	1	63048437	63100249	49	5	120000	-0.67586	0.75044	AL138847.1	76353	25kb
ENSG00000125703	1	63224806	63356184	283	11	120000	-0.11638	0.54632	ATG4C	76353	25kb
ENSG00000187140	1	63763730	63815797	142	42	120000	0.26073	0.39715	FOXD3	76353	25kb
ENSG00000088035	1	63808261	63929233	273	28	120000	-0.63273	0.73655	ALG6	76353	25kb
ENSG00000142856	1	63881441	64084392	512	20	120000	-0.33277	0.63035	ITGB3BP	76353	25kb
ENSG00000203965	1	63964043	64063364	243	20	120000	-0.42744	0.66547	EFCAB7	76353	25kb
ENSG00000116652	1	63989588	64040405	119	13	120000	-0.34755	0.63591	DLEU2L	76353	25kb
ENSG00000079739	1	64033947	64150916	353	44	120000	-0.77151	0.7798	PGM1	76353	25kb

ENSG00000185483	1	64214693	64672181	1176	84	120000	0.67062	0.25123	ROR1	76353	25kb
ENSG00000177414	1	64644310	64758051	302	22	120000	-0.25253	0.59968	UBE2U	76353	25kb
ENSG00000158966	1	64911428	65183741	624	87	120000	0.13076	0.44798	CACHD1	76353	25kb
ENSG00000162437	1	65185778	65323915	290	24	120000	-0.017069	0.50681	RAVER2	76353	25kb
ENSG00000162434	1	65273912	65457187	466	38	120000	-0.55217	0.70958	JAK1	76353	25kb
ENSG00000162433	1	65588232	65722828	350	35	120000	-0.92803	0.8233	AK4	76353	25kb
ENSG00000116675	1	65688902	65906552	603	43	120000	1.4767	0.069883	DNAJC6	76353	25kb
ENSG00000116678	1	65861248	66132242	775	36	120000	0.28282	0.38866	LEPR	76353	25kb
ENSG00000213625	1	65861270	65926690	164	34	120000	1.3787	0.083998	LEPROT	76353	25kb
ENSG00000184588	1	66233197	66865259	1490	92	120000	-1.2221	0.88917	PDE4B	76353	25kb
ENSG00000118473	1	66974066	67238982	924	32	120000	-0.75669	0.77538	SGIP1	76353	25kb
ENSG00000248458	1	67106547	67167710	203	20	120000	-1.4585	0.92765	AL139147.1	76353	25kb
ENSG00000152760	1	67193142	67269470	200	28	120000	-0.57175	0.71625	TCTEX1D1	76353	25kb
ENSG00000172410	1	67238424	67291939	143	27	120000	-0.47787	0.68363	INSL5	76353	25kb
ENSG00000152763	1	67253568	67415570	342	43	120000	-1.2111	0.88707	WDR78	76353	25kb
ENSG00000198160	1	67365578	67479302	254	14	120000	-0.35491	0.63867	MIER1	76353	25kb
ENSG00000116704	1	67440015	67544782	336	26	120000	-0.4555	0.67562	SLC35D1	76353	25kb
ENSG00000203963	1	67532848	67722536	600	38	120000	-1.1526	0.87545	C1orf141	76353	25kb
ENSG00000162594	1	67607083	67750662	397	36	120000	-1.3155	0.90583	IL23R	76353	25kb
ENSG00000081985	1	67748047	67887583	354	33	120000	-1.6483	0.95035	IL12RB2	76353	25kb
ENSG00000142864	1	67848493	67921098	157	19	120000	-1.5156	0.93519	SERBP1	76353	25kb
ENSG00000116717	1	68125744	68179021	130	17	120000	1.3267	0.092311	GADD45A	76353	25kb
ENSG00000172380	1	68142149	68324150	452	36	120000	0.58395	0.27963	GNG12	76353	25kb
ENSG00000162595	1	68486645	68542314	74	9	120000	-0.90735	0.81789	DIRAS3	76353	25kb
ENSG00000116729	1	68539142	68723803	683	81	120000	0.62876	0.26475	WLS	76353	25kb
ENSG00000116745	1	68869505	68940642	254	25	120000	2.5129	0.0059864	RPE65	76353	25kb
ENSG00000024526	1	68914835	68987904	205	24	120000	1.1086	0.13381	DEPDC1	76353	25kb
ENSG00000033122	1	70009081	70642628	1641	74	120000	1.2821	0.099909	LRRC7	76353	25kb
ENSG00000066557	1	70585488	70696303	160	17	120000	-0.2095	0.58297	LRRC40	76353	25kb
ENSG00000116754	1	70646365	70743735	142	16	120000	0.064959	0.4741	SRSF11	76353	25kb
ENSG00000118454	1	70701271	70845417	225	31	120000	0.90207	0.18351	ANKRD13C	76353	25kb
ENSG00000197568	1	70795488	70876022	153	19	120000	1.1129	0.13287	HHLA3	76353	25kb
ENSG00000116761	1	70851901	70930534	215	33	120000	0.32024	0.37439	CTH	76353	25kb
ENSG00000050628	1	71293036	71538491	677	49	120000	-1.1664	0.87827	PTGER3	76353	25kb
ENSG00000132485	1	71503974	71571980	146	22	120000	-0.0081104	0.50324	ZRANB2	76353	25kb
ENSG00000172260	1	71836623	72773417	1987	103	120000	1.614	0.053269	NEGR1	76353	25kb
ENSG00000162620	1	74466699	74688871	384	17	120000	1.3095	0.095176	LRRIQ3	76353	25kb
ENSG00000254685	1	74638896	74724770	188	19	120000	1.1899	0.11705	FPGT	76353	25kb
ENSG00000259030	1	74638919	75034666	786	64	120000	2.08	0.018761	FPGT-TNNI3K	76353	25kb
ENSG00000116783	1	74638947	75035112	786	64	120000	2.08	0.018761	TNNI3K	76353	25kb
ENSG00000162621	1	74910562	75003298	179	24	120000	1.6705	0.047415	LRRC53	76353	25kb
ENSG00000178965	1	75008795	75164422	302	29	120000	1.2179	0.11164	C1orf173	76353	25kb
ENSG00000116791	1	75146170	75224092	226	32	120000	-1.1028	0.86494	CRYZ	76353	25kb
ENSG00000162623	1	75173836	75257362	291	20	120000	-1.032	0.84896	TYW3	76353	25kb
ENSG00000162624	1	75569119	75652218	153	22	120000	-1.4768	0.93013	LHX8	76353	25kb
ENSG00000137968	1	75642816	76101801	1126	45	120000	-0.41959	0.66261	SLC44A5	76353	25kb
ENSG00000117054	1	76165036	76278260	382	22	120000	0.23199	0.40827	ACADM	76353	25kb
ENSG00000137955	1	76226879	76285764	188	23	120000	0.19815	0.42146	RABGGTB	76353	25kb
ENSG00000057468	1	76237567	76403923	621	26	120000	-0.23219	0.5918	MSH4	76353	25kb
ENSG00000154007	1	76359558	76423116	237	28	120000	-0.26664	0.60513	ASB17	76353	25kb
ENSG00000184005	1	76515404	77125286	1955	149	120000	-1.7583	0.96066	ST6GALNAC3	76353	25kb
ENSG00000117069	1	77308126	77556396	613	45	120000	-1.7391	0.95899	ST6GALNAC5	76353	25kb
ENSG00000142892	1	77529675	77710115	465	32	120000	-1.2725	0.8984	PIGK	76353	25kb
ENSG00000154027	1	77722736	78050651	1080	74	120000	-2.0911	0.98174	AK5	76353	25kb
ENSG00000036549	1	78003101	78174104	313	23	120000	-1.1275	0.87024	ZZZ3	76353	25kb
ENSG00000077254	1	78136672	78250537	173	24	120000	-0.90543	0.81738	USP33	76353	25kb
ENSG00000180488	1	78220309	78369106	230	32	120000	-1.2586	0.89591	FAM73A	76353	25kb
ENSG00000162614	1	78329198	78434580	172	23	120000	-0.70857	0.76071	NEXN	76353	25kb
ENSG00000162613	1	78384740	78469794	177	24	120000	-0.62143	0.73284	FUBP1	76353	25kb
ENSG00000162616	1	78419859	78508648	199	27	120000	-0.65693	0.74439	DNAJB4	76353	25kb
ENSG00000137960	1	78420226	78629133	515	32	120000	-0.51395	0.69636	GIPC2	76353	25kb
ENSG00000122420	1	78744568	79030434	812	51	120000	-1.1657	0.87814	PTGFR	76353	25kb
ENSG00000137959	1	79060607	79136830	155	32	120000	-1.3019	0.90352	IFI44L	76353	25kb
ENSG00000137965	1	79090481	79154763	162	20	120000	-0.82225	0.79453	IFI44	76353	25kb
ENSG00000162618	1	79330449	79497403	723	34	120000	1.6654	0.047921	ELTD1	76353	25kb
ENSG00000117114	1	81746845	82483120	2412	177	120000	-1.531	0.93712	LPHN2	76353	25kb
ENSG00000137941	1	84305711	84489833	483	23	120000	-0.16666	0.56618	TTLL7	76353	25kb
ENSG00000142875	1	84518745	84729181	363	29	120000	0.55227	0.29038	PRKACB	76353	25kb
ENSG00000203943	1	84739049	84880640	375	46	120000	0.25498	0.39937	SAMD13	76353	25kb
ENSG00000137976	1	84839215	84905701	265	23	120000	-0.52347	0.69968	DNASE2B	76353	25kb
ENSG00000117133	1	84919942	84988473	271	22	120000	0.05282	0.47894	RPF1	76353	25kb
ENSG00000174021	1	84939008	84997248	220	25	120000	0.014334	0.49428	GNG5	76353	25kb
ENSG00000117151	1	84990289	85065163	198	19	120000	0.97042	0.16592	CTBS	76353	25kb
ENSG00000180869	1	85068913	85125703	156	28	120000	0.21413	0.41522	C1orf180	76353	25kb
ENSG00000117155	1	85084390	85181486	252	23	120000	0.29491	0.38403	SSX2IP	76353	25kb
ENSG00000171517	1	85252285	85383896	477	65	120000	-0.35005	0.63685	LPAR3	76353	25kb
ENSG00000153898	1	85366268	85487796	403	38	120000	-0.1184	0.54712	MCOLN2	76353	25kb
ENSG00000162643	1	85439830	85623821	576	38	120000	0.044647	0.48219	WDR63	76353	25kb
ENSG00000055732	1	85458765	85539182	200	22	120000	-0.31878	0.62505	MCOLN3	76353	25kb
ENSG00000097096	1	85597556	85691729	287	23	120000	0.873	0.19133	SYDE2	76353	25kb
ENSG00000162642	1	85690639	85750355	194	31	120000	1.1539	0.12428	C1orf52	76353	25kb
ENSG00000142867	1	85706931	85767773	187	31	120000	1.3965	0.081278	BCL10	76353	25kb
ENSG00000153904	1	85759164	86068933	867	69	120000	0.24432	0.40349	DDAH1	76353	25kb
ENSG00000142871	1	86021444	86074645	130	27	120000	-2.5074	0.99392	CYR61	76353	25kb
ENSG00000117174	1	86090106	86199116	272	23	120000	-0.35827	0.63993	ZNHIT6	76353	25kb
ENSG00000171502	1	86169916	86647626	1632	67	120000	-0.92362	0.82216	COL24A1	76353	25kb
ENSG00000122417	1	86789344	86887023	316	21	120000	0.62665	0.26544	ODF2L	76353	25kb
ENSG00000137975	1	86864769	86947241	352	27	120000	0.29666	0.38336	CLCA2	76353	25kb
ENSG00000016490	1	86909051	86990972	271	39	120000	-0.22299	0.58823	CLCA1	76353	25kb
ENSG00000016602	1	86987761	87071437	221	36	120000	0.25559	0.39913	CLCA4	76353	25kb
ENSG00000097033	1	87145259	87238867	195	29	120000	0.3975	0.3455	SH3GLB1	76353	25kb
ENSG00000183291	1	87303132	87405107	284	17	120000	-0.10176	0.54053	SEP15	76353	25kb
ENSG00000153936	1	87355331	87627334	681	30	120000	-0.31266	0.62273	HS2ST1	76353	25kb
ENSG00000267561	1	87433692	87659884	552	31	120000	-0.22461	0.58886	RP5-1052I5.2	76353	25kb
ENSG00000143013	1	87769151	87839606	204	41	120000	-0.31751	0.62457	LMO4	76353	25kb
ENSG00000065243	1	89124905	89326938	462	14	120000	-1.38	0.91621	PKN2	76353	25kb

ENSG00000137947	1	89293615	89382627	289	17	120000	-1.5116	0.93468	GTF2B	76353	25kb
ENSG00000137944	1	89376456	89483636	225	20	120000	-1.9074	0.97176	CCBL2	76353	25kb
ENSG00000213516	1	89420139	89483643	120	18	120000	-1.7056	0.95596	RBMXL1	76353	25kb
ENSG00000117226	1	89447349	89513577	137	17	120000	-1.1993	0.88479	GBP3	76353	25kb
ENSG00000117228	1	89493002	89556043	173	17	120000	-0.9577	0.83089	GBP1	76353	25kb
ENSG00000162645	1	89546815	89641139	234	16	120000	-1.213	0.88743	GBP2	76353	25kb
ENSG00000213512	1	89572434	89666723	202	16	120000	-1.082	0.86037	GBP7	76353	25kb
ENSG00000162654	1	89621831	89689615	125	15	120000	-1.0589	0.85518	GBP4	76353	25kb
ENSG00000154451	1	89699633	89763544	101	20	120000	-1.1887	0.88273	GBP5	76353	25kb
ENSG00000183347	1	89804617	89877020	220	25	120000	0.70934	0.23906	GBP6	76353	25kb
ENSG00000197147	1	89965395	90088423	302	21	120000	-0.96937	0.83382	LRRC8B	76353	25kb
ENSG00000171488	1	90073631	90260462	361	20	120000	1.0746	0.14128	LRRC8C	76353	25kb
ENSG00000271949	1	90073699	90423809	611	50	120000	1.0356	0.1502	RP11-302M6.4	76353	25kb
ENSG00000171492	1	90261573	90427170	251	48	120000	1.0823	0.13955	LRRC8D	76353	25kb
ENSG00000162664	1	90435671	90526090	177	27	120000	0.065872	0.47374	ZNF326	76353	25kb
ENSG00000143032	1	91152096	91207794	136	28	120000	-0.24013	0.59489	BARHL2	76353	25kb
ENSG00000122482	1	91355859	91512829	261	18	120000	-0.37289	0.64538	ZNF644	76353	25kb
ENSG00000162669	1	91701323	91895426	369	29	120000	0.77603	0.21887	HFM1	76353	25kb
ENSG00000097046	1	91941408	92016321	202	26	120000	-0.72981	0.76725	CDC7	76353	25kb
ENSG00000069702	1	92120902	92396892	918	79	120000	-0.50075	0.69173	TGFBR3	76353	25kb
ENSG00000137948	1	92389928	92504983	338	16	120000	0.32222	0.37364	BRDT	76353	25kb
ENSG00000172031	1	92470539	92554093	175	17	120000	0.39686	0.34574	EPHX4	76353	25kb
ENSG00000189195	1	92520862	92638393	181	15	120000	-0.73395	0.76851	BTBD8	76353	25kb
ENSG00000069712	1	92607542	92675280	102	12	120000	-0.4493	0.67339	KIAA1107	76353	25kb
ENSG00000203910	1	92658497	92736370	79	8	120000	0.22647	0.41042	C1orf146	76353	25kb
ENSG00000174842	1	92686959	92789544	87	10	120000	0.56566	0.28581	GLMN	76353	25kb
ENSG00000122484	1	92739522	92892613	159	16	120000	0.28916	0.38623	RPAP2	76353	25kb
ENSG00000162676	1	92915319	92977433	98	11	120000	0.36363	0.35807	GFI1	76353	25kb
ENSG00000067208	1	92949253	93282961	648	15	120000	-0.072657	0.52896	EVI5	76353	25kb
ENSG00000122406	1	93272582	93332481	81	8	120000	0.30225	0.38123	RPL5	76353	25kb
ENSG00000154511	1	93282724	93452057	240	14	120000	0.29418	0.38431	FAM69A	76353	25kb
ENSG00000143033	1	93519792	93629638	202	26	120000	-0.042162	0.51682	MTF2	76353	25kb
ENSG00000117500	1	93590299	93671285	154	18	120000	-0.15921	0.56325	TMED5	76353	25kb
ENSG00000122483	1	93620476	93769287	316	17	120000	-0.2251	0.58905	CCDC18	76353	25kb
ENSG00000117505	1	93786445	93860055	168	16	120000	-0.086965	0.53465	DR1	76353	25kb
ENSG00000137942	1	93888688	94045218	219	35	120000	-0.75974	0.77629	FNBP1L	76353	25kb
ENSG00000137936	1	94002347	94337706	1007	35	120000	-1.5922	0.94433	BCAR3	76353	25kb
ENSG00000067334	1	94308373	94370474	203	22	120000	-1.2532	0.89494	DNTTIP2	76353	25kb
ENSG00000023909	1	94325761	94399966	243	27	120000	-0.58876	0.72199	GCLM	76353	25kb
ENSG00000198691	1	94433393	94611688	655	83	120000	-1.1748	0.87997	ABCA4	76353	25kb
ENSG00000137962	1	94589544	94765624	371	39	120000	-0.11762	0.54681	ARHGAP29	76353	25kb
ENSG00000117528	1	94858933	95009222	190	24	120000	0.17175	0.43182	ABCD3	76353	25kb
ENSG00000117525	1	94969781	95032356	120	29	120000	-0.34462	0.63481	F3	76353	25kb
ENSG00000143036	1	95260898	95385802	450	62	120000	-0.91419	0.81969	SLC44A3	76353	25kb
ENSG00000117519	1	95337507	95417834	290	41	120000	-0.3247	0.6273	CNN3	76353	25kb
ENSG00000172339	1	95414963	95563501	484	36	120000	-1.0983	0.86396	ALG14	76353	25kb
ENSG00000152078	1	95557894	95688163	370	36	120000	-0.68834	0.75438	TMEM56	76353	25kb
ENSG00000271092	1	95558479	95737781	504	32	120000	-0.87729	0.80983	TMEM56-RWDD3	76353	25kb
ENSG00000122481	1	95674711	95737781	177	24	120000	-1.2774	0.89928	RWDD3	76353	25kb
ENSG00000237435	1	96378457	96513436	385	26	120000	0.63749	0.2619	RP11-147C23.1	76353	25kb
ENSG00000117569	1	97162221	97314294	319	25	120000	-1.4791	0.93044	PTBP2	76353	25kb
ENSG00000188641	1	97518299	98411605	2275	128	120000	-0.43926	0.66976	DPYD	76353	25kb
ENSG00000162627	1	99102236	99251056	462	28	120000	-0.34318	0.63427	SNX7	76353	25kb
ENSG00000117598	1	99330806	99495588	604	52	120000	-1.7401	0.95908	LPPR5	76353	25kb
ENSG00000117600	1	99704509	99800146	267	30	120000	-0.6334	0.73676	LPPR4	76353	25kb
ENSG00000099260	1	100086499	100185097	258	31	120000	-0.43666	0.66882	PALMD	76353	25kb
ENSG00000156869	1	100149259	100257187	376	19	120000	-0.3415	0.63364	FRRS1	76353	25kb
ENSG00000162688	1	100290640	100414579	388	28	120000	0.1257	0.44998	AGL	76353	25kb
ENSG00000117620	1	100410345	100517535	336	20	120000	0.14136	0.44379	SLC35A3	76353	25kb
ENSG00000156875	1	100478653	100573933	275	16	120000	0.0052999	0.49789	HIAT1	76353	25kb
ENSG00000156876	1	100524119	100623511	237	22	120000	0.10869	0.45672	SASS6	76353	25kb
ENSG00000122435	1	100573706	100641053	154	19	120000	0.23835	0.40581	TRMT13	76353	25kb
ENSG00000122477	1	100589409	100668771	165	19	120000	0.1791	0.42893	LRRC39	76353	25kb
ENSG00000137992	1	100627475	100740390	243	19	120000	-0.14799	0.55882	DBT	76353	25kb
ENSG00000137996	1	100706763	100783325	176	14	120000	-0.19341	0.57668	RTCA	76353	25kb
ENSG00000079335	1	100785584	101010833	430	32	120000	-1.2176	0.8883	CDC14A	76353	25kb
ENSG00000181656	1	100978693	101032574	156	14	120000	0.5859	0.27897	GPR88	76353	25kb
ENSG00000162692	1	101160298	101229601	144	27	120000	0.24541	0.40307	VCAM1	76353	25kb
ENSG00000162694	1	101312943	101386554	89	15	120000	-0.27529	0.60845	EXTL2	76353	25kb
ENSG00000162695	1	101336632	101472309	174	17	120000	-0.72002	0.76424	SLC30A7	76353	25kb
ENSG00000117543	1	101430179	101516644	115	11	120000	-0.46559	0.67924	DPH5	76353	25kb
ENSG00000269175	1	101430761	101482653	62	8	120000	-0.5728	0.71661	AC093157.1	76353	25kb
ENSG00000170989	1	101677444	101732074	139	34	120000	-0.54721	0.70788	S1PR1	76353	25kb
ENSG00000118733	1	102243130	102487586	831	95	120000	-0.57837	0.71849	OLFM3	76353	25kb
ENSG00000060718	1	103317023	103599052	1179	42	120000	-0.43778	0.66923	COL11A1	76353	25kb
ENSG00000185946	1	104043313	104122861	104	21	120000	-0.32111	0.62594	RNPC3	76353	25kb
ENSG00000240038	1	104071437	104147156	87	19	120000	0.018672	0.49255	AMY2B	76353	25kb
ENSG00000243480	1	104134999	104193402	13	6	120000	1.1729	0.12042	AMY2A	76353	25kb
ENSG00000187733	1	104268028	104326312	42	11	120000	1.2402	0.10745	AMY1C	76353	25kb
ENSG00000198890	1	107574267	107626916	55	7	120000	0.50731	0.30597	PRMT6	76353	25kb
ENSG00000162631	1	107657629	108051080	918	92	120000	-0.47153	0.68137	NTNG1	76353	25kb
ENSG00000134215	1	108088782	108532766	1493	97	120000	1.0598	0.14462	VAV3	76353	25kb
ENSG00000085491	1	108651658	108768471	398	32	120000	-0.25648	0.60121	SLC25A24	76353	25kb
ENSG00000196427	1	108740963	108811689	68	13	120000	-0.65819	0.74479	NBPF4	76353	25kb
ENSG00000186086	1	108893421	109038624	132	8	120000	-0.16316	0.5648	NBPF6	76353	25kb
ENSG00000162636	1	109077711	109212522	251	13	120000	-0.86771	0.80722	FAM102B	76353	25kb
ENSG00000162639	1	109165912	109229148	97	11	120000	-0.86972	0.80777	HENMT1	76353	25kb
ENSG00000134186	1	109209945	109269425	78	20	120000	0.86813	0.19266	PRPF38B	76353	25kb
ENSG00000143107	1	109230279	109310365	161	26	120000	0.11	0.4562	FNDC7	76353	25kb
ENSG00000116266	1	109264296	109377148	358	17	120000	-0.31579	0.62392	STXBP3	76353	25kb
ENSG00000162641	1	109333520	109531106	665	47	120000	0.28025	0.38964	AKNAD1	76353	25kb
ENSG00000121957	1	109392972	109502167	372	25	120000	0.6357	0.26249	GPSM2	76353	25kb
ENSG00000121940	1	109447130	109531111	277	30	120000	0.46792	0.31992	CLCC1	76353	25kb
ENSG00000085433	1	109487836	109609850	348	37	120000	-0.73405	0.76854	WDR47	76353	25kb
ENSG00000197780	1	109580108	109643624	194	34	120000	-0.77429	0.78062	TAF13	76353	25kb
ENSG00000215717	1	109607425	109664556	180	28	120000	-1.1093	0.86635	TMEM167B	76353	25kb

ENSG00000179902	1	109623573	109681479	192	28	120000	-0.31863	0.625	C1orf194	76353	25kb
ENSG00000116299	1	109631301	109774401	459	50	120000	-0.7358	0.76907	KIAA1324	76353	25kb
ENSG00000031698	1	109731540	109805791	191	33	120000	-0.78275	0.78311	SARS	76353	25kb
ENSG00000143126	1	109767641	109843372	217	42	120000	0.59015	0.27754	CELSR2	76353	25kb
ENSG00000134222	1	109797178	109850808	164	33	120000	1.4435	0.074441	PSRC1	76353	25kb
ENSG00000221986	1	109809987	109874663	160	30	120000	2.1539	0.015624	MYBPHL	76353	25kb
ENSG00000134243	1	109827192	109965573	249	23	120000	2.1374	0.016283	SORT1	76353	25kb
ENSG00000143106	1	109916653	109994062	117	13	120000	2.0858	0.018498	PSMA5	76353	25kb
ENSG00000143028	1	109984180	110049759	134	11	120000	2.5446	0.0054694	SYPL2	76353	25kb
ENSG00000162650	1	110001101	110060426	136	13	120000	2.6021	0.0046334	ATXN7L2	76353	25kb
ENSG00000174151	1	110011674	110070554	142	15	120000	2.3705	0.0088829	CYB561D1	76353	25kb
ENSG00000181754	1	110021797	110077360	130	15	120000	2.0167	0.021862	AMIGO1	76353	25kb
ENSG00000156097	1	110057494	110116028	105	17	120000	1.4973	0.067163	GPR61	76353	25kb
ENSG00000065135	1	110066233	110161975	155	24	120000	1.0327	0.15086	GNAI3	76353	25kb
ENSG00000134183	1	110120889	110180679	112	21	120000	0.55767	0.28853	GNAT2	76353	25kb
ENSG00000116337	1	110133726	110199673	131	22	120000	0.41714	0.33829	AMPD2	76353	25kb
ENSG00000168765	1	110173703	110233118	110	20	120000	0.16246	0.43547	GSTM4	76353	25kb
ENSG00000213366	1	110185644	110277171	193	39	120000	0.096131	0.46171	GSTM2	76353	25kb
ENSG00000134184	1	110205436	110276661	149	31	120000	0.053634	0.47861	GSTM1	76353	25kb
ENSG00000134201	1	110229864	110343050	302	45	120000	0.47531	0.31728	GSTM5	76353	25kb
ENSG00000134202	1	110251554	110309384	155	27	120000	-0.11359	0.54522	GSTM3	76353	25kb
ENSG00000198758	1	110267702	110331649	188	30	120000	0.33452	0.36899	EPS8L3	76353	25kb
ENSG00000184371	1	110427864	110498614	214	41	120000	-2.0281	0.97872	CSF1	76353	25kb
ENSG00000168710	1	110502308	110591357	87	24	120000	-1.8151	0.96525	AHCYL1	76353	25kb
ENSG00000143093	1	110549199	110642263	122	15	120000	-1.4437	0.92559	STRIP1	76353	25kb
ENSG00000156150	1	110577616	110638322	92	10	120000	-1.6262	0.94804	ALX3	76353	25kb
ENSG00000186150	1	110630062	110681569	137	24	120000	-1.3401	0.9099	UBL4B	76353	25kb
ENSG00000197106	1	110668108	110769824	222	29	120000	-0.63225	0.73639	SLC6A17	76353	25kb
ENSG00000116396	1	110728965	110850722	374	38	120000	-0.093893	0.5374	KCNC4	76353	25kb
ENSG00000162775	1	110856128	110914299	143	17	120000	1.0016	0.15827	RBM15	76353	25kb
ENSG00000168679	1	110880470	110958704	163	20	120000	1.9107	0.028021	SLC16A4	76353	25kb
ENSG00000134248	1	110918871	110975564	141	18	120000	2.2507	0.012202	LAMTOR5	76353	25kb
ENSG00000143125	1	110968822	111024976	182	38	120000	1.2924	0.098102	PROK1	76353	25kb
ENSG00000143105	1	111034839	111086797	144	21	120000	0.051613	0.47942	KCNA10	76353	25kb
ENSG00000177301	1	111111202	111199096	199	33	120000	-0.58013	0.71909	KCNA2	76353	25kb
ENSG00000177272	1	111189310	111242655	118	17	120000	-1.5502	0.93945	KCNA3	76353	25kb
ENSG00000143119	1	111390775	111467550	317	21	120000	-0.33883	0.63263	CD53	76353	25kb
ENSG00000121931	1	111464807	111531701	154	14	120000	-0.5441	0.70682	LRIF1	76353	25kb
ENSG00000156171	1	111634955	111707838	153	16	120000	-1.2641	0.89691	DRAM2	76353	25kb
ENSG00000134255	1	111657249	111752724	178	30	120000	-1.8223	0.9658	CEPT1	76353	25kb
ENSG00000162777	1	111704796	111772157	149	37	120000	-1.0971	0.8637	DENND2D	76353	25kb
ENSG00000064886	1	111718393	111811062	322	37	120000	0.19198	0.42388	CHI3L2	76353	25kb
ENSG00000134216	1	111808484	111888188	438	35	120000	0.13989	0.44437	CHIA	76353	25kb
ENSG00000173947	1	111863910	111920635	258	27	120000	0.6079	0.27163	PIFO	76353	25kb
ENSG00000085465	1	111931936	111995399	188	22	120000	-0.42455	0.66442	OVGP1	76353	25kb
ENSG00000116455	1	111957512	112016998	153	17	120000	-0.30053	0.61811	WDR77	76353	25kb
ENSG00000116459	1	111966486	112030395	164	21	120000	-0.42561	0.6648	ATP5F1	76353	25kb
ENSG00000143110	1	111991414	112046134	147	28	120000	-0.48532	0.68627	C1orf162	76353	25kb
ENSG00000121933	1	112000970	112131584	385	59	120000	-0.58441	0.72053	ADORA3	76353	25kb
ENSG00000116473	1	112059840	112284313	677	55	120000	0.7729	0.21979	RAP1A	76353	25kb
ENSG00000197852	1	112198252	112323446	378	36	120000	0.54921	0.29143	FAM212B	76353	25kb
ENSG00000064703	1	112272867	112335638	183	20	120000	-0.49881	0.69104	DDX20	76353	25kb
ENSG00000171385	1	112288284	112556777	803	95	120000	-1.1632	0.87763	KCND3	76353	25kb
ENSG00000143079	1	112913803	113031078	284	16	120000	2.1404	0.016161	CTTNBP2NL	76353	25kb
ENSG00000134245	1	112984163	113097787	251	41	120000	1.6779	0.046687	WNT2B	76353	25kb
ENSG00000007341	1	113041140	113188447	296	22	120000	-0.10625	0.54231	ST7L	76353	25kb
ENSG00000116489	1	113136795	113239241	170	17	120000	-0.56858	0.71518	CAPZA1	76353	25kb
ENSG00000155363	1	113190763	113268368	143	24	120000	-0.47954	0.68422	MOV10	76353	25kb
ENSG00000155366	1	113218728	113275056	109	24	120000	-0.40635	0.65776	RHOC	76353	25kb
ENSG00000271810	1	113220236	113279055	116	27	120000	-0.43691	0.66891	RP11-426L16.10	76353	25kb
ENSG00000155367	1	113227616	113283099	129	29	120000	-0.25657	0.60124	PPM1J	76353	25kb
ENSG00000184599	1	113238041	113294857	138	30	120000	0.33062	0.37046	FAM19A3	76353	25kb
ENSG00000155380	1	113429469	113524635	156	19	120000	1.5332	0.06261	SLC16A1	76353	25kb
ENSG00000198799	1	113590831	113699882	222	24	120000	0.1241	0.45062	LRIG2	76353	25kb
ENSG00000081026	1	113908371	114253545	652	38	120000	0.23134	0.40852	MAGI3	76353	25kb
ENSG00000116793	1	114214453	114327111	186	20	120000	0.31006	0.37826	PHTF1	76353	25kb
ENSG00000081019	1	114279454	114380098	150	22	120000	0.0029897	0.49881	RSBN1	76353	25kb
ENSG00000134242	1	114331433	114439381	179	21	120000	0.7721	0.22003	PTPN22	76353	25kb
ENSG00000188761	1	114395790	114455169	104	16	120000	1.303	0.096296	BCL2L15	76353	25kb
ENSG00000134262	1	114412370	114472823	110	22	120000	1.2613	0.10361	AP4B1	76353	25kb
ENSG00000118655	1	114422763	114481708	109	24	120000	1.1999	0.11509	DCLRE1B	76353	25kb
ENSG00000163349	1	114446814	114545426	196	30	120000	-0.68497	0.75332	HIPK1	76353	25kb
ENSG00000116774	1	114497063	114549876	119	20	120000	-1.1336	0.87151	OLFML3	76353	25kb
ENSG00000134207	1	114606913	114721541	425	73	120000	0.22295	0.41179	SYT6	76353	25kb
ENSG00000197323	1	114910399	115078781	403	24	120000	0.70293	0.24105	TRIM33	76353	25kb
ENSG00000116752	1	115085178	115149260	117	19	120000	1.3462	0.089113	BCAS2	76353	25kb
ENSG00000175984	1	115100469	115238043	275	20	120000	1.291	0.098358	DENND2C	76353	25kb
ENSG00000116748	1	115190719	115263239	146	24	120000	1.2399	0.1075	AMPD1	76353	25kb
ENSG00000213281	1	115222090	115284515	102	20	120000	1.3284	0.092029	NRAS	76353	25kb
ENSG00000009307	1	115234534	115326297	144	19	120000	1.4883	0.068339	CSDE1	76353	25kb
ENSG00000052723	1	115287100	115348306	85	16	120000	1.2586	0.10409	SIKE1	76353	25kb
ENSG00000198765	1	115372424	115562991	448	24	120000	0.039586	0.48421	SYCP1	76353	25kb
ENSG00000134200	1	115547415	115601941	170	15	120000	0.22313	0.41172	TSHB	76353	25kb
ENSG00000134198	1	115565632	115657121	347	27	120000	0.90632	0.18238	TSPAN2	76353	25kb
ENSG00000134259	1	115803539	115905857	367	54	120000	0.98991	0.16111	NGF	76353	25kb
ENSG00000173218	1	116159574	116265845	358	35	120000	0.51123	0.30459	VANGL1	76353	25kb
ENSG00000118729	1	116217628	116336402	503	26	120000	2.0717	0.019149	CASQ2	76353	25kb
ENSG00000177551	1	116353998	116411538	195	25	120000	-0.55261	0.70974	NHLH2	76353	25kb
ENSG00000163393	1	116494119	116637675	317	24	120000	-1.0312	0.84877	SLC22A15	76353	25kb
ENSG00000173212	1	116629376	116702861	196	29	120000	-1.6277	0.94821	MAB21L3	76353	25kb
ENSG00000163399	1	116890290	116977883	131	12	120000	0.55531	0.28934	ATP1A1	76353	25kb
ENSG00000269279	1	116890890	116951718	98	10	120000	0.64277	0.26019	AL136376.1	76353	25kb
ENSG00000116815	1	117032157	117138661	137	24	120000	1.0606	0.14443	CD58	76353	25kb
ENSG00000143061	1	117092031	117235375	160	35	120000	-0.8193	0.79369	IGSF3	76353	25kb
ENSG00000203864	1	117211734	117274225	104	25	120000	-1.8041	0.96439	C1orf137	76353	25kb
ENSG00000116824	1	117272007	117336850	123	21	120000	-1.0982	0.86393	CD2	76353	25kb

ENSG00000134247	1	117427679	117557980	348	34	120000	1.6472	0.049759	PTGFRN	76353	25kb
ENSG00000134256	1	117519382	117604167	273	34	120000	1.8725	0.03057	CD101	76353	25kb
ENSG00000116830	1	117577925	117675075	260	33	120000	0.606	0.27226	TTF2	76353	25kb
ENSG00000134253	1	117628682	117690209	142	24	120000	-0.45052	0.67383	TRIM45	76353	25kb
ENSG00000134258	1	117661209	117778556	310	32	120000	0.10607	0.45776	VTCN1	76353	25kb
ENSG00000198162	1	117885071	118096494	552	23	120000	0.13403	0.44669	MAN1A2	76353	25kb
ENSG00000183508	1	118123556	118195994	258	24	120000	0.83969	0.20054	FAM46C	76353	25kb
ENSG00000196505	1	118381107	118497253	108	21	120000	-0.53648	0.70419	GDAP2	76353	25kb
ENSG00000065183	1	118447343	118534165	117	23	120000	-0.4226	0.66371	WDR3	76353	25kb
ENSG00000155761	1	118471484	118752846	543	20	120000	-0.67748	0.75095	SPAG17	76353	25kb
ENSG00000092607	1	119400669	119557179	402	35	120000	-0.10735	0.54274	TBX15	76353	25kb
ENSG00000116874	1	119548839	119708294	411	27	120000	-0.52915	0.70165	WARS2	76353	25kb
ENSG00000116882	1	119886402	119961753	189	26	120000	-0.77647	0.78126	HAO2	76353	25kb
ENSG00000203859	1	119932554	119990658	173	17	120000	-0.9788	0.83616	HSD3B2	76353	25kb
ENSG00000203857	1	120024821	120082681	98	5	120000	-0.94418	0.82746	HSD3B1	76353	25kb
ENSG00000143067	1	120137045	120215396	218	36	120000	-0.0025445	0.50102	ZNF697	76353	25kb
ENSG00000092621	1	120177421	120311838	288	35	120000	-0.16092	0.56392	PHGDH	76353	25kb
ENSG00000134240	1	120265619	120336528	189	26	120000	-1.5307	0.93708	HMGCS2	76353	25kb
ENSG00000134193	1	120311641	120379283	169	15	120000	-1.3562	0.91248	REG4	76353	25kb
ENSG00000134249	1	120411156	120464118	102	16	120000	0.022619	0.49098	ADAM30	76353	25kb
ENSG00000134250	1	120429176	120637240	175	16	120000	-0.40181	0.65609	NOTCH2	76353	25kb
ENSG00000188610	1	120812756	120880681	2	1	120000	-0.65225	0.74288	FAM72B	76353	25kb
ENSG00000198019	1	120901979	120960937	4	1	120000	0.65366	0.25667	FCGR1B	76353	25kb
ENSG00000178104	1	144811157	145101186	3	2	120000	1.0386	0.1495	PDE4DIP	76353	25kb
ENSG00000213240	1	145184119	145316972	2	1	120000	-0.55018	0.7089	NOTCH2NL	76353	25kb
ENSG00000255168	1	145184145	145344796	2	1	120000	-0.55018	0.7089	RP11-458D21.5	76353	25kb
ENSG00000163386	1	145264772	145395303	35	9	120000	-0.28972	0.61398	NBPF10	76353	25kb
ENSG00000168509	1	145388095	145442545	87	16	120000	0.097302	0.46124	HFE2	76353	25kb
ENSG00000117289	1	145413469	145467635	52	12	120000	0.19027	0.42455	TXNIP	76353	25kb
ENSG00000121851	1	145431236	145495388	44	11	120000	-0.10561	0.54205	POLR3GL	76353	25kb
ENSG00000181039	1	145445508	145500646	32	8	120000	-0.012128	0.50484	ANKRD34A	76353	25kb
ENSG00000152022	1	145452085	145526669	43	15	120000	-0.6366	0.73781	LIX1L	76353	25kb
ENSG00000131795	1	145482598	145538536	35	13	120000	-0.62126	0.73279	RBM8A	76353	25kb
ENSG00000131779	1	145491252	145548730	49	14	120000	-0.90796	0.81805	PEX11B	76353	25kb
ENSG00000143127	1	145499891	145568868	93	18	120000	-0.94583	0.82788	ITGA10	76353	25kb
ENSG00000198483	1	145524230	145593526	111	18	120000	-1.0159	0.84516	ANKRD35	76353	25kb
ENSG00000131788	1	145550233	145611546	106	16	120000	-0.95196	0.82944	PIAS3	76353	25kb
ENSG00000186364	1	145561115	145614439	84	11	120000	-0.74437	0.77167	NUDT17	76353	25kb
ENSG00000186141	1	145567605	145636025	83	13	120000	-0.50305	0.69253	POLR3C	76353	25kb
ENSG00000121848	1	145586036	145721218	181	14	120000	0.11673	0.45354	RNF115	76353	25kb
ENSG00000117281	1	145670798	145740614	90	13	120000	0.20921	0.41714	CD160	76353	25kb
ENSG00000174827	1	145701918	145789074	63	15	120000	0.3858	0.34982	PDZK1	76353	25kb
ENSG00000117262	1	145739411	145852103	17	9	120000	1.4221	0.077503	GPR89A	76353	25kb
ENSG00000186275	1	146348546	146492744	8	3	120000	-0.26546	0.60467	NBPF12	76353	25kb
ENSG00000131791	1	146601685	146669129	262	12	120000	0.43386	0.3322	PRKAB2	76353	25kb
ENSG00000131781	1	146621930	146739700	523	22	120000	0.39627	0.34595	FMO5	76353	25kb
ENSG00000131778	1	146689291	146792443	477	28	120000	-0.051404	0.5205	CHD1L	76353	25kb
ENSG00000116128	1	146988182	147123017	390	67	120000	-1.244	0.89325	BCL9	76353	25kb
ENSG00000162836	1	147076453	147167618	227	31	120000	-0.40659	0.65784	ACP6	76353	25kb
ENSG00000143140	1	147203332	147270484	228	27	120000	-1.4058	0.92011	GJA5	76353	25kb
ENSG00000121634	1	147349946	147406393	152	26	120000	-0.51583	0.69701	GJA8	76353	25kb
ENSG00000188092	1	147375506	147490753	71	14	120000	-0.21925	0.58677	GPR89B	76353	25kb
ENSG00000150337	1	149729227	149789074	27	6	120000	-1.1981	0.88456	FCGR1A	76353	25kb
ENSG00000203814	1	149729245	149808928	28	6	120000	-1.1665	0.8783	HIST2H2BF	76353	25kb
ENSG00000183598	1	149759826	149810236	24	5	120000	-0.97373	0.8349	HIST2H3D	76353	25kb
ENSG00000183941	1	149779221	149836339	25	7	120000	-1.6367	0.94915	HIST2H4A	76353	25kb
ENSG00000203811	1	149786110	149837765	18	7	120000	-1.5605	0.94068	HIST2H3C	76353	25kb
ENSG00000183558	1	149788505	149839478	15	6	120000	-1.5223	0.93604	HIST2H2AA3	76353	25kb
ENSG00000203812	1	149797643	149848191	14	6	120000	-2.0972	0.98201	HIST2H2AA4	76353	25kb
ENSG00000203852	1	149799181	149850836	18	7	120000	-1.5645	0.94115	HIST2H3A	76353	25kb
ENSG00000182217	1	149800607	149857714	28	8	120000	-1.3359	0.90921	HIST2H4B	76353	25kb
ENSG00000184678	1	149831010	149883232	70	8	120000	-0.78734	0.78446	HIST2H2BE	76353	25kb
ENSG00000184260	1	149833525	149883961	71	8	120000	-0.75297	0.77427	HIST2H2AC	76353	25kb
ENSG00000184270	1	149834019	149884466	71	8	120000	-0.75297	0.77427	HIST2H2AB	76353	25kb
ENSG00000178096	1	149834440	149897351	91	10	120000	-0.6771	0.75083	BOLA1	76353	25kb
ENSG00000159164	1	149849870	149914434	116	10	120000	-0.64543	0.74068	SV2A	76353	25kb
ENSG00000143368	1	149870209	149925236	89	10	120000	-0.58401	0.72039	SF3B4	76353	25kb
ENSG00000014914	1	149875543	149933791	87	10	120000	-0.60568	0.72764	MTMR11	76353	25kb
ENSG00000163113	1	149884705	150007625	184	18	120000	-0.72279	0.7651	OTUD7B	76353	25kb
ENSG00000136631	1	150014369	150142505	211	10	120000	-0.37536	0.6463	VPS45	76353	25kb
ENSG00000023902	1	150096373	150161916	107	10	120000	-0.28189	0.61099	PLEKHO1	76353	25kb
ENSG00000143401	1	150165717	150233504	104	10	120000	0.25702	0.39858	ANP32E	76353	25kb
ENSG00000118298	1	150204554	150262478	78	11	120000	0.061458	0.4755	CA14	76353	25kb
ENSG00000117362	1	150212804	150266980	74	10	120000	-0.13901	0.55528	APH1A	76353	25kb
ENSG00000118292	1	150215600	150278327	105	11	120000	-0.2089	0.58274	C1orf54	76353	25kb
ENSG00000159208	1	150229953	150284505	112	12	120000	-0.38807	0.65102	C1orf51	76353	25kb
ENSG00000187145	1	150241289	150306414	156	13	120000	-0.51615	0.69712	MRPS21	76353	25kb
ENSG00000117360	1	150268925	150350671	212	16	120000	-0.59853	0.72526	PRPF3	76353	25kb
ENSG00000163125	1	150310567	150474042	460	17	120000	-0.82553	0.79546	RPRD2	76353	25kb
ENSG00000143374	1	150434887	150505078	182	16	120000	-0.69584	0.75674	TARS2	76353	25kb
ENSG00000143369	1	150455538	150511265	147	17	120000	-0.52987	0.7019	ECM1	76353	25kb
ENSG00000143382	1	150496884	150558413	138	29	120000	0.37725	0.353	ADAMTSL4	76353	25kb
ENSG00000225996	1	150496897	150549367	113	27	120000	0.21079	0.41653	AL356356.1	76353	25kb
ENSG00000203804	1	150508480	150558969	109	24	120000	0.26884	0.39403	ADAMTSL4-AS1	76353	25kb
ENSG00000143384	1	150522032	150577066	127	22	120000	0.29875	0.38257	MCL1	76353	25kb
ENSG00000143420	1	150548327	150627088	168	20	120000	0.44079	0.32968	ENSA	76353	25kb
ENSG00000143457	1	150593701	150694630	261	14	120000	0.42156	0.33667	GOLPH3L	76353	25kb
ENSG00000143452	1	150645536	150718364	192	11	120000	0.39083	0.34796	HORMAD1	76353	25kb
ENSG00000163131	1	150677672	150763433	174	14	120000	0.68352	0.24714	CTSS	76353	25kb
ENSG00000143387	1	150743684	150805799	96	14	120000	0.91567	0.17992	CTSK	76353	25kb
ENSG00000143437	1	150757181	150874244	199	21	120000	0.96283	0.16782	ARNT	76353	25kb
ENSG00000143379	1	150873739	150962213	182	19	120000	0.61875	0.26804	SETDB1	76353	25kb
ENSG00000143418	1	150908059	150972479	150	21	120000	0.68399	0.24699	CERS2	76353	25kb
ENSG00000143412	1	150929493	150993110	165	28	120000	1.2065	0.11381	ANXA9	76353	25kb
ENSG00000143409	1	150944025	151005851	161	30	120000	1.6033	0.054434	FAM63A	76353	25kb
ENSG00000143363	1	150955896	151033189	184	37	120000	1.6392	0.050583	PRUNE	76353	25kb

ENSG00000163141	1	150984046	151045076	125	27	120000	1.5238	0.063773	BNIPL	76353	25kb
ENSG00000143443	1	150995216	151049462	110	23	120000	0.97869	0.16387	C1orf56	76353	25kb
ENSG00000197622	1	150998447	151067801	154	24	120000	0.49925	0.3088	CDC42SE1	76353	25kb
ENSG00000213190	1	151005234	151065970	129	21	120000	0.35414	0.36162	MLLT11	76353	25kb
ENSG00000143458	1	151018054	151123018	214	24	120000	0.36143	0.35889	GABPB2	76353	25kb
ENSG00000143434	1	151079161	151144104	110	19	120000	0.38169	0.35135	SEMA6C	76353	25kb
ENSG00000163154	1	151104105	151157225	75	17	120000	0.49791	0.30927	TNFAIP8L2	76353	25kb
ENSG00000163156	1	151104140	151167773	85	21	120000	0.32444	0.3728	SCNM1	76353	25kb
ENSG00000163155	1	151107224	151163424	74	19	120000	0.4296	0.33374	LYSMD1	76353	25kb
ENSG00000163157	1	151117462	151173760	60	19	120000	0.017921	0.49285	TMOD4	76353	25kb
ENSG00000163159	1	151117463	151192797	88	20	120000	-0.27731	0.60923	VPS72	76353	25kb
ENSG00000143398	1	151145425	151247012	124	14	120000	-0.55583	0.71084	PIP5K1A	76353	25kb
ENSG00000159352	1	151202179	151264955	74	10	120000	-0.38242	0.64893	PSMD4	76353	25kb
ENSG00000232671	1	151227500	151279405	51	8	120000	-0.20164	0.5799	RP11-126K1.2	76353	25kb
ENSG00000143373	1	151229094	151289656	60	8	120000	-0.13578	0.554	ZNF687	76353	25kb
ENSG00000143393	1	151239273	151325191	94	15	120000	0.1548	0.43849	PI4KB	76353	25kb
ENSG00000143390	1	151288116	151344833	94	17	120000	0.49832	0.30913	RFX5	76353	25kb
ENSG00000143416	1	151311778	151370209	102	21	120000	0.48238	0.31477	SELENBP1	76353	25kb
ENSG00000159377	1	151347010	151399420	82	11	120000	-0.25629	0.60114	PSMB4	76353	25kb
ENSG00000143442	1	151350200	151456941	156	13	120000	-0.54218	0.70615	POGZ	76353	25kb
ENSG00000143375	1	151457986	151536168	133	20	120000	0.46918	0.31947	CGN	76353	25kb
ENSG00000143367	1	151487781	151581059	174	27	120000	-0.14796	0.55881	TUFT1	76353	25kb
ENSG00000143376	1	151559541	151696567	203	25	120000	-0.53594	0.704	SNX27	76353	25kb
ENSG00000159409	1	151649880	151714290	110	16	120000	-0.57373	0.71692	CELF3	76353	25kb
ENSG00000240510	1	151657909	151727070	121	17	120000	-0.35802	0.63983	AL589765.1	76353	25kb
ENSG00000178796	1	151657909	151727281	121	17	120000	-0.35802	0.63983	RIIAD1	76353	25kb
ENSG00000143436	1	151707119	151761040	119	12	120000	-0.047615	0.51899	MRPL9	76353	25kb
ENSG00000143450	1	151710445	151768808	130	12	120000	-0.21292	0.5843	OAZ3	76353	25kb
ENSG00000182134	1	151717583	151788892	158	14	120000	-0.23124	0.59143	TDRKH	76353	25kb
ENSG00000213171	1	151747740	151803630	124	16	120000	-0.33111	0.62972	LINGO4	76353	25kb
ENSG00000143365	1	151753547	151829348	176	28	120000	-0.29431	0.61574	RORC	76353	25kb
ENSG00000225556	1	151785295	151838033	129	25	120000	-0.036678	0.51463	C2CD4D	76353	25kb
ENSG00000196407	1	151794739	151851173	138	25	120000	0.10663	0.45754	THEM5	76353	25kb
ENSG00000159445	1	151821060	151907284	213	20	120000	0.47226	0.31837	THEM4	76353	25kb
ENSG00000197747	1	151930391	151991866	172	13	120000	0.16213	0.4356	S100A10	76353	25kb
ENSG00000163191	1	151979982	152045383	96	17	120000	-0.012689	0.50506	S100A11	76353	25kb
ENSG00000182898	1	152031620	152086540	51	16	120000	0.36662	0.35695	TCHHL1	76353	25kb
ENSG00000159450	1	152053793	152111556	40	14	120000	0.3339	0.36923	TCHH	76353	25kb
ENSG00000215853	1	152101071	152156704	49	11	120000	0.19911	0.42109	RPTN	76353	25kb
ENSG00000197915	1	152159558	152221669	189	10	120000	-0.67226	0.74929	HRNR	76353	25kb
ENSG00000143631	1	152249651	152322679	195	11	120000	-1.1589	0.87675	FLG	76353	25kb
ENSG00000143520	1	152296211	152357482	105	10	120000	-1.12	0.86864	FLG2	76353	25kb
ENSG00000143536	1	152356719	152411739	96	10	120000	-1.2666	0.89734	CRNN	76353	25kb
ENSG00000186207	1	152458320	152509653	113	11	120000	1.2684	0.10233	LCE5A	76353	25kb
ENSG00000169509	1	152461978	152513486	123	10	120000	1.3699	0.085362	CRCT1	76353	25kb
ENSG00000185966	1	152513130	152564248	119	10	120000	1.3196	0.093482	LCE3E	76353	25kb
ENSG00000163202	1	152526857	152577980	79	12	120000	1.0923	0.13735	LCE3D	76353	25kb
ENSG00000244057	1	152548138	152598562	71	6	120000	-0.60336	0.72686	LCE3C	76353	25kb
ENSG00000187238	1	152561287	152611574	113	7	120000	-0.35097	0.6372	LCE3B	76353	25kb
ENSG00000185962	1	152570310	152620579	151	10	120000	-0.61865	0.73193	LCE3A	76353	25kb
ENSG00000187223	1	152610872	152662135	137	9	120000	-0.79959	0.78803	LCE2D	76353	25kb
ENSG00000187180	1	152622771	152674050	131	11	120000	-0.70783	0.76048	LCE2C	76353	25kb
ENSG00000159455	1	152622771	152684877	157	12	120000	-0.73028	0.76739	LCE2B	76353	25kb
ENSG00000187173	1	152645840	152696918	171	12	120000	-0.94852	0.82857	LCE2A	76353	25kb
ENSG00000187170	1	152655636	152706967	158	11	120000	-0.97428	0.83504	LCE4A	76353	25kb
ENSG00000198854	1	152666998	152717905	137	9	120000	-0.81503	0.79247	C1orf68	76353	25kb
ENSG00000203786	1	152705506	152759529	128	11	120000	-0.52038	0.6986	KPRP	76353	25kb
ENSG00000240386	1	152723848	152774445	118	9	120000	-0.48677	0.68679	LCE1F	76353	25kb
ENSG00000186226	1	152733690	152785902	129	13	120000	-0.25349	0.60005	LCE1E	76353	25kb
ENSG00000172155	1	152744227	152795657	118	14	120000	0.011297	0.49549	LCE1D	76353	25kb
ENSG00000197084	1	152752311	152804107	107	15	120000	0.1882	0.42536	LCE1C	76353	25kb
ENSG00000196734	1	152759447	152810585	83	16	120000	0.67495	0.24985	LCE1B	76353	25kb
ENSG00000186844	1	152774949	152825573	68	19	120000	1.6415	0.050351	LCE1A	76353	25kb
ENSG00000235942	1	152790344	152841459	43	13	120000	2.4076	0.0080281	LCE6A	76353	25kb
ENSG00000163206	1	152825793	152882523	67	15	120000	1.2357	0.10829	SMCP	76353	25kb
ENSG00000163207	1	152856021	152909362	93	11	120000	-0.19428	0.57702	IVL	76353	25kb
ENSG00000184148	1	152918142	152970050	130	9	120000	-1.1187	0.86836	SPRR4	76353	25kb
ENSG00000169474	1	152931557	152983289	162	6	120000	-1.223	0.88933	SPRR1A	76353	25kb
ENSG00000163209	1	152949223	153001332	194	6	120000	-0.94874	0.82862	SPRR3	76353	25kb
ENSG00000169469	1	152978678	153030376	213	6	120000	-0.38678	0.65054	SPRR1B	76353	25kb
ENSG00000163216	1	152987201	153039407	203	7	120000	-0.33937	0.63284	SPRR2D	76353	25kb
ENSG00000241794	1	153003589	153055013	201	8	120000	-0.44193	0.67073	SPRR2A	76353	25kb
ENSG00000196805	1	153017700	153138927	316	22	120000	-0.42793	0.66565	SPRR2B	76353	25kb
ENSG00000203785	1	153040611	153103660	169	21	120000	-0.37327	0.64552	SPRR2E	76353	25kb
ENSG00000244094	1	153059590	153110991	122	20	120000	0.063995	0.47449	SPRR2F	76353	25kb
ENSG00000159516	1	153097058	153148345	107	11	120000	-0.23799	0.59405	SPRR2G	76353	25kb
ENSG00000203784	1	153150919	153202596	128	16	120000	-1.4192	0.92207	LELP1	76353	25kb
ENSG00000203783	1	153165060	153216793	167	18	120000	-1.7253	0.95777	PRR9	76353	25kb
ENSG00000203782	1	153207176	153259598	154	19	120000	-1.1414	0.87314	LOR	76353	25kb
ENSG00000159527	1	153245338	153308194	182	18	120000	0.62027	0.26754	PGLYRP3	76353	25kb
ENSG00000163218	1	153277596	153346316	262	15	120000	0.74761	0.22735	PGLYRP4	76353	25kb
ENSG00000163220	1	153305330	153358503	179	17	120000	0.61579	0.26902	S100A9	76353	25kb
ENSG00000163221	1	153321184	153373125	150	14	120000	-0.44234	0.67088	S100A12	76353	25kb
ENSG00000143546	1	153337508	153388664	122	6	120000	-1.4436	0.92558	S100A8	76353	25kb
ENSG00000184330	1	153364000	153420701	134	14	120000	-1.7142	0.95676	S100A7A	76353	25kb
ENSG00000197364	1	153384534	153437425	109	16	120000	-1.9734	0.97578	S100A7L2	76353	25kb
ENSG00000143556	1	153405220	153458177	126	13	120000	-2.0271	0.97868	S100A7	76353	25kb
ENSG00000197956	1	153482075	153533720	124	9	120000	-1.2937	0.90212	S100A6	76353	25kb
ENSG00000196420	1	153484623	153539241	123	9	120000	-1.2552	0.8953	S100A5	76353	25kb
ENSG00000196154	1	153491089	153547612	122	10	120000	-1.0337	0.84935	S100A4	76353	25kb
ENSG00000188015	1	153494805	153546848	116	9	120000	-1.0323	0.84903	S100A3	76353	25kb
ENSG00000196754	1	153508584	153565366	111	11	120000	-0.54469	0.70702	S100A2	76353	25kb
ENSG00000188643	1	153554362	153610621	125	20	120000	0.087377	0.46519	S100A16	76353	25kb
ENSG00000189334	1	153561731	153614462	119	17	120000	-0.16194	0.56432	S100A14	76353	25kb
ENSG00000189171	1	153566263	153631568	138	17	120000	-0.4175	0.66184	S100A13	76353	25kb
ENSG00000160678	1	153575402	153629513	106	11	120000	-0.57044	0.71581	S100A1	76353	25kb

ENSG00000160679	1	153581525	153643782	114	9	120000	-0.75917	0.77612	CHTOP	76353	25kb
ENSG00000143553	1	153606130	153659306	94	11	120000	-1.3832	0.91669	SNAPIN	76353	25kb
ENSG00000143621	1	153609512	153668524	91	12	120000	-1.5495	0.93937	ILF2	76353	25kb
ENSG00000169418	1	153626113	153691468	99	16	120000	-1.4904	0.93195	NPR1	76353	25kb
ENSG00000143624	1	153675543	153771555	151	20	120000	-0.53187	0.70259	INTS3	76353	25kb
ENSG00000143554	1	153721830	153777633	87	22	120000	0.063994	0.47449	SLC27A3	76353	25kb
ENSG00000143614	1	153752201	153920451	275	17	120000	0.6942	0.24378	GATAD2B	76353	25kb
ENSG00000198837	1	153876977	153944172	102	12	120000	1.0074	0.15688	DENND4B	76353	25kb
ENSG00000160741	1	153895145	153956101	83	10	120000	1.0598	0.14462	CRTC2	76353	25kb
ENSG00000143570	1	153906575	153965188	66	11	120000	0.9509	0.17083	SLC39A1	76353	25kb
ENSG00000143578	1	153915010	153971839	65	14	120000	0.95274	0.17036	CREB3L4	76353	25kb
ENSG00000143543	1	153921745	153975164	60	14	120000	1.029	0.15173	JTB	76353	25kb
ENSG00000143545	1	153929127	153983834	64	15	120000	1.1152	0.13239	RAB13	76353	25kb
ENSG00000177954	1	153938235	153989626	58	13	120000	0.98402	0.16255	RPS27	76353	25kb
ENSG00000143552	1	153940161	154152592	284	19	120000	0.39486	0.34647	NUP210L	76353	25kb
ENSG00000143549	1	154102784	154192124	119	18	120000	-0.81483	0.79242	TPM3	76353	25kb
ENSG00000163263	1	154146848	154203809	61	12	120000	-0.27318	0.60764	C1orf189	76353	25kb
ENSG00000143612	1	154154182	154218104	89	14	120000	-0.38763	0.65085	C1orf43	76353	25kb
ENSG00000143569	1	154167655	154268986	147	19	120000	-0.25089	0.59905	UBAP2L	76353	25kb
ENSG00000143575	1	154219987	154273351	76	14	120000	-0.043362	0.51729	HAX1	76353	25kb
ENSG00000143595	1	154268566	154322801	139	17	120000	-0.74055	0.77052	AQP10	76353	25kb
ENSG00000143515	1	154273029	154348783	242	23	120000	-1.027	0.84779	ATP8B2	76353	25kb
ENSG00000160712	1	154352669	154466926	263	28	120000	0.14932	0.44065	IL6R	76353	25kb
ENSG00000169291	1	154417248	154499589	191	18	120000	0.57854	0.28145	SHE	76353	25kb
ENSG00000163239	1	154449695	154545623	237	17	120000	0.57711	0.28193	TDRD10	76353	25kb
ENSG00000160714	1	154496053	154556504	171	19	120000	0.64	0.26109	UBE2Q1	76353	25kb
ENSG00000160716	1	154515257	154577502	117	23	120000	1.1963	0.11579	CHRNB2	76353	25kb
ENSG00000160710	1	154529538	154625475	174	21	120000	1.6347	0.051053	ADAR	76353	25kb
ENSG00000268141	1	154560067	154610190	101	11	120000	1.3781	0.084087	AL606500.1	76353	25kb
ENSG00000143603	1	154644931	154867756	621	102	120000	1.3599	0.086934	KCNN3	76353	25kb
ENSG00000163344	1	154872210	154934467	104	11	120000	-0.33711	0.63198	PMVK	76353	25kb
ENSG00000163346	1	154891552	154953599	85	15	120000	-0.7189	0.7639	PBXIP1	76353	25kb
ENSG00000163348	1	154904502	154961329	76	15	120000	-0.97766	0.83588	PYGO2	76353	25kb
ENSG00000160691	1	154909774	154971871	78	14	120000	-0.89538	0.81471	SHC1	76353	25kb
ENSG00000173207	1	154922129	154976725	51	8	120000	-1.074	0.85859	CKS1B	76353	25kb
ENSG00000160688	1	154930814	154990587	62	11	120000	-0.89928	0.81575	FLAD1	76353	25kb
ENSG00000163352	1	154941062	154991791	56	10	120000	-0.92627	0.82285	LENEP	76353	25kb
ENSG00000160685	1	154950127	155015998	89	19	120000	-0.47107	0.6812	ZBTB7B	76353	25kb
ENSG00000163354	1	154965996	155031257	91	20	120000	-0.10022	0.53992	DCST2	76353	25kb
ENSG00000163357	1	154981300	155048406	103	23	120000	0.16887	0.43295	DCST1	76353	25kb
ENSG00000143537	1	154998042	155060252	91	21	120000	-0.0017908	0.50071	ADAM15	76353	25kb
ENSG00000243364	1	155011207	155067029	81	21	120000	-0.0061676	0.50246	EFNA4	76353	25kb
ENSG00000251246	1	155011224	155084283	116	20	120000	-0.24301	0.596	EFNA3	76353	25kb
ENSG00000143590	1	155011224	155085014	116	20	120000	-0.24301	0.596	EFNA3	76353	25kb
ENSG00000169242	1	155074936	155132333	123	14	120000	-1.1234	0.86936	EFNA1	76353	25kb
ENSG00000169241	1	155082820	155136329	115	14	120000	-1.1435	0.87358	SLC50A1	76353	25kb
ENSG00000179085	1	155087367	155138071	100	14	120000	-1.151	0.87513	DPM3	76353	25kb
ENSG00000163463	1	155116884	155170951	90	13	120000	-1.0926	0.86271	KRTCAP2	76353	25kb
ENSG00000273088	1	155116885	155184748	107	15	120000	-1.1348	0.87177	RP11-201K10.3	76353	25kb
ENSG00000163462	1	155120873	155182447	103	15	120000	-1.0888	0.86189	TRIM46	76353	25kb
ENSG00000185499	1	155133300	155187707	74	13	120000	-1.0608	0.8556	MUC1	76353	25kb
ENSG00000169231	1	155140379	155203842	86	14	120000	-1.0316	0.84887	THBS3	76353	25kb
ENSG00000173171	1	155153490	155208615	74	12	120000	-0.99187	0.83937	MTX1	76353	25kb
ENSG00000177628	1	155179243	155239490	86	10	120000	-0.68528	0.75342	GBA	76353	25kb
ENSG00000160767	1	155191996	155250274	87	8	120000	-0.38813	0.65104	FAM189B	76353	25kb
ENSG00000116521	1	155200770	155257221	84	7	120000	-0.082098	0.53272	SCAMP3	76353	25kb
ENSG00000176444	1	155207659	155273282	95	8	120000	0.27299	0.39243	CLK2	76353	25kb
ENSG00000143630	1	155222374	155284639	84	7	120000	0.59139	0.27713	HCN3	76353	25kb
ENSG00000143627	1	155234630	155296225	74	7	120000	0.73696	0.23057	PKLR	76353	25kb
ENSG00000160752	1	155253539	155315457	62	8	120000	0.78336	0.21671	FDPS	76353	25kb
ENSG00000160753	1	155265687	155325905	52	9	120000	0.80527	0.21033	RUSC1	76353	25kb
ENSG00000116539	1	155280059	155557598	216	25	120000	0.61114	0.27055	ASH1L	76353	25kb
ENSG00000125459	1	155554979	155743153	167	20	120000	-0.19483	0.57724	MSTO1	76353	25kb
ENSG00000163374	1	155604237	155683791	76	14	120000	0.019709	0.49214	YY1AP1	76353	25kb
ENSG00000132676	1	155632751	155733801	100	14	120000	-0.4854	0.68631	DAP3	76353	25kb
ENSG00000116580	1	155694508	155854191	205	14	120000	-0.42601	0.66495	GON4L	76353	25kb
ENSG00000132718	1	155804300	155879990	115	16	120000	-0.25186	0.59943	SYT11	76353	25kb
ENSG00000143622	1	155842599	155906195	97	11	120000	0.54357	0.29337	RIT1	76353	25kb
ENSG00000132680	1	155857834	155929191	100	10	120000	0.98144	0.16319	KIAA0907	76353	25kb
ENSG00000173080	1	155886480	155937625	64	7	120000	1.2297	0.1094	RXFP4	76353	25kb
ENSG00000116584	1	155891630	156001861	151	20	120000	0.85574	0.19607	ARHGEF2	76353	25kb
ENSG00000163479	1	155953839	156015750	94	17	120000	-0.13244	0.55268	SSR2	76353	25kb
ENSG00000160803	1	155980092	156048585	100	24	120000	-0.31617	0.62406	UBQLN4	76353	25kb
ENSG00000116586	1	155999543	156053301	70	21	120000	-0.22528	0.58912	LAMTOR2	76353	25kb
ENSG00000132698	1	156005951	156065295	83	22	120000	0.13341	0.44693	RAB25	76353	25kb
ENSG00000254726	1	156016804	156076789	77	18	120000	0.41844	0.33781	MEX3A	76353	25kb
ENSG00000160789	1	156027364	156134880	214	23	120000	-0.54223	0.70617	LMNA	76353	25kb
ENSG00000196189	1	156092157	156172543	199	22	120000	-1.0389	0.85057	SEMA4A	76353	25kb
ENSG00000160785	1	156138880	156207587	158	16	120000	-0.47365	0.68212	SLC25A44	76353	25kb
ENSG00000260238	1	156157773	156238123	183	21	120000	0.24951	0.40148	PMF1-BGLAP	76353	25kb
ENSG00000160783	1	156157784	156237874	183	21	120000	0.24951	0.40148	PMF1	76353	25kb
ENSG00000242252	1	156186753	156238112	121	19	120000	0.80012	0.21182	BGLAP	76353	25kb
ENSG00000160781	1	156188206	156242881	128	19	120000	0.81135	0.20858	PAQR6	76353	25kb
ENSG00000198952	1	156194015	156277620	193	20	120000	0.87297	0.19134	SMG5	76353	25kb
ENSG00000163472	1	156227726	156287976	142	10	120000	0.74973	0.22671	TMEM79	76353	25kb
ENSG00000198715	1	156234880	156290463	119	9	120000	0.52764	0.29887	C1orf85	76353	25kb
ENSG00000189030	1	156243415	156294428	124	9	120000	0.53376	0.29675	VHLL	76353	25kb
ENSG00000163468	1	156253759	156362664	228	25	120000	0.36011	0.35938	CCT3	76353	25kb
ENSG00000163467	1	156282105	156341786	108	12	120000	0.49351	0.31083	TSACC	76353	25kb
ENSG00000132677	1	156314003	156380011	128	30	120000	-0.61531	0.73083	RHBG	76353	25kb
ENSG00000125462	1	156349042	156425493	117	33	120000	-0.73863	0.76994	C1orf61	76353	25kb
ENSG00000116604	1	156408519	156495620	164	25	120000	-0.17017	0.56756	MEF2D	76353	25kb
ENSG00000183856	1	156470197	156567396	233	25	120000	0.071176	0.47163	IQGAP3	76353	25kb
ENSG00000187862	1	156524519	156581562	175	13	120000	0.18148	0.428	TTC24	76353	25kb
ENSG00000163382	1	156536554	156589091	169	13	120000	0.18716	0.42577	APOA1BP	76353	25kb
ENSG00000160818	1	156539279	156596288	170	12	120000	0.13906	0.4447	GPATCH4	76353	25kb

ENSG00000132702	1	156564086	156620517	132	13	120000	-0.17546	0.56964	HAPLN2	76353	25kb
ENSG00000132692	1	156586182	156654324	131	25	120000	-1.5575	0.94032	BCAN	76353	25kb
ENSG00000132688	1	156613555	156672189	136	24	120000	-1.5005	0.93325	NES	76353	25kb
ENSG00000143320	1	156644398	156700608	137	34	120000	-1.4121	0.92104	CRABP2	76353	25kb
ENSG00000143319	1	156666683	156723591	114	26	120000	-0.18426	0.5731	ISG20L2	76353	25kb
ENSG00000143303	1	156673234	156731752	123	23	120000	-0.22824	0.59027	RRNAD1	76353	25kb
ENSG00000143314	1	156682095	156736382	116	21	120000	-0.059698	0.5238	MRPL24	76353	25kb
ENSG00000143321	1	156686899	156761717	173	25	120000	-0.2107	0.58344	HDGF	76353	25kb
ENSG00000143294	1	156695402	156795607	237	28	120000	-0.4745	0.68243	PRCC	76353	25kb
ENSG00000027869	1	156751035	156811654	177	22	120000	-0.84958	0.80222	SH2D2A	76353	25kb
ENSG00000198400	1	156760432	156876642	284	40	120000	-1.5685	0.94162	NTRK1	76353	25kb
ENSG00000027644	1	156784855	156853810	162	32	120000	-0.89683	0.8151	INSRR	76353	25kb
ENSG00000187800	1	156838490	156911226	200	24	120000	-1.6671	0.95225	PEAR1	76353	25kb
ENSG00000160838	1	156865442	156927886	164	19	120000	-1.3646	0.91381	LRRC71	76353	25kb
ENSG00000132694	1	156879632	157040162	419	23	120000	-1.4002	0.91928	ARHGEF11	76353	25kb
ENSG00000253831	1	157036836	157094600	265	25	120000	-0.57207	0.71636	ETV3L	76353	25kb
ENSG00000117036	1	157065983	157133266	153	23	120000	-0.06116	0.52438	ETV3	76353	25kb
ENSG00000143297	1	157458167	157547310	323	28	120000	-0.68998	0.7549	FCRL5	76353	25kb
ENSG00000163518	1	157518539	157592870	234	17	120000	-0.59214	0.72312	FCRL4	76353	25kb
ENSG00000160856	1	157619111	157695647	202	20	120000	-0.62848	0.73515	FCRL3	76353	25kb
ENSG00000132704	1	157690523	157771922	151	23	120000	-0.48529	0.68627	FCRL2	76353	25kb
ENSG00000163534	1	157739193	157814895	142	16	120000	-0.91321	0.81943	FCRL1	76353	25kb
ENSG00000073754	1	157775704	157893046	352	26	120000	-1.077	0.85927	CD5L	76353	25kb
ENSG00000183853	1	157938063	158095052	490	87	120000	-1.8272	0.96617	KIRREL	76353	25kb
ENSG00000158473	1	158124737	158179686	192	14	120000	1.4873	0.068462	CD1D	76353	25kb
ENSG00000158477	1	158198927	158253059	115	18	120000	0.98763	0.16167	CD1A	76353	25kb
ENSG00000158481	1	158234576	158288420	79	15	120000	-1.1509	0.87511	CD1C	76353	25kb
ENSG00000158485	1	158272741	158326321	114	18	120000	-1.8538	0.96812	CD1B	76353	25kb
ENSG00000158488	1	158298254	158352343	198	17	120000	-1.9419	0.97393	CD1E	76353	25kb
ENSG00000186306	1	158343312	158394256	276	25	120000	-0.6226	0.73323	OR10T2	76353	25kb
ENSG00000180708	1	158364718	158415656	229	23	120000	1.255	0.10474	OR10K2	76353	25kb
ENSG00000173285	1	158410272	158461344	185	17	120000	0.72539	0.23411	OR10K1	76353	25kb
ENSG00000198965	1	158424668	158475675	217	14	120000	-0.21109	0.58359	OR10R2	76353	25kb
ENSG00000197532	1	158491918	158542895	228	11	120000	-0.84774	0.80171	OR6Y1	76353	25kb
ENSG00000186440	1	158507441	158558394	166	16	120000	-0.45744	0.67632	OR6P1	76353	25kb
ENSG00000186400	1	158523709	158574689	145	16	120000	-0.14717	0.5585	OR10X1	76353	25kb
ENSG00000198967	1	158551229	158602170	165	17	120000	0.060286	0.47596	OR10Z1	76353	25kb
ENSG00000163554	1	158555496	158681488	307	26	120000	-0.078817	0.53141	SPTA1	76353	25kb
ENSG00000196171	1	158644369	158695486	109	12	120000	-0.014727	0.50588	OR6K2	76353	25kb
ENSG00000203757	1	158661958	158712953	146	13	120000	-0.54519	0.70719	OR6K3	76353	25kb
ENSG00000180433	1	158699510	158750681	231	25	120000	-1.6075	0.94603	OR6K6	76353	25kb
ENSG00000197403	1	158710466	158761492	204	32	120000	-2.1325	0.98352	OR6N1	76353	25kb
ENSG00000188340	1	158721472	158772425	191	38	120000	-2.4849	0.99352	OR6N2	76353	25kb
ENSG00000163563	1	158776107	158844296	241	22	120000	-0.83383	0.79781	MNDA	76353	25kb
ENSG00000163564	1	158875586	158971844	357	38	120000	-1.0909	0.86235	PYHIN1	76353	25kb
ENSG00000163565	1	158944758	159049945	433	28	120000	0.33942	0.36714	IFI16	76353	25kb
ENSG00000163568	1	159007274	159141886	268	32	120000	0.19656	0.42209	AIM2	76353	25kb
ENSG00000162706	1	159116399	159198103	189	39	120000	-0.80985	0.79099	CADM3	76353	25kb
ENSG00000213088	1	159148097	159201290	151	31	120000	-1.0743	0.85866	DARC	76353	25kb
ENSG00000179639	1	159234504	159303014	186	24	120000	-1.5739	0.94225	FCER1A	76353	25kb
ENSG00000196266	1	159258460	159309449	137	18	120000	-1.5085	0.93428	OR10J3	76353	25kb
ENSG00000196184	1	159384512	159435600	134	22	120000	-1.9746	0.97584	OR10J1	76353	25kb
ENSG00000184155	1	159479793	159530842	109	15	120000	-0.80728	0.79025	OR10J5	76353	25kb
ENSG00000132703	1	159532615	159583655	165	23	120000	1.6096	0.053739	APCS	76353	25kb
ENSG00000132693	1	159657079	159709379	111	15	120000	0.74155	0.22918	CRP	76353	25kb
ENSG00000158716	1	159725722	159777333	136	31	120000	2.6623	0.0038802	DUSP23	76353	25kb
ENSG00000181036	1	159745301	159811041	201	29	120000	2.5682	0.0051112	FCRL6	76353	25kb
ENSG00000158714	1	159771540	159832039	196	34	120000	2.2256	0.01302	SLAMF8	76353	25kb
ENSG00000188004	1	159779264	159850137	226	40	120000	1.9052	0.028375	C1orf204	76353	25kb
ENSG00000243284	1	159799106	159857447	174	27	120000	0.64666	0.25893	VSIG8	76353	25kb
ENSG00000256029	1	159802690	159867843	196	28	120000	0.42377	0.33587	RP11-190A12.7	76353	25kb
ENSG00000213085	1	159817154	159894953	223	29	120000	0.16646	0.4339	CCDC19	76353	25kb
ENSG00000269620	1	159854884	159905159	136	32	120000	-0.14695	0.55842	AL590560.1	76353	25kb
ENSG00000158710	1	159862897	159920522	158	41	120000	-0.17822	0.57073	TAGLN2	76353	25kb
ENSG00000085552	1	159871829	159940386	200	48	120000	-0.737	0.76944	IGSF9	76353	25kb
ENSG00000162723	1	159896282	159949044	173	33	120000	-0.72057	0.76441	SLAMF9	76353	25kb
ENSG00000143315	1	159972462	160026783	151	20	120000	-0.27091	0.60677	PIGM	76353	25kb
ENSG00000177807	1	159982257	160065038	209	28	120000	-0.94881	0.82864	KCNJ10	76353	25kb
ENSG00000162728	1	160026360	160085353	144	19	120000	-0.70148	0.7585	KCNJ9	76353	25kb
ENSG00000162729	1	160036130	160093733	140	20	120000	-0.74178	0.77089	IGSF8	76353	25kb
ENSG00000018625	1	160060549	160138381	243	41	120000	-0.68637	0.75376	ATP1A2	76353	25kb
ENSG00000132681	1	160096360	160181767	273	52	120000	-0.37187	0.64501	ATP1A4	76353	25kb
ENSG00000143318	1	160135285	160196676	152	38	120000	-0.079919	0.53185	CASQ1	76353	25kb
ENSG00000162734	1	160150127	160210166	92	27	120000	-0.62171	0.73293	PEA15	76353	25kb
ENSG00000132716	1	160160505	160279920	163	19	120000	-1.7611	0.96089	DCAF8	76353	25kb
ENSG00000258465	1	160162254	160279920	161	19	120000	-1.7719	0.96179	DCAF8	76353	25kb
ENSG00000162735	1	160221602	160281138	80	11	120000	-1.9215	0.97267	PEX19	76353	25kb
ENSG00000122218	1	160234063	160338190	175	22	120000	-1.9047	0.97159	COPA	76353	25kb
ENSG00000162736	1	160288062	160353742	148	23	120000	-1.9359	0.97356	NCSTN	76353	25kb
ENSG00000171786	1	160311857	160367638	146	20	120000	-1.4731	0.92964	NHLH1	76353	25kb
ENSG00000162738	1	160345376	160423468	184	21	120000	-0.90135	0.8163	VANGL2	76353	25kb
ENSG00000162739	1	160429820	160518052	301	30	120000	-0.61493	0.7307	SLAMF6	76353	25kb
ENSG00000066294	1	160485885	160574306	364	22	120000	-1.4119	0.92101	CD84	76353	25kb
ENSG00000117090	1	160552890	160642085	325	41	120000	-2.0967	0.98199	SLAMF1	76353	25kb
ENSG00000117091	1	160623536	160706641	287	50	120000	-0.64701	0.74119	CD48	76353	25kb
ENSG00000026751	1	160684037	160749611	150	26	120000	0.22429	0.41126	SLAMF7	76353	25kb
ENSG00000122224	1	160740864	160823051	182	20	120000	-0.4764	0.6831	LY9	76353	25kb
ENSG00000122223	1	160774950	160857692	251	19	120000	1.2041	0.11427	CD244	76353	25kb
ENSG00000179914	1	160821329	160879960	192	11	120000	1.5625	0.059087	ITLN1	76353	25kb
ENSG00000158764	1	160889815	160949589	220	24	120000	0.34804	0.36391	ITLN2	76353	25kb
ENSG00000158769	1	160940001	161016138	215	19	120000	-0.83294	0.79756	F11R	76353	25kb
ENSG00000270149	1	160942747	161033752	238	22	120000	-0.78854	0.78481	RP11-544M22.13	76353	25kb
ENSG00000215845	1	160982421	161033780	114	18	120000	-0.94814	0.82847	TSTD1	76353	25kb
ENSG00000158773	1	160984041	161040767	122	19	120000	-0.88905	0.81301	USF1	76353	25kb
ENSG00000186517	1	160991736	161064760	157	29	120000	-0.83662	0.7986	ARHGAP30	76353	25kb
ENSG00000268154	1	161010655	161060943	111	22	120000	-0.54789	0.70812	AL591806.1	76353	25kb

ENSG00000143217	1	161015785	161084389	140	33	120000	0.19419	0.42301	PVRL4	76353	25kb
ENSG00000162755	1	161043151	161095136	105	26	120000	0.77683	0.21863	KLHDC9	76353	25kb
ENSG00000143256	1	161045346	161112901	120	25	120000	0.82523	0.20462	PFDN2	76353	25kb
ENSG00000158793	1	161062876	161120235	94	16	120000	1.0915	0.13753	NIT1	76353	25kb
ENSG00000158796	1	161065764	161127478	101	15	120000	1.0427	0.14854	DEDD	76353	25kb
ENSG00000143222	1	161097566	161153646	83	14	120000	0.87739	0.19014	UFC1	76353	25kb
ENSG00000143258	1	161104240	161160513	83	15	120000	0.69582	0.24327	USP21	76353	25kb
ENSG00000143224	1	161111200	161172803	102	19	120000	0.90485	0.18277	PPOX	76353	25kb
ENSG00000158850	1	161116100	161172287	89	19	120000	0.96326	0.16771	B4GALT3	76353	25kb
ENSG00000158859	1	161129098	161193846	123	27	120000	1.0422	0.14867	ADAMTS4	76353	25kb
ENSG00000158864	1	161141894	161209185	143	32	120000	1.4437	0.074409	NDUFS2	76353	25kb
ENSG00000158869	1	161160024	161215489	138	28	120000	1.9693	0.024462	FCER1G	76353	25kb
ENSG00000268387	1	161165160	161217296	130	26	120000	1.9972	0.022902	AL590714.1	76353	25kb
ENSG00000158874	1	161167082	161218421	132	25	120000	2.0422	0.020564	APOA2	76353	25kb
ENSG00000158882	1	161170793	161225408	151	25	120000	1.9979	0.022862	TOMM40L	76353	25kb
ENSG00000143257	1	161174456	161233092	169	27	120000	2.0031	0.022581	NR1I3	76353	25kb
ENSG00000248485	1	161203517	161280240	249	38	120000	1.981	0.023794	PCP4L1	76353	25kb
ENSG00000158887	1	161249525	161304762	196	24	120000	0.18688	0.42588	MPZ	76353	25kb
ENSG00000143252	1	161259047	161357984	405	18	120000	-0.32098	0.62589	SDHC	76353	25kb
ENSG00000188931	1	161309521	161362664	226	9	120000	-0.61213	0.72977	C1orf192	76353	25kb
ENSG00000143226	1	161450220	161518803	165	29	120000	0.86604	0.19323	FCGR2A	76353	25kb
ENSG00000173110	1	161469036	161521681	130	25	120000	1.2949	0.097684	HSPA6	76353	25kb
ENSG00000203747	1	161486549	161625917	237	36	120000	1.1199	0.13137	FCGR3A	76353	25kb
ENSG00000072694	1	161526101	161673444	246	35	120000	-0.025956	0.51035	FCGR2B	76353	25kb
ENSG00000162747	1	161567986	161626753	131	17	120000	-0.33769	0.6322	FCGR3B	76353	25kb
ENSG00000132185	1	161651762	161709142	243	41	120000	-0.39518	0.65364	FCRLA	76353	25kb
ENSG00000162746	1	161666353	161722933	244	40	120000	0.089731	0.46425	FCRLB	76353	25kb
ENSG00000081721	1	161694548	161752028	214	33	120000	0.48689	0.31317	DUSP12	76353	25kb
ENSG00000118217	1	161711084	161958860	754	28	120000	0.36551	0.35737	ATF6	76353	25kb
ENSG00000162745	1	161927982	162018644	313	59	120000	0.23582	0.40679	OLFML2B	76353	25kb
ENSG00000198929	1	162014564	162378321	1337	81	120000	0.21171	0.41617	NOS1AP	76353	25kb
ENSG00000254706	1	162310197	162376905	237	53	120000	0.30684	0.37948	RP11-565P22.6	76353	25kb
ENSG00000239887	1	162311698	162381600	252	49	120000	0.71068	0.23864	C1orf226	76353	25kb
ENSG00000171722	1	162317993	162371660	185	44	120000	0.30183	0.38139	C1orf111	76353	25kb
ENSG00000198574	1	162340056	162406928	216	30	120000	1.2511	0.10545	SH2D1B	76353	25kb
ENSG00000152332	1	162442041	162524419	208	17	120000	0.75683	0.22458	UHMK1	76353	25kb
ENSG00000117143	1	162506323	162594627	171	31	120000	0.30448	0.38038	UAP1	76353	25kb
ENSG00000162733	1	162576163	162782190	526	39	120000	0.78549	0.21608	DDR2	76353	25kb
ENSG00000132196	1	162735492	162807607	180	28	120000	0.89509	0.18537	HSD17B7	76353	25kb
ENSG00000185860	1	162769248	162863605	329	39	120000	-0.55464	0.71043	C1orf110	76353	25kb
ENSG00000117152	1	163013565	163071592	194	28	120000	-2.8673	0.99793	RGS4	76353	25kb
ENSG00000143248	1	163055911	163316577	1002	56	120000	-0.70188	0.75862	RGS5	76353	25kb
ENSG00000143228	1	163211366	163350554	601	36	120000	0.13335	0.44696	NUF2	76353	25kb
ENSG00000185630	1	164499821	164893533	1120	133	120000	-1.6754	0.95307	PBX1	76353	25kb
ENSG00000162761	1	165146104	165350952	650	69	120000	0.32186	0.37378	LMX1A	76353	25kb
ENSG00000143171	1	165345159	165439433	358	44	120000	-0.95487	0.83018	RXRG	76353	25kb
ENSG00000162763	1	165488244	165558198	299	21	120000	0.37426	0.35411	LRRC52	76353	25kb
ENSG00000143198	1	165575098	165656033	357	33	120000	0.070669	0.47183	MGST3	76353	25kb
ENSG00000143149	1	165606453	165693100	356	34	120000	-0.99726	0.84068	ALDH9A1	76353	25kb
ENSG00000143183	1	165671032	165821992	306	37	120000	-0.78959	0.78512	TMCO1	76353	25kb
ENSG00000143179	1	165771768	165905855	255	34	120000	-0.35966	0.64045	UCK2	76353	25kb
ENSG00000188859	1	166001674	166161206	439	31	120000	-0.9324	0.82444	FAM78B	76353	25kb
ENSG00000267884	1	166001679	166053709	152	23	120000	-1.3172	0.90611	AL626787.1	76353	25kb
ENSG00000143157	1	166783681	166850581	170	20	120000	0.187	0.42583	POGK	76353	25kb
ENSG00000152382	1	166800747	166870564	146	17	120000	0.30568	0.37992	TADA1	76353	25kb
ENSG00000143195	1	166857443	166969719	228	29	120000	0.38276	0.35095	ILDR2	76353	25kb
ENSG00000143194	1	166933346	167016451	221	29	120000	1.1979	0.11547	MAEL	76353	25kb
ENSG00000143167	1	166997073	167084868	302	33	120000	1.4807	0.069337	GPA33	76353	25kb
ENSG00000198842	1	167038282	167123402	320	40	120000	1.2285	0.10964	DUSP27	76353	25kb
ENSG00000143190	1	167165066	167421582	533	35	120000	-0.5087	0.69452	POU2F1	76353	25kb
ENSG00000198821	1	167374877	167512847	474	95	120000	0.6	0.27425	CD247	76353	25kb
ENSG00000143162	1	167473914	167548004	328	46	120000	0.59657	0.2754	CREG1	76353	25kb
ENSG00000198771	1	167574330	167700486	426	62	120000	-0.033254	0.51326	RCSD1	76353	25kb
ENSG00000197965	1	167665429	167786156	344	35	120000	1.5141	0.065004	MPZL1	76353	25kb
ENSG00000143199	1	167753625	167908453	471	61	120000	-0.057886	0.52308	ADCY10	76353	25kb
ENSG00000143158	1	167860967	167931278	112	11	120000	-1.0241	0.84711	MPC2	76353	25kb
ENSG00000143164	1	167880021	168070081	258	17	120000	-1.2213	0.88902	DCAF6	76353	25kb
ENSG00000143147	1	168028997	168131821	195	24	120000	0.31174	0.37762	GPR161	76353	25kb
ENSG00000143155	1	168123171	168194950	202	23	120000	1.1618	0.12265	TIPRL	76353	25kb
ENSG00000213064	1	168170176	168247263	180	21	120000	1.0314	0.15117	SFT2D2	76353	25kb
ENSG00000143178	1	168225278	168308664	223	32	120000	-0.13911	0.55532	TBX19	76353	25kb
ENSG00000143185	1	168485003	168538235	147	18	120000	-0.48263	0.68532	XCL2	76353	25kb
ENSG00000143184	1	168520711	168576315	173	23	120000	1.2129	0.11259	XCL1	76353	25kb
ENSG00000143196	1	168639697	168723502	343	37	120000	-1.5906	0.94416	DPT	76353	25kb
ENSG00000143153	1	169049935	169126960	287	33	120000	-0.56146	0.71276	ATP1B1	76353	25kb
ENSG00000143156	1	169076769	169362205	992	27	120000	0.77949	0.21785	NME7	76353	25kb
ENSG00000117475	1	169312208	169390778	245	25	120000	1.2651	0.10291	BLZF1	76353	25kb
ENSG00000117477	1	169339108	169454907	316	22	120000	1.2367	0.1081	CCDC181	76353	25kb
ENSG00000117479	1	169408147	169480241	187	33	120000	1.289	0.098691	SLC19A2	76353	25kb
ENSG00000198734	1	169458404	169580826	504	35	120000	-0.55501	0.71056	F5	76353	25kb
ENSG00000174175	1	169533087	169624431	304	29	120000	-1.698	0.95525	SELP	76353	25kb
ENSG00000000460	1	169606245	169848221	1024	31	120000	0.98983	0.16113	C1orf112	76353	25kb
ENSG00000188404	1	169634808	169705839	318	31	120000	0.76387	0.22247	SELL	76353	25kb
ENSG00000007908	1	169666781	169758846	496	21	120000	1.2505	0.10556	SELE	76353	25kb
ENSG00000171806	1	169736670	169789107	182	20	120000	1.1278	0.1297	METTL18	76353	25kb
ENSG00000000457	1	169793772	169888408	307	16	120000	1.0829	0.13942	SCYL3	76353	25kb
ENSG00000075945	1	169865467	170079349	608	16	120000	1.1687	0.12126	KIFAP3	76353	25kb
ENSG00000203740	1	170090142	170161931	254	21	120000	0.18755	0.42561	METTL11B	76353	25kb
ENSG00000120370	1	170476270	170547587	113	19	120000	1.3955	0.081438	GORAB	76353	25kb
ENSG00000116132	1	170606869	170733560	284	33	120000	-0.12814	0.55098	PRRX1	76353	25kb
ENSG00000117501	1	170879612	171058906	623	39	120000	-0.97316	0.83476	MROH9	76353	25kb
ENSG00000007933	1	171035018	171111959	275	24	120000	-0.39491	0.65354	FMO3	76353	25kb
ENSG00000117507	1	171081879	171155707	290	25	120000	0.80914	0.20922	FMO6P	76353	25kb
ENSG00000094963	1	171129347	171206822	354	22	120000	1.7008	0.044495	FMO2	76353	25kb
ENSG00000010932	1	171192638	171280117	258	27	120000	0.33947	0.36713	FMO1	76353	25kb
ENSG00000076258	1	171258347	171336223	141	13	120000	0.44723	0.32735	FMO4	76353	25kb

ENSG00000117523	1	171429651	171587650	390	25	120000	1.1076	0.13401	PRRC2C	76353	25kb
ENSG00000034971	1	171579557	171646823	218	28	120000	0.17129	0.432	MYOC	76353	25kb
ENSG00000117533	1	171644300	171736387	311	14	120000	1.0938	0.13702	VAMP4	76353	25kb
ENSG00000010165	1	171725788	171808163	303	14	120000	0.11032	0.45608	METTL13	76353	25kb
ENSG00000197959	1	171785621	172412606	1432	77	120000	0.31486	0.37643	DNM3	76353	25kb
ENSG00000135845	1	172314329	172438230	310	27	120000	-0.49654	0.69024	PIGC	76353	25kb
ENSG00000180999	1	172364828	172462971	284	26	120000	-0.26454	0.60432	C1orf105	76353	25kb
ENSG00000094975	1	172476489	172605971	200	21	120000	0.85597	0.19601	SUCO	76353	25kb
ENSG00000117560	1	172603154	172661014	138	19	120000	1.1857	0.11787	FASLG	76353	25kb
ENSG00000120337	1	172984100	173045103	97	17	120000	-0.27204	0.60721	TNFSF18	76353	25kb
ENSG00000117586	1	173127873	173201452	187	21	120000	0.11088	0.45586	TNFSF4	76353	25kb
ENSG00000117592	1	173421405	173482946	116	15	120000	1.0853	0.1389	PRDX6	76353	25kb
ENSG00000162753	1	173444603	173597233	289	15	120000	2.0234	0.021518	SLC9C2	76353	25kb
ENSG00000183831	1	173553700	173664001	107	17	120000	0.65419	0.25649	ANKRD45	76353	25kb
ENSG00000076321	1	173659080	173780840	140	16	120000	-0.12809	0.55096	KLHL20	76353	25kb
ENSG00000120334	1	173743688	173818858	79	12	120000	-0.12875	0.55122	CENPL	76353	25kb
ENSG00000117593	1	173768641	173852684	116	15	120000	-0.45421	0.67516	DARS2	76353	25kb
ENSG00000185278	1	173812220	173897687	174	16	120000	-0.51176	0.69559	ZBTB37	76353	25kb
ENSG00000117601	1	173847947	173911516	134	13	120000	-0.35321	0.63804	SERPINC1	76353	25kb
ENSG00000135870	1	173875352	174016435	251	14	120000	-0.074883	0.52985	RC3H1	76353	25kb
ENSG00000152061	1	174103548	174989445	1723	26	120000	-0.16337	0.56489	RABGAP1L	76353	25kb
ENSG00000203737	1	174392212	174443683	88	12	120000	-0.60013	0.72579	GPR52	76353	25kb
ENSG00000116161	1	174943300	175005851	97	19	120000	0.90171	0.18361	CACYBP	76353	25kb
ENSG00000120333	1	174954925	175017561	116	23	120000	0.95939	0.16868	MRPS14	76353	25kb
ENSG00000120332	1	175011994	175142202	445	37	120000	1.1964	0.11577	TNN	76353	25kb
ENSG00000235750	1	175101123	175187135	279	29	120000	1.1275	0.12978	KIAA0040	76353	25kb
ENSG00000116147	1	175259330	175737906	1386	128	120000	0.072726	0.47101	TNR	76353	25kb
ENSG00000143207	1	175888967	176201629	784	32	120000	-0.40213	0.6562	RFWD2	76353	25kb
ENSG00000116183	1	176407307	176839735	912	44	120000	0.69697	0.24291	PAPPA2	76353	25kb
ENSG00000152092	1	176801438	177159109	854	61	120000	-0.058514	0.52333	ASTN1	76353	25kb
ENSG00000198797	1	177115633	177276558	308	32	120000	0.27287	0.39248	BRINP2	76353	25kb
ENSG00000120341	1	177868091	177978438	412	48	120000	-0.16485	0.56547	SEC16B	76353	25kb
ENSG00000075391	1	178037864	178473644	709	29	120000	1.2958	0.097524	RASAL2	76353	25kb
ENSG00000240021	1	178457212	178542734	229	20	120000	-1.9848	0.97642	TEX35	76353	25kb
ENSG00000213057	1	178486887	178543024	168	12	120000	-2.0697	0.98076	C1orf220	76353	25kb
ENSG00000184909	1	178486950	178542579	167	12	120000	-2.0769	0.98109	C1ORF220	76353	25kb
ENSG00000116191	1	178669300	178914238	517	28	120000	-1.4185	0.92197	RALGPS2	76353	25kb
ENSG00000116194	1	178793840	178865187	132	18	120000	-1.3386	0.90966	ANGPTL1	76353	25kb
ENSG00000116199	1	178969939	179070697	296	23	120000	0.42578	0.33513	FAM20B	76353	25kb
ENSG00000186283	1	179025512	179092158	191	27	120000	0.32119	0.37403	TOR3A	76353	25kb
ENSG00000143322	1	179043462	179223819	419	26	120000	-0.00063975	0.50026	ABL2	76353	25kb
ENSG00000057252	1	179237925	179352815	487	30	120000	1.3948	0.081531	SOAT1	76353	25kb
ENSG00000162779	1	179309855	179548870	1208	25	120000	-0.38844	0.65116	AXDND1	76353	25kb
ENSG00000269064	1	179418716	179482805	412	19	120000	-0.55831	0.71168	AL160286.1	76353	25kb
ENSG00000116218	1	179494674	179570087	267	21	120000	-1.3199	0.90657	NPHS2	76353	25kb
ENSG00000162782	1	179535748	179685407	455	28	120000	-1.445	0.92577	TDRD5	76353	25kb
ENSG00000143340	1	179687298	179810333	391	40	120000	0.20011	0.4207	FAM163A	76353	25kb
ENSG00000169905	1	179784102	179871938	218	24	120000	0.22136	0.4124	TOR1AIP2	76353	25kb
ENSG00000258664	1	179808916	179859311	95	12	120000	-0.59443	0.72389	IFRG15	76353	25kb
ENSG00000143337	1	179826177	179919135	223	24	120000	-0.53148	0.70246	TOR1AIP1	76353	25kb
ENSG00000135837	1	179898873	180109015	538	19	120000	-0.73678	0.76937	CEP350	76353	25kb
ENSG00000116260	1	180098969	180198165	398	37	120000	0.19714	0.42186	QSOX1	76353	25kb
ENSG00000121454	1	180174421	180274380	330	51	120000	-1.3799	0.9162	LHX4	76353	25kb
ENSG00000135847	1	180219515	180497089	753	34	120000	-1.1965	0.88425	ACBD6	76353	25kb
ENSG00000143324	1	180576140	180884387	552	30	120000	-0.82945	0.79658	XPR1	76353	25kb
ENSG00000135835	1	180857290	180945750	225	47	120000	-0.77362	0.78042	KIAA1614	76353	25kb
ENSG00000234237	1	180916695	180974868	125	17	120000	-0.22629	0.58951	AL162431.1	76353	25kb
ENSG00000135823	1	180916861	181017047	211	28	120000	-0.62914	0.73537	STX6	76353	25kb
ENSG00000153029	1	180978067	181056074	236	37	120000	-0.84778	0.80172	MR1	76353	25kb
ENSG00000162783	1	181032638	181084977	160	25	120000	-0.13491	0.55366	IER5	76353	25kb
ENSG00000198216	1	181357238	181802219	1204	101	120000	0.67337	0.25036	CACNA1E	76353	25kb
ENSG00000179930	1	181998705	182055847	184	19	120000	-0.76566	0.77806	ZNF648	76353	25kb
ENSG00000135821	1	182325839	182386341	200	21	120000	0.75405	0.22541	GLUL	76353	25kb
ENSG00000203730	1	182342252	182394751	151	17	120000	0.7293	0.23291	TEDDM1	76353	25kb
ENSG00000121446	1	182394256	182554734	465	28	120000	0.033806	0.48652	RGSL1	76353	25kb
ENSG00000135828	1	182517769	182583391	159	26	120000	-0.45432	0.6752	RNASEL	76353	25kb
ENSG00000143333	1	182542758	182598543	116	23	120000	-0.89367	0.81425	RGS16	76353	25kb
ENSG00000135824	1	182590239	182678711	234	37	120000	-0.6922	0.75559	RGS8	76353	25kb
ENSG00000135838	1	182733428	182824519	150	23	120000	-0.85304	0.80318	NPL	76353	25kb
ENSG00000135829	1	182783504	182881886	128	12	120000	-0.30502	0.61982	DHX9	76353	25kb
ENSG00000157060	1	182844000	182947660	187	25	120000	0.60891	0.27129	SHCBP1L	76353	25kb
ENSG00000135862	1	182967595	183139727	628	13	120000	0.070065	0.47207	LAMC1	76353	25kb
ENSG00000058085	1	183130373	183239035	367	26	120000	1.0973	0.13626	LAMC2	76353	25kb
ENSG00000157064	1	183192372	183412737	563	33	120000	1.8038	0.035628	NMNAT2	76353	25kb
ENSG00000116698	1	183416351	183592381	322	33	120000	-0.6808	0.752	SMG7	76353	25kb
ENSG00000116701	1	183499698	183585011	197	25	120000	-1.1227	0.86921	NCF2	76353	25kb
ENSG00000162704	1	183567401	183629892	248	7	120000	-1.192	0.88337	ARPC5	76353	25kb
ENSG00000143344	1	183580220	183922665	1015	46	120000	0.38173	0.35133	RGL1	76353	25kb
ENSG00000173627	1	183590541	183647451	253	5	120000	-1.0703	0.85776	APOBEC4	76353	25kb
ENSG00000198756	1	183873796	184031863	438	45	120000	0.58192	0.28031	COLGALT2	76353	25kb
ENSG00000198860	1	183995811	184068346	119	20	120000	0.1503	0.44026	TSEN15	76353	25kb
ENSG00000116667	1	184331192	184623154	716	48	120000	-0.016733	0.50668	C1orf21	76353	25kb
ENSG00000116406	1	184634365	184749047	226	25	120000	0.055614	0.47782	EDEM3	76353	25kb
ENSG00000135842	1	184734858	184968682	508	38	120000	-0.10325	0.54112	FAM129A	76353	25kb
ENSG00000121481	1	184989496	185096740	129	18	120000	-0.14494	0.55762	RNF2	76353	25kb
ENSG00000121486	1	185062220	185151204	130	11	120000	0.16774	0.43339	TRMT1L	76353	25kb
ENSG00000116668	1	185101212	185285897	220	21	120000	-0.3626	0.64155	SWT1	76353	25kb
ENSG00000116679	1	185240520	185311461	79	13	120000	-1.6181	0.94718	IVNS1ABP	76353	25kb
ENSG00000143341	1	185678683	186185085	721	45	120000	-1.2587	0.89593	HMCN1	76353	25kb
ENSG00000116690	1	186240405	186308694	124	19	120000	0.31196	0.37754	PRG4	76353	25kb
ENSG00000047410	1	186255784	186369825	220	21	120000	0.28404	0.38819	TPR	76353	25kb
ENSG00000157181	1	186319890	186415510	180	26	120000	0.49247	0.31119	C1orf27	76353	25kb
ENSG00000268662	1	186339123	186390908	96	17	120000	0.73035	0.23259	AL596220.1	76353	25kb
ENSG00000262180	1	186344704	186395587	98	18	120000	0.79632	0.21292	OCLM	76353	25kb
ENSG00000116703	1	186387698	186455254	124	13	120000	0.50117	0.30812	PDC	76353	25kb
ENSG00000073756	1	186615923	186674559	188	15	120000	0.13289	0.44714	PTGS2	76353	25kb

ENSG00000116711	1	186773085	186983113	627	59	120000	-0.97492	0.8352	PLA2G4A	76353	25kb
ENSG00000162670	1	190041792	190471759	871	43	120000	-1.0846	0.86095	BRINP3	76353	25kb
ENSG00000150681	1	192102587	192179945	151	19	120000	-0.19489	0.57726	RGS18	76353	25kb
ENSG00000253148	1	192261122	192361415	237	26	120000	0.9671	0.16675	RGS21	76353	25kb
ENSG00000090104	1	192519857	192574161	170	30	120000	1.1541	0.12422	RGS1	76353	25kb
ENSG00000127074	1	192580275	192654390	217	23	120000	0.18596	0.42624	RGS13	76353	25kb
ENSG00000116741	1	192753169	192806403	136	21	120000	1.4163	0.078346	RGS2	76353	25kb
ENSG00000116750	1	192956380	193054237	140	18	120000	0.91079	0.1812	UCHL5	76353	25kb
ENSG00000116747	1	193003552	193085907	150	15	120000	0.88486	0.18812	TROVE2	76353	25kb
ENSG00000023572	1	193040598	193100244	116	12	120000	0.77409	0.21944	GLRX2	76353	25kb
ENSG00000134371	1	193066147	193248031	420	18	120000	0.72003	0.23575	CDC73	76353	25kb
ENSG00000162630	1	193123175	193180784	133	14	120000	0.5729	0.28336	B3GALT2	76353	25kb
ENSG00000162687	1	196169909	196603355	756	23	120000	-0.1823	0.57233	KCNT2	76353	25kb
ENSG00000000971	1	196596008	196741634	256	16	120000	-1.1334	0.87147	CFH	76353	25kb
ENSG00000116785	1	196718925	196788203	37	5	120000	-0.47106	0.6812	CFHR3	76353	25kb
ENSG00000244414	1	196763887	196826319	58	10	120000	-1.427	0.92321	CFHR1	76353	25kb
ENSG00000080910	1	196763898	196953356	328	20	120000	-1.558	0.94039	CFHR2	76353	25kb
ENSG00000134365	1	196794371	196913102	219	20	120000	-1.5776	0.94267	CFHR4	76353	25kb
ENSG00000134389	1	196921667	197003804	150	19	120000	-1.0323	0.84903	CFHR5	76353	25kb
ENSG00000143278	1	196983321	197061397	160	14	120000	-1.005	0.84256	F13B	76353	25kb
ENSG00000066279	1	197028258	197140824	195	15	120000	-0.95627	0.83053	ASPM	76353	25kb
ENSG00000177888	1	197097810	197194672	158	18	120000	-0.76426	0.77764	ZBTB41	76353	25kb
ENSG00000134376	1	197145592	197472585	530	40	120000	0.72035	0.23566	CRB1	76353	25kb
ENSG00000213047	1	197448878	197769826	532	20	120000	0.79858	0.21227	DENND1B	76353	25kb
ENSG00000203724	1	197846777	197901497	112	29	120000	1.4535	0.073046	C1orf53	76353	25kb
ENSG00000143355	1	197856037	197929608	174	30	120000	1.596	0.055242	LHX9	76353	25kb
ENSG00000151414	1	198101093	198316550	597	25	120000	-0.42546	0.66475	NEK7	76353	25kb
ENSG00000151418	1	198467352	198535075	191	20	120000	0.59366	0.27637	ATP6V1G3	76353	25kb
ENSG00000081237	1	198582801	198751545	502	36	120000	-1.1929	0.88355	PTPRC	76353	25kb
ENSG00000116833	1	199971730	200171552	673	74	120000	2.7946	0.0025979	NR5A2	76353	25kb
ENSG00000162702	1	200349068	200404184	94	14	120000	0.10567	0.45792	ZNF281	76353	25kb
ENSG00000118193	1	200495628	200614862	401	31	120000	0.84243	0.19977	KIF14	76353	25kb
ENSG00000118197	1	200568024	200664097	329	25	120000	0.1877	0.42556	DDX59	76353	25kb
ENSG00000118200	1	200683686	200854832	396	25	120000	-0.79614	0.78702	CAMSAP2	76353	25kb
ENSG00000170128	1	200817083	200868306	146	26	120000	-0.72575	0.766	GPR25	76353	25kb
ENSG00000163362	1	200835176	200909863	233	44	120000	-1.2392	0.89236	C1orf106	76353	25kb
ENSG00000116852	1	200913520	201017828	260	21	120000	-1.6257	0.948	KIF21B	76353	25kb
ENSG00000081248	1	200983642	201106694	402	60	120000	-0.53991	0.70537	CACNA1S	76353	25kb
ENSG00000232237	1	201058081	201121312	253	35	120000	0.65177	0.25727	ASCL5	76353	25kb
ENSG00000116857	1	201078900	201165702	327	43	120000	0.045184	0.48198	TMEM9	76353	25kb
ENSG00000163395	1	201134953	201223080	361	43	120000	-0.19272	0.57641	IGFN1	76353	25kb
ENSG00000081277	1	201227580	201327121	362	28	120000	0.93193	0.17569	PKP1	76353	25kb
ENSG00000118194	1	201303136	201371890	234	34	120000	0.17252	0.43151	TNNT2	76353	25kb
ENSG00000159166	1	201317372	201393736	239	41	120000	0.13386	0.44676	LAD1	76353	25kb
ENSG00000159173	1	201348625	201423994	234	42	120000	1.6309	0.051461	TNNI1	76353	25kb
ENSG00000174307	1	201409620	201463365	216	34	120000	1.5855	0.056425	PHLDA3	76353	25kb
ENSG00000159176	1	201427658	201503584	343	32	120000	0.98303	0.1628	CSRP1	76353	25kb
ENSG00000269690	1	201567013	201617738	125	30	120000	-0.19764	0.57834	AC096677.1	76353	25kb
ENSG00000134369	1	201567411	201821102	583	68	120000	-1.8024	0.96426	NAV1	76353	25kb
ENSG00000198700	1	201773269	201878422	169	23	120000	-0.23534	0.59303	IPO9	76353	25kb
ENSG00000198892	1	201832808	201886434	99	14	120000	-0.029949	0.51195	SHISA4	76353	25kb
ENSG00000163431	1	201840580	201940715	220	41	120000	-0.80554	0.78975	LMOD1	76353	25kb
ENSG00000134375	1	201899619	201964789	229	35	120000	0.18447	0.42682	TIMM17A	76353	25kb
ENSG00000176393	1	201926500	202000275	287	28	120000	1.0106	0.1561	RNPEP	76353	25kb
ENSG00000163435	1	201952073	202011316	209	31	120000	0.93158	0.17578	ELF3	76353	25kb
ENSG00000170075	1	202066986	202127720	199	39	120000	0.18145	0.42801	GPR37L1	76353	25kb
ENSG00000143862	1	202077532	202138869	217	50	120000	-0.11974	0.54766	ARL8A	76353	25kb
ENSG00000143851	1	202091141	202155716	237	45	120000	-0.10833	0.54313	PTPN7	76353	25kb
ENSG00000133067	1	202138029	202313909	448	45	120000	-1.1061	0.86566	LGR6	76353	25kb
ENSG00000077152	1	202275785	202336108	109	15	120000	-0.19508	0.57733	UBE2T	76353	25kb
ENSG00000077157	1	202292827	202586834	388	22	120000	-0.38863	0.65122	PPP1R12B	76353	25kb
ENSG00000143858	1	202534724	202704545	371	49	120000	-1.4303	0.92368	SYT2	76353	25kb
ENSG00000117139	1	202671526	202803598	339	23	120000	-0.90187	0.81644	KDM5B	76353	25kb
ENSG00000184774	1	202764394	202821353	153	18	120000	-1.1812	0.88125	RP11-480I12.4	76353	25kb
ENSG00000183155	1	202823085	202883263	121	14	120000	-1.1716	0.87933	RABIF	76353	25kb
ENSG00000117153	1	202835228	202922764	207	20	120000	-1.3034	0.90378	KLHL12	76353	25kb
ENSG00000159346	1	202884951	202952700	174	26	120000	-2.1953	0.98593	ADIPOR1	76353	25kb
ENSG00000159348	1	202905997	202961408	132	23	120000	-2.7499	0.99702	CYB5R1	76353	25kb
ENSG00000163444	1	202951514	203018976	151	31	120000	-1.6085	0.94614	TMEM183A	76353	25kb
ENSG00000143847	1	202970626	203072868	264	37	120000	-0.75829	0.77586	PPFIA4	76353	25kb
ENSG00000122180	1	203027260	203080164	138	26	120000	0.0092627	0.4963	MYOG	76353	25kb
ENSG00000163485	1	203034782	203161533	344	50	120000	1.1078	0.13398	ADORA1	76353	25kb
ENSG00000133055	1	203111939	203169941	189	33	120000	1.2247	0.11034	MYBPH	76353	25kb
ENSG00000133048	1	203123059	203180877	200	38	120000	0.65421	0.25649	CHI3L1	76353	25kb
ENSG00000133063	1	203156955	203267769	332	41	120000	0.34067	0.36668	CHIT1	76353	25kb
ENSG00000159388	1	203249619	203303730	129	26	120000	1.4411	0.074782	BTG2	76353	25kb
ENSG00000122176	1	203284756	203345617	172	38	120000	0.10791	0.45703	FMOD	76353	25kb
ENSG00000188783	1	203419956	203485480	220	29	120000	1.9199	0.027434	PRELP	76353	25kb
ENSG00000188770	1	203438271	203502992	223	39	120000	2.039	0.020725	OPTC	76353	25kb
ENSG00000058668	1	203570689	203738209	437	63	120000	-0.77412	0.78057	ATP2B4	76353	25kb
ENSG00000122188	1	203709304	203770361	165	21	120000	-0.87875	0.81023	LAX1	76353	25kb
ENSG00000058673	1	203739782	203848252	329	18	120000	-0.82419	0.79508	ZC3H11A	76353	25kb
ENSG00000257315	1	203740437	203794686	178	17	120000	-0.99801	0.84086	ZBED6	76353	25kb
ENSG00000182004	1	203805731	203864678	156	14	120000	-0.1715	0.56808	SNRPE	76353	25kb
ENSG00000143842	1	204017243	204121863	285	54	120000	-0.24792	0.5979	SOX13	76353	25kb
ENSG00000143845	1	204075190	204146307	197	44	120000	0.18654	0.42601	ETNK2	76353	25kb
ENSG00000143839	1	204098944	204160465	183	33	120000	0.25936	0.39768	REN	76353	25kb
ENSG00000170498	1	204134469	204190614	162	29	120000	-0.21824	0.58638	KISS1	76353	25kb
ENSG00000174567	1	204142288	204208220	188	29	120000	0.34028	0.36682	GOLT1A	76353	25kb
ENSG00000143850	1	204162979	204371793	627	49	120000	-0.42798	0.66567	PLEKHA6	76353	25kb
ENSG00000158615	1	204347515	204405919	146	34	120000	-0.99572	0.84031	PPP1R15B	76353	25kb
ENSG00000133056	1	204366756	204488852	294	28	120000	-0.22625	0.5895	PIK3C2B	76353	25kb
ENSG00000198625	1	204460511	204567871	222	15	120000	-0.53907	0.70508	MDM4	76353	25kb
ENSG00000170382	1	204561298	204679861	294	32	120000	-1.265	0.89706	LRRN2	76353	25kb
ENSG00000163531	1	204772779	205016950	717	74	120000	-1.5285	0.9368	NFASC	76353	25kb
ENSG00000184144	1	204987325	205072627	251	36	120000	0.036049	0.48562	CNTN2	76353	25kb

ENSG00000174529	1	205027258	205078645	149	20	120000	-0.34522	0.63503	TMEM81	76353	25kb
ENSG00000117222	1	205030270	205116143	243	24	120000	-0.53139	0.70243	RBBP5	76353	25kb
ENSG00000133059	1	205086632	205205727	274	18	120000	-0.7348	0.76877	DSTYK	76353	25kb
ENSG00000133069	1	205172304	205267471	249	22	120000	-0.76531	0.77796	TMCC2	76353	25kb
ENSG00000163545	1	205246187	205315883	248	26	120000	-1.2474	0.89387	NUAK2	76353	25kb
ENSG00000162873	1	205280220	205351218	276	26	120000	-2.1236	0.98315	KLHDC8A	76353	25kb
ENSG00000186007	1	205325506	205450082	350	56	120000	-0.62302	0.73336	LEMD1	76353	25kb
ENSG00000117266	1	205448723	205526921	325	49	120000	0.17605	0.43013	CDK18	76353	25kb
ENSG00000174514	1	205513013	205597046	255	34	120000	1.2256	0.11017	MFSD4	76353	25kb
ENSG00000158711	1	205552071	205626090	242	34	120000	0.4036	0.34325	ELK4	76353	25kb
ENSG00000158715	1	205601979	205674587	215	38	120000	-0.37355	0.64563	SLC45A3	76353	25kb
ENSG00000069275	1	205656947	205744404	222	30	120000	-1.6009	0.9453	NUCKS1	76353	25kb
ENSG00000268313	1	205657497	205709153	108	22	120000	-1.2664	0.89732	AC119673.1	76353	25kb
ENSG00000117280	1	205712114	205769588	179	26	120000	-1.4219	0.92248	RAB7L1	76353	25kb
ENSG00000133065	1	205733221	205807876	201	30	120000	-0.55313	0.70991	SLC41A1	76353	25kb
ENSG00000162877	1	205772150	205844260	195	34	120000	-1.1383	0.8725	PM20D1	76353	25kb
ENSG00000174502	1	205857176	205937588	222	37	120000	-1.8094	0.96481	SLC26A9	76353	25kb
ENSG00000196550	1	206111916	206180151	6	3	120000	1.6821	0.046271	FAM72A	76353	25kb
ENSG00000198049	1	206198976	206256639	188	16	120000	1.0286	0.15184	AVPR1B	76353	25kb
ENSG00000196533	1	206213872	206313647	221	30	120000	1.2355	0.10832	C1orf186	76353	25kb
ENSG00000196188	1	206292459	206357104	77	8	120000	0.74875	0.22701	CTSE	76353	25kb
ENSG00000163486	1	206491200	206662783	283	29	120000	-0.9215	0.8216	SRGAP2	76353	25kb
ENSG00000143466	1	206618791	206695223	238	36	120000	-1.4648	0.92852	IKBKE	76353	25kb
ENSG00000162888	1	206639449	206696061	181	27	120000	-1.4718	0.92946	C1orf147	76353	25kb
ENSG00000136653	1	206655879	206787616	350	40	120000	-1.5817	0.94314	RASSF5	76353	25kb
ENSG00000143486	1	206719620	206810904	264	30	120000	-1.2849	0.90059	EIF2D	76353	25kb
ENSG00000143479	1	206783881	206882764	264	32	120000	0.33409	0.36916	DYRK3	76353	25kb
ENSG00000162889	1	206833289	206932628	265	28	120000	0.82729	0.20404	MAPKAPK2	76353	25kb
ENSG00000136634	1	206915947	206970839	139	29	120000	0.21012	0.41679	IL10	76353	25kb
ENSG00000142224	1	206947215	207041324	234	38	120000	0.58941	0.27779	IL19	76353	25kb
ENSG00000162891	1	207013699	207067568	90	19	120000	0.58752	0.27843	IL20	76353	25kb
ENSG00000162892	1	207045788	207102484	92	10	120000	1.3179	0.09377	IL24	76353	25kb
ENSG00000162894	1	207052731	207121592	127	13	120000	1.8684	0.03085	FAIM3	76353	25kb
ENSG00000162896	1	207076863	207144811	134	17	120000	2.0764	0.018926	PIGR	76353	25kb
ENSG00000162897	1	207106310	207168970	132	17	120000	1.8516	0.032043	FCAMR	76353	25kb
ENSG00000182795	1	207166866	207231101	93	15	120000	2.253	0.012129	C1orf116	76353	25kb
ENSG00000180667	1	207192194	207251325	64	11	120000	0.36756	0.3566	YOD1	76353	25kb
ENSG00000123836	1	207197801	207279369	115	22	120000	1.0992	0.13584	PFKFB2	76353	25kb
ENSG00000123843	1	207237187	207298338	137	23	120000	1.052	0.14641	C4BPB	76353	25kb
ENSG00000123838	1	207252607	207343317	191	32	120000	1.375	0.084563	C4BPA	76353	25kb
ENSG00000196352	1	207469853	207559311	187	17	120000	1.9922	0.023176	CD55	76353	25kb
ENSG00000117322	1	207602575	207688240	204	27	120000	-0.39585	0.65389	CR2	76353	25kb
ENSG00000203710	1	207644492	207838992	424	37	120000	-0.52675	0.70082	CR1	76353	25kb
ENSG00000197721	1	207793458	207936761	480	30	120000	-0.18698	0.57416	CR1L	76353	25kb
ENSG00000117335	1	207900402	207993858	185	17	120000	1.2026	0.11457	CD46	76353	25kb
ENSG00000174059	1	208032594	208109747	273	30	120000	2.3149	0.01031	CD34	76353	25kb
ENSG00000076356	1	208170587	208442665	820	81	120000	0.039095	0.48441	PLXNA2	76353	25kb
ENSG00000008118	1	209732062	209812283	296	35	120000	1.4421	0.074638	CAMK1G	76353	25kb
ENSG00000196878	1	209763215	209850811	317	49	120000	0.50346	0.30732	LAMB3	76353	25kb
ENSG00000123689	1	209823765	209874733	138	38	120000	0.46108	0.32237	G0S2	76353	25kb
ENSG00000117594	1	209834510	209933295	214	22	120000	0.38855	0.3488	HSD11B1	76353	25kb
ENSG00000009790	1	209904377	209980668	206	20	120000	0.2305	0.40885	TRAF3IP3	76353	25kb
ENSG00000162757	1	209930661	209982904	155	19	120000	-0.045881	0.5183	C1orf74	76353	25kb
ENSG00000117595	1	209934036	210004465	226	24	120000	-0.45626	0.6759	IRF6	76353	25kb
ENSG00000117597	1	209976352	210055910	281	19	120000	-1.295	0.90234	DIEXF	76353	25kb
ENSG00000143469	1	210086538	210362636	583	25	120000	1.1397	0.12721	SYT14	76353	25kb
ENSG00000082497	1	210381144	210444976	102	18	120000	1.2204	0.11117	SERTAD4	76353	25kb
ENSG00000054392	1	210476596	210874638	1550	118	120000	0.29147	0.38535	HHAT	76353	25kb
ENSG00000143473	1	210831555	211332457	1560	96	120000	-0.65347	0.74327	KCNH1	76353	25kb
ENSG00000117625	1	211406719	211514727	321	27	120000	-0.8883	0.81281	RCOR3	76353	25kb
ENSG00000082512	1	211474957	211573288	236	32	120000	-0.40772	0.65826	TRAF5	76353	25kb
ENSG00000198570	1	211624864	211691259	266	29	120000	0.48101	0.31526	RD3	76353	25kb
ENSG00000170385	1	211719910	211777084	173	34	120000	-1.1954	0.88404	SLC30A1	76353	25kb
ENSG00000117650	1	211811114	211873960	242	35	120000	-0.57836	0.71849	NEK2	76353	25kb
ENSG00000123684	1	211891799	212029114	435	35	120000	1.3934	0.081752	LPGAT1	76353	25kb
ENSG00000143493	1	212088741	212233884	498	24	120000	-0.8909	0.81351	INTS7	76353	25kb
ENSG00000143476	1	212183919	212305742	383	31	120000	-0.88546	0.81205	DTL	76353	25kb
ENSG00000066027	1	212433879	212560200	297	22	120000	-0.41516	0.66099	PPP2R5A	76353	25kb
ENSG00000065600	1	212512273	212613243	226	32	120000	0.41312	0.33976	TMEM206	76353	25kb
ENSG00000117691	1	212581229	212644714	207	31	120000	-0.30698	0.62057	NENF	76353	25kb
ENSG00000162772	1	212713676	212819119	352	49	120000	0.71021	0.23879	ATF3	76353	25kb
ENSG00000162771	1	212772789	212825120	137	31	120000	0.26496	0.39552	FAM71A	76353	25kb
ENSG00000123685	1	212834760	212898327	231	36	120000	0.34524	0.36496	BATF3	76353	25kb
ENSG00000117697	1	212874495	212990124	491	21	120000	0.43739	0.33091	NSL1	76353	25kb
ENSG00000203705	1	212940170	213014968	321	14	120000	0.79806	0.21242	TATDN3	76353	25kb
ENSG00000185523	1	212978483	213045991	314	16	120000	1.1595	0.12312	C1orf227	76353	25kb
ENSG00000162769	1	213006597	213097705	454	20	120000	1.0098	0.15629	FLVCR1	76353	25kb
ENSG00000143494	1	213098862	213190379	217	32	120000	-1.1418	0.87323	VASH2	76353	25kb
ENSG00000174606	1	213140524	213214168	133	20	120000	-1.7573	0.96056	ANGEL2	76353	25kb
ENSG00000136643	1	213199589	213473116	333	23	120000	-1.7718	0.96179	RPS6KC1	76353	25kb
ENSG00000117707	1	214131524	214239595	260	28	120000	-0.71148	0.76161	PROX1	76353	25kb
ENSG00000143499	1	214429445	214535474	306	29	120000	1.2638	0.10314	SMYD2	76353	25kb
ENSG00000152104	1	214497039	214750792	915	71	120000	3.5472	0.00019468	PTPN14	76353	25kb
ENSG00000117724	1	214751538	214862931	399	28	120000	-0.52555	0.7004	CENPF	76353	25kb
ENSG00000082482	1	215154118	215435436	874	37	120000	-0.032292	0.51288	KCNK2	76353	25kb
ENSG00000136636	1	215715735	215820149	66	18	120000	-1.1712	0.87924	KCTD3	76353	25kb
ENSG00000042781	1	215771236	216621738	2276	146	120000	-0.29588	0.61634	USH2A	76353	25kb
ENSG00000196482	1	216651588	217336097	2316	171	120000	-1.1721	0.87941	ESRRG	76353	25kb
ENSG00000092978	1	217575334	217829424	685	58	120000	0.30522	0.3801	GPATCH2	76353	25kb
ENSG00000162814	1	217779666	218070038	860	40	120000	0.67897	0.24858	SPATA17	76353	25kb
ENSG00000067533	1	218433629	218536325	171	16	120000	0.021728	0.49133	RRP15	76353	25kb
ENSG00000092969	1	218494577	218642961	295	39	120000	0.13902	0.44472	TGFB2	76353	25kb
ENSG00000228208	1	218658438	218724320	204	17	120000	0.40668	0.34212	C1orf143	76353	25kb
ENSG00000143353	1	219322186	219411207	145	14	120000	0.25864	0.39796	LYPLAL1	76353	25kb
ENSG00000196660	1	219833769	220156989	1009	45	120000	-1.2437	0.8932	SLC30A10	76353	25kb
ENSG00000136628	1	220116943	220245000	376	15	120000	-0.16671	0.5662	EPRS	76353	25kb

ENSG00000162813	1	220205824	220288804	173	27	120000	-0.34027	0.63317	BPNT1	76353	25kb
ENSG00000067704	1	220242444	220346380	205	23	120000	-1.2512	0.89456	IARS2	76353	25kb
ENSG00000118873	1	220296635	220470796	355	27	120000	-1.1461	0.87413	RAB3GAP2	76353	25kb
ENSG00000224867	1	220578286	220633023	180	23	120000	-0.55879	0.71185	AC096644.1	76353	25kb
ENSG00000116141	1	220676568	220862803	396	20	120000	-0.41379	0.66049	MARK1	76353	25kb
ENSG00000162817	1	220838187	220897499	256	19	120000	0.87727	0.19017	C1orf115	76353	25kb
ENSG00000117791	1	220896567	220983150	267	25	120000	-0.42416	0.66428	MARC2	76353	25kb
ENSG00000186205	1	220935101	221012735	235	36	120000	-0.73106	0.76763	MARC1	76353	25kb
ENSG00000136630	1	221026699	221083401	148	23	120000	0.19852	0.42132	HLX	76353	25kb
ENSG00000143507	1	221849766	221940518	259	38	120000	-0.2308	0.59126	DUSP10	76353	25kb
ENSG00000143512	1	222670602	222746445	174	20	120000	0.38983	0.34833	HHIPL2	76353	25kb
ENSG00000143498	1	222706244	222788275	240	29	120000	0.78923	0.21499	TAF1A	76353	25kb
ENSG00000154305	1	222766428	222866354	236	24	120000	2.5552	0.0053057	MIA3	76353	25kb
ENSG00000186063	1	222816355	222911552	169	23	120000	2.3635	0.0090513	AIDA	76353	25kb
ENSG00000162819	1	222860895	222933538	108	19	120000	2.0722	0.019123	BROX	76353	25kb
ENSG00000197520	1	222885549	222949147	118	14	120000	2.2508	0.012199	FAM177B	76353	25kb
ENSG00000154309	1	222963406	223204337	669	45	120000	0.067232	0.4732	DISP1	76353	25kb
ENSG00000187554	1	223257748	223341624	318	16	120000	-1.6771	0.95324	TLR5	76353	25kb
ENSG00000143502	1	223369161	223562544	433	33	120000	-0.31728	0.62449	SUSD4	76353	25kb
ENSG00000178395	1	223541715	223593812	99	11	120000	0.49916	0.30883	C1orf65	76353	25kb
ENSG00000203697	1	223686349	223878436	453	45	120000	0.1848	0.42669	CAPN8	76353	25kb
ENSG00000162909	1	223864295	223988720	342	21	120000	-0.82575	0.79553	CAPN2	76353	25kb
ENSG00000143514	1	223942601	224058674	258	16	120000	-0.46232	0.67808	TP53BP2	76353	25kb
ENSG00000269358	1	224190760	224241371	67	14	120000	-0.99956	0.84124	AC138393.1	76353	25kb
ENSG00000143756	1	224276789	224374749	305	18	120000	0.019491	0.49222	FBXO28	76353	25kb
ENSG00000143753	1	224338458	224406143	201	24	120000	0.50778	0.30581	DEGS1	76353	25kb
ENSG00000143748	1	224390036	224543089	343	27	120000	0.10811	0.45696	NVL	76353	25kb
ENSG00000143771	1	224519552	224592161	146	15	120000	-0.63067	0.73587	CNIH4	76353	25kb
ENSG00000162923	1	224547845	224649735	181	11	120000	-0.79391	0.78638	WDR26	76353	25kb
ENSG00000143786	1	224597362	224953251	903	48	120000	-2.0524	0.97993	CNIH3	76353	25kb
ENSG00000185842	1	225058964	225611996	1697	35	120000	0.39504	0.34641	DNAH14	76353	25kb
ENSG00000143815	1	225564204	225641627	214	29	120000	-0.22341	0.58839	LBR	76353	25kb
ENSG00000203691	1	225575404	225627040	148	22	120000	-0.17811	0.57068	AC092811.1	76353	25kb
ENSG00000154380	1	225649537	225865844	519	24	120000	-0.68813	0.75432	ENAH	76353	25kb
ENSG00000143742	1	225940531	226003168	231	31	120000	1.3745	0.08465	SRP9	76353	25kb
ENSG00000143819	1	225972794	226058260	305	40	120000	-0.024933	0.50995	EPHX1	76353	25kb
ENSG00000196187	1	226008237	226095069	302	35	120000	-0.99427	0.83995	TMEM63A	76353	25kb
ENSG00000243709	1	226048982	226124082	218	27	120000	-1.1691	0.87882	LEFTY1	76353	25kb
ENSG00000255835	1	226049396	226136978	237	30	120000	-1.0802	0.85998	RP4-559A3.7	76353	25kb
ENSG00000143811	1	226082578	226136978	123	26	120000	-0.25693	0.60139	PYCR2	76353	25kb
ENSG00000143768	1	226099298	226154189	100	24	120000	0.15782	0.4373	LEFTY2	76353	25kb
ENSG00000143751	1	226145403	226212032	117	20	120000	-0.54082	0.70569	SDE2	76353	25kb
ENSG00000163041	1	226224552	226284702	116	17	120000	-1.3554	0.91235	H3F3A	76353	25kb
ENSG00000182827	1	226307380	226399431	199	27	120000	-1.1052	0.86546	ACBD3	76353	25kb
ENSG00000185155	1	226386319	226439755	94	16	120000	-0.2848	0.6121	MIXL1	76353	25kb
ENSG00000183814	1	226393850	226522570	201	28	120000	0.14879	0.44086	LIN9	76353	25kb
ENSG00000143799	1	226523392	226620780	280	19	120000	-0.8442	0.80072	PARP1	76353	25kb
ENSG00000203685	1	226711501	226821915	312	42	120000	0.14611	0.44192	C1orf95	76353	25kb
ENSG00000143772	1	226794391	226952024	314	44	120000	-1.0949	0.86323	ITPKB	76353	25kb
ENSG00000143801	1	227032885	227108806	188	23	120000	1.2142	0.11233	PSEN2	76353	25kb
ENSG00000163050	1	227060237	227200246	368	40	120000	0.78461	0.21634	ADCK3	76353	25kb
ENSG00000143776	1	227152566	227531175	1725	26	120000	0.93055	0.17604	CDC42BPA	76353	25kb
ENSG00000181450	1	227726244	227890144	387	17	120000	0.19515	0.42264	ZNF678	76353	25kb
ENSG00000143740	1	227891240	227993927	227	34	120000	-0.0084663	0.50338	SNAP47	76353	25kb
ENSG00000081692	1	227893126	227948112	110	20	120000	0.31387	0.37681	JMJD4	76353	25kb
ENSG00000185888	1	227978394	228059171	256	31	120000	-0.38687	0.65057	PRSS38	76353	25kb
ENSG00000143816	1	228081357	228160599	211	24	120000	0.38047	0.3518	WNT9A	76353	25kb
ENSG00000154342	1	228169752	228273961	192	15	120000	0.22486	0.41104	WNT3A	76353	25kb
ENSG00000143761	1	228245361	228311912	82	15	120000	-0.42468	0.66447	ARF1	76353	25kb
ENSG00000143793	1	228263427	228318112	69	14	120000	-0.41609	0.66133	C1orf35	76353	25kb
ENSG00000162910	1	228269380	228322013	69	15	120000	-0.42866	0.66591	MRPL55	76353	25kb
ENSG00000143774	1	228302663	228361685	76	13	120000	-0.86566	0.80666	GUK1	76353	25kb
ENSG00000198835	1	228312553	228372527	88	14	120000	-0.84048	0.79968	GJC2	76353	25kb
ENSG00000203684	1	228326787	228378213	84	13	120000	-1.0602	0.85546	IBA57-AS1	76353	25kb
ENSG00000181873	1	228328516	228394958	109	15	120000	-0.55357	0.71006	IBA57	76353	25kb
ENSG00000162913	1	228366207	228426365	138	8	120000	-0.4469	0.67253	C1orf145	76353	25kb
ENSG00000154358	1	228370831	228591577	568	29	120000	-0.70759	0.7604	OBSCN	76353	25kb
ENSG00000154370	1	228556374	228619541	119	15	120000	-0.25517	0.60071	TRIM11	76353	25kb
ENSG00000162931	1	228570641	228629562	111	13	120000	-0.19447	0.5771	TRIM17	76353	25kb
ENSG00000168148	1	228587546	228638026	99	14	120000	-0.069547	0.52772	HIST3H3	76353	25kb
ENSG00000181218	1	228620065	228670560	95	17	120000	0.070369	0.47195	HIST3H2A	76353	25kb
ENSG00000196890	1	228620808	228671259	94	17	120000	0.10896	0.45662	HIST3H2BB	76353	25kb
ENSG00000168159	1	228649762	228708467	106	22	120000	-0.28871	0.6136	RNF187	76353	25kb
ENSG00000116574	1	228845824	228907416	182	19	120000	0.24546	0.40305	RHOU	76353	25kb
ENSG00000177800	1	229360383	229412557	135	27	120000	-0.58303	0.72006	TMEM78	76353	25kb
ENSG00000168118	1	229381822	229466641	193	18	120000	-1.2722	0.89835	RAB4A	76353	25kb
ENSG00000213029	1	229415129	229466248	126	10	120000	-1.4578	0.92755	SPHAR	76353	25kb
ENSG00000154429	1	229431758	229504041	147	19	120000	-1.1767	0.88035	CCSAP	76353	25kb
ENSG00000143632	1	229541992	229594845	120	20	120000	-1.8869	0.97041	ACTA1	76353	25kb
ENSG00000069248	1	229552045	229669103	240	14	120000	-1.3856	0.91707	NUP133	76353	25kb
ENSG00000135776	1	229627329	229719442	163	10	120000	-1.1811	0.88123	ABCB10	76353	25kb
ENSG00000135801	1	229703858	229786794	167	19	120000	-0.54246	0.70625	TAF5L	76353	25kb
ENSG00000135763	1	229736981	229820946	186	19	120000	-0.25642	0.60119	URB2	76353	25kb
ENSG00000143641	1	230168536	230442870	1044	94	120000	1.6799	0.046484	GALNT2	76353	25kb
ENSG00000177614	1	230432392	230586475	557	63	120000	-1.4558	0.92727	PGBD5	76353	25kb
ENSG00000135775	1	230753235	230854728	363	47	120000	2.1983	0.013963	COG2	76353	25kb
ENSG00000135744	1	230813269	230875043	293	23	120000	1.3511	0.088327	AGT	76353	25kb
ENSG00000135773	1	230858130	230962749	469	51	120000	0.26703	0.39472	CAPN9	76353	25kb
ENSG00000119280	1	230947865	231030335	264	34	120000	0.49567	0.31006	C1orf198	76353	25kb
ENSG00000143643	1	231016989	231139621	410	24	120000	0.85917	0.19512	TTC13	76353	25kb
ENSG00000173409	1	231089727	231161341	260	22	120000	0.51117	0.30462	ARV1	76353	25kb
ENSG00000182118	1	231129704	231200992	269	41	120000	-0.88496	0.81191	FAM89A	76353	25kb
ENSG00000119283	1	231272858	231382302	319	42	120000	-1.0893	0.86198	TRIM67	76353	25kb
ENSG00000143633	1	231334509	231401933	198	30	120000	-0.75101	0.77368	C1orf131	76353	25kb
ENSG00000116906	1	231351953	231438719	224	27	120000	-0.64838	0.74163	GNPAT	76353	25kb
ENSG00000116903	1	231443480	231498598	120	10	120000	-0.17457	0.56929	EXOC8	76353	25kb

ENSG00000010072	1	231447850	231515769	164	11	120000	-0.074849	0.52983	SPRTN	76353	25kb
ENSG00000135766	1	231474497	231585790	386	13	120000	0.076772	0.4694	EGLN1	76353	25kb
ENSG00000116918	1	231639399	231727270	123	22	120000	0.57334	0.28321	TSNAX	76353	25kb
ENSG00000270106	1	231639399	231979990	753	77	120000	-0.68003	0.75176	TSNAX-DISC1	76353	25kb
ENSG00000162946	1	231737561	232202018	1259	118	120000	-0.86896	0.80757	DISC1	76353	25kb
ENSG00000116991	1	232508711	232722304	650	64	120000	1.8889	0.029455	SIPA1L2	76353	25kb
ENSG00000212916	1	232915643	232971092	194	24	120000	-1.3637	0.91367	MAP10	76353	25kb
ENSG00000135778	1	233061351	233144628	196	17	120000	-0.46214	0.67801	NTPCR	76353	25kb
ENSG00000135749	1	233094181	233456459	1233	77	120000	-1.6515	0.95068	PCNXL2	76353	25kb
ENSG00000143674	1	233438514	233545894	362	31	120000	-2.541	0.99447	MLK4	76353	25kb
ENSG00000135750	1	233724750	233833258	385	61	120000	0.6666	0.25251	KCNK1	76353	25kb
ENSG00000183780	1	234015679	234485262	1678	141	120000	-1.1815	0.88129	SLC35F3	76353	25kb
ENSG00000168275	1	234484202	234544795	158	23	120000	-0.24584	0.5971	COA6	76353	25kb
ENSG00000059588	1	234502059	234639849	605	69	120000	-1.6989	0.95533	TARBP1	76353	25kb
ENSG00000168264	1	234715015	234770271	162	31	120000	0.29289	0.3848	IRF2BP2	76353	25kb
ENSG00000173726	1	235247651	235317251	156	17	120000	0.37945	0.35218	TOMM20	76353	25kb
ENSG00000188739	1	235269498	235349772	168	16	120000	0.37265	0.3547	RBM34	76353	25kb
ENSG00000054267	1	235269949	235516534	534	16	120000	0.27311	0.39238	ARID4B	76353	25kb
ENSG00000152904	1	235465665	235532847	162	13	120000	0.28662	0.3872	GGPS1	76353	25kb
ENSG00000116957	1	235505675	235637283	323	25	120000	-0.37657	0.64675	TBCE	76353	25kb
ENSG00000162885	1	235588238	235692781	277	19	120000	-0.019996	0.50798	B3GALNT2	76353	25kb
ENSG00000168243	1	235685987	235839054	465	53	120000	1.289	0.098696	GNG4	76353	25kb
ENSG00000143669	1	235799341	236071940	442	29	120000	3.2156	0.00065092	LYST	76353	25kb
ENSG00000116962	1	236114130	236253462	346	38	120000	-1.1991	0.88475	NID1	76353	25kb
ENSG00000077585	1	236280832	236410165	489	45	120000	0.13976	0.44442	GPR137B	76353	25kb
ENSG00000086619	1	236353855	236470319	484	20	120000	0.060965	0.47569	ERO1LB	76353	25kb
ENSG00000186197	1	236486562	236673214	775	62	120000	0.025215	0.48994	EDARADD	76353	25kb
ENSG00000116977	1	236656300	236741281	507	32	120000	-1.3794	0.91611	LGALS8	76353	25kb
ENSG00000119285	1	236687305	236792804	632	23	120000	-0.86759	0.80719	HEATR1	76353	25kb
ENSG00000077522	1	236824754	236952931	511	41	120000	-0.26431	0.60423	ACTN2	76353	25kb
ENSG00000116984	1	236933610	237092281	431	24	120000	0.66276	0.25374	MTR	76353	25kb
ENSG00000244020	1	237142403	237192718	135	24	120000	0.22628	0.41049	MT1HL1	76353	25kb
ENSG00000198626	1	237180505	238022288	2942	244	120000	-0.19518	0.57738	RYR2	76353	25kb
ENSG00000116996	1	238020705	238079094	213	21	120000	-0.56395	0.7136	ZP4	76353	25kb
ENSG00000270188	1	238082024	238133575	204	23	120000	0.38785	0.34906	MTRNR2L11	76353	25kb
ENSG00000133019	1	239524865	240103750	1506	126	120000	-0.048496	0.51934	CHRM3	76353	25kb
ENSG00000155816	1	240152648	240663489	1885	124	120000	0.34306	0.36577	FMN2	76353	25kb
ENSG00000268365	1	240604183	240668504	271	42	120000	-1.8504	0.96787	AL646016.1	76353	25kb
ENSG00000180875	1	240627873	240800449	513	91	120000	-1.1886	0.88271	GREM2	76353	25kb
ENSG00000182901	1	240906554	241545530	2094	171	120000	0.4211	0.33684	RGS7	76353	25kb
ENSG00000091483	1	241635903	241708061	202	24	120000	-0.84813	0.80182	FH	76353	25kb
ENSG00000117009	1	241670434	241783944	328	54	120000	-0.29362	0.61548	KMO	76353	25kb
ENSG00000054277	1	241728404	241865678	402	73	120000	0.86965	0.19225	OPN3	76353	25kb
ENSG00000203668	1	241767155	241824232	196	40	120000	1.1894	0.11714	CHML	76353	25kb
ENSG00000162843	1	241790580	241990435	696	79	120000	-0.28868	0.61359	WDR64	76353	25kb
ENSG00000174371	1	241986269	242083450	379	40	120000	-1.6401	0.94951	EXO1	76353	25kb
ENSG00000197769	1	242133792	242187375	247	35	120000	0.039868	0.4841	MAP1LC3C	76353	25kb
ENSG00000180287	1	242221288	242712998	1770	122	120000	-0.41082	0.6594	PLD5	76353	25kb
ENSG00000143702	1	243262730	243443650	213	20	120000	1.0568	0.14531	CEP170	76353	25kb
ENSG00000269795	1	243363058	243420911	100	11	120000	0.5757	0.28241	AC092782.1	76353	25kb
ENSG00000054282	1	243394320	243688394	508	37	120000	0.70331	0.24093	SDCCAG8	76353	25kb
ENSG00000117020	1	243626535	244039381	609	27	120000	0.39462	0.34656	AKT3	76353	25kb
ENSG00000179456	1	244189585	244245778	123	36	120000	-0.25335	0.6	ZBTB18	76353	25kb
ENSG00000268146	1	244202632	244256211	112	32	120000	-0.31482	0.62355	AL590483.1	76353	25kb
ENSG00000173728	1	244490937	244577965	340	44	120000	-0.92397	0.82225	C1orf100	76353	25kb
ENSG00000035687	1	244546796	244640436	233	31	120000	0.083908	0.46656	ADSS	76353	25kb
ENSG00000179397	1	244592679	244829479	705	43	120000	-0.37448	0.64598	C1orf101	76353	25kb
ENSG00000121644	1	244791237	244897335	286	36	120000	-1.0944	0.86311	DESI2	76353	25kb
ENSG00000203667	1	244973624	245033359	153	18	120000	0.097912	0.461	COX20	76353	25kb
ENSG00000153187	1	244989468	245052844	136	21	120000	-0.78448	0.78362	HNRNPU	76353	25kb
ENSG00000272195	1	245107652	245159390	165	22	120000	-0.68716	0.75401	RP11-156E8.1	76353	25kb
ENSG00000203666	1	245108007	245315466	627	43	120000	-1.1186	0.86835	EFCAB2	76353	25kb
ENSG00000162849	1	245293287	245897733	2226	220	120000	-2.0159	0.9781	KIF26B	76353	25kb
ENSG00000185420	1	245887642	246695614	3335	118	120000	-0.044832	0.51788	SMYD3	76353	25kb
ENSG00000162851	1	246678862	246754626	335	32	120000	-0.20981	0.58309	TFB2M	76353	25kb
ENSG00000162852	1	246704746	246856886	516	46	120000	-0.059119	0.52357	CNST	76353	25kb
ENSG00000143653	1	246862349	246956439	321	22	120000	0.91046	0.18129	SCCPDH	76353	25kb
ENSG00000153207	1	246977400	247120280	317	27	120000	-0.084855	0.53381	AHCTF1	76353	25kb
ENSG00000197472	1	247083849	247196395	376	31	120000	0.17494	0.43056	ZNF695	76353	25kb
ENSG00000135747	1	247083849	247267113	727	37	120000	-0.29162	0.61471	ZNF670	76353	25kb
ENSG00000188295	1	247238264	247292674	233	28	120000	-1.1093	0.86635	ZNF669	76353	25kb
ENSG00000221953	1	247248462	247300719	204	26	120000	-0.7732	0.7803	C1orf229	76353	25kb
ENSG00000196418	1	247260277	247360318	365	33	120000	-0.17619	0.56993	ZNF124	76353	25kb
ENSG00000162714	1	247435714	247520148	217	27	120000	0.64171	0.26053	ZNF496	76353	25kb
ENSG00000162711	1	247554458	247637410	459	61	120000	-2.108	0.98248	NLRP3	76353	25kb
ENSG00000177535	1	247589255	247640308	332	36	120000	-1.866	0.96898	OR2B11	76353	25kb
ENSG00000169224	1	247645360	247765992	368	51	120000	0.24274	0.4041	GCSAML	76353	25kb
ENSG00000196242	1	247668434	247722141	155	28	120000	-0.62299	0.73335	OR2C3	76353	25kb
ENSG00000177489	1	247726662	247777615	163	28	120000	0.99026	0.16102	OR2G2	76353	25kb
ENSG00000177476	1	247743856	247794838	158	25	120000	1.2223	0.1108	OR2G3	76353	25kb
ENSG00000197437	1	247810320	247861365	257	13	120000	0.60715	0.27188	OR13G1	76353	25kb
ENSG00000169214	1	247850045	247901105	126	16	120000	2.0038	0.022548	OR6F1	76353	25kb
ENSG00000241128	1	247861401	247912345	139	15	120000	2.1361	0.016336	OR14A2	76353	25kb
ENSG00000153230	1	247876917	247927861	133	13	120000	2.1334	0.016447	OR14K1	76353	25kb
ENSG00000221888	1	247895676	247946982	197	18	120000	2.1371	0.016295	OR1C1	76353	25kb
ENSG00000196772	1	247953102	248004031	264	11	120000	0.9045	0.18286	OR14A16	76353	25kb
ENSG00000197591	1	247979199	248030254	272	20	120000	0.55683	0.28882	OR11L1	76353	25kb
ENSG00000162722	1	247995501	248066507	337	37	120000	1.4068	0.079742	TRIM58	76353	25kb
ENSG00000238243	1	248006277	248085449	405	47	120000	1.8917	0.029269	OR2W3	76353	25kb
ENSG00000177462	1	248059320	248110258	241	28	120000	2.2357	0.012685	OR2T8	76353	25kb
ENSG00000177275	1	248072071	248123057	201	19	120000	1.1628	0.12245	OR2AJ1	76353	25kb
ENSG00000196071	1	248075493	248289224	624	40	120000	-0.31812	0.6248	OR2L13	76353	25kb
ENSG00000196936	1	248087160	248138098	224	17	120000	0.27143	0.39303	OR2L8	76353	25kb
ENSG00000187080	1	248103535	248154641	225	19	120000	-0.19208	0.57616	OR2AK2	76353	25kb
ENSG00000197454	1	248160250	248211188	104	12	120000	0.0055927	0.49777	OR2L5	76353	25kb
ENSG00000203663	1	248176474	248227607	101	12	120000	-0.54944	0.70865	OR2L2	76353	25kb

ENSG00000198128	1	248198984	248249922	113	11	120000	-0.55286	0.70982	OR2L3	76353	25kb
ENSG00000162727	1	248283450	248334388	106	10	120000	-1.4205	0.92227	OR2M5	76353	25kb
ENSG00000198601	1	248318288	248369331	196	14	120000	0.29265	0.3849	OR2M2	76353	25kb
ENSG00000228198	1	248341332	248392392	186	10	120000	0.6262	0.26559	OR2M3	76353	25kb
ENSG00000171180	1	248377231	248428166	79	9	120000	0.11237	0.45526	OR2M4	76353	25kb
ENSG00000177212	1	248411073	248462138	109	6	120000	-0.47094	0.68116	OR2T33	76353	25kb
ENSG00000177201	1	248432918	248483880	208	5	120000	-0.17358	0.5689	OR2T12	76353	25kb
ENSG00000177186	1	248461894	248512899	261	7	120000	-0.35506	0.63873	OR2M7	76353	25kb
ENSG00000177174	1	248487077	248538015	248	12	120000	-0.75171	0.77389	OR14C36	76353	25kb
ENSG00000196944	1	248499883	248550929	242	11	120000	-0.80821	0.79052	OR2T4	76353	25kb
ENSG00000198104	1	248525910	248576836	230	8	120000	-0.54634	0.70758	OR2T6	76353	25kb
ENSG00000175143	1	248544296	248595405	202	9	120000	-0.79677	0.78721	OR2T1	76353	25kb
ENSG00000227152	1	248579508	248630434	72	13	120000	-0.39707	0.65434	OR2T7	76353	25kb
ENSG00000196240	1	248591077	248642130	77	7	120000	0.60019	0.27419	OR2T2	76353	25kb
ENSG00000196539	1	248611627	248662634	59	3	120000	0.81547	0.2074	OR2T3	76353	25kb
ENSG00000203661	1	248626885	248677898	48	3	120000	0.85903	0.19516	OR2T5	76353	25kb
ENSG00000188558	1	248659916	248710964	27	4	120000	0.11647	0.45364	OR2G6	76353	25kb
ENSG00000182783	1	248696784	248747797	15	3	120000	0.70159	0.24147	OR2T29	76353	25kb
ENSG00000183310	1	248712022	248763080	22	5	120000	1.0579	0.14506	OR2T34	76353	25kb
ENSG00000184022	1	248731052	248782100	21	6	120000	1.1369	0.1278	OR2T10	76353	25kb
ENSG00000183130	1	248764420	248815491	29	8	120000	-0.86305	0.80594	OR2T11	76353	25kb
ENSG00000177151	1	248776588	248827559	59	12	120000	-1.1786	0.88072	OR2T35	76353	25kb
ENSG00000187701	1	248788232	248839185	95	13	120000	-1.1352	0.87186	OR2T27	76353	25kb
ENSG00000189181	1	248819628	248870629	179	8	120000	-0.91794	0.82067	OR14I1	76353	25kb
ENSG00000266949	1	248877716	248928150	45	5	120000	-0.70112	0.75839	LYPD8	76353	25kb
ENSG00000175137	1	249079648	249145832	97	11	120000	-0.53109	0.70232	SH3BP5L	76353	25kb
ENSG00000171161	1	249107409	249168716	95	10	120000	-1.3398	0.90984	ZNF672	76353	25kb
ENSG00000171163	1	249119205	249178343	94	12	120000	-1.4725	0.92956	ZNF692	76353	25kb
ENSG00000185220	1	249175395	249239145	62	8	120000	0.57501	0.28264	PGBD2	76353	25kb
ENSG00000184731	2	13814	71870	137	15	120000	-0.049657	0.5198	FAM110C	76353	25kb
ENSG00000035115	2	192730	291398	198	18	120000	-0.86723	0.80709	SH3YL1	76353	25kb
ENSG00000143727	2	239140	303283	129	14	120000	-0.78128	0.78268	ACP1	76353	25kb
ENSG00000189292	2	254558	313851	120	16	120000	-0.75637	0.77529	FAM150B	76353	25kb
ENSG00000151353	2	642335	702439	216	22	120000	-1.4657	0.92864	TMEM18	76353	25kb
ENSG00000172554	2	921554	1396385	2005	77	120000	0.069593	0.47226	SNTG2	76353	25kb
ENSG00000115705	2	1352995	1572483	844	50	120000	-0.50286	0.69247	TPO	76353	25kb
ENSG00000130508	2	1610659	1773624	600	63	120000	-1.0226	0.84676	PXDN	76353	25kb
ENSG00000186487	2	1767885	2360032	1794	104	120000	1.1915	0.11673	MYT1L	76353	25kb
ENSG00000032389	2	3167696	3406653	842	39	120000	-0.073954	0.52948	TSSC1	76353	25kb
ENSG00000171853	2	3358446	3513865	547	39	120000	-0.05928	0.52364	TRAPPC12	76353	25kb
ENSG00000182551	2	3476132	3548507	219	23	120000	0.21197	0.41607	ADI1	76353	25kb
ENSG00000255767	2	3554574	3620547	165	23	120000	-0.33158	0.6299	RP13-512J5.1	76353	25kb
ENSG00000171865	2	3567383	3631206	211	28	120000	-0.21602	0.58551	RNASEH1	76353	25kb
ENSG00000171863	2	3597795	3653509	224	35	120000	-0.49532	0.68981	RPS7	76353	25kb
ENSG00000118004	2	3617426	3717048	395	46	120000	0.80169	0.21137	COLEC11	76353	25kb
ENSG00000151360	2	3680785	3775261	326	35	120000	1.0017	0.15824	ALLC	76353	25kb
ENSG00000214866	2	3726453	3861122	492	59	120000	-0.58537	0.72085	DCDC2C	76353	25kb
ENSG00000176887	2	5807799	5866516	173	30	120000	-0.07485	0.52983	SOX11	76353	25kb
ENSG00000272268	2	6086712	6139374	146	25	120000	0.45439	0.32477	DKFZP761K2322	76353	25kb
ENSG00000134326	2	6955701	7031766	124	39	120000	-0.68922	0.75466	CMPK2	76353	25kb
ENSG00000134321	2	6980937	7063370	168	41	120000	-0.15962	0.56341	RSAD2	76353	25kb
ENSG00000151692	2	7032523	7233417	714	62	120000	0.237	0.40633	RNF144A	76353	25kb
ENSG00000115738	2	8793975	8849583	172	24	120000	-1.1477	0.87446	ID2	76353	25kb
ENSG00000134313	2	8840408	9002760	311	32	120000	-0.29832	0.61727	KIDINS220	76353	25kb
ENSG00000143797	2	8967820	9168942	334	25	120000	-0.83239	0.79741	MBOAT2	76353	25kb
ENSG00000151693	2	9321894	9570812	556	37	120000	-2.2446	0.9876	ASAP2	76353	25kb
ENSG00000119185	2	9518604	9588676	140	16	120000	-1.4748	0.92986	ITGB1BP1	76353	25kb
ENSG00000119203	2	9538697	9638230	203	16	120000	-1.0303	0.84857	CPSF3	76353	25kb
ENSG00000134330	2	9588787	9661672	144	11	120000	-0.81099	0.79132	IAH1	76353	25kb
ENSG00000151694	2	9603615	9720921	237	21	120000	-1.2955	0.90243	ADAM17	76353	25kb
ENSG00000134308	2	9699101	9796143	222	34	120000	-1.6497	0.9505	YWHAQ	76353	25kb
ENSG00000115750	2	9958483	10099545	654	35	120000	0.50965	0.30515	TAF1B	76353	25kb
ENSG00000134317	2	10060341	10167411	482	46	120000	1.1835	0.11831	GRHL1	76353	25kb
ENSG00000172059	2	10157976	10219963	218	38	120000	0.064809	0.47416	KLF11	76353	25kb
ENSG00000205795	2	10171907	10246071	250	38	120000	0.18716	0.42577	CYS1	76353	25kb
ENSG00000233502	2	10198908	10251228	170	29	120000	-0.0051681	0.50206	AC104794.4	76353	25kb
ENSG00000171848	2	10237455	10296545	135	17	120000	-0.84296	0.80037	RRM2	76353	25kb
ENSG00000163009	2	10256509	10376851	409	44	120000	-0.3696	0.64416	C2orf48	76353	25kb
ENSG00000115756	2	10418015	10592743	696	91	120000	-0.37785	0.64723	HPCAL1	76353	25kb
ENSG00000115758	2	10555094	10613630	173	25	120000	-0.12275	0.54885	ODC1	76353	25kb
ENSG00000115761	2	10685892	10855101	659	27	120000	-0.046967	0.51873	NOL10	76353	25kb
ENSG00000143882	2	10836775	10950236	424	38	120000	2.4833	0.0065089	ATP6V1C2	76353	25kb
ENSG00000143870	2	10898517	11003103	590	31	120000	2.0946	0.018101	PDIA6	76353	25kb
ENSG00000232056	2	10962703	11020081	315	44	120000	1.6123	0.053451	AC092687.4	76353	25kb
ENSG00000162975	2	11027063	11079350	181	30	120000	-1.1406	0.87297	KCNF1	76353	25kb
ENSG00000150873	2	11248179	11311916	274	31	120000	-0.16997	0.56748	C2orf50	76353	25kb
ENSG00000162976	2	11270324	11344000	251	31	120000	0.87916	0.18966	PQLC3	76353	25kb
ENSG00000134318	2	11294887	11513456	654	26	120000	1.7053	0.044067	ROCK2	76353	25kb
ENSG00000169016	2	11559501	11631297	296	16	120000	0.95552	0.16966	E2F6	76353	25kb
ENSG00000269074	2	11578927	11631274	185	17	120000	1.0106	0.15609	AC099344.1	76353	25kb
ENSG00000196208	2	11649242	11807914	466	68	120000	-0.59491	0.72405	GREB1	76353	25kb
ENSG00000169006	2	11773304	11835290	253	48	120000	-1.6769	0.95322	NTSR2	76353	25kb
ENSG00000134324	2	11792721	11992535	652	70	120000	-1.1317	0.87113	LPIN1	76353	25kb
ENSG00000071575	2	12832015	12907860	182	25	120000	1.1312	0.12898	TRIB2	76353	25kb
ENSG00000162981	2	14747810	14815933	101	19	120000	-0.22036	0.5872	FAM84A	76353	25kb
ENSG00000265629	2	14750215	14815933	99	19	120000	-0.19905	0.57889	AC011897.1	76353	25kb
ENSG00000151779	2	15282032	15726454	1059	27	120000	0.89669	0.18494	NBAS	76353	25kb
ENSG00000079785	2	15706302	15796235	282	19	120000	-1.4821	0.93084	DDX1	76353	25kb
ENSG00000231031	2	15805906	15909463	382	50	120000	-1.8317	0.9665	AC008271.1	76353	25kb
ENSG00000134323	2	16055686	16112129	142	25	120000	-0.58484	0.72067	MYCN	76353	25kb
ENSG00000197872	2	16705727	16872599	489	65	120000	0.17311	0.43128	FAM49A	76353	25kb
ENSG00000214842	2	17666851	17724706	187	12	120000	-0.14521	0.55773	RAD51AP2	76353	25kb
ENSG00000163032	2	17695393	17863285	526	19	120000	-0.61238	0.72986	VSNL1	76353	25kb
ENSG00000163029	2	17820079	18006509	411	30	120000	-0.31268	0.62274	SMC6	76353	25kb
ENSG00000178295	2	17910125	17991632	190	23	120000	-0.71663	0.7632	GEN1	76353	25kb
ENSG00000151379	2	17972763	18023368	180	27	120000	0.67981	0.24831	MSGN1	76353	25kb

ENSG00000170745	2	18034114	18567882	2139	153	120000	1.2654	0.10286	KCNS3	76353	25kb
ENSG00000240857	2	18710989	18766946	162	25	120000	-1.0997	0.86428	RDH14	76353	25kb
ENSG00000250741	2	18711811	18795830	195	31	120000	-1.0338	0.84939	NT5C1B-RDH14	76353	25kb
ENSG00000185013	2	18712050	18795838	192	31	120000	-1.0486	0.85281	NT5C1B	76353	25kb
ENSG00000143867	2	19526246	19583414	119	20	120000	-0.33963	0.63293	OSR1	76353	25kb
ENSG00000183891	2	20071404	20126747	188	31	120000	-0.97	0.83398	TTC32	76353	25kb
ENSG00000118965	2	20085021	20214892	313	24	120000	-0.8125	0.79175	WDR35	76353	25kb
ENSG00000132031	2	20166872	20237455	177	25	120000	-1.4152	0.92149	MATN3	76353	25kb
ENSG00000068697	2	20207411	20276789	201	27	120000	-0.79374	0.78633	LAPTM4A	76353	25kb
ENSG00000115884	2	20375558	20450194	248	36	120000	1.6683	0.04763	SDC1	76353	25kb
ENSG00000055917	2	20423452	20576995	339	19	120000	2.0777	0.018869	PUM2	76353	25kb
ENSG00000143878	2	20621835	20674200	156	27	120000	-1.9405	0.97384	RHOB	76353	25kb
ENSG00000118960	2	20735208	20875849	619	63	120000	-1.6583	0.95137	HS1BP3	76353	25kb
ENSG00000143869	2	20841424	20898418	175	43	120000	-0.25811	0.60184	GDF7	76353	25kb
ENSG00000118961	2	20858788	21047882	413	44	120000	-1.0586	0.85511	C2orf43	76353	25kb
ENSG00000084674	2	21199301	21291945	169	30	120000	1.338	0.090446	APOB	76353	25kb
ENSG00000218819	2	21321789	21391144	183	14	120000	1.2705	0.10195	TDRD15	76353	25kb
ENSG00000119771	2	23583088	23956481	1291	117	120000	-1.4507	0.92657	KLHL29	76353	25kb
ENSG00000224361	2	23704913	23772214	268	44	120000	0.06065	0.47582	AC011239.1	76353	25kb
ENSG00000119778	2	23946534	24174984	592	20	120000	-1.3068	0.90435	ATAD2B	76353	25kb
ENSG00000173960	2	24125155	24252779	364	23	120000	-1.6489	0.95042	UBXN2A	76353	25kb
ENSG00000205639	2	24207951	24311191	263	25	120000	-1.8055	0.9645	MFSD2B	76353	25kb
ENSG00000163026	2	24227210	24297445	179	25	120000	-1.4826	0.93092	C2orf44	76353	25kb
ENSG00000119782	2	24247571	24311551	165	18	120000	-1.3413	0.91008	FKBP1B	76353	25kb
ENSG00000115128	2	24265454	24324313	171	13	120000	-1.2512	0.89456	SF3B14	76353	25kb
ENSG00000219626	2	24274396	24417509	448	26	120000	-1.7325	0.95841	FAM228B	76353	25kb
ENSG00000115129	2	24275303	24333731	177	12	120000	-1.1734	0.87968	TP53I3	76353	25kb
ENSG00000176732	2	24313241	24371347	189	14	120000	-1.2378	0.8921	PFN4	76353	25kb
ENSG00000266118	2	24322236	24438305	361	26	120000	-1.6589	0.95143	RP11-507M3.1	76353	25kb
ENSG00000186453	2	24372938	24448718	225	22	120000	-1.2688	0.89774	FAM228A	76353	25kb
ENSG00000198399	2	24400733	24608583	519	19	120000	-0.44444	0.67164	ITSN2	76353	25kb
ENSG00000084676	2	24689783	25018571	758	29	120000	-0.40242	0.65631	NCOA1	76353	25kb
ENSG00000184924	2	24987603	25041251	137	21	120000	-0.27079	0.60673	PTRHD1	76353	25kb
ENSG00000138092	2	24991005	25070245	234	27	120000	-0.34356	0.63441	CENPO	76353	25kb
ENSG00000138031	2	25017038	25167708	491	31	120000	-0.70197	0.75865	ADCY3	76353	25kb
ENSG00000115137	2	25141505	25219963	161	18	120000	-1.0614	0.85575	DNAJC27	76353	25kb
ENSG00000084710	2	25239999	25403243	349	29	120000	-0.7629	0.77724	EFR3B	76353	25kb
ENSG00000115138	2	25358722	25416772	120	14	120000	-0.58047	0.7192	POMC	76353	25kb
ENSG00000119772	2	25430845	25590459	307	46	120000	-1.2036	0.88563	DNMT3A	76353	25kb
ENSG00000138101	2	25575067	25921503	570	66	120000	0.63204	0.26368	DTNB	76353	25kb
ENSG00000143970	2	25931622	26126385	445	23	120000	-0.13357	0.55313	ASXL2	76353	25kb
ENSG00000084731	2	26124471	26230618	280	35	120000	0.13239	0.44734	KIF3C	76353	25kb
ENSG00000268412	2	26226074	26276481	111	15	120000	-0.76258	0.77714	AC013449.1	76353	25kb
ENSG00000084733	2	26231976	26385323	269	13	120000	-0.58369	0.72028	RAB10	76353	25kb
ENSG00000157833	2	26370960	26437532	122	11	120000	-0.44837	0.67306	GAREML	76353	25kb
ENSG00000084754	2	26388504	26492594	193	9	120000	-0.59926	0.7255	HADHA	76353	25kb
ENSG00000138029	2	26441038	26538336	200	15	120000	-0.073894	0.52945	HADHB	76353	25kb
ENSG00000173567	2	26506041	26594685	215	32	120000	1.2677	0.10245	GPR113	76353	25kb
ENSG00000138018	2	26506415	26643759	319	29	120000	1.5787	0.057206	EPT1	76353	25kb
ENSG00000157856	2	26599784	26704579	233	37	120000	1.9317	0.026698	DRC1	76353	25kb
ENSG00000115155	2	26655071	26806566	445	58	120000	-0.31055	0.62193	OTOF	76353	25kb
ENSG00000173557	2	26760450	26827400	185	33	120000	-0.38283	0.64908	C2orf70	76353	25kb
ENSG00000157884	2	26779070	26889236	267	34	120000	-0.6694	0.74838	CIB4	76353	25kb
ENSG00000171303	2	26890619	26981288	189	31	120000	-1.2602	0.8962	KCNK3	76353	25kb
ENSG00000213699	2	26962152	27029099	117	12	120000	-0.56632	0.71441	SLC35F6	76353	25kb
ENSG00000115163	2	26962157	27048935	204	17	120000	-0.36332	0.64182	CENPA	76353	25kb
ENSG00000157851	2	27045615	27198219	311	15	120000	-0.38514	0.64993	DPYSL5	76353	25kb
ENSG00000084764	2	27168480	27275064	147	11	120000	0.053427	0.4787	MAPRE3	76353	25kb
ENSG00000119777	2	27230778	27289563	63	9	120000	-0.0961	0.53828	TMEM214	76353	25kb
ENSG00000084693	2	27240232	27318490	85	9	120000	0.033884	0.48648	AGBL5	76353	25kb
ENSG00000228474	2	27268340	27319641	66	9	120000	-0.16472	0.56542	OST4	76353	25kb
ENSG00000138080	2	27276435	27334271	69	9	120000	-0.22281	0.58816	EMILIN1	76353	25kb
ENSG00000138030	2	27284615	27348640	85	10	120000	-0.16079	0.56387	KHK	76353	25kb
ENSG00000138028	2	27296757	27366995	98	13	120000	0.50124	0.3081	CGREF1	76353	25kb
ENSG00000143994	2	27321682	27378680	89	14	120000	1.0823	0.13956	ABHD1	76353	25kb
ENSG00000138073	2	27328624	27382543	82	14	120000	1.2248	0.11032	PREB	76353	25kb
ENSG00000186143	2	27334723	27387278	92	15	120000	1.0791	0.14027	C2orf53	76353	25kb
ENSG00000163792	2	27346872	27401378	98	19	120000	0.96906	0.16626	TCF23	76353	25kb
ENSG00000138074	2	27397455	27460826	153	15	120000	-1.3196	0.90652	SLC5A6	76353	25kb
ENSG00000138085	2	27409895	27465046	141	14	120000	-1.3184	0.90632	ATRAID	76353	25kb
ENSG00000084774	2	27415258	27491811	180	17	120000	-1.2078	0.88644	CAD	76353	25kb
ENSG00000115194	2	27451552	27523685	136	18	120000	-0.7412	0.77071	SLC30A3	76353	25kb
ENSG00000163793	2	27473289	27529367	91	13	120000	-0.23604	0.5933	DNAJC5G	76353	25kb
ENSG00000138100	2	27480260	27555307	95	15	120000	0.024516	0.49022	TRIM54	76353	25kb
ENSG00000163794	2	27505268	27556313	59	12	120000	0.41744	0.33818	UCN	76353	25kb
ENSG00000115204	2	27507360	27573547	68	14	120000	0.065215	0.474	MPV17	76353	25kb
ENSG00000115207	2	27523716	27604868	85	17	120000	-0.261	0.60295	GTF3C2	76353	25kb
ENSG00000115211	2	27562219	27618353	69	11	120000	-0.64767	0.7414	EIF2B4	76353	25kb
ENSG00000115234	2	27568389	27624995	70	12	120000	-0.61723	0.73146	SNX17	76353	25kb
ENSG00000163795	2	27575098	27628657	71	11	120000	-0.49348	0.68916	ZNF513	76353	25kb
ENSG00000115241	2	27579061	27657554	112	11	120000	-0.85683	0.80423	PPM1G	76353	25kb
ENSG00000115216	2	27625657	27690126	91	8	120000	-1.1638	0.87774	NRBP1	76353	25kb
ENSG00000157992	2	27640233	27694348	80	7	120000	-1.157	0.87637	KRTCAP3	76353	25kb
ENSG00000138002	2	27642238	27737656	131	10	120000	-0.96142	0.83183	IFT172	76353	25kb
ENSG00000115226	2	27689750	27743112	76	9	120000	-0.67978	0.75168	FNDC4	76353	25kb
ENSG00000084734	2	27694709	27771554	118	15	120000	-0.068093	0.52714	GCKR	76353	25kb
ENSG00000233438	2	27735253	27815011	133	16	120000	0.81605	0.20724	AC109829.1	76353	25kb
ENSG00000221843	2	27774389	27830588	92	11	120000	1.2229	0.11069	C2orf16	76353	25kb
ENSG00000243943	2	27780897	27883041	169	15	120000	0.76202	0.22302	ZNF512	76353	25kb
ENSG00000176714	2	27823506	27876879	99	11	120000	0.44284	0.32894	CCDC121	76353	25kb
ENSG00000198522	2	27826114	27899375	111	13	120000	0.32278	0.37343	GPN1	76353	25kb
ENSG00000119760	2	27848679	27911676	76	13	120000	-0.5471	0.70785	SUPT7L	76353	25kb
ENSG00000163798	2	27861338	27942840	70	14	120000	-0.14787	0.55878	SLC4A1AP	76353	25kb
ENSG00000205334	2	27903653	27963599	50	12	120000	0.34051	0.36673	AC074091.13	76353	25kb
ENSG00000243147	2	27969584	28235954	398	19	120000	0.61377	0.26968	MRPL33	76353	25kb
ENSG00000268656	2	27973851	28034186	85	11	120000	0.13455	0.44648	AC110084.1	76353	25kb

ENSG00000171174	2	27979231	28138965	241	18	120000	0.49866	0.30901	RBKS	76353	25kb
ENSG00000158019	2	28087808	28586768	1091	53	120000	0.23148	0.40847	BRE	76353	25kb
ENSG00000075426	2	28590315	28665179	198	37	120000	-1.4562	0.92733	FOSL2	76353	25kb
ENSG00000163803	2	28655012	28891654	880	88	120000	0.24447	0.40343	PLB1	76353	25kb
ENSG00000213639	2	28949506	29050806	186	17	120000	-0.84927	0.80214	PPP1CB	76353	25kb
ENSG00000163806	2	28980383	29098477	211	18	120000	-0.91501	0.81991	SPDYA	76353	25kb
ENSG00000171103	2	29047687	29118167	112	14	120000	-1.0625	0.85599	TRMT61B	76353	25kb
ENSG00000163811	2	29092509	29196088	292	15	120000	-1.4883	0.93166	WDR43	76353	25kb
ENSG00000189350	2	29154477	29309239	551	43	120000	-1.1642	0.87783	FAM179A	76353	25kb
ENSG00000179270	2	29258842	29322127	198	30	120000	-0.56439	0.71376	C2orf71	76353	25kb
ENSG00000115295	2	29295571	29437509	335	32	120000	0.2502	0.40122	CLIP4	76353	25kb
ENSG00000171094	2	29390640	30169432	2778	162	120000	0.046515	0.48145	ALK	76353	25kb
ENSG00000119801	2	30344807	30408399	118	21	120000	-0.46794	0.68009	YPEL5	76353	25kb
ENSG00000213626	2	30429397	30571596	450	58	120000	2.0253	0.021418	LBH	76353	25kb
ENSG00000172954	2	30645092	30892091	842	25	120000	-0.50222	0.69224	LCLAT1	76353	25kb
ENSG00000162949	2	30920637	31068408	501	66	120000	-0.8978	0.81535	CAPN13	76353	25kb
ENSG00000158089	2	31108333	31403068	1073	85	120000	0.53402	0.29666	GALNT14	76353	25kb
ENSG00000214711	2	31370924	31481724	539	45	120000	-0.23028	0.59106	CAPN14	76353	25kb
ENSG00000013016	2	31431880	31517313	343	46	120000	-0.18386	0.57294	EHD3	76353	25kb
ENSG00000158125	2	31532187	31662581	440	62	120000	1.916	0.027682	XDH	76353	25kb
ENSG00000162959	2	32065129	32261299	417	21	120000	1.0475	0.14744	MEMO1	76353	25kb
ENSG00000162961	2	32067878	32289881	483	25	120000	1.1088	0.13376	DPY30	76353	25kb
ENSG00000021574	2	32263680	32407706	346	21	120000	2.1219	0.016923	SPAST	76353	25kb
ENSG00000152683	2	32365933	32474448	261	19	120000	2.3234	0.010079	SLC30A6	76353	25kb
ENSG00000091106	2	32424522	32515923	216	27	120000	1.8276	0.033806	NLRC4	76353	25kb
ENSG00000119820	2	32477979	32566663	184	33	120000	-0.044698	0.51783	YIPF4	76353	25kb
ENSG00000115760	2	32557096	32868966	630	25	120000	-0.37933	0.64778	BIRC6	76353	25kb
ENSG00000018699	2	32828099	33071118	833	44	120000	1.1898	0.11707	TTC27	76353	25kb
ENSG00000049323	2	33147039	33649576	2126	103	120000	0.91108	0.18113	LTBP1	76353	25kb
ENSG00000152689	2	33636391	33814817	614	90	120000	-1.7458	0.95958	RASGRP3	76353	25kb
ENSG00000119812	2	33783725	33849449	186	28	120000	-1.0045	0.84244	FAM98A	76353	25kb
ENSG00000150938	2	36558069	36803278	690	64	120000	-0.5514	0.70932	CRIM1	76353	25kb
ENSG00000217075	2	36733948	36804411	186	19	120000	-0.28446	0.61197	AC007401.2	76353	25kb
ENSG00000171055	2	36753570	36898230	530	42	120000	0.044811	0.48213	FEZ2	76353	25kb
ENSG00000205221	2	36898833	37066935	693	90	120000	0.075927	0.46974	VIT	76353	25kb
ENSG00000268688	2	37043327	37093530	201	26	120000	-0.4472	0.67263	AC007382.1	76353	25kb
ENSG00000115808	2	37045783	37218615	529	28	120000	-0.94702	0.82819	STRN	76353	25kb
ENSG00000008869	2	37170526	37336485	439	32	120000	-0.68701	0.75396	HEATR5B	76353	25kb
ENSG00000152133	2	37286594	37351387	162	17	120000	-0.52538	0.70034	GPATCH11	76353	25kb
ENSG00000055332	2	37301353	37409208	457	27	120000	-0.78343	0.78331	EIF2AK2	76353	25kb
ENSG00000138068	2	37369963	37448741	421	19	120000	-0.76816	0.7788	SULT6B1	76353	25kb
ENSG00000218739	2	37398631	37468336	317	23	120000	-1.1024	0.86486	CEBPZ-AS1	76353	25kb
ENSG00000115816	2	37403755	37483856	311	24	120000	-0.80561	0.78977	CEBPZ	76353	25kb
ENSG00000003509	2	37433774	37505546	206	27	120000	-0.22739	0.58994	NDUFAF7	76353	25kb
ENSG00000115825	2	37452645	37576951	468	27	120000	-0.23438	0.59265	PRKD3	76353	25kb
ENSG00000115828	2	37546717	37625465	301	42	120000	0.3136	0.37691	QPCT	76353	25kb
ENSG00000163171	2	37844032	37990611	471	54	120000	1.4027	0.080349	CDC42EP3	76353	25kb
ENSG00000115841	2	38125330	38319285	564	30	120000	0.4565	0.32402	RMDN2	76353	25kb
ENSG00000138061	2	38269116	38362044	320	34	120000	-0.47347	0.68206	CYP1B1	76353	25kb
ENSG00000119787	2	38497022	38629427	351	24	120000	-0.3759	0.6465	ATL2	76353	25kb
ENSG00000143889	2	38764120	38855728	217	21	120000	0.14948	0.44059	HNRNPLL	76353	25kb
ENSG00000143891	2	38868052	38993379	338	37	120000	0.26828	0.39424	GALM	76353	25kb
ENSG00000115875	2	38945741	39003636	108	14	120000	0.44007	0.32994	SRSF7	76353	25kb
ENSG00000152147	2	38953676	39037142	163	16	120000	0.53996	0.29461	GEMIN6	76353	25kb
ENSG00000163214	2	38999871	39128075	291	21	120000	0.27121	0.39312	DHX57	76353	25kb
ENSG00000188010	2	39078103	39181213	216	27	120000	0.00056299	0.49978	MORN2	76353	25kb
ENSG00000214694	2	39092021	39227590	273	25	120000	-0.52386	0.69981	ARHGEF33	76353	25kb
ENSG00000269210	2	39161764	39212483	91	12	120000	-0.79764	0.78746	AC019171.1	76353	25kb
ENSG00000115904	2	39183537	39376486	381	16	120000	-0.28684	0.61288	SOS1	76353	25kb
ENSG00000205111	2	39377787	39481729	236	21	120000	0.42162	0.33665	CDKL4	76353	25kb
ENSG00000011566	2	39451407	39689453	536	23	120000	-0.24492	0.59674	MAP4K3	76353	25kb
ENSG00000152154	2	39867122	39970103	170	12	120000	1.1301	0.12921	TMEM178A	76353	25kb
ENSG00000138050	2	39938200	40031407	237	18	120000	0.36219	0.35861	THUMPD2	76353	25kb
ENSG00000183023	2	40299410	40863193	2018	120	120000	0.5858	0.27901	SLC8A1	76353	25kb
ENSG00000268898	2	40463147	40515117	185	30	120000	0.51869	0.30199	AC007377.1	76353	25kb
ENSG00000205086	2	42137508	42206406	264	22	120000	-0.21049	0.58336	C2orf91	76353	25kb
ENSG00000162878	2	42250160	42310668	253	19	120000	-0.97929	0.83628	PKDCC	76353	25kb
ENSG00000143924	2	42371490	42584688	580	20	120000	-1.2256	0.88983	EML4	76353	25kb
ENSG00000115944	2	42535686	42677228	295	18	120000	-1.5657	0.9413	COX7A2L	76353	25kb
ENSG00000171126	2	42644157	42746237	267	31	120000	-1.435	0.92436	KCNG3	76353	25kb
ENSG00000057935	2	42696709	43009087	751	33	120000	-0.16732	0.56644	MTA3	76353	25kb
ENSG00000162881	2	42964642	43016401	176	22	120000	-0.97506	0.83524	OXER1	76353	25kb
ENSG00000162882	2	42969229	43044733	260	19	120000	-1.2923	0.90187	HAAO	76353	25kb
ENSG00000115970	2	43368800	43848185	1242	46	120000	-0.29122	0.61456	THADA	76353	25kb
ENSG00000152518	2	43424541	43478748	120	22	120000	-0.79489	0.78666	ZFP36L2	76353	25kb
ENSG00000152527	2	43839412	44020126	701	49	120000	-0.48807	0.68725	PLEKHH2	76353	25kb
ENSG00000138036	2	43976178	44062149	280	22	120000	-0.44867	0.67317	DYNC2LI1	76353	25kb
ENSG00000138075	2	44014611	44091004	345	29	120000	1.2456	0.10645	ABCG5	76353	25kb
ENSG00000143921	2	44041103	44130605	433	27	120000	1.9102	0.028053	ABCG8	76353	25kb
ENSG00000138095	2	44088647	44248144	618	31	120000	1.0386	0.14949	LRPPRC	76353	25kb
ENSG00000138032	2	44370108	44496523	298	22	120000	-0.55059	0.70904	PPM1B	76353	25kb
ENSG00000138079	2	44477599	44573633	275	19	120000	0.17501	0.43053	SLC3A1	76353	25kb
ENSG00000138078	2	44518420	44614001	275	19	120000	0.41748	0.33816	PREPL	76353	25kb
ENSG00000143919	2	44564089	45024731	1141	37	120000	0.59263	0.27671	CAMKMT	76353	25kb
ENSG00000138083	2	45143902	45198216	217	35	120000	-0.51405	0.69639	SIX3	76353	25kb
ENSG00000170577	2	45207300	45261569	268	20	120000	1.2612	0.10361	SIX2	76353	25kb
ENSG00000068784	2	45590819	45864304	777	31	120000	-0.76234	0.77707	SRBD1	76353	25kb
ENSG00000171132	2	45853484	46440129	2169	199	120000	0.58811	0.27823	PRKCE	76353	25kb
ENSG00000116016	2	46495806	46638836	453	56	120000	-0.31005	0.62174	EPAS1	76353	25kb
ENSG00000187600	2	46631329	46736564	373	24	120000	-1.3993	0.91913	TMEM247	76353	25kb
ENSG00000250565	2	46692889	46794696	317	28	120000	-1.6317	0.94863	ATP6V1E2	76353	25kb
ENSG00000119729	2	46743945	46835260	226	33	120000	-1.8369	0.96689	RHOQ	76353	25kb
ENSG00000151665	2	46783076	46869258	216	25	120000	-1.3876	0.91737	PIGF	76353	25kb
ENSG00000119878	2	46818555	46877881	176	23	120000	-1.0761	0.85907	CRIPT	76353	25kb
ENSG00000171150	2	46901091	47015268	436	30	120000	2.8257	0.0023587	SOCS5	76353	25kb
ENSG00000222005	2	47018807	47074796	210	45	120000	3.4255	0.00030686	LINC01118	76353	25kb

ENSG00000239332	2	47018836	47111146	354	60	120000	2.9203	0.0017483	LINC01119	76353	25kb
ENSG00000180398	2	47104009	47193994	405	37	120000	-0.085594	0.53411	MCFD2	76353	25kb
ENSG00000068724	2	47118296	47328276	967	78	120000	0.9717	0.1656	TTC7A	76353	25kb
ENSG00000239605	2	47247677	47407517	690	69	120000	1.2959	0.097498	C2orf61	76353	25kb
ENSG00000273269	2	47268080	47428650	645	67	120000	0.88595	0.18782	RP11-761B3.1	76353	25kb
ENSG00000143933	2	47362221	47428740	211	32	120000	-0.86508	0.8065	CALM2	76353	25kb
ENSG00000119888	2	47547297	47639740	259	38	120000	-1.6375	0.94923	EPCAM	76353	25kb
ENSG00000095002	2	47605108	47814450	594	53	120000	-1.5934	0.94447	MSH2	76353	25kb
ENSG00000184261	2	47718720	47823078	289	59	120000	-2.2294	0.98711	KCNK12	76353	25kb
ENSG00000269563	2	47774601	47826998	191	53	120000	-1.4676	0.92889	AC138655.1	76353	25kb
ENSG00000116062	2	47897669	48062240	456	45	120000	0.22246	0.41198	MSH6	76353	25kb
ENSG00000138081	2	47991455	48157932	415	25	120000	0.3533	0.36193	FBXO11	76353	25kb
ENSG00000170802	2	48516776	48631433	324	22	120000	-1.1097	0.86644	FOXN2	76353	25kb
ENSG00000162869	2	48642737	48767525	408	30	120000	-1.6878	0.95428	PPP1R21	76353	25kb
ENSG00000243244	2	48731522	48851025	456	40	120000	-1.2746	0.89878	STON1	76353	25kb
ENSG00000068781	2	48732064	49028654	1142	86	120000	-0.41557	0.66114	STON1-GTF2A1L	76353	25kb
ENSG00000242441	2	48819937	48985287	629	48	120000	0.15864	0.43698	GTF2A1L	76353	25kb
ENSG00000138039	2	48834428	49007880	670	58	120000	0.16522	0.43439	LHCGR	76353	25kb
ENSG00000170820	2	49164296	49406676	873	40	120000	1.0266	0.15231	FSHR	76353	25kb
ENSG00000179915	2	50120643	51284674	3615	133	120000	0.97721	0.16423	NRXN1	76353	25kb
ENSG00000115239	2	53734810	54112170	1236	80	120000	0.42747	0.33452	GPR75-ASB3	76353	25kb
ENSG00000270898	2	53872430	54112297	705	33	120000	0.77666	0.21868	ASB3	76353	25kb
ENSG00000143942	2	53969929	54027333	168	24	120000	-0.42051	0.66294	CHAC2	76353	25kb
ENSG00000068912	2	53989181	54070956	226	27	120000	0.2998	0.38216	ERLEC1	76353	25kb
ENSG00000119737	2	54055050	54112126	158	25	120000	0.51689	0.30262	GPR75	76353	25kb
ENSG00000068878	2	54066204	54222977	435	28	120000	-0.37109	0.64471	PSME4	76353	25kb
ENSG00000170634	2	54172975	54557437	1078	47	120000	0.53673	0.29573	ACYP2	76353	25kb
ENSG00000178021	2	54455315	54508409	171	18	120000	0.045395	0.4819	TSPYL6	76353	25kb
ENSG00000177994	2	54532171	54635879	300	29	120000	0.59987	0.2743	C2orf73	76353	25kb
ENSG00000115306	2	54658422	54921812	777	31	120000	-1.7322	0.95838	SPTBN1	76353	25kb
ENSG00000214595	2	54925636	55224157	955	72	120000	0.5346	0.29646	EML6	76353	25kb
ENSG00000115310	2	55174325	55364757	612	40	120000	1.6652	0.047933	RTN4	76353	25kb
ENSG00000162994	2	55376927	55484699	306	27	120000	-0.52714	0.70095	CLHC1	76353	25kb
ENSG00000143947	2	55434039	55487989	151	24	120000	-0.59632	0.72452	RPS27A	76353	25kb
ENSG00000085760	2	55438731	55521483	243	37	120000	-0.19614	0.57775	MTIF2	76353	25kb
ENSG00000115355	2	55489978	55672057	604	26	120000	1.6961	0.044931	CCDC88A	76353	25kb
ENSG00000163001	2	55721740	55798015	225	30	120000	1.2325	0.10888	CCDC104	76353	25kb
ENSG00000138041	2	55749428	55871015	372	28	120000	0.93872	0.17394	SMEK2	76353	25kb
ENSG00000138035	2	55836400	55946045	399	29	120000	0.28622	0.38736	PNPT1	76353	25kb
ENSG00000115380	2	56068102	56176274	337	33	120000	-0.58718	0.72146	EFEMP1	76353	25kb
ENSG00000055813	2	56386258	56638308	781	75	120000	-0.37026	0.64441	CCDC85A	76353	25kb
ENSG00000028116	2	58109786	58412055	616	32	120000	-0.69183	0.75548	VRK2	76353	25kb
ENSG00000115392	2	58361378	58493507	276	25	120000	-0.59942	0.72555	FANCL	76353	25kb
ENSG00000119866	2	60653302	60805702	263	42	120000	2.0333	0.02101	BCL11A	76353	25kb
ENSG00000115421	2	60958365	61054220	233	24	120000	0.002663	0.49894	PAPOLG	76353	25kb
ENSG00000162924	2	61083656	61183745	162	26	120000	-0.86174	0.80558	REL	76353	25kb
ENSG00000162927	2	61142357	61270394	200	34	120000	-0.64097	0.73923	PUS10	76353	25kb
ENSG00000162928	2	61219360	61304125	139	24	120000	-0.28183	0.61096	PEX13	76353	25kb
ENSG00000162929	2	61268006	61416960	300	26	120000	1.0122	0.15571	KIAA1841	76353	25kb
ENSG00000237651	2	61347243	61416964	184	15	120000	1.0338	0.15061	C2orf74	76353	25kb
ENSG00000173209	2	61379553	61443338	132	14	120000	0.98324	0.16274	AHSA2	76353	25kb
ENSG00000115464	2	61389598	61722904	687	27	120000	1.0208	0.15368	USP34	76353	25kb
ENSG00000082898	2	61679984	61790761	202	18	120000	0.50534	0.30666	XPO1	76353	25kb
ENSG00000170264	2	62026989	62106278	211	25	120000	-0.38876	0.65127	FAM161A	76353	25kb
ENSG00000115484	2	62070224	62140939	200	24	120000	-0.47865	0.6839	CCT4	76353	25kb
ENSG00000173163	2	62090859	62399382	571	31	120000	0.39178	0.34761	COMMD1	76353	25kb
ENSG00000170340	2	62398248	62476866	142	22	120000	1.8746	0.030422	B3GNT2	76353	25kb
ENSG00000186889	2	62702356	62764029	227	37	120000	-0.87698	0.80975	TMEM17	76353	25kb
ENSG00000115504	2	62875986	63298622	455	40	120000	2.0225	0.021565	EHBP1	76353	25kb
ENSG00000115507	2	63252192	63309971	45	19	120000	2.9828	0.0014283	OTX1	76353	25kb
ENSG00000143951	2	63323518	64079977	1534	34	120000	0.58781	0.27833	WDPCP	76353	25kb
ENSG00000014641	2	63790743	63859331	127	14	120000	0.044643	0.4822	MDH1	76353	25kb
ENSG00000169764	2	64043074	64143696	212	33	120000	-0.43823	0.66939	UGP2	76353	25kb
ENSG00000143952	2	64094280	64271206	484	35	120000	-1.0203	0.84621	VPS54	76353	25kb
ENSG00000197329	2	64294786	64396588	271	26	120000	-0.30824	0.62105	PELI1	76353	25kb
ENSG00000119862	2	64656103	64713515	162	24	120000	2.3854	0.0085307	LGALSL	76353	25kb
ENSG00000119844	2	64726465	64845139	225	18	120000	0.7172	0.23663	AFTPH	76353	25kb
ENSG00000179833	2	64833755	65003139	337	34	120000	-1.0949	0.86323	SERTAD2	76353	25kb
ENSG00000115902	2	65190611	65275999	253	39	120000	1.6049	0.054256	SLC1A4	76353	25kb
ENSG00000011523	2	65258500	65339138	248	28	120000	-1.6114	0.94645	CEP68	76353	25kb
ENSG00000138069	2	65272835	65382240	322	23	120000	-1.6679	0.95233	RAB1A	76353	25kb
ENSG00000138071	2	65429887	65523387	245	23	120000	-1.0305	0.84862	ACTR2	76353	25kb
ENSG00000198369	2	65512985	65684771	499	34	120000	-0.75609	0.7752	SPRED2	76353	25kb
ENSG00000143995	2	66635584	66826001	468	68	120000	1.3677	0.085704	MEIS1	76353	25kb
ENSG00000143971	2	67599451	67662677	169	13	120000	-0.93819	0.82593	ETAA1	76353	25kb
ENSG00000197223	2	68243262	68363080	228	50	120000	0.70639	0.23997	C1D	76353	25kb
ENSG00000243667	2	68325068	68409692	155	20	120000	-0.42361	0.66408	WDR92	76353	25kb
ENSG00000273398	2	68333370	68513362	331	19	120000	-0.56032	0.71237	RP11-474G23.1	76353	25kb
ENSG00000115946	2	68359976	68428370	136	13	120000	-0.41072	0.65936	PNO1	76353	25kb
ENSG00000221823	2	68380989	68508369	230	15	120000	-0.35735	0.63959	PPP3R1	76353	25kb
ENSG00000119865	2	68486303	68572183	214	34	120000	-1.9915	0.97679	CNRIP1	76353	25kb
ENSG00000115956	2	68567305	68649585	436	21	120000	-0.75881	0.77602	PLEK	76353	25kb
ENSG00000204923	2	68661551	68719390	125	15	120000	-1.0194	0.846	FBXO48	76353	25kb
ENSG00000169621	2	68669693	68883004	547	19	120000	-1.5829	0.94328	APLF	76353	25kb
ENSG00000169618	2	68845721	68907708	185	26	120000	1.5628	0.059053	PROKR1	76353	25kb
ENSG00000163219	2	68881733	69078965	763	48	120000	1.1245	0.13041	ARHGAP25	76353	25kb
ENSG00000163217	2	69067613	69123649	134	19	120000	0.52456	0.29994	BMP10	76353	25kb
ENSG00000183607	2	69147364	69205102	159	20	120000	0.58188	0.28032	GKN2	76353	25kb
ENSG00000169605	2	69176705	69233106	210	16	120000	-0.74531	0.77196	GKN1	76353	25kb
ENSG00000169604	2	69215310	69501459	790	70	120000	0.33534	0.36868	ANTXR1	76353	25kb
ENSG00000198380	2	69521905	69639382	232	11	120000	-0.8038	0.78924	GFPT1	76353	25kb
ENSG00000169599	2	69597882	69689760	186	11	120000	-0.94986	0.82891	NFU1	76353	25kb
ENSG00000115977	2	69663532	69926481	438	36	120000	0.44861	0.32686	AAK1	76353	25kb
ENSG00000196975	2	69846557	70078596	526	33	120000	-0.19275	0.57642	ANXA4	76353	25kb
ENSG00000087338	2	70031774	70133528	189	14	120000	-1.1413	0.87313	GMCL1	76353	25kb
ENSG00000124380	2	70095692	70157707	105	8	120000	-0.75223	0.77404	SNRNP27	76353	25kb

ENSG00000059728	2	70099820	70195077	173	14	120000	-0.88559	0.81208	MXD1	76353	25kb
ENSG00000268328	2	70104482	70154841	88	6	120000	-0.6142	0.73046	AC019206.1	76353	25kb
ENSG00000244617	2	70162226	70214397	100	13	120000	-1.0975	0.86378	ASPRV1	76353	25kb
ENSG00000169564	2	70289585	70341332	90	6	120000	-0.84031	0.79963	PCBP1	76353	25kb
ENSG00000115998	2	70352012	70500747	306	17	120000	-1.1738	0.87976	C2orf42	76353	25kb
ENSG00000116001	2	70411576	70500792	168	8	120000	-0.61682	0.73132	TIA1	76353	25kb
ENSG00000116005	2	70459518	70533323	143	7	120000	-0.57985	0.71899	PCYOX1	76353	25kb
ENSG00000143977	2	70483494	70545903	112	8	120000	-0.52048	0.69864	SNRPG	76353	25kb
ENSG00000035141	2	70498107	70554222	116	9	120000	-0.35754	0.63966	FAM136A	76353	25kb
ENSG00000163235	2	70649412	70806325	496	53	120000	2.7025	0.0034412	TGFA	76353	25kb
ENSG00000075340	2	70809750	71020357	801	50	120000	0.49621	0.30987	ADD2	76353	25kb
ENSG00000183733	2	70979442	71042775	187	24	120000	1.0487	0.14715	FIGLA	76353	25kb
ENSG00000152672	2	71010775	71072732	292	29	120000	2.3442	0.009535	CLEC4F	76353	25kb
ENSG00000116031	2	71032347	71087952	270	21	120000	1.8214	0.034277	CD207	76353	25kb
ENSG00000116035	2	71102720	71185576	275	37	120000	0.89093	0.18648	VAX2	76353	25kb
ENSG00000116039	2	71138012	71217536	256	43	120000	0.75422	0.22536	ATP6V1B1	76353	25kb
ENSG00000258881	2	71141448	71247466	338	56	120000	0.54573	0.29263	AC007040.11	76353	25kb
ENSG00000144031	2	71180510	71237626	162	32	120000	0.44858	0.32687	ANKRD53	76353	25kb
ENSG00000144043	2	71188068	71247075	170	33	120000	0.34918	0.36348	TEX261	76353	25kb
ENSG00000124357	2	71266474	71331935	270	21	120000	-0.47391	0.68222	NAGK	76353	25kb
ENSG00000124370	2	71311814	71382369	233	27	120000	-0.62504	0.73403	MCEE	76353	25kb
ENSG00000124383	2	71332444	71402231	243	33	120000	-1.1826	0.88152	MPHOSPH10	76353	25kb
ENSG00000124374	2	71384869	71479213	340	29	120000	-1.242	0.89289	PAIP2B	76353	25kb
ENSG00000075292	2	71478691	71687199	606	28	120000	-1.0354	0.84976	ZNF638	76353	25kb
ENSG00000135636	2	71655852	71938898	958	109	120000	-1.2386	0.89226	DYSF	76353	25kb
ENSG00000003137	2	72331367	72400167	157	27	120000	0.74662	0.22765	CYP26B1	76353	25kb
ENSG00000144036	2	72378113	73078170	1049	25	120000	1.7796	0.037573	EXOC6B	76353	25kb
ENSG00000116096	2	73089489	73144287	60	10	120000	0.8626	0.19418	SPR	76353	25kb
ENSG00000135638	2	73118389	73187020	97	22	120000	2.421	0.0077382	EMX1	76353	25kb
ENSG00000144040	2	73144165	73327747	334	29	120000	0.23778	0.40603	SFXN5	76353	25kb
ENSG00000135631	2	73275510	73408849	368	25	120000	0.66296	0.25368	RAB11FIP5	76353	25kb
ENSG00000214513	2	73404386	73464641	169	21	120000	0.72873	0.23308	NOTO	76353	25kb
ENSG00000135632	2	73416350	73479365	171	22	120000	0.73965	0.22976	SMYD5	76353	25kb
ENSG00000135617	2	73430134	73485366	146	20	120000	1.0018	0.15823	PRADC1	76353	25kb
ENSG00000135624	2	73435548	73505149	157	24	120000	1.1096	0.13358	CCT7	76353	25kb
ENSG00000163013	2	73456810	73536559	140	28	120000	1.232	0.10898	FBXO41	76353	25kb
ENSG00000135625	2	73493058	73545833	66	15	120000	0.13523	0.44621	EGR4	76353	25kb
ENSG00000116127	2	73587886	73862920	497	17	120000	-1.5187	0.93558	ALMS1	76353	25kb
ENSG00000144035	2	73842959	73894520	158	12	120000	-1.0216	0.84651	NAT8	76353	25kb
ENSG00000144034	2	73931231	73989527	95	5	120000	0.36795	0.35646	TPRKB	76353	25kb
ENSG00000144048	2	73964311	74032284	44	5	120000	0.59887	0.27463	DUSP11	76353	25kb
ENSG00000187833	2	73986316	74069274	64	12	120000	-0.18044	0.5716	C2orf78	76353	25kb
ENSG00000124356	2	74031086	74125786	239	29	120000	-1.7281	0.95801	STAMBP	76353	25kb
ENSG00000163017	2	74094441	74171992	294	33	120000	-2.3175	0.98976	ACTG2	76353	25kb
ENSG00000114956	2	74128953	74211088	255	23	120000	-1.5924	0.94435	DGUOK	76353	25kb
ENSG00000187605	2	74204840	74360303	336	26	120000	1.1623	0.12255	TET3	76353	25kb
ENSG00000217702	2	74336599	74387521	158	20	120000	-2.0656	0.98057	MGC10955	76353	25kb
ENSG00000163170	2	74337525	74400121	175	21	120000	-2.0429	0.97947	BOLA3	76353	25kb
ENSG00000114978	2	74354655	74431025	160	18	120000	-1.8148	0.96522	MOB1A	76353	25kb
ENSG00000267958	2	74393517	74443627	91	12	120000	-1.9366	0.9736	CATX-2	76353	25kb
ENSG00000065911	2	74400689	74469692	121	16	120000	-1.7723	0.96183	MTHFD2	76353	25kb
ENSG00000264324	2	74413731	74615504	297	33	120000	-1.372	0.91497	RP11-287D1.3	76353	25kb
ENSG00000188687	2	74418369	74595541	272	32	120000	-1.4748	0.92987	SLC4A5	76353	25kb
ENSG00000204843	2	74563281	74644214	98	9	120000	0.10874	0.4567	DCTN1	76353	25kb
ENSG00000159239	2	74616304	74673718	56	7	120000	0.28029	0.38963	C2orf81	76353	25kb
ENSG00000005448	2	74623805	74677882	47	6	120000	0.055242	0.47797	WDR54	76353	25kb
ENSG00000114993	2	74627963	74694549	58	7	120000	-0.0069336	0.50277	RTKN	76353	25kb
ENSG00000115274	2	74657150	74713011	44	8	120000	-0.39611	0.65399	INO80B	76353	25kb
ENSG00000239779	2	74660456	74713018	41	7	120000	-0.37774	0.64719	WBP1	76353	25kb
ENSG00000115275	2	74663184	74717537	43	8	120000	-0.43143	0.66692	MOGS	76353	25kb
ENSG00000204822	2	74674085	74725449	42	7	120000	-0.51171	0.69557	MRPL53	76353	25kb
ENSG00000135637	2	74674113	74735535	51	8	120000	-0.42754	0.66551	CCDC142	76353	25kb
ENSG00000115282	2	74685200	74747013	60	9	120000	-0.49051	0.68811	TTC31	76353	25kb
ENSG00000179528	2	74699644	74755443	48	9	120000	-0.5031	0.69255	LBX2	76353	25kb
ENSG00000115289	2	74707170	74760707	44	7	120000	-0.5806	0.71925	PCGF1	76353	25kb
ENSG00000115297	2	74715590	74769274	48	8	120000	-0.39117	0.65216	TLX2	76353	25kb
ENSG00000144045	2	74720258	74778463	52	9	120000	-0.23137	0.59148	DQX1	76353	25kb
ENSG00000115307	2	74728772	74782066	49	11	120000	-0.25497	0.60063	AUP1	76353	25kb
ENSG00000115317	2	74731504	74785472	45	10	120000	-0.30947	0.62152	HTRA2	76353	25kb
ENSG00000115318	2	74734541	74807817	65	15	120000	-0.51668	0.69731	LOXL3	76353	25kb
ENSG00000115325	2	74751153	74809681	49	14	120000	-0.48614	0.68657	DOK1	76353	25kb
ENSG00000159374	2	74760010	74900465	145	21	120000	-0.11565	0.54603	M1AP	76353	25kb
ENSG00000135622	2	74856355	74934186	117	8	120000	-0.12035	0.5479	SEMA4F	76353	25kb
ENSG00000159399	2	75036108	75145486	306	38	120000	1.3436	0.089546	HK2	76353	25kb
ENSG00000115350	2	75160619	75222255	160	19	120000	-1.1549	0.87593	POLE4	76353	25kb
ENSG00000115353	2	75248590	75451826	571	64	120000	-0.044402	0.51771	TACR1	76353	25kb
ENSG00000115363	2	75671428	75821848	452	61	120000	-0.79142	0.78565	EVA1A	76353	25kb
ENSG00000115364	2	75848909	75942977	288	21	120000	-0.28165	0.61089	MRPL19	76353	25kb
ENSG00000005436	2	75854126	75963115	312	22	120000	-0.39192	0.65244	GCFC2	76353	25kb
ENSG00000176204	2	76949845	77845445	2972	135	120000	-0.23836	0.5942	LRRTM4	76353	25kb
ENSG00000143954	2	79227812	79280631	259	14	120000	-1.037	0.85012	REG3G	76353	25kb
ENSG00000172023	2	79287156	79340145	163	29	120000	-1.581	0.94307	REG1B	76353	25kb
ENSG00000115386	2	79322488	79375545	164	30	120000	-1.211	0.88706	REG1A	76353	25kb
ENSG00000172016	2	79359132	79411879	180	31	120000	-1.0682	0.85728	REG3A	76353	25kb
ENSG00000066032	2	79387357	80900905	4555	290	120000	-0.40688	0.65795	CTNNA2	76353	25kb
ENSG00000162951	2	80490483	80556874	138	36	120000	-0.28909	0.61374	LRRTM1	76353	25kb
ENSG00000163541	2	84625647	84712169	180	13	120000	1.054	0.14593	SUCLG1	76353	25kb
ENSG00000115423	2	84718579	85071713	856	24	120000	-0.099329	0.53956	DNAH6	76353	25kb
ENSG00000186854	2	85023774	85159132	379	31	120000	-0.5906	0.7226	TRABD2A	76353	25kb
ENSG00000269417	2	85097209	85147345	130	13	120000	-0.03781	0.51508	AC022210.1	76353	25kb
ENSG00000034510	2	85107749	85158795	124	13	120000	0.18746	0.42565	TMSB10	76353	25kb
ENSG00000176407	2	85173216	85311595	263	25	120000	0.53022	0.29798	KCMF1	76353	25kb
ENSG00000152284	2	85335533	85562511	816	66	120000	-0.20447	0.58101	TCF7L1	76353	25kb
ENSG00000152291	2	85520147	85580548	198	26	120000	-0.30528	0.61992	TGOLN2	76353	25kb
ENSG00000042445	2	85544211	85606743	156	24	120000	0.24408	0.40358	RETSAT	76353	25kb
ENSG00000115459	2	85556517	85643875	262	27	120000	0.37704	0.35307	ELMOD3	76353	25kb

ENSG00000042493	2	85596871	85670555	248	33	120000	0.083856	0.46659	CAPG	76353	25kb
ENSG00000152292	2	85620844	85689152	239	36	120000	-0.58448	0.72055	SH2D6	76353	25kb
ENSG00000168906	2	85741288	85797403	178	19	120000	1.7239	0.042363	MAT2A	76353	25kb
ENSG00000115486	2	85746846	85813670	208	20	120000	1.8864	0.029618	GGCX	76353	25kb
ENSG00000118640	2	85763685	85834154	189	24	120000	1.6031	0.05446	VAMP8	76353	25kb
ENSG00000168899	2	85786531	85845535	150	21	120000	1.5379	0.062035	VAMP5	76353	25kb
ENSG00000168894	2	85797848	85849736	115	20	120000	1.3534	0.087957	RNF181	76353	25kb
ENSG00000168890	2	85800671	85855319	118	20	120000	1.1473	0.12563	TMEM150A	76353	25kb
ENSG00000168883	2	85804979	85901403	165	27	120000	0.23674	0.40643	USP39	76353	25kb
ENSG00000168887	2	85808777	85864189	103	19	120000	0.33505	0.36879	C2orf68	76353	25kb
ENSG00000168878	2	85859437	85920864	116	25	120000	-0.86711	0.80706	SFTPB	76353	25kb
ENSG00000115523	2	85887298	85950977	189	20	120000	-0.29964	0.61777	GNLY	76353	25kb
ENSG00000168874	2	85953467	86040189	257	39	120000	-0.12078	0.54807	ATOH8	76353	25kb
ENSG00000115525	2	86041267	86141137	336	21	120000	0.71198	0.23824	ST3GAL5	76353	25kb
ENSG00000068654	2	86222339	86358278	322	21	120000	-0.066755	0.52661	POLR1A	76353	25kb
ENSG00000132300	2	86308305	86394280	212	20	120000	-0.14658	0.55827	PTCD3	76353	25kb
ENSG00000132305	2	86346055	86447893	269	13	120000	0.075038	0.47009	IMMT	76353	25kb
ENSG00000132313	2	86401478	86465917	201	14	120000	0.11276	0.45511	MRPL35	76353	25kb
ENSG00000068615	2	86416116	86590206	556	22	120000	-0.78433	0.78358	REEP1	76353	25kb
ENSG00000115548	2	86642770	86744839	252	18	120000	-1.3123	0.90529	KDM3A	76353	25kb
ENSG00000115561	2	86705554	86973245	510	30	120000	-1.0184	0.84576	CHMP3	76353	25kb
ENSG00000249884	2	86707791	86973245	502	35	120000	-1.0674	0.8571	RNF103-CHMP3	76353	25kb
ENSG00000239305	2	86805516	86875989	175	23	120000	-0.89537	0.81471	RNF103	76353	25kb
ENSG00000153561	2	86922296	87030164	153	18	120000	-0.54713	0.70786	RMND5A	76353	25kb
ENSG00000153563	2	86986729	87060519	182	22	120000	-0.68979	0.75484	CD8A	76353	25kb
ENSG00000172116	2	87017462	87114047	186	23	120000	0.39828	0.34521	CD8B	76353	25kb
ENSG00000229615	2	87064023	87117827	67	10	120000	1.2628	0.10333	AC111200.1	76353	25kb
ENSG00000187627	2	87110076	87266104	26	7	120000	1.8258	0.033938	RGPD1	76353	25kb
ENSG00000183281	2	87204682	87273975	1	1	120000	2.1568	0.015512	PLGLB1	76353	25kb
ENSG00000125551	2	88022606	88083312	1	1	120000	-0.086456	0.53445	PLGLB2	76353	25kb
ENSG00000185304	2	88030474	88310309	82	13	120000	-0.49015	0.68799	RGPD2	76353	25kb
ENSG00000172086	2	88301724	88380248	160	19	120000	-1.5314	0.93716	KRCC1	76353	25kb
ENSG00000115593	2	88342299	88437906	283	36	120000	-1.7416	0.95921	SMYD1	76353	25kb
ENSG00000163586	2	88397510	88452635	175	31	120000	-1.0469	0.85243	FABP1	76353	25kb
ENSG00000144115	2	88444835	88511146	164	27	120000	-1.2526	0.89483	THNSL2	76353	25kb
ENSG00000172073	2	88799169	88854102	215	20	120000	0.62508	0.26596	TEX37	76353	25kb
ENSG00000172071	2	88831259	88952094	203	28	120000	1.6206	0.052552	EIF2AK3	76353	25kb
ENSG00000153574	2	88966162	89075452	244	11	120000	0.15121	0.4399	RPIA	76353	25kb
ENSG00000163060	2	95512178	95567574	85	11	120000	-0.1972	0.57817	TEKT4	76353	25kb
ENSG00000172005	2	95666422	95744737	84	18	120000	1.2869	0.099069	MAL	76353	25kb
ENSG00000144029	2	95727952	95840179	102	19	120000	1.5375	0.062086	MRPS5	76353	25kb
ENSG00000144026	2	95788075	95856158	56	12	120000	1.4576	0.07248	ZNF514	76353	25kb
ENSG00000163067	2	95806177	95875065	65	16	120000	1.4857	0.068682	ZNF2	76353	25kb
ENSG00000155066	2	95915201	95982056	78	12	120000	1.7356	0.041315	PROM2	76353	25kb
ENSG00000115041	2	95938052	96076825	146	18	120000	2.0533	0.02002	KCNIP3	76353	25kb
ENSG00000115042	2	96043469	96107364	31	12	120000	0.78873	0.21513	FAHD2A	76353	25kb
ENSG00000144015	2	96232766	96290526	25	13	120000	0.99672	0.15945	TRIM43	76353	25kb
ENSG00000174501	2	96489587	96682541	140	18	120000	1.1497	0.12514	ANKRD36C	76353	25kb
ENSG00000186281	2	96662694	96730199	38	8	120000	1.4776	0.069761	GPAT2	76353	25kb
ENSG00000222040	2	96753707	96806984	51	9	120000	1.2489	0.10585	ADRA2B	76353	25kb
ENSG00000188886	2	96764589	96829175	74	11	120000	1.4997	0.066843	ASTL	76353	25kb
ENSG00000158050	2	96783905	96836179	62	12	120000	1.6388	0.050624	DUSP2	76353	25kb
ENSG00000084090	2	96825597	96899563	80	13	120000	0.40339	0.34333	STARD7	76353	25kb
ENSG00000135956	2	96889254	96956732	76	17	120000	1.5676	0.058487	TMEM127	76353	25kb
ENSG00000144021	2	96906870	96964087	66	16	120000	1.9655	0.024676	CIAO1	76353	25kb
ENSG00000144028	2	96915074	96996297	87	16	120000	1.6079	0.053929	SNRNP200	76353	25kb
ENSG00000198885	2	96966069	97019091	55	10	120000	1.3216	0.093146	ITPRIPL1	76353	25kb
ENSG00000121152	2	96976525	97064583	95	15	120000	1.8084	0.035273	NCAPH	76353	25kb
ENSG00000196843	2	97177480	97243375	88	20	120000	0.2383	0.40583	ARID5A	76353	25kb
ENSG00000114982	2	97233907	97333524	106	18	120000	0.45185	0.32569	KANSL3	76353	25kb
ENSG00000114988	2	97346666	97430801	95	10	120000	0.98037	0.16345	LMAN2L	76353	25kb
ENSG00000158158	2	97401639	97502628	130	11	120000	0.72611	0.23388	CNNM4	76353	25kb
ENSG00000168763	2	97456982	97524648	76	8	120000	0.18927	0.42494	CNNM3	76353	25kb
ENSG00000163126	2	97465263	97548671	81	10	120000	-0.14343	0.55702	ANKRD23	76353	25kb
ENSG00000213337	2	97477348	97548832	74	9	120000	-0.17087	0.56784	ANKRD39	76353	25kb
ENSG00000168758	2	97500453	97561494	85	13	120000	-1.0454	0.85209	SEMA4C	76353	25kb
ENSG00000168754	2	97516620	97709175	359	27	120000	-1.0721	0.85816	FAM178B	76353	25kb
ENSG00000144199	2	97724320	97785619	42	6	120000	-0.4339	0.66782	FAHD2B	76353	25kb
ENSG00000135976	2	97754233	97955258	86	8	120000	-0.6093	0.72884	ANKRD36	76353	25kb
ENSG00000135940	2	98237503	98289846	58	9	120000	0.18019	0.4285	COX5B	76353	25kb
ENSG00000115073	2	98247431	98305570	63	9	120000	0.19247	0.42369	ACTR1B	76353	25kb
ENSG00000115085	2	98305023	98381325	142	23	120000	0.46093	0.32242	ZAP70	76353	25kb
ENSG00000075568	2	98347799	98637388	559	23	120000	0.67028	0.25134	TMEM131	76353	25kb
ENSG00000168658	2	98678579	98954762	698	40	120000	0.46306	0.32166	VWA3B	76353	25kb
ENSG00000222000	2	98922852	98997468	265	21	120000	0.093539	0.46274	AC092675.3	76353	25kb
ENSG00000144191	2	98937618	99040064	274	29	120000	-0.27633	0.60885	CNGA3	76353	25kb
ENSG00000040933	2	99036317	99235853	310	13	120000	-0.87433	0.80903	INPP4A	76353	25kb
ENSG00000183513	2	99190773	99249978	90	9	120000	-1.2537	0.89503	COA5	76353	25kb
ENSG00000115446	2	99200042	99259978	95	9	120000	-1.1467	0.87424	UNC50	76353	25kb
ENSG00000071073	2	99210569	99372589	336	28	120000	-0.47224	0.68162	MGAT4A	76353	25kb
ENSG00000196872	2	99385309	99577722	490	27	120000	-0.86905	0.80759	KIAA1211L	76353	25kb
ENSG00000135951	2	99588724	99796427	522	19	120000	0.22783	0.40989	TSGA10	76353	25kb
ENSG00000273045	2	99732948	99792950	135	10	120000	0.15997	0.43645	C2ORF15	76353	25kb
ENSG00000241962	2	99732948	99964204	533	22	120000	0.062331	0.47515	C2orf15	76353	25kb
ENSG00000144182	2	99746418	99804620	136	10	120000	0.14691	0.4416	LIPT1	76353	25kb
ENSG00000273155	2	99746461	99836761	210	17	120000	-0.081001	0.53228	MRPL30	76353	25kb
ENSG00000158411	2	99752890	99822521	164	14	120000	0.040461	0.48386	MITD1	76353	25kb
ENSG00000185414	2	99772542	99839089	155	16	120000	-0.17197	0.56827	MRPL30	76353	25kb
ENSG00000185674	2	99833709	99896745	142	22	120000	0.10166	0.45951	LYG2	76353	25kb
ENSG00000144214	2	99875701	99946205	168	18	120000	0.536	0.29598	LYG1	76353	25kb
ENSG00000115514	2	99910445	99982165	175	20	120000	0.3588	0.35987	TXNDC9	76353	25kb
ENSG00000158417	2	99928816	100042789	260	18	120000	0.51557	0.30308	EIF5B	76353	25kb
ENSG00000135945	2	99991938	100131497	290	15	120000	0.42665	0.33482	REV1	76353	25kb
ENSG00000144218	2	100137323	100784201	1360	64	120000	0.87584	0.19056	AFF3	76353	25kb
ENSG00000170500	2	100864753	100964195	358	17	120000	-0.54601	0.70747	LONRF2	76353	25kb
ENSG00000269383	2	100961705	101012007	200	10	120000	-0.90942	0.81844	AC012493.2	76353	25kb

ENSG00000115526	2	100983327	101059118	252	26	120000	-0.58239	0.71985	CHST10	76353	25kb
ENSG00000204640	2	101061944	101124742	380	17	120000	-0.57907	0.71873	NMS	76353	25kb
ENSG00000115539	2	101154152	101218197	158	22	120000	0.45194	0.32566	PDCL3	76353	25kb
ENSG00000170485	2	101411614	101638291	669	72	120000	0.63439	0.26291	NPAS2	76353	25kb
ENSG00000071082	2	101593177	101665494	181	23	120000	-0.0038612	0.50154	RPL31	76353	25kb
ENSG00000204634	2	101599079	101894328	675	45	120000	0.026427	0.48946	TBC1D8	76353	25kb
ENSG00000158435	2	101844264	101911778	109	18	120000	1.1428	0.12655	CNOT11	76353	25kb
ENSG00000163162	2	101862681	101950163	149	25	120000	0.91061	0.18125	RNF149	76353	25kb
ENSG00000175874	2	101937013	102029057	222	23	120000	0.12684	0.44953	CREG2	76353	25kb
ENSG00000196460	2	101988823	102116165	454	28	120000	-0.43532	0.66833	RFX8	76353	25kb
ENSG00000071054	2	102288312	102536149	396	26	120000	-1.4331	0.92409	MAP4K4	76353	25kb
ENSG00000233404	2	102483955	102536150	74	14	120000	-1.481	0.9307	FLJ20373	76353	25kb
ENSG00000115590	2	102583306	102670006	250	22	120000	-0.41013	0.65915	IL1R2	76353	25kb
ENSG00000115594	2	102656004	102821334	509	41	120000	-0.53829	0.70481	IL1R1	76353	25kb
ENSG00000115598	2	102778433	102881462	341	22	120000	0.22106	0.41252	IL1RL2	76353	25kb
ENSG00000115602	2	102902962	102993497	340	20	120000	0.93742	0.17427	IL1RL1	76353	25kb
ENSG00000115604	2	102902989	103040218	502	24	120000	1.2544	0.10485	IL18R1	76353	25kb
ENSG00000115607	2	103010149	103094025	341	18	120000	1.1381	0.12754	IL18RAP	76353	25kb
ENSG00000180251	2	103064762	103175431	450	24	120000	0.096165	0.46169	SLC9A4	76353	25kb
ENSG00000115616	2	103211166	103352777	352	42	120000	0.66798	0.25207	SLC9A2	76353	25kb
ENSG00000135953	2	103307299	103378347	148	16	120000	1.4187	0.077989	MFSD9	76353	25kb
ENSG00000170417	2	103328367	103485352	294	20	120000	0.5536	0.28993	TMEM182	76353	25kb
ENSG00000198914	2	105446969	105501929	88	18	120000	0.65753	0.25542	POU3F3	76353	25kb
ENSG00000135972	2	105629441	105741418	320	12	120000	-1.0125	0.84435	MRPS9	76353	25kb
ENSG00000135973	2	105833200	105884924	122	22	120000	1.0653	0.14338	GPR45	76353	25kb
ENSG00000268556	2	105842759	105894601	123	26	120000	0.88586	0.18785	AC012360.1	76353	25kb
ENSG00000135966	2	105855871	105971491	340	33	120000	0.54092	0.29428	TGFBRAP1	76353	25kb
ENSG00000268809	2	105857490	105907585	135	27	120000	0.65385	0.2566	AC012360.2	76353	25kb
ENSG00000135974	2	105928816	105990668	188	23	120000	0.54468	0.29299	C2orf49	76353	25kb
ENSG00000115641	2	105949169	106079970	445	71	120000	-0.14616	0.5581	FHL2	76353	25kb
ENSG00000071051	2	106336354	106535730	724	42	120000	-1.0389	0.85057	NCK2	76353	25kb
ENSG00000119147	2	106654702	106719615	241	39	120000	-0.83583	0.79838	C2orf40	76353	25kb
ENSG00000115652	2	106684759	106835795	465	40	120000	-1.2069	0.88626	UXS1	76353	25kb
ENSG00000153165	2	106996446	107109832	77	22	120000	0.95748	0.16916	RGPD3	76353	25kb
ENSG00000269000	2	107074728	107128848	105	16	120000	0.042055	0.48323	AC108868.1	76353	25kb
ENSG00000144057	2	107393056	107528564	433	34	120000	-1.2279	0.89026	ST6GAL2	76353	25kb
ENSG00000196862	2	108418388	108532297	68	13	120000	0.010406	0.49585	RGPD4	76353	25kb
ENSG00000115665	2	108577979	108655450	189	39	120000	0.086647	0.46548	SLC5A7	76353	25kb
ENSG00000196228	2	108838651	108906807	218	26	120000	2.0753	0.01898	SULT1C3	76353	25kb
ENSG00000198203	2	108880095	108951371	227	42	120000	2.4152	0.0078637	SULT1C2	76353	25kb
ENSG00000198075	2	108969367	109029513	173	18	120000	-0.69608	0.75681	SULT1C4	76353	25kb
ENSG00000135968	2	109040017	109150871	343	18	120000	-0.033905	0.51352	GCC2	76353	25kb
ENSG00000169756	2	109125857	109328702	551	23	120000	-0.51831	0.69788	LIMS1	76353	25kb
ENSG00000153201	2	109310937	109427267	163	15	120000	-0.58928	0.72216	RANBP2	76353	25kb
ENSG00000163006	2	109378213	109526933	300	16	120000	-0.18389	0.57295	CCDC138	76353	25kb
ENSG00000135960	2	109485927	109630828	382	39	120000	-1.1373	0.8723	EDAR	76353	25kb
ENSG00000172985	2	109720804	110287207	1268	82	120000	-2.6731	0.99624	SH3RF3	76353	25kb
ENSG00000186522	2	110275559	110396783	325	21	120000	-0.47596	0.68295	SEPT10	76353	25kb
ENSG00000198142	2	110346911	110401563	165	18	120000	-0.3645	0.64226	SOWAHC	76353	25kb
ENSG00000144063	2	110816447	110899143	82	15	120000	0.15174	0.4397	MALL	76353	25kb
ENSG00000144061	2	110854888	110987643	222	17	120000	0.13939	0.44457	NPHP1	76353	25kb
ENSG00000169679	2	111370275	111460691	77	10	120000	0.36474	0.35765	BUB1	76353	25kb
ENSG00000153093	2	111465150	111900799	1165	71	120000	-0.4175	0.66184	ACOXL	76353	25kb
ENSG00000153094	2	111851955	111951024	216	22	120000	-0.70195	0.75865	BCL2L11	76353	25kb
ENSG00000153107	2	112498848	112667267	293	31	120000	2.3501	0.0093833	ANAPC1	76353	25kb
ENSG00000153208	2	112631056	112812138	611	29	120000	0.4848	0.31391	MERTK	76353	25kb
ENSG00000153214	2	112787800	112901895	320	31	120000	-0.92351	0.82213	TMEM87B	76353	25kb
ENSG00000144152	2	112870962	112970791	235	37	120000	-0.26975	0.60632	FBLN7	76353	25kb
ENSG00000144161	2	112944102	113037713	143	17	120000	-0.14085	0.55601	ZC3H8	76353	25kb
ENSG00000188177	2	113008171	113122640	205	19	120000	-0.14752	0.55864	ZC3H6	76353	25kb
ENSG00000169629	2	113102669	113217065	51	15	120000	0.018853	0.49248	RGPD8	76353	25kb
ENSG00000114999	2	113214731	113324316	233	28	120000	-0.18583	0.57371	TTL	76353	25kb
ENSG00000125630	2	113274492	113359635	217	24	120000	-0.39937	0.65519	POLR1B	76353	25kb
ENSG00000125611	2	113316817	113371852	125	20	120000	-0.29043	0.61426	CHCHD5	76353	25kb
ENSG00000144136	2	113378434	113446404	178	18	120000	0.58886	0.27798	SLC20A1	76353	25kb
ENSG00000144130	2	113454063	113525456	175	26	120000	1.8871	0.029573	NT5DC4	76353	25kb
ENSG00000169607	2	113468930	113547254	180	23	120000	0.86614	0.19321	CKAP2L	76353	25kb
ENSG00000115008	2	113506492	113567167	130	13	120000	-0.72217	0.76491	IL1A	76353	25kb
ENSG00000125538	2	113562328	113619480	104	19	120000	0.46414	0.32128	IL1B	76353	25kb
ENSG00000125571	2	113645548	113701459	211	13	120000	-0.27547	0.60852	IL37	76353	25kb
ENSG00000136688	2	113705780	113768242	134	13	120000	-0.54125	0.70583	IL36G	76353	25kb
ENSG00000136694	2	113738038	113790621	117	14	120000	-0.68426	0.75309	IL36A	76353	25kb
ENSG00000136696	2	113754668	113835444	293	20	120000	0.22556	0.41077	IL36B	76353	25kb
ENSG00000136695	2	113791215	113847959	327	25	120000	0.92105	0.17851	IL36RN	76353	25kb
ENSG00000136697	2	113800547	113858427	347	26	120000	1.1977	0.11552	IL1F10	76353	25kb
ENSG00000136689	2	113839791	113916593	379	25	120000	1.8612	0.031355	IL1RN	76353	25kb
ENSG00000125637	2	113889902	113991973	332	45	120000	1.5684	0.058398	PSD4	76353	25kb
ENSG00000125618	2	113948574	114061527	378	31	120000	0.66409	0.25332	PAX8	76353	25kb
ENSG00000136682	2	114170268	114278766	24	5	120000	-0.30535	0.61995	CBWD2	76353	25kb
ENSG00000238091	2	114179992	114230429	8	2	120000	-0.29613	0.61644	AC016745.1	76353	25kb
ENSG00000184492	2	114231661	114283728	10	5	120000	0.16603	0.43407	FOXD4L1	76353	25kb
ENSG00000144134	2	114359806	114425973	61	13	120000	0.016051	0.4936	RABL2A	76353	25kb
ENSG00000115084	2	114437588	114539400	362	24	120000	0.67024	0.25135	SLC35F5	76353	25kb
ENSG00000115091	2	114622537	114745173	387	23	120000	-1.2394	0.8924	ACTR3	76353	25kb
ENSG00000175497	2	115174876	116628328	3513	140	120000	-0.32411	0.62707	DPP10	76353	25kb
ENSG00000088205	2	118547226	118614955	183	24	120000	-1.7486	0.95982	DDX18	76353	25kb
ENSG00000125633	2	118648054	118796709	529	27	120000	-0.72205	0.76487	CCDC93	76353	25kb
ENSG00000125629	2	118821028	118893573	230	44	120000	0.64574	0.25922	INSIG2	76353	25kb
ENSG00000163064	2	119574747	119630254	148	24	120000	2.0125	0.022082	EN1	76353	25kb
ENSG00000019169	2	119674742	119777236	317	33	120000	-0.19756	0.57831	MARCO	76353	25kb
ENSG00000144119	2	119888819	119941465	193	32	120000	-0.33324	0.63052	C1QL2	76353	25kb
ENSG00000115107	2	119956384	120048228	332	40	120000	1.1594	0.12314	STEAP3	76353	25kb
ENSG00000186132	2	120034801	120149404	411	30	120000	-0.77953	0.78217	C2orf76	76353	25kb
ENSG00000155368	2	120099497	120155126	218	22	120000	-0.70001	0.75804	DBI	76353	25kb
ENSG00000171227	2	120162477	120221096	160	23	120000	-0.39082	0.65203	TMEM37	76353	25kb
ENSG00000080293	2	120172419	120307070	398	36	120000	-1.1493	0.87478	SCTR	76353	25kb

ENSG00000163075	2	120277008	120444827	356	23	120000	-0.049572	0.51977	PCDP1	76353	25kb
ENSG00000144120	2	120411743	120469083	225	17	120000	-1.276	0.89902	TMEM177	76353	25kb
ENSG00000088179	2	120492207	120766394	498	26	120000	-0.2407	0.59511	PTPN4	76353	25kb
ENSG00000115109	2	120745581	120961695	388	19	120000	-0.60637	0.72787	EPB41L5	76353	25kb
ENSG00000226479	2	120953854	121005984	101	12	120000	-0.30222	0.61876	TMEM185B	76353	25kb
ENSG00000144118	2	120972640	121077289	244	17	120000	0.84302	0.19961	RALB	76353	25kb
ENSG00000163083	2	121078719	121134384	190	37	120000	-1.7262	0.95784	INHBB	76353	25kb
ENSG00000268194	2	121196911	121248697	203	35	120000	1.1869	0.11764	LINC01101	76353	25kb
ENSG00000074047	2	121468199	121775229	872	96	120000	1.0222	0.15333	GLI2	76353	25kb
ENSG00000115112	2	121949163	122067783	322	48	120000	0.25068	0.40103	TFCP2L1	76353	25kb
ENSG00000074054	2	122070352	122432163	819	20	120000	-1.0505	0.85325	CLASP1	76353	25kb
ENSG00000155438	2	122459521	122519499	123	9	120000	-0.030993	0.51236	NIFK	76353	25kb
ENSG00000211460	2	122469679	122550429	148	15	120000	0.036048	0.48562	TSN	76353	25kb
ENSG00000155052	2	124757864	125697864	2847	133	120000	-2.1932	0.98585	CNTNAP5	76353	25kb
ENSG00000136732	2	127388509	127479246	336	46	120000	0.83804	0.201	GYPC	76353	25kb
ENSG00000237524	2	127631472	127684673	151	18	120000	-0.4353	0.66833	AC114783.1	76353	25kb
ENSG00000136717	2	127780603	127889931	449	39	120000	-0.85513	0.80376	BIN1	76353	25kb
ENSG00000186684	2	127916696	128002654	287	27	120000	0.32623	0.37213	CYP27C1	76353	25kb
ENSG00000163161	2	127989866	128076752	209	24	120000	0.72454	0.23437	ERCC3	76353	25kb
ENSG00000169967	2	128031306	128171041	318	23	120000	0.18715	0.42577	MAP3K2	76353	25kb
ENSG00000115718	2	128151003	128211822	160	20	120000	0.72485	0.23427	PROC	76353	25kb
ENSG00000163166	2	128168783	128309462	356	26	120000	0.17279	0.43141	IWS1	76353	25kb
ENSG00000169994	2	128268378	128420304	437	43	120000	-0.16861	0.56695	MYO7B	76353	25kb
ENSG00000072163	2	128370956	128464360	340	41	120000	0.5338	0.29674	LIMS2	76353	25kb
ENSG00000144230	2	128378439	128435213	211	41	120000	0.59771	0.27502	GPR17	76353	25kb
ENSG00000136709	2	128433596	128593761	444	29	120000	1.2749	0.10117	WDR33	76353	25kb
ENSG00000173349	2	128433597	128486385	145	21	120000	1.1113	0.13321	SFT2D3	76353	25kb
ENSG00000144231	2	128578840	128640731	175	21	120000	1.6363	0.050893	POLR2D	76353	25kb
ENSG00000144233	2	128594204	128668496	174	26	120000	2.4075	0.0080306	AMMECR1L	76353	25kb
ENSG00000136715	2	128673791	128810694	280	22	120000	-0.19222	0.57622	SAP130	76353	25kb
ENSG00000136731	2	128823774	128978251	493	22	120000	1.8453	0.032494	UGGT1	76353	25kb
ENSG00000136720	2	128969290	129101151	444	52	120000	-0.02128	0.50849	HS6ST1	76353	25kb
ENSG00000222014	2	130712235	130765311	105	10	120000	0.10755	0.45718	RAB6C	76353	25kb
ENSG00000196604	2	130806108	130911795	63	6	120000	-0.77421	0.7806	POTEF	76353	25kb
ENSG00000152076	2	130871860	130927707	72	6	120000	-0.59654	0.72459	CCDC74B	76353	25kb
ENSG00000136699	2	130883981	130965323	133	7	120000	-0.47203	0.68155	SMPD4	76353	25kb
ENSG00000152082	2	130914310	130973302	85	6	120000	-0.26152	0.60316	MZT2B	76353	25kb
ENSG00000152086	2	130924318	130981034	80	5	120000	-0.26217	0.6034	TUBA3E	76353	25kb
ENSG00000136710	2	131070814	131124922	89	12	120000	-0.31042	0.62188	CCDC115	76353	25kb
ENSG00000136718	2	131074798	131130383	82	12	120000	-0.48138	0.68488	IMP4	76353	25kb
ENSG00000072135	2	131088580	131157982	68	13	120000	0.46917	0.31947	PTPN18	76353	25kb
ENSG00000196834	2	131192028	131292239	4	2	120000	-1.2759	0.899	POTEI	76353	25kb
ENSG00000222038	2	131344054	131440664	1	1	120000	1.0473	0.14748	POTEJ	76353	25kb
ENSG00000173302	2	131461643	131512909	70	8	120000	-1.3045	0.90396	GPR148	76353	25kb
ENSG00000178171	2	131488008	131550707	110	14	120000	-1.0105	0.84386	AMER3	76353	25kb
ENSG00000136002	2	131569489	131829836	655	59	120000	-0.79867	0.78776	ARHGEF4	76353	25kb
ENSG00000152102	2	131780449	131876033	153	15	120000	-0.46691	0.67972	FAM168B	76353	25kb
ENSG00000115762	2	131837420	132136282	307	16	120000	-0.33382	0.63074	PLEKHB2	76353	25kb
ENSG00000188219	2	131950882	132047965	40	8	120000	0.15393	0.43883	POTEE	76353	25kb
ENSG00000178206	2	132019330	132075127	42	5	120000	-0.29832	0.61727	CYP4F31P	76353	25kb
ENSG00000173272	2	132197473	132275316	329	13	120000	2.1989	0.013941	MZT2A	76353	25kb
ENSG00000075886	2	132208666	132265507	217	12	120000	2.1596	0.015402	TUBA3D	76353	25kb
ENSG00000163040	2	132260248	132316239	202	13	120000	2.797	0.0025788	CCDC74A	76353	25kb
ENSG00000197927	2	132454948	132549973	234	19	120000	-0.24004	0.59485	C2orf27A	76353	25kb
ENSG00000186825	2	132527534	132584234	143	14	120000	-0.70716	0.76027	C2orf27B	76353	25kb
ENSG00000163046	2	132880164	133040542	180	20	120000	-0.64557	0.74072	ANKRD30BL	76353	25kb
ENSG00000183840	2	133149147	133429132	798	67	120000	0.5158	0.303	GPR39	76353	25kb
ENSG00000150551	2	133377426	133454152	129	12	120000	1.717	0.042992	LYPD1	76353	25kb
ENSG00000176771	2	133404374	134351034	2384	176	120000	-0.093149	0.53711	NCKAP5	76353	25kb
ENSG00000269686	2	133849577	133901625	150	19	120000	0.011218	0.49552	AC011755.1	76353	25kb
ENSG00000152127	2	134852554	135237192	1150	101	120000	-1.5404	0.93827	MGAT5	76353	25kb
ENSG00000152128	2	135188330	135501570	844	52	120000	-1.0747	0.85874	TMEM163	76353	25kb
ENSG00000153086	2	135571117	135684604	201	15	120000	0.44047	0.3298	ACMSD	76353	25kb
ENSG00000082258	2	135650805	135741912	150	12	120000	-0.29073	0.61437	CCNT2	76353	25kb
ENSG00000176601	2	135697061	135830038	273	20	120000	-0.37226	0.64515	MAP3K19	76353	25kb
ENSG00000115839	2	135784835	135958964	271	13	120000	-0.20427	0.58093	RAB3GAP1	76353	25kb
ENSG00000121988	2	135869486	136313806	631	15	120000	-1.2269	0.89006	ZRANB3	76353	25kb
ENSG00000048991	2	136264025	136507840	333	20	120000	-1.9528	0.97458	R3HDM1	76353	25kb
ENSG00000144224	2	136474189	136567625	182	15	120000	-0.87012	0.80788	UBXN4	76353	25kb
ENSG00000115850	2	136520410	136619750	194	16	120000	-0.61133	0.72951	LCT	76353	25kb
ENSG00000076003	2	136572196	136658996	147	13	120000	-0.32152	0.62609	MCM6	76353	25kb
ENSG00000115866	2	136639247	136768670	133	8	120000	-0.15049	0.55981	DARS	76353	25kb
ENSG00000121966	2	136846919	136900735	79	11	120000	-0.76034	0.77648	CXCR4	76353	25kb
ENSG00000144229	2	137498115	138460287	3292	112	120000	-0.26666	0.60514	THSD7B	76353	25kb
ENSG00000150540	2	138696590	138798930	222	23	120000	-0.38753	0.65082	HNMT	76353	25kb
ENSG00000144228	2	139234371	139356117	176	20	120000	-1.0636	0.85624	SPOPL	76353	25kb
ENSG00000144227	2	139403342	139562918	471	58	120000	-0.11478	0.54569	NXPH2	76353	25kb
ENSG00000168702	2	140963992	142914270	7786	259	120000	1.099	0.13589	LRP1B	76353	25kb
ENSG00000115919	2	143610067	143824890	544	35	120000	0.29541	0.38384	KYNU	76353	25kb
ENSG00000075884	2	143823931	144550921	1599	107	120000	-0.83184	0.79725	ARHGAP15	76353	25kb
ENSG00000121964	2	144670635	145115135	602	40	120000	-0.94076	0.82659	GTDC1	76353	25kb
ENSG00000169554	2	145116648	145307147	277	51	120000	0.23441	0.40733	ZEB2	76353	25kb
ENSG00000121989	2	148577086	148713393	169	16	120000	-0.97095	0.83421	ACVR2A	76353	25kb
ENSG00000115947	2	148662968	148804147	221	16	120000	-0.7895	0.78509	ORC4	76353	25kb
ENSG00000204406	2	148753580	149300805	1155	61	120000	0.26973	0.39368	MBD5	76353	25kb
ENSG00000135999	2	149377009	149570130	441	19	120000	-1.3385	0.90963	EPC2	76353	25kb
ENSG00000168280	2	149607819	149908273	304	18	120000	-0.61479	0.73065	KIF5C	76353	25kb
ENSG00000150556	2	149869621	150096776	663	47	120000	-0.52687	0.70086	LYPD6B	76353	25kb
ENSG00000187123	2	150161499	150355662	423	43	120000	0.22019	0.41286	LYPD6	76353	25kb
ENSG00000168288	2	150401148	150469330	160	16	120000	-0.10957	0.54362	MMADHC	76353	25kb
ENSG00000115963	2	151299709	151420525	268	40	120000	-2.103	0.98227	RND3	76353	25kb
ENSG00000222031	2	151832734	151930288	374	32	120000	-0.6749	0.75013	AC023469.1	76353	25kb
ENSG00000184898	2	152079454	152143393	161	16	120000	-1.2009	0.88511	RBM43	76353	25kb
ENSG00000123609	2	152101979	152171571	220	13	120000	-0.70666	0.76011	NMI	76353	25kb
ENSG00000123610	2	152189106	152261560	209	33	120000	0.64937	0.25805	TNFAIP6	76353	25kb
ENSG00000080345	2	152241397	152389527	324	24	120000	0.019138	0.49237	RIF1	76353	25kb

ENSG00000183091	2	152316850	152616001	503	34	120000	-0.14636	0.55818	NEB	76353	25kb
ENSG00000162980	2	152620498	152710006	195	23	120000	0.24124	0.40468	ARL5A	76353	25kb
ENSG00000182389	2	152664290	152980593	758	38	120000	0.017062	0.49319	CACNB4	76353	25kb
ENSG00000115145	2	152948315	153057506	233	21	120000	-0.47833	0.68379	STAM2	76353	25kb
ENSG00000157827	2	153166751	153531348	1090	64	120000	-0.12535	0.54988	FMNL2	76353	25kb
ENSG00000196504	2	153483107	153599511	291	20	120000	-1.055	0.8543	PRPF40A	76353	25kb
ENSG00000177917	2	153549407	153642767	230	30	120000	-1.2343	0.89146	ARL6IP6	76353	25kb
ENSG00000177519	2	154308852	154360322	102	23	120000	-0.28235	0.61116	RPRM	76353	25kb
ENSG00000144278	2	154703426	155335361	2012	65	120000	-1.1507	0.87508	GALNT13	76353	25kb
ENSG00000162989	2	155529811	155739863	611	63	120000	-0.43714	0.669	KCNJ3	76353	25kb
ENSG00000153234	2	157155944	157223860	87	18	120000	1.4872	0.068487	NR4A2	76353	25kb
ENSG00000115159	2	157266802	157495247	484	36	120000	-0.70819	0.76059	GPD2	76353	25kb
ENSG00000268857	2	157327597	157377655	92	16	120000	-0.64827	0.7416	AC011308.1	76353	25kb
ENSG00000136542	2	158089110	158195723	220	23	120000	-0.10728	0.54272	GALNT5	76353	25kb
ENSG00000136541	2	158150137	158209225	97	13	120000	0.40299	0.34348	ERMN	76353	25kb
ENSG00000115165	2	158246131	158370473	219	32	120000	-1.2776	0.89931	CYTIP	76353	25kb
ENSG00000123612	2	158358279	158510517	197	35	120000	-1.6371	0.94919	ACVR1C	76353	25kb
ENSG00000115170	2	158567958	158757374	311	50	120000	-1.4375	0.92471	ACVR1	76353	25kb
ENSG00000007001	2	158708214	159017666	925	75	120000	-0.31625	0.6241	UPP2	76353	25kb
ENSG00000153237	2	159002593	159338265	1281	59	120000	-1.9473	0.97425	CCDC148	76353	25kb
ENSG00000144283	2	159288476	159564391	867	28	120000	-1.0783	0.85955	PKP4	76353	25kb
ENSG00000163331	2	159626829	159744293	440	38	120000	-0.20857	0.58261	DAPL1	76353	25kb
ENSG00000115183	2	159800146	160114170	964	55	120000	-0.77419	0.78059	TANC1	76353	25kb
ENSG00000196151	2	160067304	160168310	447	26	120000	-1.9002	0.9713	WDSUB1	76353	25kb
ENSG00000123636	2	160150490	160498203	1077	60	120000	-1.0469	0.85243	BAZ2B	76353	25kb
ENSG00000136536	2	160544000	160652538	375	20	120000	-0.10391	0.54138	MARCH7	76353	25kb
ENSG00000241399	2	160600364	160679753	274	20	120000	0.37762	0.35286	CD302	76353	25kb
ENSG00000248672	2	160603362	160786221	629	38	120000	0.018406	0.49266	LY75-CD302	76353	25kb
ENSG00000054219	2	160603362	160786260	629	38	120000	0.018406	0.49266	LY75	76353	25kb
ENSG00000153246	2	160763519	160944121	604	37	120000	-0.9587	0.83114	PLA2R1	76353	25kb
ENSG00000115221	2	160931177	161153399	627	46	120000	-1.7976	0.96388	ITGB6	76353	25kb
ENSG00000153250	2	161103662	161375305	632	39	120000	0.78409	0.21649	RBMS1	76353	25kb
ENSG00000136560	2	161968419	162117732	237	22	120000	-1.4706	0.9293	TANK	76353	25kb
ENSG00000115233	2	162139549	162293228	230	25	120000	-0.92608	0.8228	PSMD14	76353	25kb
ENSG00000136535	2	162247605	162307381	100	17	120000	-1.2029	0.88548	TBR1	76353	25kb
ENSG00000144290	2	162255843	162866792	1215	32	120000	-1.725	0.95773	SLC4A10	76353	25kb
ENSG00000197635	2	162823751	162956052	296	28	120000	-1.4099	0.92071	DPP4	76353	25kb
ENSG00000115263	2	162974392	163033914	127	16	120000	-0.10024	0.53992	GCG	76353	25kb
ENSG00000078098	2	163002194	163126661	210	11	120000	-0.64429	0.74031	FAP	76353	25kb
ENSG00000115267	2	163098589	163200213	200	7	120000	-0.82939	0.79656	IFIH1	76353	25kb
ENSG00000115271	2	163150350	163253105	110	12	120000	-0.63071	0.73588	GCA	76353	25kb
ENSG00000184611	2	163202917	163720240	1038	65	120000	0.92469	0.17756	KCNH7	76353	25kb
ENSG00000182263	2	164424906	164617522	446	70	120000	0.29492	0.38403	FIGN	76353	25kb
ENSG00000115290	2	165324322	165503358	308	35	120000	-0.15084	0.55995	GRB14	76353	25kb
ENSG00000082438	2	165485134	165725189	649	37	120000	1.3941	0.081649	COBLL1	76353	25kb
ENSG00000169507	2	165727696	165837035	301	26	120000	-1.3597	0.91303	SLC38A11	76353	25kb
ENSG00000153253	2	165919032	166085577	313	25	120000	-1.2229	0.88931	SCN3A	76353	25kb
ENSG00000136531	2	166070912	166273818	509	37	120000	-0.74575	0.77209	SCN2A	76353	25kb
ENSG00000178662	2	166301157	166570917	525	36	120000	-0.17935	0.57117	CSRNP3	76353	25kb
ENSG00000115339	2	166579101	166676192	169	28	120000	0.55766	0.28854	GALNT3	76353	25kb
ENSG00000123607	2	166688985	166835353	299	25	120000	0.61258	0.27008	TTC21B	76353	25kb
ENSG00000144285	2	166820670	167009523	593	30	120000	-0.28575	0.61247	SCN1A	76353	25kb
ENSG00000169432	2	167026695	167257503	691	52	120000	1.2191	0.1114	SCN9A	76353	25kb
ENSG00000136546	2	167235083	167375757	376	28	120000	0.73406	0.23146	SCN7A	76353	25kb
ENSG00000163092	2	167719997	168141263	1337	40	120000	1.7557	0.039572	XIRP2	76353	25kb
ENSG00000172318	2	168650182	168755551	331	29	120000	-0.048161	0.51921	B3GALT1	76353	25kb
ENSG00000198648	2	168785530	169129651	1046	52	120000	-0.60396	0.72706	STK39	76353	25kb
ENSG00000172292	2	169287372	169656644	951	90	120000	1.4939	0.067597	CERS6	76353	25kb
ENSG00000163072	2	169618049	169747024	307	33	120000	-0.029371	0.51172	NOSTRIN	76353	25kb
ENSG00000152253	2	169665642	169794881	335	34	120000	-0.4145	0.66075	SPC25	76353	25kb
ENSG00000152254	2	169732750	169791505	158	25	120000	-1.1814	0.88127	G6PC2	76353	25kb
ENSG00000073734	2	169754448	169912832	471	43	120000	0.29292	0.38479	ABCB11	76353	25kb
ENSG00000073737	2	169896299	169977677	128	27	120000	1.6328	0.051258	DHRS9	76353	25kb
ENSG00000081479	2	169958619	170244195	864	88	120000	1.0888	0.13812	LRP2	76353	25kb
ENSG00000163093	2	170310688	170407432	256	27	120000	-0.33756	0.63215	BBS5	76353	25kb
ENSG00000251569	2	170310990	170407432	255	27	120000	-0.32855	0.62875	RP11-724O16.1	76353	25kb
ENSG00000239474	2	170341212	170407772	164	28	120000	-0.2312	0.59142	KLHL41	76353	25kb
ENSG00000138399	2	170361259	170455385	266	26	120000	0.16154	0.43583	FASTKD1	76353	25kb
ENSG00000138398	2	170415850	170522916	355	20	120000	-0.082081	0.53271	PPIG	76353	25kb
ENSG00000154479	2	170476935	170575943	340	16	120000	-0.064992	0.52591	CCDC173	76353	25kb
ENSG00000144362	2	170525975	170583218	220	16	120000	-0.079649	0.53174	PHOSPHO2	76353	25kb
ENSG00000213160	2	170525998	170658499	572	31	120000	-0.23675	0.59357	KLHL23	76353	25kb
ENSG00000138385	2	170623443	170693574	269	27	120000	-0.24644	0.59733	SSB	76353	25kb
ENSG00000138382	2	170641591	170706441	233	32	120000	-0.35614	0.63913	METTL5	76353	25kb
ENSG00000144357	2	170658968	170965641	1082	28	120000	-0.34482	0.63488	UBR3	76353	25kb
ENSG00000071909	2	171009655	171536681	1859	182	120000	-1.4983	0.93297	MYO3B	76353	25kb
ENSG00000222033	2	171543961	171596077	181	22	120000	0.088554	0.46472	LINC01124	76353	25kb
ENSG00000204335	2	171546861	171599588	191	19	120000	0.13549	0.44611	SP5	76353	25kb
ENSG00000204334	2	171615296	171680481	203	20	120000	-0.065403	0.52607	ERICH2	76353	25kb
ENSG00000128683	2	171644723	171742661	282	27	120000	-0.26977	0.60633	GAD1	76353	25kb
ENSG00000115806	2	171759974	171848639	196	18	120000	2.0358	0.020886	GORASP2	76353	25kb
ENSG00000198586	2	171822333	172112824	708	30	120000	2.0256	0.021401	TLK1	76353	25kb
ENSG00000123600	2	172148919	172316312	351	26	120000	-0.006036	0.50241	METTL8	76353	25kb
ENSG00000115827	2	172265727	172366562	261	16	120000	0.028354	0.48869	DCAF17	76353	25kb
ENSG00000071967	2	172353757	172439643	290	26	120000	0.60884	0.27131	CYBRD1	76353	25kb
ENSG00000077380	2	172518919	172629930	236	25	120000	-0.81307	0.79191	DYNC1I2	76353	25kb
ENSG00000268821	2	172535844	172592314	120	17	120000	-0.77147	0.77979	AC068039.1	76353	25kb
ENSG00000115840	2	172615880	172889766	633	31	120000	-0.036475	0.51455	SLC25A12	76353	25kb
ENSG00000128708	2	172753958	172873599	293	24	120000	0.30656	0.37959	HAT1	76353	25kb
ENSG00000172878	2	172839490	172972158	331	37	120000	0.44041	0.32982	METAP1D	76353	25kb
ENSG00000144355	2	172924468	172979405	121	32	120000	0.032877	0.48689	DLX1	76353	25kb
ENSG00000115844	2	172939167	172992628	150	37	120000	0.2182	0.41364	DLX2	76353	25kb
ENSG00000091409	2	173267082	173396181	527	33	120000	-0.48853	0.68741	ITGA6	76353	25kb
ENSG00000152256	2	173395101	173514823	411	26	120000	-0.61799	0.73171	PDK1	76353	25kb
ENSG00000091428	2	173575002	173942621	1066	83	120000	0.97426	0.16496	RAPGEF4	76353	25kb
ENSG00000091436	2	173915163	174157738	865	52	120000	0.30826	0.37894	MLTK	76353	25kb

ENSG00000144354	2	174194548	174258725	213	34	120000	0.0069329	0.49723	CDCA7	76353	25kb
ENSG00000172845	2	174746187	174855430	332	25	120000	-1.562	0.94085	SP3	76353	25kb
ENSG00000138430	2	174912175	175138426	657	29	120000	-0.58601	0.72106	OLA1	76353	25kb
ENSG00000217236	2	175174674	175228220	121	23	120000	-0.5349	0.70364	SP9	76353	25kb
ENSG00000268241	2	175175601	175227151	117	23	120000	-0.50744	0.69408	AC018470.1	76353	25kb
ENSG00000138433	2	175187750	175285443	226	23	120000	0.052627	0.47901	CIR1	76353	25kb
ENSG00000144306	2	175235458	175319303	235	18	120000	0.79507	0.21329	SCRN3	76353	25kb
ENSG00000163328	2	175271966	175376822	285	28	120000	0.45199	0.32564	GPR155	76353	25kb
ENSG00000115935	2	175399300	175572644	359	43	120000	0.033377	0.48669	WIPF1	76353	25kb
ENSG00000138435	2	175587320	175654200	192	25	120000	0.058714	0.47659	CHRNA1	76353	25kb
ENSG00000128656	2	175639091	175895097	381	24	120000	1.3279	0.092106	CHN1	76353	25kb
ENSG00000115966	2	175911978	176058110	339	20	120000	-1.0043	0.84237	ATF2	76353	25kb
ENSG00000154518	2	176015986	176074335	152	16	120000	-0.46138	0.67774	ATP5G3	76353	25kb
ENSG00000144320	2	176763620	176892567	322	24	120000	-0.50955	0.69482	KIAA1715	76353	25kb
ENSG00000174279	2	176917200	176973641	140	28	120000	0.097918	0.461	EVX2	76353	25kb
ENSG00000128714	2	176932619	176985666	114	25	120000	0.06139	0.47552	HOXD13	76353	25kb
ENSG00000170178	2	176939458	176991408	113	26	120000	0.015166	0.49395	HOXD12	76353	25kb
ENSG00000128713	2	176943944	176999722	120	28	120000	-0.081908	0.53264	HOXD11	76353	25kb
ENSG00000128710	2	176948518	177009670	145	33	120000	-0.17779	0.57056	HOXD10	76353	25kb
ENSG00000128709	2	176962088	177014853	119	27	120000	-0.35013	0.63688	HOXD9	76353	25kb
ENSG00000175879	2	176969422	177022423	112	25	120000	-0.57508	0.71738	HOXD8	76353	25kb
ENSG00000128652	2	176976340	177062830	173	24	120000	-0.96703	0.83324	HOXD3	76353	25kb
ENSG00000170166	2	176990950	177042954	116	17	120000	-0.95032	0.82902	HOXD4	76353	25kb
ENSG00000128645	2	177028307	177080688	123	18	120000	-0.79304	0.78612	HOXD1	76353	25kb
ENSG00000128654	2	177109123	177227753	222	25	120000	2.0716	0.019153	MTX2	76353	25kb
ENSG00000170144	2	178052291	178113686	68	11	120000	-0.45937	0.67702	HNRNPA3	76353	25kb
ENSG00000116044	2	178067323	178282425	517	31	120000	0.86392	0.19382	NFE2L2	76353	25kb
ENSG00000018510	2	178232372	178433564	445	21	120000	0.019936	0.49205	AGPS	76353	25kb
ENSG00000196659	2	178388726	178442742	113	11	120000	-0.26606	0.6049	TTC30B	76353	25kb
ENSG00000197557	2	178452720	178508694	248	25	120000	-1.2774	0.89926	TTC30A	76353	25kb
ENSG00000128655	2	178462980	178998066	1881	53	120000	0.71847	0.23623	PDE11A	76353	25kb
ENSG00000155636	2	178952151	179028738	197	36	120000	0.2141	0.41523	RBM45	76353	25kb
ENSG00000079156	2	179034208	179289160	725	55	120000	0.92429	0.17767	OSBPL6	76353	25kb
ENSG00000180228	2	179271141	179341239	105	16	120000	1.529	0.063134	PRKRA	76353	25kb
ENSG00000204311	2	179291163	179351117	81	15	120000	1.3428	0.08967	DFNB59	76353	25kb
ENSG00000079150	2	179303391	179368327	103	17	120000	1.5006	0.06673	FKBP7	76353	25kb
ENSG00000116095	2	179320195	179406189	158	26	120000	1.3517	0.088244	PLEKHA3	76353	25kb
ENSG00000155657	2	179365716	179720529	632	39	120000	0.57342	0.28318	TTN	76353	25kb
ENSG00000163492	2	179669484	179939813	812	57	120000	-1.477	0.93017	CCDC141	76353	25kb
ENSG00000187231	2	179941419	180154517	528	28	120000	-0.63971	0.73882	SESTD1	76353	25kb
ENSG00000144331	2	180281709	180751232	1687	121	120000	0.28153	0.38915	ZNF385B	76353	25kb
ENSG00000163510	2	180784603	180896840	289	21	120000	-1.0998	0.86429	CWC22	76353	25kb
ENSG00000170035	2	181806975	181966312	527	15	120000	0.39831	0.3452	UBE2E3	76353	25kb
ENSG00000115232	2	182296929	182425914	356	38	120000	-0.62138	0.73283	ITGA4	76353	25kb
ENSG00000188452	2	182376403	182570392	492	34	120000	0.068035	0.47288	CERKL	76353	25kb
ENSG00000162992	2	182512815	182570603	122	14	120000	0.80311	0.21096	NEUROD1	76353	25kb
ENSG00000138434	2	182731560	182820465	126	11	120000	-0.64568	0.74076	SSFA2	76353	25kb
ENSG00000150722	2	182793968	183021125	482	53	120000	0.84042	0.20034	PPP1R1C	76353	25kb
ENSG00000115252	2	182979763	183412919	1354	59	120000	0.44638	0.32766	PDE1A	76353	25kb
ENSG00000077232	2	183555999	183684191	208	16	120000	-0.78572	0.78398	DNAJC10	76353	25kb
ENSG00000162998	2	183673002	183756890	214	31	120000	1.8358	0.033191	FRZB	76353	25kb
ENSG00000061676	2	183748843	183928586	462	31	120000	-0.16041	0.56372	NCKAP1	76353	25kb
ENSG00000162999	2	183918287	183989733	220	27	120000	-0.32707	0.62819	DUSP19	76353	25kb
ENSG00000163002	2	183957241	184051408	514	23	120000	-0.42533	0.6647	NUP35	76353	25kb
ENSG00000170396	2	185438093	185829219	833	31	120000	1.5907	0.055843	ZNF804A	76353	25kb
ENSG00000188738	2	186578355	186723017	330	16	120000	-0.5401	0.70543	FSIP2	76353	25kb
ENSG00000065548	2	187325883	187399090	142	16	120000	1.1235	0.13061	ZC3H15	76353	25kb
ENSG00000213953	2	187336840	187390393	98	14	120000	0.99368	0.16019	AC018867.1	76353	25kb
ENSG00000268846	2	187342219	187392356	97	13	120000	1.0842	0.13914	AC018867.2	76353	25kb
ENSG00000138448	2	187429792	187570628	242	29	120000	-0.79791	0.78754	ITGAV	76353	25kb
ENSG00000144369	2	187533698	187655685	263	23	120000	-0.9086	0.81822	FAM171B	76353	25kb
ENSG00000163012	2	187667562	187738935	227	16	120000	0.59491	0.27595	ZSWIM2	76353	25kb
ENSG00000064989	2	188182856	188338187	369	20	120000	-0.47284	0.68184	CALCRL	76353	25kb
ENSG00000003436	2	188303957	188455487	332	32	120000	-0.10428	0.54153	TFPI	76353	25kb
ENSG00000144366	2	189131396	189485653	754	27	120000	-0.47835	0.6838	GULP1	76353	25kb
ENSG00000174325	2	189573882	189679831	311	14	120000	1.7469	0.040329	DIRC1	76353	25kb
ENSG00000168542	2	189814046	189902472	219	37	120000	0.39125	0.34781	COL3A1	76353	25kb
ENSG00000204262	2	189871622	190069605	541	22	120000	0.69752	0.24274	COL5A2	76353	25kb
ENSG00000115368	2	190281159	190365291	302	12	120000	0.82538	0.20458	WDR75	76353	25kb
ENSG00000138449	2	190400305	190473484	156	22	120000	0.94811	0.17154	SLC40A1	76353	25kb
ENSG00000138381	2	190501111	190560557	137	20	120000	0.12198	0.45146	ASNSD1	76353	25kb
ENSG00000151687	2	190514016	190650919	275	21	120000	-0.27153	0.60701	ANKAR	76353	25kb
ENSG00000128694	2	190586386	190652953	122	14	120000	-0.27932	0.61	OSGEPL1	76353	25kb
ENSG00000128699	2	190610049	190674097	133	15	120000	-0.45282	0.67466	ORMDL1	76353	25kb
ENSG00000231145	2	190617909	190668157	107	13	120000	-0.39446	0.65338	AC013468.1	76353	25kb
ENSG00000064933	2	190624107	190767355	300	21	120000	-0.31515	0.62367	PMS1	76353	25kb
ENSG00000187699	2	190719335	191093210	875	69	120000	0.56927	0.28459	C2orf88	76353	25kb
ENSG00000138379	2	190895423	190952455	121	18	120000	1.8075	0.035346	MSTN	76353	25kb
ENSG00000198130	2	191029461	191233919	585	20	120000	0.97056	0.16588	HIBCH	76353	25kb
ENSG00000151689	2	191183196	191261391	194	21	120000	0.62577	0.26573	INPP1	76353	25kb
ENSG00000151690	2	191248081	191398931	337	22	120000	1.2599	0.10385	MFSD6	76353	25kb
ENSG00000189362	2	191344068	191424448	174	19	120000	1.7335	0.0415	TMEM194B	76353	25kb
ENSG00000138386	2	191486472	191582492	209	28	120000	1.204	0.1143	NAB1	76353	25kb
ENSG00000115419	2	191720553	191855278	230	25	120000	1.6073	0.053989	GLS	76353	25kb
ENSG00000115415	2	191804084	191910686	226	35	120000	0.62571	0.26575	STAT1	76353	25kb
ENSG00000138378	2	191869302	192041322	363	46	120000	0.47852	0.31614	STAT4	76353	25kb
ENSG00000128641	2	192084911	192315115	484	27	120000	0.046735	0.48136	MYO1B	76353	25kb
ENSG00000173559	2	192517794	192586385	140	19	120000	0.64631	0.25904	NABP1	76353	25kb
ENSG00000168497	2	192674028	192736981	143	28	120000	0.63715	0.26202	SDPR	76353	25kb
ENSG00000144339	2	192788769	193085435	609	74	120000	0.92809	0.17668	TMEFF2	76353	25kb
ENSG00000196950	2	196415701	196627426	417	42	120000	0.56072	0.2875	SLC39A10	76353	25kb
ENSG00000118997	2	196577427	196958536	751	29	120000	0.82049	0.20597	DNAH7	76353	25kb
ENSG00000081320	2	196973290	197066227	292	16	120000	0.5628	0.28679	STK17B	76353	25kb
ENSG00000138411	2	197034094	197483416	1228	77	120000	-1.1758	0.88017	HECW2	76353	25kb
ENSG00000144395	2	197479278	197653214	351	19	120000	0.79656	0.21285	CCDC150	76353	25kb
ENSG00000119041	2	197602756	197689449	107	17	120000	1.9973	0.022898	GTF3C3	76353	25kb

ENSG00000187944	2	197644726	197700000	59	11	120000	2.4081	0.008017	C2orf66	76353	25kb
ENSG00000197121	2	197672728	197817520	248	19	120000	2.1273	0.016696	PGAP1	76353	25kb
ENSG00000065413	2	197806741	198200897	1056	70	120000	0.58214	0.28024	ANKRD44	76353	25kb
ENSG00000115524	2	198229508	198324815	111	12	120000	-0.76068	0.77658	SF3B1	76353	25kb
ENSG00000115520	2	198293147	198365032	72	10	120000	-0.79412	0.78644	COQ10B	76353	25kb
ENSG00000144381	2	198326305	198406461	79	11	120000	-0.52837	0.70138	HSPD1	76353	25kb
ENSG00000115541	2	198339718	198393181	54	9	120000	-0.39563	0.65381	HSPE1	76353	25kb
ENSG00000270757	2	198340137	198440450	113	14	120000	-0.26868	0.60591	HSPE1-MOB4	76353	25kb
ENSG00000115540	2	198355295	198443423	102	14	120000	-0.23977	0.59475	MOB4	76353	25kb
ENSG00000162944	2	198407948	198565769	211	17	120000	-0.74434	0.77166	RFTN2	76353	25kb
ENSG00000222017	2	198532830	198664547	151	13	120000	-0.75011	0.77341	AC011997.1	76353	25kb
ENSG00000247626	2	198545087	198598113	49	6	120000	-0.78195	0.78288	MARS2	76353	25kb
ENSG00000152430	2	198566603	198676486	137	13	120000	-0.6883	0.75437	BOLL	76353	25kb
ENSG00000115896	2	198644426	199462305	1575	82	120000	-1.2462	0.89365	PLCL1	76353	25kb
ENSG00000119042	2	200109223	200360989	348	27	120000	-1.3124	0.9053	SATB2	76353	25kb
ENSG00000226124	2	200600267	200740896	291	37	120000	-0.91863	0.82086	FTCDNL1	76353	25kb
ENSG00000178074	2	200750979	200845658	177	21	120000	0.6582	0.2552	C2orf69	76353	25kb
ENSG00000162971	2	200769698	200845459	135	18	120000	0.69489	0.24356	TYW5	76353	25kb
ENSG00000162972	2	200795040	200898263	186	24	120000	0.52768	0.29886	C2orf47	76353	25kb
ENSG00000196141	2	201145604	201371986	524	46	120000	0.30209	0.38129	SPATS2L	76353	25kb
ENSG00000155729	2	201328675	201409507	192	38	120000	0.89284	0.18597	KCTD18	76353	25kb
ENSG00000163535	2	201349731	201473505	277	24	120000	1.0522	0.14635	SGOL2	76353	25kb
ENSG00000138356	2	201425591	201566787	396	42	120000	1.573	0.057863	AOX1	76353	25kb
ENSG00000082153	2	201650317	201713569	98	13	120000	0.851	0.19738	BZW1	76353	25kb
ENSG00000013441	2	201692732	201754422	109	24	120000	-0.14762	0.55868	CLK1	76353	25kb
ENSG00000240344	2	201710630	201779026	102	18	120000	-0.96744	0.83334	PPIL3	76353	25kb
ENSG00000196290	2	201729050	201793655	89	14	120000	-1.1109	0.8667	NIF3L1	76353	25kb
ENSG00000115942	2	201748696	201853403	126	16	120000	-0.95456	0.8301	ORC2	76353	25kb
ENSG00000155744	2	201813441	201961394	235	18	120000	-0.7005	0.75819	FAM126B	76353	25kb
ENSG00000119013	2	201911156	201975473	133	12	120000	-0.46939	0.6806	NDUFB3	76353	25kb
ENSG00000003402	2	201955827	202066410	223	23	120000	-0.41277	0.66011	CFLAR	76353	25kb
ENSG00000003400	2	202022604	202119129	215	23	120000	0.14819	0.4411	CASP10	76353	25kb
ENSG00000064012	2	202073166	202177434	255	25	120000	-0.27551	0.60854	CASP8	76353	25kb
ENSG00000155749	2	202127994	202247121	275	22	120000	0.42466	0.33554	ALS2CR12	76353	25kb
ENSG00000115993	2	202216930	202341302	236	22	120000	0.62285	0.26669	TRAK2	76353	25kb
ENSG00000082146	2	202227581	202370569	258	17	120000	0.49756	0.3094	STRADB	76353	25kb
ENSG00000155754	2	202327148	202508901	455	25	120000	-0.00043261	0.50017	ALS2CR11	76353	25kb
ENSG00000155755	2	202459907	202533293	169	26	120000	-0.97349	0.83484	TMEM237	76353	25kb
ENSG00000082126	2	202484593	202588417	271	44	120000	0.53627	0.29589	MPP4	76353	25kb
ENSG00000003393	2	202540277	202670912	379	29	120000	0.64351	0.25995	ALS2	76353	25kb
ENSG00000138395	2	202630184	202785273	358	49	120000	-0.67409	0.74987	CDK15	76353	25kb
ENSG00000155760	2	202874310	202928160	149	22	120000	0.38707	0.34935	FZD7	76353	25kb
ENSG00000182329	2	202912978	203087585	441	39	120000	-1.4389	0.92491	AC079354.1	76353	25kb
ENSG00000116030	2	203045903	203128331	178	20	120000	-0.081827	0.53261	SUMO1	76353	25kb
ENSG00000267889	2	203079292	203129435	98	15	120000	0.31661	0.37577	AC079354.2	76353	25kb
ENSG00000055044	2	203105439	203193389	151	13	120000	-0.06281	0.52504	NOP58	76353	25kb
ENSG00000204217	2	203216659	203457474	391	22	120000	-0.96641	0.83308	BMPR2	76353	25kb
ENSG00000138439	2	203474901	203659480	235	31	120000	-0.99859	0.841	FAM117B	76353	25kb
ENSG00000163596	2	203615690	203761708	281	17	120000	0.06753	0.47308	ICA1L	76353	25kb
ENSG00000138442	2	203714505	203904521	355	16	120000	0.31316	0.37708	WDR12	76353	25kb
ENSG00000138380	2	203751937	203876786	231	15	120000	0.40334	0.34335	CARF	76353	25kb
ENSG00000144426	2	203854602	204116101	382	23	120000	-0.76662	0.77835	NBEAL1	76353	25kb
ENSG00000119004	2	204078663	204188009	185	18	120000	-1.0219	0.84659	CYP20A1	76353	25kb
ENSG00000138443	2	204167942	204337446	245	30	120000	-1.342	0.9102	ABI2	76353	25kb
ENSG00000173166	2	204234068	204425133	269	24	120000	-1.1907	0.88311	RAPH1	76353	25kb
ENSG00000178562	2	204546198	204628635	192	22	120000	-0.95187	0.82942	CD28	76353	25kb
ENSG00000163599	2	204707509	204763683	146	20	120000	0.30787	0.37909	CTLA4	76353	25kb
ENSG00000163600	2	204776471	204851300	228	35	120000	-0.023012	0.50918	ICOS	76353	25kb
ENSG00000116117	2	205385516	206509886	2712	155	120000	-0.84408	0.80069	PARD3B	76353	25kb
ENSG00000118257	2	206521714	206687857	379	75	120000	-0.69001	0.75491	NRP2	76353	25kb
ENSG00000114933	2	206833445	206976027	347	27	120000	-0.62375	0.7336	INO80D	76353	25kb
ENSG00000023228	2	206954541	207049327	237	16	120000	-1.4247	0.92288	NDUFS1	76353	25kb
ENSG00000114942	2	206999309	207052652	128	12	120000	-1.334	0.9089	EEF1B2	76353	25kb
ENSG00000183671	2	207015040	207107771	242	25	120000	-0.44077	0.67031	GPR1	76353	25kb
ENSG00000204186	2	207114387	207204148	139	13	120000	0.084864	0.46618	ZDBF2	76353	25kb
ENSG00000269633	2	207195463	207245843	96	11	120000	-0.1576	0.56261	AC017081.1	76353	25kb
ENSG00000114948	2	207283263	207510851	469	47	120000	-0.34426	0.63467	ADAM23	76353	25kb
ENSG00000232125	2	207491345	207608120	427	41	120000	-0.34293	0.63417	DYTN	76353	25kb
ENSG00000138400	2	207577487	207655271	285	15	120000	-1.0532	0.85389	MDH1B	76353	25kb
ENSG00000118246	2	207605081	207682233	240	20	120000	-0.88	0.81057	FASTKD2	76353	25kb
ENSG00000144410	2	207779278	207859198	198	34	120000	-0.10023	0.53992	CPO	76353	25kb
ENSG00000118263	2	207913861	208056991	420	48	120000	-1.1963	0.88421	KLF7	76353	25kb
ENSG00000118260	2	208369461	208493155	176	16	120000	0.78837	0.21524	CREB1	76353	25kb
ENSG00000144401	2	208420355	208515652	174	16	120000	0.51357	0.30378	METTL21A	76353	25kb
ENSG00000163249	2	208551264	208651563	272	16	120000	0.70483	0.24046	CCNYL1	76353	25kb
ENSG00000163251	2	208602310	208659287	132	19	120000	0.9901	0.16106	FZD5	76353	25kb
ENSG00000178385	2	208668027	208915284	476	44	120000	-1.8768	0.96973	PLEKHM3	76353	25kb
ENSG00000118231	2	208961331	209014225	271	20	120000	-1.34	0.90988	CRYGD	76353	25kb
ENSG00000163254	2	208967861	209019554	244	26	120000	-1.2585	0.8959	CRYGC	76353	25kb
ENSG00000182187	2	208982297	209035892	250	32	120000	-0.663	0.74634	CRYGB	76353	25kb
ENSG00000168582	2	209000464	209053300	237	38	120000	-0.52389	0.69982	CRYGA	76353	25kb
ENSG00000188674	2	209005067	209079797	315	37	120000	-0.91066	0.81876	C2orf80	76353	25kb
ENSG00000138413	2	209075951	209155798	187	25	120000	-1.1247	0.86963	IDH1	76353	25kb
ENSG00000115020	2	209105991	209248475	387	17	120000	-1.2487	0.89411	PIKFYVE	76353	25kb
ENSG00000144407	2	209199438	209744227	1409	74	120000	-0.6349	0.73725	PTH2R	76353	25kb
ENSG00000078018	2	210263782	210623842	914	36	120000	-0.86757	0.80719	MAP2	76353	25kb
ENSG00000144406	2	210611717	210889024	543	33	120000	-0.74346	0.7714	UNC80	76353	25kb
ENSG00000197713	2	210842289	210911300	83	14	120000	-0.4665	0.67957	RPE	76353	25kb
ENSG00000144445	2	210861147	211061107	170	30	120000	-0.080653	0.53214	KANSL1L	76353	25kb
ENSG00000115361	2	211027663	211115215	81	18	120000	-0.31213	0.62253	ACADL	76353	25kb
ENSG00000168530	2	211129874	211204914	145	15	120000	-0.25427	0.60036	MYL1	76353	25kb
ENSG00000115365	2	211270973	211367376	216	17	120000	0.4072	0.34193	LANCL1	76353	25kb
ENSG00000021826	2	211317406	211568831	648	48	120000	-0.75123	0.77374	CPS1	76353	25kb
ENSG00000178568	2	212215446	213428565	4354	205	120000	0.90081	0.18385	ERBB4	76353	25kb
ENSG00000030419	2	213839429	214042151	571	32	120000	0.31596	0.37602	IKZF2	76353	25kb
ENSG00000144451	2	214124113	215300225	3469	99	120000	0.76655	0.22167	SPAG16	76353	25kb

ENSG00000174453	2	215250789	215468683	601	35	120000	-0.58599	0.72106	VWC2L	76353	25kb
ENSG00000138376	2	215565370	215699428	608	41	120000	0.13193	0.44752	BARD1	76353	25kb
ENSG00000144452	2	215771266	216028151	673	65	120000	-2.8439	0.99777	ABCA12	76353	25kb
ENSG00000138363	2	216151540	216239487	283	20	120000	-0.60225	0.7265	ATIC	76353	25kb
ENSG00000115414	2	216200163	216325895	373	33	120000	0.016769	0.49331	FN1	76353	25kb
ENSG00000118242	2	216784213	216923819	470	78	120000	2.8264	0.0023537	MREG	76353	25kb
ENSG00000115425	2	216836052	216972678	405	56	120000	-0.13097	0.5521	PECR	76353	25kb
ENSG00000163449	2	216921589	216992506	139	19	120000	-0.27821	0.60957	TMEM169	76353	25kb
ENSG00000079246	2	216947187	217096026	321	29	120000	-0.74262	0.77114	XRCC5	76353	25kb
ENSG00000144583	2	217097588	217261750	431	51	120000	-1.8782	0.96982	MARCH4	76353	25kb
ENSG00000138375	2	217252137	217372776	374	26	120000	0.86939	0.19232	SMARCAL1	76353	25kb
ENSG00000197756	2	217337912	217468903	363	44	120000	0.75506	0.22511	RPL37A	76353	25kb
ENSG00000115457	2	217472551	217554159	197	30	120000	0.81377	0.20789	IGFBP2	76353	25kb
ENSG00000115461	2	217511828	217585248	198	26	120000	0.52629	0.29934	IGFBP5	76353	25kb
ENSG00000118245	2	217699181	217749787	143	26	120000	0.49623	0.30987	TNP1	76353	25kb
ENSG00000223874	2	217710495	217761362	153	28	120000	0.14261	0.4433	AC007557.1	76353	25kb
ENSG00000231672	2	218123742	218646316	1563	88	120000	-0.045047	0.51797	DIRC3	76353	25kb
ENSG00000079308	2	218639512	218892718	748	111	120000	1.7384	0.041067	TNS1	76353	25kb
ENSG00000188282	2	218874683	218980304	258	24	120000	0.77201	0.22005	RUFY4	76353	25kb
ENSG00000180871	2	218965012	219026976	114	9	120000	0.31316	0.37708	CXCR2	76353	25kb
ENSG00000163464	2	219002568	219056718	97	13	120000	-0.23545	0.59307	CXCR1	76353	25kb
ENSG00000163466	2	219056817	219144079	190	18	120000	-0.21025	0.58326	ARPC2	76353	25kb
ENSG00000179921	2	219099219	219153582	137	12	120000	0.31971	0.3746	GPBAR1	76353	25kb
ENSG00000127837	2	219103850	219159980	142	11	120000	0.38679	0.34946	AAMP	76353	25kb
ENSG00000127838	2	219110115	219236516	338	24	120000	0.40993	0.34093	PNKD	76353	25kb
ENSG00000135926	2	219113915	219182309	164	11	120000	0.59569	0.27569	TMBIM1	76353	25kb
ENSG00000158428	2	219196579	219257822	153	25	120000	-0.22809	0.59021	C2orf62	76353	25kb
ENSG00000018280	2	219221752	219286617	140	30	120000	-1.1868	0.88235	SLC11A1	76353	25kb
ENSG00000144579	2	219237979	219295664	109	25	120000	-0.99545	0.84024	CTDSP1	76353	25kb
ENSG00000127831	2	219258815	219343018	146	17	120000	0.10845	0.45682	VIL1	76353	25kb
ENSG00000135913	2	219289974	219458084	289	11	120000	0.42792	0.33435	USP37	76353	25kb
ENSG00000144580	2	219408303	219486803	138	9	120000	0.42689	0.33473	RQCD1	76353	25kb
ENSG00000115556	2	219447488	219526907	139	10	120000	0.087898	0.46498	PLCD4	76353	25kb
ENSG00000115568	2	219477639	219549378	129	10	120000	0.14871	0.44089	ZNF142	76353	25kb
ENSG00000074582	2	219498487	219553166	98	9	120000	0.29888	0.38252	BCS1L	76353	25kb
ENSG00000163481	2	219503587	219562134	102	10	120000	0.38274	0.35096	RNF25	76353	25kb
ENSG00000163482	2	219511749	219592439	122	13	120000	0.78914	0.21501	STK36	76353	25kb
ENSG00000135912	2	219550568	219645139	179	7	120000	1.7994	0.035978	TTLL4	76353	25kb
ENSG00000135929	2	219621472	219705016	158	8	120000	1.8179	0.034543	CYP27A1	76353	25kb
ENSG00000115592	2	219662106	219721809	83	14	120000	1.7988	0.036028	PRKAG3	76353	25kb
ENSG00000115596	2	219699544	219763955	70	14	120000	0.57423	0.28291	WNT6	76353	25kb
ENSG00000135925	2	219720085	219789303	68	12	120000	0.21565	0.41463	WNT10A	76353	25kb
ENSG00000171450	2	219799377	219851876	75	10	120000	0.70209	0.24131	CDK5R2	76353	25kb
ENSG00000163497	2	219820809	219875379	77	13	120000	0.76671	0.22163	FEV	76353	25kb
ENSG00000163499	2	219829911	219883143	63	14	120000	0.93876	0.17393	CRYBA2	76353	25kb
ENSG00000181378	2	219842568	219931249	158	19	120000	-1.0481	0.85269	CCDC108	76353	25kb
ENSG00000163501	2	219894142	219950189	136	11	120000	-1.4225	0.92256	IHH	76353	25kb
ENSG00000187736	2	219915039	220050587	272	14	120000	-1.1538	0.87571	NHEJ1	76353	25kb
ENSG00000213901	2	219915051	220060549	286	15	120000	-1.1469	0.87428	SLC23A3	76353	25kb
ENSG00000115649	2	220011619	220067828	121	13	120000	-0.98075	0.83664	CNPPD1	76353	25kb
ENSG00000144567	2	220015947	220075201	124	16	120000	-0.87456	0.80909	FAM134A	76353	25kb
ENSG00000158552	2	220034959	220099373	133	21	120000	-0.51507	0.69675	ZFAND2B	76353	25kb
ENSG00000115657	2	220049490	220108712	115	21	120000	-0.46935	0.68059	ABCB6	76353	25kb
ENSG00000198925	2	220049494	220119439	133	22	120000	-0.46951	0.68065	ATG9A	76353	25kb
ENSG00000163516	2	220069479	220126391	104	14	120000	-0.20218	0.58011	ANKZF1	76353	25kb
ENSG00000163521	2	220076328	220135200	120	14	120000	-0.31664	0.62424	GLB1L	76353	25kb
ENSG00000115661	2	220085177	220140059	125	14	120000	-0.30202	0.61868	STK16	76353	25kb
ENSG00000127824	2	220089433	220167892	174	21	120000	0.12553	0.45005	TUBA4A	76353	25kb
ENSG00000135924	2	220118989	220176622	132	19	120000	0.51598	0.30293	DNAJB2	76353	25kb
ENSG00000054356	2	220129345	220199370	178	21	120000	1.192	0.11664	PTPRN	76353	25kb
ENSG00000182698	2	220167131	220222899	169	18	120000	1.2814	0.10003	RESP18	76353	25kb
ENSG00000123992	2	220213268	220289744	178	25	120000	0.4782	0.31625	DNPEP	76353	25kb
ENSG00000175084	2	220258099	220316461	122	24	120000	-1.1613	0.87725	DES	76353	25kb
ENSG00000072195	2	220274568	220388009	229	38	120000	-0.66876	0.74818	SPEG	76353	25kb
ENSG00000144591	2	220338589	220396710	113	14	120000	-0.21658	0.58573	GMPPA	76353	25kb
ENSG00000072182	2	220353892	220428494	178	26	120000	-0.95023	0.829	ASIC4	76353	25kb
ENSG00000123989	2	220378669	220433509	143	22	120000	-0.90649	0.81766	CHPF	76353	25kb
ENSG00000188760	2	220383385	220440317	157	25	120000	-0.8419	0.80008	TMEM198	76353	25kb
ENSG00000124006	2	220390451	220461581	185	32	120000	-0.92635	0.82287	OBSL1	76353	25kb
ENSG00000123999	2	220408884	220465435	148	32	120000	-0.74281	0.7712	INHA	76353	25kb
ENSG00000144589	2	220437582	220506173	121	35	120000	-1.2465	0.89372	STK11IP	76353	25kb
ENSG00000114923	2	220467049	220531702	115	22	120000	-0.76777	0.77869	SLC4A3	76353	25kb
ENSG00000116106	2	222257747	222463922	502	49	120000	-0.76239	0.77709	EPHA4	76353	25kb
ENSG00000135903	2	223039607	223188715	455	55	120000	-1.7107	0.95643	PAX3	76353	25kb
ENSG00000163081	2	223137866	223194936	135	31	120000	-2.0727	0.9809	CCDC140	76353	25kb
ENSG00000163082	2	223264236	223450667	474	62	120000	0.67736	0.24909	SGPP2	76353	25kb
ENSG00000116120	2	223410255	223546056	361	27	120000	-0.35943	0.64036	FARSB	76353	25kb
ENSG00000124003	2	223511505	223599649	267	35	120000	-0.9808	0.83665	MOGAT1	76353	25kb
ENSG00000123983	2	223700652	223834357	360	30	120000	-0.18409	0.57303	ACSL3	76353	25kb
ENSG00000152049	2	223891532	224088117	786	39	120000	0.039583	0.48421	KCNE4	76353	25kb
ENSG00000171951	2	224436658	224492221	110	17	120000	-0.99907	0.84112	SCG2	76353	25kb
ENSG00000152056	2	224591403	224727744	481	38	120000	0.6849	0.2467	AP1S3	76353	25kb
ENSG00000085449	2	224695433	224835104	329	18	120000	-0.5574	0.71137	WDFY1	76353	25kb
ENSG00000135900	2	224797121	224857431	147	19	120000	0.66792	0.25209	MRPL44	76353	25kb
ENSG00000135919	2	224814829	224929036	373	36	120000	1.0549	0.14573	SERPINE2	76353	25kb
ENSG00000124019	2	225218415	225291802	311	21	120000	1.4979	0.067076	FAM124B	76353	25kb
ENSG00000036257	2	225309867	225475110	447	23	120000	0.64226	0.26035	CUL3	76353	25kb
ENSG00000135905	2	225604807	225932162	848	57	120000	-1.2539	0.89506	DOCK10	76353	25kb
ENSG00000144460	2	226240364	226543734	562	45	120000	2.2402	0.012538	NYAP2	76353	25kb
ENSG00000169047	2	227571033	227689475	264	27	120000	1.4711	0.070635	IRS1	76353	25kb
ENSG00000144468	2	227675297	227888931	438	19	120000	0.24575	0.40294	RHBDD1	76353	25kb
ENSG00000081052	2	227842427	228053829	640	48	120000	0.16316	0.43519	COL4A4	76353	25kb
ENSG00000169031	2	228004281	228204508	917	70	120000	0.9516	0.17065	COL4A3	76353	25kb
ENSG00000168958	2	228164867	228247550	314	23	120000	-1.252	0.89472	MFF	76353	25kb
ENSG00000168955	2	228201872	228271711	329	38	120000	-0.91522	0.81996	TM4SF20	76353	25kb
ENSG00000173744	2	228311868	228450930	455	25	120000	2.0956	0.018057	AGFG1	76353	25kb

ENSG00000042304	2	228449806	228523036	429	39	120000	1.4439	0.074384	C2orf83	76353	25kb
ENSG00000135917	2	228524926	228607728	432	38	120000	-0.63979	0.73885	SLC19A3	76353	25kb
ENSG00000115009	2	228653558	228707272	138	37	120000	-0.11585	0.54611	CCL20	76353	25kb
ENSG00000123977	2	228710770	228814060	395	24	120000	-1.1825	0.88149	DAW1	76353	25kb
ENSG00000153820	2	228819666	229071361	765	35	120000	0.2443	0.4035	SPHKAP	76353	25kb
ENSG00000153823	2	229690242	230161001	1427	127	120000	-0.2138	0.58465	PID1	76353	25kb
ENSG00000187957	2	230197345	230604274	1461	112	120000	0.47393	0.31778	DNER	76353	25kb
ENSG00000153827	2	230603554	230812955	276	20	120000	0.11411	0.45457	TRIP12	76353	25kb
ENSG00000153832	2	230762018	230902825	219	28	120000	-0.68105	0.75208	FBXO36	76353	25kb
ENSG00000163053	2	230874698	230958715	255	30	120000	-1.5254	0.93643	SLC16A14	76353	25kb
ENSG00000135899	2	231007009	231115444	418	36	120000	-0.22132	0.58758	SP110	76353	25kb
ENSG00000079263	2	231042826	231248762	693	51	120000	0.48018	0.31555	SP140	76353	25kb
ENSG00000185404	2	231166899	231293447	486	38	120000	0.67643	0.24938	SP140L	76353	25kb
ENSG00000067066	2	231255657	231433805	686	49	120000	-0.11536	0.54592	SP100	76353	25kb
ENSG00000135932	2	231552560	231710790	318	27	120000	1.3616	0.086656	CAB39	76353	25kb
ENSG00000135916	2	231704354	231768963	259	41	120000	2.2175	0.013294	ITM2C	76353	25kb
ENSG00000135898	2	231747033	231850781	429	65	120000	0.062661	0.47502	GPR55	76353	25kb
ENSG00000173699	2	231835836	231914770	345	38	120000	0.48338	0.31441	SPATA3	76353	25kb
ENSG00000204128	2	231877205	231939434	210	33	120000	-0.40454	0.65709	C2orf72	76353	25kb
ENSG00000173692	2	231896578	232062541	335	24	120000	-0.70577	0.75983	PSMD1	76353	25kb
ENSG00000135914	2	231947944	232014832	101	16	120000	-0.60173	0.72632	HTR2B	76353	25kb
ENSG00000135931	2	232038260	232264548	524	36	120000	-0.29311	0.61528	ARMC9	76353	25kb
ENSG00000156966	2	232235254	232290875	146	19	120000	-0.23604	0.5933	B3GNT7	76353	25kb
ENSG00000233538	2	232291906	232342864	147	24	120000	-0.89217	0.81385	AC017104.2	76353	25kb
ENSG00000115053	2	232293242	232373352	244	31	120000	-1.2532	0.89493	NCL	76353	25kb
ENSG00000171596	2	232362871	232420206	188	20	120000	-1.8934	0.97085	NMUR1	76353	25kb
ENSG00000177673	2	232432575	232483994	137	22	120000	-2.3745	0.99121	C2orf57	76353	25kb
ENSG00000269363	2	232546517	232596621	157	17	120000	0.28424	0.38811	MGC4771	76353	25kb
ENSG00000187514	2	232546605	232603251	169	18	120000	0.36358	0.35808	PTMA	76353	25kb
ENSG00000156973	2	232572135	232675982	238	21	120000	0.23624	0.40662	PDE6D	76353	25kb
ENSG00000144524	2	232621381	232698963	142	17	120000	0.1258	0.44994	COPS7B	76353	25kb
ENSG00000163273	2	232761530	232816113	149	34	120000	-1.2901	0.90149	NPPC	76353	25kb
ENSG00000144535	2	232800955	233234060	1090	33	120000	-0.61546	0.73087	DIS3L2	76353	25kb
ENSG00000163283	2	233218244	233272599	154	27	120000	0.012731	0.49492	ALPP	76353	25kb
ENSG00000163286	2	233246553	233300424	173	19	120000	1.4879	0.068387	ALPPL2	76353	25kb
ENSG00000163295	2	233295833	233349742	225	32	120000	1.6993	0.044628	ALPI	76353	25kb
ENSG00000171551	2	233319537	233377538	200	41	120000	0.87047	0.19202	ECEL1	76353	25kb
ENSG00000237412	2	233360173	233415422	174	31	120000	1.2999	0.09681	PRSS56	76353	25kb
ENSG00000135902	2	233365703	233426377	193	30	120000	1.0162	0.15476	CHRND	76353	25kb
ENSG00000196811	2	233379437	233436113	182	22	120000	0.56125	0.28731	CHRNG	76353	25kb
ENSG00000221944	2	233387779	233440226	155	17	120000	0.36325	0.35821	TIGD1	76353	25kb
ENSG00000135930	2	233389762	233473354	219	23	120000	0.10938	0.45645	EIF4E2	76353	25kb
ENSG00000115468	2	233445767	233572491	378	53	120000	-1.6551	0.95105	EFHD1	76353	25kb
ENSG00000204120	2	233537009	233750285	571	30	120000	-0.31243	0.62264	GIGYF2	76353	25kb
ENSG00000115474	2	233606174	233666278	140	16	120000	0.089023	0.46453	KCNJ13	76353	25kb
ENSG00000182600	2	233696980	233768418	197	23	120000	-0.3253	0.62752	C2orf82	76353	25kb
ENSG00000066248	2	233718396	233902982	641	56	120000	-0.13432	0.55343	NGEF	76353	25kb
ENSG00000222001	2	233852324	233905595	160	30	120000	0.72301	0.23484	AC106876.2	76353	25kb
ENSG00000115488	2	233872382	233924767	142	22	120000	0.69577	0.24329	NEU2	76353	25kb
ENSG00000168918	2	233899677	234141549	744	75	120000	1.5208	0.064152	INPP5D	76353	25kb
ENSG00000085978	2	234093697	234229320	385	48	120000	-0.021493	0.50857	ATG16L1	76353	25kb
ENSG00000130561	2	234191462	234280701	250	31	120000	1.3102	0.09507	SAG	76353	25kb
ENSG00000077044	2	234238153	234405750	346	27	120000	1.8902	0.029369	DGKD	76353	25kb
ENSG00000085982	2	234359166	234500428	273	15	120000	0.93281	0.17546	USP40	76353	25kb
ENSG00000241635	2	234501291	234706956	673	41	120000	-1.3273	0.90779	UGT1A8	76353	25kb
ENSG00000242515	2	234520100	234706951	608	40	120000	-1.3224	0.90699	UGT1A10	76353	25kb
ENSG00000241119	2	234555499	234706946	535	37	120000	-1.2627	0.89665	UGT1A9	76353	25kb
ENSG00000244122	2	234565584	234706945	511	34	120000	-1.1424	0.87336	UGT1A7	76353	25kb
ENSG00000167165	2	234575253	234706946	487	37	120000	-1.2544	0.89515	UGT1A6	76353	25kb
ENSG00000240224	2	234596638	234706945	398	36	120000	-1.036	0.8499	UGT1A5	76353	25kb
ENSG00000244474	2	234602424	234706945	372	35	120000	-0.93541	0.82521	UGT1A4	76353	25kb
ENSG00000243135	2	234612754	234706945	348	34	120000	-0.86933	0.80767	UGT1A3	76353	25kb
ENSG00000242366	2	234643894	234706945	221	35	120000	-1.0633	0.85619	UGT1A1	76353	25kb
ENSG00000185038	2	234659370	234767069	397	62	120000	-0.87489	0.80918	MROH2A	76353	25kb
ENSG00000123485	2	234717062	234788212	283	27	120000	-0.66622	0.74737	HJURP	76353	25kb
ENSG00000144481	2	234801043	234953166	555	68	120000	-0.96405	0.83249	TRPM8	76353	25kb
ENSG00000072080	2	234934323	235010778	327	31	120000	-0.67929	0.75152	SPP2	76353	25kb
ENSG00000188042	2	235376685	235430697	171	35	120000	-1.2963	0.90256	ARL4C	76353	25kb
ENSG00000130147	2	235835617	235989358	507	75	120000	1.8573	0.031634	SH3BP4	76353	25kb
ENSG00000157985	2	236377733	237065444	1739	142	120000	0.40755	0.3418	AGAP1	76353	25kb
ENSG00000222007	2	236657499	236717031	181	6	120000	0.14477	0.44244	AC064874.1	76353	25kb
ENSG00000168505	2	237048879	237102012	169	12	120000	-0.73004	0.76732	GBX2	76353	25kb
ENSG00000182177	2	237077095	237198052	400	26	120000	-1.3769	0.91574	ASB18	76353	25kb
ENSG00000132321	2	237207794	237441185	491	37	120000	-0.62635	0.73446	IQCA1	76353	25kb
ENSG00000144476	2	237451430	237516001	165	19	120000	0.12611	0.44982	ACKR3	76353	25kb
ENSG00000198612	2	237968955	238034109	155	18	120000	0.77107	0.22033	COPS8	76353	25kb
ENSG00000224132	2	238140734	238191319	106	12	120000	0.46266	0.3218	AC112715.2	76353	25kb
ENSG00000163359	2	238207646	238348018	459	56	120000	-0.022474	0.50897	COL6A3	76353	25kb
ENSG00000222022	2	238305063	238358919	146	23	120000	-0.31831	0.62488	AC112721.1	76353	25kb
ENSG00000115648	2	238369071	238488961	558	32	120000	-0.64179	0.7395	MLPH	76353	25kb
ENSG00000071677	2	238450217	238500818	236	26	120000	-0.34918	0.63652	PRLH	76353	25kb
ENSG00000124839	2	238457965	238535257	383	43	120000	-0.78684	0.78431	RAB17	76353	25kb
ENSG00000234949	2	238474663	238529624	292	35	120000	-0.55022	0.70891	AC104667.3	76353	25kb
ENSG00000124831	2	238511219	238747325	864	49	120000	-0.96489	0.8327	LRRFIP1	76353	25kb
ENSG00000177483	2	238682032	238776451	255	25	120000	-0.25173	0.59937	RBM44	76353	25kb
ENSG00000132329	2	238742536	238845756	372	70	120000	-0.58794	0.72171	RAMP1	76353	25kb
ENSG00000184182	2	238850469	238976236	365	16	120000	-0.41276	0.66011	UBE2F	76353	25kb
ENSG00000258984	2	238850656	239033053	595	22	120000	-0.77791	0.78169	UBE2F-SCLY	76353	25kb
ENSG00000132330	2	238944530	239033054	322	20	120000	-1.2735	0.89857	SCLY	76353	25kb
ENSG00000144488	2	238983798	239066928	359	36	120000	-0.96805	0.83349	ESPNL	76353	25kb
ENSG00000168427	2	239022363	239086588	272	42	120000	-0.21332	0.58446	KLHL30	76353	25kb
ENSG00000178752	2	239042623	239102541	235	36	120000	0.28802	0.38667	FAM132B	76353	25kb
ENSG00000132323	2	239054042	239137370	332	27	120000	0.46102	0.32239	ILKAP	76353	25kb
ENSG00000186235	2	239108193	239165318	235	24	120000	0.45643	0.32404	AC016757.3	76353	25kb
ENSG00000144485	2	239121908	239174303	190	17	120000	0.36829	0.35633	HES6	76353	25kb
ENSG00000132326	2	239127679	239223743	309	25	120000	0.32658	0.37199	PER2	76353	25kb

ENSG00000268396	2	239167329	239217498	127	21	120000	0.41521	0.339	AC012485.1	76353	25kb
ENSG00000204104	2	239204082	239334541	377	24	120000	-0.56645	0.71446	TRAF3IP1	76353	25kb
ENSG00000065802	2	239310383	239385891	251	25	120000	-0.85231	0.80298	ASB1	76353	25kb
ENSG00000233608	2	239731673	239820893	234	47	120000	-1.1351	0.87184	TWIST2	76353	25kb
ENSG00000068024	2	239944864	240348348	1390	119	120000	-0.042445	0.51693	HDAC4	76353	25kb
ENSG00000213828	2	240058493	240109053	174	20	120000	-0.0021513	0.50086	AC017028.1	76353	25kb
ENSG00000222020	2	240298130	240349058	169	40	120000	0.26179	0.39674	AC062017.1	76353	25kb
ENSG00000196758	2	240474995	240532788	245	49	120000	3.2756	0.00052716	AC079612.1	76353	25kb
ENSG00000220256	2	240659554	240749577	431	39	120000	-1.3833	0.91672	AC093802.1	76353	25kb
ENSG00000130414	2	240806867	240989819	774	63	120000	-1.2211	0.88898	NDUFA10	76353	25kb
ENSG00000182083	2	240943841	240994906	195	38	120000	-1.3718	0.91494	OR6B2	76353	25kb
ENSG00000221961	2	240956230	241007399	190	37	120000	-1.2767	0.89915	PRR21	76353	25kb
ENSG00000178586	2	240959494	241010489	193	36	120000	-1.2085	0.88658	OR6B3	76353	25kb
ENSG00000267963	2	241028907	241089304	313	25	120000	-1.7671	0.96139	AC013469.1	76353	25kb
ENSG00000172428	2	241040980	241101224	328	32	120000	-1.9728	0.97574	MYEOV2	76353	25kb
ENSG00000178602	2	241053446	241108979	307	32	120000	-1.7313	0.9583	OTOS	76353	25kb
ENSG00000063660	2	241350088	241432493	370	45	120000	0.46353	0.32149	GPC1	76353	25kb
ENSG00000218416	2	241363732	241421131	255	37	120000	0.45157	0.32579	AC110619.2	76353	25kb
ENSG00000144504	2	241393839	241533626	481	63	120000	-0.15279	0.56072	ANKMY1	76353	25kb
ENSG00000188542	2	241474471	241528431	145	29	120000	-1.1007	0.86449	DUSP28	76353	25kb
ENSG00000142327	2	241480221	241545789	227	31	120000	-1.4523	0.9268	RNPEPL1	76353	25kb
ENSG00000142330	2	241501133	241582122	342	41	120000	-1.8783	0.96983	CAPN10	76353	25kb
ENSG00000178623	2	241519848	241595676	382	46	120000	-1.8297	0.96635	GPR35	76353	25kb
ENSG00000185176	2	241590835	241647323	187	23	120000	-0.14818	0.5589	AQP12B	76353	25kb
ENSG00000268822	2	241599478	241649705	131	21	120000	0.23186	0.40832	AC011298.1	76353	25kb
ENSG00000184945	2	241606262	241662900	136	30	120000	0.30649	0.37962	AQP12A	76353	25kb
ENSG00000130294	2	241628181	241784725	422	52	120000	-0.20171	0.57993	KIF1A	76353	25kb
ENSG00000172482	2	241782896	241844919	318	13	120000	-0.87857	0.81018	AGXT	76353	25kb
ENSG00000172478	2	241800465	241861306	311	15	120000	-1.0039	0.84228	C2orf54	76353	25kb
ENSG00000226321	2	241833202	241957728	391	38	120000	-0.61314	0.73011	AC104809.3	76353	25kb
ENSG00000162804	2	241913255	242059983	434	44	120000	-0.45949	0.67706	SNED1	76353	25kb
ENSG00000122085	2	241986584	242066747	254	21	120000	-0.0015008	0.5006	MTERFD2	76353	25kb
ENSG00000115687	2	242020514	242114679	314	23	120000	-0.28134	0.61077	PASK	76353	25kb
ENSG00000115685	2	242063991	242148067	278	23	120000	0.051919	0.4793	PPP1R7	76353	25kb
ENSG00000146205	2	242102924	242189792	247	44	120000	-0.30737	0.62072	ANO7	76353	25kb
ENSG00000115677	2	242141679	242281476	330	32	120000	-0.16976	0.5674	HDLBP	76353	25kb
ENSG00000268485	2	242142319	242193850	124	28	120000	-0.9162	0.82022	AC104841.2	76353	25kb
ENSG00000168385	2	242229515	242318442	183	21	120000	-0.02448	0.50977	SEPT2	76353	25kb
ENSG00000006607	2	242270658	242459256	455	29	120000	-0.33154	0.62988	FARP2	76353	25kb
ENSG00000115694	2	242407089	242474145	194	30	120000	-0.6653	0.74707	STK25	76353	25kb
ENSG00000176720	2	242473136	242538546	225	35	120000	-0.15444	0.56137	BOK	76353	25kb
ENSG00000176946	2	242498820	242601864	300	22	120000	-0.20562	0.58145	THAP4	76353	25kb
ENSG00000168397	2	242551628	242638272	335	26	120000	-0.64988	0.74212	ATG4B	76353	25kb
ENSG00000168393	2	242590157	242651406	286	20	120000	-0.74414	0.77161	DTYMK	76353	25kb
ENSG00000168395	2	242616450	242693893	326	31	120000	-0.8366	0.79859	ING5	76353	25kb
ENSG00000180902	2	242648994	242733231	300	35	120000	-0.052636	0.52099	D2HGDH	76353	25kb
ENSG00000154252	2	242691240	242768623	259	33	120000	0.0080978	0.49677	GAL3ST2	76353	25kb
ENSG00000204099	2	242724920	242783739	182	31	120000	-1.766	0.9613	NEU4	76353	25kb
ENSG00000188389	2	242767033	242826060	206	25	120000	-1.4061	0.92015	PDCD1	76353	25kb
ENSG00000188011	2	242786752	242840975	238	22	120000	-1.1721	0.87941	CXXC11	76353	25kb
ENSG00000216921	2	242811136	242869702	273	19	120000	-1.6621	0.95176	AC131097.4	76353	25kb
ENSG00000134121	3	213279	476090	1032	86	120000	0.60726	0.27184	CHL1	76353	25kb
ENSG00000134115	3	1109260	1470901	1652	142	120000	0.59648	0.27543	CNTN6	76353	25kb
ENSG00000144619	3	2115497	3124645	4304	336	120000	-0.55189	0.70949	CNTN4	76353	25kb
ENSG00000091181	3	3086233	3193297	529	101	120000	-0.67174	0.74913	IL5RA	76353	25kb
ENSG00000072756	3	3143600	3217563	428	48	120000	-0.56987	0.71562	TRNT1	76353	25kb
ENSG00000113851	3	3165676	3246394	432	32	120000	-0.32072	0.62579	CRBN	76353	25kb
ENSG00000144455	3	3717498	4533965	3069	138	120000	-0.19124	0.57583	SUMF1	76353	25kb
ENSG00000175928	3	3816121	3914387	303	40	120000	0.72889	0.23304	LRRN1	76353	25kb
ENSG00000170364	3	4319988	4384251	181	27	120000	0.46049	0.32258	SETMAR	76353	25kb
ENSG00000150995	3	4510032	4914524	1469	167	120000	1.7172	0.042975	ITPR1	76353	25kb
ENSG00000235978	3	4830978	4953977	424	48	120000	1.097	0.13632	AC018816.3	76353	25kb
ENSG00000134107	3	4995801	5052008	180	32	120000	0.38954	0.34844	BHLHE40	76353	25kb
ENSG00000134108	3	5138905	5247596	256	26	120000	-0.66514	0.74702	ARL8B	76353	25kb
ENSG00000268509	3	5204227	5254982	73	18	120000	-1.3137	0.90553	AC026202.1	76353	25kb
ENSG00000134109	3	5204331	5286642	219	43	120000	-0.82211	0.79449	EDEM1	76353	25kb
ENSG00000196277	3	6786688	7808215	3336	234	120000	-0.54484	0.70707	GRM7	76353	25kb
ENSG00000071282	3	8518393	8634805	411	33	120000	0.6301	0.26431	LMCD1	76353	25kb
ENSG00000125046	3	8636086	8811726	766	128	120000	0.16401	0.43486	SSUH2	76353	25kb
ENSG00000182533	3	8750486	8908492	689	80	120000	-0.032131	0.51282	CAV3	76353	25kb
ENSG00000180914	3	8767094	8836314	334	59	120000	0.82567	0.2045	OXTR	76353	25kb
ENSG00000070950	3	8792088	9030457	938	50	120000	0.17467	0.43067	RAD18	76353	25kb
ENSG00000196220	3	8997275	9429737	1321	160	120000	0.08804	0.46492	SRGAP3	76353	25kb
ENSG00000134077	3	9379526	9453475	180	23	120000	-1.8381	0.96697	THUMPD3	76353	25kb
ENSG00000168137	3	9414299	9545924	259	24	120000	-1.8784	0.96984	SETD5	76353	25kb
ENSG00000156959	3	9518481	9620486	295	23	120000	0.30997	0.37829	LHFPL4	76353	25kb
ENSG00000163719	3	9666117	9769077	190	31	120000	0.6134	0.26981	MTMR14	76353	25kb
ENSG00000144550	3	9720491	9796592	153	43	120000	0.51118	0.30461	CPNE9	76353	25kb
ENSG00000156983	3	9748413	9814702	125	33	120000	0.019502	0.49222	BRPF1	76353	25kb
ENSG00000114026	3	9766628	9854903	173	29	120000	0.12431	0.45053	OGG1	76353	25kb
ENSG00000134072	3	9774026	9836676	124	22	120000	0.11667	0.45356	CAMK1	76353	25kb
ENSG00000171148	3	9796544	9859695	134	20	120000	0.44989	0.3264	TADA3	76353	25kb
ENSG00000241553	3	9809179	9874410	175	21	120000	0.68311	0.24727	ARPC4	76353	25kb
ENSG00000250151	3	9809766	9902085	242	30	120000	0.47567	0.31716	ARPC4-TTLL3	76353	25kb
ENSG00000214021	3	9824770	9921822	263	32	120000	0.4005	0.34439	TTLL3	76353	25kb
ENSG00000156990	3	9854533	9911286	176	28	120000	0.43564	0.33155	RPUSD3	76353	25kb
ENSG00000187288	3	9883398	9946938	163	30	120000	0.24739	0.4023	CIDEC	76353	25kb
ENSG00000171135	3	9907238	9961033	137	16	120000	0.12306	0.45103	JAGN1	76353	25kb
ENSG00000163701	3	9919296	9983086	161	23	120000	-0.31001	0.62172	IL17RE	76353	25kb
ENSG00000163702	3	9933758	10000314	163	25	120000	-0.96612	0.83301	IL17RC	76353	25kb
ENSG00000163703	3	9950506	10012097	132	22	120000	-1.7103	0.95639	CRELD1	76353	25kb
ENSG00000163704	3	9962226	10019078	130	16	120000	-1.3588	0.91289	PRRT3	76353	25kb
ENSG00000125037	3	9979221	10077800	254	8	120000	-1.1552	0.876	EMC3	76353	25kb
ENSG00000144554	3	10043098	10168614	339	9	120000	-0.53724	0.70445	FANCD2	76353	25kb
ENSG00000163705	3	10098001	10174915	221	10	120000	-0.16777	0.56662	FANCD2OS	76353	25kb
ENSG00000254999	3	10132276	10193874	219	14	120000	-0.21181	0.58387	BRK1	76353	25kb

ENSG00000134086	3	10157692	10218904	219	24	120000	-0.44016	0.67009	VHL	76353	25kb
ENSG00000134070	3	10181549	10310427	415	44	120000	-0.81493	0.79244	IRAK2	76353	25kb
ENSG00000157014	3	10264707	10347902	224	27	120000	0.095035	0.46214	TATDN2	76353	25kb
ENSG00000272410	3	10266056	10352480	241	30	120000	0.1015	0.45958	RP11-438J1.1	76353	25kb
ENSG00000157017	3	10302359	10359631	166	23	120000	0.18402	0.427	GHRL	76353	25kb
ENSG00000157020	3	10309815	10387862	217	30	120000	-0.22978	0.59087	SEC13	76353	25kb
ENSG00000157087	3	10340707	10774716	1561	134	120000	-0.05525	0.52203	ATP2B2	76353	25kb
ENSG00000132164	3	10832885	11007419	511	41	120000	-0.22994	0.59093	SLC6A11	76353	25kb
ENSG00000157103	3	11009410	11105933	303	65	120000	1.6479	0.049682	SLC6A1	76353	25kb
ENSG00000196639	3	11153779	11330243	506	50	120000	-0.83021	0.79679	HRH1	76353	25kb
ENSG00000197548	3	11288995	11624139	720	26	120000	0.4351	0.33174	ATG7	76353	25kb
ENSG00000144560	3	11572544	11787220	719	65	120000	0.5885	0.2781	VGLL4	76353	25kb
ENSG00000144559	3	11806916	11913393	294	36	120000	2.1546	0.015598	TAMM41	76353	25kb
ENSG00000157150	3	12169551	12225851	138	18	120000	-1.3522	0.91184	TIMP4	76353	25kb
ENSG00000132170	3	12303867	12500855	462	43	120000	2.4173	0.0078175	PPARG	76353	25kb
ENSG00000154743	3	12500931	12606122	362	19	120000	0.011385	0.49546	TSEN2	76353	25kb
ENSG00000225526	3	12531433	12627558	299	21	120000	0.31003	0.37827	C3orf83	76353	25kb
ENSG00000075975	3	12573513	12650212	190	22	120000	0.78711	0.21561	MKRN2	76353	25kb
ENSG00000132155	3	12600100	12730725	317	19	120000	1.3408	0.089991	RAF1	76353	25kb
ENSG00000088726	3	12750024	12835956	266	22	120000	-0.57192	0.71631	TMEM40	76353	25kb
ENSG00000144712	3	12812971	12938415	419	39	120000	1.4308	0.076251	CAND2	76353	25kb
ENSG00000144713	3	12850984	12908087	180	18	120000	1.5395	0.061845	RPL32	76353	25kb
ENSG00000144711	3	12913719	13139617	734	86	120000	1.2648	0.10297	IQSEC1	76353	25kb
ENSG00000132182	3	13332737	13486809	566	26	120000	-0.91775	0.82063	NUP210	76353	25kb
ENSG00000163517	3	13496224	13572916	234	46	120000	1.1688	0.12125	HDAC11	76353	25kb
ENSG00000163520	3	13548824	13704922	594	61	120000	1.4529	0.073119	FBLN2	76353	25kb
ENSG00000154764	3	13832755	13946618	374	54	120000	0.47127	0.31872	WNT7A	76353	25kb
ENSG00000180438	3	13953756	14149311	357	20	120000	-0.57388	0.71698	TPRXL	76353	25kb
ENSG00000163528	3	14128580	14191370	133	19	120000	-1.098	0.86389	CHCHD4	76353	25kb
ENSG00000170876	3	14141440	14210179	146	23	120000	-0.72271	0.76507	TMEM43	76353	25kb
ENSG00000268279	3	14151717	14214548	134	22	120000	-0.58554	0.72091	RP11-434D12.1	76353	25kb
ENSG00000154767	3	14161647	14245283	176	25	120000	-0.18333	0.57273	XPC	76353	25kb
ENSG00000170860	3	14194858	14267619	169	33	120000	0.065348	0.47395	LSM3	76353	25kb
ENSG00000131389	3	14419076	14555857	412	80	120000	-1.4821	0.93085	SLC6A6	76353	25kb
ENSG00000154781	3	14668271	14739166	200	24	120000	0.14191	0.44357	CCDC174	76353	25kb
ENSG00000131379	3	14691606	14839541	414	57	120000	-0.13633	0.55422	C3orf20	76353	25kb
ENSG00000154783	3	14835469	15000895	623	41	120000	0.69155	0.24461	FGD5	76353	25kb
ENSG00000177463	3	14964091	15120107	310	23	120000	-0.58045	0.71919	NR2C2	76353	25kb
ENSG00000131368	3	15058967	15131842	140	17	120000	-0.34678	0.63562	MRPS25	76353	25kb
ENSG00000131381	3	15086580	15165670	155	15	120000	-0.1832	0.57268	ZFYVE20	76353	25kb
ENSG00000131375	3	15222659	15319425	154	23	120000	0.72065	0.23556	CAPN7	76353	25kb
ENSG00000131370	3	15271360	15407875	336	55	120000	-0.50347	0.69268	SH3BP5	76353	25kb
ENSG00000206562	3	15397782	15507073	263	21	120000	-0.23051	0.59115	METTL6	76353	25kb
ENSG00000144597	3	15443862	15509120	156	17	120000	-0.034401	0.51372	EAF1	76353	25kb
ENSG00000206561	3	15466640	15588258	321	27	120000	0.05334	0.47873	COLQ	76353	25kb
ENSG00000131373	3	15577211	15668338	171	28	120000	0.58512	0.27923	HACL1	76353	25kb
ENSG00000169814	3	15617848	15712329	190	25	120000	0.3657	0.3573	BTD	76353	25kb
ENSG00000206560	3	15683743	15926278	496	18	120000	0.29257	0.38492	ANKRD28	76353	25kb
ENSG00000131386	3	16191156	16298499	385	51	120000	-0.055433	0.5221	GALNT15	76353	25kb
ENSG00000154813	3	16274485	16331479	184	20	120000	-0.056603	0.52257	DPH3	76353	25kb
ENSG00000154814	3	16281706	16416806	444	36	120000	-0.14478	0.55756	OXNAD1	76353	25kb
ENSG00000131378	3	16330081	16580533	764	67	120000	-1.3839	0.91681	RFTN1	76353	25kb
ENSG00000092345	3	16603299	16736813	589	59	120000	-1.7897	0.96325	DAZL	76353	25kb
ENSG00000154822	3	16819159	17157086	898	43	120000	1.1521	0.12463	PLCL2	76353	25kb
ENSG00000131374	3	17173654	18511309	2919	57	120000	-1.1151	0.8676	TBC1D5	76353	25kb
ENSG00000182568	3	18361879	18512080	315	43	120000	-0.72477	0.7657	SATB1	76353	25kb
ENSG00000183960	3	19164946	19602138	615	56	120000	-0.24451	0.59658	KCNH8	76353	25kb
ENSG00000163576	3	19895964	20013517	364	28	120000	-0.49107	0.68831	EFHB	76353	25kb
ENSG00000144566	3	19963571	20051667	318	25	120000	-0.37674	0.64682	RAB5A	76353	25kb
ENSG00000183977	3	19996453	20078822	266	23	120000	-0.36829	0.64367	PP2D1	76353	25kb
ENSG00000114166	3	20056515	20220896	610	39	120000	-1.4819	0.93082	KAT2B	76353	25kb
ENSG00000129810	3	20177085	20252784	276	22	120000	-0.92004	0.82122	SGOL1	76353	25kb
ENSG00000151789	3	21434915	22439812	4622	252	120000	0.36219	0.35861	ZNF385D	76353	25kb
ENSG00000182247	3	23219511	23658284	1430	29	120000	0.43088	0.33328	UBE2E2	76353	25kb
ENSG00000170142	3	23822394	23957807	281	29	120000	-0.84327	0.80046	UBE2E1	76353	25kb
ENSG00000197885	3	23908151	24013082	273	18	120000	0.30406	0.38054	NKIRAS1	76353	25kb
ENSG00000174748	3	23933036	23990183	180	16	120000	0.43006	0.33358	RPL15	76353	25kb
ENSG00000174738	3	23961751	24047109	248	19	120000	0.49383	0.31071	NR1D2	76353	25kb
ENSG00000151090	3	24133651	24561773	1220	127	120000	0.06728	0.47318	THRB	76353	25kb
ENSG00000077092	3	25190823	25664423	1861	114	120000	-0.4255	0.66476	RARB	76353	25kb
ENSG00000077097	3	25614475	25731398	288	23	120000	0.3826	0.35101	TOP2B	76353	25kb
ENSG00000151092	3	25735435	25856530	169	14	120000	0.7844	0.2164	NGLY1	76353	25kb
ENSG00000151093	3	25799408	25861025	92	14	120000	0.73523	0.2311	OXSM	76353	25kb
ENSG00000230891	3	25875024	25940191	156	17	120000	1.0582	0.14498	LINC00692	76353	25kb
ENSG00000179796	3	26639297	26777267	302	32	120000	0.098835	0.46063	LRRC3B	76353	25kb
ENSG00000163491	3	27126576	27435951	941	31	120000	1.6556	0.048899	NEK10	76353	25kb
ENSG00000033867	3	27389214	27550911	523	25	120000	1.0824	0.13954	SLC4A7	76353	25kb
ENSG00000163508	3	27732440	27789206	168	23	120000	1.0209	0.15364	EOMES	76353	25kb
ENSG00000187118	3	28258075	28391633	231	23	120000	0.29085	0.38558	CMC1	76353	25kb
ENSG00000163512	3	28331494	28415618	116	13	120000	0.2547	0.39948	AZI2	76353	25kb
ENSG00000206559	3	28365637	28604613	334	20	120000	-0.086229	0.53436	ZCWPW2	76353	25kb
ENSG00000144642	3	29297473	30076886	2990	184	120000	0.57898	0.2813	RBMS3	76353	25kb
ENSG00000163513	3	30622994	30760634	456	64	120000	-0.97734	0.8358	TGFBR2	76353	25kb
ENSG00000144644	3	30742692	30961257	689	46	120000	-0.11765	0.54683	GADL1	76353	25kb
ENSG00000163527	3	31549130	31704112	306	21	120000	-0.53549	0.70384	STT3B	76353	25kb
ENSG00000144645	3	31674382	32144072	1481	137	120000	-0.060737	0.52422	OSBPL10	76353	25kb
ENSG00000197385	3	31998263	32058120	185	20	120000	-0.2049	0.58118	ZNF860	76353	25kb
ENSG00000152642	3	32122181	32235205	422	30	120000	-0.87598	0.80948	GPD1L	76353	25kb
ENSG00000170293	3	32255171	32436817	669	91	120000	1.3839	0.083194	CMTM8	76353	25kb
ENSG00000153551	3	32408163	32549559	503	50	120000	-0.93561	0.82526	CMTM7	76353	25kb
ENSG00000091317	3	32497804	32569900	213	35	120000	-0.68661	0.75383	CMTM6	76353	25kb
ENSG00000144635	3	32542463	32637366	242	36	120000	-1.0096	0.84366	DYNC1LI1	76353	25kb
ENSG00000182973	3	32701637	32840367	530	24	120000	-0.287	0.61295	CNOT10	76353	25kb
ENSG00000206557	3	32834510	32964318	320	49	120000	0.49703	0.30958	TRIM71	76353	25kb
ENSG00000183813	3	32968066	33022841	142	19	120000	0.028497	0.48863	CCR4	76353	25kb
ENSG00000170266	3	33013100	33163722	526	39	120000	-0.57782	0.71831	GLB1	76353	25kb

ENSG00000188167	3	33106913	33163293	144	16	120000	-0.70956	0.76101	TMPPE	76353	25kb
ENSG00000170275	3	33130471	33214265	230	31	120000	-0.28513	0.61223	CRTAP	76353	25kb
ENSG00000173705	3	33166537	33285707	348	35	120000	0.48551	0.31366	SUSD5	76353	25kb
ENSG00000153558	3	33293517	33470154	402	24	120000	0.14327	0.44304	FBXL2	76353	25kb
ENSG00000153560	3	33404828	33507863	319	17	120000	-0.142	0.55646	UBP1	76353	25kb
ENSG00000163539	3	33512737	33784848	466	33	120000	0.16325	0.43516	CLASP2	76353	25kb
ENSG00000170248	3	33814844	33936194	357	20	120000	-0.68704	0.75397	PDCD6IP	76353	25kb
ENSG00000172995	3	35655437	35860988	441	34	120000	1.2451	0.10655	ARPP21	76353	25kb
ENSG00000144681	3	36396836	36614499	842	46	120000	-0.32953	0.62912	STAC	76353	25kb
ENSG00000163673	3	36728913	36806352	192	40	120000	-0.22781	0.5901	DCLK3	76353	25kb
ENSG00000168016	3	36843311	37011548	356	29	120000	0.30392	0.38059	TRANK1	76353	25kb
ENSG00000178567	3	37002357	37059795	89	9	120000	0.41875	0.3377	EPM2AIP1	76353	25kb
ENSG00000076242	3	37009823	37132380	212	14	120000	0.15666	0.43776	MLH1	76353	25kb
ENSG00000093167	3	37069117	37250180	319	14	120000	0.057958	0.47689	LRRFIP2	76353	25kb
ENSG00000144674	3	37259668	37433242	330	15	120000	1.5332	0.062611	GOLGA4	76353	25kb
ENSG00000198590	3	37402760	37501988	186	22	120000	1.2805	0.10019	C3orf35	76353	25kb
ENSG00000144668	3	37468606	37890005	1062	92	120000	0.20856	0.41739	ITGA9	76353	25kb
ENSG00000144677	3	37878451	38050960	361	32	120000	-1.235	0.89159	CTDSPL	76353	25kb
ENSG00000136059	3	38004550	38073679	176	22	120000	-0.59118	0.7228	VILL	76353	25kb
ENSG00000187091	3	38023987	38096253	209	28	120000	-0.13541	0.55385	PLCD1	76353	25kb
ENSG00000008226	3	38055696	38190516	288	23	120000	0.4068	0.34208	DLEC1	76353	25kb
ENSG00000060971	3	38119620	38203733	132	22	120000	-0.32666	0.62804	ACAA1	76353	25kb
ENSG00000172936	3	38154969	38209513	66	14	120000	-0.58086	0.71933	MYD88	76353	25kb
ENSG00000172939	3	38181580	38321979	187	23	120000	-0.27996	0.61024	OXSR1	76353	25kb
ENSG00000172940	3	38282303	38344806	163	10	120000	1.1235	0.13061	SLC22A13	76353	25kb
ENSG00000144671	3	38298785	38385066	285	27	120000	1.617	0.05294	SLC22A14	76353	25kb
ENSG00000093217	3	38363251	38487839	388	29	120000	0.59596	0.2756	XYLB	76353	25kb
ENSG00000114739	3	38470342	38559633	172	10	120000	-0.74821	0.77283	ACVR2B	76353	25kb
ENSG00000157036	3	38512618	38608652	209	21	120000	-1.0998	0.86429	EXOG	76353	25kb
ENSG00000183873	3	38564548	38716164	403	61	120000	-1.6512	0.95065	SCN5A	76353	25kb
ENSG00000185313	3	38713293	38860501	479	49	120000	0.024076	0.4904	SCN10A	76353	25kb
ENSG00000168356	3	38862260	39017052	373	24	120000	-1.4093	0.92062	SCN11A	76353	25kb
ENSG00000114742	3	39068489	39163155	275	22	120000	-0.88234	0.8112	WDR48	76353	25kb
ENSG00000114745	3	39113150	39174854	177	23	120000	-1.1873	0.88244	GORASP1	76353	25kb
ENSG00000168026	3	39124152	39205394	231	30	120000	-1.2043	0.88577	TTC21A	76353	25kb
ENSG00000144655	3	39158346	39221053	183	27	120000	-1.2607	0.8963	CSRNP1	76353	25kb
ENSG00000168334	3	39199701	39259087	155	29	120000	-0.61188	0.72969	XIRP1	76353	25kb
ENSG00000168329	3	39279985	39348226	262	35	120000	-0.084157	0.53353	CX3CR1	76353	25kb
ENSG00000179934	3	39346197	39400171	140	24	120000	-1.4798	0.93054	CCR8	76353	25kb
ENSG00000144659	3	39399839	39463842	272	23	120000	-1.1359	0.87201	SLC25A38	76353	25kb
ENSG00000168028	3	39423180	39479033	272	12	120000	-1.0423	0.85137	RPSA	76353	25kb
ENSG00000168314	3	39483689	39595970	451	15	120000	0.826	0.2044	MOBP	76353	25kb
ENSG00000170011	3	39825405	40326812	1423	72	120000	0.29216	0.38508	MYRIP	76353	25kb
ENSG00000114784	3	40326175	40378915	116	9	120000	-0.68305	0.75271	EIF1B	76353	25kb
ENSG00000168032	3	40403647	40495110	201	12	120000	-0.68405	0.75303	ENTPD3	76353	25kb
ENSG00000188846	3	40473783	40528861	141	12	120000	-0.73564	0.76903	RPL14	76353	25kb
ENSG00000177873	3	40493604	40556727	126	13	120000	-0.47076	0.68109	ZNF619	76353	25kb
ENSG00000177842	3	40522483	40585227	98	18	120000	-0.13398	0.55329	ZNF620	76353	25kb
ENSG00000172888	3	40541369	40641176	168	19	120000	-0.76745	0.77859	ZNF621	76353	25kb
ENSG00000168036	3	41211328	41326587	192	21	120000	-0.89668	0.81506	CTNNB1	76353	25kb
ENSG00000168038	3	41263090	42028922	2266	41	120000	-0.74111	0.77069	ULK4	76353	25kb
ENSG00000182606	3	42030294	42292381	717	52	120000	1.5238	0.063785	TRAK1	76353	25kb
ENSG00000187094	3	42274317	42332699	126	21	120000	1.9115	0.02797	CCK	76353	25kb
ENSG00000157093	3	42413570	42477092	141	27	120000	-0.72993	0.76728	LYZL4	76353	25kb
ENSG00000114812	3	42505791	42604059	271	40	120000	0.53894	0.29496	VIPR1	76353	25kb
ENSG00000093183	3	42564461	42667572	264	25	120000	-0.025268	0.51008	SEC22C	76353	25kb
ENSG00000008324	3	42598332	42661606	158	16	120000	-0.35855	0.64003	SS18L2	76353	25kb
ENSG00000114857	3	42617106	42715227	233	21	120000	-0.76874	0.77898	NKTR	76353	25kb
ENSG00000114853	3	42670176	42734072	142	23	120000	-1.0247	0.84724	ZBTB47	76353	25kb
ENSG00000157119	3	42702011	42759036	118	25	120000	-1.3525	0.91188	KLHL40	76353	25kb
ENSG00000010282	3	42709155	42769319	124	23	120000	-1.2597	0.89612	HHATL	76353	25kb
ENSG00000244607	3	42709155	42839745	260	23	120000	-1.2464	0.8937	CCDC13	76353	25kb
ENSG00000181061	3	42773669	42871023	180	22	120000	-1.028	0.84801	HIGD1A	76353	25kb
ENSG00000144648	3	42821244	42954466	282	28	120000	-0.87759	0.80992	ACKR2	76353	25kb
ENSG00000273291	3	42825938	43122363	625	41	120000	-1.0513	0.85344	KRBOX1	76353	25kb
ENSG00000240747	3	42825975	43009284	349	33	120000	-0.92518	0.82256	KRBOX1	76353	25kb
ENSG00000180432	3	42872497	42942633	176	23	120000	-0.69639	0.75691	CYP8B1	76353	25kb
ENSG00000182983	3	42922223	42985825	113	19	120000	-0.67041	0.7487	ZNF662	76353	25kb
ENSG00000144649	3	42995759	43126703	303	25	120000	-0.83472	0.79806	FAM198A	76353	25kb
ENSG00000144647	3	43095724	43172568	142	21	120000	-0.66653	0.74746	POMGNT2	76353	25kb
ENSG00000163788	3	43303004	43491256	262	27	120000	0.71856	0.23621	SNRK	76353	25kb
ENSG00000160746	3	43371351	43758086	698	38	120000	0.8478	0.19827	ANO10	76353	25kb
ENSG00000011198	3	43706605	43800863	244	26	120000	-0.080476	0.53207	ABHD5	76353	25kb
ENSG00000173769	3	44258378	44398590	290	13	120000	0.020953	0.49164	TOPAZ1	76353	25kb
ENSG00000179152	3	44354611	44475943	228	23	120000	0.2713	0.39308	TCAIM	76353	25kb
ENSG00000185219	3	44456262	44544162	130	18	120000	0.062899	0.47492	ZNF445	76353	25kb
ENSG00000178917	3	44515462	44577128	74	11	120000	0.01104	0.4956	ZNF852	76353	25kb
ENSG00000196345	3	44571685	44649975	157	11	120000	-0.21763	0.58614	ZKSCAN7	76353	25kb
ENSG00000273303	3	44582051	44660665	145	11	120000	-0.31105	0.62212	RP11-944L7.5	76353	25kb
ENSG00000144792	3	44594715	44666186	112	11	120000	-0.53761	0.70458	ZNF660	76353	25kb
ENSG00000186448	3	44601380	44714963	159	15	120000	-0.44527	0.67194	ZNF197	76353	25kb
ENSG00000169981	3	44665219	44727283	65	12	120000	-0.52196	0.69915	ZNF35	76353	25kb
ENSG00000196653	3	44729135	44790323	92	16	120000	-0.50008	0.69149	ZNF502	76353	25kb
ENSG00000186446	3	44746088	44803575	82	14	120000	-0.43054	0.6666	ZNF501	76353	25kb
ENSG00000163807	3	44754153	44828154	92	13	120000	-0.42326	0.66395	KIAA1143	76353	25kb
ENSG00000163808	3	44778209	44939868	243	31	120000	-0.79686	0.78723	KIF15	76353	25kb
ENSG00000169964	3	44878361	44932162	102	19	120000	-0.5467	0.70771	TMEM42	76353	25kb
ENSG00000163810	3	44891100	44981482	212	25	120000	-0.39725	0.65441	TGM4	76353	25kb
ENSG00000163812	3	44931749	45042677	216	21	120000	-0.11717	0.54664	ZDHHC3	76353	25kb
ENSG00000075914	3	44991733	45102558	212	19	120000	0.28697	0.38707	EXOSC7	76353	25kb
ENSG00000163815	3	45018040	45102563	179	16	120000	0.32553	0.37239	CLEC3B	76353	25kb
ENSG00000163814	3	45098770	45212914	482	80	120000	0.91543	0.17998	CDCP1	76353	25kb
ENSG00000249992	3	45240958	45292770	285	21	120000	-0.25274	0.59976	TMEM158	76353	25kb
ENSG00000011376	3	45404998	45615913	545	35	120000	-0.8079	0.79042	LARS2	76353	25kb
ENSG00000144791	3	45571886	45752830	631	35	120000	-0.86496	0.80647	LIMD1	76353	25kb
ENSG00000211456	3	45705548	45811916	318	16	120000	-0.20311	0.58048	SACM1L	76353	25kb

ENSG00000163817	3	45771942	45863027	205	44	120000	-0.3496	0.63668	SLC6A20	76353	25kb
ENSG00000163818	3	45839808	45982534	313	31	120000	-0.60191	0.72638	LZTFL1	76353	25kb
ENSG00000173585	3	45902996	45969667	163	25	120000	-0.93464	0.82501	CCR9	76353	25kb
ENSG00000163820	3	45934396	46062316	367	25	120000	0.14567	0.44209	FYCO1	76353	25kb
ENSG00000172215	3	45957425	46014845	165	18	120000	0.26732	0.39461	CXCR6	76353	25kb
ENSG00000173578	3	46033516	46094234	207	15	120000	0.23639	0.40656	XCR1	76353	25kb
ENSG00000183625	3	46180096	46333197	367	19	120000	-1.1523	0.87541	CCR3	76353	25kb
ENSG00000163823	3	46218200	46274887	141	6	120000	-0.77754	0.78158	CCR1	76353	25kb
ENSG00000121807	3	46370225	46427419	168	17	120000	-0.7384	0.76987	CCR2	76353	25kb
ENSG00000160791	3	46386633	46442697	165	18	120000	-0.66875	0.74817	CCR5	76353	25kb
ENSG00000121797	3	46423654	46479488	240	20	120000	-0.95252	0.82958	CCRL2	76353	25kb
ENSG00000012223	3	46452136	46551724	365	30	120000	-1.0989	0.8641	LTF	76353	25kb
ENSG00000163825	3	46513981	46567439	119	15	120000	0.37899	0.35235	RTP3	76353	25kb
ENSG00000163827	3	46531913	46646589	412	29	120000	-0.32731	0.62828	LRRC2	76353	25kb
ENSG00000268324	3	46573888	46626178	221	21	120000	-0.49839	0.6909	LUZPP1	76353	25kb
ENSG00000241186	3	46591045	46693033	458	36	120000	0.73395	0.23149	TDGF1	76353	25kb
ENSG00000178038	3	46685487	46760194	244	37	120000	0.10814	0.45694	ALS2CL	76353	25kb
ENSG00000181585	3	46717823	46777376	186	22	120000	-0.54438	0.70691	TMIE	76353	25kb
ENSG00000206549	3	46728605	46879064	317	19	120000	-1.0518	0.85356	PRSS50	76353	25kb
ENSG00000261603	3	46736073	46802919	226	17	120000	-0.95647	0.83058	PRSS46	76353	25kb
ENSG00000188086	3	46758582	46879048	230	17	120000	-1.1727	0.87954	PRSS45	76353	25kb
ENSG00000178055	3	46846032	46900585	106	13	120000	-1.5332	0.93739	PRSS42	76353	25kb
ENSG00000160808	3	46874362	46948659	147	26	120000	-1.213	0.88744	MYL3	76353	25kb
ENSG00000160801	3	46894236	46970287	144	25	120000	-0.9405	0.82652	PTH1R	76353	25kb
ENSG00000268537	3	46902699	46955171	95	21	120000	-0.49212	0.68868	AC109583.1	76353	25kb
ENSG00000160799	3	46938216	47048500	260	18	120000	-0.70129	0.75844	CCDC12	76353	25kb
ENSG00000160796	3	46996173	47076193	188	15	120000	-0.78927	0.78502	NBEAL2	76353	25kb
ENSG00000181555	3	47032919	47230457	263	16	120000	-1.5266	0.93657	SETD2	76353	25kb
ENSG00000088727	3	47244516	47349941	132	18	120000	-1.0201	0.84615	KIF9	76353	25kb
ENSG00000114648	3	47299407	47413306	144	22	120000	-0.7687	0.77897	KLHL18	76353	25kb
ENSG00000076201	3	47397501	47479931	99	12	120000	-0.41521	0.661	PTPN23	76353	25kb
ENSG00000114650	3	47430203	47543616	145	16	120000	-0.094963	0.53783	SCAP	76353	25kb
ENSG00000163832	3	47512130	47580251	109	17	120000	-0.64974	0.74207	ELP6	76353	25kb
ENSG00000114646	3	47578729	47647282	105	13	120000	0.16085	0.43611	CSPG5	76353	25kb
ENSG00000173473	3	47601762	47848596	298	16	120000	-0.036922	0.51473	SMARCC1	76353	25kb
ENSG00000132153	3	47819399	47916685	98	14	120000	-0.43034	0.66652	DHX30	76353	25kb
ENSG00000047849	3	47867182	48155769	289	15	120000	-0.1614	0.56411	MAP4	76353	25kb
ENSG00000164045	3	48173636	48254892	105	11	120000	0.62807	0.26498	CDC25A	76353	25kb
ENSG00000164047	3	48239837	48291981	57	12	120000	0.38608	0.34972	CAMP	76353	25kb
ENSG00000164048	3	48257590	48365743	177	10	120000	0.40321	0.3434	ZNF589	76353	25kb
ENSG00000172113	3	48309754	48368175	109	5	120000	0.46506	0.32094	NME6	76353	25kb
ENSG00000229453	3	48323336	48394831	153	6	120000	0.40247	0.34367	SPINK8	76353	25kb
ENSG00000164049	3	48388709	48467666	132	12	120000	-0.067552	0.52693	FBXW12	76353	25kb
ENSG00000164050	3	48420261	48496594	101	7	120000	-0.34032	0.63319	PLXNB1	76353	25kb
ENSG00000164051	3	48448574	48506866	78	8	120000	-0.26028	0.60268	CCDC51	76353	25kb
ENSG00000232112	3	48456667	48510616	76	9	120000	-0.26023	0.60266	TMA7	76353	25kb
ENSG00000164053	3	48463114	48532115	107	15	120000	-0.27154	0.60701	ATRIP	76353	25kb
ENSG00000213689	3	48481445	48534044	80	14	120000	-0.1504	0.55978	TREX1	76353	25kb
ENSG00000164054	3	48484197	48567259	117	20	120000	0.094199	0.46248	SHISA5	76353	25kb
ENSG00000114268	3	48530117	48624448	112	17	120000	-0.39901	0.65506	PFKFB4	76353	25kb
ENSG00000145040	3	48574160	48626206	63	13	120000	-1.0427	0.85145	UCN2	76353	25kb
ENSG00000114270	3	48576506	48657700	80	16	120000	-1.1408	0.87302	COL7A1	76353	25kb
ENSG00000010256	3	48611435	48673409	52	12	120000	-0.75756	0.77564	UQCRC1	76353	25kb
ENSG00000183396	3	48633192	48684288	46	7	120000	-0.17042	0.56766	TMEM89	76353	25kb
ENSG00000225697	3	48638156	48697926	56	7	120000	0.082138	0.46727	SLC26A6	76353	25kb
ENSG00000008300	3	48648902	48725348	82	7	120000	0.32309	0.37331	CELSR3	76353	25kb
ENSG00000213672	3	48676364	48748797	91	8	120000	0.4891	0.31239	NCKIPSD	76353	25kb
ENSG00000068745	3	48700436	48802786	132	14	120000	0.30316	0.38088	IP6K2	76353	25kb
ENSG00000114302	3	48757030	48910279	130	17	120000	-0.21814	0.58634	PRKAR2A	76353	25kb
ENSG00000178537	3	48869369	48961426	89	11	120000	-0.07556	0.53012	SLC25A20	76353	25kb
ENSG00000221883	3	48930221	48981818	63	11	120000	-0.05024	0.52003	ARIH2OS	76353	25kb
ENSG00000177479	3	48931254	49048815	120	15	120000	-0.051893	0.52069	ARIH2	76353	25kb
ENSG00000178467	3	49002319	49069587	48	10	120000	-0.14381	0.55718	P4HTM	76353	25kb
ENSG00000178252	3	49019495	49078386	41	8	120000	-0.30426	0.61953	WDR6	76353	25kb
ENSG00000178149	3	49027921	49084726	41	8	120000	-0.31067	0.62197	DALRD3	76353	25kb
ENSG00000178057	3	49032892	49085928	37	8	120000	-0.31567	0.62387	NDUFAF3	76353	25kb
ENSG00000178035	3	49036758	49091841	41	10	120000	-0.44087	0.67035	IMPDH2	76353	25kb
ENSG00000198218	3	49042140	49156796	93	14	120000	-0.21736	0.58604	QRICH1	76353	25kb
ENSG00000172053	3	49108365	49167553	56	12	120000	0.0023289	0.49907	QARS	76353	25kb
ENSG00000172046	3	49120479	49183371	58	13	120000	0.0011566	0.49954	USP19	76353	25kb
ENSG00000172037	3	49133547	49195551	64	12	120000	0.084472	0.46634	LAMB2	76353	25kb
ENSG00000177352	3	49174968	49228754	71	11	120000	0.51977	0.30161	CCDC71	76353	25kb
ENSG00000185909	3	49184044	49238917	72	10	120000	0.59057	0.2774	KLHDC8B	76353	25kb
ENSG00000236980	3	49190065	49254291	84	11	120000	0.59969	0.27436	C3orf84	76353	25kb
ENSG00000173421	3	49210861	49320537	141	15	120000	0.49101	0.31171	CCDC36	76353	25kb
ENSG00000225399	3	49272518	49323744	65	11	120000	0.50357	0.30728	RP11-3B7.1	76353	25kb
ENSG00000188315	3	49281035	49340342	70	12	120000	0.60397	0.27293	C3orf62	76353	25kb
ENSG00000114316	3	49290264	49403145	137	17	120000	0.25021	0.40121	USP4	76353	25kb
ENSG00000233276	3	49369609	49421033	75	11	120000	-0.27105	0.60683	GPX1	76353	25kb
ENSG00000067560	3	49371578	49475431	189	19	120000	-0.21599	0.5855	RHOA	76353	25kb
ENSG00000145022	3	49424639	49478908	116	15	120000	-0.09718	0.53871	TCTA	76353	25kb
ENSG00000145020	3	49429211	49485186	118	15	120000	-0.10323	0.54111	AMT	76353	25kb
ENSG00000145029	3	49435379	49491759	114	16	120000	-0.12225	0.54865	NICN1	76353	25kb
ENSG00000173402	3	49481146	49598048	202	21	120000	-0.41522	0.66101	DAG1	76353	25kb
ENSG00000164061	3	49566922	49733978	221	21	120000	-0.056217	0.52242	BSN	76353	25kb
ENSG00000164062	3	49686435	49746396	80	13	120000	0.26599	0.39512	APEH	76353	25kb
ENSG00000173531	3	49696380	49751934	80	13	120000	0.49733	0.30948	MST1	76353	25kb
ENSG00000164068	3	49701932	49783962	112	15	120000	0.8184	0.20656	RNF123	76353	25kb
ENSG00000176020	3	49729267	49786349	83	11	120000	1.0248	0.15274	AMIGO3	76353	25kb
ENSG00000173540	3	49729277	49786384	83	11	120000	1.0248	0.15274	GMPPB	76353	25kb
ENSG00000176095	3	49736727	49848975	167	16	120000	1.2854	0.099334	IP6K1	76353	25kb
ENSG00000187492	3	49803165	49862268	90	14	120000	1.6416	0.050332	CDHR4	76353	25kb
ENSG00000185614	3	49815687	49867463	66	12	120000	1.6711	0.04735	FAM212A	76353	25kb
ENSG00000182179	3	49817640	49876379	80	13	120000	1.6176	0.052875	UBA7	76353	25kb
ENSG00000183763	3	49841034	49919007	99	14	120000	1.3335	0.091176	TRAIP	76353	25kb
ENSG00000164076	3	49870421	49932655	78	12	120000	0.97546	0.16467	CAMKV	76353	25kb

ENSG00000164078	3	49899435	49966299	81	10	120000	-0.094631	0.5377	MST1R	76353	25kb
ENSG00000228008	3	49916278	49979370	74	10	120000	-0.094507	0.53765	CTD-2330K9.3	76353	25kb
ENSG00000164077	3	49921302	49992606	89	11	120000	-0.072567	0.52892	MON1A	76353	25kb
ENSG00000004534	3	49952440	50162478	311	13	120000	-8.519e-05	0.50003	RBM6	76353	25kb
ENSG00000003756	3	50101341	50181454	110	14	120000	-0.26397	0.6041	RBM5	76353	25kb
ENSG00000001617	3	50167478	50251508	133	18	120000	-0.061084	0.52435	SEMA3F	76353	25kb
ENSG00000114349	3	50204045	50258949	82	12	120000	-0.18017	0.57149	GNAT1	76353	25kb
ENSG00000114353	3	50238724	50321787	94	21	120000	-0.03717	0.51483	GNAI2	76353	25kb
ENSG00000179564	3	50291458	50350545	55	13	120000	0.26104	0.39703	LSMEM2	76353	25kb
ENSG00000214706	3	50300163	50355349	48	13	120000	0.49001	0.31206	IFRD2	76353	25kb
ENSG00000186792	3	50305262	50361899	43	12	120000	0.35627	0.36082	HYAL3	76353	25kb
ENSG00000243477	3	50308833	50361852	38	11	120000	0.28614	0.38738	NAT6	76353	25kb
ENSG00000114378	3	50312320	50374812	45	11	120000	0.18523	0.42652	HYAL1	76353	25kb
ENSG00000068001	3	50330221	50385337	40	10	120000	0.21415	0.41522	HYAL2	76353	25kb
ENSG00000114383	3	50332458	50390682	42	11	120000	0.073687	0.47063	TUSC2	76353	25kb
ENSG00000068028	3	50342219	50403411	36	9	120000	0.27587	0.39132	RASSF1	76353	25kb
ENSG00000004838	3	50353541	50409283	34	8	120000	0.46034	0.32263	ZMYND10	76353	25kb
ENSG00000114388	3	50359761	50413522	35	7	120000	0.31929	0.37475	NPRL2	76353	25kb
ENSG00000114395	3	50363126	50420891	42	9	120000	0.58196	0.2803	CYB561D2	76353	25kb
ENSG00000272104	3	50363323	50430354	58	14	120000	0.36628	0.35708	XXcos-LUCA11.5	76353	25kb
ENSG00000126062	3	50367180	50422041	42	8	120000	0.58692	0.27863	TMEM115	76353	25kb
ENSG00000007402	3	50375233	50566675	307	20	120000	-0.27197	0.60718	CACNA2D2	76353	25kb
ENSG00000088543	3	50570462	50633458	86	10	120000	-0.43537	0.66835	C3orf18	76353	25kb
ENSG00000114735	3	50581583	50647366	93	11	120000	-0.36629	0.64292	HEMK1	76353	25kb
ENSG00000114737	3	50618921	50674262	82	11	120000	-0.51082	0.69526	CISH	76353	25kb
ENSG00000114738	3	50623951	50711720	160	16	120000	-0.21769	0.58616	MAPKAPK3	76353	25kb
ENSG00000088538	3	50687672	51446629	1306	19	120000	0.23103	0.40864	DOCK3	76353	25kb
ENSG00000145050	3	51397478	51451828	61	9	120000	-0.94764	0.82834	MANF	76353	25kb
ENSG00000179837	3	51403731	51460330	65	9	120000	-0.82445	0.79516	RBM15B	76353	25kb
ENSG00000145041	3	51408298	51559010	185	16	120000	-0.93461	0.82501	VPRBP	76353	25kb
ENSG00000164080	3	51550160	51727683	163	26	120000	-0.9488	0.82864	RAD54L2	76353	25kb
ENSG00000164081	3	51671709	51763339	87	26	120000	-0.89117	0.81358	TEX264	76353	25kb
ENSG00000164082	3	51716086	51777629	77	19	120000	-1.065	0.85655	GRM2	76353	25kb
ENSG00000214686	3	51787580	51838009	43	12	120000	-0.98105	0.83672	IQCF6	76353	25kb
ENSG00000229972	3	51826620	51889876	111	9	120000	-0.2339	0.59247	IQCF3	76353	25kb
ENSG00000184345	3	51870645	51922440	133	12	120000	0.30773	0.37914	IQCF2	76353	25kb
ENSG00000214681	3	51882737	51934600	122	15	120000	0.27556	0.39144	IQCF5	76353	25kb
ENSG00000173389	3	51903892	51962351	120	21	120000	-0.21024	0.58326	IQCF1	76353	25kb
ENSG00000114767	3	51942446	52000957	90	23	120000	0.10777	0.45709	RRP9	76353	25kb
ENSG00000041880	3	51951361	52007883	74	18	120000	0.46709	0.32022	PARP3	76353	25kb
ENSG00000180929	3	51964330	52016509	64	14	120000	0.25677	0.39868	GPR62	76353	25kb
ENSG00000090097	3	51966470	52027426	78	17	120000	0.4137	0.33955	PCBP4	76353	25kb
ENSG00000272762	3	51969317	52033032	78	19	120000	0.28043	0.38958	RP11-155D18.14	76353	25kb
ENSG00000114779	3	51977526	52042425	77	21	120000	0.41024	0.34081	ABHD14B	76353	25kb
ENSG00000248487	3	51980442	52040212	68	19	120000	0.5314	0.29757	ABHD14A	76353	25kb
ENSG00000114786	3	51984066	52048199	77	21	120000	0.17155	0.43189	ABHD14A-ACY1	76353	25kb
ENSG00000243989	3	51984066	52048213	77	21	120000	0.17155	0.43189	ACY1	76353	25kb
ENSG00000162244	3	52002616	52054958	69	18	120000	-0.26167	0.60321	RPL29	76353	25kb
ENSG00000164086	3	52057935	52115566	89	16	120000	0.029572	0.4882	DUSP7	76353	25kb
ENSG00000256097	3	52072076	52122567	66	12	120000	-0.28506	0.6122	LINC00696	76353	25kb
ENSG00000164087	3	52084269	52213706	178	18	120000	-0.50786	0.69422	POC1A	76353	25kb
ENSG00000023330	3	52207102	52273343	101	21	120000	-1.9307	0.97324	ALAS1	76353	25kb
ENSG00000239732	3	52230096	52298183	90	14	120000	-1.9515	0.9745	TLR9	76353	25kb
ENSG00000173366	3	52230097	52290206	76	14	120000	-1.9869	0.97654	TLR9	76353	25kb
ENSG00000247596	3	52237626	52298276	82	14	120000	-1.922	0.9727	TWF2	76353	25kb
ENSG00000164088	3	52254841	52309613	86	13	120000	-1.7896	0.96324	PPM1M	76353	25kb
ENSG00000164091	3	52263437	52347036	144	16	120000	-1.93	0.9732	WDR82	76353	25kb
ENSG00000168237	3	52296105	52354272	99	13	120000	-1.9107	0.97198	GLYCTK	76353	25kb
ENSG00000114841	3	52325335	52459507	168	15	120000	-1.665	0.95204	DNAH1	76353	25kb
ENSG00000163930	3	52410029	52469366	58	11	120000	-1.9792	0.9761	BAP1	76353	25kb
ENSG00000010318	3	52419673	52482657	58	12	120000	-2.0497	0.9798	PHF7	76353	25kb
ENSG00000010319	3	52442069	52504101	88	13	120000	-1.5996	0.94515	SEMA3G	76353	25kb
ENSG00000114854	3	52460118	52513086	91	10	120000	-1.378	0.91589	TNNC1	76353	25kb
ENSG00000010322	3	52464134	52552087	138	14	120000	-1.1967	0.8843	NISCH	76353	25kb
ENSG00000010327	3	52504354	52583511	135	19	120000	-1.0331	0.84923	STAB1	76353	25kb
ENSG00000168268	3	52533386	52594070	111	16	120000	-0.8088	0.79068	NT5DC2	76353	25kb
ENSG00000168273	3	52543029	52638253	213	15	120000	-0.46684	0.67969	SMIM4	76353	25kb
ENSG00000163939	3	52554368	52744933	407	14	120000	-0.27712	0.60916	PBRM1	76353	25kb
ENSG00000163938	3	52690172	52753508	125	10	120000	-0.14409	0.55729	GNL3	76353	25kb
ENSG00000016864	3	52703505	52765048	118	10	120000	-0.20011	0.5793	GLT8D1	76353	25kb
ENSG00000114902	3	52713971	52767182	102	10	120000	-0.2218	0.58776	SPCS1	76353	25kb
ENSG00000114904	3	52719800	52829965	213	13	120000	-0.32172	0.62617	NEK4	76353	25kb
ENSG00000055957	3	52786603	52851078	114	12	120000	-0.37042	0.64447	ITIH1	76353	25kb
ENSG00000162267	3	52803784	52868025	137	15	120000	0.16158	0.43582	ITIH3	76353	25kb
ENSG00000055955	3	52821991	52890495	200	18	120000	0.21614	0.41444	ITIH4	76353	25kb
ENSG00000243696	3	52822298	52894745	210	19	120000	0.23209	0.40824	RP5-966M1.6	76353	25kb
ENSG00000272573	3	52842130	52894235	169	15	120000	0.36368	0.35805	MUSTN1	76353	25kb
ENSG00000248592	3	52842137	52956578	279	14	120000	0.71467	0.23741	TMEM110-MUSTN1	76353	25kb
ENSG00000213533	3	52845235	52956612	274	14	120000	0.73735	0.23045	TMEM110	76353	25kb
ENSG00000163935	3	52912588	53105766	428	23	120000	0.40296	0.34349	SFMBT1	76353	25kb
ENSG00000272305	3	52978135	53158469	432	24	120000	0.23949	0.40536	RP11-894J14.5	76353	25kb
ENSG00000163933	3	53097499	53189478	227	18	120000	0.89082	0.18651	RFT1	76353	25kb
ENSG00000163932	3	53165025	53251733	221	33	120000	0.82149	0.20568	PRKCD	76353	25kb
ENSG00000163931	3	53233723	53315068	300	20	120000	0.99542	0.15977	TKT	76353	25kb
ENSG00000162290	3	53292447	53406654	176	20	120000	0.89084	0.18651	DCP1A	76353	25kb
ENSG00000157388	3	53503683	53872760	880	88	120000	0.22678	0.4103	CACNA1D	76353	25kb
ENSG00000016391	3	53821362	53905417	250	47	120000	-1.7129	0.95664	CHDH	76353	25kb
ENSG00000056736	3	53855607	53924827	215	33	120000	-1.1531	0.87556	IL17RB	76353	25kb
ENSG00000113812	3	53876093	53941229	204	22	120000	-0.66515	0.74702	ACTR8	76353	25kb
ENSG00000113811	3	53893437	53951015	167	17	120000	-0.45292	0.6747	SELK	76353	25kb
ENSG00000157445	3	54131574	55133584	2937	212	120000	-2.1877	0.98565	CACNA2D3	76353	25kb
ENSG00000144771	3	54927264	55026115	289	24	120000	-0.48667	0.68675	LRTM1	76353	25kb
ENSG00000114251	3	55474743	55548973	220	36	120000	-0.59768	0.72497	WNT5A	76353	25kb
ENSG00000187672	3	55517336	56527391	2970	115	120000	-1.8877	0.97047	ERC2	76353	25kb
ENSG00000180376	3	56566189	56680846	458	28	120000	-0.56063	0.71247	CCDC66	76353	25kb
ENSG00000163946	3	56629161	56742265	436	24	120000	-0.63187	0.73626	FAM208A	76353	25kb

ENSG00000163947	3	56736446	57138357	1267	108	120000	-0.97347	0.83484	ARHGEF3	76353	25kb
ENSG00000186451	3	57069469	57134460	201	32	120000	-0.1446	0.55749	SPATA12	76353	25kb
ENSG00000144730	3	57099010	57229334	314	25	120000	-0.22436	0.58876	IL17RD	76353	25kb
ENSG00000163666	3	57206944	57285549	102	14	120000	-0.092973	0.53704	HESX1	76353	25kb
ENSG00000157500	3	57236765	57332496	144	18	120000	0.20354	0.41936	APPL1	76353	25kb
ENSG00000239388	3	57277375	57351710	133	16	120000	0.20477	0.41888	ASB14	76353	25kb
ENSG00000174844	3	57302727	57555071	751	30	120000	-0.15369	0.56107	DNAH12	76353	25kb
ENSG00000174840	3	57517003	57577571	149	17	120000	0.51229	0.30422	PDE12	76353	25kb
ENSG00000168374	3	57532090	57608947	181	18	120000	0.43273	0.3326	ARF4	76353	25kb
ENSG00000174839	3	57586184	57703816	270	18	120000	0.97465	0.16487	DENND6A	76353	25kb
ENSG00000163681	3	57716177	57939895	323	21	120000	1.2144	0.11229	SLMAP	76353	25kb
ENSG00000136068	3	57969127	58182982	528	37	120000	-1.2341	0.89141	FLNB	76353	25kb
ENSG00000163687	3	58152984	58225424	229	31	120000	-1.1641	0.87781	DNASE1L3	76353	25kb
ENSG00000163686	3	58198233	58306420	380	24	120000	-0.84053	0.79969	ABHD6	76353	25kb
ENSG00000163684	3	58266974	58335422	289	14	120000	-1.4813	0.93074	RPP14	76353	25kb
ENSG00000255154	3	58278341	58328847	226	14	120000	-1.4195	0.92212	RPP14	76353	25kb
ENSG00000168297	3	58293607	58436748	574	18	120000	-1.6032	0.94555	PXK	76353	25kb
ENSG00000168291	3	58388357	58444584	234	18	120000	-1.557	0.94026	PDHB	76353	25kb
ENSG00000168301	3	58452841	58513087	116	25	120000	-1.2572	0.89566	KCTD6	76353	25kb
ENSG00000168306	3	58465863	58548046	172	33	120000	-1.0568	0.85469	ACOX2	76353	25kb
ENSG00000168309	3	58524844	58638337	433	51	120000	1.2002	0.11504	FAM107A	76353	25kb
ENSG00000198643	3	58594673	58677575	363	38	120000	2.6045	0.0046002	FAM3D	76353	25kb
ENSG00000163689	3	58678092	59060810	494	31	120000	-0.096814	0.53856	C3orf67	76353	25kb
ENSG00000189283	3	59710036	61262133	6537	372	120000	-1.1412	0.87311	FHIT	76353	25kb
ENSG00000269415	3	59932215	59982583	310	25	120000	0.49577	0.31003	NPCDR1	76353	25kb
ENSG00000144724	3	61522243	62308288	2392	179	120000	-2.4109	0.99204	PTPRG	76353	25kb
ENSG00000114405	3	62279648	62346888	86	22	120000	-0.68828	0.75436	C3orf14	76353	25kb
ENSG00000153266	3	62330356	62384999	83	22	120000	-0.09516	0.53791	FEZF2	76353	25kb
ENSG00000163618	3	62359022	62886054	1965	118	120000	1.2235	0.11057	CADPS	76353	25kb
ENSG00000163630	3	63188991	63627597	1657	120	120000	-0.11064	0.54405	SYNPR	76353	25kb
ENSG00000188817	3	63613344	63689696	281	33	120000	-0.28518	0.61225	SNTN	76353	25kb
ENSG00000163632	3	63780038	63859312	140	22	120000	2.0345	0.020952	C3orf49	76353	25kb
ENSG00000163634	3	63794546	63874579	133	23	120000	2.0358	0.020886	THOC7	76353	25kb
ENSG00000163635	3	63825233	64014138	350	31	120000	0.8687	0.1925	ATXN7	76353	25kb
ENSG00000163636	3	63971225	64034658	153	27	120000	0.68681	0.2461	PSMD6	76353	25kb
ENSG00000163637	3	64054543	64456152	1289	109	120000	0.78737	0.21553	PRICKLE2	76353	25kb
ENSG00000163638	3	64476333	64698676	630	70	120000	0.52181	0.3009	ADAMTS9	76353	25kb
ENSG00000151276	3	65314200	66049509	2493	194	120000	-1.534	0.93749	MAGI1	76353	25kb
ENSG00000144741	3	66094285	66463540	844	53	120000	-1.1446	0.87381	SLC25A26	76353	25kb
ENSG00000144749	3	66404221	66576687	447	47	120000	-0.35518	0.63877	LRIG1	76353	25kb
ENSG00000163376	3	67023727	67086634	111	20	120000	0.95557	0.16965	KBTBD8	76353	25kb
ENSG00000172340	3	67385884	67730038	1272	66	120000	-0.62341	0.73349	SUCLG2	76353	25kb
ENSG00000183662	3	68028359	68619776	1967	95	120000	-2.4785	0.9934	FAM19A1	76353	25kb
ENSG00000163377	3	68755917	69006761	708	31	120000	1.1074	0.13405	FAM19A4	76353	25kb
ENSG00000163378	3	68999365	69088112	224	26	120000	0.35586	0.36097	EOGT	76353	25kb
ENSG00000144747	3	69043978	69126484	157	17	120000	-1.2318	0.89099	TMF1	76353	25kb
ENSG00000144744	3	69078881	69154559	163	22	120000	-0.61349	0.73022	UBA3	76353	25kb
ENSG00000144746	3	69109095	69180217	169	24	120000	-0.092358	0.53679	ARL6IP5	76353	25kb
ENSG00000163380	3	69131023	69197183	175	25	120000	0.47186	0.31851	LMOD3	76353	25kb
ENSG00000114541	3	69194141	69616734	1443	105	120000	0.91697	0.17958	FRMD4B	76353	25kb
ENSG00000187098	3	69763586	70042488	646	36	120000	-0.14521	0.55773	MITF	76353	25kb
ENSG00000114861	3	70978844	71658140	1385	124	120000	-2.0811	0.98129	FOXP1	76353	25kb
ENSG00000163412	3	71699567	71828924	384	50	120000	1.4227	0.077416	EIF4E3	76353	25kb
ENSG00000170837	3	71778201	71830647	142	21	120000	0.82135	0.20572	GPR27	76353	25kb
ENSG00000163421	3	71795807	71859357	181	34	120000	-0.36603	0.64283	PROK2	76353	25kb
ENSG00000163602	3	72395976	72521069	222	38	120000	-1.006	0.8428	RYBP	76353	25kb
ENSG00000144736	3	72773428	72936065	249	16	120000	-0.10144	0.5404	SHQ1	76353	25kb
ENSG00000172986	3	72912224	73072289	460	34	120000	-0.30826	0.62106	GXYLT2	76353	25kb
ENSG00000163605	3	73020936	73143350	394	18	120000	-0.62696	0.73466	PPP4R2	76353	25kb
ENSG00000255423	3	73085810	73137488	140	12	120000	-0.44355	0.67132	EBLN2	76353	25kb
ENSG00000121440	3	73406584	73699091	757	79	120000	-0.83895	0.79925	PDZRN3	76353	25kb
ENSG00000113805	3	74286719	74595291	768	76	120000	-0.63135	0.7361	CNTN3	76353	25kb
ENSG00000227124	3	75733794	75859734	175	19	120000	-0.82048	0.79403	ZNF717	76353	25kb
ENSG00000185008	3	75930846	77724115	5275	243	120000	0.39871	0.34505	ROBO2	76353	25kb
ENSG00000169855	3	78621390	79841965	2857	108	120000	-0.80566	0.78978	ROBO1	76353	25kb
ENSG00000114480	3	81513850	81836312	575	30	120000	0.84263	0.19972	GBE1	76353	25kb
ENSG00000175161	3	84983132	86148579	2979	67	120000	1.0163	0.15474	CADM2	76353	25kb
ENSG00000206538	3	86962119	87065269	189	21	120000	-0.62426	0.73377	VGLL3	76353	25kb
ENSG00000083937	3	87251421	87329698	201	25	120000	-1.2074	0.88636	CHMP2B	76353	25kb
ENSG00000064835	3	87283554	87350737	212	19	120000	-0.9276	0.82319	POU1F1	76353	25kb
ENSG00000179097	3	88014846	88067919	145	16	120000	-0.076496	0.53049	HTR1F	76353	25kb
ENSG00000163320	3	88076094	88224035	382	18	120000	-0.64364	0.7401	CGGBP1	76353	25kb
ENSG00000229729	3	88083424	88213123	330	15	120000	-0.54962	0.70871	RP11-159G9.5	76353	25kb
ENSG00000175105	3	88163254	88218815	111	7	120000	-0.32	0.62552	ZNF654	76353	25kb
ENSG00000179021	3	88173893	88242879	152	8	120000	-0.25178	0.59939	C3orf38	76353	25kb
ENSG00000044524	3	89131674	89556284	832	28	120000	0.6192	0.26789	EPHA3	76353	25kb
ENSG00000184500	3	93566881	93717910	139	25	120000	-0.38389	0.64947	PROS1	76353	25kb
ENSG00000169379	3	93673983	93799512	97	18	120000	-1.0205	0.84625	ARL13B	76353	25kb
ENSG00000178750	3	93708213	93772454	51	12	120000	-1.2454	0.8935	STX19	76353	25kb
ENSG00000178700	3	93741680	93807233	48	15	120000	-1.0313	0.84881	DHFRL1	76353	25kb
ENSG00000178694	3	93756760	93872389	101	24	120000	-0.90522	0.81733	NSUN3	76353	25kb
ENSG00000269028	3	96310981	96362000	189	14	120000	0.33135	0.37019	MTRNR2L12	76353	25kb
ENSG00000080224	3	96508425	97496304	1360	72	120000	-0.44011	0.67007	EPHA6	76353	25kb
ENSG00000113966	3	97458365	97544953	146	18	120000	-0.13756	0.55471	ARL6	76353	25kb
ENSG00000233280	3	97515884	97619132	135	13	120000	0.99375	0.16017	CRYBG3	76353	25kb
ENSG00000080200	3	97570819	97688810	151	15	120000	1.1912	0.11678	CRYBG3	76353	25kb
ENSG00000170854	3	97635662	97716301	129	20	120000	1.4443	0.074334	MINA	76353	25kb
ENSG00000196578	3	97781017	97831946	304	13	120000	1.9892	0.02334	OR5AC2	76353	25kb
ENSG00000231192	3	97826542	97877483	203	6	120000	2.1054	0.017627	OR5H1	76353	25kb
ENSG00000236032	3	97843170	97894249	196	8	120000	2.0215	0.021614	OR5H14	76353	25kb
ENSG00000233412	3	97862544	97913485	286	6	120000	1.8853	0.029696	OR5H15	76353	25kb
ENSG00000230301	3	97958088	98009200	298	14	120000	-0.60092	0.72605	OR5H6	76353	25kb
ENSG00000197938	3	97976732	98027676	266	13	120000	-0.8664	0.80687	OR5H2	76353	25kb
ENSG00000196098	3	98047698	98098663	128	17	120000	-0.53323	0.70306	OR5K4	76353	25kb
ENSG00000206536	3	98084510	98135475	178	15	120000	1.4554	0.072774	OR5K3	76353	25kb
ENSG00000232382	3	98163324	98214420	131	16	120000	-0.27118	0.60688	OR5K1	76353	25kb

ENSG00000231861	3	98191448	98242496	117	23	120000	-0.75905	0.77609	OR5K2	76353	25kb
ENSG00000080822	3	98191756	98266910	175	34	120000	-0.85707	0.8043	CLDND1	76353	25kb
ENSG00000080819	3	98214976	98337567	321	42	120000	0.33444	0.36903	CPOX	76353	25kb
ENSG00000154165	3	98225743	98276960	111	24	120000	-0.78667	0.78426	GPR15	76353	25kb
ENSG00000064225	3	98426080	98565045	356	29	120000	0.1091	0.45656	ST3GAL6	76353	25kb
ENSG00000057019	3	98489785	98645533	438	25	120000	0.3115	0.37771	DCBLD2	76353	25kb
ENSG00000144810	3	99332319	99543070	488	42	120000	0.37431	0.35409	COL8A1	76353	25kb
ENSG00000184220	3	99511678	99922447	515	23	120000	-0.36365	0.64194	CMSS1	76353	25kb
ENSG00000168386	3	99523985	99858357	416	23	120000	-0.26941	0.60619	FILIP1L	76353	25kb
ENSG00000235156	3	99879531	99938035	83	17	120000	-0.93006	0.82383	TMEM30C	76353	25kb
ENSG00000036054	3	99954844	100069095	217	24	120000	0.16507	0.43445	TBC1D23	76353	25kb
ENSG00000114021	3	100028545	100100710	155	27	120000	0.83466	0.20196	NIT2	76353	25kb
ENSG00000154174	3	100057275	100145242	177	24	120000	0.46427	0.32123	TOMM70A	76353	25kb
ENSG00000206535	3	100095037	100200163	175	29	120000	-0.11192	0.54456	LNP1	76353	25kb
ENSG00000181458	3	100186463	100321288	363	20	120000	-0.21758	0.58612	TMEM45A	76353	25kb
ENSG00000144820	3	100303433	100439323	346	23	120000	1.4084	0.079503	GPR128	76353	25kb
ENSG00000114354	3	100403205	100492810	155	15	120000	2.4179	0.0078061	TFG	76353	25kb
ENSG00000154175	3	100443000	100737359	812	52	120000	0.39545	0.34626	ABI3BP	76353	25kb
ENSG00000081148	3	100916390	101064404	283	17	120000	-0.62587	0.7343	IMPG2	76353	25kb
ENSG00000138468	3	101018049	101257085	618	16	120000	-0.71807	0.76364	SENP7	76353	25kb
ENSG00000174173	3	101255706	101310290	138	17	120000	-1.6607	0.95161	TRMT10C	76353	25kb
ENSG00000081154	3	101267939	101338281	174	18	120000	-1.578	0.94272	PCNP	76353	25kb
ENSG00000066422	3	101342733	101421339	130	15	120000	-0.36083	0.64089	ZBTB11	76353	25kb
ENSG00000114391	3	101374935	101430626	83	12	120000	-0.46126	0.6777	RPL24	76353	25kb
ENSG00000182504	3	101417769	101514406	137	15	120000	-0.67499	0.75016	CEP97	76353	25kb
ENSG00000144815	3	101473046	101572073	134	17	120000	-1.0724	0.85823	NXPE3	76353	25kb
ENSG00000144802	3	101521835	101604866	115	20	120000	-0.65592	0.74406	NFKBIZ	76353	25kb
ENSG00000170044	3	101793088	102223685	1014	60	120000	-0.342	0.63382	ZPLD1	76353	25kb
ENSG00000170017	3	105060753	105320744	804	39	120000	-0.38952	0.65155	ALCAM	76353	25kb
ENSG00000114423	3	105349305	105613396	721	36	120000	-0.21743	0.58606	CBLB	76353	25kb
ENSG00000138483	3	107071188	107122481	161	27	120000	-0.38782	0.65092	CCDC54	76353	25kb
ENSG00000114439	3	107216783	107555171	653	38	120000	0.14192	0.44357	BBX	76353	25kb
ENSG00000196776	3	107737145	107834872	224	36	120000	-0.98785	0.83839	CD47	76353	25kb
ENSG00000114446	3	107854659	107966417	454	11	120000	1.4726	0.070425	IFT57	76353	25kb
ENSG00000114455	3	107990376	108122132	452	31	120000	-0.55861	0.71179	HHLA2	76353	25kb
ENSG00000144821	3	108074216	108273169	475	44	120000	-1.1596	0.87689	MYH15	76353	25kb
ENSG00000163507	3	108243716	108333491	129	18	120000	-0.92737	0.82313	KIAA1524	76353	25kb
ENSG00000198919	3	108283529	108438693	202	21	120000	-0.8111	0.79135	DZIP3	76353	25kb
ENSG00000163515	3	108437271	108501231	169	22	120000	-0.01715	0.50684	RETNLB	76353	25kb
ENSG00000163519	3	108516545	108598852	242	36	120000	0.49816	0.30919	TRAT1	76353	25kb
ENSG00000138472	3	108601639	108697742	369	35	120000	-2.2109	0.98648	GUCA1C	76353	25kb
ENSG00000114487	3	108652086	108861989	647	21	120000	-1.7358	0.9587	MORC1	76353	25kb
ENSG00000163530	3	108987635	109060364	236	31	120000	0.95764	0.16912	DPPA2	76353	25kb
ENSG00000121570	3	109019988	109081419	180	23	120000	0.49488	0.31034	DPPA4	76353	25kb
ENSG00000206532	3	110582231	110637323	133	18	120000	0.32844	0.37129	RP11-553A10.1	76353	25kb
ENSG00000177707	3	110763918	111019410	595	43	120000	-0.25698	0.6014	PVRL3	76353	25kb
ENSG00000153283	3	110986566	111409597	1185	65	120000	0.90309	0.18324	CD96	76353	25kb
ENSG00000177494	3	111286747	111339290	96	21	120000	0.66724	0.25231	ZBED2	76353	25kb
ENSG00000240891	3	111368523	111590294	706	52	120000	-0.7668	0.7784	PLCXD2	76353	25kb
ENSG00000144824	3	111426344	111720364	1068	59	120000	-0.6812	0.75213	PHLDB2	76353	25kb
ENSG00000144827	3	111672857	111737210	282	28	120000	-1.0452	0.85202	ABHD10	76353	25kb
ENSG00000144834	3	111692511	111757734	344	25	120000	-0.85587	0.80396	TAGLN3	76353	25kb
ENSG00000176040	3	111728690	111825116	384	30	120000	-0.68909	0.75462	TMPRSS7	76353	25kb
ENSG00000114529	3	111780182	111874851	241	27	120000	-0.61619	0.73111	C3orf52	76353	25kb
ENSG00000174500	3	111814688	111877152	182	19	120000	-0.77793	0.7817	GCSAM	76353	25kb
ENSG00000172139	3	111834734	112038105	793	24	120000	-0.52279	0.69944	SLC9C1	76353	25kb
ENSG00000091972	3	112026194	112106659	263	21	120000	-0.57307	0.7167	CD200	76353	25kb
ENSG00000186265	3	112157815	112243408	186	25	120000	0.43881	0.3304	BTLA	76353	25kb
ENSG00000144848	3	112226356	112305893	249	16	120000	0.37832	0.3526	ATG3	76353	25kb
ENSG00000138459	3	112255556	112329424	222	15	120000	0.47891	0.316	SLC35A5	76353	25kb
ENSG00000091986	3	112298407	112393377	248	39	120000	0.73265	0.23189	CCDC80	76353	25kb
ENSG00000206531	3	112509556	112590703	322	23	120000	0.44316	0.32882	CD200R1L	76353	25kb
ENSG00000163606	3	112615056	112718969	379	33	120000	0.056773	0.47736	CD200R1	76353	25kb
ENSG00000163607	3	112684765	112758907	143	28	120000	0.73003	0.23269	GTPBP8	76353	25kb
ENSG00000163608	3	112696287	112763708	115	21	120000	0.91137	0.18105	C3orf17	76353	25kb
ENSG00000144857	3	112904850	113031303	472	61	120000	0.24744	0.40228	BOC	76353	25kb
ENSG00000206530	3	112980777	113185457	709	29	120000	-0.042578	0.51698	WDR52	76353	25kb
ENSG00000163611	3	113136565	113259034	427	21	120000	0.85075	0.19745	SPICE1	76353	25kb
ENSG00000072858	3	113226143	113373425	432	38	120000	0.094627	0.46231	SIDT1	76353	25kb
ENSG00000176542	3	113342232	113440493	116	11	120000	0.15253	0.43939	KIAA2018	76353	25kb
ENSG00000121579	3	113410307	113490147	101	8	120000	-0.19298	0.57651	NAA50	76353	25kb
ENSG00000114573	3	113440866	113555903	156	11	120000	-0.40809	0.6584	ATP6V1A	76353	25kb
ENSG00000178075	3	113522029	113691021	330	46	120000	-0.042679	0.51702	GRAMD1C	76353	25kb
ENSG00000184307	3	113641748	113709248	113	30	120000	-0.82186	0.79442	ZDHHC23	76353	25kb
ENSG00000163617	3	113657984	113800460	215	28	120000	-0.73727	0.76952	KIAA1407	76353	25kb
ENSG00000151576	3	113699680	113832269	231	26	120000	-1.0216	0.84652	QTRTD1	76353	25kb
ENSG00000151577	3	113822499	113943254	330	32	120000	-1.8541	0.96814	DRD3	76353	25kb
ENSG00000174255	3	113928483	113981425	167	34	120000	-0.81599	0.79275	ZNF80	76353	25kb
ENSG00000181847	3	113970760	114054135	225	40	120000	0.29401	0.38438	TIGIT	76353	25kb
ENSG00000181722	3	114031941	114891118	1643	105	120000	-0.54425	0.70687	ZBTB20	76353	25kb
ENSG00000172020	3	115317171	115465337	397	75	120000	-0.85492	0.8037	GAP43	76353	25kb
ENSG00000185565	3	115496235	117741095	6645	323	120000	0.016639	0.49336	LSAMP	76353	25kb
ENSG00000144847	3	118594404	118889915	761	46	120000	0.74988	0.22666	IGSF11	76353	25kb
ENSG00000163424	3	118839997	118903889	255	23	120000	0.46045	0.3226	C3orf30	76353	25kb
ENSG00000251012	3	118841222	118931654	333	36	120000	0.49518	0.31024	RP11-484M3.5	76353	25kb
ENSG00000114638	3	118867364	118949000	293	40	120000	1.0481	0.14729	UPK1B	76353	25kb
ENSG00000121578	3	118905579	118984950	272	25	120000	1.1947	0.11609	B4GALT4	76353	25kb
ENSG00000031081	3	118988220	119164561	609	60	120000	-0.22749	0.58998	ARHGAP31	76353	25kb
ENSG00000176142	3	119123347	119212677	254	19	120000	-0.78367	0.78338	TMEM39A	76353	25kb
ENSG00000163389	3	119162785	119238555	218	25	120000	-1.1241	0.86952	POGLUT1	76353	25kb
ENSG00000113845	3	119192379	119268937	253	25	120000	-1.0102	0.8438	TIMMDC1	76353	25kb
ENSG00000121594	3	119218140	119303449	320	33	120000	-1.6358	0.94906	CD80	76353	25kb
ENSG00000144843	3	119273115	119333792	214	17	120000	-1.1534	0.87563	ADPRH	76353	25kb
ENSG00000144837	3	119291689	119373658	285	28	120000	-1.2843	0.90048	PLA1A	76353	25kb
ENSG00000121577	3	119330304	119409171	302	23	120000	-1.34	0.90988	POPDC2	76353	25kb
ENSG00000138495	3	119348360	119421301	291	19	120000	-1.5594	0.94055	COX17	76353	25kb

ENSG00000183833	3	119396869	119510949	320	26	120000	-1.8785	0.96984	MAATS1	76353	25kb
ENSG00000144852	3	119474331	119562332	213	35	120000	-0.5328	0.70291	NR1I2	76353	25kb
ENSG00000082701	3	119515170	119838264	531	31	120000	0.45952	0.32293	GSK3B	76353	25kb
ENSG00000175697	3	119858492	120028941	511	27	120000	-0.062921	0.52509	GPR156	76353	25kb
ENSG00000163428	3	120018356	120093186	212	23	120000	-0.58586	0.72101	LRRC58	76353	25kb
ENSG00000163430	3	120086140	120195100	314	31	120000	0.57207	0.28364	FSTL1	76353	25kb
ENSG00000065518	3	120290156	120346347	185	17	120000	-0.2681	0.60569	NDUFB4	76353	25kb
ENSG00000113924	3	120322020	120426418	346	36	120000	-0.1983	0.5786	HGD	76353	25kb
ENSG00000144840	3	120380528	120486840	258	32	120000	0.064457	0.4743	RABL3	76353	25kb
ENSG00000153767	3	120436484	120526916	204	29	120000	-0.019121	0.50763	GTF2E1	76353	25kb
ENSG00000145087	3	120601919	121168608	1608	29	120000	-0.67333	0.74963	STXBP5L	76353	25kb
ENSG00000051341	3	121125278	121289853	332	20	120000	-1.2825	0.90016	POLQ	76353	25kb
ENSG00000186103	3	121264551	121330481	183	25	120000	-0.70222	0.75873	ARGFX	76353	25kb
ENSG00000163833	3	121286966	121374139	268	32	120000	0.46221	0.32197	FBXO40	76353	25kb
ENSG00000180353	3	121325246	121404774	193	27	120000	0.72261	0.23496	HCLS1	76353	25kb
ENSG00000173230	3	121357046	121493602	283	19	120000	-0.26741	0.60542	GOLGB1	76353	25kb
ENSG00000173226	3	121463610	121578926	359	16	120000	-0.56621	0.71438	IQCB1	76353	25kb
ENSG00000145088	3	121529030	121630373	282	15	120000	-0.92248	0.82186	EAF2	76353	25kb
ENSG00000163406	3	121587936	121687949	281	11	120000	-0.59747	0.7249	SLC15A2	76353	25kb
ENSG00000145103	3	121681170	121766051	158	26	120000	-0.33262	0.63029	ILDR1	76353	25kb
ENSG00000114013	3	121749213	121864983	237	43	120000	-0.95162	0.82936	CD86	76353	25kb
ENSG00000036828	3	121877530	122030342	572	35	120000	-0.66942	0.74839	CASR	76353	25kb
ENSG00000121552	3	122019091	122085819	205	29	120000	-0.72683	0.76633	CSTA	76353	25kb
ENSG00000160124	3	122053438	122127078	203	27	120000	-1.2281	0.89029	CCDC58	76353	25kb
ENSG00000114023	3	122078023	122156181	211	25	120000	-0.76027	0.77645	FAM162A	76353	25kb
ENSG00000196981	3	122105715	122159882	140	19	120000	-0.47691	0.68329	WDR5B	76353	25kb
ENSG00000114030	3	122115796	122258792	312	20	120000	-0.081166	0.53235	KPNA1	76353	25kb
ENSG00000138496	3	122221771	122308424	244	28	120000	0.0838	0.46661	PARP9	76353	25kb
ENSG00000163840	3	122258085	122319050	244	28	120000	-0.20478	0.58113	DTX3L	76353	25kb
ENSG00000173200	3	122271449	122382894	400	49	120000	0.040048	0.48403	PARP15	76353	25kb
ENSG00000173193	3	122374465	122474687	358	28	120000	1.3167	0.093976	PARP14	76353	25kb
ENSG00000169087	3	122433846	122537671	304	21	120000	0.51843	0.30208	HSPBAP1	76353	25kb
ENSG00000138463	3	122488642	122624986	329	25	120000	0.86366	0.19389	DIRC2	76353	25kb
ENSG00000082684	3	122603041	122772452	525	59	120000	0.90021	0.184	SEMA5B	76353	25kb
ENSG00000065485	3	122760909	122969074	641	53	120000	0.82258	0.20537	PDIA5	76353	25kb
ENSG00000121542	3	122895774	123017977	371	10	120000	0.23356	0.40766	SEC22A	76353	25kb
ENSG00000173175	3	122976143	123193605	491	37	120000	0.61081	0.27066	ADCY5	76353	25kb
ENSG00000206527	3	123184667	123329032	384	26	120000	0.43848	0.33052	PTPLB	76353	25kb
ENSG00000065534	3	123303896	123628178	705	69	120000	0.18214	0.42774	MYLK	76353	25kb
ENSG00000175455	3	123591152	123705564	272	19	120000	1.1942	0.11621	CCDC14	76353	25kb
ENSG00000065371	3	123662368	123736025	141	23	120000	0.66467	0.25313	ROPN1	76353	25kb
ENSG00000160145	3	123773870	124470172	2184	108	120000	-0.45512	0.67549	KALRN	76353	25kb
ENSG00000114491	3	124424213	124489040	248	24	120000	0.9911	0.16082	UMPS	76353	25kb
ENSG00000082781	3	124455795	124645265	634	36	120000	-0.013921	0.50555	ITGB5	76353	25kb
ENSG00000173702	3	124599289	124697663	332	52	120000	-1.5959	0.94475	MUC13	76353	25kb
ENSG00000173706	3	124659554	124799802	409	46	120000	-1.4682	0.92897	HEG1	76353	25kb
ENSG00000221955	3	124776480	125023021	828	82	120000	-0.20327	0.58054	SLC12A8	76353	25kb
ENSG00000163848	3	124919405	125119198	387	25	120000	-0.21968	0.58694	ZNF148	76353	25kb
ENSG00000114520	3	125140495	125264041	368	27	120000	-0.82714	0.79592	SNX4	76353	25kb
ENSG00000144909	3	125222702	125338934	407	28	120000	-1.8086	0.96475	OSBPL11	76353	25kb
ENSG00000189366	3	125623118	125680882	255	14	120000	0.13282	0.44717	ALG1L	76353	25kb
ENSG00000114547	3	125662987	125727297	163	22	120000	0.69581	0.24327	ROPN1B	76353	25kb
ENSG00000114544	3	125700198	125845404	500	28	120000	0.44445	0.32836	SLC41A3	76353	25kb
ENSG00000144908	3	125797412	125941837	619	43	120000	0.8753	0.1907	ALDH1L1	76353	25kb
ENSG00000163884	3	126036478	126101285	167	24	120000	-0.83621	0.79848	KLF15	76353	25kb
ENSG00000163885	3	126088782	126180399	346	57	120000	-1.672	0.95274	CCDC37	76353	25kb
ENSG00000070476	3	126131444	126219762	348	32	120000	-1.5544	0.93995	ZXDC	76353	25kb
ENSG00000159650	3	126175124	126261616	339	40	120000	-1.4969	0.9328	UROC1	76353	25kb
ENSG00000180767	3	126218126	126287134	254	26	120000	-0.52562	0.70042	CHST13	76353	25kb
ENSG00000180697	3	126220842	126302808	294	30	120000	-0.20882	0.58271	C3orf22	76353	25kb
ENSG00000197763	3	126265622	126398998	428	36	120000	-0.038677	0.51543	TXNRD3	76353	25kb
ENSG00000206483	3	126265625	126352398	271	29	120000	0.088734	0.46465	TXNRD3NB	76353	25kb
ENSG00000159685	3	126398063	126704249	764	29	120000	0.76348	0.22259	CHCHD6	76353	25kb
ENSG00000114554	3	126682437	126781235	335	26	120000	0.47735	0.31656	PLXNA1	76353	25kb
ENSG00000214324	3	126886974	126942025	179	34	120000	0.20393	0.41921	C3orf56	76353	25kb
ENSG00000163870	3	127266912	127342094	76	19	120000	-0.43909	0.6697	TPRA1	76353	25kb
ENSG00000073111	3	127292066	127366276	69	18	120000	-0.68402	0.75302	MCM2	76353	25kb
ENSG00000114631	3	127323024	127416652	124	21	120000	-0.33342	0.63059	PODXL2	76353	25kb
ENSG00000114626	3	127366778	127424768	114	21	120000	-0.38375	0.64942	ABTB1	76353	25kb
ENSG00000074416	3	127382909	127567051	474	67	120000	-0.29883	0.61746	MGLL	76353	25kb
ENSG00000187715	3	127609075	127731514	354	33	120000	1.0677	0.14282	KBTBD12	76353	25kb
ENSG00000058262	3	127745484	127815526	174	7	120000	0.3455	0.36486	SEC61A1	76353	25kb
ENSG00000175792	3	127758621	127897757	286	11	120000	0.75311	0.22569	RUVBL1	76353	25kb
ENSG00000132394	3	127847297	128152485	592	24	120000	0.97905	0.16378	EEFSEC	76353	25kb
ENSG00000179407	3	128156282	128211091	208	37	120000	0.15519	0.43834	DNAJB8	76353	25kb
ENSG00000179348	3	128173270	128237028	221	44	120000	0.051141	0.47961	GATA2	76353	25kb
ENSG00000198685	3	128265843	128319929	177	27	120000	-0.057472	0.52292	C3orf27	76353	25kb
ENSG00000163902	3	128313817	128424918	336	21	120000	-0.74797	0.77276	RPN1	76353	25kb
ENSG00000075785	3	128419965	128558639	399	22	120000	-0.34586	0.63528	RAB7A	76353	25kb
ENSG00000177646	3	128573439	128659910	357	20	120000	-0.53634	0.70414	ACAD9	76353	25kb
ENSG00000114656	3	128603709	128746533	395	30	120000	-0.81907	0.79363	KIAA1257	76353	25kb
ENSG00000187695	3	128603717	128715173	350	26	120000	-0.77344	0.78037	RP11-723O4.6	76353	25kb
ENSG00000114654	3	128695472	128784585	121	23	120000	-0.47083	0.68112	EFCC1	76353	25kb
ENSG00000169704	3	128754610	128806249	62	15	120000	-1.2966	0.90261	GP9	76353	25kb
ENSG00000172780	3	128781412	128866644	96	12	120000	-1.1619	0.87737	RAB43	76353	25kb
ENSG00000261796	3	128781418	128904879	135	17	120000	-1.2005	0.88502	ISY1-RAB43	76353	25kb
ENSG00000240682	3	128821258	128905136	92	15	120000	-1.1012	0.86458	ISY1	76353	25kb
ENSG00000169714	3	128863327	128927765	77	15	120000	-0.63449	0.73712	CNBP	76353	25kb
ENSG00000181789	3	128943449	129021614	136	10	120000	0.69366	0.24395	COPG1	76353	25kb
ENSG00000183624	3	128972671	129050029	146	9	120000	0.40284	0.34353	HMCES	76353	25kb
ENSG00000184897	3	129008615	129060120	111	9	120000	0.31945	0.37469	H1FX	76353	25kb
ENSG00000172771	3	129095164	129172494	195	16	120000	-0.77025	0.77942	EFCAB12	76353	25kb
ENSG00000129071	3	129124787	129183878	138	8	120000	-0.94501	0.82767	MBD4	76353	25kb
ENSG00000163913	3	129133968	129264198	285	10	120000	-0.75185	0.77393	IFT122	76353	25kb
ENSG00000163914	3	129222483	129279012	111	12	120000	-0.44175	0.67067	RHO	76353	25kb
ENSG00000178804	3	129237057	129295310	153	18	120000	-0.50891	0.69459	H1FOO	76353	25kb

ENSG00000004399	3	129249018	129350661	312	27	120000	-0.39481	0.65351	PLXND1	76353	25kb
ENSG00000172765	3	129341635	129637419	387	18	120000	-0.7586	0.77596	TMCC1	76353	25kb
ENSG00000170893	3	129668148	129721781	198	25	120000	0.87948	0.18957	TRH	76353	25kb
ENSG00000172752	3	130039359	130228688	667	35	120000	0.79755	0.21257	COL6A5	76353	25kb
ENSG00000206384	3	130254178	130421999	328	28	120000	1.2668	0.10261	COL6A6	76353	25kb
ENSG00000196455	3	130372779	130490673	199	6	120000	2.0345	0.020948	PIK3R4	76353	25kb
ENSG00000017260	3	130544439	130760556	359	29	120000	-0.18131	0.57194	ATP2C1	76353	25kb
ENSG00000034533	3	130707719	130771493	115	14	120000	0.47083	0.31888	ASTE1	76353	25kb
ENSG00000114670	3	130720694	131094309	799	29	120000	0.70362	0.24084	NEK11	76353	25kb
ENSG00000198585	3	131075515	131132674	166	16	120000	-0.038626	0.51541	NUDT16	76353	25kb
ENSG00000114686	3	131156056	131246827	198	25	120000	0.59601	0.27558	MRPL3	76353	25kb
ENSG00000196353	3	131227399	132029254	3012	109	120000	-0.40747	0.65817	CPNE4	76353	25kb
ENSG00000014257	3	132011211	132112142	451	29	120000	0.22533	0.41086	ACPP	76353	25kb
ENSG00000138246	3	132111370	132282876	369	31	120000	-0.30748	0.62076	DNAJC13	76353	25kb
ENSG00000240303	3	132251982	132404567	258	22	120000	-0.21988	0.58702	ACAD11	76353	25kb
ENSG00000113971	3	132251986	132466303	386	32	120000	-0.33806	0.63234	NPHP3	76353	25kb
ENSG00000129048	3	132291081	132362811	113	16	120000	-0.26302	0.60373	ACKR4	76353	25kb
ENSG00000081307	3	132348290	132421941	96	15	120000	-0.70182	0.75861	UBA5	76353	25kb
ENSG00000144868	3	132732235	133141636	1312	58	120000	-1.3994	0.91915	TMEM108	76353	25kb
ENSG00000170819	3	133093839	133219066	405	58	120000	0.32766	0.37158	BFSP2	76353	25kb
ENSG00000091527	3	133267574	133334105	172	24	120000	-1.4105	0.92081	CDV3	76353	25kb
ENSG00000163781	3	133292019	133405737	329	23	120000	-1.3689	0.91448	TOPBP1	76353	25kb
ENSG00000091513	3	133439800	133522850	236	27	120000	-0.050252	0.52004	TF	76353	25kb
ENSG00000144867	3	133477877	133569616	236	31	120000	0.49589	0.30999	SRPRB	76353	25kb
ENSG00000154917	3	133518083	133639680	375	59	120000	-0.38772	0.65089	RAB6B	76353	25kb
ENSG00000221972	3	133621992	133673656	133	29	120000	0.070395	0.47194	C3orf36	76353	25kb
ENSG00000174640	3	133626540	133796028	503	68	120000	0.50675	0.30617	SLCO2A1	76353	25kb
ENSG00000163785	3	133759147	133994689	614	31	120000	1.0756	0.14105	RYK	76353	25kb
ENSG00000114019	3	134049716	134119321	125	20	120000	1.3289	0.091945	AMOTL2	76353	25kb
ENSG00000129055	3	134171548	134230558	108	7	120000	-0.039653	0.51582	ANAPC13	76353	25kb
ENSG00000182923	3	134179585	134318859	289	7	120000	-0.0039995	0.5016	CEP63	76353	25kb
ENSG00000154928	3	134291643	135004309	2105	147	120000	-0.61873	0.73195	EPHB1	76353	25kb
ENSG00000174611	3	134296980	134395478	234	15	120000	-0.95141	0.8293	KY	76353	25kb
ENSG00000073711	3	135659515	135891733	373	24	120000	-0.69093	0.7552	PPP2R3A	76353	25kb
ENSG00000174579	3	135842764	135941083	108	19	120000	-0.29371	0.61551	MSL2	76353	25kb
ENSG00000114054	3	135944148	136081738	192	23	120000	-0.88771	0.81265	PCCB	76353	25kb
ENSG00000118007	3	136030077	136496220	703	30	120000	-0.86603	0.80676	STAG1	76353	25kb
ENSG00000168917	3	136512489	136599734	200	11	120000	0.82039	0.206	SLC35G2	76353	25kb
ENSG00000158092	3	136556050	136693665	287	13	120000	0.85089	0.19742	NCK1	76353	25kb
ENSG00000174564	3	136640072	136754927	227	17	120000	0.36159	0.35883	IL20RB	76353	25kb
ENSG00000168875	3	137458579	137509396	112	19	120000	-0.9844	0.83754	SOX14	76353	25kb
ENSG00000066405	3	137692577	137777494	180	22	120000	0.73372	0.23156	CLDN18	76353	25kb
ENSG00000158163	3	137755832	137859660	227	19	120000	0.20626	0.41829	DZIP1L	76353	25kb
ENSG00000118017	3	137817560	137876229	109	9	120000	1.2733	0.10145	A4GNT	76353	25kb
ENSG00000138231	3	137854854	137918791	103	5	120000	1.065	0.14344	DBR1	76353	25kb
ENSG00000114098	3	137881109	138042231	152	30	120000	-0.15089	0.55997	ARMC8	76353	25kb
ENSG00000181322	3	137955279	138073728	132	23	120000	-1.4395	0.92499	NME9	76353	25kb
ENSG00000158186	3	138041539	138149375	201	17	120000	-1.5722	0.94205	MRAS	76353	25kb
ENSG00000158220	3	138128428	138225528	163	25	120000	-0.28962	0.61395	ESYT3	76353	25kb
ENSG00000114107	3	138188186	138338380	257	14	120000	-0.082427	0.53285	CEP70	76353	25kb
ENSG00000158234	3	138302448	138377218	103	11	120000	-0.070782	0.52821	FAIM	76353	25kb
ENSG00000051382	3	138347860	138578780	301	11	120000	-0.16499	0.56552	PIK3CB	76353	25kb
ENSG00000183770	3	138638066	138690982	130	16	120000	-0.63155	0.73616	FOXL2	76353	25kb
ENSG00000206262	3	138641076	138697293	140	17	120000	-0.46779	0.68003	C3orf72	76353	25kb
ENSG00000206260	3	138697804	138750110	85	12	120000	2.102	0.017776	PRR23A	76353	25kb
ENSG00000175110	3	138699648	139101065	855	51	120000	1.7121	0.043437	MRPS22	76353	25kb
ENSG00000184814	3	138712873	138764768	97	13	120000	2.6768	0.0037162	PRR23B	76353	25kb
ENSG00000233701	3	138735944	138788734	93	9	120000	2.7574	0.0029133	PRR23C	76353	25kb
ENSG00000184432	3	139049442	139133574	199	16	120000	0.099639	0.46032	COPB2	76353	25kb
ENSG00000114113	3	139146726	139224589	207	16	120000	-0.063488	0.52531	RBP2	76353	25kb
ENSG00000114115	3	139211276	139283671	230	25	120000	1.1702	0.12097	RBP1	76353	25kb
ENSG00000163864	3	139254022	139421859	429	50	120000	0.080228	0.46803	NMNAT3	76353	25kb
ENSG00000158258	3	139629027	140321239	2122	164	120000	0.97822	0.16398	CLSTN2	76353	25kb
ENSG00000155890	3	140371881	140444992	263	13	120000	-1.2006	0.88505	TRIM42	76353	25kb
ENSG00000114120	3	140635672	140723775	214	14	120000	-1.0775	0.85938	SLC25A36	76353	25kb
ENSG00000175093	3	140745244	140892453	297	30	120000	-0.28995	0.61407	SPSB4	76353	25kb
ENSG00000155893	3	140922568	141038748	376	36	120000	1.2352	0.10837	ACPL2	76353	25kb
ENSG00000177311	3	141018055	141193634	403	40	120000	0.058809	0.47655	ZBTB38	76353	25kb
ENSG00000155903	3	141180889	141359184	347	34	120000	-0.20562	0.58145	RASA2	76353	25kb
ENSG00000114125	3	141432046	141491402	154	16	120000	0.53159	0.29751	RNF7	76353	25kb
ENSG00000114124	3	141471990	141562332	240	31	120000	-0.43213	0.66718	GRK7	76353	25kb
ENSG00000069849	3	141569966	141670356	329	24	120000	0.25865	0.39795	ATP1B3	76353	25kb
ENSG00000114126	3	141638277	141893386	812	21	120000	-0.56345	0.71344	TFDP2	76353	25kb
ENSG00000175066	3	141857414	141969449	303	28	120000	0.85924	0.19511	GK5	76353	25kb
ENSG00000114127	3	142000449	142191904	261	21	120000	-1.0206	0.84627	XRN1	76353	25kb
ENSG00000175054	3	142143077	142322668	401	21	120000	-0.27113	0.60685	ATR	76353	25kb
ENSG00000120756	3	142290229	142457506	387	24	120000	-0.56004	0.71227	PLS1	76353	25kb
ENSG00000144935	3	142417916	142551730	256	16	120000	-0.65261	0.743	TRPC1	76353	25kb
ENSG00000163710	3	142509764	142633045	348	40	120000	-1.9207	0.97261	PCOLCE2	76353	25kb
ENSG00000188582	3	142643006	142707178	208	37	120000	-0.29715	0.61682	PAQR9	76353	25kb
ENSG00000163714	3	142658339	142804567	327	34	120000	-1.2855	0.90068	U2SURP	76353	25kb
ENSG00000175040	3	142813173	142866800	135	15	120000	-0.99051	0.83904	CHST2	76353	25kb
ENSG00000181804	3	142959064	143592373	2040	173	120000	1.0287	0.15181	SLC9A9	76353	25kb
ENSG00000181744	3	143665640	143792561	377	15	120000	-0.077552	0.53091	C3orf58	76353	25kb
ENSG00000232013	3	145757358	145809156	137	14	120000	0.17666	0.42989	AC107021.1	76353	25kb
ENSG00000152952	3	145762227	145906440	392	18	120000	-0.1502	0.5597	PLOD2	76353	25kb
ENSG00000114698	3	145885126	145993966	397	27	120000	-0.70912	0.76087	PLSCR4	76353	25kb
ENSG00000163746	3	146084208	146238778	425	20	120000	0.77471	0.21925	PLSCR2	76353	25kb
ENSG00000188313	3	146207967	146287651	302	28	120000	-1.1296	0.87067	PLSCR1	76353	25kb
ENSG00000231213	3	146269342	146349003	417	29	120000	0.67139	0.25098	PLSCR5	76353	25kb
ENSG00000174963	3	147078833	147149647	140	25	120000	0.26179	0.39674	ZIC4	76353	25kb
ENSG00000152977	3	147086209	147253080	356	27	120000	1.2722	0.10165	ZIC1	76353	25kb
ENSG00000144891	3	148390571	148485795	268	34	120000	1.6682	0.047635	AGTR1	76353	25kb
ENSG00000153002	3	148483889	148602974	385	53	120000	-0.85502	0.80373	CPB1	76353	25kb
ENSG00000163751	3	148558043	148639983	284	24	120000	-0.83184	0.79725	CPA3	76353	25kb
ENSG00000163754	3	148684128	148770419	199	29	120000	-1.4397	0.92502	GYG1	76353	25kb

ENSG00000071794	3	148722914	148829341	256	21	120000	0.11363	0.45477	HLTF	76353	25kb
ENSG00000163755	3	148822371	148916519	333	36	120000	-0.40112	0.65583	HPS3	76353	25kb
ENSG00000047457	3	148855197	148964842	383	47	120000	-1.455	0.92717	CP	76353	25kb
ENSG00000163762	3	149011285	149077201	213	38	120000	-0.051143	0.52039	TM4SF18	76353	25kb
ENSG00000169908	3	149061809	149120652	194	28	120000	0.40456	0.3429	TM4SF1	76353	25kb
ENSG00000169903	3	149166761	149246068	469	37	120000	-1.8334	0.96663	TM4SF4	76353	25kb
ENSG00000018408	3	149210022	149479501	912	80	120000	1.5541	0.060076	WWTR1	76353	25kb
ENSG00000114744	3	149431257	149495286	148	19	120000	1.2996	0.096864	COMMD2	76353	25kb
ENSG00000206199	3	149453892	149711172	590	35	120000	1.6524	0.049226	ANKUB1	76353	25kb
ENSG00000082996	3	149505495	149704926	423	29	120000	1.5405	0.061723	RNF13	76353	25kb
ENSG00000070087	3	149657691	149793575	347	29	120000	2.6164	0.0044435	PFN2	76353	25kb
ENSG00000268175	3	149664066	149715535	117	20	120000	2.5199	0.0058701	AC117395.1	76353	25kb
ENSG00000196428	3	150101122	150209218	287	23	120000	-0.54526	0.70721	TSC22D2	76353	25kb
ENSG00000120742	3	150234781	150346015	312	28	120000	-1.3575	0.91269	SERP1	76353	25kb
ENSG00000144895	3	150239465	150327029	256	26	120000	-1.3081	0.90459	EIF2A	76353	25kb
ENSG00000198843	3	150295662	150373222	217	30	120000	-1.0725	0.85826	SELT	76353	25kb
ENSG00000163645	3	150352672	150446758	267	39	120000	-1.1766	0.88032	FAM194A	76353	25kb
ENSG00000181788	3	150433914	150506264	141	14	120000	-0.64271	0.73979	SIAH2	76353	25kb
ENSG00000214237	3	150563832	150637089	190	44	120000	-0.44128	0.67049	FAM188B2	76353	25kb
ENSG00000260234	3	150575784	150684530	325	60	120000	-0.87619	0.80954	RP11-166N6.3	76353	25kb
ENSG00000163646	3	150618950	150715786	275	39	120000	-0.33956	0.6329	CLRN1	76353	25kb
ENSG00000144893	3	150778484	151179860	1042	52	120000	0.96771	0.16659	MED12L	76353	25kb
ENSG00000174946	3	150890619	150945988	165	19	120000	0.0071033	0.49717	GPR171	76353	25kb
ENSG00000174944	3	150904905	151021255	321	29	120000	0.80588	0.21016	P2RY14	76353	25kb
ENSG00000138271	3	150986891	151059740	223	23	120000	1.1732	0.12036	GPR87	76353	25kb
ENSG00000181631	3	151019100	151072336	162	19	120000	1.2333	0.10872	P2RY13	76353	25kb
ENSG00000169313	3	151030168	151127600	312	27	120000	1.6153	0.053126	P2RY12	76353	25kb
ENSG00000152580	3	151118172	151201497	235	34	120000	0.35069	0.36291	IGSF10	76353	25kb
ENSG00000197953	3	151426704	151504127	294	19	120000	-0.8702	0.8079	AADACL2	76353	25kb
ENSG00000114771	3	151506825	151571276	271	23	120000	-0.8366	0.79859	AADAC	76353	25kb
ENSG00000198829	3	151566431	151627405	178	28	120000	0.13959	0.44449	SUCNR1	76353	25kb
ENSG00000152601	3	151936617	152208569	414	31	120000	-0.81501	0.79247	MBNL1	76353	25kb
ENSG00000221962	3	152032484	152083779	79	14	120000	-0.16723	0.56641	TMEM14E	76353	25kb
ENSG00000169860	3	152527736	152580841	116	12	120000	-1.3616	0.91333	P2RY1	76353	25kb
ENSG00000181467	3	152855029	152911265	80	17	120000	-0.80944	0.79087	RAP2B	76353	25kb
ENSG00000237787	3	153177284	153245486	210	23	120000	-0.77707	0.78144	C3orf79	76353	25kb
ENSG00000114790	3	153813792	154000616	416	23	120000	-1.0807	0.86008	ARHGEF26	76353	25kb
ENSG00000174953	3	153965335	154067286	181	20	120000	-0.18167	0.57208	DHX36	76353	25kb
ENSG00000174948	3	154030461	154172504	325	30	120000	0.052281	0.47915	GPR149	76353	25kb
ENSG00000196549	3	154716913	154926497	482	69	120000	0.14249	0.44335	MME	76353	25kb
ENSG00000114805	3	155068369	155487856	1091	40	120000	-1.9766	0.97596	PLCH1	76353	25kb
ENSG00000214919	3	155434933	155486515	128	19	120000	-0.85272	0.80309	AC104472.1	76353	25kb
ENSG00000174928	3	155455401	155549140	240	28	120000	-0.40724	0.65809	C3orf33	76353	25kb
ENSG00000169359	3	155513813	155597218	218	27	120000	0.21427	0.41517	SLC33A1	76353	25kb
ENSG00000163655	3	155563325	155686815	325	16	120000	-0.52718	0.70096	GMPS	76353	25kb
ENSG00000169282	3	155730490	156281545	1487	76	120000	0.047571	0.48103	KCNAB1	76353	25kb
ENSG00000114850	3	156232929	156297973	216	25	120000	1.7236	0.042395	SSR3	76353	25kb
ENSG00000163659	3	156366024	156449559	222	22	120000	0.83663	0.2014	TIPARP	76353	25kb
ENSG00000197980	3	156518270	156788918	758	29	120000	0.35051	0.36298	LEKR1	76353	25kb
ENSG00000163660	3	156839297	156903549	164	16	120000	1.4469	0.073968	CCNL1	76353	25kb
ENSG00000197415	3	156952531	157276408	883	39	120000	0.24089	0.40482	VEPH1	76353	25kb
ENSG00000163661	3	157129578	157186417	147	23	120000	0.17505	0.43052	PTX3	76353	25kb
ENSG00000174899	3	157236035	157420538	552	38	120000	0.13014	0.44823	C3orf55	76353	25kb
ENSG00000168779	3	157789948	157849292	127	13	120000	0.66014	0.25458	SHOX2	76353	25kb
ENSG00000174891	3	157798644	158288519	1379	24	120000	0.13099	0.44789	RSRC1	76353	25kb
ENSG00000178053	3	158263952	158350041	290	19	120000	1.0603	0.14451	MLF1	76353	25kb
ENSG00000168827	3	158337067	158435364	321	13	120000	0.57481	0.28271	GFM1	76353	25kb
ENSG00000079257	3	158338611	158415482	254	13	120000	0.63818	0.26168	LXN	76353	25kb
ENSG00000118849	3	158389681	158475485	276	16	120000	0.10662	0.45754	RARRES1	76353	25kb
ENSG00000118855	3	158424987	158572508	404	35	120000	-0.60681	0.72801	MFSD1	76353	25kb
ENSG00000250588	3	158655024	159640155	2049	115	120000	1.0449	0.14804	IQCJ-SCHIP1	76353	25kb
ENSG00000214216	3	158655717	159009096	722	50	120000	1.5384	0.061981	IQCJ	76353	25kb
ENSG00000151967	3	159532650	159640149	167	33	120000	1.3244	0.092693	SCHIP1	76353	25kb
ENSG00000168811	3	159681537	159738806	200	23	120000	2.1457	0.015949	IL12A	76353	25kb
ENSG00000242107	3	159708811	159774210	246	28	120000	1.6463	0.04985	LINC01100	76353	25kb
ENSG00000180044	3	159918423	159970999	105	22	120000	-0.018737	0.50747	C3orf80	76353	25kb
ENSG00000248710	3	159920241	160192617	441	38	120000	-0.37082	0.64461	RP11-432B6.3	76353	25kb
ENSG00000068885	3	159949774	160142668	280	27	120000	-0.41833	0.66215	IFT80	76353	25kb
ENSG00000113810	3	160092062	160177750	153	19	120000	-0.14428	0.55736	SMC4	76353	25kb
ENSG00000213186	3	160125233	160228561	201	23	120000	-0.54402	0.70679	TRIM59	76353	25kb
ENSG00000186432	3	160187783	160308376	210	20	120000	-1.0943	0.86308	KPNA4	76353	25kb
ENSG00000179674	3	160369948	160421233	167	17	120000	-1.8207	0.96567	ARL14	76353	25kb
ENSG00000163590	3	160448390	160821695	844	34	120000	0.6901	0.24506	PPM1L	76353	25kb
ENSG00000169255	3	160776671	160848172	154	15	120000	1.4729	0.070383	B3GALNT1	76353	25kb
ENSG00000169251	3	160797484	160996320	437	23	120000	0.78241	0.21699	NMD3	76353	25kb
ENSG00000196542	3	161037580	161115668	298	20	120000	0.18348	0.42721	SPTSSB	76353	25kb
ENSG00000182447	3	161189596	161246730	236	15	120000	-0.93807	0.8259	OTOL1	76353	25kb
ENSG00000090402	3	164671686	164821283	311	12	120000	0.33085	0.37038	SI	76353	25kb
ENSG00000121871	3	164879508	164939897	104	14	120000	-0.99197	0.83939	SLITRK3	76353	25kb
ENSG00000114200	3	165465692	165580260	413	26	120000	0.22112	0.4125	BCHE	76353	25kb
ENSG00000169064	3	166933075	167124134	480	34	120000	-1.0074	0.84313	ZBBX	76353	25kb
ENSG00000114204	3	167134577	167221792	250	22	120000	0.24792	0.4021	SERPINI2	76353	25kb
ENSG00000174776	3	167171472	167396771	380	39	120000	0.50228	0.30774	WDR49	76353	25kb
ENSG00000114209	3	167376086	167477727	149	10	120000	0.55961	0.28787	PDCD10	76353	25kb
ENSG00000163536	3	167428031	167568356	384	27	120000	-0.74958	0.77325	SERPINI1	76353	25kb
ENSG00000173905	3	167701465	167838763	331	24	120000	0.78794	0.21537	GOLIM4	76353	25kb
ENSG00000085276	3	168776287	169406406	1774	126	120000	-0.92847	0.82342	MECOM	76353	25kb
ENSG00000184378	3	169459709	169512683	80	10	120000	-0.84495	0.80093	ACTRT3	76353	25kb
ENSG00000085274	3	169465619	169532504	115	11	120000	-1.0544	0.85415	MYNN	76353	25kb
ENSG00000171757	3	169486216	169555774	142	17	120000	-0.63882	0.73853	LRRC34	76353	25kb
ENSG00000188306	3	169514710	169580563	162	19	120000	0.32768	0.37158	LRRIQ4	76353	25kb
ENSG00000114248	3	169531967	169612718	209	29	120000	0.66317	0.25361	LRRC31	76353	25kb
ENSG00000187033	3	169604360	169681963	255	18	120000	2.2659	0.011728	SAMD7	76353	25kb
ENSG00000008952	3	169659423	169741161	191	21	120000	1.519	0.064383	SEC62	76353	25kb
ENSG00000173890	3	169730717	169828191	214	17	120000	0.94565	0.17216	GPR160	76353	25kb
ENSG00000173889	3	169779520	169924537	238	19	120000	0.62851	0.26484	PHC3	76353	25kb

ENSG00000163558	3	169915153	170048769	420	17	120000	0.29305	0.38474	PRKCI	76353	25kb
ENSG00000136603	3	170050466	170139623	225	24	120000	-0.04752	0.51895	SKIL	76353	25kb
ENSG00000013297	3	170111653	170603169	1190	90	120000	1.5895	0.055977	CLDN11	76353	25kb
ENSG00000013293	3	170152372	170328863	371	44	120000	2.7419	0.0030545	SLC7A14	76353	25kb
ENSG00000163584	3	170557664	170613272	131	17	120000	0.35292	0.36208	RPL22L1	76353	25kb
ENSG00000163577	3	170581204	170651482	127	17	120000	0.45195	0.32565	EIF5A2	76353	25kb
ENSG00000163581	3	170689137	170769539	198	15	120000	2.0096	0.022237	SLC2A2	76353	25kb
ENSG00000154310	3	170754128	171203197	1231	139	120000	0.28976	0.386	TNIK	76353	25kb
ENSG00000075651	3	171293195	171553740	707	28	120000	1.7522	0.039867	PLD1	76353	25kb
ENSG00000183657	3	171484580	171552714	204	24	120000	0.53212	0.29732	PP13439	76353	25kb
ENSG00000186329	3	171536139	171681505	423	42	120000	1.5901	0.055906	TMEM212	76353	25kb
ENSG00000075420	3	171732418	172144455	1110	112	120000	-0.56617	0.71436	FNDC3B	76353	25kb
ENSG00000267979	3	171994438	172044686	134	26	120000	-1.5338	0.93746	AC092964.2	76353	25kb
ENSG00000203443	3	172009218	172064513	127	26	120000	-0.63379	0.73689	AC092964.1	76353	25kb
ENSG00000121853	3	172137923	172191246	156	21	120000	-0.82567	0.79551	GHSR	76353	25kb
ENSG00000121858	3	172198298	172266297	278	28	120000	-0.6444	0.74034	TNFSF10	76353	25kb
ENSG00000144959	3	172323039	172454008	412	57	120000	-0.33376	0.63072	NCEH1	76353	25kb
ENSG00000269496	3	172336483	172386740	130	25	120000	-0.17699	0.57024	AC007919.2	76353	25kb
ENSG00000114346	3	172443472	172564264	280	17	120000	-0.25463	0.6005	ECT2	76353	25kb
ENSG00000144962	3	172582148	172884058	771	57	120000	-0.75304	0.77429	SPATA16	76353	25kb
ENSG00000169760	3	173089074	174029434	2537	146	120000	-0.6299	0.73562	NLGN1	76353	25kb
ENSG00000177694	3	174131363	175548428	4559	254	120000	-1.3527	0.91193	NAALADL2	76353	25kb
ENSG00000177565	3	176712143	176940261	634	46	120000	-0.14703	0.55845	TBL1XR1	76353	25kb
ENSG00000197584	3	177965720	178587217	1759	101	120000	0.51315	0.30392	KCNMB2	76353	25kb
ENSG00000172667	3	178710011	178815067	207	28	120000	1.0857	0.1388	ZMAT3	76353	25kb
ENSG00000121879	3	178840902	178982881	377	28	120000	-0.49353	0.68918	PIK3CA	76353	25kb
ENSG00000171121	3	178932530	179009790	262	26	120000	-0.72413	0.76551	KCNMB3	76353	25kb
ENSG00000121864	3	179015779	179078323	133	20	120000	-0.63377	0.73688	ZNF639	76353	25kb
ENSG00000171109	3	179040480	179137719	165	20	120000	-1.0048	0.84251	MFN1	76353	25kb
ENSG00000114450	3	179091990	179194378	193	29	120000	-1.0601	0.85546	GNB4	76353	25kb
ENSG00000136518	3	179255668	179331196	111	11	120000	0.36673	0.35691	ACTL6A	76353	25kb
ENSG00000136522	3	179281073	179347442	102	9	120000	0.26207	0.39663	MRPL47	76353	25kb
ENSG00000136521	3	179297478	179370435	124	16	120000	0.7037	0.24081	NDUFB5	76353	25kb
ENSG00000058056	3	179345543	179532189	463	54	120000	-0.1192	0.54744	USP13	76353	25kb
ENSG00000114757	3	179487746	179779841	794	48	120000	-0.25772	0.60169	PEX5L	76353	25kb
ENSG00000163728	3	180294918	180360616	88	19	120000	1.4162	0.078352	TTC14	76353	25kb
ENSG00000145075	3	180295646	180613793	702	60	120000	2.6767	0.0037174	CCDC39	76353	25kb
ENSG00000114416	3	180560929	180725541	355	23	120000	3.1508	0.00081408	FXR1	76353	25kb
ENSG00000205981	3	180676497	180732562	79	13	120000	2.726	0.0032057	DNAJC19	76353	25kb
ENSG00000181449	3	181404714	181457221	79	19	120000	0.65079	0.25759	SOX2	76353	25kb
ENSG00000058063	3	182486288	182664423	345	17	120000	-0.51709	0.69745	ATP11B	76353	25kb
ENSG00000043093	3	182630862	182728741	152	20	120000	-1.0768	0.85921	DCUN1D1	76353	25kb
ENSG00000078070	3	182708006	182858863	333	20	120000	-0.7869	0.78433	MCCC1	76353	25kb
ENSG00000078081	3	182815001	182906627	315	26	120000	-0.84549	0.80108	LAMP3	76353	25kb
ENSG00000053524	3	182870831	183171566	921	66	120000	-1.1086	0.86619	MCF2L2	76353	25kb
ENSG00000176597	3	182946032	183041292	235	19	120000	-0.91893	0.82093	B3GNT5	76353	25kb
ENSG00000172578	3	183180319	183298477	317	41	120000	2.349	0.0094122	KLHL6	76353	25kb
ENSG00000114796	3	183328356	183427265	265	8	120000	-1.3491	0.91135	KLHL24	76353	25kb
ENSG00000163872	3	183390606	183555413	334	23	120000	-0.18997	0.57533	YEATS2	76353	25kb
ENSG00000180834	3	183508664	183568382	155	18	120000	-0.25358	0.60009	MAP6D1	76353	25kb
ENSG00000175193	3	183522173	183627721	344	21	120000	0.40729	0.3419	PARL	76353	25kb
ENSG00000114770	3	183612722	183760803	431	27	120000	1.0313	0.15119	ABCC5	76353	25kb
ENSG00000186090	3	183724332	183782157	180	28	120000	0.65082	0.25758	HTR3D	76353	25kb
ENSG00000178084	3	183745835	183803459	197	30	120000	0.50166	0.30795	HTR3C	76353	25kb
ENSG00000186038	3	183789852	183849783	152	19	120000	0.34785	0.36398	HTR3E	76353	25kb
ENSG00000145191	3	183827826	184427546	1961	182	120000	-1.3832	0.9167	EIF2B5	76353	25kb
ENSG00000161202	3	183848176	183916398	135	19	120000	-1.2422	0.89291	DVL3	76353	25kb
ENSG00000161203	3	183867477	183926879	122	18	120000	-1.6141	0.94675	AP2M1	76353	25kb
ENSG00000161204	3	183878811	183936800	128	20	120000	-1.7808	0.96253	ABCF3	76353	25kb
ENSG00000145198	3	183923217	183985117	132	18	120000	-1.2094	0.88674	VWA5B2	76353	25kb
ENSG00000214160	3	183935089	183992336	127	16	120000	-0.8651	0.80651	ALG3	76353	25kb
ENSG00000145194	3	183942438	184035819	187	27	120000	0.34438	0.36528	ECE2	76353	25kb
ENSG00000163888	3	183952001	184004251	103	17	120000	0.11155	0.45559	CAMK2N2	76353	25kb
ENSG00000175166	3	183991497	184051842	108	21	120000	1.5786	0.057209	PSMD2	76353	25kb
ENSG00000114867	3	184007283	184078146	143	27	120000	0.92643	0.17711	EIF4G1	76353	25kb
ENSG00000175182	3	184028714	184089063	124	28	120000	0.30786	0.37909	FAM131A	76353	25kb
ENSG00000114859	3	184038973	184104439	146	35	120000	-0.049203	0.51962	CLCN2	76353	25kb
ENSG00000163882	3	184054506	184111384	131	31	120000	-0.3517	0.63747	POLR2H	76353	25kb
ENSG00000090534	3	184064723	184120932	146	38	120000	-0.63548	0.73744	THPO	76353	25kb
ENSG00000090539	3	184072861	184133524	175	41	120000	-0.49515	0.68975	CHRD	76353	25kb
ENSG00000224153	3	184094325	184182436	344	47	120000	-0.51652	0.69725	RP11-433C9.2	76353	25kb
ENSG00000182580	3	184254572	184325197	253	32	120000	-0.897	0.81514	EPHB3	76353	25kb
ENSG00000177383	3	184403156	184454836	185	33	120000	0.32125	0.37401	MAGEF1	76353	25kb
ENSG00000156931	3	184504931	184795402	635	34	120000	0.084212	0.46644	VPS8	76353	25kb
ENSG00000187068	3	184770838	184895802	423	29	120000	-0.51842	0.69792	C3orf70	76353	25kb
ENSG00000113790	3	184883412	185024778	302	20	120000	-2.2543	0.98791	EHHADH	76353	25kb
ENSG00000073803	3	184975729	185231885	574	31	120000	-0.89613	0.81491	MAP3K13	76353	25kb
ENSG00000163900	3	185169284	185241845	120	14	120000	0.67943	0.24843	TMEM41A	76353	25kb
ENSG00000163898	3	185199050	185295401	218	20	120000	0.012478	0.49502	LIPH	76353	25kb
ENSG00000163904	3	185275284	185376339	199	16	120000	-0.61753	0.73156	SENP2	76353	25kb
ENSG00000073792	3	185336527	185567844	387	43	120000	-0.50823	0.69435	IGF2BP2	76353	25kb
ENSG00000163915	3	185405316	185472575	91	23	120000	-0.10298	0.54101	C3orf65	76353	25kb
ENSG00000136527	3	185608694	185680924	164	25	120000	-0.37377	0.64571	TRA2B	76353	25kb
ENSG00000244405	3	185739097	185853107	231	30	120000	-0.17985	0.57137	ETV5	76353	25kb
ENSG00000058866	3	185798457	186105026	755	115	120000	-1.1664	0.87827	DGKG	76353	25kb
ENSG00000213139	3	186231230	186289491	110	16	120000	0.80396	0.21071	CRYGS	76353	25kb
ENSG00000113838	3	186238862	186313332	152	14	120000	1.1978	0.11549	TBCCD1	76353	25kb
ENSG00000090520	3	186260192	186340061	201	21	120000	1.3716	0.085092	DNAJB11	76353	25kb
ENSG00000145192	3	186305712	186364107	159	26	120000	0.96828	0.16645	AHSG	76353	25kb
ENSG00000090512	3	186328758	186395930	201	36	120000	0.13126	0.44779	FETUB	76353	25kb
ENSG00000113905	3	186353005	186421029	170	28	120000	-0.42922	0.66612	HRG	76353	25kb
ENSG00000113889	3	186410065	186486743	360	33	120000	-1.2344	0.89147	KNG1	76353	25kb
ENSG00000156976	3	186475994	186532689	122	26	120000	-0.51623	0.69715	EIF4A2	76353	25kb
ENSG00000163918	3	186482669	186549847	139	26	120000	-0.27041	0.60658	RFC4	76353	25kb
ENSG00000181092	3	186535463	186601252	192	40	120000	0.073893	0.47055	ADIPOQ	76353	25kb
ENSG00000073849	3	186623274	186821341	738	91	120000	1.982	0.023738	ST6GAL1	76353	25kb

ENSG00000163923	3	186813736	186923696	299	40	120000	-1.0833	0.86065	RPL39L	76353	25kb
ENSG00000175077	3	186890274	186944253	140	25	120000	-1.1791	0.88082	RTP1	76353	25kb
ENSG00000127241	3	186910942	187034810	396	60	120000	-0.60586	0.72769	MASP1	76353	25kb
ENSG00000136514	3	187061120	187114864	178	30	120000	-0.75924	0.77615	RTP4	76353	25kb
ENSG00000157005	3	187361694	187413187	184	28	120000	1.689	0.045607	SST	76353	25kb
ENSG00000198471	3	187391047	187445345	238	28	120000	1.5517	0.060369	RTP2	76353	25kb
ENSG00000228804	3	187395101	187476637	316	35	120000	1.5007	0.066719	RP11-211G3.3	76353	25kb
ENSG00000113916	3	187414165	187488515	258	33	120000	0.859	0.19517	BCL6	76353	25kb
ENSG00000145012	3	187846072	188633460	2295	230	120000	-1.1422	0.87332	LPP	76353	25kb
ENSG00000213132	3	187871331	187923598	182	41	120000	-1.7387	0.95896	AC022498.1	76353	25kb
ENSG00000188001	3	188640003	189068093	899	90	120000	0.44597	0.32781	TPRG1	76353	25kb
ENSG00000073282	3	189324205	189640068	1246	126	120000	-1.529	0.93687	TP63	76353	25kb
ENSG00000090530	3	189649517	189865226	804	49	120000	-1.2778	0.89933	LEPREL1	76353	25kb
ENSG00000163347	3	189998490	190065264	334	29	120000	0.066619	0.47344	CLDN1	76353	25kb
ENSG00000113946	3	190015330	190154932	641	40	120000	-0.41083	0.6594	CLDN16	76353	25kb
ENSG00000198398	3	190121444	190192665	235	31	120000	-0.92355	0.82214	TMEM207	76353	25kb
ENSG00000196083	3	190206840	190400843	600	84	120000	0.15022	0.4403	IL1RAP	76353	25kb
ENSG00000205835	3	190545666	190635218	285	37	120000	0.27197	0.39282	GMNC	76353	25kb
ENSG00000188729	3	190892030	191008404	455	34	120000	0.36092	0.35908	OSTN	76353	25kb
ENSG00000188958	3	190959957	191073325	483	43	120000	0.26001	0.39743	UTS2B	76353	25kb
ENSG00000152492	3	191021866	191141459	519	24	120000	-0.55919	0.71199	CCDC50	76353	25kb
ENSG00000253548	3	191153952	191204245	187	15	120000	-0.17708	0.57028	PYDC2	76353	25kb
ENSG00000114279	3	191832184	192510553	1906	152	120000	0.45559	0.32434	FGF12	76353	25kb
ENSG00000180611	3	192489604	192660950	531	38	120000	1.1082	0.13389	MB21D2	76353	25kb
ENSG00000127252	3	192933914	193020527	176	19	120000	-0.13145	0.55229	HRASLS	76353	25kb
ENSG00000187527	3	192967579	193121632	474	36	120000	-1.9763	0.97594	ATP13A5	76353	25kb
ENSG00000127249	3	193094866	193335900	627	55	120000	-0.0074219	0.50296	ATP13A4	76353	25kb
ENSG00000198836	3	193285933	193440612	440	26	120000	0.71398	0.23762	OPA1	76353	25kb
ENSG00000114315	3	193828934	193881521	211	31	120000	1.0361	0.15008	HES1	76353	25kb
ENSG00000178772	3	194035494	194097057	326	33	120000	-1.0383	0.85044	CPN2	76353	25kb
ENSG00000172061	3	194050976	194115472	392	38	120000	-1.2114	0.88713	LRRC15	76353	25kb
ENSG00000178732	3	194090550	194144995	211	22	120000	-1.8077	0.96467	GP5	76353	25kb
ENSG00000133657	3	194098401	194244093	482	48	120000	0.36154	0.35885	ATP13A3	76353	25kb
ENSG00000145014	3	194283402	194379418	458	42	120000	0.4575	0.32366	TMEM44	76353	25kb
ENSG00000041802	3	194336517	194418206	352	25	120000	0.33682	0.36813	LSG1	76353	25kb
ENSG00000185112	3	194381622	194434762	216	27	120000	0.11641	0.45366	FAM43A	76353	25kb
ENSG00000173950	3	194764008	195016896	926	68	120000	-0.3221	0.62631	XXYLT1	76353	25kb
ENSG00000114331	3	194970465	195188807	641	19	120000	-1.4099	0.92072	ACAP2	76353	25kb
ENSG00000184203	3	195216221	195295209	194	22	120000	-0.30376	0.61935	PPP1R2	76353	25kb
ENSG00000189058	3	195270573	195336076	234	37	120000	-1.8551	0.96821	APOD	76353	25kb
ENSG00000176945	3	195422753	195492994	134	24	120000	-1.5312	0.93714	MUC20	76353	25kb
ENSG00000145113	3	195448636	195564148	366	35	120000	-0.9132	0.81943	MUC4	76353	25kb
ENSG00000061938	3	195565235	195663816	313	42	120000	-1.4659	0.92866	TNK2	76353	25kb
ENSG00000072274	3	195729054	195834060	425	36	120000	-0.8989	0.81565	TFRC	76353	25kb
ENSG00000163958	3	195899320	195963308	275	29	120000	-0.4058	0.65756	ZDHHC19	76353	25kb
ENSG00000163959	3	195913358	195995049	294	30	120000	0.13998	0.44434	SLC51A	76353	25kb
ENSG00000161217	3	195916093	196039828	396	33	120000	0.63983	0.26114	PCYT1A	76353	25kb
ENSG00000272741	3	195950101	196070093	318	19	120000	1.1477	0.12554	RP11-447L10.1	76353	25kb
ENSG00000213123	3	195993090	196070170	206	19	120000	1.2762	0.10094	TCTEX1D2	76353	25kb
ENSG00000273331	3	196017953	196090244	176	22	120000	1.0769	0.14075	TM4SF19	76353	25kb
ENSG00000145107	3	196021213	196090374	171	22	120000	1.0769	0.14077	TM4SF19	76353	25kb
ENSG00000163960	3	196049533	196184345	266	26	120000	1.6619	0.048268	UBXN7	76353	25kb
ENSG00000163961	3	196170654	196255639	237	29	120000	1.2736	0.1014	RNF168	76353	25kb
ENSG00000214097	3	196208748	196267237	188	19	120000	0.066485	0.4735	SMCO1	76353	25kb
ENSG00000185798	3	196256056	196320545	176	22	120000	-0.49248	0.68881	WDR53	76353	25kb
ENSG00000174013	3	196270482	196340930	224	44	120000	-0.59937	0.72554	FBXO45	76353	25kb
ENSG00000174004	3	196341557	196413875	313	67	120000	-1.5758	0.94247	NRROS	76353	25kb
ENSG00000163964	3	196341646	196487878	584	77	120000	-1.5703	0.94183	PIGX	76353	25kb
ENSG00000174007	3	196408148	196464164	167	20	120000	0.032502	0.48704	CEP19	76353	25kb
ENSG00000180370	3	196441728	196584518	504	41	120000	-1.3257	0.90753	PAK2	76353	25kb
ENSG00000119231	3	196569727	196686585	317	26	120000	1.9931	0.023128	SENP5	76353	25kb
ENSG00000114503	3	196637273	196694468	163	30	120000	1.7526	0.039832	NCBP2	76353	25kb
ENSG00000119227	3	196648214	196720931	203	40	120000	1.0475	0.14742	PIGZ	76353	25kb
ENSG00000163975	3	196690492	196781687	236	49	120000	1.0547	0.14578	MFI2	76353	25kb
ENSG00000075711	3	196744431	197051171	778	32	120000	1.3755	0.084493	DLG1	76353	25kb
ENSG00000161267	3	197211654	197325194	494	54	120000	-1.544	0.93871	BDH1	76353	25kb
ENSG00000145016	3	197373264	197501598	220	19	120000	-1.1971	0.88436	KIAA0226	76353	25kb
ENSG00000122068	3	197439050	197539467	216	23	120000	-1.4349	0.92434	FYTTD1	76353	25kb
ENSG00000186001	3	197493097	197640307	345	40	120000	-1.4486	0.92627	LRCH3	76353	25kb
ENSG00000114473	3	197590946	197712013	341	22	120000	-1.1511	0.87515	IQCG	76353	25kb
ENSG00000182899	3	197651858	197708481	167	18	120000	-1.057	0.85476	RPL35A	76353	25kb
ENSG00000185621	3	197662071	197795591	359	19	120000	-1.243	0.89307	LMLN	76353	25kb
ENSG00000197701	4	28179	113099	109	15	120000	-0.10301	0.54102	ZNF595	76353	25kb
ENSG00000186777	4	239464	324110	281	25	120000	-0.62603	0.73435	ZNF732	76353	25kb
ENSG00000131127	4	306603	403653	322	23	120000	-0.59677	0.72467	ZNF141	76353	25kb
ENSG00000182903	4	394604	517945	382	25	120000	-1.24	0.89251	ZNF721	76353	25kb
ENSG00000174227	4	467989	558985	320	22	120000	-0.91149	0.81898	PIGG	76353	25kb
ENSG00000133256	4	594373	689571	268	17	120000	0.88333	0.18853	PDE6B	76353	25kb
ENSG00000169020	4	641225	693127	139	18	120000	1.2519	0.1053	ATP5I	76353	25kb
ENSG00000215375	4	642369	700822	164	25	120000	1.0929	0.13722	MYL5	76353	25kb
ENSG00000169026	4	650618	708230	156	34	120000	0.70507	0.24038	MFSD7	76353	25kb
ENSG00000185619	4	674537	789428	452	41	120000	-1.0533	0.8539	PCGF3	76353	25kb
ENSG00000168993	4	753745	844986	409	38	120000	-1.2779	0.89936	CPLX1	76353	25kb
ENSG00000178950	4	818064	951161	497	41	120000	-1.0815	0.86026	GAK	76353	25kb
ENSG00000127419	4	901175	977444	196	31	120000	-1.8078	0.96468	TMEM175	76353	25kb
ENSG00000145214	4	927675	1005683	235	40	120000	-1.5195	0.93568	DGKQ	76353	25kb
ENSG00000145217	4	947861	1012228	204	36	120000	-1.3107	0.90503	SLC26A1	76353	25kb
ENSG00000127415	4	955785	1023316	222	40	120000	-1.0468	0.85241	IDUA	76353	25kb
ENSG00000127418	4	978724	1045685	234	44	120000	0.43052	0.33341	FGFRL1	76353	25kb
ENSG00000178222	4	1025038	1132350	445	22	120000	0.1471	0.44153	RNF212	76353	25kb
ENSG00000159674	4	1135720	1227750	266	30	120000	-0.87782	0.80998	SPON2	76353	25kb
ENSG00000159692	4	1180236	1268741	274	25	120000	-1.5526	0.93974	CTBP1	76353	25kb
ENSG00000090316	4	1258639	1358935	482	26	120000	-1.8257	0.96605	MAEA	76353	25kb
ENSG00000163945	4	1316054	1406837	302	19	120000	-1.0367	0.85006	UVSSA	76353	25kb
ENSG00000179979	4	1360340	1414780	142	18	120000	-0.71739	0.76343	CRIPAK	76353	25kb
ENSG00000235608	4	1371720	1425119	136	18	120000	-0.55484	0.7105	NKX1-1	76353	25kb

ENSG00000174137	4	1594642	1711029	386	29	120000	0.42797	0.33434	FAM53A	76353	25kb
ENSG00000163950	4	1669527	1739282	260	23	120000	0.40706	0.34198	SLBP	76353	25kb
ENSG00000168936	4	1692679	1748085	240	21	120000	0.26522	0.39542	TMEM129	76353	25kb
ENSG00000013810	4	1698227	1771898	315	21	120000	0.14594	0.44199	TACC3	76353	25kb
ENSG00000068078	4	1770034	1835599	136	38	120000	1.0755	0.14109	FGFR3	76353	25kb
ENSG00000168924	4	1788206	1882974	117	40	120000	-0.017838	0.50712	LETM1	76353	25kb
ENSG00000109685	4	1848151	2008934	140	39	120000	-0.067688	0.52698	WHSC1	76353	25kb
ENSG00000185049	4	1959441	2068630	154	27	120000	-0.082552	0.5329	NELFA	76353	25kb
ENSG00000243449	4	2018689	2070697	88	17	120000	-0.44888	0.67324	C4orf48	76353	25kb
ENSG00000185818	4	2036239	2095816	79	12	120000	-0.82405	0.79504	NAT8L	76353	25kb
ENSG00000130997	4	2048645	2268848	492	16	120000	-0.85958	0.80499	POLN	76353	25kb
ENSG00000214367	4	2204191	2268891	141	12	120000	-0.78752	0.78451	HAUS3	76353	25kb
ENSG00000123933	4	2224159	2289021	163	21	120000	-1.1096	0.86641	MXD4	76353	25kb
ENSG00000159733	4	2246309	2445390	754	63	120000	-0.9668	0.83318	ZFYVE28	76353	25kb
ENSG00000249428	4	2426700	2489668	200	32	120000	-0.065192	0.52599	RP11-503N18.3	76353	25kb
ENSG00000063978	4	2438947	2652047	474	45	120000	0.8509	0.19741	RNF4	76353	25kb
ENSG00000125386	4	2601988	2759292	414	29	120000	0.96255	0.16789	FAM193A	76353	25kb
ENSG00000168884	4	2718375	2783103	228	35	120000	-0.15708	0.56241	TNIP2	76353	25kb
ENSG00000087266	4	2769750	2867825	335	39	120000	-2.2672	0.98831	SH3BP2	76353	25kb
ENSG00000087274	4	2820584	2956803	281	33	120000	-2.013	0.97794	ADD1	76353	25kb
ENSG00000109736	4	2907288	2961586	124	20	120000	-1.473	0.92962	MFSD10	76353	25kb
ENSG00000087269	4	2914660	2990112	168	19	120000	-1.5478	0.93917	NOP14	76353	25kb
ENSG00000125388	4	2940335	3067474	387	25	120000	-1.6058	0.94584	GRK4	76353	25kb
ENSG00000197386	4	3051408	3270676	526	27	120000	0.70346	0.24088	HTT	76353	25kb
ENSG00000188981	4	3221096	3298465	234	24	120000	0.93538	0.1748	MSANTD1	76353	25kb
ENSG00000159788	4	3269755	3466640	518	52	120000	0.88203	0.18888	RGS12	76353	25kb
ENSG00000109758	4	3418614	3476211	188	39	120000	-0.12884	0.55126	HGFAC	76353	25kb
ENSG00000175920	4	3440033	3528200	480	44	120000	1.9169	0.027628	DOK7	76353	25kb
ENSG00000163956	4	3483103	3559286	355	29	120000	2.3071	0.010525	LRPAP1	76353	25kb
ENSG00000268781	4	3486523	3536678	313	23	120000	2.2723	0.011534	AL590235.1	76353	25kb
ENSG00000216560	4	3553596	3617438	97	24	120000	0.68954	0.24524	LINC00955	76353	25kb
ENSG00000184160	4	3743075	3795251	206	38	120000	-0.15285	0.56074	ADRA2C	76353	25kb
ENSG00000163982	4	4165530	4253616	500	36	120000	-1.3905	0.91781	OTOP1	76353	25kb
ENSG00000132406	4	4212269	4274950	290	24	120000	-0.45664	0.67604	TMEM128	76353	25kb
ENSG00000145220	4	4244428	4316896	349	34	120000	-0.81862	0.7935	LYAR	76353	25kb
ENSG00000168826	4	4266924	4348513	350	25	120000	-0.58549	0.72089	ZBTB49	76353	25kb
ENSG00000168824	4	4324867	4445785	493	62	120000	-1.074	0.85858	NSG1	76353	25kb
ENSG00000168818	4	4392469	4569073	485	25	120000	-0.45333	0.67484	STX18	76353	25kb
ENSG00000163132	4	4836393	4890663	280	37	120000	-1.0491	0.85294	MSX1	76353	25kb
ENSG00000170891	4	4991313	5046199	232	35	120000	1.1553	0.12398	CYTL1	76353	25kb
ENSG00000152953	4	5028169	5527725	2168	119	120000	1.3288	0.09195	STK32B	76353	25kb
ENSG00000082929	4	5501296	5554528	172	20	120000	-0.33265	0.6303	C4orf6	76353	25kb
ENSG00000173040	4	5519499	5736275	849	67	120000	0.72578	0.23399	EVC2	76353	25kb
ENSG00000072840	4	5687924	5855772	837	103	120000	0.53357	0.29682	EVC	76353	25kb
ENSG00000072832	4	5724811	5919785	856	107	120000	0.063615	0.47464	CRMP1	76353	25kb
ENSG00000181215	4	5933845	6016545	512	50	120000	1.8603	0.031421	C4orf50	76353	25kb
ENSG00000152969	4	6002926	6227318	797	116	120000	0.89004	0.18672	JAKMIP1	76353	25kb
ENSG00000109501	4	6246576	6329992	363	22	120000	0.92076	0.17859	WFS1	76353	25kb
ENSG00000074211	4	6297305	6590327	1197	132	120000	1.2081	0.11351	PPP2R2C	76353	25kb
ENSG00000013288	4	6551902	6650089	284	39	120000	-0.44321	0.67119	MAN2B2	76353	25kb
ENSG00000179010	4	6616818	6669472	172	33	120000	-1.3262	0.90762	MRFAP1	76353	25kb
ENSG00000268791	4	6668782	6719189	173	37	120000	-0.90817	0.81811	AC093323.1	76353	25kb
ENSG00000163993	4	6669796	6723897	175	38	120000	-0.96452	0.83261	S100P	76353	25kb
ENSG00000178988	4	6684428	6736607	196	44	120000	-0.91802	0.8207	MRFAP1L1	76353	25kb
ENSG00000186222	4	6692842	6744387	203	42	120000	-1.4115	0.92096	BLOC1S4	76353	25kb
ENSG00000170871	4	6758102	6910897	343	57	120000	0.62242	0.26683	KIAA0232	76353	25kb
ENSG00000132405	4	6885969	7059845	619	40	120000	-0.23064	0.5912	TBC1D14	76353	25kb
ENSG00000173013	4	7017579	7069728	161	22	120000	-0.14995	0.5596	CCDC96	76353	25kb
ENSG00000173011	4	7018626	7084679	222	26	120000	-0.16203	0.56436	TADA2B	76353	25kb
ENSG00000109519	4	7035633	7094924	199	24	120000	0.11891	0.45267	GRPEL1	76353	25kb
ENSG00000184985	4	7169265	7769554	3315	291	120000	1.5013	0.066636	SORCS2	76353	25kb
ENSG00000178597	4	7407022	7461700	354	62	120000	1.379	0.08395	PSAPL1	76353	25kb
ENSG00000196526	4	7735441	7966653	1148	49	120000	-1.2203	0.88883	AFAP1	76353	25kb
ENSG00000228919	4	7915728	7967022	215	26	120000	-0.54771	0.70806	AC097381.1	76353	25kb
ENSG00000163995	4	7942039	8185559	935	132	120000	-0.49467	0.68959	ABLIM2	76353	25kb
ENSG00000125089	4	8158799	8268530	388	55	120000	-0.46834	0.68023	SH3TC1	76353	25kb
ENSG00000170801	4	8246492	8333838	422	50	120000	0.9772	0.16423	HTRA3	76353	25kb
ENSG00000087008	4	8343009	8467450	431	34	120000	0.6014	0.27379	ACOX3	76353	25kb
ENSG00000155275	4	8412867	8520258	339	23	120000	0.43945	0.33017	TRMT44	76353	25kb
ENSG00000155269	4	8535452	8646486	568	70	120000	-1.5225	0.93605	GPR78	76353	25kb
ENSG00000109625	4	8569387	8646488	395	49	120000	-1.2871	0.90097	CPZ	76353	25kb
ENSG00000215612	4	8822802	8898543	243	46	120000	0.90732	0.18212	HMX1	76353	25kb
ENSG00000229924	4	9147135	9203453	40	8	120000	-1.3023	0.90359	FAM90A26	76353	25kb
ENSG00000231396	4	9187383	9238975	1	1	120000	-0.20188	0.57999	USP17L10	76353	25kb
ENSG00000232399	4	9187383	9253214	1	1	120000	-0.20188	0.57999	USP17L13	76353	25kb
ENSG00000233136	4	9192131	9243723	1	1	120000	-0.20188	0.57999	USP17L11	76353	25kb
ENSG00000227551	4	9196878	9248470	1	1	120000	-0.20188	0.57999	USP17L12	76353	25kb
ENSG00000235780	4	9320874	9372466	26	4	120000	-0.48526	0.68625	USP17L27	76353	25kb
ENSG00000231051	4	9325619	9377211	68	8	120000	-0.63632	0.73772	USP17L28	76353	25kb
ENSG00000231637	4	9330364	9381956	92	11	120000	-0.79762	0.78745	USP17L29	76353	25kb
ENSG00000228856	4	9339855	9391447	160	15	120000	-0.90494	0.81725	USP17L30	76353	25kb
ENSG00000219492	4	9360743	9415709	276	15	120000	-0.94948	0.82881	RP11-1396O13.13	76353	25kb
ENSG00000186146	4	9421260	9477240	119	13	120000	-1.2824	0.90015	DEFB131	76353	25kb
ENSG00000109667	4	9747777	10081560	1421	37	120000	-2.086	0.98151	SLC2A9	76353	25kb
ENSG00000169676	4	9758258	9810632	143	15	120000	-1.316	0.90592	DRD5	76353	25kb
ENSG00000071127	4	10050963	10143573	479	27	120000	-0.57621	0.71776	WDR1	76353	25kb
ENSG00000178163	4	10416498	10484034	161	27	120000	0.93116	0.17588	ZNF518B	76353	25kb
ENSG00000109684	4	10463019	10711489	901	48	120000	0.46715	0.32019	CLNK	76353	25kb
ENSG00000002587	4	11369774	11456389	340	59	120000	1.6299	0.051564	HS3ST1	76353	25kb
ENSG00000157869	4	13337978	13510989	576	15	120000	1.8413	0.032791	RAB28	76353	25kb
ENSG00000109705	4	13517454	13571674	153	29	120000	1.4603	0.07211	NKX3-2	76353	25kb
ENSG00000038219	4	13545362	13654347	245	23	120000	0.23279	0.40796	BOD1L1	76353	25kb
ENSG00000137449	4	14979298	15096777	294	21	120000	-1.0456	0.85212	CPEB2	76353	25kb
ENSG00000163145	4	15316442	15472790	487	32	120000	0.11722	0.45334	C1QTNF7	76353	25kb
ENSG00000048342	4	15446489	15628180	424	40	120000	-0.11338	0.54514	CC2D2A	76353	25kb
ENSG00000118564	4	15581162	15708302	327	20	120000	0.25099	0.40091	FBXL5	76353	25kb

ENSG00000237765	4	15658285	15732188	235	26	120000	-0.29317	0.6153	FAM200B	76353	25kb
ENSG00000109743	4	15679573	15764936	288	23	120000	-0.57172	0.71625	BST1	76353	25kb
ENSG00000004468	4	15754898	15879853	350	26	120000	-0.92854	0.82344	CD38	76353	25kb
ENSG00000137440	4	15912192	15965363	179	35	120000	0.87392	0.19108	FGFBP1	76353	25kb
ENSG00000137441	4	15936866	15995932	207	38	120000	-0.022474	0.50897	FGFBP2	76353	25kb
ENSG00000007062	4	15939699	16111001	563	72	120000	0.15315	0.43914	PROM1	76353	25kb
ENSG00000169762	4	16137128	16254033	358	29	120000	0.3517	0.36253	TAPT1	76353	25kb
ENSG00000169744	4	16478164	16925432	1278	127	120000	-0.46505	0.67905	LDB2	76353	25kb
ENSG00000151552	4	17436884	17538857	438	47	120000	0.98557	0.16217	QDPR	76353	25kb
ENSG00000249581	4	17491788	17553727	242	40	120000	0.40634	0.34225	CLRN2	76353	25kb
ENSG00000002549	4	17553815	17634595	215	10	120000	0.51386	0.30367	LAP3	76353	25kb
ENSG00000118579	4	17591254	17660728	218	9	120000	0.2069	0.41805	MED28	76353	25kb
ENSG00000047662	4	17605929	17808135	502	21	120000	-0.28687	0.61289	FAM184B	76353	25kb
ENSG00000163257	4	17777278	17837381	108	11	120000	-1.6324	0.94871	DCAF16	76353	25kb
ENSG00000109805	4	17787525	17871485	143	10	120000	-1.5293	0.93691	NCAPG	76353	25kb
ENSG00000178177	4	17817822	18048499	532	16	120000	-1.492	0.93215	LCORL	76353	25kb
ENSG00000145147	4	20229883	20647184	1086	113	120000	0.77384	0.21951	SLIT2	76353	25kb
ENSG00000163138	4	20672905	20779530	323	23	120000	0.75717	0.22447	PACRGL	76353	25kb
ENSG00000185774	4	20705239	21975422	4515	196	120000	-0.61827	0.7318	KCNIP4	76353	25kb
ENSG00000152990	4	22321694	22542677	822	64	120000	2.0835	0.018603	GPR125	76353	25kb
ENSG00000109819	4	23731664	23930712	577	86	120000	1.7014	0.044434	PPARGC1A	76353	25kb
ENSG00000109606	4	24494064	24611173	299	30	120000	-0.31284	0.6228	DHX15	76353	25kb
ENSG00000109610	4	24766534	24827464	178	20	120000	-0.79628	0.78706	SOD3	76353	25kb
ENSG00000181982	4	24782739	25006826	717	63	120000	1.6983	0.044726	CCDC149	76353	25kb
ENSG00000153012	4	24975469	25057501	335	48	120000	-0.99546	0.84024	LGI2	76353	25kb
ENSG00000109618	4	25096627	25187204	223	38	120000	-1.6943	0.9549	SEPSECS	76353	25kb
ENSG00000038210	4	25137263	25305714	471	33	120000	-1.9005	0.97131	PI4K2B	76353	25kb
ENSG00000168228	4	25289407	25397005	323	21	120000	-1.4781	0.9303	ZCCHC4	76353	25kb
ENSG00000053900	4	25353835	25445120	289	18	120000	-0.83605	0.79844	ANAPC4	76353	25kb
ENSG00000157765	4	25631923	25705370	195	42	120000	-0.89155	0.81368	SLC34A2	76353	25kb
ENSG00000091490	4	25724055	25890382	491	80	120000	2.4548	0.0070476	SEL1L3	76353	25kb
ENSG00000250317	4	25838452	25956435	338	37	120000	1.0453	0.14793	SMIM20	76353	25kb
ENSG00000168214	4	26140077	26461541	613	57	120000	0.0084484	0.49663	RBPJ	76353	25kb
ENSG00000163394	4	26458022	26517084	200	32	120000	0.48632	0.31337	CCKAR	76353	25kb
ENSG00000109680	4	26553059	26781973	290	22	120000	2.0941	0.018126	TBC1D19	76353	25kb
ENSG00000109689	4	26834300	27052003	451	34	120000	-0.20206	0.58006	STIM2	76353	25kb
ENSG00000250038	4	28338901	28429486	294	21	120000	0.9776	0.16414	RP11-180C1.1	76353	25kb
ENSG00000169851	4	30697037	31173422	1373	66	120000	-1.2691	0.8978	PCDH7	76353	25kb
ENSG00000047365	4	35924843	36271131	961	65	120000	-0.25201	0.59948	ARAP2	76353	25kb
ENSG00000197057	4	36258244	36372378	414	45	120000	-0.26164	0.6032	DTHD1	76353	25kb
ENSG00000174145	4	37220842	37476087	854	78	120000	0.878	0.18997	KIAA1239	76353	25kb
ENSG00000154274	4	37430563	37650117	833	82	120000	0.23188	0.40831	C4orf19	76353	25kb
ENSG00000181826	4	37567422	37712998	531	67	120000	0.84487	0.19909	RELL1	76353	25kb
ENSG00000169299	4	37803255	37889558	360	25	120000	-1.3225	0.90701	PGM2	76353	25kb
ENSG00000065882	4	37867708	38165796	967	96	120000	1.2013	0.11482	TBC1D1	76353	25kb
ENSG00000250254	4	37937056	37987746	171	28	120000	-0.14431	0.55737	PTTG2	76353	25kb
ENSG00000196355	4	38603029	38691430	262	27	120000	0.5904	0.27746	AC021860.1	76353	25kb
ENSG00000109787	4	38640817	38727663	244	39	120000	1.3678	0.085687	KLF3	76353	25kb
ENSG00000174123	4	38748860	38809611	304	20	120000	-0.17194	0.56826	TLR10	76353	25kb
ENSG00000174125	4	38767298	38883438	474	25	120000	-0.62445	0.73383	TLR1	76353	25kb
ENSG00000174130	4	38800336	38883438	297	16	120000	-0.57522	0.71743	TLR6	76353	25kb
ENSG00000197712	4	38844298	38972360	480	37	120000	-0.95938	0.83132	FAM114A1	76353	25kb
ENSG00000121895	4	38943365	39059542	487	61	120000	-0.36156	0.64116	TMEM156	76353	25kb
ENSG00000109790	4	39021659	39153477	389	14	120000	-0.69356	0.75602	KLHL5	76353	25kb
ENSG00000157796	4	39159024	39312430	267	11	120000	-0.93979	0.82634	WDR19	76353	25kb
ENSG00000035928	4	39264076	39392995	209	15	120000	-0.61797	0.7317	RFC1	76353	25kb
ENSG00000134962	4	39383473	39478156	287	35	120000	0.6635	0.2535	KLB	76353	25kb
ENSG00000163682	4	39430744	39485568	207	26	120000	0.78426	0.21644	RPL9	76353	25kb
ENSG00000121897	4	39435620	39504273	267	23	120000	0.76059	0.22345	LIAS	76353	25kb
ENSG00000109814	4	39475375	39554931	326	33	120000	-0.40583	0.65757	UGDH	76353	25kb
ENSG00000163683	4	39522950	39665710	376	38	120000	-0.59276	0.72333	SMIM14	76353	25kb
ENSG00000078140	4	39674664	39809412	397	29	120000	-1.5601	0.94064	UBE2K	76353	25kb
ENSG00000121892	4	39799483	40004576	595	34	120000	0.063095	0.47485	PDS5A	76353	25kb
ENSG00000078177	4	40033446	40184872	415	34	120000	-1.0673	0.85708	N4BP2	76353	25kb
ENSG00000168421	4	40167673	40273587	322	53	120000	-0.46041	0.67739	RHOH	76353	25kb
ENSG00000174343	4	40312346	40382234	314	25	120000	-0.87815	0.81007	CHRNA9	76353	25kb
ENSG00000163694	4	40400272	40657892	756	109	120000	-2.3758	0.99125	RBM47	76353	25kb
ENSG00000179299	4	40726914	40837002	255	22	120000	-0.5936	0.72361	NSUN7	76353	25kb
ENSG00000163697	4	40787044	41243731	1584	100	120000	-0.047145	0.5188	APBB2	76353	25kb
ENSG00000154277	4	41233430	41295472	227	27	120000	2.2	0.013905	UCHL1	76353	25kb
ENSG00000064042	4	41336624	41727061	1004	90	120000	0.22704	0.4102	LIMCH1	76353	25kb
ENSG00000109132	4	41721099	41775987	191	21	120000	1.0475	0.14743	PHOX2B	76353	25kb
ENSG00000109133	4	41912137	41987589	224	17	120000	0.31864	0.375	TMEM33	76353	25kb
ENSG00000182308	4	41958713	42013476	196	16	120000	1.248	0.10602	DCAF4L1	76353	25kb
ENSG00000014824	4	41967489	42117474	523	14	120000	0.56192	0.28709	SLC30A9	76353	25kb
ENSG00000188848	4	42087955	42179895	266	20	120000	1.1745	0.12009	BEND4	76353	25kb
ENSG00000178343	4	42374856	42429504	205	28	120000	0.78068	0.2175	SHISA3	76353	25kb
ENSG00000124406	4	42385390	42684122	1016	44	120000	0.62231	0.26687	ATP8A1	76353	25kb
ENSG00000215203	4	42870284	43057675	620	40	120000	-0.23598	0.59328	GRXCR1	76353	25kb
ENSG00000183783	4	44150926	44475824	778	54	120000	-0.97098	0.83422	KCTD8	76353	25kb
ENSG00000177752	4	44599086	44705573	390	22	120000	-0.64382	0.74015	YIPF7	76353	25kb
ENSG00000151806	4	44655444	44727943	202	12	120000	-1.1428	0.87344	GUF1	76353	25kb
ENSG00000163281	4	44659217	44753612	250	13	120000	-0.94759	0.82833	GNPDA2	76353	25kb
ENSG00000163285	4	46012786	46151098	413	22	120000	0.097176	0.46129	GABRG1	76353	25kb
ENSG00000151834	4	46225444	46502247	589	26	120000	-0.96652	0.83311	GABRA2	76353	25kb
ENSG00000170516	4	46711844	46936252	483	28	120000	0.43715	0.331	COX7B2	76353	25kb
ENSG00000109158	4	46895917	47021424	393	26	120000	-1.3128	0.90537	GABRA4	76353	25kb
ENSG00000163288	4	46970740	47453461	1140	81	120000	-2.0899	0.98169	GABRB1	76353	25kb
ENSG00000169019	4	47427885	47490736	151	20	120000	-0.50524	0.6933	COMMD8	76353	25kb
ENSG00000145246	4	47462305	47620503	527	46	120000	0.53406	0.29665	ATP10D	76353	25kb
ENSG00000145244	4	47571015	47865123	709	37	120000	-0.27945	0.61005	CORIN	76353	25kb
ENSG00000170448	4	47824257	47941653	275	15	120000	-0.64528	0.74063	NFXL1	76353	25kb
ENSG00000163293	4	47891159	48067188	498	28	120000	-1.068	0.85723	NIPAL1	76353	25kb
ENSG00000198515	4	47912994	48043689	391	23	120000	-0.95794	0.83095	CNGA1	76353	25kb
ENSG00000074966	4	48043410	48161273	269	23	120000	-0.44714	0.67261	TXK	76353	25kb
ENSG00000135605	4	48112800	48296881	600	38	120000	-0.5861	0.72109	TEC	76353	25kb

ENSG00000109171	4	48318339	48453229	340	21	120000	-0.38325	0.64923	SLAIN2	76353	25kb
ENSG00000145248	4	48460360	48516213	110	14	120000	0.50126	0.30809	SLC10A4	76353	25kb
ENSG00000182223	4	48467269	48521406	94	13	120000	0.3339	0.36923	ZAR1	76353	25kb
ENSG00000075539	4	48474378	48807339	461	28	120000	-0.29551	0.6162	FRYL	76353	25kb
ENSG00000109180	4	48782229	48888834	143	21	120000	-0.27449	0.60815	OCIAD1	76353	25kb
ENSG00000145247	4	48862036	48933954	107	16	120000	-1.0809	0.86014	OCIAD2	76353	25kb
ENSG00000109182	4	48963264	49089098	213	18	120000	-0.92343	0.82211	CWH43	76353	25kb
ENSG00000109184	4	52684166	52808003	166	13	120000	-1.3169	0.90606	DCUN1D4	76353	25kb
ENSG00000188993	4	52834868	52908786	101	13	120000	-0.83614	0.79846	LRRC66	76353	25kb
ENSG00000163069	4	52861872	52929648	78	12	120000	-0.47563	0.68283	SGCB	76353	25kb
ENSG00000163071	4	52892497	52988458	197	22	120000	0.98242	0.16295	SPATA18	76353	25kb
ENSG00000109189	4	53432138	53550502	163	9	120000	0.59501	0.27592	USP46	76353	25kb
ENSG00000226887	4	53563785	53642807	155	14	120000	0.5495	0.29133	ERVMER34-1	76353	25kb
ENSG00000128045	4	53703457	53758000	156	17	120000	2.3442	0.0095327	RASL11B	76353	25kb
ENSG00000184178	4	53714149	54257242	1220	70	120000	1.9183	0.027535	SCFD2	76353	25kb
ENSG00000145216	4	54218810	55186439	2609	148	120000	-0.72011	0.76427	FIP1L1	76353	25kb
ENSG00000072201	4	54300468	54592572	926	72	120000	0.075626	0.46986	LNX1	76353	25kb
ENSG00000109220	4	54850956	54955857	133	13	120000	0.18621	0.42614	CHIC2	76353	25kb
ENSG00000269506	4	54902213	54952377	62	11	120000	0.071991	0.4713	AC110792.1	76353	25kb
ENSG00000180613	4	54940690	54993672	95	14	120000	-1.1919	0.88334	GSX2	76353	25kb
ENSG00000134853	4	55070264	55189414	271	21	120000	-0.50618	0.69364	PDGFRA	76353	25kb
ENSG00000157404	4	55499085	55631881	373	38	120000	-0.89194	0.81379	KIT	76353	25kb
ENSG00000128052	4	55919644	56016756	256	42	120000	-0.3886	0.65121	KDR	76353	25kb
ENSG00000128039	4	56187276	56264263	207	27	120000	-0.90046	0.81606	SRD5A3	76353	25kb
ENSG00000134851	4	56237124	56344564	306	19	120000	-0.41404	0.66058	TMEM165	76353	25kb
ENSG00000134852	4	56269070	56438305	566	17	120000	-0.089664	0.53572	CLOCK	76353	25kb
ENSG00000163440	4	56397692	56483379	318	20	120000	-0.25557	0.60086	PDCL2	76353	25kb
ENSG00000109255	4	56436396	56527865	317	24	120000	-0.46748	0.67992	NMU	76353	25kb
ENSG00000090989	4	56694782	56796200	252	27	120000	-0.084066	0.5335	EXOC1	76353	25kb
ENSG00000174799	4	56790037	56924529	301	25	120000	0.83471	0.20194	CEP135	76353	25kb
ENSG00000109265	4	57011361	57219791	695	85	120000	-0.10158	0.54045	KIAA1211	76353	25kb
ENSG00000157426	4	57179456	57278666	285	17	120000	0.50683	0.30614	AASDH	76353	25kb
ENSG00000128059	4	57234528	57326781	245	25	120000	0.48542	0.31369	PPAT	76353	25kb
ENSG00000268171	4	57251682	57302131	129	15	120000	0.65423	0.25648	AC068620.1	76353	25kb
ENSG00000128050	4	57276907	57352534	199	22	120000	0.31255	0.37731	PAICS	76353	25kb
ENSG00000174780	4	57308081	57394839	244	22	120000	0.33343	0.3694	SRP72	76353	25kb
ENSG00000196503	4	57346509	57415614	205	24	120000	0.60479	0.27266	ARL9	76353	25kb
ENSG00000249693	4	57371775	57494673	367	45	120000	0.68238	0.2475	THEGL	76353	25kb
ENSG00000171476	4	57489155	57573065	315	42	120000	0.54279	0.29364	HOPX	76353	25kb
ENSG00000128040	4	57651026	57712908	273	36	120000	-0.066658	0.52657	SPINK2	76353	25kb
ENSG00000084093	4	57749075	57827010	244	22	120000	0.36029	0.35931	REST	76353	25kb
ENSG00000084092	4	57804536	57869989	207	27	120000	0.60928	0.27117	NOA1	76353	25kb
ENSG00000047315	4	57818888	57922334	395	36	120000	1.2345	0.10851	POLR2B	76353	25kb
ENSG00000163453	4	57871939	58001551	559	48	120000	1.2227	0.11073	IGFBP7	76353	25kb
ENSG00000150471	4	62041976	62969053	2050	132	120000	1.3852	0.082996	LPHN3	76353	25kb
ENSG00000205678	4	65115975	65300186	598	24	120000	0.299	0.38247	TECRL	76353	25kb
ENSG00000145242	4	66160281	66561213	1503	73	120000	-1.3949	0.91848	EPHA5	76353	25kb
ENSG00000145241	4	68312521	68436324	389	24	120000	-0.4838	0.68574	CENPC	76353	25kb
ENSG00000035720	4	68399446	68498055	333	38	120000	-1.2554	0.89533	STAP1	76353	25kb
ENSG00000033178	4	68453370	68591897	420	22	120000	-0.24115	0.59528	UBA6	76353	25kb
ENSG00000109163	4	68580046	68645078	173	20	120000	-0.48679	0.6868	GNRHR	76353	25kb
ENSG00000153802	4	68661594	68774750	272	30	120000	-0.82874	0.79637	TMPRSS11D	76353	25kb
ENSG00000187054	4	68750103	68854858	293	24	120000	0.20918	0.41715	TMPRSS11A	76353	25kb
ENSG00000198092	4	68893916	69020598	383	16	120000	0.097616	0.46112	TMPRSS11F	76353	25kb
ENSG00000226894	4	69029242	69108798	184	12	120000	-0.26599	0.60488	TMPRSS11BNL	76353	25kb
ENSG00000185873	4	69067371	69136438	135	10	120000	-0.26139	0.6031	TMPRSS11B	76353	25kb
ENSG00000083896	4	69151105	69240807	143	20	120000	-0.13084	0.55205	YTHDC1	76353	25kb
ENSG00000087128	4	69288167	69388322	387	32	120000	0.50032	0.30842	TMPRSS11E	76353	25kb
ENSG00000197888	4	69377902	69459245	26	6	120000	1.0028	0.15797	UGT2B17	76353	25kb
ENSG00000196620	4	69487348	69561346	263	33	120000	1.2678	0.10243	UGT2B15	76353	25kb
ENSG00000109181	4	69656711	69721914	265	15	120000	-0.74723	0.77254	UGT2B10	76353	25kb
ENSG00000135220	4	69769181	69842509	304	15	120000	-0.11159	0.54443	UGT2A3	76353	25kb
ENSG00000171234	4	69892081	70003705	500	16	120000	-0.62641	0.73448	UGT2B7	76353	25kb
ENSG00000213759	4	70040669	70105449	333	18	120000	-1.7317	0.95834	UGT2B11	76353	25kb
ENSG00000135226	4	70121217	70185768	40	9	120000	-1.3816	0.91645	UGT2B28	76353	25kb
ENSG00000156096	4	70320883	70416732	499	28	120000	-1.518	0.93549	UGT2B4	76353	25kb
ENSG00000271271	4	70429135	70530360	471	30	120000	-0.67143	0.74903	UGT2A2	76353	25kb
ENSG00000173610	4	70429135	70543965	514	32	120000	-0.40124	0.65588	UGT2A1	76353	25kb
ENSG00000270386	4	70429912	70543967	505	31	120000	-0.36676	0.6431	UGT2A1	76353	25kb
ENSG00000173597	4	70561880	70678679	409	28	120000	1.0444	0.14815	SULT1B1	76353	25kb
ENSG00000109193	4	70651498	70750870	279	29	120000	1.1324	0.12872	SULT1E1	76353	25kb
ENSG00000126545	4	70771799	70837289	169	18	120000	-0.13947	0.55546	CSN1S1	76353	25kb
ENSG00000135222	4	70795974	70851725	144	16	120000	-0.37071	0.64457	CSN2	76353	25kb
ENSG00000126549	4	70836647	70893287	122	17	120000	0.35298	0.36205	STATH	76353	25kb
ENSG00000205649	4	70869130	70927255	174	20	120000	0.38843	0.34885	HTN3	76353	25kb
ENSG00000126550	4	70891119	70949562	193	20	120000	0.56394	0.2864	HTN1	76353	25kb
ENSG00000187533	4	70974333	71067516	326	36	120000	1.2277	0.10979	C4orf40	76353	25kb
ENSG00000109205	4	71037213	71095293	191	17	120000	0.031585	0.4874	ODAM	76353	25kb
ENSG00000181617	4	71066788	71125969	264	12	120000	-0.2962	0.61646	FDCSP	76353	25kb
ENSG00000171209	4	71083305	71142145	291	13	120000	0.27041	0.39342	CSN3	76353	25kb
ENSG00000145309	4	71175683	71227833	299	19	120000	0.92062	0.17862	CABS1	76353	25kb
ENSG00000109208	4	71201478	71257875	360	19	120000	0.6951	0.2435	SMR3A	76353	25kb
ENSG00000171201	4	71210810	71280961	370	25	120000	0.57048	0.28418	SMR3B	76353	25kb
ENSG00000171199	4	71238599	71300911	251	30	120000	0.17541	0.43038	PROL1	76353	25kb
ENSG00000171195	4	71271209	71373714	442	25	120000	0.51542	0.30313	MUC7	76353	25kb
ENSG00000187689	4	71359257	71423459	377	18	120000	0.92024	0.17872	AMTN	76353	25kb
ENSG00000178522	4	71432973	71498005	229	39	120000	1.3618	0.086631	AMBN	76353	25kb
ENSG00000132464	4	71469461	71577533	235	32	120000	1.9112	0.027989	ENAM	76353	25kb
ENSG00000132465	4	71496258	71572534	129	23	120000	1.7517	0.039913	IGJ	76353	25kb
ENSG00000132467	4	71529196	71581267	96	13	120000	1.1092	0.13367	UTP3	76353	25kb
ENSG00000018189	4	71544921	71698032	433	16	120000	1.0183	0.15427	RUFY3	76353	25kb
ENSG00000132463	4	71656499	71730662	199	15	120000	1.2045	0.1142	GRSF1	76353	25kb
ENSG00000173542	4	71743043	71913166	283	19	120000	0.20982	0.4169	MOB1B	76353	25kb
ENSG00000156136	4	71833255	71921631	151	17	120000	-0.85801	0.80456	DCK	76353	25kb
ENSG00000080493	4	72028003	72462804	810	59	120000	0.45098	0.326	SLC4A4	76353	25kb
ENSG00000145321	4	72582410	72694758	252	29	120000	1.8207	0.034328	GC	76353	25kb

ENSG00000056291	4	72872521	73038784	449	17	120000	0.071628	0.47145	NPFFR2	76353	25kb
ENSG00000156140	4	73121686	73459516	655	48	120000	0.63644	0.26224	ADAMTS3	76353	25kb
ENSG00000163626	4	73896797	73960472	50	10	120000	1.8495	0.032192	COX18	76353	25kb
ENSG00000132466	4	73914093	74149515	191	30	120000	2.0413	0.020611	ANKRD17	76353	25kb
ENSG00000163631	4	74237831	74312129	165	16	120000	1.4046	0.080073	ALB	76353	25kb
ENSG00000081051	4	74271855	74346891	177	20	120000	0.93172	0.17574	AFP	76353	25kb
ENSG00000079557	4	74322400	74394691	198	19	120000	-0.53204	0.70265	AFM	76353	25kb
ENSG00000169435	4	74412267	74511348	236	22	120000	0.37792	0.35275	RASSF6	76353	25kb
ENSG00000169429	4	74581223	74634433	152	16	120000	1.1607	0.12287	IL8	76353	25kb
ENSG00000124875	4	74677214	74739781	147	28	120000	-0.66181	0.74595	CXCL6	76353	25kb
ENSG00000109272	4	74693906	74744872	137	24	120000	-0.8644	0.80632	PF4V1	76353	25kb
ENSG00000163739	4	74710110	74761959	167	20	120000	-0.82796	0.79615	CXCL1	76353	25kb
ENSG00000163737	4	74821794	74872841	105	11	120000	-0.13262	0.55275	PF4	76353	25kb
ENSG00000163736	4	74827755	74878914	98	10	120000	-0.19394	0.57689	PPBP	76353	25kb
ENSG00000163735	4	74836359	74889496	97	13	120000	-0.45632	0.67592	CXCL5	76353	25kb
ENSG00000163734	4	74877306	74929524	50	17	120000	-0.70787	0.76049	CXCL3	76353	25kb
ENSG00000081041	4	74937752	74990010	64	14	120000	-0.40901	0.65874	CXCL2	76353	25kb
ENSG00000163738	4	74954891	75193816	364	31	120000	-1.9802	0.97616	MTHFD2L	76353	25kb
ENSG00000269559	4	74998327	75049085	59	10	120000	-1.3999	0.91923	AC093677.1	76353	25kb
ENSG00000182585	4	75149190	75206024	151	19	120000	-1.9481	0.9743	EPGN	76353	25kb
ENSG00000124882	4	75205860	75279468	324	21	120000	-1.1202	0.86868	EREG	76353	25kb
ENSG00000109321	4	75285851	75345726	75	14	120000	-0.71298	0.76207	AREG	76353	25kb
ENSG00000205595	4	75455629	75515486	81	11	120000	-0.32432	0.62715	AREGB	76353	25kb
ENSG00000174808	4	75644969	75744896	329	24	120000	0.8182	0.20662	BTC	76353	25kb
ENSG00000169116	4	75833305	76000325	312	34	120000	-2.3714	0.99114	PARM1	76353	25kb
ENSG00000163743	4	76379247	76464974	179	19	120000	0.57351	0.28315	RCHY1	76353	25kb
ENSG00000174796	4	76414156	76500683	225	29	120000	0.32609	0.37218	THAP6	76353	25kb
ENSG00000174792	4	76456258	76516095	171	28	120000	1.0543	0.14586	C4orf26	76353	25kb
ENSG00000138769	4	76478215	76580900	236	25	120000	2.1465	0.015917	CDKL2	76353	25kb
ENSG00000138757	4	76542966	76674709	305	18	120000	1.1114	0.1332	G3BP2	76353	25kb
ENSG00000138768	4	76624777	76760382	305	17	120000	-0.56563	0.71418	USO1	76353	25kb
ENSG00000156194	4	76756020	76848724	283	29	120000	-0.55356	0.71006	PPEF2	76353	25kb
ENSG00000138744	4	76806809	76887204	284	27	120000	-0.69091	0.75519	NAAA	76353	25kb
ENSG00000198301	4	76837103	76937115	254	19	120000	-0.016235	0.50648	SDAD1	76353	25kb
ENSG00000268788	4	76846883	76903526	144	16	120000	0.12063	0.45199	AC110615.1	76353	25kb
ENSG00000138755	4	76897428	76953641	146	13	120000	-0.18484	0.57332	CXCL9	76353	25kb
ENSG00000156219	4	76907337	77058955	721	23	120000	0.11058	0.45598	ART3	76353	25kb
ENSG00000169245	4	76917273	76969650	195	8	120000	-0.28067	0.61052	CXCL10	76353	25kb
ENSG00000169248	4	76929835	76987568	280	6	120000	-0.21374	0.58462	CXCL11	76353	25kb
ENSG00000138750	4	77010812	77094668	432	14	120000	0.63403	0.26303	NUP54	76353	25kb
ENSG00000138760	4	77054890	77160046	458	30	120000	1.4252	0.077056	SCARB2	76353	25kb
ENSG00000189157	4	77110193	77229933	442	33	120000	0.5583	0.28832	FAM47E	76353	25kb
ENSG00000272414	4	77147874	77257282	396	26	120000	0.736	0.23087	FAM47E-STBD1	76353	25kb
ENSG00000118804	4	77147886	77257752	396	26	120000	0.736	0.23087	FAM47E-STBD1	76353	25kb
ENSG00000163749	4	77209154	77368021	286	21	120000	1.4898	0.068139	CCDC158	76353	25kb
ENSG00000138771	4	77331253	77729406	1094	62	120000	0.27685	0.39095	SHROOM3	76353	25kb
ENSG00000186212	4	77791082	77844002	170	20	120000	1.7119	0.04346	SOWAHB	76353	25kb
ENSG00000138758	4	77845856	77986537	355	28	120000	0.80781	0.2096	SEPT11	76353	25kb
ENSG00000118816	4	77943311	78022158	143	21	120000	-0.10795	0.54298	CCNI	76353	25kb
ENSG00000138764	4	78053304	78379542	1594	36	120000	1.8801	0.030049	CCNG2	76353	25kb
ENSG00000156234	4	78407907	78557988	242	49	120000	-0.15227	0.56051	CXCL13	76353	25kb
ENSG00000138767	4	78609541	78765769	265	19	120000	-0.053803	0.52145	CNOT6L	76353	25kb
ENSG00000169288	4	78758674	78898944	340	18	120000	-0.13432	0.55342	MRPL1	76353	25kb
ENSG00000138759	4	78953724	79490423	1815	60	120000	0.021784	0.49131	FRAS1	76353	25kb
ENSG00000138772	4	79447673	79556597	326	31	120000	-0.57299	0.71667	ANXA3	76353	25kb
ENSG00000138756	4	79672496	79862526	307	14	120000	-0.63955	0.73877	BMP2K	76353	25kb
ENSG00000163291	4	79783281	79885592	208	14	120000	-0.60995	0.72905	PAQR3	76353	25kb
ENSG00000156269	4	80121848	80272204	280	26	120000	-0.10043	0.54	NAA11	76353	25kb
ENSG00000196475	4	80302508	80354372	86	20	120000	-0.42099	0.66312	GK2	76353	25kb
ENSG00000163297	4	80797303	81071608	679	48	120000	-0.31713	0.62443	ANTXR2	76353	25kb
ENSG00000152784	4	81080033	81150483	264	22	120000	-1.1759	0.88018	PRDM8	76353	25kb
ENSG00000138675	4	81162753	81282834	302	32	120000	-1.0481	0.8527	FGF5	76353	25kb
ENSG00000197826	4	81231874	81909910	1489	43	120000	-0.41299	0.66019	C4orf22	76353	25kb
ENSG00000152785	4	81927119	82003685	179	22	120000	1.3991	0.080897	BMP3	76353	25kb
ENSG00000138669	4	81984837	82161218	400	30	120000	1.9542	0.025337	PRKG2	76353	25kb
ENSG00000138670	4	82322547	82990397	2155	108	120000	1.9155	0.027712	RASGEF1B	76353	25kb
ENSG00000138668	4	83248651	83320656	202	26	120000	-0.068831	0.52744	HNRNPD	76353	25kb
ENSG00000152795	4	83318717	83376294	158	22	120000	0.10509	0.45815	HNRNPDL	76353	25kb
ENSG00000145293	4	83326715	83407244	202	33	120000	-0.53532	0.70379	ENOPH1	76353	25kb
ENSG00000249242	4	83379323	83508510	323	48	120000	-1.9084	0.97183	TMEM150C	76353	25kb
ENSG00000145284	4	83525692	83745010	975	64	120000	-1.991	0.97676	SCD5	76353	25kb
ENSG00000138674	4	83714814	83847319	282	15	120000	-0.03498	0.51395	SEC31A	76353	25kb
ENSG00000168152	4	83796837	83866122	144	14	120000	-0.188	0.57456	THAP9	76353	25kb
ENSG00000189308	4	83806126	83959079	313	14	120000	-0.36874	0.64384	LIN54	76353	25kb
ENSG00000138663	4	83930600	84021971	141	23	120000	0.67359	0.25029	COPS4	76353	25kb
ENSG00000145287	4	83986201	84083228	172	42	120000	1.1891	0.1172	PLAC8	76353	25kb
ENSG00000173085	4	84157689	84231067	235	17	120000	0.4378	0.33076	COQ2	76353	25kb
ENSG00000173083	4	84188614	84281306	204	26	120000	1.9644	0.024741	HPSE	76353	25kb
ENSG00000163312	4	84303496	84402009	196	20	120000	0.14908	0.44075	HELQ	76353	25kb
ENSG00000163319	4	84352085	84415888	125	10	120000	-0.089837	0.53579	MRPS18C	76353	25kb
ENSG00000163322	4	84357092	84469501	295	10	120000	0.40705	0.34199	FAM175A	76353	25kb
ENSG00000138678	4	84432067	84552028	355	16	120000	0.49548	0.31013	AGPAT9	76353	25kb
ENSG00000163623	4	85388140	85444603	87	18	120000	1.389	0.082411	NKX6-1	76353	25kb
ENSG00000163624	4	85479132	85597491	202	22	120000	0.53412	0.29663	CDS1	76353	25kb
ENSG00000163625	4	85565704	85912544	374	25	120000	-0.12308	0.54898	WDFY3	76353	25kb
ENSG00000138639	4	86371267	86948823	1773	81	120000	-0.71741	0.76344	ARHGAP24	76353	25kb
ENSG00000109339	4	86911276	87540284	1680	61	120000	-0.30667	0.62045	MAPK10	76353	25kb
ENSG00000163629	4	87490468	87761324	672	17	120000	0.085933	0.46576	PTPN13	76353	25kb
ENSG00000145283	4	87719621	87795416	130	17	120000	0.31156	0.37769	SLC10A6	76353	25kb
ENSG00000163633	4	87772358	87882354	265	23	120000	0.87112	0.19184	C4orf36	76353	25kb
ENSG00000172493	4	87831154	88087206	590	38	120000	0.046055	0.48163	AFF1	76353	25kb
ENSG00000145332	4	88056255	88186466	282	25	120000	-1.0086	0.84342	KLHL8	76353	25kb
ENSG00000170509	4	88199941	88269058	229	28	120000	-0.72273	0.76508	HSD17B13	76353	25kb
ENSG00000198189	4	88232762	88337538	275	25	120000	-0.10519	0.54189	HSD17B11	76353	25kb
ENSG00000170502	4	88318734	88405606	218	26	120000	1.4961	0.067312	NUDT9	76353	25kb
ENSG00000152583	4	88369487	88477213	339	21	120000	0.075523	0.4699	SPARCL1	76353	25kb

ENSG00000152591	4	88504681	88563062	146	27	120000	0.61381	0.26967	DSPP	76353	25kb
ENSG00000152592	4	88546459	88610513	186	20	120000	1.0889	0.13811	DMP1	76353	25kb
ENSG00000029559	4	88695733	88758074	225	36	120000	-0.42547	0.66475	IBSP	76353	25kb
ENSG00000152595	4	88717563	88792969	338	28	120000	-0.20045	0.57944	MEPE	76353	25kb
ENSG00000118785	4	88871819	88929562	189	22	120000	-1.777	0.96222	SPP1	76353	25kb
ENSG00000118762	4	88903820	89023929	311	32	120000	-1.7211	0.95739	PKD2	76353	25kb
ENSG00000118777	4	88986416	89177474	564	50	120000	-0.70335	0.75908	ABCG2	76353	25kb
ENSG00000163644	4	89153772	89230921	187	34	120000	-0.4923	0.68875	PPM1K	76353	25kb
ENSG00000138642	4	89274891	89389263	193	49	120000	-0.68308	0.75272	HERC6	76353	25kb
ENSG00000138646	4	89353268	89452314	77	27	120000	-1.7966	0.9638	HERC5	76353	25kb
ENSG00000145337	4	89417136	89469964	69	22	120000	-1.5887	0.94394	PYURF	76353	25kb
ENSG00000138641	4	89417199	89654693	505	33	120000	-1.0721	0.85816	HERC3	76353	25kb
ENSG00000255072	4	89417724	89467940	62	20	120000	-1.5346	0.93756	PIGY	76353	25kb
ENSG00000177432	4	89592066	89644386	127	20	120000	-0.40298	0.65652	NAP1L5	76353	25kb
ENSG00000138640	4	89622106	90057549	1309	50	120000	-1.0259	0.84752	FAM13A	76353	25kb
ENSG00000180346	4	90008968	90061050	188	23	120000	-0.92338	0.8221	TIGD2	76353	25kb
ENSG00000185477	4	90132537	90254161	347	51	120000	-1.857	0.96834	GPRIN3	76353	25kb
ENSG00000145335	4	90620250	90784466	610	26	120000	-0.09876	0.53934	SNCA	76353	25kb
ENSG00000138722	4	90775683	90900780	323	26	120000	1.6598	0.048473	MMRN1	76353	25kb
ENSG00000184305	4	91023686	92548064	4271	165	120000	0.22663	0.41035	CCSER1	76353	25kb
ENSG00000249049	4	92215185	92271457	180	19	120000	-0.52013	0.69851	RP11-763F8.1	76353	25kb
ENSG00000248511	4	93193402	93250329	86	22	120000	-1.1864	0.88227	RP11-9B6.1	76353	25kb
ENSG00000152208	4	93200550	94720707	3772	126	120000	-0.18288	0.57255	GRID2	76353	25kb
ENSG00000172238	4	94725042	94776221	178	25	120000	-0.92271	0.82192	ATOH1	76353	25kb
ENSG00000163104	4	95103762	95237443	276	12	120000	0.28614	0.38739	SMARCAD1	76353	25kb
ENSG00000163106	4	95194686	95289027	200	14	120000	-0.25617	0.60109	HPGDS	76353	25kb
ENSG00000163110	4	95348037	95614377	632	35	120000	0.63654	0.26221	PDLIM5	76353	25kb
ENSG00000138696	4	95654119	96104599	1336	57	120000	-0.69789	0.75738	BMPR1B	76353	25kb
ENSG00000182168	4	96058655	96495357	1433	137	120000	-0.0021098	0.50084	UNC5C	76353	25kb
ENSG00000163114	4	96736239	96787625	249	15	120000	2.3522	0.0093317	PDHA2	76353	25kb
ENSG00000163116	4	98080244	99089391	2547	33	120000	-1.2995	0.90311	STPG2	76353	25kb
ENSG00000138698	4	99157535	99390012	490	18	120000	0.52808	0.29872	RAP1GDS1	76353	25kb
ENSG00000168785	4	99366518	99604780	672	78	120000	-0.16019	0.56363	TSPAN5	76353	25kb
ENSG00000151247	4	99767835	99876788	319	21	120000	0.42777	0.33441	EIF4E	76353	25kb
ENSG00000164024	4	99891771	100008964	342	19	120000	0.67814	0.24884	METAP1	76353	25kb
ENSG00000197894	4	99967132	100034952	282	15	120000	0.2905	0.38572	ADH5	76353	25kb
ENSG00000198099	4	100019808	100103949	385	14	120000	0.55803	0.28841	ADH4	76353	25kb
ENSG00000172955	4	100098795	100165694	172	24	120000	1.7797	0.037563	ADH6	76353	25kb
ENSG00000187758	4	100172524	100237185	196	22	120000	1.367	0.085813	ADH1A	76353	25kb
ENSG00000196616	4	100201121	100267558	237	25	120000	1.1646	0.12209	ADH1B	76353	25kb
ENSG00000196344	4	100308418	100381894	235	44	120000	-0.91146	0.81897	ADH7	76353	25kb
ENSG00000138813	4	100407161	100488460	329	28	120000	-0.46906	0.68049	C4orf17	76353	25kb
ENSG00000145331	4	100442866	100510189	274	18	120000	-0.025674	0.51024	TRMT10A	76353	25kb
ENSG00000138823	4	100459918	100570156	439	32	120000	-0.15531	0.56171	MTTP	76353	25kb
ENSG00000248713	4	100532686	100600805	233	32	120000	0.26648	0.39493	RP11-766F14.2	76353	25kb
ENSG00000070190	4	100712990	100816311	303	32	120000	-1.2653	0.89712	DAPP1	76353	25kb
ENSG00000109270	4	100774493	100840647	122	21	120000	-0.64255	0.73974	LAMTOR3	76353	25kb
ENSG00000164031	4	100792405	100892883	167	21	120000	-0.61244	0.72988	DNAJB14	76353	25kb
ENSG00000164032	4	100844243	100896545	94	16	120000	-0.57381	0.71695	H2AFZ	76353	25kb
ENSG00000145358	4	101082027	101136939	276	29	120000	1.9881	0.023399	DDIT4L	76353	25kb
ENSG00000164035	4	101291498	101826283	1768	54	120000	1.8639	0.031165	EMCN	76353	25kb
ENSG00000138814	4	101919566	102294435	845	50	120000	1.9223	0.027286	PPP3CA	76353	25kb
ENSG00000254531	4	102243937	102295040	78	6	120000	0.2765	0.39108	AP001816.1	76353	25kb
ENSG00000153064	4	102307443	103020969	1925	92	120000	-0.59939	0.72554	BANK1	76353	25kb
ENSG00000138821	4	103147198	103377415	744	44	120000	0.22586	0.41066	SLC39A8	76353	25kb
ENSG00000109320	4	103397486	103563459	356	15	120000	-0.047948	0.51912	NFKB1	76353	25kb
ENSG00000109323	4	103527660	103707151	326	22	120000	-0.80985	0.79099	MANBA	76353	25kb
ENSG00000109332	4	103690540	103815053	257	9	120000	-0.7412	0.77071	UBE2D3	76353	25kb
ENSG00000145354	4	103765135	103835399	113	6	120000	-0.74211	0.77099	CISD2	76353	25kb
ENSG00000164037	4	103781205	103965896	321	11	120000	-0.64352	0.74006	SLC9B1	76353	25kb
ENSG00000164038	4	103916025	104031986	189	25	120000	-0.59543	0.72422	SLC9B2	76353	25kb
ENSG00000164039	4	103975592	104046040	123	17	120000	-0.012449	0.50497	BDH2	76353	25kb
ENSG00000138778	4	104001963	104144566	287	14	120000	-0.16771	0.56659	CENPE	76353	25kb
ENSG00000169836	4	104482188	104665973	488	36	120000	-0.70698	0.76021	TACR3	76353	25kb
ENSG00000168772	4	105364469	105441058	122	22	120000	0.67603	0.24951	CXXC4	76353	25kb
ENSG00000168769	4	106042032	106225973	423	37	120000	-0.48386	0.68576	TET2	76353	25kb
ENSG00000138777	4	106265234	106420238	584	25	120000	-0.21448	0.58491	PPA2	76353	25kb
ENSG00000236699	4	106448777	106654250	555	41	120000	1.3026	0.096352	ARHGEF38	76353	25kb
ENSG00000138785	4	106578784	106842143	574	20	120000	-0.53525	0.70376	INTS12	76353	25kb
ENSG00000138780	4	106604935	106793885	405	15	120000	-0.66953	0.74842	GSTCD	76353	25kb
ENSG00000168743	4	106790932	106950184	462	42	120000	-1.1294	0.87063	NPNT	76353	25kb
ENSG00000145348	4	106937756	107267652	695	22	120000	-0.76901	0.77906	TBCK	76353	25kb
ENSG00000164022	4	107211701	107295383	182	17	120000	-0.89583	0.81483	AIMP1	76353	25kb
ENSG00000155011	4	107817959	108229963	987	103	120000	0.23193	0.4083	DKK2	76353	25kb
ENSG00000138801	4	108486433	108666608	480	33	120000	-0.068936	0.52748	PAPSS1	76353	25kb
ENSG00000164023	4	108720719	108861203	325	47	120000	-0.68727	0.75404	SGMS2	76353	25kb
ENSG00000155016	4	108827525	108899613	189	18	120000	0.33779	0.36776	CYP2U1	76353	25kb
ENSG00000138796	4	108885870	108981331	221	14	120000	-0.24768	0.59781	HADH	76353	25kb
ENSG00000138795	4	108943701	109115112	324	24	120000	-0.26247	0.60352	LEF1	76353	25kb
ENSG00000109475	4	109516722	109576568	153	16	120000	1.324	0.092759	RPL34	76353	25kb
ENSG00000198856	4	109546740	109613976	152	10	120000	0.17023	0.43242	OSTC	76353	25kb
ENSG00000164089	4	109638196	109709210	170	25	120000	2.1432	0.016048	ETNPPL	76353	25kb
ENSG00000188517	4	109706877	110248813	1654	92	120000	2.0094	0.022248	COL25A1	76353	25kb
ENSG00000138802	4	110329928	110487052	295	13	120000	-0.81316	0.79194	SEC24B	76353	25kb
ENSG00000005059	4	110456361	110634874	406	29	120000	0.85624	0.19593	CCDC109B	76353	25kb
ENSG00000138794	4	110584785	110649739	104	13	120000	0.95173	0.17062	CASP6	76353	25kb
ENSG00000123739	4	110606145	110676233	126	21	120000	0.95887	0.16881	PLA2G12A	76353	25kb
ENSG00000205403	4	110636852	110748335	272	24	120000	-0.0066151	0.50264	CFI	76353	25kb
ENSG00000109534	4	110711666	110770893	162	18	120000	-0.14987	0.55957	GAR1	76353	25kb
ENSG00000180245	4	110724150	110790760	153	14	120000	-0.31964	0.62538	RRH	76353	25kb
ENSG00000183423	4	110744358	110818471	196	16	120000	-0.72806	0.76671	LRIT3	76353	25kb
ENSG00000138798	4	110809040	110958422	313	17	120000	0.099836	0.46024	EGF	76353	25kb
ENSG00000170522	4	110942002	111145355	659	79	120000	0.32176	0.37382	ELOVL6	76353	25kb
ENSG00000138792	4	111261889	111511441	654	54	120000	0.16313	0.43521	ENPEP	76353	25kb
ENSG00000164093	4	111513579	111588279	119	15	120000	-0.066291	0.52643	PITX2	76353	25kb
ENSG00000174749	4	113041553	113141412	184	18	120000	-0.8778	0.80997	C4orf32	76353	25kb

ENSG00000138660	4	113127893	113216203	157	13	120000	-0.46953	0.68066	AP1AR	76353	25kb
ENSG00000145365	4	113170698	113232059	102	10	120000	-0.24471	0.59666	TIFA	76353	25kb
ENSG00000073331	4	113181665	113388776	552	66	120000	-0.37374	0.6457	ALPK1	76353	25kb
ENSG00000178403	4	113409672	113462328	136	19	120000	-1.1203	0.86871	NEUROG2	76353	25kb
ENSG00000138658	4	113435492	113583151	290	24	120000	-0.58689	0.72136	C4orf21	76353	25kb
ENSG00000174720	4	113533120	113603748	150	25	120000	-0.44959	0.6735	LARP7	76353	25kb
ENSG00000145362	4	113714265	114329896	1750	143	120000	0.80446	0.21057	ANK2	76353	25kb
ENSG00000145349	4	114347188	114708083	849	63	120000	0.18431	0.42688	CAMK2D	76353	25kb
ENSG00000180801	4	114796440	114925883	339	31	120000	0.013749	0.49452	ARSJ	76353	25kb
ENSG00000174607	4	115494611	115624380	311	30	120000	0.70566	0.2402	UGT8	76353	25kb
ENSG00000138653	4	115723919	116060032	871	46	120000	-0.15416	0.56126	NDST4	76353	25kb
ENSG00000270394	4	117195016	117246520	137	11	120000	-0.18162	0.57206	MTRNR2L13	76353	25kb
ENSG00000174599	4	117979718	118031736	136	21	120000	-0.23741	0.59383	TRAM1L1	76353	25kb
ENSG00000164100	4	118929773	119204803	864	28	120000	0.8399	0.20048	NDST3	76353	25kb
ENSG00000164099	4	119176193	119299158	326	32	120000	0.80735	0.20973	PRSS12	76353	25kb
ENSG00000145388	4	119581523	119661588	168	16	120000	-0.018417	0.50735	METTL14	76353	25kb
ENSG00000150961	4	119618978	119784838	601	21	120000	0.51122	0.3046	SEC24D	76353	25kb
ENSG00000172403	4	119784996	120007402	705	65	120000	-0.46726	0.67984	SYNPO2	76353	25kb
ENSG00000172399	4	120031939	120133944	352	29	120000	0.49302	0.311	MYOZ2	76353	25kb
ENSG00000178636	4	120088928	120158799	193	24	120000	-0.67456	0.75002	RP11-455G16.1	76353	25kb
ENSG00000145390	4	120108742	120241672	397	22	120000	-0.53613	0.70407	USP53	76353	25kb
ENSG00000164096	4	120193207	120250600	200	19	120000	-0.4083	0.65847	C4orf3	76353	25kb
ENSG00000145384	4	120213405	120268545	234	14	120000	-0.58287	0.72001	FABP2	76353	25kb
ENSG00000138735	4	120390550	120575146	524	18	120000	-0.32616	0.62785	PDE5A	76353	25kb
ENSG00000164109	4	120951763	121013229	132	20	120000	-0.71004	0.76116	MAD2L1	76353	25kb
ENSG00000138738	4	121581074	121869025	1131	43	120000	-0.99476	0.84007	PRDM5	76353	25kb
ENSG00000173376	4	121931768	122019176	243	26	120000	-0.22473	0.5889	NDNF	76353	25kb
ENSG00000050730	4	122027563	122173621	427	33	120000	-0.9771	0.83574	TNIP3	76353	25kb
ENSG00000186867	4	122225467	122327214	517	33	120000	0.18687	0.42588	QRFPR	76353	25kb
ENSG00000164111	4	122564110	122643268	295	27	120000	1.6627	0.048181	ANXA5	76353	25kb
ENSG00000164112	4	122655088	122711582	235	20	120000	-0.29091	0.61444	TMEM155	76353	25kb
ENSG00000226757	4	122660740	122712962	222	18	120000	-0.26526	0.60459	AC079341.1	76353	25kb
ENSG00000123737	4	122697472	122763176	146	17	120000	-0.89723	0.8152	EXOSC9	76353	25kb
ENSG00000145386	4	122712599	122770087	119	15	120000	-0.99689	0.84059	CCNA2	76353	25kb
ENSG00000138686	4	122720595	122816652	179	20	120000	-0.93905	0.82615	BBS7	76353	25kb
ENSG00000138741	4	122775182	122897909	230	33	120000	-0.4622	0.67803	TRPC3	76353	25kb
ENSG00000138688	4	123048488	123308913	417	28	120000	0.14256	0.44332	KIAA1109	76353	25kb
ENSG00000164113	4	123275121	123375957	182	23	120000	-0.20821	0.58247	ADAD1	76353	25kb
ENSG00000109471	4	123347625	123402880	109	19	120000	-0.63952	0.73876	IL2	76353	25kb
ENSG00000138684	4	123508783	123567224	179	20	120000	0.29848	0.38267	IL21	76353	25kb
ENSG00000181004	4	123628857	123691098	154	13	120000	0.32509	0.37256	BBS12	76353	25kb
ENSG00000138685	4	123722863	123844391	432	41	120000	0.28341	0.38843	FGF2	76353	25kb
ENSG00000170917	4	123784852	123869123	291	23	120000	-1.1286	0.87046	NUDT6	76353	25kb
ENSG00000145375	4	123819229	124265605	1450	30	120000	-1.4939	0.9324	SPATA5	76353	25kb
ENSG00000164056	4	124292950	124349910	135	26	120000	0.46226	0.32195	SPRY1	76353	25kb
ENSG00000151458	4	125560207	125658887	214	31	120000	-1.6536	0.95089	ANKRD50	76353	25kb
ENSG00000196159	4	126212554	126439087	729	55	120000	-0.5113	0.69543	FAT4	76353	25kb
ENSG00000164066	4	128519426	128672892	264	21	120000	-0.81874	0.79353	INTU	76353	25kb
ENSG00000151475	4	128626533	128720448	113	13	120000	-0.12675	0.55043	SLC25A31	76353	25kb
ENSG00000164070	4	128677976	128786888	115	14	120000	-0.3214	0.62605	HSPA4L	76353	25kb
ENSG00000142731	4	128777016	128845350	80	11	120000	0.0032835	0.49869	PLK4	76353	25kb
ENSG00000164073	4	128813960	128912150	136	11	120000	-0.18095	0.5718	MFSD8	76353	25kb
ENSG00000164074	4	128861461	128985866	181	12	120000	-0.20169	0.57992	C4orf29	76353	25kb
ENSG00000138709	4	128957423	129169086	413	18	120000	0.041533	0.48344	LARP1B	76353	25kb
ENSG00000164040	4	129165397	129234984	116	18	120000	-1.7479	0.95976	PGRMC2	76353	25kb
ENSG00000077684	4	129705779	129821379	178	29	120000	-1.3072	0.90443	JADE1	76353	25kb
ENSG00000151466	4	129761076	130039764	538	26	120000	-1.0341	0.84946	SCLT1	76353	25kb
ENSG00000151470	4	129989472	130062795	196	19	120000	-0.0065567	0.50262	C4orf33	76353	25kb
ENSG00000248369	4	133331957	133393390	149	14	120000	-0.050399	0.5201	RP11-62N21.1	76353	25kb
ENSG00000138650	4	134045470	134154356	377	29	120000	1.4253	0.077039	PCDH10	76353	25kb
ENSG00000254535	4	135092488	135147903	131	14	120000	0.22679	0.41029	PABPC4L	76353	25kb
ENSG00000189184	4	138415072	138478648	198	28	120000	0.48906	0.3124	PCDH18	76353	25kb
ENSG00000151012	4	139060251	139188503	338	34	120000	0.39022	0.34819	SLC7A11	76353	25kb
ENSG00000151014	4	139911943	139991906	112	25	120000	-0.79888	0.78782	CCRN4L	76353	25kb
ENSG00000109381	4	139924266	140123372	348	29	120000	-0.6525	0.74296	ELF2	76353	25kb
ENSG00000137463	4	140162317	140226492	159	14	120000	0.20584	0.41846	MGARP	76353	25kb
ENSG00000109390	4	140163034	140248705	216	17	120000	0.19249	0.42368	NDUFC1	76353	25kb
ENSG00000164134	4	140197609	140366187	308	14	120000	-0.069205	0.52759	NAA15	76353	25kb
ENSG00000172007	4	140349386	140422763	155	15	120000	0.15272	0.43931	RAB33B	76353	25kb
ENSG00000145391	4	140392095	140552853	431	71	120000	0.2501	0.40125	SETD7	76353	25kb
ENSG00000085871	4	140561922	140686899	409	52	120000	-0.57537	0.71748	MGST2	76353	25kb
ENSG00000196782	4	140612907	141100338	1213	130	120000	-1.0471	0.85248	MAML3	76353	25kb
ENSG00000153130	4	141153440	141331880	425	43	120000	-1.1694	0.87887	SCOC	76353	25kb
ENSG00000153132	4	141284609	141374122	221	16	120000	-0.24524	0.59687	CLGN	76353	25kb
ENSG00000205301	4	141338416	141444531	281	21	120000	1.4195	0.077876	RP11-542P2.1	76353	25kb
ENSG00000179387	4	141420312	141499924	161	16	120000	0.75317	0.22567	ELMOD2	76353	25kb
ENSG00000109424	4	141455588	141514959	135	18	120000	0.38161	0.35137	UCP1	76353	25kb
ENSG00000109436	4	141516919	141702274	470	49	120000	-0.88281	0.81133	TBC1D9	76353	25kb
ENSG00000170153	4	141755961	142159031	1207	100	120000	-0.88098	0.81084	RNF150	76353	25kb
ENSG00000109445	4	142117041	142180851	168	11	120000	-0.72186	0.76481	ZNF330	76353	25kb
ENSG00000164136	4	142532752	142680140	339	23	120000	0.80917	0.20921	IL15	76353	25kb
ENSG00000109452	4	142919313	143793585	2474	103	120000	0.75158	0.22615	INPP4B	76353	25kb
ENSG00000170185	4	144081070	144169983	196	11	120000	-1.092	0.86259	USP38	76353	25kb
ENSG00000109458	4	144232915	144420721	373	42	120000	-0.94542	0.82778	GAB1	76353	25kb
ENSG00000153147	4	144409616	144503639	187	14	120000	-0.78081	0.78254	SMARCA5	76353	25kb
ENSG00000183090	4	144473455	144646828	348	27	120000	0.52024	0.30145	FREM3	76353	25kb
ENSG00000197465	4	144767020	144851716	199	19	120000	0.44717	0.32737	GYPE	76353	25kb
ENSG00000250361	4	144892257	145086844	607	28	120000	0.18473	0.42672	GYPB	76353	25kb
ENSG00000170180	4	145005457	145086904	302	22	120000	0.34479	0.36512	GYPA	76353	25kb
ENSG00000164161	4	145542173	145691423	297	21	120000	0.2304	0.40889	HHIP	76353	25kb
ENSG00000164162	4	145863264	146044693	196	33	120000	0.12142	0.45168	ANAPC10	76353	25kb
ENSG00000164163	4	145994084	146075331	90	20	120000	0.50071	0.30829	ABCE1	76353	25kb
ENSG00000164164	4	146006990	146126313	123	25	120000	0.57197	0.28367	OTUD4	76353	25kb
ENSG00000170365	4	146377346	146504231	245	25	120000	0.90883	0.18172	SMAD1	76353	25kb
ENSG00000151611	4	146514415	146606187	245	29	120000	0.79788	0.21247	MMAA	76353	25kb
ENSG00000237136	4	146576356	146717184	368	37	120000	1.3004	0.096726	C4orf51	76353	25kb

ENSG00000151612	4	146653779	146884787	473	62	120000	1.7347	0.041393	ZNF827	76353	25kb
ENSG00000164167	4	147071837	147146152	247	17	120000	-0.70845	0.76067	LSM6	76353	25kb
ENSG00000250673	4	147120709	147175208	141	21	120000	-0.67074	0.74881	RP11-6L6.2	76353	25kb
ENSG00000120519	4	147150127	147468123	636	34	120000	0.29724	0.38314	SLC10A7	76353	25kb
ENSG00000151615	4	147535045	147588626	133	15	120000	-0.32799	0.62854	POU4F2	76353	25kb
ENSG00000137473	4	147602790	147892034	676	27	120000	-0.0039769	0.50159	TTC29	76353	25kb
ENSG00000151617	4	148377069	148491106	250	30	120000	1.4754	0.070053	EDNRA	76353	25kb
ENSG00000164168	4	148513534	148618195	171	19	120000	0.12567	0.45	TMEM184C	76353	25kb
ENSG00000164169	4	148533936	148630381	128	12	120000	-0.071147	0.52836	PRMT10	76353	25kb
ENSG00000071205	4	148628214	149018931	818	65	120000	0.74084	0.2294	ARHGAP10	76353	25kb
ENSG00000151623	4	148974913	149390850	1065	87	120000	-0.2879	0.61329	NR3C2	76353	25kb
ENSG00000170390	4	150974426	151203609	673	39	120000	-0.78082	0.78255	DCLK2	76353	25kb
ENSG00000198589	4	151160594	151961879	1494	46	120000	2.1309	0.01655	LRBA	76353	25kb
ENSG00000181541	4	151478077	151530843	87	15	120000	2.3559	0.0092383	MAB21L2	76353	25kb
ENSG00000145425	4	151995725	152050804	101	11	120000	0.91159	0.18099	RPS3A	76353	25kb
ENSG00000109686	4	151998903	152271784	634	30	120000	-0.094849	0.53778	SH3D19	76353	25kb
ENSG00000189099	4	152173323	152237605	166	15	120000	-0.85228	0.80297	PRSS48	76353	25kb
ENSG00000164142	4	152305368	152609784	676	17	120000	-1.0144	0.8448	FAM160A1	76353	25kb
ENSG00000059691	4	152566656	152707175	296	16	120000	-0.86106	0.8054	PET112	76353	25kb
ENSG00000109670	4	153217410	153482253	544	31	120000	0.10878	0.45669	FBXW7	76353	25kb
ENSG00000170006	4	153514784	153626317	307	56	120000	1.2229	0.11068	TMEM154	76353	25kb
ENSG00000169989	4	153665506	153725916	132	17	120000	-0.13993	0.55564	TIGD4	76353	25kb
ENSG00000164144	4	153676089	153864615	396	23	120000	0.22457	0.41116	ARFIP1	76353	25kb
ENSG00000137460	4	153832504	153925848	250	52	120000	-0.61713	0.73143	FHDC1	76353	25kb
ENSG00000109654	4	154048494	154285472	620	64	120000	0.019935	0.49205	TRIM2	76353	25kb
ENSG00000121211	4	154240801	154361270	352	26	120000	-0.86043	0.80522	MND1	76353	25kb
ENSG00000121210	4	154362498	154582863	631	74	120000	0.78649	0.21579	KIAA0922	76353	25kb
ENSG00000137462	4	154597652	154651851	149	24	120000	1.3889	0.082438	TLR2	76353	25kb
ENSG00000145428	4	154606277	154706387	316	20	120000	1.1528	0.1245	RNF175	76353	25kb
ENSG00000145423	4	154676744	154735272	194	17	120000	0.43034	0.33347	SFRP2	76353	25kb
ENSG00000197410	4	155128399	155437930	918	62	120000	-1.0301	0.84852	DCHS2	76353	25kb
ENSG00000171566	4	155431158	155496587	208	20	120000	0.9456	0.17218	PLRG1	76353	25kb
ENSG00000171564	4	155459108	155517238	129	15	120000	1.6493	0.049544	FGB	76353	25kb
ENSG00000171560	4	155479278	155536918	93	15	120000	2.2235	0.013092	FGA	76353	25kb
ENSG00000171557	4	155500286	155559119	91	20	120000	1.0095	0.15636	FGG	76353	25kb
ENSG00000121207	4	155523097	155699270	319	23	120000	0.48925	0.31233	LRAT	76353	25kb
ENSG00000151962	4	155677365	155774965	254	17	120000	0.84056	0.2003	RBM46	76353	25kb
ENSG00000185149	4	156104781	156163230	214	26	120000	-1.2139	0.8876	NPY2R	76353	25kb
ENSG00000164114	4	156238810	156323122	297	19	120000	-0.21419	0.5848	MAP9	76353	25kb
ENSG00000164116	4	156562863	156678501	405	43	120000	-1.1525	0.87544	GUCY1A3	76353	25kb
ENSG00000061918	4	156655144	156753743	320	49	120000	-1.5376	0.93793	GUCY1B3	76353	25kb
ENSG00000256394	4	156725881	156812425	319	35	120000	-0.53534	0.70379	ASIC5	76353	25kb
ENSG00000151790	4	156750890	156866558	444	34	120000	0.39532	0.3463	TDO2	76353	25kb
ENSG00000256043	4	156820270	156900069	349	38	120000	2.016	0.0219	CTSO	76353	25kb
ENSG00000251283	4	157530646	157588606	219	23	120000	0.7673	0.22145	RP11-171N4.2	76353	25kb
ENSG00000145431	4	157656606	157917546	443	41	120000	-0.25916	0.60225	PDGFC	76353	25kb
ENSG00000109738	4	157972209	158118242	523	29	120000	-1.4521	0.92676	GLRB	76353	25kb
ENSG00000120251	4	158100334	158312227	375	35	120000	-1.2175	0.88829	GRIA2	76353	25kb
ENSG00000164125	4	159020626	159119470	211	16	120000	0.55241	0.29033	FAM198B	76353	25kb
ENSG00000164124	4	159097756	159201563	209	38	120000	-0.31219	0.62255	TMEM144	76353	25kb
ENSG00000171509	4	159211463	159599524	987	28	120000	-0.093377	0.5372	RXFP1	76353	25kb
ENSG00000205208	4	159562831	159618407	65	15	120000	-0.82819	0.79622	C4orf46	76353	25kb
ENSG00000171503	4	159568277	159655775	99	23	120000	-0.11773	0.54686	ETFDH	76353	25kb
ENSG00000171497	4	159605286	159669548	88	18	120000	0.67613	0.24948	PPID	76353	25kb
ENSG00000052795	4	159665290	159854201	295	14	120000	1.3402	0.090096	FNIP2	76353	25kb
ENSG00000164123	4	159789286	159984912	485	28	120000	0.81388	0.20786	C4orf45	76353	25kb
ENSG00000109756	4	160000330	160306321	555	36	120000	0.35553	0.3611	RAPGEF2	76353	25kb
ENSG00000168843	4	162280049	163110187	2898	119	120000	0.62976	0.26443	FSTL5	76353	25kb
ENSG00000145414	4	164006225	164113073	221	25	120000	-0.68329	0.75279	NAF1	76353	25kb
ENSG00000164128	4	164220113	164290984	256	25	120000	0.22524	0.4109	NPY1R	76353	25kb
ENSG00000164129	4	164240091	164298086	246	28	120000	0.29573	0.38372	NPY5R	76353	25kb
ENSG00000151005	4	164367257	164420047	117	22	120000	-0.40667	0.65787	TKTL2	76353	25kb
ENSG00000198498	4	164390594	164466691	173	15	120000	-0.48425	0.6859	TMA16	76353	25kb
ENSG00000145416	4	164420450	165330202	2962	130	120000	-1.5485	0.93924	MARCH1	76353	25kb
ENSG00000183439	4	165850598	165923820	218	21	120000	-1.095	0.86323	TRIM61	76353	25kb
ENSG00000250486	4	165853100	165905273	147	18	120000	-1.3152	0.90579	FAM218A	76353	25kb
ENSG00000176979	4	165928105	165987896	153	25	120000	-0.89855	0.81555	TRIM60	76353	25kb
ENSG00000170088	4	165970574	166154701	604	44	120000	-0.37748	0.64709	TMEM192	76353	25kb
ENSG00000109466	4	166103770	166269308	420	36	120000	0.12596	0.44988	KLHL2	76353	25kb
ENSG00000052802	4	166223775	166289312	243	30	120000	-0.64805	0.74152	MSMO1	76353	25kb
ENSG00000109472	4	166257346	166444472	754	34	120000	-0.71723	0.76338	CPE	76353	25kb
ENSG00000038295	4	166769410	167050047	746	83	120000	-0.11892	0.54733	TLL1	76353	25kb
ENSG00000196104	4	167629535	168180947	1692	70	120000	-0.38441	0.64966	SPOCK3	76353	25kb
ENSG00000109511	4	168988666	169133841	349	25	120000	-0.19156	0.57595	ANXA10	76353	25kb
ENSG00000137628	4	169112444	169264958	425	25	120000	0.93272	0.17548	DDX60	76353	25kb
ENSG00000181381	4	169252886	169483937	909	59	120000	-1.1467	0.87425	DDX60L	76353	25kb
ENSG00000129116	4	169393217	169874608	1451	114	120000	0.044704	0.48217	PALLD	76353	25kb
ENSG00000145439	4	169759921	169956426	404	25	120000	0.4757	0.31715	CBR4	76353	25kb
ENSG00000154447	4	169990407	170217256	589	45	120000	-1.257	0.89563	SH3RF1	76353	25kb
ENSG00000137601	4	170289426	170558780	603	20	120000	-0.88637	0.81229	NEK1	76353	25kb
ENSG00000109572	4	170508784	170669824	416	32	120000	0.066412	0.47353	CLCN3	76353	25kb
ENSG00000056050	4	170625616	170704104	187	38	120000	-0.045844	0.51828	C4orf27	76353	25kb
ENSG00000198948	4	170882748	170979182	241	28	120000	1.4002	0.080729	MFAP3L	76353	25kb
ENSG00000109576	4	170956373	171037850	213	29	120000	0.78116	0.21736	AADAT	76353	25kb
ENSG00000174473	4	172708405	173987710	3146	132	120000	-1.6917	0.95464	GALNTL6	76353	25kb
ENSG00000109586	4	174064904	174270118	447	39	120000	0.44682	0.3275	GALNT7	76353	25kb
ENSG00000164104	4	174227846	174281276	96	14	120000	1.1422	0.12669	HMGB2	76353	25kb
ENSG00000164105	4	174266120	174323683	93	13	120000	1.4929	0.067735	SAP30	76353	25kb
ENSG00000164106	4	174280852	174352531	118	20	120000	0.63995	0.2611	SCRG1	76353	25kb
ENSG00000164107	4	174421120	174476380	131	24	120000	-1.5916	0.94427	HAND2	76353	25kb
ENSG00000164117	4	175132809	175230415	196	28	120000	-1.0491	0.85292	FBXO8	76353	25kb
ENSG00000164118	4	175179828	175279531	222	29	120000	-0.81204	0.79162	CEP44	76353	25kb
ENSG00000164120	4	175386328	175469305	287	39	120000	0.050736	0.47977	HPGD	76353	25kb
ENSG00000145451	4	175533065	175775465	899	70	120000	1.7095	0.043682	GLRA3	76353	25kb
ENSG00000168594	4	175725819	175924331	742	29	120000	-0.60932	0.72884	ADAM29	76353	25kb
ENSG00000150625	4	176529085	176948815	1296	104	120000	-1.8585	0.96845	GPM6A	76353	25kb

ENSG00000150627	4	176961985	177128978	591	34	120000	0.92131	0.17844	WDR17	76353	25kb
ENSG00000150628	4	177080789	177141822	193	38	120000	-1.0152	0.845	SPATA4	76353	25kb
ENSG00000164122	4	177109824	177223722	395	44	120000	-1.0257	0.84749	ASB5	76353	25kb
ENSG00000129128	4	177216115	177278396	303	23	120000	1.0902	0.13781	SPCS3	76353	25kb
ENSG00000150630	4	177579689	177738881	444	26	120000	1.0971	0.13629	VEGFC	76353	25kb
ENSG00000249008	4	178138693	178194927	197	31	120000	0.095441	0.46198	RP11-487E13.1	76353	25kb
ENSG00000109674	4	178205990	178309097	339	59	120000	0.035516	0.48583	NEIL3	76353	25kb
ENSG00000038002	4	178326924	178388657	155	28	120000	-0.093316	0.53717	AGA	76353	25kb
ENSG00000231171	4	178624911	178936904	1001	69	120000	0.55882	0.28814	LINC01098	76353	25kb
ENSG00000218336	4	183040140	183749177	2598	239	120000	-1.0116	0.84413	TENM3	76353	25kb
ENSG00000129187	4	183786213	183864089	357	43	120000	0.84263	0.19972	DCTD	76353	25kb
ENSG00000151718	4	183995446	184266930	534	48	120000	-0.3958	0.65387	WWC2	76353	25kb
ENSG00000177300	4	184214220	184266927	150	24	120000	0.41911	0.33757	CLDN22	76353	25kb
ENSG00000185758	4	184217917	184268579	143	24	120000	0.44618	0.32773	CLDN24	76353	25kb
ENSG00000168564	4	184340744	184394351	309	22	120000	0.54841	0.29171	CDKN2AIP	76353	25kb
ENSG00000168556	4	184401147	184457249	194	32	120000	0.57254	0.28348	ING2	76353	25kb
ENSG00000182552	4	184535788	184605378	309	44	120000	-2.2357	0.98731	RWDD4	76353	25kb
ENSG00000168538	4	184555420	184659745	423	45	120000	-0.87781	0.80998	TRAPPC11	76353	25kb
ENSG00000173320	4	184749584	184969679	649	91	120000	-1.9307	0.97324	STOX2	76353	25kb
ENSG00000164303	4	184984859	185167383	624	69	120000	0.37913	0.3523	ENPP6	76353	25kb
ENSG00000168310	4	185283867	185420734	485	95	120000	0.61705	0.2686	IRF2	76353	25kb
ENSG00000164305	4	185523850	185595663	239	29	120000	0.93654	0.1745	CASP3	76353	25kb
ENSG00000164306	4	185545767	185641117	408	19	120000	0.77806	0.21827	PRIMPOL	76353	25kb
ENSG00000151725	4	185590772	185680287	445	12	120000	0.69289	0.24419	CENPU	76353	25kb
ENSG00000151726	4	185651749	185772972	431	43	120000	-0.74431	0.77166	ACSL1	76353	25kb
ENSG00000251139	4	185709773	185767443	214	26	120000	-1.2791	0.89956	RP11-701P16.2	76353	25kb
ENSG00000187821	4	185914995	185966958	211	34	120000	-1.6038	0.94562	HELT	76353	25kb
ENSG00000151729	4	186039395	186096536	150	19	120000	-0.13422	0.55339	SLC25A4	76353	25kb
ENSG00000164323	4	186055819	186155658	324	16	120000	0.38518	0.35005	KIAA1430	76353	25kb
ENSG00000109762	4	186100391	186316339	613	38	120000	0.3066	0.37957	SNX25	76353	25kb
ENSG00000109771	4	186260032	186342053	218	29	120000	0.47768	0.31644	LRP2BP	76353	25kb
ENSG00000186352	4	186292175	186346782	157	23	120000	0.15521	0.43833	ANKRD37	76353	25kb
ENSG00000109775	4	186295694	186372139	218	25	120000	0.54484	0.29293	UFSP2	76353	25kb
ENSG00000205129	4	186322394	186395980	234	37	120000	2.0658	0.019425	C4orf47	76353	25kb
ENSG00000168491	4	186341336	186417913	274	41	120000	2.1345	0.016402	CCDC110	76353	25kb
ENSG00000249679	4	186367670	186418417	199	35	120000	2.1314	0.016529	RP11-279O9.4	76353	25kb
ENSG00000154553	4	186397851	186481766	245	34	120000	2.1536	0.015637	PDLIM3	76353	25kb
ENSG00000154556	4	186481598	186902806	1787	175	120000	1.2046	0.11417	SORBS2	76353	25kb
ENSG00000164342	4	186965306	187034223	290	41	120000	0.84855	0.19807	TLR3	76353	25kb
ENSG00000109794	4	187000573	187118821	414	45	120000	0.6179	0.26832	FAM149A	76353	25kb
ENSG00000269302	4	187086913	187137626	182	23	120000	-0.92272	0.82192	AC110771.1	76353	25kb
ENSG00000145476	4	187087674	187159610	241	30	120000	-0.85303	0.80318	CYP4V2	76353	25kb
ENSG00000164344	4	187105133	187204625	339	32	120000	-0.9128	0.81933	KLKB1	76353	25kb
ENSG00000088926	4	187162099	187235835	238	39	120000	-0.71277	0.76201	F11	76353	25kb
ENSG00000272297	4	187322700	187501464	776	73	120000	-0.91828	0.82076	RP11-215A19.2	76353	25kb
ENSG00000168412	4	187429809	187501721	243	38	120000	0.44521	0.32808	MTNR1A	76353	25kb
ENSG00000083857	4	187483937	187672876	600	76	120000	1.2498	0.10568	FAT1	76353	25kb
ENSG00000179059	4	188891925	188951204	241	41	120000	-1.6203	0.94742	ZFP42	76353	25kb
ENSG00000179046	4	188987427	189055757	463	46	120000	-0.90268	0.81665	TRIML2	76353	25kb
ENSG00000184108	4	189035573	189093897	340	35	120000	0.25709	0.39855	TRIML1	76353	25kb
ENSG00000109536	4	190836943	190909359	87	5	120000	-1.2382	0.89218	FRG1	76353	25kb
ENSG00000205097	4	190920506	190973412	2	1	120000	-1.6409	0.94959	FRG2	76353	25kb
ENSG00000153404	5	115373	215085	367	21	120000	1.1659	0.12183	PLEKHG4B	76353	25kb
ENSG00000185028	5	166626	220468	219	22	120000	1.0455	0.14791	LRRC14B	76353	25kb
ENSG00000164366	5	171986	243330	350	20	120000	1.127	0.12987	CCDC127	76353	25kb
ENSG00000073578	5	193356	281815	400	12	120000	1.0318	0.15109	SDHA	76353	25kb
ENSG00000249915	5	246736	378971	396	21	120000	0.929	0.17645	PDCD6	76353	25kb
ENSG00000063438	5	279291	463406	630	43	120000	0.65034	0.25774	AHRR	76353	25kb
ENSG00000221990	5	416645	468258	241	22	120000	0.65131	0.25742	C5orf55	76353	25kb
ENSG00000180104	5	418273	497052	399	27	120000	0.21519	0.41481	EXOC3	76353	25kb
ENSG00000188242	5	441239	498213	281	23	120000	-0.39559	0.65379	CTD-2228K2.5	76353	25kb
ENSG00000066230	5	448425	549447	448	48	120000	-0.24219	0.59568	SLC9A3	76353	25kb
ENSG00000112877	5	587387	692283	376	26	120000	-0.29225	0.61495	CEP72	76353	25kb
ENSG00000171368	5	635883	718510	240	30	120000	-0.35965	0.64045	TPPP	76353	25kb
ENSG00000268885	5	642759	693839	181	24	120000	-0.26941	0.60619	AC026740.1	76353	25kb
ENSG00000188818	5	685471	876101	308	33	120000	-1.4985	0.933	ZDHHC11	76353	25kb
ENSG00000206077	5	685475	792067	130	33	120000	-1.0193	0.84597	ZDHHC11B	76353	25kb
ENSG00000028310	5	825406	917939	199	11	120000	-0.68546	0.75347	BRD9	76353	25kb
ENSG00000071539	5	867758	944472	194	9	120000	-0.34486	0.6349	TRIP13	76353	25kb
ENSG00000250173	5	954480	1006415	138	23	120000	2.082	0.018669	RP11-661C8.3	76353	25kb
ENSG00000145506	5	983944	1064058	299	31	120000	1.3872	0.082689	NKD2	76353	25kb
ENSG00000113504	5	1025499	1137150	447	60	120000	-1.017	0.84542	SLC12A7	76353	25kb
ENSG00000174358	5	1176710	1250232	311	52	120000	-0.93744	0.82573	SLC6A19	76353	25kb
ENSG00000164363	5	1200470	1271304	270	44	120000	-0.80493	0.78957	SLC6A18	76353	25kb
ENSG00000164362	5	1228262	1320184	273	56	120000	2.3014	0.010685	TERT	76353	25kb
ENSG00000049656	5	1292859	1370214	254	30	120000	2.6345	0.0042131	CLPTM1L	76353	25kb
ENSG00000142319	5	1367909	1470545	343	50	120000	1.3305	0.091678	SLC6A3	76353	25kb
ENSG00000153395	5	1431595	1549092	383	54	120000	0.65092	0.25755	LPCAT1	76353	25kb
ENSG00000171421	5	1773500	1826480	223	23	120000	-1.0323	0.84904	MRPL36	76353	25kb
ENSG00000145494	5	1776514	1841719	281	20	120000	-0.63198	0.7363	NDUFS6	76353	25kb
ENSG00000113430	5	1852541	1912350	288	39	120000	-0.84381	0.80061	IRX4	76353	25kb
ENSG00000170561	5	2720959	2777969	233	30	120000	0.23064	0.4088	IRX2	76353	25kb
ENSG00000186493	5	2727245	2780508	223	27	120000	0.31034	0.37815	C5orf38	76353	25kb
ENSG00000170549	5	3571168	3626517	264	26	120000	-0.33273	0.63033	IRX1	76353	25kb
ENSG00000145536	5	5115443	5345417	1030	117	120000	-0.14257	0.55668	ADAMTS16	76353	25kb
ENSG00000164151	5	5395777	5515347	268	26	120000	0.51722	0.3025	KIAA0947	76353	25kb
ENSG00000133398	5	6346994	6403707	193	24	120000	-0.27908	0.60991	MED10	76353	25kb
ENSG00000215218	5	6423736	6520022	348	53	120000	0.41513	0.33902	UBE2QL1	76353	25kb
ENSG00000037474	5	6574352	6658404	369	32	120000	-1.2335	0.89131	NSUN2	76353	25kb
ENSG00000145545	5	6608456	6694675	328	32	120000	-0.88994	0.81325	SRD5A1	76353	25kb
ENSG00000112941	5	6689718	6782161	381	32	120000	0.065192	0.47401	PAPD7	76353	25kb
ENSG00000078295	5	7371321	7855194	1317	99	120000	0.52146	0.30102	ADCY2	76353	25kb
ENSG00000215217	5	7805491	7876603	221	40	120000	0.81269	0.2082	C5orf49	76353	25kb
ENSG00000124275	5	7826299	7931138	504	41	120000	1.6735	0.047113	MTRR	76353	25kb
ENSG00000124279	5	7834272	7894150	249	43	120000	1.8165	0.034644	FASTKD3	76353	25kb
ENSG00000112902	5	9010138	9571187	1920	170	120000	-0.31569	0.62388	SEMA5A	76353	25kb

ENSG00000169777	5	9604109	9737490	421	65	120000	-0.42714	0.66536	TAS2R1	76353	25kb
ENSG00000150756	5	10201442	10275009	305	29	120000	1.5104	0.065465	FAM173B	76353	25kb
ENSG00000150753	5	10225033	10291524	304	25	120000	1.3466	0.089062	CCT5	76353	25kb
ENSG00000164237	5	10250987	10333138	269	23	120000	1.1268	0.12992	CMBL	76353	25kb
ENSG00000145495	5	10328815	10465500	198	18	120000	0.078839	0.46858	MARCH6	76353	25kb
ENSG00000145491	5	10416636	10497141	183	24	120000	0.37136	0.35518	ROPN1L	76353	25kb
ENSG00000249160	5	10480108	10547196	197	43	120000	0.74304	0.22873	RP11-1C1.5	76353	25kb
ENSG00000164236	5	10539442	10675308	429	51	120000	-1.0842	0.86086	ANKRD33B	76353	25kb
ENSG00000112977	5	10654342	10786384	426	31	120000	0.038713	0.48456	DAP	76353	25kb
ENSG00000169862	5	10946952	11929155	2942	225	120000	-0.18972	0.57524	CTNND2	76353	25kb
ENSG00000039139	5	13665440	13969652	1015	59	120000	2.0551	0.019936	DNAH5	76353	25kb
ENSG00000038382	5	14118811	14557235	1115	64	120000	-0.17353	0.56888	TRIO	76353	25kb
ENSG00000145569	5	14556884	14640116	138	25	120000	-1.0082	0.84332	FAM105A	76353	25kb
ENSG00000154124	5	14639773	14724820	173	20	120000	-0.6982	0.75748	FAM105B	76353	25kb
ENSG00000154122	5	14679910	14896887	533	49	120000	-1.3459	0.91084	ANKH	76353	25kb
ENSG00000183580	5	15475305	15964900	1055	57	120000	0.88274	0.18869	FBXL7	76353	25kb
ENSG00000183654	5	16042248	16205871	321	41	120000	-0.17432	0.56919	MARCH11	76353	25kb
ENSG00000173545	5	16426628	16490901	168	21	120000	-0.6581	0.74476	ZNF622	76353	25kb
ENSG00000154153	5	16448147	16642167	550	55	120000	-1.3075	0.90448	FAM134B	76353	25kb
ENSG00000145555	5	16640395	16961372	1062	125	120000	-2.7683	0.99718	MYO10	76353	25kb
ENSG00000176788	5	17040707	17301943	906	97	120000	-0.16529	0.56564	BASP1	76353	25kb
ENSG00000145526	5	19448060	20600982	3131	119	120000	0.50484	0.30683	CDH18	76353	25kb
ENSG00000154162	5	21725782	22878731	2881	118	120000	0.71176	0.23831	CDH12	76353	25kb
ENSG00000164256	5	23482264	23553706	127	25	120000	1.7654	0.038752	PRDM9	76353	25kb
ENSG00000248874	5	23926457	24203372	1003	30	120000	-0.38357	0.64935	C5orf17	76353	25kb
ENSG00000040731	5	24462209	24670087	725	48	120000	0.43522	0.3317	CDH10	76353	25kb
ENSG00000113100	5	26855709	27146257	718	43	120000	-0.40434	0.65702	CDH9	76353	25kb
ENSG00000113361	5	31168857	31354253	502	55	120000	0.070012	0.47209	CDH6	76353	25kb
ENSG00000113360	5	31375604	31557303	534	46	120000	-1.0183	0.84574	DROSHA	76353	25kb
ENSG00000082213	5	31507373	31580165	238	33	120000	0.80946	0.20912	C5orf22	76353	25kb
ENSG00000133401	5	31614517	32136037	1946	235	120000	1.2622	0.10344	PDZD2	76353	25kb
ENSG00000113384	5	32099810	32199456	209	25	120000	1.4198	0.077833	GOLPH3	76353	25kb
ENSG00000150712	5	32202100	32338115	368	21	120000	-0.029828	0.5119	MTMR12	76353	25kb
ENSG00000056097	5	32329456	32469867	381	30	120000	-1.4027	0.91965	ZFR	76353	25kb
ENSG00000113387	5	32506739	32629185	357	37	120000	-0.85503	0.80373	SUB1	76353	25kb
ENSG00000113389	5	32664176	32816819	422	48	120000	1.0505	0.14675	NPR3	76353	25kb
ENSG00000181495	5	32763945	32816819	116	16	120000	2.2562	0.012029	AC026703.1	76353	25kb
ENSG00000113407	5	33415802	33494644	202	20	120000	-1.5598	0.94059	TARS	76353	25kb
ENSG00000151388	5	33498640	33917297	1322	95	120000	0.15973	0.43655	ADAMTS12	76353	25kb
ENSG00000182631	5	33911491	33964023	149	20	120000	-0.81771	0.79324	RXFP3	76353	25kb
ENSG00000164175	5	33919721	34009835	221	43	120000	1.0551	0.14568	SLC45A2	76353	25kb
ENSG00000242110	5	33961283	34033220	190	28	120000	1.6962	0.044925	AMACR	76353	25kb
ENSG00000273294	5	33962279	34149633	325	32	120000	1.6821	0.046278	RP11-1084J3.4	76353	25kb
ENSG00000082196	5	33994553	34068937	191	20	120000	1.7142	0.043249	C1QTNF3	76353	25kb
ENSG00000039560	5	34631342	34857732	822	82	120000	-0.88034	0.81066	RAI14	76353	25kb
ENSG00000205838	5	34813938	34924561	291	35	120000	-0.9663	0.83305	TTC23L	76353	25kb
ENSG00000113456	5	34880369	34944094	124	20	120000	-0.94766	0.82835	RAD1	76353	25kb
ENSG00000113460	5	34890481	34951101	115	20	120000	-0.81647	0.79289	BRIX1	76353	25kb
ENSG00000168724	5	34904698	34984069	157	21	120000	-0.89276	0.81401	DNAJC21	76353	25kb
ENSG00000113492	5	34973206	35073198	350	40	120000	-1.8123	0.96503	AGXT2	76353	25kb
ENSG00000113494	5	35023861	35255794	756	59	120000	0.68719	0.24598	PRLR	76353	25kb
ENSG00000269027	5	35026012	35076198	180	29	120000	-1.6854	0.95405	AC010368.2	76353	25kb
ENSG00000152582	5	35592946	35839713	761	40	120000	-0.3832	0.64922	SPEF2	76353	25kb
ENSG00000168685	5	35827797	35904705	252	19	120000	-1.5461	0.93896	IL7R	76353	25kb
ENSG00000152611	5	35879397	35963881	261	23	120000	-1.4384	0.92484	CAPSL	76353	25kb
ENSG00000145626	5	35926112	36026130	255	23	120000	-0.26925	0.60613	UGT3A1	76353	25kb
ENSG00000168671	5	36010119	36096460	249	32	120000	-0.060361	0.52407	UGT3A2	76353	25kb
ENSG00000164187	5	36073514	36177063	199	28	120000	-0.35585	0.63902	LMBRD2	76353	25kb
ENSG00000145604	5	36127091	36209421	187	30	120000	-0.21498	0.58511	SKP2	76353	25kb
ENSG00000152620	5	36167694	36267381	233	22	120000	0.045119	0.48201	NADK2	76353	25kb
ENSG00000164188	5	36223536	36327216	234	25	120000	0.26125	0.39695	RANBP3L	76353	25kb
ENSG00000079215	5	36581457	36713436	360	61	120000	2.012	0.022112	SLC1A3	76353	25kb
ENSG00000164190	5	36851861	37091515	286	23	120000	0.62265	0.26676	NIPBL	76353	25kb
ENSG00000197603	5	37081330	37274530	239	20	120000	1.0213	0.15356	C5orf42	76353	25kb
ENSG00000113569	5	37263239	37396283	209	17	120000	2.8515	0.0021755	NUP155	76353	25kb
ENSG00000082068	5	37354314	37778537	926	33	120000	1.5513	0.06042	WDR70	76353	25kb
ENSG00000168621	5	37787779	37864788	265	28	120000	1.4353	0.075605	GDNF	76353	25kb
ENSG00000164318	5	38233511	38490123	735	69	120000	0.25638	0.39883	EGFLAM	76353	25kb
ENSG00000113594	5	38450065	38633456	287	31	120000	-1.285	0.9006	LIFR	76353	25kb
ENSG00000145623	5	38820960	38970698	407	44	120000	0.55517	0.28939	OSMR	76353	25kb
ENSG00000164327	5	38913021	39099510	385	19	120000	-0.14216	0.55652	RICTOR	76353	25kb
ENSG00000082074	5	39080338	39299630	583	42	120000	-0.70929	0.76093	FYB	76353	25kb
ENSG00000269215	5	39080358	39132350	121	23	120000	1.375	0.084566	AC008964.1	76353	25kb
ENSG00000113600	5	39259364	39449970	589	49	120000	-1.7113	0.95649	C9	76353	25kb
ENSG00000153071	5	39346780	39487402	455	50	120000	-1.7342	0.95856	DAB2	76353	25kb
ENSG00000171522	5	40654600	40718837	162	16	120000	-0.49202	0.68865	PTGER4	76353	25kb
ENSG00000113638	5	40689577	40781077	182	19	120000	-0.8497	0.80225	TTC33	76353	25kb
ENSG00000132356	5	40734481	40823476	176	21	120000	-0.90178	0.81641	PRKAA1	76353	25kb
ENSG00000145592	5	40800364	40860437	126	24	120000	-0.78168	0.7828	RPL37	76353	25kb
ENSG00000132357	5	40816286	40885275	167	19	120000	-0.71162	0.76165	CARD6	76353	25kb
ENSG00000112936	5	40884354	41008041	479	40	120000	-1.3903	0.91778	C7	76353	25kb
ENSG00000171495	5	40973119	41096444	498	44	120000	0.73896	0.22997	MROH2B	76353	25kb
ENSG00000039537	5	41117336	41286540	511	35	120000	-0.26732	0.60539	C6	76353	25kb
ENSG00000182836	5	41282056	41535730	594	39	120000	-1.9594	0.97496	PLCXD3	76353	25kb
ENSG00000083720	5	41705167	41895621	258	15	120000	-1.2521	0.89473	OXCT1	76353	25kb
ENSG00000205765	5	41879290	41946738	107	14	120000	-0.45176	0.67428	C5orf51	76353	25kb
ENSG00000151876	5	41900356	41966845	120	9	120000	0.026597	0.48939	FBXO4	76353	25kb
ENSG00000112964	5	42398879	42746979	730	44	120000	0.98329	0.16273	GHR	76353	25kb
ENSG00000198865	5	42731903	42827462	270	14	120000	-0.038882	0.51551	CCDC152	76353	25kb
ENSG00000250722	5	42774982	42912494	403	31	120000	-0.34816	0.63614	SEPP1	76353	25kb
ENSG00000177721	5	43014335	43068272	108	16	120000	-0.39032	0.65185	ANXA2R	76353	25kb
ENSG00000172262	5	43040278	43217123	507	25	120000	-0.77344	0.78037	ZNF131	76353	25kb
ENSG00000177453	5	43167173	43305952	343	38	120000	-1.4248	0.92289	NIM1K	76353	25kb
ENSG00000112972	5	43264497	43338614	182	10	120000	-0.57851	0.71854	HMGCS1	76353	25kb
ENSG00000151882	5	43351747	43437493	227	24	120000	-0.63981	0.73885	CCL28	76353	25kb
ENSG00000151881	5	43419354	43508995	219	20	120000	-0.67811	0.75115	C5orf28	76353	25kb

ENSG00000172244	5	43461803	43540247	175	18	120000	-0.56122	0.71268	C5orf34	76353	25kb
ENSG00000172239	5	43501369	43582860	175	17	120000	-0.59185	0.72302	PAIP1	76353	25kb
ENSG00000112992	5	43577794	43732507	356	16	120000	-0.22453	0.58883	NNT	76353	25kb
ENSG00000070193	5	44278646	44414808	332	25	120000	0.31826	0.37514	FGF10	76353	25kb
ENSG00000112996	5	44784027	44845530	120	7	120000	0.19329	0.42337	MRPS30	76353	25kb
ENSG00000164588	5	45234349	45721253	570	31	120000	0.28866	0.38642	HCN1	76353	25kb
ENSG00000170571	5	49667026	49764082	191	11	120000	0.072213	0.47122	EMB	76353	25kb
ENSG00000151883	5	49936733	50167356	437	25	120000	-0.85338	0.80328	PARP8	76353	25kb
ENSG00000016082	5	50653921	50715564	120	16	120000	1.4458	0.074116	ISL1	76353	25kb
ENSG00000248789	5	51278865	51401821	359	21	120000	0.45678	0.32391	CTD-2203A3.1	76353	25kb
ENSG00000213949	5	52058730	52280040	770	70	120000	0.45588	0.32424	ITGA1	76353	25kb
ENSG00000152684	5	52058774	52124880	252	21	120000	0.52781	0.29882	PELO	76353	25kb
ENSG00000164171	5	52260156	52415609	595	34	120000	-0.031073	0.51239	ITGA2	76353	25kb
ENSG00000164172	5	52366509	52430893	250	25	120000	0.74339	0.22862	MOCS2	76353	25kb
ENSG00000134363	5	52751239	52807964	168	30	120000	-1.2413	0.89274	FST	76353	25kb
ENSG00000164258	5	52831463	53004168	504	25	120000	-1.0159	0.84515	NDUFS4	76353	25kb
ENSG00000185305	5	53154775	53631412	1471	109	120000	0.77883	0.21804	ARL15	76353	25kb
ENSG00000169271	5	53726445	53777207	257	34	120000	-0.036961	0.51474	HSPB3	76353	25kb
ENSG00000178996	5	53788589	53867415	339	30	120000	-1.2409	0.89267	SNX18	76353	25kb
ENSG00000249016	5	54227063	54278674	195	29	120000	-0.21587	0.58546	RP11-45H22.3	76353	25kb
ENSG00000164283	5	54248692	54343499	295	33	120000	-0.27978	0.61018	ESM1	76353	25kb
ENSG00000113088	5	54295081	54355398	144	23	120000	-0.33501	0.63119	GZMK	76353	25kb
ENSG00000145649	5	54373476	54431080	112	12	120000	0.16794	0.43332	GZMA	76353	25kb
ENSG00000164287	5	54383799	54494005	271	16	120000	0.376	0.35346	CDC20B	76353	25kb
ENSG00000164294	5	54430946	54487899	142	13	120000	0.58661	0.27873	GPX8	76353	25kb
ENSG00000234602	5	54490442	54548143	176	20	120000	0.091152	0.46369	MCIDAS	76353	25kb
ENSG00000152669	5	54501980	54554508	140	17	120000	0.001954	0.49922	CCNO	76353	25kb
ENSG00000067248	5	54527073	54628550	199	18	120000	-0.41086	0.65941	DHX29	76353	25kb
ENSG00000039123	5	54578588	54746409	354	10	120000	-0.10004	0.53984	SKIV2L2	76353	25kb
ENSG00000067113	5	54695682	54855878	375	15	120000	0.021367	0.49148	PPAP2A	76353	25kb
ENSG00000177058	5	54896673	55094022	878	25	120000	0.26802	0.39434	SLC38A9	76353	25kb
ENSG00000152670	5	55008845	55137985	379	28	120000	-0.06547	0.5261	DDX4	76353	25kb
ENSG00000164509	5	55122207	55243678	295	34	120000	-0.17635	0.56999	IL31RA	76353	25kb
ENSG00000268280	5	55199468	55249569	103	26	120000	-0.79576	0.78691	AC008914.1	76353	25kb
ENSG00000134352	5	55205923	55315821	205	29	120000	-1.1483	0.87457	IL6ST	76353	25kb
ENSG00000164512	5	55370507	55554186	547	56	120000	-0.67632	0.75058	ANKRD55	76353	25kb
ENSG00000248727	5	55728622	55802773	357	49	120000	-0.86994	0.80783	CTC-236F12.4	76353	25kb
ENSG00000225940	5	55782394	55927059	641	80	120000	-1.2572	0.89567	AC022431.2	76353	25kb
ENSG00000095015	5	56086401	56216979	338	19	120000	-0.23803	0.59407	MAP3K1	76353	25kb
ENSG00000155542	5	56180087	56246359	193	18	120000	-0.44391	0.67145	SETD9	76353	25kb
ENSG00000155545	5	56190429	56292502	325	27	120000	-0.75848	0.77592	MIER3	76353	25kb
ENSG00000062194	5	56444775	56585506	219	27	120000	0.29271	0.38487	GPBP1	76353	25kb
ENSG00000169067	5	56750846	56803636	158	26	120000	-0.81704	0.79305	ACTBL2	76353	25kb
ENSG00000145632	5	57724809	57781087	232	28	120000	0.23189	0.40831	PLK2	76353	25kb
ENSG00000175857	5	57762262	57817917	201	19	120000	0.87759	0.19008	GAPT	76353	25kb
ENSG00000250127	5	57812394	57879070	123	16	120000	0.95251	0.17042	CTD-2117L12.1	76353	25kb
ENSG00000152932	5	57853048	58180213	1012	72	120000	0.86318	0.19402	RAB3C	76353	25kb
ENSG00000113448	5	58239865	59842947	3918	165	120000	1.5153	0.064843	PDE4D	76353	25kb
ENSG00000224838	5	59701565	59751939	131	15	120000	0.76538	0.22202	FKSG52	76353	25kb
ENSG00000035499	5	59867739	60021017	286	17	120000	2.1442	0.016009	DEPDC1B	76353	25kb
ENSG00000164181	5	60022618	60165216	338	26	120000	1.3812	0.083616	ELOVL7	76353	25kb
ENSG00000049167	5	60144658	60265900	281	18	120000	0.62778	0.26507	ERCC8	76353	25kb
ENSG00000164182	5	60215956	60473853	572	21	120000	0.74005	0.22963	NDUFAF2	76353	25kb
ENSG00000269267	5	60386538	60436762	105	13	120000	0.70391	0.24074	AC008498.1	76353	25kb
ENSG00000188725	5	60428536	60483301	105	14	120000	0.8729	0.19136	SMIM15	76353	25kb
ENSG00000130449	5	60603100	60866997	433	35	120000	-1.0032	0.84213	ZSWIM6	76353	25kb
ENSG00000178722	5	60908535	61072590	487	45	120000	-0.55261	0.70973	C5orf64	76353	25kb
ENSG00000068796	5	61576989	61858076	558	19	120000	0.47062	0.31895	KIF2A	76353	25kb
ENSG00000086189	5	61658081	61724766	139	15	120000	0.51461	0.30341	DIMT1	76353	25kb
ENSG00000086200	5	61674799	61949409	470	22	120000	0.4488	0.32679	IPO11	76353	25kb
ENSG00000268942	5	61783070	61833309	81	14	120000	0.72457	0.23436	CKS1B	76353	25kb
ENSG00000186105	5	61849562	61902275	79	12	120000	0.27343	0.39226	LRRC70	76353	25kb
ENSG00000178394	5	63231183	63283334	94	15	120000	-2.0916	0.98177	HTR1A	76353	25kb
ENSG00000164197	5	63436671	63693696	361	17	120000	-0.94961	0.82885	RNF180	76353	25kb
ENSG00000186479	5	63777084	63933139	353	37	120000	-1.2967	0.90263	RGS7BP	76353	25kb
ENSG00000145642	5	63961135	64038917	126	18	120000	-0.48782	0.68716	FAM159B	76353	25kb
ENSG00000153006	5	63988971	64089512	143	15	120000	-0.20106	0.57967	SREK1IP1	76353	25kb
ENSG00000153015	5	64039757	64339590	550	22	120000	0.62015	0.26758	CWC27	76353	25kb
ENSG00000049192	5	64419563	64802747	890	52	120000	0.45346	0.32511	ADAMTS6	76353	25kb
ENSG00000123219	5	64788593	64883998	287	14	120000	-0.25422	0.60034	CENPK	76353	25kb
ENSG00000113593	5	64834063	64908376	217	14	120000	0.15161	0.43975	PPWD1	76353	25kb
ENSG00000113595	5	64860507	64946802	251	16	120000	0.31523	0.37629	TRIM23	76353	25kb
ENSG00000113597	5	64895543	64987060	241	16	120000	0.16465	0.43461	TRAPPC13	76353	25kb
ENSG00000253251	5	64895592	64951718	168	13	120000	0.25289	0.40018	CTC-534A2.2	76353	25kb
ENSG00000197860	5	64936755	65043862	246	23	120000	-0.57559	0.71755	SGTB	76353	25kb
ENSG00000123213	5	64993023	65192553	570	43	120000	-0.85023	0.8024	NLN	76353	25kb
ENSG00000112851	5	65197303	65403377	273	21	120000	0.7646	0.22226	ERBB2IP	76353	25kb
ENSG00000153914	5	65410799	65504443	156	17	120000	0.48943	0.31227	SREK1	76353	25kb
ENSG00000069020	5	65867176	66490423	1627	106	120000	-0.17499	0.56946	MAST4	76353	25kb
ENSG00000134061	5	66453103	66517627	162	29	120000	0.15514	0.43836	CD180	76353	25kb
ENSG00000253787	5	67460704	67519235	225	31	120000	0.92489	0.17751	RP11-404L6.2	76353	25kb
ENSG00000145675	5	67486548	67622649	345	50	120000	0.26303	0.39626	PIK3R1	76353	25kb
ENSG00000145740	5	68364473	68451896	226	25	120000	-0.7008	0.75829	SLC30A5	76353	25kb
ENSG00000134057	5	68437837	68499072	149	14	120000	0.0039039	0.49844	CCNB1	76353	25kb
ENSG00000153044	5	68460375	68531184	184	12	120000	0.35984	0.35948	CENPH	76353	25kb
ENSG00000134056	5	68488587	68550956	158	13	120000	0.10639	0.45764	MRPS36	76353	25kb
ENSG00000134058	5	68505668	68598250	246	19	120000	0.012343	0.49508	CDK7	76353	25kb
ENSG00000183323	5	68551002	68653636	224	21	120000	0.099029	0.46056	CCDC125	76353	25kb
ENSG00000085231	5	68621811	68690840	118	15	120000	0.37725	0.35299	TAF9	76353	25kb
ENSG00000152942	5	68640120	68735628	164	13	120000	0.57933	0.28118	RAD17	76353	25kb
ENSG00000152939	5	68685939	68765157	131	12	120000	0.4463	0.32769	MARVELD2	76353	25kb
ENSG00000197822	5	68763119	68878931	75	16	120000	-1.2039	0.88568	OCLN	76353	25kb
ENSG00000183474	5	68831035	68915550	2	1	120000	0.88142	0.18905	GTF2H2C	76353	25kb
ENSG00000205572	5	69296074	69363940	1	1	120000	-1.0652	0.85661	SERF1B	76353	25kb
ENSG00000205571	5	69320350	69399349	1	1	120000	-1.0652	0.85661	SMN2	76353	25kb
ENSG00000172062	5	70195768	70274769	1	1	120000	-0.31189	0.62244	SMN1	76353	25kb

ENSG00000249437	5	70239310	70345941	8	3	120000	-0.91377	0.81958	NAIP	76353	25kb
ENSG00000145736	5	70305784	70388516	6	3	120000	-0.66959	0.74844	GTF2H2	76353	25kb
ENSG00000145734	5	70726442	70888649	357	13	120000	-0.85872	0.80475	BDP1	76353	25kb
ENSG00000131844	5	70858115	70979531	327	29	120000	-0.80088	0.7884	MCCC2	76353	25kb
ENSG00000164326	5	70989990	71041875	187	28	120000	-0.91398	0.81964	CARTPT	76353	25kb
ENSG00000131711	5	71378061	71530395	343	58	120000	-0.75097	0.77367	MAP1B	76353	25kb
ENSG00000113048	5	71490236	71641473	204	24	120000	-0.11739	0.54673	MRPS27	76353	25kb
ENSG00000049883	5	71591194	71681052	111	17	120000	-0.37648	0.64672	PTCD2	76353	25kb
ENSG00000178175	5	71713479	71828554	267	21	120000	2.7772	0.0027412	ZNF366	76353	25kb
ENSG00000083312	5	72087139	72237560	259	17	120000	-1.1728	0.87956	TNPO1	76353	25kb
ENSG00000157107	5	72226808	72411349	299	16	120000	-0.58312	0.72009	FCHO2	76353	25kb
ENSG00000157111	5	72391119	72452644	204	24	120000	-1.1756	0.88013	TMEM171	76353	25kb
ENSG00000164325	5	72444022	72495970	172	26	120000	-0.25666	0.60128	TMEM174	76353	25kb
ENSG00000251493	5	72715654	72769352	92	15	120000	-0.99557	0.84027	FOXD1	76353	25kb
ENSG00000145741	5	72769233	72826460	207	18	120000	-0.5629	0.71325	BTF3	76353	25kb
ENSG00000164331	5	72823160	72886511	196	22	120000	0.32893	0.3711	ANKRA2	76353	25kb
ENSG00000164338	5	72836268	72902794	168	15	120000	0.21251	0.41586	UTP15	76353	25kb
ENSG00000268217	5	72869203	72921845	135	15	120000	0.27555	0.39145	AC008387.1	76353	25kb
ENSG00000214944	5	72896983	73262818	1094	79	120000	0.94234	0.17301	ARHGEF28	76353	25kb
ENSG00000171617	5	73898234	73962249	166	28	120000	0.6359	0.26242	ENC1	76353	25kb
ENSG00000049860	5	73910848	74043472	366	56	120000	-0.71216	0.76182	HEXB	76353	25kb
ENSG00000164347	5	73992029	74088196	204	22	120000	-0.76228	0.77705	GFM2	76353	25kb
ENSG00000164346	5	74037817	74097737	144	16	120000	-0.63862	0.73847	NSA2	76353	25kb
ENSG00000198780	5	74048399	74187776	303	16	120000	-0.72845	0.76683	FAM169A	76353	25kb
ENSG00000176928	5	74298289	74351724	173	19	120000	-0.054475	0.52172	GCNT4	76353	25kb
ENSG00000145700	5	74339100	74557703	568	22	120000	0.14499	0.44236	ANKRD31	76353	25kb
ENSG00000113161	5	74607154	74682929	161	21	120000	-0.80694	0.79015	HMGCR	76353	25kb
ENSG00000113163	5	74639311	74832963	325	21	120000	-0.33255	0.63026	COL4A3BP	76353	25kb
ENSG00000122008	5	74782581	74921969	218	20	120000	-0.058178	0.5232	POLK	76353	25kb
ENSG00000189045	5	74882284	74992671	280	23	120000	1.1516	0.12473	ANKDD1B	76353	25kb
ENSG00000152359	5	74944949	75038313	275	20	120000	1.7902	0.036714	POC5	76353	25kb
ENSG00000122012	5	75353997	75674764	1029	74	120000	1.5067	0.065947	SV2C	76353	25kb
ENSG00000145703	5	75674074	76028957	1135	104	120000	-0.49736	0.69053	IQGAP2	76353	25kb
ENSG00000164220	5	75886307	75944259	285	26	120000	-1.3129	0.90539	F2RL2	76353	25kb
ENSG00000181104	5	75986868	76056606	208	21	120000	-0.80517	0.78964	F2R	76353	25kb
ENSG00000164251	5	76089758	76156140	168	24	120000	0.25828	0.39809	F2RL1	76353	25kb
ENSG00000171643	5	76120826	76242475	316	34	120000	-0.60521	0.72748	S100Z	76353	25kb
ENSG00000145708	5	76223538	76301983	246	35	120000	-0.93371	0.82477	CRHBP	76353	25kb
ENSG00000164252	5	76300076	76386059	233	32	120000	-0.31599	0.62399	AGGF1	76353	25kb
ENSG00000132846	5	76342897	76408148	172	28	120000	0.32338	0.3732	ZBED3	76353	25kb
ENSG00000113231	5	76481274	76750632	784	59	120000	1.2065	0.11382	PDE8B	76353	25kb
ENSG00000164253	5	76696795	76941436	837	46	120000	-0.1073	0.54272	WDR41	76353	25kb
ENSG00000171540	5	76899538	76960513	127	25	120000	-0.63405	0.73698	OTP	76353	25kb
ENSG00000171530	5	76961991	77189604	810	42	120000	0.06869	0.47262	TBCA	76353	25kb
ENSG00000132842	5	77271349	77615579	731	31	120000	0.29896	0.38249	AP3B1	76353	25kb
ENSG00000085365	5	77631339	77801562	415	18	120000	0.30758	0.3792	SCAMP1	76353	25kb
ENSG00000145685	5	77756038	78090844	754	74	120000	-0.703	0.75897	LHFPL2	76353	25kb
ENSG00000113273	5	78048032	78306910	811	51	120000	0.035429	0.48587	ARSB	76353	25kb
ENSG00000132837	5	78268438	78556861	715	44	120000	1.9536	0.025375	DMGDH	76353	25kb
ENSG00000132840	5	78340540	78410289	213	19	120000	1.4542	0.072951	BHMT2	76353	25kb
ENSG00000145692	5	78382602	78453108	163	20	120000	1.2695	0.10213	BHMT	76353	25kb
ENSG00000268012	5	78498714	78548911	86	11	120000	0.77754	0.21842	AC016559.1	76353	25kb
ENSG00000152409	5	78507012	78648038	286	18	120000	1.3002	0.096766	JMY	76353	25kb
ENSG00000152413	5	78643459	78835040	442	38	120000	1.2438	0.10679	HOMER1	76353	25kb
ENSG00000164329	5	78882943	79007471	326	23	120000	-0.78139	0.78271	PAPD4	76353	25kb
ENSG00000164309	5	78960700	79121063	460	62	120000	-1.309	0.90474	CMYA5	76353	25kb
ENSG00000177034	5	79250584	79312082	131	13	120000	-0.31287	0.62281	MTX3	76353	25kb
ENSG00000113296	5	79262134	79404110	395	27	120000	0.12015	0.45218	THBS4	76353	25kb
ENSG00000164300	5	79382050	79576898	703	75	120000	-0.99964	0.84126	SERINC5	76353	25kb
ENSG00000164299	5	79590444	79642661	231	27	120000	-0.15858	0.563	SPZ1	76353	25kb
ENSG00000039319	5	79678832	79800169	286	11	120000	0.40705	0.34198	ZFYVE16	76353	25kb
ENSG00000152380	5	79758788	79863382	222	32	120000	-0.34205	0.63384	FAM151B	76353	25kb
ENSG00000189127	5	79827574	79891307	178	33	120000	-0.22552	0.58921	ANKRD34B	76353	25kb
ENSG00000228716	5	79897047	79975802	340	12	120000	-0.45252	0.67455	DHFR	76353	25kb
ENSG00000271043	5	79920819	79971855	280	7	120000	-0.401	0.65579	MTRNR2L2	76353	25kb
ENSG00000113318	5	79925467	80197279	774	27	120000	-0.8038	0.78924	MSH3	76353	25kb
ENSG00000113319	5	80231491	80550975	921	94	120000	-0.073392	0.52925	RASGRF2	76353	25kb
ENSG00000131730	5	80504104	80587216	177	22	120000	-0.23884	0.59438	CKMT2	76353	25kb
ENSG00000131732	5	80572409	80634116	153	24	120000	-0.65054	0.74233	ZCCHC9	76353	25kb
ENSG00000172497	5	80600824	80714998	342	46	120000	-0.96603	0.83298	ACOT12	76353	25kb
ENSG00000145687	5	80683840	81072616	841	48	120000	-0.8922	0.81386	SSBP2	76353	25kb
ENSG00000152348	5	81242844	81597676	560	39	120000	-0.41187	0.65978	ATG10	76353	25kb
ENSG00000186468	5	81544177	81599396	128	20	120000	-0.12989	0.55167	RPS23	76353	25kb
ENSG00000205464	5	81550281	81707796	308	27	120000	0.50248	0.30767	ATP6AP1L	76353	25kb
ENSG00000174695	5	82323665	82398682	162	14	120000	-0.93718	0.82567	TMEM167A	76353	25kb
ENSG00000152422	5	82348317	82674606	748	36	120000	-1.0759	0.85901	XRCC4	76353	25kb
ENSG00000038427	5	82742284	82903122	401	49	120000	-1.2008	0.88509	VCAN	76353	25kb
ENSG00000145681	5	82908624	83042432	377	47	120000	-0.99772	0.84079	HAPLN1	76353	25kb
ENSG00000164176	5	83211373	83705611	1022	92	120000	-0.34589	0.63529	EDIL3	76353	25kb
ENSG00000127184	5	85888721	85941779	65	16	120000	0.6878	0.24579	COX7C	76353	25kb
ENSG00000233828	5	86487423	86559822	103	22	120000	-0.30655	0.62041	AC008394.1	76353	25kb
ENSG00000145715	5	86538705	86712748	234	22	120000	-0.035655	0.51422	RASA1	76353	25kb
ENSG00000134480	5	86662311	86733836	84	14	120000	-0.26069	0.60284	CCNH	76353	25kb
ENSG00000164180	5	87460450	87590293	203	25	120000	0.47018	0.31911	TMEM161B	76353	25kb
ENSG00000081189	5	87988975	88224922	407	34	120000	-0.6461	0.74089	MEF2C	76353	25kb
ENSG00000153140	5	89663078	89730603	73	14	120000	-0.10958	0.54363	CETN3	76353	25kb
ENSG00000176055	5	89729020	89795585	117	19	120000	-1.6316	0.94862	MBLAC2	76353	25kb
ENSG00000113356	5	89742565	89835370	175	25	120000	-1.0617	0.85582	POLR3G	76353	25kb
ENSG00000176018	5	89786428	89850401	121	15	120000	-0.1832	0.57268	LYSMD3	76353	25kb
ENSG00000164199	5	89800161	90485038	1553	75	120000	0.55439	0.28965	GPR98	76353	25kb
ENSG00000113369	5	90639541	90704176	112	18	120000	-0.46419	0.67874	ARRDC3	76353	25kb
ENSG00000175745	5	92894043	92955321	69	16	120000	-1.279	0.89955	NR2F1	76353	25kb
ENSG00000113391	5	92928775	93472404	715	28	120000	-0.44164	0.67062	FAM172A	76353	25kb
ENSG00000248483	5	93045508	93102343	80	12	120000	0.61797	0.2683	POU5F2	76353	25kb
ENSG00000185261	5	93463671	93979309	797	76	120000	0.1178	0.45311	KIAA0825	76353	25kb
ENSG00000133302	5	93929052	94100141	435	29	120000	0.27689	0.39093	ANKRD32	76353	25kb

ENSG00000175471	5	94014446	94645279	1570	123	120000	1.507	0.065907	MCTP1	76353	25kb
ENSG00000153347	5	94702048	94811158	347	32	120000	-1.3292	0.90812	FAM81B	76353	25kb
ENSG00000198677	5	94774599	94915711	282	15	120000	-0.79744	0.7874	TTC37	76353	25kb
ENSG00000164291	5	94865778	94965768	160	22	120000	-1.1619	0.87736	ARSK	76353	25kb
ENSG00000178015	5	94930782	94982846	88	25	120000	-1.4005	0.91932	GPR150	76353	25kb
ENSG00000175449	5	94957458	95045477	241	28	120000	-1.3104	0.90497	RFESD	76353	25kb
ENSG00000145757	5	94962885	95059415	247	29	120000	-1.3796	0.91614	SPATA9	76353	25kb
ENSG00000164292	5	95024226	95185087	423	55	120000	-0.6915	0.75537	RHOBTB3	76353	25kb
ENSG00000173221	5	95062023	95183709	304	40	120000	-0.27542	0.6085	GLRX	76353	25kb
ENSG00000236882	5	95162936	95220837	207	36	120000	0.77491	0.2192	C5orf27	76353	25kb
ENSG00000118985	5	95195802	95322775	274	17	120000	0.73318	0.23173	ELL2	76353	25kb
ENSG00000175426	5	95701119	95794847	189	29	120000	-0.43457	0.66806	PCSK1	76353	25kb
ENSG00000153113	5	95835971	96140299	1040	70	120000	0.74021	0.22959	CAST	76353	25kb
ENSG00000164307	5	96071521	96168803	411	28	120000	0.092559	0.46313	ERAP1	76353	25kb
ENSG00000247121	5	96124731	96296513	729	35	120000	-0.15587	0.56193	CTD-2260A17.2	76353	25kb
ENSG00000164308	5	96186643	96280420	416	25	120000	-0.31469	0.6235	ERAP2	76353	25kb
ENSG00000113441	5	96246098	96398219	436	22	120000	0.73119	0.23233	LNPEP	76353	25kb
ENSG00000251606	5	96399779	96544354	394	57	120000	-0.53953	0.70524	CTD-2215E18.1	76353	25kb
ENSG00000145721	5	96402574	96503576	252	38	120000	-0.29632	0.61651	LIX1	76353	25kb
ENSG00000058729	5	96471571	96543964	234	41	120000	-0.014496	0.50578	RIOK2	76353	25kb
ENSG00000174136	5	98079354	98159347	174	25	120000	0.82973	0.20335	RGMB	76353	25kb
ENSG00000153922	5	98165908	98287240	233	23	120000	-0.62743	0.73481	CHD1	76353	25kb
ENSG00000174132	5	99846009	99947445	189	16	120000	-1.4415	0.92527	FAM174A	76353	25kb
ENSG00000113532	5	100117639	100263970	327	16	120000	-0.68745	0.7541	ST8SIA4	76353	25kb
ENSG00000173930	5	101544690	101657253	461	37	120000	0.06401	0.47448	SLCO4C1	76353	25kb
ENSG00000268424	5	101544696	101596279	181	29	120000	0.90429	0.18292	AC008948.1	76353	25kb
ENSG00000205359	5	101682486	101859720	602	23	120000	-0.1331	0.55294	SLCO6A1	76353	25kb
ENSG00000145730	5	102064685	102391809	769	25	120000	0.56725	0.28527	PAM	76353	25kb
ENSG00000145723	5	102396704	102480855	266	10	120000	0.87647	0.19039	GIN1	76353	25kb
ENSG00000145725	5	102430853	102573500	321	13	120000	0.83917	0.20069	PPIP5K2	76353	25kb
ENSG00000181751	5	102569403	102639361	133	15	120000	0.097825	0.46104	C5orf30	76353	25kb
ENSG00000112874	5	102859556	102923494	174	26	120000	0.10473	0.4583	NUDT12	76353	25kb
ENSG00000184349	5	106687590	107031596	866	102	120000	-0.15575	0.56188	EFNA5	76353	25kb
ENSG00000145743	5	107169736	107742799	1319	46	120000	0.11981	0.45232	FBXL17	76353	25kb
ENSG00000151422	5	108058523	108557542	1798	55	120000	0.6668	0.25245	FER	76353	25kb
ENSG00000198961	5	108645410	108770695	419	17	120000	-0.41242	0.65998	PJA2	76353	25kb
ENSG00000112893	5	109000067	109230326	809	31	120000	-1.636	0.94908	MAN2A1	76353	25kb
ENSG00000254106	5	109193883	109245452	174	29	120000	-0.96191	0.83195	AC011366.3	76353	25kb
ENSG00000186952	5	109599934	110099657	1274	31	120000	-1.2459	0.89361	TMEM232	76353	25kb
ENSG00000164209	5	110048837	110125857	181	25	120000	-1.4174	0.92181	SLC25A46	76353	25kb
ENSG00000145777	5	110380760	110438722	135	27	120000	0.76246	0.22289	TSLP	76353	25kb
ENSG00000134987	5	110402414	110491200	261	24	120000	0.53647	0.29582	WDR36	76353	25kb
ENSG00000152495	5	110534351	110855584	832	52	120000	0.49278	0.31109	CAMK4	76353	25kb
ENSG00000164211	5	110806731	110873288	139	25	120000	0.85474	0.19635	STARD4	76353	25kb
ENSG00000134986	5	110973318	111358161	1069	60	120000	-0.39622	0.65403	NREP	76353	25kb
ENSG00000129595	5	111453138	111780013	916	53	120000	-0.24388	0.59634	EPB41L4A	76353	25kb
ENSG00000268358	5	111730280	111781310	86	12	120000	0.48636	0.31336	EPB41L4A-AS2	76353	25kb
ENSG00000134982	5	112018195	112206936	500	27	120000	-0.94658	0.82807	APC	76353	25kb
ENSG00000258864	5	112137910	112228279	272	18	120000	-0.89311	0.8141	CTC-554D6.1	76353	25kb
ENSG00000272869	5	112171885	112253776	264	16	120000	-0.67085	0.74884	CTC-487M23.8	76353	25kb
ENSG00000153037	5	112171919	112230485	192	17	120000	-0.62263	0.73324	SRP19	76353	25kb
ENSG00000129625	5	112187084	112283236	306	19	120000	-0.48924	0.68766	REEP5	76353	25kb
ENSG00000212643	5	112202313	112253791	175	15	120000	-0.48623	0.6866	ZRSR1	76353	25kb
ENSG00000172795	5	112287399	112381667	289	27	120000	-2.2392	0.98743	DCP2	76353	25kb
ENSG00000171444	5	112332796	112849527	1990	66	120000	-1.7784	0.96233	MCC	76353	25kb
ENSG00000212122	5	112743251	112795728	222	23	120000	-0.25217	0.59954	TSSK1B	76353	25kb
ENSG00000047188	5	112824380	112955982	533	19	120000	-0.70064	0.75824	YTHDC2	76353	25kb
ENSG00000080709	5	113671642	113857337	451	51	120000	-0.057795	0.52304	KCNN2	76353	25kb
ENSG00000152503	5	114435459	114541243	262	33	120000	1.0074	0.15687	TRIM36	76353	25kb
ENSG00000164219	5	114521527	114623569	206	28	120000	1.0323	0.15096	PGGT1B	76353	25kb
ENSG00000164221	5	114577885	114657528	166	28	120000	0.83023	0.2032	CCDC112	76353	25kb
ENSG00000145780	5	114831608	114905591	139	20	120000	0.087072	0.46531	FEM1C	76353	25kb
ENSG00000251201	5	114889339	114986858	217	20	120000	0.92632	0.17714	TMED7-TICAM2	76353	25kb
ENSG00000243414	5	114889339	114986876	217	20	120000	0.92632	0.17714	TICAM2	76353	25kb
ENSG00000134970	5	114924205	114993689	192	14	120000	0.83283	0.20247	TMED7	76353	25kb
ENSG00000129596	5	115115430	115177651	131	29	120000	-1.0279	0.84801	CDO1	76353	25kb
ENSG00000145782	5	115138893	115202555	95	23	120000	-0.65142	0.74261	ATG12	76353	25kb
ENSG00000177879	5	115152178	115274778	251	44	120000	-0.35826	0.63993	AP3S1	76353	25kb
ENSG00000172901	5	115273151	115388316	563	53	120000	0.67754	0.24903	AQPEP	76353	25kb
ENSG00000268714	5	115333088	115419625	427	52	120000	1.4309	0.076231	CTD-2287O16.3	76353	25kb
ENSG00000268223	5	115362163	115420815	290	25	120000	2.105	0.017647	ARL14EPL	76353	25kb
ENSG00000145781	5	115395688	115773459	1733	37	120000	3.1507	0.00081438	COMMD10	76353	25kb
ENSG00000092421	5	115754312	115935630	619	81	120000	0.33679	0.36814	SEMA6A	76353	25kb
ENSG00000169570	5	118148017	118349240	397	36	120000	-1.7013	0.95556	DTWD2	76353	25kb
ENSG00000172869	5	118348467	118609833	388	39	120000	-1.8151	0.96525	DMXL1	76353	25kb
ENSG00000145779	5	118579387	118760383	536	43	120000	-0.4444	0.67162	TNFAIP8	76353	25kb
ENSG00000133835	5	118763138	118997894	746	39	120000	-0.54295	0.70642	HSD17B4	76353	25kb
ENSG00000164334	5	118940254	118996517	222	20	120000	-0.87186	0.80836	FAM170A	76353	25kb
ENSG00000184838	5	119774973	120048027	1047	65	120000	0.0019094	0.49924	PRR16	76353	25kb
ENSG00000181867	5	121162650	121213519	169	23	120000	-0.13947	0.55546	FTMT	76353	25kb
ENSG00000151304	5	121272656	121436265	304	21	120000	0.47046	0.31901	SRFBP1	76353	25kb
ENSG00000113083	5	121373890	121438980	131	21	120000	-0.029399	0.51173	LOX	76353	25kb
ENSG00000164185	5	121440208	121540312	172	35	120000	0.54494	0.2929	ZNF474	76353	25kb
ENSG00000064692	5	121622049	121824914	287	25	120000	-0.17193	0.56825	SNCAIP	76353	25kb
ENSG00000205302	5	122085691	122190803	341	20	120000	-1.4788	0.9304	SNX2	76353	25kb
ENSG00000064652	5	122154134	122390049	732	27	120000	-0.13284	0.55284	SNX24	76353	25kb
ENSG00000168938	5	122333945	122397436	230	27	120000	0.6986	0.2424	PPIC	76353	25kb
ENSG00000061455	5	122399816	122554960	566	46	120000	-0.28816	0.61339	PRDM6	76353	25kb
ENSG00000168944	5	122655579	122784286	343	18	120000	-0.26773	0.60554	CEP120	76353	25kb
ENSG00000151292	5	122822793	122977739	247	19	120000	-0.55437	0.71034	CSNK1G3	76353	25kb
ENSG00000168916	5	123947608	124109500	486	60	120000	-1.3037	0.90384	ZNF608	76353	25kb
ENSG00000155324	5	125670824	125857186	560	75	120000	-0.4302	0.66647	GRAMD3	76353	25kb
ENSG00000164904	5	125852533	125956110	301	42	120000	0.48337	0.31442	ALDH7A1	76353	25kb
ENSG00000164902	5	125910960	125987944	235	27	120000	0.40638	0.34223	PHAX	76353	25kb
ENSG00000196900	5	125942414	125996979	172	25	120000	-0.18813	0.57461	C5orf48	76353	25kb
ENSG00000113368	5	126087315	126197712	359	32	120000	1.6692	0.047534	LMNB1	76353	25kb

ENSG00000173926	5	126178406	126391500	554	19	120000	-0.0076988	0.50307	MARCH3	76353	25kb
ENSG00000164241	5	126353250	126434184	220	15	120000	-0.23944	0.59462	C5orf63	76353	25kb
ENSG00000145794	5	126601523	126826429	737	30	120000	0.39887	0.345	MEGF10	76353	25kb
ENSG00000164244	5	126828301	126915781	240	20	120000	0.35092	0.36282	PRRC1	76353	25kb
ENSG00000205279	5	126959736	127019322	207	26	120000	0.26553	0.3953	CTXN3	76353	25kb
ENSG00000064651	5	127394458	127550380	233	20	120000	0.47914	0.31592	SLC12A2	76353	25kb
ENSG00000138829	5	127568601	128019878	1318	35	120000	-0.25312	0.59991	FBN2	76353	25kb
ENSG00000113396	5	127848706	128394335	2071	50	120000	-0.84237	0.80021	SLC27A6	76353	25kb
ENSG00000066583	5	128405444	128474721	229	29	120000	1.0357	0.15017	ISOC1	76353	25kb
ENSG00000145808	5	128770958	129099376	852	34	120000	1.506	0.066036	ADAMTS19	76353	25kb
ENSG00000186367	5	129058772	129127425	154	21	120000	0.38275	0.35095	KIAA1024L	76353	25kb
ENSG00000198108	5	129215165	129547327	565	23	120000	0.0040854	0.49837	CHSY3	76353	25kb
ENSG00000169567	5	130469720	130532428	163	11	120000	-1.0852	0.86108	HINT1	76353	25kb
ENSG00000186687	5	130481503	130566119	199	13	120000	-0.97565	0.83538	LYRM7	76353	25kb
ENSG00000158985	5	130556186	130759140	253	16	120000	-0.95785	0.83093	CDC42SE2	76353	25kb
ENSG00000158987	5	130734614	130995929	359	14	120000	-0.73794	0.76972	RAPGEF6	76353	25kb
ENSG00000273217	5	130736584	131157676	572	15	120000	-0.72805	0.76671	CTC-432M15.3	76353	25kb
ENSG00000217128	5	130952407	131157710	268	12	120000	-0.65871	0.74496	FNIP1	76353	25kb
ENSG00000164398	5	131117683	131372936	378	19	120000	-1.0699	0.85767	ACSL6	76353	25kb
ENSG00000164399	5	131371222	131423897	96	11	120000	-1.1329	0.87138	IL3	76353	25kb
ENSG00000164400	5	131384483	131436859	91	13	120000	-0.90807	0.81808	CSF2	76353	25kb
ENSG00000072682	5	131502531	131656008	361	26	120000	-0.13208	0.55254	P4HA2	76353	25kb
ENSG00000131435	5	131568364	131634147	179	18	120000	-0.49628	0.69015	PDLIM4	76353	25kb
ENSG00000197208	5	131605136	131704899	264	17	120000	0.62768	0.26511	SLC22A4	76353	25kb
ENSG00000197375	5	131680444	131756306	201	18	120000	0.40322	0.34339	SLC22A5	76353	25kb
ENSG00000197536	5	131721328	131836736	315	24	120000	-0.59455	0.72393	C5orf56	76353	25kb
ENSG00000125347	5	131792301	131851490	175	18	120000	-1.124	0.86949	IRF1	76353	25kb
ENSG00000113525	5	131852136	131917530	87	14	120000	-0.88484	0.81188	IL5	76353	25kb
ENSG00000113522	5	131866711	132005313	182	14	120000	-0.71716	0.76336	RAD50	76353	25kb
ENSG00000169194	5	131966955	132021802	96	16	120000	-1.4255	0.92299	IL13	76353	25kb
ENSG00000113520	5	131984678	132043368	118	14	120000	-1.376	0.91559	IL4	76353	25kb
ENSG00000131437	5	132003320	132098330	152	10	120000	-0.91044	0.81871	KIF3A	76353	25kb
ENSG00000205089	5	132058137	132114856	75	5	120000	0.13945	0.44455	CCNI2	76353	25kb
ENSG00000164402	5	132061509	132167933	143	14	120000	-0.048235	0.51924	SEPT8	76353	25kb
ENSG00000198944	5	132124033	132177488	77	17	120000	-0.49006	0.68795	SOWAHA	76353	25kb
ENSG00000164403	5	132132833	132191590	98	15	120000	-0.34335	0.63433	SHROOM1	76353	25kb
ENSG00000164404	5	132171873	132227576	118	14	120000	0.10091	0.45981	GDF9	76353	25kb
ENSG00000164405	5	132177252	132228723	112	13	120000	0.12026	0.45214	UQCRQ	76353	25kb
ENSG00000164406	5	132183014	132235738	100	12	120000	0.17042	0.43234	LEAP2	76353	25kb
ENSG00000072364	5	132186071	132324326	299	15	120000	0.34725	0.3642	AFF4	76353	25kb
ENSG00000155329	5	132307677	132387296	190	17	120000	1.3359	0.090798	ZCCHC10	76353	25kb
ENSG00000170606	5	132362654	132467141	270	18	120000	1.2948	0.09769	HSPA4	76353	25kb
ENSG00000053108	5	132507147	132973255	1330	143	120000	1.3027	0.096343	FSTL4	76353	25kb
ENSG00000113583	5	133266201	133329478	117	23	120000	-0.57888	0.71867	C5orf15	76353	25kb
ENSG00000213585	5	133282606	133365824	171	25	120000	-0.38169	0.64865	VDAC1	76353	25kb
ENSG00000081059	5	133425402	133512556	174	19	120000	-0.80762	0.79035	TCF7	76353	25kb
ENSG00000113558	5	133459633	133537729	148	14	120000	-0.46978	0.68075	SKP1	76353	25kb
ENSG00000272772	5	133477861	133586762	231	14	120000	-0.66847	0.74808	CTD-2410N18.5	76353	25kb
ENSG00000113575	5	133505025	133586833	194	12	120000	-0.75281	0.77422	PPP2CA	76353	25kb
ENSG00000006837	5	133516305	133731738	384	12	120000	-0.421	0.66312	CDKL3	76353	25kb
ENSG00000119048	5	133681870	133752683	103	7	120000	-0.070929	0.52827	UBE2B	76353	25kb
ENSG00000237190	5	133712778	133772589	72	7	120000	-0.24569	0.59704	CDKN2AIPNL	76353	25kb
ENSG00000043143	5	133835003	133943918	256	30	120000	1.4243	0.077178	JADE2	76353	25kb
ENSG00000152700	5	133911834	134009961	140	22	120000	-0.9103	0.81867	SAR1B	76353	25kb
ENSG00000113615	5	133959479	134088513	147	23	120000	-0.82247	0.7946	SEC24A	76353	25kb
ENSG00000164615	5	134049191	134112847	61	16	120000	-1.7107	0.95643	CAMLG	76353	25kb
ENSG00000145833	5	134069469	134215823	154	34	120000	-1.494	0.93241	DDX46	76353	25kb
ENSG00000181904	5	134156370	134220427	70	22	120000	-1.073	0.85836	C5orf24	76353	25kb
ENSG00000113621	5	134184493	134262215	102	23	120000	-0.17949	0.57122	TXNDC15	76353	25kb
ENSG00000132570	5	134215596	134368649	240	32	120000	-0.52499	0.7002	PCBD2	76353	25kb
ENSG00000152705	5	134278596	134372392	157	20	120000	-0.88225	0.81118	CATSPER3	76353	25kb
ENSG00000069011	5	134338425	134395503	145	16	120000	-0.75517	0.77493	PITX1	76353	25kb
ENSG00000224186	5	134343970	134716744	901	110	120000	0.62432	0.26621	C5orf66	76353	25kb
ENSG00000113648	5	134644590	134760604	185	23	120000	-0.75434	0.77468	H2AFY	76353	25kb
ENSG00000251380	5	134754905	134808038	186	31	120000	-0.68622	0.75371	C5orf20	76353	25kb
ENSG00000255833	5	134754908	134813089	200	32	120000	-0.63541	0.73742	TIFAB	76353	25kb
ENSG00000181965	5	134844991	134896639	114	18	120000	1.2673	0.10252	NEUROG1	76353	25kb
ENSG00000145824	5	134881373	134939969	129	20	120000	1.376	0.084411	CXCL14	76353	25kb
ENSG00000145832	5	135145338	135249326	398	40	120000	1.3357	0.09082	SLC25A48	76353	25kb
ENSG00000145839	5	135202935	135256516	217	30	120000	0.85024	0.1976	IL9	76353	25kb
ENSG00000145826	5	135232968	135315723	354	21	120000	0.9271	0.17694	LECT2	76353	25kb
ENSG00000120708	5	135339584	135424507	207	19	120000	-0.65321	0.74319	TGFBI	76353	25kb
ENSG00000113658	5	135443534	135549435	236	9	120000	-0.2533	0.59998	SMAD5	76353	25kb
ENSG00000069018	5	135523999	135757730	535	27	120000	-0.77587	0.78109	TRPC7	76353	25kb
ENSG00000152377	5	136285987	136959068	1768	99	120000	-1.2187	0.88852	SPOCK1	76353	25kb
ENSG00000146021	5	136928189	137096779	328	31	120000	-0.80027	0.78822	KLHL3	76353	25kb
ENSG00000177733	5	137062075	137115039	50	15	120000	-0.89782	0.81536	HNRNPA0	76353	25kb
ENSG00000120729	5	137178480	137248540	76	16	120000	-1.0773	0.85932	MYOT	76353	25kb
ENSG00000078795	5	137198657	137303436	159	19	120000	-1.1304	0.87085	PKD2L2	76353	25kb
ENSG00000031003	5	137248649	137412650	318	17	120000	-1.2591	0.896	FAM13B	76353	25kb
ENSG00000061492	5	137394581	137453054	122	18	120000	-1.7027	0.95569	WNT8A	76353	25kb
ENSG00000112981	5	137425866	137500132	107	20	120000	-1.5047	0.9338	NME5	76353	25kb
ENSG00000112983	5	137450455	137539675	117	18	120000	-1.1142	0.86741	BRD8	76353	25kb
ENSG00000112984	5	137489408	137548404	73	13	120000	-1.1603	0.87704	KIF20A	76353	25kb
ENSG00000094880	5	137498339	137574032	109	17	120000	-1.1174	0.8681	CDC23	76353	25kb
ENSG00000146013	5	137563068	137635360	122	21	120000	-1.4044	0.91991	GFRA3	76353	25kb
ENSG00000158402	5	137595954	137699044	197	24	120000	-0.23504	0.59291	CDC25C	76353	25kb
ENSG00000120709	5	137642624	137710416	141	21	120000	0.46759	0.32004	FAM53C	76353	25kb
ENSG00000120733	5	137663285	137797717	310	24	120000	1.319	0.09358	KDM3B	76353	25kb
ENSG00000132563	5	137749706	137807658	155	25	120000	1.256	0.10456	REEP2	76353	25kb
ENSG00000120738	5	137776179	137830004	139	22	120000	0.23454	0.40728	EGR1	76353	25kb
ENSG00000120705	5	137816784	137903989	160	21	120000	0.10015	0.46011	ETF1	76353	25kb
ENSG00000113013	5	137865571	137936133	128	14	120000	0.41312	0.33976	HSPA9	76353	25kb
ENSG00000044115	5	137921656	138295723	634	43	120000	-0.90364	0.81691	CTNNA1	76353	25kb
ENSG00000146006	5	138179612	138236057	82	4	120000	-0.73463	0.76872	LRRTM2	76353	25kb
ENSG00000120725	5	138257409	138654246	509	22	120000	0.4449	0.3282	SIL1	76353	25kb

ENSG00000015479	5	138584441	138692360	100	11	120000	-1.0432	0.85157	MATR3	76353	25kb
ENSG00000120727	5	138652276	138730406	84	14	120000	-1.4184	0.92196	PAIP2	76353	25kb
ENSG00000170482	5	138677885	138745242	69	12	120000	-1.409	0.92058	SLC23A1	76353	25kb
ENSG00000170476	5	138698169	138750770	51	7	120000	-1.0959	0.86344	MZB1	76353	25kb
ENSG00000228672	5	138702635	138755885	53	7	120000	-1.0763	0.8591	PROB1	76353	25kb
ENSG00000170469	5	138707252	138764777	63	7	120000	-1.007	0.84304	SPATA24	76353	25kb
ENSG00000170464	5	138719279	138805180	74	8	120000	-0.8423	0.80019	DNAJC18	76353	25kb
ENSG00000249751	5	138759245	138867328	71	13	120000	-0.3073	0.62069	ECSCR	76353	25kb
ENSG00000269071	5	138827108	138877506	53	13	120000	0.022633	0.49097	AC138517.1	76353	25kb
ENSG00000184584	5	138830119	138887520	70	16	120000	0.03661	0.4854	TMEM173	76353	25kb
ENSG00000131508	5	138881016	139033018	259	17	120000	-0.55576	0.71081	UBE2D2	76353	25kb
ENSG00000171604	5	139001884	139088467	141	21	120000	-0.47833	0.68379	CXXC5	76353	25kb
ENSG00000146005	5	139150406	139249051	163	24	120000	-0.15146	0.56019	PSD2	76353	25kb
ENSG00000158458	5	139201364	139447884	446	56	120000	1.9788	0.023921	NRG2	76353	25kb
ENSG00000185129	5	139462362	139521321	51	11	120000	1.098	0.13611	PURA	76353	25kb
ENSG00000182700	5	139480521	139533391	44	10	120000	1.6102	0.053673	IGIP	76353	25kb
ENSG00000120306	5	139529227	139686637	273	13	120000	2.1537	0.015632	CYSTM1	76353	25kb
ENSG00000113068	5	139599624	139707706	184	14	120000	2.4145	0.0078775	PFDN1	76353	25kb
ENSG00000113070	5	139687428	139751216	101	20	120000	1.1897	0.11709	HBEGF	76353	25kb
ENSG00000113073	5	139714787	139779728	72	18	120000	0.11087	0.45586	SLC4A9	76353	25kb
ENSG00000131503	5	139756399	139954163	158	25	120000	0.17849	0.42917	ANKHD1	76353	25kb
ENSG00000254996	5	139756493	139954154	158	25	120000	0.17849	0.42917	ANKHD1-EIF4EBP3	76353	25kb
ENSG00000213523	5	139891925	139962895	71	15	120000	0.23223	0.40818	SRA1	76353	25kb
ENSG00000243056	5	139902251	139954163	56	13	120000	0.11234	0.45528	EIF4EBP3	76353	25kb
ENSG00000113108	5	139912853	139998337	120	15	120000	-0.45721	0.67624	APBB3	76353	25kb
ENSG00000176087	5	139919041	139973688	62	14	120000	-0.00099612	0.5004	SLC35A4	76353	25kb
ENSG00000170458	5	139986313	140038286	108	12	120000	-0.93139	0.82417	CD14	76353	25kb
ENSG00000131495	5	139993325	140052370	122	14	120000	-0.92181	0.82169	NDUFA2	76353	25kb
ENSG00000113119	5	139994012	140049993	116	13	120000	-1.1066	0.86577	TMCO6	76353	25kb
ENSG00000113141	5	140001643	140067064	127	13	120000	-1.0968	0.86364	IK	76353	25kb
ENSG00000120314	5	140019261	140078709	103	10	120000	-0.78499	0.78377	WDR55	76353	25kb
ENSG00000256453	5	140025379	140078171	92	9	120000	-0.70566	0.7598	DND1	76353	25kb
ENSG00000170445	5	140027758	140096609	118	9	120000	-0.6313	0.73608	HARS	76353	25kb
ENSG00000112855	5	140046011	140103889	99	9	120000	-0.63724	0.73801	HARS2	76353	25kb
ENSG00000146007	5	140053265	140111248	104	9	120000	-0.58191	0.71968	ZMAT2	76353	25kb
ENSG00000204970	5	140140876	140416929	530	25	120000	-1.1143	0.86742	PCDHA1	76353	25kb
ENSG00000204969	5	140149444	140416929	514	23	120000	-0.98537	0.83778	PCDHA2	76353	25kb
ENSG00000255408	5	140155783	140416929	507	24	120000	-1.1235	0.86939	PCDHA3	76353	25kb
ENSG00000204967	5	140161659	140416929	494	27	120000	-1.0375	0.85024	PCDHA4	76353	25kb
ENSG00000204965	5	140176222	140416929	462	27	120000	-0.992	0.8394	PCDHA5	76353	25kb
ENSG00000081842	5	140182563	140416929	448	24	120000	-0.88557	0.81208	PCDHA6	76353	25kb
ENSG00000204963	5	140188969	140416929	426	27	120000	-0.91564	0.82007	PCDHA7	76353	25kb
ENSG00000204962	5	140195907	140416929	417	24	120000	-0.82735	0.79598	PCDHA8	76353	25kb
ENSG00000204961	5	140202048	140416929	404	24	120000	-0.84217	0.80015	PCDHA9	76353	25kb
ENSG00000250120	5	140210595	140416929	400	23	120000	-0.79818	0.78762	PCDHA10	76353	25kb
ENSG00000249034	5	140215341	140268224	80	14	120000	-0.49638	0.69019	AC005609.1	76353	25kb
ENSG00000249158	5	140223689	140416929	379	24	120000	-0.69729	0.75719	PCDHA11	76353	25kb
ENSG00000251664	5	140230058	140416929	373	23	120000	-0.70487	0.75955	PCDHA12	76353	25kb
ENSG00000239389	5	140236793	140416929	362	24	120000	-0.84306	0.8004	PCDHA13	76353	25kb
ENSG00000248383	5	140281302	140416929	289	24	120000	-0.94406	0.82743	PCDHAC1	76353	25kb
ENSG00000243232	5	140320820	140416936	218	23	120000	-1.0331	0.84923	PCDHAC2	76353	25kb
ENSG00000171815	5	140405979	140458512	92	17	120000	-0.37274	0.64533	PCDHB1	76353	25kb
ENSG00000112852	5	140449227	140501962	114	9	120000	0.30648	0.37962	PCDHB2	76353	25kb
ENSG00000113205	5	140455234	140508406	112	9	120000	0.32999	0.37071	PCDHB3	76353	25kb
ENSG00000081818	5	140476581	140530201	123	17	120000	-0.27249	0.60738	PCDHB4	76353	25kb
ENSG00000113209	5	140489800	140542703	122	22	120000	-0.98761	0.83833	PCDHB5	76353	25kb
ENSG00000113211	5	140504683	140557868	138	19	120000	-1.4385	0.92486	PCDHB6	76353	25kb
ENSG00000255622	5	140510577	140563639	145	18	120000	-1.5285	0.9368	PCDHB17	76353	25kb
ENSG00000113212	5	140527243	140580957	162	17	120000	-1.3698	0.91462	PCDHB7	76353	25kb
ENSG00000120322	5	140532371	140585081	150	16	120000	-1.4012	0.91942	PCDHB8	76353	25kb
ENSG00000196963	5	140535980	140590793	149	16	120000	-1.3547	0.91224	PCDHB16	76353	25kb
ENSG00000120324	5	140546942	140600215	151	18	120000	-1.2758	0.89898	PCDHB10	76353	25kb
ENSG00000197479	5	140554183	140607618	147	20	120000	-1.139	0.87266	PCDHB11	76353	25kb
ENSG00000120328	5	140563269	140616696	149	20	120000	-0.9623	0.83205	PCDHB12	76353	25kb
ENSG00000187372	5	140568509	140621993	146	20	120000	-0.7912	0.78559	PCDHB13	76353	25kb
ENSG00000120327	5	140577931	140630858	132	19	120000	-0.69443	0.75629	PCDHB14	76353	25kb
ENSG00000113248	5	140600147	140652799	129	18	120000	-0.020529	0.50819	PCDHB15	76353	25kb
ENSG00000120329	5	140657196	140708612	162	20	120000	0.087385	0.46518	SLC25A2	76353	25kb
ENSG00000178913	5	140673057	140725330	155	22	120000	-0.095533	0.53805	TAF7	76353	25kb
ENSG00000204956	5	140685252	140917546	543	40	120000	0.48786	0.31282	PCDHGA1	76353	25kb
ENSG00000081853	5	140693539	140917546	514	36	120000	0.53274	0.29711	PCDHGA2	76353	25kb
ENSG00000254245	5	140698601	140917546	496	45	120000	0.61751	0.26845	PCDHGA3	76353	25kb
ENSG00000254221	5	140704828	140917546	474	37	120000	0.63803	0.26173	PCDHGB1	76353	25kb
ENSG00000262576	5	140709768	140917546	465	43	120000	0.62497	0.26599	PCDHGA4	76353	25kb
ENSG00000253910	5	140714703	140917546	456	42	120000	0.63896	0.26142	PCDHGB2	76353	25kb
ENSG00000253485	5	140718898	140917546	451	35	120000	0.6569	0.25562	PCDHGA5	76353	25kb
ENSG00000262209	5	140724831	140917546	436	38	120000	0.69191	0.2445	PCDHGB3	76353	25kb
ENSG00000253731	5	140728651	140917546	431	37	120000	0.67063	0.25123	PCDHGA6	76353	25kb
ENSG00000253537	5	140737467	140917546	413	36	120000	0.5859	0.27897	PCDHGA7	76353	25kb
ENSG00000253953	5	140742452	140917546	399	38	120000	0.55134	0.2907	PCDHGB4	76353	25kb
ENSG00000253767	5	140747381	140917546	391	35	120000	0.52231	0.30073	PCDHGA8	76353	25kb
ENSG00000261934	5	140757520	140917546	367	34	120000	0.50796	0.30574	PCDHGA9	76353	25kb
ENSG00000253305	5	140762770	140917546	355	33	120000	0.48391	0.31423	PCDHGB6	76353	25kb
ENSG00000253846	5	140767743	140917546	340	33	120000	0.48095	0.31528	PCDHGA10	76353	25kb
ENSG00000254122	5	140772427	140917546	331	31	120000	0.45979	0.32283	PCDHGB7	76353	25kb
ENSG00000253873	5	140775762	140916835	318	30	120000	0.41021	0.34083	PCDHGA11	76353	25kb
ENSG00000253159	5	140785185	140917546	294	29	120000	0.36702	0.3568	PCDHGA12	76353	25kb
ENSG00000240184	5	140830580	140917542	191	31	120000	0.2344	0.40734	PCDHGC3	76353	25kb
ENSG00000242419	5	140839741	140917546	165	29	120000	0.3084	0.37889	PCDHGC4	76353	25kb
ENSG00000240764	5	140843808	140917546	150	27	120000	0.39297	0.34717	PCDHGC5	76353	25kb
ENSG00000131504	5	140869583	141023622	278	27	120000	0.19189	0.42392	DIAPH1	76353	25kb
ENSG00000171720	5	140975443	141041437	156	32	120000	-0.25196	0.59946	HDAC3	76353	25kb
ENSG00000164620	5	140991517	141045644	138	31	120000	-0.28277	0.61132	RELL2	76353	25kb
ENSG00000197948	5	140993869	141055986	159	35	120000	-0.31304	0.62287	FCHSD1	76353	25kb
ENSG00000120318	5	141007968	141086788	261	46	120000	-0.58859	0.72193	ARAP3	76353	25kb
ENSG00000156453	5	141207938	141283811	236	36	120000	1.0788	0.14034	PCDH1	76353	25kb

ENSG00000081791	5	141278373	141346612	175	26	120000	0.047093	0.48122	KIAA0141	76353	25kb
ENSG00000113555	5	141298150	141374304	195	27	120000	0.29793	0.38288	PCDH12	76353	25kb
ENSG00000013561	5	141312893	141394856	231	22	120000	0.12786	0.44913	RNF14	76353	25kb
ENSG00000113552	5	141346314	141417606	238	27	120000	0.10981	0.45628	GNPDA1	76353	25kb
ENSG00000131507	5	141463070	141559005	331	24	120000	0.42569	0.33517	NDFIP1	76353	25kb
ENSG00000187678	5	141664992	141731020	188	23	120000	1.1355	0.12809	SPRY4	76353	25kb
ENSG00000113578	5	141946743	142102617	519	73	120000	-0.30561	0.62005	FGF1	76353	25kb
ENSG00000145819	5	142124949	142633576	1230	108	120000	2.5495	0.0053941	ARHGAP26	76353	25kb
ENSG00000113580	5	142632496	142840077	314	27	120000	2.0414	0.020608	NR3C1	76353	25kb
ENSG00000158497	5	143166726	143225284	152	19	120000	-1.9464	0.9742	HMHB1	76353	25kb
ENSG00000145817	5	143512723	143575278	161	15	120000	-0.51856	0.69797	YIPF5	76353	25kb
ENSG00000183775	5	143525396	143890249	637	30	120000	-0.086325	0.5344	KCTD16	76353	25kb
ENSG00000186314	5	144826362	145239932	1045	49	120000	0.97233	0.16544	PRELID2	76353	25kb
ENSG00000204928	5	145214296	145277531	153	31	120000	-0.59808	0.72511	GRXCR2	76353	25kb
ENSG00000156463	5	145291142	145486354	482	26	120000	-0.3474	0.63585	SH3RF2	76353	25kb
ENSG00000173261	5	145438949	145508932	182	8	120000	0.15463	0.43856	PLAC8L1	76353	25kb
ENSG00000133706	5	145467601	145587223	394	8	120000	0.096072	0.46173	LARS	76353	25kb
ENSG00000091009	5	145558113	145743814	392	25	120000	0.72904	0.23299	RBM27	76353	25kb
ENSG00000091010	5	145693587	145745083	134	20	120000	0.41019	0.34083	POU4F3	76353	25kb
ENSG00000113649	5	145801874	145916524	317	20	120000	-0.53087	0.70224	TCERG1	76353	25kb
ENSG00000173250	5	145867666	145920753	163	18	120000	-0.12869	0.5512	GPR151	76353	25kb
ENSG00000156475	5	145942936	146489347	1223	80	120000	1.5715	0.058033	PPP2R2B	76353	25kb
ENSG00000169302	5	146589526	146792415	725	57	120000	1.1034	0.13493	STK32A	76353	25kb
ENSG00000113657	5	146745374	146914619	208	39	120000	-0.64723	0.74126	DPYSL3	76353	25kb
ENSG00000176049	5	146942990	147187338	629	35	120000	-0.22428	0.58873	JAKMIP2	76353	25kb
ENSG00000164266	5	147179131	147236349	160	29	120000	0.019735	0.49213	SPINK1	76353	25kb
ENSG00000164265	5	147225245	147286754	207	27	120000	0.52074	0.30128	SCGB3A2	76353	25kb
ENSG00000178776	5	147235289	147311101	263	30	120000	0.37356	0.35437	C5orf46	76353	25kb
ENSG00000133710	5	147380246	147541852	636	22	120000	0.86753	0.19283	SPINK5	76353	25kb
ENSG00000196800	5	147524296	147579961	240	7	120000	-1.1945	0.88387	SPINK14	76353	25kb
ENSG00000178172	5	147557357	147619700	170	7	120000	-1.0768	0.85921	SPINK6	76353	25kb
ENSG00000214510	5	147622743	147690817	112	13	120000	-0.55682	0.71117	SPINK13	76353	25kb
ENSG00000145879	5	147666982	147720485	86	8	120000	-1.3891	0.91761	SPINK7	76353	25kb
ENSG00000268126	5	147673550	147726891	104	7	120000	-1.4123	0.92107	AC091948.1	76353	25kb
ENSG00000204909	5	147675766	147744412	128	8	120000	-1.3435	0.91044	SPINK9	76353	25kb
ENSG00000145868	5	147738498	147847399	176	16	120000	-0.74669	0.77238	FBXO38	76353	25kb
ENSG00000164270	5	147805595	148081798	685	46	120000	-1.0274	0.84788	HTR4	76353	25kb
ENSG00000169252	5	148181156	148233196	155	29	120000	0.11233	0.45528	ADRB2	76353	25kb
ENSG00000169247	5	148278202	148467726	541	45	120000	0.86905	0.19241	SH3TC2	76353	25kb
ENSG00000173210	5	148496046	148665105	435	37	120000	-1.1901	0.883	ABLIM3	76353	25kb
ENSG00000157510	5	148626434	148746365	268	44	120000	0.37303	0.35456	AFAP1L1	76353	25kb
ENSG00000164284	5	148699993	148759146	108	17	120000	0.62242	0.26683	GRPEL2	76353	25kb
ENSG00000145882	5	148712570	148774216	117	14	120000	0.60842	0.27146	PCYOX1L	76353	25kb
ENSG00000127743	5	148725887	148808765	246	37	120000	-0.45893	0.67686	IL17B	76353	25kb
ENSG00000113712	5	148846760	148956007	177	26	120000	0.7899	0.21479	CSNK1A1	76353	25kb
ENSG00000183111	5	148906510	149039531	356	31	120000	-0.015528	0.50619	ARHGEF37	76353	25kb
ENSG00000155846	5	149084861	149259585	547	45	120000	-0.53284	0.70293	PPARGC1B	76353	25kb
ENSG00000132915	5	149212519	149349356	369	52	120000	0.69264	0.24427	PDE6A	76353	25kb
ENSG00000155850	5	149315300	149398018	181	27	120000	-0.016284	0.5065	SLC26A2	76353	25kb
ENSG00000164296	5	149347681	149405730	113	12	120000	0.2819	0.38901	TIGD6	76353	25kb
ENSG00000113716	5	149354884	149457386	249	23	120000	-0.23142	0.59151	HMGXB3	76353	25kb
ENSG00000182578	5	149407854	149517935	303	45	120000	-0.059356	0.52367	CSF1R	76353	25kb
ENSG00000113721	5	149468400	149560435	248	50	120000	-0.15974	0.56346	PDGFRB	76353	25kb
ENSG00000113722	5	149521358	149589120	210	34	120000	-1.14	0.87285	CDX1	76353	25kb
ENSG00000011083	5	149544520	149627351	246	33	120000	0.089843	0.46421	SLC6A7	76353	25kb
ENSG00000070808	5	149574054	149694854	312	49	120000	0.3804	0.35182	CAMK2A	76353	25kb
ENSG00000183876	5	149650906	149743870	195	33	120000	0.76365	0.22254	ARSI	76353	25kb
ENSG00000070814	5	149712202	149804871	202	30	120000	0.75404	0.22541	TCOF1	76353	25kb
ENSG00000019582	5	149756200	149817492	143	32	120000	0.29564	0.38375	CD74	76353	25kb
ENSG00000164587	5	149797753	149854319	171	17	120000	-0.40829	0.65847	RPS14	76353	25kb
ENSG00000070614	5	149840381	149962773	301	33	120000	0.68142	0.2478	NDST1	76353	25kb
ENSG00000171992	5	149955642	150063782	237	32	120000	-0.39059	0.65195	SYNPO	76353	25kb
ENSG00000164591	5	150015436	150083927	97	11	120000	-1.1678	0.87856	MYOZ3	76353	25kb
ENSG00000086589	5	150045356	150105669	84	8	120000	-0.98869	0.83859	RBM22	76353	25kb
ENSG00000132912	5	150063002	150163671	155	7	120000	-0.48788	0.68718	DCTN4	76353	25kb
ENSG00000256235	5	150132508	150201296	167	12	120000	-0.97908	0.83623	SMIM3	76353	25kb
ENSG00000269636	5	150132867	150201297	167	12	120000	-0.97908	0.83623	AC010441.1	76353	25kb
ENSG00000237693	5	150201085	150305298	283	11	120000	-0.66039	0.7455	IRGM	76353	25kb
ENSG00000145908	5	150248954	150309545	189	11	120000	-0.58138	0.71951	ZNF300	76353	25kb
ENSG00000211445	5	150375124	150433554	156	26	120000	0.38575	0.34984	GPX3	76353	25kb
ENSG00000145901	5	150384506	150498138	382	50	120000	0.4371	0.33102	TNIP1	76353	25kb
ENSG00000197043	5	150455273	150562443	446	55	120000	-0.73751	0.7696	ANXA6	76353	25kb
ENSG00000198624	5	150535613	150628706	374	54	120000	-2.2663	0.98828	CCDC69	76353	25kb
ENSG00000196743	5	150566711	150675001	401	48	120000	-1.8891	0.97056	GM2A	76353	25kb
ENSG00000186334	5	150631323	150708327	301	24	120000	-1.3418	0.91017	SLC36A3	76353	25kb
ENSG00000186335	5	150669539	150752151	316	27	120000	-1.0833	0.86066	SLC36A2	76353	25kb
ENSG00000123643	5	150791607	150896942	344	23	120000	0.12529	0.45015	SLC36A1	76353	25kb
ENSG00000086570	5	150858654	150973505	374	38	120000	-0.23538	0.59304	FAT2	76353	25kb
ENSG00000113140	5	151015657	151091726	182	27	120000	0.80195	0.21129	SPARC	76353	25kb
ENSG00000177556	5	151096877	151177093	162	18	120000	-0.19186	0.57607	ATOX1	76353	25kb
ENSG00000145907	5	151125606	151217346	201	17	120000	-0.29386	0.61557	G3BP1	76353	25kb
ENSG00000145888	5	151177074	151329403	345	20	120000	-0.66111	0.74573	GLRA1	76353	25kb
ENSG00000132911	5	151746093	151837929	357	12	120000	-0.17657	0.57008	NMUR2	76353	25kb
ENSG00000155511	5	152844175	153218429	1267	73	120000	-0.84925	0.80213	GRIA1	76353	25kb
ENSG00000055147	5	153344688	153443496	223	13	120000	-0.38118	0.64846	FAM114A2	76353	25kb
ENSG00000037749	5	153393466	153625038	623	37	120000	-1.1739	0.87978	MFAP3	76353	25kb
ENSG00000164574	5	153545290	153825544	938	68	120000	-2.082	0.98133	GALNT10	76353	25kb
ENSG00000164576	5	153800517	153865614	156	34	120000	-1.2963	0.90257	SAP30L	76353	25kb
ENSG00000113196	5	153829532	153882824	143	31	120000	-0.6877	0.75418	HAND1	76353	25kb
ENSG00000155506	5	154067462	154222167	284	27	120000	-0.6949	0.75644	LARP1	76353	25kb
ENSG00000170271	5	154173051	154263812	155	16	120000	-1.2343	0.89145	FAXDC2	76353	25kb
ENSG00000155508	5	154212113	154281353	137	13	120000	-1.5962	0.94477	CNOT8	76353	25kb
ENSG00000082516	5	154241976	154342769	237	19	120000	-1.615	0.94684	GEMIN5	76353	25kb
ENSG00000082515	5	154295630	154373971	175	21	120000	-1.5454	0.93887	MRPL22	76353	25kb
ENSG00000226650	5	154368260	154422685	200	16	120000	-1.214	0.88763	KIF4B	76353	25kb
ENSG00000170624	5	155272354	156219799	2685	116	120000	-0.69406	0.75618	SGCD	76353	25kb

ENSG00000145850	5	156321293	156415266	264	24	120000	-0.15562	0.56183	TIMD4	76353	25kb
ENSG00000113249	5	156431424	156511130	318	26	120000	-1.0005	0.84147	HAVCR1	76353	25kb
ENSG00000135077	5	156487843	156594880	326	23	120000	-0.92883	0.82351	HAVCR2	76353	25kb
ENSG00000155868	5	156539423	156611030	205	21	120000	-0.58069	0.71927	MED7	76353	25kb
ENSG00000113263	5	156544944	156707201	539	42	120000	-0.18879	0.57487	ITK	76353	25kb
ENSG00000170613	5	156563857	156618275	164	18	120000	-0.39958	0.65527	FAM71B	76353	25kb
ENSG00000055163	5	156668089	156847606	559	31	120000	1.6858	0.045916	CYFIP2	76353	25kb
ENSG00000172568	5	156743608	156797729	187	13	120000	1.4628	0.071758	FNDC9	76353	25kb
ENSG00000135074	5	156797542	157027783	783	35	120000	1.3667	0.085857	ADAM19	76353	25kb
ENSG00000172548	5	156862027	156926725	179	22	120000	1.2598	0.10388	NIPAL4	76353	25kb
ENSG00000039600	5	157027687	157123488	308	28	120000	0.65971	0.25472	SOX30	76353	25kb
ENSG00000187658	5	157073561	157132151	198	27	120000	0.38542	0.34996	C5orf52	76353	25kb
ENSG00000113272	5	157133205	157193456	195	27	120000	0.050065	0.48004	THG1L	76353	25kb
ENSG00000268432	5	157135003	157186727	176	26	120000	-0.13207	0.55254	AC026407.1	76353	25kb
ENSG00000155858	5	157145703	157212717	198	33	120000	0.82507	0.20467	LSM11	76353	25kb
ENSG00000113282	5	157187751	157311183	368	24	120000	0.70009	0.24194	CLINT1	76353	25kb
ENSG00000164330	5	158097928	158551769	923	70	120000	-1.5758	0.94246	EBF1	76353	25kb
ENSG00000145860	5	158559417	158662061	225	30	120000	0.78537	0.21612	RNF145	76353	25kb
ENSG00000164332	5	158665089	158738044	148	25	120000	0.26978	0.39366	UBLCP1	76353	25kb
ENSG00000113302	5	158716791	158782895	138	24	120000	1.0632	0.14385	IL12B	76353	25kb
ENSG00000170214	5	159318790	159424551	299	40	120000	0.78257	0.21694	ADRA1B	76353	25kb
ENSG00000113312	5	159411120	159517550	230	23	120000	1.0556	0.14559	TTC1	76353	25kb
ENSG00000170234	5	159463808	159571430	222	22	120000	0.88758	0.18738	PWWP2A	76353	25kb
ENSG00000170231	5	159589374	159690742	432	37	120000	-0.44883	0.67322	FABP6	76353	25kb
ENSG00000135083	5	159653659	159797403	409	49	120000	-1.649	0.95042	CCNJL	76353	25kb
ENSG00000145861	5	159749758	159822648	178	27	120000	-2.0222	0.97842	C1QTNF2	76353	25kb
ENSG00000221886	5	159795155	159852104	118	18	120000	-0.50105	0.69183	C5orf54	76353	25kb
ENSG00000164609	5	159803648	159873718	161	27	120000	-0.73647	0.76928	SLU7	76353	25kb
ENSG00000164611	5	159823829	159880748	145	28	120000	-0.93293	0.82457	PTTG1	76353	25kb
ENSG00000118322	5	159965127	160304221	1003	73	120000	1.5248	0.063656	ATP10B	76353	25kb
ENSG00000145864	5	160690436	161001050	839	48	120000	-0.55182	0.70946	GABRB2	76353	25kb
ENSG00000145863	5	160949069	161154599	477	36	120000	-1.1255	0.86981	GABRA6	76353	25kb
ENSG00000022355	5	161249197	161351975	264	18	120000	-1.1904	0.88305	GABRA1	76353	25kb
ENSG00000113327	5	161469546	161607542	261	35	120000	-2.1774	0.98527	GABRG2	76353	25kb
ENSG00000113328	5	162839575	162898157	113	16	120000	0.099214	0.46048	CCNG1	76353	25kb
ENSG00000268800	5	162841447	162898821	105	15	120000	0.14323	0.44305	AC112205.1	76353	25kb
ENSG00000170584	5	162848532	162912146	108	16	120000	0.3774	0.35294	NUDCD2	76353	25kb
ENSG00000072571	5	162862209	162943947	159	20	120000	0.36264	0.35844	HMMR	76353	25kb
ENSG00000038274	5	162905120	162971342	195	30	120000	0.37024	0.3556	MAT2B	76353	25kb
ENSG00000145934	5	166686804	167716162	2467	233	120000	-0.77874	0.78193	TENM2	76353	25kb
ENSG00000113645	5	167693656	167924308	754	95	120000	-0.64393	0.74019	WWC1	76353	25kb
ENSG00000113643	5	167888450	167971304	294	19	120000	1.3537	0.087916	RARS	76353	25kb
ENSG00000188573	5	167931121	167982639	173	13	120000	1.4793	0.069529	FBLL1	76353	25kb
ENSG00000120137	5	167950500	168031605	139	17	120000	3.1298	0.00087453	PANK3	76353	25kb
ENSG00000184347	5	168063745	168753133	2393	197	120000	-0.40927	0.65883	SLIT3	76353	25kb
ENSG00000040275	5	168985638	169056782	202	14	120000	1.3862	0.082844	SPDL1	76353	25kb
ENSG00000134516	5	169039251	169535386	1577	132	120000	-0.68457	0.75319	DOCK2	76353	25kb
ENSG00000204767	5	169266268	169432744	423	63	120000	-2.8123	0.99754	FAM196B	76353	25kb
ENSG00000168269	5	169507901	169561727	244	31	120000	1.4165	0.078309	FOXI1	76353	25kb
ENSG00000234511	5	169634451	169704579	149	30	120000	1.5289	0.063151	C5orf58	76353	25kb
ENSG00000043462	5	169648241	169750231	240	66	120000	1.2921	0.09816	LCP2	76353	25kb
ENSG00000182132	5	169755491	170188636	1492	164	120000	0.79552	0.21316	KCNIP1	76353	25kb
ENSG00000145936	5	169776675	169841681	269	44	120000	0.89349	0.1858	KCNMB1	76353	25kb
ENSG00000094755	5	170165354	170266051	339	43	120000	-0.43689	0.6689	GABRP	76353	25kb
ENSG00000204764	5	170263874	170752019	968	27	120000	-0.98034	0.83654	RANBP17	76353	25kb
ENSG00000164438	5	170711288	170764138	122	20	120000	-1.0867	0.86142	TLX3	76353	25kb
ENSG00000181163	5	170789120	170863141	200	20	120000	0.049411	0.4803	NPM1	76353	25kb
ENSG00000156427	5	170821660	170909627	250	44	120000	0.44896	0.32673	FGF18	76353	25kb
ENSG00000253110	5	171175656	171226743	152	21	120000	-0.23173	0.59163	CTB-78H18.1	76353	25kb
ENSG00000185662	5	171187876	171246602	178	29	120000	-0.85166	0.8028	SMIM23	76353	25kb
ENSG00000072803	5	171263553	171458877	312	30	120000	0.097323	0.46123	FBXW11	76353	25kb
ENSG00000072786	5	171444077	171640390	670	67	120000	-0.88138	0.81094	STK10	76353	25kb
ENSG00000214360	5	171596176	171655456	189	28	120000	-0.17814	0.57069	EFCAB9	76353	25kb
ENSG00000168246	5	171611644	171736075	346	21	120000	1.1435	0.12642	UBTD2	76353	25kb
ENSG00000174705	5	171727185	171906527	599	34	120000	1.6645	0.048011	SH3PXD2B	76353	25kb
ENSG00000268553	5	172011245	172061436	161	33	120000	-2.0907	0.98172	AC027309.1	76353	25kb
ENSG00000214357	5	172043269	172143543	272	38	120000	-1.0747	0.85874	NEURL1B	76353	25kb
ENSG00000120129	5	172170093	172223198	190	38	120000	-1.8246	0.96597	DUSP1	76353	25kb
ENSG00000113719	5	172236278	172404688	586	84	120000	-0.76468	0.77777	ERGIC1	76353	25kb
ENSG00000037241	5	172360732	172421774	186	31	120000	-0.13075	0.55201	RPL26L1	76353	25kb
ENSG00000113732	5	172385760	172487448	266	32	120000	-1.2819	0.90006	ATP6V0E1	76353	25kb
ENSG00000164463	5	172458355	172591291	314	24	120000	-0.8686	0.80747	CREBRF	76353	25kb
ENSG00000113734	5	172546445	172616390	179	24	120000	-1.1879	0.88257	BNIP1	76353	25kb
ENSG00000183072	5	172634112	172687360	139	26	120000	-1.496	0.93268	NKX2-5	76353	25kb
ENSG00000113739	5	172716716	172781506	203	48	120000	-0.69237	0.75565	STC2	76353	25kb
ENSG00000145919	5	173009517	173068663	175	25	120000	-0.019479	0.50777	BOD1	76353	25kb
ENSG00000113742	5	173290283	173413979	363	25	120000	-1.1201	0.86865	CPEB4	76353	25kb
ENSG00000185056	5	173375782	173458143	256	18	120000	0.0025817	0.49897	C5orf47	76353	25kb
ENSG00000170091	5	173447607	173695504	834	96	120000	1.4344	0.075724	NSG2	76353	25kb
ENSG00000120149	5	174126536	174182896	176	40	120000	-0.57182	0.71628	MSX2	76353	25kb
ENSG00000184845	5	174842042	174896211	170	30	120000	0.70784	0.23952	DRD1	76353	25kb
ENSG00000164466	5	174879065	174981745	377	32	120000	-0.24688	0.5975	SFXN1	76353	25kb
ENSG00000113749	5	175060033	175138245	154	27	120000	-1.3321	0.90859	HRH2	76353	25kb
ENSG00000145920	5	175198313	175336023	363	37	120000	0.81965	0.20621	CPLX2	76353	25kb
ENSG00000051596	5	175319876	175486683	65	18	120000	-1.3738	0.91524	THOC3	76353	25kb
ENSG00000248469	5	175451681	175514024	9	5	120000	-0.8842	0.81171	RP11-826N14.2	76353	25kb
ENSG00000182230	5	175462692	175584261	112	14	120000	-1.2585	0.89589	FAM153B	76353	25kb
ENSG00000170085	5	175640365	175797994	267	17	120000	-1.0635	0.85623	SIMC1	76353	25kb
ENSG00000122203	5	175748064	175813971	161	24	120000	-0.97591	0.83545	KIAA1191	76353	25kb
ENSG00000175414	5	175767471	175853866	188	33	120000	-1.2298	0.89062	ARL10	76353	25kb
ENSG00000048162	5	175785949	175840976	113	22	120000	-1.1696	0.87893	NOP16	76353	25kb
ENSG00000146066	5	175790748	175841772	109	21	120000	-1.0236	0.84699	HIGD2A	76353	25kb
ENSG00000175416	5	175794456	175868570	159	31	120000	-0.92278	0.82194	CLTB	76353	25kb
ENSG00000113194	5	175849629	175962075	240	29	120000	-0.011498	0.50459	FAF2	76353	25kb
ENSG00000146083	5	175928698	175990026	175	31	120000	0.17884	0.42903	RNF44	76353	25kb
ENSG00000074276	5	175944512	176047975	309	33	120000	-0.29247	0.61504	CDHR2	76353	25kb

ENSG00000169258	5	175997803	176062134	161	28	120000	0.52038	0.3014	GPRIN1	76353	25kb
ENSG00000074317	5	176022085	176082530	117	26	120000	2.5439	0.0054815	SNCB	76353	25kb
ENSG00000175766	5	176032683	176098642	177	29	120000	2.6282	0.004292	EIF4E1B	76353	25kb
ENSG00000048140	5	176049388	176111058	200	29	120000	2.9029	0.0018485	TSPAN17	76353	25kb
ENSG00000113763	5	176212478	176332897	261	53	120000	-1.0714	0.858	UNC5A	76353	25kb
ENSG00000160883	5	176282870	176351333	169	35	120000	-1.0197	0.84608	HK3	76353	25kb
ENSG00000087206	5	176307006	176474634	533	23	120000	-0.78898	0.78494	UIMC1	76353	25kb
ENSG00000113761	5	176424697	176533190	385	27	120000	-0.85557	0.80388	ZNF346	76353	25kb
ENSG00000160867	5	176488887	176550145	190	32	120000	-0.90116	0.81625	FGFR4	76353	25kb
ENSG00000165671	5	176535026	176752216	484	26	120000	-0.11107	0.54422	NSD1	76353	25kb
ENSG00000169228	5	176703199	176755745	118	22	120000	-0.025416	0.51014	RAB24	76353	25kb
ENSG00000213347	5	176703462	176764758	130	24	120000	-0.13008	0.55175	MXD3	76353	25kb
ENSG00000169230	5	176705775	176758960	121	23	120000	-0.12671	0.55042	PRELID1	76353	25kb
ENSG00000169223	5	176733563	176803853	120	19	120000	-0.74696	0.77246	LMAN2	76353	25kb
ENSG00000169220	5	176759838	176824602	97	13	120000	-1.2235	0.88943	RGS14	76353	25kb
ENSG00000131183	5	176781236	176850849	103	20	120000	-1.5566	0.94022	SLC34A1	76353	25kb
ENSG00000196570	5	176802108	176852637	70	19	120000	-1.2772	0.89923	PFN3	76353	25kb
ENSG00000131187	5	176804141	176861577	77	22	120000	-1.3896	0.91767	F12	76353	25kb
ENSG00000198055	5	176805205	176894902	133	23	120000	-1.299	0.90303	GRK6	76353	25kb
ENSG00000131188	5	176848446	176908283	96	11	120000	-0.7713	0.77974	PRR7	76353	25kb
ENSG00000113758	5	176858609	176926402	107	16	120000	-0.70362	0.75916	DBN1	76353	25kb
ENSG00000196923	5	176885395	176949607	117	29	120000	-0.37075	0.64459	PDLIM7	76353	25kb
ENSG00000146094	5	176903908	176963275	98	24	120000	0.028827	0.4885	DOK3	76353	25kb
ENSG00000183258	5	176913578	176969470	95	20	120000	0.28455	0.38799	DDX41	76353	25kb
ENSG00000146067	5	176921789	177006542	152	22	120000	0.52284	0.30054	FAM193B	76353	25kb
ENSG00000184840	5	176994159	177048125	166	18	120000	0.44106	0.32958	TMED9	76353	25kb
ENSG00000027847	5	177002101	177062348	180	20	120000	0.49562	0.31008	B4GALT7	76353	25kb
ENSG00000170074	5	177109982	177235399	66	11	120000	0.083061	0.4669	FAM153A	76353	25kb
ENSG00000249109	5	177184198	177246397	10	3	120000	-0.8662	0.80681	RP11-1026M7.2	76353	25kb
ENSG00000175325	5	177394236	177448243	157	31	120000	0.098557	0.46075	PROP1	76353	25kb
ENSG00000204677	5	177408406	177507560	181	29	120000	-1.2806	0.89982	FAM153C	76353	25kb
ENSG00000145911	5	177515444	177578088	169	27	120000	-0.79902	0.78786	N4BP3	76353	25kb
ENSG00000145916	5	177532997	177602566	216	26	120000	-1.1474	0.87439	RMND5B	76353	25kb
ENSG00000145912	5	177551461	177605968	184	25	120000	-1.1837	0.88174	NHP2	76353	25kb
ENSG00000197451	5	177606508	177663164	249	47	120000	-1.8894	0.97058	HNRNPAB	76353	25kb
ENSG00000175309	5	177610498	177684792	311	47	120000	-1.2142	0.88766	PHYKPL	76353	25kb
ENSG00000050767	5	177639619	178042556	1540	147	120000	-0.97142	0.83433	COL23A1	76353	25kb
ENSG00000113240	5	178004665	178082616	227	22	120000	-0.67581	0.75042	CLK4	76353	25kb
ENSG00000169131	5	178113593	178182703	254	20	120000	0.20978	0.41692	ZNF354A	76353	25kb
ENSG00000178338	5	178261954	178340123	507	25	120000	-1.3524	0.91187	ZNF354B	76353	25kb
ENSG00000198939	5	178297895	178385213	365	19	120000	-1.2565	0.89554	ZFP2	76353	25kb
ENSG00000178187	5	178343192	178418434	307	24	120000	-0.53347	0.70314	ZNF454	76353	25kb
ENSG00000113262	5	178380328	178448207	260	30	120000	-0.12156	0.54838	GRM6	76353	25kb
ENSG00000234284	5	178425753	178487065	188	15	120000	0.54464	0.293	ZNF879	76353	25kb
ENSG00000177932	5	178462416	178535538	168	19	120000	-0.0487	0.51942	ZNF354C	76353	25kb
ENSG00000087116	5	178512852	178797431	1031	143	120000	-0.11223	0.54468	ADAMTS2	76353	25kb
ENSG00000176783	5	178952559	179062027	313	24	120000	-0.11823	0.54706	RUFY1	76353	25kb
ENSG00000169045	5	179016179	179086785	174	25	120000	-0.82341	0.79486	HNRNPH1	76353	25kb
ENSG00000204661	5	179043545	179097047	124	26	120000	-0.93529	0.82518	C5orf60	76353	25kb
ENSG00000228259	5	179053298	179104445	107	27	120000	-1.2747	0.8988	AC136604.1	76353	25kb
ENSG00000204659	5	179080584	179132975	146	28	120000	-0.82548	0.79545	CBY3	76353	25kb
ENSG00000127022	5	179080629	179182926	272	28	120000	-0.86239	0.80576	CANX	76353	25kb
ENSG00000161021	5	179134851	179248512	287	23	120000	-0.65185	0.74275	MAML1	76353	25kb
ENSG00000213316	5	179195981	179248648	138	23	120000	-0.60606	0.72776	LTC4S	76353	25kb
ENSG00000161013	5	179199597	179258952	171	31	120000	-0.95783	0.83093	MGAT4B	76353	25kb
ENSG00000161011	5	179208388	179290078	244	39	120000	-1.5347	0.93757	SQSTM1	76353	25kb
ENSG00000161010	5	179236436	179314173	211	31	120000	-1.3582	0.9128	C5orf45	76353	25kb
ENSG00000245317	5	179261013	179313283	117	18	120000	-1.166	0.87819	CTC-241N9.1	76353	25kb
ENSG00000197226	5	179264066	179359859	193	21	120000	-0.8745	0.80908	TBC1D9B	76353	25kb
ENSG00000113269	5	179313651	179524118	517	39	120000	0.78472	0.21631	RNF130	76353	25kb
ENSG00000146090	5	179502795	179661153	658	89	120000	-0.29295	0.61522	RASGEF1C	76353	25kb
ENSG00000050748	5	179635143	179744099	431	36	120000	0.58566	0.27905	MAPK9	76353	25kb
ENSG00000131459	5	179702690	179805387	407	40	120000	1.1461	0.12588	GFPT2	76353	25kb
ENSG00000113300	5	179896412	180030405	516	25	120000	0.10823	0.45691	CNOT6	76353	25kb
ENSG00000161055	5	179992103	180043540	170	31	120000	0.18544	0.42644	SCGB3A1	76353	25kb
ENSG00000037280	5	180003506	180101624	405	58	120000	1.4842	0.068874	FLT4	76353	25kb
ENSG00000174339	5	180141042	180192099	128	19	120000	-0.30367	0.61931	OR2Y1	76353	25kb
ENSG00000131446	5	180192541	180267652	297	26	120000	-1.7661	0.96131	MGAT1	76353	25kb
ENSG00000196670	5	180249611	180313286	237	29	120000	-1.8824	0.97011	ZFP62	76353	25kb
ENSG00000113303	5	180301077	180402906	264	13	120000	-1.3573	0.91265	BTNL8	76353	25kb
ENSG00000168903	5	180390845	180458727	111	12	120000	0.016775	0.49331	BTNL3	76353	25kb
ENSG00000165810	5	180442225	180513523	269	32	120000	-0.53545	0.70383	BTNL9	76353	25kb
ENSG00000185372	5	180526357	180577304	186	26	120000	-2.0667	0.98062	OR2V1	76353	25kb
ENSG00000182613	5	180556943	180607890	222	29	120000	-1.8709	0.96932	OR2V2	76353	25kb
ENSG00000146054	5	180595924	180657293	296	30	120000	-0.61348	0.73022	TRIM7	76353	25kb
ENSG00000146063	5	180624499	180687809	251	37	120000	-0.32229	0.62639	TRIM41	76353	25kb
ENSG00000204628	5	180638909	180700096	212	28	120000	-0.78205	0.78291	GNB2L1	76353	25kb
ENSG00000183718	5	180656417	180713119	171	19	120000	-0.93918	0.82618	TRIM52	76353	25kb
ENSG00000268397	5	180657720	180709578	157	19	120000	-0.94871	0.82862	AC008443.1	76353	25kb
ENSG00000112679	6	266630	376355	111	35	120000	-0.84426	0.80074	DUSP22	76353	25kb
ENSG00000137265	6	366739	436447	240	44	120000	-1.7382	0.95891	IRF4	76353	25kb
ENSG00000112685	6	460133	718117	895	65	120000	0.52035	0.30141	EXOC2	76353	25kb
ENSG00000188996	6	630939	681963	166	20	120000	0.98101	0.16329	HUS1B	76353	25kb
ENSG00000176515	6	1055164	1130181	254	28	120000	0.31759	0.3754	AL033381.1	76353	25kb
ENSG00000164379	6	1287675	1339992	196	34	120000	0.37467	0.35395	FOXQ1	76353	25kb
ENSG00000137273	6	1365069	1420832	196	45	120000	-0.41185	0.65978	FOXF2	76353	25kb
ENSG00000054598	6	1585681	1639127	162	45	120000	-0.14139	0.55622	FOXC1	76353	25kb
ENSG00000112699	6	1599041	2270926	1686	128	120000	-0.0071049	0.50283	GMDS	76353	25kb
ENSG00000164385	6	2597147	2659837	209	36	120000	-0.48945	0.68774	C6orf195	76353	25kb
ENSG00000145949	6	2638863	2776200	600	56	120000	0.47986	0.31566	MYLK4	76353	25kb
ENSG00000124535	6	2740648	2812186	255	28	120000	0.10466	0.45832	WRNIP1	76353	25kb
ENSG00000021355	6	2807566	2867240	274	35	120000	0.067753	0.47299	SERPINB1	76353	25kb
ENSG00000170542	6	2862500	2928514	243	45	120000	0.35183	0.36248	SERPINB9	76353	25kb
ENSG00000124570	6	2923393	2997090	266	31	120000	-0.73258	0.76809	SERPINB6	76353	25kb
ENSG00000124588	6	2963221	3044996	454	39	120000	-1.3254	0.90748	NQO2	76353	25kb
ENSG00000137275	6	3039225	3140421	242	44	120000	0.79882	0.2122	RIPK1	76353	25kb

ENSG00000137274	6	3093608	3178812	156	22	120000	0.57643	0.28216	BPHL	76353	25kb
ENSG00000137267	6	3128903	3182760	116	13	120000	0.31281	0.37721	TUBB2A	76353	25kb
ENSG00000137285	6	3199495	3256964	101	14	120000	-0.96042	0.83158	TUBB2B	76353	25kb
ENSG00000180822	6	3206637	3328607	444	33	120000	-1.2126	0.88736	PSMG4	76353	25kb
ENSG00000137266	6	3244196	3482256	955	80	120000	-0.14626	0.55814	SLC22A23	76353	25kb
ENSG00000168994	6	3697848	3777260	409	40	120000	-0.15705	0.5624	PXDC1	76353	25kb
ENSG00000145945	6	3824620	3876551	173	28	120000	1.741	0.040845	FAM50B	76353	25kb
ENSG00000145965	6	3957909	4009372	165	29	120000	-1.801	0.96415	C6ORF50	76353	25kb
ENSG00000112739	6	3996501	4090217	283	22	120000	-0.052677	0.52101	PRPF4B	76353	25kb
ENSG00000145975	6	4024668	4112578	261	20	120000	-0.011239	0.50448	FAM217A	76353	25kb
ENSG00000185689	6	4054440	4156185	308	25	120000	0.25257	0.4003	C6orf201	76353	25kb
ENSG00000198721	6	4090923	4160831	219	28	120000	0.4457	0.32791	ECI2	76353	25kb
ENSG00000153046	6	4681393	4980785	907	85	120000	-2.1138	0.98273	CDYL	76353	25kb
ENSG00000124787	6	4969966	5029297	297	17	120000	-0.86822	0.80736	RPP40	76353	25kb
ENSG00000219607	6	5060720	5112455	215	24	120000	-1.023	0.84685	PPP1R3G	76353	25kb
ENSG00000214113	6	5077827	5286172	749	48	120000	0.64431	0.25969	LYRM4	76353	25kb
ENSG00000145982	6	5236277	5796813	1872	174	120000	1.5996	0.054839	FARS2	76353	25kb
ENSG00000124785	6	5973232	6032200	245	36	120000	-0.44159	0.67061	NRN1	76353	25kb
ENSG00000124491	6	6119318	6346246	829	115	120000	0.44498	0.32817	F13A1	76353	25kb
ENSG00000112799	6	6563341	6680216	516	66	120000	-1.0099	0.84373	LY86	76353	25kb
ENSG00000124782	6	7082830	7277213	453	54	120000	1.1048	0.13461	RREB1	76353	25kb
ENSG00000124783	6	7243539	7372679	535	37	120000	-0.92109	0.8215	SSR1	76353	25kb
ENSG00000164304	6	7301889	7414976	413	35	120000	-1.3383	0.9096	CAGE1	76353	25kb
ENSG00000124784	6	7364729	7443270	234	15	120000	-0.27789	0.60945	RIOK1	76353	25kb
ENSG00000096696	6	7516808	7611950	299	38	120000	-0.50915	0.69468	DSP	76353	25kb
ENSG00000168566	6	7565432	7637200	199	31	120000	0.27513	0.39161	SNRNP48	76353	25kb
ENSG00000153162	6	7702030	7906655	677	68	120000	-1.8654	0.96894	BMP6	76353	25kb
ENSG00000239264	6	7856483	8051646	711	66	120000	-1.6129	0.94661	TXNDC5	76353	25kb
ENSG00000259040	6	7856755	8089597	831	60	120000	-1.6598	0.95152	BLOC1S5-TXNDC5	76353	25kb
ENSG00000188428	6	7988800	8089647	388	24	120000	-0.63096	0.73597	BLOC1S5	76353	25kb
ENSG00000265818	6	7990959	8127763	501	33	120000	-0.92127	0.82155	EEF1E1-BLOC1S5	76353	25kb
ENSG00000124802	6	8048593	8127811	250	32	120000	-1.5545	0.93997	EEF1E1	76353	25kb
ENSG00000124786	6	8388301	8460794	303	17	120000	0.090915	0.46378	SLC35B3	76353	25kb
ENSG00000181355	6	9571343	10236841	2036	117	120000	0.4609	0.32244	OFCC1	76353	25kb
ENSG00000137203	6	10368419	10444892	213	50	120000	-0.88383	0.8116	TFAP2A	76353	25kb
ENSG00000111846	6	10467456	10654601	647	57	120000	-0.38071	0.64829	GCNT2	76353	25kb
ENSG00000205318	6	10608993	10672501	132	16	120000	0.76986	0.22069	GCNT6	76353	25kb
ENSG00000137434	6	10646651	10720030	73	15	120000	0.35363	0.36181	C6orf52	76353	25kb
ENSG00000111845	6	10669928	10735015	63	14	120000	-1.0028	0.84203	PAK1IP1	76353	25kb
ENSG00000111843	6	10698148	10756362	86	13	120000	-0.82572	0.79552	TMEM14C	76353	25kb
ENSG00000137210	6	10722992	10877986	335	40	120000	-0.090468	0.53604	TMEM14B	76353	25kb
ENSG00000272162	6	10723027	10955656	590	58	120000	0.59537	0.2758	RP11-637O19.3	76353	25kb
ENSG00000153157	6	10723027	11004553	702	68	120000	0.45672	0.32394	SYCP2L	76353	25kb
ENSG00000111837	6	10737956	10863764	266	32	120000	-0.19847	0.57866	MAK	76353	25kb
ENSG00000124827	6	10848456	10907174	188	30	120000	1.5773	0.057369	GCM2	76353	25kb
ENSG00000197977	6	10955992	11069547	232	20	120000	0.21313	0.41561	ELOVL2	76353	25kb
ENSG00000224531	6	11069266	11163964	314	27	120000	-0.013905	0.50555	SMIM13	76353	25kb
ENSG00000244476	6	11077722	11136965	213	24	120000	0.2582	0.39812	ERVFRD-1	76353	25kb
ENSG00000111859	6	11158531	11407581	926	98	120000	-1.416	0.92161	NEDD9	76353	25kb
ENSG00000205269	6	11512938	11608757	328	25	120000	-0.078175	0.53116	TMEM170B	76353	25kb
ENSG00000111863	6	11687287	11832279	488	52	120000	-1.5618	0.94083	ADTRP	76353	25kb
ENSG00000095951	6	11983995	12190232	585	47	120000	-0.86391	0.80618	HIVEP1	76353	25kb
ENSG00000078401	6	12265596	12322427	157	29	120000	-0.59883	0.72536	EDN1	76353	25kb
ENSG00000112137	6	12692893	13313645	1602	82	120000	-1.181	0.8812	PHACTR1	76353	25kb
ENSG00000145979	6	13241774	13353815	504	27	120000	-1.4856	0.93131	TBC1D7	76353	25kb
ENSG00000145990	6	13333062	13512894	565	56	120000	1.0671	0.14297	GFOD1	76353	25kb
ENSG00000187461	6	13444513	13511415	192	27	120000	2.031	0.02113	AL583828.1	76353	25kb
ENSG00000124523	6	13549816	13639790	152	25	120000	0.38811	0.34897	SIRT5	76353	25kb
ENSG00000225921	6	13590559	13657971	98	18	120000	-0.3251	0.62745	NOL7	76353	25kb
ENSG00000268059	6	13590728	13646126	86	17	120000	-0.39796	0.65467	AL441883.1	76353	25kb
ENSG00000010017	6	13596730	13736796	228	31	120000	-0.46091	0.67757	RANBP9	76353	25kb
ENSG00000050393	6	13761789	13839800	213	32	120000	1.3867	0.082772	MCUR1	76353	25kb
ENSG00000180537	6	13899677	14005533	237	20	120000	0.16212	0.43561	RNF182	76353	25kb
ENSG00000112149	6	14092872	14162149	236	37	120000	2.1782	0.014694	CD83	76353	25kb
ENSG00000008083	6	15221527	15547252	871	62	120000	-1.6856	0.95407	JARID2	76353	25kb
ENSG00000047579	6	15498032	15688289	458	34	120000	0.65481	0.25629	DTNBP1	76353	25kb
ENSG00000007944	6	16104356	16173479	125	24	120000	0.21396	0.41529	MYLIP	76353	25kb
ENSG00000137198	6	16213811	16320780	326	36	120000	-0.22263	0.58809	GMPR	76353	25kb
ENSG00000124788	6	16274343	16786722	1520	177	120000	-0.36623	0.6429	ATXN1	76353	25kb
ENSG00000230873	6	17077489	17156603	204	28	120000	-0.36155	0.64116	STMND1	76353	25kb
ENSG00000112183	6	17256577	17319106	173	30	120000	-0.22866	0.59043	RBM24	76353	25kb
ENSG00000112186	6	17368447	17583023	554	48	120000	2.4189	0.0077838	CAP2	76353	25kb
ENSG00000137414	6	17575586	17636950	160	28	120000	2.8192	0.0024072	FAM8A1	76353	25kb
ENSG00000124789	6	17590269	17731656	381	36	120000	2.4331	0.0074854	NUP153	76353	25kb
ENSG00000137177	6	17734414	18012854	765	37	120000	-0.3333	0.63055	KIF13A	76353	25kb
ENSG00000187566	6	18095718	18147851	212	24	120000	1.27	0.10205	NHLRC1	76353	25kb
ENSG00000137364	6	18103542	18180305	291	24	120000	0.7949	0.21334	TPMT	76353	25kb
ENSG00000165097	6	18130560	18249084	379	37	120000	-0.50283	0.69246	KDM1B	76353	25kb
ENSG00000124795	6	18199099	18290054	237	33	120000	-0.65791	0.7447	DEK	76353	25kb
ENSG00000137393	6	18343779	18494105	760	44	120000	0.48205	0.31488	RNF144B	76353	25kb
ENSG00000172201	6	19812617	19865915	191	45	120000	0.19136	0.42412	ID4	76353	25kb
ENSG00000172197	6	20075935	20237670	521	78	120000	-1.2946	0.90228	MBOAT1	76353	25kb
ENSG00000112242	6	20377398	20518941	310	45	120000	1.0139	0.15532	E2F3	76353	25kb
ENSG00000145996	6	20509688	21257635	2287	89	120000	-0.15749	0.56257	CDKAL1	76353	25kb
ENSG00000124766	6	21568972	21623847	63	9	120000	0.065544	0.47387	SOX4	76353	25kb
ENSG00000172179	6	22262480	22322730	204	35	120000	1.3158	0.094124	PRL	76353	25kb
ENSG00000112273	6	22544678	22596892	192	23	120000	-0.61552	0.73089	HDGFL1	76353	25kb
ENSG00000152954	6	24101350	24180128	284	40	120000	0.16607	0.43405	NRSN1	76353	25kb
ENSG00000146038	6	24146984	24383280	913	55	120000	-1.5321	0.93725	DCDC2	76353	25kb
ENSG00000146049	6	24332131	24383512	171	26	120000	-0.5096	0.69483	KAAG1	76353	25kb
ENSG00000124532	6	24378153	24450810	407	28	120000	-0.82147	0.79431	MRS2	76353	25kb
ENSG00000112293	6	24399793	24520433	705	39	120000	-0.72064	0.76443	GPLD1	76353	25kb
ENSG00000112294	6	24470080	24562435	432	31	120000	-0.4795	0.68421	ALDH5A1	76353	25kb
ENSG00000137261	6	24519332	24671383	551	52	120000	1.4719	0.07052	KIAA0319	76353	25kb
ENSG00000111802	6	24625205	24692261	257	21	120000	1.1861	0.11779	TDP2	76353	25kb
ENSG00000112304	6	24642263	24730293	278	28	120000	0.56892	0.28471	ACOT13	76353	25kb

ENSG00000112308	6	24680089	24746064	184	34	120000	0.28003	0.38973	C6orf62	76353	25kb
ENSG00000112312	6	24750159	24811327	199	36	120000	1.5137	0.065049	GMNN	76353	25kb
ENSG00000260286	6	24772549	24824117	156	22	120000	1.9321	0.026675	C6orf229	76353	25kb
ENSG00000111913	6	24772601	25067238	1011	83	120000	0.77296	0.21977	FAM65B	76353	25kb
ENSG00000079691	6	25254306	25645758	1597	80	120000	-1.8311	0.96646	LRRC16A	76353	25kb
ENSG00000079689	6	25627464	25727011	354	33	120000	-1.2118	0.88721	SCGN	76353	25kb
ENSG00000164508	6	25701291	25751790	228	15	120000	-0.85916	0.80487	HIST1H2AA	76353	25kb
ENSG00000146047	6	25702137	25752573	225	15	120000	-0.85879	0.80477	HIST1H2BA	76353	25kb
ENSG00000146039	6	25729927	25806419	279	18	120000	-0.93559	0.82526	SLC17A4	76353	25kb
ENSG00000124568	6	25758125	25857287	294	20	120000	-0.73192	0.76789	SLC17A1	76353	25kb
ENSG00000124564	6	25808294	25907514	289	22	120000	-0.81946	0.79374	SLC17A3	76353	25kb
ENSG00000112337	6	25887982	25955946	206	17	120000	-0.61798	0.73171	SLC17A2	76353	25kb
ENSG00000112343	6	25938030	26012384	172	19	120000	-0.75085	0.77363	TRIM38	76353	25kb
ENSG00000124610	6	25992260	26043040	116	19	120000	-0.618	0.73171	HIST1H1A	76353	25kb
ENSG00000198366	6	25995718	26046186	119	20	120000	-0.58184	0.71966	HIST1H3A	76353	25kb
ENSG00000196176	6	25996907	26047278	121	20	120000	-0.61458	0.73058	HIST1H4A	76353	25kb
ENSG00000124529	6	26002124	26052480	124	19	120000	-0.53363	0.7032	HIST1H4B	76353	25kb
ENSG00000124693	6	26006817	26057288	129	19	120000	-0.65137	0.7426	HIST1H3B	76353	25kb
ENSG00000137259	6	26008320	26058796	128	19	120000	-0.61775	0.73163	HIST1H2AB	76353	25kb
ENSG00000196226	6	26018455	26068885	122	17	120000	-0.62888	0.73529	HIST1H2BB	76353	25kb
ENSG00000196532	6	26020639	26071097	124	17	120000	-0.59246	0.72323	HIST1H3C	76353	25kb
ENSG00000187837	6	26030968	26081699	140	19	120000	-0.40309	0.65656	HIST1H1C	76353	25kb
ENSG00000010704	6	26062509	26123571	203	25	120000	-1.0106	0.8439	HFE	76353	25kb
ENSG00000197061	6	26079104	26129518	178	22	120000	-1.2612	0.89638	HIST1H4C	76353	25kb
ENSG00000187475	6	26082640	26133364	176	21	120000	-1.3116	0.90518	HIST1H1T	76353	25kb
ENSG00000180596	6	26090101	26149154	186	22	120000	-1.4513	0.92666	HIST1H2BC	76353	25kb
ENSG00000180573	6	26099373	26164344	176	23	120000	-1.6826	0.95377	HIST1H2AC	76353	25kb
ENSG00000168298	6	26131559	26182343	96	25	120000	-1.4586	0.92766	HIST1H1E	76353	25kb
ENSG00000158373	6	26133349	26196577	154	23	120000	-1.2011	0.88514	HIST1H2BD	76353	25kb
ENSG00000197697	6	26158958	26209454	183	17	120000	-0.67455	0.75002	HIST1H2BE	76353	25kb
ENSG00000188987	6	26163938	26214304	197	17	120000	-0.56976	0.71558	HIST1H4D	76353	25kb
ENSG00000197409	6	26172068	26224521	225	16	120000	-0.49688	0.69036	HIST1H3D	76353	25kb
ENSG00000196866	6	26174079	26224471	220	15	120000	-0.47608	0.68299	HIST1H2AD	76353	25kb
ENSG00000197846	6	26174748	26225942	225	15	120000	-0.45162	0.67423	HIST1H2BF	76353	25kb
ENSG00000198518	6	26179858	26231266	226	15	120000	-0.4576	0.67638	HIST1H4E	76353	25kb
ENSG00000187990	6	26191428	26241872	211	14	120000	-0.39848	0.65486	HIST1H2BG	76353	25kb
ENSG00000168274	6	26192165	26242711	206	14	120000	-0.38663	0.65048	HIST1H2AE	76353	25kb
ENSG00000196966	6	26200383	26250844	188	14	120000	-0.12904	0.55134	HIST1H3E	76353	25kb
ENSG00000124575	6	26209440	26260216	153	13	120000	-0.02594	0.51035	HIST1H1D	76353	25kb
ENSG00000198327	6	26215561	26265976	149	14	120000	0.0091156	0.49636	HIST1H4F	76353	25kb
ENSG00000124578	6	26221886	26272259	143	16	120000	0.14056	0.44411	HIST1H4G	76353	25kb
ENSG00000256316	6	26225370	26275835	135	17	120000	0.25551	0.39916	HIST1H3F	76353	25kb
ENSG00000197459	6	26226879	26277303	139	18	120000	0.27065	0.39333	HIST1H2BH	76353	25kb
ENSG00000256018	6	26246146	26296612	147	17	120000	0.074523	0.4703	HIST1H3G	76353	25kb
ENSG00000168242	6	26248144	26298622	147	17	120000	0.033034	0.48682	HIST1H2BI	76353	25kb
ENSG00000158406	6	26256283	26310762	178	18	120000	0.021185	0.49155	HIST1H4H	76353	25kb
ENSG00000186470	6	26340387	26403546	325	22	120000	-0.49899	0.69111	BTN3A2	76353	25kb
ENSG00000124508	6	26358324	26420102	328	22	120000	-0.66164	0.7459	BTN2A2	76353	25kb
ENSG00000026950	6	26377465	26440444	282	19	120000	-0.88449	0.81178	BTN3A1	76353	25kb
ENSG00000111801	6	26415700	26478643	223	18	120000	-0.45318	0.67479	BTN3A3	76353	25kb
ENSG00000112763	6	26433150	26501849	217	28	120000	-0.37733	0.64703	BTN2A1	76353	25kb
ENSG00000124557	6	26476449	26535650	159	18	120000	-0.62319	0.73342	BTN1A1	76353	25kb
ENSG00000182952	6	26513633	26571482	142	11	120000	-0.40862	0.65859	HMGN4	76353	25kb
ENSG00000146109	6	26572180	26625278	163	19	120000	-0.37227	0.64515	ABT1	76353	25kb
ENSG00000181315	6	26611518	26684980	163	14	120000	-0.42276	0.66377	ZNF322	76353	25kb
ENSG00000124635	6	27068676	27125541	96	25	120000	-0.81225	0.79168	HIST1H2BJ	76353	25kb
ENSG00000196787	6	27075832	27128070	92	25	120000	-0.98483	0.83765	HIST1H2AG	76353	25kb
ENSG00000197903	6	27081073	27139619	101	23	120000	-0.94781	0.82839	HIST1H2BK	76353	25kb
ENSG00000198339	6	27082076	27133418	93	23	120000	-0.95278	0.82965	HIST1H4I	76353	25kb
ENSG00000184825	6	27089861	27140317	91	22	120000	-0.82038	0.794	HIST1H2AH	76353	25kb
ENSG00000112812	6	27190480	27249403	170	25	120000	-0.079227	0.53157	PRSS16	76353	25kb
ENSG00000158553	6	27228682	27304949	284	29	120000	-1.4908	0.93199	POM121L2	76353	25kb
ENSG00000124613	6	27317394	27396683	349	17	120000	-1.6731	0.95284	ZNF391	76353	25kb
ENSG00000096654	6	27393522	27465897	203	14	120000	-1.2209	0.88894	ZNF184	76353	25kb
ENSG00000185130	6	27750257	27800709	94	19	120000	-0.064842	0.52585	HIST1H2BL	76353	25kb
ENSG00000196747	6	27750899	27801429	93	19	120000	-0.065348	0.52605	HIST1H2AI	76353	25kb
ENSG00000203813	6	27752842	27803314	94	19	120000	-0.073518	0.5293	HIST1H3H	76353	25kb
ENSG00000182611	6	27757112	27807607	97	19	120000	-0.087432	0.53484	HIST1H2AJ	76353	25kb
ENSG00000196374	6	27757822	27808267	95	18	120000	-0.070301	0.52802	HIST1H2BM	76353	25kb
ENSG00000197238	6	27766884	27817257	95	14	120000	-0.10023	0.53992	HIST1H4J	76353	25kb
ENSG00000197914	6	27773952	27824305	82	12	120000	-0.10374	0.54131	HIST1H4K	76353	25kb
ENSG00000184348	6	27780658	27831117	83	14	120000	-0.21304	0.58435	HIST1H2AK	76353	25kb
ENSG00000233822	6	27781323	27848487	121	16	120000	-0.18263	0.57245	HIST1H2BN	76353	25kb
ENSG00000198374	6	27808034	27858606	99	14	120000	-0.21987	0.58701	HIST1H2AL	76353	25kb
ENSG00000184357	6	27809570	27860359	101	14	120000	-0.2411	0.59526	HIST1H1B	76353	25kb
ENSG00000182572	6	27814623	27865099	100	15	120000	-0.2305	0.59115	HIST1H3I	76353	25kb
ENSG00000198558	6	27815926	27866289	99	16	120000	-0.32317	0.62672	HIST1H4L	76353	25kb
ENSG00000197153	6	27833093	27885884	127	20	120000	-0.84126	0.7999	HIST1H3J	76353	25kb
ENSG00000233224	6	27835477	27885963	115	19	120000	-0.9034	0.81684	HIST1H2AM	76353	25kb
ENSG00000196331	6	27836203	27886669	115	20	120000	-0.90805	0.81808	HIST1H2BO	76353	25kb
ENSG00000168131	6	27853963	27905174	111	23	120000	-0.65989	0.74534	OR2B2	76353	25kb
ENSG00000124657	6	27900019	27950960	105	17	120000	0.85361	0.19666	OR2B6	76353	25kb
ENSG00000197279	6	28023753	28082341	193	15	120000	-1.0532	0.85388	ZNF165	76353	25kb
ENSG00000196812	6	28067338	28122860	170	13	120000	-1.141	0.87306	ZSCAN16	76353	25kb
ENSG00000198315	6	28084688	28152250	203	14	120000	-1.2403	0.89257	ZKSCAN8	76353	25kb
ENSG00000137185	6	28167664	28226260	136	18	120000	-0.067258	0.52681	ZSCAN9	76353	25kb
ENSG00000187626	6	28187401	28252011	141	19	120000	-0.12771	0.55081	ZKSCAN4	76353	25kb
ENSG00000189134	6	28202098	28253736	100	16	120000	-0.33864	0.63256	NKAPL	76353	25kb
ENSG00000137338	6	28224314	28295326	163	23	120000	0.033686	0.48656	PGBD1	76353	25kb
ENSG00000235109	6	28267470	28349048	263	22	120000	-0.017858	0.50712	ZSCAN31	76353	25kb
ENSG00000189298	6	28292691	28361947	239	24	120000	0.17408	0.4309	ZKSCAN3	76353	25kb
ENSG00000158691	6	28321732	28392511	251	18	120000	0.56884	0.28473	ZSCAN12	76353	25kb
ENSG00000187987	6	28374707	28436279	195	20	120000	0.17479	0.43062	ZSCAN23	76353	25kb
ENSG00000198704	6	28446073	28520992	166	23	120000	-1.5024	0.93351	GPX6	76353	25kb
ENSG00000224586	6	28468702	28527729	149	24	120000	-1.3471	0.91103	GPX5	76353	25kb
ENSG00000232040	6	28514407	28608989	214	29	120000	-0.18079	0.57173	SCAND3	76353	25kb

ENSG00000204713	6	28845779	28916766	308	25	120000	-1.1741	0.87981	TRIM27	76353	25kb
ENSG00000204709	6	28886654	28937314	199	24	120000	-1.4835	0.93102	C6orf100	76353	25kb
ENSG00000197935	6	28937562	28998093	198	27	120000	-0.20259	0.58027	ZNF311	76353	25kb
ENSG00000204704	6	28986990	29038017	194	24	120000	-0.13141	0.55227	OR2W1	76353	25kb
ENSG00000204703	6	29028985	29080090	170	22	120000	-0.14097	0.55605	OR2B3	76353	25kb
ENSG00000204702	6	29043386	29094658	162	21	120000	-0.18771	0.57445	OR2J1	76353	25kb
ENSG00000204701	6	29054668	29105603	158	19	120000	-0.26891	0.606	OR2J3	76353	25kb
ENSG00000204700	6	29116311	29167351	198	18	120000	0.90991	0.18144	OR2J2	76353	25kb
ENSG00000204695	6	29249403	29300519	182	26	120000	-0.69143	0.75535	OR14J1	76353	25kb
ENSG00000243729	6	29298007	29424744	584	34	120000	-1.0445	0.85186	OR5V1	76353	25kb
ENSG00000112462	6	29316200	29368068	251	28	120000	-0.5896	0.72227	OR12D3	76353	25kb
ENSG00000168787	6	29339416	29390448	244	21	120000	-0.27264	0.60743	OR12D2	76353	25kb
ENSG00000204694	6	29368281	29449848	349	21	120000	-0.78965	0.78513	OR11A1	76353	25kb
ENSG00000206474	6	29382083	29433731	240	20	120000	-0.82357	0.79491	OR10C1	76353	25kb
ENSG00000204688	6	29399958	29457105	258	17	120000	-0.74517	0.77191	OR2H1	76353	25kb
ENSG00000204687	6	29429474	29480738	155	23	120000	-0.8803	0.81065	MAS1L	76353	25kb
ENSG00000213886	6	29498292	29552702	206	27	120000	-0.7861	0.78409	UBD	76353	25kb
ENSG00000204681	6	29498406	29626753	483	46	120000	-0.71979	0.76417	GABBR1	76353	25kb
ENSG00000204657	6	29530683	29581745	178	26	120000	0.38206	0.35121	OR2H2	76353	25kb
ENSG00000204655	6	29599758	29665149	485	35	120000	-0.94735	0.82827	MOG	76353	25kb
ENSG00000204644	6	29615169	29673887	552	28	120000	-1.0045	0.84242	ZFP57	76353	25kb
ENSG00000204642	6	29665552	29731305	743	28	120000	-0.23182	0.59166	HLA-F	76353	25kb
ENSG00000204632	6	29769744	29823902	919	29	120000	0.27686	0.39094	HLA-G	76353	25kb
ENSG00000206503	6	29884037	29938661	853	31	120000	-1.1027	0.86491	HLA-A	76353	25kb
ENSG00000066379	6	30001676	30057686	350	22	120000	-0.74111	0.77069	ZNRD1	76353	25kb
ENSG00000204619	6	30009486	30063110	352	21	120000	-0.59042	0.72255	PPP1R11	76353	25kb
ENSG00000204618	6	30013043	30068664	371	24	120000	-0.57046	0.71582	RNF39	76353	25kb
ENSG00000204616	6	30045674	30105883	447	37	120000	-1.0669	0.857	TRIM31	76353	25kb
ENSG00000204614	6	30078885	30141512	486	20	120000	-1.446	0.92592	TRIM40	76353	25kb
ENSG00000204613	6	30094722	30153711	398	20	120000	-1.5794	0.94288	TRIM10	76353	25kb
ENSG00000204610	6	30105993	30165473	348	23	120000	-1.7333	0.95848	TRIM15	76353	25kb
ENSG00000234127	6	30127232	30206204	338	26	120000	-1.5646	0.94116	TRIM26	76353	25kb
ENSG00000204599	6	30269256	30336506	240	34	120000	-1.146	0.8741	TRIM39	76353	25kb
ENSG00000248167	6	30272359	30339631	250	36	120000	-1.0334	0.8493	TRIM39-RPP21	76353	25kb
ENSG00000241370	6	30287908	30339661	184	32	120000	-0.87994	0.81055	RPP21	76353	25kb
ENSG00000204592	6	30432244	30486982	167	22	120000	-0.64656	0.74104	HLA-E	76353	25kb
ENSG00000204590	6	30484154	30549951	150	15	120000	-0.39541	0.65373	GNL1	76353	25kb
ENSG00000204576	6	30499663	30556500	114	14	120000	-0.29804	0.61716	PRR3	76353	25kb
ENSG00000204574	6	30514153	30589956	186	16	120000	-0.65879	0.74498	ABCF1	76353	25kb
ENSG00000204569	6	30543177	30611389	187	16	120000	-0.67187	0.74917	PPP1R10	76353	25kb
ENSG00000204568	6	30560486	30619172	167	15	120000	-0.75553	0.77503	MRPS18B	76353	25kb
ENSG00000137343	6	30569619	30639600	162	18	120000	-0.77887	0.78197	ATAT1	76353	25kb
ENSG00000204564	6	30589816	30645987	146	19	120000	-1.0041	0.84234	C6orf136	76353	25kb
ENSG00000204560	6	30595896	30665814	201	30	120000	-0.85438	0.80355	DHX16	76353	25kb
ENSG00000146112	6	30619166	30680672	232	25	120000	-0.40286	0.65648	PPP1R18	76353	25kb
ENSG00000137404	6	30630824	30684197	224	23	120000	-0.33568	0.63144	NRM	76353	25kb
ENSG00000137337	6	30642584	30710666	338	22	120000	0.78766	0.21545	MDC1	76353	25kb
ENSG00000196230	6	30662978	30718203	291	17	120000	0.94768	0.17165	TUBB	76353	25kb
ENSG00000137312	6	30670486	30735510	335	26	120000	0.737	0.23056	FLOT1	76353	25kb
ENSG00000137331	6	30685976	30737331	260	24	120000	0.79138	0.21436	IER3	76353	25kb
ENSG00000204580	6	30819198	30892933	295	19	120000	-0.068856	0.52745	DDR1	76353	25kb
ENSG00000213780	6	30850961	30906883	248	21	120000	0.018368	0.49267	GTF2H4	76353	25kb
ENSG00000137411	6	30851019	30919236	286	21	120000	-0.1554	0.56175	VARS2	76353	25kb
ENSG00000196260	6	30874130	30924952	215	22	120000	-0.14371	0.55714	SFTA2	76353	25kb
ENSG00000168631	6	30883749	30946998	302	34	120000	-0.55398	0.7102	DPCR1	76353	25kb
ENSG00000204544	6	30926495	30982680	374	21	120000	-0.051488	0.52053	MUC21	76353	25kb
ENSG00000261272	6	30953251	31028179	757	42	120000	0.70511	0.24037	MUC22	76353	25kb
ENSG00000204542	6	31054000	31105336	591	26	120000	0.75308	0.2257	C6orf15	76353	25kb
ENSG00000204540	6	31057527	31132869	829	39	120000	0.66517	0.25297	PSORS1C1	76353	25kb
ENSG00000204539	6	31057867	31113223	656	33	120000	0.65742	0.25546	CDSN	76353	25kb
ENSG00000204538	6	31080313	31132127	557	35	120000	0.44789	0.32712	PSORS1C2	76353	25kb
ENSG00000204536	6	31085216	31151015	673	40	120000	0.531	0.29771	CCHCR1	76353	25kb
ENSG00000137310	6	31101319	31159936	622	38	120000	0.48062	0.31539	TCF19	76353	25kb
ENSG00000204531	6	31107119	31173508	746	37	120000	0.3176	0.37539	POU5F1	76353	25kb
ENSG00000206344	6	31140537	31196745	767	32	120000	0.20254	0.41975	HCG27	76353	25kb
ENSG00000204525	6	31211526	31264907	1165	44	120000	-0.78536	0.78388	HLA-C	76353	25kb
ENSG00000234745	6	31296649	31349965	1360	53	120000	-0.62037	0.73249	HLA-B	76353	25kb
ENSG00000204520	6	31346356	31408092	1025	31	120000	-0.91694	0.82041	MICA	76353	25kb
ENSG00000204516	6	31437658	31503901	642	35	120000	-0.2042	0.5809	MICB	76353	25kb
ENSG00000204511	6	31471494	31523009	371	33	120000	-0.77865	0.78191	MCCD1	76353	25kb
ENSG00000198563	6	31472996	31535225	406	36	120000	-0.93538	0.8252	DDX39B	76353	25kb
ENSG00000254870	6	31472996	31539385	417	39	120000	-1.0046	0.84245	ATP6V1G2-DDX39B	76353	25kb
ENSG00000213760	6	31487239	31541204	305	30	120000	-1.2562	0.89548	ATP6V1G2	76353	25kb
ENSG00000204498	6	31489647	31551606	315	33	120000	-1.292	0.90183	NFKBIL1	76353	25kb
ENSG00000226979	6	31514831	31567101	238	44	120000	-1.554	0.93991	LTA	76353	25kb
ENSG00000232810	6	31518344	31571113	230	43	120000	-1.6054	0.94579	TNF	76353	25kb
ENSG00000227507	6	31523302	31575299	213	45	120000	-1.8602	0.96857	LTB	76353	25kb
ENSG00000204482	6	31528901	31581686	212	46	120000	-1.8326	0.96657	LST1	76353	25kb
ENSG00000204475	6	31531672	31585762	215	49	120000	-1.7676	0.96144	NCR3	76353	25kb
ENSG00000204472	6	31557961	31609798	209	42	120000	-1.0363	0.84998	AIF1	76353	25kb
ENSG00000204469	6	31563497	31630548	239	40	120000	-0.60238	0.72654	PRRC2A	76353	25kb
ENSG00000204463	6	31581805	31645482	220	33	120000	-0.26845	0.60582	BAG6	76353	25kb
ENSG00000204444	6	31595193	31650987	176	30	120000	-0.20328	0.58054	APOM	76353	25kb
ENSG00000204439	6	31601075	31653549	156	28	120000	-0.23153	0.59155	C6orf47	76353	25kb
ENSG00000204438	6	31604006	31659060	153	28	120000	-0.2529	0.59983	GPANK1	76353	25kb
ENSG00000204435	6	31608013	31663120	135	25	120000	-0.27494	0.60832	CSNK2B	76353	25kb
ENSG00000263020	6	31608879	31666323	142	25	120000	-0.2886	0.61356	CSNK2B-LY6G5B-1181	76353	25kb
ENSG00000240053	6	31612944	31666553	131	24	120000	-0.3125	0.62267	LY6G5B	76353	25kb
ENSG00000204428	6	31619461	31676817	138	25	120000	-0.43149	0.66694	LY6G5C	76353	25kb
ENSG00000204427	6	31629726	31696221	151	27	120000	-0.76394	0.77755	ABHD16A	76353	25kb
ENSG00000204422	6	31629739	31706849	168	29	120000	-0.93932	0.82622	XXbac-BPG32J3.20	76353	25kb
ENSG00000204424	6	31649640	31710581	122	26	120000	-1.5022	0.93347	LY6G6F	76353	25kb
ENSG00000250641	6	31649681	31710695	122	26	120000	-1.5022	0.93347	MEGT1	76353	25kb
ENSG00000255552	6	31654548	31706842	105	23	120000	-1.2472	0.89383	LY6G6E	76353	25kb
ENSG00000244355	6	31658133	31710581	113	25	120000	-1.608	0.94608	LY6G6D	76353	25kb
ENSG00000204420	6	31661371	31719491	130	28	120000	-1.6737	0.9529	C6orf25	76353	25kb

ENSG00000204421	6	31661425	31714622	122	26	120000	-1.7376	0.95886	LY6G6C	76353	25kb
ENSG00000213722	6	31669815	31723394	112	25	120000	-2.0465	0.97965	DDAH2	76353	25kb
ENSG00000213719	6	31673358	31732540	125	26	120000	-1.7903	0.9633	CLIC1	76353	25kb
ENSG00000204410	6	31682725	31757622	151	29	120000	-2.2952	0.98914	MSH5	76353	25kb
ENSG00000255152	6	31682797	31757628	151	29	120000	-2.2952	0.98914	MSH5-SAPCD1	76353	25kb
ENSG00000228727	6	31705576	31757628	110	23	120000	-2.1351	0.98362	SAPCD1	76353	25kb
ENSG00000204396	6	31708367	31770108	136	28	120000	-2.3916	0.99161	VWA7	76353	25kb
ENSG00000204394	6	31720295	31788730	153	35	120000	-2.7849	0.99732	VARS	76353	25kb
ENSG00000204392	6	31740173	31799761	133	34	120000	-3.2435	0.99941	LSM2	76353	25kb
ENSG00000204390	6	31752396	31808437	141	35	120000	-3.3527	0.9996	HSPA1L	76353	25kb
ENSG00000204389	6	31758291	31810723	137	34	120000	-3.3054	0.99953	HSPA1A	76353	25kb
ENSG00000204388	6	31770512	31823031	143	35	120000	-3.5558	0.99981	HSPA1B	76353	25kb
ENSG00000204387	6	31777385	31832541	146	36	120000	-3.7006	0.99989	C6orf48	76353	25kb
ENSG00000204386	6	31800436	31855683	174	37	120000	-3.1162	0.99908	NEU1	76353	25kb
ENSG00000204385	6	31805969	31871823	202	41	120000	-3.0881	0.99899	SLC44A4	76353	25kb
ENSG00000204371	6	31822536	31890464	196	38	120000	-2.8492	0.99781	EHMT2	76353	25kb
ENSG00000166278	6	31840562	31938449	268	35	120000	-2.5846	0.99513	C2	76353	25kb
ENSG00000204366	6	31842384	31894769	143	35	120000	-2.8296	0.99767	ZBTB12	76353	25kb
ENSG00000244255	6	31870475	31944825	188	32	120000	-2.2765	0.98859	CFB	76353	25kb
ENSG00000243649	6	31870475	31944861	189	32	120000	-2.2803	0.9887	CFB	76353	25kb
ENSG00000204356	6	31894864	31951887	140	26	120000	-2.0702	0.98078	NELFE	76353	25kb
ENSG00000204351	6	31901857	31962532	127	23	120000	-1.9337	0.97342	SKIV2L	76353	25kb
ENSG00000204348	6	31912587	31965069	101	25	120000	-1.9703	0.9756	DXO	76353	25kb
ENSG00000204344	6	31913868	31975598	107	26	120000	-1.9941	0.97693	STK19	76353	25kb
ENSG00000244731	6	31924801	31995458	81	23	120000	-1.7662	0.96132	C4A	76353	25kb
ENSG00000268923	6	31948945	31999881	11	6	120000	-1.6191	0.94729	AL645922.1	76353	25kb
ENSG00000224389	6	31957539	32028195	83	26	120000	-1.5106	0.93455	C4B	76353	25kb
ENSG00000231852	6	31981042	32034447	96	25	120000	-1.5135	0.93492	CYP21A2	76353	25kb
ENSG00000168477	6	31983931	32108111	252	33	120000	-1.5175	0.93543	TNXB	76353	25kb
ENSG00000213676	6	32040953	32121030	174	39	120000	-1.6052	0.94578	ATF6B	76353	25kb
ENSG00000204315	6	32071484	32123068	115	29	120000	-1.6046	0.94571	FKBPL	76353	25kb
ENSG00000204314	6	32091136	32147150	138	29	120000	-1.5418	0.93844	PRRT1	76353	25kb
ENSG00000221988	6	32096218	32159011	160	30	120000	-1.1261	0.86995	PPT2	76353	25kb
ENSG00000258388	6	32096622	32164755	172	32	120000	-1.2392	0.89236	PPT2-EGFL8	76353	25kb
ENSG00000241404	6	32107360	32161058	134	26	120000	-0.9602	0.83152	EGFL8	76353	25kb
ENSG00000204310	6	32110989	32170873	153	29	120000	-1.0088	0.84345	AGPAT1	76353	25kb
ENSG00000204308	6	32121131	32176930	162	30	120000	-1.0226	0.84674	RNF5	76353	25kb
ENSG00000204305	6	32123745	32177101	156	30	120000	-0.9309	0.82405	AGER	76353	25kb
ENSG00000204304	6	32127512	32182963	167	31	120000	-0.82251	0.79461	PBX2	76353	25kb
ENSG00000213654	6	32133543	32188300	186	32	120000	-0.54426	0.70687	GPSM3	76353	25kb
ENSG00000204301	6	32137620	32216844	453	50	120000	-1.1298	0.87071	NOTCH4	76353	25kb
ENSG00000204296	6	32231303	32364684	1134	32	120000	-0.64861	0.74171	C6orf10	76353	25kb
ENSG00000204290	6	32336740	32399905	715	30	120000	-1.9255	0.97292	BTNL2	76353	25kb
ENSG00000204287	6	32382619	32437823	782	24	120000	-1.7435	0.95938	HLA-DRA	76353	25kb
ENSG00000198502	6	32460120	32523064	20	2	120000	0.42149	0.3367	HLA-DRB5	76353	25kb
ENSG00000196126	6	32521546	32582625	518	21	120000	-0.59853	0.72526	HLA-DRB1	76353	25kb
ENSG00000196735	6	32570956	32639839	1422	38	120000	0.36049	0.35924	HLA-DQA1	76353	25kb
ENSG00000179344	6	32602244	32661160	957	34	120000	1.8409	0.032815	HLA-DQB1	76353	25kb
ENSG00000237541	6	32684119	32739992	873	17	120000	1.2061	0.11389	HLA-DQA2	76353	25kb
ENSG00000232629	6	32698875	32756311	808	21	120000	1.4489	0.073683	HLA-DQB2	76353	25kb
ENSG00000241106	6	32755540	32809825	529	30	120000	0.57875	0.28138	HLA-DOB	76353	25kb
ENSG00000250264	6	32756544	32831599	685	44	120000	0.40317	0.34341	TAP2	76353	25kb
ENSG00000204267	6	32764610	32831557	590	45	120000	0.32868	0.3712	TAP2	76353	25kb
ENSG00000204264	6	32783494	32837480	427	41	120000	0.13872	0.44484	PSMB8	76353	25kb
ENSG00000240065	6	32786913	32852362	493	41	120000	-0.05555	0.52215	PSMB9	76353	25kb
ENSG00000168394	6	32787986	32846755	456	40	120000	-0.024179	0.50965	TAP1	76353	25kb
ENSG00000242574	6	32877406	32933847	255	30	120000	1.253	0.1051	HLA-DMB	76353	25kb
ENSG00000248993	6	32880141	32945899	335	36	120000	1.3754	0.084505	XXbac-BPG181M17.5	76353	25kb
ENSG00000204257	6	32891390	32961871	417	43	120000	1.3414	0.089888	HLA-DMA	76353	25kb
ENSG00000204256	6	32911437	32974282	383	42	120000	0.78432	0.21643	BRD2	76353	25kb
ENSG00000204252	6	32946955	33002389	450	57	120000	-0.27837	0.60964	HLA-DOA	76353	25kb
ENSG00000231389	6	33007346	33073552	889	28	120000	-0.50024	0.69155	HLA-DPA1	76353	25kb
ENSG00000223865	6	33018703	33079978	878	19	120000	-0.70701	0.76022	HLA-DPB1	76353	25kb
ENSG00000204248	6	33105458	33185276	397	28	120000	1.7825	0.037331	COL11A2	76353	25kb
ENSG00000204231	6	33136365	33193630	151	23	120000	2.4221	0.0077148	RXRB	76353	25kb
ENSG00000112473	6	33143222	33197216	130	22	120000	2.0244	0.021467	SLC39A7	76353	25kb
ENSG00000204228	6	33147419	33199608	132	24	120000	2.087	0.018446	HSD17B8	76353	25kb
ENSG00000204227	6	33151272	33205499	140	28	120000	2.4615	0.0069186	RING1	76353	25kb
ENSG00000223501	6	33193049	33264824	187	27	120000	1.5057	0.066072	VPS52	76353	25kb
ENSG00000231500	6	33214787	33269287	144	19	120000	1.0299	0.15153	RPS18	76353	25kb
ENSG00000235863	6	33219917	33277609	145	20	120000	0.92856	0.17656	B3GALT4	76353	25kb
ENSG00000227057	6	33221885	33282304	151	21	120000	0.99963	0.15874	WDR46	76353	25kb
ENSG00000204220	6	33232079	33291178	140	21	120000	0.84313	0.19958	PFDN6	76353	25kb
ENSG00000237441	6	33234431	33292101	133	20	120000	0.79566	0.21311	RGL2	76353	25kb
ENSG00000231925	6	33242471	33307164	135	19	120000	0.57241	0.28352	TAPBP	76353	25kb
ENSG00000236104	6	33257183	33310719	104	15	120000	0.34616	0.36461	ZBTB22	76353	25kb
ENSG00000204209	6	33261335	33322046	138	21	120000	1.1615	0.12272	DAXX	76353	25kb
ENSG00000237649	6	33334313	33402701	136	23	120000	2.2101	0.013548	KIFC1	76353	25kb
ENSG00000112511	6	33353176	33409230	110	22	120000	1.9628	0.024833	PHF1	76353	25kb
ENSG00000112514	6	33359219	33411094	101	21	120000	1.8904	0.029354	CUTA	76353	25kb
ENSG00000197283	6	33362847	33446466	170	25	120000	1.4659	0.071337	SYNGAP1	76353	25kb
ENSG00000213588	6	33397356	33450325	111	17	120000	1.1761	0.11978	ZBTB9	76353	25kb
ENSG00000030110	6	33515329	33573019	301	24	120000	-1.1222	0.86911	BAK1	76353	25kb
ENSG00000204188	6	33526515	33581803	234	34	120000	-1.7004	0.95548	GGNBP1	76353	25kb
ENSG00000096433	6	33563142	33689351	544	47	120000	-1.4925	0.93222	ITPR3	76353	25kb
ENSG00000137288	6	33637070	33704504	266	20	120000	-0.68994	0.75488	UQCC2	76353	25kb
ENSG00000269490	6	33638196	33688474	209	23	120000	-0.67324	0.7496	SBP1	76353	25kb
ENSG00000161896	6	33664444	33739762	319	24	120000	-1.2345	0.8915	IP6K3	76353	25kb
ENSG00000161904	6	33713979	33781913	287	21	120000	-2.0078	0.97767	LEMD2	76353	25kb
ENSG00000096395	6	33737450	33796788	254	22	120000	-1.4176	0.92184	MLN	76353	25kb
ENSG00000124493	6	33964628	34148399	732	62	120000	1.1921	0.11661	GRM4	76353	25kb
ENSG00000137309	6	34179650	34239008	186	19	120000	0.59688	0.27529	HMGA1	76353	25kb
ENSG00000186577	6	34189157	34242247	160	17	120000	0.56024	0.28766	C6orf1	76353	25kb
ENSG00000272325	6	34222456	34385451	390	19	120000	1.0338	0.15062	NUDT3	76353	25kb
ENSG00000270800	6	34231547	34418825	448	24	120000	0.87339	0.19122	RPS10-NUDT3	76353	25kb
ENSG00000124614	6	34360231	34418902	135	20	120000	0.35216	0.36236	RPS10	76353	25kb

ENSG00000124507	6	34408916	34528006	305	42	120000	0.31783	0.37531	PACSIN1	76353	25kb
ENSG00000124664	6	34480579	34549110	203	32	120000	1.3977	0.081097	SPDEF	76353	25kb
ENSG00000196821	6	34530065	34689636	362	28	120000	0.3989	0.34498	C6orf106	76353	25kb
ENSG00000124562	6	34700183	34766571	221	12	120000	0.18678	0.42592	SNRPC	76353	25kb
ENSG00000065060	6	34734857	34875915	391	24	120000	0.012203	0.49513	UHRF1BP1	76353	25kb
ENSG00000064995	6	34820555	34880866	154	20	120000	0.1482	0.44109	TAF11	76353	25kb
ENSG00000064999	6	34832042	35084179	614	40	120000	-0.4793	0.68414	ANKS1A	76353	25kb
ENSG00000124678	6	35060848	35141387	270	18	120000	-0.45283	0.67466	TCP11	76353	25kb
ENSG00000146197	6	35157190	35245856	180	18	120000	-0.26717	0.60533	SCUBE3	76353	25kb
ENSG00000065029	6	35201686	35288762	180	18	120000	-0.43658	0.66879	ZNF76	76353	25kb
ENSG00000023892	6	35240595	35314548	163	19	120000	-0.8364	0.79853	DEF6	76353	25kb
ENSG00000112033	6	35285335	35420968	349	31	120000	-1.6822	0.95374	PPARD	76353	25kb
ENSG00000112039	6	35395138	35459880	199	32	120000	-1.4145	0.9214	FANCE	76353	25kb
ENSG00000198755	6	35411185	35463562	147	26	120000	-1.2063	0.88615	RPL10A	76353	25kb
ENSG00000007866	6	35416374	35489853	204	36	120000	-1.0718	0.8581	TEAD3	76353	25kb
ENSG00000112041	6	35440651	35505715	190	31	120000	-0.66655	0.74747	TULP1	76353	25kb
ENSG00000096060	6	35516362	35721360	453	33	120000	-0.57301	0.71668	FKBP5	76353	25kb
ENSG00000157343	6	35679809	35741856	164	26	120000	1.6033	0.054438	ARMC12	76353	25kb
ENSG00000196748	6	35719371	35772329	172	24	120000	2.0829	0.018631	CLPSL2	76353	25kb
ENSG00000204140	6	35723794	35786452	196	27	120000	1.8584	0.031554	CLPSL1	76353	25kb
ENSG00000137392	6	35737759	35790088	141	22	120000	1.3649	0.086137	CLPS	76353	25kb
ENSG00000197753	6	35748070	35826651	208	17	120000	0.28718	0.38699	LHFPL5	76353	25kb
ENSG00000096063	6	35775743	35914119	292	12	120000	-0.34312	0.63425	SRPK1	76353	25kb
ENSG00000112053	6	35886291	36017645	320	32	120000	0.18041	0.42842	SLC26A8	76353	25kb
ENSG00000112062	6	35970488	36104013	325	24	120000	0.066274	0.47358	MAPK14	76353	25kb
ENSG00000156711	6	36070586	36132842	105	22	120000	-1.0097	0.84369	MAPK13	76353	25kb
ENSG00000096070	6	36139521	36225567	123	25	120000	-2.2457	0.98764	BRPF3	76353	25kb
ENSG00000180316	6	36185980	36301372	280	52	120000	0.44154	0.32941	PNPLA1	76353	25kb
ENSG00000189325	6	36258534	36329662	200	50	120000	0.055705	0.47779	C6orf222	76353	25kb
ENSG00000010030	6	36297419	36381164	271	41	120000	-0.62951	0.73549	ETV7	76353	25kb
ENSG00000179165	6	36333328	36435666	324	23	120000	-0.58127	0.71947	PXT1	76353	25kb
ENSG00000112078	6	36385544	36483920	274	14	120000	-0.40797	0.65835	KCTD20	76353	25kb
ENSG00000112079	6	36436669	36540247	280	17	120000	-0.399	0.65505	STK38	76353	25kb
ENSG00000112081	6	36537145	36598377	206	15	120000	0.86707	0.19295	SRSF3	76353	25kb
ENSG00000124762	6	36619305	36680116	257	27	120000	1.1443	0.12625	CDKN1A	76353	25kb
ENSG00000255587	6	36658256	36724870	281	29	120000	0.48171	0.31501	RAB44	76353	25kb
ENSG00000124772	6	36683552	36832778	541	62	120000	-0.59861	0.72528	CPNE5	76353	25kb
ENSG00000137168	6	36797603	36867800	231	29	120000	-1.3524	0.91188	PPIL1	76353	25kb
ENSG00000198663	6	36814646	36921740	343	23	120000	-1.61	0.9463	C6orf89	76353	25kb
ENSG00000164530	6	36897209	36957613	246	33	120000	-0.1499	0.55958	PI16	76353	25kb
ENSG00000137409	6	36910917	36979074	286	26	120000	0.83998	0.20046	MTCH1	76353	25kb
ENSG00000146192	6	36948422	37021846	272	31	120000	0.74075	0.22942	FGD2	76353	25kb
ENSG00000226976	6	36987607	37038177	169	18	120000	-0.82153	0.79433	COX6A1P2	76353	25kb
ENSG00000137193	6	37112979	37168202	137	18	120000	-0.27595	0.60871	PIM1	76353	25kb
ENSG00000172738	6	37154956	37250931	212	26	120000	0.28557	0.3876	TMEM217	76353	25kb
ENSG00000065491	6	37200548	37325746	219	19	120000	-0.04929	0.51966	TBC1D22B	76353	25kb
ENSG00000112130	6	37296748	37387514	169	34	120000	-2.2926	0.98906	RNF8	76353	25kb
ENSG00000137200	6	37375995	37475603	188	23	120000	-1.461	0.928	CMTR1	76353	25kb
ENSG00000198937	6	37425696	37492698	187	20	120000	-0.83412	0.79789	CCDC167	76353	25kb
ENSG00000112139	6	37575284	37692082	497	65	120000	-1.0689	0.85745	MDGA1	76353	25kb
ENSG00000156639	6	37762275	38147400	1074	25	120000	0.83971	0.20054	ZFAND3	76353	25kb
ENSG00000183826	6	38111227	38632924	1267	76	120000	-0.8462	0.80128	BTBD9	76353	25kb
ENSG00000124767	6	38618701	38695917	247	37	120000	-0.26337	0.60387	GLO1	76353	25kb
ENSG00000124721	6	38658117	39023301	1266	63	120000	0.32257	0.37351	DNAH8	76353	25kb
ENSG00000112164	6	38991574	39080519	285	34	120000	-1.0538	0.85402	GLP1R	76353	25kb
ENSG00000112167	6	39046840	39107965	222	32	120000	-0.10587	0.54216	SAYSD1	76353	25kb
ENSG00000164626	6	39131749	39222226	328	50	120000	-0.65733	0.74452	KCNK5	76353	25kb
ENSG00000124780	6	39241777	39307329	337	36	120000	-0.097961	0.53902	KCNK17	76353	25kb
ENSG00000095981	6	39257474	39315744	258	35	120000	0.25315	0.40007	KCNK16	76353	25kb
ENSG00000164627	6	39272766	39718181	1301	71	120000	0.16036	0.4363	KIF6	76353	25kb
ENSG00000146122	6	39735142	39897648	532	50	120000	0.51105	0.30466	DAAM2	76353	25kb
ENSG00000124615	6	39842354	39927290	279	28	120000	-0.41948	0.66257	MOCS1	76353	25kb
ENSG00000156564	6	40334325	40580204	948	66	120000	-0.99883	0.84106	LRFN2	76353	25kb
ENSG00000124602	6	40969772	41031928	150	15	120000	0.72757	0.23344	UNC5CL	76353	25kb
ENSG00000124596	6	40976366	41090526	242	21	120000	1.2314	0.1091	OARD1	76353	25kb
ENSG00000112212	6	40985293	41037076	121	11	120000	0.87527	0.19071	TSPO2	76353	25kb
ENSG00000124701	6	40996043	41057250	133	12	120000	0.56555	0.28585	APOBEC2	76353	25kb
ENSG00000001167	6	41015684	41092715	159	13	120000	1.082	0.13962	NFYA	76353	25kb
ENSG00000161911	6	41092080	41147075	61	8	120000	1.7842	0.037192	TREML1	76353	25kb
ENSG00000095970	6	41101244	41155924	65	11	120000	1.5131	0.065126	TREM2	76353	25kb
ENSG00000112195	6	41133015	41193932	186	29	120000	0.4409	0.32964	TREML2	76353	25kb
ENSG00000188056	6	41171062	41231620	268	18	120000	0.79388	0.21363	TREML4	76353	25kb
ENSG00000124731	6	41210664	41279457	257	28	120000	0.10618	0.45772	TREM1	76353	25kb
ENSG00000096264	6	41278393	41343625	318	36	120000	0.24495	0.40325	NCR2	76353	25kb
ENSG00000137166	6	41489164	41595122	312	37	120000	-0.81483	0.79242	FOXP4	76353	25kb
ENSG00000112559	6	41579620	41646984	237	58	120000	2.7369	0.0031008	MDFI	76353	25kb
ENSG00000112561	6	41626716	41728997	336	51	120000	0.9726	0.16538	TFEB	76353	25kb
ENSG00000268275	6	41628964	41679127	186	34	120000	1.0612	0.14429	AL035588.1	76353	25kb
ENSG00000096088	6	41679449	41746847	177	38	120000	0.56027	0.28765	PGC	76353	25kb
ENSG00000137218	6	41712914	41779280	168	30	120000	0.7857	0.21602	FRS3	76353	25kb
ENSG00000124593	6	41723087	41782879	138	24	120000	0.64023	0.26101	PRICKLE4	76353	25kb
ENSG00000214736	6	41730400	41782636	113	20	120000	0.52268	0.3006	TOMM6	76353	25kb
ENSG00000164663	6	41732634	41888099	287	22	120000	0.35639	0.36077	USP49	76353	25kb
ENSG00000124641	6	41848092	41913877	108	20	120000	0.6951	0.2435	MED20	76353	25kb
ENSG00000112578	6	41863926	41925784	117	21	120000	0.98404	0.16255	BYSL	76353	25kb
ENSG00000112576	6	41877671	42043095	512	61	120000	1.5972	0.055113	CCND3	76353	25kb
ENSG00000137413	6	41993251	42080199	331	58	120000	1.0281	0.15195	TAF8	76353	25kb
ENSG00000188112	6	42043856	42135357	392	65	120000	-0.94229	0.82698	C6orf132	76353	25kb
ENSG00000048545	6	42098144	42172794	309	38	120000	-1.5208	0.93584	GUCA1A	76353	25kb
ENSG00000214732	6	42098279	42156156	231	42	120000	-1.3542	0.91216	RP1-139D8.6	76353	25kb
ENSG00000112599	6	42127139	42187654	264	18	120000	-1.997	0.97709	GUCA1B	76353	25kb
ENSG00000048544	6	42149539	42210603	224	14	120000	-1.571	0.9419	MRPS10	76353	25kb
ENSG00000124496	6	42167669	42444789	691	62	120000	-0.3736	0.64565	TRERF1	76353	25kb
ENSG00000024048	6	42506800	42686242	365	21	120000	-1.785	0.96287	UBR2	76353	25kb
ENSG00000112619	6	42639340	42715312	185	23	120000	-1.744	0.95942	PRPH2	76353	25kb
ENSG00000124659	6	42687219	42739558	94	18	120000	-0.45495	0.67543	TBCC	76353	25kb

ENSG00000112624	6	42724765	42861298	257	30	120000	0.30945	0.37849	GLTSCR1L	76353	25kb
ENSG00000146223	6	42822356	42882663	164	16	120000	0.15144	0.43981	RPL7L1	76353	25kb
ENSG00000221821	6	42833005	42883554	141	14	120000	0.15682	0.43769	C6orf226	76353	25kb
ENSG00000171611	6	42858727	42918573	153	15	120000	-0.35371	0.63822	PTCRA	76353	25kb
ENSG00000137161	6	42871938	42932025	162	20	120000	-0.13298	0.55289	CNPY3	76353	25kb
ENSG00000124713	6	42903496	42956618	176	19	120000	0.51455	0.30343	GNMT	76353	25kb
ENSG00000124587	6	42906608	42971958	215	25	120000	0.57317	0.28327	PEX6	76353	25kb
ENSG00000112640	6	42927237	43005080	212	25	120000	0.47253	0.31828	PPP2R5D	76353	25kb
ENSG00000124733	6	42954832	43006706	129	17	120000	-0.053671	0.5214	MEA1	76353	25kb
ENSG00000124702	6	42956951	43014036	131	16	120000	-0.033936	0.51354	KLHDC3	76353	25kb
ENSG00000124541	6	42964383	43026894	143	19	120000	-0.30218	0.61874	RRP36	76353	25kb
ENSG00000044090	6	42980355	43046683	146	18	120000	-0.16015	0.56362	CUL7	76353	25kb
ENSG00000137171	6	42983515	43067837	176	21	120000	-0.28858	0.61355	KLC4	76353	25kb
ENSG00000112651	6	42996767	43052544	124	16	120000	-0.066194	0.52639	MRPL2	76353	25kb
ENSG00000112655	6	43019006	43154457	304	28	120000	0.20379	0.41926	PTK7	76353	25kb
ENSG00000112658	6	43114037	43174243	128	16	120000	0.1733	0.43121	SRF	76353	25kb
ENSG00000112659	6	43124913	43217325	205	25	120000	-0.32119	0.62597	CUL9	76353	25kb
ENSG00000112667	6	43168367	43222222	120	21	120000	-0.8856	0.81208	DNPH1	76353	25kb
ENSG00000146216	6	43186418	43280997	216	35	120000	-0.68342	0.75283	TTBK1	76353	25kb
ENSG00000137204	6	43238432	43298276	113	19	120000	-0.019147	0.50764	SLC22A7	76353	25kb
ENSG00000146215	6	43242448	43301535	104	18	120000	0.077675	0.46904	CRIP3	76353	25kb
ENSG00000171467	6	43249872	43362216	195	25	120000	0.39722	0.3456	ZNF318	76353	25kb
ENSG00000124574	6	43370104	43443168	159	27	120000	1.4525	0.073176	ABCC10	76353	25kb
ENSG00000171462	6	43393090	43449370	117	27	120000	0.59585	0.27564	DLK2	76353	25kb
ENSG00000137221	6	43420261	43499294	151	17	120000	-0.2749	0.6083	TJAP1	76353	25kb
ENSG00000204052	6	43449707	43503424	96	10	120000	-0.39676	0.65423	LRRC73	76353	25kb
ENSG00000171453	6	43452440	43522323	115	12	120000	-0.47217	0.6816	POLR1C	76353	25kb
ENSG00000137207	6	43454565	43509728	92	11	120000	-0.50245	0.69232	YIPF3	76353	25kb
ENSG00000124571	6	43465072	43568812	151	16	120000	-0.46244	0.67812	XPO5	76353	25kb
ENSG00000170734	6	43518887	43611701	134	17	120000	-0.34417	0.63464	POLH	76353	25kb
ENSG00000172432	6	43548053	43621899	106	14	120000	-0.33768	0.6322	GTPBP2	76353	25kb
ENSG00000124688	6	43572277	43633689	129	15	120000	0.22977	0.40914	MAD2L1BP	76353	25kb
ENSG00000172426	6	43587783	43665336	190	19	120000	0.76698	0.22155	RSPH9	76353	25kb
ENSG00000096080	6	43614040	43680528	183	18	120000	0.90151	0.18366	MRPS18A	76353	25kb
ENSG00000112715	6	43712921	43779224	183	46	120000	-1.0298	0.84845	VEGFA	76353	25kb
ENSG00000181577	6	43943317	43998695	250	38	120000	-1.2394	0.89239	C6orf223	76353	25kb
ENSG00000180992	6	44056194	44120194	243	27	120000	0.96332	0.16769	MRPL14	76353	25kb
ENSG00000137216	6	44069651	44148256	305	33	120000	1.0358	0.15016	TMEM63B	76353	25kb
ENSG00000137225	6	44101548	44177139	266	50	120000	0.93205	0.17566	CAPN11	76353	25kb
ENSG00000112759	6	44162242	44226888	179	37	120000	0.39333	0.34704	SLC29A1	76353	25kb
ENSG00000096384	6	44189824	44246620	156	34	120000	0.22471	0.4111	HSP90AB1	76353	25kb
ENSG00000157593	6	44196833	44250291	165	36	120000	0.21484	0.41494	SLC35B2	76353	25kb
ENSG00000146232	6	44200903	44258500	190	35	120000	0.47705	0.31666	NFKBIE	76353	25kb
ENSG00000178233	6	44213203	44300243	264	28	120000	0.57174	0.28375	TMEM151B	76353	25kb
ENSG00000272442	6	44215931	44371694	418	50	120000	-0.14727	0.55854	RP11-444E17.6	76353	25kb
ENSG00000146221	6	44221480	44290458	209	25	120000	0.61619	0.26888	TCTE1	76353	25kb
ENSG00000124608	6	44242391	44306063	211	29	120000	0.52079	0.30126	AARS2	76353	25kb
ENSG00000249481	6	44285397	44369904	204	31	120000	-1.0615	0.85577	SPATS1	76353	25kb
ENSG00000096401	6	44330262	44443163	237	38	120000	-0.88236	0.81121	CDC5L	76353	25kb
ENSG00000267849	6	44375472	44425720	92	17	120000	-1.3762	0.91562	AL133262.1	76353	25kb
ENSG00000196284	6	44752054	45370690	1863	31	120000	-0.28619	0.61264	SUPT3H	76353	25kb
ENSG00000124813	6	45270894	45657086	1060	70	120000	-1.7799	0.96245	RUNX2	76353	25kb
ENSG00000112782	6	45843045	46073132	666	70	120000	0.59345	0.27644	CLIC5	76353	25kb
ENSG00000001561	6	46072730	46139436	186	13	120000	0.69597	0.24322	ENPP4	76353	25kb
ENSG00000112796	6	46101924	46163708	166	17	120000	0.8326	0.20254	ENPP5	76353	25kb
ENSG00000172348	6	46163475	46484709	664	46	120000	0.26781	0.39442	RCAN2	76353	25kb
ENSG00000146233	6	46492541	46645523	358	27	120000	1.4852	0.068751	CYP39A1	76353	25kb
ENSG00000153291	6	46595678	46670930	94	19	120000	-0.034382	0.51371	SLC25A27	76353	25kb
ENSG00000180113	6	46630612	46697056	75	15	120000	-0.22655	0.58961	TDRD6	76353	25kb
ENSG00000146070	6	46646938	46728430	136	22	120000	-0.080493	0.53208	PLA2G7	76353	25kb
ENSG00000230062	6	46689654	46752243	150	24	120000	0.15949	0.43664	ANKRD66	76353	25kb
ENSG00000112818	6	46736127	46832515	273	22	120000	1.7259	0.042179	MEP1A	76353	25kb
ENSG00000069122	6	46795249	46947680	564	42	120000	0.84651	0.19863	GPR116	76353	25kb
ENSG00000153292	6	46940440	47035099	296	33	120000	0.89116	0.18642	GPR110	76353	25kb
ENSG00000146072	6	47174268	47302641	341	51	120000	-0.0088829	0.50354	TNFRSF21	76353	25kb
ENSG00000198087	6	47420525	47619999	514	19	120000	-1.581	0.94306	CD2AP	76353	25kb
ENSG00000164393	6	47599172	47690533	395	11	120000	-0.34184	0.63377	GPR111	76353	25kb
ENSG00000153294	6	47628600	47714757	367	18	120000	-0.09082	0.53618	GPR115	76353	25kb
ENSG00000124818	6	47724718	47825516	326	26	120000	1.2517	0.10534	OPN5	76353	25kb
ENSG00000244694	6	47820764	48061425	766	45	120000	0.35999	0.35943	PTCHD4	76353	25kb
ENSG00000146085	6	49373073	49455904	170	13	120000	-0.16817	0.56678	MUT	76353	25kb
ENSG00000031691	6	49406091	49485820	192	20	120000	0.073969	0.47052	CENPQ	76353	25kb
ENSG00000203972	6	49442671	49519760	178	24	120000	0.43834	0.33057	GLYATL3	76353	25kb
ENSG00000197261	6	49493113	49554620	178	25	120000	0.14408	0.44272	C6orf141	76353	25kb
ENSG00000112077	6	49547871	49629552	261	21	120000	-0.84245	0.80023	RHAG	76353	25kb
ENSG00000124490	6	49635073	49706274	233	25	120000	-0.91353	0.81952	CRISP2	76353	25kb
ENSG00000096006	6	49670097	49737150	187	20	120000	-1.0578	0.85493	CRISP3	76353	25kb
ENSG00000170950	6	49728366	49780053	115	18	120000	-0.82218	0.79451	PGK2	76353	25kb
ENSG00000124812	6	49776970	49869809	245	19	120000	0.13894	0.44475	CRISP1	76353	25kb
ENSG00000214643	6	49888814	49942157	159	15	120000	-1.0826	0.8605	DEFB133	76353	25kb
ENSG00000177684	6	49902962	49956818	159	16	120000	-1.2794	0.89963	DEFB114	76353	25kb
ENSG00000214642	6	49911390	49962338	142	16	120000	-1.4291	0.92352	DEFB113	76353	25kb
ENSG00000203970	6	49951851	50014694	123	18	120000	-0.51072	0.69522	DEFB110	76353	25kb
ENSG00000180872	6	49986288	50041364	120	15	120000	-0.37257	0.64526	DEFB112	76353	25kb
ENSG00000008197	6	50656541	50765701	183	19	120000	0.28812	0.38663	TFAP2D	76353	25kb
ENSG00000008196	6	50761436	50840326	190	24	120000	0.50857	0.30553	TFAP2B	76353	25kb
ENSG00000170927	6	51455098	51977423	1675	70	120000	-1.6791	0.95343	PKHD1	76353	25kb
ENSG00000112115	6	52026185	52080436	228	23	120000	-1.228	0.89029	IL17A	76353	25kb
ENSG00000112116	6	52076479	52134335	179	31	120000	0.034949	0.48606	IL17F	76353	25kb
ENSG00000112118	6	52103807	52174679	232	34	120000	0.59396	0.27627	MCM3	76353	25kb
ENSG00000170915	6	52201219	52297575	348	49	120000	-1.6106	0.94636	PAQR8	76353	25kb
ENSG00000096093	6	52260106	52412892	517	38	120000	-1.2739	0.89865	EFHC1	76353	25kb
ENSG00000065308	6	52337200	52466713	465	47	120000	-0.17578	0.56977	TRAM2	76353	25kb
ENSG00000096092	6	52510907	52576386	230	28	120000	0.51723	0.3025	TMEM14A	76353	25kb
ENSG00000244067	6	52589897	52653367	195	22	120000	-0.79213	0.78586	GSTA2	76353	25kb
ENSG00000243955	6	52631462	52693708	153	14	120000	-0.21295	0.58432	GSTA1	76353	25kb

ENSG00000182793	6	52671453	52735893	167	16	120000	-0.55022	0.70891	GSTA5	76353	25kb
ENSG00000174156	6	52736437	52799483	204	22	120000	1.2735	0.10143	GSTA3	76353	25kb
ENSG00000170899	6	52817751	52885176	242	33	120000	1.2473	0.10614	GSTA4	76353	25kb
ENSG00000112144	6	52841077	52951600	236	32	120000	0.39046	0.3481	ICK	76353	25kb
ENSG00000112146	6	52891789	52990671	180	28	120000	-1.1886	0.8827	FBXO9	76353	25kb
ENSG00000137270	6	52966762	53038627	261	26	120000	-1.4758	0.93	GCM1	76353	25kb
ENSG00000012660	6	53107196	53238947	399	22	120000	-0.41107	0.65949	ELOVL5	76353	25kb
ENSG00000001084	6	53337139	53506768	566	64	120000	1.3394	0.090216	GCLC	76353	25kb
ENSG00000124743	6	53487699	53555506	298	22	120000	-0.46689	0.67971	KLHL31	76353	25kb
ENSG00000137269	6	53634295	53813919	458	25	120000	-0.87454	0.80909	LRRC1	76353	25kb
ENSG00000146147	6	53769780	54156078	938	59	120000	-1.0251	0.84735	MLIP	76353	25kb
ENSG00000137251	6	54147657	54279950	368	32	120000	-0.26314	0.60378	TINAG	76353	25kb
ENSG00000168143	6	54686569	54831820	514	27	120000	-1.79	0.96328	FAM83B	76353	25kb
ENSG00000137252	6	55014050	55172418	516	30	120000	-0.52971	0.70184	HCRTR2	76353	25kb
ENSG00000187871	6	55167267	55292291	727	31	120000	0.46338	0.32155	GFRAL	76353	25kb
ENSG00000146151	6	55274167	55469012	838	37	120000	1.2222	0.11081	HMGCLL1	76353	25kb
ENSG00000112175	6	55593443	55765362	455	46	120000	0.98261	0.1629	BMP5	76353	25kb
ENSG00000124749	6	55896388	56283892	1526	60	120000	-0.60518	0.72747	COL21A1	76353	25kb
ENSG00000151914	6	56297785	56844426	1056	33	120000	0.010659	0.49575	DST	76353	25kb
ENSG00000151917	6	56794773	56917140	254	16	120000	-0.46751	0.67993	BEND6	76353	25kb
ENSG00000168116	6	56886347	56945023	85	13	120000	0.2863	0.38733	KIAA1586	76353	25kb
ENSG00000112200	6	56926642	57060105	151	12	120000	0.43063	0.33337	ZNF451	76353	25kb
ENSG00000112208	6	57012124	57079631	65	10	120000	0.18644	0.42605	BAG2	76353	25kb
ENSG00000112210	6	57028607	57112078	118	22	120000	0.25994	0.39745	RAB23	76353	25kb
ENSG00000146143	6	57154603	57538375	318	39	120000	-0.26963	0.60628	PRIM2	76353	25kb
ENSG00000255633	6	62259008	62309534	333	16	120000	0.77067	0.22045	MTRNR2L9	76353	25kb
ENSG00000112232	6	62364865	63021132	1545	36	120000	0.87897	0.18971	KHDRBS2	76353	25kb
ENSG00000198225	6	63896351	63947929	200	10	120000	-0.74512	0.7719	FKBP1C	76353	25kb
ENSG00000146166	6	63960856	64054882	139	17	120000	-0.20009	0.57929	LGSN	76353	25kb
ENSG00000112245	6	64206666	64318492	235	17	120000	-0.82323	0.79481	PTP4A1	76353	25kb
ENSG00000118482	6	64320725	64514229	301	19	120000	-0.18343	0.57277	PHF3	76353	25kb
ENSG00000188107	6	64404876	66442118	5951	135	120000	-0.84203	0.80012	EYS	76353	25kb
ENSG00000135298	6	69320259	70124403	2195	79	120000	-0.076225	0.53038	BAI3	76353	25kb
ENSG00000168216	6	70360694	70532003	552	19	120000	0.38773	0.34911	LMBRD1	76353	25kb
ENSG00000082293	6	70551463	70944679	1009	68	120000	0.048452	0.48068	COL19A1	76353	25kb
ENSG00000112280	6	70899764	71037786	557	48	120000	-1.1868	0.88234	COL9A1	76353	25kb
ENSG00000082269	6	71097644	71295877	411	11	120000	-1.1272	0.87016	FAM135A	76353	25kb
ENSG00000154079	6	71251620	71324272	205	14	120000	-1.4153	0.92151	C6orf57	76353	25kb
ENSG00000112305	6	71352479	71596718	511	26	120000	1.1003	0.1356	SMAP1	76353	25kb
ENSG00000112309	6	71541382	71691741	382	48	120000	0.8115	0.20854	B3GAT2	76353	25kb
ENSG00000119900	6	71973506	72043653	309	17	120000	0.29549	0.38381	OGFRL1	76353	25kb
ENSG00000079841	6	72571406	73137845	1421	52	120000	0.72897	0.23301	RIMS1	76353	25kb
ENSG00000185760	6	73306520	73933574	1689	120	120000	-0.97109	0.83425	KCNQ5	76353	25kb
ENSG00000243501	6	73894469	73997919	386	30	120000	-0.24512	0.59682	RP11-257K9.8	76353	25kb
ENSG00000256980	6	73908267	73960493	189	18	120000	-0.24823	0.59802	KHDC1L	76353	25kb
ENSG00000135314	6	73926037	74045088	365	28	120000	0.53028	0.29796	KHDC1	76353	25kb
ENSG00000203909	6	74037785	74089018	143	25	120000	1.4459	0.074099	DPPA5	76353	25kb
ENSG00000203908	6	74047400	74098894	133	24	120000	1.3159	0.094099	KHDC3L	76353	25kb
ENSG00000203907	6	74053278	74129856	166	25	120000	0.77341	0.21964	OOEP	76353	25kb
ENSG00000080007	6	74079471	74152292	136	27	120000	0.44504	0.32815	DDX43	76353	25kb
ENSG00000164430	6	74098238	74186999	188	36	120000	0.52398	0.30015	MB21D1	76353	25kb
ENSG00000135297	6	74146301	74243959	225	24	120000	0.41238	0.34003	MTO1	76353	25kb
ENSG00000156508	6	74200473	74258520	138	15	120000	0.092129	0.4633	EEF1A1	76353	25kb
ENSG00000119899	6	74278102	74388878	483	18	120000	-0.40611	0.65767	SLC17A5	76353	25kb
ENSG00000156535	6	74380508	74563040	499	43	120000	-0.90152	0.81634	CD109	76353	25kb
ENSG00000111799	6	75769042	75940767	256	38	120000	0.86158	0.19446	COL12A1	76353	25kb
ENSG00000112695	6	75922391	75985039	109	9	120000	0.92576	0.17729	COX7A2	76353	25kb
ENSG00000112697	6	75937640	76019684	138	16	120000	0.67707	0.24918	TMEM30A	76353	25kb
ENSG00000118407	6	75976575	76228454	376	26	120000	-0.45931	0.677	FILIP1	76353	25kb
ENSG00000112701	6	76286225	76452997	391	23	120000	0.42371	0.33589	SENP6	76353	25kb
ENSG00000196586	6	76433909	76654254	418	31	120000	0.099189	0.46049	MYO6	76353	25kb
ENSG00000112706	6	76605832	76807395	427	57	120000	-0.66549	0.74713	IMPG1	76353	25kb
ENSG00000135312	6	78146948	78198490	105	19	120000	1.6087	0.053839	HTR1B	76353	25kb
ENSG00000269964	6	78375375	78661691	769	24	120000	0.408	0.34164	MEI4	76353	25kb
ENSG00000146243	6	79552189	79681157	239	21	120000	1.3407	0.090015	IRAK1BP1	76353	25kb
ENSG00000146247	6	79620584	79812953	356	22	120000	1.0971	0.13631	PHIP	76353	25kb
ENSG00000118418	6	79885962	79969406	188	18	120000	0.708	0.23947	HMGN3	76353	25kb
ENSG00000135338	6	80169708	80272175	273	21	120000	-0.86264	0.80583	LCA5	76353	25kb
ENSG00000198478	6	80316000	80438372	384	44	120000	-0.3976	0.65454	SH3BGRL2	76353	25kb
ENSG00000118402	6	80599529	80682297	286	22	120000	0.96278	0.16783	ELOVL4	76353	25kb
ENSG00000112742	6	80688604	80777244	303	14	120000	1.0214	0.15354	TTK	76353	25kb
ENSG00000083123	6	80791364	81080987	814	23	120000	0.61553	0.2691	BCKDHB	76353	25kb
ENSG00000112773	6	82176156	82487491	813	46	120000	-0.27326	0.60767	FAM46A	76353	25kb
ENSG00000269494	6	82237307	82287537	118	11	120000	-0.61158	0.72959	AL359693.1	76353	25kb
ENSG00000005700	6	82854700	82982471	319	19	120000	-1.034	0.84943	IBTK	76353	25kb
ENSG00000146242	6	83047923	83105545	120	14	120000	-1.2422	0.89292	TPBG	76353	25kb
ENSG00000118420	6	83577117	83800560	553	24	120000	0.3182	0.37517	UBE3D	76353	25kb
ENSG00000083097	6	83752385	83906069	227	19	120000	0.9043	0.18292	DOPEY1	76353	25kb
ENSG00000214479	6	83757692	83807854	79	10	120000	0.9077	0.18202	AL139333.1	76353	25kb
ENSG00000013375	6	83845869	83928655	141	23	120000	0.70185	0.24139	PGM3	76353	25kb
ENSG00000013392	6	83878098	83933651	109	20	120000	0.1967	0.42203	RWDD2A	76353	25kb
ENSG00000065833	6	83895108	84165797	598	22	120000	0.43147	0.33306	ME1	76353	25kb
ENSG00000146250	6	84197194	84260423	182	24	120000	0.68818	0.24567	PRSS35	76353	25kb
ENSG00000065609	6	84237599	84444410	389	31	120000	0.066353	0.47355	SNAP91	76353	25kb
ENSG00000203877	6	84537985	84592234	112	7	120000	0.36441	0.35778	RIPPLY2	76353	25kb
ENSG00000065615	6	84544362	84702143	299	14	120000	0.24175	0.40449	CYB5R4	76353	25kb
ENSG00000135324	6	84718475	84825600	253	23	120000	-1.6447	0.94998	MRAP2	76353	25kb
ENSG00000135315	6	84808960	84962353	268	20	120000	-1.1181	0.86823	KIAA1009	76353	25kb
ENSG00000112837	6	85372069	85499237	322	25	120000	-0.59219	0.72314	TBX18	76353	25kb
ENSG00000135318	6	86134809	86230500	197	17	120000	-0.41283	0.66013	NT5E	76353	25kb
ENSG00000135317	6	86190214	86328874	206	25	120000	-1.5172	0.93539	SNX14	76353	25kb
ENSG00000271793	6	86242696	86349952	166	24	120000	-1.4777	0.93026	RP11-321N4.5	76353	25kb
ENSG00000135316	6	86293053	86378510	128	19	120000	-1.4074	0.92034	SYNCRIP	76353	25kb
ENSG00000168830	6	87622024	87751349	337	27	120000	0.68193	0.24764	HTR1E	76353	25kb
ENSG00000135346	6	87770216	87829824	161	18	120000	0.14972	0.44049	CGA	76353	25kb
ENSG00000188994	6	87837551	87998914	423	16	120000	-0.91453	0.81978	ZNF292	76353	25kb

ENSG00000164411	6	87967696	88063996	268	12	120000	-1.1433	0.87354	GJB7	76353	25kb
ENSG00000111850	6	88007301	88077043	199	14	120000	-1.2764	0.89909	SMIM8	76353	25kb
ENSG00000226524	6	88007323	88134459	405	21	120000	-1.3644	0.91378	RP1-102H19.8	76353	25kb
ENSG00000203872	6	88029567	88100181	207	17	120000	-1.4709	0.92934	C6orf163	76353	25kb
ENSG00000203871	6	88081840	88134467	193	14	120000	-0.79862	0.78774	C6orf164	76353	25kb
ENSG00000272514	6	88092701	88199183	346	20	120000	-0.94424	0.82748	C6ORF165	76353	25kb
ENSG00000213204	6	88092730	88246352	499	27	120000	-0.8365	0.79856	C6orf165	76353	25kb
ENSG00000164414	6	88155341	88247054	282	19	120000	-0.60447	0.72723	SLC35A1	76353	25kb
ENSG00000146282	6	88199096	88324721	387	17	120000	-0.44565	0.67207	RARS2	76353	25kb
ENSG00000135336	6	88274839	88402169	315	19	120000	-0.22493	0.58898	ORC3	76353	25kb
ENSG00000135334	6	88359790	88436927	190	25	120000	0.056669	0.4774	AKIRIN2	76353	25kb
ENSG00000118434	6	88732507	88801550	106	12	120000	0.99604	0.15961	SPACA1	76353	25kb
ENSG00000118432	6	88824583	88901078	238	45	120000	0.40783	0.3417	CNR1	76353	25kb
ENSG00000111880	6	89294985	89698348	827	28	120000	2.1654	0.015178	RNGTT	76353	25kb
ENSG00000203863	6	89649246	89701685	133	16	120000	0.75981	0.22368	AL079342.1	76353	25kb
ENSG00000146278	6	89765470	89819879	181	17	120000	-0.95345	0.82982	PNRC1	76353	25kb
ENSG00000154548	6	89780678	89852800	210	19	120000	-0.56849	0.71515	SRSF12	76353	25kb
ENSG00000146281	6	89830769	89900284	210	20	120000	-0.21209	0.58398	PM20D2	76353	25kb
ENSG00000146276	6	89862220	89965997	416	35	120000	-1.4109	0.92087	GABRR1	76353	25kb
ENSG00000111886	6	89941927	90050018	382	56	120000	-2.1001	0.98214	GABRR2	76353	25kb
ENSG00000198833	6	90011344	90087567	182	35	120000	-1.0215	0.8465	UBE2J1	76353	25kb
ENSG00000025039	6	90049355	90146989	212	39	120000	0.58522	0.2792	RRAGD	76353	25kb
ENSG00000135299	6	90117889	90368553	532	37	120000	0.37294	0.3546	ANKRD6	76353	25kb
ENSG00000083099	6	90252863	90373472	258	25	120000	0.22687	0.41026	LYRM2	76353	25kb
ENSG00000112159	6	90327218	90554442	495	33	120000	0.41618	0.33864	MDN1	76353	25kb
ENSG00000135355	6	90579188	90630839	198	31	120000	1.6843	0.046059	GJA10	76353	25kb
ENSG00000112182	6	90611248	91031627	907	97	120000	-1.2521	0.89473	BACH2	76353	25kb
ENSG00000135341	6	91198292	91321764	379	29	120000	0.24127	0.40467	MAP3K7	76353	25kb
ENSG00000135333	6	93924738	94154265	571	54	120000	-1.2835	0.90034	EPHA7	76353	25kb
ENSG00000172469	6	96000419	96082333	304	18	120000	0.89621	0.18507	MANEA	76353	25kb
ENSG00000172461	6	96438860	96688488	1023	55	120000	-0.60259	0.72661	FUT9	76353	25kb
ENSG00000014123	6	96944471	97028152	150	18	120000	0.42952	0.33377	UFL1	76353	25kb
ENSG00000112214	6	96985424	97089512	280	16	120000	1.07	0.14231	FHL5	76353	25kb
ENSG00000112218	6	97217002	97310353	290	24	120000	0.66006	0.25461	GPR63	76353	25kb
ENSG00000123545	6	97312189	97370757	177	25	120000	1.0246	0.15277	NDUFAF4	76353	25kb
ENSG00000186231	6	97347605	97613630	804	52	120000	1.1637	0.12226	KLHL32	76353	25kb
ENSG00000146263	6	97565037	97756093	379	21	120000	-0.040498	0.51615	MMS22L	76353	25kb
ENSG00000184486	6	99257580	99311660	140	25	120000	0.20624	0.4183	POU3F2	76353	25kb
ENSG00000112234	6	99291420	99420849	241	26	120000	0.93236	0.17558	FBXL4	76353	25kb
ENSG00000146267	6	99694045	99822938	450	30	120000	0.98304	0.16279	FAXC	76353	25kb
ENSG00000132423	6	99792276	99867080	222	25	120000	0.96529	0.1672	COQ3	76353	25kb
ENSG00000132424	6	99820927	99898207	205	23	120000	1.1767	0.11966	PNISR	76353	25kb
ENSG00000123552	6	99855190	99994604	344	23	120000	2.0037	0.022551	USP45	76353	25kb
ENSG00000112237	6	99965256	100041849	223	23	120000	2.2866	0.011109	CCNC	76353	25kb
ENSG00000112238	6	100029606	100088454	213	30	120000	2.0035	0.022564	PRDM13	76353	25kb
ENSG00000152034	6	100342786	100467123	268	32	120000	-0.50183	0.69211	MCHR2	76353	25kb
ENSG00000112246	6	100807891	100937805	323	33	120000	-0.69216	0.75558	SIM1	76353	25kb
ENSG00000112249	6	100931070	101354248	1158	27	120000	-0.21082	0.58348	ASCC3	76353	25kb
ENSG00000164418	6	101821664	102542958	2115	131	120000	-0.59233	0.72319	GRIK2	76353	25kb
ENSG00000085382	6	105150968	105332794	401	18	120000	0.33808	0.36765	HACE1	76353	25kb
ENSG00000187772	6	105379923	105556207	277	37	120000	-0.34004	0.63309	LIN28B	76353	25kb
ENSG00000112276	6	105519697	105610049	226	21	120000	-0.43081	0.6667	BVES	76353	25kb
ENSG00000132429	6	105581155	105652870	184	21	120000	-1.0891	0.86194	POPDC3	76353	25kb
ENSG00000085377	6	105700440	105875959	464	41	120000	-1.1212	0.8689	PREP	76353	25kb
ENSG00000057657	6	106509195	106582814	168	33	120000	0.89565	0.18522	PRDM1	76353	25kb
ENSG00000057663	6	106607351	106798666	489	31	120000	0.46352	0.32149	ATG5	76353	25kb
ENSG00000112297	6	106934730	107043326	518	35	120000	-0.82707	0.7959	AIM1	76353	25kb
ENSG00000130347	6	106993903	107102373	430	26	120000	-0.54432	0.70689	RTN4IP1	76353	25kb
ENSG00000130348	6	107052453	107141292	325	29	120000	-1.4665	0.92875	QRSL1	76353	25kb
ENSG00000130349	6	107324407	107397546	257	22	120000	0.286	0.38744	C6orf203	76353	25kb
ENSG00000178409	6	107361386	107461473	305	42	120000	0.096723	0.46147	BEND3	76353	25kb
ENSG00000164494	6	107448761	107805768	772	49	120000	2.8781	0.0020004	PDSS2	76353	25kb
ENSG00000112320	6	107786162	108006357	461	56	120000	0.31145	0.37773	SOBP	76353	25kb
ENSG00000146285	6	108000308	108170521	559	41	120000	-0.22369	0.5885	SCML4	76353	25kb
ENSG00000025796	6	108163960	108304393	266	21	120000	-0.93408	0.82487	SEC63	76353	25kb
ENSG00000081087	6	108337613	108512058	471	61	120000	0.86074	0.19469	OSTM1	76353	25kb
ENSG00000112333	6	108462262	108535013	126	29	120000	2.1327	0.016474	NR2E1	76353	25kb
ENSG00000112335	6	108507426	108607464	181	23	120000	0.73494	0.23119	SNX3	76353	25kb
ENSG00000135537	6	108591098	108872999	492	35	120000	0.74684	0.22758	LACE1	76353	25kb
ENSG00000118689	6	108856038	109030977	315	26	120000	-0.39782	0.65462	FOXO3	76353	25kb
ENSG00000118690	6	109144619	109320186	462	39	120000	0.46984	0.31923	ARMC2	76353	25kb
ENSG00000080546	6	109282640	109441022	302	13	120000	0.63957	0.26123	SESN1	76353	25kb
ENSG00000183137	6	109391313	109510135	158	16	120000	0.7474	0.22741	CEP57L1	76353	25kb
ENSG00000135535	6	109662717	109728762	183	16	120000	-0.74801	0.77277	CD164	76353	25kb
ENSG00000185250	6	109686418	109787374	260	16	120000	-0.70067	0.75824	PPIL6	76353	25kb
ENSG00000135587	6	109736966	109790122	134	17	120000	-0.65568	0.74399	SMPD2	76353	25kb
ENSG00000135596	6	109740265	109812171	176	21	120000	-0.86065	0.80528	MICAL1	76353	25kb
ENSG00000112365	6	109758797	109829440	173	22	120000	-0.86961	0.80774	ZBTB24	76353	25kb
ENSG00000155085	6	109789059	110037420	707	21	120000	-0.45005	0.67366	AK9	76353	25kb
ENSG00000112367	6	109987499	110171631	411	34	120000	-1.2459	0.89361	FIG4	76353	25kb
ENSG00000146360	6	110274514	110326921	125	25	120000	0.33993	0.36695	GPR6	76353	25kb
ENSG00000112290	6	110396022	110526207	323	14	120000	-0.67162	0.74909	WASF1	76353	25kb
ENSG00000168438	6	110476344	110600478	324	21	120000	-1.2482	0.89401	CDC40	76353	25kb
ENSG00000053328	6	110542131	110704475	451	37	120000	-0.68213	0.75242	METTL24	76353	25kb
ENSG00000203797	6	110687974	110761765	300	35	120000	-1.4678	0.92892	DDO	76353	25kb
ENSG00000004809	6	110720890	110822844	442	55	120000	-0.10916	0.54346	SLC22A16	76353	25kb
ENSG00000155111	6	110906181	111162161	553	27	120000	-1.2105	0.88695	CDK19	76353	25kb
ENSG00000123505	6	111170973	111241916	262	17	120000	-0.46927	0.68056	AMD1	76353	25kb
ENSG00000155115	6	111254763	111314093	269	13	120000	0.33601	0.36843	GTF3C6	76353	25kb
ENSG00000197498	6	111278218	111374466	436	22	120000	0.36306	0.35828	RPF2	76353	25kb
ENSG00000112394	6	111383781	111577397	340	28	120000	1.3962	0.081334	SLC16A10	76353	25kb
ENSG00000173214	6	111555551	111617370	137	24	120000	1.2263	0.11004	KIAA1919	76353	25kb
ENSG00000009413	6	111595234	111829918	499	31	120000	0.55499	0.28945	REV3L	76353	25kb
ENSG00000056972	6	111852657	111952481	262	27	120000	0.93619	0.17459	TRAF3IP2	76353	25kb
ENSG00000010810	6	111956535	112219655	789	44	120000	-0.30862	0.62119	FYN	76353	25kb
ENSG00000112761	6	112350275	112417171	128	13	120000	0.32677	0.37192	WISP3	76353	25kb

ENSG00000074935	6	112366980	112433732	144	9	120000	0.09041	0.46398	TUBE1	76353	25kb
ENSG00000203778	6	112383802	112448993	138	7	120000	-0.12168	0.54842	FAM229B	76353	25kb
ENSG00000112769	6	112404963	112601141	512	44	120000	1.6	0.054799	LAMA4	76353	25kb
ENSG00000251258	6	112643532	112697498	143	25	120000	-1.5979	0.94497	RFPL4B	76353	25kb
ENSG00000155130	6	114153541	114209648	116	21	120000	0.17555	0.43033	MARCKS	76353	25kb
ENSG00000196591	6	114229192	114357472	363	32	120000	-0.66836	0.74805	HDAC2	76353	25kb
ENSG00000249853	6	114351750	114689209	703	70	120000	-0.48283	0.68539	HS3ST5	76353	25kb
ENSG00000111816	6	116227312	116406921	446	31	120000	0.62513	0.26594	FRK	76353	25kb
ENSG00000178425	6	116397012	116595660	488	35	120000	0.064127	0.47443	NT5DC1	76353	25kb
ENSG00000123500	6	116415086	116504910	193	25	120000	0.072842	0.47097	COL10A1	76353	25kb
ENSG00000234188	6	116417320	116468093	116	18	120000	-0.22134	0.58759	AL121963.1	76353	25kb
ENSG00000187189	6	116546151	116600261	154	18	120000	0.45291	0.32531	TSPYL4	76353	25kb
ENSG00000111817	6	116550336	116787424	553	43	120000	0.28622	0.38736	DSE	76353	25kb
ENSG00000189241	6	116572741	116626066	149	15	120000	0.53789	0.29533	TSPYL1	76353	25kb
ENSG00000188820	6	116757533	116809934	143	19	120000	-0.42243	0.66364	FAM26F	76353	25kb
ENSG00000173626	6	116791152	116891773	284	20	120000	-0.13865	0.55514	TRAPPC3L	76353	25kb
ENSG00000178033	6	116807809	116870955	185	20	120000	-0.046406	0.51851	FAM26E	76353	25kb
ENSG00000164451	6	116825174	116905031	208	18	120000	0.038276	0.48473	FAM26D	76353	25kb
ENSG00000111832	6	116867530	116943838	192	22	120000	-0.026616	0.51062	RWDD1	76353	25kb
ENSG00000111834	6	116912642	116979148	176	20	120000	1.225	0.11029	RSPH4A	76353	25kb
ENSG00000153975	6	116931781	117014957	184	20	120000	1.7194	0.042771	ZUFSP	76353	25kb
ENSG00000196911	6	116977350	117088029	201	26	120000	2.318	0.010225	KPNA5	76353	25kb
ENSG00000183807	6	117048363	117111886	146	27	120000	1.9333	0.026597	FAM162B	76353	25kb
ENSG00000173612	6	117088248	117175200	204	26	120000	-0.35576	0.63899	GPRC6A	76353	25kb
ENSG00000185002	6	117173375	117278326	415	21	120000	-0.0017889	0.50071	RFX6	76353	25kb
ENSG00000170162	6	117561721	117619728	166	33	120000	-0.32179	0.6262	VGLL2	76353	25kb
ENSG00000047936	6	117584463	117772018	645	53	120000	0.36161	0.35882	ROS1	76353	25kb
ENSG00000047932	6	117614374	117948691	1095	60	120000	0.96798	0.16653	GOPC	76353	25kb
ENSG00000164465	6	117749980	117916021	577	36	120000	1.1637	0.12228	DCBLD1	76353	25kb
ENSG00000153989	6	117971665	118056803	232	39	120000	0.77961	0.21781	NUS1	76353	25kb
ENSG00000196376	6	118203689	118663839	1237	80	120000	0.26673	0.39484	SLC35F1	76353	25kb
ENSG00000111860	6	118756935	119056238	825	31	120000	0.35204	0.3624	CEP85L	76353	25kb
ENSG00000198523	6	118844461	118906893	158	19	120000	-0.25978	0.60248	PLN	76353	25kb
ENSG00000111877	6	119109605	119281327	608	29	120000	-0.56937	0.71545	MCM9	76353	25kb
ENSG00000111875	6	119190384	119255332	191	20	120000	-0.38633	0.65037	ASF1A	76353	25kb
ENSG00000111879	6	119255928	119495552	775	49	120000	-0.072421	0.52887	FAM184A	76353	25kb
ENSG00000111885	6	119473374	119695926	792	30	120000	-0.47377	0.68217	MAN1A1	76353	25kb
ENSG00000146350	6	121375640	121680891	1155	26	120000	0.040339	0.48391	TBC1D32	76353	25kb
ENSG00000152661	6	121731838	121795873	245	32	120000	1.2236	0.11055	GJA1	76353	25kb
ENSG00000025156	6	122695691	122779264	206	20	120000	-0.37249	0.64524	HSF2	76353	25kb
ENSG00000111897	6	122739499	122817967	168	17	120000	-1.0264	0.84765	SERINC1	76353	25kb
ENSG00000135549	6	122768076	123072518	779	45	120000	-1.0164	0.84529	PKIB	76353	25kb
ENSG00000164434	6	123075620	123130219	165	36	120000	1.5167	0.064668	FABP7	76353	25kb
ENSG00000172594	6	123085315	123155865	197	47	120000	1.7686	0.038483	SMPDL3A	76353	25kb
ENSG00000146352	6	123292116	123419072	318	30	120000	-0.38482	0.64982	CLVS2	76353	25kb
ENSG00000186439	6	123512483	123983238	1344	68	120000	0.60589	0.27229	TRDN	76353	25kb
ENSG00000188580	6	124100286	125171803	2873	228	120000	-0.78777	0.78458	NKAIN2	76353	25kb
ENSG00000146373	6	125258691	125438779	402	57	120000	-0.5507	0.70908	RNF217	76353	25kb
ENSG00000111907	6	125415195	125610553	610	41	120000	-0.019569	0.50781	TPD52L1	76353	25kb
ENSG00000111906	6	125516108	125648282	402	31	120000	0.20597	0.41841	HDDC2	76353	25kb
ENSG00000135547	6	126043810	126107415	84	10	120000	0.35666	0.36067	HEY2	76353	25kb
ENSG00000111912	6	126077307	126277266	342	31	120000	-0.0071948	0.50287	NCOA7	76353	25kb
ENSG00000111911	6	126252927	126326387	94	9	120000	0.001666	0.49934	HINT3	76353	25kb
ENSG00000066651	6	126282576	126385422	100	7	120000	0.27857	0.39029	TRMT11	76353	25kb
ENSG00000203760	6	126636320	126695021	59	12	120000	-0.069276	0.52761	CENPW	76353	25kb
ENSG00000146374	6	127414749	127543910	236	22	120000	0.40911	0.34123	RSPO3	76353	25kb
ENSG00000118518	6	127562755	127634712	143	16	120000	-1.3435	0.91045	RNF146	76353	25kb
ENSG00000093144	6	127584855	127689754	212	18	120000	-1.2973	0.90274	ECHDC1	76353	25kb
ENSG00000255330	6	127734551	127865146	367	32	120000	-1.8796	0.96992	SOGA3	76353	25kb
ENSG00000214338	6	127734551	127865500	369	31	120000	-1.8367	0.96687	SOGA3	76353	25kb
ENSG00000189367	6	127736488	127805536	148	10	120000	-1.1741	0.87982	KIAA0408	76353	25kb
ENSG00000184530	6	127815600	127937962	442	30	120000	-1.2359	0.89176	C6orf58	76353	25kb
ENSG00000172673	6	128004217	128264776	502	35	120000	0.17118	0.43204	THEMIS	76353	25kb
ENSG00000152894	6	128264924	128866870	1193	86	120000	0.31709	0.37559	PTPRK	76353	25kb
ENSG00000196569	6	129179342	129862714	1801	82	120000	-2.318	0.98978	LAMA2	76353	25kb
ENSG00000146376	6	129872277	130056370	586	63	120000	-2.29	0.98899	ARHGAP18	76353	25kb
ENSG00000203756	6	130127389	130207692	404	30	120000	0.358	0.36017	TMEM244	76353	25kb
ENSG00000198945	6	130309844	130487594	480	42	120000	0.28298	0.3886	L3MBTL3	76353	25kb
ENSG00000164483	6	130440460	130711570	1193	35	120000	-0.86331	0.80602	SAMD3	76353	25kb
ENSG00000164484	6	130661879	130789208	410	25	120000	-1.7287	0.95807	TMEM200A	76353	25kb
ENSG00000256162	6	131123546	131183275	213	30	120000	-1.9574	0.97485	SMLR1	76353	25kb
ENSG00000079819	6	131135487	131409462	880	36	120000	-1.7385	0.95894	EPB41L2	76353	25kb
ENSG00000118507	6	131431806	131629675	453	46	120000	0.28294	0.38861	AKAP7	76353	25kb
ENSG00000118520	6	131869284	131930472	114	16	120000	-1.0503	0.85322	ARG1	76353	25kb
ENSG00000112282	6	131870106	131974369	152	20	120000	-1.0879	0.86168	MED23	76353	25kb
ENSG00000154269	6	131924582	132093553	337	32	120000	-0.14276	0.55676	ENPP3	76353	25kb
ENSG00000180658	6	131996520	132047635	100	14	120000	0.34625	0.36458	OR2A4	76353	25kb
ENSG00000236761	6	132004581	132057206	112	14	120000	0.0080368	0.49679	CTAGE9	76353	25kb
ENSG00000197594	6	132104156	132241295	366	41	120000	-0.1315	0.55231	ENPP1	76353	25kb
ENSG00000118523	6	132244316	132297513	128	26	120000	-0.78592	0.78404	CTGF	76353	25kb
ENSG00000079931	6	132592194	132747684	376	39	120000	-1.2884	0.9012	MOXD1	76353	25kb
ENSG00000079950	6	132742006	132859337	354	42	120000	0.97367	0.16511	STX7	76353	25kb
ENSG00000146385	6	132848832	132899860	225	36	120000	0.047611	0.48101	TAAR8	76353	25kb
ENSG00000146383	6	132866461	132917498	238	42	120000	1.1572	0.12359	TAAR6	76353	25kb
ENSG00000135569	6	132884731	132935877	276	34	120000	1.7368	0.041213	TAAR5	76353	25kb
ENSG00000146378	6	132913161	132970414	319	41	120000	1.0099	0.15628	TAAR2	76353	25kb
ENSG00000146399	6	132941123	132992142	239	25	120000	-1.4617	0.92809	TAAR1	76353	25kb
ENSG00000112299	6	132977729	133060188	351	34	120000	-1.0498	0.85309	VNN1	76353	25kb
ENSG00000093134	6	133018926	133080904	273	31	120000	0.028852	0.48849	VNN3	76353	25kb
ENSG00000112303	6	133040009	133109598	264	27	120000	0.19974	0.42084	VNN2	76353	25kb
ENSG00000146409	6	133065509	133144701	303	26	120000	-0.23161	0.59158	SLC18B1	76353	25kb
ENSG00000112306	6	133110580	133163703	218	21	120000	0.25636	0.39884	RPS12	76353	25kb
ENSG00000112319	6	133536736	133878258	958	64	120000	0.78745	0.21551	EYA4	76353	25kb
ENSG00000118526	6	134185276	134241691	162	21	120000	0.71522	0.23724	TCF21	76353	25kb
ENSG00000028839	6	134248308	134336570	212	29	120000	-0.42322	0.66393	TBPL1	76353	25kb
ENSG00000146411	6	134284835	134398774	229	43	120000	0.17041	0.43234	SLC2A12	76353	25kb

ENSG00000118515	6	134465384	134664250	574	80	120000	1.1135	0.13274	SGK1	76353	25kb
ENSG00000118514	6	135213528	135296260	152	15	120000	-0.035636	0.51421	ALDH8A1	76353	25kb
ENSG00000112339	6	135256516	135449194	403	19	120000	-0.43202	0.66714	HBS1L	76353	25kb
ENSG00000118513	6	135477453	135565311	183	33	120000	-0.02645	0.51055	MYB	76353	25kb
ENSG00000135541	6	135579670	135843914	466	38	120000	1.9545	0.025319	AHI1	76353	25kb
ENSG00000171408	6	136147834	136541712	666	69	120000	0.81466	0.20763	PDE7B	76353	25kb
ENSG00000146410	6	136527162	136596473	41	20	120000	0.097017	0.46136	MTFR2	76353	25kb
ENSG00000029363	6	136553001	136635989	60	14	120000	-0.56457	0.71382	BCLAF1	76353	25kb
ENSG00000135525	6	136638875	136896957	348	19	120000	-1.1142	0.8674	MAP7	76353	25kb
ENSG00000197442	6	136853185	137138656	511	37	120000	-1.814	0.96516	MAP3K5	76353	25kb
ENSG00000112357	6	137118717	137260075	307	22	120000	-1.0358	0.84986	PEX7	76353	25kb
ENSG00000182747	6	137218402	137271777	141	17	120000	-0.58939	0.7222	SLC35D3	76353	25kb
ENSG00000016402	6	137296108	137391298	336	31	120000	-0.32323	0.62674	IL20RA	76353	25kb
ENSG00000164485	6	137439968	137519785	242	35	120000	2.0923	0.018207	IL22RA2	76353	25kb
ENSG00000027697	6	137493621	137565586	149	31	120000	2.497	0.0062632	IFNGR1	76353	25kb
ENSG00000177468	6	137788336	137840531	210	37	120000	-1.1998	0.88488	OLIG3	76353	25kb
ENSG00000118503	6	138163351	138229449	120	17	120000	1.0396	0.14926	TNFAIP3	76353	25kb
ENSG00000112378	6	138384642	138453648	266	33	120000	-1.2646	0.89698	PERP	76353	25kb
ENSG00000112379	6	138458058	138690800	673	40	120000	-1.9362	0.97358	KIAA1244	76353	25kb
ENSG00000254440	6	138512129	138564627	144	12	120000	-0.74551	0.77202	PBOV1	76353	25kb
ENSG00000262543	6	138674042	138729212	196	9	120000	-0.50126	0.69191	RP3-422G23.4	76353	25kb
ENSG00000051620	6	138699668	138768334	158	15	120000	-0.10689	0.54256	HEBP2	76353	25kb
ENSG00000135540	6	138718180	139038708	919	77	120000	-0.30919	0.62141	NHSL1	76353	25kb
ENSG00000024862	6	139069657	139139456	223	30	120000	0.18187	0.42784	CCDC28A	76353	25kb
ENSG00000203734	6	139092063	139250207	496	58	120000	1.5219	0.064022	ECT2L	76353	25kb
ENSG00000135597	6	139199630	139334398	423	21	120000	2.226	0.013007	REPS1	76353	25kb
ENSG00000146386	6	139324819	139389439	198	31	120000	0.85297	0.19684	ABRACL	76353	25kb
ENSG00000112406	6	139431249	139526939	290	14	120000	0.17063	0.43226	HECA	76353	25kb
ENSG00000164440	6	139536198	139638276	270	32	120000	0.9367	0.17446	TXLNB	76353	25kb
ENSG00000164442	6	139668393	139720757	154	28	120000	0.784	0.21652	CITED2	76353	25kb
ENSG00000135577	6	142354467	142434936	158	14	120000	0.24879	0.40176	NMBR	76353	25kb
ENSG00000203733	6	142429227	142481288	92	8	120000	-0.3364	0.63172	GJE1	76353	25kb
ENSG00000009844	6	142443367	142570826	262	14	120000	-0.83004	0.79674	VTA1	76353	25kb
ENSG00000112414	6	142597991	142792403	313	32	120000	-0.89614	0.81491	GPR126	76353	25kb
ENSG00000010818	6	143047604	143291338	611	64	120000	-0.60122	0.72615	HIVEP2	76353	25kb
ENSG00000146416	6	143356633	143686441	667	61	120000	-1.4364	0.92456	AIG1	76353	25kb
ENSG00000189007	6	143718969	143796810	184	14	120000	-0.47091	0.68115	ADAT2	76353	25kb
ENSG00000268402	6	143734575	143789295	125	8	120000	-0.35165	0.63745	AL031320.1	76353	25kb
ENSG00000034693	6	143746944	143836147	180	13	120000	-0.21364	0.58459	PEX3	76353	25kb
ENSG00000001036	6	143790948	143857827	148	22	120000	-0.46338	0.67845	FUCA2	76353	25kb
ENSG00000112419	6	143832982	144177322	1070	63	120000	0.70831	0.23938	PHACTR2	76353	25kb
ENSG00000257065	6	144103229	144190754	218	31	120000	0.20343	0.4194	RP3-468K18.5	76353	25kb
ENSG00000135521	6	144139481	144209949	182	24	120000	-0.29513	0.61605	LTV1	76353	25kb
ENSG00000118491	6	144160573	144284483	287	38	120000	-1.1716	0.87932	ZC2HC1B	76353	25kb
ENSG00000118495	6	144236437	144410735	456	58	120000	-0.71061	0.76134	PLAGL1	76353	25kb
ENSG00000169976	6	144391018	144441754	134	24	120000	0.067524	0.47308	SF3B5	76353	25kb
ENSG00000135604	6	144446663	144534507	192	38	120000	-0.023729	0.50947	STX11	76353	25kb
ENSG00000152818	6	144581837	145199170	1315	83	120000	-0.30205	0.61869	UTRN	76353	25kb
ENSG00000112425	6	145797719	146082160	779	17	120000	0.5028	0.30755	EPM2A	76353	25kb
ENSG00000118496	6	146089638	146160889	126	15	120000	0.95001	0.17105	FBXO30	76353	25kb
ENSG00000146414	6	146160381	146310559	211	12	120000	0.11042	0.45604	SHPRH	76353	25kb
ENSG00000152822	6	146323782	146783734	873	45	120000	-0.39533	0.6537	GRM1	76353	25kb
ENSG00000118508	6	146839829	146901101	78	15	120000	-0.33296	0.63042	RAB32	76353	25kb
ENSG00000118492	6	146895101	147161598	674	67	120000	0.95421	0.16999	ADGB	76353	25kb
ENSG00000164506	6	147500561	147731866	346	22	120000	-1.6989	0.95533	STXBP5	76353	25kb
ENSG00000203727	6	147805063	148083683	1092	73	120000	-1.6976	0.95521	SAMD5	76353	25kb
ENSG00000111961	6	148568440	148898186	1207	130	120000	-0.13273	0.5528	SASH1	76353	25kb
ENSG00000111962	6	149043464	149423126	1124	113	120000	-0.50439	0.69301	UST	76353	25kb
ENSG00000055208	6	149514777	149757749	815	43	120000	0.012792	0.4949	TAB2	76353	25kb
ENSG00000177688	6	149696495	149747177	166	16	120000	0.12287	0.4511	SUMO4	76353	25kb
ENSG00000178199	6	149743794	149831197	283	17	120000	0.65273	0.25697	ZC3H12D	76353	25kb
ENSG00000131013	6	149800869	149892174	133	11	120000	0.29068	0.38565	PPIL4	76353	25kb
ENSG00000055211	6	149862430	149937884	86	10	120000	-0.039485	0.51575	GINM1	76353	25kb
ENSG00000186625	6	149891009	149995108	166	6	120000	0.028646	0.48857	KATNA1	76353	25kb
ENSG00000131023	6	149954289	150064392	174	6	120000	0.0022199	0.49911	LATS1	76353	25kb
ENSG00000120253	6	150020451	150095801	128	5	120000	-0.01078	0.5043	NUP43	76353	25kb
ENSG00000120265	6	150045579	150157556	226	6	120000	-0.004267	0.5017	PCMT1	76353	25kb
ENSG00000120256	6	150114934	150211199	221	13	120000	-0.12761	0.55077	LRP11	76353	25kb
ENSG00000164520	6	150179511	150244238	192	13	120000	0.10804	0.45698	RAET1E	76353	25kb
ENSG00000203722	6	150213014	150269257	166	23	120000	-0.093015	0.53705	RAET1G	76353	25kb
ENSG00000131015	6	150238136	150295371	189	22	120000	-0.33209	0.63009	ULBP2	76353	25kb
ENSG00000111981	6	150260143	150319846	242	24	120000	-0.17671	0.57013	ULBP1	76353	25kb
ENSG00000155918	6	150314470	150371668	171	19	120000	0.026097	0.48959	RAET1L	76353	25kb
ENSG00000131019	6	150359286	150415231	261	20	120000	-0.59195	0.72306	ULBP3	76353	25kb
ENSG00000198729	6	150439212	150596493	594	68	120000	-0.42215	0.66354	PPP1R14C	76353	25kb
ENSG00000009765	6	150665028	150752105	456	38	120000	1.1358	0.12801	IYD	76353	25kb
ENSG00000120278	6	150895999	151189799	1085	70	120000	0.4713	0.31871	PLEKHG1	76353	25kb
ENSG00000120254	6	151161685	151448023	1215	100	120000	0.22697	0.41022	MTHFD1L	76353	25kb
ENSG00000131016	6	151536134	151704692	576	69	120000	2.0586	0.019768	AKAP12	76353	25kb
ENSG00000181472	6	151660252	151737683	205	30	120000	2.0619	0.019607	ZBTB2	76353	25kb
ENSG00000155906	6	151700989	151798259	286	20	120000	0.14334	0.44301	RMND1	76353	25kb
ENSG00000146476	6	151748422	151816236	197	19	120000	-0.56082	0.71254	C6orf211	76353	25kb
ENSG00000120262	6	151790165	151967328	648	47	120000	1.3679	0.085669	CCDC170	76353	25kb
ENSG00000091831	6	151952826	152475754	1649	105	120000	-0.53598	0.70401	ESR1	76353	25kb
ENSG00000131018	6	152417819	152983936	1882	133	120000	-1.3906	0.91783	SYNE1	76353	25kb
ENSG00000120279	6	152994030	153070702	295	17	120000	-0.30051	0.61811	MYCT1	76353	25kb
ENSG00000146469	6	153046933	153105900	178	35	120000	-0.95574	0.8304	VIP	76353	25kb
ENSG00000112029	6	153266664	153329714	237	21	120000	0.22765	0.40996	FBXO5	76353	25kb
ENSG00000112031	6	153283497	153348820	191	19	120000	0.27428	0.39193	MTRF1L	76353	25kb
ENSG00000091844	6	153300594	153477384	558	31	120000	0.56351	0.28654	RGS17	76353	25kb
ENSG00000213121	6	153527455	153693623	586	49	120000	1.8754	0.030369	AL590867.1	76353	25kb
ENSG00000112038	6	154306631	154593001	870	71	120000	-1.0978	0.86386	OPRM1	76353	25kb
ENSG00000074706	6	154450631	154702926	898	85	120000	1.8594	0.031482	IPCEF1	76353	25kb
ENSG00000153721	6	154683638	154856793	697	80	120000	1.0406	0.14903	CNKSR3	76353	25kb
ENSG00000213079	6	155029459	155180192	299	19	120000	-1.4856	0.93131	SCAF8	76353	25kb
ENSG00000146426	6	155128831	155603857	1582	165	120000	0.90299	0.18327	TIAM2	76353	25kb

ENSG00000029639	6	155553643	155660627	443	30	120000	-0.48911	0.68762	TFB1M	76353	25kb
ENSG00000171217	6	155560147	155622682	293	20	120000	-0.52625	0.70064	CLDN20	76353	25kb
ENSG00000074771	6	155691504	155802037	317	56	120000	-1.1815	0.88129	NOX3	76353	25kb
ENSG00000049618	6	157074063	157556913	1075	81	120000	0.7166	0.23681	ARID1B	76353	25kb
ENSG00000215712	6	157685418	157769633	181	17	120000	1.3917	0.081999	TMEM242	76353	25kb
ENSG00000175048	6	157777165	158124178	1254	96	120000	-0.60236	0.72653	ZDHHC14	76353	25kb
ENSG00000130340	6	158219296	158391109	563	20	120000	-0.25686	0.60136	SNX9	76353	25kb
ENSG00000078269	6	158377888	158545208	533	49	120000	0.1276	0.44923	SYNJ2	76353	25kb
ENSG00000122335	6	158505536	158614312	247	24	120000	0.84445	0.19921	SERAC1	76353	25kb
ENSG00000272047	6	158564384	158645376	242	26	120000	-0.11153	0.5444	GTF2H5	76353	25kb
ENSG00000130338	6	158708692	158957860	834	33	120000	0.71365	0.23772	TULP4	76353	25kb
ENSG00000146433	6	158932468	159081460	448	35	120000	-0.5812	0.71945	TMEM181	76353	25kb
ENSG00000214239	6	159022471	159074322	149	17	120000	0.016189	0.49354	AL591025.1	76353	25kb
ENSG00000146425	6	159032506	159090771	152	18	120000	0.13482	0.44638	DYNLT1	76353	25kb
ENSG00000164674	6	159046046	159210908	548	53	120000	-0.22268	0.58811	SYTL3	76353	25kb
ENSG00000092820	6	159161773	159265444	382	23	120000	-0.59975	0.72566	EZR	76353	25kb
ENSG00000203711	6	159265971	159356385	351	47	120000	0.80911	0.20923	C6orf99	76353	25kb
ENSG00000130363	6	159368903	159446219	381	13	120000	0.38697	0.34939	RSPH3	76353	25kb
ENSG00000164691	6	159430500	159491184	192	18	120000	0.53662	0.29576	TAGAP	76353	25kb
ENSG00000164694	6	159565429	159718141	644	24	120000	-0.18751	0.57437	FNDC1	76353	25kb
ENSG00000112096	6	160065089	160208561	411	25	120000	0.45759	0.32362	SOD2	76353	25kb
ENSG00000146457	6	160121617	160202351	210	20	120000	0.87672	0.19032	WTAP	76353	25kb
ENSG00000120437	6	160156360	160225144	226	17	120000	1.0525	0.14629	ACAT2	76353	25kb
ENSG00000120438	6	160174530	160235781	208	17	120000	0.9104	0.18131	TCP1	76353	25kb
ENSG00000112110	6	160185844	160244468	197	19	120000	0.72068	0.23555	MRPL18	76353	25kb
ENSG00000146453	6	160196298	160266736	250	32	120000	0.37611	0.35342	PNLDC1	76353	25kb
ENSG00000130368	6	160302974	160354108	105	14	120000	-0.86881	0.80752	MAS1	76353	25kb
ENSG00000197081	6	160365131	160559539	481	58	120000	-1.1499	0.87491	IGF2R	76353	25kb
ENSG00000175003	6	160517821	160604750	223	44	120000	-0.52227	0.69926	SLC22A1	76353	25kb
ENSG00000112499	6	160567093	160723670	558	44	120000	0.47013	0.31913	SLC22A2	76353	25kb
ENSG00000146477	6	160744300	160901014	371	26	120000	1.5125	0.06521	SLC22A3	76353	25kb
ENSG00000198670	6	160927515	161112407	319	26	120000	-0.34622	0.63541	LPA	76353	25kb
ENSG00000122194	6	161098270	161199347	291	25	120000	-1.0671	0.85704	PLG	76353	25kb
ENSG00000085511	6	161387759	161563417	553	31	120000	-1.7686	0.96152	MAP3K4	76353	25kb
ENSG00000026652	6	161526011	161720093	621	68	120000	-0.85723	0.80434	AGPAT4	76353	25kb
ENSG00000185345	6	161743452	163173803	5218	277	120000	0.79312	0.21385	PARK2	76353	25kb
ENSG00000112530	6	163123164	163761524	2007	151	120000	0.7396	0.22977	PACRG	76353	25kb
ENSG00000225683	6	163561446	163637840	269	34	120000	1.0814	0.13975	AL078585.1	76353	25kb
ENSG00000112531	6	163810032	164024628	454	32	120000	-0.95946	0.83134	QKI	76353	25kb
ENSG00000112539	6	165668153	165748096	245	29	120000	2.2245	0.013059	C6orf118	76353	25kb
ENSG00000112541	6	165715776	166425091	2662	221	120000	1.844	0.032592	PDE10A	76353	25kb
ENSG00000256391	6	166282330	166334725	287	24	120000	2.0962	0.018031	SDIM1	76353	25kb
ENSG00000164458	6	166546144	166607188	311	24	120000	0.46103	0.32239	T	76353	25kb
ENSG00000176381	6	166694168	166746936	194	25	120000	0.26672	0.39484	PRR18	76353	25kb
ENSG00000198818	6	166708216	166781079	200	20	120000	0.20742	0.41784	SFT2D1	76353	25kb
ENSG00000060762	6	166753407	166821486	122	12	120000	0.4509	0.32603	MPC1	76353	25kb
ENSG00000071242	6	166797852	167344939	1894	178	120000	-0.59824	0.72516	RPS6KA2	76353	25kb
ENSG00000269048	6	166920369	166972709	168	23	120000	-1.0202	0.84617	Z98049.1	76353	25kb
ENSG00000249141	6	167246582	167394612	525	47	120000	-0.22264	0.58809	RP11-514O12.4	76353	25kb
ENSG00000026297	6	167317992	167395679	268	24	120000	-1.1704	0.87908	RNASET2	76353	25kb
ENSG00000213066	6	167387670	167491201	458	14	120000	-0.73252	0.76808	FGFR1OP	76353	25kb
ENSG00000112486	6	167500295	167578184	279	30	120000	-0.68214	0.75242	CCR6	76353	25kb
ENSG00000166984	6	167534902	167635401	395	43	120000	1.0291	0.15172	TCP10L2	76353	25kb
ENSG00000120436	6	167544759	167596817	150	20	120000	-0.32121	0.62598	GPR31	76353	25kb
ENSG00000112494	6	167659657	167754507	413	39	120000	0.25859	0.39797	UNC93A	76353	25kb
ENSG00000120440	6	167713574	167797991	307	32	120000	-0.044991	0.51794	TTLL2	76353	25kb
ENSG00000203690	6	167745521	167822998	279	37	120000	-0.50703	0.69393	TCP10	76353	25kb
ENSG00000146521	6	168160217	168222539	292	25	120000	-1.8738	0.96952	C6orf123	76353	25kb
ENSG00000269155	6	168171210	168223927	260	21	120000	-1.6996	0.9554	AL009178.1	76353	25kb
ENSG00000130396	6	168202602	168397703	620	29	120000	-0.12474	0.54964	MLLT4	76353	25kb
ENSG00000125337	6	168371921	168470769	458	57	120000	0.74076	0.22942	KIF25	76353	25kb
ENSG00000153303	6	168431425	168507237	369	49	120000	0.19679	0.422	FRMD1	76353	25kb
ENSG00000164488	6	168668510	168745434	327	46	120000	-0.014449	0.50576	DACT2	76353	25kb
ENSG00000112562	6	168816831	169098984	1053	82	120000	-1.6395	0.94944	SMOC2	76353	25kb
ENSG00000186340	6	169590875	169679139	401	53	120000	-0.35958	0.64042	THBS2	76353	25kb
ENSG00000184465	6	169832307	170127159	847	32	120000	-1.0173	0.84549	WDR27	76353	25kb
ENSG00000185127	6	170077233	170131401	109	16	120000	-0.22268	0.58811	C6orf120	76353	25kb
ENSG00000130024	6	170079001	170149151	161	18	120000	-0.25907	0.60221	PHF10	76353	25kb
ENSG00000184786	6	170115210	170176655	159	20	120000	-0.38079	0.64832	TCTE3	76353	25kb
ENSG00000130023	6	170126718	170206680	267	19	120000	-0.4546	0.6753	ERMARD	76353	25kb
ENSG00000198719	6	170566294	170624561	181	33	120000	0.12081	0.45192	DLL1	76353	25kb
ENSG00000112584	6	170574791	170741153	528	26	120000	1.1407	0.127	FAM120B	76353	25kb
ENSG00000008018	6	170819205	170887429	175	14	120000	-1.4445	0.9257	PSMB1	76353	25kb
ENSG00000112592	6	170838390	170906958	189	14	120000	-1.3924	0.9181	TBP	76353	25kb
ENSG00000071994	6	170859383	170918780	161	13	120000	-1.3995	0.91918	PDCD2	76353	25kb
ENSG00000177706	7	167969	325711	669	67	120000	-0.4941	0.68938	FAM20C	76353	25kb
ENSG00000249852	7	202554	254557	170	33	120000	-0.70186	0.75862	AC145676.2	76353	25kb
ENSG00000269396	7	277918	329070	317	42	120000	0.032491	0.48704	AC187652.1	76353	25kb
ENSG00000197461	7	511895	584933	371	56	120000	-0.081984	0.53267	PDGFA	76353	25kb
ENSG00000188191	7	563834	792287	818	77	120000	2.1777	0.014715	PRKAR1B	76353	25kb
ENSG00000164818	7	741338	854190	399	25	120000	2.3931	0.0083527	HEATR2	76353	25kb
ENSG00000164828	7	830528	961072	525	41	120000	1.9863	0.023499	SUN1	76353	25kb
ENSG00000239857	7	891189	961073	288	40	120000	1.8739	0.030474	GET4	76353	25kb
ENSG00000105963	7	912540	1020043	441	69	120000	0.37121	0.35524	ADAP1	76353	25kb
ENSG00000240230	7	913415	1040235	516	64	120000	0.31295	0.37716	COX19	76353	25kb
ENSG00000073067	7	997835	1054276	252	25	120000	-1.4379	0.92477	CYP2W1	76353	25kb
ENSG00000146540	7	1011622	1202896	887	36	120000	-1.0142	0.84476	C7orf50	76353	25kb
ENSG00000164849	7	1059212	1123897	328	21	120000	-0.27501	0.60835	GPR146	76353	25kb
ENSG00000164850	7	1096844	1158451	288	26	120000	-0.75965	0.77627	GPER1	76353	25kb
ENSG00000178381	7	1166707	1225395	352	22	120000	-1.3921	0.91805	ZFAND2A	76353	25kb
ENSG00000164853	7	1247543	1301954	184	33	120000	1.0249	0.15269	UNCX	76353	25kb
ENSG00000164877	7	1443101	1524138	236	26	120000	-1.2811	0.89992	MICALL2	76353	25kb
ENSG00000164880	7	1484913	1570489	264	13	120000	-0.48656	0.68672	INTS1	76353	25kb
ENSG00000198517	7	1545350	1607679	227	16	120000	0.26674	0.39483	MAFK	76353	25kb
ENSG00000164855	7	1556871	1625457	251	22	120000	0.39374	0.34689	TMEM184A	76353	25kb
ENSG00000157778	7	1581966	1635641	185	33	120000	0.90989	0.18144	PSMG3	76353	25kb

ENSG00000225968	7	1702755	1812590	307	45	120000	0.41917	0.33755	ELFN1	76353	25kb
ENSG00000205971	7	1707446	1758961	122	18	120000	0.9575	0.16916	AC074389.6	76353	25kb
ENSG00000002822	7	1830429	2297878	1979	43	120000	-0.66255	0.74619	MAD1L1	76353	25kb
ENSG00000176349	7	1853222	1914567	281	17	120000	-0.35027	0.63693	AC110781.3	76353	25kb
ENSG00000122687	7	2248866	2306840	223	33	120000	0.18193	0.42782	FTSJ2	76353	25kb
ENSG00000106268	7	2256857	2315781	231	31	120000	0.38808	0.34898	NUDT1	76353	25kb
ENSG00000106266	7	2266405	2418953	436	50	120000	0.15276	0.43929	SNX8	76353	25kb
ENSG00000106263	7	2368721	2445380	186	22	120000	0.010734	0.49572	EIF3B	76353	25kb
ENSG00000136213	7	2418223	2499242	220	36	120000	-0.8351	0.79817	CHST12	76353	25kb
ENSG00000106003	7	2527163	2593811	235	43	120000	0.97159	0.16563	LFNG	76353	25kb
ENSG00000106009	7	2552511	2620361	241	39	120000	0.33579	0.36851	BRAT1	76353	25kb
ENSG00000106012	7	2573632	2679368	455	46	120000	0.1569	0.43766	IQCE	76353	25kb
ENSG00000136295	7	2646585	2729436	277	44	120000	0.5826	0.28008	TTYH3	76353	25kb
ENSG00000174945	7	2694156	2840134	545	49	120000	0.74637	0.22772	AMZ1	76353	25kb
ENSG00000146535	7	2742746	2908958	599	31	120000	0.17481	0.43061	GNA12	76353	25kb
ENSG00000198286	7	2920775	3108579	828	121	120000	-0.69109	0.75524	CARD11	76353	25kb
ENSG00000217455	7	3154868	3239287	511	46	120000	3.3616	0.00038748	AC091801.1	76353	25kb
ENSG00000146555	7	3316080	4333632	4079	120	120000	-0.73595	0.76912	SDK1	76353	25kb
ENSG00000164916	7	4658388	4836074	607	66	120000	-1.0743	0.85867	FOXK1	76353	25kb
ENSG00000242802	7	4790253	4858943	302	18	120000	-0.25589	0.60098	AP5Z1	76353	25kb
ENSG00000157927	7	4811686	4948350	600	47	120000	-0.53254	0.70283	RADIL	76353	25kb
ENSG00000218823	7	4872364	4926625	228	26	120000	-0.11362	0.54523	PAPOLB	76353	25kb
ENSG00000136297	7	4920620	5023844	385	41	120000	-0.28328	0.61152	MMD2	76353	25kb
ENSG00000272968	7	4998349	5137852	582	29	120000	0.26374	0.39599	RBAK-RBAKDN	76353	25kb
ENSG00000146587	7	5060452	5134119	305	25	120000	0.79505	0.21329	RBAK	76353	25kb
ENSG00000157954	7	5204819	5298457	410	44	120000	0.90375	0.18306	WIPI2	76353	25kb
ENSG00000164638	7	5289000	5371501	351	48	120000	-0.42323	0.66394	SLC29A4	76353	25kb
ENSG00000182095	7	5321421	5490045	642	87	120000	-0.0062686	0.5025	TNRC18	76353	25kb
ENSG00000155034	7	5445966	5578429	473	54	120000	-1.1721	0.87942	FBXL18	76353	25kb
ENSG00000075624	7	5541782	5628415	311	43	120000	-0.98925	0.83873	ACTB	76353	25kb
ENSG00000075618	7	5607439	5671286	239	40	120000	1.1609	0.12284	FSCN1	76353	25kb
ENSG00000011275	7	5634678	5846370	795	30	120000	2.2882	0.011062	RNF216	76353	25kb
ENSG00000122543	7	5894458	5950993	263	36	120000	-0.7816	0.78278	OCM	76353	25kb
ENSG00000122674	7	5913356	5990605	156	35	120000	-0.7512	0.77373	CCZ1	76353	25kb
ENSG00000155026	7	5940181	6035314	96	28	120000	-1.7237	0.95762	RSPH10B	76353	25kb
ENSG00000122512	7	5987870	6073756	240	32	120000	-1.2385	0.89224	PMS2	76353	25kb
ENSG00000106305	7	6023876	6088465	288	25	120000	-1.129	0.87055	AIMP2	76353	25kb
ENSG00000086232	7	6036881	6123861	379	31	120000	-1.3213	0.9068	EIF2AK1	76353	25kb
ENSG00000157999	7	6046007	6101017	235	28	120000	-1.2372	0.892	ANKRD61	76353	25kb
ENSG00000106346	7	6119515	6226195	320	28	120000	-0.77578	0.78106	USP42	76353	25kb
ENSG00000008256	7	6176407	6337275	475	38	120000	-0.14169	0.55634	CYTH3	76353	25kb
ENSG00000178397	7	6344040	6413612	295	33	120000	-0.4864	0.68666	FAM220A	76353	25kb
ENSG00000136238	7	6389154	6468608	346	26	120000	-0.9418	0.82685	RAC1	76353	25kb
ENSG00000164535	7	6423757	6548821	480	45	120000	-1.5359	0.93772	DAGLB	76353	25kb
ENSG00000136240	7	6460584	6548873	331	33	120000	-1.6701	0.95255	KDELR2	76353	25kb
ENSG00000269781	7	6497922	6549873	170	22	120000	-1.8078	0.96468	FLJ20306	76353	25kb
ENSG00000215045	7	6512093	6616067	325	39	120000	0.23316	0.40782	GRID2IP	76353	25kb
ENSG00000136247	7	6592065	6654005	192	19	120000	0.31235	0.37739	ZDHHC4	76353	25kb
ENSG00000146576	7	6604648	6673357	206	21	120000	0.35967	0.35955	C7orf26	76353	25kb
ENSG00000236609	7	6630248	6688921	192	23	120000	0.24275	0.4041	ZNF853	76353	25kb
ENSG00000198580	7	6688376	6740991	168	26	120000	-0.27405	0.60798	AC073343.1	76353	25kb
ENSG00000164631	7	6703064	6771554	243	28	120000	0.2577	0.39832	ZNF12	76353	25kb
ENSG00000169402	7	6768740	6863996	70	12	120000	0.3731	0.35454	RSPH10B2	76353	25kb
ENSG00000146574	7	6808765	6891401	146	17	120000	0.96349	0.16765	CCZ1B	76353	25kb
ENSG00000106392	7	7171565	7313282	649	36	120000	-1.6994	0.95538	C1GALT1	76353	25kb
ENSG00000215018	7	7370834	7600484	717	31	120000	-0.56929	0.71542	COL28A1	76353	25kb
ENSG00000164654	7	7581503	7673560	312	20	120000	-0.25543	0.6008	MIOS	76353	25kb
ENSG00000106399	7	7651149	7783238	539	34	120000	0.47891	0.316	RPA3	76353	25kb
ENSG00000219545	7	7655342	8068689	1779	149	120000	0.40239	0.3437	RPA3-AS1	76353	25kb
ENSG00000106415	7	7983425	8158902	611	40	120000	0.20333	0.41944	GLCCI1	76353	25kb
ENSG00000003147	7	8127814	8327317	807	94	120000	0.57747	0.28181	ICA1	76353	25kb
ENSG00000122584	7	8448585	8817593	1577	100	120000	-1.064	0.85634	NXPH1	76353	25kb
ENSG00000189043	7	10946578	11004883	185	29	120000	1.7657	0.03872	NDUFA4	76353	25kb
ENSG00000106443	7	10988499	11234250	679	35	120000	1.9489	0.025653	PHF14	76353	25kb
ENSG00000005108	7	11384984	11896824	2057	133	120000	-1.222	0.88915	THSD7A	76353	25kb
ENSG00000106460	7	12225867	12307993	406	20	120000	0.89008	0.18671	TMEM106B	76353	25kb
ENSG00000146530	7	12345511	12468567	506	23	120000	-0.1681	0.56675	VWDE	76353	25kb
ENSG00000006747	7	12585203	12718228	572	61	120000	0.85337	0.19673	SCIN	76353	25kb
ENSG00000122644	7	12701481	12755559	250	38	120000	0.10371	0.4587	ARL4A	76353	25kb
ENSG00000006468	7	13905853	14056050	531	60	120000	-0.2152	0.58519	ETV1	76353	25kb
ENSG00000136267	7	14159674	15039402	2884	172	120000	-0.43432	0.66797	DGKB	76353	25kb
ENSG00000187546	7	15214943	15626640	1365	90	120000	-1.9372	0.97364	AGMO	76353	25kb
ENSG00000106511	7	15625837	15751437	456	37	120000	0.79158	0.2143	MEOX2	76353	25kb
ENSG00000214960	7	16105817	16485947	1158	48	120000	-0.30826	0.62106	ISPD	76353	25kb
ENSG00000171243	7	16476106	16595205	443	38	120000	-0.97417	0.83501	SOSTDC1	76353	25kb
ENSG00000205858	7	16541505	16646193	355	45	120000	-1.7259	0.95782	LRRC72	76353	25kb
ENSG00000106524	7	16614401	16710442	330	42	120000	-1.2021	0.88534	ANKMY2	76353	25kb
ENSG00000136261	7	16660756	16771148	423	55	120000	-0.70056	0.75821	BZW2	76353	25kb
ENSG00000106537	7	16768160	16849161	412	57	120000	1.0681	0.14274	TSPAN13	76353	25kb
ENSG00000267906	7	16803866	16854246	233	40	120000	0.76098	0.22333	AC073333.1	76353	25kb
ENSG00000106541	7	16806435	16898057	471	41	120000	0.67456	0.24998	AGR2	76353	25kb
ENSG00000173467	7	16874029	16946611	340	46	120000	-0.32609	0.62782	AGR3	76353	25kb
ENSG00000106546	7	17313246	17410776	116	25	120000	1.2886	0.098776	AHR	76353	25kb
ENSG00000071189	7	17805385	18005124	468	22	120000	-0.091055	0.53628	SNX13	76353	25kb
ENSG00000229937	7	18041405	18092486	137	20	120000	0.45472	0.32466	PRPS1L1	76353	25kb
ENSG00000048052	7	18101572	19067039	2461	181	120000	1.2617	0.10353	HDAC9	76353	25kb
ENSG00000122691	7	19035614	19182295	367	34	120000	-0.018329	0.50731	TWIST1	76353	25kb
ENSG00000146618	7	19159405	19210044	139	20	120000	1.1778	0.11944	FERD3L	76353	25kb
ENSG00000105849	7	19710085	19773710	252	18	120000	1.8557	0.031745	TWISTNB	76353	25kb
ENSG00000173452	7	19733933	19838221	389	22	120000	0.83999	0.20046	TMEM196	76353	25kb
ENSG00000183742	7	20149278	20282027	360	34	120000	0.83343	0.2023	MACC1	76353	25kb
ENSG00000105855	7	20345325	20480377	444	32	120000	-0.26926	0.60614	ITGB8	76353	25kb
ENSG00000004846	7	20629830	20841658	701	52	120000	-0.4552	0.67552	ABCB5	76353	25kb
ENSG00000164651	7	20798906	20851505	75	15	120000	-0.3922	0.65255	SP8	76353	25kb
ENSG00000105866	7	21442652	21579440	466	45	120000	-0.70775	0.76045	SP4	76353	25kb
ENSG00000105877	7	21557833	21966457	1766	125	120000	0.24409	0.40358	DNAH11	76353	25kb

ENSG00000164649	7	21915518	22010702	336	42	120000	0.24327	0.4039	CDCA7L	76353	25kb
ENSG00000136237	7	22132856	22421763	939	74	120000	0.4634	0.32154	RAPGEF5	76353	25kb
ENSG00000105889	7	22434063	22697544	762	64	120000	0.49464	0.31043	STEAP1B	76353	25kb
ENSG00000136244	7	22740503	22796621	253	24	120000	-0.99054	0.83904	IL6	76353	25kb
ENSG00000196683	7	22827251	22887470	271	22	120000	0.50696	0.30609	TOMM7	76353	25kb
ENSG00000122591	7	22955878	23078749	360	22	120000	-0.83157	0.79717	FAM126A	76353	25kb
ENSG00000122550	7	23120353	23242533	354	12	120000	-1.4868	0.93146	KLHL7	76353	25kb
ENSG00000214871	7	23185760	23259503	252	10	120000	-1.2761	0.89903	AC005082.1	76353	25kb
ENSG00000136243	7	23196446	23265630	200	11	120000	-1.0742	0.85863	NUPL2	76353	25kb
ENSG00000136235	7	23250586	23339727	154	18	120000	-0.32524	0.6275	GPNMB	76353	25kb
ENSG00000156928	7	23313358	23376348	109	12	120000	0.47865	0.3161	MALSU1	76353	25kb
ENSG00000136231	7	23324828	23535086	438	48	120000	-0.19813	0.57853	IGF2BP3	76353	25kb
ENSG00000164548	7	23519399	23596660	168	23	120000	-0.70404	0.7593	TRA2A	76353	25kb
ENSG00000169193	7	23611998	23709327	152	20	120000	-0.11169	0.54446	CCDC126	76353	25kb
ENSG00000188732	7	23694749	23767868	215	30	120000	-0.38583	0.65019	FAM221A	76353	25kb
ENSG00000196335	7	23724786	23897132	568	29	120000	-1.1305	0.87086	STK31	76353	25kb
ENSG00000122585	7	24298782	24356484	246	30	120000	-0.098047	0.53905	NPY	76353	25kb
ENSG00000105926	7	24587887	24758812	536	14	120000	0.27028	0.39347	MPP6	76353	25kb
ENSG00000105928	7	24712972	24834244	389	28	120000	0.25412	0.3997	DFNA5	76353	25kb
ENSG00000070882	7	24811158	25046253	711	83	120000	-1.3201	0.9066	OSBPL3	76353	25kb
ENSG00000172115	7	25134710	25189980	174	12	120000	-0.31112	0.62214	CYCS	76353	25kb
ENSG00000153790	7	25149316	25244975	338	23	120000	-0.62216	0.73308	C7orf31	76353	25kb
ENSG00000105954	7	25239189	25293105	218	32	120000	-1.6352	0.94899	NPVF	76353	25kb
ENSG00000050344	7	26166860	26251745	199	26	120000	-0.21836	0.58643	NFE2L3	76353	25kb
ENSG00000122566	7	26204547	26266149	142	15	120000	-0.41847	0.6622	HNRNPA2B1	76353	25kb
ENSG00000122565	7	26215782	26277976	133	13	120000	-0.54623	0.70754	CBX3	76353	25kb
ENSG00000086300	7	26306541	26438949	394	41	120000	-1.6721	0.95275	SNX10	76353	25kb
ENSG00000122548	7	26547740	26603407	278	23	120000	-1.3388	0.90968	KIAA0087	76353	25kb
ENSG00000222004	7	26652490	26711889	179	24	120000	1.7361	0.041277	C7orf71	76353	25kb
ENSG00000005020	7	26681681	27059858	886	24	120000	0.63483	0.26277	SKAP2	76353	25kb
ENSG00000105991	7	27107612	27160615	97	20	120000	-0.091269	0.53636	HOXA1	76353	25kb
ENSG00000105996	7	27114721	27167430	106	22	120000	0.083039	0.46691	HOXA2	76353	25kb
ENSG00000105997	7	27120803	27217200	195	28	120000	0.11698	0.45344	HOXA3	76353	25kb
ENSG00000197576	7	27143126	27195418	118	16	120000	-0.15574	0.56188	HOXA4	76353	25kb
ENSG00000106004	7	27155671	27208287	123	18	120000	-0.018422	0.50735	HOXA5	76353	25kb
ENSG00000106006	7	27160015	27215222	121	19	120000	-0.04877	0.51945	HOXA6	76353	25kb
ENSG00000122592	7	27168335	27222555	108	20	120000	0.050562	0.47984	HOXA7	76353	25kb
ENSG00000078399	7	27177054	27235117	115	25	120000	0.10737	0.45725	HOXA9	76353	25kb
ENSG00000257184	7	27178154	27244632	135	29	120000	0.10351	0.45878	RP1-170O19.20	76353	25kb
ENSG00000253293	7	27185210	27244880	119	27	120000	0.11095	0.45583	HOXA10	76353	25kb
ENSG00000005073	7	27196129	27249842	103	26	120000	0.16416	0.4348	HOXA11	76353	25kb
ENSG00000106031	7	27208122	27264725	111	24	120000	-0.46965	0.6807	HOXA13	76353	25kb
ENSG00000106038	7	27257164	27315112	130	16	120000	-1.4095	0.92065	EVX1	76353	25kb
ENSG00000106049	7	27540061	27727614	566	22	120000	0.2245	0.41118	HIBADH	76353	25kb
ENSG00000106052	7	27753950	27909183	399	31	120000	-0.81244	0.79173	TAX1BP1	76353	25kb
ENSG00000153814	7	27845192	28245362	1042	88	120000	-0.67106	0.74891	JAZF1	76353	25kb
ENSG00000146592	7	28313940	28890511	1778	170	120000	-0.81596	0.79274	CREB5	76353	25kb
ENSG00000106066	7	29009847	29260067	903	89	120000	0.24124	0.40469	CPVL	76353	25kb
ENSG00000106069	7	29136890	29578944	1489	141	120000	0.10433	0.45845	CHN2	76353	25kb
ENSG00000176532	7	29578427	29631911	199	25	120000	1.1879	0.11744	PRR15	76353	25kb
ENSG00000122574	7	29821102	29981682	584	46	120000	0.064503	0.47428	WIPF3	76353	25kb
ENSG00000136193	7	29934719	30054905	314	26	120000	-0.085842	0.5342	SCRN1	76353	25kb
ENSG00000106080	7	30025203	30091300	113	12	120000	-0.61266	0.72995	FKBP14	76353	25kb
ENSG00000106086	7	30042020	30195096	356	27	120000	-0.63556	0.73747	PLEKHA8	76353	25kb
ENSG00000180354	7	30149426	30227378	235	37	120000	-0.36055	0.64078	MTURN	76353	25kb
ENSG00000180233	7	30298923	30477118	288	36	120000	-0.41899	0.66239	ZNRF2	76353	25kb
ENSG00000106100	7	30439143	30543400	226	32	120000	-1.3782	0.91593	NOD1	76353	25kb
ENSG00000006625	7	30511237	30616095	276	26	120000	-1.1654	0.87806	GGCT	76353	25kb
ENSG00000106105	7	30609297	30698649	218	25	120000	-1.1185	0.86833	GARS	76353	25kb
ENSG00000106113	7	30667200	30764745	261	47	120000	-1.4312	0.92381	CRHR2	76353	25kb
ENSG00000241644	7	30712601	30822218	363	59	120000	-0.2457	0.59704	INMT	76353	25kb
ENSG00000254959	7	30766753	30956696	673	49	120000	-0.73767	0.76964	INMT-FAM188B	76353	25kb
ENSG00000106125	7	30786033	30957002	619	42	120000	-0.99764	0.84077	FAM188B	76353	25kb
ENSG00000250424	7	30868010	30988427	417	54	120000	-1.3372	0.90942	AQP1	76353	25kb
ENSG00000240583	7	30868010	30990131	423	54	120000	-1.3401	0.90989	AQP1	76353	25kb
ENSG00000106128	7	30953284	31057869	383	53	120000	-0.82075	0.7941	GHRHR	76353	25kb
ENSG00000078549	7	31067076	31176089	327	58	120000	-1.2722	0.89835	ADCYAP1R1	76353	25kb
ENSG00000164600	7	31352075	31405508	98	23	120000	0.052601	0.47903	NEUROD6	76353	25kb
ENSG00000180347	7	31528704	31723334	554	33	120000	-1.2494	0.89424	CCDC129	76353	25kb
ENSG00000106341	7	31701329	31773069	190	31	120000	-1.4653	0.92859	PPP1R17	76353	25kb
ENSG00000154678	7	31765793	32363941	2170	136	120000	-2.12	0.983	PDE1C	76353	25kb
ENSG00000106355	7	32499951	32559895	224	21	120000	0.083436	0.46675	LSM5	76353	25kb
ENSG00000105778	7	32510038	33103516	1779	42	120000	-0.34976	0.63674	AVL9	76353	25kb
ENSG00000170852	7	32882784	32958743	145	19	120000	-1.0493	0.85298	KBTBD2	76353	25kb
ENSG00000122642	7	32972017	33071543	265	30	120000	-1.313	0.9054	FKBP9	76353	25kb
ENSG00000122643	7	33028742	33127409	307	16	120000	-0.91834	0.82078	NT5C3A	76353	25kb
ENSG00000164610	7	33109409	33174013	184	23	120000	-0.80765	0.79035	RP9	76353	25kb
ENSG00000122507	7	33143856	33670680	1527	56	120000	0.62293	0.26667	BBS9	76353	25kb
ENSG00000173862	7	33740593	33793068	135	20	120000	-0.05695	0.52271	RP11-89N17.1	76353	25kb
ENSG00000164619	7	33919523	34220484	654	68	120000	0.38949	0.34846	BMPER	76353	25kb
ENSG00000214841	7	34660709	34724772	349	26	120000	2.8746	0.002023	AC005493.1	76353	25kb
ENSG00000187258	7	34672851	34942944	961	58	120000	1.4221	0.077494	NPSR1	76353	25kb
ENSG00000173852	7	34943488	35102883	470	23	120000	-0.55725	0.71132	DPY19L1	76353	25kb
ENSG00000164532	7	35217042	35318758	263	12	120000	-0.6009	0.72605	TBX20	76353	25kb
ENSG00000122557	7	35647269	35760181	250	24	120000	0.38804	0.34899	HERPUD2	76353	25kb
ENSG00000122545	7	35815542	35969917	271	14	120000	-1.1001	0.86437	SEPT7	76353	25kb
ENSG00000205745	7	36093694	36150346	253	25	120000	-1.4662	0.9287	PP13004	76353	25kb
ENSG00000122547	7	36167758	36366152	768	72	120000	0.22182	0.41223	EEPD1	76353	25kb
ENSG00000164542	7	36338830	36454734	389	42	120000	0.93108	0.17591	KIAA0895	76353	25kb
ENSG00000011426	7	36404415	36518400	365	38	120000	0.99351	0.16023	ANLN	76353	25kb
ENSG00000136250	7	36527456	36789154	780	83	120000	-0.33591	0.63153	AOAH	76353	25kb
ENSG00000155849	7	36868961	37513852	1944	147	120000	0.463	0.32168	ELMO1	76353	25kb
ENSG00000187037	7	37698399	37898390	600	35	120000	-1.9782	0.97605	GPR141	76353	25kb
ENSG00000086289	7	37698446	38016543	1151	72	120000	-1.5874	0.94379	EPDR1	76353	25kb
ENSG00000086288	7	37863199	37965003	512	38	120000	-1.0445	0.85188	NME8	76353	25kb
ENSG00000106483	7	37920543	38090297	716	64	120000	-0.41138	0.6596	SFRP4	76353	25kb

ENSG00000010270	7	38192824	38295272	312	24	120000	0.78281	0.21687	STARD3NL	76353	25kb
ENSG00000078053	7	38398305	38696167	1288	78	120000	-0.57225	0.71642	AMPH	76353	25kb
ENSG00000164556	7	38699946	38751637	195	36	120000	-0.1789	0.57099	FAM183B	76353	25kb
ENSG00000006715	7	38737563	38996994	885	40	120000	1.0901	0.13784	VPS41	76353	25kb
ENSG00000106536	7	38992598	39557694	1623	72	120000	0.022296	0.49111	POU6F2	76353	25kb
ENSG00000241127	7	39580975	39674919	276	30	120000	1.5497	0.060606	YAE1D1	76353	25kb
ENSG00000006451	7	39638082	39772723	296	29	120000	2.4352	0.0074414	RALA	76353	25kb
ENSG00000065883	7	39964636	40161733	353	22	120000	0.89019	0.18668	CDK13	76353	25kb
ENSG00000168303	7	40140622	40199258	80	14	120000	0.72033	0.23566	MPLKIP	76353	25kb
ENSG00000175600	7	40149575	40925362	1401	89	120000	0.61219	0.27021	SUGCT	76353	25kb
ENSG00000122641	7	41699712	41767706	104	21	120000	-1.1626	0.8775	INHBA	76353	25kb
ENSG00000106571	7	41975548	42302469	1001	94	120000	2.6916	0.003556	GLI3	76353	25kb
ENSG00000136197	7	42923325	42976904	138	10	120000	-0.041427	0.51652	C7orf25	76353	25kb
ENSG00000256646	7	42923872	42996773	200	15	120000	1.0112	0.15596	PSMA2	76353	25kb
ENSG00000106588	7	42931460	42996822	191	14	120000	1.0155	0.15493	PSMA2	76353	25kb
ENSG00000106591	7	42946799	43013557	182	17	120000	1.6826	0.046222	MRPL32	76353	25kb
ENSG00000002746	7	43127198	43630600	1461	121	120000	0.88678	0.1876	HECW1	76353	25kb
ENSG00000164543	7	43597357	43691385	263	14	120000	1.1489	0.12531	STK17A	76353	25kb
ENSG00000106603	7	43623055	43794316	490	18	120000	1.1039	0.13483	COA1	76353	25kb
ENSG00000106605	7	43773279	43871939	222	23	120000	0.71249	0.23808	BLVRA	76353	25kb
ENSG00000062582	7	43881157	43934492	115	21	120000	-0.77423	0.7806	MRPS24	76353	25kb
ENSG00000270617	7	43881157	43971188	230	28	120000	-1.0347	0.8496	URGCP-MRPS24	76353	25kb
ENSG00000106608	7	43890493	43991010	272	21	120000	-1.1032	0.86502	URGCP	76353	25kb
ENSG00000078967	7	43941037	44020735	207	22	120000	-1.1847	0.88193	UBE2D4	76353	25kb
ENSG00000136206	7	44015488	44074721	40	9	120000	-1.0606	0.85556	SPDYE1	76353	25kb
ENSG00000136279	7	44059239	44134055	161	14	120000	0.18053	0.42837	DBNL	76353	25kb
ENSG00000164708	7	44077326	44130186	136	11	120000	0.23782	0.40601	PGAM2	76353	25kb
ENSG00000122678	7	44086846	44147139	159	17	120000	0.64129	0.26067	POLM	76353	25kb
ENSG00000106624	7	44118960	44179161	153	23	120000	0.86269	0.19415	AEBP1	76353	25kb
ENSG00000106628	7	44129286	44188957	155	23	120000	0.50336	0.30736	POLD2	76353	25kb
ENSG00000106631	7	44153463	44205931	140	23	120000	-0.084407	0.53363	MYL7	76353	25kb
ENSG00000106633	7	44158872	44262769	296	37	120000	-0.05326	0.52124	GCK	76353	25kb
ENSG00000106636	7	44215567	44278893	194	27	120000	0.14219	0.44347	YKT6	76353	25kb
ENSG00000058404	7	44231749	44399176	436	46	120000	0.96872	0.16634	CAMK2B	76353	25kb
ENSG00000015676	7	44393720	44555479	335	10	120000	0.60271	0.27335	NUDCD3	76353	25kb
ENSG00000015520	7	44527134	44605914	175	24	120000	-0.061765	0.52462	NPC1L1	76353	25kb
ENSG00000136271	7	44580016	44639650	171	20	120000	-0.87116	0.80817	DDX56	76353	25kb
ENSG00000158604	7	44592493	44646886	160	18	120000	-0.73014	0.76735	TMED4	76353	25kb
ENSG00000105953	7	44621171	44773665	342	31	120000	-0.10169	0.5405	OGDH	76353	25kb
ENSG00000122515	7	44763180	44834477	147	26	120000	0.78591	0.21596	ZMIZ2	76353	25kb
ENSG00000196262	7	44811279	44889163	206	22	120000	0.94202	0.17309	PPIA	76353	25kb
ENSG00000105968	7	44841390	44912682	177	19	120000	0.9316	0.17577	H2AFV	76353	25kb
ENSG00000146676	7	44890896	44949960	114	18	120000	0.95398	0.17005	PURB	76353	25kb
ENSG00000136286	7	44977261	45043697	164	28	120000	1.8276	0.033804	MYO1G	76353	25kb
ENSG00000136280	7	45014074	45141068	422	23	120000	1.0949	0.13678	CCM2	76353	25kb
ENSG00000136274	7	45095037	45153513	193	21	120000	0.45382	0.32498	NACAD	76353	25kb
ENSG00000136270	7	45114699	45176646	217	28	120000	-0.032963	0.51315	TBRG4	76353	25kb
ENSG00000122679	7	45172390	45250901	411	30	120000	2.1382	0.016251	RAMP3	76353	25kb
ENSG00000164742	7	45588739	45787715	585	54	120000	-0.58073	0.71929	ADCY1	76353	25kb
ENSG00000267988	7	45877684	45931045	174	30	120000	-0.87702	0.80976	AC096582.1	76353	25kb
ENSG00000146678	7	45902956	45958267	164	26	120000	-0.80525	0.78966	IGFBP1	76353	25kb
ENSG00000146674	7	45926949	45986473	135	23	120000	-0.66317	0.74639	IGFBP3	76353	25kb
ENSG00000233539	7	46688383	46824449	501	52	120000	-0.35711	0.63949	AC011294.3	76353	25kb
ENSG00000136205	7	47289752	47647156	980	109	120000	-1.5462	0.93897	TNS3	76353	25kb
ENSG00000221845	7	47669842	47726246	210	32	120000	0.15163	0.43974	C7orf65	76353	25kb
ENSG00000136273	7	47710328	48044178	1212	81	120000	0.4856	0.31363	HUS1	76353	25kb
ENSG00000158683	7	47789250	48013088	760	56	120000	-0.29681	0.61669	PKD1L1	76353	25kb
ENSG00000136275	7	47809889	47884445	247	33	120000	-1.2744	0.89873	C7orf69	76353	25kb
ENSG00000164744	7	48001745	48093716	220	17	120000	-0.76546	0.778	SUN3	76353	25kb
ENSG00000164746	7	48050108	48125901	157	17	120000	-0.53011	0.70198	C7orf57	76353	25kb
ENSG00000183696	7	48103225	48173330	119	17	120000	0.087968	0.46495	UPP1	76353	25kb
ENSG00000179869	7	48186055	48712092	1946	76	120000	-1.0199	0.84611	ABCA13	76353	25kb
ENSG00000267970	7	48862362	48916221	156	27	120000	1.2321	0.10896	AC004899.1	76353	25kb
ENSG00000188730	7	49788257	49986546	423	44	120000	-0.32476	0.62732	VWC2	76353	25kb
ENSG00000042813	7	49865017	50185925	802	23	120000	0.16499	0.43448	ZPBP	76353	25kb
ENSG00000164500	7	50110632	50224426	362	19	120000	-0.013013	0.50519	C7orf72	76353	25kb
ENSG00000185811	7	50318720	50497799	417	60	120000	0.18119	0.42811	IKZF1	76353	25kb
ENSG00000132436	7	50486831	50543088	196	21	120000	-0.35367	0.63821	FIGNL1	76353	25kb
ENSG00000132437	7	50501134	50658154	632	29	120000	-0.092589	0.53688	DDC	76353	25kb
ENSG00000106070	7	50632760	50886159	827	38	120000	-0.24212	0.59566	GRB10	76353	25kb
ENSG00000106078	7	51058909	51409515	733	53	120000	-1.5779	0.94271	COBL	76353	25kb
ENSG00000221900	7	53078349	53129617	147	25	120000	-0.01123	0.50448	POM121L12	76353	25kb
ENSG00000170419	7	54585018	54663773	278	23	120000	1.6417	0.050323	VSTM2A	76353	25kb
ENSG00000132432	7	54794943	54852667	255	14	120000	-0.83541	0.79826	SEC61G	76353	25kb
ENSG00000146648	7	55061714	55349313	893	98	120000	-1.3705	0.91473	EGFR	76353	25kb
ENSG00000132434	7	55408141	55526435	349	22	120000	-1.4778	0.93026	LANCL2	76353	25kb
ENSG00000154978	7	55478749	55665681	789	23	120000	0.3403	0.36682	VOPP1	76353	25kb
ENSG00000154997	7	55836237	55955482	357	32	120000	1.2533	0.10505	SEPT14	76353	25kb
ENSG00000249773	7	55929970	56047932	316	28	120000	0.78611	0.2159	MRPS17	76353	25kb
ENSG00000178665	7	55930169	56034918	274	25	120000	0.93092	0.17595	ZNF713	76353	25kb
ENSG00000146729	7	55994486	56092874	264	23	120000	0.30251	0.38113	GBAS	76353	25kb
ENSG00000239789	7	55994512	56049192	158	24	120000	0.3824	0.35108	MRPS17	76353	25kb
ENSG00000146733	7	56053744	56144297	218	27	120000	-0.12943	0.55149	PSPH	76353	25kb
ENSG00000146731	7	56094323	56156682	164	23	120000	-0.49279	0.68892	CCT6A	76353	25kb
ENSG00000129103	7	56106695	56173363	185	23	120000	-0.80339	0.78913	SUMF2	76353	25kb
ENSG00000164776	7	56123440	56185689	196	23	120000	-0.87509	0.80924	PHKG1	76353	25kb
ENSG00000106153	7	56144262	56199269	196	26	120000	-0.49606	0.69007	CHCHD2	76353	25kb
ENSG00000185290	7	56157374	56209093	176	26	120000	-0.38843	0.65115	NUPR1L	76353	25kb
ENSG00000185177	7	57162321	57232571	213	16	120000	-1.1458	0.87405	ZNF479	76353	25kb
ENSG00000182111	7	57484883	57558252	230	12	120000	1.1383	0.12749	ZNF716	76353	25kb
ENSG00000230000	7	62784239	62837151	31	6	120000	0.1121	0.45537	AC006455.1	76353	25kb
ENSG00000268181	7	62833414	62883860	22	4	120000	-0.12164	0.54841	AC073188.1	76353	25kb
ENSG00000257482	7	63480821	63563927	302	11	120000	1.3274	0.092186	ZNF727	76353	25kb
ENSG00000197123	7	63663852	63752309	380	26	120000	-0.66658	0.74748	ZNF679	76353	25kb
ENSG00000234444	7	63742837	63840238	501	32	120000	-0.497	0.6904	ZNF736	76353	25kb
ENSG00000173041	7	63955262	64048484	309	15	120000	-1.2165	0.88811	ZNF680	76353	25kb

ENSG00000196247	7	64101511	64196404	250	24	120000	-1.4241	0.92279	ZNF107	76353	25kb
ENSG00000197008	7	64229766	64319054	317	20	120000	-0.96387	0.83244	ZNF138	76353	25kb
ENSG00000198039	7	64305550	64416344	409	17	120000	0.05561	0.47783	ZNF273	76353	25kb
ENSG00000152926	7	64407154	64492062	284	15	120000	1.0118	0.15581	ZNF117	76353	25kb
ENSG00000213462	7	64426187	64492121	215	16	120000	0.995	0.15987	ERV3-1	76353	25kb
ENSG00000146757	7	64813712	64891038	241	17	120000	0.49487	0.31035	ZNF92	76353	25kb
ENSG00000269558	7	65087081	65138280	15	5	120000	0.75243	0.2259	AC104057.1	76353	25kb
ENSG00000196715	7	65313254	65449550	270	16	120000	1.8836	0.029811	VKORC1L1	76353	25kb
ENSG00000169919	7	65400671	65472301	141	14	120000	1.575	0.057626	GUSB	76353	25kb
ENSG00000126522	7	65515785	65583545	161	16	120000	1.295	0.097667	ASL	76353	25kb
ENSG00000249319	7	65527756	65642228	285	15	120000	1.4451	0.074222	AC068533.7	76353	25kb
ENSG00000241258	7	65554591	65644555	237	19	120000	1.4601	0.072134	CRCP	76353	25kb
ENSG00000169902	7	65645186	65910530	515	19	120000	1.1673	0.12155	TPST1	76353	25kb
ENSG00000267941	7	65914543	65965709	128	14	120000	0.96624	0.16696	AC008267.1	76353	25kb
ENSG00000243335	7	66068868	66301446	742	23	120000	1.1244	0.13042	KCTD7	76353	25kb
ENSG00000154710	7	66122151	66301451	581	19	120000	1.147	0.12568	RABGEF1	76353	25kb
ENSG00000106609	7	66361212	66448538	212	20	120000	1.1208	0.13118	TMEM248	76353	25kb
ENSG00000126524	7	66427664	66485588	168	36	120000	1.3416	0.08987	SBDS	76353	25kb
ENSG00000198874	7	66435160	66729501	1196	40	120000	0.74944	0.2268	TYW1	76353	25kb
ENSG00000158321	7	69038905	70283054	2589	92	120000	0.24468	0.40335	AUTS2	76353	25kb
ENSG00000185274	7	70572155	71203585	2261	143	120000	-0.27134	0.60693	WBSCR17	76353	25kb
ENSG00000183166	7	71219476	71937136	2124	79	120000	-1.5182	0.93552	CALN1	76353	25kb
ENSG00000196313	7	72324936	72446979	51	21	120000	0.28541	0.38766	POM121	76353	25kb
ENSG00000155428	7	72405016	72464997	7	4	120000	-0.97277	0.83467	TRIM74	76353	25kb
ENSG00000267985	7	72434478	72489996	1	1	120000	0.043496	0.48265	AC005488.1	76353	25kb
ENSG00000130305	7	72691514	72747864	35	12	120000	-1.5877	0.94382	NSUN5	76353	25kb
ENSG00000146755	7	72701535	72767085	83	10	120000	-1.3841	0.91684	TRIM50	76353	25kb
ENSG00000077800	7	72717167	72797634	149	12	120000	-1.3263	0.90764	FKBP6	76353	25kb
ENSG00000188763	7	72823109	72875450	67	13	120000	-0.73829	0.76983	FZD9	76353	25kb
ENSG00000009954	7	72829728	72961608	202	19	120000	-0.033366	0.51331	BAZ1B	76353	25kb
ENSG00000106635	7	72925686	72997332	132	16	120000	0.4871	0.31309	BCL7B	76353	25kb
ENSG00000106638	7	72958262	73018121	111	15	120000	0.83167	0.2028	TBL2	76353	25kb
ENSG00000009950	7	72982524	73063873	197	22	120000	0.63553	0.26254	MLXIPL	76353	25kb
ENSG00000176428	7	73057155	73111442	180	17	120000	0.65585	0.25596	VPS37D	76353	25kb
ENSG00000176410	7	73070299	73122783	158	14	120000	0.23399	0.4075	DNAJC30	76353	25kb
ENSG00000071462	7	73072355	73144491	196	20	120000	0.40466	0.34286	WBSCR22	76353	25kb
ENSG00000106089	7	73088536	73159002	177	26	120000	-0.21307	0.58436	STX1A	76353	25kb
ENSG00000106077	7	73125424	73178197	89	24	120000	-0.48759	0.68708	ABHD11	76353	25kb
ENSG00000165215	7	73158328	73209600	83	14	120000	-0.60213	0.72646	CLDN3	76353	25kb
ENSG00000189143	7	73188872	73272014	165	10	120000	-0.72629	0.76617	CLDN4	76353	25kb
ENSG00000165171	7	73223920	73281865	129	6	120000	-0.74393	0.77154	WBSCR27	76353	25kb
ENSG00000175877	7	73250489	73305223	106	11	120000	-0.48517	0.68622	WBSCR28	76353	25kb
ENSG00000049540	7	73417119	73509237	192	32	120000	-0.093718	0.53733	ELN	76353	25kb
ENSG00000106683	7	73472263	73561855	195	24	120000	1.2288	0.10958	LIMK1	76353	25kb
ENSG00000106682	7	73563575	73636431	101	14	120000	1.1324	0.12874	EIF4H	76353	25kb
ENSG00000086730	7	73588982	73669161	93	19	120000	0.42603	0.33504	LAT2	76353	25kb
ENSG00000049541	7	73620829	73693774	113	18	120000	0.52021	0.30146	RFC2	76353	25kb
ENSG00000106665	7	73678805	73845273	387	31	120000	0.64842	0.25836	CLIP2	76353	25kb
ENSG00000006704	7	73843120	74041931	389	51	120000	-0.7976	0.78745	GTF2IRD1	76353	25kb
ENSG00000077809	7	74046994	74200026	153	22	120000	1.1413	0.12687	GTF2I	76353	25kb
ENSG00000158517	7	74163309	74228659	3	1	120000	1.1498	0.12512	NCF1	76353	25kb
ENSG00000196275	7	74185483	74292847	9	3	120000	1.7081	0.043812	GTF2IRD2	76353	25kb
ENSG00000183086	7	74354083	74463803	9	4	120000	0.98312	0.16277	GATSL1	76353	25kb
ENSG00000174374	7	74416226	74515064	26	7	120000	0.33182	0.37001	WBSCR16	76353	25kb
ENSG00000174428	7	74483364	74590623	17	6	120000	0.71768	0.23648	GTF2IRD2B	76353	25kb
ENSG00000198750	7	74782499	74892509	4	2	120000	-0.16535	0.56566	GATSL2	76353	25kb
ENSG00000268891	7	74974908	75030425	1	1	120000	0.71102	0.23853	AC006014.1	76353	25kb
ENSG00000178809	7	74999337	75065279	47	12	120000	-0.21625	0.58561	TRIM73	76353	25kb
ENSG00000135213	7	75021066	75140548	113	20	120000	0.57926	0.28121	POM121C	76353	25kb
ENSG00000127946	7	75137621	75393280	832	80	120000	1.2428	0.10698	HIP1	76353	25kb
ENSG00000006606	7	75373851	75444214	387	39	120000	0.099765	0.46027	CCL26	76353	25kb
ENSG00000106178	7	75415983	75477674	309	30	120000	0.32851	0.37126	CCL24	76353	25kb
ENSG00000005486	7	75446920	75543244	221	34	120000	-0.67083	0.74884	RHBDD2	76353	25kb
ENSG00000127948	7	75503518	75641173	249	45	120000	-0.31548	0.6238	POR	76353	25kb
ENSG00000127952	7	75600656	75702322	240	25	120000	-0.099646	0.53969	STYXL1	76353	25kb
ENSG00000146701	7	75652369	75721826	170	16	120000	0.20032	0.42062	MDH2	76353	25kb
ENSG00000177679	7	75806216	75941605	261	24	120000	-0.57393	0.71699	SRRM3	76353	25kb
ENSG00000106211	7	75906861	75958612	117	20	120000	-0.61115	0.72945	HSPB1	76353	25kb
ENSG00000170027	7	75931116	76013348	217	25	120000	-0.34057	0.63329	YWHAG	76353	25kb
ENSG00000146700	7	75993651	76064012	255	20	120000	-0.98477	0.83763	SRCRB4D	76353	25kb
ENSG00000188372	7	76001835	76096388	245	27	120000	-1.0419	0.85126	ZP3	76353	25kb
ENSG00000091073	7	76065993	76160312	71	8	120000	-0.61607	0.73108	DTX2	76353	25kb
ENSG00000243566	7	76114745	76673340	831	71	120000	-1.2114	0.88712	UPK3B	76353	25kb
ENSG00000146707	7	76214303	76281578	64	10	120000	-0.71448	0.76254	POMZP3	76353	25kb
ENSG00000135205	7	76726751	76983850	725	38	120000	0.39024	0.34818	CCDC146	76353	25kb
ENSG00000127951	7	76797688	76854143	107	17	120000	-0.018987	0.50757	FGL2	76353	25kb
ENSG00000186088	7	76915068	77070717	550	36	120000	1.1948	0.11607	GSAP	76353	25kb
ENSG00000127947	7	77141592	77294388	413	22	120000	-1.0782	0.85953	PTPN12	76353	25kb
ENSG00000187257	7	77300760	77437339	351	22	120000	-0.83634	0.79852	RSBN1L	76353	25kb
ENSG00000135211	7	77398045	77452897	143	20	120000	-0.46632	0.67951	TMEM60	76353	25kb
ENSG00000006576	7	77403122	77611818	518	28	120000	-0.42563	0.66481	PHTF2	76353	25kb
ENSG00000187391	7	77621393	79107890	5462	276	120000	1.5366	0.062197	MAGI2	76353	25kb
ENSG00000127955	7	79738271	79873718	339	45	120000	0.20438	0.41903	GNAI1	76353	25kb
ENSG00000135218	7	79973891	80333593	990	68	120000	-0.10138	0.54038	CD36	76353	25kb
ENSG00000214415	7	80062987	80166336	282	32	120000	0.91788	0.17934	GNAT3	76353	25kb
ENSG00000075223	7	80346854	80576675	520	59	120000	0.67631	0.24942	SEMA3C	76353	25kb
ENSG00000237896	7	80779824	80853431	295	30	120000	-0.38144	0.64856	AC005008.2	76353	25kb
ENSG00000019991	7	81303322	81424754	255	23	120000	-0.21035	0.5833	HGF	76353	25kb
ENSG00000153956	7	81550760	82098114	1655	117	120000	1.1593	0.12317	CACNA2D1	76353	25kb
ENSG00000186472	7	82358329	82817246	1285	64	120000	0.41065	0.34066	PCLO	76353	25kb
ENSG00000170381	7	82968222	83303326	1069	47	120000	1.8984	0.028821	SEMA3E	76353	25kb
ENSG00000075213	7	83560093	84147040	1751	86	120000	-0.31689	0.62434	SEMA3A	76353	25kb
ENSG00000153993	7	84599869	84841171	399	30	120000	-0.83732	0.79879	SEMA3D	76353	25kb
ENSG00000198822	7	86248230	86519200	530	39	120000	0.27295	0.39245	GRM3	76353	25kb
ENSG00000164659	7	86481222	86714015	554	27	120000	-0.32486	0.62735	KIAA1324L	76353	25kb
ENSG00000135164	7	86756677	86850653	258	14	120000	-0.67673	0.75071	DMTF1	76353	25kb

ENSG00000135185	7	86800478	86874903	199	16	120000	-0.7609	0.77664	TMEM243	76353	25kb
ENSG00000005469	7	86949997	87054111	254	20	120000	0.71616	0.23695	CROT	76353	25kb
ENSG00000005471	7	87006013	87134751	292	18	120000	0.77134	0.22025	ABCB4	76353	25kb
ENSG00000085563	7	87108175	87367611	529	43	120000	1.0477	0.14739	ABCB1	76353	25kb
ENSG00000105784	7	87231864	87486611	369	17	120000	0.032056	0.48721	RUNDC3B	76353	25kb
ENSG00000075303	7	87437883	87530672	166	15	120000	-0.50823	0.69436	SLC25A40	76353	25kb
ENSG00000006634	7	87480531	87563856	153	17	120000	-0.55455	0.7104	DBF4	76353	25kb
ENSG00000008277	7	87538458	87857204	507	35	120000	0.76458	0.22226	ADAM22	76353	25kb
ENSG00000075142	7	87809433	87881308	110	20	120000	0.39029	0.34816	SRI	76353	25kb
ENSG00000127954	7	87880744	87961206	128	10	120000	0.42385	0.33584	STEAP4	76353	25kb
ENSG00000182348	7	88363682	88991346	2046	89	120000	-0.30827	0.62106	ZNF804B	76353	25kb
ENSG00000164645	7	88398420	88450035	184	16	120000	-0.063323	0.52525	C7orf62	76353	25kb
ENSG00000164647	7	89758689	89819143	155	14	120000	-0.37586	0.64649	STEAP1	76353	25kb
ENSG00000157214	7	89771904	89892451	337	22	120000	-0.65358	0.74331	STEAP2	76353	25kb
ENSG00000105792	7	89849488	89965377	298	21	120000	0.0176	0.49298	C7orf63	76353	25kb
ENSG00000105793	7	89939537	90045769	297	20	120000	0.033473	0.48665	GTPBP10	76353	25kb
ENSG00000157224	7	89988035	90167716	549	35	120000	-1.0271	0.84781	CLDN12	76353	25kb
ENSG00000058091	7	90070738	90864905	2341	83	120000	0.77794	0.2183	CDK14	76353	25kb
ENSG00000157240	7	90868783	90923123	118	18	120000	-1.3007	0.90332	FZD1	76353	25kb
ENSG00000127989	7	91296323	91535034	432	21	120000	0.64812	0.25846	MTERF	76353	25kb
ENSG00000127914	7	91545181	91764987	385	9	120000	0.073411	0.47074	AKAP9	76353	25kb
ENSG00000001630	7	91716465	91797266	133	7	120000	-0.44488	0.6718	CYP51A1	76353	25kb
ENSG00000240720	7	91716473	91833845	168	10	120000	-0.63219	0.73637	LRRD1	76353	25kb
ENSG00000001631	7	91803283	91900480	97	14	120000	-1.6518	0.95071	KRIT1	76353	25kb
ENSG00000001629	7	91850548	92055698	218	26	120000	-1.5807	0.94303	ANKIB1	76353	25kb
ENSG00000157259	7	92051767	92113150	135	21	120000	-1.0936	0.86293	GATAD1	76353	25kb
ENSG00000242950	7	92072694	92132300	117	17	120000	-0.70697	0.76021	ERVW-1	76353	25kb
ENSG00000127980	7	92091334	92182845	182	18	120000	-0.59276	0.72333	PEX1	76353	25kb
ENSG00000127993	7	92133087	92192319	107	12	120000	-0.46392	0.67865	RBM48	76353	25kb
ENSG00000234545	7	92165107	92244708	158	19	120000	-0.20657	0.58183	FAM133B	76353	25kb
ENSG00000105810	7	92209235	92490908	362	39	120000	-0.88897	0.81299	CDK6	76353	25kb
ENSG00000205413	7	92703829	92772336	222	29	120000	-0.022766	0.50908	SAMD9	76353	25kb
ENSG00000177409	7	92734368	92802682	175	29	120000	-1.0577	0.85491	SAMD9L	76353	25kb
ENSG00000188175	7	92792899	92880837	165	26	120000	-1.2275	0.89018	HEPACAM2	76353	25kb
ENSG00000004766	7	92836653	93013338	272	34	120000	-0.90252	0.81661	CCDC132	76353	25kb
ENSG00000004948	7	93028799	93229042	511	36	120000	-0.95932	0.8313	CALCR	76353	25kb
ENSG00000127928	7	93195885	93565577	1018	65	120000	-1.3325	0.90865	GNGT1	76353	25kb
ENSG00000105825	7	93489709	93545303	211	23	120000	-0.87569	0.8094	TFPI2	76353	25kb
ENSG00000127920	7	93526011	93582922	177	27	120000	-0.31782	0.62469	GNG11	76353	25kb
ENSG00000105829	7	93567074	93658694	160	31	120000	1.0471	0.14753	BET1	76353	25kb
ENSG00000164692	7	93998873	94085544	229	40	120000	0.015638	0.49376	COL1A2	76353	25kb
ENSG00000127995	7	94113531	94211331	189	19	120000	0.46093	0.32242	CASD1	76353	25kb
ENSG00000127990	7	94189542	94310521	171	18	120000	0.25	0.4013	SGCE	76353	25kb
ENSG00000242265	7	94260637	94324007	114	16	120000	-0.045341	0.51808	PEG10	76353	25kb
ENSG00000158528	7	94511514	94950727	772	38	120000	0.36908	0.35603	PPP1R9A	76353	25kb
ENSG00000005421	7	94901988	95050673	293	33	120000	-0.45126	0.6741	PON1	76353	25kb
ENSG00000105852	7	94964256	95050680	143	14	120000	-0.51949	0.69829	PON3	76353	25kb
ENSG00000105854	7	95009175	95089510	216	27	120000	-1.1161	0.86782	PON2	76353	25kb
ENSG00000005981	7	95082756	95194544	352	38	120000	-0.96574	0.83291	ASB4	76353	25kb
ENSG00000269449	7	95146457	95198854	193	23	120000	-1.4311	0.9238	AC002451.1	76353	25kb
ENSG00000004799	7	95187811	95250803	211	22	120000	-1.8432	0.96735	PDK4	76353	25kb
ENSG00000158560	7	95376866	95764634	963	88	120000	0.22487	0.41104	DYNC1I1	76353	25kb
ENSG00000004864	7	95724532	95976459	321	18	120000	-1.5169	0.93535	SLC25A13	76353	25kb
ENSG00000197851	7	96085938	96157835	186	27	120000	-0.74096	0.77064	C7orf76	76353	25kb
ENSG00000127922	7	96085938	96364203	608	36	120000	-1.3144	0.90565	SHFM1	76353	25kb
ENSG00000006377	7	96609860	96665351	113	29	120000	0.1297	0.4484	DLX6	76353	25kb
ENSG00000105880	7	96624704	96679409	102	22	120000	-0.62939	0.73545	DLX5	76353	25kb
ENSG00000196636	7	96720902	96836075	229	20	120000	0.86639	0.19314	ACN9	76353	25kb
ENSG00000006128	7	97336220	97394784	259	13	120000	-1.1137	0.8673	TAC1	76353	25kb
ENSG00000070669	7	97456430	97526854	204	26	120000	-0.46045	0.6774	ASNS	76353	25kb
ENSG00000135175	7	97588996	97645481	260	25	120000	-1.1184	0.8683	OCM2	76353	25kb
ENSG00000164715	7	97711197	97863945	429	30	120000	0.3891	0.3486	LMTK2	76353	25kb
ENSG00000180535	7	97815739	97867291	130	22	120000	-0.045635	0.5182	BHLHA15	76353	25kb
ENSG00000205356	7	97818936	97906563	222	24	120000	0.61806	0.26827	TECPR1	76353	25kb
ENSG00000164713	7	97856691	97962162	287	14	120000	0.80464	0.21051	BRI3	76353	25kb
ENSG00000006453	7	97895963	98055380	384	16	120000	0.55278	0.29021	BAIAP2L1	76353	25kb
ENSG00000106236	7	98221609	98284180	135	23	120000	-0.96913	0.83376	NPTX2	76353	25kb
ENSG00000166448	7	98419111	98493394	186	27	120000	-1.2918	0.90179	TMEM130	76353	25kb
ENSG00000196367	7	98450556	98635866	387	19	120000	-0.71451	0.76255	TRRAP	76353	25kb
ENSG00000198742	7	98600061	98766723	367	45	120000	-0.9497	0.82887	SMURF1	76353	25kb
ENSG00000185467	7	98746197	98830129	326	21	120000	1.2436	0.10683	KPNA7	76353	25kb
ENSG00000241685	7	98898521	99010787	214	22	120000	0.8342	0.20208	ARPC1A	76353	25kb
ENSG00000130429	7	98946872	99017424	137	16	120000	0.61232	0.27016	ARPC1B	76353	25kb
ENSG00000106244	7	98964671	99031452	128	15	120000	0.54222	0.29383	PDAP1	76353	25kb
ENSG00000106245	7	98981264	99042239	108	16	120000	0.63377	0.26311	BUD31	76353	25kb
ENSG00000106246	7	98989362	99088787	171	18	120000	0.20951	0.41703	PTCD1	76353	25kb
ENSG00000248919	7	98992372	99088820	164	17	120000	0.15841	0.43707	ATP5J2-PTCD1	76353	25kb
ENSG00000160917	7	99011545	99079994	120	14	120000	0.16414	0.43481	CPSF4	76353	25kb
ENSG00000214314	7	99015552	99065747	100	12	120000	0.18989	0.4247	AC073063.1	76353	25kb
ENSG00000241468	7	99021098	99088954	118	13	120000	-0.14672	0.55832	ATP5J2	76353	25kb
ENSG00000198556	7	99045464	99126273	124	16	120000	0.28325	0.38849	ZNF789	76353	25kb
ENSG00000160908	7	99059142	99122947	93	16	120000	0.40875	0.34136	ZNF394	76353	25kb
ENSG00000196652	7	99077274	99157323	100	17	120000	0.55596	0.28912	ZKSCAN5	76353	25kb
ENSG00000221909	7	99118931	99181159	75	15	120000	0.47172	0.31856	FAM200A	76353	25kb
ENSG00000197343	7	99131029	99199076	95	14	120000	0.45261	0.32542	ZNF655	76353	25kb
ENSG00000272647	7	99131291	99230433	135	18	120000	0.43875	0.33042	GS1-259H13.10	76353	25kb
ENSG00000197037	7	99189569	99255030	86	13	120000	0.73616	0.23082	ZSCAN25	76353	25kb
ENSG00000106258	7	99220817	99302621	111	15	120000	0.5536	0.28993	CYP3A5	76353	25kb
ENSG00000160870	7	99277660	99357819	134	9	120000	-0.50563	0.69344	CYP3A7	76353	25kb
ENSG00000160868	7	99329604	99406888	79	15	120000	-0.55296	0.70985	CYP3A4	76353	25kb
ENSG00000021461	7	99400636	99488718	159	20	120000	0.97513	0.16475	CYP3A43	76353	25kb
ENSG00000244623	7	99448610	99499680	113	16	120000	1.4798	0.06946	OR2AE1	76353	25kb
ENSG00000146833	7	99449581	99542223	205	20	120000	1.3405	0.090039	TRIM4	76353	25kb
ENSG00000176402	7	99495892	99552243	138	15	120000	1.0916	0.1375	GJC3	76353	25kb
ENSG00000160862	7	99539343	99598780	132	17	120000	1.6019	0.054587	AZGP1	76353	25kb
ENSG00000106261	7	99588204	99664312	108	17	120000	1.1344	0.12831	ZKSCAN1	76353	25kb

ENSG00000166529	7	99622390	99687661	93	15	120000	0.53242	0.29722	ZSCAN21	76353	25kb
ENSG00000166526	7	99636656	99705171	106	14	120000	1.3329	0.09129	ZNF3	76353	25kb
ENSG00000168090	7	99661577	99714823	94	15	120000	1.9337	0.026576	COPS6	76353	25kb
ENSG00000166508	7	99665351	99724563	96	15	120000	1.8833	0.029828	MCM7	76353	25kb
ENSG00000221838	7	99674172	99732968	92	14	120000	2.142	0.016096	AP4M1	76353	25kb
ENSG00000106290	7	99679693	99742464	93	13	120000	2.1259	0.016755	TAF6	76353	25kb
ENSG00000166997	7	99692236	99748134	83	14	120000	1.9027	0.028543	CNPY4	76353	25kb
ENSG00000214309	7	99699317	99751118	70	13	120000	1.6263	0.051942	MBLAC1	76353	25kb
ENSG00000188186	7	99721530	99778567	74	14	120000	1.1741	0.12017	LAMTOR4	76353	25kb
ENSG00000146826	7	99727043	99781338	80	15	120000	0.91982	0.17883	C7orf43	76353	25kb
ENSG00000197093	7	99731867	99791373	91	14	120000	0.32086	0.37416	GAL3ST4	76353	25kb
ENSG00000213420	7	99742229	99799995	86	12	120000	-0.14558	0.55787	GPC2	76353	25kb
ENSG00000066923	7	99750186	99844111	181	17	120000	-0.68843	0.75441	STAG3	76353	25kb
ENSG00000160844	7	99773283	99894855	207	21	120000	-0.69402	0.75617	GATS	76353	25kb
ENSG00000213413	7	99790864	99844113	115	12	120000	-1.0107	0.84392	PVRIG	76353	25kb
ENSG00000214300	7	99880325	99944819	46	9	120000	0.30003	0.38208	SPDYE3	76353	25kb
ENSG00000121716	7	99908737	99990356	86	8	120000	-0.32412	0.62708	PILRB	76353	25kb
ENSG00000085514	7	99940153	100022719	110	10	120000	-0.52859	0.70146	PILRA	76353	25kb
ENSG00000078487	7	99973449	100051615	105	9	120000	-0.86578	0.80669	ZCWPW1	76353	25kb
ENSG00000146834	7	100001413	100056741	76	7	120000	-0.79112	0.78556	MEPCE	76353	25kb
ENSG00000160813	7	100007905	100059188	69	7	120000	-0.65708	0.74444	PPP1R35	76353	25kb
ENSG00000185955	7	100029238	100086894	73	9	120000	-0.8275	0.79602	C7orf61	76353	25kb
ENSG00000166925	7	100035982	100101902	87	8	120000	-0.64699	0.74118	TSC22D4	76353	25kb
ENSG00000166924	7	100056550	100117422	78	10	120000	-0.58628	0.72116	NYAP1	76353	25kb
ENSG00000106351	7	100111834	100190842	102	12	120000	0.052437	0.47909	AGFG2	76353	25kb
ENSG00000205307	7	100144855	100196270	67	12	120000	0.17462	0.43069	SAP25	76353	25kb
ENSG00000077454	7	100144855	100208776	81	16	120000	0.16888	0.43295	LRCH4	76353	25kb
ENSG00000106336	7	100156605	100223740	102	20	120000	0.4155	0.33889	FBXO24	76353	25kb
ENSG00000106333	7	100174800	100230798	82	16	120000	0.44685	0.32749	PCOLCE	76353	25kb
ENSG00000106330	7	100184725	100238007	77	13	120000	0.61063	0.27072	MOSPD3	76353	25kb
ENSG00000106327	7	100193039	100265402	112	17	120000	0.36107	0.35903	TFR2	76353	25kb
ENSG00000077080	7	100215720	100279084	106	19	120000	-0.30563	0.62006	ACTL6B	76353	25kb
ENSG00000172354	7	100246154	100301797	108	19	120000	-0.39357	0.65305	GNB2	76353	25kb
ENSG00000146830	7	100252130	100312071	125	18	120000	-0.52861	0.70146	GIGYF1	76353	25kb
ENSG00000172336	7	100278676	100330118	114	16	120000	-0.38021	0.6481	POP7	76353	25kb
ENSG00000130427	7	100293423	100346323	101	16	120000	-0.31175	0.62238	EPO	76353	25kb
ENSG00000196411	7	100375187	100450121	183	25	120000	0.58212	0.28024	EPHB4	76353	25kb
ENSG00000146828	7	100399442	100489631	216	31	120000	0.12144	0.45167	SLC12A9	76353	25kb
ENSG00000087077	7	100439760	100496076	121	16	120000	-0.4205	0.66294	TRIP6	76353	25kb
ENSG00000087087	7	100447733	100511285	162	16	120000	-0.613	0.73006	SRRT	76353	25kb
ENSG00000176125	7	100461346	100512339	141	15	120000	-0.66172	0.74592	UFSP1	76353	25kb
ENSG00000087085	7	100462615	100519594	152	17	120000	-0.61006	0.72909	ACHE	76353	25kb
ENSG00000169894	7	100522187	100636118	226	45	120000	0.78255	0.21695	MUC3A	76353	25kb
ENSG00000228273	7	100522257	100575424	140	29	120000	0.63417	0.26298	MUC3A	76353	25kb
ENSG00000205277	7	100587904	100687230	173	25	120000	0.62217	0.26692	MUC12	76353	25kb
ENSG00000169876	7	100638353	100727020	236	27	120000	-0.19313	0.57657	MUC17	76353	25kb
ENSG00000169871	7	100703720	100760017	226	25	120000	-0.5176	0.69763	TRIM56	76353	25kb
ENSG00000106366	7	100745370	100807547	202	34	120000	0.99934	0.15881	SERPINE1	76353	25kb
ENSG00000106367	7	100772678	100829877	181	28	120000	1.7119	0.043461	AP1S1	76353	25kb
ENSG00000128564	7	100780790	100833874	164	25	120000	1.3701	0.085329	VGF	76353	25kb
ENSG00000167011	7	100788774	100848557	172	24	120000	0.86637	0.19314	NAT16	76353	25kb
ENSG00000106384	7	100813288	100869302	160	30	120000	0.55871	0.28818	MOGAT3	76353	25kb
ENSG00000106397	7	100824258	100886701	148	27	120000	-0.16906	0.56713	PLOD3	76353	25kb
ENSG00000106400	7	100835949	100892471	130	22	120000	-0.4706	0.68104	ZNHIT1	76353	25kb
ENSG00000106404	7	100850373	100907101	198	16	120000	-1.1281	0.87036	CLDN15	76353	25kb
ENSG00000214253	7	100857739	100920597	251	15	120000	-1.098	0.86389	FIS1	76353	25kb
ENSG00000128581	7	100931975	100990104	238	20	120000	-1.7716	0.96177	RABL5	76353	25kb
ENSG00000106436	7	101231605	101297576	229	31	120000	-0.69955	0.75789	MYL10	76353	25kb
ENSG00000257923	7	101433959	101952249	1288	73	120000	0.16124	0.43595	CUX1	76353	25kb
ENSG00000160999	7	101903405	101987178	210	37	120000	-0.19855	0.57869	SH2B2	76353	25kb
ENSG00000128563	7	101979319	102092123	222	22	120000	0.30273	0.38105	PRKRIP1	76353	25kb
ENSG00000160991	7	102048553	102122268	221	28	120000	0.081917	0.46736	ORAI2	76353	25kb
ENSG00000160993	7	102071685	102130323	153	22	120000	-0.22634	0.58953	ALKBH4	76353	25kb
ENSG00000161036	7	102080376	102138615	131	18	120000	-0.1899	0.57531	LRWD1	76353	25kb
ENSG00000005075	7	102088565	102144354	115	16	120000	-0.16297	0.56473	POLR2J	76353	25kb
ENSG00000170667	7	102097892	102183228	89	13	120000	-0.029898	0.51193	RASA4B	76353	25kb
ENSG00000168255	7	102153365	102238103	1	1	120000	-0.37203	0.64506	POLR2J3	76353	25kb
ENSG00000270249	7	102156948	102257891	1	1	120000	-0.37203	0.64506	RP11-514P8.7	76353	25kb
ENSG00000205238	7	102166679	102227755	1	1	120000	-0.37203	0.64506	SPDYE2	76353	25kb
ENSG00000228049	7	102252474	102337076	17	5	120000	-0.21575	0.58541	POLR2J2	76353	25kb
ENSG00000267645	7	102252496	102337088	17	5	120000	-0.21575	0.58541	POLR2J2	76353	25kb
ENSG00000222011	7	102364418	102474672	179	28	120000	0.048693	0.48058	FAM185A	76353	25kb
ENSG00000161040	7	102428308	102740286	507	27	120000	-0.29696	0.61675	FBXL13	76353	25kb
ENSG00000128606	7	102528438	102610396	122	17	120000	0.10419	0.45851	LRRC17	76353	25kb
ENSG00000170632	7	102690328	102765205	151	12	120000	-0.94665	0.82809	ARMC10	76353	25kb
ENSG00000161048	7	102715223	102815007	319	16	120000	-0.98145	0.83681	NAPEPLD	76353	25kb
ENSG00000105819	7	102912869	102994958	124	22	120000	-0.44971	0.67354	PMPCB	76353	25kb
ENSG00000105821	7	102927921	103010320	133	23	120000	-0.53038	0.70207	DNAJC2	76353	25kb
ENSG00000161057	7	102959701	103034842	130	22	120000	0.14231	0.44342	PSMC2	76353	25kb
ENSG00000170615	7	102968177	103111624	356	36	120000	0.0066159	0.49736	SLC26A5	76353	25kb
ENSG00000189056	7	103087231	103654963	2209	119	120000	-0.72116	0.7646	RELN	76353	25kb
ENSG00000164815	7	103741788	103873495	410	19	120000	-0.95122	0.82925	ORC5	76353	25kb
ENSG00000187416	7	103944104	104574001	1883	114	120000	-0.68053	0.75191	LHFPL3	76353	25kb
ENSG00000005483	7	104629626	104779808	214	18	120000	-1.2148	0.88778	KMT2E	76353	25kb
ENSG00000135250	7	104726151	105064755	616	22	120000	-0.28142	0.6108	SRPK2	76353	25kb
ENSG00000091127	7	105055108	105187714	456	43	120000	-0.93877	0.82608	PUS7	76353	25kb
ENSG00000135249	7	105147532	105233124	302	17	120000	-0.93691	0.8256	RINT1	76353	25kb
ENSG00000185055	7	105180567	105266322	187	33	120000	-0.87576	0.80942	EFCAB10	76353	25kb
ENSG00000146776	7	105220514	105542050	879	123	120000	-0.68015	0.7518	ATXN7L1	76353	25kb
ENSG00000128536	7	105492242	105701877	741	110	120000	-0.6411	0.73927	CDHR3	76353	25kb
ENSG00000008282	7	105705949	105778022	242	31	120000	-0.33762	0.63218	SYPL1	76353	25kb
ENSG00000105835	7	105863731	105951772	205	23	120000	-0.68015	0.7518	NAMPT	76353	25kb
ENSG00000253276	7	106272211	106326442	189	40	120000	-2.0714	0.98084	CCDC71L	76353	25kb
ENSG00000105851	7	106480723	106572590	219	34	120000	-1.4168	0.92173	PIK3CG	76353	25kb
ENSG00000005249	7	106660094	106827256	367	27	120000	0.15914	0.43678	PRKAR2B	76353	25kb
ENSG00000105856	7	106784406	106867974	151	12	120000	1.0347	0.15041	HBP1	76353	25kb

ENSG00000164597	7	106817000	107229959	802	19	120000	0.9901	0.16106	COG5	76353	25kb
ENSG00000172209	7	107085463	107141098	110	11	120000	0.62736	0.26521	GPR22	76353	25kb
ENSG00000105865	7	107178929	107243906	102	10	120000	0.65415	0.25651	DUS4L	76353	25kb
ENSG00000075790	7	107195422	107294615	141	15	120000	0.5359	0.29601	BCAP29	76353	25kb
ENSG00000091137	7	107276080	107383254	194	31	120000	1.0656	0.1433	SLC26A4	76353	25kb
ENSG00000105879	7	107359142	107427112	148	19	120000	0.034908	0.48608	CBLL1	76353	25kb
ENSG00000091138	7	107380912	107468670	268	37	120000	-0.13269	0.55278	SLC26A3	76353	25kb
ENSG00000091140	7	107506415	107597175	201	17	120000	-0.22747	0.58997	DLD	76353	25kb
ENSG00000091136	7	107539244	107668700	310	45	120000	-0.61792	0.73169	LAMB1	76353	25kb
ENSG00000091128	7	107638993	107795801	341	34	120000	-1.6309	0.94854	LAMB4	76353	25kb
ENSG00000091129	7	107763068	108122161	1181	53	120000	0.71655	0.23683	NRCAM	76353	25kb
ENSG00000135241	7	108085866	108235110	418	24	120000	1.4814	0.069252	PNPLA8	76353	25kb
ENSG00000177683	7	108169987	108235194	169	18	120000	-0.099118	0.53948	THAP5	76353	25kb
ENSG00000128590	7	108185012	108240294	153	14	120000	-0.55726	0.71132	DNAJB9	76353	25kb
ENSG00000205174	7	108499032	108549644	142	16	120000	-0.17373	0.56896	C7orf66	76353	25kb
ENSG00000269185	7	108515389	108565655	222	11	120000	0.55263	0.29026	FLJ00325	76353	25kb
ENSG00000184903	7	110278110	111227573	1903	79	120000	0.23484	0.40717	IMMP2L	76353	25kb
ENSG00000173114	7	110706062	110790510	173	17	120000	0.813	0.20811	LRRN3	76353	25kb
ENSG00000128512	7	111341166	111871466	1275	137	120000	-1.2675	0.89752	DOCK4	76353	25kb
ENSG00000198839	7	111821643	112008151	499	29	120000	-0.16719	0.56639	ZNF277	76353	25kb
ENSG00000006652	7	112038023	112146072	345	41	120000	-1.1769	0.88039	IFRD1	76353	25kb
ENSG00000181016	7	112095908	112156117	205	31	120000	-0.50408	0.6929	LSMEM1	76353	25kb
ENSG00000146802	7	112377437	112455647	131	13	120000	-1.3822	0.91655	TMEM168	76353	25kb
ENSG00000164603	7	112434202	112604971	261	24	120000	0.67955	0.24839	C7orf60	76353	25kb
ENSG00000164604	7	112693386	112752833	176	25	120000	-0.25446	0.60043	GPR85	76353	25kb
ENSG00000214194	7	112731773	112783668	140	15	120000	-0.69129	0.75531	LINC00998	76353	25kb
ENSG00000236294	7	113066127	113116457	108	12	120000	-0.42984	0.66635	TSRM	76353	25kb
ENSG00000154415	7	113491832	113740975	468	22	120000	-0.24388	0.59634	PPP1R3A	76353	25kb
ENSG00000128573	7	113701382	114358827	1146	66	120000	0.61867	0.26807	FOXP2	76353	25kb
ENSG00000135272	7	114537209	114684256	293	41	120000	0.3051	0.38015	MDFIC	76353	25kb
ENSG00000105967	7	115550202	115824950	677	39	120000	-1.6492	0.95045	TFEC	76353	25kb
ENSG00000135269	7	115825547	115923837	330	21	120000	-1.0967	0.86361	TES	76353	25kb
ENSG00000105971	7	115902434	116173595	719	46	120000	-1.704	0.95581	CAV2	76353	25kb
ENSG00000105974	7	116139839	116226233	227	24	120000	-1.0877	0.86164	CAV1	76353	25kb
ENSG00000105976	7	116287444	116463440	335	42	120000	-0.7223	0.76494	MET	76353	25kb
ENSG00000198898	7	116426124	116587103	291	23	120000	-0.5459	0.70743	CAPZA2	76353	25kb
ENSG00000004866	7	116568292	116895157	532	53	120000	-0.32319	0.62672	ST7	76353	25kb
ENSG00000214188	7	116568953	116763860	243	46	120000	-0.19286	0.57647	ST7-OT4	76353	25kb
ENSG00000228368	7	116580900	116632526	67	19	120000	-0.54843	0.7083	AC106873.4	76353	25kb
ENSG00000105989	7	116891685	116988343	237	35	120000	-1.2237	0.88946	WNT2	76353	25kb
ENSG00000154438	7	116978276	117093177	149	17	120000	0.62066	0.26741	ASZ1	76353	25kb
ENSG00000001626	7	117080838	117381025	504	23	120000	-0.080923	0.53225	CFTR	76353	25kb
ENSG00000077063	7	117325705	117539193	444	57	120000	-0.81403	0.79219	CTTNBP2	76353	25kb
ENSG00000128534	7	117799086	117857878	229	11	120000	-0.26349	0.60391	NAA38	76353	25kb
ENSG00000106013	7	117829727	117907785	220	13	120000	-0.37133	0.6448	ANKRD7	76353	25kb
ENSG00000184408	7	119888722	120415385	974	51	120000	-0.16445	0.56531	KCND2	76353	25kb
ENSG00000106025	7	120402376	120523456	222	21	120000	-1.238	0.89214	TSPAN12	76353	25kb
ENSG00000071243	7	120565803	120642270	143	15	120000	-1.1118	0.86689	ING3	76353	25kb
ENSG00000106034	7	120603731	120962498	809	35	120000	-1.0453	0.85207	CPED1	76353	25kb
ENSG00000002745	7	120940421	121006158	147	18	120000	-1.1571	0.87639	WNT16	76353	25kb
ENSG00000196937	7	120963905	121061418	243	24	120000	-0.89714	0.81518	FAM3C	76353	25kb
ENSG00000106278	7	121488143	121727090	553	42	120000	-0.068282	0.52722	PTPRZ1	76353	25kb
ENSG00000008311	7	121690701	121809334	226	26	120000	0.13355	0.44688	AASS	76353	25kb
ENSG00000128610	7	121916448	121975745	153	29	120000	-0.10952	0.5436	FEZF1	76353	25kb
ENSG00000081803	7	121933481	122551813	1443	71	120000	-0.1337	0.55318	CADPS2	76353	25kb
ENSG00000188050	7	122312766	122364210	123	17	120000	-0.24738	0.59769	RNF133	76353	25kb
ENSG00000235631	7	122316718	122368021	122	18	120000	-0.16125	0.56405	RNF148	76353	25kb
ENSG00000128519	7	122609759	122660754	150	16	120000	-1.4378	0.92476	TAS2R16	76353	25kb
ENSG00000081800	7	122728585	122865040	323	27	120000	-1.994	0.97692	SLC13A1	76353	25kb
ENSG00000164675	7	123067454	123200131	190	21	120000	-0.87266	0.80858	IQUB	76353	25kb
ENSG00000128609	7	123152051	123223309	143	20	120000	-1.0028	0.84203	NDUFA5	76353	25kb
ENSG00000146809	7	123182064	123302951	247	33	120000	-0.19393	0.57688	ASB15	76353	25kb
ENSG00000170807	7	123270861	123329344	108	15	120000	1.0977	0.13616	LMOD2	76353	25kb
ENSG00000106299	7	123296989	123414121	280	17	120000	1.234	0.1086	WASL	76353	25kb
ENSG00000106302	7	123444037	123542532	181	25	120000	-0.76086	0.77663	HYAL4	76353	25kb
ENSG00000106304	7	123540286	123636468	198	26	120000	2.291	0.010982	SPAM1	76353	25kb
ENSG00000234224	7	123645973	123698523	134	15	120000	1.0752	0.14115	TMEM229A	76353	25kb
ENSG00000170775	7	124361051	124430681	146	27	120000	1.2025	0.11458	GPR37	76353	25kb
ENSG00000128513	7	124437440	124595037	492	10	120000	-0.0028437	0.50113	POT1	76353	25kb
ENSG00000179603	7	126053652	126918348	2241	69	120000	-0.35173	0.63748	GRM8	76353	25kb
ENSG00000048405	7	126961844	127096978	412	31	120000	-0.64713	0.74123	ZNF800	76353	25kb
ENSG00000179562	7	127195672	127258665	144	21	120000	-1.1174	0.86808	GCC1	76353	25kb
ENSG00000004059	7	127203399	127256759	120	19	120000	-1.1012	0.86459	ARF5	76353	25kb
ENSG00000106328	7	127206463	127267198	132	20	120000	-1.1246	0.86963	FSCN3	76353	25kb
ENSG00000106331	7	127225346	127280982	109	18	120000	-1.0492	0.85295	PAX4	76353	25kb
ENSG00000197157	7	127267234	127757661	819	35	120000	0.62401	0.26631	SND1	76353	25kb
ENSG00000128594	7	127642124	127697160	83	21	120000	0.91856	0.17916	LRRC4	76353	25kb
ENSG00000174697	7	127856337	127922681	168	30	120000	0.76583	0.22189	LEP	76353	25kb
ENSG00000106344	7	127912738	128008962	223	38	120000	1.9836	0.023648	RBM28	76353	25kb
ENSG00000224940	7	127965379	128026739	142	35	120000	1.2527	0.10516	PRRT4	76353	25kb
ENSG00000106348	7	128007331	128075306	211	33	120000	0.91865	0.17914	IMPDH1	76353	25kb
ENSG00000135245	7	128070903	128123472	152	26	120000	0.87394	0.19108	HILPDA	76353	25kb
ENSG00000165055	7	128091783	128171656	277	27	120000	-0.43103	0.66678	METTL2B	76353	25kb
ENSG00000205085	7	128287342	128351929	214	20	120000	-0.66054	0.74555	FAM71F2	76353	25kb
ENSG00000135248	7	128324115	128396797	254	32	120000	-0.40672	0.65789	FAM71F1	76353	25kb
ENSG00000128595	7	128354346	128436861	253	27	120000	0.044619	0.48221	CALU	76353	25kb
ENSG00000128617	7	128387545	128440844	146	18	120000	0.71864	0.23618	OPN1SW	76353	25kb
ENSG00000128596	7	128405811	128487186	209	25	120000	0.81684	0.20701	CCDC136	76353	25kb
ENSG00000128591	7	128445431	128524328	228	27	120000	1.4809	0.069323	FLNC	76353	25kb
ENSG00000128524	7	128477880	128530898	159	23	120000	1.6871	0.045793	ATP6V1F	76353	25kb
ENSG00000128604	7	128552666	128615089	179	32	120000	0.33769	0.3678	IRF5	76353	25kb
ENSG00000064419	7	128569948	128720198	389	30	120000	-0.065173	0.52598	TNPO3	76353	25kb
ENSG00000158457	7	128759712	128833671	206	32	120000	-0.12945	0.5515	TSPAN33	76353	25kb
ENSG00000128602	7	128803713	128878386	195	31	120000	-0.88879	0.81294	SMO	76353	25kb
ENSG00000158467	7	128839864	129095052	551	30	120000	0.028915	0.48847	AHCYL2	76353	25kb
ENSG00000128578	7	129049274	129153240	198	23	120000	-0.16186	0.56429	STRIP2	76353	25kb

ENSG00000240204	7	129117320	129177773	239	43	120000	1.2393	0.10761	SMKR1	76353	25kb
ENSG00000106459	7	129226555	129421922	512	33	120000	-0.34577	0.63524	NRF1	76353	25kb
ENSG00000186591	7	129445572	129617789	590	27	120000	0.83371	0.20222	UBE2H	76353	25kb
ENSG00000091732	7	129633126	129716291	239	21	120000	-0.97288	0.83469	ZC3HC1	76353	25kb
ENSG00000128607	7	129685350	129800560	276	27	120000	0.39962	0.34472	KLHDC10	76353	25kb
ENSG00000146842	7	129779555	129872610	225	30	120000	-0.58362	0.72026	TMEM209	76353	25kb
ENSG00000165120	7	129822700	129881683	127	17	120000	-0.62472	0.73392	SSMEM1	76353	25kb
ENSG00000158516	7	129881667	129954638	212	29	120000	-0.83075	0.79694	CPA2	76353	25kb
ENSG00000128510	7	129907974	129989020	282	29	120000	-1.4343	0.92426	CPA4	76353	25kb
ENSG00000158525	7	129959630	130033571	258	25	120000	-0.40775	0.65827	CPA5	76353	25kb
ENSG00000091704	7	129995180	130052955	150	23	120000	-0.12002	0.54777	CPA1	76353	25kb
ENSG00000106477	7	130008612	130107274	204	35	120000	-1.6346	0.94894	CEP41	76353	25kb
ENSG00000106484	7	130101012	130171133	108	14	120000	-1.0664	0.85688	MEST	76353	25kb
ENSG00000158623	7	130121089	130378598	323	21	120000	-0.46687	0.6797	COPG2	76353	25kb
ENSG00000213265	7	130328488	130397268	165	23	120000	-0.51294	0.696	TSGA13	76353	25kb
ENSG00000174595	7	130392401	130443888	144	26	120000	-0.74599	0.77216	KLF14	76353	25kb
ENSG00000128585	7	130769855	131206395	1092	100	120000	-1.4826	0.9309	MKLN1	76353	25kb
ENSG00000128567	7	131160021	131267976	299	57	120000	1.4109	0.079132	PODXL	76353	25kb
ENSG00000221866	7	131783091	132358447	1714	146	120000	-0.47092	0.68115	PLXNA4	76353	25kb
ENSG00000183470	7	132308553	132438528	298	36	120000	1.2239	0.11049	AC009365.3	76353	25kb
ENSG00000106554	7	132444629	132791848	671	30	120000	0.87055	0.192	CHCHD3	76353	25kb
ENSG00000131558	7	132912829	133776342	1702	49	120000	0.85604	0.19599	EXOC4	76353	25kb
ENSG00000155530	7	133787052	133974343	429	19	120000	2.2529	0.012132	LRGUK	76353	25kb
ENSG00000205060	7	133949084	134026803	228	11	120000	-0.18019	0.5715	SLC35B4	76353	25kb
ENSG00000085662	7	134102102	134169036	163	32	120000	0.089649	0.46428	AKR1B1	76353	25kb
ENSG00000198074	7	134187344	134251160	253	21	120000	-0.96204	0.83198	AKR1B10	76353	25kb
ENSG00000227471	7	134208888	134289627	380	24	120000	-0.40369	0.65678	AKR1B15	76353	25kb
ENSG00000172331	7	134306560	134389565	268	16	120000	-0.44566	0.67208	BPGM	76353	25kb
ENSG00000122786	7	134404003	134680479	764	53	120000	-0.65509	0.7438	CALD1	76353	25kb
ENSG00000146856	7	134646259	134857715	572	23	120000	0.67273	0.25056	AGBL3	76353	25kb
ENSG00000122783	7	134752115	134880547	393	17	120000	0.27707	0.39086	C7orf49	76353	25kb
ENSG00000229974	7	134807797	134857916	148	11	120000	0.65288	0.25692	AC083862.1	76353	25kb
ENSG00000146859	7	134807824	134875967	223	17	120000	0.43356	0.3323	TMEM140	76353	25kb
ENSG00000105875	7	134843590	134921316	231	16	120000	0.1524	0.43944	WDR91	76353	25kb
ENSG00000146857	7	134891731	134968244	176	30	120000	-0.2894	0.61386	STRA8	76353	25kb
ENSG00000080802	7	135021547	135219875	390	37	120000	2.3496	0.0093973	CNOT4	76353	25kb
ENSG00000155561	7	135217667	135358505	574	22	120000	0.72465	0.23433	NUP205	76353	25kb
ENSG00000243317	7	135322244	135403166	287	27	120000	0.082181	0.46725	C7orf73	76353	25kb
ENSG00000164707	7	135340985	135439006	322	33	120000	1.1646	0.12209	SLC13A4	76353	25kb
ENSG00000189320	7	135388096	135458594	221	33	120000	1.824	0.034076	FAM180A	76353	25kb
ENSG00000105887	7	135586509	135687101	354	29	120000	0.76058	0.22345	MTPN	76353	25kb
ENSG00000236338	7	135586542	135638214	145	28	120000	0.66845	0.25192	AC015987.2	76353	25kb
ENSG00000267697	7	135587022	135637198	140	27	120000	0.64583	0.25919	LUZP6	76353	25kb
ENSG00000181072	7	136528416	136730002	591	40	120000	-0.49795	0.69074	CHRM2	76353	25kb
ENSG00000105894	7	136887088	137053611	443	56	120000	0.94626	0.17201	PTN	76353	25kb
ENSG00000157680	7	137040783	137556838	1050	83	120000	0.26223	0.39657	DGKI	76353	25kb
ENSG00000182158	7	137534725	137711813	401	58	120000	-0.1846	0.57323	CREB3L2	76353	25kb
ENSG00000122787	7	137662070	137827732	431	51	120000	0.45841	0.32333	AKR1D1	76353	25kb
ENSG00000122779	7	138120079	138299738	391	29	120000	0.10932	0.45647	TRIM24	76353	25kb
ENSG00000157703	7	138254030	138411097	527	78	120000	-0.46991	0.68079	SVOPL	76353	25kb
ENSG00000105929	7	138366040	138509305	555	77	120000	-0.63884	0.73854	ATP6V0A4	76353	25kb
ENSG00000214128	7	138457695	138547846	266	29	120000	-0.0013087	0.50052	TMEM213	76353	25kb
ENSG00000122778	7	138491126	138691064	585	52	120000	-0.8006	0.78832	KIAA1549	76353	25kb
ENSG00000146858	7	138685452	138745775	208	17	120000	-1.062	0.85588	ZC3HAV1L	76353	25kb
ENSG00000105939	7	138703266	138819465	332	35	120000	-2.0429	0.97947	ZC3HAV1	76353	25kb
ENSG00000105948	7	138793490	138901732	216	17	120000	-1.6238	0.94779	TTC26	76353	25kb
ENSG00000157741	7	138890102	139017982	191	21	120000	-1.07	0.8577	UBN2	76353	25kb
ENSG00000164898	7	138999203	139056065	173	10	120000	-0.82818	0.79622	C7orf55	76353	25kb
ENSG00000146963	7	139000105	139133198	419	11	120000	-0.92137	0.82157	C7orf55-LUC7L2	76353	25kb
ENSG00000269955	7	139001106	139132345	415	11	120000	-0.94653	0.82806	LUC7L2	76353	25kb
ENSG00000188883	7	139112436	139193458	288	36	120000	-2.3218	0.98988	KLRG2	76353	25kb
ENSG00000236279	7	139183602	139254730	154	31	120000	-1.0043	0.84237	CLEC2L	76353	25kb
ENSG00000064393	7	139221316	139502577	526	56	120000	-0.4345	0.66804	HIPK2	76353	25kb
ENSG00000059377	7	139451850	139745125	707	93	120000	-1.6378	0.94927	TBXAS1	76353	25kb
ENSG00000059378	7	139698544	139788521	292	35	120000	-1.1691	0.87881	PARP12	76353	25kb
ENSG00000006459	7	139759546	139901835	315	27	120000	0.65833	0.25516	KDM7A	76353	25kb
ENSG00000157800	7	139968493	140129233	634	57	120000	-1.9101	0.97194	SLC37A3	76353	25kb
ENSG00000146955	7	140078843	140151050	249	35	120000	-1.7803	0.96249	RAB19	76353	25kb
ENSG00000133606	7	140127840	140204369	265	21	120000	-1.9002	0.9713	MKRN1	76353	25kb
ENSG00000146966	7	140193220	140398793	479	43	120000	0.24173	0.4045	DENND2A	76353	25kb
ENSG00000133597	7	140347953	140421061	122	12	120000	0.94625	0.17201	ADCK2	76353	25kb
ENSG00000090266	7	140365577	140447590	122	14	120000	0.44138	0.32947	NDUFB2	76353	25kb
ENSG00000157764	7	140394127	140649564	391	16	120000	-0.54929	0.7086	BRAF	76353	25kb
ENSG00000090263	7	140680854	140740028	113	12	120000	0.56476	0.28612	MRPS33	76353	25kb
ENSG00000261115	7	140749032	141205180	1038	94	120000	0.36916	0.356	TMEM178B	76353	25kb
ENSG00000006530	7	141225989	141380044	277	16	120000	-1.1854	0.88206	AGK	76353	25kb
ENSG00000257093	7	141331528	141426953	162	23	120000	-1.0388	0.85055	KIAA1147	76353	25kb
ENSG00000214102	7	141383153	141456071	103	11	120000	-0.92089	0.82145	WEE2	76353	25kb
ENSG00000106028	7	141413121	141512722	188	7	120000	-1.1548	0.87592	SSBP1	76353	25kb
ENSG00000127362	7	141438897	141489997	118	5	120000	-1.213	0.88744	TAS2R3	76353	25kb
ENSG00000127364	7	141453242	141504235	115	4	120000	-1.1548	0.87592	TAS2R4	76353	25kb
ENSG00000127366	7	141465017	141516166	95	5	120000	-1.0566	0.85464	TAS2R5	76353	25kb
ENSG00000165076	7	141511086	141566287	139	10	120000	-0.62534	0.73413	PRSS37	76353	25kb
ENSG00000257335	7	141582613	141831547	620	78	120000	0.010363	0.49587	MGAM	76353	25kb
ENSG00000258083	7	141593617	141644709	128	16	120000	-0.46459	0.67889	OR9A4	76353	25kb
ENSG00000258227	7	141602157	141671807	167	17	120000	-0.61772	0.73162	CLEC5A	76353	25kb
ENSG00000257138	7	141647431	141698573	97	24	120000	0.49816	0.30919	TAS2R38	76353	25kb
ENSG00000257743	7	141786549	141947124	490	65	120000	-0.98278	0.83714	RP11-1220K2.2	76353	25kb
ENSG00000258223	7	141926963	141982878	271	14	120000	0.22684	0.41027	PRSS58	76353	25kb
ENSG00000270672	7	142349104	142400550	170	11	120000	0.10092	0.45981	MTRNR2L6	76353	25kb
ENSG00000204983	7	142432319	142485923	118	12	120000	-0.13512	0.55374	PRSS1	76353	25kb
ENSG00000106123	7	142527792	142593847	214	20	120000	-0.8014	0.78855	EPHB6	76353	25kb
ENSG00000165125	7	142543956	142608507	185	17	120000	-0.021401	0.50854	TRPV6	76353	25kb
ENSG00000127412	7	142580267	142655905	215	19	120000	0.39563	0.34619	TRPV5	76353	25kb
ENSG00000165131	7	142611440	142662955	153	16	120000	0.17714	0.4297	C7orf34	76353	25kb
ENSG00000197993	7	142613201	142684768	231	21	120000	-0.46773	0.68001	KEL	76353	25kb

ENSG00000179468	7	142698245	142749282	104	14	120000	-2.2053	0.98629	OR9A2	76353	25kb
ENSG00000225781	7	142724417	142775444	143	18	120000	-2.1361	0.98366	OR6V1	76353	25kb
ENSG00000159763	7	142804170	142861839	147	14	120000	-1.1928	0.88353	PIP	76353	25kb
ENSG00000236398	7	142855512	142906528	87	9	120000	-0.95071	0.82912	TAS2R39	76353	25kb
ENSG00000221937	7	142894130	142945162	57	11	120000	-1.1675	0.87849	TAS2R40	76353	25kb
ENSG00000268170	7	142894163	142944360	56	11	120000	-1.1639	0.87776	AC073342.1	76353	25kb
ENSG00000197448	7	142916186	142992947	53	17	120000	-0.71878	0.76386	GSTK1	76353	25kb
ENSG00000178826	7	142952050	143010141	39	14	120000	-0.89789	0.81538	TMEM139	76353	25kb
ENSG00000106144	7	142960308	143029789	71	23	120000	-1.0522	0.85365	CASP2	76353	25kb
ENSG00000188037	7	142988219	143074176	169	39	120000	-1.8862	0.97037	CLCN1	76353	25kb
ENSG00000159784	7	143025493	143084863	152	34	120000	-1.5053	0.93388	FAM131B	76353	25kb
ENSG00000159840	7	143053173	143113204	148	40	120000	-0.0061248	0.50244	ZYX	76353	25kb
ENSG00000146904	7	143062382	143130985	192	44	120000	0.73699	0.23056	EPHA1	76353	25kb
ENSG00000185899	7	143115546	143166502	132	25	120000	0.77229	0.21997	TAS2R60	76353	25kb
ENSG00000221855	7	143149966	143200889	169	21	120000	-0.22235	0.58798	TAS2R41	76353	25kb
ENSG00000271079	7	143243894	143296480	2	1	120000	0.38422	0.35041	CTAGE15	76353	25kb
ENSG00000170379	7	143293043	143452502	30	7	120000	-0.47213	0.68158	FAM115C	76353	25kb
ENSG00000271321	7	143427182	143479789	22	6	120000	-0.89926	0.81574	CTAGE6	76353	25kb
ENSG00000198420	7	143523468	143624291	92	19	120000	1.9896	0.023318	FAM115A	76353	25kb
ENSG00000221910	7	143607259	143658372	165	14	120000	1.8239	0.034083	OR2F2	76353	25kb
ENSG00000213215	7	143632027	143683108	161	17	120000	1.5523	0.060294	OR2F1	76353	25kb
ENSG00000221813	7	143676022	143727068	144	10	120000	1.1064	0.13427	OR6B1	76353	25kb
ENSG00000221836	7	143722461	143773473	221	13	120000	1.7394	0.040979	OR2A5	76353	25kb
ENSG00000221933	7	143746275	143797288	157	12	120000	2.2699	0.011608	OR2A25	76353	25kb
ENSG00000221858	7	143767141	143818186	139	11	120000	1.7927	0.036509	OR2A12	76353	25kb
ENSG00000221989	7	143781607	143832657	157	10	120000	1.618	0.052829	OR2A2	76353	25kb
ENSG00000221938	7	143801151	143852162	181	7	120000	1.4456	0.074148	OR2A14	76353	25kb
ENSG00000225932	7	143855559	143908146	54	7	120000	1.9916	0.023205	CTAGE4	76353	25kb
ENSG00000213214	7	143858176	144016230	55	9	120000	1.9675	0.024566	ARHGEF35	76353	25kb
ENSG00000212807	7	143904004	143954936	4	2	120000	0.3529	0.36208	OR2A42	76353	25kb
ENSG00000243896	7	143930700	143981815	6	3	120000	0.40421	0.34303	OR2A7	76353	25kb
ENSG00000244693	7	143938794	143991381	6	3	120000	0.40421	0.34303	CTAGE8	76353	25kb
ENSG00000221970	7	143990218	144041150	2	1	120000	-1.234	0.8914	OR2A1	76353	25kb
ENSG00000050327	7	144027381	144102725	62	9	120000	0.17177	0.43181	ARHGEF5	76353	25kb
ENSG00000106410	7	144069333	144132320	135	16	120000	1.2681	0.10239	NOBOX	76353	25kb
ENSG00000196511	7	144124034	144558488	1153	71	120000	0.4458	0.32787	TPK1	76353	25kb
ENSG00000174469	7	145788453	148143090	8238	329	120000	-0.093456	0.53723	CNTNAP2	76353	25kb
ENSG00000170279	7	148262657	148337952	214	44	120000	-0.11207	0.54461	C7orf33	76353	25kb
ENSG00000055130	7	148370006	148523128	324	26	120000	0.90966	0.1815	CUL1	76353	25kb
ENSG00000106462	7	148479475	148606413	271	26	120000	1.1098	0.13355	EZH2	76353	25kb
ENSG00000155660	7	148675154	148750733	242	29	120000	-0.93823	0.82594	PDIA4	76353	25kb
ENSG00000197362	7	148741735	148812874	169	20	120000	-1.464	0.9284	ZNF786	76353	25kb
ENSG00000204947	7	148774876	148848438	179	14	120000	-0.89288	0.81404	ZNF425	76353	25kb
ENSG00000197024	7	148798508	148905116	294	15	120000	-1.1472	0.87435	ZNF398	76353	25kb
ENSG00000170265	7	148867577	148948339	233	32	120000	-1.4982	0.93295	ZNF282	76353	25kb
ENSG00000170260	7	148911742	148977700	206	30	120000	-1.2376	0.89206	ZNF212	76353	25kb
ENSG00000204946	7	148934262	149019393	251	32	120000	-1.374	0.91528	ZNF783	76353	25kb
ENSG00000196453	7	149103454	149183214	166	29	120000	0.7581	0.2242	ZNF777	76353	25kb
ENSG00000181220	7	149144885	149219908	120	17	120000	-0.89055	0.81342	ZNF746	76353	25kb
ENSG00000133619	7	149386872	149456664	220	30	120000	-0.69947	0.75787	KRBA1	76353	25kb
ENSG00000181444	7	149436271	149495568	198	35	120000	0.38829	0.3489	ZNF467	76353	25kb
ENSG00000106479	7	149510456	149589568	218	24	120000	0.4468	0.32751	ZNF862	76353	25kb
ENSG00000171130	7	149545057	149602784	147	15	120000	0.49252	0.31118	ATP6V0E2	76353	25kb
ENSG00000106526	7	149916005	150045814	300	23	120000	-0.24082	0.59515	ACTR3C	76353	25kb
ENSG00000127399	7	149994728	150060239	158	11	120000	-1.4502	0.9265	LRRC61	76353	25kb
ENSG00000188707	7	150001938	150054808	137	9	120000	-1.3772	0.91577	ZBED6CL	76353	25kb
ENSG00000106538	7	150010408	150063763	146	11	120000	-1.6742	0.95295	RARRES2	76353	25kb
ENSG00000214022	7	150040278	150096133	170	20	120000	-1.1822	0.88144	REPIN1	76353	25kb
ENSG00000196456	7	150040879	150134558	312	31	120000	-0.4597	0.67714	ZNF775	76353	25kb
ENSG00000171115	7	150122718	150201480	220	32	120000	-0.081648	0.53254	GIMAP8	76353	25kb
ENSG00000179144	7	150186918	150243161	249	25	120000	-0.51263	0.6959	GIMAP7	76353	25kb
ENSG00000133574	7	150239365	150296041	333	13	120000	-0.51137	0.69545	GIMAP4	76353	25kb
ENSG00000133561	7	150297463	150354473	222	18	120000	-0.47737	0.68345	GIMAP6	76353	25kb
ENSG00000106560	7	150357785	150415729	133	17	120000	0.42075	0.33697	GIMAP2	76353	25kb
ENSG00000213203	7	150388645	150446372	156	19	120000	0.47816	0.31627	GIMAP1	76353	25kb
ENSG00000196329	7	150394341	150472121	233	30	120000	0.63863	0.26153	GIMAP5	76353	25kb
ENSG00000106565	7	150463373	150523448	255	34	120000	0.12292	0.45109	TMEM176B	76353	25kb
ENSG00000002933	7	150472491	150527208	222	38	120000	0.078698	0.46864	TMEM176A	76353	25kb
ENSG00000002726	7	150496715	150583592	437	33	120000	0.32262	0.37349	AOC1	76353	25kb
ENSG00000055118	7	150617049	150700403	168	27	120000	-0.61171	0.72964	KCNH2	76353	25kb
ENSG00000164867	7	150663083	150736676	166	36	120000	-0.36078	0.64087	NOS3	76353	25kb
ENSG00000181652	7	150684297	150746586	141	34	120000	-0.19921	0.57895	ATG9B	76353	25kb
ENSG00000197150	7	150700510	150769869	170	35	120000	-0.85953	0.80497	ABCB8	76353	25kb
ENSG00000213199	7	150720379	150774843	134	25	120000	-1.0476	0.85259	ASIC3	76353	25kb
ENSG00000164885	7	150725899	150780617	125	23	120000	-1.2411	0.89272	CDK5	76353	25kb
ENSG00000164889	7	150729297	150798614	164	25	120000	-1.3441	0.91053	SLC4A2	76353	25kb
ENSG00000164896	7	150748711	150802953	132	19	120000	-1.64	0.94949	FASTK	76353	25kb
ENSG00000164897	7	150753167	150805633	127	18	120000	-1.6121	0.94653	TMUB1	76353	25kb
ENSG00000133612	7	150757918	150866523	237	35	120000	-2.1438	0.98398	AGAP3	76353	25kb
ENSG00000164900	7	150820676	150896832	174	29	120000	-1.5006	0.93328	GBX1	76353	25kb
ENSG00000146926	7	150847785	150909919	154	30	120000	-0.9574	0.83082	ASB10	76353	25kb
ENSG00000033050	7	150879923	150949316	188	30	120000	-0.30905	0.62136	ABCF2	76353	25kb
ENSG00000033100	7	150904575	150960908	171	22	120000	-0.58993	0.72238	CHPF2	76353	25kb
ENSG00000082014	7	150910850	150999982	332	46	120000	-1.5671	0.94146	SMARCD3	76353	25kb
ENSG00000013374	7	151013785	151100535	311	46	120000	-0.71554	0.76286	NUB1	76353	25kb
ENSG00000187260	7	151047995	151132813	301	47	120000	-1.3719	0.91495	WDR86	76353	25kb
ENSG00000127377	7	151100921	151162899	190	35	120000	-0.39244	0.65263	CRYGN	76353	25kb
ENSG00000106615	7	151138098	151242206	224	31	120000	0.26046	0.39726	RHEB	76353	25kb
ENSG00000106617	7	151228197	151599210	1339	157	120000	1.1308	0.12906	PRKAG2	76353	25kb
ENSG00000106648	7	151628464	151742019	428	49	120000	-1.9538	0.97464	GALNTL5	76353	25kb
ENSG00000178234	7	151697759	151844425	227	18	120000	-0.040955	0.51633	GALNT11	76353	25kb
ENSG00000055609	7	151807010	152158090	388	18	120000	-0.15631	0.56211	KMT2C	76353	25kb
ENSG00000196584	7	152316864	152398250	340	34	120000	-0.17241	0.56844	XRCC2	76353	25kb
ENSG00000133627	7	152431834	152577463	373	35	120000	-0.092201	0.53673	ACTR3B	76353	25kb
ENSG00000130226	7	153559182	154710995	3820	338	120000	0.54434	0.2931	DPP6	76353	25kb
ENSG00000214106	7	154695208	154766618	170	21	120000	0.61187	0.27031	PAXIP1-AS2	76353	25kb

ENSG00000157212	7	154710397	154819794	209	16	120000	0.22627	0.4105	PAXIP1	76353	25kb
ENSG00000220575	7	154833777	154888267	207	18	120000	-1.2265	0.88999	HTR5A-AS1	76353	25kb
ENSG00000157219	7	154837034	154902459	269	15	120000	-0.96115	0.83176	HTR5A	76353	25kb
ENSG00000217825	7	154963273	155015165	299	25	120000	0.24571	0.40295	AC099552.4	76353	25kb
ENSG00000186480	7	155064486	155126945	260	26	120000	2.1225	0.016899	INSIG1	76353	25kb
ENSG00000204960	7	155124680	155185629	353	42	120000	1.1556	0.12392	BLACE	76353	25kb
ENSG00000218672	7	155149771	155214051	365	53	120000	0.13841	0.44496	AC008060.7	76353	25kb
ENSG00000164778	7	155225824	155282526	239	46	120000	-0.1292	0.5514	EN2	76353	25kb
ENSG00000146910	7	155241901	155351557	380	83	120000	-2.148	0.98414	CNPY1	76353	25kb
ENSG00000216895	7	155378925	155462899	227	27	120000	0.89106	0.18645	AC009403.2	76353	25kb
ENSG00000184863	7	155412145	155599179	477	19	120000	1.5159	0.064766	RBM33	76353	25kb
ENSG00000164690	7	155567680	155629967	194	41	120000	-0.80966	0.79093	SHH	76353	25kb
ENSG00000204876	7	155730326	155784037	242	46	120000	0.25504	0.39935	AC021218.2	76353	25kb
ENSG00000244291	7	156406057	156458348	146	24	120000	0.013993	0.49442	C7orf13	76353	25kb
ENSG00000105982	7	156407975	156494824	245	40	120000	0.21749	0.41391	RNF32	76353	25kb
ENSG00000105983	7	156436646	156710924	874	32	120000	0.12384	0.45072	LMBR1	76353	25kb
ENSG00000146909	7	156717417	156790876	377	25	120000	-0.16866	0.56697	NOM1	76353	25kb
ENSG00000130675	7	156761745	156828345	275	38	120000	-0.71172	0.76168	MNX1	76353	25kb
ENSG00000269446	7	156877674	156928764	139	25	120000	-0.52075	0.69873	AC006967.1	76353	25kb
ENSG00000009335	7	156906607	157087066	545	26	120000	1.0271	0.15218	UBE3C	76353	25kb
ENSG00000105993	7	157103075	157235133	589	42	120000	1.5354	0.062338	DNAJB6	76353	25kb
ENSG00000236669	7	157293477	157345019	205	23	120000	1.7587	0.039317	AC006372.1	76353	25kb
ENSG00000155093	7	157306750	158405480	3910	261	120000	1.2171	0.11179	PTPRN2	76353	25kb
ENSG00000222012	7	157381715	157436457	136	28	120000	2.0227	0.021551	AC005481.5	76353	25kb
ENSG00000146918	7	158399003	158522520	363	18	120000	2.3408	0.0096223	NCAPG2	76353	25kb
ENSG00000117868	7	158498686	158647944	604	22	120000	1.7203	0.042691	ESYT2	76353	25kb
ENSG00000126870	7	158624269	158774438	502	41	120000	1.5529	0.060218	WDR60	76353	25kb
ENSG00000106018	7	158795866	158962649	594	38	120000	0.16678	0.43377	VIPR2	76353	25kb
ENSG00000250210	8	117091	175563	60	10	120000	0.12361	0.45081	RP11-585F1.10	76353	25kb
ENSG00000172748	8	157137	222342	227	28	120000	-0.18335	0.57274	ZNF596	76353	25kb
ENSG00000147364	8	331428	446225	545	40	120000	0.65063	0.25764	FBXO25	76353	25kb
ENSG00000180190	8	414803	520781	493	43	120000	0.087267	0.46523	TDRP	76353	25kb
ENSG00000104714	8	539746	713106	895	46	120000	1.3084	0.095377	ERICH1	76353	25kb
ENSG00000198010	8	1424532	1681642	1094	69	120000	-1.2017	0.88527	DLGAP2	76353	25kb
ENSG00000182372	8	1678944	1759738	307	22	120000	0.37712	0.35304	CLN8	76353	25kb
ENSG00000104728	8	1747142	1931807	953	116	120000	-0.27894	0.60985	ARHGEF10	76353	25kb
ENSG00000176595	8	1897044	1980102	408	53	120000	0.34559	0.36483	KBTBD11	76353	25kb
ENSG00000036448	8	1968155	2138475	1346	95	120000	0.22646	0.41042	MYOM2	76353	25kb
ENSG00000183117	8	2767875	4877494	16631	617	120000	-0.36771	0.64345	CSMD1	76353	25kb
ENSG00000147316	8	6239113	6526144	1630	108	120000	0.56762	0.28515	MCPH1	76353	25kb
ENSG00000091879	8	6332172	6445930	675	63	120000	0.11936	0.4525	ANGPT2	76353	25kb
ENSG00000155189	8	6540878	6642184	439	46	120000	-0.0090169	0.5036	AGPAT5	76353	25kb
ENSG00000164825	8	6703097	6760544	454	45	120000	-0.95402	0.82996	DEFB1	76353	25kb
ENSG00000164822	8	6757215	6808598	336	51	120000	-0.40603	0.65764	DEFA6	76353	25kb
ENSG00000164821	8	6768344	6820860	281	52	120000	-1.1895	0.88287	DEFA4	76353	25kb
ENSG00000206047	8	6810171	6862602	123	24	120000	-1.4899	0.93187	DEFA1	76353	25kb
ENSG00000240247	8	6829288	6900778	173	27	120000	0.96858	0.16638	DEFA1B	76353	25kb
ENSG00000239839	8	6848391	6900823	171	27	120000	0.97461	0.16488	DEFA3	76353	25kb
ENSG00000164816	8	6887831	6939256	363	38	120000	0.21495	0.4149	DEFA5	76353	25kb
ENSG00000215372	8	7188039	7268080	2	1	120000	-0.74867	0.77297	ZNF705G	76353	25kb
ENSG00000182319	8	8150258	8269008	463	43	120000	-0.50163	0.69203	SGK223	76353	25kb
ENSG00000253958	8	8534448	8586616	252	14	120000	0.49972	0.30864	CLDN23	76353	25kb
ENSG00000147324	8	8615864	8776155	775	40	120000	-0.6265	0.73451	MFHAS1	76353	25kb
ENSG00000104626	8	8834657	8999256	650	34	120000	0.46986	0.31923	ERI1	76353	25kb
ENSG00000173281	8	8968765	9034084	212	26	120000	0.4578	0.32355	PPP1R3B	76353	25kb
ENSG00000253426	8	8984252	9050646	220	36	120000	1.3016	0.096531	RP11-10A14.4	76353	25kb
ENSG00000173273	8	9388424	9664856	1030	22	120000	-0.45041	0.67379	TNKS	76353	25kb
ENSG00000175806	8	9886778	10311401	1835	73	120000	-1.4604	0.92791	MSRA	76353	25kb
ENSG00000184647	8	10358056	10436676	374	28	120000	0.33153	0.37012	PRSS55	76353	25kb
ENSG00000183638	8	10438859	10594697	555	62	120000	-0.24215	0.59567	RP1L1	76353	25kb
ENSG00000171060	8	10505147	10583103	211	33	120000	-0.041535	0.51657	C8orf74	76353	25kb
ENSG00000171056	8	10556278	10722357	743	27	120000	1.2159	0.11201	SOX7	76353	25kb
ENSG00000258724	8	10557909	10722357	741	28	120000	1.2317	0.10904	SOX7	76353	25kb
ENSG00000254093	8	10597473	10722394	601	23	120000	1.2072	0.11368	PINX1	76353	25kb
ENSG00000171044	8	10728555	11083875	1217	42	120000	-1.9593	0.97496	XKR6	76353	25kb
ENSG00000215346	8	10958980	11012745	197	24	120000	-1.9883	0.97661	AF131215.5	76353	25kb
ENSG00000104643	8	11116925	11210646	289	17	120000	0.16692	0.43372	MTMR9	76353	25kb
ENSG00000177710	8	11163397	11214717	165	10	120000	-0.94527	0.82774	SLC35G5	76353	25kb
ENSG00000184608	8	11200911	11321167	580	41	120000	-1.4166	0.9217	C8orf12	76353	25kb
ENSG00000154319	8	11253972	11357224	526	42	120000	-1.4803	0.9306	FAM167A	76353	25kb
ENSG00000136573	8	11326510	11447113	551	32	120000	-1.8687	0.96917	BLK	76353	25kb
ENSG00000136574	8	11509468	11642511	503	49	120000	-0.52685	0.70085	GATA4	76353	25kb
ENSG00000255394	8	11593896	11645309	277	28	120000	0.01686	0.49327	C8orf49	76353	25kb
ENSG00000154328	8	11602148	11669855	396	36	120000	-0.41257	0.66004	NEIL2	76353	25kb
ENSG00000079459	8	11628082	11721818	550	36	120000	-0.92687	0.823	FDFT1	76353	25kb
ENSG00000255046	8	11630437	11685077	318	30	120000	-0.54325	0.70652	RP11-297N6.4	76353	25kb
ENSG00000164733	8	11675033	11751957	416	31	120000	-1.323	0.90709	CTSB	76353	25kb
ENSG00000205884	8	11806446	11857108	219	27	120000	-0.17905	0.57105	DEFB136	76353	25kb
ENSG00000205883	8	11814830	11867099	212	28	120000	-0.40705	0.65802	DEFB135	76353	25kb
ENSG00000205882	8	11825692	11878821	184	25	120000	-0.66383	0.7466	DEFB134	76353	25kb
ENSG00000255098	8	11845545	11898043	187	18	120000	-0.49576	0.68997	RP11-481A20.11	76353	25kb
ENSG00000233050	8	11896898	11954256	1	1	120000	-1.8767	0.96972	DEFB130	76353	25kb
ENSG00000215343	8	11936898	11998025	11	4	120000	-1.6004	0.94524	ZNF705D	76353	25kb
ENSG00000223443	8	11969677	12021586	13	3	120000	-1.1349	0.87179	USP17L2	76353	25kb
ENSG00000232948	8	12143471	12200825	7	2	120000	-0.074916	0.52986	DEFB130	76353	25kb
ENSG00000154359	8	12554403	12638582	341	31	120000	1.1301	0.12922	LONRF1	76353	25kb
ENSG00000250305	8	12778151	12914012	821	46	120000	-1.1275	0.87023	KIAA1456	76353	25kb
ENSG00000164741	8	12915870	13398167	2593	93	120000	-1.2969	0.90267	DLC1	76353	25kb
ENSG00000164743	8	13399352	13450796	318	28	120000	0.064731	0.47419	C8orf48	76353	25kb
ENSG00000185053	8	13922373	15120848	6422	200	120000	-1.7024	0.95566	SGCZ	76353	25kb
ENSG00000104723	8	15249724	15649158	1859	76	120000	0.13932	0.4446	TUSC3	76353	25kb
ENSG00000038945	8	15940387	16449999	1979	85	120000	1.9261	0.027047	MSR1	76353	25kb
ENSG00000078579	8	16824678	16884690	438	44	120000	0.14662	0.44172	FGF20	76353	25kb
ENSG00000155970	8	16859747	17005153	533	59	120000	-0.382	0.64877	MICU3	76353	25kb
ENSG00000104219	8	16988538	17107308	367	46	120000	0.18672	0.42594	ZDHHC2	76353	25kb
ENSG00000198791	8	17061737	17129387	182	23	120000	0.53466	0.29644	CNOT7	76353	25kb

ENSG00000155975	8	17079080	17184936	384	31	120000	0.77065	0.22046	VPS37A	76353	25kb
ENSG00000003987	8	17130539	17296037	755	49	120000	1.0735	0.14152	MTMR7	76353	25kb
ENSG00000003989	8	17329597	17453082	845	101	120000	-0.66071	0.7456	SLC7A2	76353	25kb
ENSG00000104213	8	17408942	17526580	861	72	120000	-1.2403	0.89256	PDGFRL	76353	25kb
ENSG00000129422	8	17476304	17683426	1227	90	120000	0.16535	0.43433	MTUS1	76353	25kb
ENSG00000104760	8	17696889	17792874	606	21	120000	-1.104	0.86519	FGL1	76353	25kb
ENSG00000078674	8	17755349	17910478	746	27	120000	-0.96306	0.83224	PCM1	76353	25kb
ENSG00000104763	8	17888934	17967494	429	37	120000	-1.4785	0.93036	ASAH1	76353	25kb
ENSG00000171428	8	18002986	18106198	578	41	120000	-1.6143	0.94677	NAT1	76353	25kb
ENSG00000156006	8	18223755	18283728	356	23	120000	-1.2849	0.90059	NAT2	76353	25kb
ENSG00000156011	8	18359811	18967240	3072	170	120000	-1.0526	0.85373	PSD3	76353	25kb
ENSG00000104611	8	19146128	19278729	455	55	120000	-0.92431	0.82234	SH2D4A	76353	25kb
ENSG00000147408	8	19236672	19640540	1629	96	120000	-0.7028	0.75891	CSGALNACT1	76353	25kb
ENSG00000104613	8	19649651	19734594	298	31	120000	1.1111	0.13326	INTS10	76353	25kb
ENSG00000175445	8	19734228	19849769	449	34	120000	1.5562	0.059832	LPL	76353	25kb
ENSG00000036565	8	19977366	20065717	303	40	120000	-1.2553	0.89531	SLC18A1	76353	25kb
ENSG00000147416	8	20029878	20109330	228	45	120000	-0.82302	0.79475	ATP6V1B2	76353	25kb
ENSG00000061337	8	20078676	20186474	345	58	120000	-0.045952	0.51833	LZTS1	76353	25kb
ENSG00000168546	8	21522915	21694869	422	69	120000	-0.62642	0.73448	GFRA2	76353	25kb
ENSG00000147443	8	21741384	21796371	139	21	120000	-0.59025	0.72249	DOK2	76353	25kb
ENSG00000130227	8	21752180	21889096	319	23	120000	0.11976	0.45234	XPO7	76353	25kb
ENSG00000158806	8	21856636	21919408	158	36	120000	0.59204	0.27691	NPM2	76353	25kb
ENSG00000158815	8	21874909	21931320	171	34	120000	1.0133	0.15546	FGF17	76353	25kb
ENSG00000158856	8	21881506	21965038	286	31	120000	0.7321	0.23205	DMTN	76353	25kb
ENSG00000158863	8	21921670	21987409	241	29	120000	0.52218	0.30077	FAM160B2	76353	25kb
ENSG00000173566	8	21939383	21991932	174	24	120000	0.090564	0.46392	NUDT18	76353	25kb
ENSG00000168453	8	21946928	22015897	188	25	120000	0.083554	0.46671	HR	76353	25kb
ENSG00000168476	8	21970533	22024464	149	29	120000	0.15822	0.43714	REEP4	76353	25kb
ENSG00000168481	8	21979338	22039597	158	36	120000	0.0022438	0.4991	LGI3	76353	25kb
ENSG00000168484	8	21989426	22046992	149	33	120000	0.28848	0.38649	SFTPC	76353	25kb
ENSG00000168487	8	21997249	22094839	291	50	120000	0.80025	0.21178	BMP1	76353	25kb
ENSG00000168490	8	22052222	22114854	200	38	120000	0.81933	0.2063	PHYHIP	76353	25kb
ENSG00000168495	8	22077617	22137113	162	18	120000	0.33934	0.36717	POLR3D	76353	25kb
ENSG00000197181	8	22107810	22240076	338	23	120000	0.093067	0.46293	PIWIL2	76353	25kb
ENSG00000104635	8	22199762	22316642	364	26	120000	-0.20443	0.58099	SLC39A14	76353	25kb
ENSG00000120910	8	22273332	22423652	302	15	120000	1.1083	0.13386	PPP3CC	76353	25kb
ENSG00000120896	8	22377499	22458301	171	31	120000	0.77983	0.21775	SORBS3	76353	25kb
ENSG00000120913	8	22410792	22480538	179	27	120000	-0.20023	0.57935	PDLIM2	76353	25kb
ENSG00000248235	8	22421787	22484597	174	22	120000	-0.43175	0.66704	AC037459.4	76353	25kb
ENSG00000241852	8	22432114	22486663	159	17	120000	-0.4487	0.67317	C8orf58	76353	25kb
ENSG00000158941	8	22437145	22504027	196	20	120000	-0.62257	0.73322	CCAR2	76353	25kb
ENSG00000147439	8	22452931	22551661	240	21	120000	-0.57331	0.71678	BIN3	76353	25kb
ENSG00000179388	8	22520172	22575815	141	28	120000	0.59673	0.27534	EGR3	76353	25kb
ENSG00000134020	8	22545769	22882513	1016	112	120000	0.28351	0.38839	PEBP4	76353	25kb
ENSG00000008853	8	22819930	22902712	222	43	120000	-1.0688	0.85741	RHOBTB2	76353	25kb
ENSG00000120889	8	22852646	22951692	278	43	120000	-1.0159	0.84517	TNFRSF10B	76353	25kb
ENSG00000173535	8	22916868	22999950	239	41	120000	-0.95558	0.83036	TNFRSF10C	76353	25kb
ENSG00000173530	8	22968101	23046543	200	47	120000	-1.2703	0.89801	TNFRSF10D	76353	25kb
ENSG00000104689	8	23022965	23107639	266	32	120000	-0.59286	0.72336	TNFRSF10A	76353	25kb
ENSG00000147457	8	23076150	23144512	252	32	120000	-0.93514	0.82514	CHMP7	76353	25kb
ENSG00000104679	8	23102633	23178792	318	39	120000	-1.0418	0.85124	R3HCC1	76353	25kb
ENSG00000134013	8	23129702	23307841	805	53	120000	-0.96358	0.83237	LOXL2	76353	25kb
ENSG00000197217	8	23218296	23340208	406	31	120000	-1.3644	0.91378	ENTPD4	76353	25kb
ENSG00000147454	8	23361318	23457976	535	37	120000	0.025603	0.48979	SLC25A37	76353	25kb
ENSG00000268085	8	23387287	23445651	369	30	120000	0.21012	0.41679	AC051642.1	76353	25kb
ENSG00000215298	8	23405157	23457974	321	25	120000	0.47158	0.31861	FP15737	76353	25kb
ENSG00000167034	8	23511206	23565440	189	30	120000	-0.58088	0.71934	NKX3-1	76353	25kb
ENSG00000180053	8	23534964	23589111	213	35	120000	-0.1589	0.56313	NKX2-6	76353	25kb
ENSG00000159167	8	23674428	23737320	221	29	120000	1.5356	0.062319	STC1	76353	25kb
ENSG00000042980	8	24126553	24241531	423	21	120000	0.38112	0.35156	ADAM28	76353	25kb
ENSG00000134028	8	24216798	24288526	208	24	120000	-0.11226	0.54469	ADAMDEC1	76353	25kb
ENSG00000069206	8	24273443	24409483	391	26	120000	-0.60776	0.72833	ADAM7	76353	25kb
ENSG00000104722	8	24745525	24801607	129	17	120000	-0.20393	0.5808	NEFM	76353	25kb
ENSG00000147459	8	25017238	25300598	747	70	120000	-0.22338	0.58838	DOCK5	76353	25kb
ENSG00000147437	8	25251776	25307170	123	10	120000	0.99345	0.16025	GNRH1	76353	25kb
ENSG00000104756	8	25260366	25340992	208	13	120000	1.0942	0.13693	KCTD9	76353	25kb
ENSG00000184661	8	25291513	25390436	272	13	120000	0.96994	0.16604	CDCA2	76353	25kb
ENSG00000221818	8	25674246	25927913	758	80	120000	-0.9054	0.81737	EBF2	76353	25kb
ENSG00000221914	8	26124007	26255196	324	35	120000	-0.77387	0.7805	PPP2R2A	76353	25kb
ENSG00000104765	8	26215414	26388152	606	59	120000	-0.63331	0.73673	BNIP3L	76353	25kb
ENSG00000240694	8	26337202	26396608	231	30	120000	-0.36637	0.64295	PNMA2	76353	25kb
ENSG00000092964	8	26346791	26540694	781	38	120000	-1.5235	0.93618	DPYSL2	76353	25kb
ENSG00000120907	8	26580667	26749790	749	76	120000	-2.3024	0.98934	ADRA1A	76353	25kb
ENSG00000253903	8	26918694	26969766	249	27	120000	-0.53815	0.70476	RP11-521M14.2	76353	25kb
ENSG00000015592	8	27067840	27140937	197	29	120000	-1.7482	0.95979	STMN4	76353	25kb
ENSG00000104228	8	27117404	27193836	145	20	120000	-0.83528	0.79822	TRIM35	76353	25kb
ENSG00000120899	8	27143999	27341903	552	31	120000	-1.8542	0.96815	PTK2B	76353	25kb
ENSG00000120903	8	27292279	27362400	232	30	120000	-1.661	0.95164	CHRNA2	76353	25kb
ENSG00000120915	8	27323296	27428081	320	30	120000	-1.0519	0.85358	EPHX2	76353	25kb
ENSG00000120885	8	27429434	27497548	191	31	120000	1.3921	0.081939	CLU	76353	25kb
ENSG00000168077	8	27466385	27559293	300	21	120000	2.6155	0.0044543	SCARA3	76353	25kb
ENSG00000147419	8	27565835	27655170	308	17	120000	0.40783	0.3417	CCDC25	76353	25kb
ENSG00000171320	8	27604466	27695157	360	15	120000	0.87504	0.19078	ESCO2	76353	25kb
ENSG00000168078	8	27642137	27720612	340	12	120000	0.64269	0.26021	PBK	76353	25kb
ENSG00000168079	8	27702399	27875244	725	66	120000	-0.49668	0.69029	SCARA5	76353	25kb
ENSG00000189233	8	27854481	27966388	295	45	120000	0.33946	0.36713	NUGGC	76353	25kb
ENSG00000134014	8	27922190	28073673	386	31	120000	0.34002	0.36692	ELP3	76353	25kb
ENSG00000168081	8	28149503	28225872	219	27	120000	-0.50231	0.69228	PNOC	76353	25kb
ENSG00000186918	8	28178102	28285218	203	21	120000	-0.74867	0.77297	ZNF395	76353	25kb
ENSG00000214050	8	28180804	28372835	390	55	120000	-0.58293	0.72003	FBXO16	76353	25kb
ENSG00000104290	8	28326729	28456775	272	19	120000	0.86851	0.19256	FZD3	76353	25kb
ENSG00000012232	8	28432986	28638116	428	50	120000	-0.07145	0.52848	EXTL3	76353	25kb
ENSG00000104299	8	28600178	28772759	290	25	120000	1.079	0.1403	INTS9	76353	25kb
ENSG00000147421	8	28722911	28947281	269	23	120000	2.8809	0.001983	HMBOX1	76353	25kb
ENSG00000197892	8	28899796	29145641	475	42	120000	0.66775	0.25215	KIF13B	76353	25kb
ENSG00000120875	8	29165581	29233185	220	22	120000	-1.069	0.85747	DUSP4	76353	25kb

ENSG00000133872	8	29895528	29965723	157	17	120000	-0.70745	0.76036	TMEM66	76353	25kb
ENSG00000104660	8	29927914	30059724	279	19	120000	-0.59593	0.72439	LEPROTL1	76353	25kb
ENSG00000177669	8	29964340	30027202	138	17	120000	-0.75914	0.77612	MBOAT4	76353	25kb
ENSG00000104671	8	29988813	30066156	162	13	120000	-0.33598	0.63156	DCTN6	76353	25kb
ENSG00000157110	8	30216944	30454778	473	37	120000	0.051215	0.47958	RBPMS	76353	25kb
ENSG00000197265	8	30410835	30540768	260	22	120000	-0.62324	0.73344	GTF2E2	76353	25kb
ENSG00000253457	8	30471117	30528581	125	17	120000	-0.70244	0.7588	SMIM18	76353	25kb
ENSG00000104687	8	30510583	30610443	260	28	120000	0.03585	0.4857	GSR	76353	25kb
ENSG00000104695	8	30606973	30696830	201	21	120000	-0.98375	0.83738	PPP2CB	76353	25kb
ENSG00000133863	8	30664060	30773122	237	19	120000	-1.1277	0.87028	TEX15	76353	25kb
ENSG00000172733	8	30828321	30916231	183	26	120000	-1.0531	0.85385	PURG	76353	25kb
ENSG00000165392	8	30866317	31056285	401	36	120000	0.69335	0.24405	WRN	76353	25kb
ENSG00000157168	8	31471902	32647548	3743	153	120000	0.95228	0.17048	NRG1	76353	25kb
ENSG00000172728	8	33203342	33355940	424	28	120000	-0.83808	0.79901	FUT10	76353	25kb
ENSG00000129696	8	33305904	33396119	277	14	120000	-0.24244	0.59578	TTI2	76353	25kb
ENSG00000198042	8	33317268	33383778	204	14	120000	-0.18098	0.57181	MAK16	76353	25kb
ENSG00000133874	8	33380273	33449643	200	18	120000	-1.3947	0.91845	RNF122	76353	25kb
ENSG00000133878	8	33423856	33482624	153	13	120000	-0.72248	0.765	DUSP26	76353	25kb
ENSG00000156687	8	35067975	35679068	1187	57	120000	-0.032659	0.51303	UNC5D	76353	25kb
ENSG00000233863	8	35624365	35678384	71	5	120000	-1.2787	0.8995	AC012215.1	76353	25kb
ENSG00000215262	8	36616842	36818646	327	24	120000	-0.85372	0.80337	KCNU1	76353	25kb
ENSG00000183779	8	37528269	37582537	38	19	120000	-0.053063	0.52116	ZNF703	76353	25kb
ENSG00000183154	8	37567279	37619944	32	18	120000	0.099394	0.46041	RP11-863K10.7	76353	25kb
ENSG00000147475	8	37569117	37641619	40	22	120000	-0.098755	0.53933	ERLIN2	76353	25kb
ENSG00000147471	8	37595111	37662283	56	15	120000	0.60189	0.27362	PROSC	76353	25kb
ENSG00000020181	8	37616709	37727414	156	40	120000	-0.62219	0.73309	GPR124	76353	25kb
ENSG00000104221	8	37675786	37732422	73	20	120000	-1.6107	0.94638	BRF2	76353	25kb
ENSG00000156675	8	37691136	37781985	82	24	120000	-2.3056	0.98943	RAB11FIP1	76353	25kb
ENSG00000169154	8	37766799	37822647	71	9	120000	-0.17028	0.5676	GOT1L1	76353	25kb
ENSG00000188778	8	37795516	37849483	71	8	120000	-0.21987	0.58701	ADRB3	76353	25kb
ENSG00000187840	8	37862859	37942883	128	17	120000	-1.441	0.92521	EIF4EBP1	76353	25kb
ENSG00000129691	8	37937760	38026594	122	19	120000	-0.99266	0.83956	ASH2L	76353	25kb
ENSG00000147465	8	37976167	38033783	84	22	120000	-0.69355	0.75602	STAR	76353	25kb
ENSG00000175324	8	37995839	38059248	93	12	120000	0.42317	0.33608	LSM1	76353	25kb
ENSG00000156735	8	38009051	38095819	104	11	120000	0.49232	0.31125	BAG4	76353	25kb
ENSG00000085788	8	38057736	38158076	104	11	120000	0.96486	0.16731	DDHD2	76353	25kb
ENSG00000147535	8	38095648	38151761	73	9	120000	1.1442	0.12628	PPAPDC1B	76353	25kb
ENSG00000147548	8	38102215	38264790	198	15	120000	0.97808	0.16402	WHSC1L1	76353	25kb
ENSG00000165046	8	38218725	38292045	88	10	120000	0.93374	0.17522	LETM2	76353	25kb
ENSG00000077782	8	38243656	38351352	180	28	120000	-0.013493	0.50538	FGFR1	76353	25kb
ENSG00000196166	8	38343352	38411180	169	22	120000	0.23839	0.40579	C8orf86	76353	25kb
ENSG00000147526	8	38560704	38735546	361	34	120000	0.09205	0.46333	TACC1	76353	25kb
ENSG00000169499	8	38733753	38856428	313	51	120000	-2.0794	0.98121	PLEKHA2	76353	25kb
ENSG00000169495	8	38806683	38871181	145	17	120000	-0.85257	0.80305	HTRA4	76353	25kb
ENSG00000169490	8	38821327	38879343	124	13	120000	-0.83287	0.79754	TM2D2	76353	25kb
ENSG00000168615	8	38829388	38987663	328	22	120000	-0.58207	0.71974	ADAM9	76353	25kb
ENSG00000197140	8	38939509	39167430	491	24	120000	0.36646	0.35701	ADAM32	76353	25kb
ENSG00000168619	8	39417008	39612583	414	32	120000	1.3147	0.094302	ADAM18	76353	25kb
ENSG00000104755	8	39576254	39720808	317	29	120000	0.21238	0.4159	ADAM2	76353	25kb
ENSG00000131203	8	39734794	39810963	257	22	120000	-0.23515	0.59296	IDO1	76353	25kb
ENSG00000188676	8	39767133	39898910	552	39	120000	-0.6332	0.7367	IDO2	76353	25kb
ENSG00000176907	8	39985989	40037821	138	30	120000	-0.61102	0.72941	C8orf4	76353	25kb
ENSG00000165061	8	40363109	40780352	1452	99	120000	1.4071	0.079696	ZMAT4	76353	25kb
ENSG00000104332	8	41094481	41192016	233	31	120000	-0.81544	0.79259	SFRP1	76353	25kb
ENSG00000147533	8	41322915	41393499	286	25	120000	-0.40482	0.6572	GOLGA7	76353	25kb
ENSG00000147536	8	41361725	41427565	231	33	120000	0.89746	0.18474	GINS4	76353	25kb
ENSG00000158669	8	41409706	41507520	309	23	120000	0.74476	0.22821	AGPAT6	76353	25kb
ENSG00000165066	8	41477697	41533855	210	29	120000	-0.13458	0.55353	NKX6-3	76353	25kb
ENSG00000029534	8	41485739	41779280	910	84	120000	0.17795	0.42938	ANK1	76353	25kb
ENSG00000083168	8	41761997	41934508	250	22	120000	1.3964	0.081294	KAT6A	76353	25kb
ENSG00000070718	8	41985464	42054191	103	10	120000	0.87412	0.19103	AP3M2	76353	25kb
ENSG00000104368	8	42007236	42090242	124	15	120000	0.52492	0.29982	PLAT	76353	25kb
ENSG00000104365	8	42103820	42214973	190	20	120000	-0.28224	0.61112	IKBKB	76353	25kb
ENSG00000070501	8	42170972	42254326	152	17	120000	-0.15509	0.56162	POLB	76353	25kb
ENSG00000104371	8	42206586	42259750	100	13	120000	-0.3175	0.62457	DKK4	76353	25kb
ENSG00000078668	8	42224142	42288415	112	16	120000	-0.71332	0.76218	VDAC3	76353	25kb
ENSG00000168575	8	42248993	42422069	342	31	120000	1.2241	0.11046	SLC20A2	76353	25kb
ENSG00000176209	8	42371298	42434603	179	16	120000	1.4408	0.074819	SMIM19	76353	25kb
ENSG00000147432	8	42527519	42617550	326	12	120000	-0.51451	0.69655	CHRNB3	76353	25kb
ENSG00000147434	8	42582763	42676535	205	26	120000	1.4021	0.08044	CHRNA6	76353	25kb
ENSG00000131931	8	42666817	42723468	95	15	120000	0.19743	0.42174	THAP1	76353	25kb
ENSG00000120925	8	42679780	42777433	158	16	120000	-0.10787	0.54295	RNF170	76353	25kb
ENSG00000168172	8	42727075	42910682	326	13	120000	-0.034043	0.51358	HOOK3	76353	25kb
ENSG00000254673	8	42848567	42957477	167	16	120000	0.13933	0.44459	RP11-598P20.5	76353	25kb
ENSG00000168522	8	42864337	42965931	147	15	120000	0.20841	0.41746	FNTA	76353	25kb
ENSG00000185900	8	42923658	43003577	127	15	120000	0.26367	0.39602	POMK	76353	25kb
ENSG00000165102	8	42970556	43082998	159	24	120000	-0.1224	0.54871	HGSNAT	76353	25kb
ENSG00000164808	8	48148167	48673868	430	38	120000	-0.9086	0.81822	SPIDR	76353	25kb
ENSG00000221869	8	48624471	48676648	40	14	120000	-0.21847	0.58647	CEBPD	76353	25kb
ENSG00000253729	8	48660669	48897743	198	27	120000	-0.69882	0.75767	PRKDC	76353	25kb
ENSG00000104738	8	48847745	48915720	40	13	120000	-0.39552	0.65377	MCM4	76353	25kb
ENSG00000169139	8	48895960	49002268	68	17	120000	-0.8601	0.80513	UBE2V2	76353	25kb
ENSG00000034239	8	49598348	49672870	115	24	120000	1.929	0.026867	EFCAB1	76353	25kb
ENSG00000019549	8	49805249	49859299	81	21	120000	2.6019	0.0046348	SNAI2	76353	25kb
ENSG00000168333	8	49941870	50013649	148	14	120000	-0.47976	0.6843	C8orf22	76353	25kb
ENSG00000253206	8	50423945	50491973	126	9	120000	-0.46951	0.68065	RP11-738G5.2	76353	25kb
ENSG00000147481	8	50797349	51731678	2800	65	120000	-1.1199	0.86861	SNTG1	76353	25kb
ENSG00000147485	8	52207138	52747005	1909	66	120000	-0.77502	0.78084	PXDNL	76353	25kb
ENSG00000168300	8	52705140	52836735	471	19	120000	-0.22172	0.58773	PCMTD1	76353	25kb
ENSG00000232941	8	52705143	52755969	180	15	120000	-0.16951	0.5673	AC090186.1	76353	25kb
ENSG00000147488	8	52998399	53398519	1096	90	120000	-0.26205	0.60336	ST18	76353	25kb
ENSG00000196711	8	53421597	53503067	149	18	120000	0.69029	0.24501	FAM150A	76353	25kb
ENSG00000023287	8	53510016	53683403	218	31	120000	0.18516	0.42655	RB1CC1	76353	25kb
ENSG00000183729	8	53825991	53878677	274	14	120000	-0.12732	0.55066	NPBWR1	76353	25kb
ENSG00000082556	8	54113284	54189257	336	22	120000	0.19614	0.42225	OPRK1	76353	25kb
ENSG00000047249	8	54603117	54781118	217	15	120000	0.53817	0.29523	ATP6V1H	76353	25kb

ENSG00000147509	8	54739368	54896863	214	32	120000	1.251	0.10546	RGS20	76353	25kb
ENSG00000187735	8	54854112	54960089	184	13	120000	2.0648	0.01947	TCEA1	76353	25kb
ENSG00000120992	8	54933938	55039577	187	11	120000	2.1744	0.014837	LYPLA1	76353	25kb
ENSG00000137547	8	55022770	55085461	124	11	120000	0.96644	0.16691	MRPL15	76353	25kb
ENSG00000164736	8	55345495	55398448	135	23	120000	-0.39823	0.65477	SOX17	76353	25kb
ENSG00000104237	8	55503627	55568394	116	17	120000	-1.002	0.84183	RP1	76353	25kb
ENSG00000206579	8	55989949	56479613	1415	94	120000	-0.49325	0.68908	XKR4	76353	25kb
ENSG00000253976	8	56022763	56079918	158	26	120000	0.97983	0.16358	RP11-386G21.1	76353	25kb
ENSG00000253857	8	56049043	56102287	101	21	120000	1.3551	0.087693	RP11-386G21.2	76353	25kb
ENSG00000167904	8	56583983	56710966	331	30	120000	1.5429	0.061433	TMEM68	76353	25kb
ENSG00000137574	8	56660701	56763007	280	17	120000	1.3673	0.085764	TGS1	76353	25kb
ENSG00000254087	8	56767372	56948940	572	38	120000	-0.031097	0.5124	LYN	76353	25kb
ENSG00000008988	8	56954854	57012069	175	11	120000	-1.5576	0.94033	RPS20	76353	25kb
ENSG00000172680	8	57000501	57051541	142	13	120000	-1.5302	0.93701	MOS	76353	25kb
ENSG00000181690	8	57048463	57148883	155	19	120000	-0.20738	0.58214	PLAG1	76353	25kb
ENSG00000170791	8	57099245	57156357	70	16	120000	-0.073232	0.52919	CHCHD7	76353	25kb
ENSG00000170786	8	57187569	57258335	187	16	120000	-0.47564	0.68283	SDR16C5	76353	25kb
ENSG00000181195	8	57324233	57384293	187	15	120000	0.20646	0.41821	PENK	76353	25kb
ENSG00000104331	8	57845492	57931403	181	21	120000	-1.2382	0.89219	IMPAD1	76353	25kb
ENSG00000169122	8	58882068	59141838	694	59	120000	-0.28156	0.61086	FAM110B	76353	25kb
ENSG00000215114	8	59298823	59389060	269	24	120000	-0.61629	0.73115	UBXN2B	76353	25kb
ENSG00000167910	8	59377737	59437795	158	22	120000	-1.5692	0.9417	CYP7A1	76353	25kb
ENSG00000137575	8	59440483	59520419	166	12	120000	-0.56392	0.7136	SDCBP	76353	25kb
ENSG00000035681	8	59471063	59597403	249	26	120000	-0.5591	0.71195	NSMAF	76353	25kb
ENSG00000198846	8	59692977	60056767	977	97	120000	0.4822	0.31483	TOX	76353	25kb
ENSG00000178538	8	61074906	61218971	481	35	120000	-0.91234	0.8192	CA8	76353	25kb
ENSG00000104388	8	61404416	61561186	313	13	120000	-0.69667	0.75699	RAB2A	76353	25kb
ENSG00000171316	8	61566337	61804465	464	28	120000	-0.066177	0.52638	CHD7	76353	25kb
ENSG00000177182	8	61944717	62439204	1437	83	120000	0.76436	0.22233	CLVS1	76353	25kb
ENSG00000198363	8	62388116	62652155	677	45	120000	0.27577	0.39136	ASPH	76353	25kb
ENSG00000185942	8	63136150	63937211	2397	72	120000	0.64267	0.26022	NKAIN3	76353	25kb
ENSG00000137563	8	63902638	63976730	286	18	120000	-0.15564	0.56184	GGH	76353	25kb
ENSG00000137561	8	63936112	64023612	291	21	120000	0.56632	0.28559	TTPA	76353	25kb
ENSG00000185728	8	64056112	64150346	179	20	120000	-0.17146	0.56807	YTHDF3	76353	25kb
ENSG00000180828	8	65467814	65521181	91	16	120000	0.55458	0.28959	BHLHE22	76353	25kb
ENSG00000172817	8	65475320	65736318	513	27	120000	-0.1692	0.56718	CYP7B1	76353	25kb
ENSG00000104442	8	66489694	66571442	263	12	120000	0.0064843	0.49741	ARMC1	76353	25kb
ENSG00000066855	8	66531969	66708496	456	29	120000	0.14258	0.44331	MTFR1	76353	25kb
ENSG00000205268	8	66604745	66779557	430	28	120000	-0.24761	0.59778	PDE7A	76353	25kb
ENSG00000147570	8	66908795	67037751	435	41	120000	-1.1572	0.87641	DNAJC5B	76353	25kb
ENSG00000147573	8	67014131	67112720	250	19	120000	-0.24929	0.59843	TRIM55	76353	25kb
ENSG00000147571	8	67063620	67115960	133	11	120000	-0.1008	0.54014	CRH	76353	25kb
ENSG00000179041	8	67316263	67367966	148	23	120000	-0.47502	0.68261	RRS1	76353	25kb
ENSG00000147576	8	67317420	67408836	289	29	120000	-0.74295	0.77125	ADHFE1	76353	25kb
ENSG00000169085	8	67347238	67455759	282	37	120000	-0.63952	0.73876	C8orf46	76353	25kb
ENSG00000185697	8	67449410	67551482	135	11	120000	0.51672	0.30268	MYBL1	76353	25kb
ENSG00000175073	8	67515722	67604452	118	11	120000	0.68611	0.24632	VCPIP1	76353	25kb
ENSG00000270024	8	67554831	67797707	374	15	120000	1.7358	0.041304	C8orf44-SGK3	76353	25kb
ENSG00000213865	8	67554850	67622799	95	11	120000	0.59759	0.27506	C8orf44	76353	25kb
ENSG00000104205	8	67599653	67799257	321	13	120000	1.799	0.036012	SGK3	76353	25kb
ENSG00000178460	8	67757984	67859283	123	14	120000	1.4559	0.072708	MCMDC2	76353	25kb
ENSG00000261787	8	67833736	67899825	66	10	120000	1.23	0.10934	TCF24	76353	25kb
ENSG00000178125	8	67851334	67993839	149	13	120000	0.99895	0.15891	PPP1R42	76353	25kb
ENSG00000121022	8	67930314	68021018	104	9	120000	0.97297	0.16528	COPS5	76353	25kb
ENSG00000104218	8	67949661	68133498	211	18	120000	0.93804	0.17411	CSPP1	76353	25kb
ENSG00000066777	8	68060747	68280912	247	29	120000	1.0887	0.13814	ARFGEF1	76353	25kb
ENSG00000165078	8	68309360	68683620	920	76	120000	-0.8811	0.81087	CPA6	76353	25kb
ENSG00000046889	8	68839353	69174265	1103	93	120000	0.26887	0.39401	PREX2	76353	25kb
ENSG00000248801	8	69190703	69268726	185	24	120000	-0.93093	0.82405	RP11-664D7.4	76353	25kb
ENSG00000165084	8	69217957	69756257	1478	99	120000	-1.2645	0.89697	C8orf34	76353	25kb
ENSG00000137573	8	70353859	70598150	694	104	120000	-1.5285	0.93681	SULF1	76353	25kb
ENSG00000137571	8	70554282	70772299	595	59	120000	-1.4974	0.93286	SLCO5A1	76353	25kb
ENSG00000269197	8	70825403	70875635	178	25	120000	-0.73853	0.7699	AC090574.1	76353	25kb
ENSG00000147596	8	70938886	71008928	189	31	120000	-0.25527	0.60074	PRDM14	76353	25kb
ENSG00000140396	8	70996997	71341040	546	24	120000	-0.72734	0.76649	NCOA2	76353	25kb
ENSG00000067167	8	71460677	71545622	203	20	120000	-0.2632	0.6038	TRAM1	76353	25kb
ENSG00000213002	8	71460681	71511671	105	15	120000	-0.070177	0.52797	AC120194.1	76353	25kb
ENSG00000246366	8	71495812	71600514	275	18	120000	-0.90417	0.81705	RP11-382J12.1	76353	25kb
ENSG00000147592	8	71522553	71606409	239	21	120000	-0.99559	0.84028	LACTB2	76353	25kb
ENSG00000221947	8	71556600	71727606	502	18	120000	-1.2471	0.89382	XKR9	76353	25kb
ENSG00000104313	8	72084668	72299467	549	80	120000	0.8103	0.20888	EYA1	76353	25kb
ENSG00000253379	8	72290675	72529260	563	43	120000	-0.47225	0.68163	RP11-1102P16.1	76353	25kb
ENSG00000235531	8	72715402	73055628	1330	87	120000	0.45224	0.32555	RP11-383H13.1	76353	25kb
ENSG00000178860	8	72728784	72781703	176	28	120000	-0.10189	0.54058	MSC	76353	25kb
ENSG00000104321	8	72907152	73012852	456	39	120000	-0.90251	0.81661	TRPA1	76353	25kb
ENSG00000182674	8	73424626	73875584	1290	72	120000	-0.23258	0.59196	KCNB2	76353	25kb
ENSG00000147601	8	73896099	73985357	180	19	120000	-0.64418	0.74027	TERF1	76353	25kb
ENSG00000164764	8	73951775	74061323	318	31	120000	-0.02645	0.51055	SBSPON	76353	25kb
ENSG00000147604	8	74177506	74233024	157	21	120000	-0.76461	0.77775	RPL7	76353	25kb
ENSG00000121039	8	74181847	74262516	219	28	120000	-0.42486	0.66453	RDH10	76353	25kb
ENSG00000040341	8	74307604	74684943	1045	49	120000	-0.61865	0.73193	STAU2	76353	25kb
ENSG00000258677	8	74575926	74816089	454	28	120000	-2.0889	0.98164	RP11-463D19.2	76353	25kb
ENSG00000104343	8	74667332	74816145	221	26	120000	-1.383	0.91667	UBE2W	76353	25kb
ENSG00000154582	8	74826404	74909522	252	24	120000	-0.17144	0.56806	TCEB1	76353	25kb
ENSG00000175606	8	74859672	74920018	196	18	120000	-0.11912	0.54741	TMEM70	76353	25kb
ENSG00000154589	8	74878587	74966322	244	24	120000	-0.21044	0.58334	LY96	76353	25kb
ENSG00000104369	8	75121935	75258563	345	34	120000	-1.1121	0.86696	JPH1	76353	25kb
ENSG00000104381	8	75208365	75426107	489	43	120000	-0.27596	0.60871	GDAP1	76353	25kb
ENSG00000254349	8	75487010	75760548	676	45	120000	0.1553	0.43829	RP11-758M4.1	76353	25kb
ENSG00000137558	8	75711772	75792264	192	21	120000	0.7264	0.2338	PI15	76353	25kb
ENSG00000121005	8	75871750	75971793	317	23	120000	0.33355	0.36936	CRISPLD1	76353	25kb
ENSG00000164749	8	76295149	76504078	786	38	120000	-1.0499	0.85311	HNF4G	76353	25kb
ENSG00000091656	8	77568454	77804521	499	49	120000	-1.1521	0.87537	ZFHX4	76353	25kb
ENSG00000164751	8	77867494	77938280	190	24	120000	0.26127	0.39694	PEX2	76353	25kb
ENSG00000171033	8	79403374	79542502	324	20	120000	-0.74234	0.77106	PKIA	76353	25kb
ENSG00000104427	8	79553282	79657000	246	17	120000	0.77505	0.21915	ZC2HC1A	76353	25kb

ENSG00000104432	8	79562978	79742758	452	24	120000	1.5658	0.058702	IL7	76353	25kb
ENSG00000104435	8	80498049	80603410	331	34	120000	-0.19861	0.57872	STMN2	76353	25kb
ENSG00000164683	8	80651245	80705098	116	26	120000	-0.71982	0.76418	HEY1	76353	25kb
ENSG00000147586	8	80805952	80967524	208	22	120000	0.2601	0.39739	MRPS28	76353	25kb
ENSG00000076554	8	80845571	81168467	760	37	120000	-0.023492	0.50937	TPD52	76353	25kb
ENSG00000205189	8	81372854	81463500	240	34	120000	0.076918	0.46934	ZBTB10	76353	25kb
ENSG00000164684	8	81515686	81812016	435	25	120000	1.0673	0.14292	ZNF704	76353	25kb
ENSG00000076641	8	81855045	82049303	502	65	120000	0.65472	0.25632	PAG1	76353	25kb
ENSG00000164687	8	82167598	82222012	85	12	120000	-0.061182	0.52439	FABP5	76353	25kb
ENSG00000147588	8	82327561	82384758	161	15	120000	-1.6698	0.95252	PMP2	76353	25kb
ENSG00000205186	8	82345576	82398814	163	15	120000	-1.2502	0.89438	FABP9	76353	25kb
ENSG00000170323	8	82365654	82420498	158	19	120000	-1.1274	0.8702	FABP4	76353	25kb
ENSG00000197416	8	82412216	82468613	136	25	120000	-0.16447	0.56532	FABP12	76353	25kb
ENSG00000133731	8	82545196	82623928	252	20	120000	-1.9019	0.97141	IMPA1	76353	25kb
ENSG00000253598	8	82580842	82633409	166	20	120000	-1.5227	0.93608	SLC10A5	76353	25kb
ENSG00000104231	8	82588569	82670138	258	18	120000	-1.9159	0.97231	ZFAND1	76353	25kb
ENSG00000164695	8	82619669	82696750	231	31	120000	-2.1862	0.9856	CHMP4C	76353	25kb
ENSG00000104497	8	82686816	82780101	349	18	120000	-1.8847	0.97027	SNX16	76353	25kb
ENSG00000184672	8	85070022	85859079	1844	56	120000	-1.0634	0.8562	RALYL	76353	25kb
ENSG00000133739	8	85994382	86083311	155	15	120000	1.0281	0.15196	LRRCC1	76353	25kb
ENSG00000133740	8	86064460	86154387	128	14	120000	1.9993	0.02279	E2F5	76353	25kb
ENSG00000176731	8	86101311	86157650	76	10	120000	2.2979	0.010783	C8orf59	76353	25kb
ENSG00000185015	8	86107816	86221302	167	15	120000	2.5254	0.0057789	CA13	76353	25kb
ENSG00000258256	8	86109675	86182946	95	11	120000	2.5292	0.0057166	RP11-219B4.5	76353	25kb
ENSG00000257962	8	86133524	86187077	74	10	120000	2.5495	0.0053935	RP11-219B4.6	76353	25kb
ENSG00000133742	8	86214837	86316243	192	26	120000	0.98762	0.16167	CA1	76353	25kb
ENSG00000164879	8	86260665	86386269	227	25	120000	-0.090861	0.5362	CA3	76353	25kb
ENSG00000104267	8	86351081	86418722	91	15	120000	0.51963	0.30166	CA2	76353	25kb
ENSG00000205176	8	86543695	86600726	16	4	120000	-0.11884	0.5473	REXO1L1P	76353	25kb
ENSG00000147614	8	86974552	87191457	613	30	120000	0.001596	0.49936	ATP6V0D2	76353	25kb
ENSG00000147613	8	87035602	87125850	207	22	120000	0.10488	0.45823	PSKH2	76353	25kb
ENSG00000164893	8	87201281	87358375	464	33	120000	0.88877	0.18706	SLC7A13	76353	25kb
ENSG00000123124	8	87329967	87515649	518	21	120000	0.33573	0.36854	WWP1	76353	25kb
ENSG00000176623	8	87455486	87551586	289	18	120000	-0.17823	0.57073	RMDN1	76353	25kb
ENSG00000085719	8	87472059	87598726	376	32	120000	-0.026011	0.51038	CPNE3	76353	25kb
ENSG00000170289	8	87541205	87780903	690	61	120000	-0.59929	0.72551	CNGB3	76353	25kb
ENSG00000176571	8	87853670	88652447	2143	75	120000	-1.0708	0.85788	CNBD1	76353	25kb
ENSG00000176566	8	88857973	88911296	85	19	120000	-0.00693	0.50276	DCAF4L2	76353	25kb
ENSG00000156103	8	89019237	89365254	814	62	120000	0.57726	0.28188	MMP16	76353	25kb
ENSG00000104312	8	90744975	90828291	222	17	120000	-0.99303	0.83965	RIPK2	76353	25kb
ENSG00000164823	8	90889087	90965116	177	22	120000	0.11908	0.45261	OSGIN2	76353	25kb
ENSG00000104320	8	90920564	91040456	276	16	120000	0.088791	0.46462	NBN	76353	25kb
ENSG00000104325	8	90988633	91089320	221	17	120000	-0.54601	0.70747	DECR1	76353	25kb
ENSG00000104327	8	91045836	91132703	176	19	120000	-0.72738	0.7665	CALB1	76353	25kb
ENSG00000180694	8	91609223	91828860	457	19	120000	-0.97101	0.83423	TMEM64	76353	25kb
ENSG00000123119	8	91778778	91996636	441	21	120000	-0.94254	0.82704	NECAB1	76353	25kb
ENSG00000253250	8	91945865	92022485	122	14	120000	-0.87093	0.8081	C8orf88	76353	25kb
ENSG00000155099	8	91981024	92078292	191	22	120000	-1.0415	0.85117	TMEM55A	76353	25kb
ENSG00000155100	8	92057424	92124323	111	10	120000	-0.75182	0.77392	OTUD6B	76353	25kb
ENSG00000214954	8	92089060	92256464	348	17	120000	-1.201	0.88512	LRRC69	76353	25kb
ENSG00000147606	8	92196722	92435378	463	40	120000	-0.0029117	0.50116	SLC26A7	76353	25kb
ENSG00000079102	8	92942203	93140514	243	15	120000	-0.73584	0.76909	RUNX1T1	76353	25kb
ENSG00000205133	8	93870758	94054901	376	37	120000	-0.53025	0.70203	TRIQK	76353	25kb
ENSG00000212999	8	93870865	93923013	76	22	120000	0.41247	0.34	AC117834.1	76353	25kb
ENSG00000254318	8	94121324	94204079	208	28	120000	-1.3049	0.90403	C8orf87	76353	25kb
ENSG00000268080	8	94216867	94267388	127	22	120000	0.13842	0.44495	AC016885.1	76353	25kb
ENSG00000188343	8	94685789	94768755	141	18	120000	1.4983	0.067032	FAM92A1	76353	25kb
ENSG00000183808	8	94716584	94778245	87	10	120000	1.416	0.078395	RBM12B	76353	25kb
ENSG00000212998	8	94727349	94778001	71	8	120000	1.3565	0.087473	RBM12B-AS1	76353	25kb
ENSG00000164953	8	94742072	94856462	123	14	120000	0.76486	0.22218	TMEM67	76353	25kb
ENSG00000164951	8	94845035	94963294	301	21	120000	0.17582	0.43022	PDP1	76353	25kb
ENSG00000079112	8	95114399	95254531	444	48	120000	-0.32577	0.6277	CDH17	76353	25kb
ENSG00000164949	8	95236481	95299578	205	27	120000	-0.32781	0.62847	GEM	76353	25kb
ENSG00000197275	8	95359188	95512337	471	23	120000	2.033	0.021026	RAD54B	76353	25kb
ENSG00000265817	8	95365605	95474180	317	17	120000	2.1404	0.016162	FSBP	76353	25kb
ENSG00000164944	8	95474921	95590757	440	25	120000	0.5861	0.2789	KIAA1429	76353	25kb
ENSG00000212997	8	95533771	95585630	200	20	120000	0.030367	0.48789	AC023632.1	76353	25kb
ENSG00000104413	8	95628302	95744694	380	31	120000	-1.0896	0.86205	ESRP1	76353	25kb
ENSG00000156162	8	95706931	95831064	371	26	120000	-0.048578	0.51937	DPY19L4	76353	25kb
ENSG00000164941	8	95800539	95918974	355	24	120000	1.4215	0.077579	INTS8	76353	25kb
ENSG00000175305	8	95866998	95933906	198	24	120000	1.5213	0.064091	CCNE2	76353	25kb
ENSG00000156170	8	95882995	96153683	777	39	120000	0.90972	0.18149	NDUFAF6	76353	25kb
ENSG00000164938	8	95913200	95986639	265	16	120000	1.4582	0.072386	TP53INP1	76353	25kb
ENSG00000175895	8	96121032	96193912	187	27	120000	-0.23449	0.5927	PLEKHF2	76353	25kb
ENSG00000156172	8	96232147	96306429	346	28	120000	0.2059	0.41843	C8orf37	76353	25kb
ENSG00000156466	8	97129562	97198020	119	19	120000	0.76388	0.22247	GDF6	76353	25kb
ENSG00000156467	8	97213148	97272862	130	18	120000	0.7921	0.21415	UQCRB	76353	25kb
ENSG00000156469	8	97226626	97298838	128	15	120000	0.71828	0.23629	MTERFD1	76353	25kb
ENSG00000156471	8	97248943	97374223	309	26	120000	-0.11758	0.5468	PTDSS1	76353	25kb
ENSG00000169439	8	97480579	97649000	467	60	120000	-0.14288	0.55681	SDC2	76353	25kb
ENSG00000104324	8	97632455	98186882	873	66	120000	0.87888	0.18973	CPQ	76353	25kb
ENSG00000180543	8	98260717	98315176	174	20	120000	0.39945	0.34478	TSPYL5	76353	25kb
ENSG00000147649	8	98631407	98765998	289	20	120000	0.40047	0.34441	MTDH	76353	25kb
ENSG00000104341	8	98762285	98890241	528	31	120000	-0.34603	0.63534	LAPTM4B	76353	25kb
ENSG00000132561	8	98856068	99073944	631	54	120000	1.0128	0.15557	MATN2	76353	25kb
ENSG00000156482	8	99012079	99083697	169	24	120000	1.9562	0.025224	RPL30	76353	25kb
ENSG00000177459	8	99051539	99130838	218	46	120000	2.3455	0.0095015	C8orf47	76353	25kb
ENSG00000132541	8	99089572	99154469	230	35	120000	0.098611	0.46072	HRSP12	76353	25kb
ENSG00000104356	8	99104525	99197062	364	36	120000	-0.34521	0.63503	POP1	76353	25kb
ENSG00000104361	8	99177061	99331621	401	53	120000	-0.13999	0.55567	NIPAL2	76353	25kb
ENSG00000104375	8	99388631	99980055	777	45	120000	-0.57962	0.71891	STK3	76353	25kb
ENSG00000156486	8	99414250	99470076	84	19	120000	0.10974	0.45631	KCNS2	76353	25kb
ENSG00000164920	8	99931631	99989332	79	15	120000	1.1116	0.13316	OSR2	76353	25kb
ENSG00000132549	8	100000494	100914808	1587	25	120000	0.89226	0.18613	VPS13B	76353	25kb
ENSG00000164919	8	100860428	100931290	127	15	120000	0.069618	0.47225	COX6C	76353	25kb
ENSG00000132554	8	100948164	101168496	369	31	120000	0.85068	0.19747	RGS22	76353	25kb

ENSG00000156509	8	101120588	101183028	92	8	120000	0.5937	0.27636	FBXO43	76353	25kb
ENSG00000147669	8	101137812	101191230	83	7	120000	0.68184	0.24767	POLR2K	76353	25kb
ENSG00000104450	8	101145134	101296506	250	17	120000	0.70657	0.23992	SPAG1	76353	25kb
ENSG00000034677	8	101244288	101373446	263	18	120000	0.47575	0.31713	RNF19A	76353	25kb
ENSG00000186106	8	101496980	101597012	235	37	120000	-0.2971	0.6168	ANKRD46	76353	25kb
ENSG00000174226	8	101560116	101700643	512	54	120000	1.2842	0.099534	SNX31	76353	25kb
ENSG00000070756	8	101673044	101760037	222	25	120000	0.69834	0.24248	PABPC1	76353	25kb
ENSG00000164924	8	101903753	101990616	202	24	120000	-0.31524	0.62371	YWHAZ	76353	25kb
ENSG00000120963	8	102165106	102243421	145	17	120000	-0.44956	0.67348	ZNF706	76353	25kb
ENSG00000083307	8	102479660	102706954	678	68	120000	-1.0683	0.85732	GRHL2	76353	25kb
ENSG00000104490	8	102673771	103162135	1421	87	120000	0.42617	0.33499	NCALD	76353	25kb
ENSG00000048392	8	103191730	103276346	263	28	120000	-0.33548	0.63137	RRM2B	76353	25kb
ENSG00000104517	8	103240240	103450069	558	33	120000	-0.93069	0.82399	UBR5	76353	25kb
ENSG00000253633	8	103515968	103575896	308	33	120000	-1.903	0.97148	KB-1980E6.3	76353	25kb
ENSG00000155087	8	103538800	103598245	276	39	120000	-2.4891	0.9936	ODF1	76353	25kb
ENSG00000155090	8	103636007	103693130	176	26	120000	-1.3641	0.91374	KLF10	76353	25kb
ENSG00000155096	8	103813585	103931092	309	18	120000	0.52419	0.30007	AZIN1	76353	25kb
ENSG00000253320	8	103851528	104015104	475	44	120000	0.928	0.1767	KB-1507C5.2	76353	25kb
ENSG00000155097	8	104008291	104110279	310	30	120000	0.65163	0.25732	ATP6V1C1	76353	25kb
ENSG00000236939	8	104120191	104178703	259	30	120000	-0.38132	0.64852	C8orf56	76353	25kb
ENSG00000164929	8	104127938	104267533	490	48	120000	0.74962	0.22674	BAALC	76353	25kb
ENSG00000164930	8	104285661	104370094	281	19	120000	0.52464	0.29992	FZD6	76353	25kb
ENSG00000164932	8	104358743	104420225	236	23	120000	-1.2397	0.89246	CTHRC1	76353	25kb
ENSG00000164933	8	104385863	104452417	211	17	120000	-2.065	0.98054	SLC25A32	76353	25kb
ENSG00000164934	8	104401942	104480681	225	22	120000	-1.7687	0.96153	DCAF13	76353	25kb
ENSG00000176406	8	104487976	105293322	2487	34	120000	0.54582	0.29259	RIMS2	76353	25kb
ENSG00000147647	8	105317552	105504281	580	62	120000	0.32776	0.37155	DPYS	76353	25kb
ENSG00000164935	8	105326315	105393917	256	34	120000	0.93432	0.17507	DCSTAMP	76353	25kb
ENSG00000147650	8	105476459	105626252	253	18	120000	2.4577	0.0069913	LRP12	76353	25kb
ENSG00000253350	8	105577961	106456488	2230	121	120000	1.1935	0.11633	RP11-127H5.1	76353	25kb
ENSG00000169946	8	106305920	106841760	1198	95	120000	0.56564	0.28582	ZFPM2	76353	25kb
ENSG00000164830	8	107257473	107789922	1442	72	120000	0.28748	0.38687	OXR1	76353	25kb
ENSG00000174429	8	107746711	107807473	141	24	120000	0.5851	0.27924	ABRA	76353	25kb
ENSG00000154188	8	108236721	108535283	921	64	120000	-1.8309	0.96644	ANGPT1	76353	25kb
ENSG00000147655	8	108886544	109120913	593	39	120000	-0.57995	0.71902	RSPO2	76353	25kb
ENSG00000104408	8	109188445	109472562	889	25	120000	0.77322	0.2197	EIF3E	76353	25kb
ENSG00000104412	8	109430830	109524145	275	14	120000	1.4354	0.075592	EMC2	76353	25kb
ENSG00000164841	8	109594079	109824844	667	46	120000	0.49511	0.31026	TMEM74	76353	25kb
ENSG00000174417	8	110073850	110156813	229	25	120000	-0.84429	0.80075	TRHR	76353	25kb
ENSG00000120526	8	110228148	110371614	341	25	120000	0.17627	0.43004	NUDCD1	76353	25kb
ENSG00000120533	8	110321553	110383182	150	26	120000	0.48895	0.31244	ENY2	76353	25kb
ENSG00000205038	8	110349706	110567559	515	18	120000	-0.17124	0.56798	PKHD1L1	76353	25kb
ENSG00000147654	8	110526940	110603225	154	20	120000	-0.80859	0.79063	EBAG9	76353	25kb
ENSG00000147642	8	110561207	110729020	399	30	120000	-2.304	0.98939	SYBU	76353	25kb
ENSG00000248050	8	110631344	110685313	143	18	120000	-1.9645	0.97527	RP11-422N16.3	76353	25kb
ENSG00000164794	8	110950874	111013076	113	16	120000	-0.1267	0.55041	KCNV1	76353	25kb
ENSG00000164796	8	113210157	114474328	3055	73	120000	-0.66911	0.74829	CSMD3	76353	25kb
ENSG00000253207	8	114587539	114654187	166	21	120000	0.403	0.34347	RP11-67H2.1	76353	25kb
ENSG00000104447	8	116395724	116846899	635	26	120000	1.21	0.11313	TRPS1	76353	25kb
ENSG00000147677	8	117629369	117804164	385	24	120000	-0.24654	0.59737	EIF3H	76353	25kb
ENSG00000147679	8	117753742	117886702	318	36	120000	-0.8518	0.80284	UTP23	76353	25kb
ENSG00000164754	8	117833174	117912105	219	21	120000	-0.59109	0.72277	RAD21	76353	25kb
ENSG00000205002	8	117925438	117981726	159	26	120000	0.85839	0.19534	AARD	76353	25kb
ENSG00000164756	8	117937512	118213953	530	41	120000	-0.52167	0.69905	SLC30A8	76353	25kb
ENSG00000164758	8	118507952	118577501	189	24	120000	0.0045041	0.4982	MED30	76353	25kb
ENSG00000182197	8	118781729	119149092	903	62	120000	-1.3471	0.91103	EXT1	76353	25kb
ENSG00000177570	8	119176698	119659234	1303	100	120000	-0.092866	0.537	SAMD12	76353	25kb
ENSG00000225885	8	119269378	119437844	477	40	120000	-0.2117	0.58383	AC023590.1	76353	25kb
ENSG00000164761	8	119910796	119989439	259	22	120000	1.314	0.09443	TNFRSF11B	76353	25kb
ENSG00000184374	8	119982691	120143821	703	29	120000	0.22186	0.41221	COLEC10	76353	25kb
ENSG00000147676	8	120152273	120282913	341	35	120000	-0.72665	0.76628	MAL2	76353	25kb
ENSG00000136999	8	120403546	120461593	210	11	120000	-1.5589	0.9405	NOV	76353	25kb
ENSG00000136960	8	120544326	120710693	369	36	120000	-0.76489	0.77783	ENPP2	76353	25kb
ENSG00000064313	8	120718015	120870103	412	23	120000	1.6123	0.053449	TAF2	76353	25kb
ENSG00000136982	8	120821216	120893250	293	24	120000	0.72761	0.23343	DSCC1	76353	25kb
ENSG00000155792	8	120860957	121088152	944	38	120000	0.5741	0.28295	DEPTOR	76353	25kb
ENSG00000187955	8	121047019	121409275	888	63	120000	1.0719	0.14188	COL14A1	76353	25kb
ENSG00000172172	8	121368000	121482642	203	19	120000	0.21568	0.41462	MRPL13	76353	25kb
ENSG00000172167	8	121432640	121579373	323	18	120000	0.6373	0.26196	MTBP	76353	25kb
ENSG00000172164	8	121522985	121850513	902	63	120000	0.88609	0.18778	SNTB1	76353	25kb
ENSG00000170961	8	122599356	122678630	244	34	120000	0.0060055	0.4976	HAS2	76353	25kb
ENSG00000178764	8	123768633	124011750	623	104	120000	-0.67338	0.74965	ZHX2	76353	25kb
ENSG00000136986	8	124000404	124079663	213	33	120000	1.0288	0.15179	DERL1	76353	25kb
ENSG00000156787	8	124029208	124189393	426	41	120000	-0.73129	0.7677	TBC1D31	76353	25kb
ENSG00000147689	8	124166200	124247314	195	17	120000	-1.4486	0.92627	FAM83A	76353	25kb
ENSG00000189376	8	124207196	124278638	48	14	120000	-2.5368	0.99441	C8orf76	76353	25kb
ENSG00000259305	8	124213431	124304627	48	20	120000	-2.4521	0.9929	ZHX1-C8ORF76	76353	25kb
ENSG00000165156	8	124235696	124312781	44	17	120000	-2.2281	0.98706	ZHX1	76353	25kb
ENSG00000156802	8	124307090	124453590	285	37	120000	-0.27175	0.60709	ATAD2	76353	25kb
ENSG00000156795	8	124403965	124504470	359	15	120000	0.043107	0.48281	WDYHV1	76353	25kb
ENSG00000156804	8	124485129	124578446	270	46	120000	0.21827	0.41361	FBXO32	76353	25kb
ENSG00000175946	8	124632767	124690190	228	36	120000	1.1671	0.12159	KLHL38	76353	25kb
ENSG00000104537	8	124668034	124774647	317	44	120000	1.0461	0.14776	ANXA13	76353	25kb
ENSG00000176853	8	124755696	124852692	242	30	120000	0.17192	0.43175	FAM91A1	76353	25kb
ENSG00000214814	8	124839227	125157302	739	59	120000	-1.8728	0.96945	FER1L6	76353	25kb
ENSG00000164983	8	125299231	125409933	354	25	120000	-0.49315	0.68905	TMEM65	76353	25kb
ENSG00000183665	8	125438048	125499391	139	25	120000	-0.16413	0.56518	TRMT12	76353	25kb
ENSG00000170881	8	125461979	125525155	127	17	120000	-0.16479	0.56544	RNF139	76353	25kb
ENSG00000147687	8	125475726	125576699	250	32	120000	-0.18042	0.57159	TATDN1	76353	25kb
ENSG00000147684	8	125526344	125605751	276	26	120000	-0.081321	0.53241	NDUFB9	76353	25kb
ENSG00000170873	8	125538031	125765730	847	94	120000	0.042137	0.48319	MTSS1	76353	25kb
ENSG00000180938	8	125960540	126016631	175	15	120000	0.80672	0.20991	ZNF572	76353	25kb
ENSG00000104549	8	125985739	126059525	235	21	120000	0.58513	0.27923	SQLE	76353	25kb
ENSG00000164961	8	126011502	126129082	394	16	120000	-0.09416	0.53751	KIAA0196	76353	25kb
ENSG00000156831	8	126078921	126404362	782	50	120000	2.3761	0.0087473	NSMCE2	76353	25kb
ENSG00000173334	8	126417563	126475647	142	34	120000	1.3361	0.090766	TRIB1	76353	25kb

ENSG00000168672	8	127539687	127595638	144	25	120000	0.24579	0.40292	FAM84B	76353	25kb
ENSG00000212993	8	128401535	128457311	210	25	120000	1.807	0.035384	POU5F1B	76353	25kb
ENSG00000136997	8	128722680	128778674	157	34	120000	0.5788	0.28136	MYC	76353	25kb
ENSG00000256655	8	128934126	128985591	173	27	120000	-1.1461	0.87413	TMEM75	76353	25kb
ENSG00000147697	8	130735442	130824134	334	29	120000	-0.40414	0.65695	GSDMC	76353	25kb
ENSG00000153310	8	130826839	131054375	503	40	120000	-0.42311	0.66389	FAM49B	76353	25kb
ENSG00000153317	8	131039353	131480906	1393	40	120000	-0.45263	0.67459	ASAP1	76353	25kb
ENSG00000155897	8	131767547	132079672	1072	63	120000	0.030061	0.48801	ADCY8	76353	25kb
ENSG00000132294	8	132891335	133050889	462	34	120000	-0.33539	0.63134	EFR3A	76353	25kb
ENSG00000253117	8	133011467	133096627	246	37	120000	-0.18126	0.57192	OC90	76353	25kb
ENSG00000258417	8	133011467	133122902	390	53	120000	-1.1976	0.88447	OC90	76353	25kb
ENSG00000132297	8	133048733	133148406	409	44	120000	-1.4174	0.92182	HHLA1	76353	25kb
ENSG00000184156	8	133108108	133518200	1498	114	120000	-1.8148	0.96522	KCNQ3	76353	25kb
ENSG00000129295	8	133559320	133712838	353	34	120000	-1.5035	0.93365	LRRC6	76353	25kb
ENSG00000165071	8	133672253	133797958	378	25	120000	0.095556	0.46194	TMEM71	76353	25kb
ENSG00000129292	8	133762618	133886052	268	17	120000	-0.3985	0.65487	PHF20L1	76353	25kb
ENSG00000042832	8	133854203	134172147	1046	54	120000	-0.5711	0.71603	TG	76353	25kb
ENSG00000155926	8	134023973	134140298	424	29	120000	-0.54166	0.70597	SLA	76353	25kb
ENSG00000104415	8	134178282	134267587	293	58	120000	-0.53415	0.70338	WISP1	76353	25kb
ENSG00000104419	8	134224414	134339265	434	57	120000	-0.57142	0.71614	NDRG1	76353	25kb
ENSG00000008513	8	134442091	134609183	668	102	120000	0.53602	0.29597	ST3GAL1	76353	25kb
ENSG00000066827	8	135465031	135750292	931	56	120000	-0.65213	0.74284	ZFAT	76353	25kb
ENSG00000131773	8	136444700	136693965	582	34	120000	1.2242	0.11044	KHDRBS3	76353	25kb
ENSG00000147724	8	139117266	139534065	1403	135	120000	2.0304	0.021157	FAM135B	76353	25kb
ENSG00000169436	8	139575478	139951249	1330	134	120000	-0.96263	0.83213	COL22A1	76353	25kb
ENSG00000169427	8	140588081	140740299	461	69	120000	0.45527	0.32446	KCNK9	76353	25kb
ENSG00000167632	8	140717586	141493678	2326	177	120000	-1.2023	0.88538	TRAPPC9	76353	25kb
ENSG00000250733	8	140918416	140971190	175	23	120000	-0.66371	0.74656	C8orf17	76353	25kb
ENSG00000104472	8	141496397	141552236	140	33	120000	1.6788	0.046591	CHRAC1	76353	25kb
ENSG00000123908	8	141516264	141670718	449	53	120000	-0.086426	0.53444	AGO2	76353	25kb
ENSG00000169398	8	141642999	142037315	720	19	120000	1.1089	0.13374	PTK2	76353	25kb
ENSG00000105339	8	142102377	142230907	368	54	120000	-0.29821	0.61723	DENND3	76353	25kb
ENSG00000022567	8	142192265	142343404	532	54	120000	-0.72929	0.76709	SLC45A4	76353	25kb
ENSG00000254019	8	142239664	142291916	166	27	120000	-0.60914	0.72878	RP11-10J21.3	76353	25kb
ENSG00000204882	8	142341600	142402367	177	30	120000	0.16366	0.435	GPR20	76353	25kb
ENSG00000184489	8	142377093	142466620	299	34	120000	0.69213	0.24443	PTP4A3	76353	25kb
ENSG00000226490	8	142499738	142553837	246	36	120000	-0.82239	0.79457	AC138647.1	76353	25kb
ENSG00000171045	8	143268441	143509601	963	43	120000	-1.7251	0.95775	TSNARE1	76353	25kb
ENSG00000181790	8	143505791	143651370	389	56	120000	1.6923	0.045299	BAI1	76353	25kb
ENSG00000198576	8	143667405	143721833	171	20	120000	1.0311	0.15124	ARC	76353	25kb
ENSG00000167653	8	143726726	143789142	259	16	120000	0.19609	0.42227	PSCA	76353	25kb
ENSG00000160886	8	143756529	143811545	168	11	120000	-0.0036878	0.50147	LY6K	76353	25kb
ENSG00000130193	8	143783621	143843345	149	15	120000	0.60988	0.27097	THEM6	76353	25kb
ENSG00000126233	8	143797362	143848829	139	16	120000	0.46461	0.32111	SLURP1	76353	25kb
ENSG00000197353	8	143806568	143858952	145	18	120000	0.66046	0.25448	LYPD2	76353	25kb
ENSG00000180155	8	143820752	143884640	238	25	120000	-0.092718	0.53694	LYNX1	76353	25kb
ENSG00000167656	8	143841296	143893008	228	22	120000	-0.36294	0.64167	LY6D	76353	25kb
ENSG00000104499	8	143890663	144022922	494	27	120000	-0.46628	0.67949	GML	76353	25kb
ENSG00000160882	8	143928772	143986262	188	12	120000	0.010585	0.49578	CYP11B1	76353	25kb
ENSG00000179142	8	143966975	144024259	198	13	120000	-0.26602	0.60489	CYP11B2	76353	25kb
ENSG00000160932	8	144074399	144130249	173	23	120000	-0.17178	0.5682	LY6E	76353	25kb
ENSG00000177335	8	144095626	144166359	204	19	120000	0.074593	0.47027	C8orf31	76353	25kb
ENSG00000261667	8	144136874	144189418	155	11	120000	0.33531	0.3687	RP11-520P18.5	76353	25kb
ENSG00000176956	8	144214331	144267128	175	26	120000	-0.16909	0.56714	LY6H	76353	25kb
ENSG00000182851	8	144270068	144324044	204	25	120000	-1.134	0.8716	GPIHBP1	76353	25kb
ENSG00000181638	8	144303991	144369875	207	15	120000	-1.1873	0.88244	ZFP41	76353	25kb
ENSG00000264668	8	144304280	144383573	256	13	120000	-1.1924	0.88345	ZFP41	76353	25kb
ENSG00000250571	8	144324603	144384101	176	11	120000	-1.1193	0.86849	GLI4	76353	25kb
ENSG00000185730	8	144346846	144405231	221	12	120000	-1.107	0.86585	ZNF696	76353	25kb
ENSG00000184428	8	144361554	144467149	340	21	120000	-1.3243	0.9073	TOP1MT	76353	25kb
ENSG00000158106	8	144426057	144491390	193	29	120000	-1.1211	0.86887	RHPN1	76353	25kb
ENSG00000182759	8	144476352	144537576	259	30	120000	-1.1418	0.87323	MAFA	76353	25kb
ENSG00000014164	8	144494825	144648623	547	41	120000	0.090795	0.46383	ZC3H3	76353	25kb
ENSG00000104518	8	144610377	144670232	249	29	120000	0.39867	0.34507	GSDMD	76353	25kb
ENSG00000204839	8	144623357	144680141	244	30	120000	0.22775	0.40992	MROH6	76353	25kb
ENSG00000147813	8	144631955	144685819	224	28	120000	0.16258	0.43542	NAPRT1	76353	25kb
ENSG00000104529	8	144636867	144706711	275	26	120000	-0.20628	0.58172	EEF1D	76353	25kb
ENSG00000179886	8	144655074	144707485	195	24	120000	-0.43376	0.66777	TIGD5	76353	25kb
ENSG00000104524	8	144661083	144716943	184	24	120000	-0.56136	0.71272	PYCRL	76353	25kb
ENSG00000104522	8	144669788	144725218	171	28	120000	-0.7356	0.76901	TSTA3	76353	25kb
ENSG00000183309	8	144693183	144760900	182	29	120000	-0.76621	0.77822	ZNF623	76353	25kb
ENSG00000181135	8	144741622	144821068	237	39	120000	0.23047	0.40886	ZNF707	76353	25kb
ENSG00000255181	8	144763864	144815279	154	29	120000	0.37948	0.35216	CCDC166	76353	25kb
ENSG00000181085	8	144773429	144829628	162	24	120000	-0.29884	0.61747	MAPK15	76353	25kb
ENSG00000180921	8	144781103	144840971	151	20	120000	-0.77001	0.77935	FAM83H	76353	25kb
ENSG00000180900	8	144848090	144922549	156	27	120000	0.18157	0.42796	SCRIB	76353	25kb
ENSG00000179950	8	144873514	144937029	138	23	120000	0.39235	0.3474	PUF60	76353	25kb
ENSG00000185189	8	144890764	144949200	113	26	120000	0.27031	0.39346	NRBP2	76353	25kb
ENSG00000227184	8	144914497	144977632	149	47	120000	0.97118	0.16573	EPPK1	76353	25kb
ENSG00000178209	8	144964321	145075902	285	16	120000	0.57747	0.28181	PLEC	76353	25kb
ENSG00000178685	8	145026321	145111940	131	13	120000	0.82459	0.2048	PARP10	76353	25kb
ENSG00000178719	8	145039226	145092583	88	8	120000	0.578	0.28163	GRINA	76353	25kb
ENSG00000186583	8	145061582	145126933	101	16	120000	0.48538	0.3137	SPATC1	76353	25kb
ENSG00000178814	8	145081167	145143735	85	14	120000	-0.22181	0.58777	OPLAH	76353	25kb
ENSG00000178896	8	145108529	145160550	90	7	120000	-0.38315	0.6492	EXOSC4	76353	25kb
ENSG00000197858	8	145112493	145166119	97	7	120000	-0.46346	0.67848	GPAA1	76353	25kb
ENSG00000179091	8	145124930	145177428	99	6	120000	-0.24174	0.59551	CYC1	76353	25kb
ENSG00000179526	8	145128536	145188027	117	6	120000	-0.33382	0.63074	SHARPIN	76353	25kb
ENSG00000179632	8	145134402	145187514	112	6	120000	-0.30983	0.62166	MAF1	76353	25kb
ENSG00000179698	8	145137629	145198218	108	6	120000	-0.30251	0.61887	KIAA1875	76353	25kb
ENSG00000235173	8	145167672	145220746	55	4	120000	-0.26743	0.60543	FAM203A	76353	25kb
ENSG00000179832	8	145177919	145341843	122	17	120000	-0.40768	0.65824	MROH1	76353	25kb
ENSG00000170727	8	145461055	145540082	85	7	120000	-0.80644	0.79001	BOP1	76353	25kb
ENSG00000188686	8	145465549	145517470	43	7	120000	-0.86429	0.80629	SCXA	76353	25kb
ENSG00000185122	8	145490280	145563385	116	11	120000	-0.6387	0.73849	HSF1	76353	25kb
ENSG00000185000	8	145514954	145575573	99	14	120000	-0.55661	0.7111	DGAT1	76353	25kb

ENSG00000170616	8	145529228	145584943	85	19	120000	-0.52063	0.69869	SCRT1	76353	25kb
ENSG00000214597	8	145550878	145603574	75	15	120000	-0.12188	0.5485	TMEM249	76353	25kb
ENSG00000271698	8	145551888	145604269	74	14	120000	-0.13257	0.55273	GS1-393G12.13	76353	25kb
ENSG00000185803	8	145552795	145609932	75	14	120000	-0.31472	0.62351	SLC52A2	76353	25kb
ENSG00000182325	8	145554091	145608036	71	13	120000	-0.29279	0.61516	FBXL6	76353	25kb
ENSG00000173137	8	145571790	145643457	111	15	120000	-0.38612	0.65029	ADCK5	76353	25kb
ENSG00000071894	8	145593444	145659753	111	16	120000	0.46855	0.3197	CPSF1	76353	25kb
ENSG00000147804	8	145610126	145667279	121	14	120000	0.83004	0.20326	SLC39A4	76353	25kb
ENSG00000160948	8	145624000	145678931	127	15	120000	1.4164	0.078333	VPS28	76353	25kb
ENSG00000160949	8	145629165	145694827	182	23	120000	1.5803	0.057022	TONSL	76353	25kb
ENSG00000187954	8	145649965	145716060	171	22	120000	1.5375	0.062091	CYHR1	76353	25kb
ENSG00000167702	8	145666426	145724585	138	20	120000	1.2429	0.10695	KIFC2	76353	25kb
ENSG00000160973	8	145673795	145726718	119	15	120000	1.0699	0.14234	FOXH1	76353	25kb
ENSG00000160972	8	145678352	145752504	146	18	120000	0.85716	0.19568	PPP1R16A	76353	25kb
ENSG00000167701	8	145703356	145757557	85	13	120000	0.62924	0.2646	GPT	76353	25kb
ENSG00000167700	8	145709457	145761596	80	14	120000	0.68708	0.24602	MFSD3	76353	25kb
ENSG00000160957	8	145711667	145768229	81	16	120000	0.65142	0.25739	RECQL4	76353	25kb
ENSG00000160959	8	145718376	145775557	75	15	120000	0.57456	0.28279	LRRC14	76353	25kb
ENSG00000254402	8	145722761	145777416	70	14	120000	0.67119	0.25105	LRRC24	76353	25kb
ENSG00000213563	8	145726117	145779516	72	15	120000	0.591	0.27726	C8orf82	76353	25kb
ENSG00000147799	8	145729563	145936194	304	27	120000	0.13403	0.44669	ARHGAP39	76353	25kb
ENSG00000198169	8	145921298	146006802	130	18	120000	-0.33772	0.63221	ZNF251	76353	25kb
ENSG00000196378	8	145973499	146037730	182	17	120000	-1.2082	0.88651	ZNF34	76353	25kb
ENSG00000161016	8	145990150	146042972	158	17	120000	-1.5832	0.94332	RPL8	76353	25kb
ENSG00000197363	8	145999261	146061554	184	16	120000	-1.475	0.92989	ZNF517	76353	25kb
ENSG00000147789	8	146027849	146097894	191	16	120000	-1.298	0.90286	ZNF7	76353	25kb
ENSG00000170619	8	146041427	146104121	155	12	120000	-1.0801	0.85994	COMMD5	76353	25kb
ENSG00000196150	8	146051632	146152553	210	22	120000	-1.1354	0.8719	ZNF250	76353	25kb
ENSG00000170631	8	146130744	146201274	168	22	120000	-0.72093	0.76452	ZNF16	76353	25kb
ENSG00000182307	8	146252764	146306416	124	5	120000	0.90433	0.18291	C8orf33	76353	25kb
ENSG00000170122	9	91237	143417	31	7	120000	0.92287	0.17804	FOXD4	76353	25kb
ENSG00000172785	9	96041	213979	128	18	120000	1.8515	0.03205	CBWD1	76353	25kb
ENSG00000183784	9	188108	240893	176	19	120000	1.5857	0.056404	C9orf66	76353	25kb
ENSG00000107099	9	189854	490259	1439	77	120000	0.21437	0.41513	DOCK8	76353	25kb
ENSG00000107104	9	445291	771105	1609	61	120000	1.0971	0.1363	KANK1	76353	25kb
ENSG00000137090	9	816690	994090	861	75	120000	0.29205	0.38513	DMRT1	76353	25kb
ENSG00000064218	9	951964	1016731	232	37	120000	1.62	0.052612	DMRT3	76353	25kb
ENSG00000173253	9	1025354	1082552	262	42	120000	1.804	0.035618	DMRT2	76353	25kb
ENSG00000080503	9	1990342	2218624	906	125	120000	0.39335	0.34703	SMARCA2	76353	25kb
ENSG00000147852	9	2596834	2685053	351	59	120000	2.2244	0.013061	VLDLR	76353	25kb
ENSG00000168263	9	2692502	2755037	235	41	120000	-0.47647	0.68313	KCNV2	76353	25kb
ENSG00000080608	9	2695469	2869241	521	64	120000	-1.4213	0.92239	KIAA0020	76353	25kb
ENSG00000080298	9	3193297	3551004	859	47	120000	-0.72592	0.76606	RFX3	76353	25kb
ENSG00000203286	9	3427305	3494181	107	17	120000	-0.42015	0.66281	AL365202.1	76353	25kb
ENSG00000107249	9	3799127	4373392	2375	208	120000	-2.4549	0.99295	GLIS3	76353	25kb
ENSG00000106688	9	4465444	4612469	499	76	120000	0.71032	0.23875	SLC1A1	76353	25kb
ENSG00000106686	9	4528386	4691674	664	74	120000	-0.011922	0.50476	SPATA6L	76353	25kb
ENSG00000205808	9	4637298	4690256	282	26	120000	-0.49358	0.6892	PPAPDC2	76353	25kb
ENSG00000106993	9	4654559	4733398	361	33	120000	0.18412	0.42696	CDC37L1	76353	25kb
ENSG00000147853	9	4686155	4767043	386	50	120000	1.134	0.1284	AK3	76353	25kb
ENSG00000120158	9	4767869	4910917	510	44	120000	-1.5098	0.93445	RCL1	76353	25kb
ENSG00000269305	9	4834120	4884260	174	26	120000	-0.72893	0.76698	AL158147.2	76353	25kb
ENSG00000096968	9	4960033	5153183	490	26	120000	-0.4023	0.65627	JAK2	76353	25kb
ENSG00000268155	9	5052163	5109580	158	10	120000	-0.37405	0.64581	AL161450.1	76353	25kb
ENSG00000120210	9	5106979	5210668	246	8	120000	0.10643	0.45762	INSL6	76353	25kb
ENSG00000120211	9	5206419	5260304	104	6	120000	0.71724	0.23661	INSL4	76353	25kb
ENSG00000107014	9	5274868	5329969	136	16	120000	-0.53015	0.70199	RLN2	76353	25kb
ENSG00000107018	9	5309969	5364873	152	20	120000	-0.45576	0.67572	RLN1	76353	25kb
ENSG00000107020	9	5332973	5462878	439	41	120000	1.0341	0.15054	PLGRKT	76353	25kb
ENSG00000120217	9	5425503	5495566	242	40	120000	-0.074969	0.52988	CD274	76353	25kb
ENSG00000197646	9	5485545	5596282	311	43	120000	0.71645	0.23686	PDCD1LG2	76353	25kb
ENSG00000107036	9	5604025	5801557	501	32	120000	1.1451	0.12608	KIAA1432	76353	25kb
ENSG00000099219	9	5740076	5858117	304	23	120000	0.88172	0.18896	ERMP1	76353	25kb
ENSG00000183354	9	5856596	6032901	482	36	120000	0.43181	0.33294	KIAA2026	76353	25kb
ENSG00000120215	9	5865802	5935606	186	28	120000	-0.59558	0.72427	MLANA	76353	25kb
ENSG00000137040	9	5986043	6040618	131	18	120000	0.66886	0.25179	RANBP6	76353	25kb
ENSG00000137033	9	6190805	6282983	252	27	120000	-0.99552	0.84026	IL33	76353	25kb
ENSG00000170777	9	6303349	6356900	126	12	120000	-0.97631	0.83555	TPD52L3	76353	25kb
ENSG00000147854	9	6388151	6532054	359	21	120000	-0.67976	0.75167	UHRF2	76353	25kb
ENSG00000215066	9	6442618	6495375	143	16	120000	-0.44535	0.67197	C9orf38	76353	25kb
ENSG00000178445	9	6507464	6670650	665	65	120000	-1.4255	0.92299	GLDC	76353	25kb
ENSG00000107077	9	6695863	7200648	2305	120	120000	0.76757	0.22137	KDM4C	76353	25kb
ENSG00000137038	9	7771490	7913380	524	64	120000	-0.41586	0.66125	TMEM261	76353	25kb
ENSG00000153707	9	8289246	10637723	10422	490	120000	-1.6453	0.95004	PTPRD	76353	25kb
ENSG00000107165	9	12660439	12735290	341	22	120000	0.42051	0.33706	TYRP1	76353	25kb
ENSG00000153714	9	12750020	12847130	383	59	120000	-0.42015	0.66281	LURAP1L	76353	25kb
ENSG00000107186	9	13080703	13304589	373	41	120000	-0.98717	0.83822	MPDZ	76353	25kb
ENSG00000147862	9	14056842	14423982	912	149	120000	-0.82793	0.79615	NFIB	76353	25kb
ENSG00000175893	9	14586069	14718469	529	46	120000	0.27314	0.39237	ZDHHC21	76353	25kb
ENSG00000147869	9	14694722	14747715	236	37	120000	-0.13183	0.55244	CER1	76353	25kb
ENSG00000164946	9	14709664	14935993	898	102	120000	1.5593	0.059458	FREM1	76353	25kb
ENSG00000155158	9	15138620	15332358	622	70	120000	0.18253	0.42758	TTC39B	76353	25kb
ENSG00000164975	9	15397702	15490951	278	21	120000	0.54461	0.29301	SNAPC3	76353	25kb
ENSG00000164985	9	15439064	15536017	282	21	120000	-0.50756	0.69412	PSIP1	76353	25kb
ENSG00000164989	9	15527895	16086661	1550	21	120000	0.0021529	0.49914	CCDC171	76353	25kb
ENSG00000205549	9	16178933	16301311	529	60	120000	0.55359	0.28993	C9orf92	76353	25kb
ENSG00000173068	9	16384501	16895841	1635	105	120000	-0.031094	0.5124	BNC2	76353	25kb
ENSG00000044459	9	17109980	17528921	1440	37	120000	-0.38735	0.65075	CNTLN	76353	25kb
ENSG00000107295	9	17554080	17822127	1217	69	120000	-0.29056	0.6143	SH3GL2	76353	25kb
ENSG00000178031	9	18448892	18935948	1887	153	120000	-0.0068681	0.50274	ADAMTSL1	76353	25kb
ENSG00000155875	9	18902656	19058251	776	45	120000	-0.67655	0.75065	FAM154A	76353	25kb
ENSG00000155876	9	19024372	19076019	228	21	120000	-0.55784	0.71152	RRAGA	76353	25kb
ENSG00000147874	9	19028141	19128117	404	24	120000	-0.69109	0.75525	HAUS6	76353	25kb
ENSG00000147872	9	19083373	19174288	351	50	120000	-1.5196	0.9357	PLIN2	76353	25kb
ENSG00000137145	9	19205433	19399139	534	24	120000	0.06065	0.47582	DENND4C	76353	25kb
ENSG00000137154	9	19350713	19405252	97	12	120000	0.46263	0.32181	RPS6	76353	25kb

ENSG00000177076	9	19383925	19477018	265	41	120000	-1.2543	0.89513	ACER2	76353	25kb
ENSG00000155886	9	19482450	19811926	951	81	120000	0.035956	0.48566	SLC24A2	76353	25kb
ENSG00000171843	9	20316663	20647542	607	77	120000	-0.42011	0.6628	MLLT3	76353	25kb
ENSG00000188352	9	20633308	21020954	1035	45	120000	-0.1094	0.54356	FOCAD	76353	25kb
ENSG00000188921	9	20970306	21056635	247	31	120000	-0.013097	0.50522	PTPLAD2	76353	25kb
ENSG00000171855	9	21052104	21102943	155	25	120000	1.4804	0.06938	IFNB1	76353	25kb
ENSG00000177047	9	21115631	21167144	186	34	120000	-0.3465	0.63552	IFNW1	76353	25kb
ENSG00000137080	9	21140636	21191659	182	20	120000	0.54074	0.29434	IFNA21	76353	25kb
ENSG00000236637	9	21161617	21212598	223	17	120000	0.44996	0.32637	IFNA4	76353	25kb
ENSG00000214042	9	21176468	21227204	243	15	120000	0.15206	0.43957	IFNA7	76353	25kb
ENSG00000186803	9	21181180	21232142	247	15	120000	0.070418	0.47193	IFNA10	76353	25kb
ENSG00000147885	9	21191372	21242310	253	12	120000	0.027316	0.4891	IFNA16	76353	25kb
ENSG00000234829	9	21202242	21253221	228	13	120000	-0.03671	0.51464	IFNA17	76353	25kb
ENSG00000228083	9	21214201	21264978	198	13	120000	-0.24533	0.5969	IFNA14	76353	25kb
ENSG00000147873	9	21279613	21330312	147	13	120000	-0.17038	0.56765	IFNA5	76353	25kb
ENSG00000198642	9	21304670	21360379	143	13	120000	0.2276	0.40998	KLHL9	76353	25kb
ENSG00000120235	9	21324834	21376377	152	10	120000	0.36529	0.35745	IFNA6	76353	25kb
ENSG00000233816	9	21342371	21393075	134	11	120000	0.33721	0.36798	IFNA13	76353	25kb
ENSG00000188379	9	21359254	21410396	144	15	120000	0.0042867	0.49829	IFNA2	76353	25kb
ENSG00000120242	9	21384146	21435184	143	24	120000	0.18186	0.42785	IFNA8	76353	25kb
ENSG00000197919	9	21415440	21466315	146	24	120000	1.2302	0.10931	IFNA1	76353	25kb
ENSG00000184995	9	21455841	21507312	121	17	120000	0.29705	0.38321	IFNE	76353	25kb
ENSG00000099810	9	21777542	21956646	426	42	120000	0.79457	0.21343	MTAP	76353	25kb
ENSG00000264545	9	21777635	22057985	626	59	120000	0.26417	0.39583	RP11-145E5.5	76353	25kb
ENSG00000224854	9	21942137	21992738	90	18	120000	-1.739	0.95899	C9orf53	76353	25kb
ENSG00000147889	9	21942751	22020300	142	27	120000	-2.0519	0.97991	CDKN2A	76353	25kb
ENSG00000147883	9	21977902	22034362	114	15	120000	-1.5841	0.94341	CDKN2B	76353	25kb
ENSG00000176399	9	22421840	22480739	181	20	120000	-0.38098	0.64839	DMRTA1	76353	25kb
ENSG00000107105	9	23665102	23851335	504	59	120000	1.3426	0.089706	ELAVL2	76353	25kb
ENSG00000205442	9	24517950	24570944	157	8	120000	-0.053988	0.52153	IZUMO3	76353	25kb
ENSG00000198680	9	25651396	25703856	225	39	120000	-1.4272	0.92324	TUSC1	76353	25kb
ENSG00000120159	9	26815683	26917802	293	23	120000	0.089388	0.46439	CAAP1	76353	25kb
ENSG00000137055	9	26879081	26972461	311	21	120000	0.074066	0.47048	PLAA	76353	25kb
ENSG00000096872	9	26922037	27087928	440	30	120000	-0.51048	0.69514	IFT74	76353	25kb
ENSG00000184434	9	26968134	27030691	176	24	120000	-0.68104	0.75208	LRRC19	76353	25kb
ENSG00000120156	9	27084139	27255173	865	80	120000	-1.0922	0.86264	TEK	76353	25kb
ENSG00000120160	9	27259656	27322137	264	25	120000	-0.50859	0.69448	EQTN	76353	25kb
ENSG00000120162	9	27300207	27554779	884	92	120000	1.2379	0.10787	MOB3B	76353	25kb
ENSG00000147896	9	27499312	27551496	218	23	120000	1.3546	0.087768	IFNK	76353	25kb
ENSG00000147894	9	27521544	27598864	315	29	120000	1.4189	0.077957	C9orf72	76353	25kb
ENSG00000174482	9	27923076	28695283	2697	197	120000	0.067679	0.47302	LINGO2	76353	25kb
ENSG00000122729	9	32359618	32479767	312	40	120000	0.53108	0.29768	ACO1	76353	25kb
ENSG00000107201	9	32430300	32551322	272	26	120000	0.61355	0.26975	DDX58	76353	25kb
ENSG00000197579	9	32515542	32577551	118	14	120000	0.40624	0.34228	TOPORS	76353	25kb
ENSG00000165264	9	32527997	32598160	118	20	120000	0.54451	0.29304	NDUFB6	76353	25kb
ENSG00000241043	9	32541787	32593619	90	17	120000	0.5401	0.29457	GVQW1	76353	25kb
ENSG00000122728	9	32604452	32660667	67	11	120000	1.2221	0.11084	TAF1L	76353	25kb
ENSG00000188133	9	32758497	32812397	187	14	120000	1.0599	0.1446	TMEM215	76353	25kb
ENSG00000137074	9	32947604	33050166	369	11	120000	1.1626	0.1225	APTX	76353	25kb
ENSG00000086061	9	33000209	33064905	287	16	120000	1.1087	0.13379	DNAJA1	76353	25kb
ENSG00000122692	9	33016762	33101665	294	20	120000	0.4274	0.33454	SMU1	76353	25kb
ENSG00000086062	9	33079080	33192354	375	36	120000	-1.3957	0.9186	B4GALT1	76353	25kb
ENSG00000122711	9	33193363	33273565	233	23	120000	0.9752	0.16473	SPINK4	76353	25kb
ENSG00000107262	9	33222818	33289761	131	12	120000	0.94254	0.17296	BAG1	76353	25kb
ENSG00000086065	9	33239940	33306977	95	9	120000	0.45535	0.32443	CHMP5	76353	25kb
ENSG00000086102	9	33265509	33396155	176	27	120000	0.19884	0.4212	NFX1	76353	25kb
ENSG00000165269	9	33359765	33427643	184	34	120000	0.96726	0.16671	AQP7	76353	25kb
ENSG00000165272	9	33416152	33472609	242	38	120000	0.75009	0.2266	AQP3	76353	25kb
ENSG00000165271	9	33436439	33498928	171	26	120000	0.72946	0.23286	NOL6	76353	25kb
ENSG00000230453	9	33499392	33598007	295	42	120000	-0.13596	0.55407	ANKRD18B	76353	25kb
ENSG00000010438	9	33725515	33824230	279	28	120000	0.26353	0.39607	PRSS3	76353	25kb
ENSG00000107341	9	33792565	33945402	382	21	120000	-1.5351	0.93763	UBE2R2	76353	25kb
ENSG00000137073	9	33896691	34073947	499	25	120000	-1.7161	0.95693	UBAP2	76353	25kb
ENSG00000198876	9	34061385	34152397	258	19	120000	-1.1697	0.87894	DCAF12	76353	25kb
ENSG00000165006	9	34154003	34277521	231	20	120000	-0.85703	0.80429	UBAP1	76353	25kb
ENSG00000186638	9	34227379	34354198	240	22	120000	-0.80552	0.78974	KIF24	76353	25kb
ENSG00000164978	9	34304504	34368709	128	17	120000	-0.95745	0.83083	NUDT2	76353	25kb
ENSG00000164976	9	34341668	34401851	117	20	120000	-0.92177	0.82168	KIAA1161	76353	25kb
ENSG00000164972	9	34354017	34422830	154	22	120000	-1.2056	0.88602	C9orf24	76353	25kb
ENSG00000164970	9	34373182	34483568	234	31	120000	-1.1158	0.86774	FAM219A	76353	25kb
ENSG00000122735	9	34432412	34545982	197	25	120000	-0.71559	0.76288	DNAI1	76353	25kb
ENSG00000168913	9	34496038	34548039	96	20	120000	-0.96882	0.83368	ENHO	76353	25kb
ENSG00000122756	9	34526430	34615121	218	33	120000	-0.16982	0.56742	CNTFR	76353	25kb
ENSG00000164967	9	34585483	34637101	100	16	120000	0.097987	0.46097	RPP25L	76353	25kb
ENSG00000137100	9	34588548	34645515	110	18	120000	0.084842	0.46619	DCTN3	76353	25kb
ENSG00000205143	9	34596376	34653104	104	17	120000	0.15097	0.44	ARID3C	76353	25kb
ENSG00000147955	9	34609719	34662806	87	14	120000	0.30658	0.37958	SIGMAR1	76353	25kb
ENSG00000213930	9	34613130	34676032	131	17	120000	-0.010426	0.50416	GALT	76353	25kb
ENSG00000258728	9	34621642	34682110	134	18	120000	-0.070084	0.52794	GALT	76353	25kb
ENSG00000137070	9	34625699	34686889	138	18	120000	-0.11164	0.54445	IL11RA	76353	25kb
ENSG00000213927	9	34636877	34689045	120	15	120000	-0.1913	0.57585	CCL27	76353	25kb
ENSG00000187186	9	34639160	34691109	119	15	120000	-0.26513	0.60455	RP11-195F19.5	76353	25kb
ENSG00000172724	9	34664564	34716274	144	24	120000	-0.83192	0.79727	CCL19	76353	25kb
ENSG00000137077	9	34684002	34735121	138	27	120000	-0.85076	0.80255	CCL21	76353	25kb
ENSG00000205108	9	34698052	34754464	158	20	120000	-1.0389	0.85058	FAM205A	76353	25kb
ENSG00000122733	9	34932484	35007541	137	29	120000	-1.1016	0.86467	KIAA1045	76353	25kb
ENSG00000137094	9	34964638	35023897	108	20	120000	-0.6345	0.73712	DNAJB5	76353	25kb
ENSG00000174038	9	35016092	35070988	100	16	120000	-0.65973	0.74529	C9orf131	76353	25kb
ENSG00000269510	9	35017390	35067824	89	15	120000	-0.58648	0.72122	FLJ00273	76353	25kb
ENSG00000165280	9	35031061	35098246	127	18	120000	-0.55626	0.71098	VCP	76353	25kb
ENSG00000221829	9	35048832	35105013	105	15	120000	-0.29539	0.61615	FANCG	76353	25kb
ENSG00000165282	9	35063685	35121591	112	22	120000	-0.15719	0.56245	PIGO	76353	25kb
ENSG00000165283	9	35074888	35128154	111	23	120000	0.2315	0.40846	STOML2	76353	25kb
ENSG00000005238	9	35079109	35141338	151	26	120000	0.14901	0.44077	FAM214B	76353	25kb
ENSG00000198722	9	35136999	35430335	462	29	120000	-0.73691	0.76941	UNC13B	76353	25kb
ENSG00000268482	9	35336056	35386262	61	8	120000	0.11816	0.45297	AL160274.1	76353	25kb

ENSG00000198853	9	35465124	35586895	197	29	120000	0.45599	0.3242	RUSC2	76353	25kb
ENSG00000215187	9	35536828	35588896	88	13	120000	-0.14016	0.55573	FAM166B	76353	25kb
ENSG00000107140	9	35580367	35635038	109	15	120000	-0.58998	0.7224	TESK1	76353	25kb
ENSG00000137101	9	35584530	35671807	239	35	120000	-0.96421	0.83253	CD72	76353	25kb
ENSG00000137078	9	35624292	35675937	165	27	120000	-0.89996	0.81593	SIT1	76353	25kb
ENSG00000159884	9	35633301	35686508	179	30	120000	-1.1799	0.88099	CCDC107	76353	25kb
ENSG00000137135	9	35633872	35700863	209	35	120000	-1.2728	0.89846	ARHGEF39	76353	25kb
ENSG00000107159	9	35648853	35706156	162	25	120000	-1.1451	0.87392	CA9	76353	25kb
ENSG00000198467	9	35656989	35716017	135	24	120000	-1.3355	0.90915	TPM2	76353	25kb
ENSG00000137076	9	35671945	35757392	134	25	120000	-1.7192	0.95721	TLN1	76353	25kb
ENSG00000107175	9	35707332	35762001	62	16	120000	-1.3853	0.91701	CREB3	76353	25kb
ENSG00000070610	9	35711863	35774983	71	18	120000	-1.2176	0.8883	GBA2	76353	25kb
ENSG00000107185	9	35724203	35783572	78	18	120000	-1.0339	0.84942	RGP1	76353	25kb
ENSG00000215183	9	35727987	35781610	69	17	120000	-1.1305	0.87088	MSMP	76353	25kb
ENSG00000159899	9	35767151	35834729	113	14	120000	-0.92477	0.82246	NPR2	76353	25kb
ENSG00000137098	9	35783042	35837269	85	10	120000	-1.0905	0.86225	SPAG8	76353	25kb
ENSG00000137133	9	35787957	35840351	83	10	120000	-1.2183	0.88844	HINT2	76353	25kb
ENSG00000137103	9	35789448	35879844	155	19	120000	-1.2281	0.8903	TMEM8B	76353	25kb
ENSG00000204930	9	35791388	35853744	92	12	120000	-1.2086	0.88659	FAM221B	76353	25kb
ENSG00000168828	9	35844375	35895461	112	20	120000	-0.5826	0.71992	OR13J1	76353	25kb
ENSG00000196196	9	35881189	35932138	145	30	120000	-0.20443	0.58099	HRCT1	76353	25kb
ENSG00000122718	9	35932105	35983151	208	28	120000	1.4774	0.069785	OR2S2	76353	25kb
ENSG00000122707	9	36011430	36149448	375	33	120000	0.77453	0.21931	RECK	76353	25kb
ENSG00000122694	9	36111732	36188910	255	32	120000	0.60332	0.27315	GLIPR2	76353	25kb
ENSG00000185972	9	36144389	36196329	154	21	120000	1.2751	0.10113	CCIN	76353	25kb
ENSG00000122705	9	36165853	36329778	466	39	120000	0.623	0.26664	CLTA	76353	25kb
ENSG00000159921	9	36189438	36302053	284	22	120000	0.60706	0.2719	GNE	76353	25kb
ENSG00000137075	9	36311393	36512545	368	42	120000	0.43616	0.33136	RNF38	76353	25kb
ENSG00000165304	9	36547859	36702678	248	13	120000	1.7533	0.039775	MELK	76353	25kb
ENSG00000196092	9	36808272	37059103	906	85	120000	-1.3692	0.91454	PAX5	76353	25kb
ENSG00000147905	9	37095536	37383146	399	26	120000	-0.33673	0.63184	ZCCHC7	76353	25kb
ENSG00000137106	9	37397663	37461987	163	32	120000	0.20504	0.41877	GRHPR	76353	25kb
ENSG00000168795	9	37413111	37490396	189	34	120000	0.23934	0.40542	ZBTB5	76353	25kb
ENSG00000137054	9	37460932	37528694	247	18	120000	0.38968	0.34839	POLR1E	76353	25kb
ENSG00000147912	9	37485889	37613871	406	25	120000	0.96642	0.16692	FBXO10	76353	25kb
ENSG00000256966	9	37487544	37617466	407	25	120000	0.92732	0.17688	RP11-613M10.8	76353	25kb
ENSG00000175768	9	37557643	37617639	145	18	120000	0.78406	0.2165	TOMM5	76353	25kb
ENSG00000255872	9	37563410	38093684	1494	68	120000	-0.52811	0.70129	RP11-613M10.9	76353	25kb
ENSG00000070601	9	37625997	37771901	490	56	120000	-1.4277	0.92331	FRMPD1	76353	25kb
ENSG00000165275	9	37728804	37803969	206	20	120000	0.3125	0.37733	TRMT10B	76353	25kb
ENSG00000107371	9	37741975	37826434	240	17	120000	0.74485	0.22818	EXOSC3	76353	25kb
ENSG00000122741	9	37775499	37892663	298	11	120000	1.0378	0.14968	DCAF10	76353	25kb
ENSG00000122696	9	37854400	37929350	145	19	120000	0.46721	0.32017	SLC25A51	76353	25kb
ENSG00000107338	9	37894131	38094210	554	29	120000	-1.75	0.95994	SHB	76353	25kb
ENSG00000137124	9	38367661	38423658	269	50	120000	-0.019313	0.5077	ALDH1B1	76353	25kb
ENSG00000137142	9	38383991	38449444	292	40	120000	-0.19569	0.57757	IGFBPL1	76353	25kb
ENSG00000273170	9	38515566	38602204	202	43	120000	0.16532	0.43435	ANKRD18A	76353	25kb
ENSG00000180071	9	38515566	38645657	327	56	120000	0.54991	0.29119	ANKRD18A	76353	25kb
ENSG00000106714	9	39047764	39313312	139	15	120000	-1.2348	0.89155	CNTNAP3	76353	25kb
ENSG00000204814	9	44842571	44895528	44	3	120000	-0.54215	0.70614	RP11-160N1.10	76353	25kb
ENSG00000196873	9	70831397	70939929	1	1	120000	-0.87002	0.80785	CBWD3	76353	25kb
ENSG00000187559	9	70892276	70945000	1	1	120000	-0.87002	0.80785	FOXD4L3	76353	25kb
ENSG00000154330	9	70918224	71170977	417	37	120000	0.38746	0.34921	PGM5	76353	25kb
ENSG00000181778	9	71126496	71180783	118	24	120000	0.97296	0.16529	TMEM252	76353	25kb
ENSG00000107242	9	71295575	71649092	1054	57	120000	-0.69106	0.75524	PIP5K1B	76353	25kb
ENSG00000187866	9	71369964	71423609	144	23	120000	-0.71103	0.76147	FAM122A	76353	25kb
ENSG00000165059	9	71602469	71654039	151	36	120000	0.13566	0.44604	PRKACG	76353	25kb
ENSG00000165060	9	71625175	71740094	396	29	120000	0.86454	0.19365	FXN	76353	25kb
ENSG00000119139	9	71711209	71895124	693	49	120000	-0.39734	0.65444	TJP2	76353	25kb
ENSG00000269295	9	71844331	71894642	245	40	120000	0.31862	0.37501	AL358113.1	76353	25kb
ENSG00000135063	9	71914488	72032371	392	39	120000	0.5737	0.28308	FAM189A2	76353	25kb
ENSG00000107282	9	72017446	72312222	667	44	120000	-0.94476	0.82761	APBA1	76353	25kb
ENSG00000188647	9	72299438	72399875	174	17	120000	1.247	0.1062	PTAR1	76353	25kb
ENSG00000204711	9	72410709	72546148	380	38	120000	-0.72935	0.76711	C9orf135	76353	25kb
ENSG00000165072	9	72633497	72866886	648	21	120000	0.10168	0.45951	MAMDC2	76353	25kb
ENSG00000198887	9	72848937	72994804	373	20	120000	0.10044	0.46	SMC5	76353	25kb
ENSG00000119138	9	72974503	73054540	167	29	120000	-0.6467	0.74109	KLF9	76353	25kb
ENSG00000083067	9	73118979	74086820	2954	136	120000	-0.086056	0.53429	TRPM3	76353	25kb
ENSG00000135048	9	74273282	74456606	526	40	120000	0.26075	0.39714	TMEM2	76353	25kb
ENSG00000107362	9	74452368	74550847	128	16	120000	-0.17617	0.56992	ABHD17B	76353	25kb
ENSG00000155621	9	74501426	74625970	228	40	120000	-0.69689	0.75706	C9orf85	76353	25kb
ENSG00000204669	9	74641292	74712733	190	29	120000	-1.0596	0.85533	C9orf57	76353	25kb
ENSG00000119125	9	74704511	74897109	553	31	120000	-1.5352	0.93763	GDA	76353	25kb
ENSG00000107372	9	74941341	75005163	167	18	120000	-0.94891	0.82867	ZFAND5	76353	25kb
ENSG00000165091	9	75111717	75476267	1049	31	120000	-0.97515	0.83526	TMC1	76353	25kb
ENSG00000165092	9	75490578	75720358	459	37	120000	-0.57537	0.71748	ALDH1A1	76353	25kb
ENSG00000135046	9	75741673	75810309	208	37	120000	-1.2852	0.90064	ANXA1	76353	25kb
ENSG00000198963	9	77087281	77333093	585	54	120000	-0.093544	0.53726	RORB	76353	25kb
ENSG00000119121	9	77312411	77528010	442	62	120000	0.21871	0.41344	TRPM6	76353	25kb
ENSG00000135045	9	77536497	77592802	144	26	120000	-0.94117	0.82669	C9orf40	76353	25kb
ENSG00000156017	9	77570936	77668339	227	26	120000	-1.5533	0.93983	C9orf41	76353	25kb
ENSG00000106733	9	77650489	77728133	295	36	120000	-0.93246	0.82445	NMRK1	76353	25kb
ENSG00000134996	9	77678459	77787181	374	44	120000	-0.086189	0.53434	OSTF1	76353	25kb
ENSG00000099139	9	78480560	79002255	1890	225	120000	-0.15274	0.5607	PCSK5	76353	25kb
ENSG00000135002	9	78975433	79034433	199	21	120000	1.7226	0.042481	RFK	76353	25kb
ENSG00000187210	9	79009752	79147332	500	40	120000	0.54333	0.29345	GCNT1	76353	25kb
ENSG00000106772	9	79201292	79546003	1050	88	120000	-1.1144	0.86745	PRUNE2	76353	25kb
ENSG00000204612	9	79609571	79660869	198	33	120000	-0.46968	0.68071	FOXB2	76353	25kb
ENSG00000197969	9	79767269	80061457	865	35	120000	0.41756	0.33814	VPS13A	76353	25kb
ENSG00000156049	9	80012995	80288223	808	106	120000	3.0296	0.0012244	GNA14	76353	25kb
ENSG00000156052	9	80306003	80671374	609	26	120000	0.94954	0.17117	GNAQ	76353	25kb
ENSG00000148019	9	80825978	80919606	241	31	120000	0.46437	0.32119	CEP78	76353	25kb
ENSG00000135069	9	80887059	80970009	300	33	120000	-0.39237	0.65261	PSAT1	76353	25kb
ENSG00000106829	9	82161688	82366658	413	41	120000	0.0017862	0.49929	TLE4	76353	25kb
ENSG00000196781	9	84173598	84329220	498	91	120000	-1.2318	0.89099	TLE1	76353	25kb
ENSG00000214929	9	84578687	84635171	61	12	120000	-1.0057	0.84272	SPATA31D1	76353	25kb

ENSG00000165105	9	85569500	85703092	197	19	120000	-0.79136	0.78563	RASEF	76353	25kb
ENSG00000172159	9	85832905	86178461	1279	95	120000	0.054332	0.47834	FRMD3	76353	25kb
ENSG00000148057	9	86212964	86284045	188	23	120000	-1.491	0.93202	IDNK	76353	25kb
ENSG00000135018	9	86249878	86348118	260	17	120000	-1.2733	0.89854	UBQLN1	76353	25kb
ENSG00000165113	9	86329336	86469431	269	13	120000	-0.41572	0.66119	GKAP1	76353	25kb
ENSG00000165115	9	86426613	86561342	232	16	120000	0.58694	0.27862	KIF27	76353	25kb
ENSG00000165118	9	86528226	86596901	124	16	120000	0.47827	0.31623	C9orf64	76353	25kb
ENSG00000165119	9	86557998	86620569	92	14	120000	0.34357	0.36559	HNRNPK	76353	25kb
ENSG00000178966	9	86570626	86643985	120	14	120000	0.14888	0.44082	RMI1	76353	25kb
ENSG00000197506	9	86865372	86980672	443	46	120000	1.2523	0.10523	SLC28A3	76353	25kb
ENSG00000148053	9	87258466	87663505	1085	73	120000	-0.90118	0.81625	NTRK2	76353	25kb
ENSG00000135049	9	88136455	88381944	693	20	120000	0.56205	0.28704	AGTPBP1	76353	25kb
ENSG00000135040	9	88531061	88662213	179	31	120000	0.18824	0.42534	NAA35	76353	25kb
ENSG00000135052	9	88616061	88740088	326	25	120000	0.83924	0.20067	GOLM1	76353	25kb
ENSG00000187753	9	88810180	88899572	172	26	120000	1.4966	0.067247	C9orf153	76353	25kb
ENSG00000135070	9	88854461	88922676	117	14	120000	1.1867	0.11767	ISCA1	76353	25kb
ENSG00000083223	9	88877648	88994369	220	18	120000	0.60844	0.27145	ZCCHC6	76353	25kb
ENSG00000180447	9	89534279	89587104	167	22	120000	-0.60835	0.72852	GAS1	76353	25kb
ENSG00000204446	9	89738559	89799471	169	16	120000	-0.80918	0.79079	C9orf170	76353	25kb
ENSG00000196730	9	90087143	90348548	917	88	120000	0.81111	0.20865	DAPK1	76353	25kb
ENSG00000135047	9	90315434	90371308	201	24	120000	0.97141	0.16567	CTSL	76353	25kb
ENSG00000177992	9	90472741	90528814	229	30	120000	-0.047563	0.51897	SPATA31E1	76353	25kb
ENSG00000156345	9	90556356	90614668	119	16	120000	0.68441	0.24686	CDK20	76353	25kb
ENSG00000106723	9	90978334	91118609	249	8	120000	-1.169	0.8788	SPIN1	76353	25kb
ENSG00000130045	9	91125016	91224189	236	31	120000	0.94313	0.17281	NXNL2	76353	25kb
ENSG00000186354	9	91580778	91636055	166	32	120000	1.8508	0.032101	C9orf47	76353	25kb
ENSG00000213694	9	91581362	91644925	202	39	120000	1.6631	0.04815	S1PR3	76353	25kb
ENSG00000148082	9	91603060	91818682	603	47	120000	-0.31464	0.62348	SHC3	76353	25kb
ENSG00000123975	9	91901113	91956618	130	25	120000	-0.4294	0.66618	CKS2	76353	25kb
ENSG00000187742	9	91908421	91999557	224	32	120000	0.012975	0.49482	SECISBP2	76353	25kb
ENSG00000187764	9	91950702	92138045	659	61	120000	1.4746	0.070154	SEMA4D	76353	25kb
ENSG00000130222	9	92194928	92246470	179	24	120000	-0.71274	0.762	GADD45G	76353	25kb
ENSG00000165023	9	93347114	93430386	360	35	120000	0.58053	0.28078	DIRAS2	76353	25kb
ENSG00000165025	9	93539069	93685831	629	73	120000	0.32158	0.37389	SYK	76353	25kb
ENSG00000148090	9	93951097	94149195	491	29	120000	1.0198	0.1539	AUH	76353	25kb
ENSG00000165030	9	94146327	94211144	203	39	120000	0.27621	0.39119	NFIL3	76353	25kb
ENSG00000169071	9	94300373	94737444	1609	90	120000	-0.65906	0.74507	ROR2	76353	25kb
ENSG00000090054	9	94769281	94902666	456	30	120000	-0.18607	0.5738	SPTLC1	76353	25kb
ENSG00000196305	9	94947489	95081038	325	19	120000	0.1753	0.43042	IARS	76353	25kb
ENSG00000198000	9	95034640	95112918	177	16	120000	0.29922	0.38239	NOL8	76353	25kb
ENSG00000188312	9	95062766	95407815	829	23	120000	0.96468	0.16735	CENPP	76353	25kb
ENSG00000106809	9	95121249	95191978	175	18	120000	0.53935	0.29482	OGN	76353	25kb
ENSG00000127083	9	95151527	95211743	165	19	120000	0.68361	0.24711	OMD	76353	25kb
ENSG00000106819	9	95193487	95269788	187	18	120000	0.92858	0.17655	ASPN	76353	25kb
ENSG00000106823	9	95231365	95323937	225	19	120000	1.1453	0.12604	ECM2	76353	25kb
ENSG00000127080	9	95350466	95457547	290	18	120000	1.178	0.1194	IPPK	76353	25kb
ENSG00000185963	9	95448645	95552094	252	15	120000	0.71506	0.23729	BICD2	76353	25kb
ENSG00000127081	9	95582874	95665304	238	29	120000	-1.598	0.94498	ZNF484	76353	25kb
ENSG00000127084	9	95684733	95823518	410	38	120000	0.1995	0.42093	FGD3	76353	25kb
ENSG00000157303	9	95795989	95872420	233	37	120000	1.6991	0.044646	SUSD3	76353	25kb
ENSG00000165233	9	95833500	95900565	228	37	120000	2.1149	0.01722	C9orf89	76353	25kb
ENSG00000131669	9	95858771	95921570	205	34	120000	2.384	0.0085622	NINJ1	76353	25kb
ENSG00000165238	9	95922198	96107854	563	36	120000	-1.4531	0.9269	WNK2	76353	25kb
ENSG00000204352	9	96055481	96133696	249	34	120000	-2.0029	0.9774	C9orf129	76353	25kb
ENSG00000188938	9	96183776	96240874	186	22	120000	-0.83789	0.79895	FAM120AOS	76353	25kb
ENSG00000048828	9	96189004	96353397	479	26	120000	-0.38373	0.64941	FAM120A	76353	25kb
ENSG00000197724	9	96313689	96466869	584	29	120000	-0.33687	0.63189	PHF2	76353	25kb
ENSG00000131668	9	96688905	96742654	159	17	120000	1.6079	0.053924	BARX1	76353	25kb
ENSG00000158079	9	96768076	96897138	210	27	120000	-1.1675	0.8785	PTPDC1	76353	25kb
ENSG00000175787	9	96996593	97088736	279	26	120000	0.051589	0.47943	ZNF169	76353	25kb
ENSG00000130950	9	97052605	97115926	110	20	120000	0.17226	0.43161	NUTM2F	76353	25kb
ENSG00000148110	9	97111833	97248324	312	19	120000	0.56477	0.28611	HIATL1	76353	25kb
ENSG00000130957	9	97296002	97381075	354	21	120000	-0.15004	0.55963	FBP2	76353	25kb
ENSG00000165140	9	97340415	97427531	328	26	120000	-0.073431	0.52927	FBP1	76353	25kb
ENSG00000148120	9	97463983	97874441	884	31	120000	0.88408	0.18833	C9orf3	76353	25kb
ENSG00000158169	9	97836336	98104991	317	25	120000	-1.3942	0.91838	FANCC	76353	25kb
ENSG00000185920	9	98180262	98304339	291	21	120000	2.6656	0.0038421	PTCH1	76353	25kb
ENSG00000268926	9	98509605	98562013	199	32	120000	-1.9143	0.97221	DKFZP434H0512	76353	25kb
ENSG00000182150	9	98612983	98801842	447	26	120000	-1.5012	0.93335	ERCC6L2	76353	25kb
ENSG00000130948	9	98972588	99089434	387	40	120000	-0.44326	0.67121	HSD17B3	76353	25kb
ENSG00000130958	9	99057988	99170992	369	22	120000	0.43623	0.33134	SLC35D2	76353	25kb
ENSG00000165244	9	99123223	99205611	194	11	120000	0.77661	0.21869	ZNF367	76353	25kb
ENSG00000130956	9	99187483	99278618	162	10	120000	-0.47459	0.68246	HABP4	76353	25kb
ENSG00000081377	9	99227523	99407112	193	26	120000	-0.42568	0.66483	CDC14B	76353	25kb
ENSG00000158122	9	99376859	99442585	44	22	120000	-1.2365	0.89186	AAED1	76353	25kb
ENSG00000081386	9	99493147	99565411	120	6	120000	0.25602	0.39897	ZNF510	76353	25kb
ENSG00000196597	9	99553754	99662905	133	15	120000	-0.34258	0.63404	ZNF782	76353	25kb
ENSG00000196312	9	99635348	99800862	118	18	120000	-1.389	0.91758	HIATL2	76353	25kb
ENSG00000188152	9	99665592	99727535	28	8	120000	-0.68466	0.75322	NUTM2G	76353	25kb
ENSG00000136943	9	99769940	99826925	75	7	120000	-1.3948	0.91846	CTSV	76353	25kb
ENSG00000197816	9	99975765	100165806	514	15	120000	1.4365	0.075436	CCDC180	76353	25kb
ENSG00000196116	9	100149232	100283407	295	25	120000	-0.052092	0.52077	TDRD7	76353	25kb
ENSG00000136842	9	100238462	100389030	295	46	120000	-1.08	0.85992	TMOD1	76353	25kb
ENSG00000136925	9	100337362	100420962	102	20	120000	-0.21003	0.58318	TSTD2	76353	25kb
ENSG00000136937	9	100370908	100461030	80	18	120000	-0.22536	0.58915	NCBP1	76353	25kb
ENSG00000136936	9	100412191	100484639	104	14	120000	-0.38221	0.64885	XPA	76353	25kb
ENSG00000178919	9	100590536	100643986	177	12	120000	-0.65142	0.74261	FOXE1	76353	25kb
ENSG00000136932	9	100641771	100709852	163	15	120000	-0.98033	0.83654	C9orf156	76353	25kb
ENSG00000136929	9	100664073	100732138	136	13	120000	-0.73394	0.76851	HEMGN	76353	25kb
ENSG00000136938	9	100720643	100803225	131	22	120000	-0.38515	0.64994	ANP32B	76353	25kb
ENSG00000095380	9	100794021	100870357	158	20	120000	0.42956	0.33376	NANS	76353	25kb
ENSG00000106785	9	100806557	100906494	198	31	120000	0.66143	0.25417	TRIM14	76353	25kb
ENSG00000106789	9	100858257	100979922	442	58	120000	0.99302	0.16035	CORO2A	76353	25kb
ENSG00000095383	9	100936311	101042915	489	38	120000	0.056355	0.47753	TBC1D2	76353	25kb
ENSG00000136928	9	101025391	101496479	1762	147	120000	-0.18765	0.57442	GABBR2	76353	25kb
ENSG00000165138	9	101468611	101584247	227	23	120000	-1.2367	0.89189	ANKS6	76353	25kb

ENSG00000119514	9	101544981	101637363	292	34	120000	-1.3346	0.90899	GALNT12	76353	25kb
ENSG00000204291	9	101680461	101858069	461	48	120000	-0.92852	0.82343	COL15A1	76353	25kb
ENSG00000106799	9	101841320	101941474	193	23	120000	0.11197	0.45542	TGFBR1	76353	25kb
ENSG00000119523	9	101953708	102009238	117	8	120000	-0.79222	0.78588	ALG2	76353	25kb
ENSG00000106803	9	101959346	102017897	133	7	120000	-0.60721	0.72814	SEC61B	76353	25kb
ENSG00000119508	9	102559137	102654173	109	16	120000	1.9745	0.024162	NR4A3	76353	25kb
ENSG00000136874	9	102643915	102757618	209	23	120000	-0.6576	0.7446	STX17	76353	25kb
ENSG00000023318	9	102716461	102886322	282	20	120000	-0.27296	0.60756	ERP44	76353	25kb
ENSG00000119509	9	102836538	103088282	524	17	120000	-0.24883	0.59825	INVS	76353	25kb
ENSG00000136891	9	103039359	103140221	212	18	120000	-0.82813	0.7962	TEX10	76353	25kb
ENSG00000066697	9	103164438	103238511	187	14	120000	-0.041779	0.51666	MSANTD3	76353	25kb
ENSG00000251349	9	103179553	103364918	436	22	120000	1.0969	0.13635	MSANTD3-TMEFF1	76353	25kb
ENSG00000241697	9	103179560	103364918	436	22	120000	1.0969	0.13635	TMEFF1	76353	25kb
ENSG00000170681	9	103315361	103375188	175	24	120000	1.3439	0.089495	MURC	76353	25kb
ENSG00000148123	9	103765991	104112417	1098	66	120000	-0.64301	0.73989	LPPR1	76353	25kb
ENSG00000136881	9	104097699	104170801	243	16	120000	-0.64605	0.74088	BAAT	76353	25kb
ENSG00000136897	9	104124915	104185896	173	21	120000	0.0036954	0.49853	MRPL50	76353	25kb
ENSG00000136870	9	104136155	104197942	169	20	120000	0.35127	0.36269	ZNF189	76353	25kb
ENSG00000136872	9	104157860	104223105	220	26	120000	0.35824	0.36008	ALDOB	76353	25kb
ENSG00000165152	9	104210453	104320819	340	39	120000	-1.1128	0.86711	TMEM246	76353	25kb
ENSG00000155827	9	104271133	104350622	204	31	120000	-0.9968	0.84057	RNF20	76353	25kb
ENSG00000198785	9	104306635	104525862	765	31	120000	-0.91951	0.82109	GRIN3A	76353	25kb
ENSG00000188386	9	104328897	104382283	169	26	120000	-0.53077	0.70221	PPP3R2	76353	25kb
ENSG00000155833	9	105732593	105805770	285	21	120000	-2.0472	0.97968	CYLC2	76353	25kb
ENSG00000136824	9	106831541	106928698	265	15	120000	0.64136	0.26064	SMC2	76353	25kb
ENSG00000186881	9	107241455	107292547	163	22	120000	-0.30149	0.61848	OR13F1	76353	25kb
ENSG00000148136	9	107263534	107314490	155	25	120000	-0.37919	0.64773	OR13C4	76353	25kb
ENSG00000204246	9	107273030	107324137	145	24	120000	-0.32881	0.62885	OR13C3	76353	25kb
ENSG00000186943	9	107306449	107357411	149	14	120000	-0.58018	0.7191	OR13C8	76353	25kb
ENSG00000255800	9	107335650	107386788	233	10	120000	-0.78057	0.78247	OR13C5	76353	25kb
ENSG00000257019	9	107341924	107392951	263	8	120000	-0.95474	0.83014	OR13C2	76353	25kb
ENSG00000136839	9	107354529	107405485	248	12	120000	-1.0812	0.8602	OR13C9	76353	25kb
ENSG00000179055	9	107431660	107482766	186	14	120000	-1.1027	0.86492	OR13D1	76353	25kb
ENSG00000136783	9	107484969	107547403	177	26	120000	0.96994	0.16604	NIPSNAP3A	76353	25kb
ENSG00000165028	9	107501438	107564738	198	34	120000	1.6004	0.054752	NIPSNAP3B	76353	25kb
ENSG00000165029	9	107518283	107715518	767	100	120000	0.47387	0.3178	ABCA1	76353	25kb
ENSG00000070214	9	107981903	108226452	585	35	120000	-1.5814	0.94311	SLC44A1	76353	25kb
ENSG00000106701	9	108185077	108339714	349	32	120000	-0.28981	0.61402	FSD1L	76353	25kb
ENSG00000106692	9	108295411	108428399	283	35	120000	-0.11916	0.54743	FKTN	76353	25kb
ENSG00000186051	9	108399738	108450367	137	30	120000	-0.26552	0.6047	TAL2	76353	25kb
ENSG00000095209	9	108431825	108563893	388	16	120000	-0.93808	0.8259	TMEM38B	76353	25kb
ENSG00000148143	9	109600378	109800915	341	47	120000	0.63107	0.264	ZNF462	76353	25kb
ENSG00000242631	9	109660630	109711455	77	23	120000	0.7618	0.22309	RP11-508N12.4	76353	25kb
ENSG00000119318	9	110020418	110119475	317	24	120000	0.53778	0.29536	RAD23B	76353	25kb
ENSG00000136826	9	110222133	110277763	168	24	120000	0.68837	0.24561	KLF4	76353	25kb
ENSG00000214645	9	110514471	110565419	154	26	120000	-0.96565	0.83289	AL162389.1	76353	25kb
ENSG00000148156	9	111591871	111644239	254	27	120000	-0.047728	0.51903	ACTL7B	76353	25kb
ENSG00000187003	9	111599603	111651035	253	27	120000	-0.30185	0.61862	ACTL7A	76353	25kb
ENSG00000070061	9	111604797	111721396	476	41	120000	-0.82965	0.79663	IKBKAP	76353	25kb
ENSG00000119328	9	111671461	111738024	196	21	120000	-0.80875	0.79067	FAM206A	76353	25kb
ENSG00000119326	9	111679851	111800809	338	23	120000	-0.96109	0.83175	CTNNAL1	76353	25kb
ENSG00000106771	9	111752432	111907225	535	26	120000	-0.7758	0.78107	TMEM245	76353	25kb
ENSG00000260230	9	111867573	111954571	321	29	120000	-0.30823	0.62105	FRRS1L	76353	25kb
ENSG00000095203	9	111909255	112108244	566	65	120000	1.2426	0.10701	EPB41L4B	76353	25kb
ENSG00000070159	9	112112746	112285590	469	41	120000	0.3105	0.37809	PTPN3	76353	25kb
ENSG00000243444	9	112378068	112738755	1349	93	120000	-0.20176	0.57995	PALM2	76353	25kb
ENSG00000157654	9	112517589	112959792	1430	127	120000	-0.51612	0.69711	PALM2-AKAP2	76353	25kb
ENSG00000241978	9	112517769	112959792	1428	128	120000	-0.52587	0.70051	AKAP2	76353	25kb
ENSG00000188959	9	112927328	112995469	241	33	120000	1.607	0.054032	C9orf152	76353	25kb
ENSG00000136810	9	112981091	113043920	210	44	120000	-0.30615	0.62025	TXN	76353	25kb
ENSG00000204193	9	113040801	113125127	311	47	120000	-1.1476	0.87443	TXNDC8	76353	25kb
ENSG00000165124	9	113102531	113367160	1037	71	120000	-2.1517	0.98429	SVEP1	76353	25kb
ENSG00000030304	9	113406051	113588859	556	55	120000	-0.35256	0.63779	MUSK	76353	25kb
ENSG00000198121	9	113610543	113825981	654	53	120000	0.35124	0.3627	LPAR1	76353	25kb
ENSG00000171133	9	114064763	114115800	100	23	120000	1.2878	0.098905	OR2K2	76353	25kb
ENSG00000136813	9	114097972	114272025	419	26	120000	0.79586	0.21306	KIAA0368	76353	25kb
ENSG00000173258	9	114262439	114365124	323	38	120000	0.43978	0.33005	ZNF483	76353	25kb
ENSG00000106853	9	114287002	114387135	349	44	120000	0.4999	0.30857	PTGR1	76353	25kb
ENSG00000059769	9	114368632	114457508	301	31	120000	1.1758	0.11984	DNAJC25	76353	25kb
ENSG00000244115	9	114368666	114457509	300	31	120000	1.1759	0.11981	DNAJC25-GNG10	76353	25kb
ENSG00000242616	9	114398615	114457526	200	25	120000	1.2619	0.10349	GNG10	76353	25kb
ENSG00000165181	9	114423453	114582288	564	23	120000	1.3691	0.085478	C9orf84	76353	25kb
ENSG00000148154	9	114634046	114722649	433	43	120000	1.3682	0.085629	UGCG	76353	25kb
ENSG00000106868	9	114778065	114962688	494	56	120000	-0.096439	0.53841	SUSD1	76353	25kb
ENSG00000119314	9	114955715	115120947	469	14	120000	-0.41752	0.66185	PTBP3	76353	25kb
ENSG00000119471	9	115117217	115259690	433	23	120000	-0.15466	0.56146	HSDL2	76353	25kb
ENSG00000230185	9	115171096	115274484	284	14	120000	0.33209	0.36991	C9orf147	76353	25kb
ENSG00000165185	9	115224127	115456677	544	37	120000	0.44235	0.32912	KIAA1958	76353	25kb
ENSG00000148153	9	115421206	115505516	180	26	120000	0.18144	0.42801	INIP	76353	25kb
ENSG00000148158	9	115488118	115668951	514	20	120000	-0.076268	0.5304	SNX30	76353	25kb
ENSG00000119457	9	115616200	115678193	225	26	120000	0.86189	0.19438	SLC46A2	76353	25kb
ENSG00000136866	9	115775660	115844039	136	31	120000	0.42286	0.3362	ZFP37	76353	25kb
ENSG00000136867	9	115888222	115951417	237	42	120000	0.30564	0.37994	SLC31A2	76353	25kb
ENSG00000119321	9	115898286	116008641	413	35	120000	1.6265	0.051917	FKBP15	76353	25kb
ENSG00000136868	9	115958808	116053674	329	29	120000	1.8503	0.032133	SLC31A1	76353	25kb
ENSG00000176386	9	115993115	116062869	198	24	120000	1.1256	0.13018	CDC26	76353	25kb
ENSG00000136875	9	116012623	116080185	187	25	120000	0.59985	0.2743	PRPF4	76353	25kb
ENSG00000165188	9	116034373	116090656	144	20	120000	0.24295	0.40402	RNF183	76353	25kb
ENSG00000148225	9	116050502	116127578	282	21	120000	0.38332	0.35074	WDR31	76353	25kb
ENSG00000119411	9	116086821	116158513	327	44	120000	0.20322	0.41948	BSPRY	76353	25kb
ENSG00000119431	9	116110699	116164279	239	48	120000	0.2593	0.3977	HDHD3	76353	25kb
ENSG00000148218	9	116123597	116188613	240	51	120000	2.6897	0.0035757	ALAD	76353	25kb
ENSG00000148229	9	116144515	116197952	148	24	120000	3.0127	0.0012945	POLE3	76353	25kb
ENSG00000157653	9	116147334	116216964	166	21	120000	3.1664	0.00077167	C9orf43	76353	25kb
ENSG00000138835	9	116182011	116385018	563	20	120000	1.7156	0.04312	RGS3	76353	25kb
ENSG00000157657	9	116613562	116843871	647	58	120000	-0.031375	0.51251	ZNF618	76353	25kb

ENSG00000106927	9	116797407	116865752	225	35	120000	-0.99724	0.84067	AMBP	76353	25kb
ENSG00000136883	9	116823406	116886507	237	37	120000	-1.0213	0.84644	KIF12	76353	25kb
ENSG00000196739	9	116892840	117099791	725	114	120000	-0.78379	0.78342	COL27A1	76353	25kb
ENSG00000229314	9	117060336	117113755	116	30	120000	-1.43	0.92365	ORM1	76353	25kb
ENSG00000228278	9	117067149	117120532	100	26	120000	-1.0831	0.86062	ORM2	76353	25kb
ENSG00000106948	9	117071436	117181685	239	33	120000	0.67077	0.25118	AKNA	76353	25kb
ENSG00000095397	9	117139360	117292730	479	38	120000	0.81349	0.20797	DFNB31	76353	25kb
ENSG00000136888	9	117325026	117385653	210	29	120000	0.76759	0.22137	ATP6V1G1	76353	25kb
ENSG00000157693	9	117348486	117433702	359	26	120000	0.78025	0.21762	C9orf91	76353	25kb
ENSG00000181634	9	117521915	117593406	141	30	120000	-0.56197	0.71293	TNFSF15	76353	25kb
ENSG00000106952	9	117631003	117717697	214	23	120000	0.7056	0.24022	TNFSF8	76353	25kb
ENSG00000041982	9	117757806	117905536	464	44	120000	2.6956	0.0035131	TNC	76353	25kb
ENSG00000173077	9	117879097	118189923	1066	62	120000	0.65892	0.25497	DEC1	76353	25kb
ENSG00000182752	9	118891083	119189601	510	79	120000	1.8602	0.031429	PAPPA	76353	25kb
ENSG00000256040	9	119135439	119187885	125	20	120000	-1.1854	0.88208	PAPPA-AS1	76353	25kb
ENSG00000148219	9	119162504	120202348	3158	172	120000	-0.24894	0.5983	ASTN2	76353	25kb
ENSG00000119401	9	119424581	119488579	152	21	120000	0.52218	0.30077	TRIM32	76353	25kb
ENSG00000136869	9	120441610	120504149	154	26	120000	-1.157	0.87637	TLR4	76353	25kb
ENSG00000078725	9	121890736	122156745	540	48	120000	0.53377	0.29675	BRINP1	76353	25kb
ENSG00000260970	9	122232653	122311403	280	29	120000	1.1688	0.12123	RP11-295D22.1	76353	25kb
ENSG00000136861	9	123126147	123367448	337	29	120000	-0.24225	0.59571	CDK5RAP2	76353	25kb
ENSG00000106780	9	123338091	123501748	216	15	120000	0.10953	0.45639	MEGF9	76353	25kb
ENSG00000119402	9	123489256	123580690	143	14	120000	-0.0058817	0.50235	FBXW2	76353	25kb
ENSG00000095261	9	123552774	123630262	113	11	120000	-0.060402	0.52408	PSMD5	76353	25kb
ENSG00000119403	9	123592977	123664606	135	16	120000	-0.033919	0.51353	PHF19	76353	25kb
ENSG00000056558	9	123639671	123716451	221	15	120000	0.022596	0.49099	TRAF1	76353	25kb
ENSG00000106804	9	123689616	123837554	345	23	120000	-0.92168	0.82165	C5	76353	25kb
ENSG00000119397	9	123812141	123964888	339	21	120000	-1.0959	0.86345	CNTRL	76353	25kb
ENSG00000119396	9	123915415	124010292	196	26	120000	-1.2648	0.89703	RAB14	76353	25kb
ENSG00000148180	9	123945072	124120121	436	34	120000	-0.40688	0.65795	GSN	76353	25kb
ENSG00000148175	9	124076355	124157531	223	32	120000	0.43159	0.33302	STOM	76353	25kb
ENSG00000267987	9	124078625	124135410	173	25	120000	0.68936	0.2453	AL161784.1	76353	25kb
ENSG00000136848	9	124304336	124572809	631	87	120000	-0.81912	0.79364	DAB2IP	76353	25kb
ENSG00000175764	9	124559207	124880885	1132	70	120000	-0.32626	0.62789	TTLL11	76353	25kb
ENSG00000119421	9	124869745	124947098	279	28	120000	-0.27586	0.60867	NDUFA8	76353	25kb
ENSG00000185681	9	124897190	124987367	246	42	120000	-0.39832	0.6548	MORN5	76353	25kb
ENSG00000106852	9	124939856	125016905	188	44	120000	0.054104	0.47843	LHX6	76353	25kb
ENSG00000119446	9	124974903	125052118	136	26	120000	0.25221	0.40044	RBM18	76353	25kb
ENSG00000148187	9	125001882	125110743	144	27	120000	-0.13959	0.55551	MRRF	76353	25kb
ENSG00000095303	9	125107824	125182982	193	29	120000	0.11204	0.45539	PTGS1	76353	25kb
ENSG00000269738	9	125123344	125173928	142	23	120000	0.49688	0.30964	AL162424.1	76353	25kb
ENSG00000136834	9	125214220	125265235	157	28	120000	0.542	0.29391	OR1J1	76353	25kb
ENSG00000197233	9	125248081	125299022	164	33	120000	0.58946	0.27778	OR1J2	76353	25kb
ENSG00000239590	9	125256420	125307361	174	29	120000	0.89124	0.1864	OR1J4	76353	25kb
ENSG00000171505	9	125263637	125314572	199	27	120000	0.70914	0.23912	OR1N1	76353	25kb
ENSG00000171501	9	125290391	125341493	312	21	120000	0.39135	0.34777	OR1N2	76353	25kb
ENSG00000171496	9	125304778	125355838	320	22	120000	0.39475	0.34651	OR1L8	76353	25kb
ENSG00000165202	9	125351948	125403026	185	21	120000	1.1402	0.1271	OR1Q1	76353	25kb
ENSG00000171484	9	125365858	125416852	164	26	120000	2.0561	0.019885	OR1B1	76353	25kb
ENSG00000173679	9	125398845	125449927	95	16	120000	1.5817	0.056858	OR1L1	76353	25kb
ENSG00000171481	9	125412315	125463432	142	12	120000	0.69936	0.24216	OR1L3	76353	25kb
ENSG00000136939	9	125461269	125512204	152	8	120000	0.49719	0.30953	OR1L4	76353	25kb
ENSG00000171459	9	125487019	125538062	90	8	120000	0.77499	0.21917	OR1L6	76353	25kb
ENSG00000148215	9	125526150	125577237	128	14	120000	0.631	0.26402	OR5C1	76353	25kb
ENSG00000136940	9	125535668	125615910	176	24	120000	0.96835	0.16643	PDCL	76353	25kb
ENSG00000165204	9	125537370	125588395	129	16	120000	0.66576	0.25278	OR1K1	76353	25kb
ENSG00000056586	9	125581835	125692620	199	27	120000	1.5292	0.063109	RC3H2	76353	25kb
ENSG00000186130	9	125645335	125700609	109	17	120000	1.3456	0.089209	ZBTB6	76353	25kb
ENSG00000171448	9	125652845	125718779	122	19	120000	1.6715	0.047309	ZBTB26	76353	25kb
ENSG00000011454	9	125678112	125892145	334	24	120000	1.8571	0.031647	RABGAP1	76353	25kb
ENSG00000188394	9	125771806	125822975	73	13	120000	1.8472	0.032361	GPR21	76353	25kb
ENSG00000165209	9	125846779	126055855	278	28	120000	0.96416	0.16748	STRBP	76353	25kb
ENSG00000148204	9	126093449	126167603	233	40	120000	-0.014205	0.50567	CRB2	76353	25kb
ENSG00000119522	9	126116933	126717431	1478	65	120000	0.61354	0.26976	DENND1A	76353	25kb
ENSG00000106689	9	126738949	126820580	244	37	120000	-0.45464	0.67531	LHX2	76353	25kb
ENSG00000119408	9	126994885	127140586	385	30	120000	-1.5363	0.93776	NEK6	76353	25kb
ENSG00000136930	9	127090745	127202723	226	30	120000	-1.0039	0.84229	PSMB7	76353	25kb
ENSG00000180264	9	127188423	127264379	161	23	120000	0.29976	0.38218	GPR144	76353	25kb
ENSG00000136931	9	127218516	127294709	120	15	120000	0.62492	0.26601	NR5A1	76353	25kb
ENSG00000148200	9	127254554	127558589	237	26	120000	2.1365	0.01632	NR6A1	76353	25kb
ENSG00000185585	9	127514437	127602164	178	27	120000	0.095854	0.46182	OLFML2A	76353	25kb
ENSG00000136918	9	127590755	127645160	156	18	120000	-0.93523	0.82516	WDR38	76353	25kb
ENSG00000136942	9	127595159	127649260	135	17	120000	-1.0603	0.85549	RPL35	76353	25kb
ENSG00000136950	9	127599409	127665003	137	15	120000	-1.0738	0.85854	ARPC5L	76353	25kb
ENSG00000136935	9	127615646	127735771	166	14	120000	-0.98984	0.83887	GOLGA1	76353	25kb
ENSG00000173611	9	127679887	127930785	393	22	120000	0.29275	0.38486	SCAI	76353	25kb
ENSG00000119414	9	127883852	127977218	192	13	120000	1.2822	0.099881	PPP6C	76353	25kb
ENSG00000136933	9	127937821	128021437	160	16	120000	1.6469	0.049788	RABEPK	76353	25kb
ENSG00000044574	9	127972132	128028609	90	15	120000	2.0502	0.020171	HSPA5	76353	25kb
ENSG00000165219	9	127999073	128154486	246	17	120000	2.4789	0.0065888	GAPVD1	76353	25kb
ENSG00000119487	9	128174672	128494513	543	27	120000	-0.45815	0.67658	MAPKAP1	76353	25kb
ENSG00000167081	9	128484624	128754656	362	21	120000	-1.0572	0.85479	PBX3	76353	25kb
ENSG00000196814	9	129064128	129294320	615	41	120000	-0.027478	0.51096	MVB12B	76353	25kb
ENSG00000136944	9	129351722	129488311	424	56	120000	-0.29466	0.61587	LMX1B	76353	25kb
ENSG00000169155	9	129542285	129625489	113	26	120000	0.011053	0.49559	ZBTB43	76353	25kb
ENSG00000177125	9	129597944	129673157	167	27	120000	1.702	0.04438	ZBTB34	76353	25kb
ENSG00000136828	9	129652053	130010445	604	24	120000	2.2076	0.013635	RALGPS1	76353	25kb
ENSG00000136859	9	129824611	129910162	120	15	120000	1.7932	0.03647	ANGPTL2	76353	25kb
ENSG00000136895	9	129961544	130180939	356	29	120000	3.1572	0.00079633	GARNL3	76353	25kb
ENSG00000136856	9	130134421	130195703	115	14	120000	1.2816	0.099984	SLC2A8	76353	25kb
ENSG00000196152	9	130161661	130232651	155	10	120000	0.44651	0.32761	ZNF79	76353	25kb
ENSG00000197958	9	130184953	130238684	129	8	120000	0.10995	0.45622	RPL12	76353	25kb
ENSG00000148356	9	130188765	130290780	231	15	120000	0.19454	0.42287	LRSAM1	76353	25kb
ENSG00000136830	9	130242618	130366268	325	34	120000	1.6398	0.050522	FAM129B	76353	25kb
ENSG00000136854	9	130349544	130482460	150	23	120000	0.6884	0.2456	STXBP1	76353	25kb
ENSG00000187024	9	130430257	130512152	111	19	120000	-0.24487	0.59672	PTRH1	76353	25kb

ENSG00000160401	9	130444268	130503281	78	14	120000	-0.26592	0.60485	C9orf117	76353	25kb
ENSG00000167094	9	130453345	130518879	105	17	120000	-0.38617	0.65032	TTC16	76353	25kb
ENSG00000160404	9	130468803	130522604	102	16	120000	-0.51393	0.69635	TOR2A	76353	25kb
ENSG00000095370	9	130475596	130566020	191	23	120000	1.3837	0.083227	SH2D3C	76353	25kb
ENSG00000136807	9	130522958	130578066	110	13	120000	2.1239	0.01684	CDK9	76353	25kb
ENSG00000136877	9	130531876	130601606	133	17	120000	1.9174	0.027593	FPGS	76353	25kb
ENSG00000106991	9	130552291	130642035	209	40	120000	1.1221	0.13091	ENG	76353	25kb
ENSG00000106992	9	130603759	130665022	150	33	120000	-1.1058	0.86559	AK1	76353	25kb
ENSG00000160408	9	130622600	130692687	152	39	120000	-1.1803	0.88107	ST6GALNAC6	76353	25kb
ENSG00000136840	9	130645165	130704317	125	25	120000	-0.89615	0.81491	ST6GALNAC4	76353	25kb
ENSG00000167103	9	130658158	130718076	131	19	120000	-0.6766	0.75067	PIP5KL1	76353	25kb
ENSG00000136908	9	130672378	130725763	108	11	120000	-0.18216	0.57227	DPM2	76353	25kb
ENSG00000167106	9	130677858	130767792	226	18	120000	-0.24531	0.59689	FAM102A	76353	25kb
ENSG00000171169	9	130798512	130855485	135	8	120000	0.3731	0.35454	NAIF1	76353	25kb
ENSG00000148339	9	130805480	130896524	187	10	120000	0.052193	0.47919	SLC25A25	76353	25kb
ENSG00000148334	9	130857972	130915741	89	18	120000	-0.53081	0.70222	PTGES2	76353	25kb
ENSG00000232850	9	130865612	130916481	73	18	120000	-0.5026	0.69238	AL590708.2	76353	25kb
ENSG00000148346	9	130886350	130940734	93	18	120000	0.28429	0.38809	LCN2	76353	25kb
ENSG00000171159	9	130897539	130951207	103	15	120000	0.49027	0.31197	C9orf16	76353	25kb
ENSG00000148337	9	130903343	130991662	196	24	120000	0.69791	0.24262	CIZ1	76353	25kb
ENSG00000106976	9	130940658	131042527	208	29	120000	0.6924	0.24434	DNM1	76353	25kb
ENSG00000167110	9	130993108	131063274	116	16	120000	0.10149	0.45958	GOLGA2	76353	25kb
ENSG00000175854	9	131012658	131076269	119	16	120000	-0.52496	0.70019	SWI5	76353	25kb
ENSG00000268211	9	131037367	131089212	104	14	120000	-0.82784	0.79612	AL359091.2	76353	25kb
ENSG00000167112	9	131046382	131110021	126	17	120000	-1.0249	0.8473	TRUB2	76353	25kb
ENSG00000167113	9	131059815	131121351	127	18	120000	-1.1467	0.87424	COQ4	76353	25kb
ENSG00000167114	9	131077925	131148749	135	21	120000	-1.3728	0.9151	SLC27A4	76353	25kb
ENSG00000167118	9	131108598	131178015	128	24	120000	-1.2225	0.88923	URM1	76353	25kb
ENSG00000167123	9	131149030	131224626	171	16	120000	-1.2617	0.89648	CERCAM	76353	25kb
ENSG00000136811	9	131192465	131288571	154	9	120000	-1.6818	0.9537	ODF2	76353	25kb
ENSG00000119392	9	131241979	131329567	113	13	120000	-2.0222	0.97842	GLE1	76353	25kb
ENSG00000197694	9	131289866	131420941	195	20	120000	-1.7682	0.96149	SPTAN1	76353	25kb
ENSG00000119333	9	131370940	131444066	118	17	120000	-1.5038	0.93368	WDR34	76353	25kb
ENSG00000119335	9	131420703	131483679	87	22	120000	-0.043325	0.51728	SET	76353	25kb
ENSG00000160447	9	131439802	131508197	83	23	120000	0.28802	0.38667	PKN3	76353	25kb
ENSG00000160446	9	131458148	131511406	60	18	120000	0.44319	0.32881	ZDHHC12	76353	25kb
ENSG00000160445	9	131467065	131559693	113	17	120000	-0.10792	0.54297	ZER1	76353	25kb
ENSG00000107021	9	131524483	131597711	177	16	120000	0.58443	0.27946	TBC1D13	76353	25kb
ENSG00000167136	9	131555753	131609956	161	14	120000	0.71686	0.23673	ENDOG	76353	25kb
ENSG00000198917	9	131556930	131617100	167	14	120000	0.78974	0.21484	C9orf114	76353	25kb
ENSG00000171097	9	131570221	131669773	233	16	120000	0.080521	0.46791	CCBL1	76353	25kb
ENSG00000136802	9	131619391	131705318	180	19	120000	-0.94163	0.82681	LRRC8A	76353	25kb
ENSG00000175287	9	131658174	131729320	119	18	120000	-1.115	0.86757	PHYHD1	76353	25kb
ENSG00000251184	9	131678757	131744300	75	7	120000	-0.81042	0.79115	RP11-101E3.5	76353	25kb
ENSG00000175283	9	131682809	131734898	65	6	120000	-0.89939	0.81578	DOLK	76353	25kb
ENSG00000095319	9	131684978	131794375	124	9	120000	-1.1384	0.87252	NUP188	76353	25kb
ENSG00000148341	9	131744315	131815582	92	14	120000	-0.9335	0.82472	SH3GLB2	76353	25kb
ENSG00000148343	9	131773900	131859361	108	21	120000	-0.80198	0.78872	FAM73B	76353	25kb
ENSG00000167130	9	131818379	131877717	80	9	120000	-0.88879	0.81294	DOLPP1	76353	25kb
ENSG00000095321	9	131832089	131898468	112	6	120000	-0.47712	0.68336	CRAT	76353	25kb
ENSG00000119383	9	131848229	131936225	181	7	120000	-0.37054	0.64451	PPP2R4	76353	25kb
ENSG00000188483	9	131912835	131965540	103	13	120000	-0.47009	0.68085	IER5L	76353	25kb
ENSG00000148335	9	132346163	132423209	250	44	120000	0.90705	0.18219	NTMT1	76353	25kb
ENSG00000179058	9	132349504	132408055	192	30	120000	1.3719	0.085047	C9orf50	76353	25kb
ENSG00000148331	9	132374171	132429444	172	29	120000	1.2097	0.1132	ASB6	76353	25kb
ENSG00000167157	9	132402920	132509875	376	48	120000	0.32082	0.37417	PRRX2	76353	25kb
ENSG00000148344	9	132475610	132540326	252	35	120000	0.19867	0.42126	PTGES	76353	25kb
ENSG00000136816	9	132540432	132598560	257	12	120000	1.6038	0.054384	TOR1B	76353	25kb
ENSG00000136827	9	132550223	132611413	294	13	120000	1.4354	0.075589	TOR1A	76353	25kb
ENSG00000136819	9	132564569	132623142	315	14	120000	1.0814	0.13976	C9orf78	76353	25kb
ENSG00000136878	9	132571977	132669107	545	18	120000	0.66465	0.25314	USP20	76353	25kb
ENSG00000187239	9	132624466	132830473	774	37	120000	0.33208	0.36991	FNBP1	76353	25kb
ENSG00000148358	9	132790705	132927448	516	39	120000	1.4482	0.073782	GPR107	76353	25kb
ENSG00000196979	9	132877889	132931498	277	34	120000	1.2263	0.11005	AL360004.1	76353	25kb
ENSG00000107130	9	132909857	133024583	467	62	120000	0.473	0.31811	NCS1	76353	25kb
ENSG00000148357	9	133021882	133334510	597	93	120000	-0.84453	0.80081	HMCN2	76353	25kb
ENSG00000215428	9	133249029	133307639	242	50	120000	-0.075987	0.53029	AL354898.1	76353	25kb
ENSG00000130707	9	133295316	133401661	374	63	120000	1.0194	0.15401	ASS1	76353	25kb
ENSG00000107164	9	133429352	133538739	283	31	120000	0.45286	0.32532	FUBP3	76353	25kb
ENSG00000130711	9	133514981	133583368	188	33	120000	1.0877	0.13836	PRDM12	76353	25kb
ENSG00000130713	9	133544108	133605248	179	19	120000	0.13658	0.44568	EXOSC2	76353	25kb
ENSG00000097007	9	133564333	133788062	618	52	120000	-0.8478	0.80173	ABL1	76353	25kb
ENSG00000188710	9	133743815	133794225	214	33	120000	-1.3195	0.9065	QRFP	76353	25kb
ENSG00000130720	9	133752825	133839673	342	49	120000	0.48828	0.31267	FIBCD1	76353	25kb
ENSG00000050555	9	133859469	133994860	446	65	120000	1.8536	0.031895	LAMC3	76353	25kb
ENSG00000126878	9	133946863	134023539	240	33	120000	-0.84801	0.80178	AIF1L	76353	25kb
ENSG00000126883	9	133975948	134135057	336	27	120000	-1.4345	0.92429	NUP214	76353	25kb
ENSG00000126882	9	134108463	134176934	162	33	120000	0.7271	0.23358	FAM78A	76353	25kb
ENSG00000160539	9	134140081	134209649	176	24	120000	-0.12734	0.55066	PPAPDC3	76353	25kb
ENSG00000130723	9	134244480	134400584	380	27	120000	-1.2007	0.88507	PRRC2B	76353	25kb
ENSG00000130714	9	134353289	134424193	204	17	120000	-0.62664	0.73455	POMT1	76353	25kb
ENSG00000130717	9	134374188	134431655	205	21	120000	-0.49802	0.69076	UCK1	76353	25kb
ENSG00000107263	9	134427157	134640461	566	29	120000	-0.1674	0.56647	RAPGEF1	76353	25kb
ENSG00000160563	9	134710494	134980295	465	45	120000	-2.2153	0.98663	MED27	76353	25kb
ENSG00000196358	9	135012334	135144921	341	55	120000	-0.24903	0.59833	NTNG2	76353	25kb
ENSG00000107290	9	135111743	135255372	484	29	120000	1.0526	0.14625	SETX	76353	25kb
ENSG00000125482	9	135226008	135307209	209	33	120000	1.889	0.029445	TTF1	76353	25kb
ENSG00000188523	9	135260430	135473704	612	85	120000	1.0467	0.14762	C9orf171	76353	25kb
ENSG00000125492	9	135432572	135490653	137	33	120000	0.066616	0.47344	BARHL1	76353	25kb
ENSG00000125485	9	135443384	135570788	300	28	120000	0.63473	0.2628	DDX31	76353	25kb
ENSG00000125484	9	135520422	135595342	197	20	120000	1.0353	0.15025	GTF3C4	76353	25kb
ENSG00000165695	9	135575965	135779164	520	45	120000	1.6806	0.046424	AK8	76353	25kb
ENSG00000165698	9	135728414	135790588	154	21	120000	1.3208	0.09328	C9orf9	76353	25kb
ENSG00000165699	9	135741735	135845020	221	30	120000	1.1039	0.13482	TSC1	76353	25kb
ENSG00000165702	9	135795932	135892083	354	61	120000	-0.36534	0.64257	GFI1B	76353	25kb
ENSG00000148308	9	135881076	135958890	221	31	120000	-2.3416	0.9904	GTF3C5	76353	25kb

ENSG00000170835	9	135912365	135972248	117	18	120000	-1.2826	0.90019	CEL	76353	25kb
ENSG00000160271	9	135948107	136064301	343	49	120000	-1.2127	0.88738	RALGDS	76353	25kb
ENSG00000148288	9	136003340	136064332	209	41	120000	-0.63938	0.73871	GBGT1	76353	25kb
ENSG00000171102	9	136055664	136109630	204	24	120000	-0.27145	0.60698	OBP2B	76353	25kb
ENSG00000148296	9	136172552	136228235	226	32	120000	0.089054	0.46452	SURF6	76353	25kb
ENSG00000148297	9	136180160	136239986	202	26	120000	-0.28406	0.61182	MED22	76353	25kb
ENSG00000148303	9	136190069	136243281	164	19	120000	-0.29536	0.61614	RPL7A	76353	25kb
ENSG00000148290	9	136193610	136248552	151	18	120000	-0.24746	0.59772	SURF1	76353	25kb
ENSG00000148291	9	136198428	136253045	133	16	120000	-0.27718	0.60918	SURF2	76353	25kb
ENSG00000148248	9	136203325	136267970	178	15	120000	-0.43689	0.6689	SURF4	76353	25kb
ENSG00000198870	9	136218117	136296220	262	10	120000	-0.31605	0.62402	C9orf96	76353	25kb
ENSG00000148300	9	136246186	136308164	242	16	120000	-0.22778	0.59009	REXO4	76353	25kb
ENSG00000160323	9	136254478	136349508	351	30	120000	0.78391	0.21655	ADAMTS13	76353	25kb
ENSG00000160325	9	136300089	136360970	212	36	120000	1.3559	0.087562	CACFD1	76353	25kb
ENSG00000160326	9	136311217	136369259	197	36	120000	1.3562	0.087521	SLC2A6	76353	25kb
ENSG00000187616	9	136354708	136418734	307	46	120000	-0.14599	0.55803	TMEM8C	76353	25kb
ENSG00000197859	9	136372286	136465641	326	51	120000	-0.12784	0.55086	ADAMTSL2	76353	25kb
ENSG00000196990	9	136419079	136476319	149	25	120000	0.73591	0.23089	FAM163B	76353	25kb
ENSG00000123454	9	136476482	136549466	344	60	120000	-0.56388	0.71358	DBH	76353	25kb
ENSG00000123453	9	136503682	136630077	502	65	120000	-0.76881	0.779	SARDH	76353	25kb
ENSG00000160293	9	136602016	136882726	1240	119	120000	-0.69705	0.75711	VAV2	76353	25kb
ENSG00000169925	9	136870427	136958657	330	27	120000	-0.86188	0.80562	BRD3	76353	25kb
ENSG00000196363	9	136975487	137050093	383	22	120000	-1.4936	0.93236	WDR5	76353	25kb
ENSG00000186350	9	137183944	137357431	565	48	120000	-1.0816	0.86029	RXRA	76353	25kb
ENSG00000130635	9	137508620	137761686	1161	115	120000	-0.96546	0.83284	COL5A1	76353	25kb
ENSG00000160339	9	137747654	137804924	255	37	120000	-2.0097	0.97777	FCN2	76353	25kb
ENSG00000085265	9	137776431	137834809	206	35	120000	-2.0778	0.98114	FCN1	76353	25kb
ENSG00000130558	9	137942268	138038025	445	61	120000	-1.5188	0.93559	OLFM1	76353	25kb
ENSG00000269408	9	138125512	138176275	265	65	120000	0.66864	0.25186	AL390778.1	76353	25kb
ENSG00000178243	9	138210095	138263408	156	20	120000	0.33885	0.36736	C9orf62	76353	25kb
ENSG00000196422	9	138345925	138405739	203	45	120000	0.60715	0.27188	PPP1R26	76353	25kb
ENSG00000160345	9	138362027	138418580	203	35	120000	0.57391	0.28302	C9orf116	76353	25kb
ENSG00000122140	9	138366830	138421519	202	34	120000	0.62007	0.26761	MRPS2	76353	25kb
ENSG00000160349	9	138388284	138443378	203	35	120000	1.0465	0.14767	LCN1	76353	25kb
ENSG00000122136	9	138412985	138466815	190	42	120000	0.39857	0.34511	OBP2A	76353	25kb
ENSG00000122133	9	138428602	138483801	247	38	120000	0.073563	0.47068	PAEP	76353	25kb
ENSG00000204007	9	138490502	138556386	312	47	120000	-0.79592	0.78696	GLT6D1	76353	25kb
ENSG00000148386	9	138530168	138583268	212	39	120000	0.67125	0.25103	LCN9	76353	25kb
ENSG00000165643	9	138560253	138616374	211	31	120000	0.69078	0.24485	SOHLH1	76353	25kb
ENSG00000107147	9	138569031	138709992	495	60	120000	-0.02918	0.51164	KCNT1	76353	25kb
ENSG00000130559	9	138675333	138824074	287	23	120000	-1.3639	0.91369	CAMSAP1	76353	25kb
ENSG00000130560	9	138799815	138878226	293	26	120000	-0.94658	0.82807	UBAC1	76353	25kb
ENSG00000148411	9	138873383	139012131	527	73	120000	0.96263	0.16787	NACC2	76353	25kb
ENSG00000238227	9	138981427	139035731	221	40	120000	-0.72108	0.76457	C9orf69	76353	25kb
ENSG00000107187	9	139063096	139121955	210	45	120000	-0.64995	0.74214	LHX3	76353	25kb
ENSG00000165661	9	139073179	139162687	342	44	120000	0.68575	0.24644	QSOX2	76353	25kb
ENSG00000267845	9	139194089	139244640	107	23	120000	-0.28356	0.61163	DKFZP434A062	76353	25kb
ENSG00000160360	9	139196932	139279057	240	39	120000	-0.29543	0.61617	GPSM1	76353	25kb
ENSG00000213221	9	139228932	139283241	200	28	120000	-0.45195	0.67435	DNLZ	76353	25kb
ENSG00000187796	9	139231355	139293133	224	29	120000	-0.34215	0.63388	CARD9	76353	25kb
ENSG00000165684	9	139245029	139318249	294	22	120000	-0.081407	0.53244	SNAPC4	76353	25kb
ENSG00000165689	9	139271377	139330061	250	27	120000	-0.095807	0.53816	SDCCAG3	76353	25kb
ENSG00000165688	9	139280110	139343213	243	28	120000	-0.059157	0.52359	PMPCA	76353	25kb
ENSG00000148384	9	139298071	139359274	223	28	120000	-0.19941	0.57903	INPP5E	76353	25kb
ENSG00000148396	9	139309549	139397141	266	21	120000	-0.12693	0.5505	SEC16A	76353	25kb
ENSG00000196366	9	139352947	139405518	163	26	120000	0.079863	0.46817	C9orf163	76353	25kb
ENSG00000148400	9	139363896	139465314	337	49	120000	0.62418	0.26625	NOTCH1	76353	25kb
ENSG00000172889	9	139528308	139592130	190	33	120000	0.34499	0.36505	EGFL7	76353	25kb
ENSG00000169692	9	139542595	139606875	172	29	120000	0.15994	0.43646	AGPAT2	76353	25kb
ENSG00000165716	9	139582022	139643502	195	30	120000	-1.05	0.85313	FAM69B	76353	25kb
ENSG00000187922	9	139607619	139662808	209	25	120000	-1.3968	0.91876	LCN10	76353	25kb
ENSG00000204003	9	139607619	139667905	218	25	120000	-1.44	0.92506	LCN6	76353	25kb
ENSG00000267206	9	139613463	139667980	201	22	120000	-1.3729	0.9151	LCN6	76353	25kb
ENSG00000204001	9	139623838	139677678	204	20	120000	-1.3411	0.91005	LCN8	76353	25kb
ENSG00000177984	9	139629086	139685707	221	18	120000	-1.309	0.90474	LCN15	76353	25kb
ENSG00000244187	9	139660807	139712709	168	13	120000	-1.0561	0.85454	TMEM141	76353	25kb
ENSG00000272896	9	139660831	139720047	175	14	120000	-1.0435	0.85165	RP11-216L13.17	76353	25kb
ENSG00000213213	9	139665802	139727193	172	14	120000	-0.92761	0.8232	CCDC183	76353	25kb
ENSG00000196642	9	139677374	139760639	154	18	120000	-1.1371	0.87226	RABL6	76353	25kb
ENSG00000232434	9	139713867	139766797	60	12	120000	-0.72102	0.76455	C9orf172	76353	25kb
ENSG00000054148	9	139718176	139770488	61	12	120000	-0.85219	0.80294	PHPT1	76353	25kb
ENSG00000177943	9	139720395	139780249	81	11	120000	-0.65412	0.74348	MAMDC4	76353	25kb
ENSG00000107223	9	139731571	139785738	78	9	120000	-0.72856	0.76687	EDF1	76353	25kb
ENSG00000127191	9	139751364	139846059	231	17	120000	-0.79668	0.78718	TRAF2	76353	25kb
ENSG00000159069	9	139809887	139864148	195	24	120000	-0.68009	0.75178	FBXW5	76353	25kb
ENSG00000176919	9	139814698	139866426	193	23	120000	-0.57607	0.71772	C8G	76353	25kb
ENSG00000184925	9	139819003	139874949	207	24	120000	-0.38892	0.65133	LCN12	76353	25kb
ENSG00000198454	9	139838695	139891442	185	20	120000	0.032835	0.4869	C9orf141	76353	25kb
ENSG00000107317	9	139846956	139904887	188	20	120000	0.22163	0.4123	PTGDS	76353	25kb
ENSG00000214402	9	139851356	139905862	172	19	120000	0.29809	0.38282	LCNL1	76353	25kb
ENSG00000148362	9	139861870	139913436	148	18	120000	0.38233	0.35111	C9orf142	76353	25kb
ENSG00000169583	9	139864087	139916255	139	18	120000	0.39508	0.34639	CLIC3	76353	25kb
ENSG00000107331	9	139876686	139948367	168	25	120000	0.41422	0.33936	ABCA2	76353	25kb
ENSG00000180539	9	139896916	139956234	133	23	120000	0.14143	0.44376	C9orf139	76353	25kb
ENSG00000180549	9	139899626	139952462	123	23	120000	0.14977	0.44047	FUT7	76353	25kb
ENSG00000107281	9	139908922	139965655	132	20	120000	0.35555	0.36109	NPDC1	76353	25kb
ENSG00000054179	9	139917550	139973497	131	21	120000	0.40194	0.34386	ENTPD2	76353	25kb
ENSG00000186193	9	139931581	139990040	130	24	120000	0.71739	0.23657	SAPCD2	76353	25kb
ENSG00000197355	9	139946953	140003991	131	22	120000	0.53133	0.29759	UAP1L1	76353	25kb
ENSG00000268996	9	139955807	140006121	119	19	120000	0.60755	0.27174	AL807752.1	76353	25kb
ENSG00000177239	9	139956379	140028635	181	29	120000	0.87048	0.19202	MAN1B1	76353	25kb
ENSG00000176978	9	139979994	140034629	147	27	120000	0.99272	0.16042	DPP7	76353	25kb
ENSG00000176884	9	140007842	140088207	173	28	120000	0.48588	0.31353	GRIN1	76353	25kb
ENSG00000184709	9	140038210	140089503	87	10	120000	-0.31332	0.62298	LRRC26	76353	25kb
ENSG00000185863	9	140040310	140091861	84	9	120000	-0.34213	0.63387	TMEM210	76353	25kb
ENSG00000176248	9	140044236	140107989	111	13	120000	-0.61389	0.73036	ANAPC2	76353	25kb

ENSG00000176101	9	140058099	140109822	94	11	120000	-0.86388	0.80617	SSNA1	76353	25kb
ENSG00000176058	9	140061069	140123645	121	17	120000	-0.91544	0.82002	TPRN	76353	25kb
ENSG00000187713	9	140073534	140125090	102	15	120000	-0.89092	0.81352	TMEM203	76353	25kb
ENSG00000188566	9	140075147	140136461	133	21	120000	-0.96693	0.83321	NDOR1	76353	25kb
ENSG00000212864	9	140089707	140141033	105	21	120000	-1.0097	0.84369	RNF208	76353	25kb
ENSG00000197191	9	140094087	140145763	119	24	120000	-1.0312	0.84877	C9orf169	76353	25kb
ENSG00000233198	9	140097018	140149090	124	25	120000	-0.89012	0.8133	RNF224	76353	25kb
ENSG00000198569	9	140100209	140156006	136	25	120000	-0.77082	0.77959	SLC34A3	76353	25kb
ENSG00000188229	9	140110665	140163159	136	19	120000	-0.73979	0.77029	TUBB4B	76353	25kb
ENSG00000188163	9	140113036	140167222	134	18	120000	-0.71003	0.76116	FAM166A	76353	25kb
ENSG00000197768	9	140120713	140172934	137	16	120000	-0.68664	0.75385	C9orf173	76353	25kb
ENSG00000188986	9	140124625	140192998	166	22	120000	-0.26969	0.6063	NELFB	76353	25kb
ENSG00000198113	9	140147201	140202093	117	16	120000	0.16779	0.43337	TOR4A	76353	25kb
ENSG00000198435	9	140169083	140221703	91	28	120000	1.7945	0.036365	NRARP	76353	25kb
ENSG00000187609	9	140176348	140342714	387	75	120000	0.18622	0.42614	EXD3	76353	25kb
ENSG00000188747	9	140292802	140353858	112	23	120000	-0.040619	0.5162	NOXA1	76353	25kb
ENSG00000188833	9	140303816	140361268	92	18	120000	0.33599	0.36844	ENTPD8	76353	25kb
ENSG00000165802	9	140317022	140378786	96	24	120000	0.23702	0.40632	NSMF	76353	25kb
ENSG00000130653	9	140329404	140469986	217	61	120000	0.52793	0.29877	PNPLA7	76353	25kb
ENSG00000182154	9	140420651	140472007	84	24	120000	0.89013	0.1867	MRPL41	76353	25kb
ENSG00000148399	9	140424356	140498387	125	22	120000	1.3264	0.092354	DPH7	76353	25kb
ENSG00000165724	9	140451531	140509942	99	13	120000	1.4059	0.079874	ZMYND19	76353	25kb
ENSG00000197070	9	140475106	140534812	115	11	120000	1.0435	0.14835	ARRDC1	76353	25kb
ENSG00000203993	9	140484784	140538358	109	11	120000	0.87996	0.18944	C9orf37	76353	25kb
ENSG00000181090	9	140488444	140789468	731	58	120000	0.53402	0.29667	EHMT1	76353	25kb
ENSG00000148408	9	140747241	141044076	592	48	120000	0.50505	0.30676	CACNA1B	76353	25kb
ENSG00000173876	10	67828	145103	131	19	120000	0.12766	0.44921	TUBB8	76353	25kb
ENSG00000015171	10	155405	325577	300	25	120000	0.22667	0.41034	ZMYND11	76353	25kb
ENSG00000151240	10	295130	760683	1103	60	120000	-0.92971	0.82374	DIP2C	76353	25kb
ENSG00000180525	10	670888	736109	103	12	120000	-0.45365	0.67496	PRR26	76353	25kb
ENSG00000107929	10	830484	1002564	444	25	120000	1.3356	0.090832	LARP4B	76353	25kb
ENSG00000205740	10	992097	1059281	194	22	120000	-1.528	0.93674	AL359878.1	76353	25kb
ENSG00000107937	10	1009338	1090876	261	20	120000	-1.1831	0.88161	GTPBP4	76353	25kb
ENSG00000148377	10	1039847	1096799	180	22	120000	-1.0207	0.84631	IDI2	76353	25kb
ENSG00000067064	10	1060848	1120110	168	25	120000	0.22222	0.41207	IDI1	76353	25kb
ENSG00000047056	10	1070478	1203237	413	37	120000	1.5597	0.059416	WDR37	76353	25kb
ENSG00000185736	10	1203073	1804670	2458	212	120000	0.19102	0.42425	ADARB2	76353	25kb
ENSG00000067057	10	3083525	3204904	762	72	120000	-0.23297	0.59211	PFKP	76353	25kb
ENSG00000107959	10	3154920	3240003	527	34	120000	-0.43465	0.66809	PITRM1	76353	25kb
ENSG00000067082	10	3793188	3852473	256	26	120000	-0.21606	0.58553	KLF6	76353	25kb
ENSG00000165568	10	4803821	4915254	664	44	120000	-0.49869	0.691	AKR1E2	76353	25kb
ENSG00000187134	10	4909796	5050475	600	51	120000	-0.81122	0.79138	AKR1C1	76353	25kb
ENSG00000151632	10	5004967	5085223	351	41	120000	0.30368	0.38069	AKR1C2	76353	25kb
ENSG00000196139	10	5052546	5174878	637	44	120000	0.88101	0.18916	AKR1C3	76353	25kb
ENSG00000196326	10	5171335	5269449	482	24	120000	0.050258	0.47996	AKR1CL1	76353	25kb
ENSG00000198610	10	5212425	5285912	418	26	120000	0.75467	0.22522	AKR1C4	76353	25kb
ENSG00000178473	10	5381972	5441169	264	30	120000	-0.96891	0.83371	UCN3	76353	25kb
ENSG00000178462	10	5410061	5471793	203	21	120000	-0.5762	0.71776	TUBAL3	76353	25kb
ENSG00000173848	10	5429514	5525426	266	16	120000	-0.46665	0.67963	NET1	76353	25kb
ENSG00000178372	10	5515658	5566533	190	37	120000	-0.60859	0.7286	CALML5	76353	25kb
ENSG00000178363	10	5541924	5593225	156	41	120000	0.13802	0.44511	CALML3	76353	25kb
ENSG00000196372	10	5655830	5733558	399	51	120000	1.311	0.09493	ASB13	76353	25kb
ENSG00000108021	10	5701801	5830703	400	33	120000	1.2274	0.10984	FAM208B	76353	25kb
ENSG00000057608	10	5782186	5909095	382	36	120000	1.582	0.056825	GDI2	76353	25kb
ENSG00000134461	10	5878580	5956869	329	19	120000	1.4555	0.072767	ANKRD16	76353	25kb
ENSG00000134452	10	5906535	6004556	426	43	120000	0.096299	0.46164	FBXO18	76353	25kb
ENSG00000134470	10	5965855	6045150	348	61	120000	-1.4258	0.92303	IL15RA	76353	25kb
ENSG00000134460	10	6027652	6129288	434	56	120000	1.4107	0.07917	IL2RA	76353	25kb
ENSG00000134453	10	6105950	6184420	343	43	120000	-0.37475	0.64608	RBM17	76353	25kb
ENSG00000170525	10	6161881	6302495	651	133	120000	-0.06482	0.52584	PFKFB3	76353	25kb
ENSG00000212743	10	6367278	6419723	116	27	120000	-1.3901	0.91776	DKFZP667F0711	76353	25kb
ENSG00000065675	10	6444105	6647263	608	76	120000	-0.50053	0.69165	PRKCQ	76353	25kb
ENSG00000198879	10	7175586	7478450	1050	83	120000	-0.30029	0.61802	SFMBT2	76353	25kb
ENSG00000123243	10	7576232	7733961	680	85	120000	-0.20572	0.5815	ITIH5	76353	25kb
ENSG00000151655	10	7720232	7816483	390	50	120000	-0.2805	0.61045	ITIH2	76353	25kb
ENSG00000151657	10	7767925	7854990	291	31	120000	-0.3346	0.63104	KIN	76353	25kb
ENSG00000165629	10	7805092	7874778	238	27	120000	0.13122	0.4478	ATP5C1	76353	25kb
ENSG00000165632	10	7835467	8083590	797	48	120000	-1.2502	0.89439	TAF3	76353	25kb
ENSG00000107485	10	8070567	8142161	308	45	120000	-0.8756	0.80938	GATA3	76353	25kb
ENSG00000048740	10	11022259	11403674	1301	117	120000	-0.14724	0.55853	CELF2	76353	25kb
ENSG00000148429	10	11470945	11678753	556	35	120000	0.39731	0.34557	USP6NL	76353	25kb
ENSG00000134463	10	11759365	11831069	355	25	120000	-0.66405	0.74667	ECHDC3	76353	25kb
ENSG00000148426	10	11840338	11939276	372	47	120000	-0.78897	0.78494	PROSER2	76353	25kb
ENSG00000151461	10	11937021	12110169	602	31	120000	-0.75042	0.7735	UPF2	76353	25kb
ENSG00000181192	10	12085971	12190224	361	35	120000	-1.3405	0.90996	DHTKD1	76353	25kb
ENSG00000065665	10	12146636	12236960	274	22	120000	-0.93728	0.82569	SEC61A2	76353	25kb
ENSG00000165609	10	12182324	12263143	218	28	120000	-0.83298	0.79757	NUDT5	76353	25kb
ENSG00000151465	10	12212964	12317588	281	30	120000	-0.85603	0.80401	CDC123	76353	25kb
ENSG00000183049	10	12366481	12902545	2008	199	120000	-0.82828	0.79624	CAMK1D	76353	25kb
ENSG00000151468	10	12913627	13166652	1065	97	120000	-0.26785	0.60559	CCDC3	76353	25kb
ENSG00000123240	10	13116449	13205291	257	36	120000	0.19441	0.42293	OPTN	76353	25kb
ENSG00000065328	10	13178554	13278104	369	64	120000	-2.0517	0.9799	MCM10	76353	25kb
ENSG00000165623	10	13238767	13301334	321	56	120000	-0.11833	0.5471	UCMA	76353	25kb
ENSG00000107537	10	13294796	13369412	312	43	120000	0.27155	0.39298	PHYH	76353	25kb
ENSG00000226209	10	13325145	13375447	200	27	120000	-0.71138	0.76157	AL138764.1	76353	25kb
ENSG00000086475	10	13334424	13415297	297	26	120000	0.23897	0.40556	SEPHS1	76353	25kb
ENSG00000165626	10	13455484	13595974	410	32	120000	-0.33413	0.63086	BEND7	76353	25kb
ENSG00000165630	10	13603927	13697868	271	19	120000	-0.20424	0.58092	PRPF18	76353	25kb
ENSG00000239665	10	13627047	13735299	374	33	120000	-0.045781	0.51826	RP11-295P9.3	76353	25kb
ENSG00000151474	10	13660706	14529141	3656	271	120000	-0.55838	0.71171	FRMD4A	76353	25kb
ENSG00000065809	10	14535556	14841896	1205	109	120000	-0.45109	0.67404	FAM107B	76353	25kb
ENSG00000185267	10	14836249	14905574	171	26	120000	0.31782	0.37531	CDNF	76353	25kb
ENSG00000187522	10	14855163	14938740	136	22	120000	0.68781	0.24579	HSPA14	76353	25kb
ENSG00000152455	10	14895819	14971314	129	24	120000	0.64957	0.25798	SUV39H2	76353	25kb
ENSG00000152457	10	14914358	15021431	325	37	120000	0.85281	0.19688	DCLRE1C	76353	25kb
ENSG00000197889	10	14976438	15055049	370	31	120000	1.0934	0.13711	MEIG1	76353	25kb

ENSG00000152463	10	15049226	15140851	424	45	120000	1.3866	0.082787	OLAH	76353	25kb
ENSG00000176244	10	15094522	15155775	236	36	120000	2.6611	0.0038948	ACBD7	76353	25kb
ENSG00000176236	10	15112384	15164318	222	31	120000	2.5928	0.00476	C10orf111	76353	25kb
ENSG00000152464	10	15114179	15206817	362	36	120000	2.6553	0.0039616	RPP38	76353	25kb
ENSG00000152465	10	15119583	15235692	415	43	120000	2.3905	0.0084126	NMT2	76353	25kb
ENSG00000148468	10	15228642	15438061	736	66	120000	0.17937	0.42882	FAM171A1	76353	25kb
ENSG00000077943	10	15530948	15787124	845	50	120000	-0.20469	0.58109	ITGA8	76353	25kb
ENSG00000148481	10	15795169	15927519	203	24	120000	1.2869	0.099063	FAM188A	76353	25kb
ENSG00000165983	10	16453942	16580736	490	35	120000	0.1013	0.45966	PTER	76353	25kb
ENSG00000165985	10	16530742	16589004	206	30	120000	-1.0976	0.86382	C1QL3	76353	25kb
ENSG00000148484	10	16607610	16884527	876	86	120000	-0.42846	0.66584	RSU1	76353	25kb
ENSG00000107611	10	16840963	17196830	1423	104	120000	-0.70091	0.75832	CUBN	76353	25kb
ENSG00000107614	10	17159253	17269053	395	22	120000	0.19935	0.42099	TRDMT1	76353	25kb
ENSG00000026025	10	17245258	17304592	176	25	120000	-0.039874	0.5159	VIM	76353	25kb
ENSG00000148488	10	17335382	17521329	956	49	120000	1.3585	0.087146	ST8SIA6	76353	25kb
ENSG00000165996	10	17606958	17684376	278	25	120000	-0.47423	0.68233	PTPLA	76353	25kb
ENSG00000136738	10	17661124	17782913	496	29	120000	-0.75327	0.77436	STAM	76353	25kb
ENSG00000148483	10	17769251	17867867	56	10	120000	-0.018321	0.50731	TMEM236	76353	25kb
ENSG00000183748	10	17826362	17978178	34	16	120000	0.9465	0.17195	MRC1L1	76353	25kb
ENSG00000184040	10	18016218	18114855	12	6	120000	-1.2846	0.90053	TMEM236	76353	25kb
ENSG00000120586	10	18073352	18225091	44	9	120000	1.065	0.14345	MRC1	76353	25kb
ENSG00000148482	10	18215768	18357221	610	53	120000	-0.014124	0.50563	SLC39A12	76353	25kb
ENSG00000165995	10	18404606	18855798	1727	124	120000	-0.47673	0.68322	CACNB2	76353	25kb
ENSG00000241058	10	18809490	18965551	536	19	120000	-1.066	0.85678	NSUN6	76353	25kb
ENSG00000271786	10	18906405	18969123	202	20	120000	-1.5502	0.93945	RP11-139J15.7	76353	25kb
ENSG00000165997	10	18923334	18995568	258	28	120000	-2.2668	0.9883	ARL5B	76353	25kb
ENSG00000204740	10	19467779	20104330	2505	101	120000	1.4031	0.08029	MALRD1	76353	25kb
ENSG00000120594	10	20080168	20603785	2135	99	120000	0.14495	0.44237	PLXDC2	76353	25kb
ENSG00000078114	10	21043902	21488116	1120	71	120000	2.1231	0.016874	NEBL	76353	25kb
ENSG00000204683	10	21389692	21460488	164	19	120000	2.246	0.012353	C10orf113	76353	25kb
ENSG00000204682	10	21756587	21811191	84	16	120000	1.3044	0.096043	CASC10	76353	25kb
ENSG00000180592	10	21777407	21839611	60	14	120000	0.56699	0.28536	SKIDA1	76353	25kb
ENSG00000078403	10	21798094	22057559	252	31	120000	-0.81287	0.79185	MLLT10	76353	25kb
ENSG00000136770	10	22020466	22317698	476	31	120000	-0.63662	0.73781	DNAJC1	76353	25kb
ENSG00000223601	10	22472743	22523950	126	12	120000	0.94535	0.17224	EBLN1	76353	25kb
ENSG00000148444	10	22579903	22634235	40	13	120000	-1.1655	0.8781	COMMD3	76353	25kb
ENSG00000269897	10	22580317	22643471	46	14	120000	-1.2728	0.89845	COMMD3-BMI1	76353	25kb
ENSG00000168283	10	22585140	22645413	44	11	120000	-1.344	0.91053	BMI1	76353	25kb
ENSG00000077327	10	22609399	22768153	233	17	120000	-0.3022	0.61875	SPAG6	76353	25kb
ENSG00000150867	10	22798778	23028484	710	38	120000	0.9253	0.17741	PIP4K2A	76353	25kb
ENSG00000165309	10	23191953	23351518	387	30	120000	0.55168	0.29058	ARMC3	76353	25kb
ENSG00000148450	10	23359435	23435942	170	28	120000	-0.8945	0.81447	MSRB2	76353	25kb
ENSG00000168267	10	23456256	23508181	71	20	120000	-1.3128	0.90537	PTF1A	76353	25kb
ENSG00000204566	10	23467745	23553745	141	22	120000	-1.761	0.96088	C10orf115	76353	25kb
ENSG00000179133	10	23531124	23658774	278	18	120000	-1.2153	0.88788	C10orf67	76353	25kb
ENSG00000165312	10	23703198	23756308	99	10	120000	0.5819	0.28032	OTUD1	76353	25kb
ENSG00000120549	10	23958675	24861772	2579	156	120000	1.8225	0.034186	KIAA1217	76353	25kb
ENSG00000269879	10	24745273	24795632	152	19	120000	3.0125	0.0012956	AL353583.1	76353	25kb
ENSG00000107863	10	24847538	25037597	350	30	120000	1.2624	0.1034	ARHGAP21	76353	25kb
ENSG00000099256	10	25112536	25266533	519	45	120000	1.2237	0.11052	PRTFDC1	76353	25kb
ENSG00000151023	10	25245908	25330089	178	18	120000	-0.4137	0.66045	ENKUR	76353	25kb
ENSG00000185875	10	25280587	25340593	129	12	120000	-0.69281	0.75579	THNSL1	76353	25kb
ENSG00000151025	10	25438991	25916155	1781	63	120000	0.85252	0.19696	GPR158	76353	25kb
ENSG00000095777	10	26198196	26526456	1305	26	120000	1.5833	0.056675	MYO3A	76353	25kb
ENSG00000136750	10	26480236	26618487	429	18	120000	1.049	0.14708	GAD2	76353	25kb
ENSG00000077420	10	26702132	26881732	623	50	120000	1.3819	0.083497	APBB1IP	76353	25kb
ENSG00000148459	10	26961588	27060727	346	25	120000	-1.4161	0.92163	PDSS1	76353	25kb
ENSG00000136754	10	27010522	27175016	467	26	120000	-0.6046	0.72728	ABI1	76353	25kb
ENSG00000107890	10	27255843	27414421	520	24	120000	0.12759	0.44924	ANKRD26	76353	25kb
ENSG00000136758	10	27374383	27469195	365	19	120000	0.024912	0.49006	YME1L1	76353	25kb
ENSG00000120539	10	27418753	27500853	315	18	120000	-0.042528	0.51696	MASTL	76353	25kb
ENSG00000107897	10	27459146	27556059	479	22	120000	-0.43278	0.66741	ACBD5	76353	25kb
ENSG00000182077	10	27662116	27728297	261	17	120000	1.5798	0.057073	PTCHD3	76353	25kb
ENSG00000099246	10	27768197	27856143	192	17	120000	-1.4226	0.92257	RAB18	76353	25kb
ENSG00000150051	10	27936804	28059989	298	30	120000	-0.26545	0.60467	MKX	76353	25kb
ENSG00000169126	10	28039115	28312977	624	37	120000	-0.064852	0.52585	ARMC4	76353	25kb
ENSG00000150054	10	28314922	28648415	1277	55	120000	0.66604	0.25269	MPP7	76353	25kb
ENSG00000095787	10	28796422	28937041	292	18	120000	0.55816	0.28837	WAC	76353	25kb
ENSG00000095739	10	28941271	28996868	158	25	120000	1.7876	0.036922	BAMBI	76353	25kb
ENSG00000204365	10	29110337	29195827	304	25	120000	-0.61769	0.73161	C10orf126	76353	25kb
ENSG00000120563	10	29552990	29632257	393	45	120000	-0.023425	0.50934	LYZL1	76353	25kb
ENSG00000197321	10	29721267	30050710	1233	96	120000	-0.12079	0.54807	SVIL	76353	25kb
ENSG00000165757	10	30276729	30429423	360	43	120000	-1.2883	0.90118	KIAA1462	76353	25kb
ENSG00000107951	10	30573730	30688377	406	42	120000	0.2448	0.4033	MTPAP	76353	25kb
ENSG00000107968	10	30697866	30775762	155	15	120000	-1.0778	0.85944	MAP3K8	76353	25kb
ENSG00000151033	10	30875718	30943691	292	28	120000	-0.51528	0.69682	LYZL2	76353	25kb
ENSG00000183621	10	31084136	31345866	776	33	120000	0.43645	0.33125	ZNF438	76353	25kb
ENSG00000148516	10	31582424	31843742	348	16	120000	0.071452	0.47152	ZEB1	76353	25kb
ENSG00000165322	10	32069365	32242742	327	21	120000	-1.4045	0.91991	ARHGAP12	76353	25kb
ENSG00000170759	10	32272938	32370359	240	20	120000	-1.1765	0.88031	KIF5B	76353	25kb
ENSG00000120616	10	32531679	32692726	454	34	120000	-0.53523	0.70376	EPC1	76353	25kb
ENSG00000216937	10	32710068	32888492	645	28	120000	-1.0143	0.84478	CCDC7	76353	25kb
ENSG00000150076	10	32807297	33196802	1427	31	120000	-1.1897	0.88292	C10ORF68	76353	25kb
ENSG00000150093	10	33164247	33319720	452	41	120000	1.34	0.090121	ITGB1	76353	25kb
ENSG00000099250	10	33441420	33650190	795	70	120000	-0.58497	0.72072	NRP1	76353	25kb
ENSG00000148498	10	34373488	35129253	1702	70	120000	-0.85107	0.80263	PARD3	76353	25kb
ENSG00000108094	10	35272479	35404570	304	11	120000	-0.18268	0.57248	CUL2	76353	25kb
ENSG00000095794	10	35390719	35526886	292	10	120000	-0.10666	0.54247	CREM	76353	25kb
ENSG00000108100	10	35510953	35885852	580	28	120000	0.46777	0.31998	CCNY	76353	25kb
ENSG00000177291	10	35869338	35922863	160	30	120000	-1.404	0.91984	GJD4	76353	25kb
ENSG00000177283	10	35902177	35955362	114	18	120000	-0.79009	0.78526	FZD8	76353	25kb
ENSG00000229644	10	36785649	36838162	110	14	120000	0.95264	0.17039	NAMPTL	76353	25kb
ENSG00000148513	10	37389785	37698039	595	25	120000	3.2488	0.00057951	ANKRD30A	76353	25kb
ENSG00000256892	10	37865366	37916859	190	16	120000	1.425	0.077078	MTRNR2L7	76353	25kb
ENSG00000198105	10	38066751	38172034	341	13	120000	1.1505	0.12497	ZNF248	76353	25kb
ENSG00000175395	10	38213500	38290561	189	15	120000	1.4883	0.068341	ZNF25	76353	25kb

ENSG00000189180	10	38274578	38379016	270	16	120000	1.6838	0.046113	ZNF33A	76353	25kb
ENSG00000075407	10	38358264	38439472	243	16	120000	0.80867	0.20935	ZNF37A	76353	25kb
ENSG00000225119	10	38692074	38761820	69	10	120000	0.47687	0.31673	LINC00999	76353	25kb
ENSG00000196693	10	43059555	43158992	253	19	120000	0.79521	0.21325	ZNF33B	76353	25kb
ENSG00000165733	10	43253249	43355385	327	21	120000	-0.46156	0.6778	BMS1	76353	25kb
ENSG00000165731	10	43547475	43650799	360	29	120000	-0.60412	0.72712	RET	76353	25kb
ENSG00000169826	10	43608934	43705756	232	20	120000	-0.46255	0.67816	CSGALNACT2	76353	25kb
ENSG00000198915	10	43664983	43787367	393	31	120000	0.24455	0.4034	RASGEF1A	76353	25kb
ENSG00000150201	10	43842090	43896783	161	23	120000	1.4188	0.077984	FXYD4	76353	25kb
ENSG00000169813	10	43856065	43929614	228	25	120000	0.98147	0.16318	HNRNPF	76353	25kb
ENSG00000243660	10	43907282	44016517	391	23	120000	0.50793	0.30575	ZNF487	76353	25kb
ENSG00000196793	10	44026792	44095066	210	17	120000	-0.16932	0.56723	ZNF239	76353	25kb
ENSG00000198298	10	44076855	44138351	193	31	120000	-0.8395	0.79941	ZNF485	76353	25kb
ENSG00000169740	10	44114307	44169304	179	17	120000	0.34602	0.36466	ZNF32	76353	25kb
ENSG00000107562	10	44768038	44906941	560	54	120000	-0.22815	0.59024	CXCL12	76353	25kb
ENSG00000269515	10	44848687	44903739	271	33	120000	-0.50811	0.69431	AL137026.1	76353	25kb
ENSG00000187783	10	45381648	45457450	241	32	120000	0.94912	0.17128	TMEM72	76353	25kb
ENSG00000107551	10	45429855	45516339	261	36	120000	0.41647	0.33853	RASSF4	76353	25kb
ENSG00000165507	10	45441429	45499258	171	33	120000	0.3231	0.37331	C10orf10	76353	25kb
ENSG00000165511	10	45468146	45521336	157	19	120000	0.77332	0.21967	C10orf25	76353	25kb
ENSG00000165512	10	45470923	45525774	161	19	120000	0.79746	0.21259	ZNF22	76353	25kb
ENSG00000256574	10	45773102	45836056	234	13	120000	-0.94236	0.827	OR13A1	76353	25kb
ENSG00000012779	10	45844661	45966561	352	29	120000	-0.67404	0.74986	ALOX5	76353	25kb
ENSG00000165406	10	45925035	46115354	610	19	120000	0.30102	0.3817	MARCH8	76353	25kb
ENSG00000172671	10	46086043	46193228	266	10	120000	0.82623	0.20434	ZFAND4	76353	25kb
ENSG00000172661	10	46197648	46313409	71	6	120000	0.9116	0.18099	FAM21C	76353	25kb
ENSG00000204179	10	46524955	46666045	5	2	120000	-0.75673	0.77539	PTPN20A	76353	25kb
ENSG00000204176	10	46930444	46996400	2	1	120000	0.81023	0.2089	SYT15	76353	25kb
ENSG00000150165	10	47132983	47199093	2	1	120000	-0.82347	0.79488	ANXA8L1	76353	25kb
ENSG00000189090	10	47152204	47206681	2	1	120000	-0.82347	0.79488	FAM25B	76353	25kb
ENSG00000198250	10	47632581	47726443	395	32	120000	-0.30293	0.61903	ANTXRL	76353	25kb
ENSG00000186807	10	47721936	47788041	2	1	120000	-0.39299	0.65284	ANXA8L2	76353	25kb
ENSG00000215033	10	47721962	47795871	2	1	120000	-0.39299	0.65284	AL603965.1	76353	25kb
ENSG00000152726	10	47869023	47974412	11	4	120000	-0.83555	0.7983	FAM21B	76353	25kb
ENSG00000072444	10	47976603	48080018	9	3	120000	-0.76397	0.77756	ASAH2C	76353	25kb
ENSG00000165390	10	48230253	48304200	4	2	120000	1.202	0.11468	ANXA8	76353	25kb
ENSG00000215020	10	48230279	48304199	4	2	120000	1.202	0.11468	AL591684.1	76353	25kb
ENSG00000165388	10	48330024	48398866	125	27	120000	1.5726	0.057907	ZNF488	76353	25kb
ENSG00000107618	10	48356487	48415991	100	29	120000	2.037	0.020824	RBP3	76353	25kb
ENSG00000128802	10	48388092	48441853	119	27	120000	1.7057	0.044029	GDF2	76353	25kb
ENSG00000107623	10	48400815	48463976	165	38	120000	1.0284	0.15188	GDF10	76353	25kb
ENSG00000183675	10	48711874	48852966	2	1	120000	0.37777	0.3528	PTPN20B	76353	25kb
ENSG00000188279	10	49174649	49232818	5	2	120000	0.8467	0.19858	FAM25C	76353	25kb
ENSG00000170324	10	49339601	49507941	236	27	120000	-0.098561	0.53926	FRMPD2	76353	25kb
ENSG00000107643	10	49489698	49672403	450	30	120000	-0.69341	0.75597	MAPK8	76353	25kb
ENSG00000128805	10	49629077	49889310	904	81	120000	-0.36169	0.64121	ARHGAP22	76353	25kb
ENSG00000128815	10	49867921	50216001	1154	85	120000	-1.4988	0.93304	WDFY4	76353	25kb
ENSG00000165383	10	50092528	50168242	279	35	120000	-0.46568	0.67928	LRRC18	76353	25kb
ENSG00000165633	10	50197290	50348554	534	26	120000	-0.6148	0.73066	VSTM4	76353	25kb
ENSG00000172538	10	50314199	50367053	213	21	120000	-0.66469	0.74688	FAM170B	76353	25kb
ENSG00000204161	10	50337773	50421630	325	30	120000	-0.41004	0.65911	C10orf128	76353	25kb
ENSG00000177354	10	50482187	50560537	301	33	120000	0.46566	0.32073	C10orf71	76353	25kb
ENSG00000165606	10	50547237	50628497	228	30	120000	0.95164	0.17064	DRGX	76353	25kb
ENSG00000225830	10	50638414	50772584	337	22	120000	1.6697	0.047489	ERCC6	76353	25kb
ENSG00000243251	10	50698151	50772086	216	25	120000	1.3649	0.086147	PGBD3	76353	25kb
ENSG00000258838	10	50698247	50772584	218	25	120000	1.3762	0.084375	ERCC6-PGBD3	76353	25kb
ENSG00000070748	10	50792141	50926925	408	53	120000	2.792	0.0026192	CHAT	76353	25kb
ENSG00000187714	10	50793347	50845765	133	39	120000	0.54945	0.29135	SLC18A3	76353	25kb
ENSG00000178645	10	50862697	50943307	269	20	120000	2.8537	0.0021606	C10orf53	76353	25kb
ENSG00000197444	10	50917689	50995425	217	22	120000	2.115	0.017215	OGDHL	76353	25kb
ENSG00000227345	10	51001325	51155715	131	17	120000	0.038207	0.48476	PARG	76353	25kb
ENSG00000204169	10	51439162	51511327	81	9	120000	-0.54282	0.70637	AGAP7	76353	25kb
ENSG00000138294	10	51524498	51587517	144	19	120000	0.62428	0.26622	MSMB	76353	25kb
ENSG00000138293	10	51540108	51615734	112	25	120000	1.3069	0.095625	NCOA4	76353	25kb
ENSG00000138297	10	51567080	51648365	39	10	120000	0.57226	0.28357	TIMM23	76353	25kb
ENSG00000204149	10	51723078	51795259	95	21	120000	-0.21188	0.5839	AGAP6	76353	25kb
ENSG00000099290	10	51802648	51918269	107	11	120000	-0.53441	0.70347	FAM21A	76353	25kb
ENSG00000188611	10	51917538	52033370	143	17	120000	0.20231	0.41984	ASAH2	76353	25kb
ENSG00000198964	10	52040360	52409923	915	47	120000	-0.037207	0.51484	SGMS1	76353	25kb
ENSG00000269165	10	52069298	52120876	113	22	120000	0.5024	0.30769	AC069547.1	76353	25kb
ENSG00000269700	10	52127766	52179321	149	19	120000	-0.055666	0.5222	AC069547.2	76353	25kb
ENSG00000204147	10	52474078	52601255	235	18	120000	-0.98147	0.83682	ASAH2B	76353	25kb
ENSG00000148584	10	52534169	52670435	215	33	120000	-0.59952	0.72559	A1CF	76353	25kb
ENSG00000185532	10	52725945	54083110	4394	241	120000	-1.3135	0.90549	PRKG1	76353	25kb
ENSG00000177613	10	53430247	53484355	181	24	120000	-0.31485	0.62356	CSTF2T	76353	25kb
ENSG00000107984	10	54049056	54102802	120	21	120000	0.38021	0.35189	DKK1	76353	25kb
ENSG00000165471	10	54500140	54556460	253	23	120000	0.15503	0.4384	MBL2	76353	25kb
ENSG00000150275	10	55537531	57412702	6412	224	120000	-0.28848	0.61351	PCDH15	76353	25kb
ENSG00000249860	10	57333750	57385488	125	15	120000	1.7247	0.042291	MTRNR2L5	76353	25kb
ENSG00000122952	10	58091989	58146036	69	13	120000	1.0272	0.15217	ZWINT	76353	25kb
ENSG00000151151	10	59926278	60052694	512	10	120000	2.1346	0.016398	IPMK	76353	25kb
ENSG00000122873	10	60003818	60074346	243	16	120000	2.1042	0.01768	CISD1	76353	25kb
ENSG00000072401	10	60069735	60155513	199	31	120000	0.99632	0.15955	UBE2D1	76353	25kb
ENSG00000108064	10	60119782	60183981	167	31	120000	-0.1414	0.55622	TFAM	76353	25kb
ENSG00000122870	10	60247900	60616195	837	49	120000	1.1006	0.13553	BICC1	76353	25kb
ENSG00000165443	10	60911350	61032534	198	15	120000	0.1084	0.45684	PHYHIPL	76353	25kb
ENSG00000148541	10	60980890	61147939	395	35	120000	-0.61779	0.73164	FAM13C	76353	25kb
ENSG00000165449	10	61385523	61520760	356	45	120000	0.56773	0.28511	SLC16A9	76353	25kb
ENSG00000108091	10	61523521	61691414	604	26	120000	1.246	0.10638	CCDC6	76353	25kb
ENSG00000235931	10	61690188	61745671	259	21	120000	0.6384	0.26161	C10orf40	76353	25kb
ENSG00000151150	10	61761056	62518248	2322	93	120000	-1.1749	0.87998	ANK3	76353	25kb
ENSG00000170312	10	62513089	62579610	245	31	120000	-0.48335	0.68557	CDK1	76353	25kb
ENSG00000072422	10	62604196	62786198	495	49	120000	-0.95118	0.82924	RHOBTB1	76353	25kb
ENSG00000196932	10	63141401	63238208	258	25	120000	0.79991	0.21188	TMEM26	76353	25kb
ENSG00000183346	10	63397719	63551524	485	30	120000	0.14764	0.44131	C10orf107	76353	25kb
ENSG00000150347	10	63636059	63881703	582	74	120000	2.4279	0.0075936	ARID5B	76353	25kb

ENSG00000182010	10	63917794	64053466	357	20	120000	-0.93468	0.82502	RTKN2	76353	25kb
ENSG00000138311	10	64108951	64456771	1030	81	120000	-1.7007	0.9555	ZNF365	76353	25kb
ENSG00000181915	10	64539516	64593238	133	19	120000	-1.0349	0.84964	ADO	76353	25kb
ENSG00000122877	10	64546756	64704660	469	30	120000	-0.96985	0.83394	EGR2	76353	25kb
ENSG00000148572	10	64868050	64939783	173	20	120000	-0.56746	0.7148	NRBF2	76353	25kb
ENSG00000171988	10	64901981	65250722	707	26	120000	-0.34735	0.63584	JMJD1C	76353	25kb
ENSG00000165476	10	65256123	65409883	373	27	120000	-0.41939	0.66254	REEP3	76353	25kb
ENSG00000183230	10	67647276	69480927	6689	200	120000	-0.93475	0.82504	CTNNA3	76353	25kb
ENSG00000198739	10	68660764	68884588	588	45	120000	0.068732	0.4726	LRRTM3	76353	25kb
ENSG00000108176	10	69531427	69622924	259	19	120000	-0.80458	0.78947	DNAJC12	76353	25kb
ENSG00000096717	10	69619427	69703147	212	18	120000	-1.0209	0.84635	SIRT1	76353	25kb
ENSG00000148634	10	69656665	69860105	411	26	120000	-1.4019	0.91952	HERC4	76353	25kb
ENSG00000138347	10	69840912	69996774	582	44	120000	0.53103	0.2977	MYPN	76353	25kb
ENSG00000179774	10	69965386	70016871	184	20	120000	0.56902	0.28467	ATOH7	76353	25kb
ENSG00000108187	10	70017417	70117806	331	15	120000	0.030491	0.48784	PBLD	76353	25kb
ENSG00000096746	10	70065931	70127948	142	15	120000	-0.36189	0.64128	HNRNPH3	76353	25kb
ENSG00000204130	10	70075864	70192051	267	14	120000	-0.7976	0.78745	RUFY2	76353	25kb
ENSG00000138346	10	70148821	70256879	355	19	120000	-0.898	0.81541	DNA2	76353	25kb
ENSG00000122912	10	70212756	70312231	338	19	120000	-0.45089	0.67397	SLC25A16	76353	25kb
ENSG00000138336	10	70295413	70479239	552	22	120000	0.17105	0.43209	TET1	76353	25kb
ENSG00000060339	10	70455769	70577134	266	16	120000	-0.21349	0.58453	CCAR1	76353	25kb
ENSG00000165730	10	70562298	70680188	340	30	120000	-0.86112	0.80542	STOX1	76353	25kb
ENSG00000107625	10	70636034	70731603	242	26	120000	-1.5715	0.94196	DDX50	76353	25kb
ENSG00000165732	10	70690884	70769829	200	22	120000	-1.144	0.87368	DDX21	76353	25kb
ENSG00000198954	10	70723487	70801738	231	28	120000	-0.81147	0.79145	KIAA1279	76353	25kb
ENSG00000122862	10	70822862	70889567	200	20	120000	-0.80803	0.79046	SRGN	76353	25kb
ENSG00000122958	10	70858268	70957617	240	19	120000	-0.99747	0.84073	VPS26A	76353	25kb
ENSG00000156502	10	70914988	70993855	251	29	120000	0.34868	0.36366	SUPV3L1	76353	25kb
ENSG00000156510	10	70955059	71052315	349	43	120000	0.92507	0.17747	HKDC1	76353	25kb
ENSG00000156515	10	71004740	71186638	661	82	120000	-0.25724	0.6015	HK1	76353	25kb
ENSG00000075073	10	71138659	71201623	208	26	120000	1.0571	0.14524	TACR2	76353	25kb
ENSG00000099282	10	71186229	71292425	375	25	120000	1.6987	0.044687	TSPAN15	76353	25kb
ENSG00000122859	10	71306454	71357994	147	27	120000	-0.4756	0.68282	NEUROG3	76353	25kb
ENSG00000171224	10	71365007	71418352	271	42	120000	1.1078	0.13397	C10orf35	76353	25kb
ENSG00000235645	10	71419655	71477208	301	37	120000	-0.022205	0.50886	RP11-242G20.1	76353	25kb
ENSG00000197467	10	71536644	71749031	974	135	120000	-0.87124	0.80819	COL13A1	76353	25kb
ENSG00000099284	10	71787552	71897036	332	43	120000	-1.0736	0.8585	H2AFY2	76353	25kb
ENSG00000042286	10	71832979	71917690	294	53	120000	0.9064	0.18236	AIFM2	76353	25kb
ENSG00000156521	10	71872737	71931432	210	34	120000	1.5156	0.064811	TYSND1	76353	25kb
ENSG00000079332	10	71882045	71955279	242	29	120000	1.5328	0.062669	SAR1A	76353	25kb
ENSG00000180817	10	71937586	72018667	295	26	120000	0.54383	0.29328	PPA1	76353	25kb
ENSG00000148734	10	71982085	72068432	345	49	120000	-0.24795	0.59791	NPFFR1	76353	25kb
ENSG00000172731	10	72033729	72167382	517	45	120000	-1.0965	0.86358	LRRC20	76353	25kb
ENSG00000148730	10	72139135	72213374	187	26	120000	-1.3218	0.90689	EIF4EBP2	76353	25kb
ENSG00000156574	10	72167071	72232707	147	26	120000	-1.701	0.95553	NODAL	76353	25kb
ENSG00000197604	10	72169585	72221314	129	21	120000	-1.5459	0.93894	AC022532.1	76353	25kb
ENSG00000107719	10	72213577	72353205	516	55	120000	-1.5208	0.93584	PALD1	76353	25kb
ENSG00000180644	10	72332104	72387531	160	28	120000	-1.5139	0.93498	PRF1	76353	25kb
ENSG00000138316	10	72407559	72547197	694	65	120000	-0.38119	0.64847	ADAMTS14	76353	25kb
ENSG00000166220	10	72505995	72570157	360	42	120000	-0.93394	0.82483	TBATA	76353	25kb
ENSG00000166224	10	72550717	72665930	408	33	120000	-1.5718	0.942	SGPL1	76353	25kb
ENSG00000166228	10	72617037	72673541	190	39	120000	-1.5489	0.9393	PCBD1	76353	25kb
ENSG00000107731	10	72947327	73087621	499	71	120000	0.74367	0.22854	UNC5B	76353	25kb
ENSG00000198246	10	73054015	73148142	368	47	120000	0.28433	0.38808	SLC29A3	76353	25kb
ENSG00000107736	10	73131691	73600702	1514	165	120000	-1.4397	0.92502	CDH23	76353	25kb
ENSG00000214688	10	73446458	73522581	232	42	120000	-1.378	0.9159	C10orf105	76353	25kb
ENSG00000107738	10	73482316	73558255	252	41	120000	-1.321	0.90675	C10orf54	76353	25kb
ENSG00000197746	10	73551055	73636126	251	35	120000	1.0285	0.15185	PSAP	76353	25kb
ENSG00000122863	10	73699123	73798322	290	21	120000	-0.40801	0.65837	CHST3	76353	25kb
ENSG00000107742	10	73793793	73873790	212	20	120000	-0.55028	0.70894	SPOCK2	76353	25kb
ENSG00000138303	10	73831278	74001892	326	24	120000	-0.084702	0.53375	ASCC1	76353	25kb
ENSG00000166295	10	73950787	74020618	183	11	120000	-0.24753	0.59775	ANAPC16	76353	25kb
ENSG00000168209	10	74008678	74060794	117	12	120000	1.2368	0.10808	DDIT4	76353	25kb
ENSG00000148719	10	74067588	74139988	146	11	120000	0.54872	0.2916	DNAJB12	76353	25kb
ENSG00000107745	10	74102098	74410899	493	15	120000	0.76945	0.22081	MICU1	76353	25kb
ENSG00000156026	10	74426889	74672452	243	21	120000	0.72637	0.23381	MCU	76353	25kb
ENSG00000138315	10	74628339	74717791	84	14	120000	-0.41306	0.66022	OIT3	76353	25kb
ENSG00000138308	10	74669928	74739536	77	15	120000	0.60557	0.2724	PLA2G12B	76353	25kb
ENSG00000122884	10	74741975	74881732	231	7	120000	-1.2115	0.88715	P4HA1	76353	25kb
ENSG00000166321	10	74845217	74916586	152	3	120000	-1.2196	0.88869	NUDT13	76353	25kb
ENSG00000122882	10	74864913	74953813	199	4	120000	-1.2463	0.89368	ECD	76353	25kb
ENSG00000138286	10	74902924	75029262	261	4	120000	-1.1857	0.88214	FAM149B1	76353	25kb
ENSG00000213551	10	74918120	75033620	243	5	120000	-1.1542	0.8758	DNAJC9	76353	25kb
ENSG00000182180	10	74981510	75037451	128	4	120000	-1.1272	0.87017	MRPS16	76353	25kb
ENSG00000156042	10	74988517	75143617	363	5	120000	-1.1349	0.87179	TTC18	76353	25kb
ENSG00000138279	10	75109859	75198834	112	7	120000	-0.60935	0.72885	ANXA7	76353	25kb
ENSG00000166343	10	75158339	75218319	56	6	120000	-0.75978	0.77631	MSS51	76353	25kb
ENSG00000107758	10	75171186	75280782	98	14	120000	-0.54179	0.70602	PPP3CB	76353	25kb
ENSG00000166348	10	75232296	75410711	274	14	120000	-1.0639	0.85631	USP54	76353	25kb
ENSG00000177791	10	75366412	75426515	97	10	120000	-1.1447	0.87384	MYOZ1	76353	25kb
ENSG00000166317	10	75379639	75448561	75	10	120000	-0.88112	0.81087	SYNPO2L	76353	25kb
ENSG00000172650	10	75409033	75482639	51	8	120000	0.14691	0.4416	AGAP5	76353	25kb
ENSG00000176986	10	75479120	75556919	105	13	120000	0.16095	0.43606	SEC24C	76353	25kb
ENSG00000267964	10	75506935	75557378	54	9	120000	-0.13318	0.55298	AC022400.2	76353	25kb
ENSG00000196968	10	75507049	75565009	63	9	120000	-0.2128	0.58426	FUT11	76353	25kb
ENSG00000172586	10	75516805	75568410	55	8	120000	-0.32807	0.62857	CHCHD1	76353	25kb
ENSG00000214655	10	75520340	75586551	78	10	120000	-0.034043	0.51358	ZSWIM8	76353	25kb
ENSG00000272916	10	75531272	75596409	74	11	120000	-0.044755	0.51785	RP11-574K11.31	76353	25kb
ENSG00000166507	10	75536669	75596589	70	11	120000	0.003074	0.49877	NDST2	76353	25kb
ENSG00000148660	10	75547259	75659343	154	15	120000	0.17335	0.43119	CAMK2G	76353	25kb
ENSG00000122861	10	75643935	75702255	124	12	120000	0.029982	0.48804	PLAU	76353	25kb
ENSG00000222047	10	75644727	75707535	138	15	120000	-0.063776	0.52543	C10orf55	76353	25kb
ENSG00000035403	10	75732872	75904918	296	27	120000	-1.3666	0.91413	VCL	76353	25kb
ENSG00000185009	10	75856524	75935821	164	14	120000	-0.93708	0.82564	AP3M1	76353	25kb
ENSG00000156110	10	75885960	76494061	984	27	120000	-0.47567	0.68285	ADK	76353	25kb
ENSG00000156650	10	76560340	76817380	394	25	120000	-0.85848	0.80469	KAT6B	76353	25kb

ENSG00000188716	10	76772594	76843272	186	31	120000	-1.352	0.91181	DUPD1	76353	25kb
ENSG00000079393	10	76829192	76893979	205	26	120000	-1.4804	0.93061	DUSP13	76353	25kb
ENSG00000156671	10	76834344	76966881	327	24	120000	-1.837	0.9669	SAMD8	76353	25kb
ENSG00000165637	10	76944912	77016206	163	28	120000	-1.7654	0.96125	VDAC2	76353	25kb
ENSG00000165644	10	76968727	77020788	113	23	120000	-1.312	0.90524	COMTD1	76353	25kb
ENSG00000165655	10	77132588	77186664	70	23	120000	-0.46957	0.68067	ZNF503	76353	25kb
ENSG00000148655	10	77335998	78344925	2140	159	120000	-1.9394	0.97378	C10orf11	76353	25kb
ENSG00000156113	10	78604359	79423353	2249	170	120000	-0.14249	0.55665	KCNMA1	76353	25kb
ENSG00000151208	10	79525549	79711378	540	23	120000	0.74647	0.22769	DLG5	76353	25kb
ENSG00000204049	10	79601633	79654368	152	13	120000	0.7875	0.2155	AL391421.1	76353	25kb
ENSG00000148606	10	79709907	79814303	185	25	120000	0.086826	0.4654	POLR3A	76353	25kb
ENSG00000138326	10	79768518	79841570	110	16	120000	0.58849	0.2781	RPS24	76353	25kb
ENSG00000108175	10	80803792	81101276	998	134	120000	-0.41055	0.6593	ZMIZ1	76353	25kb
ENSG00000108179	10	81082225	81140093	189	21	120000	1.6924	0.045286	PPIF	76353	25kb
ENSG00000165424	10	81117081	81230383	469	69	120000	0.96325	0.16771	ZCCHC24	76353	25kb
ENSG00000235705	10	81241126	81295678	151	19	120000	0.72312	0.2348	AL133481.1	76353	25kb
ENSG00000253626	10	81247357	81301188	145	20	120000	0.57545	0.28249	EIF5AL1	76353	25kb
ENSG00000185303	10	81290608	81345153	231	26	120000	-0.98266	0.83711	SFTPA2	76353	25kb
ENSG00000122852	10	81345695	81400196	251	25	120000	-1.5001	0.9332	SFTPA1	76353	25kb
ENSG00000188199	10	81437983	81499437	82	9	120000	0.27117	0.39313	NUTM2B	76353	25kb
ENSG00000228570	10	81576114	81635632	72	13	120000	-1.7081	0.9562	NUTM2E	76353	25kb
ENSG00000133661	10	81672496	81767370	534	26	120000	-0.52217	0.69922	SFTPD	76353	25kb
ENSG00000133678	10	81813402	81877313	218	24	120000	-0.86731	0.80711	TMEM254	76353	25kb
ENSG00000189129	10	81866438	81930115	329	37	120000	-0.76663	0.77835	PLAC9	76353	25kb
ENSG00000122359	10	81885645	81990328	408	32	120000	-1.2999	0.90319	ANXA11	76353	25kb
ENSG00000204038	10	81984466	82038395	170	21	120000	-0.48608	0.68655	AL359195.1	76353	25kb
ENSG00000151224	10	82006576	82074440	255	21	120000	-0.71878	0.76386	MAT1A	76353	25kb
ENSG00000170788	10	82070861	82141511	239	17	120000	-0.24356	0.59622	DYDC1	76353	25kb
ENSG00000133665	10	82079501	82152829	250	16	120000	-0.29459	0.61585	DYDC2	76353	25kb
ENSG00000122378	10	82142585	82217753	271	16	120000	-1.2087	0.88661	FAM213A	76353	25kb
ENSG00000108219	10	82188922	82317879	348	30	120000	-1.6878	0.95427	TSPAN14	76353	25kb
ENSG00000178217	10	82272658	82431316	547	53	120000	-0.56564	0.71418	SH2D4B	76353	25kb
ENSG00000185737	10	83610070	84771935	3312	182	120000	-0.16976	0.5674	NRG3	76353	25kb
ENSG00000165678	10	85874196	85938001	206	29	120000	0.64721	0.25875	GHITM	76353	25kb
ENSG00000188373	10	85908494	85970050	166	36	120000	-0.59972	0.72565	C10orf99	76353	25kb
ENSG00000148600	10	85929410	86004377	240	32	120000	-0.7372	0.7695	CDHR1	76353	25kb
ENSG00000204033	10	85955254	86010345	181	24	120000	-0.65715	0.74446	LRIT2	76353	25kb
ENSG00000148602	10	85966349	86026217	203	31	120000	-0.2223	0.58796	LRIT1	76353	25kb
ENSG00000148604	10	85979809	86044716	210	35	120000	-0.38073	0.6483	RGR	76353	25kb
ENSG00000107771	10	86063342	86303273	751	28	120000	-0.62514	0.73406	CCSER2	76353	25kb
ENSG00000182771	10	87334312	88151250	2455	150	120000	1.6007	0.05472	GRID1	76353	25kb
ENSG00000062650	10	88170013	88306572	354	25	120000	-1.0675	0.85713	WAPAL	76353	25kb
ENSG00000122375	10	88389314	88451605	297	27	120000	-0.084097	0.53351	OPN4	76353	25kb
ENSG00000122367	10	88403206	88520825	428	47	120000	-0.55005	0.70886	LDB3	76353	25kb
ENSG00000107779	10	88491407	88717595	567	32	120000	-0.18564	0.57364	BMPR1A	76353	25kb
ENSG00000173269	10	88670297	88754238	238	38	120000	2.0968	0.018007	MMRN2	76353	25kb
ENSG00000173267	10	88693375	88748017	163	24	120000	1.9995	0.022778	SNCG	76353	25kb
ENSG00000148671	10	88702949	88768691	189	27	120000	2.2329	0.012777	ADIRF	76353	25kb
ENSG00000188100	10	88755063	88809487	129	25	120000	3.7062	0.00010521	FAM25A	76353	25kb
ENSG00000148672	10	88785243	88879623	218	23	120000	4.0602	2.4517e-05	GLUD1	76353	25kb
ENSG00000122376	10	88828918	88976225	266	21	120000	4.3647	6.3647e-06	FAM35A	76353	25kb
ENSG00000184923	10	88960205	89021665	42	13	120000	3.6317	0.00014078	NUTM2A	76353	25kb
ENSG00000214562	10	89092425	89155452	45	13	120000	3.585	0.00016857	NUTM2D	76353	25kb
ENSG00000107789	10	89239632	89338217	196	31	120000	1.7466	0.04035	MINPP1	76353	25kb
ENSG00000198682	10	89394370	89532462	412	38	120000	0.17181	0.43179	PAPSS2	76353	25kb
ENSG00000138138	10	89486269	89626100	251	19	120000	0.92833	0.17662	ATAD1	76353	25kb
ENSG00000227268	10	89593918	89648194	84	21	120000	0.86019	0.19484	KLLN	76353	25kb
ENSG00000171862	10	89597870	89756687	226	35	120000	1.6262	0.051949	PTEN	76353	25kb
ENSG00000184719	10	90008621	90369287	778	46	120000	-0.25724	0.6015	RNLS	76353	25kb
ENSG00000204022	10	90321510	90391733	87	15	120000	-0.12381	0.54927	LIPJ	76353	25kb
ENSG00000182333	10	90399198	90463571	163	25	120000	0.98392	0.16258	LIPF	76353	25kb
ENSG00000204021	10	90459301	90537543	337	17	120000	1.2437	0.1068	LIPK	76353	25kb
ENSG00000204020	10	90496163	90562999	269	18	120000	0.19552	0.42249	LIPN	76353	25kb
ENSG00000173239	10	90537487	90605303	176	31	120000	-0.07142	0.52847	LIPM	76353	25kb
ENSG00000152766	10	90556889	90636575	191	34	120000	-0.60041	0.72588	ANKRD22	76353	25kb
ENSG00000138134	10	90614491	90759910	362	35	120000	-0.45056	0.67385	STAMBPL1	76353	25kb
ENSG00000107796	10	90669831	90776147	303	37	120000	-0.38243	0.64893	ACTA2	76353	25kb
ENSG00000026103	10	90725414	90800542	292	30	120000	-2.024	0.97852	FAS	76353	25kb
ENSG00000138135	10	90940694	90992071	130	20	120000	-1.0854	0.86112	CH25H	76353	25kb
ENSG00000107798	10	90948326	91199314	682	61	120000	-0.54841	0.7083	LIPA	76353	25kb
ENSG00000119922	10	91036712	91094033	127	23	120000	-0.043952	0.51753	IFIT2	76353	25kb
ENSG00000119917	10	91062651	91125728	206	19	120000	-0.045207	0.51803	IFIT3	76353	25kb
ENSG00000204010	10	91112813	91169962	137	23	120000	0.40463	0.34287	IFIT1B	76353	25kb
ENSG00000185745	10	91127303	91188745	164	23	120000	0.014671	0.49415	IFIT1	76353	25kb
ENSG00000152778	10	91149343	91205758	157	25	120000	0.21824	0.41362	IFIT5	76353	25kb
ENSG00000152779	10	91165051	91341398	492	65	120000	-0.3739	0.64576	SLC16A12	76353	25kb
ENSG00000152782	10	91317745	91430215	406	37	120000	-1.3003	0.90325	PANK1	76353	25kb
ENSG00000138182	10	91436367	91559700	423	18	120000	-0.8175	0.79318	KIF20B	76353	25kb
ENSG00000148680	10	92475578	92642671	432	27	120000	2.0342	0.020967	HTR7	76353	25kb
ENSG00000148688	10	92606473	92693312	188	28	120000	2.3424	0.0095792	RPP30	76353	25kb
ENSG00000148677	10	92646853	92706033	164	34	120000	1.741	0.040845	ANKRD1	76353	25kb
ENSG00000180628	10	92954908	93069088	258	17	120000	-0.62601	0.73434	PCGF5	76353	25kb
ENSG00000165338	10	93145096	93299586	238	15	120000	-0.62033	0.73248	HECTD2	76353	25kb
ENSG00000119938	10	93363199	93417811	183	19	120000	0.0775	0.46911	PPP1R3C	76353	25kb
ENSG00000107854	10	93533069	93650033	260	15	120000	0.11649	0.45363	TNKS2	76353	25kb
ENSG00000174721	10	93641346	93694240	132	25	120000	-0.062239	0.52481	FGFBP3	76353	25kb
ENSG00000095564	10	93658526	93815082	283	19	120000	-0.066769	0.52662	BTAF1	76353	25kb
ENSG00000107864	10	93781449	94075844	392	22	120000	-0.3906	0.65195	CPEB3	76353	25kb
ENSG00000198060	10	94025920	94138721	158	14	120000	-0.37995	0.64801	MARCH5	76353	25kb
ENSG00000119912	10	94186441	94358833	420	33	120000	-0.26148	0.60314	IDE	76353	25kb
ENSG00000138160	10	94328043	94440150	377	25	120000	-0.64187	0.73952	KIF11	76353	25kb
ENSG00000152804	10	94422945	94480403	133	17	120000	0.12528	0.45015	HHEX	76353	25kb
ENSG00000138190	10	94565935	94844250	637	29	120000	-0.047284	0.51886	EXOC6	76353	25kb
ENSG00000187553	10	94796021	94853454	146	24	120000	0.82786	0.20387	CYP26C1	76353	25kb
ENSG00000095596	10	94808232	94862647	136	32	120000	0.60738	0.2718	CYP26A1	76353	25kb
ENSG00000138119	10	95041186	95267074	919	68	120000	0.67504	0.24983	MYOF	76353	25kb

ENSG00000138180	10	95231389	95313849	348	33	120000	1.0398	0.14921	CEP55	76353	25kb
ENSG00000186188	10	95301422	95389237	324	47	120000	0.014029	0.4944	FFAR4	76353	25kb
ENSG00000138207	10	95326444	95386501	228	42	120000	-0.37428	0.6459	RBP4	76353	25kb
ENSG00000095464	10	95347345	95450767	378	54	120000	0.65532	0.25613	PDE6C	76353	25kb
ENSG00000148690	10	95402640	95487329	307	17	120000	1.2235	0.11056	FRA10AC1	76353	25kb
ENSG00000108231	10	95492566	95582916	248	51	120000	-0.11071	0.54408	LGI1	76353	25kb
ENSG00000176273	10	95628730	95740819	175	17	120000	0.74481	0.22819	SLC35G1	76353	25kb
ENSG00000138193	10	95728746	96117580	780	57	120000	-0.24073	0.59512	PLCE1	76353	25kb
ENSG00000173145	10	96050004	96147716	266	22	120000	0.32436	0.37283	NOC3L	76353	25kb
ENSG00000108239	10	96137261	96320687	300	17	120000	0.90303	0.18326	TBC1D12	76353	25kb
ENSG00000119969	10	96280547	96398662	225	19	120000	1.1882	0.11737	HELLS	76353	25kb
ENSG00000108242	10	96418251	96520947	235	19	120000	0.97162	0.16562	CYP2C18	76353	25kb
ENSG00000165841	10	96422911	96638017	537	20	120000	0.93376	0.17522	CYP2C19	76353	25kb
ENSG00000138109	10	96673415	96774147	350	21	120000	0.3428	0.36587	CYP2C9	76353	25kb
ENSG00000138115	10	96771530	96854254	285	21	120000	-0.0042487	0.50169	CYP2C8	76353	25kb
ENSG00000173124	10	96928957	97013685	266	16	120000	0.0047975	0.49809	C10orf129	76353	25kb
ENSG00000107438	10	96972329	97075781	374	48	120000	-0.80941	0.79086	PDLIM1	76353	25kb
ENSG00000095637	10	97046528	97346171	1064	82	120000	0.75605	0.22481	SORBS1	76353	25kb
ENSG00000059573	10	97340696	97441463	326	25	120000	-0.42645	0.66511	ALDH18A1	76353	25kb
ENSG00000119977	10	97398158	97478900	226	29	120000	-0.41503	0.66094	TCTN3	76353	25kb
ENSG00000138185	10	97446536	97662023	617	25	120000	0.0021045	0.49916	ENTPD1	76353	25kb
ENSG00000270099	10	97595305	97709601	316	28	120000	0.13783	0.44519	RP11-248J23.7	76353	25kb
ENSG00000173088	10	97642360	97723480	244	33	120000	0.11189	0.45546	C10orf131	76353	25kb
ENSG00000269948	10	97684725	97769785	270	31	120000	1.5339	0.062529	RP11-248J23.6	76353	25kb
ENSG00000269920	10	97708786	97817432	281	35	120000	1.7395	0.040973	RP11-690P14.4	76353	25kb
ENSG00000188649	10	97708786	97817441	281	35	120000	1.7395	0.040973	CC2D2B	76353	25kb
ENSG00000107443	10	97778151	97845627	112	19	120000	1.875	0.030396	CCNJ	76353	25kb
ENSG00000095585	10	97926458	98056344	395	61	120000	1.597	0.055127	BLNK	76353	25kb
ENSG00000107447	10	98039085	98123321	263	18	120000	2.6349	0.004208	DNTT	76353	25kb
ENSG00000197430	10	98077973	98144092	211	24	120000	2.1776	0.014719	OPALIN	76353	25kb
ENSG00000095587	10	98099363	98298675	586	54	120000	1.6337	0.051162	TLL2	76353	25kb
ENSG00000077147	10	98252866	98372209	322	20	120000	0.27278	0.39251	TM9SF3	76353	25kb
ENSG00000155629	10	98328069	98505271	480	65	120000	0.70822	0.2394	PIK3AP1	76353	25kb
ENSG00000196233	10	98567017	98765800	252	19	120000	0.27745	0.39072	LCOR	76353	25kb
ENSG00000155640	10	98716041	98770582	56	13	120000	-0.014115	0.50563	C10orf12	76353	25kb
ENSG00000187122	10	98732795	98970677	630	82	120000	0.25931	0.3977	SLIT1	76353	25kb
ENSG00000269891	10	98732795	99077413	1005	84	120000	-0.2715	0.607	ARHGAP19-SLIT1	76353	25kb
ENSG00000213390	10	98953996	99077413	421	24	120000	-0.68263	0.75258	ARHGAP19	76353	25kb
ENSG00000165879	10	99054022	99106672	186	18	120000	-0.89862	0.81557	FRAT1	76353	25kb
ENSG00000181274	10	99067255	99119458	165	18	120000	-0.77813	0.78176	FRAT2	76353	25kb
ENSG00000052749	10	99091115	99186127	293	15	120000	-0.48284	0.6854	RRP12	76353	25kb
ENSG00000224474	10	99154228	99210831	143	16	120000	-0.73557	0.769	AL355490.1	76353	25kb
ENSG00000171314	10	99160917	99218198	113	19	120000	-0.87616	0.80953	PGAM1	76353	25kb
ENSG00000171311	10	99170899	99230774	108	20	120000	-1.2567	0.89557	EXOSC1	76353	25kb
ENSG00000171307	10	99180927	99242127	98	20	120000	-1.2968	0.90265	ZDHHC16	76353	25kb
ENSG00000155229	10	99193081	99283551	167	24	120000	-1.6625	0.9518	MMS19	76353	25kb
ENSG00000165886	10	99233625	99355966	262	26	120000	-0.79076	0.78546	UBTD1	76353	25kb
ENSG00000165887	10	99307198	99368641	145	21	120000	0.39102	0.34789	ANKRD2	76353	25kb
ENSG00000241935	10	99319080	99397559	173	33	120000	0.31993	0.37451	HOGA1	76353	25kb
ENSG00000249967	10	99319131	99458667	326	38	120000	0.08395	0.46655	PI4K2A	76353	25kb
ENSG00000155252	10	99319131	99461191	333	38	120000	0.053061	0.47884	PI4K2A	76353	25kb
ENSG00000203942	10	99324450	99375690	118	25	120000	0.65734	0.25548	C10orf62	76353	25kb
ENSG00000171160	10	99349310	99418344	135	21	120000	0.19437	0.42294	MORN4	76353	25kb
ENSG00000119986	10	99412181	99472080	163	22	120000	0.52957	0.29821	AVPI1	76353	25kb
ENSG00000155254	10	99447930	99502907	168	29	120000	1.8592	0.031502	MARVELD1	76353	25kb
ENSG00000155256	10	99471878	99545664	214	19	120000	2.0488	0.020243	ZFYVE27	76353	25kb
ENSG00000120057	10	99501508	99556709	153	12	120000	2.0927	0.018186	SFRP5	76353	25kb
ENSG00000155265	10	99584996	99656337	155	32	120000	0.5442	0.29315	GOLGA7B	76353	25kb
ENSG00000095713	10	99599757	99815585	402	55	120000	-0.3731	0.64546	CRTAC1	76353	25kb
ENSG00000269783	10	99602889	99656294	124	28	120000	0.058288	0.47676	GOLGA7B	76353	25kb
ENSG00000166024	10	99869387	100029654	295	17	120000	0.3681	0.3564	R3HCC1L	76353	25kb
ENSG00000138131	10	99982447	100053007	170	30	120000	-0.23381	0.59243	LOXL4	76353	25kb
ENSG00000119943	10	100118322	100199941	294	17	120000	-0.15129	0.56013	PYROXD2	76353	25kb
ENSG00000107521	10	100150955	100231684	350	28	120000	-0.38737	0.65076	HPS1	76353	25kb
ENSG00000172987	10	100193875	101020619	1975	66	120000	-0.27108	0.60684	HPSE2	76353	25kb
ENSG00000119946	10	101063856	101179087	276	28	120000	-0.64922	0.7419	CNNM1	76353	25kb
ENSG00000120053	10	101131627	101215381	142	26	120000	0.19686	0.42197	GOT1	76353	25kb
ENSG00000119919	10	101267690	101321278	176	16	120000	-1.0015	0.8417	NKX2-3	76353	25kb
ENSG00000155287	10	101345282	101405366	174	20	120000	-1.0681	0.85725	SLC25A28	76353	25kb
ENSG00000198018	10	101394263	101490997	172	12	120000	-1.5317	0.93721	ENTPD7	76353	25kb
ENSG00000119929	10	101437315	101540891	182	26	120000	-1.0166	0.84532	CUTC	76353	25kb
ENSG00000014919	10	101446601	101516857	129	18	120000	-1.0851	0.86107	COX15	76353	25kb
ENSG00000023839	10	101517489	101636949	276	20	120000	-1.0047	0.84248	ABCC2	76353	25kb
ENSG00000107554	10	101610334	101794676	603	29	120000	-1.3121	0.90525	DNMBP	76353	25kb
ENSG00000120054	10	101776950	101866634	273	25	120000	-0.79477	0.78663	CPN1	76353	25kb
ENSG00000107566	10	101884851	101973091	206	22	120000	-1.347	0.911	ERLIN1	76353	25kb
ENSG00000213341	10	101923055	102014376	214	17	120000	-1.5507	0.93951	CHUK	76353	25kb
ENSG00000095485	10	101967055	102052437	207	15	120000	-1.6826	0.95378	CWF19L1	76353	25kb
ENSG00000196072	10	102008713	102071469	155	20	120000	-1.095	0.86323	BLOC1S2	76353	25kb
ENSG00000107593	10	102022903	102115243	225	40	120000	-1.0888	0.86187	PKD2L1	76353	25kb
ENSG00000099194	10	102081881	102149591	178	28	120000	-1.4522	0.92677	SCD	76353	25kb
ENSG00000075290	10	102197798	102268501	131	6	120000	-1.6341	0.94889	WNT8B	76353	25kb
ENSG00000075826	10	102221399	102314628	185	8	120000	-1.6147	0.94681	SEC31B	76353	25kb
ENSG00000255339	10	102240385	102314638	149	7	120000	-1.5605	0.94068	NDUFB8	76353	25kb
ENSG00000166136	10	102242203	102314757	146	7	120000	-1.5551	0.94004	NDUFB8	76353	25kb
ENSG00000166135	10	102263829	102344755	173	6	120000	-1.4595	0.92778	HIF1AN	76353	25kb
ENSG00000075891	10	102470360	102614698	324	38	120000	0.69339	0.24403	PAX2	76353	25kb
ENSG00000119906	10	102647326	102749893	159	18	120000	-0.8	0.78814	FAM178A	76353	25kb
ENSG00000055950	10	102704215	102772272	114	15	120000	-0.54524	0.70721	MRPL43	76353	25kb
ENSG00000095539	10	102704275	102770628	109	14	120000	-0.54202	0.7061	SEMA4G	76353	25kb
ENSG00000107815	10	102722124	102779158	113	16	120000	-0.33457	0.63102	C10orf2	76353	25kb
ENSG00000107816	10	102731375	102792593	145	26	120000	-0.45033	0.67376	LZTS2	76353	25kb
ENSG00000186862	10	102742440	102815890	164	38	120000	-0.43123	0.66685	PDZD7	76353	25kb
ENSG00000107819	10	102765991	102825998	124	39	120000	-0.013608	0.50543	SFXN3	76353	25kb
ENSG00000107821	10	102796598	102852888	115	21	120000	-0.15699	0.56238	KAZALD1	76353	25kb
ENSG00000236311	10	102824078	102915883	283	31	120000	-0.44483	0.67178	TLX1NB	76353	25kb

ENSG00000269259	10	102857536	102908624	155	27	120000	-0.059013	0.52353	HUG1	76353	25kb
ENSG00000107807	10	102864257	102922545	204	29	120000	-0.32171	0.62616	TLX1	76353	25kb
ENSG00000138136	10	102961733	103014551	111	25	120000	-0.49405	0.68936	LBX1	76353	25kb
ENSG00000166167	10	103088820	103342078	517	17	120000	-1.5223	0.93603	BTRC	76353	25kb
ENSG00000166171	10	103305317	103394425	162	16	120000	-1.4846	0.93118	DPCD	76353	25kb
ENSG00000166169	10	103313639	103373027	113	14	120000	-1.5425	0.93852	POLL	76353	25kb
ENSG00000107829	10	103345423	103480052	175	17	120000	-1.6184	0.94722	FBXW4	76353	25kb
ENSG00000107831	10	103504899	103560854	50	14	120000	-0.38923	0.65145	FGF8	76353	25kb
ENSG00000107833	10	103516082	103568170	57	12	120000	-0.045281	0.51806	NPM3	76353	25kb
ENSG00000198408	10	103519200	103603696	105	17	120000	-0.40275	0.65643	MGEA5	76353	25kb
ENSG00000120049	10	103560731	103628677	92	16	120000	-0.30437	0.61958	KCNIP2	76353	25kb
ENSG00000120029	10	103580356	103840950	329	26	120000	0.15405	0.43879	C10orf76	76353	25kb
ENSG00000166189	10	103800147	103852792	81	22	120000	0.44306	0.32886	HPS6	76353	25kb
ENSG00000198728	10	103842317	103905210	105	20	120000	0.46955	0.31934	LDB1	76353	25kb
ENSG00000148840	10	103867787	103935082	102	16	120000	0.49147	0.31155	PPRC1	76353	25kb
ENSG00000166197	10	103886933	103948627	85	17	120000	0.64794	0.25851	NOLC1	76353	25kb
ENSG00000119915	10	103961085	104014346	82	14	120000	-0.34163	0.63369	ELOVL3	76353	25kb
ENSG00000107859	10	103964943	104026231	92	16	120000	-0.23253	0.59193	PITX3	76353	25kb
ENSG00000107862	10	103980289	104167656	288	28	120000	-0.56003	0.71227	GBF1	76353	25kb
ENSG00000077150	10	104128867	104187281	80	22	120000	-0.11132	0.54432	NFKB2	76353	25kb
ENSG00000059915	10	104137376	104206296	107	24	120000	-0.031785	0.51268	PSD	76353	25kb
ENSG00000107872	10	104153946	104207893	85	20	120000	0.011373	0.49546	FBXL15	76353	25kb
ENSG00000107874	10	104158002	104217418	99	25	120000	0.1347	0.44642	CUEDC2	76353	25kb
ENSG00000120055	10	104184594	104236300	123	25	120000	-0.22171	0.58773	C10orf95	76353	25kb
ENSG00000138111	10	104196149	104261802	179	17	120000	-0.54426	0.70687	TMEM180	76353	25kb
ENSG00000138107	10	104213986	104287482	218	15	120000	-0.70461	0.75947	ACTR1A	76353	25kb
ENSG00000107882	10	104238744	104418292	399	18	120000	-0.67485	0.75011	SUFU	76353	25kb
ENSG00000171206	10	104379253	104443164	136	22	120000	0.51158	0.30447	TRIM8	76353	25kb
ENSG00000138175	10	104408488	104499164	173	25	120000	0.62093	0.26732	ARL3	76353	25kb
ENSG00000156398	10	104449295	104528249	167	19	120000	0.46808	0.31987	SFXN2	76353	25kb
ENSG00000166272	10	104478727	104601021	315	26	120000	1.205	0.11411	WBP1L	76353	25kb
ENSG00000148795	10	104565288	104622290	144	19	120000	0.89095	0.18648	CYP17A1	76353	25kb
ENSG00000166275	10	104588980	104649718	159	18	120000	0.35264	0.36218	C10orf32	76353	25kb
ENSG00000270316	10	104589029	104686656	258	17	120000	0.085261	0.46603	C10orf32-ASMT	76353	25kb
ENSG00000214435	10	104604273	104686656	221	21	120000	-0.19516	0.57737	AS3MT	76353	25kb
ENSG00000148842	10	104653050	104874978	546	22	120000	-0.3866	0.65047	CNNM2	76353	25kb
ENSG00000076685	10	104820940	104978056	387	23	120000	-0.78503	0.78378	NT5C2	76353	25kb
ENSG00000235376	10	104980644	105032773	78	14	120000	-0.92437	0.82235	RPEL1	76353	25kb
ENSG00000148798	10	105011920	105075108	92	14	120000	-0.82393	0.79501	INA	76353	25kb
ENSG00000156374	10	105037553	105135891	133	16	120000	-0.78865	0.78484	PCGF6	76353	25kb
ENSG00000148835	10	105102724	105173822	85	12	120000	-0.72864	0.76689	TAF5	76353	25kb
ENSG00000173915	10	105123798	105181223	75	13	120000	-0.84194	0.80009	USMG5	76353	25kb
ENSG00000148843	10	105131405	105231049	158	26	120000	-0.77968	0.78221	PDCD11	76353	25kb
ENSG00000138172	10	105181543	105237660	105	16	120000	-0.34673	0.6356	CALHM2	76353	25kb
ENSG00000185933	10	105188144	105243645	109	16	120000	-0.3304	0.62945	CALHM1	76353	25kb
ENSG00000183128	10	105207561	105263997	153	23	120000	0.50633	0.30631	CALHM3	76353	25kb
ENSG00000107954	10	105228736	105377309	355	42	120000	0.55515	0.2894	NEURL1	76353	25kb
ENSG00000107957	10	105323285	105640301	784	68	120000	-0.31396	0.62322	SH3PXD2A	76353	25kb
ENSG00000107960	10	105617300	105702963	198	24	120000	-0.23436	0.59265	OBFC1	76353	25kb
ENSG00000065613	10	105701959	105813991	320	20	120000	-1.218	0.88839	SLK	76353	25kb
ENSG00000065618	10	105766044	105870760	209	33	120000	-0.8533	0.80325	COL17A1	76353	25kb
ENSG00000156384	10	105856816	105911143	100	11	120000	0.81516	0.20749	SFR1	76353	25kb
ENSG00000197748	10	105864646	106017120	336	26	120000	0.051967	0.47928	WDR96	76353	25kb
ENSG00000148834	10	105970114	106052217	177	25	120000	-1.4539	0.92702	GSTO1	76353	25kb
ENSG00000065621	10	106003631	106089703	170	23	120000	-0.93628	0.82543	GSTO2	76353	25kb
ENSG00000148841	10	106046894	106123162	145	20	120000	-1.025	0.84733	ITPRIP	76353	25kb
ENSG00000120051	10	106088522	106239848	268	32	120000	-0.28837	0.61347	CCDC147	76353	25kb
ENSG00000156395	10	106375859	107049993	1940	58	120000	-0.44694	0.67254	SORCS3	76353	25kb
ENSG00000108018	10	108308421	108949292	1795	125	120000	0.27091	0.39323	SORCS1	76353	25kb
ENSG00000108039	10	111599524	111708311	222	27	120000	-1.1098	0.86645	XPNPEP1	76353	25kb
ENSG00000203876	10	111676029	111738649	107	7	120000	-0.30213	0.61872	RP11-451M19.3	76353	25kb
ENSG00000148700	10	111731126	111920323	283	21	120000	-0.88922	0.81306	ADD3	76353	25kb
ENSG00000119950	10	111942363	112072123	333	24	120000	-0.39943	0.65521	MXI1	76353	25kb
ENSG00000119953	10	112025488	112089709	153	23	120000	-0.89857	0.81556	SMNDC1	76353	25kb
ENSG00000138166	10	112232596	112296302	128	21	120000	-2.099	0.98209	DUSP5	76353	25kb
ENSG00000108055	10	112302449	112389394	281	21	120000	-0.88525	0.81199	SMC3	76353	25kb
ENSG00000203867	10	112379155	112624227	805	76	120000	0.52614	0.2994	RBM20	76353	25kb
ENSG00000150593	10	112606565	112684764	165	25	120000	-1.1833	0.88165	PDCD4	76353	25kb
ENSG00000214413	10	112633488	112704032	154	22	120000	-0.93256	0.82448	BBIP1	76353	25kb
ENSG00000108061	10	112654301	112798425	292	24	120000	-0.74725	0.77254	SHOC2	76353	25kb
ENSG00000150594	10	112811790	112865658	122	26	120000	-0.1805	0.57162	ADRA2A	76353	25kb
ENSG00000119927	10	113884624	114000135	273	40	120000	-0.29047	0.61427	GPAM	76353	25kb
ENSG00000119913	10	114018493	114089793	215	31	120000	0.51733	0.30246	TECTB	76353	25kb
ENSG00000197142	10	114108776	114213138	244	28	120000	0.7578	0.22428	ACSL5	76353	25kb
ENSG00000023041	10	114165058	114231672	138	32	120000	1.2293	0.10947	ZDHHC6	76353	25kb
ENSG00000151532	10	114181756	114603503	939	112	120000	0.24101	0.40477	VTI1A	76353	25kb
ENSG00000148737	10	114685009	114952437	568	78	120000	0.30022	0.38201	TCF7L2	76353	25kb
ENSG00000148702	10	115285596	115374361	328	51	120000	-0.25046	0.59888	HABP2	76353	25kb
ENSG00000197893	10	115323475	115448886	472	47	120000	-0.70532	0.75969	NRAP	76353	25kb
ENSG00000165806	10	115413942	115515662	318	23	120000	-0.46921	0.68054	CASP7	76353	25kb
ENSG00000148735	10	115486213	115568188	273	20	120000	-1.461	0.92799	PLEKHS1	76353	25kb
ENSG00000198924	10	115569488	115639142	94	24	120000	-0.63243	0.73645	DCLRE1A	76353	25kb
ENSG00000196865	10	115589420	115701953	138	32	120000	0.91873	0.17912	NHLRC2	76353	25kb
ENSG00000234631	10	115649530	115701949	79	22	120000	1.5405	0.061716	AL162407.1	76353	25kb
ENSG00000043591	10	115778806	115831667	159	13	120000	0.72056	0.23559	ADRB1	76353	25kb
ENSG00000165813	10	115855621	115958979	223	16	120000	-1.1418	0.87322	C10orf118	76353	25kb
ENSG00000095627	10	115914029	116017063	262	14	120000	-1.3839	0.9168	TDRD1	76353	25kb
ENSG00000165816	10	115974018	116076272	269	33	120000	-1.7022	0.95564	VWA2	76353	25kb
ENSG00000169129	10	116029583	116189515	470	75	120000	0.55089	0.29085	AFAP1L2	76353	25kb
ENSG00000099204	10	116165872	116469762	889	74	120000	-0.70457	0.75946	ABLIM1	76353	25kb
ENSG00000151553	10	116556503	116684591	355	24	120000	0.032501	0.48704	FAM160B1	76353	25kb
ENSG00000165832	10	116672952	116762430	231	23	120000	0.036858	0.4853	TRUB1	76353	25kb
ENSG00000107518	10	116828124	117733503	1955	50	120000	0.93647	0.17452	ATRNL1	76353	25kb
ENSG00000151892	10	117791444	118057979	806	75	120000	0.46223	0.32196	GFRA1	76353	25kb
ENSG00000182645	10	118058940	118164541	281	29	120000	-1.2221	0.88916	CCDC172	76353	25kb
ENSG00000203837	10	118162379	118262469	376	23	120000	0.8916	0.1863	PNLIPRP3	76353	25kb

ENSG00000175535	10	118280443	118352367	145	14	120000	0.39528	0.34632	PNLIP	76353	25kb
ENSG00000187021	10	118324897	118393687	230	32	120000	0.63817	0.26168	PNLIPRP1	76353	25kb
ENSG00000165863	10	118398207	118454775	153	28	120000	0.10556	0.45797	C10orf82	76353	25kb
ENSG00000165868	10	118405703	118527085	373	33	120000	0.37441	0.35405	HSPA12A	76353	25kb
ENSG00000188316	10	118584023	118696299	134	24	120000	-1.2228	0.88929	ENO4	76353	25kb
ENSG00000187164	10	118618742	118911097	405	33	120000	-0.24688	0.5975	KIAA1598	76353	25kb
ENSG00000148704	10	118863032	118922812	108	18	120000	-0.16973	0.56739	VAX1	76353	25kb
ENSG00000186795	10	118932000	118994810	193	23	120000	-0.79447	0.78654	KCNK18	76353	25kb
ENSG00000165646	10	118975604	119063941	245	38	120000	-1.6657	0.95212	SLC18A2	76353	25kb
ENSG00000165650	10	119015000	119159978	400	20	120000	-0.5763	0.71779	PDZD8	76353	25kb
ENSG00000170370	10	119276955	119334056	181	23	120000	-0.5209	0.69878	EMX2	76353	25kb
ENSG00000107560	10	119739427	119831114	152	19	120000	0.61138	0.27047	RAB11FIP2	76353	25kb
ENSG00000165669	10	120040401	120126840	148	34	120000	0.36355	0.3581	FAM204A	76353	25kb
ENSG00000119973	10	120327916	120380160	251	35	120000	-0.6377	0.73817	PRLHR	76353	25kb
ENSG00000151893	10	120408679	120539761	436	31	120000	0.81466	0.20763	CACUL1	76353	25kb
ENSG00000188613	10	120764228	120818854	98	14	120000	0.65557	0.25605	NANOS1	76353	25kb
ENSG00000107581	10	120769356	120865316	179	17	120000	0.44955	0.32652	EIF3A	76353	25kb
ENSG00000119979	10	120838598	120922496	212	17	120000	0.3742	0.35413	FAM45A	76353	25kb
ENSG00000183605	10	120875279	120950179	186	22	120000	0.077663	0.46905	SFXN4	76353	25kb
ENSG00000165672	10	120902215	120963345	181	19	120000	0.50571	0.30653	PRDX3	76353	25kb
ENSG00000198873	10	120942101	121240131	992	84	120000	1.692	0.045323	GRK5	76353	25kb
ENSG00000148908	10	121234340	121327220	277	35	120000	1.1858	0.11784	RGS10	76353	25kb
ENSG00000151923	10	121309199	121381541	186	24	120000	0.17253	0.43151	TIAL1	76353	25kb
ENSG00000151929	10	121385882	121462331	251	39	120000	-0.2083	0.5825	BAG3	76353	25kb
ENSG00000198825	10	121460609	121613652	342	18	120000	-0.72092	0.76452	INPP5F	76353	25kb
ENSG00000197771	10	121563972	121677068	360	15	120000	-0.88728	0.81254	MCMBP	76353	25kb
ENSG00000107651	10	121627223	121727014	352	20	120000	-0.9354	0.82521	SEC23IP	76353	25kb
ENSG00000203805	10	122191466	122374367	650	62	120000	-0.45605	0.67582	PPAPDC1A	76353	25kb
ENSG00000177234	10	122332721	122384629	187	39	120000	-0.97156	0.83437	C10orf85	76353	25kb
ENSG00000120008	10	122585687	122694036	387	23	120000	-0.98197	0.83694	WDR11	76353	25kb
ENSG00000066468	10	123212848	123382972	378	74	120000	0.54809	0.29181	FGFR2	76353	25kb
ENSG00000107669	10	123474939	123713316	833	45	120000	0.36031	0.35931	ATE1	76353	25kb
ENSG00000107672	10	123691603	123759732	255	14	120000	0.58741	0.27846	NSMCE4A	76353	25kb
ENSG00000138162	10	123723689	124039060	1255	136	120000	0.79287	0.21393	TACC2	76353	25kb
ENSG00000138152	10	124005821	124122677	428	33	120000	1.0971	0.13631	BTBD16	76353	25kb
ENSG00000107679	10	124109212	124216867	297	25	120000	-2.2997	0.98927	PLEKHA1	76353	25kb
ENSG00000254636	10	124189169	124241868	165	27	120000	-2.2607	0.98811	ARMS2	76353	25kb
ENSG00000166033	10	124196041	124299424	410	35	120000	-1.3165	0.906	HTRA1	76353	25kb
ENSG00000187908	10	124295181	124428252	520	28	120000	-1.6121	0.94653	DMBT1	76353	25kb
ENSG00000183559	10	124432225	124484338	144	27	120000	0.56436	0.28625	C10orf120	76353	25kb
ENSG00000138161	10	124566665	124664146	194	22	120000	-0.65996	0.74536	CUZD1	76353	25kb
ENSG00000213185	10	124583594	124664157	128	21	120000	-0.3602	0.64065	FAM24B	76353	25kb
ENSG00000203795	10	124645217	124697627	108	12	120000	-0.20948	0.58296	FAM24A	76353	25kb
ENSG00000119965	10	124665419	124738919	147	20	120000	-0.45742	0.67631	C10orf88	76353	25kb
ENSG00000179988	10	124688897	124782029	233	28	120000	-0.68253	0.75255	PSTK	76353	25kb
ENSG00000095574	10	124725322	124793333	211	23	120000	-0.45505	0.67546	IKZF5	76353	25kb
ENSG00000196177	10	124743495	124842827	329	30	120000	-0.719	0.76393	ACADSB	76353	25kb
ENSG00000188620	10	124870478	124922257	120	27	120000	0.53496	0.29634	HMX3	76353	25kb
ENSG00000188816	10	124882638	124935188	104	20	120000	0.13368	0.44683	HMX2	76353	25kb
ENSG00000154473	10	124888793	124949886	121	17	120000	0.21841	0.41356	BUB3	76353	25kb
ENSG00000154478	10	125400871	125479123	281	36	120000	-0.5068	0.69385	GPR26	76353	25kb
ENSG00000121898	10	125440723	125724783	1229	130	120000	-0.59821	0.72515	CPXM2	76353	25kb
ENSG00000182022	10	125742184	125878206	358	45	120000	0.056507	0.47747	CHST15	76353	25kb
ENSG00000065154	10	126060872	126132545	230	29	120000	1.4647	0.071508	OAT	76353	25kb
ENSG00000229544	10	126110592	126163753	169	31	120000	1.0009	0.15845	NKX1-2	76353	25kb
ENSG00000107902	10	126125403	126331457	984	76	120000	-0.2582	0.60187	LHPP	76353	25kb
ENSG00000258539	10	126280649	126505296	578	46	120000	0.51315	0.30392	RP11-12J10.3	76353	25kb
ENSG00000189319	10	126282861	126457838	486	48	120000	0.48375	0.31428	FAM53B	76353	25kb
ENSG00000203791	10	126411718	126505439	182	22	120000	0.95565	0.16962	METTL10	76353	25kb
ENSG00000165660	10	126465354	126550239	149	21	120000	0.70592	0.24012	FAM175B	76353	25kb
ENSG00000019995	10	126605692	126701758	226	22	120000	-0.48196	0.68508	ZRANB1	76353	25kb
ENSG00000175029	10	126651421	126874739	806	80	120000	-0.92672	0.82297	CTBP2	76353	25kb
ENSG00000175018	10	127240091	127396713	367	43	120000	2.4871	0.0064386	TEX36	76353	25kb
ENSG00000107938	10	127383084	127477712	156	27	120000	0.7739	0.2195	EDRF1	76353	25kb
ENSG00000154485	10	127430022	127489390	112	13	120000	0.54543	0.29273	MMP21	76353	25kb
ENSG00000188690	10	127452146	127536817	162	12	120000	0.071441	0.47152	UROS	76353	25kb
ENSG00000107949	10	127487115	127567264	122	12	120000	0.079474	0.46833	BCCIP	76353	25kb
ENSG00000089876	10	127499906	127610005	131	14	120000	0.18305	0.42738	DHX32	76353	25kb
ENSG00000203780	10	127560108	127723161	367	18	120000	-0.09125	0.53635	FANK1	76353	25kb
ENSG00000148848	10	127675950	128102024	1492	122	120000	-1.3862	0.91715	ADAM12	76353	25kb
ENSG00000154493	10	128088566	128384079	867	102	120000	0.77994	0.21771	C10orf90	76353	25kb
ENSG00000150760	10	128568978	129275781	2098	157	120000	0.52389	0.30018	DOCK1	76353	25kb
ENSG00000188916	10	128908694	129019422	252	22	120000	0.086879	0.46538	FAM196A	76353	25kb
ENSG00000214285	10	129322613	129375935	297	24	120000	2.1081	0.017511	NPS	76353	25kb
ENSG00000186766	10	129510499	129564450	285	19	120000	0.57159	0.2838	FOXI2	76353	25kb
ENSG00000180745	10	129651105	129716211	285	46	120000	0.7236	0.23465	CLRN3	76353	25kb
ENSG00000132334	10	129680325	129909119	906	105	120000	-0.34323	0.63429	PTPRE	76353	25kb
ENSG00000148773	10	129869923	129949649	340	30	120000	0.53917	0.29489	MKI67	76353	25kb
ENSG00000170430	10	131240448	131591271	1405	71	120000	1.0229	0.15317	MGMT	76353	25kb
ENSG00000268421	10	131283884	131334525	304	14	120000	-0.3978	0.65461	AL355531.2	76353	25kb
ENSG00000108001	10	131608547	131787105	447	102	120000	0.14	0.44433	EBF3	76353	25kb
ENSG00000108010	10	131909663	132007785	386	32	120000	1.2798	0.1003	GLRX3	76353	25kb
ENSG00000176769	10	132865654	133134984	1359	140	120000	-0.94229	0.82698	TCERG1L	76353	25kb
ENSG00000189275	10	133579734	133647535	210	19	120000	-0.84251	0.80025	AL450307.1	76353	25kb
ENSG00000175470	10	133722955	133798331	169	15	120000	-0.85454	0.8036	PPP2R2D	76353	25kb
ENSG00000176171	10	133756578	133820435	207	21	120000	-1.0941	0.86304	BNIP3	76353	25kb
ENSG00000188385	10	133893175	134023313	613	49	120000	-1.1531	0.87556	JAKMIP3	76353	25kb
ENSG00000151640	10	133975404	134044280	245	24	120000	0.21661	0.41426	DPYSL4	76353	25kb
ENSG00000165752	10	133995996	134170351	717	64	120000	0.87514	0.19075	STK32C	76353	25kb
ENSG00000148814	10	134120614	134220010	378	30	120000	0.62606	0.26564	LRRC27	76353	25kb
ENSG00000171813	10	134185672	134256367	208	40	120000	0.73074	0.23247	PWWP2B	76353	25kb
ENSG00000180066	10	134232831	134287912	163	23	120000	1.1817	0.11867	C10orf91	76353	25kb
ENSG00000068383	10	134326324	134621979	919	44	120000	-0.68651	0.7538	INPP5A	76353	25kb
ENSG00000148826	10	134573297	134624556	168	34	120000	0.68191	0.24765	NKX6-2	76353	25kb
ENSG00000171811	10	134596896	134781327	783	49	120000	0.066954	0.47331	TTC40	76353	25kb
ENSG00000197177	10	134859433	134970179	467	60	120000	-0.87249	0.80853	GPR123	76353	25kb

ENSG00000171798	10	134948951	135064916	494	47	120000	0.28931	0.38617	KNDC1	76353	25kb
ENSG00000171794	10	135018778	135070062	205	35	120000	0.70222	0.24127	UTF1	76353	25kb
ENSG00000151650	10	135025908	135080433	201	35	120000	0.44872	0.32682	VENTX	76353	25kb
ENSG00000151651	10	135050907	135115372	226	28	120000	-0.11855	0.54718	ADAM8	76353	25kb
ENSG00000130640	10	135068135	135150841	210	22	120000	-0.23694	0.59365	TUBGCP2	76353	25kb
ENSG00000198546	10	135096979	135191033	291	31	120000	-0.35659	0.6393	ZNF511	76353	25kb
ENSG00000130643	10	135113927	135175475	186	23	120000	-0.85011	0.80237	CALY	76353	25kb
ENSG00000165828	10	135135650	135191187	187	22	120000	-0.082743	0.53297	PRAP1	76353	25kb
ENSG00000148803	10	135143658	135196529	196	22	120000	0.31584	0.37606	FUOM	76353	25kb
ENSG00000127884	10	135150984	135212193	243	30	120000	0.58973	0.27768	ECHS1	76353	25kb
ENSG00000148832	10	135167695	135230198	250	39	120000	1.0264	0.15234	PAOX	76353	25kb
ENSG00000268882	10	135167720	135218102	206	32	120000	0.84431	0.19925	AL360181.1	76353	25kb
ENSG00000254536	10	135179338	135258999	280	32	120000	1.2529	0.10511	RP11-108K14.8	76353	25kb
ENSG00000148824	10	135182598	135259811	274	30	120000	1.2028	0.11453	MTG1	76353	25kb
ENSG00000203772	10	135209170	135407916	694	47	120000	0.068451	0.47271	SPRN	76353	25kb
ENSG00000130649	10	135308910	135399724	337	19	120000	-0.080984	0.53227	CYP2E1	76353	25kb
ENSG00000268925	10	135316026	135367661	204	20	120000	-0.30762	0.62081	AL161645.2	76353	25kb
ENSG00000171772	10	135342404	135407876	257	11	120000	0.18178	0.42788	SYCE1	76353	25kb
ENSG00000225899	10	135412399	135465299	97	10	120000	-0.62302	0.73337	FRG2B	76353	25kb
ENSG00000177951	11	142784	232428	167	22	120000	-0.71304	0.76209	BET1L	76353	25kb
ENSG00000188076	11	168080	219573	97	19	120000	-0.76916	0.7791	SCGB1C1	76353	25kb
ENSG00000177947	11	171738	225261	130	21	120000	-0.76989	0.77932	ODF3	76353	25kb
ENSG00000177963	11	182511	240113	206	23	120000	-0.66214	0.74606	RIC8A	76353	25kb
ENSG00000142082	11	190458	261931	339	24	120000	-0.70274	0.75889	SIRT3	76353	25kb
ENSG00000185627	11	211546	277983	334	25	120000	-0.44915	0.67334	PSMD13	76353	25kb
ENSG00000174885	11	253365	310359	241	49	120000	-0.26236	0.60348	NLRP6	76353	25kb
ENSG00000142102	11	264135	321107	207	50	120000	0.049581	0.48023	ATHL1	76353	25kb
ENSG00000206013	11	273200	324526	175	45	120000	-0.28735	0.61308	IFITM5	76353	25kb
ENSG00000185201	11	282631	340272	224	52	120000	-0.77818	0.78177	IFITM2	76353	25kb
ENSG00000185885	11	288506	340272	197	45	120000	-1.2251	0.88973	IFITM1	76353	25kb
ENSG00000142089	11	294669	352537	255	47	120000	-0.97226	0.83454	IFITM3	76353	25kb
ENSG00000182272	11	344796	407116	268	37	120000	0.1523	0.43947	B4GALNT4	76353	25kb
ENSG00000184363	11	367614	429908	185	26	120000	-0.20195	0.58002	PKP3	76353	25kb
ENSG00000185187	11	380716	442455	188	26	120000	-0.16563	0.56578	SIGIRR	76353	25kb
ENSG00000185101	11	392933	467011	187	32	120000	-0.24385	0.59633	ANO9	76353	25kb
ENSG00000174915	11	423268	516393	276	21	120000	-0.19056	0.57557	PTDSS2	76353	25kb
ENSG00000023191	11	469512	532300	245	24	120000	-0.079892	0.53184	RNH1	76353	25kb
ENSG00000174775	11	507242	562287	254	24	120000	0.17042	0.43234	HRAS	76353	25kb
ENSG00000161328	11	512527	579916	337	31	120000	-0.042184	0.51682	LRRC56	76353	25kb
ENSG00000185522	11	529855	585779	268	24	120000	0.05397	0.47848	C11orf35	76353	25kb
ENSG00000099849	11	535404	589021	256	24	120000	-0.051758	0.52064	RASSF7	76353	25kb
ENSG00000070047	11	551486	637222	312	23	120000	-0.3031	0.61909	PHRF1	76353	25kb
ENSG00000185507	11	587553	640999	159	20	120000	-0.52504	0.70022	IRF7	76353	25kb
ENSG00000099834	11	591565	651078	196	27	120000	-0.79665	0.78717	CDHR5	76353	25kb
ENSG00000070031	11	601431	652143	183	27	120000	-0.86232	0.80574	SCT	76353	25kb
ENSG00000069696	11	612293	665706	206	31	120000	-1.0166	0.84533	DRD4	76353	25kb
ENSG00000177030	11	619233	731715	389	35	120000	-1.0128	0.84442	DEAF1	76353	25kb
ENSG00000177106	11	669438	752727	251	36	120000	-0.89863	0.81558	EPS8L2	76353	25kb
ENSG00000177042	11	670428	730028	200	28	120000	-0.86218	0.80571	TMEM80	76353	25kb
ENSG00000177156	11	722329	790024	162	21	120000	-0.33961	0.63293	TALDO1	76353	25kb
ENSG00000177225	11	742220	802488	139	20	120000	-0.39865	0.65492	PDDC1	76353	25kb
ENSG00000255284	11	752578	809297	135	19	120000	-0.24644	0.59733	AP006621.5	76353	25kb
ENSG00000184524	11	762104	815123	134	19	120000	-0.15811	0.56282	CEND1	76353	25kb
ENSG00000177542	11	765475	823316	146	21	120000	0.027305	0.48911	SLC25A22	76353	25kb
ENSG00000177595	11	774179	834753	164	26	120000	-0.071514	0.52851	PIDD	76353	25kb
ENSG00000177600	11	784647	837880	149	24	120000	-0.2431	0.59604	RPLP2	76353	25kb
ENSG00000177666	11	793902	850573	171	21	120000	-0.44872	0.67318	PNPLA2	76353	25kb
ENSG00000177685	11	801144	856991	170	21	120000	-0.32997	0.62929	EFCAB4A	76353	25kb
ENSG00000177697	11	807843	864831	164	20	120000	-0.057955	0.52311	CD151	76353	25kb
ENSG00000177700	11	812356	867545	165	20	120000	0.0093493	0.49627	POLR2L	76353	25kb
ENSG00000214063	11	817808	892116	209	23	120000	0.36743	0.35665	TSPAN4	76353	25kb
ENSG00000177830	11	842357	940058	220	27	120000	0.93979	0.17366	CHID1	76353	25kb
ENSG00000183020	11	899894	1037239	396	22	120000	-0.47479	0.68253	AP2A2	76353	25kb
ENSG00000184956	11	987821	1061706	181	29	120000	-0.86687	0.80699	MUC6	76353	25kb
ENSG00000198788	11	1049875	1129419	357	33	120000	-1.7366	0.95877	MUC2	76353	25kb
ENSG00000215182	11	1126580	1247364	271	25	120000	-2.0123	0.97791	MUC5AC	76353	25kb
ENSG00000117983	11	1219296	1308406	355	35	120000	-0.98843	0.83853	MUC5B	76353	25kb
ENSG00000078902	11	1270601	1355884	300	24	120000	0.38383	0.35055	TOLLIP	76353	25kb
ENSG00000267950	11	1270809	1321033	190	18	120000	0.044791	0.48214	AC136297.1	76353	25kb
ENSG00000174672	11	1386129	1508919	556	47	120000	-0.851	0.80262	BRSK2	76353	25kb
ENSG00000182208	11	1465687	1547477	296	28	120000	-0.77436	0.78064	MOB2	76353	25kb
ENSG00000184545	11	1550274	1618150	210	20	120000	0.11524	0.45413	DUSP8	76353	25kb
ENSG00000205869	11	1580572	1631513	182	18	120000	0.39628	0.34595	KRTAP5-1	76353	25kb
ENSG00000205867	11	1593409	1644524	213	24	120000	0.29202	0.38514	KRTAP5-2	76353	25kb
ENSG00000196224	11	1603795	1654693	250	29	120000	0.06273	0.47499	KRTAP5-3	76353	25kb
ENSG00000241598	11	1617188	1668368	313	26	120000	-0.20326	0.58053	KRTAP5-4	76353	25kb
ENSG00000185940	11	1626033	1677160	366	28	120000	-0.33172	0.62995	KRTAP5-5	76353	25kb
ENSG00000205864	11	1693425	1743985	177	29	120000	0.52486	0.29984	KRTAP5-6	76353	25kb
ENSG00000244242	11	1728640	1796821	215	29	120000	0.52304	0.30047	IFITM10	76353	25kb
ENSG00000250644	11	1743897	1805281	188	24	120000	-0.043984	0.51754	RP11-295K3.1	76353	25kb
ENSG00000117984	11	1748982	1810222	185	23	120000	0.00062702	0.49975	CTSD	76353	25kb
ENSG00000149043	11	1823709	1883751	221	38	120000	-1.0559	0.8545	SYT8	76353	25kb
ENSG00000130598	11	1835219	1887910	206	39	120000	-1.1688	0.87875	TNNI2	76353	25kb
ENSG00000130592	11	1849200	1938497	325	54	120000	-1.8903	0.97064	LSP1	76353	25kb
ENSG00000184682	11	1885375	1937084	172	38	120000	-1.2612	0.89638	C11orf89	76353	25kb
ENSG00000130595	11	1915792	1984936	212	45	120000	-2.1904	0.98575	TNNT3	76353	25kb
ENSG00000214026	11	1943508	2030752	362	37	120000	-1.8965	0.97105	MRPL23	76353	25kb
ENSG00000167244	11	2125342	2195833	216	35	120000	1.148	0.12549	IGF2	76353	25kb
ENSG00000129965	11	2128768	2207439	256	34	120000	0.82586	0.20444	INS-IGF2	76353	25kb
ENSG00000254647	11	2156009	2207571	165	29	120000	1.0291	0.15172	INS	76353	25kb
ENSG00000180176	11	2160159	2218107	218	31	120000	0.6835	0.24714	TH	76353	25kb
ENSG00000183734	11	2264725	2317182	237	31	120000	-0.37925	0.64775	ASCL2	76353	25kb
ENSG00000110665	11	2291875	2349279	293	29	120000	0.34729	0.36419	C11orf21	76353	25kb
ENSG00000064201	11	2298227	2364430	315	27	120000	0.64528	0.25937	TSPAN32	76353	25kb
ENSG00000110651	11	2372407	2443649	221	35	120000	1.8111	0.035062	CD81	76353	25kb
ENSG00000184281	11	2396718	2450106	148	31	120000	0.79253	0.21403	TSSC4	76353	25kb

ENSG00000070985	11	2400745	2469275	191	40	120000	0.21288	0.41571	TRPM5	76353	25kb
ENSG00000053918	11	2440914	2895339	1342	177	120000	0.94185	0.17313	KCNQ1	76353	25kb
ENSG00000129757	11	2879443	2932111	164	43	120000	2.5859	0.0048558	CDKN1C	76353	25kb
ENSG00000254827	11	2884010	2949970	210	52	120000	2.557	0.0052797	SLC22A18AS	76353	25kb
ENSG00000110628	11	2895951	2971476	233	51	120000	1.8481	0.032297	SLC22A18	76353	25kb
ENSG00000181649	11	2924503	2975685	164	32	120000	1.5769	0.057404	PHLDA2	76353	25kb
ENSG00000205531	11	2940667	3038607	306	23	120000	0.65634	0.2558	NAP1L4	76353	25kb
ENSG00000183562	11	2986093	3037563	182	21	120000	0.59357	0.2764	AC131971.1	76353	25kb
ENSG00000110619	11	2997152	3103843	336	21	120000	-0.11924	0.54746	CARS	76353	25kb
ENSG00000021762	11	3083346	3212969	575	75	120000	-0.80946	0.79088	OSBPL5	76353	25kb
ENSG00000182170	11	3214174	3265043	301	40	120000	1.306	0.095769	MRGPRG	76353	25kb
ENSG00000184350	11	3223928	3278616	250	41	120000	0.80183	0.21133	MRGPRE	76353	25kb
ENSG00000005801	11	3335491	3425448	246	22	120000	-0.25423	0.60034	ZNF195	76353	25kb
ENSG00000167311	11	3634733	3688546	240	34	120000	0.52456	0.29994	ART5	76353	25kb
ENSG00000129744	11	3641358	3710646	282	35	120000	0.49071	0.31182	ART1	76353	25kb
ENSG00000129749	11	3661817	3717614	209	32	120000	0.66231	0.25389	CHRNA10	76353	25kb
ENSG00000110713	11	3667313	3844022	533	30	120000	0.9546	0.16989	NUP98	76353	25kb
ENSG00000148985	11	3793954	3872601	244	31	120000	0.36096	0.35906	PGAP2	76353	25kb
ENSG00000177105	11	3823208	3887213	168	27	120000	-0.028329	0.5113	RHOG	76353	25kb
ENSG00000167323	11	3850757	4139439	687	32	120000	0.36149	0.35887	STIM1	76353	25kb
ENSG00000167325	11	4090937	4185106	335	20	120000	0.49722	0.30952	RRM1	76353	25kb
ENSG00000221996	11	4363493	4414616	106	25	120000	-2.0403	0.97934	OR52B4	76353	25kb
ENSG00000132109	11	4381127	4439926	240	29	120000	-2.1388	0.98378	TRIM21	76353	25kb
ENSG00000181963	11	4445525	4496591	221	35	120000	-0.28474	0.61208	OR52K2	76353	25kb
ENSG00000196778	11	4485109	4536138	218	16	120000	0.062379	0.47513	OR52K1	76353	25kb
ENSG00000197790	11	4541421	4592374	319	27	120000	-0.111	0.54419	OR52M1	76353	25kb
ENSG00000171987	11	4567653	4624050	306	34	120000	-0.64793	0.74148	C11orf40	76353	25kb
ENSG00000226288	11	4583021	4634135	245	31	120000	-0.62933	0.73543	OR52I2	76353	25kb
ENSG00000232268	11	4590037	4641243	213	21	120000	-0.4753	0.68271	OR52I1	76353	25kb
ENSG00000167333	11	4594902	4654489	245	19	120000	-0.39714	0.65437	TRIM68	76353	25kb
ENSG00000197428	11	4635945	4687068	290	25	120000	-1.3155	0.90583	OR51D1	76353	25kb
ENSG00000180785	11	4639650	4701718	360	29	120000	-1.4242	0.9228	OR51E1	76353	25kb
ENSG00000167332	11	4676401	4744084	345	33	120000	-0.26162	0.60319	OR51E2	76353	25kb
ENSG00000167346	11	4701157	5038659	1441	70	120000	1.7251	0.042254	MMP26	76353	25kb
ENSG00000188069	11	4765209	4816168	302	16	120000	1.0925	0.1373	OR51F1	76353	25kb
ENSG00000176937	11	4799663	4850847	304	14	120000	1.1654	0.12193	OR52R1	76353	25kb
ENSG00000176925	11	4817551	4868686	241	20	120000	1.4478	0.073837	OR51F2	76353	25kb
ENSG00000176922	11	4844427	4895514	158	18	120000	1.8585	0.031548	OR51S1	76353	25kb
ENSG00000176904	11	4855886	4906794	132	17	120000	2.1772	0.014732	OR51H1P	76353	25kb
ENSG00000176900	11	4878049	4929113	143	22	120000	2.4888	0.0064079	OR51T1	76353	25kb
ENSG00000176895	11	4903600	4954538	205	35	120000	1.9645	0.024736	OR51A7	76353	25kb
ENSG00000176893	11	4910900	4961922	237	39	120000	1.4799	0.069446	OR51G2	76353	25kb
ENSG00000176879	11	4919531	4970637	246	36	120000	1.2481	0.10599	OR51G1	76353	25kb
ENSG00000205497	11	4942355	4993356	252	22	120000	1.0092	0.15643	OR51A4	76353	25kb
ENSG00000205496	11	4951002	5001943	220	23	120000	0.93689	0.17441	OR51A2	76353	25kb
ENSG00000176798	11	4995213	5046160	172	27	120000	0.58535	0.27915	OR51L1	76353	25kb
ENSG00000205495	11	5042756	5093691	385	13	120000	0.14902	0.44077	OR52J3	76353	25kb
ENSG00000176787	11	5054880	5105857	348	20	120000	0.15584	0.43808	OR52E2	76353	25kb
ENSG00000171944	11	5127922	5178872	128	20	120000	1.4	0.080753	OR52A5	76353	25kb
ENSG00000182070	11	5147239	5232612	364	28	120000	0.0757	0.46983	OR52A1	76353	25kb
ENSG00000176742	11	5195965	5246930	282	26	120000	-0.16611	0.56596	OR51V1	76353	25kb
ENSG00000244734	11	5221694	5275625	210	26	120000	-0.22362	0.58848	HBB	76353	25kb
ENSG00000223609	11	5228908	5281600	170	24	120000	-0.55414	0.71026	HBD	76353	25kb
ENSG00000213934	11	5244313	5296122	151	15	120000	-0.96085	0.83169	HBG1	76353	25kb
ENSG00000196565	11	5249420	5692019	2162	111	120000	0.36293	0.35833	HBG2	76353	25kb
ENSG00000213931	11	5264582	5551847	1392	66	120000	-0.16426	0.56524	HBE1	76353	25kb
ENSG00000183251	11	5297244	5348226	215	26	120000	0.75816	0.22418	OR51B4	76353	25kb
ENSG00000184881	11	5319541	5370582	277	25	120000	1.1699	0.12102	OR51B2	76353	25kb
ENSG00000242180	11	5338744	5389809	316	26	120000	0.76573	0.22192	OR51B5	76353	25kb
ENSG00000176239	11	5347738	5398676	300	21	120000	0.11827	0.45293	OR51B6	76353	25kb
ENSG00000184698	11	5385607	5436664	376	20	120000	-1.3694	0.91457	OR51M1	76353	25kb
ENSG00000184321	11	5398827	5449777	369	19	120000	-1.1532	0.87559	OR51J1	76353	25kb
ENSG00000167360	11	5418341	5469436	316	19	120000	-0.44767	0.67281	OR51Q1	76353	25kb
ENSG00000167359	11	5436800	5487744	267	22	120000	0.15248	0.43941	OR51I1	76353	25kb
ENSG00000187918	11	5449638	5500707	249	30	120000	0.35479	0.36137	OR51I2	76353	25kb
ENSG00000181609	11	5484915	5535979	175	29	120000	0.28714	0.387	OR52D1	76353	25kb
ENSG00000175520	11	5503530	5556215	205	32	120000	0.34629	0.36456	UBQLN3	76353	25kb
ENSG00000175518	11	5510623	5562935	209	26	120000	0.52587	0.29949	UBQLNL	76353	25kb
ENSG00000181616	11	5540719	5591779	299	18	120000	2.3204	0.010159	OR52H1	76353	25kb
ENSG00000187747	11	5577107	5628114	329	29	120000	0.60675	0.27201	OR52B6	76353	25kb
ENSG00000121236	11	5592339	5659188	331	51	120000	-1.0862	0.86131	TRIM6	76353	25kb
ENSG00000258588	11	5592955	5690628	450	61	120000	-1.4621	0.92815	TRIM6-TRIM34	76353	25kb
ENSG00000258659	11	5615994	5690628	294	42	120000	-1.9067	0.97172	TRIM34	76353	25kb
ENSG00000132256	11	5659425	5984849	1613	66	120000	-0.2367	0.59355	TRIM5	76353	25kb
ENSG00000132274	11	5685919	5783319	555	48	120000	-0.93543	0.82522	TRIM22	76353	25kb
ENSG00000181023	11	5732681	5783768	286	24	120000	-0.39402	0.65322	OR56B1	76353	25kb
ENSG00000181074	11	5750923	5801959	215	23	120000	-0.27157	0.60702	OR52N4	76353	25kb
ENSG00000181009	11	5773864	5824897	192	15	120000	0.12061	0.452	OR52N5	76353	25kb
ENSG00000181001	11	5784084	5835046	205	14	120000	0.34076	0.36664	OR52N1	76353	25kb
ENSG00000180988	11	5816544	5867578	362	20	120000	0.53161	0.2975	OR52N2	76353	25kb
ENSG00000205409	11	5837158	5888182	312	17	120000	0.63734	0.26195	OR52E6	76353	25kb
ENSG00000183269	11	5852904	5903964	292	11	120000	0.30713	0.37937	OR52E8	76353	25kb
ENSG00000180974	11	5880501	5931527	259	17	120000	0.23121	0.40857	OR52E4	76353	25kb
ENSG00000184478	11	5943570	5994591	185	23	120000	0.30386	0.38062	OR56A3	76353	25kb
ENSG00000183313	11	5982122	6033215	183	32	120000	-0.26595	0.60486	OR52L1	76353	25kb
ENSG00000183389	11	5998216	6049424	216	26	120000	-0.036951	0.51474	OR56A4	76353	25kb
ENSG00000180934	11	6022901	6073971	203	21	120000	0.46114	0.32235	OR56A1	76353	25kb
ENSG00000180919	11	6103914	6155065	236	8	120000	-0.17568	0.56973	OR56B4	76353	25kb
ENSG00000180913	11	6124840	6175706	112	14	120000	0.43052	0.33341	OR56B3P	76353	25kb
ENSG00000180909	11	6148006	6198818	138	26	120000	0.35278	0.36212	OR52B1P	76353	25kb
ENSG00000255307	11	6165560	6216638	126	24	120000	0.78882	0.21511	OR52B2	76353	25kb
ENSG00000175485	11	6195376	6246489	133	22	120000	0.41922	0.33753	OR52W1	76353	25kb
ENSG00000180878	11	6201796	6257362	124	21	120000	-0.015471	0.50617	C11orf42	76353	25kb
ENSG00000051009	11	6207565	6280941	141	22	120000	-0.11974	0.54765	FAM160A2	76353	25kb
ENSG00000132259	11	6230995	6290659	144	21	120000	-0.25881	0.60211	CNGA4	76353	25kb
ENSG00000110148	11	6255966	6318357	199	47	120000	-0.68154	0.75223	CCKBR	76353	25kb

ENSG00000170955	11	6315176	6366877	188	43	120000	-0.85018	0.80239	PRKCDBP	76353	25kb
ENSG00000166311	11	6386655	6441228	145	32	120000	2.4262	0.0076282	SMPD1	76353	25kb
ENSG00000166313	11	6391355	6465644	173	34	120000	1.5358	0.062299	APBB1	76353	25kb
ENSG00000110169	11	6427279	6488847	134	26	120000	-0.47911	0.68407	HPX	76353	25kb
ENSG00000110171	11	6444843	6520689	163	25	120000	-0.13583	0.55402	TRIM3	76353	25kb
ENSG00000132254	11	6471910	6527666	124	15	120000	0.38567	0.34987	ARFIP2	76353	25kb
ENSG00000132286	11	6477677	6530909	124	13	120000	0.38969	0.34838	TIMM10B	76353	25kb
ENSG00000265264	11	6477715	6555208	190	16	120000	0.3164	0.37585	TIMM10B	76353	25kb
ENSG00000179532	11	6493490	6639988	366	44	120000	-0.32386	0.62698	DNHD1	76353	25kb
ENSG00000132275	11	6591305	6649850	134	27	120000	-0.13408	0.55333	RRP8	76353	25kb
ENSG00000166333	11	6599961	6657102	132	26	120000	-0.1801	0.57146	ILK	76353	25kb
ENSG00000166337	11	6602526	6658898	131	26	120000	-0.17688	0.5702	TAF10	76353	25kb
ENSG00000166340	11	6609000	6665692	139	27	120000	-0.13077	0.55202	TPP1	76353	25kb
ENSG00000166341	11	6617556	6702085	217	42	120000	-0.071188	0.52838	DCHS1	76353	25kb
ENSG00000158042	11	6677013	6729632	180	24	120000	0.24152	0.40458	MRPL17	76353	25kb
ENSG00000188124	11	6764218	6815286	215	17	120000	-0.049369	0.51969	OR2AG2	76353	25kb
ENSG00000170803	11	6781248	6832245	190	17	120000	-0.10662	0.54246	OR2AG1	76353	25kb
ENSG00000184933	11	6790756	6842128	175	16	120000	-0.044664	0.51781	OR6A2	76353	25kb
ENSG00000166363	11	6841883	6892936	277	13	120000	-0.28209	0.61106	OR10A5	76353	25kb
ENSG00000170790	11	6865924	6916976	273	13	120000	-0.39785	0.65463	OR10A2	76353	25kb
ENSG00000170782	11	6872856	6923850	268	19	120000	-0.41484	0.66087	OR10A4	76353	25kb
ENSG00000166368	11	6887721	6938830	221	23	120000	-0.24437	0.59653	OR2D2	76353	25kb
ENSG00000178358	11	6917205	6968264	215	17	120000	0.4559	0.32423	OR2D3	76353	25kb
ENSG00000149054	11	6922635	7030863	348	29	120000	0.11909	0.4526	ZNF215	76353	25kb
ENSG00000149050	11	6995549	7066599	241	25	120000	-0.4005	0.65561	ZNF214	76353	25kb
ENSG00000158077	11	7016677	7117539	406	22	120000	-0.78252	0.78305	NLRP14	76353	25kb
ENSG00000170748	11	7085165	7137379	212	21	120000	-0.77268	0.78015	RBMXL2	76353	25kb
ENSG00000170743	11	7235009	7515273	1060	93	120000	-0.4421	0.67079	SYT9	76353	25kb
ENSG00000183801	11	7481619	7557608	304	37	120000	-0.98527	0.83775	OLFML1	76353	25kb
ENSG00000166387	11	7509529	7703358	788	74	120000	-1.4056	0.92008	PPFIBP2	76353	25kb
ENSG00000166394	11	7661331	7723453	321	32	120000	0.3093	0.37855	CYB5R2	76353	25kb
ENSG00000183303	11	7792452	7843520	211	19	120000	0.52473	0.29989	OR5P2	76353	25kb
ENSG00000182334	11	7821584	7872519	203	16	120000	0.83376	0.20221	OR5P3	76353	25kb
ENSG00000175393	11	7924180	7975209	234	27	120000	-0.45711	0.67621	OR10A6	76353	25kb
ENSG00000170683	11	7935081	7986141	200	24	120000	-0.35434	0.63846	OR10A3	76353	25kb
ENSG00000182261	11	7955971	8011973	189	30	120000	-0.57601	0.7177	NLRP10	76353	25kb
ENSG00000175390	11	7966798	8048409	260	40	120000	-0.84372	0.80059	EIF3F	76353	25kb
ENSG00000166402	11	8015791	8152659	434	65	120000	0.013834	0.49448	TUB	76353	25kb
ENSG00000166405	11	8102597	8215602	441	32	120000	-0.21707	0.58592	RIC3	76353	25kb
ENSG00000166407	11	8220851	8315263	416	67	120000	-0.25397	0.60024	LMO1	76353	25kb
ENSG00000130413	11	8388418	8640836	790	27	120000	0.29401	0.38437	STK33	76353	25kb
ENSG00000166436	11	8608584	8718413	296	23	120000	0.36307	0.35828	TRIM66	76353	25kb
ENSG00000166441	11	8678958	8761306	193	39	120000	0.7273	0.23352	RPL27A	76353	25kb
ENSG00000166444	11	8689898	8957498	784	42	120000	0.080976	0.46773	ST5	76353	25kb
ENSG00000166452	11	8907686	8966631	231	19	120000	-0.87458	0.8091	AKIP1	76353	25kb
ENSG00000176029	11	8916623	8979553	239	19	120000	-0.87979	0.81051	C11orf16	76353	25kb
ENSG00000176009	11	8934119	8989580	206	18	120000	-0.88284	0.81134	ASCL3	76353	25kb
ENSG00000175348	11	8943841	9011558	222	21	120000	-0.53239	0.70277	TMEM9B	76353	25kb
ENSG00000175352	11	8977123	9050596	208	25	120000	-0.38673	0.65052	NRIP3	76353	25kb
ENSG00000175356	11	9016071	9184661	458	53	120000	-0.33971	0.63296	SCUBE2	76353	25kb
ENSG00000184014	11	9135372	9311937	404	22	120000	0.17813	0.42931	DENND5A	76353	25kb
ENSG00000166471	11	9277201	9361327	255	18	120000	0.14732	0.44144	TMEM41B	76353	25kb
ENSG00000205339	11	9381169	9494673	247	25	120000	1.9582	0.025102	IPO7	76353	25kb
ENSG00000268403	11	9456747	9507010	103	13	120000	1.5203	0.064212	AC132192.1	76353	25kb
ENSG00000166478	11	9456866	9575071	227	24	120000	1.0565	0.14537	ZNF143	76353	25kb
ENSG00000166483	11	9570228	9640004	164	29	120000	-1.2768	0.89916	WEE1	76353	25kb
ENSG00000133789	11	9660624	9799538	414	21	120000	-0.48061	0.6846	SWAP70	76353	25kb
ENSG00000133812	11	9775214	10340754	1804	41	120000	0.88257	0.18873	SBF2	76353	25kb
ENSG00000148926	11	10301227	10353944	118	13	120000	0.588	0.27827	ADM	76353	25kb
ENSG00000133805	11	10304860	10554126	892	83	120000	0.2038	0.41925	AMPD3	76353	25kb
ENSG00000255823	11	10504434	10555723	193	34	120000	1.2271	0.1099	MTRNR2L8	76353	25kb
ENSG00000110315	11	10508225	10587777	277	30	120000	1.8389	0.032967	RNF141	76353	25kb
ENSG00000133800	11	10553513	10658236	332	31	120000	1.9375	0.02634	LYVE1	76353	25kb
ENSG00000072952	11	10569638	10740535	538	48	120000	1.715	0.04317	MRVI1	76353	25kb
ENSG00000198730	11	10747534	10826290	208	42	120000	0.041172	0.48358	CTR9	76353	25kb
ENSG00000110321	11	10793597	10855657	147	20	120000	0.50872	0.30548	EIF4G2	76353	25kb
ENSG00000236287	11	10808621	10905343	265	25	120000	-0.22932	0.59069	ZBED5	76353	25kb
ENSG00000110328	11	11267423	11668552	1806	181	120000	0.4746	0.31754	GALNT18	76353	25kb
ENSG00000254598	11	11348489	11399904	270	50	120000	0.84573	0.19885	CSNK2A3	76353	25kb
ENSG00000268242	11	11722060	11772257	181	27	120000	0.34883	0.36361	AC131935.1	76353	25kb
ENSG00000170242	11	11837970	12005870	402	30	120000	1.22	0.11123	USP47	76353	25kb
ENSG00000050165	11	11959653	12056316	323	54	120000	1.2106	0.11302	DKK3	76353	25kb
ENSG00000133816	11	12090543	12310334	930	111	120000	1.5354	0.062337	MICAL2	76353	25kb
ENSG00000133808	11	12272627	12405691	542	44	120000	0.41518	0.339	MICALCL	76353	25kb
ENSG00000197702	11	12373732	12577348	669	95	120000	1.7708	0.038295	PARVA	76353	25kb
ENSG00000187079	11	12670969	12991298	607	42	120000	-0.15202	0.56042	TEAD1	76353	25kb
ENSG00000133794	11	13273199	13433813	409	43	120000	-0.59259	0.72327	ARNTL	76353	25kb
ENSG00000148925	11	13384548	13509844	310	17	120000	-0.96033	0.83156	BTBD10	76353	25kb
ENSG00000152266	11	13488602	13542728	150	13	120000	-1.1559	0.87614	PTH	76353	25kb
ENSG00000197601	11	13665217	13778893	278	29	120000	-0.74886	0.77303	FAR1	76353	25kb
ENSG00000133818	11	14274472	14411052	167	28	120000	-0.47577	0.68288	RRAS2	76353	25kb
ENSG00000129083	11	14439986	14546573	190	16	120000	-1.904	0.97154	COPB1	76353	25kb
ENSG00000256206	11	14490329	14566890	106	13	120000	-1.8783	0.96983	PSMA1	76353	25kb
ENSG00000129084	11	14490329	14690181	262	25	120000	-2.3773	0.99128	PSMA1	76353	25kb
ENSG00000152270	11	14640269	14917350	322	25	120000	-2.117	0.98287	PDE3B	76353	25kb
ENSG00000186104	11	14874553	14938798	77	14	120000	-1.2987	0.90297	CYP2R1	76353	25kb
ENSG00000175868	11	14901543	15128888	405	18	120000	-0.55926	0.71201	CALCB	76353	25kb
ENSG00000110680	11	14963214	15018900	119	10	120000	-0.90704	0.81781	CALCA	76353	25kb
ENSG00000188487	11	15108970	15293754	567	65	120000	-0.64159	0.73943	INSC	76353	25kb
ENSG00000110693	11	15962995	16786138	1561	65	120000	0.15119	0.43991	SOX6	76353	25kb
ENSG00000110696	11	16609679	16803428	302	23	120000	0.64538	0.25934	C11orf58	76353	25kb
ENSG00000166689	11	16774842	17060990	879	61	120000	0.16225	0.43555	PLEKHA7	76353	25kb
ENSG00000110700	11	17070936	17124334	107	16	120000	-0.16675	0.56622	RPS13	76353	25kb
ENSG00000011405	11	17074277	17254530	358	24	120000	-0.17763	0.57049	PIK3C2A	76353	25kb
ENSG00000070081	11	17204700	17396521	406	24	120000	-0.42484	0.66452	NUCB2	76353	25kb
ENSG00000188211	11	17348273	17423888	211	22	120000	-1.122	0.86907	NCR3LG1	76353	25kb

ENSG00000187486	11	17382406	17435878	172	24	120000	-1.0863	0.86132	KCNJ11	76353	25kb
ENSG00000006071	11	17389432	17523449	520	50	120000	0.75026	0.22655	ABCC8	76353	25kb
ENSG00000006611	11	17490442	17590963	356	53	120000	1.2871	0.099027	USH1C	76353	25kb
ENSG00000188162	11	17543920	17693697	388	42	120000	0.68549	0.24652	OTOG	76353	25kb
ENSG00000129152	11	17716115	17768678	167	26	120000	-0.25187	0.59943	MYOD1	76353	25kb
ENSG00000129159	11	17731359	17829602	249	49	120000	0.055337	0.47794	KCNC1	76353	25kb
ENSG00000129158	11	17784595	18059709	509	44	120000	-0.51107	0.69535	SERGEF	76353	25kb
ENSG00000129167	11	18014111	18088973	122	11	120000	1.1606	0.12291	TPH1	76353	25kb
ENSG00000166788	11	18066482	18152638	200	14	120000	1.7483	0.040202	SAAL1	76353	25kb
ENSG00000179826	11	18117502	18185027	214	24	120000	1.7599	0.039212	MRGPRX3	76353	25kb
ENSG00000255470	11	18136733	18236042	368	23	120000	1.2192	0.11139	RP11-113D6.6	76353	25kb
ENSG00000179817	11	18169384	18220827	212	13	120000	1.436	0.075495	MRGPRX4	76353	25kb
ENSG00000189332	11	18205685	18261109	201	20	120000	0.36424	0.35784	RP11-113D6.10	76353	25kb
ENSG00000148965	11	18227896	18283440	183	26	120000	-1.0735	0.85847	SAA4	76353	25kb
ENSG00000134339	11	18235770	18295190	196	33	120000	-0.87185	0.80836	SAA2	76353	25kb
ENSG00000173432	11	18262721	18316524	170	29	120000	0.071397	0.47154	SAA1	76353	25kb
ENSG00000110756	11	18275223	18368745	240	26	120000	1.726	0.042173	HPS5	76353	25kb
ENSG00000110768	11	18318842	18413591	220	25	120000	2.0688	0.019284	GTF2H1	76353	25kb
ENSG00000134333	11	18390935	18454972	234	21	120000	-0.66364	0.74654	LDHA	76353	25kb
ENSG00000166796	11	18408854	18498605	329	17	120000	-1.0798	0.85987	LDHC	76353	25kb
ENSG00000166800	11	18452371	18526147	220	26	120000	-1.4119	0.92101	LDHAL6A	76353	25kb
ENSG00000074319	11	18464883	18573779	299	27	120000	-0.77336	0.78035	TSG101	76353	25kb
ENSG00000151116	11	18526156	18635294	301	24	120000	-0.45539	0.67559	UEVLD	76353	25kb
ENSG00000179119	11	18602948	18681338	257	27	120000	-0.71816	0.76367	SPTY2D1	76353	25kb
ENSG00000151117	11	18689669	18751332	206	48	120000	-1.0006	0.84149	TMEM86A	76353	25kb
ENSG00000179057	11	18700852	18772777	218	52	120000	-1.056	0.85452	IGSF22	76353	25kb
ENSG00000110786	11	18724475	18839268	375	70	120000	-2.0919	0.98177	PTPN5	76353	25kb
ENSG00000170255	11	18930360	18986054	144	20	120000	-1.3621	0.91342	MRGPRX1	76353	25kb
ENSG00000183695	11	19051002	19107228	243	20	120000	-1.2037	0.88565	MRGPRX2	76353	25kb
ENSG00000177054	11	19113646	19222969	314	39	120000	-0.038079	0.51519	ZDHHC13	76353	25kb
ENSG00000129170	11	19178578	19257120	217	45	120000	1.0871	0.13849	CSRP3	76353	25kb
ENSG00000129173	11	19220610	19288167	175	27	120000	-0.091352	0.53639	E2F8	76353	25kb
ENSG00000166833	11	19347271	20168144	2535	267	120000	-0.79092	0.78551	NAV2	76353	25kb
ENSG00000109851	11	20152701	20207159	198	48	120000	1.3176	0.093814	DBX1	76353	25kb
ENSG00000109854	11	20360231	20430329	288	28	120000	-0.051964	0.52072	HTATIP2	76353	25kb
ENSG00000185238	11	20384076	20555840	540	35	120000	-0.57725	0.71812	PRMT3	76353	25kb
ENSG00000165970	11	20595946	20705831	447	49	120000	-0.44426	0.67157	SLC6A5	76353	25kb
ENSG00000165973	11	20666117	21622227	3214	275	120000	0.013508	0.49461	NELL1	76353	25kb
ENSG00000171714	11	22189722	22329903	229	21	120000	-1.2455	0.89353	ANO5	76353	25kb
ENSG00000091664	11	22334643	22426049	283	39	120000	0.62294	0.26666	SLC17A6	76353	25kb
ENSG00000183161	11	22619079	22672387	80	14	120000	-0.6264	0.73447	FANCF	76353	25kb
ENSG00000229387	11	22621860	22672369	78	14	120000	-0.59013	0.72245	AC103801.2	76353	25kb
ENSG00000148935	11	22622188	22859601	570	48	120000	-1.9399	0.97381	GAS2	76353	25kb
ENSG00000198168	11	22810345	22876845	154	17	120000	-1.4408	0.92519	SVIP	76353	25kb
ENSG00000255359	11	22843477	22906972	198	22	120000	-0.5769	0.718	CCDC179	76353	25kb
ENSG00000187398	11	24493516	25129150	2811	114	120000	0.26794	0.39437	LUZP2	76353	25kb
ENSG00000134343	11	26185829	26709835	2700	81	120000	-0.518	0.69777	ANO3	76353	25kb
ENSG00000169550	11	26555580	26618780	351	33	120000	-1.1184	0.86829	MUC15	76353	25kb
ENSG00000148942	11	26663566	26769974	399	49	120000	-0.17829	0.57075	SLC5A12	76353	25kb
ENSG00000176971	11	26990628	27043630	182	27	120000	0.28848	0.38649	FIBIN	76353	25kb
ENSG00000129151	11	27037272	27174356	424	30	120000	-0.50005	0.69148	BBOX1	76353	25kb
ENSG00000109881	11	27327374	27410415	223	31	120000	0.44606	0.32778	CCDC34	76353	25kb
ENSG00000205213	11	27362508	27519322	298	33	120000	0.59782	0.27498	LGR4	76353	25kb
ENSG00000148943	11	27491123	27553320	92	13	120000	-0.49042	0.68808	LIN7C	76353	25kb
ENSG00000176697	11	27651440	27768605	175	25	120000	0.17569	0.43027	BDNF	76353	25kb
ENSG00000121621	11	28017167	28154855	193	20	120000	0.20707	0.41798	KIF18A	76353	25kb
ENSG00000169519	11	28104795	28380054	353	20	120000	0.48936	0.31229	METTL15	76353	25kb
ENSG00000182255	11	30006288	30063570	159	22	120000	-0.48945	0.68774	KCNA4	76353	25kb
ENSG00000131808	11	30227563	30281808	118	9	120000	-0.62225	0.73311	FSHB	76353	25kb
ENSG00000152219	11	30319598	30384774	159	16	120000	0.58098	0.28063	ARL14EP	76353	25kb
ENSG00000066382	11	30381040	30633419	602	48	120000	-0.5028	0.69245	MPPED2	76353	25kb
ENSG00000170959	11	30826916	31416357	822	28	120000	-0.44777	0.67284	DCDC1	76353	25kb
ENSG00000170946	11	31366387	31478396	147	16	120000	-0.080931	0.53225	DNAJC24	76353	25kb
ENSG00000148950	11	31428948	31556192	146	12	120000	-0.046181	0.51842	IMMP1L	76353	25kb
ENSG00000109911	11	31506297	31830546	424	21	120000	-0.044803	0.51787	ELP4	76353	25kb
ENSG00000007372	11	31781340	31864509	109	18	120000	1.2556	0.10463	PAX6	76353	25kb
ENSG00000049449	11	31808939	32152301	813	73	120000	1.0247	0.15275	RCN1	76353	25kb
ENSG00000184937	11	32384321	32482176	404	24	120000	-0.49265	0.68887	WT1	76353	25kb
ENSG00000149100	11	32580344	32652808	225	28	120000	-0.69246	0.75567	EIF3M	76353	25kb
ENSG00000186714	11	32598792	32841204	684	26	120000	-0.65457	0.74363	CCDC73	76353	25kb
ENSG00000135378	11	32826489	32904669	306	20	120000	-0.77833	0.78181	PRRG4	76353	25kb
ENSG00000060749	11	32889724	33039862	366	19	120000	0.13005	0.44826	QSER1	76353	25kb
ENSG00000121690	11	33012410	33080128	294	17	120000	-0.74854	0.77293	DEPDC7	76353	25kb
ENSG00000176148	11	33035963	33152489	439	23	120000	-0.31577	0.62391	TCP11L1	76353	25kb
ENSG00000176102	11	33073734	33208917	401	23	120000	0.26587	0.39517	CSTF3	76353	25kb
ENSG00000110422	11	33253218	33403569	308	33	120000	1.7987	0.036029	HIPK3	76353	25kb
ENSG00000110427	11	33538618	33720648	454	42	120000	-1.971	0.97564	KIAA1549L	76353	25kb
ENSG00000205177	11	33694807	33747347	210	38	120000	-1.6148	0.94683	C11orf91	76353	25kb
ENSG00000085063	11	33694807	33782991	312	38	120000	-1.7245	0.95769	CD59	76353	25kb
ENSG00000110429	11	33737485	33821089	258	22	120000	-0.7075	0.76037	FBXO3	76353	25kb
ENSG00000135363	11	33855122	33938836	218	46	120000	1.2442	0.10672	LMO2	76353	25kb
ENSG00000184566	11	33877189	33928338	142	29	120000	1.9211	0.027358	AC132216.1	76353	25kb
ENSG00000135387	11	34048230	34147703	275	14	120000	-0.71639	0.76313	CAPRIN1	76353	25kb
ENSG00000135372	11	34102149	34194217	182	10	120000	-0.39569	0.65383	NAT10	76353	25kb
ENSG00000166016	11	34147535	34404555	797	111	120000	1.2737	0.10138	ABTB2	76353	25kb
ENSG00000121691	11	34435472	34518609	351	22	120000	-1.3494	0.9114	CAT	76353	25kb
ENSG00000135374	11	34475340	34560352	249	30	120000	-1.3297	0.90819	ELF5	76353	25kb
ENSG00000135373	11	34617640	34707604	243	42	120000	-1.6139	0.94672	EHF	76353	25kb
ENSG00000149089	11	34849641	34963046	591	19	120000	0.33303	0.36956	APIP	76353	25kb
ENSG00000110435	11	34912376	35067138	637	22	120000	-0.12586	0.55008	PDHX	76353	25kb
ENSG00000269766	11	35125091	35175215	181	28	120000	-1.3387	0.90966	AL356215.1	76353	25kb
ENSG00000026508	11	35135417	35278949	496	65	120000	-2.4257	0.99236	CD44	76353	25kb
ENSG00000110436	11	35247753	35466610	677	46	120000	-1.7238	0.95763	SLC1A2	76353	25kb
ENSG00000149090	11	35428370	35576848	563	65	120000	-1.104	0.86519	PAMR1	76353	25kb
ENSG00000179431	11	35614735	35667419	106	19	120000	-2.0217	0.9784	FJX1	76353	25kb
ENSG00000166326	11	35659353	35854775	225	33	120000	-0.38651	0.65044	TRIM44	76353	25kb

ENSG00000179241	11	35940531	36278686	1167	114	120000	-0.31492	0.62359	LDLRAD3	76353	25kb
ENSG00000110442	11	36270051	36335999	233	32	120000	0.14631	0.44184	COMMD9	76353	25kb
ENSG00000135362	11	36292838	36511754	795	78	120000	0.050623	0.47981	PRR5L	76353	25kb
ENSG00000175104	11	36483577	36556822	164	23	120000	-0.27349	0.60776	TRAF6	76353	25kb
ENSG00000166349	11	36507259	36639706	293	24	120000	-0.54458	0.70698	RAG1	76353	25kb
ENSG00000175097	11	36572124	36644829	167	10	120000	-0.71265	0.76197	RAG2	76353	25kb
ENSG00000166352	11	36591051	36719823	416	18	120000	-0.62718	0.73473	C11orf74	76353	25kb
ENSG00000148948	11	40110753	41506323	3751	198	120000	0.0059659	0.49762	LRRC4C	76353	25kb
ENSG00000166181	11	43308513	43391079	152	26	120000	-0.39262	0.6527	API5	76353	25kb
ENSG00000052841	11	43355482	43541483	422	38	120000	-1.004	0.8423	TTC17	76353	25kb
ENSG00000149084	11	43552986	43903167	784	42	120000	-0.4593	0.67699	HSD17B12	76353	25kb
ENSG00000166199	11	43877361	43966816	345	36	120000	1.1723	0.12054	ALKBH3	76353	25kb
ENSG00000187479	11	43921892	43990888	273	45	120000	0.979	0.16379	C11orf96	76353	25kb
ENSG00000205126	11	44044531	44106527	230	37	120000	1.218	0.11161	ACCSL	76353	25kb
ENSG00000110455	11	44062475	44130772	203	36	120000	-0.54115	0.7058	ACCS	76353	25kb
ENSG00000151348	11	44092099	44291979	426	34	120000	-0.71575	0.76293	EXT2	76353	25kb
ENSG00000052850	11	44256994	44356716	299	48	120000	-1.7229	0.95755	ALX4	76353	25kb
ENSG00000085117	11	44560977	44666913	409	65	120000	-1.2569	0.89561	CD82	76353	25kb
ENSG00000157570	11	44723015	44978972	871	101	120000	0.27658	0.39105	TSPAN18	76353	25kb
ENSG00000175274	11	44882454	44997840	361	62	120000	0.5792	0.28123	TP53I11	76353	25kb
ENSG00000019485	11	45090564	45272734	400	40	120000	-1.2013	0.88518	PRDM11	76353	25kb
ENSG00000019505	11	45236852	45332870	252	21	120000	0.20228	0.41985	SYT13	76353	25kb
ENSG00000175264	11	45645427	45712172	210	20	120000	-0.25958	0.60241	CHST1	76353	25kb
ENSG00000205106	11	45767983	45818909	148	21	120000	-1.382	0.91652	CTD-2210P24.4	76353	25kb
ENSG00000181830	11	45800623	45859566	172	28	120000	-1.5909	0.94419	SLC35C1	76353	25kb
ENSG00000121671	11	45843669	45929798	178	25	120000	-1.1505	0.87504	CRY2	76353	25kb
ENSG00000121653	11	45882202	45953016	127	30	120000	0.32255	0.37352	MAPK8IP1	76353	25kb
ENSG00000234776	11	45903085	45953833	99	24	120000	0.7355	0.23102	C11orf94	76353	25kb
ENSG00000121680	11	45906220	45965363	107	24	120000	1.1032	0.13498	PEX16	76353	25kb
ENSG00000165905	11	45918172	45975647	90	18	120000	1.4214	0.077602	GYLTL1B	76353	25kb
ENSG00000135365	11	45925871	46167985	417	28	120000	1.5612	0.059237	PHF21A	76353	25kb
ENSG00000157613	11	46274212	46367972	137	25	120000	1.3829	0.083345	CREB3L1	76353	25kb
ENSG00000149091	11	46329455	46427104	134	24	120000	2.0495	0.020204	DGKZ	76353	25kb
ENSG00000110492	11	46377306	46430375	53	14	120000	2.1824	0.014541	MDK	76353	25kb
ENSG00000180720	11	46381640	46433107	49	14	120000	2.2015	0.013851	CHRM4	76353	25kb
ENSG00000110497	11	46392964	46640675	223	23	120000	2.1108	0.017395	AMBRA1	76353	25kb
ENSG00000180423	11	46599411	46664459	55	8	120000	2.1703	0.014992	HARBI1	76353	25kb
ENSG00000175224	11	46613826	46721368	97	14	120000	2.2135	0.013432	ATG13	76353	25kb
ENSG00000175220	11	46673630	46747165	98	14	120000	2.0296	0.021198	ARHGAP1	76353	25kb
ENSG00000175213	11	46697368	46752462	90	13	120000	2.1366	0.016316	ZNF408	76353	25kb
ENSG00000180210	11	46715730	46786056	113	14	120000	2.0081	0.022318	F2	76353	25kb
ENSG00000175216	11	46739598	46892847	223	20	120000	2.2321	0.012806	CKAP5	76353	25kb
ENSG00000134569	11	46853419	46965193	163	19	120000	2.3231	0.010086	LRP4	76353	25kb
ENSG00000149179	11	46933240	47210936	377	22	120000	2.3328	0.0098285	C11orf49	76353	25kb
ENSG00000149182	11	47160848	47223676	110	16	120000	2.3026	0.010649	ARFGAP2	76353	25kb
ENSG00000165912	11	47174076	47232994	108	16	120000	2.2729	0.011517	PACSIN3	76353	25kb
ENSG00000134574	11	47211493	47285767	163	18	120000	1.0819	0.13965	DDB2	76353	25kb
ENSG00000134575	11	47235853	47295457	144	14	120000	0.48904	0.31241	ACP2	76353	25kb
ENSG00000025434	11	47244851	47315396	174	16	120000	-0.003352	0.50134	NR1H3	76353	25kb
ENSG00000110514	11	47265712	47376582	254	19	120000	0.0054336	0.49783	MADD	76353	25kb
ENSG00000134571	11	47327957	47399253	175	25	120000	0.19729	0.4218	MYBPC3	76353	25kb
ENSG00000066336	11	47351411	47425127	192	22	120000	0.26419	0.39582	SPI1	76353	25kb
ENSG00000165915	11	47403683	47463047	164	16	120000	0.69327	0.24407	SLC39A13	76353	25kb
ENSG00000165916	11	47415320	47472993	154	15	120000	0.87493	0.19081	PSMC3	76353	25kb
ENSG00000165917	11	47434308	47495730	119	13	120000	1.1353	0.12812	RAPSN	76353	25kb
ENSG00000149187	11	47462496	47612121	199	31	120000	1.0348	0.15038	CELF1	76353	25kb
ENSG00000213619	11	47561888	47631114	105	22	120000	0.48911	0.31238	NDUFS3	76353	25kb
ENSG00000110536	11	47561982	47620013	88	21	120000	0.5726	0.28346	PTPMT1	76353	25kb
ENSG00000123444	11	47568749	47625567	82	20	120000	0.28702	0.38705	KBTBD4	76353	25kb
ENSG00000231880	11	47574277	47624823	67	17	120000	0.11406	0.4546	KBTBD4	76353	25kb
ENSG00000196666	11	47583198	47635746	74	19	120000	0.12973	0.44839	FAM180B	76353	25kb
ENSG00000172247	11	47586216	47641211	80	19	120000	0.2191	0.41329	C1QTNF4	76353	25kb
ENSG00000109919	11	47613867	47689175	103	17	120000	0.17435	0.4308	MTCH2	76353	25kb
ENSG00000165923	11	47656143	47761941	182	20	120000	0.27032	0.39346	AGBL2	76353	25kb
ENSG00000109920	11	47713072	47813995	192	20	120000	0.21318	0.4156	FNBP4	76353	25kb
ENSG00000030066	11	47774639	47895107	255	16	120000	0.45491	0.32459	NUP160	76353	25kb
ENSG00000149177	11	47977113	48214670	477	32	120000	0.41214	0.34012	PTPRJ	76353	25kb
ENSG00000175619	11	48213344	48264314	190	18	120000	0.058539	0.47666	OR4B1	76353	25kb
ENSG00000172208	11	48241608	48292622	185	15	120000	0.036791	0.48533	OR4X2	76353	25kb
ENSG00000176567	11	48260413	48311330	179	13	120000	-0.19834	0.57861	OR4X1	76353	25kb
ENSG00000176555	11	48302775	48353704	140	13	120000	0.16695	0.4337	OR4S1	76353	25kb
ENSG00000176547	11	48321472	48372507	115	16	120000	0.53379	0.29674	OR4C3	76353	25kb
ENSG00000176540	11	48362037	48413017	88	13	120000	1.1975	0.11555	OR4C5	76353	25kb
ENSG00000237388	11	48485269	48536332	177	18	120000	0.47734	0.31656	OR4A47	76353	25kb
ENSG00000182053	11	49025504	49084579	167	16	120000	0.59436	0.27613	TRIM49B	76353	25kb
ENSG00000214891	11	49050266	49105664	173	16	120000	0.82527	0.20461	TRIM64C	76353	25kb
ENSG00000086205	11	49143187	49255222	397	20	120000	2.6889	0.0035841	FOLH1	76353	25kb
ENSG00000258817	11	49948943	49999971	182	17	120000	2.7585	0.0029036	OR4C13	76353	25kb
ENSG00000221954	11	49978009	50029071	165	13	120000	2.8419	0.0022423	OR4C12	76353	25kb
ENSG00000221840	11	51386378	51437448	320	16	120000	1.6438	0.050109	OR4A5	76353	25kb
ENSG00000185926	11	51490282	51541211	307	19	120000	2.0698	0.019234	OR4C46	76353	25kb
ENSG00000150244	11	55004658	55063595	276	16	120000	0.62339	0.26652	TRIM48	76353	25kb
ENSG00000181961	11	55085627	55136707	209	20	120000	0.8166	0.20708	OR4A16	76353	25kb
ENSG00000181958	11	55110360	55161394	235	20	120000	0.90754	0.18206	OR4A15	76353	25kb
ENSG00000181939	11	55296783	55347895	332	13	120000	0.23556	0.40689	OR4C15	76353	25kb
ENSG00000181935	11	55314604	55365536	331	13	120000	0.34063	0.36669	OR4C16	76353	25kb
ENSG00000172188	11	55345830	55396874	129	13	120000	0.65472	0.25632	OR4C11	76353	25kb
ENSG00000181927	11	55380834	55431772	28	7	120000	1.2315	0.10906	OR4P4	76353	25kb
ENSG00000174982	11	55393380	55444315	34	9	120000	0.96873	0.16634	OR4S2	76353	25kb
ENSG00000181903	11	55407614	55458671	44	10	120000	0.81935	0.20629	OR4C6	76353	25kb
ENSG00000198877	11	55515914	55566858	274	14	120000	0.42381	0.33585	OR5D13	76353	25kb
ENSG00000186113	11	55538032	55588976	158	18	120000	0.57014	0.28429	OR5D14	76353	25kb
ENSG00000186117	11	55553854	55604952	172	19	120000	0.47531	0.31728	OR5L1	76353	25kb
ENSG00000186119	11	55562086	55613073	176	20	120000	0.49697	0.30961	OR5D18	76353	25kb
ENSG00000205030	11	55569695	55620630	168	21	120000	0.45292	0.3253	OR5L2	76353	25kb
ENSG00000205029	11	55581228	55632214	154	21	120000	0.4549	0.32459	OR5D16	76353	25kb

ENSG00000124900	11	55625773	55684286	296	14	120000	-0.09535	0.53798	TRIM51	76353	25kb
ENSG00000187612	11	55656126	55707058	381	8	120000	-0.27871	0.60977	OR5W2	76353	25kb
ENSG00000167825	11	55677932	55728876	377	8	120000	-0.30804	0.62097	OR5I1	76353	25kb
ENSG00000174970	11	55709975	55760990	359	6	120000	-0.29331	0.61536	OR10AG1	76353	25kb
ENSG00000149133	11	55736157	55787101	330	7	120000	-0.29349	0.61543	OR5F1	76353	25kb
ENSG00000181785	11	55772895	55823869	390	9	120000	-0.33701	0.63194	OR5AS1	76353	25kb
ENSG00000172154	11	55835753	55886762	343	6	120000	-0.54028	0.7055	OR8I2	76353	25kb
ENSG00000181767	11	55847519	55898457	356	8	120000	-0.44404	0.67149	OR8H2	76353	25kb
ENSG00000181761	11	55864849	55915787	281	7	120000	-0.29819	0.61722	OR8H3	76353	25kb
ENSG00000167822	11	55879247	55930194	211	9	120000	-0.16777	0.56662	OR8J3	76353	25kb
ENSG00000181752	11	55901870	55952793	141	14	120000	0.032252	0.48714	OR8K5	76353	25kb
ENSG00000174957	11	55919094	55970032	147	15	120000	-0.04998	0.51993	OR5J2	76353	25kb
ENSG00000181718	11	55974490	56025737	152	13	120000	-0.23132	0.59147	OR5T2	76353	25kb
ENSG00000172489	11	55994676	56045698	151	12	120000	-0.19166	0.576	OR5T3	76353	25kb
ENSG00000181698	11	56018029	56069146	154	14	120000	-0.22079	0.58737	OR5T1	76353	25kb
ENSG00000181693	11	56032529	56083566	178	17	120000	-0.058526	0.52334	OR8H1	76353	25kb
ENSG00000181689	11	56060783	56111721	188	16	120000	-0.10769	0.54288	OR8K3	76353	25kb
ENSG00000150261	11	56088421	56139507	188	16	120000	-0.11948	0.54755	OR8K1	76353	25kb
ENSG00000172487	11	56102691	56153764	192	17	120000	-0.17555	0.56967	OR8J1	76353	25kb
ENSG00000172199	11	56118100	56169029	206	19	120000	-0.3724	0.6452	OR8U1	76353	25kb
ENSG00000174942	11	56159734	56210708	227	18	120000	-0.76932	0.77915	OR5R1	76353	25kb
ENSG00000150269	11	56204945	56255877	176	14	120000	-0.43454	0.66805	OR5M9	76353	25kb
ENSG00000174937	11	56211964	56263014	170	15	120000	-0.44887	0.67324	OR5M3	76353	25kb
ENSG00000181371	11	56232825	56283871	185	18	120000	-0.22999	0.59095	OR5M8	76353	25kb
ENSG00000255223	11	56284746	56335757	207	19	120000	-0.15799	0.56277	OR5M11	76353	25kb
ENSG00000254834	11	56319212	56370263	164	16	120000	-0.41671	0.66155	OR5M10	76353	25kb
ENSG00000255012	11	56355031	56405978	136	17	120000	-0.65551	0.74393	OR5M1	76353	25kb
ENSG00000172464	11	56383942	56434947	140	19	120000	-0.097512	0.53884	OR5AP2	76353	25kb
ENSG00000172459	11	56406138	56457163	158	21	120000	-0.10507	0.54184	OR5AR1	76353	25kb
ENSG00000174914	11	56442864	56493781	170	21	120000	0.24091	0.40481	OR9G1	76353	25kb
ENSG00000172457	11	56485303	56536287	175	23	120000	0.55867	0.28819	OR9G4	76353	25kb
ENSG00000181273	11	56731347	56782342	144	19	120000	2.2181	0.013274	OR5AK2	76353	25kb
ENSG00000183908	11	56924221	56984191	139	17	120000	0.81689	0.207	LRRC55	76353	25kb
ENSG00000134817	11	56976051	57029709	134	20	120000	-0.10659	0.54244	APLNR	76353	25kb
ENSG00000149115	11	57042112	57117426	194	20	120000	-0.31586	0.62395	TNKS1BP1	76353	25kb
ENSG00000149136	11	57068459	57128351	151	19	120000	-0.20419	0.5809	SSRP1	76353	25kb
ENSG00000109991	11	57080848	57163073	194	27	120000	-0.49555	0.68989	P2RX3	76353	25kb
ENSG00000156575	11	57119242	57173623	114	17	120000	-0.79701	0.78728	PRG3	76353	25kb
ENSG00000186652	11	57129267	57183130	113	18	120000	-0.73103	0.76762	PRG2	76353	25kb
ENSG00000254979	11	57129838	57216532	186	32	120000	-0.26698	0.60526	RP11-872D17.8	76353	25kb
ENSG00000134802	11	57149427	57220053	140	25	120000	-0.067195	0.52679	SLC43A3	76353	25kb
ENSG00000186907	11	57203022	57270007	93	18	120000	-0.0062197	0.50248	RTN4RL2	76353	25kb
ENSG00000149150	11	57227007	57308259	124	22	120000	-0.85269	0.80309	SLC43A1	76353	25kb
ENSG00000134809	11	57270936	57323276	110	16	120000	-0.86053	0.80525	TIMM10	76353	25kb
ENSG00000214872	11	57283979	57343108	128	16	120000	-0.42443	0.66437	SMTNL1	76353	25kb
ENSG00000156587	11	57294129	57360757	157	17	120000	-0.51987	0.69842	UBE2L6	76353	25kb
ENSG00000149131	11	57339860	57407326	137	18	120000	-0.87586	0.80945	SERPING1	76353	25kb
ENSG00000166793	11	57387560	57442417	79	12	120000	-1.1115	0.86682	YPEL4	76353	25kb
ENSG00000172409	11	57391465	57454340	101	15	120000	-1.1531	0.87558	CLP1	76353	25kb
ENSG00000156599	11	57410219	57493659	116	14	120000	-0.9192	0.821	ZDHHC5	76353	25kb
ENSG00000156603	11	57446186	57504693	86	12	120000	-0.75255	0.77414	MED19	76353	25kb
ENSG00000213593	11	57455072	57533445	102	14	120000	-0.59825	0.72516	TMX2	76353	25kb
ENSG00000254462	11	57455077	57584058	173	21	120000	-0.45427	0.67518	TMX2-CTNND1	76353	25kb
ENSG00000211450	11	57483825	57535986	70	12	120000	-0.67118	0.74895	C11orf31	76353	25kb
ENSG00000254732	11	57484635	57585715	137	19	120000	-0.40912	0.65877	RP11-691N7.6	76353	25kb
ENSG00000233436	11	57485986	57544253	82	13	120000	-0.61746	0.73153	BTBD18	76353	25kb
ENSG00000198561	11	57495715	57612018	158	20	120000	-0.46316	0.67838	CTNND1	76353	25kb
ENSG00000186509	11	57766353	57974088	691	30	120000	-1.711	0.95646	OR9Q1	76353	25kb
ENSG00000172381	11	57773402	57824403	210	19	120000	-2.5324	0.99434	OR6Q1	76353	25kb
ENSG00000172377	11	57860972	57911916	166	17	120000	-1.7112	0.95648	OR9I1	76353	25kb
ENSG00000186513	11	57932906	57983990	144	14	120000	-0.13812	0.55493	OR9Q2	76353	25kb
ENSG00000197887	11	57945674	57996653	164	17	120000	-0.42521	0.66466	OR1S2	76353	25kb
ENSG00000172774	11	57957217	58008195	175	18	120000	-0.71188	0.76173	OR1S1	76353	25kb
ENSG00000180475	11	57970354	58021390	200	19	120000	-1.0294	0.84835	OR10Q1	76353	25kb
ENSG00000172772	11	58009264	58060732	199	14	120000	-1.7604	0.96083	OR10W1	76353	25kb
ENSG00000197786	11	58100597	58151542	149	11	120000	-0.87153	0.80827	OR5B17	76353	25kb
ENSG00000172769	11	58144937	58195882	216	10	120000	0.66198	0.25399	OR5B3	76353	25kb
ENSG00000172365	11	58164738	58215786	204	8	120000	1.1515	0.12475	OR5B2	76353	25kb
ENSG00000172362	11	58181593	58232646	171	10	120000	1.205	0.1141	OR5B12	76353	25kb
ENSG00000198283	11	58249648	58300578	62	11	120000	0.75328	0.22564	OR5B21	76353	25kb
ENSG00000110031	11	58269344	58370693	147	19	120000	0.78246	0.21697	LPXN	76353	25kb
ENSG00000186660	11	58321584	58413515	180	18	120000	0.77954	0.21783	ZFP91	76353	25kb
ENSG00000255073	11	58321645	58417112	199	17	120000	0.80531	0.21032	ZFP91-CNTF	76353	25kb
ENSG00000269570	11	58353589	58403759	88	14	120000	0.43426	0.33205	AP001350.1	76353	25kb
ENSG00000242689	11	58365146	58418198	138	11	120000	0.76682	0.22159	CNTF	76353	25kb
ENSG00000149124	11	58382899	58524447	505	29	120000	0.063961	0.4745	GLYAT	76353	25kb
ENSG00000156689	11	58576542	58696688	494	22	120000	-0.19854	0.57869	GLYATL2	76353	25kb
ENSG00000166840	11	58647871	58836000	532	26	120000	-1.452	0.92674	GLYATL1	76353	25kb
ENSG00000224130	11	58647897	58701071	174	21	120000	-0.73744	0.76957	AP001652.1	76353	25kb
ENSG00000189057	11	58849658	58919883	112	14	120000	-1.1024	0.86487	FAM111B	76353	25kb
ENSG00000166801	11	58885221	58947512	137	8	120000	-0.35807	0.63985	FAM111A	76353	25kb
ENSG00000110042	11	58913903	59001060	199	19	120000	-0.48476	0.68608	DTX4	76353	25kb
ENSG00000197629	11	58950983	59005424	101	11	120000	-0.5685	0.71515	MPEG1	76353	25kb
ENSG00000176495	11	59106885	59157921	171	13	120000	-1.0117	0.84415	OR5AN1	76353	25kb
ENSG00000172324	11	59164416	59215449	158	16	120000	-0.18046	0.5716	OR5A2	76353	25kb
ENSG00000172320	11	59185617	59236667	206	16	120000	-0.050905	0.5203	OR5A1	76353	25kb
ENSG00000166884	11	59199411	59250464	198	17	120000	0.29499	0.384	OR4D6	76353	25kb
ENSG00000254466	11	59219846	59270869	166	22	120000	0.52506	0.29977	OR4D10	76353	25kb
ENSG00000176200	11	59246049	59296984	100	12	120000	0.42923	0.33388	OR4D11	76353	25kb
ENSG00000172742	11	59257386	59308330	86	10	120000	0.13145	0.44771	OR4D9	76353	25kb
ENSG00000110048	11	59316871	59408617	178	15	120000	-0.40098	0.65578	OSBP	76353	25kb
ENSG00000166889	11	59379189	59461453	130	12	120000	-0.79467	0.7866	PATL1	76353	25kb
ENSG00000172289	11	59455296	59506337	136	11	120000	-0.53837	0.70484	OR10V1	76353	25kb
ENSG00000166900	11	59455929	59598354	387	13	120000	-0.75458	0.77475	STX3	76353	25kb
ENSG00000166902	11	59548608	59603345	121	12	120000	-0.96961	0.83388	MRPL16	76353	25kb
ENSG00000134812	11	59571741	59637974	92	18	120000	0.0042933	0.49829	GIF	76353	25kb

ENSG00000134827	11	59595273	59659048	59	16	120000	0.88069	0.18924	TCN1	76353	25kb
ENSG00000214788	11	59680928	59737807	44	20	120000	0.61591	0.26898	OOSP1	76353	25kb
ENSG00000149507	11	59782748	59840517	141	24	120000	-1.6745	0.95298	OOSP2	76353	25kb
ENSG00000149516	11	59799060	59863601	179	25	120000	-2.0049	0.97751	MS4A3	76353	25kb
ENSG00000149534	11	59830734	59888444	161	18	120000	-1.6795	0.95347	MS4A2	76353	25kb
ENSG00000110077	11	59914081	59977139	144	14	120000	-1.239	0.89233	MS4A6A	76353	25kb
ENSG00000214787	11	59943726	60035561	260	16	120000	-1.5327	0.93733	MS4A4E	76353	25kb
ENSG00000110079	11	60023014	60101445	295	24	120000	-1.0333	0.84926	MS4A4A	76353	25kb
ENSG00000166926	11	60077304	60189069	396	23	120000	-0.28621	0.61264	MS4A6E	76353	25kb
ENSG00000166927	11	60120955	60188424	181	16	120000	0.09284	0.46302	MS4A7	76353	25kb
ENSG00000166928	11	60121003	60210161	245	22	120000	-0.13141	0.55228	MS4A14	76353	25kb
ENSG00000166930	11	60172062	60247687	214	26	120000	-0.46301	0.67832	MS4A5	76353	25kb
ENSG00000156738	11	60198225	60263233	197	19	120000	0.27492	0.39169	MS4A1	76353	25kb
ENSG00000071203	11	60235251	60299903	235	22	120000	0.37743	0.35293	MS4A12	76353	25kb
ENSG00000204979	11	60257886	60335194	258	16	120000	0.059435	0.4763	MS4A13	76353	25kb
ENSG00000166959	11	60442047	60508284	80	11	120000	0.53534	0.29621	MS4A8	76353	25kb
ENSG00000214782	11	60471789	60536685	124	24	120000	-0.47851	0.68386	MS4A18	76353	25kb
ENSG00000166961	11	60499426	60569205	185	47	120000	-0.92406	0.82227	MS4A15	76353	25kb
ENSG00000172689	11	60527821	60593778	171	32	120000	-0.5031	0.69255	MS4A10	76353	25kb
ENSG00000110104	11	60584544	60643554	223	32	120000	0.15011	0.44034	CCDC86	76353	25kb
ENSG00000183134	11	60593413	60648444	197	30	120000	0.30331	0.38083	PTGDR2	76353	25kb
ENSG00000149506	11	60610035	60668166	157	25	120000	0.43075	0.33333	ZP1	76353	25kb
ENSG00000110107	11	60633202	60699060	131	14	120000	0.71104	0.23853	PRPF19	76353	25kb
ENSG00000110108	11	60656346	60715915	139	13	120000	0.57446	0.28283	TMEM109	76353	25kb
ENSG00000006118	11	60666935	60729631	163	21	120000	0.23123	0.40857	TMEM132A	76353	25kb
ENSG00000110446	11	60679556	60745002	182	28	120000	-0.29749	0.61695	SLC15A3	76353	25kb
ENSG00000013725	11	60714115	60812849	323	44	120000	-1.0571	0.85476	CD6	76353	25kb
ENSG00000110448	11	60844867	60920324	191	19	120000	-0.79403	0.78641	CD5	76353	25kb
ENSG00000167987	11	60872728	60954089	207	16	120000	-1.1893	0.88284	VPS37C	76353	25kb
ENSG00000229859	11	60945779	61005570	50	10	120000	-1.5173	0.9354	PGA3	76353	25kb
ENSG00000229183	11	60964688	61043929	40	14	120000	-1.1863	0.88225	PGA4	76353	25kb
ENSG00000256713	11	60983514	61043920	27	12	120000	-0.69965	0.75793	PGA5	76353	25kb
ENSG00000167992	11	61000762	61087896	51	19	120000	-1.4497	0.92642	VWCE	76353	25kb
ENSG00000167986	11	61041923	61135068	70	20	120000	-1.156	0.87617	DDB1	76353	25kb
ENSG00000149476	11	61075682	61145767	60	13	120000	-0.29804	0.61716	DAK	76353	25kb
ENSG00000162144	11	61091217	61154771	58	11	120000	-0.22546	0.58919	CYB561A3	76353	25kb
ENSG00000149483	11	61104473	61161981	47	10	120000	-0.23763	0.59392	TMEM138	76353	25kb
ENSG00000187049	11	61134159	61191335	49	11	120000	-0.68272	0.75261	TMEM216	76353	25kb
ENSG00000149532	11	61145121	61222503	88	10	120000	-0.74683	0.77242	CPSF7	76353	25kb
ENSG00000256591	11	61171692	61278294	185	20	120000	-0.66252	0.74618	RP11-286N22.8	76353	25kb
ENSG00000167985	11	61172514	61240001	86	9	120000	-0.58125	0.71947	SDHAF2	76353	25kb
ENSG00000162148	11	61223592	61283403	130	16	120000	-0.40199	0.65615	PPP1R32	76353	25kb
ENSG00000204950	11	61251272	61303482	138	19	120000	-0.50706	0.69394	LRRC10B	76353	25kb
ENSG00000011347	11	61257785	61373620	213	30	120000	-0.83746	0.79883	SYT7	76353	25kb
ENSG00000134780	11	61422905	61539473	240	32	120000	-1.3965	0.91871	DAGLA	76353	25kb
ENSG00000124920	11	61495114	61580990	148	26	120000	-1.1745	0.8799	MYRF	76353	25kb
ENSG00000134825	11	61510973	61585274	126	23	120000	-0.85014	0.80238	TMEM258	76353	25kb
ENSG00000168496	11	61535109	61589716	68	10	120000	-0.27396	0.60794	FEN1	76353	25kb
ENSG00000134824	11	61535452	61659826	266	22	120000	-0.27854	0.6097	FADS2	76353	25kb
ENSG00000149485	11	61542099	61621790	146	15	120000	-0.2879	0.61329	FADS1	76353	25kb
ENSG00000221968	11	61615991	61684523	178	27	120000	0.10461	0.45834	FADS3	76353	25kb
ENSG00000167994	11	61639773	61712741	168	32	120000	0.068171	0.47282	RAB3IL1	76353	25kb
ENSG00000167995	11	61692293	61757987	200	35	120000	0.1014	0.45962	BEST1	76353	25kb
ENSG00000167996	11	61702190	61760132	184	31	120000	0.51902	0.30187	FTH1	76353	25kb
ENSG00000269089	11	61710453	61761755	170	27	120000	0.68001	0.24825	AP003733.1	76353	25kb
ENSG00000149503	11	61866445	61945635	301	16	120000	0.77785	0.21833	INCENP	76353	25kb
ENSG00000168515	11	61932688	61986011	197	19	120000	0.12432	0.45053	SCGB1D1	76353	25kb
ENSG00000124939	11	61951140	62006408	190	26	120000	0.10775	0.4571	SCGB2A1	76353	25kb
ENSG00000124935	11	61984682	62037280	178	26	120000	0.013565	0.49459	SCGB1D2	76353	25kb
ENSG00000110484	11	62012627	62065628	161	22	120000	-0.4177	0.66192	SCGB2A2	76353	25kb
ENSG00000197745	11	62038754	62091536	160	17	120000	-0.97946	0.83632	SCGB1D4	76353	25kb
ENSG00000162174	11	62079920	62185882	315	19	120000	-1.3835	0.91674	ASRGL1	76353	25kb
ENSG00000149021	11	62147575	62215667	254	28	120000	-1.0631	0.85614	SCGB1A1	76353	25kb
ENSG00000124942	11	62176016	62348707	384	59	120000	-0.71152	0.76162	AHNAK	76353	25kb
ENSG00000254772	11	62302073	62367401	86	12	120000	0.54879	0.29158	EEF1G	76353	25kb
ENSG00000255508	11	62302075	62384003	111	12	120000	0.49357	0.3108	MIR3654	76353	25kb
ENSG00000149016	11	62317517	62384649	89	10	120000	0.6367	0.26216	TUT1	76353	25kb
ENSG00000149480	11	62335686	62394312	74	10	120000	0.33739	0.36791	MTA2	76353	25kb
ENSG00000149499	11	62344690	62405237	75	11	120000	0.088452	0.46476	EML3	76353	25kb
ENSG00000149489	11	62354194	62407592	61	9	120000	0.12938	0.44853	ROM1	76353	25kb
ENSG00000149541	11	62357768	62414647	63	9	120000	0.19124	0.42417	B3GAT3	76353	25kb
ENSG00000089597	11	62367298	62439104	87	11	120000	-0.39785	0.65463	GANAB	76353	25kb
ENSG00000185085	11	62389320	62445774	69	10	120000	-0.77317	0.78029	INTS5	76353	25kb
ENSG00000255432	11	62392166	62457650	82	13	120000	-0.49673	0.69031	RP11-831H9.11	76353	25kb
ENSG00000162194	11	62405287	62464727	82	13	120000	-0.21055	0.58338	C11orf48	76353	25kb
ENSG00000214756	11	62407781	62460968	71	11	120000	-0.17973	0.57132	METTL12	76353	25kb
ENSG00000204922	11	62412745	62466159	84	12	120000	-0.18853	0.57477	C11orf83	76353	25kb
ENSG00000162191	11	62418970	62471567	88	12	120000	-0.10979	0.54371	UBXN1	76353	25kb
ENSG00000177363	11	62428874	62482371	85	12	120000	0.04136	0.4835	LRRN4CL	76353	25kb
ENSG00000168000	11	62432747	62502317	110	13	120000	0.18211	0.42775	BSCL2	76353	25kb
ENSG00000234857	11	62432747	62519856	131	14	120000	0.17225	0.43162	HNRNPUL2-BSCL2	76353	25kb
ENSG00000162188	11	62450130	62501673	81	9	120000	0.35169	0.36254	GNG3	76353	25kb
ENSG00000214753	11	62455102	62519821	95	11	120000	0.29358	0.38454	HNRNPUL2	76353	25kb
ENSG00000162222	11	62470541	62532765	83	12	120000	0.22125	0.41245	TTC9C	76353	25kb
ENSG00000185670	11	62490791	62546660	80	13	120000	0.19575	0.4224	ZBTB3	76353	25kb
ENSG00000168002	11	62504016	62559182	73	14	120000	0.0037145	0.49852	POLR2G	76353	25kb
ENSG00000162227	11	62513775	62579814	85	14	120000	-0.047544	0.51896	TAF6L	76353	25kb
ENSG00000168569	11	62514101	62584493	92	15	120000	-0.079994	0.53188	TMEM223	76353	25kb
ENSG00000185475	11	62529887	62582877	70	11	120000	-0.072882	0.52905	TMEM179B	76353	25kb
ENSG00000162231	11	62534595	62598774	92	11	120000	-0.080152	0.53194	NXF1	76353	25kb
ENSG00000162236	11	62549369	62624560	132	9	120000	0.09498	0.46217	STX5	76353	25kb
ENSG00000133316	11	62574814	62634281	113	7	120000	0.12254	0.45124	WDR74	76353	25kb
ENSG00000168003	11	62598518	62681352	154	19	120000	0.52703	0.29909	SLC3A2	76353	25kb
ENSG00000168539	11	62651151	62714279	120	26	120000	0.23775	0.40604	CHRM1	76353	25kb
ENSG00000197901	11	62678857	62777455	182	24	120000	-0.6917	0.75544	SLC22A6	76353	25kb
ENSG00000149452	11	62731626	62808311	155	34	120000	-0.52942	0.70174	SLC22A8	76353	25kb

ENSG00000197658	11	62822412	62936693	438	21	120000	0.65294	0.2569	SLC22A24	76353	25kb
ENSG00000184999	11	62880339	63162190	807	25	120000	1.1414	0.12685	SLC22A10	76353	25kb
ENSG00000196600	11	62906248	63022124	357	19	120000	1.4459	0.0741	SLC22A25	76353	25kb
ENSG00000149742	11	63112261	63202766	259	26	120000	1.8498	0.032174	SLC22A9	76353	25kb
ENSG00000168004	11	63203876	63283666	173	27	120000	1.9554	0.025269	HRASLS5	76353	25kb
ENSG00000133317	11	63248556	63309246	86	22	120000	0.36628	0.35708	LGALS12	76353	25kb
ENSG00000133321	11	63279281	63338934	86	16	120000	-0.58453	0.72057	RARRES3	76353	25kb
ENSG00000133328	11	63295242	63355855	84	18	120000	-0.50727	0.69402	HRASLS2	76353	25kb
ENSG00000176485	11	63315667	63409355	144	32	120000	-0.52281	0.69945	PLA2G16	76353	25kb
ENSG00000184743	11	63366559	63464393	138	15	120000	0.23826	0.40584	ATL3	76353	25kb
ENSG00000133318	11	63423918	63552363	175	13	120000	-0.046595	0.51858	RTN3	76353	25kb
ENSG00000168005	11	63555860	63620190	130	22	120000	-0.87324	0.80873	C11orf84	76353	25kb
ENSG00000072518	11	63581400	63703491	194	18	120000	0.19258	0.42364	MARK2	76353	25kb
ENSG00000167771	11	63653693	63709316	75	10	120000	0.028761	0.48853	RCOR2	76353	25kb
ENSG00000110583	11	63681431	63749800	129	11	120000	-0.31047	0.6219	NAA40	76353	25kb
ENSG00000176340	11	63717079	63769015	97	8	120000	-0.34359	0.63442	COX8A	76353	25kb
ENSG00000256100	11	63717092	63780818	120	9	120000	-0.45706	0.67619	AP000721.4	76353	25kb
ENSG00000167770	11	63728325	63794283	138	12	120000	-0.67397	0.74983	OTUB1	76353	25kb
ENSG00000133315	11	63741030	63958578	476	56	120000	1.2601	0.10382	MACROD1	76353	25kb
ENSG00000126500	11	63845660	63911645	188	28	120000	1.2731	0.10149	FLRT1	76353	25kb
ENSG00000168439	11	63927744	63997015	144	20	120000	1.4925	0.06779	STIP1	76353	25kb
ENSG00000149781	11	63949150	64016354	138	22	120000	1.6958	0.044957	FERMT3	76353	25kb
ENSG00000149743	11	63966271	64018726	104	21	120000	1.6082	0.053896	TRPT1	76353	25kb
ENSG00000149761	11	63968413	64023158	105	21	120000	1.6717	0.047297	NUDT22	76353	25kb
ENSG00000110011	11	63972750	64026758	108	21	120000	1.6782	0.046659	DNAJC4	76353	25kb
ENSG00000173511	11	63977010	64031259	105	21	120000	1.6223	0.052371	VEGFB	76353	25kb
ENSG00000173486	11	63983475	64036604	102	21	120000	1.8258	0.033942	FKBP2	76353	25kb
ENSG00000173457	11	63986956	64039413	100	21	120000	1.7122	0.043429	PPP1R14B	76353	25kb
ENSG00000149782	11	63993995	64061622	111	23	120000	1.8652	0.031077	PLCB3	76353	25kb
ENSG00000002330	11	64012302	64077176	94	22	120000	1.7779	0.03771	BAD	76353	25kb
ENSG00000173264	11	64012534	64081972	99	22	120000	1.763	0.038953	GPR137	76353	25kb
ENSG00000182450	11	64033774	64092503	74	19	120000	1.4977	0.067109	KCNK4	76353	25kb
ENSG00000219435	11	64042863	64097242	71	19	120000	1.4764	0.069925	TEX40	76353	25kb
ENSG00000173153	11	64048044	64109215	82	19	120000	1.4987	0.066972	ESRRA	76353	25kb
ENSG00000173113	11	64058932	64110556	69	16	120000	1.4396	0.074994	TRMT112	76353	25kb
ENSG00000126432	11	64060560	64114283	81	18	120000	1.4701	0.070769	PRDX5	76353	25kb
ENSG00000168071	11	64082695	64150006	135	16	120000	1.5978	0.05504	CCDC88B	76353	25kb
ENSG00000162302	11	64101620	64164687	143	18	120000	1.1979	0.11547	RPS6KA4	76353	25kb
ENSG00000181908	11	64191546	64244126	105	15	120000	-0.32543	0.62757	AP003774.4	76353	25kb
ENSG00000168065	11	64298098	64365347	117	17	120000	1.1936	0.11633	SLC22A11	76353	25kb
ENSG00000197891	11	64333113	64394820	94	12	120000	-0.57673	0.71794	SLC22A12	76353	25kb
ENSG00000110076	11	64348646	64515660	239	20	120000	0.077637	0.46906	NRXN2	76353	25kb
ENSG00000068831	11	64469383	64537928	99	13	120000	1.4707	0.070693	RASGRP2	76353	25kb
ENSG00000068976	11	64488861	64552769	86	9	120000	1.5263	0.063469	PYGM	76353	25kb
ENSG00000168066	11	64507078	64571258	74	8	120000	1.6085	0.053857	SF1	76353	25kb
ENSG00000168067	11	64531290	64595713	83	19	120000	0.69458	0.24366	MAP4K2	76353	25kb
ENSG00000133895	11	64545982	64603766	95	22	120000	0.41689	0.33838	MEN1	76353	25kb
ENSG00000171219	11	64565859	64637041	152	28	120000	-0.54174	0.706	CDC42BPG	76353	25kb
ENSG00000110047	11	64594114	64680768	163	20	120000	-0.53644	0.70417	EHD1	76353	25kb
ENSG00000110046	11	64637007	64709722	128	16	120000	-0.83054	0.79688	ATG2A	76353	25kb
ENSG00000068971	11	64660025	64726945	130	13	120000	-0.54491	0.70709	PPP2R5B	76353	25kb
ENSG00000149735	11	64676943	64728360	109	12	120000	-0.43069	0.66665	GPHA2	76353	25kb
ENSG00000168070	11	64679989	64764557	200	17	120000	-0.16478	0.56544	C11orf85	76353	25kb
ENSG00000168062	11	64730415	64789517	142	17	120000	0.33895	0.36732	BATF2	76353	25kb
ENSG00000213465	11	64756585	64814656	148	23	120000	0.69339	0.24403	ARL2	76353	25kb
ENSG00000273003	11	64756654	64833042	179	27	120000	0.82628	0.20432	RP11-399J13.3	76353	25kb
ENSG00000110025	11	64769880	64833044	151	22	120000	0.93117	0.17588	SNX15	76353	25kb
ENSG00000168061	11	64783373	64837300	133	18	120000	0.88016	0.18939	SAC3D1	76353	25kb
ENSG00000168060	11	64787290	64851021	159	21	120000	0.89949	0.1842	NAALADL1	76353	25kb
ENSG00000146670	11	64808772	64876636	126	23	120000	1.6157	0.053078	CDCA5	76353	25kb
ENSG00000162300	11	64826695	64880870	109	21	120000	1.4598	0.072166	ZFPL1	76353	25kb
ENSG00000187066	11	64827451	64881847	109	20	120000	1.4058	0.079886	TMEM262	76353	25kb
ENSG00000149823	11	64831796	64904332	130	23	120000	1.7571	0.039449	VPS51	76353	25kb
ENSG00000149809	11	64854317	64908856	90	17	120000	2.1483	0.015847	TM7SF2	76353	25kb
ENSG00000174276	11	64858875	64910170	82	17	120000	1.9929	0.023134	ZNHIT2	76353	25kb
ENSG00000149806	11	64863100	64914945	82	18	120000	1.8067	0.035403	FAU	76353	25kb
ENSG00000162298	11	64864252	64927004	103	22	120000	1.6141	0.053254	SYVN1	76353	25kb
ENSG00000149792	11	64864655	64919843	90	19	120000	1.7066	0.043949	MRPL49	76353	25kb
ENSG00000204710	11	64912625	64965715	94	21	120000	0.44554	0.32796	SPDYC	76353	25kb
ENSG00000254614	11	64920077	64974305	98	20	120000	0.31471	0.37649	AP003068.23	76353	25kb
ENSG00000014216	11	64923037	65004477	165	24	120000	0.19482	0.42277	CAPN1	76353	25kb
ENSG00000014138	11	65004233	65098060	112	14	120000	-0.24391	0.59635	POLA2	76353	25kb
ENSG00000149798	11	65057289	65114900	73	16	120000	-0.86524	0.80655	CDC42EP2	76353	25kb
ENSG00000133884	11	65076225	65145720	101	21	120000	-1.0494	0.853	DPF2	76353	25kb
ENSG00000173825	11	65097238	65150084	79	19	120000	-1.315	0.90574	TIGD3	76353	25kb
ENSG00000162241	11	65117663	65176172	78	22	120000	-1.4889	0.93174	SLC25A45	76353	25kb
ENSG00000126391	11	65129070	65205996	82	20	120000	-1.5872	0.94377	FRMD8	76353	25kb
ENSG00000173727	11	65197728	65259028	119	10	120000	0.53479	0.2964	AP000769.1	76353	25kb
ENSG00000142186	11	65267548	65331175	61	15	120000	2.1662	0.015149	SCYL1	76353	25kb
ENSG00000168056	11	65281276	65351401	83	16	120000	1.9606	0.024961	LTBP3	76353	25kb
ENSG00000173465	11	65312901	65366413	89	16	120000	1.5591	0.059487	SSSCA1	76353	25kb
ENSG00000176973	11	65314820	65366669	88	15	120000	1.5023	0.066511	FAM89B	76353	25kb
ENSG00000173442	11	65318509	65385121	112	20	120000	1.0454	0.14791	EHBP1L1	76353	25kb
ENSG00000269018	11	65333665	65384947	93	17	120000	0.68804	0.24572	AP001362.1	76353	25kb
ENSG00000173338	11	65335326	65388467	95	17	120000	0.59258	0.27673	KCNK7	76353	25kb
ENSG00000173327	11	65340226	65407853	112	21	120000	-0.37776	0.64719	MAP3K11	76353	25kb
ENSG00000197136	11	65358244	65429910	109	20	120000	-1.0933	0.86288	PCNXL3	76353	25kb
ENSG00000213445	11	65380568	65443401	91	16	120000	-1.3967	0.91875	SIPA1	76353	25kb
ENSG00000173039	11	65396067	65455565	99	14	120000	-1.2101	0.88688	RELA	76353	25kb
ENSG00000172977	11	65454467	65512075	154	16	120000	-0.79399	0.7864	KAT5	76353	25kb
ENSG00000172922	11	65457367	65513418	148	16	120000	-0.8074	0.79028	RNASEH2C	76353	25kb
ENSG00000254470	11	65518364	65573273	107	15	120000	-0.27178	0.6071	AP5B1	76353	25kb
ENSG00000172818	11	65529493	65589690	155	24	120000	0.085218	0.46604	OVOL1	76353	25kb
ENSG00000172757	11	65565493	65654497	210	25	120000	0.16731	0.43356	CFL1	76353	25kb
ENSG00000172803	11	65576112	65649367	177	22	120000	0.13325	0.447	SNX32	76353	25kb
ENSG00000172732	11	65599597	65660124	115	17	120000	0.26095	0.39707	MUS81	76353	25kb

ENSG00000172638	11	65608912	65666063	119	16	120000	0.25745	0.39842	EFEMP2	76353	25kb
ENSG00000172543	11	65622280	65676212	121	17	120000	0.063407	0.47472	CTSW	76353	25kb
ENSG00000172500	11	65626212	65681010	117	18	120000	0.088057	0.46492	FIBP	76353	25kb
ENSG00000175602	11	65632875	65684105	106	18	120000	0.28542	0.38766	CCDC85B	76353	25kb
ENSG00000175592	11	65634520	65693044	123	20	120000	0.11227	0.4553	FOSL1	76353	25kb
ENSG00000175573	11	65659279	65711588	97	15	120000	-0.63886	0.73854	C11orf68	76353	25kb
ENSG00000175550	11	65661728	65714032	95	15	120000	-0.76828	0.77884	DRAP1	76353	25kb
ENSG00000175467	11	65704160	65772299	160	14	120000	-0.6707	0.74879	SART1	76353	25kb
ENSG00000175376	11	65739016	65794647	172	17	120000	-0.61556	0.73091	EIF1AD	76353	25kb
ENSG00000175334	11	65744550	65796620	155	16	120000	-0.61092	0.72937	BANF1	76353	25kb
ENSG00000175315	11	65754312	65805976	149	18	120000	-0.45239	0.67451	CST6	76353	25kb
ENSG00000175294	11	65759223	65818988	159	19	120000	-0.19756	0.5783	CATSPER1	76353	25kb
ENSG00000175229	11	65784423	65841651	107	11	120000	0.4685	0.31971	GAL3ST3	76353	25kb
ENSG00000087365	11	65793200	65861779	120	14	120000	0.38188	0.35127	SF3B2	76353	25kb
ENSG00000255038	11	65809748	65862090	80	13	120000	0.23461	0.40726	RP11-1167A19.2	76353	25kb
ENSG00000175115	11	65812834	66037218	356	21	120000	-0.034406	0.51372	PACS1	76353	25kb
ENSG00000174996	11	65999765	66060331	90	14	120000	0.1167	0.45355	KLC2	76353	25kb
ENSG00000174903	11	66011004	66069963	92	18	120000	-0.18951	0.57515	RAB1B	76353	25kb
ENSG00000174871	11	66020661	66077772	93	20	120000	-0.48331	0.68556	CNIH2	76353	25kb
ENSG00000174851	11	66027051	66081641	100	21	120000	-0.57166	0.71623	YIF1A	76353	25kb
ENSG00000179292	11	66034341	66089135	97	22	120000	-0.78973	0.78516	TMEM151A	76353	25kb
ENSG00000174807	11	66056958	66109515	120	21	120000	-1.1449	0.87388	CD248	76353	25kb
ENSG00000174791	11	66072713	66129311	124	17	120000	-0.86748	0.80716	RIN1	76353	25kb
ENSG00000174744	11	66079804	66137596	132	16	120000	-0.67502	0.75017	BRMS1	76353	25kb
ENSG00000174684	11	66087843	66140163	121	14	120000	-0.55729	0.71133	B3GNT1	76353	25kb
ENSG00000255468	11	66090421	66157275	151	16	120000	-0.47599	0.68296	RP11-867G23.8	76353	25kb
ENSG00000174669	11	66104992	66164685	136	12	120000	-0.38604	0.65027	SLC29A2	76353	25kb
ENSG00000174576	11	66163475	66219178	87	18	120000	-0.35057	0.63705	NPAS4	76353	25kb
ENSG00000174547	11	66177546	66259209	119	17	120000	0.40374	0.3432	MRPL11	76353	25kb
ENSG00000174516	11	66209216	66269808	81	10	120000	0.61506	0.26926	PELI3	76353	25kb
ENSG00000254986	11	66222484	66302130	135	9	120000	0.68074	0.24802	DPP3	76353	25kb
ENSG00000256349	11	66251550	66326084	145	10	120000	0.50765	0.30585	CTD-3074O7.11	76353	25kb
ENSG00000174483	11	66253077	66326098	145	10	120000	0.50765	0.30585	BBS1	76353	25kb
ENSG00000174165	11	66263108	66338709	160	12	120000	0.38577	0.34983	ZDHHC24	76353	25kb
ENSG00000174080	11	66305934	66361312	101	13	120000	-0.29557	0.61622	CTSF	76353	25kb
ENSG00000182791	11	66332640	66385554	70	13	120000	-0.65166	0.74269	CCDC87	76353	25kb
ENSG00000173992	11	66335292	66398490	70	13	120000	-0.70509	0.75962	CCS	76353	25kb
ENSG00000239306	11	66359053	66419818	53	13	120000	-0.7387	0.76995	RBM14	76353	25kb
ENSG00000248643	11	66359097	66438940	71	16	120000	-0.442	0.67075	RBM14-RBM4	76353	25kb
ENSG00000173933	11	66359097	66459153	96	19	120000	-0.33138	0.62982	RBM4	76353	25kb
ENSG00000173914	11	66407469	66470392	59	15	120000	0.054153	0.47841	RBM4B	76353	25kb
ENSG00000173898	11	66427719	66521697	76	22	120000	-0.49102	0.68829	SPTBN2	76353	25kb
ENSG00000173715	11	66486922	66635987	130	25	120000	-1.3459	0.91083	C11orf80	76353	25kb
ENSG00000173653	11	66585306	66639017	47	15	120000	-2.0741	0.98096	RCE1	76353	25kb
ENSG00000173599	11	66590704	66750847	240	31	120000	-2.5081	0.99393	PC	76353	25kb
ENSG00000173621	11	66599118	66652946	90	12	120000	-1.9884	0.97662	LRFN4	76353	25kb
ENSG00000173237	11	66717748	66769475	52	18	120000	-2.0944	0.98189	C11orf86	76353	25kb
ENSG00000173227	11	66749249	66843334	117	32	120000	-2.3203	0.98984	SYT12	76353	25kb
ENSG00000173156	11	66799289	66864484	91	27	120000	-2.4249	0.99234	RHOD	76353	25kb
ENSG00000173120	11	66861740	67050558	219	27	120000	-1.8743	0.96955	KDM2A	76353	25kb
ENSG00000179038	11	66938391	66989638	44	13	120000	-1.8108	0.96491	AP001885.1	76353	25kb
ENSG00000173020	11	67008881	67079027	80	17	120000	-2.1131	0.9827	ADRBK1	76353	25kb
ENSG00000172932	11	67031018	67094956	93	19	120000	-2.184	0.98552	ANKRD13D	76353	25kb
ENSG00000172830	11	67045919	67105078	91	17	120000	-2.2625	0.98817	SSH3	76353	25kb
ENSG00000175482	11	67093248	67149443	68	12	120000	-1.9565	0.9748	POLD4	76353	25kb
ENSG00000175505	11	67106639	67166648	75	12	120000	-2.1677	0.98491	CLCF1	76353	25kb
ENSG00000172613	11	67134176	67190881	99	14	120000	-2.3472	0.99054	RAD9A	76353	25kb
ENSG00000172531	11	67140654	67213654	115	19	120000	-2.8237	0.99763	PPP1CA	76353	25kb
ENSG00000175463	11	67146386	67202560	94	16	120000	-2.662	0.99612	TBC1D10C	76353	25kb
ENSG00000172508	11	67157439	67218078	105	17	120000	-2.8208	0.9976	CARNS1	76353	25kb
ENSG00000175634	11	67170931	67227872	92	15	120000	-2.6525	0.99601	RPS6KB2	76353	25kb
ENSG00000213402	11	67177981	67230538	77	12	120000	-2.4272	0.99239	PTPRCAP	76353	25kb
ENSG00000172725	11	67180519	67236292	79	13	120000	-2.2358	0.98732	CORO1B	76353	25kb
ENSG00000175514	11	67193772	67245200	69	10	120000	-2.0028	0.9774	GPR152	76353	25kb
ENSG00000175544	11	67194877	67251699	78	12	120000	-1.9584	0.97491	CABP4	76353	25kb
ENSG00000172663	11	67206824	67261743	81	15	120000	-1.9873	0.97656	TMEM134	76353	25kb
ENSG00000110711	11	67225512	67283574	80	20	120000	-2.2294	0.98711	AIP	76353	25kb
ENSG00000110697	11	67234239	67298734	92	21	120000	-2.014	0.978	PITPNM1	76353	25kb
ENSG00000167797	11	67248968	67301120	76	17	120000	-1.7633	0.96108	CDK2AP2	76353	25kb
ENSG00000167791	11	67261383	67315899	84	15	120000	-0.93862	0.82604	CABP2	76353	25kb
ENSG00000084207	11	67326066	67379131	149	14	120000	-0.058371	0.52327	GSTP1	76353	25kb
ENSG00000184224	11	67345351	67399177	153	15	120000	0.046796	0.48134	C11orf72	76353	25kb
ENSG00000167792	11	67349323	67405006	158	15	120000	0.2297	0.40916	NDUFV1	76353	25kb
ENSG00000167799	11	67370409	67422401	168	23	120000	0.46178	0.32212	NUDT8	76353	25kb
ENSG00000167800	11	67373774	67432031	179	24	120000	0.33129	0.37021	TBX10	76353	25kb
ENSG00000132744	11	67385026	67443130	206	26	120000	0.53992	0.29463	ACY3	76353	25kb
ENSG00000132746	11	67404633	67473671	197	25	120000	0.10791	0.45703	ALDH3B2	76353	25kb
ENSG00000110057	11	67733575	67797452	159	17	120000	-0.99183	0.83936	UNC93B1	76353	25kb
ENSG00000006534	11	67751048	67821744	157	25	120000	-0.49699	0.6904	ALDH3B1	76353	25kb
ENSG00000110717	11	67773084	67829111	131	22	120000	-0.3098	0.62164	NDUFS8	76353	25kb
ENSG00000110719	11	67781483	67843362	131	22	120000	0.01078	0.4957	TCIRG1	76353	25kb
ENSG00000110721	11	67795326	67913911	196	27	120000	0.18585	0.42628	CHKA	76353	25kb
ENSG00000110066	11	67897330	68006295	204	25	120000	0.60025	0.27417	SUV420H1	76353	25kb
ENSG00000171067	11	68003803	68064469	139	20	120000	0.59777	0.275	C11orf24	76353	25kb
ENSG00000162337	11	68055077	68241743	431	41	120000	0.57233	0.28355	LRP5	76353	25kb
ENSG00000110075	11	68203186	68407802	469	24	120000	1.0243	0.15286	PPP6R3	76353	25kb
ENSG00000069482	11	68426247	68483643	129	18	120000	-0.28881	0.61364	GAL	76353	25kb
ENSG00000132749	11	68449908	68544032	171	18	120000	-0.77092	0.77962	MTL5	76353	25kb
ENSG00000110090	11	68497088	68636878	305	32	120000	-0.48471	0.68606	CPT1A	76353	25kb
ENSG00000197345	11	68633744	68696303	144	15	120000	0.64644	0.259	MRPL21	76353	25kb
ENSG00000132740	11	68646310	68733070	243	18	120000	0.22842	0.40966	IGHMBP2	76353	25kb
ENSG00000172938	11	68722490	68773455	164	20	120000	0.11455	0.4544	MRGPRD	76353	25kb
ENSG00000172935	11	68746863	68805877	166	25	120000	0.23506	0.40708	MRGPRF	76353	25kb
ENSG00000162341	11	68791365	68883072	339	29	120000	-1.102	0.86477	TPCN2	76353	25kb
ENSG00000172927	11	69036605	69207494	332	42	120000	-0.42569	0.66483	MYEOV	76353	25kb
ENSG00000110092	11	69430855	69494242	150	24	120000	0.67668	0.24931	CCND1	76353	25kb

ENSG00000149716	11	69442844	69515184	168	22	120000	0.31819	0.37517	ORAOV1	76353	25kb
ENSG00000162344	11	69488000	69544410	157	29	120000	-1.5878	0.94383	FGF19	76353	25kb
ENSG00000268351	11	69562254	69612718	215	44	120000	-0.0036882	0.50147	AP001888.1	76353	25kb
ENSG00000075388	11	69562797	69615171	226	44	120000	-0.068711	0.52739	FGF4	76353	25kb
ENSG00000186895	11	69599992	69658792	259	24	120000	0.6136	0.26974	FGF3	76353	25kb
ENSG00000131620	11	69899408	70060634	649	70	120000	-0.59088	0.7227	ANO1	76353	25kb
ENSG00000168040	11	70024269	70078496	133	25	120000	-0.37585	0.64649	FADD	76353	25kb
ENSG00000131626	11	70091806	70255509	320	15	120000	1.3005	0.096713	PPFIA1	76353	25kb
ENSG00000085733	11	70219510	70307690	164	20	120000	1.5077	0.065821	CTTN	76353	25kb
ENSG00000162105	11	70288961	70988623	2020	160	120000	-0.67815	0.75116	SHANK2	76353	25kb
ENSG00000172893	11	71114239	71188914	276	13	120000	-0.18771	0.57445	DHCR7	76353	25kb
ENSG00000172890	11	71139155	71260153	399	23	120000	0.097478	0.46117	NADSYN1	76353	25kb
ENSG00000244411	11	71213313	71264210	161	18	120000	-0.60593	0.72772	KRTAP5-7	76353	25kb
ENSG00000241233	11	71224071	71275253	198	17	120000	-1.1284	0.87042	KRTAP5-8	76353	25kb
ENSG00000254997	11	71234466	71285653	229	19	120000	-1.2006	0.88504	KRTAP5-9	76353	25kb
ENSG00000204572	11	71251609	71304980	231	27	120000	-0.96868	0.83365	KRTAP5-10	76353	25kb
ENSG00000204571	11	71267901	71339399	305	28	120000	-1.0514	0.85345	KRTAP5-11	76353	25kb
ENSG00000187811	11	71267956	71343487	322	27	120000	-1.0772	0.8593	AP000867.1	76353	25kb
ENSG00000158483	11	71473556	71537282	229	14	120000	-1.1099	0.86649	FAM86C1	76353	25kb
ENSG00000184276	11	71519246	71573756	191	15	120000	-0.10332	0.54114	DEFB108B	76353	25kb
ENSG00000254469	11	71551555	71664700	404	9	120000	0.59823	0.27484	RP11-849H4.2	76353	25kb
ENSG00000137522	11	71614747	71733643	301	10	120000	0.45018	0.32629	RNF121	76353	25kb
ENSG00000137496	11	71684587	71741761	130	11	120000	0.25746	0.39841	IL18BP	76353	25kb
ENSG00000137497	11	71688910	71816739	275	11	120000	0.43042	0.33344	NUMA1	76353	25kb
ENSG00000184154	11	71766382	71846828	205	12	120000	0.68822	0.24566	LRTOMT	76353	25kb
ENSG00000149357	11	71771941	71839433	153	9	120000	0.66458	0.25316	LAMTOR1	76353	25kb
ENSG00000110200	11	71792424	71848826	155	12	120000	0.8013	0.21148	ANAPC15	76353	25kb
ENSG00000110203	11	71800915	71875936	248	27	120000	0.91254	0.18074	FOLR3	76353	25kb
ENSG00000110195	11	71875602	71932345	150	20	120000	0.89146	0.18634	FOLR1	76353	25kb
ENSG00000165457	11	71902645	71957994	125	20	120000	-0.73273	0.76814	FOLR2	76353	25kb
ENSG00000165458	11	71909745	71975149	137	23	120000	-0.70332	0.75907	INPPL1	76353	25kb
ENSG00000165462	11	71925121	71981708	113	20	120000	-1.0357	0.84982	PHOX2A	76353	25kb
ENSG00000162129	11	71978469	72170692	316	14	120000	-0.19424	0.57701	CLPB	76353	25kb
ENSG00000186642	11	72262185	72410635	440	47	120000	0.18686	0.42589	PDE2A	76353	25kb
ENSG00000186635	11	72371114	72529644	394	32	120000	-0.71821	0.76369	ARAP1	76353	25kb
ENSG00000214530	11	72440774	72529726	209	18	120000	-0.22161	0.58769	STARD10	76353	25kb
ENSG00000168010	11	72500353	72579719	113	18	120000	-1.2708	0.8981	ATG16L2	76353	25kb
ENSG00000137478	11	72522790	72878306	356	38	120000	-1.1141	0.86738	FCHSD2	76353	25kb
ENSG00000175591	11	72904343	72972397	159	45	120000	1.0509	0.14665	P2RY2	76353	25kb
ENSG00000171631	11	72950550	73034662	185	23	120000	-1.0342	0.84949	P2RY6	76353	25kb
ENSG00000110237	11	72994334	73105136	245	20	120000	-1.3659	0.91402	ARHGEF17	76353	25kb
ENSG00000054967	11	73062309	73133519	129	15	120000	-1.0505	0.85325	RELT	76353	25kb
ENSG00000054965	11	73086532	73334234	375	24	120000	-1.0362	0.84996	FAM168A	76353	25kb
ENSG00000021300	11	73332223	73398864	190	30	120000	-0.69273	0.75576	PLEKHB1	76353	25kb
ENSG00000175582	11	73361683	73497182	342	25	120000	-1.1483	0.87458	RAB6A	76353	25kb
ENSG00000175581	11	73473361	73601178	378	24	120000	-0.86064	0.80528	MRPL48	76353	25kb
ENSG00000181924	11	73558712	73613033	181	13	120000	-0.49299	0.68899	COA4	76353	25kb
ENSG00000175575	11	73562744	73663790	268	15	120000	-0.37729	0.64702	PAAF1	76353	25kb
ENSG00000187726	11	73636364	73706411	178	23	120000	-0.32633	0.62791	DNAJB13	76353	25kb
ENSG00000175567	11	73660712	73719352	176	23	120000	-0.19499	0.5773	UCP2	76353	25kb
ENSG00000175564	11	73686326	73745480	116	20	120000	0.54757	0.29199	UCP3	76353	25kb
ENSG00000168014	11	73698763	73907255	436	25	120000	1.643	0.050195	C2CD3	76353	25kb
ENSG00000214517	11	73857144	73990748	294	18	120000	1.3442	0.08945	PPME1	76353	25kb
ENSG00000149380	11	73921846	74047702	302	20	120000	0.78434	0.21642	P4HA3	76353	25kb
ENSG00000165434	11	74016363	74134518	296	19	120000	1.6559	0.048876	PGM2L1	76353	25kb
ENSG00000175538	11	74140886	74203774	181	29	120000	0.96498	0.16728	KCNE3	76353	25kb
ENSG00000175536	11	74177757	74229778	155	17	120000	-1.0412	0.8511	LIPT2	76353	25kb
ENSG00000077514	11	74179896	74405162	696	43	120000	0.96342	0.16767	POLD3	76353	25kb
ENSG00000054938	11	74382474	74467430	219	47	120000	0.097704	0.46108	CHRDL2	76353	25kb
ENSG00000166439	11	74434913	74578458	267	19	120000	-0.92826	0.82336	RNF169	76353	25kb
ENSG00000166435	11	74493784	74685245	304	20	120000	-0.66743	0.74775	XRRA1	76353	25kb
ENSG00000118363	11	74635292	74715076	131	18	120000	-0.54152	0.70593	SPCS2	76353	25kb
ENSG00000162139	11	74674179	74754938	164	23	120000	-0.54103	0.70576	NEU3	76353	25kb
ENSG00000171561	11	74774758	74825799	105	15	120000	0.17484	0.4306	OR2AT4	76353	25kb
ENSG00000137491	11	74786608	74942594	417	33	120000	0.11611	0.45378	SLCO2B1	76353	25kb
ENSG00000261594	11	74926950	74979742	85	12	120000	-1.0108	0.84395	TPBGL	76353	25kb
ENSG00000137486	11	74950226	75087873	313	45	120000	-1.2161	0.88802	ARRB1	76353	25kb
ENSG00000149273	11	75085530	75158324	158	27	120000	-1.1406	0.87298	RPS3	76353	25kb
ENSG00000149243	11	75108438	75166674	107	20	120000	-1.2615	0.89643	KLHL35	76353	25kb
ENSG00000158555	11	75120685	75261948	379	33	120000	-0.27256	0.6074	GDPD5	76353	25kb
ENSG00000149257	11	75248101	75308828	218	37	120000	0.38905	0.34862	SERPINH1	76353	25kb
ENSG00000171533	11	75272963	75405165	280	25	120000	0.0063894	0.49745	MAP6	76353	25kb
ENSG00000166391	11	75403864	75469003	167	22	120000	-1.0561	0.85455	MOGAT2	76353	25kb
ENSG00000062282	11	75445557	75537579	148	9	120000	-1.064	0.85634	DGAT2	76353	25kb
ENSG00000198382	11	75501212	75879239	625	27	120000	-1.0136	0.8446	UVRAG	76353	25kb
ENSG00000085741	11	75872369	75946780	218	37	120000	-1.3004	0.90327	WNT11	76353	25kb
ENSG00000137492	11	76036000	76117015	185	29	120000	-2.3847	0.99145	PRKRIR	76353	25kb
ENSG00000179240	11	76067357	76150663	214	35	120000	-2.029	0.97877	RP11-111M22.2	76353	25kb
ENSG00000158636	11	76130967	76289069	323	24	120000	-1.0373	0.8502	C11orf30	76353	25kb
ENSG00000137507	11	76343568	76406791	245	49	120000	-0.86524	0.80655	LRRC32	76353	25kb
ENSG00000182704	11	76468295	76534198	158	26	120000	0.33774	0.36778	TSKU	76353	25kb
ENSG00000078124	11	76546911	76762841	584	16	120000	-0.031429	0.51254	ACER3	76353	25kb
ENSG00000198488	11	76720385	76778096	169	24	120000	-0.16059	0.56379	B3GNT6	76353	25kb
ENSG00000149260	11	76752979	76862201	325	51	120000	-1.318	0.90625	CAPN5	76353	25kb
ENSG00000254550	11	76788886	76839377	139	28	120000	-1.1906	0.88309	OMP	76353	25kb
ENSG00000137474	11	76814310	76951284	461	51	120000	0.41576	0.33879	MYO7A	76353	25kb
ENSG00000178795	11	76902603	77037732	451	27	120000	1.3289	0.091936	GDPD4	76353	25kb
ENSG00000149269	11	77007752	77210680	486	26	120000	1.6749	0.04698	PAK1	76353	25kb
ENSG00000268635	11	77159416	77213074	138	21	120000	1.455	0.072838	DKFZP434E1119	76353	25kb
ENSG00000074201	11	77200981	77373850	349	34	120000	0.21906	0.4133	CLNS1A	76353	25kb
ENSG00000178301	11	77275436	77346400	157	11	120000	-0.22761	0.59003	AQP11	76353	25kb
ENSG00000048649	11	77346041	77557063	412	17	120000	0.24867	0.40181	RSF1	76353	25kb
ENSG00000087884	11	77507155	77654478	270	15	120000	0.49735	0.30947	AAMDC	76353	25kb
ENSG00000149262	11	77564766	77730724	386	27	120000	0.59577	0.27566	INTS4	76353	25kb
ENSG00000151364	11	77701761	77782237	295	27	120000	0.71228	0.23815	KCTD14	76353	25kb
ENSG00000259112	11	77703017	77815911	400	28	120000	0.93747	0.17426	NDUFC2-KCTD14	76353	25kb

ENSG00000151365	11	77749907	77804397	185	15	120000	1.2929	0.098026	THRSP	76353	25kb
ENSG00000151366	11	77754350	77816265	216	15	120000	1.3865	0.0828	NDUFC2	76353	25kb
ENSG00000159063	11	77786982	77875706	340	11	120000	1.4636	0.071654	ALG8	76353	25kb
ENSG00000188997	11	77857295	77924868	130	25	120000	0.052125	0.47921	KCTD21	76353	25kb
ENSG00000118369	11	77874858	77950757	164	20	120000	0.56818	0.28496	USP35	76353	25kb
ENSG00000033327	11	77901343	78154394	456	10	120000	0.34725	0.3642	GAB2	76353	25kb
ENSG00000137513	11	78122007	78310919	420	11	120000	-0.28102	0.61065	NARS2	76353	25kb
ENSG00000149256	11	78338876	79176992	2432	233	120000	0.24047	0.40498	TENM4	76353	25kb
ENSG00000182103	11	82418053	82469906	158	22	120000	0.80187	0.21131	FAM181B	76353	25kb
ENSG00000137509	11	82509544	82706626	493	32	120000	0.26264	0.39641	PRCP	76353	25kb
ENSG00000165490	11	82586017	82694319	254	19	120000	-0.2594	0.60234	C11orf82	76353	25kb
ENSG00000137502	11	82659175	82807965	314	28	120000	-0.48844	0.68738	RAB30	76353	25kb
ENSG00000165494	11	82843030	82923493	135	20	120000	-0.49456	0.68954	PCF11	76353	25kb
ENSG00000137494	11	82879781	82996736	256	23	120000	-0.029039	0.51158	ANKRD42	76353	25kb
ENSG00000137500	11	82945139	83022450	182	16	120000	0.39767	0.34544	CCDC90B	76353	25kb
ENSG00000150672	11	83141055	85363966	6030	191	120000	-0.44907	0.67331	DLG2	76353	25kb
ENSG00000171204	11	85314629	85372580	112	16	120000	-1.0572	0.85479	TMEM126B	76353	25kb
ENSG00000171202	11	85334011	85392591	99	13	120000	-1.0117	0.84416	TMEM126A	76353	25kb
ENSG00000137504	11	85345752	85418951	122	20	120000	-0.98124	0.83676	CREBZF	76353	25kb
ENSG00000179071	11	85369893	85422320	86	17	120000	-0.79397	0.78639	CCDC89	76353	25kb
ENSG00000137501	11	85380267	85547184	374	25	120000	-0.3172	0.62445	SYTL2	76353	25kb
ENSG00000215504	11	85538600	85590986	152	10	120000	1.2552	0.1047	AP000974.1	76353	25kb
ENSG00000150676	11	85541144	85656064	351	24	120000	1.0707	0.14216	CCDC83	76353	25kb
ENSG00000073921	11	85643727	85805924	456	27	120000	1.2593	0.10396	PICALM	76353	25kb
ENSG00000074266	11	85930586	86014855	187	20	120000	1.1669	0.12162	EED	76353	25kb
ENSG00000149196	11	85988253	86081969	287	20	120000	1.3608	0.086786	C11orf73	76353	25kb
ENSG00000149201	11	86060778	86159151	378	33	120000	0.36155	0.35884	CCDC81	76353	25kb
ENSG00000151376	11	86127150	86408678	1075	53	120000	-0.34188	0.63378	ME3	76353	25kb
ENSG00000150687	11	86477101	86688952	735	68	120000	0.61942	0.26782	PRSS23	76353	25kb
ENSG00000174804	11	86631721	86691433	188	22	120000	0.43343	0.33235	FZD4	76353	25kb
ENSG00000166575	11	86723886	87059800	1585	43	120000	0.82321	0.2052	TMEM135	76353	25kb
ENSG00000123892	11	87821410	87933635	447	37	120000	0.25954	0.39761	RAB38	76353	25kb
ENSG00000109861	11	88001760	88095955	303	29	120000	-0.11641	0.54634	CTSC	76353	25kb
ENSG00000168959	11	88212744	88824113	1852	48	120000	-0.91785	0.82065	GRM5	76353	25kb
ENSG00000077498	11	88885620	89053927	409	21	120000	0.33679	0.36814	TYR	76353	25kb
ENSG00000086991	11	89032524	89347779	669	45	120000	0.30841	0.37888	NOX4	76353	25kb
ENSG00000214414	11	89418467	89476040	129	9	120000	0.20667	0.41813	TRIM77	76353	25kb
ENSG00000168930	11	89505823	89566743	14	3	120000	1.2597	0.10388	TRIM49	76353	25kb
ENSG00000189253	11	89577450	89634185	3	2	120000	1.0277	0.15204	TRIM64B	76353	25kb
ENSG00000223417	11	89619279	89679935	22	5	120000	2.0364	0.020857	TRIM49D1	76353	25kb
ENSG00000204449	11	89739274	89831558	10	5	120000	0.82616	0.20436	TRIM49C	76353	25kb
ENSG00000077616	11	89839683	89951062	420	22	120000	0.19757	0.42169	NAALAD2	76353	25kb
ENSG00000110172	11	89909328	89981532	244	26	120000	0.37556	0.35362	CHORDC1	76353	25kb
ENSG00000165323	11	92060262	92654618	1300	61	120000	-0.58517	0.72078	FAT3	76353	25kb
ENSG00000134640	11	92677886	92743232	187	27	120000	0.73991	0.22968	MTNR1B	76353	25kb
ENSG00000180773	11	92852341	92956130	211	26	120000	0.63897	0.26142	SLC36A4	76353	25kb
ENSG00000165325	11	93038137	93196653	475	28	120000	-0.8612	0.80543	CCDC67	76353	25kb
ENSG00000166002	11	93186638	93301674	368	35	120000	0.40257	0.34363	SMCO4	76353	25kb
ENSG00000166004	11	93369805	93488522	239	24	120000	-0.56013	0.7123	KIAA1731	76353	25kb
ENSG00000166012	11	93438114	93542557	220	19	120000	-0.28726	0.61304	TAF1D	76353	25kb
ENSG00000182919	11	93449757	93522915	176	15	120000	-0.53917	0.70511	C11orf54	76353	25kb
ENSG00000042429	11	93492393	93572861	173	16	120000	-0.16827	0.56681	MED17	76353	25kb
ENSG00000214376	11	93526398	93608697	260	24	120000	-0.18088	0.57177	VSTM5	76353	25kb
ENSG00000181333	11	93729527	93871917	328	27	120000	-0.44764	0.67279	HEPHL1	76353	25kb
ENSG00000110218	11	93837094	93940138	267	23	120000	0.45706	0.32381	PANX1	76353	25kb
ENSG00000183560	11	94013759	94065887	181	28	120000	-1.2141	0.88764	FOLR4	76353	25kb
ENSG00000123901	11	94085477	94159585	290	24	120000	-0.23927	0.59455	GPR83	76353	25kb
ENSG00000020922	11	94127895	94252074	330	21	120000	-0.33343	0.6306	MRE11A	76353	25kb
ENSG00000168876	11	94201795	94257749	137	15	120000	-0.63231	0.73641	ANKRD49	76353	25kb
ENSG00000134627	11	94252006	94379587	361	34	120000	-1.3813	0.91641	PIWIL4	76353	25kb
ENSG00000196371	11	94252017	94308063	134	19	120000	-1.0177	0.84559	FUT4	76353	25kb
ENSG00000166025	11	94414597	94634918	537	43	120000	-0.61426	0.73048	AMOTL1	76353	25kb
ENSG00000150316	11	94670787	94731776	196	32	120000	0.2138	0.41535	CWC15	76353	25kb
ENSG00000186280	11	94681845	94757682	199	27	120000	-0.66668	0.74751	KDM4D	76353	25kb
ENSG00000235268	11	94733422	94785760	94	21	120000	-0.43216	0.66719	KDM4E	76353	25kb
ENSG00000149218	11	94797974	94890809	329	42	120000	0.58972	0.27769	ENDOD1	76353	25kb
ENSG00000149212	11	94873704	94990705	191	40	120000	1.6107	0.053622	SESN3	76353	25kb
ENSG00000077458	11	95477106	95548573	221	21	120000	-0.71475	0.76262	FAM76B	76353	25kb
ENSG00000166037	11	95498129	95590857	290	15	120000	-0.73149	0.76776	CEP57	76353	25kb
ENSG00000087053	11	95541046	95683479	377	22	120000	-1.465	0.92855	MTMR2	76353	25kb
ENSG00000184384	11	95684762	96101344	1343	139	120000	-0.33501	0.63119	MAML2	76353	25kb
ENSG00000149231	11	96060933	96148087	159	22	120000	1.4803	0.069393	CCDC82	76353	25kb
ENSG00000183340	11	96098153	96265738	395	35	120000	1.2926	0.09807	JRKL	76353	25kb
ENSG00000149972	11	98866683	100254616	5633	239	120000	-1.6075	0.94602	CNTN5	76353	25kb
ENSG00000165895	11	100533384	100887668	1511	66	120000	-0.28392	0.61177	ARHGAP42	76353	25kb
ENSG00000170647	11	100837811	100889663	237	30	120000	-0.024696	0.50985	TMEM133	76353	25kb
ENSG00000082175	11	100875355	101026255	494	31	120000	1.0736	0.14151	PGR	76353	25kb
ENSG00000137672	11	101297295	101768293	1809	51	120000	1.7717	0.038218	TRPC6	76353	25kb
ENSG00000187151	11	101736405	101812253	201	31	120000	1.1183	0.13171	ANGPTL5	76353	25kb
ENSG00000110318	11	101760746	101896789	332	23	120000	0.22181	0.41223	KIAA1377	76353	25kb
ENSG00000137691	11	101893174	101980291	197	18	120000	-0.67441	0.74997	C11orf70	76353	25kb
ENSG00000137693	11	101956192	102129154	380	24	120000	-1.3362	0.90926	YAP1	76353	25kb
ENSG00000023445	11	102163215	102235134	122	15	120000	0.76143	0.2232	BIRC3	76353	25kb
ENSG00000110330	11	102192942	102274401	162	16	120000	2.3554	0.0092512	BIRC2	76353	25kb
ENSG00000152558	11	102242063	102366115	304	30	120000	2.0258	0.021395	TMEM123	76353	25kb
ENSG00000255482	11	102312986	102394532	260	32	120000	0.46608	0.32058	RP11-315O6.2	76353	25kb
ENSG00000137673	11	102366239	102426484	229	45	120000	0.084843	0.46619	MMP7	76353	25kb
ENSG00000137674	11	102422566	102521063	538	39	120000	-0.42892	0.66601	MMP20	76353	25kb
ENSG00000255798	11	102527041	102579644	221	28	120000	-0.0047343	0.50189	RP11-817J15.3	76353	25kb
ENSG00000137675	11	102537218	102601537	310	28	120000	0.57973	0.28105	MMP27	76353	25kb
ENSG00000118113	11	102557526	102622781	309	31	120000	0.9527	0.17037	MMP8	76353	25kb
ENSG00000166670	11	102616234	102676359	228	46	120000	0.22266	0.4119	MMP10	76353	25kb
ENSG00000196611	11	102635651	102693891	228	43	120000	0.48019	0.31555	MMP1	76353	25kb
ENSG00000149968	11	102681532	102739534	181	19	120000	1.3517	0.088244	MMP3	76353	25kb
ENSG00000137745	11	102788724	102851463	170	35	120000	-0.079718	0.53177	MMP13	76353	25kb
ENSG00000137692	11	102907805	102987944	130	22	120000	1.6235	0.052237	DCUN1D5	76353	25kb

ENSG00000187240	11	102955160	103375591	1841	34	120000	1.3902	0.082236	DYNC2H1	76353	25kb
ENSG00000170962	11	103752914	104060107	966	73	120000	-1.8854	0.97031	PDGFD	76353	25kb
ENSG00000170967	11	103882308	103934922	143	24	120000	-1.5316	0.93719	DDI1	76353	25kb
ENSG00000204403	11	104731445	104794397	148	15	120000	-0.20249	0.58023	CASP12	76353	25kb
ENSG00000196954	11	104788593	104865163	217	20	120000	-1.6595	0.95149	CASP4	76353	25kb
ENSG00000137757	11	104839962	104918895	250	25	120000	-0.022137	0.50883	CASP5	76353	25kb
ENSG00000137752	11	104871170	104997158	421	25	120000	-0.30185	0.61862	CASP1	76353	25kb
ENSG00000204397	11	104887053	104997158	382	23	120000	-0.3651	0.64248	CARD16	76353	25kb
ENSG00000255221	11	104938196	104997158	196	15	120000	-0.79322	0.78618	CARD17	76353	25kb
ENSG00000255501	11	104983448	105035453	207	12	120000	-0.070454	0.52808	CARD18	76353	25kb
ENSG00000152578	11	105455721	105877819	919	47	120000	0.26798	0.39436	GRIA4	76353	25kb
ENSG00000170903	11	105841350	105918130	163	18	120000	-0.5849	0.72069	MSANTD4	76353	25kb
ENSG00000182359	11	105896825	105973492	150	24	120000	-1.1474	0.87438	KBTBD3	76353	25kb
ENSG00000149313	11	105921228	105994437	144	20	120000	-1.2741	0.89869	AASDHPPT	76353	25kb
ENSG00000152402	11	106519738	106914250	1161	63	120000	0.3103	0.37817	GUCY1A2	76353	25kb
ENSG00000152404	11	107172071	107353572	690	26	120000	2.1296	0.016603	CWF19L2	76353	25kb
ENSG00000137760	11	107348452	107461472	361	27	120000	-0.85131	0.8027	ALKBH8	76353	25kb
ENSG00000110675	11	107436817	107562505	388	43	120000	0.57413	0.28294	ELMOD1	76353	25kb
ENSG00000268467	11	107437471	107488949	155	18	120000	0.95901	0.16878	AP000889.3	76353	25kb
ENSG00000170290	11	107553104	107615419	159	28	120000	-1.1167	0.86794	SLN	76353	25kb
ENSG00000268602	11	107557998	107620135	153	22	120000	-1.0065	0.8429	AP002353.1	76353	25kb
ENSG00000214306	11	107618129	107672344	252	18	120000	-0.2508	0.59901	AP001024.2	76353	25kb
ENSG00000214305	11	107625219	107677316	249	17	120000	-0.87876	0.81023	AP001024.1	76353	25kb
ENSG00000110660	11	107636717	107824019	685	41	120000	-2.1309	0.98345	SLC35F2	76353	25kb
ENSG00000179331	11	107774229	107859208	232	38	120000	-0.60239	0.72654	RAB39A	76353	25kb
ENSG00000166266	11	107854459	108003503	309	22	120000	0.094761	0.46225	CUL5	76353	25kb
ENSG00000075239	11	107967243	108043503	141	23	120000	0.51243	0.30417	ACAT1	76353	25kb
ENSG00000149308	11	108002942	108118369	177	19	120000	0.39072	0.348	NPAT	76353	25kb
ENSG00000149311	11	108068211	108264829	260	12	120000	0.066864	0.47335	ATM	76353	25kb
ENSG00000269042	11	108105255	108161480	72	8	120000	0.040671	0.48378	AP001925.1	76353	25kb
ENSG00000166323	11	108154246	108363258	321	14	120000	0.12911	0.44864	C11orf65	76353	25kb
ENSG00000178202	11	108317832	108394159	125	19	120000	-0.025891	0.51033	KDELC2	76353	25kb
ENSG00000110723	11	108351158	108489465	330	35	120000	1.3455	0.089231	EXPH5	76353	25kb
ENSG00000178105	11	108510752	108836657	632	27	120000	0.3813	0.35149	DDX10	76353	25kb
ENSG00000185742	11	109267846	109324840	54	13	120000	-0.7165	0.76316	C11orf87	76353	25kb
ENSG00000149289	11	109939087	110067566	356	23	120000	0.31625	0.37591	ZC3H12C	76353	25kb
ENSG00000137710	11	110020605	110192447	337	22	120000	0.57742	0.28183	RDX	76353	25kb
ENSG00000137714	11	110275607	110360605	233	18	120000	-1.4106	0.92082	FDX1	76353	25kb
ENSG00000137727	11	110422766	110608912	465	28	120000	-0.82427	0.79511	ARHGAP20	76353	25kb
ENSG00000150750	11	111101707	111182126	254	34	120000	0.46116	0.32234	C11orf53	76353	25kb
ENSG00000196167	11	111139114	111200770	207	28	120000	0.527	0.2991	COLCA1	76353	25kb
ENSG00000214290	11	111144565	111204460	213	29	120000	0.3465	0.36448	COLCA2	76353	25kb
ENSG00000110777	11	111197977	111351355	569	34	120000	-0.21752	0.5861	POU2AF1	76353	25kb
ENSG00000255093	11	111297064	111353876	173	32	120000	-0.2048	0.58114	RP11-794P6.2	76353	25kb
ENSG00000137707	11	111313251	111408079	202	29	120000	0.67489	0.24987	BTG4	76353	25kb
ENSG00000183644	11	111360510	111432756	124	14	120000	0.57093	0.28402	C11orf88	76353	25kb
ENSG00000204381	11	111386005	111457264	109	14	120000	-0.085598	0.53411	LAYN	76353	25kb
ENSG00000170145	11	111448115	111626577	228	13	120000	-0.036029	0.51437	SIK2	76353	25kb
ENSG00000137713	11	111572632	111662151	84	18	120000	1.5987	0.054943	PPP2R1B	76353	25kb
ENSG00000086848	11	111627919	111767305	166	18	120000	1.7991	0.036004	ALG9	76353	25kb
ENSG00000258529	11	111632010	111775149	177	17	120000	1.7741	0.038023	ALG9	76353	25kb
ENSG00000255561	11	111719780	111776967	82	9	120000	1.3268	0.092286	FDXACB1	76353	25kb
ENSG00000137720	11	111724659	111781699	81	8	120000	1.2138	0.11242	C11orf1	76353	25kb
ENSG00000109846	11	111754289	111819446	86	11	120000	1.2305	0.10925	CRYAB	76353	25kb
ENSG00000170276	11	111757966	111814574	68	11	120000	1.3588	0.087101	HSPB2	76353	25kb
ENSG00000254445	11	111758460	111822595	78	12	120000	1.519	0.064378	HSPB2-C11orf52	76353	25kb
ENSG00000149300	11	111763756	111822596	72	12	120000	1.5189	0.064395	C11orf52	76353	25kb
ENSG00000150764	11	111772868	111918308	162	19	120000	1.2667	0.10263	DIXDC1	76353	25kb
ENSG00000150768	11	111870538	111960114	107	12	120000	1.1048	0.13462	DLAT	76353	25kb
ENSG00000150773	11	111909734	111969998	85	12	120000	1.1957	0.11591	PIH1D2	76353	25kb
ENSG00000150776	11	111919810	111980874	88	13	120000	1.5385	0.061969	C11orf57	76353	25kb
ENSG00000150779	11	111930524	111982522	76	12	120000	1.6681	0.047649	TIMM8B	76353	25kb
ENSG00000204370	11	111932497	112015353	137	27	120000	1.5718	0.058002	SDHD	76353	25kb
ENSG00000255292	11	111932627	112089528	308	36	120000	1.3106	0.094992	SDHD	76353	25kb
ENSG00000150782	11	111988974	112059840	155	23	120000	0.46935	0.31941	IL18	76353	25kb
ENSG00000150783	11	112013095	112068282	128	19	120000	0.51392	0.30365	TEX12	76353	25kb
ENSG00000272790	11	112016253	112096394	182	22	120000	0.90792	0.18196	AP002884.3	76353	25kb
ENSG00000197580	11	112021190	112120422	208	26	120000	1.131	0.12902	BCO2	76353	25kb
ENSG00000150787	11	112072088	112165678	237	15	120000	1.8197	0.0344	PTS	76353	25kb
ENSG00000188771	11	112093876	112156583	177	9	120000	1.7538	0.039735	PLET1	76353	25kb
ENSG00000268472	11	112105988	112157117	159	8	120000	1.7534	0.039767	AP002884.2	76353	25kb
ENSG00000149294	11	112806997	113174158	1065	65	120000	0.48646	0.31332	NCAM1	76353	25kb
ENSG00000149292	11	113160251	113279266	274	27	120000	-0.6425	0.73972	TTC12	76353	25kb
ENSG00000170209	11	113233513	113296140	172	17	120000	-0.64737	0.7413	ANKK1	76353	25kb
ENSG00000149295	11	113255318	113371413	315	31	120000	-1.5057	0.93393	DRD2	76353	25kb
ENSG00000166682	11	113533272	113602095	268	20	120000	-0.81532	0.79255	TMPRSS5	76353	25kb
ENSG00000086827	11	113578909	113669533	298	24	120000	-0.5977	0.72498	ZW10	76353	25kb
ENSG00000228607	11	113625469	113676222	150	19	120000	-0.48101	0.68474	CLDN25	76353	25kb
ENSG00000048028	11	113643596	113771292	275	20	120000	-1.0122	0.84427	USP28	76353	25kb
ENSG00000149305	11	113750399	113842287	252	27	120000	-0.61602	0.73106	HTR3B	76353	25kb
ENSG00000166736	11	113820603	113886035	192	37	120000	-1.8591	0.96849	HTR3A	76353	25kb
ENSG00000109906	11	113905315	114146398	729	103	120000	-2.2514	0.98782	ZBTB16	76353	25kb
ENSG00000166741	11	114103509	114209007	293	47	120000	-0.58423	0.72047	NNMT	76353	25kb
ENSG00000180425	11	114237165	114296139	123	19	120000	-0.53264	0.70286	C11orf71	76353	25kb
ENSG00000076053	11	114245752	114309925	166	21	120000	-0.263	0.60372	RBM7	76353	25kb
ENSG00000255663	11	114246404	114339654	260	20	120000	0.031848	0.4873	RP11-212D19.4	76353	25kb
ENSG00000076043	11	114285108	114346001	209	21	120000	0.28221	0.38889	REXO2	76353	25kb
ENSG00000095110	11	114367437	114455617	190	12	120000	-0.42078	0.66304	NXPE1	76353	25kb
ENSG00000137634	11	114416313	114491484	214	27	120000	0.26741	0.39458	NXPE4	76353	25kb
ENSG00000204361	11	114524108	114604362	244	30	120000	1.3851	0.083005	NXPE2	76353	25kb
ENSG00000182985	11	115014938	115400675	707	63	120000	0.09456	0.46233	CADM1	76353	25kb
ENSG00000137656	11	116593886	116668704	228	29	120000	1.3765	0.08433	BUD13	76353	25kb
ENSG00000109917	11	116623436	116683766	189	33	120000	1.7995	0.035971	ZNF259	76353	25kb
ENSG00000110243	11	116635083	116688136	159	32	120000	1.9462	0.025815	APOA5	76353	25kb
ENSG00000110244	11	116666419	116719022	182	28	120000	0.21984	0.413	APOA4	76353	25kb
ENSG00000110245	11	116675422	116728788	191	29	120000	-0.17711	0.57029	APOC3	76353	25kb

ENSG00000118137	11	116681467	116733666	186	29	120000	-0.53468	0.70357	APOA1	76353	25kb
ENSG00000160584	11	116689118	116994153	1076	32	120000	-0.66643	0.74743	SIK3	76353	25kb
ENSG00000168092	11	116989983	117072610	267	19	120000	-0.62916	0.73538	PAFAH1B2	76353	25kb
ENSG00000149577	11	117024449	117093160	208	24	120000	-0.88109	0.81087	SIDT2	76353	25kb
ENSG00000149591	11	117045037	117100498	171	22	120000	-0.78167	0.7828	TAGLN	76353	25kb
ENSG00000160613	11	117050053	117128241	226	35	120000	-0.8912	0.81359	PCSK7	76353	25kb
ENSG00000167257	11	117078341	117182161	259	29	120000	-0.90087	0.81617	RNF214	76353	25kb
ENSG00000186318	11	117131402	117211975	179	30	120000	-0.35224	0.63767	BACE1	76353	25kb
ENSG00000110274	11	117160273	117308984	393	39	120000	0.2835	0.3884	CEP164	76353	25kb
ENSG00000177103	11	117273489	117713240	1555	142	120000	-1.0007	0.84151	DSCAML1	76353	25kb
ENSG00000137731	11	117646559	117724413	240	47	120000	-0.93939	0.82623	FXYD2	76353	25kb
ENSG00000255245	11	117665878	117772382	339	61	120000	-0.26457	0.60433	FXYD6-FXYD2	76353	25kb
ENSG00000137726	11	117682693	117773201	292	55	120000	0.19278	0.42356	FXYD6	76353	25kb
ENSG00000137747	11	117746358	117825174	274	39	120000	-0.0091301	0.50364	TMPRSS13	76353	25kb
ENSG00000110324	11	117832063	117897196	221	18	120000	0.024472	0.49024	IL10RA	76353	25kb
ENSG00000137648	11	117922753	118017605	344	28	120000	-1.2522	0.89475	TMPRSS4	76353	25kb
ENSG00000177098	11	117979092	118048603	207	33	120000	-0.90698	0.81779	SCN4B	76353	25kb
ENSG00000149575	11	118007666	118072388	202	30	120000	0.027732	0.48894	SCN2B	76353	25kb
ENSG00000160593	11	118039455	118120809	257	16	120000	0.47319	0.31804	AMICA1	76353	25kb
ENSG00000160588	11	118072409	118148065	233	18	120000	0.50669	0.30619	MPZL3	76353	25kb
ENSG00000149573	11	118099118	118160251	221	20	120000	0.15977	0.43653	MPZL2	76353	25kb
ENSG00000198851	11	118150260	118211890	169	14	120000	-0.68544	0.75347	CD3E	76353	25kb
ENSG00000167286	11	118184669	118238459	102	18	120000	-0.68864	0.75448	CD3D	76353	25kb
ENSG00000160654	11	118190059	118250876	103	16	120000	-0.47475	0.68252	CD3G	76353	25kb
ENSG00000110344	11	118205300	118294926	147	18	120000	-0.017038	0.5068	UBE4A	76353	25kb
ENSG00000254873	11	118242810	118297610	83	12	120000	0.38846	0.34884	RP11-770J1.5	76353	25kb
ENSG00000167283	11	118246869	118327211	106	15	120000	0.52301	0.30048	ATP5L	76353	25kb
ENSG00000255384	11	118278836	118330921	57	10	120000	0.69745	0.24276	RP11-770J1.4	76353	25kb
ENSG00000118058	11	118282205	118422539	186	23	120000	1.8623	0.031283	KMT2A	76353	25kb
ENSG00000172425	11	118373187	118426912	94	10	120000	1.8824	0.029893	TTC36	76353	25kb
ENSG00000149582	11	118376756	118442995	121	11	120000	1.7825	0.03733	TMEM25	76353	25kb
ENSG00000118096	11	118390243	118468685	156	10	120000	1.6104	0.053659	IFT46	76353	25kb
ENSG00000095139	11	118418105	118498748	142	13	120000	1.5002	0.066783	ARCN1	76353	25kb
ENSG00000019144	11	118452155	118553741	177	21	120000	0.68793	0.24575	PHLDB1	76353	25kb
ENSG00000118094	11	118503026	118575399	213	8	120000	0.49625	0.30986	TREH	76353	25kb
ENSG00000110367	11	118595034	118686858	215	17	120000	-0.48543	0.68631	DDX6	76353	25kb
ENSG00000160683	11	118729475	118793508	124	33	120000	-0.33676	0.63185	CXCR5	76353	25kb
ENSG00000186174	11	118739584	118821317	184	43	120000	-0.49828	0.69086	BCL9L	76353	25kb
ENSG00000110375	11	118770873	118854269	188	29	120000	-0.40902	0.65874	UPK2	76353	25kb
ENSG00000176302	11	118817417	118877001	149	14	120000	-0.14703	0.55845	FOXR1	76353	25kb
ENSG00000186166	11	118843852	118911501	180	17	120000	0.081111	0.46768	CCDC84	76353	25kb
ENSG00000118181	11	118861422	118914401	141	15	120000	0.19723	0.42182	RPS25	76353	25kb
ENSG00000196655	11	118864142	118921164	140	16	120000	0.26718	0.39466	TRAPPC4	76353	25kb
ENSG00000137700	11	118869824	118926616	127	15	120000	0.37082	0.35539	SLC37A4	76353	25kb
ENSG00000149428	11	118889900	118952913	138	17	120000	0.13431	0.44658	HYOU1	76353	25kb
ENSG00000160695	11	118913403	118977688	134	16	120000	-0.012225	0.50488	VPS11	76353	25kb
ENSG00000256269	11	118930576	118989259	137	18	120000	0.0042581	0.4983	HMBS	76353	25kb
ENSG00000188486	11	118939564	118991177	118	18	120000	0.091863	0.4634	H2AFX	76353	25kb
ENSG00000172269	11	118942213	119004041	139	18	120000	0.15545	0.43823	DPAGT1	76353	25kb
ENSG00000172375	11	118947908	119014252	144	17	120000	0.28687	0.38711	C2CD2L	76353	25kb
ENSG00000172273	11	118967297	119031752	143	14	120000	0.70772	0.23956	HINFP	76353	25kb
ENSG00000172350	11	118994722	119058360	103	12	120000	1.0366	0.14995	ABCG4	76353	25kb
ENSG00000268192	11	119002590	119052930	82	11	120000	1.1682	0.12136	AP002956.1	76353	25kb
ENSG00000160703	11	119012277	119079725	98	15	120000	0.90747	0.18208	NLRX1	76353	25kb
ENSG00000172367	11	119031166	119085932	66	13	120000	-0.040586	0.51619	PDZD3	76353	25kb
ENSG00000248712	11	119035347	119092479	76	12	120000	-0.3139	0.6232	CCDC153	76353	25kb
ENSG00000110395	11	119051752	119203859	196	23	120000	-0.84748	0.80164	CBL	76353	25kb
ENSG00000076706	11	119154241	119217231	85	24	120000	-0.67845	0.75126	MCAM	76353	25kb
ENSG00000173456	11	119180237	119233023	97	17	120000	1.0736	0.14149	RNF26	76353	25kb
ENSG00000259159	11	119184652	119242368	121	20	120000	1.5663	0.058634	MFRP	76353	25kb
ENSG00000235718	11	119184652	119242383	121	20	120000	1.5663	0.058634	MFRP	76353	25kb
ENSG00000223953	11	119184652	119242383	121	20	120000	1.5663	0.058634	C1QTNF5	76353	25kb
ENSG00000036672	11	119200925	119277436	148	23	120000	1.5666	0.058602	USP2	76353	25kb
ENSG00000154096	11	119263090	119320695	125	9	120000	-0.83495	0.79813	THY1	76353	25kb
ENSG00000110400	11	119469120	119624794	381	42	120000	-0.032977	0.51315	PVRL1	76353	25kb
ENSG00000137699	11	119956983	120081237	368	41	120000	-1.4191	0.92206	TRIM29	76353	25kb
ENSG00000176984	11	120014685	120067387	128	20	120000	-0.85476	0.80366	AP000679.2	76353	25kb
ENSG00000184232	11	120056475	120126041	213	35	120000	-0.82446	0.79516	OAF	76353	25kb
ENSG00000137709	11	120082349	120215653	338	39	120000	-0.08546	0.53405	POU2F3	76353	25kb
ENSG00000181264	11	120170838	120229391	98	15	120000	-0.39247	0.65264	TMEM136	76353	25kb
ENSG00000196914	11	120182787	120385645	364	26	120000	-1.0312	0.84878	ARHGEF12	76353	25kb
ENSG00000269601	11	120304880	120355047	91	12	120000	-0.60759	0.72827	AP000758.1	76353	25kb
ENSG00000149403	11	120357468	120884613	1506	158	120000	0.55211	0.29044	GRIK4	76353	25kb
ENSG00000204306	11	120357511	120410732	146	36	120000	-1.7499	0.95993	AP002348.1	76353	25kb
ENSG00000154114	11	120869781	120986484	188	26	120000	0.39321	0.34708	TBCEL	76353	25kb
ENSG00000109927	11	120946882	121087202	365	26	120000	-0.25919	0.60226	TECTA	76353	25kb
ENSG00000109929	11	121138162	121204403	166	25	120000	-0.86621	0.80681	SC5D	76353	25kb
ENSG00000137642	11	121297912	121529402	451	68	120000	-0.62289	0.73332	SORL1	76353	25kb
ENSG00000259571	11	121961062	122011923	129	22	120000	1.6372	0.050791	BLID	76353	25kb
ENSG00000154127	11	122501383	122710181	679	83	120000	-0.6685	0.74809	UBASH3B	76353	25kb
ENSG00000109943	11	122684208	122768347	283	36	120000	-0.19859	0.57871	CRTAM	76353	25kb
ENSG00000109944	11	122728391	122855506	422	45	120000	-0.96142	0.83183	C11orf63	76353	25kb
ENSG00000188909	11	122823278	122877428	161	14	120000	-0.29804	0.61717	BSX	76353	25kb
ENSG00000109971	11	122903197	122958938	209	22	120000	1.0618	0.14416	HSPA8	76353	25kb
ENSG00000166250	11	122918035	123090989	694	65	120000	0.044768	0.48215	CLMP	76353	25kb
ENSG00000254667	11	123276058	123331427	172	47	120000	0.26829	0.39424	AP000783.1	76353	25kb
ENSG00000023171	11	123371344	123523482	523	77	120000	0.91345	0.1805	GRAMD1B	76353	25kb
ENSG00000166257	11	123474895	123550952	347	47	120000	1.8879	0.029523	SCN3B	76353	25kb
ENSG00000166261	11	123569885	123637383	184	20	120000	-0.26878	0.60595	ZNF202	76353	25kb
ENSG00000221931	11	123599196	123650253	168	16	120000	-1.0955	0.86334	OR6X1	76353	25kb
ENSG00000196099	11	123651043	123702095	180	29	120000	0.018027	0.49281	OR6M1	76353	25kb
ENSG00000204300	11	123728633	123781349	200	25	120000	-0.51017	0.69503	TMEM225	76353	25kb
ENSG00000181518	11	123752109	123803183	190	23	120000	-0.030841	0.5123	OR8D4	76353	25kb
ENSG00000171014	11	123785250	123836344	185	18	120000	0.23119	0.40858	OR4D5	76353	25kb
ENSG00000181499	11	123788492	123839580	182	18	120000	0.23435	0.40736	OR6T1	76353	25kb
ENSG00000196248	11	123822368	123873488	170	21	120000	0.087352	0.4652	OR10S1	76353	25kb

ENSG00000198674	11	123839870	123890868	180	23	120000	-0.098419	0.5392	OR10G6	76353	25kb
ENSG00000254737	11	123861282	123912217	162	21	120000	-0.82006	0.79391	OR10G4	76353	25kb
ENSG00000236981	11	123868720	123919655	143	19	120000	-1.1007	0.86449	OR10G9	76353	25kb
ENSG00000234560	11	123875297	123926341	139	20	120000	-1.1104	0.86658	OR10G8	76353	25kb
ENSG00000182634	11	123883773	123934717	156	22	120000	-1.5812	0.94309	OR10G7	76353	25kb
ENSG00000110002	11	123961069	124043428	254	33	120000	0.40273	0.34357	VWA5A	76353	25kb
ENSG00000197309	11	124030923	124081952	212	16	120000	-0.17716	0.57031	OR10D3	76353	25kb
ENSG00000255298	11	124109723	124160763	306	13	120000	0.1025	0.45918	OR8G5	76353	25kb
ENSG00000196341	11	124154708	124205733	147	14	120000	-0.46098	0.67759	OR8D1	76353	25kb
ENSG00000197263	11	124164134	124215184	151	14	120000	-0.65252	0.74297	OR8D2	76353	25kb
ENSG00000204293	11	124227291	124278258	151	11	120000	-0.93041	0.82392	OR8B2	76353	25kb
ENSG00000196661	11	124241278	124292264	165	13	120000	-1.0275	0.84792	OR8B3	76353	25kb
ENSG00000198657	11	124268807	124319789	184	20	120000	-0.63073	0.73589	OR8B4	76353	25kb
ENSG00000197125	11	124285022	124336054	210	22	120000	-0.7776	0.7816	OR8B8	76353	25kb
ENSG00000170953	11	124387578	124438575	182	22	120000	1.7166	0.043027	OR8B12	76353	25kb
ENSG00000196119	11	124414893	124466037	179	25	120000	1.7493	0.040116	OR8A1	76353	25kb
ENSG00000154143	11	124456386	124515252	112	21	120000	-0.63939	0.73872	PANX3	76353	25kb
ENSG00000154144	11	124467732	124530287	106	18	120000	-0.84644	0.80135	TBRG1	76353	25kb
ENSG00000110013	11	124478009	124590603	185	21	120000	-0.77161	0.77983	SIAE	76353	25kb
ENSG00000064199	11	124518694	124592414	117	13	120000	-0.74009	0.77038	SPA17	76353	25kb
ENSG00000154146	11	124584742	124642106	103	24	120000	-0.98217	0.83699	NRGN	76353	25kb
ENSG00000019102	11	124592368	124647134	96	21	120000	-1.2789	0.89954	VSIG2	76353	25kb
ENSG00000149564	11	124597026	124657186	98	20	120000	-1.3127	0.90536	ESAM	76353	25kb
ENSG00000120458	11	124611394	124695569	218	33	120000	-1.1714	0.87928	MSANTD2	76353	25kb
ENSG00000154134	11	124710282	124776366	217	42	120000	-1.025	0.84732	ROBO3	76353	25kb
ENSG00000154133	11	124728587	124793396	191	36	120000	-1.4337	0.92417	ROBO4	76353	25kb
ENSG00000254943	11	124736200	124787022	155	28	120000	-1.3566	0.91254	RP11-664I21.6	76353	25kb
ENSG00000165478	11	124764089	124831308	194	42	120000	0.034936	0.48607	HEPACAM	76353	25kb
ENSG00000221932	11	124764146	124815573	145	34	120000	-0.51867	0.698	HEPN1	76353	25kb
ENSG00000149548	11	124799017	124936385	387	25	120000	1.4694	0.070864	CCDC15	76353	25kb
ENSG00000134955	11	124907963	124984131	293	40	120000	0.21357	0.41544	SLC37A2	76353	25kb
ENSG00000150433	11	124941398	125006659	274	38	120000	-0.14575	0.55794	TMEM218	76353	25kb
ENSG00000165495	11	125009583	125328285	924	98	120000	0.45794	0.3235	PKNOX2	76353	25kb
ENSG00000149557	11	125290646	125391213	242	31	120000	-0.22795	0.59016	FEZ1	76353	25kb
ENSG00000255537	11	125340110	125394424	148	21	120000	-0.40834	0.65849	AP000708.1	76353	25kb
ENSG00000149547	11	125414112	125479575	198	21	120000	-0.15319	0.56087	EI24	76353	25kb
ENSG00000134910	11	125436607	125520110	253	15	120000	-0.15706	0.5624	STT3A	76353	25kb
ENSG00000149554	11	125470036	125571150	300	17	120000	-0.48177	0.68502	CHEK1	76353	25kb
ENSG00000134940	11	125516417	125576018	162	19	120000	-1.2325	0.89112	ACRV1	76353	25kb
ENSG00000171053	11	125591188	125644762	232	20	120000	-1.4715	0.92942	PATE1	76353	25kb
ENSG00000196844	11	125621008	125673714	183	16	120000	-1.0072	0.84307	PATE2	76353	25kb
ENSG00000236027	11	125633006	125686495	167	16	120000	-0.91663	0.82033	PATE3	76353	25kb
ENSG00000237353	11	125678211	125734964	193	15	120000	-0.86435	0.8063	PATE4	76353	25kb
ENSG00000198331	11	125728509	125795543	196	23	120000	-0.41583	0.66123	HYLS1	76353	25kb
ENSG00000110060	11	125738381	125798116	178	22	120000	-0.22755	0.59	PUS3	76353	25kb
ENSG00000109832	11	125748271	125818158	214	31	120000	-0.21975	0.58697	DDX25	76353	25kb
ENSG00000064309	11	125800691	125958230	542	52	120000	-0.90582	0.81748	CDON	76353	25kb
ENSG00000165526	11	126046993	126106587	205	26	120000	0.57899	0.2813	RPUSD4	76353	25kb
ENSG00000197798	11	126056309	126157881	261	22	120000	-0.42321	0.66393	FAM118B	76353	25kb
ENSG00000182934	11	126107814	126164039	127	19	120000	-0.17792	0.57061	SRPR	76353	25kb
ENSG00000110074	11	126113950	126173026	138	20	120000	-0.33229	0.63016	FOXRED1	76353	25kb
ENSG00000150455	11	126127960	126193740	181	23	120000	-0.83796	0.79897	TIRAP	76353	25kb
ENSG00000255062	11	126139193	126199213	170	23	120000	-1.0329	0.84918	RP11-712L6.5	76353	25kb
ENSG00000110063	11	126148647	126240644	251	34	120000	-1.4278	0.92332	DCPS	76353	25kb
ENSG00000110080	11	126200535	126335239	374	62	120000	-1.3527	0.91193	ST3GAL4	76353	25kb
ENSG00000149571	11	126268254	126898355	2184	184	120000	-0.27965	0.61013	KIRREL3	76353	25kb
ENSG00000134954	11	128303656	128482453	404	59	120000	0.29305	0.38474	ETS1	76353	25kb
ENSG00000151702	11	128531430	128708162	559	67	120000	1.5479	0.060822	FLI1	76353	25kb
ENSG00000151704	11	128681210	128762268	261	36	120000	1.4488	0.073697	KCNJ1	76353	25kb
ENSG00000120457	11	128736251	128815930	263	47	120000	-1.2973	0.90274	KCNJ5	76353	25kb
ENSG00000174370	11	128744460	128800930	194	36	120000	-1.0919	0.86256	C11orf45	76353	25kb
ENSG00000120471	11	128779626	128838040	201	35	120000	-1.2016	0.88523	TP53AIP1	76353	25kb
ENSG00000134909	11	128809955	129174219	781	25	120000	-1.1919	0.88334	ARHGAP32	76353	25kb
ENSG00000043039	11	129220835	129347171	408	65	120000	1.0205	0.15374	BARX2	76353	25kb
ENSG00000151715	11	129660714	129754898	249	31	120000	-0.71131	0.76155	TMEM45B	76353	25kb
ENSG00000170322	11	129708531	129790485	186	26	120000	-1.3378	0.90951	NFRKB	76353	25kb
ENSG00000170325	11	129744601	129897730	430	45	120000	-1.0483	0.85276	PRDM10	76353	25kb
ENSG00000267981	11	129911836	129969012	193	12	120000	0.77734	0.21848	AP003041.2	76353	25kb
ENSG00000084234	11	129914732	130039699	346	21	120000	0.27604	0.39126	APLP2	76353	25kb
ENSG00000149418	11	130004457	130105271	274	40	120000	-0.55263	0.70974	ST14	76353	25kb
ENSG00000196323	11	130071572	130209581	391	22	120000	1.9266	0.027013	ZBTB44	76353	25kb
ENSG00000134917	11	130249820	130323888	223	45	120000	0.80341	0.21087	ADAMTS8	76353	25kb
ENSG00000166106	11	130293869	130371532	241	51	120000	0.73847	0.23011	ADAMTS15	76353	25kb
ENSG00000175728	11	130517851	130612247	209	40	120000	-1.0395	0.8507	C11orf44	76353	25kb
ENSG00000120451	11	130720331	130811404	371	18	120000	0.81361	0.20793	SNX19	76353	25kb
ENSG00000182667	11	131215373	132231716	3161	269	120000	0.055952	0.47769	NTM	76353	25kb
ENSG00000183715	11	132259871	133427414	3908	296	120000	-1.3713	0.91486	OPCML	76353	25kb
ENSG00000166118	11	133685526	133740433	197	22	120000	-0.095985	0.53823	SPATA19	76353	25kb
ENSG00000080854	11	133753459	133851880	288	35	120000	-0.20428	0.58093	IGSF9B	76353	25kb
ENSG00000166086	11	133913820	134046896	381	23	120000	0.02835	0.48869	JAM3	76353	25kb
ENSG00000151503	11	133995014	134120348	255	24	120000	0.040308	0.48392	NCAPD3	76353	25kb
ENSG00000151502	11	134069539	134142686	129	23	120000	-0.82052	0.79404	VPS26B	76353	25kb
ENSG00000151500	11	134093173	134148264	109	19	120000	-1.0813	0.86023	THYN1	76353	25kb
ENSG00000151498	11	134098389	134160749	144	24	120000	-1.4184	0.92196	ACAD8	76353	25kb
ENSG00000166105	11	134119139	134214458	237	41	120000	-2.1351	0.98362	GLB1L3	76353	25kb
ENSG00000149328	11	134176768	134273235	298	62	120000	0.38497	0.35013	GLB1L2	76353	25kb
ENSG00000109956	11	134223398	134306812	363	42	120000	0.57042	0.2842	B3GAT1	76353	25kb
ENSG00000268936	11	134830246	134881693	300	19	120000	-0.14196	0.55645	AP003062.1	76353	25kb
ENSG00000268640	12	122052	172377	6	2	120000	0.023853	0.49048	AC026369.1	76353	25kb
ENSG00000120645	12	150931	312626	525	67	120000	-1.6802	0.95354	IQSEC3	76353	25kb
ENSG00000111181	12	274243	348736	305	44	120000	-0.63606	0.73763	SLC6A12	76353	25kb
ENSG00000010379	12	304789	397039	291	42	120000	0.058721	0.47659	SLC6A13	76353	25kb
ENSG00000073614	12	364295	523620	415	21	120000	0.20245	0.41978	KDM5A	76353	25kb
ENSG00000120647	12	473439	576811	290	34	120000	-0.24777	0.59785	CCDC77	76353	25kb
ENSG00000139044	12	544530	697675	650	93	120000	-1.2406	0.89262	B4GALNT3	76353	25kb
ENSG00000171840	12	648462	797945	605	51	120000	-0.64649	0.74102	NINJ2	76353	25kb

ENSG00000060237	12	836759	1045618	488	42	120000	-0.71667	0.76321	WNK1	76353	25kb
ENSG00000002016	12	996243	1124219	344	50	120000	-1.9068	0.97173	RAD52	76353	25kb
ENSG00000082805	12	1074675	1630099	1891	64	120000	-0.27613	0.60878	ERC1	76353	25kb
ENSG00000111186	12	1614057	1781409	715	135	120000	-0.55306	0.70989	WNT5B	76353	25kb
ENSG00000171823	12	1650159	1728331	266	61	120000	-1.1854	0.88206	FBXL14	76353	25kb
ENSG00000006831	12	1772740	1922844	440	29	120000	0.93988	0.17364	ADIPOR2	76353	25kb
ENSG00000151062	12	1876123	2053002	606	69	120000	1.0714	0.14198	CACNA2D4	76353	25kb
ENSG00000166159	12	1904433	1970918	240	52	120000	1.1253	0.13022	LRTM2	76353	25kb
ENSG00000151065	12	2030220	2138701	391	25	120000	0.13286	0.44715	DCP1B	76353	25kb
ENSG00000151067	12	2054952	2827108	2203	198	120000	-0.13545	0.55387	CACNA1C	76353	25kb
ENSG00000004478	12	2879119	2939576	119	15	120000	-0.36667	0.64307	FKBP4	76353	25kb
ENSG00000111203	12	2896788	2993957	202	21	120000	-0.53718	0.70443	ITFG2	76353	25kb
ENSG00000053702	12	2909514	2969710	138	12	120000	-0.64331	0.73999	NRIP2	76353	25kb
ENSG00000206044	12	2933397	2991213	137	19	120000	-0.57018	0.71572	AC005841.1	76353	25kb
ENSG00000111206	12	2941847	3011206	192	20	120000	-0.79768	0.78747	FOXM1	76353	25kb
ENSG00000171792	12	2960424	3023626	190	12	120000	-0.65432	0.74355	RHNO1	76353	25kb
ENSG00000078246	12	2961389	3075306	326	13	120000	-0.88865	0.81291	TULP3	76353	25kb
ENSG00000197905	12	3043496	3174839	514	47	120000	-0.24928	0.59843	TEAD4	76353	25kb
ENSG00000011105	12	3161521	3420730	1158	117	120000	-0.079995	0.53188	TSPAN9	76353	25kb
ENSG00000111218	12	3465515	3728139	682	109	120000	-1.5688	0.94166	PRMT8	76353	25kb
ENSG00000130038	12	3690799	3898985	792	87	120000	0.97177	0.16558	EFCAB4B	76353	25kb
ENSG00000111224	12	3875213	4007608	422	35	120000	-0.18863	0.57481	PARP11	76353	25kb
ENSG00000118971	12	4357938	4439516	199	63	120000	0.51533	0.30316	CCND2	76353	25kb
ENSG00000078237	12	4405371	4487338	220	42	120000	0.65344	0.25674	C12orf5	76353	25kb
ENSG00000118972	12	4452393	4513894	170	30	120000	0.93891	0.17389	FGF23	76353	25kb
ENSG00000111241	12	4512321	4579780	203	28	120000	-0.77653	0.78128	FGF6	76353	25kb
ENSG00000047621	12	4571894	4672674	149	16	120000	0.27626	0.39117	C12orf4	76353	25kb
ENSG00000111247	12	4622950	4694214	145	23	120000	0.72098	0.23546	RAD51AP1	76353	25kb
ENSG00000010219	12	4646370	4748325	325	32	120000	0.24248	0.4042	DYRK4	76353	25kb
ENSG00000272921	12	4689110	4796809	368	30	120000	0.61446	0.26945	RP11-500M8.7	76353	25kb
ENSG00000111254	12	4699674	4783213	305	23	120000	-0.0098696	0.50394	AKAP3	76353	25kb
ENSG00000139180	12	4733261	4823454	339	32	120000	2.2862	0.011121	NDUFA9	76353	25kb
ENSG00000255639	12	4741800	4878747	425	32	120000	1.8664	0.030989	RP11-234B24.6	76353	25kb
ENSG00000130035	12	4804507	4985277	595	50	120000	1.6795	0.046527	GALNT8	76353	25kb
ENSG00000151079	12	4893342	4985277	358	42	120000	0.69797	0.2426	KCNA6	76353	25kb
ENSG00000111262	12	4994071	5065527	206	38	120000	0.62756	0.26515	KCNA1	76353	25kb
ENSG00000130037	12	5128085	5180949	184	37	120000	0.39584	0.34611	KCNA5	76353	25kb
ENSG00000185652	12	5516278	5655702	437	65	120000	1.3714	0.085124	NTF3	76353	25kb
ENSG00000047617	12	5616035	6080398	1585	125	120000	1.3774	0.084195	ANO2	76353	25kb
ENSG00000110799	12	6033040	6258936	780	65	120000	1.5245	0.063693	VWF	76353	25kb
ENSG00000010278	12	6283881	6372425	205	22	120000	0.20104	0.42033	CD9	76353	25kb
ENSG00000008323	12	6394602	6462672	196	34	120000	-0.090954	0.53624	PLEKHG6	76353	25kb
ENSG00000067182	12	6412923	6476280	160	44	120000	-1.3124	0.9053	TNFRSF1A	76353	25kb
ENSG00000111319	12	6431009	6511896	223	60	120000	-0.44365	0.67135	SCNN1A	76353	25kb
ENSG00000111321	12	6459211	6525733	195	47	120000	0.31435	0.37663	LTBR	76353	25kb
ENSG00000139193	12	6529033	6585884	199	32	120000	-0.39914	0.6551	CD27	76353	25kb
ENSG00000139192	12	6535856	6600683	237	29	120000	-0.66885	0.7482	TAPBPL	76353	25kb
ENSG00000139190	12	6546403	6605153	222	21	120000	-0.68504	0.75334	VAMP1	76353	25kb
ENSG00000111639	12	6576150	6628007	212	17	120000	-0.74044	0.77048	MRPL51	76353	25kb
ENSG00000010292	12	6577522	6666121	305	20	120000	-1.179	0.8808	NCAPD2	76353	25kb
ENSG00000111640	12	6618093	6672537	144	21	120000	-1.5154	0.93516	GAPDH	76353	25kb
ENSG00000010295	12	6622541	6690239	157	24	120000	-1.5442	0.93873	IFFO1	76353	25kb
ENSG00000111641	12	6641029	6702857	125	18	120000	-1.4696	0.92917	NOP2	76353	25kb
ENSG00000111642	12	6654249	6741642	146	16	120000	-0.72806	0.76671	CHD4	76353	25kb
ENSG00000184574	12	6703001	6770613	120	20	120000	-0.56558	0.71416	LPAR5	76353	25kb
ENSG00000111644	12	6722241	6781626	92	18	120000	-0.36658	0.64303	ACRBP	76353	25kb
ENSG00000111653	12	6734446	6797314	89	17	120000	-0.1054	0.54197	ING4	76353	25kb
ENSG00000126746	12	6750643	6823738	111	23	120000	0.8275	0.20398	ZNF384	76353	25kb
ENSG00000139200	12	6777958	6834966	93	20	120000	1.6384	0.050669	PIANP	76353	25kb
ENSG00000111652	12	6807907	6866041	202	26	120000	0.78126	0.21733	COPS7A	76353	25kb
ENSG00000089693	12	6832170	6901641	223	30	120000	0.63778	0.26181	MLF2	76353	25kb
ENSG00000159335	12	6849682	6905116	162	28	120000	0.99934	0.15881	PTMS	76353	25kb
ENSG00000089692	12	6856678	6912621	147	29	120000	1.7043	0.044162	LAG3	76353	25kb
ENSG00000010610	12	6871024	6954974	225	48	120000	1.5131	0.065127	CD4	76353	25kb
ENSG00000250510	12	6905711	6964136	149	33	120000	0.67704	0.24919	GPR162	76353	25kb
ENSG00000111664	12	6924118	6981557	110	30	120000	-0.37234	0.64518	GNB3	76353	25kb
ENSG00000111665	12	6928957	6986230	100	26	120000	-0.28738	0.61309	CDCA3	76353	25kb
ENSG00000111667	12	6936292	7000796	106	16	120000	-0.56265	0.71316	USP5	76353	25kb
ENSG00000111669	12	6951283	7005112	74	6	120000	-0.45691	0.67613	TPI1	76353	25kb
ENSG00000111671	12	6955099	7023522	119	6	120000	-0.57685	0.71798	SPSB2	76353	25kb
ENSG00000010626	12	6957733	7048407	145	8	120000	-0.50448	0.69304	LRRC23	76353	25kb
ENSG00000111674	12	6997909	7057861	112	8	120000	-0.3559	0.63904	ENO2	76353	25kb
ENSG00000111676	12	7008626	7076484	111	15	120000	-0.52207	0.69919	ATN1	76353	25kb
ENSG00000111678	12	7027141	7080166	74	23	120000	-0.39118	0.65217	C12orf57	76353	25kb
ENSG00000111679	12	7030631	7095479	88	25	120000	-0.31756	0.62459	PTPN6	76353	25kb
ENSG00000126749	12	7047408	7130520	109	17	120000	-0.41846	0.66219	EMG1	76353	25kb
ENSG00000215021	12	7049490	7104988	75	18	120000	-0.21761	0.58613	PHB2	76353	25kb
ENSG00000111684	12	7060348	7150814	127	11	120000	-0.47058	0.68103	LPCAT3	76353	25kb
ENSG00000182326	12	7071351	7203336	178	17	120000	-0.5012	0.69188	C1S	76353	25kb
ENSG00000159403	12	7162513	7270203	125	24	120000	-0.45139	0.67415	C1R	76353	25kb
ENSG00000139178	12	7217183	7286869	130	23	120000	-0.22675	0.58969	C1RL	76353	25kb
ENSG00000139194	12	7251280	7306538	126	20	120000	-0.20351	0.58063	RBP5	76353	25kb
ENSG00000139182	12	7257294	7336541	178	26	120000	-0.065047	0.52593	CLSTN3	76353	25kb
ENSG00000139197	12	7316281	7396170	222	29	120000	1.3216	0.093148	PEX5	76353	25kb
ENSG00000215009	12	7431880	7506320	154	16	120000	0.98955	0.1612	ACSM4	76353	25kb
ENSG00000177675	12	7474281	7657493	377	47	120000	1.1544	0.12417	CD163L1	76353	25kb
ENSG00000177575	12	7598409	7681489	193	35	120000	1.8385	0.032991	CD163	76353	25kb
ENSG00000111701	12	7776996	7843499	297	34	120000	0.71158	0.23836	APOBEC1	76353	25kb
ENSG00000184344	12	7817378	7873372	248	49	120000	-0.42461	0.66444	GDF3	76353	25kb
ENSG00000187569	12	7839050	7895155	245	46	120000	-0.73161	0.7678	DPPA3	76353	25kb
ENSG00000198178	12	7857011	7929201	242	32	120000	-0.40601	0.65763	CLEC4C	76353	25kb
ENSG00000205857	12	7892812	7951717	194	29	120000	-0.096095	0.53828	NANOGNB	76353	25kb
ENSG00000111704	12	7915390	7973655	235	26	120000	-0.2963	0.6165	NANOG	76353	25kb
ENSG00000173262	12	7940108	8068744	512	45	120000	0.73223	0.23201	SLC2A14	76353	25kb
ENSG00000059804	12	8046826	8113871	178	27	120000	0.60716	0.27187	SLC2A3	76353	25kb
ENSG00000065970	12	8160299	8233099	147	26	120000	0.67423	0.25008	FOXJ2	76353	25kb

ENSG00000171860	12	8185898	8244067	109	24	120000	1.2214	0.11096	C3AR1	76353	25kb
ENSG00000089818	12	8209807	8275367	113	23	120000	1.0081	0.1567	NECAP1	76353	25kb
ENSG00000111729	12	8251228	8316203	145	22	120000	-0.022136	0.50883	CLEC4A	76353	25kb
ENSG00000196946	12	8265733	8366133	164	19	120000	0.0019683	0.49921	ZNF705A	76353	25kb
ENSG00000171847	12	8348856	8405214	117	28	120000	0.17169	0.43184	FAM90A1	76353	25kb
ENSG00000205846	12	8583522	8655926	206	7	120000	-0.65185	0.74275	CLEC6A	76353	25kb
ENSG00000166527	12	8637071	8699962	147	19	120000	-0.26856	0.60587	CLEC4D	76353	25kb
ENSG00000166523	12	8660901	8718559	187	22	120000	-0.55015	0.70889	CLEC4E	76353	25kb
ENSG00000111732	12	8729762	8790467	147	26	120000	-1.4881	0.93164	AICDA	76353	25kb
ENSG00000197614	12	8764942	8840484	153	14	120000	0.54052	0.29442	MFAP5	76353	25kb
ENSG00000166532	12	8809196	8960691	170	31	120000	0.50918	0.30531	RIMKLB	76353	25kb
ENSG00000166535	12	8950068	9064597	326	37	120000	-1.8306	0.96642	A2ML1	76353	25kb
ENSG00000111752	12	9041492	9119063	214	20	120000	-0.33679	0.63186	PHC1	76353	25kb
ENSG00000003056	12	9067959	9127551	142	14	120000	0.0001347	0.49995	M6PR	76353	25kb
ENSG00000139187	12	9077640	9188356	277	16	120000	-0.69884	0.75767	KLRG1	76353	25kb
ENSG00000175899	12	9195260	9293825	200	15	120000	-0.37231	0.64517	A2M	76353	25kb
ENSG00000126838	12	9276436	9385966	274	26	120000	-1.7558	0.96044	PZP	76353	25kb
ENSG00000111796	12	9722147	9785482	194	25	120000	0.86782	0.19275	KLRB1	76353	25kb
ENSG00000069493	12	9792565	9873413	363	25	120000	3.143	0.00083618	CLEC2D	76353	25kb
ENSG00000184293	12	9843456	9910895	298	19	120000	2.058	0.019796	CLECL1	76353	25kb
ENSG00000110848	12	9880082	9938497	235	23	120000	1.3538	0.087906	CD69	76353	25kb
ENSG00000150045	12	9955077	10022606	207	35	120000	-0.32423	0.62712	KLRF1	76353	25kb
ENSG00000110852	12	9980583	10047735	225	34	120000	-0.27498	0.60833	CLEC2B	76353	25kb
ENSG00000256797	12	10009088	10073432	223	34	120000	-0.66875	0.74817	KLRF2	76353	25kb
ENSG00000188393	12	10026272	10109980	288	38	120000	-1.5405	0.93828	CLEC2A	76353	25kb
ENSG00000172322	12	10078915	10173293	401	32	120000	-1.3286	0.908	CLEC12A	76353	25kb
ENSG00000165682	12	10113241	10191023	339	30	120000	-1.2842	0.90047	CLEC1B	76353	25kb
ENSG00000256660	12	10138226	10196218	223	35	120000	-1.291	0.90165	CLEC12B	76353	25kb
ENSG00000197992	12	10158276	10243565	223	38	120000	-0.75005	0.77339	CLEC9A	76353	25kb
ENSG00000150048	12	10197153	10289226	276	18	120000	-0.4269	0.66527	CLEC1A	76353	25kb
ENSG00000172243	12	10244376	10307857	233	27	120000	-0.9989	0.84108	CLEC7A	76353	25kb
ENSG00000173391	12	10285902	10349737	250	30	120000	-1.532	0.93724	OLR1	76353	25kb
ENSG00000165685	12	10298141	10369400	332	34	120000	-1.9241	0.97283	TMEM52B	76353	25kb
ENSG00000139112	12	10340057	10400727	257	32	120000	-1.0521	0.85363	GABARAPL1	76353	25kb
ENSG00000134539	12	10353657	10494850	475	37	120000	0.082761	0.46702	KLRD1	76353	25kb
ENSG00000213809	12	10499952	10569473	202	20	120000	0.21697	0.41411	KLRK1	76353	25kb
ENSG00000255819	12	10499952	10587745	218	22	120000	0.23731	0.40621	KLRC4-KLRK1	76353	25kb
ENSG00000183542	12	10534983	10587356	95	12	120000	0.40621	0.34229	KLRC4	76353	25kb
ENSG00000205810	12	10539911	10598194	88	11	120000	0.3482	0.36385	KLRC3	76353	25kb
ENSG00000255641	12	10539911	10613600	142	14	120000	0.10357	0.45875	NKG2-E	76353	25kb
ENSG00000205809	12	10554453	10619899	130	16	120000	0.31554	0.37617	KLRC2	76353	25kb
ENSG00000134545	12	10569863	10632284	136	15	120000	0.1052	0.45811	KLRC1	76353	25kb
ENSG00000180574	12	10633201	10700734	254	17	120000	-0.68731	0.75406	EIF2S3L	76353	25kb
ENSG00000111196	12	10733612	10791222	204	22	120000	-0.038085	0.51519	MAGOHB	76353	25kb
ENSG00000060140	12	10746538	10851917	313	26	120000	-0.35794	0.6398	STYK1	76353	25kb
ENSG00000060138	12	10826683	10900911	193	30	120000	-1.3564	0.91251	YBX3	76353	25kb
ENSG00000121377	12	10929131	10980226	96	23	120000	-0.14407	0.55728	TAS2R7	76353	25kb
ENSG00000121314	12	10933650	10984892	104	22	120000	-0.099358	0.53957	TAS2R8	76353	25kb
ENSG00000121381	12	10936693	10987767	102	20	120000	-0.20825	0.58248	TAS2R9	76353	25kb
ENSG00000111215	12	10952559	11349212	1536	23	120000	-0.01658	0.50661	PRR4	76353	25kb
ENSG00000121318	12	10952916	11003957	101	16	120000	-0.38154	0.6486	TAS2R10	76353	25kb
ENSG00000231887	12	11008610	11061854	168	10	120000	-0.48341	0.6856	PRH1	76353	25kb
ENSG00000212128	12	11035525	11087161	157	11	120000	-0.49766	0.69064	TAS2R13	76353	25kb
ENSG00000134551	12	11056835	11109940	217	11	120000	-0.21	0.58317	PRH2	76353	25kb
ENSG00000212127	12	11065005	11349172	1248	22	120000	0.12075	0.45194	TAS2R14	76353	25kb
ENSG00000212126	12	11113512	11164511	266	9	120000	0.6385	0.26157	TAS2R50	76353	25kb
ENSG00000255837	12	11124094	11175474	253	10	120000	0.79007	0.21474	TAS2R20	76353	25kb
ENSG00000212124	12	11149218	11200219	250	15	120000	0.62793	0.26503	TAS2R19	76353	25kb
ENSG00000256436	12	11157986	11209006	248	16	120000	0.48785	0.31283	TAS2R31	76353	25kb
ENSG00000268301	12	11162087	11212275	240	16	120000	0.4099	0.34094	AC018630.1	76353	25kb
ENSG00000226761	12	11188964	11239893	131	15	120000	0.28774	0.38677	TAS2R46	76353	25kb
ENSG00000255374	12	11218886	11269912	158	13	120000	-0.35817	0.63989	TAS2R43	76353	25kb
ENSG00000256188	12	11260557	11312243	265	8	120000	-0.42267	0.66373	TAS2R30	76353	25kb
ENSG00000186136	12	11313599	11364543	253	18	120000	0.61283	0.27	TAS2R42	76353	25kb
ENSG00000197870	12	11393857	11447739	136	12	120000	0.5077	0.30583	PRB3	76353	25kb
ENSG00000230657	12	11435017	11488369	158	23	120000	1.4365	0.075434	PRB4	76353	25kb
ENSG00000251655	12	11479757	11573500	247	35	120000	0.91121	0.18109	PRB1	76353	25kb
ENSG00000121335	12	11519476	11678975	488	37	120000	-0.74507	0.77188	PRB2	76353	25kb
ENSG00000139083	12	11777788	12073336	842	116	120000	-0.43861	0.66953	ETV6	76353	25kb
ENSG00000121380	12	12177778	12389018	642	71	120000	0.61253	0.27009	BCL2L14	76353	25kb
ENSG00000070018	12	12243959	12444946	503	30	120000	-0.67515	0.75021	LRP6	76353	25kb
ENSG00000111261	12	12457198	12528475	293	32	120000	2.7754	0.002757	MANSC1	76353	25kb
ENSG00000205791	12	12483342	12535001	167	25	120000	2.9658	0.0015095	LOH12CR2	76353	25kb
ENSG00000165714	12	12485013	12644840	508	43	120000	1.8335	0.033363	LOH12CR1	76353	25kb
ENSG00000111266	12	12603829	12740317	358	38	120000	1.4317	0.07611	DUSP16	76353	25kb
ENSG00000111269	12	12739761	12823042	175	27	120000	1.0634	0.14381	CREBL2	76353	25kb
ENSG00000183150	12	12788825	12874141	218	39	120000	0.59462	0.27605	GPR19	76353	25kb
ENSG00000111276	12	12842992	12900305	136	25	120000	0.14476	0.44245	CDKN1B	76353	25kb
ENSG00000178878	12	12853851	13007909	505	73	120000	-1.6514	0.95068	APOLD1	76353	25kb
ENSG00000213782	12	12941250	13007915	259	28	120000	-1.3563	0.9125	DDX47	76353	25kb
ENSG00000013588	12	13018716	13095871	250	49	120000	0.149	0.44078	GPRC5A	76353	25kb
ENSG00000111291	12	13068709	13130081	177	34	120000	-0.038161	0.51522	GPRC5D	76353	25kb
ENSG00000013583	12	13102798	13178207	198	24	120000	-0.77211	0.77998	HEBP1	76353	25kb
ENSG00000084444	12	13172218	13320455	302	34	120000	-0.57324	0.71676	KIAA1467	76353	25kb
ENSG00000111305	12	13211494	13281619	110	21	120000	-0.13333	0.55303	GSG1	76353	25kb
ENSG00000134531	12	13324650	13394708	210	41	120000	-0.42394	0.6642	EMP1	76353	25kb
ENSG00000180861	12	13499023	13565101	252	20	120000	-1.2428	0.89303	C12orf36	76353	25kb
ENSG00000273079	12	13668165	14158053	1632	115	120000	-0.95157	0.82934	GRIN2B	76353	25kb
ENSG00000171681	12	14493610	14676697	434	31	120000	-1.3334	0.90879	ATF7IP	76353	25kb
ENSG00000121316	12	14631595	14746283	255	34	120000	-2.1104	0.98259	PLBD1	76353	25kb
ENSG00000070019	12	14740576	14874519	245	32	120000	-0.67276	0.74945	GUCY2C	76353	25kb
ENSG00000197837	12	14895933	14949065	148	19	120000	-0.66427	0.74674	HIST4H4	76353	25kb
ENSG00000246705	12	14902270	14955936	147	19	120000	-0.58466	0.72061	H2AFJ	76353	25kb
ENSG00000084463	12	14914410	14981474	167	21	120000	-0.48835	0.68735	WBP11	76353	25kb
ENSG00000182993	12	14931506	15084520	393	21	120000	-0.64243	0.7397	C12orf60	76353	25kb
ENSG00000179256	12	14932584	14992116	132	20	120000	-0.72608	0.76611	SMCO3	76353	25kb

ENSG00000111339	12	14953503	15021429	178	14	120000	-1.0563	0.85458	ART4	76353	25kb
ENSG00000111341	12	15009115	15063860	132	6	120000	-0.92204	0.82174	MGP	76353	25kb
ENSG00000139055	12	15041969	15117016	221	16	120000	0.24791	0.4021	ERP27	76353	25kb
ENSG00000111348	12	15069951	15139662	250	27	120000	0.10951	0.4564	ARHGDIB	76353	25kb
ENSG00000139053	12	15100956	15159799	203	22	120000	-0.63247	0.73646	PDE6H	76353	25kb
ENSG00000134533	12	15235717	15526609	596	38	120000	-0.062974	0.52511	RERG	76353	25kb
ENSG00000151490	12	15450331	15775333	731	65	120000	0.166	0.43408	PTPRO	76353	25kb
ENSG00000151491	12	15748092	16060263	350	47	120000	-1.2237	0.88948	EPS8	76353	25kb
ENSG00000023734	12	16010325	16081412	65	18	120000	0.090773	0.46384	STRAP	76353	25kb
ENSG00000023697	12	16039106	16215220	275	43	120000	1.7779	0.037706	DERA	76353	25kb
ENSG00000188991	12	16316419	16455619	517	25	120000	0.076163	0.46964	SLC15A5	76353	25kb
ENSG00000008394	12	16475076	16787193	592	57	120000	-0.38228	0.64887	MGST1	76353	25kb
ENSG00000048540	12	16676307	16788528	130	39	120000	-0.20025	0.57936	LMO3	76353	25kb
ENSG00000111404	12	18208803	18498041	910	47	120000	-0.28754	0.61315	RERGL	76353	25kb
ENSG00000139144	12	18375548	18826348	1398	69	120000	0.38437	0.35035	PIK3C2G	76353	25kb
ENSG00000139151	12	18811103	18915991	370	30	120000	-0.25519	0.60071	PLCZ1	76353	25kb
ENSG00000177938	12	18866045	18917121	153	24	120000	-0.78366	0.78338	CAPZA3	76353	25kb
ENSG00000052126	12	19257648	19554334	797	43	120000	-1.1737	0.87974	PLEKHA5	76353	25kb
ENSG00000139154	12	19531979	19898735	1738	74	120000	-1.1972	0.88438	AEBP2	76353	25kb
ENSG00000172572	12	20497179	20862315	1177	91	120000	-0.82069	0.79409	PDE3A	76353	25kb
ENSG00000139155	12	20823289	20931320	422	37	120000	-1.6289	0.94833	SLCO1C1	76353	25kb
ENSG00000111700	12	20938636	21268040	1353	33	120000	-1.2952	0.90238	SLCO1B3	76353	25kb
ENSG00000257046	12	20943608	21268179	1327	32	120000	-1.2432	0.8931	LST3	76353	25kb
ENSG00000205754	12	20943673	21270679	1333	32	120000	-1.2547	0.8952	SLCO1B7	76353	25kb
ENSG00000257062	12	21175113	21354829	659	26	120000	-0.10101	0.54023	RP11-125O5.2	76353	25kb
ENSG00000134538	12	21259136	21417180	564	26	120000	0.30828	0.37894	SLCO1B1	76353	25kb
ENSG00000084453	12	21392534	21597528	747	50	120000	-0.020862	0.50832	SLCO1A2	76353	25kb
ENSG00000121351	12	21482893	21557912	272	30	120000	-0.29357	0.61546	IAPP	76353	25kb
ENSG00000121350	12	21565549	21648300	373	18	120000	-1.5647	0.94118	PYROXD1	76353	25kb
ENSG00000004700	12	21596845	21679603	384	28	120000	-1.949	0.97435	RECQL	76353	25kb
ENSG00000111711	12	21629715	21696342	259	27	120000	-1.7496	0.9599	GOLT1B	76353	25kb
ENSG00000134548	12	21654241	21715311	284	25	120000	-0.42732	0.66543	C12orf39	76353	25kb
ENSG00000111713	12	21664123	21782781	444	34	120000	-0.20136	0.57979	GYS2	76353	25kb
ENSG00000111716	12	21763276	21935791	354	17	120000	-1.4915	0.93208	LDHB	76353	25kb
ENSG00000121361	12	21892889	21953515	44	12	120000	-0.36614	0.64287	KCNJ8	76353	25kb
ENSG00000069431	12	21925335	22119336	380	42	120000	-0.11526	0.54588	ABCC9	76353	25kb
ENSG00000111726	12	22174108	22243608	190	25	120000	-0.15397	0.56118	CMAS	76353	25kb
ENSG00000111728	12	22191707	22614975	1215	73	120000	0.43984	0.33003	ST8SIA1	76353	25kb
ENSG00000111731	12	22576517	22722480	203	9	120000	0.27504	0.39164	C2CD5	76353	25kb
ENSG00000139163	12	22753009	22868599	239	21	120000	-0.37056	0.64452	ETNK1	76353	25kb
ENSG00000134532	12	23657440	24128966	1233	137	120000	0.52228	0.30074	SOX5	76353	25kb
ENSG00000060982	12	24939295	25127393	774	63	120000	-0.087938	0.53504	BCAT1	76353	25kb
ENSG00000268865	12	25030243	25082615	165	35	120000	-0.031013	0.51237	AC026310.1	76353	25kb
ENSG00000226397	12	25121358	25175453	219	38	120000	0.32243	0.37356	C12orf77	76353	25kb
ENSG00000118308	12	25148936	25286268	533	62	120000	0.15273	0.4393	LRMP	76353	25kb
ENSG00000118307	12	25236354	25373096	450	26	120000	-0.37007	0.64433	CASC1	76353	25kb
ENSG00000205707	12	25323150	25387579	188	18	120000	-1.0188	0.84586	LYRM5	76353	25kb
ENSG00000133703	12	25332723	25428870	286	29	120000	-1.0031	0.84209	KRAS	76353	25kb
ENSG00000268076	12	25356515	25415840	188	23	120000	-1.1625	0.87748	AC087239.1	76353	25kb
ENSG00000152936	12	25537241	25826513	1074	72	120000	-0.10924	0.54349	IFLTD1	76353	25kb
ENSG00000123094	12	26086962	26257825	687	43	120000	-0.8564	0.80411	RASSF8	76353	25kb
ENSG00000123095	12	26247959	26303060	174	29	120000	0.03775	0.48494	BHLHE41	76353	25kb
ENSG00000123096	12	26249924	26477223	805	70	120000	0.55541	0.28931	SSPN	76353	25kb
ENSG00000123104	12	26465342	27011131	1771	117	120000	-1.0026	0.84197	ITPR2	76353	25kb
ENSG00000064102	12	27033114	27116259	218	37	120000	0.7384	0.23013	ASUN	76353	25kb
ENSG00000111790	12	27066316	27144583	179	35	120000	1.4572	0.072526	FGFR1OP2	76353	25kb
ENSG00000064115	12	27101128	27192367	251	41	120000	1.1567	0.12369	TM7SF3	76353	25kb
ENSG00000152944	12	27150479	27244276	305	28	120000	0.16771	0.43341	MED21	76353	25kb
ENSG00000214700	12	27208991	27260447	183	21	120000	-0.33994	0.63305	C12orf71	76353	25kb
ENSG00000211455	12	27371901	27503892	398	38	120000	-0.53306	0.703	STK38L	76353	25kb
ENSG00000029153	12	27460787	27601241	414	48	120000	-0.018282	0.50729	ARNTL2	76353	25kb
ENSG00000165935	12	27594743	27680118	367	26	120000	-0.0398	0.51587	SMCO2	76353	25kb
ENSG00000110841	12	27651364	27873497	677	59	120000	2.1277	0.01668	PPFIBP1	76353	25kb
ENSG00000174236	12	27824428	27875566	87	17	120000	-0.64652	0.74103	REP15	76353	25kb
ENSG00000061794	12	27838706	27934228	214	20	120000	-0.57354	0.71686	MRPS35	76353	25kb
ENSG00000205693	12	27890671	27949209	154	15	120000	-0.52856	0.70144	MANSC4	76353	25kb
ENSG00000087448	12	27907953	27980973	253	25	120000	-1.1119	0.86692	KLHL42	76353	25kb
ENSG00000087494	12	28086017	28150638	217	27	120000	0.41057	0.3407	PTHLH	76353	25kb
ENSG00000123106	12	28261182	28757883	1311	24	120000	-0.40278	0.65644	CCDC91	76353	25kb
ENSG00000064763	12	29277036	29518913	787	33	120000	0.35958	0.35958	FAR2	76353	25kb
ENSG00000087502	12	29465285	29559122	246	28	120000	0.54956	0.29131	ERGIC2	76353	25kb
ENSG00000257599	12	29517227	29665421	497	40	120000	0.95991	0.16855	OVCH1-AS1	76353	25kb
ENSG00000187950	12	29540407	29675619	457	41	120000	0.90356	0.18311	OVCH1	76353	25kb
ENSG00000133687	12	29628773	29962692	1272	102	120000	0.87629	0.19044	TMTC1	76353	25kb
ENSG00000133704	12	30756922	30873920	307	15	120000	-0.46808	0.68014	IPO8	76353	25kb
ENSG00000110888	12	30837486	30932885	246	25	120000	-1.6959	0.95505	CAPRIN2	76353	25kb
ENSG00000110900	12	31054362	31174534	406	68	120000	0.13221	0.44741	TSPAN11	76353	25kb
ENSG00000013573	12	31201779	31282725	188	16	120000	-0.45109	0.67404	DDX11	76353	25kb
ENSG00000139146	12	31408518	31504992	128	27	120000	-0.93835	0.82597	FAM60A	76353	25kb
ENSG00000177340	12	31452250	31503879	57	16	120000	-0.61481	0.73066	AC024940.1	76353	25kb
ENSG00000170456	12	31510157	31769031	904	31	120000	-1.5566	0.94022	DENND5B	76353	25kb
ENSG00000139160	12	31775094	31851048	287	37	120000	-1.5978	0.94495	METTL20	76353	25kb
ENSG00000151743	12	31799071	31907108	289	31	120000	-0.99534	0.84021	AMN1	76353	25kb
ENSG00000188375	12	31919123	31970175	239	32	120000	-2.8117	0.99754	H3F3C	76353	25kb
ENSG00000174718	12	32087304	32171039	334	44	120000	-0.65694	0.74439	KIAA1551	76353	25kb
ENSG00000151746	12	32234769	32561567	1358	64	120000	0.77458	0.21929	BICD1	76353	25kb
ENSG00000139132	12	32527463	32823984	1115	56	120000	1.151	0.12488	FGD4	76353	25kb
ENSG00000087470	12	32807134	32923486	407	11	120000	0.6116	0.2704	DNM1L	76353	25kb
ENSG00000139131	12	32855424	32933836	221	16	120000	0.55256	0.29028	YARS2	76353	25kb
ENSG00000057294	12	32918679	33074774	540	40	120000	0.30763	0.37918	PKP2	76353	25kb
ENSG00000110975	12	33502173	33617754	335	22	120000	-1.8111	0.96494	SYT10	76353	25kb
ENSG00000139133	12	34150216	34207629	137	13	120000	-0.91643	0.82028	ALG10	76353	25kb
ENSG00000175548	12	38685380	38742784	204	16	120000	0.093456	0.46277	ALG10B	76353	25kb
ENSG00000139117	12	39015624	39326232	889	35	120000	1.0028	0.15799	CPNE8	76353	25kb
ENSG00000139116	12	39662030	39862192	326	21	120000	1.1097	0.13357	KIF21A	76353	25kb
ENSG00000173208	12	39918835	40038553	248	33	120000	1.4811	0.069288	ABCD2	76353	25kb

ENSG00000180116	12	39994969	40327102	751	36	120000	-0.046024	0.51835	C12orf40	76353	25kb
ENSG00000151229	12	40123823	40524891	981	64	120000	-0.99069	0.83908	SLC2A13	76353	25kb
ENSG00000188906	12	40565546	40788087	793	41	120000	0.33338	0.36942	LRRK2	76353	25kb
ENSG00000205592	12	40762197	40989632	1139	36	120000	-0.37332	0.64555	MUC19	76353	25kb
ENSG00000018236	12	41061244	41491220	1356	56	120000	-0.4408	0.67032	CNTN1	76353	25kb
ENSG00000165966	12	41557250	41993392	1490	49	120000	0.61133	0.27049	PDZRN4	76353	25kb
ENSG00000151233	12	42450647	42563681	239	26	120000	-0.22546	0.58919	GXYLT1	76353	25kb
ENSG00000015153	12	42525906	42657151	260	21	120000	0.29959	0.38225	YAF2	76353	25kb
ENSG00000269367	12	42599050	42653075	100	14	120000	0.25606	0.39895	AC020629.1	76353	25kb
ENSG00000134283	12	42607249	42878517	670	22	120000	0.43619	0.33135	PPHLN1	76353	25kb
ENSG00000139168	12	42680880	42744920	182	14	120000	0.79336	0.21378	ZCRB1	76353	25kb
ENSG00000139174	12	42827140	43009157	537	53	120000	-0.53752	0.70455	PRICKLE1	76353	25kb
ENSG00000173157	12	43722669	43970724	811	31	120000	1.7589	0.039294	ADAMTS20	76353	25kb
ENSG00000129317	12	44097410	44177620	243	19	120000	-0.091087	0.53629	PUS7L	76353	25kb
ENSG00000198001	12	44127747	44208346	208	22	120000	-0.17513	0.56951	IRAK4	76353	25kb
ENSG00000151239	12	44162526	44225178	157	26	120000	-0.70458	0.75947	TWF1	76353	25kb
ENSG00000139173	12	44204770	44808545	1476	45	120000	-0.8007	0.78835	TMEM117	76353	25kb
ENSG00000184613	12	44877058	45340631	1147	67	120000	1.6473	0.049747	NELL2	76353	25kb
ENSG00000185610	12	45383455	45469882	288	19	120000	-0.72342	0.76529	DBX2	76353	25kb
ENSG00000177119	12	45584770	45859187	671	27	120000	-0.36609	0.64285	ANO6	76353	25kb
ENSG00000189079	12	46098448	46326823	246	28	120000	-1.0256	0.84745	ARID2	76353	25kb
ENSG00000139218	12	46287914	46410903	151	23	120000	-0.56031	0.71237	SCAF11	76353	25kb
ENSG00000111371	12	46551846	46688800	284	49	120000	-0.60646	0.72789	SLC38A1	76353	25kb
ENSG00000134294	12	46726972	46791650	111	13	120000	0.39662	0.34582	SLC38A2	76353	25kb
ENSG00000139209	12	47133546	47251191	356	38	120000	0.095074	0.46213	SLC38A4	76353	25kb
ENSG00000139211	12	47444490	47498734	143	27	120000	0.55349	0.28996	AMIGO2	76353	25kb
ENSG00000179715	12	47448386	47655443	658	65	120000	-1.0619	0.85586	PCED1B	76353	25kb
ENSG00000005175	12	48032070	48124844	224	23	120000	-0.98215	0.83699	RPAP3	76353	25kb
ENSG00000111405	12	48078517	48144350	189	24	120000	-0.83283	0.79753	ENDOU	76353	25kb
ENSG00000079337	12	48103455	48189823	296	30	120000	-0.71969	0.76414	RAPGEF3	76353	25kb
ENSG00000211584	12	48122699	48201536	273	34	120000	-0.79908	0.78788	SLC48A1	76353	25kb
ENSG00000061273	12	48151505	48251915	297	42	120000	-1.178	0.8806	HDAC7	76353	25kb
ENSG00000268069	12	48153706	48204787	170	29	120000	-0.50687	0.69388	AC004466.1	76353	25kb
ENSG00000111424	12	48210320	48361831	429	61	120000	-0.4722	0.68161	VDR	76353	25kb
ENSG00000134291	12	48332352	48387661	194	28	120000	-1.046	0.85221	TMEM106C	76353	25kb
ENSG00000139219	12	48341748	48423269	297	27	120000	-0.8558	0.80395	COL2A1	76353	25kb
ENSG00000257955	12	48388554	48444165	188	23	120000	-0.38164	0.64864	RP1-228P16.5	76353	25kb
ENSG00000079387	12	48411681	48525091	323	20	120000	-0.22157	0.58768	SENP1	76353	25kb
ENSG00000152556	12	48473922	48565187	244	25	120000	-0.25789	0.60175	PFKM	76353	25kb
ENSG00000177981	12	48516571	48599996	264	17	120000	-0.90114	0.81624	ASB8	76353	25kb
ENSG00000177875	12	48552366	48604709	215	17	120000	-0.84326	0.80046	C12orf68	76353	25kb
ENSG00000269514	12	48567170	48620814	235	15	120000	-0.78522	0.78384	DKFZP779L1853	76353	25kb
ENSG00000172640	12	48571081	48622170	230	15	120000	-0.77711	0.78145	OR10AD1	76353	25kb
ENSG00000187166	12	48697763	48749062	157	17	120000	-0.51971	0.69837	H1FNT	76353	25kb
ENSG00000167528	12	48708791	48770197	149	18	120000	-0.64072	0.73915	ZNF641	76353	25kb
ENSG00000269282	12	48734919	48786738	137	22	120000	-0.62808	0.73502	AC024257.1	76353	25kb
ENSG00000139223	12	48841448	48891843	203	18	120000	-0.3804	0.64817	ANP32D	76353	25kb
ENSG00000177627	12	48851286	48915295	260	16	120000	-0.48252	0.68528	C12orf54	76353	25kb
ENSG00000197376	12	48876137	48948680	291	18	120000	-1.1684	0.87867	OR8S1	76353	25kb
ENSG00000167531	12	48936467	48988849	154	22	120000	-1.1309	0.87094	LALBA	76353	25kb
ENSG00000139620	12	49022184	49101021	183	16	120000	0.39119	0.34783	KANSL2	76353	25kb
ENSG00000129315	12	49057247	49135681	177	17	120000	0.57468	0.28275	CCNT1	76353	25kb
ENSG00000257987	12	49096218	49184569	191	28	120000	0.21093	0.41647	LINC00935	76353	25kb
ENSG00000174233	12	49134975	49207820	145	25	120000	-0.50922	0.6947	ADCY6	76353	25kb
ENSG00000167535	12	49182577	49247724	114	22	120000	0.18943	0.42488	CACNB3	76353	25kb
ENSG00000174243	12	49198547	49271625	128	28	120000	0.31201	0.37751	DDX23	76353	25kb
ENSG00000172602	12	49225928	49284681	128	22	120000	-0.055348	0.52207	RND1	76353	25kb
ENSG00000272822	12	49272286	49376148	265	14	120000	-1.2528	0.89486	RP11-302B13.5	76353	25kb
ENSG00000139537	12	49272893	49350623	214	15	120000	-1.087	0.86149	CCDC65	76353	25kb
ENSG00000134285	12	49290301	49345257	147	10	120000	-1.0447	0.85193	FKBP11	76353	25kb
ENSG00000255863	12	49290767	49358446	194	12	120000	-1.1632	0.87763	AC073610.5	76353	25kb
ENSG00000134287	12	49304506	49376334	187	13	120000	-1.2099	0.88684	ARF3	76353	25kb
ENSG00000169884	12	49334123	49390546	123	14	120000	-1.1886	0.88271	WNT10B	76353	25kb
ENSG00000125084	12	49347398	49400459	85	15	120000	-1.1895	0.88289	WNT1	76353	25kb
ENSG00000181418	12	49363932	49418092	54	16	120000	-0.36129	0.64106	DDN	76353	25kb
ENSG00000181929	12	49371057	49437980	60	12	120000	0.14174	0.44364	PRKAG1	76353	25kb
ENSG00000167548	12	49387758	49478557	87	12	120000	0.37841	0.35256	KMT2D	76353	25kb
ENSG00000167550	12	49433468	49488808	61	11	120000	0.40289	0.34351	RHEBL1	76353	25kb
ENSG00000139549	12	49458204	49513602	69	11	120000	-0.49409	0.68938	DHH	76353	25kb
ENSG00000139636	12	49465919	49529683	83	10	120000	-0.88948	0.81313	LMBR1L	76353	25kb
ENSG00000123416	12	49496565	49550180	69	4	120000	-1.2453	0.89349	TUBA1B	76353	25kb
ENSG00000167552	12	49553579	49608107	79	8	120000	-0.97148	0.83435	TUBA1A	76353	25kb
ENSG00000167553	12	49557519	49692114	259	31	120000	-0.068459	0.52729	TUBA1C	76353	25kb
ENSG00000135406	12	49662035	49717465	174	17	120000	0.85802	0.19544	PRPH	76353	25kb
ENSG00000135451	12	49692019	49750514	158	17	120000	0.60552	0.27242	TROAP	76353	25kb
ENSG00000186897	12	49701200	49755971	138	17	120000	0.21267	0.41579	C1QL4	76353	25kb
ENSG00000178401	12	49715700	49776309	133	19	120000	0.25755	0.39838	DNAJC22	76353	25kb
ENSG00000123352	12	49735367	49946205	422	26	120000	1.5558	0.059874	SPATS2	76353	25kb
ENSG00000135519	12	49907940	49977091	121	16	120000	1.1444	0.12622	KCNH3	76353	25kb
ENSG00000187778	12	49925327	49986936	111	12	120000	0.81597	0.20726	MCRS1	76353	25kb
ENSG00000110844	12	49937001	50063449	177	16	120000	0.72277	0.23491	PRPF40B	76353	25kb
ENSG00000135436	12	49951668	50024422	102	12	120000	0.34844	0.36375	FAM186B	76353	25kb
ENSG00000161791	12	50006724	50126948	197	15	120000	0.47089	0.31886	FMNL3	76353	25kb
ENSG00000139644	12	50076508	50183717	265	18	120000	-0.038804	0.51548	TMBIM6	76353	25kb
ENSG00000167566	12	50159929	50247533	243	29	120000	0.55532	0.28934	NCKAP5L	76353	25kb
ENSG00000186666	12	50206573	50261912	177	20	120000	0.55949	0.28792	BCDIN3D	76353	25kb
ENSG00000135472	12	50235679	50323000	285	37	120000	-1.0956	0.86337	FAIM2	76353	25kb
ENSG00000167580	12	50319524	50377664	146	41	120000	-2.1536	0.98437	AQP2	76353	25kb
ENSG00000161798	12	50330653	50384464	136	33	120000	-1.6323	0.94869	AQP5	76353	25kb
ENSG00000086159	12	50335977	50395922	136	29	120000	-1.3381	0.90956	AQP6	76353	25kb
ENSG00000161800	12	50345706	50451919	200	22	120000	0.020417	0.49186	RACGAP1	76353	25kb
ENSG00000110881	12	50426331	50502394	115	19	120000	0.63208	0.26367	ASIC1	76353	25kb
ENSG00000066117	12	50453755	50519495	104	13	120000	0.43379	0.33222	SMARCD1	76353	25kb
ENSG00000167588	12	50472602	50530102	86	10	120000	0.10269	0.4591	GPD1	76353	25kb
ENSG00000178449	12	50480762	50539240	88	9	120000	-0.088284	0.53517	COX14	76353	25kb
ENSG00000139624	12	50498575	50586288	132	10	120000	-0.32691	0.62813	CERS5	76353	25kb

ENSG00000050405	12	50544571	50702329	210	14	120000	-0.75459	0.77475	LIMA1	76353	25kb
ENSG00000269529	12	50665489	50716718	89	6	120000	-1.082	0.86038	AC140061.12	76353	25kb
ENSG00000185958	12	50695013	50815405	224	19	120000	-1.6998	0.95541	FAM186A	76353	25kb
ENSG00000161813	12	50761166	50898787	280	18	120000	-1.7163	0.95695	LARP4	76353	25kb
ENSG00000066084	12	50873768	51167450	575	23	120000	-0.86966	0.80776	DIP2B	76353	25kb
ENSG00000123268	12	51132493	51239905	206	22	120000	-1.0557	0.85445	ATF1	76353	25kb
ENSG00000186452	12	51211703	51306667	241	18	120000	-1.034	0.84943	TMPRSS12	76353	25kb
ENSG00000185432	12	51292255	51351300	178	24	120000	-0.63862	0.73846	METTL7A	76353	25kb
ENSG00000214511	12	51322705	51389289	217	17	120000	-0.41545	0.66109	HIGD1C	76353	25kb
ENSG00000110911	12	51348184	51447349	302	16	120000	-0.33942	0.63285	SLC11A2	76353	25kb
ENSG00000050426	12	51416745	51479207	165	19	120000	-0.10977	0.54371	LETMD1	76353	25kb
ENSG00000110925	12	51429990	51502447	198	21	120000	0.032303	0.48712	CSRNP2	76353	25kb
ENSG00000135457	12	51462446	51591926	356	25	120000	-0.47909	0.68406	TFCP2	76353	25kb
ENSG00000184271	12	51555719	51636477	208	33	120000	-0.77445	0.78067	POU6F1	76353	25kb
ENSG00000269339	12	51594860	51645085	106	16	120000	-0.746	0.77217	AC139768.1	76353	25kb
ENSG00000183283	12	51607076	51690146	221	24	120000	-0.1544	0.56135	DAZAP2	76353	25kb
ENSG00000170545	12	51613342	51689674	203	22	120000	-0.083333	0.53321	SMAGP	76353	25kb
ENSG00000110934	12	51649822	51743452	300	18	120000	-0.2793	0.60999	BIN2	76353	25kb
ENSG00000139610	12	51697227	51765463	189	19	120000	-0.60488	0.72737	CELA1	76353	25kb
ENSG00000139629	12	51720031	51811651	227	47	120000	0.017011	0.49321	GALNT6	76353	25kb
ENSG00000050438	12	51760101	51927980	368	34	120000	0.78304	0.2168	SLC4A8	76353	25kb
ENSG00000196876	12	51959050	52231648	486	34	120000	-0.76289	0.77724	SCN8A	76353	25kb
ENSG00000260415	12	52178789	52231636	118	19	120000	0.17106	0.43209	AC068987.1	76353	25kb
ENSG00000167612	12	52256744	52310448	181	35	120000	-0.62282	0.7333	ANKRD33	76353	25kb
ENSG00000139567	12	52275692	52342145	212	38	120000	-0.47712	0.68336	ACVRL1	76353	25kb
ENSG00000135503	12	52320451	52415862	190	34	120000	-1.0052	0.84261	ACVR1B	76353	25kb
ENSG00000161835	12	52375724	52434673	129	35	120000	-0.81804	0.79333	GRASP	76353	25kb
ENSG00000123358	12	52391616	52478291	223	43	120000	-0.44595	0.67218	NR4A1	76353	25kb
ENSG00000123395	12	52438030	52496278	176	22	120000	-0.61751	0.73155	C12orf44	76353	25kb
ENSG00000167767	12	52537780	52610784	296	43	120000	-1.0507	0.8533	KRT80	76353	25kb
ENSG00000135480	12	52601304	52670970	255	32	120000	0.02915	0.48837	KRT7	76353	25kb
ENSG00000170442	12	52618084	52727947	446	39	120000	1.6473	0.049749	KRT86	76353	25kb
ENSG00000205426	12	52654697	52710318	224	21	120000	2.0515	0.020111	KRT81	76353	25kb
ENSG00000170523	12	52683085	52740182	242	26	120000	1.9525	0.025438	KRT83	76353	25kb
ENSG00000267873	12	52684128	52734790	226	23	120000	1.9303	0.026788	AC121757.1	76353	25kb
ENSG00000135443	12	52728790	52786265	268	21	120000	1.5686	0.058373	KRT85	76353	25kb
ENSG00000161849	12	52746596	52804436	255	20	120000	1.5971	0.055123	KRT84	76353	25kb
ENSG00000161850	12	52762715	52825139	276	22	120000	1.4209	0.077677	KRT82	76353	25kb
ENSG00000170454	12	52792854	52853309	197	21	120000	0.85755	0.19557	KRT75	76353	25kb
ENSG00000185479	12	52815435	52870910	230	13	120000	0.3173	0.37551	KRT6B	76353	25kb
ENSG00000170465	12	52837300	52892569	282	10	120000	-0.21597	0.58549	KRT6C	76353	25kb
ENSG00000205420	12	52855958	52912041	303	13	120000	-0.12338	0.5491	KRT6A	76353	25kb
ENSG00000186081	12	52883359	52939471	239	28	120000	1.3962	0.081322	KRT5	76353	25kb
ENSG00000268716	12	52897915	52947980	221	27	120000	1.777	0.037783	AC055736.1	76353	25kb
ENSG00000139648	12	52912693	52971931	294	41	120000	1.5927	0.055617	KRT71	76353	25kb
ENSG00000170484	12	52934566	52992609	285	33	120000	1.0679	0.14278	KRT74	76353	25kb
ENSG00000170486	12	52954373	53020322	288	26	120000	1.5161	0.064743	KRT72	76353	25kb
ENSG00000186049	12	52976354	53037343	253	20	120000	1.4011	0.080589	KRT73	76353	25kb
ENSG00000172867	12	53013342	53070948	242	29	120000	0.61672	0.26871	KRT2	76353	25kb
ENSG00000167768	12	53043520	53099191	212	23	120000	-0.22562	0.58925	KRT1	76353	25kb
ENSG00000189182	12	53058410	53122247	239	32	120000	0.14571	0.44208	KRT77	76353	25kb
ENSG00000185069	12	53136939	53196129	219	32	120000	0.78677	0.21571	KRT76	76353	25kb
ENSG00000186442	12	53158469	53214901	216	40	120000	1.9367	0.026393	KRT3	76353	25kb
ENSG00000170477	12	53175333	53233335	192	38	120000	2.2258	0.013014	KRT4	76353	25kb
ENSG00000185640	12	53190194	53253079	247	30	120000	0.49548	0.31013	KRT79	76353	25kb
ENSG00000170423	12	53206588	53267876	255	25	120000	0.2501	0.40126	KRT78	76353	25kb
ENSG00000170421	12	53265977	53368738	439	32	120000	0.67777	0.24896	KRT8	76353	25kb
ENSG00000111057	12	53317655	53371685	159	28	120000	0.6311	0.26399	KRT18	76353	25kb
ENSG00000063046	12	53374942	53460993	224	23	120000	-0.57268	0.71657	EIF4B	76353	25kb
ENSG00000111077	12	53415753	53483156	172	24	120000	-0.70448	0.75943	TENC1	76353	25kb
ENSG00000167778	12	53433388	53498204	141	31	120000	-0.54412	0.70682	SPRYD3	76353	25kb
ENSG00000167779	12	53466220	53521129	139	15	120000	-0.67916	0.75148	IGFBP6	76353	25kb
ENSG00000167780	12	53472302	53543322	173	18	120000	-0.60896	0.72873	SOAT2	76353	25kb
ENSG00000139631	12	53526447	53600135	134	14	120000	-0.62569	0.73424	CSAD	76353	25kb
ENSG00000139651	12	53549484	53609596	98	13	120000	-0.67383	0.74979	ZNF740	76353	25kb
ENSG00000139626	12	53560102	53626091	115	20	120000	-0.68094	0.75205	ITGB7	76353	25kb
ENSG00000172819	12	53579354	53651764	134	30	120000	0.24883	0.40175	RARG	76353	25kb
ENSG00000182544	12	53620035	53673189	118	16	120000	0.60636	0.27214	MFSD5	76353	25kb
ENSG00000135476	12	53637083	53712427	153	20	120000	0.36348	0.35812	ESPL1	76353	25kb
ENSG00000123349	12	53664075	53718234	90	13	120000	0.061884	0.47533	PFDN5	76353	25kb
ENSG00000139637	12	53668470	53725961	94	12	120000	0.079847	0.46818	C12orf10	76353	25kb
ENSG00000094914	12	53676240	53743648	115	17	120000	0.11982	0.45231	AAAS	76353	25kb
ENSG00000170374	12	53695362	53764099	119	21	120000	0.097215	0.46128	SP7	76353	25kb
ENSG00000185591	12	53748960	53835230	158	15	120000	-0.71068	0.76136	SP1	76353	25kb
ENSG00000135409	12	53792639	53850318	87	8	120000	-0.77245	0.78008	AMHR2	76353	25kb
ENSG00000205352	12	53810389	53865429	68	8	120000	-0.72716	0.76644	PRR13	76353	25kb
ENSG00000197111	12	53810525	53899946	99	13	120000	-0.84191	0.80008	PCBP2	76353	25kb
ENSG00000139625	12	53849274	53918847	66	19	120000	-0.76835	0.77886	MAP3K12	76353	25kb
ENSG00000139546	12	53869705	53925215	64	16	120000	-0.99307	0.83966	TARBP2	76353	25kb
ENSG00000267281	12	53875472	54044763	306	17	120000	0.86423	0.19373	RP11-793H13.10	76353	25kb
ENSG00000139574	12	53875474	53926422	60	16	120000	-0.6501	0.74219	NPFF	76353	25kb
ENSG00000170653	12	53876640	54045199	307	18	120000	0.87188	0.19164	ATF7	76353	25kb
ENSG00000135390	12	54001510	54096192	240	18	120000	1.3381	0.090434	ATP5G2	76353	25kb
ENSG00000012822	12	54079903	54146529	172	29	120000	0.57077	0.28408	CALCOCO1	76353	25kb
ENSG00000123364	12	54307535	54365328	169	24	120000	-1.3578	0.91274	HOXC13	76353	25kb
ENSG00000123407	12	54323618	54377740	134	19	120000	-1.1358	0.87197	HOXC12	76353	25kb
ENSG00000123388	12	54341910	54396427	123	23	120000	-0.26669	0.60514	HOXC11	76353	25kb
ENSG00000180818	12	54353849	54409063	98	18	120000	0.34458	0.3652	HOXC10	76353	25kb
ENSG00000273049	12	54354629	54453672	171	31	120000	0.00055499	0.49978	RP11-834C11.12	76353	25kb
ENSG00000197757	12	54359408	54449607	143	27	120000	0.018485	0.49263	HOXC6	76353	25kb
ENSG00000180806	12	54363679	54422121	93	22	120000	0.39155	0.34769	HOXC9	76353	25kb
ENSG00000037965	12	54377832	54432570	90	18	120000	0.61488	0.26932	HOXC8	76353	25kb
ENSG00000198353	12	54385715	54474813	139	27	120000	0.54344	0.29341	HOXC4	76353	25kb
ENSG00000172789	12	54401637	54454142	85	15	120000	-0.1329	0.55286	HOXC5	76353	25kb
ENSG00000273266	12	54422661	54474814	84	19	120000	0.063324	0.47475	HOXC4	76353	25kb
ENSG00000123415	12	54533529	54607778	145	27	120000	-0.073825	0.52943	SMUG1	76353	25kb

ENSG00000094916	12	54599724	54698886	101	16	120000	-0.28798	0.61332	CBX5	76353	25kb
ENSG00000135486	12	54648977	54705872	52	11	120000	-0.82221	0.79452	HNRNPA1	76353	25kb
ENSG00000123405	12	54660895	54719905	54	12	120000	-0.93825	0.82594	NFE2	76353	25kb
ENSG00000111481	12	54669986	54770633	110	17	120000	-0.065123	0.52596	COPZ1	76353	25kb
ENSG00000139572	12	54731229	54783271	69	13	120000	0.77883	0.21804	GPR84	76353	25kb
ENSG00000161642	12	54737917	54810082	87	22	120000	0.42768	0.33444	ZNF385A	76353	25kb
ENSG00000161638	12	54764045	54838244	109	25	120000	0.60916	0.27121	ITGA5	76353	25kb
ENSG00000170627	12	54824734	54892386	186	21	120000	1.1213	0.13108	GTSF1	76353	25kb
ENSG00000123338	12	54866495	54962726	270	44	120000	1.2119	0.11278	NCKAP1L	76353	25kb
ENSG00000123360	12	54918134	54998023	228	42	120000	0.43098	0.33324	PDE1B	76353	25kb
ENSG00000135447	12	54944171	55007443	199	26	120000	-0.050448	0.52012	PPP1R1A	76353	25kb
ENSG00000135413	12	54999595	55053679	149	23	120000	-0.31377	0.62315	LACRT	76353	25kb
ENSG00000161634	12	55013375	55067277	153	29	120000	-0.95761	0.83087	DCD	76353	25kb
ENSG00000172551	12	55199303	55277177	387	21	120000	0.87969	0.18951	MUCL1	76353	25kb
ENSG00000135426	12	55316802	55403530	386	25	120000	0.62654	0.26548	TESPA1	76353	25kb
ENSG00000123307	12	55388729	55448798	199	36	120000	-0.14379	0.55717	NEUROD4	76353	25kb
ENSG00000170605	12	55498465	55549586	223	4	120000	-1.0335	0.84931	OR9K2	76353	25kb
ENSG00000179919	12	55589809	55640759	116	5	120000	-0.80637	0.78998	OR10A7	76353	25kb
ENSG00000197706	12	55615982	55667086	94	8	120000	-0.77178	0.77988	OR6C74	76353	25kb
ENSG00000188324	12	55663072	55714016	96	16	120000	-0.95792	0.83095	OR6C6	76353	25kb
ENSG00000205330	12	55689346	55740408	135	13	120000	-1.0346	0.84957	OR6C1	76353	25kb
ENSG00000205329	12	55700485	55751420	152	11	120000	-1.0129	0.84444	OR6C3	76353	25kb
ENSG00000187857	12	55733895	55784833	147	9	120000	-1.2896	0.90141	OR6C75	76353	25kb
ENSG00000205328	12	55769214	55820289	123	10	120000	-1.5371	0.93787	OR6C65	76353	25kb
ENSG00000185821	12	55795038	55845976	110	11	120000	-1.8383	0.96699	OR6C76	76353	25kb
ENSG00000179695	12	55820998	55871936	133	12	120000	-1.73	0.95818	OR6C2	76353	25kb
ENSG00000184954	12	55837984	55888922	142	13	120000	-1.6781	0.95334	OR6C70	76353	25kb
ENSG00000205327	12	55861147	55912100	160	14	120000	-0.65828	0.74482	OR6C68	76353	25kb
ENSG00000179626	12	55919983	55971028	107	15	120000	-0.053797	0.52145	OR6C4	76353	25kb
ENSG00000179615	12	55943199	55994128	132	16	120000	0.056483	0.47748	OR2AP1	76353	25kb
ENSG00000175398	12	56005644	56056638	136	19	120000	0.56686	0.28541	OR10P1	76353	25kb
ENSG00000170439	12	56050330	56103395	116	19	120000	0.97112	0.16574	METTL7B	76353	25kb
ENSG00000135424	12	56053352	56134827	146	26	120000	1.0685	0.14266	ITGA7	76353	25kb
ENSG00000135441	12	56084820	56138871	74	14	120000	1.2601	0.10382	BLOC1S1	76353	25kb
ENSG00000258311	12	56084820	56143487	77	15	120000	1.285	0.099397	RP11-644F5.10	76353	25kb
ENSG00000135437	12	56089151	56143489	71	16	120000	1.3161	0.094064	RDH5	76353	25kb
ENSG00000135404	12	56094107	56148491	63	17	120000	1.2138	0.11241	CD63	76353	25kb
ENSG00000135414	12	56112064	56175911	59	17	120000	0.95794	0.16905	GDF11	76353	25kb
ENSG00000205323	12	56121247	56236540	125	23	120000	1.3088	0.095293	SARNP	76353	25kb
ENSG00000257390	12	56126059	56246330	128	24	120000	1.3344	0.091037	RP11-762I7.5	76353	25kb
ENSG00000123353	12	56186703	56240663	66	17	120000	1.2295	0.10945	ORMDL2	76353	25kb
ENSG00000135392	12	56189744	56249608	71	18	120000	1.0747	0.14126	DNAJC14	76353	25kb
ENSG00000123342	12	56204217	56261750	61	19	120000	1.003	0.15793	MMP19	76353	25kb
ENSG00000170473	12	56270197	56351402	89	12	120000	-0.87438	0.80905	WIBG	76353	25kb
ENSG00000065357	12	56296103	56372811	89	20	120000	-0.78434	0.78358	DGKA	76353	25kb
ENSG00000185664	12	56322889	56392101	90	19	120000	-0.17943	0.5712	PMEL	76353	25kb
ENSG00000123374	12	56335553	56391568	79	16	120000	-0.037288	0.51487	CDK2	76353	25kb
ENSG00000111540	12	56342697	56413490	108	18	120000	0.17375	0.43103	RAB5B	76353	25kb
ENSG00000139531	12	56365964	56425425	97	14	120000	0.26155	0.39683	SUOX	76353	25kb
ENSG00000123411	12	56376443	56457219	134	18	120000	0.11052	0.456	IKZF4	76353	25kb
ENSG00000197728	12	56410637	56463116	94	14	120000	-0.34309	0.63424	RPS26	76353	25kb
ENSG00000065361	12	56448641	56522289	135	23	120000	-0.80337	0.78912	ERBB3	76353	25kb
ENSG00000257411	12	56470115	56528073	82	20	120000	-0.65663	0.74429	RP11-603J24.9	76353	25kb
ENSG00000170515	12	56473103	56532691	86	22	120000	-0.80982	0.79098	PA2G4	76353	25kb
ENSG00000229117	12	56485370	56536727	73	22	120000	-1.3861	0.91715	RPL41	76353	25kb
ENSG00000135482	12	56486943	56541278	74	23	120000	-1.4133	0.92122	ZC3H10	76353	25kb
ENSG00000139641	12	56487034	56563455	93	26	120000	-1.4761	0.93004	ESYT1	76353	25kb
ENSG00000196465	12	56521040	56578431	60	20	120000	-1.5446	0.93878	MYL6B	76353	25kb
ENSG00000092841	12	56526945	56582280	56	19	120000	-1.4102	0.92076	MYL6	76353	25kb
ENSG00000139613	12	56531767	56608351	93	20	120000	-1.7793	0.9624	SMARCC2	76353	25kb
ENSG00000181852	12	56573285	56640717	99	20	120000	-0.69319	0.7559	RNF41	76353	25kb
ENSG00000139579	12	56590799	56648638	85	17	120000	-0.11249	0.54478	NABP2	76353	25kb
ENSG00000139540	12	56598833	56656630	82	16	120000	0.47942	0.31582	SLC39A5	76353	25kb
ENSG00000139645	12	56606591	56677175	94	13	120000	0.86902	0.19242	ANKRD52	76353	25kb
ENSG00000135469	12	56635642	56689750	76	7	120000	1.104	0.13479	COQ10A	76353	25kb
ENSG00000062485	12	56640483	56719176	115	10	120000	1.1405	0.12703	CS	76353	25kb
ENSG00000144785	12	56654700	56734843	117	10	120000	1.1552	0.124	RP11-977G19.10	76353	25kb
ENSG00000257727	12	56678626	56735120	84	8	120000	1.2055	0.114	CNPY2	76353	25kb
ENSG00000135473	12	56685007	56752837	90	7	120000	1.2533	0.10504	PAN2	76353	25kb
ENSG00000110944	12	56707663	56759193	58	6	120000	1.3246	0.092651	IL23A	76353	25kb
ENSG00000170581	12	56710381	56778939	71	10	120000	1.2468	0.10624	STAT2	76353	25kb
ENSG00000175336	12	56729353	56781607	45	9	120000	1.1912	0.11679	APOF	76353	25kb
ENSG00000111602	12	56785903	56868187	172	24	120000	0.16941	0.43274	TIMELESS	76353	25kb
ENSG00000135517	12	56818286	56887950	148	20	120000	0.075957	0.46973	MIP	76353	25kb
ENSG00000176422	12	56837301	56889763	91	18	120000	0.41526	0.33898	SPRYD4	76353	25kb
ENSG00000135423	12	56839736	56907198	104	21	120000	0.37907	0.35232	GLS2	76353	25kb
ENSG00000076067	12	56890713	57009745	238	33	120000	1.3546	0.087767	RBMS2	76353	25kb
ENSG00000076108	12	56964380	57055600	147	25	120000	0.32238	0.37358	BAZ2A	76353	25kb
ENSG00000110955	12	57006959	57064798	104	14	120000	0.77763	0.21839	ATP5B	76353	25kb
ENSG00000110958	12	57032127	57107159	152	18	120000	0.92897	0.17645	PTGES3	76353	25kb
ENSG00000196531	12	57081212	57150412	104	15	120000	1.0709	0.14211	NACA	76353	25kb
ENSG00000198056	12	57100380	57171157	97	13	120000	1.0106	0.1561	PRIM1	76353	25kb
ENSG00000025423	12	57120945	57206574	141	17	120000	0.12823	0.44898	HSD17B6	76353	25kb
ENSG00000170426	12	57291938	57353189	140	22	120000	1.4358	0.075525	SDR9C7	76353	25kb
ENSG00000139547	12	57320219	57378158	126	12	120000	0.35841	0.36002	RDH16	76353	25kb
ENSG00000166856	12	57363230	57415468	87	8	120000	0.58771	0.27836	GPR182	76353	25kb
ENSG00000268162	12	57365745	57416335	83	8	120000	0.59757	0.27506	HBCBP	76353	25kb
ENSG00000166860	12	57367618	57425230	99	10	120000	0.5682	0.28495	ZBTB39	76353	25kb
ENSG00000166863	12	57378784	57447667	123	12	120000	0.81769	0.20677	TAC3	76353	25kb
ENSG00000166866	12	57397301	57469982	133	12	120000	0.71986	0.2358	MYO1A	76353	25kb
ENSG00000166881	12	57424426	57506846	146	18	120000	0.58157	0.28043	TMEM194A	76353	25kb
ENSG00000166886	12	57457677	57514259	97	19	120000	0.52438	0.30001	NAB2	76353	25kb
ENSG00000166888	12	57464191	57550922	147	28	120000	0.098582	0.46073	STAT6	76353	25kb
ENSG00000123384	12	57497276	57632134	187	29	120000	-0.7294	0.76712	LRP1	76353	25kb
ENSG00000182379	12	57585578	57645232	75	13	120000	-1.1938	0.88372	NXPH4	76353	25kb
ENSG00000182199	12	57598110	57653718	59	15	120000	-1.2634	0.89677	SHMT2	76353	25kb

ENSG00000185633	12	57603686	57659498	60	16	120000	-1.1827	0.88154	NDUFA4L2	76353	25kb
ENSG00000185482	12	57612236	57669976	61	15	120000	-1.0956	0.86337	STAC3	76353	25kb
ENSG00000258830	12	57618392	57715267	113	16	120000	-1.1251	0.86972	RP11-123K3.4	76353	25kb
ENSG00000179912	12	57618392	57849788	287	17	120000	-1.1517	0.87527	R3HDM2	76353	25kb
ENSG00000268367	12	57785198	57835536	69	10	120000	-0.99004	0.83892	AC126614.1	76353	25kb
ENSG00000175189	12	57803543	57869611	118	17	120000	-0.51679	0.69735	INHBC	76353	25kb
ENSG00000139269	12	57821106	57878063	114	19	120000	-0.40603	0.65764	INHBE	76353	25kb
ENSG00000111087	12	57828918	57891045	114	19	120000	-0.39539	0.65372	GLI1	76353	25kb
ENSG00000123329	12	57841038	57907597	114	16	120000	-0.33966	0.63294	ARHGAP9	76353	25kb
ENSG00000166986	12	57844228	57936352	142	16	120000	-0.46009	0.67728	MARS	76353	25kb
ENSG00000175197	12	57885371	57939300	67	5	120000	-0.91474	0.81984	DDIT3	76353	25kb
ENSG00000166987	12	57889493	57948931	77	7	120000	-0.90607	0.81755	MBD6	76353	25kb
ENSG00000175203	12	57898885	57966114	90	12	120000	-1.0415	0.85118	DCTN2	76353	25kb
ENSG00000155980	12	57918781	58005415	128	23	120000	-0.85731	0.80436	KIF5A	76353	25kb
ENSG00000166908	12	57959957	58022198	105	24	120000	-0.17365	0.56893	PIP4K2C	76353	25kb
ENSG00000178498	12	57973405	58028587	100	21	120000	0.19327	0.42337	DTX3	76353	25kb
ENSG00000240771	12	57978963	58038162	103	20	120000	0.18838	0.42529	ARHGEF25	76353	25kb
ENSG00000135502	12	57988310	58044934	90	16	120000	0.10972	0.45632	SLC26A10	76353	25kb
ENSG00000135454	12	57992193	58052138	112	14	120000	-0.18376	0.5729	B4GALNT1	76353	25kb
ENSG00000135506	12	58062738	58140340	155	16	120000	-0.54308	0.70646	OS9	76353	25kb
ENSG00000135439	12	58093980	58160940	89	19	120000	-0.35087	0.63716	AGAP2	76353	25kb
ENSG00000255737	12	58095054	58147139	80	16	120000	-0.35625	0.63917	AGAP2-AS1	76353	25kb
ENSG00000135452	12	58106796	58168994	77	15	120000	-0.29958	0.61775	TSPAN31	76353	25kb
ENSG00000135446	12	58116510	58174796	72	13	120000	-0.18257	0.57243	CDK4	76353	25kb
ENSG00000139266	12	58123881	58179190	70	11	120000	-0.046482	0.51854	MARCH9	76353	25kb
ENSG00000111012	12	58131117	58187769	67	9	120000	-0.16059	0.56379	CYP27B1	76353	25kb
ENSG00000037897	12	58137254	58191576	67	8	120000	-0.084052	0.53349	METTL1	76353	25kb
ENSG00000123427	12	58140275	58201324	73	8	120000	-0.25187	0.59943	METTL21B	76353	25kb
ENSG00000257921	12	58141811	58205829	79	9	120000	-0.22601	0.5894	RP11-571M6.15	76353	25kb
ENSG00000123297	12	58151372	58226854	93	10	120000	-0.3801	0.64806	TSFM	76353	25kb
ENSG00000135407	12	58166159	58237487	96	11	120000	-0.52863	0.70147	AVIL	76353	25kb
ENSG00000175215	12	58188710	58265522	94	13	120000	-0.8116	0.79149	CTDSP2	76353	25kb
ENSG00000166896	12	58310324	58376052	173	13	120000	0.50596	0.30644	XRCC6BP1	76353	25kb
ENSG00000258231	12	58912907	59231842	843	68	120000	-0.3101	0.62176	RP11-362K2.2	76353	25kb
ENSG00000139263	12	59240931	59339303	334	27	120000	-1.1252	0.86975	LRIG3	76353	25kb
ENSG00000257230	12	59892059	59942965	123	22	120000	0.29392	0.38441	RP11-272B17.2	76353	25kb
ENSG00000118596	12	59964848	60201395	605	29	120000	-0.21699	0.58589	SLC16A7	76353	25kb
ENSG00000198673	12	62077040	62697931	1757	102	120000	-0.45974	0.67715	FAM19A2	76353	25kb
ENSG00000135655	12	62629119	62836211	482	28	120000	1.5506	0.060495	USP15	76353	25kb
ENSG00000061987	12	62835597	63016363	480	32	120000	0.18524	0.42652	MON2	76353	25kb
ENSG00000221949	12	62970531	63022214	117	25	120000	1.2735	0.10142	C12orf61	76353	25kb
ENSG00000111110	12	63012762	63353817	1151	129	120000	1.4875	0.068442	PPM1H	76353	25kb
ENSG00000166148	12	63514014	63569722	201	21	120000	0.56037	0.28761	AVPR1A	76353	25kb
ENSG00000177990	12	63927693	64087719	550	27	120000	0.13764	0.44526	DPY19L2	76353	25kb
ENSG00000118600	12	64148583	64228338	204	22	120000	0.28127	0.38925	TMEM5	76353	25kb
ENSG00000196935	12	64213073	64566613	713	80	120000	-0.45133	0.67412	SRGAP1	76353	25kb
ENSG00000174206	12	64555096	64641076	121	18	120000	0.51126	0.30458	C12orf66	76353	25kb
ENSG00000185306	12	64635217	64809972	426	32	120000	0.89427	0.18559	C12orf56	76353	25kb
ENSG00000184575	12	64773130	64869907	159	21	120000	-0.26504	0.60451	XPOT	76353	25kb
ENSG00000183735	12	64820660	64920888	196	29	120000	-0.13366	0.55316	TBK1	76353	25kb
ENSG00000153179	12	64979293	65116347	331	28	120000	2.3746	0.0087836	RASSF3	76353	25kb
ENSG00000268028	12	65065189	65115329	113	24	120000	1.1708	0.12083	AC025262.1	76353	25kb
ENSG00000135677	12	65082225	65178227	154	20	120000	-0.52866	0.70148	GNS	76353	25kb
ENSG00000111490	12	65149589	65299812	218	34	120000	0.083061	0.4669	TBC1D30	76353	25kb
ENSG00000156076	12	65419406	65540346	198	24	120000	0.83297	0.20243	WIF1	76353	25kb
ENSG00000174106	12	65538351	65667107	167	21	120000	1.664	0.048058	LEMD3	76353	25kb
ENSG00000174099	12	65647423	65907024	428	30	120000	-0.015406	0.50615	MSRB3	76353	25kb
ENSG00000149948	12	66192911	66385075	367	42	120000	0.38836	0.34888	HMGA2	76353	25kb
ENSG00000197301	12	66220120	66300947	148	22	120000	0.83444	0.20202	RP11-366L20.2	76353	25kb
ENSG00000267942	12	66266518	66342967	118	27	120000	0.71886	0.23611	AC090673.2	76353	25kb
ENSG00000139233	12	66491842	66549548	149	14	120000	-0.43941	0.66982	LLPH	76353	25kb
ENSG00000228144	12	66492697	66588765	249	20	120000	-0.37615	0.6466	TMBIM4	76353	25kb
ENSG00000155957	12	66492709	66588852	250	20	120000	-0.3623	0.64144	TMBIM4	76353	25kb
ENSG00000090376	12	66557659	66673402	266	36	120000	0.0093727	0.49626	IRAK3	76353	25kb
ENSG00000127311	12	66671325	66762423	208	13	120000	-1.8154	0.96527	HELB	76353	25kb
ENSG00000155974	12	66716211	67222966	1530	115	120000	1.165	0.122	GRIP1	76353	25kb
ENSG00000111530	12	67638061	67738731	324	16	120000	-0.97266	0.83464	CAND1	76353	25kb
ENSG00000127334	12	68017118	68084186	134	18	120000	-0.15981	0.56349	DYRK2	76353	25kb
ENSG00000111537	12	68523548	68578527	172	17	120000	2.4563	0.0070191	IFNG	76353	25kb
ENSG00000111536	12	68570131	68644601	249	30	120000	2.1281	0.016663	IL26	76353	25kb
ENSG00000127318	12	68617022	68672387	217	25	120000	1.4769	0.069857	IL22	76353	25kb
ENSG00000111554	12	68641223	68751161	286	29	120000	1.3702	0.085316	MDM1	76353	25kb
ENSG00000127314	12	68979619	69079372	190	23	120000	0.1466	0.44172	RAP1B	76353	25kb
ENSG00000111581	12	69055514	69161785	237	30	120000	0.29527	0.38389	NUP107	76353	25kb
ENSG00000175782	12	69114886	69212508	226	34	120000	-0.19705	0.57811	SLC35E3	76353	25kb
ENSG00000251695	12	69161125	69212743	136	23	120000	-0.69901	0.75773	AC124890.1	76353	25kb
ENSG00000135679	12	69176956	69264214	209	35	120000	-0.61483	0.73067	MDM2	76353	25kb
ENSG00000135678	12	69210977	69390350	541	71	120000	-1.3968	0.91876	CPM	76353	25kb
ENSG00000111605	12	69608317	69693138	179	20	120000	2.6597	0.00391	CPSF6	76353	25kb
ENSG00000090382	12	69717121	69773014	184	16	120000	2.0899	0.018314	LYZ	76353	25kb
ENSG00000127337	12	69728483	69809576	281	20	120000	2.4571	0.0070034	YEATS4	76353	25kb
ENSG00000166225	12	69839129	69998562	388	21	120000	-1.2812	0.89994	FRS2	76353	25kb
ENSG00000166226	12	69954114	70020350	165	20	120000	-1.7865	0.96299	CCT2	76353	25kb
ENSG00000198812	12	69977351	70029942	175	24	120000	-1.5401	0.93823	LRRC10	76353	25kb
ENSG00000127325	12	70012140	70118256	440	33	120000	-0.72521	0.76584	BEST3	76353	25kb
ENSG00000127328	12	70107461	70241984	330	23	120000	0.39361	0.34693	RAB3IP	76353	25kb
ENSG00000258052	12	70170449	70274143	323	24	120000	0.41323	0.33972	AC025263.3	76353	25kb
ENSG00000166268	12	70194084	70377877	687	43	120000	1.0156	0.15491	MYRFL	76353	25kb
ENSG00000111596	12	70611774	70773773	453	26	120000	0.44457	0.32831	CNOT2	76353	25kb
ENSG00000135643	12	70735056	70853072	408	47	120000	-0.40386	0.65684	KCNMB4	76353	25kb
ENSG00000127329	12	70885630	71056220	653	73	120000	-1.2669	0.89741	PTPRB	76353	25kb
ENSG00000153233	12	71006853	71339623	1042	93	120000	-1.9561	0.97477	PTPRR	76353	25kb
ENSG00000258053	12	71416182	71537027	314	16	120000	-2.0775	0.98112	CTD-2021H9.3	76353	25kb
ENSG00000127324	12	71493865	71860678	1035	61	120000	-0.31477	0.62353	TSPAN8	76353	25kb
ENSG00000139292	12	71808550	72005090	678	43	120000	-0.045628	0.5182	LGR5	76353	25kb
ENSG00000133858	12	71978252	72086505	162	17	120000	-0.38435	0.64964	ZFC3H1	76353	25kb

ENSG00000173451	12	72031789	72099419	98	14	120000	-0.4787	0.68393	THAP2	76353	25kb
ENSG00000258064	12	72042984	72117748	123	18	120000	-0.65681	0.74435	RP11-293I14.2	76353	25kb
ENSG00000139291	12	72054867	72122836	129	20	120000	-0.81944	0.79373	TMEM19	76353	25kb
ENSG00000080371	12	72123654	72209699	178	16	120000	0.35201	0.36242	RAB21	76353	25kb
ENSG00000121749	12	72208487	72345629	285	21	120000	-0.51	0.69497	TBC1D15	76353	25kb
ENSG00000139287	12	72307626	72605398	747	44	120000	1.563	0.059024	TPH2	76353	25kb
ENSG00000072657	12	72456046	73084422	1361	70	120000	-0.67479	0.7501	TRHDE	76353	25kb
ENSG00000253719	12	74906551	74960223	215	21	120000	0.44637	0.32767	ATXN7L3B	76353	25kb
ENSG00000166006	12	75408857	75628648	533	41	120000	1.2411	0.10728	KCNC2	76353	25kb
ENSG00000180881	12	75644759	75809708	295	23	120000	-0.73658	0.76931	CAPS2	76353	25kb
ENSG00000173401	12	75703419	75789340	148	16	120000	-0.68033	0.75185	GLIPR1L1	76353	25kb
ENSG00000180481	12	75759850	75851468	235	11	120000	-0.90347	0.81686	GLIPR1L2	76353	25kb
ENSG00000139278	12	75849460	75922633	194	16	120000	-0.60829	0.7285	GLIPR1	76353	25kb
ENSG00000111615	12	75865684	75930416	155	16	120000	-0.55546	0.71071	KRR1	76353	25kb
ENSG00000139289	12	76394227	76452712	173	39	120000	-1.2857	0.90073	PHLDA1	76353	25kb
ENSG00000187109	12	76413670	76503813	160	24	120000	0.25445	0.39958	NAP1L1	76353	25kb
ENSG00000179941	12	76713254	76767222	90	20	120000	-0.80082	0.78838	BBS10	76353	25kb
ENSG00000091039	12	76720577	76978589	491	27	120000	-0.71544	0.76283	OSBPL8	76353	25kb
ENSG00000186908	12	77132368	77272476	474	23	120000	0.92299	0.17801	ZDHHC17	76353	25kb
ENSG00000175183	12	77227495	77297840	206	24	120000	0.44127	0.32951	CSRP2	76353	25kb
ENSG00000165891	12	77390027	77484360	308	48	120000	-0.71045	0.76129	E2F7	76353	25kb
ENSG00000268519	12	77924297	77975568	267	17	120000	1.0406	0.14904	AC073528.1	76353	25kb
ENSG00000067798	12	78199685	78631790	1385	94	120000	0.8071	0.2098	NAV3	76353	25kb
ENSG00000067715	12	79232773	79870788	1430	76	120000	0.25048	0.40111	SYT1	76353	25kb
ENSG00000177425	12	79943759	80109877	171	15	120000	-0.86771	0.80722	PAWR	76353	25kb
ENSG00000058272	12	80142343	80354240	310	13	120000	0.17752	0.42955	PPP1R12A	76353	25kb
ENSG00000269531	12	80173742	80227321	74	9	120000	0.023807	0.4905	AC073569.1	76353	25kb
ENSG00000165899	12	80578233	80797870	469	40	120000	1.8413	0.032789	OTOGL	76353	25kb
ENSG00000139304	12	80774774	81097802	830	58	120000	1.6555	0.048916	PTPRQ	76353	25kb
ENSG00000111046	12	81076277	81128253	102	10	120000	0.88575	0.18788	MYF6	76353	25kb
ENSG00000111049	12	81085691	81138452	111	14	120000	0.97208	0.16551	MYF5	76353	25kb
ENSG00000111052	12	81161299	81356704	543	40	120000	0.69188	0.24451	LIN7A	76353	25kb
ENSG00000111058	12	81306594	81675533	1152	42	120000	1.7202	0.042695	ACSS3	76353	25kb
ENSG00000139220	12	81627045	82178332	1276	65	120000	0.51887	0.30192	PPFIA2	76353	25kb
ENSG00000133773	12	82592460	82777584	457	25	120000	-1.7091	0.95628	CCDC59	76353	25kb
ENSG00000127720	12	82727276	82898015	403	13	120000	-1.0656	0.8567	METTL25	76353	25kb
ENSG00000179104	12	83055659	83553649	1402	117	120000	-0.42671	0.66521	TMTC2	76353	25kb
ENSG00000072041	12	85228492	85332394	205	28	120000	1.1718	0.12065	SLC6A15	76353	25kb
ENSG00000231738	12	85383094	85455055	97	16	120000	-0.39906	0.65508	TSPAN19	76353	25kb
ENSG00000133640	12	85405092	85682002	338	19	120000	-0.78185	0.78285	LRRIQ1	76353	25kb
ENSG00000180318	12	85648885	85720562	105	12	120000	-0.59132	0.72285	ALX1	76353	25kb
ENSG00000198774	12	86173331	86255348	328	16	120000	-0.58834	0.72185	RASSF9	76353	25kb
ENSG00000133636	12	86243073	86301770	249	18	120000	-1.0081	0.8433	NTS	76353	25kb
ENSG00000182050	12	86347516	87257681	2282	51	120000	-0.51618	0.69714	MGAT4C	76353	25kb
ENSG00000165805	12	88348816	88452814	188	17	120000	0.32489	0.37263	C12orf50	76353	25kb
ENSG00000133641	12	88402623	88468937	101	16	120000	0.11103	0.4558	C12orf29	76353	25kb
ENSG00000198707	12	88417793	88560993	207	17	120000	-0.19916	0.57893	CEP290	76353	25kb
ENSG00000139324	12	88511073	88618664	164	12	120000	-0.8417	0.80002	TMTC3	76353	25kb
ENSG00000049130	12	88861570	88999628	247	14	120000	-0.47295	0.68188	KITLG	76353	25kb
ENSG00000139318	12	89716009	89772048	159	22	120000	-1.2138	0.88759	DUSP6	76353	25kb
ENSG00000139323	12	89788495	89944801	382	26	120000	2.013	0.022058	POC1B	76353	25kb
ENSG00000257594	12	89888185	89945039	167	16	120000	2.2627	0.011828	GALNT4	76353	25kb
ENSG00000259075	12	89888185	89945039	167	16	120000	2.2627	0.011828	POC1B-GALNT4	76353	25kb
ENSG00000070961	12	89956828	90128077	269	26	120000	1.2713	0.10181	ATP2B1	76353	25kb
ENSG00000197651	12	91274399	91373953	253	25	120000	-1.2134	0.88751	CCER1	76353	25kb
ENSG00000083782	12	91332456	91423803	313	15	120000	-0.1041	0.54145	EPYC	76353	25kb
ENSG00000139330	12	91419268	91476760	252	20	120000	-0.89951	0.81581	KERA	76353	25kb
ENSG00000139329	12	91471406	91530608	188	20	120000	-1.4466	0.92599	LUM	76353	25kb
ENSG00000011465	12	91514025	91601900	91	17	120000	-0.57962	0.71891	DCN	76353	25kb
ENSG00000257242	12	92353756	92561690	626	76	120000	-0.48306	0.68547	C12orf79	76353	25kb
ENSG00000133639	12	92511286	92564673	136	25	120000	-0.044365	0.51769	BTG1	76353	25kb
ENSG00000205057	12	92788870	92846924	179	29	120000	-0.022219	0.50886	CLLU1OS	76353	25kb
ENSG00000257127	12	92790307	92849778	183	29	120000	0.15676	0.43772	CLLU1	76353	25kb
ENSG00000214215	12	93071619	93127325	107	13	120000	0.24996	0.40131	C12orf74	76353	25kb
ENSG00000187510	12	93090281	93191231	197	19	120000	0.17883	0.42903	PLEKHG7	76353	25kb
ENSG00000102189	12	93139413	93348107	440	26	120000	1.0939	0.137	EEA1	76353	25kb
ENSG00000173598	12	93746659	93822024	159	27	120000	-0.27548	0.60853	NUDT4	76353	25kb
ENSG00000177889	12	93774449	93861038	176	32	120000	-0.16594	0.5659	UBE2N	76353	25kb
ENSG00000198015	12	93836264	93922545	283	17	120000	-0.40207	0.65618	MRPL42	76353	25kb
ENSG00000120833	12	93938590	94002263	195	30	120000	0.54034	0.29448	SOCS2	76353	25kb
ENSG00000169372	12	94046151	94313616	694	69	120000	-1.0571	0.85476	CRADD	76353	25kb
ENSG00000136040	12	94517499	94726451	684	83	120000	-1.7612	0.9609	PLXNC1	76353	25kb
ENSG00000258365	12	94646534	94701620	180	28	120000	-0.60312	0.72678	RP11-1105G2.3	76353	25kb
ENSG00000173588	12	94675225	94878764	400	29	120000	-1.0136	0.8446	CCDC41	76353	25kb
ENSG00000057704	12	94935900	95069338	523	58	120000	-0.32453	0.62723	TMCC3	76353	25kb
ENSG00000184752	12	95265831	95422546	688	50	120000	0.25587	0.39903	NDUFA12	76353	25kb
ENSG00000120798	12	95389005	95492479	273	25	120000	-0.069341	0.52764	NR2C1	76353	25kb
ENSG00000180263	12	95445525	95636258	542	29	120000	-0.69153	0.75538	FGD6	76353	25kb
ENSG00000028203	12	95586522	95721566	411	31	120000	-0.9489	0.82866	VEZT	76353	25kb
ENSG00000258247	12	95745984	95799672	218	28	120000	0.38714	0.34933	RP11-167N24.6	76353	25kb
ENSG00000111142	12	95842296	95934615	339	22	120000	1.6064	0.054089	METAP2	76353	25kb
ENSG00000136014	12	95885336	95970266	321	39	120000	2.711	0.0033537	USP44	76353	25kb
ENSG00000074527	12	96026583	96209930	609	57	120000	1.06	0.14456	NTN4	76353	25kb
ENSG00000258292	12	96171875	96242085	189	22	120000	1.1678	0.12145	RP11-536G4.1	76353	25kb
ENSG00000139343	12	96227706	96322606	321	29	120000	0.15735	0.43749	SNRPF	76353	25kb
ENSG00000165972	12	96235826	96361752	454	39	120000	-0.13194	0.55248	CCDC38	76353	25kb
ENSG00000139344	12	96312071	96387370	288	23	120000	-0.47815	0.68373	AMDHD1	76353	25kb
ENSG00000084110	12	96341440	96415143	273	27	120000	-0.6121	0.72976	HAL	76353	25kb
ENSG00000111144	12	96369606	96462298	342	37	120000	-0.24194	0.59559	LTA4H	76353	25kb
ENSG00000111145	12	96563160	96688613	341	48	120000	1.5148	0.064917	ELK3	76353	25kb
ENSG00000059758	12	96647039	96819338	422	36	120000	1.242	0.10713	CDK17	76353	25kb
ENSG00000188596	12	96858349	97294333	1193	39	120000	1.8888	0.029459	C12orf55	76353	25kb
ENSG00000139350	12	97276001	97372129	207	20	120000	2.9062	0.0018293	NEDD1	76353	25kb
ENSG00000245017	12	98854322	98922633	215	30	120000	-0.50079	0.69174	RP11-181C3.1	76353	25kb
ENSG00000120802	12	98884290	98969157	245	34	120000	-0.1494	0.55938	TMPO	76353	25kb
ENSG00000075415	12	98962369	99020946	162	32	120000	1.3433	0.08959	SLC25A3	76353	25kb

ENSG00000166130	12	98982183	99063891	183	26	120000	0.9143	0.18028	IKBIP	76353	25kb
ENSG00000120868	12	99013919	99154204	289	28	120000	-0.012891	0.50514	APAF1	76353	25kb
ENSG00000185046	12	99095235	100403432	3225	186	120000	-1.0292	0.8483	ANKS1B	76353	25kb
ENSG00000180219	12	100016531	100068824	128	20	120000	1.0777	0.14059	FAM71C	76353	25kb
ENSG00000111647	12	100397233	100561626	420	29	120000	-0.34969	0.63672	UHRF1BP1L	76353	25kb
ENSG00000075089	12	100567900	100660643	211	27	120000	-0.11954	0.54758	ACTR6	76353	25kb
ENSG00000166153	12	100572447	100685857	234	30	120000	-0.2381	0.5941	DEPDC4	76353	25kb
ENSG00000136021	12	100635918	100760502	208	22	120000	-0.13451	0.5535	SCYL2	76353	25kb
ENSG00000179520	12	100725857	100840837	259	30	120000	0.40768	0.34175	SLC17A8	76353	25kb
ENSG00000012504	12	100842486	100983191	257	31	120000	-1.2239	0.8895	NR1H4	76353	25kb
ENSG00000139354	12	100942461	101047064	209	28	120000	-0.5881	0.72177	GAS2L3	76353	25kb
ENSG00000151572	12	101086304	101547419	1394	103	120000	-0.99299	0.83964	ANO4	76353	25kb
ENSG00000256870	12	101524271	101629185	367	32	120000	1.0596	0.14466	SLC5A8	76353	25kb
ENSG00000120800	12	101648887	101805394	355	29	120000	-0.12696	0.55051	UTP20	76353	25kb
ENSG00000120805	12	101761898	101826598	158	26	120000	-0.59829	0.72518	ARL1	76353	25kb
ENSG00000166211	12	101844199	101905775	202	34	120000	-0.83051	0.79688	SPIC	76353	25kb
ENSG00000196091	12	101937131	102104796	715	75	120000	-0.85676	0.80421	MYBPC1	76353	25kb
ENSG00000111666	12	102065725	102162918	293	35	120000	-0.30701	0.62058	CHPT1	76353	25kb
ENSG00000139351	12	102097426	102158250	111	17	120000	0.3289	0.37111	SYCP3	76353	25kb
ENSG00000111670	12	102114275	102249716	297	21	120000	0.097777	0.46105	GNPTAB	76353	25kb
ENSG00000136048	12	102246129	102430908	606	57	120000	0.060128	0.47603	DRAM1	76353	25kb
ENSG00000120860	12	102381705	102480927	211	31	120000	0.3303	0.37059	CCDC53	76353	25kb
ENSG00000075188	12	102442967	102538902	207	26	120000	0.36783	0.3565	NUP37	76353	25kb
ENSG00000185480	12	102488956	102616298	258	17	120000	0.52784	0.29881	PARPBP	76353	25kb
ENSG00000183395	12	102565237	102616623	106	13	120000	0.53925	0.29486	PMCH	76353	25kb
ENSG00000017427	12	102764645	102899423	247	37	120000	0.20528	0.41868	IGF1	76353	25kb
ENSG00000171759	12	103205663	103377188	476	36	120000	-0.16877	0.56701	PAH	76353	25kb
ENSG00000139352	12	103326464	103379294	143	29	120000	0.53355	0.29683	ASCL1	76353	25kb
ENSG00000257860	12	103520620	103587087	193	25	120000	0.17636	0.43	RP11-552I14.1	76353	25kb
ENSG00000179088	12	103606369	103914749	695	43	120000	-1.2744	0.89874	C12orf42	76353	25kb
ENSG00000136011	12	103956051	104185505	838	83	120000	-0.57561	0.71756	STAB2	76353	25kb
ENSG00000111696	12	104139231	104259975	532	31	120000	-1.2486	0.8941	NT5DC3	76353	25kb
ENSG00000257327	12	104210229	104263442	252	22	120000	-0.53287	0.70294	RP11-650K20.3	76353	25kb
ENSG00000166598	12	104298885	104372423	316	32	120000	0.20182	0.42003	HSP90B1	76353	25kb
ENSG00000204954	12	104318980	104384486	268	27	120000	-0.17371	0.56895	C12orf73	76353	25kb
ENSG00000139372	12	104334582	104407652	269	27	120000	-0.21129	0.58367	TDG	76353	25kb
ENSG00000120820	12	104357762	104482961	375	41	120000	0.20103	0.42034	GLT8D2	76353	25kb
ENSG00000111727	12	104433235	104523639	207	35	120000	0.072263	0.4712	HCFC2	76353	25kb
ENSG00000120837	12	104485855	104557067	168	24	120000	0.41262	0.33994	NFYB	76353	25kb
ENSG00000198431	12	104584557	104769061	564	31	120000	-0.13183	0.55244	TXNRD1	76353	25kb
ENSG00000255150	12	104672517	104723980	103	15	120000	0.46658	0.3204	EID3	76353	25kb
ENSG00000171310	12	104824073	105180792	1225	134	120000	0.45578	0.32427	CHST11	76353	25kb
ENSG00000136052	12	105171331	105377522	595	26	120000	-0.073429	0.52927	SLC41A2	76353	25kb
ENSG00000151131	12	105355088	105468515	410	33	120000	0.33342	0.36941	C12orf45	76353	25kb
ENSG00000136010	12	105388568	105503355	406	36	120000	-0.17383	0.569	ALDH1L2	76353	25kb
ENSG00000136051	12	105476102	105587912	313	20	120000	-0.075826	0.53022	KIAA1033	76353	25kb
ENSG00000136044	12	105542074	105655016	340	23	120000	-0.36213	0.64137	APPL2	76353	25kb
ENSG00000235162	12	105604068	105814875	704	48	120000	-0.75274	0.7742	C12orf75	76353	25kb
ENSG00000074590	12	106432118	106558811	341	53	120000	-0.088911	0.53542	NUAK1	76353	25kb
ENSG00000136026	12	106606655	106723057	336	58	120000	0.39599	0.34606	CKAP4	76353	25kb
ENSG00000166046	12	106670707	106765793	235	28	120000	1.0716	0.14195	TCP11L2	76353	25kb
ENSG00000013503	12	106726436	106928976	470	31	120000	0.89582	0.18517	POLR3B	76353	25kb
ENSG00000257545	12	106864736	107193696	702	53	120000	0.45999	0.32276	RP11-144F15.1	76353	25kb
ENSG00000111783	12	106951685	107181581	459	33	120000	0.57894	0.28132	RFX4	76353	25kb
ENSG00000111785	12	107143373	107308090	304	25	120000	-0.2024	0.5802	RIC8B	76353	25kb
ENSG00000151135	12	107324497	107397556	195	28	120000	-0.86681	0.80698	C12orf23	76353	25kb
ENSG00000120832	12	107346069	107405944	138	25	120000	-0.74947	0.77321	MTERFD3	76353	25kb
ENSG00000008405	12	107360142	107512607	348	21	120000	-0.71283	0.76202	CRY1	76353	25kb
ENSG00000151136	12	107687190	108078419	1066	88	120000	1.4401	0.074915	BTBD11	76353	25kb
ENSG00000136045	12	108054509	108131944	179	20	120000	-0.17172	0.56817	PWP1	76353	25kb
ENSG00000110851	12	108101643	108180049	202	18	120000	-0.00077501	0.50031	PRDM4	76353	25kb
ENSG00000187855	12	108143162	108195421	190	27	120000	-0.6634	0.74646	ASCL4	76353	25kb
ENSG00000075035	12	108498248	108669314	514	45	120000	0.57497	0.28266	WSCD2	76353	25kb
ENSG00000174600	12	108656821	108758118	329	55	120000	-2.4887	0.99359	CMKLR1	76353	25kb
ENSG00000198855	12	108883962	108944614	224	27	120000	0.32257	0.37351	FICD	76353	25kb
ENSG00000075856	12	108891357	108980176	314	23	120000	-0.14354	0.55707	SART3	76353	25kb
ENSG00000136003	12	108931358	108988160	191	22	120000	-1.2124	0.88732	ISCU	76353	25kb
ENSG00000183160	12	108958622	109017096	180	29	120000	-0.80911	0.79077	TMEM119	76353	25kb
ENSG00000110876	12	108990686	109052735	155	34	120000	-0.35658	0.6393	SELPLG	76353	25kb
ENSG00000110880	12	109013885	109150372	280	27	120000	-0.49163	0.68851	CORO1C	76353	25kb
ENSG00000084112	12	109151466	109276366	230	32	120000	0.07774	0.46902	SSH1	76353	25kb
ENSG00000110887	12	109227708	109319819	244	43	120000	0.36045	0.35925	DAO	76353	25kb
ENSG00000166111	12	109279658	109484045	457	49	120000	-0.70567	0.7598	SVOP	76353	25kb
ENSG00000135093	12	109435894	109550831	195	36	120000	-0.75986	0.77633	USP30	76353	25kb
ENSG00000189046	12	109500996	109556436	111	26	120000	-0.51193	0.69565	ALKBH2	76353	25kb
ENSG00000076248	12	109510379	109573797	158	26	120000	0.11757	0.4532	UNG	76353	25kb
ENSG00000076555	12	109529400	109731031	561	48	120000	1.0817	0.1397	ACACB	76353	25kb
ENSG00000139445	12	109690784	109772025	145	24	120000	-0.7603	0.77646	FOXN4	76353	25kb
ENSG00000174527	12	109760708	109918328	630	28	120000	2.0082	0.022309	MYO1H	76353	25kb
ENSG00000110906	12	109861461	109940349	289	20	120000	0.62885	0.26472	KCTD10	76353	25kb
ENSG00000151148	12	109890207	109999507	391	22	120000	0.61548	0.26912	UBE3B	76353	25kb
ENSG00000139428	12	109966542	110036679	259	18	120000	0.52451	0.29996	MMAB	76353	25kb
ENSG00000110921	12	109986060	110060067	286	23	120000	0.58586	0.27898	MVK	76353	25kb
ENSG00000139438	12	110127033	110233312	264	30	120000	2.1679	0.015085	FAM222A	76353	25kb
ENSG00000111199	12	110195890	110296212	216	41	120000	2.3332	0.00982	TRPV4	76353	25kb
ENSG00000139433	12	110263748	110343293	171	23	120000	1.0787	0.14036	GLTP	76353	25kb
ENSG00000139437	12	110313069	110446646	264	17	120000	1.5708	0.058119	TCHP	76353	25kb
ENSG00000139436	12	110342607	110459194	222	20	120000	1.7143	0.043237	GIT2	76353	25kb
ENSG00000076513	12	110411991	110502568	156	21	120000	1.9753	0.024118	ANKRD13A	76353	25kb
ENSG00000174456	12	110440872	110536491	178	28	120000	1.5005	0.066738	C12orf76	76353	25kb
ENSG00000122970	12	110537140	110681602	167	26	120000	1.4029	0.08032	IFT81	76353	25kb
ENSG00000174437	12	110693561	110813898	143	22	120000	1.0071	0.15695	ATP2A2	76353	25kb
ENSG00000196510	12	110785705	110866535	135	17	120000	0.69688	0.24294	ANAPC7	76353	25kb
ENSG00000111229	12	110847630	110913227	93	11	120000	0.48617	0.31342	ARPC3	76353	25kb
ENSG00000111231	12	110865289	110932073	103	12	120000	0.36962	0.35583	GPN3	76353	25kb
ENSG00000204856	12	110881169	110953190	102	12	120000	0.35132	0.36267	FAM216A	76353	25kb

ENSG00000111237	12	110903902	110964922	91	11	120000	0.31444	0.37659	VPS29	76353	25kb
ENSG00000151164	12	110914460	110994891	113	14	120000	0.4026	0.34362	RAD9B	76353	25kb
ENSG00000196850	12	110944120	111046125	116	18	120000	0.79879	0.21221	PPTC7	76353	25kb
ENSG00000204852	12	111026832	111112235	85	13	120000	0.86528	0.19344	TCTN1	76353	25kb
ENSG00000122986	12	111040646	111167755	166	14	120000	0.48208	0.31488	HVCN1	76353	25kb
ENSG00000186298	12	111132485	111205744	115	10	120000	0.70695	0.2398	PPP1CC	76353	25kb
ENSG00000173093	12	111259573	111370339	319	35	120000	1.596	0.055246	CCDC63	76353	25kb
ENSG00000111245	12	111323623	111383526	175	21	120000	2.4185	0.0077925	MYL2	76353	25kb
ENSG00000111249	12	111446828	111813358	476	39	120000	-0.32307	0.62668	CUX2	76353	25kb
ENSG00000198324	12	111773455	111831925	87	9	120000	0.27875	0.39022	FAM109A	76353	25kb
ENSG00000111252	12	111818752	111914427	83	8	120000	0.066596	0.47345	SH2B3	76353	25kb
ENSG00000204842	12	111865018	112062480	175	14	120000	0.011179	0.49554	ATXN2	76353	25kb
ENSG00000089234	12	112054950	112148790	83	10	120000	-0.41801	0.66203	BRAP	76353	25kb
ENSG00000111271	12	112098857	112219903	103	10	120000	-0.55054	0.70903	ACAD10	76353	25kb
ENSG00000257767	12	112166694	112254222	79	9	120000	-0.72777	0.76662	RP11-162P23.2	76353	25kb
ENSG00000111275	12	112179691	112272782	89	9	120000	-0.69017	0.75496	ALDH2	76353	25kb
ENSG00000089022	12	112254782	112359343	189	7	120000	-0.37115	0.64474	MAPKAPK5	76353	25kb
ENSG00000198270	12	112344086	112475970	199	10	120000	-0.45543	0.6756	TMEM116	76353	25kb
ENSG00000089248	12	112426120	112486255	86	8	120000	-0.25722	0.60149	ERP29	76353	25kb
ENSG00000111300	12	112439500	112571826	162	15	120000	-0.20275	0.58034	NAA25	76353	25kb
ENSG00000135148	12	112538305	112616407	79	12	120000	-0.17584	0.56979	TRAFD1	76353	25kb
ENSG00000173064	12	112572992	112844896	276	16	120000	-0.48449	0.68598	HECTD4	76353	25kb
ENSG00000089009	12	112817994	112881642	65	11	120000	-0.15114	0.56007	RPL6	76353	25kb
ENSG00000179295	12	112831155	112972717	172	18	120000	-0.39589	0.65391	PTPN11	76353	25kb
ENSG00000089169	12	112983184	113361686	709	75	120000	-1.3281	0.90793	RPH3A	76353	25kb
ENSG00000089127	12	113319582	113394990	227	13	120000	1.0331	0.15077	OAS1	76353	25kb
ENSG00000111331	12	113351157	113436054	285	17	120000	1.3704	0.085286	OAS3	76353	25kb
ENSG00000111335	12	113391200	113474528	273	19	120000	1.7358	0.041298	OAS2	76353	25kb
ENSG00000135144	12	113469514	113560833	347	41	120000	0.45997	0.32277	DTX1	76353	25kb
ENSG00000111344	12	113511624	113599044	313	21	120000	0.40276	0.34356	RASAL1	76353	25kb
ENSG00000186710	12	113562663	113622081	141	14	120000	0.3847	0.35023	CCDC42B	76353	25kb
ENSG00000123064	12	113569979	113648284	167	12	120000	0.80093	0.21159	DDX54	76353	25kb
ENSG00000139405	12	113598331	113655173	106	7	120000	1.0505	0.14673	RITA1	76353	25kb
ENSG00000166578	12	113608246	113683899	138	8	120000	1.06	0.14456	IQCD	76353	25kb
ENSG00000186815	12	113633855	113761390	244	12	120000	1.0876	0.13839	TPCN1	76353	25kb
ENSG00000089060	12	113711564	113822298	244	27	120000	0.51052	0.30484	SLC8B1	76353	25kb
ENSG00000151176	12	113771371	113852203	160	19	120000	-0.43422	0.66793	PLBD2	76353	25kb
ENSG00000135094	12	113805250	113889106	170	10	120000	-0.38332	0.64926	SDS	76353	25kb
ENSG00000139410	12	113835042	113901081	130	9	120000	-0.26838	0.6058	SDSL	76353	25kb
ENSG00000089116	12	113874839	113935085	132	16	120000	1.1949	0.11607	LHX5	76353	25kb
ENSG00000122965	12	114229543	114429176	819	48	120000	0.44306	0.32886	RBM19	76353	25kb
ENSG00000089225	12	114766736	114871247	350	48	120000	-0.70161	0.75854	TBX5	76353	25kb
ENSG00000135111	12	115083059	115146969	182	50	120000	0.032538	0.48702	TBX3	76353	25kb
ENSG00000257726	12	115775817	115827550	123	23	120000	0.8918	0.18625	RP11-116D17.1	76353	25kb
ENSG00000123066	12	116370711	116740143	651	40	120000	-0.61393	0.73037	MED13L	76353	25kb
ENSG00000171471	12	116972186	117039425	206	43	120000	0.44056	0.32976	MAP1LC3B2	76353	25kb
ENSG00000111412	12	117128593	117200875	233	40	120000	1.3651	0.086118	C12orf49	76353	25kb
ENSG00000135119	12	117151096	117316436	445	40	120000	0.94225	0.17303	RNFT2	76353	25kb
ENSG00000135116	12	117268949	117344246	150	23	120000	0.25882	0.39789	HRK	76353	25kb
ENSG00000174989	12	117323761	117493953	418	20	120000	-0.72084	0.7645	FBXW8	76353	25kb
ENSG00000088992	12	117451728	117562284	311	28	120000	-1.0277	0.84795	TESC	76353	25kb
ENSG00000135108	12	117556146	117653336	289	18	120000	0.98302	0.1628	FBXO21	76353	25kb
ENSG00000089250	12	117620947	117914975	765	68	120000	2.2217	0.013153	NOS1	76353	25kb
ENSG00000171435	12	117865817	118431788	1913	195	120000	-0.45476	0.67536	KSR2	76353	25kb
ENSG00000111445	12	118426393	118495935	193	30	120000	0.67671	0.2493	RFC5	76353	25kb
ENSG00000176871	12	118445712	118525235	244	45	120000	0.48279	0.31462	WSB2	76353	25kb
ENSG00000176834	12	118476398	118598831	346	53	120000	-0.06772	0.527	VSIG10	76353	25kb
ENSG00000089220	12	118548663	118608389	159	27	120000	0.010401	0.49585	PEBP1	76353	25kb
ENSG00000135090	12	118562606	118835750	561	28	120000	0.92352	0.17787	TAOK3	76353	25kb
ENSG00000111707	12	118789185	118880840	253	13	120000	1.2775	0.10072	SUDS3	76353	25kb
ENSG00000139767	12	119394300	119625856	792	66	120000	1.134	0.1284	SRRM4	76353	25kb
ENSG00000152137	12	119591447	119683936	242	48	120000	0.054387	0.47831	HSPB8	76353	25kb
ENSG00000183273	12	119747517	120003852	662	72	120000	-1.3543	0.91218	CCDC60	76353	25kb
ENSG00000224982	12	120006264	120104363	240	20	120000	1.3245	0.092665	TMEM233	76353	25kb
ENSG00000111725	12	120080558	120144435	109	16	120000	0.67865	0.24868	PRKAB1	76353	25kb
ENSG00000122966	12	120098595	120340095	577	38	120000	-0.56756	0.71483	CIT	76353	25kb
ENSG00000255692	12	120112573	120163816	123	19	120000	0.11356	0.45479	RP1-127H14.3	76353	25kb
ENSG00000135127	12	120402673	120557298	307	19	120000	1.2489	0.10586	CCDC64	76353	25kb
ENSG00000111737	12	120507899	120580306	134	24	120000	1.3491	0.08866	RAB35	76353	25kb
ENSG00000089154	12	120540007	120657513	168	21	120000	0.15138	0.43984	GCN1L1	76353	25kb
ENSG00000089157	12	120609489	120664038	66	11	120000	-0.18395	0.57298	RPLP0	76353	25kb
ENSG00000089159	12	120623250	120728574	155	24	120000	0.16191	0.43569	PXN	76353	25kb
ENSG00000089163	12	120715119	120776052	131	17	120000	0.21223	0.41596	SIRT4	76353	25kb
ENSG00000170890	12	120734914	120790592	122	13	120000	-0.066448	0.52649	PLA2G1B	76353	25kb
ENSG00000135097	12	120754133	120831983	149	15	120000	-0.52169	0.69906	MSI1	76353	25kb
ENSG00000111775	12	120850893	120903545	147	24	120000	-1.2519	0.8947	COX6A1	76353	25kb
ENSG00000111780	12	120851001	120922809	197	27	120000	-1.1914	0.88325	AL021546.6	76353	25kb
ENSG00000170855	12	120856764	120909215	153	24	120000	-1.1939	0.88374	TRIAP1	76353	25kb
ENSG00000257218	12	120859241	120924389	183	24	120000	-1.2637	0.89683	GATC	76353	25kb
ENSG00000111786	12	120874471	120932596	155	19	120000	-1.2716	0.89824	SRSF9	76353	25kb
ENSG00000088986	12	120882653	120961296	225	22	120000	-1.5098	0.93445	DYNLL1	76353	25kb
ENSG00000110871	12	120916077	120997237	263	19	120000	-1.5463	0.93899	COQ5	76353	25kb
ENSG00000022840	12	120946283	121040397	285	20	120000	-1.3911	0.91791	RNF10	76353	25kb
ENSG00000167272	12	120991567	121044201	154	21	120000	-1.0914	0.86244	POP5	76353	25kb
ENSG00000157782	12	121053355	121130127	277	33	120000	-0.51554	0.69691	CABP1	76353	25kb
ENSG00000110917	12	121099672	121164667	235	33	120000	-1.3042	0.90391	MLEC	76353	25kb
ENSG00000175970	12	121123238	121186443	235	29	120000	-0.93866	0.82605	UNC119B	76353	25kb
ENSG00000122971	12	121138538	121202811	232	32	120000	-0.91439	0.81974	ACADS	76353	25kb
ENSG00000157837	12	121175313	121367174	614	29	120000	-0.98309	0.83722	SPPL3	76353	25kb
ENSG00000272214	12	121382641	121435095	191	24	120000	1.1945	0.11613	AC079602.1	76353	25kb
ENSG00000135100	12	121391346	121465315	272	23	120000	0.59422	0.27618	HNF1A	76353	25kb
ENSG00000157895	12	121415225	121479305	234	31	120000	-0.50243	0.69232	C12orf43	76353	25kb
ENSG00000135114	12	121433095	121502045	246	38	120000	-0.68079	0.752	OASL	76353	25kb
ENSG00000089041	12	121545622	121648876	476	36	120000	-0.17559	0.56969	P2RX7	76353	25kb
ENSG00000135124	12	121622660	121696909	254	22	120000	-0.51216	0.69573	P2RX4	76353	25kb
ENSG00000110931	12	121650497	121761111	308	39	120000	0.035452	0.48586	CAMKK2	76353	25kb

ENSG00000089053	12	121721048	121862699	361	36	120000	-0.80077	0.78837	ANAPC5	76353	25kb
ENSG00000170633	12	121812844	121893389	247	19	120000	-1.0825	0.86049	RNF34	76353	25kb
ENSG00000089094	12	121841900	122043920	397	41	120000	-0.61357	0.73025	KDM2B	76353	25kb
ENSG00000182500	12	122039455	122105583	141	20	120000	0.81082	0.20874	ORAI1	76353	25kb
ENSG00000139714	12	122064024	122135537	200	23	120000	0.11547	0.45403	MORN3	76353	25kb
ENSG00000188735	12	122125658	122245907	303	32	120000	-0.13715	0.55454	TMEM120B	76353	25kb
ENSG00000139725	12	122190664	122266309	186	21	120000	-0.24206	0.59563	RHOF	76353	25kb
ENSG00000139718	12	122217086	122295562	151	19	120000	-0.27636	0.60887	SETD1B	76353	25kb
ENSG00000158104	12	122252433	122326502	146	19	120000	-1.0053	0.84262	HPD	76353	25kb
ENSG00000110801	12	122301637	122381203	202	22	120000	-1.7239	0.95764	PSMD9	76353	25kb
ENSG00000256950	12	122301715	122384376	215	23	120000	-1.6544	0.95098	RP11-87C12.2	76353	25kb
ENSG00000158023	12	122330768	122466833	357	23	120000	-1.4074	0.92035	WDR66	76353	25kb
ENSG00000110987	12	122432328	122524948	211	29	120000	-1.0273	0.84785	BCL7A	76353	25kb
ENSG00000175727	12	122491628	122656894	328	16	120000	0.18313	0.42735	MLXIP	76353	25kb
ENSG00000158113	12	122627285	122713018	217	25	120000	2.2142	0.013409	LRRC43	76353	25kb
ENSG00000204671	12	122631577	122683746	162	17	120000	1.8052	0.035524	IL31	76353	25kb
ENSG00000176383	12	122663090	122718499	92	17	120000	3.1488	0.00081961	B3GNT4	76353	25kb
ENSG00000184047	12	122667210	122737081	120	18	120000	3.1952	0.00069869	DIABLO	76353	25kb
ENSG00000256861	12	122667326	122775970	200	21	120000	3.2574	0.00056224	RP11-512M8.5	76353	25kb
ENSG00000139719	12	122689111	122776068	157	15	120000	3.2151	0.00065188	VPS33A	76353	25kb
ENSG00000130779	12	122730979	122932179	344	25	120000	2.3694	0.0089084	CLIP1	76353	25kb
ENSG00000033030	12	122932417	123010518	141	13	120000	-0.18858	0.57479	ZCCHC8	76353	25kb
ENSG00000111011	12	122964190	123036547	114	14	120000	-0.086989	0.53466	RSRC2	76353	25kb
ENSG00000184445	12	122986793	123135943	257	18	120000	0.71445	0.23748	KNTC1	76353	25kb
ENSG00000196917	12	123079824	123240390	338	36	120000	0.98921	0.16128	HCAR1	76353	25kb
ENSG00000182782	12	123160840	123212890	136	12	120000	0.63578	0.26246	HCAR2	76353	25kb
ENSG00000255398	12	123174303	123226439	108	12	120000	0.72951	0.23285	HCAR3	76353	25kb
ENSG00000139726	12	123212321	123280611	130	13	120000	0.78296	0.21683	DENR	76353	25kb
ENSG00000130783	12	123233874	123337075	306	16	120000	0.24049	0.40498	CCDC62	76353	25kb
ENSG00000130787	12	123294000	123372507	219	17	120000	-0.080466	0.53207	HIP1R	76353	25kb
ENSG00000139722	12	123324882	123405991	160	17	120000	0.3626	0.35845	VPS37B	76353	25kb
ENSG00000150967	12	123380498	123491196	121	24	120000	0.31788	0.37529	ABCB9	76353	25kb
ENSG00000111325	12	123434127	123489590	56	12	120000	-0.5161	0.69711	OGFOD2	76353	25kb
ENSG00000182196	12	123439607	123492456	52	11	120000	-0.49173	0.68855	ARL6IP4	76353	25kb
ENSG00000090975	12	123443027	123659562	262	22	120000	-0.3914	0.65225	PITPNM2	76353	25kb
ENSG00000051825	12	123611867	123753561	382	13	120000	-0.41516	0.66099	MPHOSPH9	76353	25kb
ENSG00000130921	12	123692463	123767506	223	16	120000	-0.6093	0.72884	C12orf65	76353	25kb
ENSG00000111328	12	123720528	123781881	179	17	120000	-0.45846	0.67669	CDK2AP1	76353	25kb
ENSG00000139697	12	123748656	123874390	407	20	120000	0.056074	0.47764	SBNO1	76353	25kb
ENSG00000183955	12	123843320	123918905	203	26	120000	-0.1865	0.57397	SETD8	76353	25kb
ENSG00000150977	12	123874936	123946264	175	34	120000	0.55553	0.28927	RILPL2	76353	25kb
ENSG00000184209	12	123917188	123982701	144	18	120000	1.6374	0.050769	SNRNP35	76353	25kb
ENSG00000188026	12	123930925	124043265	301	27	120000	2.5146	0.0059583	RILPL1	76353	25kb
ENSG00000086598	12	124044078	124108116	232	19	120000	1.0333	0.15074	TMED2	76353	25kb
ENSG00000111364	12	124061624	124130488	225	19	120000	0.73351	0.23162	DDX55	76353	25kb
ENSG00000111361	12	124079953	124143313	192	18	120000	0.83931	0.20065	EIF2B1	76353	25kb
ENSG00000111358	12	124093375	124172153	248	17	120000	0.6447	0.25956	GTF2H3	76353	25kb
ENSG00000168778	12	124130660	124217948	304	10	120000	0.43244	0.33271	TCTN2	76353	25kb
ENSG00000185344	12	124171865	124271302	317	18	120000	0.79881	0.2122	ATP6V0A2	76353	25kb
ENSG00000197653	12	124222042	124445753	554	43	120000	-0.45016	0.6737	DNAH10	76353	25kb
ENSG00000119242	12	124378207	124482378	234	19	120000	-0.68094	0.75204	CCDC92	76353	25kb
ENSG00000250091	12	124385971	124444531	125	13	120000	-0.62281	0.7333	DNAH10OS	76353	25kb
ENSG00000179195	12	124431392	124524986	179	20	120000	-0.59815	0.72513	ZNF664	76353	25kb
ENSG00000178882	12	124432788	124825570	1171	99	120000	-1.4358	0.92447	FAM101A	76353	25kb
ENSG00000196498	12	124783961	125077135	988	134	120000	-1.5502	0.93945	NCOR2	76353	25kb
ENSG00000073060	12	125236402	125392214	498	90	120000	0.21734	0.41397	SCARB1	76353	25kb
ENSG00000150991	12	125371150	125426914	172	36	120000	0.82635	0.2043	UBC	76353	25kb
ENSG00000150990	12	125406371	125498668	487	55	120000	1.1429	0.12655	DHX37	76353	25kb
ENSG00000184992	12	125453246	125540777	345	38	120000	-0.072986	0.52909	BRI3BP	76353	25kb
ENSG00000081760	12	125524925	125652873	360	39	120000	0.84666	0.19859	AACS	76353	25kb
ENSG00000139364	12	125646382	126171917	1714	166	120000	-0.047541	0.51896	TMEM132B	76353	25kb
ENSG00000181234	12	128726948	129217460	1699	214	120000	1.5574	0.059688	TMEM132C	76353	25kb
ENSG00000139370	12	129252739	129333528	329	27	120000	0.12173	0.45155	SLC15A4	76353	25kb
ENSG00000151948	12	129312972	129494509	763	82	120000	-0.67805	0.75113	GLT1D1	76353	25kb
ENSG00000151952	12	129531270	130413211	3330	321	120000	2.2823	0.011237	TMEM132D	76353	25kb
ENSG00000111432	12	130622004	130675285	258	53	120000	-0.65303	0.74313	FZD10	76353	25kb
ENSG00000125207	12	130797432	130882182	325	40	120000	-1.3065	0.90431	PIWIL1	76353	25kb
ENSG00000060709	12	130855682	131225826	1665	186	120000	-1.8938	0.97087	RIMBP2	76353	25kb
ENSG00000111450	12	131249145	131348811	430	29	120000	2.5841	0.0048815	STX2	76353	25kb
ENSG00000132341	12	131331424	131387223	198	20	120000	3.0899	0.001001	RAN	76353	25kb
ENSG00000111452	12	131413452	131651014	1073	85	120000	-0.78665	0.78426	GPR133	76353	25kb
ENSG00000268091	12	131489221	131539769	234	31	120000	0.57301	0.28332	AC078925.1	76353	25kb
ENSG00000267819	12	131755067	131807763	117	29	120000	0.53717	0.29558	AC092850.1	76353	25kb
ENSG00000061936	12	132170626	132309282	398	33	120000	-0.75166	0.77387	SFSWAP	76353	25kb
ENSG00000198598	12	132287938	132361328	263	33	120000	-0.63631	0.73771	MMP17	76353	25kb
ENSG00000177169	12	132354196	132432712	163	21	120000	-1.1271	0.87016	ULK1	76353	25kb
ENSG00000177192	12	132388745	132453406	125	20	120000	-0.76978	0.77928	PUS1	76353	25kb
ENSG00000183495	12	132409508	132590005	280	19	120000	-0.15412	0.56124	EP400	76353	25kb
ENSG00000185684	12	132543828	132638029	184	18	120000	-0.00062735	0.50025	EP400NL	76353	25kb
ENSG00000185163	12	132596139	132653880	133	16	120000	-0.90566	0.81744	DDX51	76353	25kb
ENSG00000184967	12	132603993	132662013	145	20	120000	-1.074	0.8586	NOC4L	76353	25kb
ENSG00000182870	12	132655924	132930935	685	72	120000	0.17671	0.42987	GALNT9	76353	25kb
ENSG00000269676	12	133024334	133075726	204	39	120000	0.60915	0.27121	MUC8	76353	25kb
ENSG00000112787	12	133041137	133186774	632	51	120000	-0.5936	0.72361	FBRSL1	76353	25kb
ENSG00000187848	12	133170366	133223972	227	21	120000	-0.18903	0.57496	P2RX2	76353	25kb
ENSG00000177084	12	133175348	133288951	367	18	120000	-0.1435	0.55705	POLE	76353	25kb
ENSG00000176894	12	133239192	133322276	249	25	120000	0.12282	0.45113	PXMP2	76353	25kb
ENSG00000256632	12	133239635	133316498	221	22	120000	-0.0060016	0.50239	RP13-672B3.2	76353	25kb
ENSG00000247077	12	133262405	133324228	189	26	120000	0.26573	0.39522	PGAM5	76353	25kb
ENSG00000176915	12	133277254	133363474	321	37	120000	1.0624	0.14404	ANKLE2	76353	25kb
ENSG00000090615	12	133320495	133430444	405	37	120000	2.0394	0.020703	GOLGA3	76353	25kb
ENSG00000072609	12	133373773	133557890	524	26	120000	2.4501	0.0071415	CHFR	76353	25kb
ENSG00000196458	12	133473019	133557892	116	21	120000	2.8571	0.0021375	ZNF605	76353	25kb
ENSG00000196387	12	133631424	133709130	79	10	120000	1.3442	0.089436	ZNF140	76353	25kb
ENSG00000214029	12	133669740	133732059	139	13	120000	1.4095	0.079348	ZNF891	76353	25kb
ENSG00000256223	12	133682161	133761051	211	16	120000	1.2865	0.099129	ZNF10	76353	25kb

ENSG00000256825	12	133682570	133803955	343	16	120000	1.206	0.11391	CTD-2140B24.4	76353	25kb
ENSG00000090612	12	133682570	133808698	353	16	120000	1.1612	0.12279	ZNF268	76353	25kb
ENSG00000255748	12	133762246	133812772	153	14	120000	0.92544	0.17737	AC226150.4	76353	25kb
ENSG00000227059	12	133769898	133837681	202	20	120000	1.3722	0.085002	ANHX	76353	25kb
ENSG00000198033	13	19722910	19780992	436	33	120000	-0.53738	0.7045	TUBA3C	76353	25kb
ENSG00000121388	13	19731173	19786496	417	28	120000	-0.77356	0.78041	RP11-408E5.4	76353	25kb
ENSG00000132958	13	19972017	20135903	411	27	120000	-0.88142	0.81096	TPTE2	76353	25kb
ENSG00000196199	13	20182788	20272599	180	18	120000	0.28152	0.38915	MPHOSPH8	76353	25kb
ENSG00000121390	13	20223896	20382142	358	21	120000	0.54327	0.29347	PSPC1	76353	25kb
ENSG00000269591	13	20243547	20294240	88	13	120000	0.4168	0.33841	AL354808.2	76353	25kb
ENSG00000132950	13	20372622	20462776	308	12	120000	0.58775	0.27835	ZMYM5	76353	25kb
ENSG00000121741	13	20507810	20690968	378	27	120000	-0.88336	0.81148	ZMYM2	76353	25kb
ENSG00000121743	13	20687394	20760188	320	54	120000	-1.1017	0.86471	GJA3	76353	25kb
ENSG00000165474	13	20736609	20792037	209	37	120000	-0.6478	0.74144	GJB2	76353	25kb
ENSG00000121742	13	20771110	20831534	214	32	120000	-1.5528	0.93976	GJB6	76353	25kb
ENSG00000165475	13	20952806	21124996	642	67	120000	-1.3263	0.90763	CRYL1	76353	25kb
ENSG00000032742	13	21115585	21290503	471	29	120000	0.14399	0.44275	IFT88	76353	25kb
ENSG00000172458	13	21251266	21322237	223	27	120000	0.78481	0.21628	IL17D	76353	25kb
ENSG00000150456	13	21277870	21373097	281	18	120000	0.47442	0.3176	N6AMT2	76353	25kb
ENSG00000132953	13	21326469	21502187	451	23	120000	-0.18833	0.57469	XPO4	76353	25kb
ENSG00000150457	13	21522171	21660686	430	40	120000	0.38185	0.35129	LATS2	76353	25kb
ENSG00000150459	13	21689653	21748221	228	27	120000	0.3826	0.35101	SAP18	76353	25kb
ENSG00000165480	13	21702734	21775741	225	25	120000	-0.097961	0.53902	SKA3	76353	25kb
ENSG00000173141	13	21725784	21778223	158	20	120000	-0.58082	0.71932	MRP63	76353	25kb
ENSG00000180776	13	21925263	22058509	342	23	120000	-2.0199	0.97831	ZDHHC20	76353	25kb
ENSG00000165487	13	22041836	22203353	430	28	120000	-0.84483	0.8009	MICU2	76353	25kb
ENSG00000102678	13	22220522	22303637	187	36	120000	-0.57514	0.7174	FGF9	76353	25kb
ENSG00000102683	13	23730091	23924304	1096	81	120000	-1.9346	0.97348	SGCG	76353	25kb
ENSG00000151835	13	23877965	24032841	572	52	120000	-0.41601	0.6613	SACS	76353	25kb
ENSG00000127863	13	24119509	24275232	523	42	120000	-1.2248	0.88968	TNFRSF19	76353	25kb
ENSG00000027001	13	24279328	24488558	652	32	120000	-0.15368	0.56107	MIPEP	76353	25kb
ENSG00000268735	13	24297243	24348901	137	16	120000	-0.47231	0.68165	AL139080.1	76353	25kb
ENSG00000205863	13	24440238	24501794	249	32	120000	0.13844	0.44495	C1QTNF9B	76353	25kb
ENSG00000273167	13	24528839	24920169	1636	187	120000	-1.2426	0.893	RP11-307N16.6	76353	25kb
ENSG00000182957	13	24528944	24906212	1591	185	120000	-1.2027	0.88545	SPATA13	76353	25kb
ENSG00000240654	13	24856304	24921673	217	33	120000	-0.9192	0.82101	C1QTNF9	76353	25kb
ENSG00000205850	13	24870264	24920566	180	25	120000	-1.0179	0.84564	AL359736.1	76353	25kb
ENSG00000102699	13	24970064	25111948	662	20	120000	-0.81306	0.79191	PARP4	76353	25kb
ENSG00000075673	13	25229549	25310921	394	44	120000	-0.45346	0.67489	ATP12A	76353	25kb
ENSG00000132972	13	25313290	25479059	464	27	120000	0.19363	0.42323	RNF17	76353	25kb
ENSG00000151849	13	25432171	25522018	335	26	120000	-0.15533	0.56172	CENPJ	76353	25kb
ENSG00000269099	13	25566541	25616675	206	22	120000	-0.17475	0.56936	LSP1	76353	25kb
ENSG00000151846	13	25645300	25698389	168	17	120000	-0.71966	0.76413	PABPC3	76353	25kb
ENSG00000165566	13	25710822	25771426	156	36	120000	0.96466	0.16736	AMER2	76353	25kb
ENSG00000139505	13	25777307	25887147	278	29	120000	0.2708	0.39327	MTMR6	76353	25kb
ENSG00000139496	13	25850662	25948938	149	33	120000	1.2497	0.1057	NUPL1	76353	25kb
ENSG00000132932	13	25921209	26624989	2239	143	120000	0.68239	0.2475	ATP8A2	76353	25kb
ENSG00000215549	13	26417061	26480095	130	13	120000	0.42097	0.33689	AL138815.1	76353	25kb
ENSG00000268344	13	26427422	26477679	83	14	120000	0.15954	0.43662	AL138815.2	76353	25kb
ENSG00000180730	13	26593735	26650169	260	33	120000	-0.35081	0.63713	SHISA2	76353	25kb
ENSG00000127870	13	26681253	26821791	524	41	120000	-0.28095	0.61063	RNF6	76353	25kb
ENSG00000132964	13	26803276	27004375	521	23	120000	-0.83666	0.79861	CDK8	76353	25kb
ENSG00000132970	13	27106840	27288085	599	33	120000	-0.49981	0.6914	WASF3	76353	25kb
ENSG00000132975	13	27304341	27359922	210	22	120000	-0.40654	0.65783	GPR12	76353	25kb
ENSG00000152484	13	27615293	27771033	440	37	120000	-1.0135	0.8446	USP12	76353	25kb
ENSG00000122026	13	27800446	27855828	140	24	120000	-1.0149	0.84492	RPL21	76353	25kb
ENSG00000122035	13	27819464	27872827	157	24	120000	-0.088505	0.53526	RASL11A	76353	25kb
ENSG00000122034	13	27973681	28034958	260	23	120000	-0.73445	0.76866	GTF3A	76353	25kb
ENSG00000122033	13	27984776	28049739	268	19	120000	-0.75268	0.77418	MTIF3	76353	25kb
ENSG00000139517	13	28095050	28219541	420	40	120000	-0.0603	0.52404	LNX2	76353	25kb
ENSG00000186184	13	28169903	28266548	413	41	120000	-0.14455	0.55747	POLR1D	76353	25kb
ENSG00000169840	13	28341780	28393905	155	27	120000	-0.56264	0.71316	GSX1	76353	25kb
ENSG00000139515	13	28469157	28525368	210	25	120000	-0.41379	0.66048	PDX1	76353	25kb
ENSG00000180389	13	28494343	28544727	153	21	120000	-0.84708	0.80152	ATP5EP2	76353	25kb
ENSG00000165556	13	28511274	28570276	195	25	120000	-1.4714	0.92941	CDX2	76353	25kb
ENSG00000183463	13	28526851	28587791	179	31	120000	-1.6907	0.95456	URAD	76353	25kb
ENSG00000122025	13	28552411	28699729	563	37	120000	-0.072875	0.52905	FLT3	76353	25kb
ENSG00000152520	13	28687643	28894475	363	27	120000	-0.23557	0.59312	PAN3	76353	25kb
ENSG00000102755	13	28849489	29094265	644	64	120000	-0.75851	0.77593	FLT1	76353	25kb
ENSG00000132963	13	29208241	29278062	179	24	120000	1.1691	0.12119	POMP	76353	25kb
ENSG00000139508	13	29249201	29318107	149	21	120000	1.0327	0.15087	SLC46A3	76353	25kb
ENSG00000132938	13	29573748	30102892	1859	101	120000	-1.3182	0.90628	MTUS2	76353	25kb
ENSG00000139514	13	30058547	30194825	471	45	120000	0.42668	0.33481	SLC7A1	76353	25kb
ENSG00000122042	13	30313508	30449821	340	20	120000	-0.83656	0.79858	UBL3	76353	25kb
ENSG00000102781	13	30751767	30906621	440	49	120000	0.23979	0.40525	KATNAL1	76353	25kb
ENSG00000189403	13	31007884	31216734	448	75	120000	1.7135	0.043307	HMGB1	76353	25kb
ENSG00000132952	13	31166830	31258686	184	20	120000	0.87442	0.19095	USPL1	76353	25kb
ENSG00000132965	13	31284645	31363556	329	40	120000	0.1078	0.45708	ALOX5AP	76353	25kb
ENSG00000102802	13	31455328	31524709	232	36	120000	0.87993	0.18945	MEDAG	76353	25kb
ENSG00000175664	13	31481840	31574639	265	31	120000	0.60756	0.27174	TEX26	76353	25kb
ENSG00000120694	13	31685762	31761525	211	26	120000	-1.5678	0.94154	HSPH1	76353	25kb
ENSG00000187676	13	31749073	31931413	473	33	120000	-0.95429	0.83003	B3GALTL	76353	25kb
ENSG00000133105	13	32288674	32402009	341	27	120000	-1.3561	0.91247	RXFP2	76353	25kb
ENSG00000073910	13	32580437	32895794	947	88	120000	-0.032072	0.51279	FRY	76353	25kb
ENSG00000189167	13	32852837	32914481	174	27	120000	-0.345	0.63495	ZAR1L	76353	25kb
ENSG00000139618	13	32864611	32998805	345	35	120000	0.42287	0.33619	BRCA2	76353	25kb
ENSG00000139597	13	32949861	33027315	163	30	120000	0.4651	0.32093	N4BP2L1	76353	25kb
ENSG00000244754	13	32981554	33137970	236	25	120000	0.0044264	0.49823	N4BP2L2	76353	25kb
ENSG00000083642	13	33135564	33377157	435	13	120000	0.93115	0.17589	PDS5B	76353	25kb
ENSG00000133116	13	33565207	33665282	366	29	120000	0.89063	0.18656	KL	76353	25kb
ENSG00000133121	13	33652272	33949767	858	66	120000	0.43481	0.33185	STARD13	76353	25kb
ENSG00000133119	13	34367186	34565695	542	34	120000	-1.6574	0.95128	RFC3	76353	25kb
ENSG00000172915	13	35491424	36272159	1986	68	120000	0.10194	0.4594	NBEA	76353	25kb
ENSG00000180660	13	36022926	36075832	120	26	120000	-1.8593	0.96851	MAB21L1	76353	25kb
ENSG00000133083	13	36320478	36730443	1347	108	120000	0.81281	0.20816	DCLK1	76353	25kb
ENSG00000120669	13	36717345	36896979	665	31	120000	-0.51435	0.6965	SOHLH2	76353	25kb

ENSG00000250709	13	36717931	36896979	664	31	120000	-0.53338	0.70312	CCDC169-SOHLH2	76353	25kb
ENSG00000242715	13	36776182	36896977	486	12	120000	-0.17982	0.57135	CCDC169	76353	25kb
ENSG00000133104	13	36850775	36969317	299	21	120000	-0.76459	0.77774	SPG20	76353	25kb
ENSG00000120664	13	36895568	36968738	178	23	120000	-1.2426	0.89299	SPG20OS	76353	25kb
ENSG00000133101	13	36980967	37042019	140	24	120000	-1.1893	0.88284	CCNA1	76353	25kb
ENSG00000180440	13	37223049	37296976	166	41	120000	1.0084	0.15662	SERTM1	76353	25kb
ENSG00000133111	13	37368361	37428241	128	21	120000	-0.40342	0.65668	RFXAP	76353	25kb
ENSG00000120693	13	37393968	37519902	278	38	120000	0.046154	0.48159	SMAD9	76353	25kb
ENSG00000120697	13	37498912	37599398	171	17	120000	-0.36646	0.64299	ALG5	76353	25kb
ENSG00000120699	13	37547953	37608750	82	11	120000	-0.084676	0.53374	EXOSC8	76353	25kb
ENSG00000102710	13	37558449	37658850	157	11	120000	-0.83053	0.79688	SUPT20H	76353	25kb
ENSG00000180138	13	37652398	37704803	168	14	120000	-1.0522	0.85364	CSNK1A1L	76353	25kb
ENSG00000133110	13	38111720	38197981	313	21	120000	-1.3711	0.91482	POSTN	76353	25kb
ENSG00000133107	13	38185773	38469562	971	68	120000	-0.69334	0.75595	TRPC4	76353	25kb
ENSG00000120686	13	38898986	38962140	112	5	120000	-0.25446	0.60043	UFM1	76353	25kb
ENSG00000150893	13	39236266	39485074	694	64	120000	0.67535	0.24973	FREM2	76353	25kb
ENSG00000133115	13	39515062	39590203	404	28	120000	0.20696	0.41802	STOML3	76353	25kb
ENSG00000120685	13	39559003	39637252	184	15	120000	0.91545	0.17998	PROSER1	76353	25kb
ENSG00000188811	13	39587443	39649246	123	15	120000	0.61349	0.26977	NHLRC3	76353	25kb
ENSG00000183722	13	39892029	40202665	1004	82	120000	0.72759	0.23343	LHFP	76353	25kb
ENSG00000133103	13	40204764	40390802	517	21	120000	0.05701	0.47727	COG6	76353	25kb
ENSG00000269120	13	41086138	41136323	135	27	120000	-0.88678	0.8124	AL133318.1	76353	25kb
ENSG00000150907	13	41104804	41265734	344	29	120000	-0.50226	0.69226	FOXO1	76353	25kb
ENSG00000102738	13	41278432	41370309	130	17	120000	-0.60154	0.72626	MRPS31	76353	25kb
ENSG00000102743	13	41338548	41409247	164	20	120000	0.11112	0.45576	SLC25A15	76353	25kb
ENSG00000120690	13	41481056	41660576	436	27	120000	1.0138	0.15535	ELF1	76353	25kb
ENSG00000120688	13	41610410	41683137	87	20	120000	-0.64124	0.73932	WBP4	76353	25kb
ENSG00000165572	13	41676705	41731882	89	15	120000	-1.3573	0.91265	KBTBD6	76353	25kb
ENSG00000120696	13	41738969	41793702	84	22	120000	-0.35285	0.6379	KBTBD7	76353	25kb
ENSG00000120662	13	41765505	41862742	236	24	120000	0.096553	0.46154	MTRF1	76353	25kb
ENSG00000172766	13	41860341	41976166	261	12	120000	0.38653	0.34955	NAA16	76353	25kb
ENSG00000102760	13	42006695	42070018	200	22	120000	0.33329	0.36946	RGCC	76353	25kb
ENSG00000102763	13	42115973	42560256	1075	54	120000	-1.0544	0.85414	VWA8	76353	25kb
ENSG00000102780	13	42589176	42855714	951	52	120000	-2.0629	0.98044	DGKH	76353	25kb
ENSG00000023516	13	42821289	42922396	279	22	120000	-0.87585	0.80944	AKAP11	76353	25kb
ENSG00000120659	13	43111872	43207149	249	28	120000	0.11412	0.45457	TNFSF11	76353	25kb
ENSG00000179813	13	43330686	43390683	160	16	120000	0.31827	0.37514	FAM216B	76353	25kb
ENSG00000133106	13	43435524	43591407	468	65	120000	1.295	0.097654	EPSTI1	76353	25kb
ENSG00000120675	13	43572339	43708045	329	18	120000	1.4802	0.06941	DNAJC15	76353	25kb
ENSG00000120658	13	43762654	44386044	1560	90	120000	-0.9877	0.83835	ENOX1	76353	25kb
ENSG00000151773	13	44373045	44478827	418	33	120000	1.271	0.10186	CCDC122	76353	25kb
ENSG00000179630	13	44428420	44493068	265	18	120000	1.3633	0.086396	LACC1	76353	25kb
ENSG00000139656	13	44692679	44760393	119	18	120000	-0.46373	0.67858	SMIM2	76353	25kb
ENSG00000151778	13	44922801	44996850	142	19	120000	0.15673	0.43773	SERP2	76353	25kb
ENSG00000102804	13	44982655	45176283	384	18	120000	-0.98744	0.83829	TSC22D1	76353	25kb
ENSG00000083635	13	45488384	45588618	162	28	120000	-0.091762	0.53656	NUFIP1	76353	25kb
ENSG00000133114	13	45538687	45632746	215	26	120000	-0.19929	0.57898	GPALPP1	76353	25kb
ENSG00000188342	13	45669650	45883237	383	19	120000	-1.5204	0.93579	GTF2F2	76353	25kb
ENSG00000180332	13	45741988	45800175	96	10	120000	-1.2735	0.89858	KCTD4	76353	25kb
ENSG00000133112	13	45886008	45940505	204	24	120000	0.12614	0.44981	TPT1	76353	25kb
ENSG00000174032	13	45942451	46017590	160	19	120000	0.17541	0.43038	SLC25A30	76353	25kb
ENSG00000136152	13	46014060	46135765	338	23	120000	-0.38876	0.65127	COG3	76353	25kb
ENSG00000165837	13	46083657	46214874	341	34	120000	-0.69334	0.75595	FAM194B	76353	25kb
ENSG00000174015	13	46251446	46313694	158	31	120000	1.6109	0.053605	SPERT	76353	25kb
ENSG00000215475	13	46329405	46450871	398	48	120000	0.93467	0.17498	SIAH3	76353	25kb
ENSG00000123200	13	46503600	46651894	334	16	120000	0.26287	0.39633	ZC3H13	76353	25kb
ENSG00000080618	13	46602321	46704211	294	20	120000	1.1964	0.11577	CPB2	76353	25kb
ENSG00000136167	13	46675055	46811006	426	53	120000	0.98607	0.16205	LCP1	76353	25kb
ENSG00000173988	13	46761083	46876501	366	31	120000	0.15918	0.43676	LRRC63	76353	25kb
ENSG00000102445	13	46891139	47037325	356	19	120000	-0.50392	0.69284	KIAA0226L	76353	25kb
ENSG00000136141	13	47102303	47352175	659	45	120000	1.7164	0.043045	LRCH1	76353	25kb
ENSG00000139684	13	47320391	47396367	159	23	120000	-0.13474	0.55359	ESD	76353	25kb
ENSG00000102468	13	47380685	47496169	379	48	120000	-0.66298	0.74633	HTR2A	76353	25kb
ENSG00000136143	13	48485622	48637125	590	23	120000	2.3851	0.008538	SUCLA2	76353	25kb
ENSG00000136159	13	48586703	48646358	210	24	120000	2.2379	0.012613	NUDT15	76353	25kb
ENSG00000136146	13	48602459	48694267	306	23	120000	1.752	0.039886	MED4	76353	25kb
ENSG00000136156	13	48782294	48862063	106	15	120000	0.40437	0.34297	ITM2B	76353	25kb
ENSG00000139687	13	48852887	49081122	283	19	120000	-0.56494	0.71394	RB1	76353	25kb
ENSG00000139679	13	48938707	49043840	135	15	120000	-0.64013	0.73896	LPAR6	76353	25kb
ENSG00000136161	13	49038095	49132369	106	14	120000	-0.46778	0.68003	RCBTB2	76353	25kb
ENSG00000152207	13	49255951	49308498	133	22	120000	0.65811	0.25523	CYSLTR2	76353	25kb
ENSG00000102531	13	49525048	49808915	372	22	120000	-0.68124	0.75214	FNDC3A	76353	25kb
ENSG00000102539	13	49769474	49821513	55	10	120000	-0.44235	0.67088	MLNR	76353	25kb
ENSG00000102543	13	49797047	49892618	159	14	120000	-0.21489	0.58507	CDADC1	76353	25kb
ENSG00000102547	13	49857786	50043262	484	47	120000	0.049458	0.48028	CAB39L	76353	25kb
ENSG00000215462	13	49982495	50045554	196	28	120000	0.23096	0.40867	AL136218.1	76353	25kb
ENSG00000136169	13	49993429	50094138	261	19	120000	0.68555	0.2465	SETDB2	76353	25kb
ENSG00000136147	13	50044746	50128123	302	20	120000	0.27048	0.3934	PHF11	76353	25kb
ENSG00000136144	13	50081082	50184719	468	24	120000	-0.071318	0.52843	RCBTB1	76353	25kb
ENSG00000152213	13	50177435	50233008	185	25	120000	-0.42915	0.66609	ARL11	76353	25kb
ENSG00000123179	13	50209859	50290611	274	27	120000	-0.83881	0.79921	EBPL	76353	25kb
ENSG00000102753	13	50248447	50392057	473	34	120000	-1.1128	0.86711	KPNA3	76353	25kb
ENSG00000123178	13	50461842	50535626	161	23	120000	-0.33525	0.63128	SPRYD7	76353	25kb
ENSG00000204977	13	50545024	50619617	124	15	120000	-0.23561	0.59313	TRIM13	76353	25kb
ENSG00000198553	13	50564390	50620058	100	15	120000	-0.27508	0.60837	KCNRG	76353	25kb
ENSG00000176124	13	50631307	51322372	1748	109	120000	-0.47638	0.6831	DLEU1	76353	25kb
ENSG00000186047	13	51260144	51443075	483	46	120000	-0.27406	0.60798	DLEU7	76353	25kb
ENSG00000136104	13	51458814	51569592	235	40	120000	-0.23853	0.59426	RNASEH2B	76353	25kb
ENSG00000150510	13	51771503	51883377	257	44	120000	-0.037915	0.51512	FAM124A	76353	25kb
ENSG00000253309	13	51884909	51963871	160	13	120000	1.1844	0.11812	SERPINE3	76353	25kb
ENSG00000102786	13	51903213	52053400	287	18	120000	1.7112	0.043523	INTS6	76353	25kb
ENSG00000139668	13	52133644	52361171	290	31	120000	-0.15921	0.56325	WDFY2	76353	25kb
ENSG00000102796	13	52317129	52403293	158	31	120000	-0.48725	0.68696	DHRS12	76353	25kb
ENSG00000123171	13	52411117	52465366	167	24	120000	2.4164	0.0078365	CCDC70	76353	25kb
ENSG00000123191	13	52481809	52610630	283	20	120000	2.2594	0.01193	ATP7B	76353	25kb
ENSG00000253710	13	52561534	52628800	140	16	120000	1.3244	0.092685	ALG11	76353	25kb

ENSG00000253797	13	52573827	52632736	106	14	120000	1.3539	0.087879	UTP14C	76353	25kb
ENSG00000197168	13	52586093	52728214	289	19	120000	1.5047	0.066197	NEK5	76353	25kb
ENSG00000136098	13	52681775	52758996	166	21	120000	1.7788	0.037639	NEK3	76353	25kb
ENSG00000136114	13	52926305	53005629	191	10	120000	1.3686	0.085562	THSD1	76353	25kb
ENSG00000136100	13	52961737	53049763	194	11	120000	1.4066	0.079776	VPS36	76353	25kb
ENSG00000136108	13	53004564	53075763	128	10	120000	1.2446	0.10665	CKAP2	76353	25kb
ENSG00000139675	13	53166605	53242919	238	8	120000	0.50825	0.30564	HNRNPA1L2	76353	25kb
ENSG00000165416	13	53201844	53300044	276	10	120000	0.21823	0.41362	SUGT1	76353	25kb
ENSG00000136110	13	53252399	53338947	267	22	120000	-0.04243	0.51692	LECT1	76353	25kb
ENSG00000136099	13	53393109	53447775	180	28	120000	0.19077	0.42435	PCDH8	76353	25kb
ENSG00000102837	13	53577894	53651192	213	43	120000	-2.1671	0.98489	OLFM4	76353	25kb
ENSG00000204919	13	57690052	57743073	65	14	120000	-0.144	0.55725	PRR20A	76353	25kb
ENSG00000204918	13	57696622	57749643	58	12	120000	0.010565	0.49579	PRR20B	76353	25kb
ENSG00000229665	13	57703195	57756216	47	11	120000	0.30278	0.38103	PRR20C	76353	25kb
ENSG00000227151	13	57709766	57762787	39	9	120000	0.35879	0.35988	PRR20D	76353	25kb
ENSG00000234278	13	57716331	57769352	30	7	120000	0.46994	0.3192	PRR20E	76353	25kb
ENSG00000118946	13	58180944	58328445	303	37	120000	1.3025	0.096377	PCDH17	76353	25kb
ENSG00000139734	13	60214717	60763121	1350	35	120000	-0.72988	0.76727	DIAPH3	76353	25kb
ENSG00000083544	13	60945591	61173012	542	25	120000	-0.36177	0.64124	TDRD3	76353	25kb
ENSG00000197991	13	61958818	62027220	250	29	120000	0.78405	0.21651	PCDH20	76353	25kb
ENSG00000226974	13	64295934	64346323	79	10	120000	0.0065527	0.49739	AL445989.1	76353	25kb
ENSG00000184226	13	66851967	67829468	2284	136	120000	-0.10269	0.5409	PCDH9	76353	25kb
ENSG00000150361	13	70249726	70707591	1922	77	120000	-1.0667	0.85694	KLHL1	76353	25kb
ENSG00000165659	13	71987098	72466330	824	88	120000	0.42743	0.33453	DACH1	76353	25kb
ENSG00000204899	13	73257495	73326825	220	25	120000	-0.9219	0.82171	MZT1	76353	25kb
ENSG00000136122	13	73277061	73355336	209	25	120000	-0.96203	0.83198	BORA	76353	25kb
ENSG00000083520	13	73304540	73381234	186	28	120000	-0.89307	0.81409	DIS3	76353	25kb
ENSG00000083535	13	73331197	73615591	713	31	120000	-0.5378	0.70464	PIBF1	76353	25kb
ENSG00000102554	13	73604114	73676676	218	42	120000	0.67276	0.25055	KLF5	76353	25kb
ENSG00000118922	13	74235226	74733394	1525	147	120000	-1.4431	0.9255	KLF12	76353	25kb
ENSG00000177596	13	74962094	75018252	264	26	120000	-0.76966	0.77925	AL355390.1	76353	25kb
ENSG00000136111	13	75833808	76081250	723	85	120000	-0.41257	0.66004	TBC1D4	76353	25kb
ENSG00000188243	13	76074350	76148575	212	24	120000	-0.88515	0.81196	COMMD6	76353	25kb
ENSG00000118939	13	76098619	76205085	322	20	120000	-1.1063	0.8657	UCHL3	76353	25kb
ENSG00000136153	13	76169570	76459004	807	57	120000	-1.6196	0.94734	LMO7	76353	25kb
ENSG00000178734	13	76420187	76482947	266	22	120000	-0.71966	0.76413	C13orf45	76353	25kb
ENSG00000178695	13	77429312	77485540	173	31	120000	3.2399	0.00059785	KCTD12	76353	25kb
ENSG00000102794	13	77497632	77557777	203	35	120000	2.6958	0.0035115	IRG1	76353	25kb
ENSG00000102805	13	77539795	77601652	188	22	120000	1.9897	0.023313	CLN5	76353	25kb
ENSG00000005812	13	77541740	77626330	233	27	120000	1.7573	0.039431	FBXL3	76353	25kb
ENSG00000005810	13	77593792	77926185	644	32	120000	0.20233	0.41983	MYCBP2	76353	25kb
ENSG00000136155	13	78084809	78244398	397	34	120000	-0.42023	0.66284	SCEL	76353	25kb
ENSG00000139737	13	78247023	78363377	268	29	120000	-0.59742	0.72489	SLAIN1	76353	25kb
ENSG00000136160	13	78444616	78518903	237	24	120000	-0.43198	0.66712	EDNRB	76353	25kb
ENSG00000152192	13	79147497	79202673	114	15	120000	1.6589	0.04857	POU4F1	76353	25kb
ENSG00000152193	13	79163426	79258314	253	22	120000	1.0716	0.14196	RNF219	76353	25kb
ENSG00000139746	13	79860962	80005612	360	10	120000	0.051913	0.4793	RBM26	76353	25kb
ENSG00000102471	13	80030287	80155210	229	20	120000	-0.83473	0.79806	NDFIP2	76353	25kb
ENSG00000136158	13	80885111	80940086	117	25	120000	-1.2497	0.8943	SPRY2	76353	25kb
ENSG00000178235	13	84426344	84481528	95	18	120000	-1.0709	0.85789	SLITRK1	76353	25kb
ENSG00000184564	13	86341925	86398623	307	16	120000	0.5676	0.28515	SLITRK6	76353	25kb
ENSG00000165300	13	88299870	88356871	147	19	120000	-0.47505	0.68262	SLITRK5	76353	25kb
ENSG00000179399	13	92025929	93544490	4283	200	120000	3.0728	0.0010604	GPC5	76353	25kb
ENSG00000183098	13	93854095	95084655	3448	204	120000	-0.19588	0.57765	GPC6	76353	25kb
ENSG00000080166	13	95064558	95156936	298	29	120000	-0.3093	0.62145	DCT	76353	25kb
ENSG00000088451	13	95201308	95273511	171	18	120000	-0.54567	0.70735	TGDS	76353	25kb
ENSG00000152749	13	95229157	95311899	193	24	120000	-0.60295	0.72673	GPR180	76353	25kb
ENSG00000125285	13	95336886	95389389	131	21	120000	-0.35103	0.63722	SOX21	76353	25kb
ENSG00000125257	13	95647083	95978687	1371	115	120000	-0.55437	0.71034	ABCC4	76353	25kb
ENSG00000134873	13	96060858	96257013	689	47	120000	-1.0427	0.85146	CLDN10	76353	25kb
ENSG00000134874	13	96205457	96321957	185	31	120000	0.16622	0.43399	DZIP1	76353	25kb
ENSG00000102580	13	96304393	96472243	190	29	120000	0.66362	0.25347	DNAJC3	76353	25kb
ENSG00000102595	13	96428834	96730736	592	25	120000	0.28063	0.3895	UGGT2	76353	25kb
ENSG00000185352	13	96718093	97510671	1872	75	120000	-0.33477	0.6311	HS6ST3	76353	25kb
ENSG00000165621	13	97612973	97671984	174	19	120000	-1.0386	0.85051	OXGR1	76353	25kb
ENSG00000139793	13	97848688	98071374	472	45	120000	-0.91212	0.81915	MBNL2	76353	25kb
ENSG00000125249	13	98061476	98146382	127	17	120000	0.33344	0.3694	RAP2A	76353	25kb
ENSG00000065150	13	98580912	98701551	222	31	120000	-1.5685	0.94161	IPO5	76353	25kb
ENSG00000152767	13	98769816	99127027	1332	156	120000	0.69675	0.24298	FARP1	76353	25kb
ENSG00000139797	13	98803039	98854519	148	29	120000	0.54607	0.29251	RNF113B	76353	25kb
ENSG00000102572	13	99077455	99255194	610	41	120000	0.3623	0.35857	STK24	76353	25kb
ENSG00000088386	13	99311055	99429908	539	71	120000	-0.057909	0.52309	SLC15A1	76353	25kb
ENSG00000088387	13	99420741	99763879	1215	41	120000	0.36499	0.35756	DOCK9	76353	25kb
ENSG00000134882	13	99828028	100063688	579	29	120000	-0.28885	0.61365	UBAC2	76353	25kb
ENSG00000125245	13	99881968	99938998	100	20	120000	-0.28199	0.61102	GPR18	76353	25kb
ENSG00000169508	13	99921784	99984659	143	26	120000	-0.18765	0.57442	GPR183	76353	25kb
ENSG00000125304	13	100128671	100241260	289	23	120000	0.74819	0.22717	TM9SF2	76353	25kb
ENSG00000125246	13	100233919	100574387	1004	95	120000	-1.377	0.91574	CLYBL	76353	25kb
ENSG00000139800	13	100590218	100649163	143	30	120000	-1.6582	0.95137	ZIC5	76353	25kb
ENSG00000043355	13	100609026	100664018	118	31	120000	-1.6458	0.9501	ZIC2	76353	25kb
ENSG00000175198	13	100716269	101207686	994	54	120000	-0.2949	0.61596	PCCA	76353	25kb
ENSG00000134864	13	101158810	101266782	182	20	120000	-1.1357	0.87197	GGACT	76353	25kb
ENSG00000125247	13	101231181	101352347	333	24	120000	-0.73336	0.76833	TMTC4	76353	25kb
ENSG00000102452	13	101681130	102093843	1474	98	120000	-0.25327	0.59997	NALCN	76353	25kb
ENSG00000198542	13	102079966	102400456	952	63	120000	0.62706	0.26531	ITGBL1	76353	25kb
ENSG00000102466	13	102347134	103079124	1941	125	120000	1.9015	0.028621	FGF14	76353	25kb
ENSG00000134900	13	103224353	103356521	537	28	120000	0.68966	0.24521	TPP2	76353	25kb
ENSG00000139780	13	103313094	103371854	255	36	120000	-0.14601	0.55804	METTL21C	76353	25kb
ENSG00000175820	13	103356801	103414159	140	20	120000	1.1793	0.11914	CCDC168	76353	25kb
ENSG00000151287	13	103393340	103451161	132	16	120000	0.73846	0.23012	TEX30	76353	25kb
ENSG00000134901	13	103411631	103476357	161	18	120000	-0.030069	0.51199	KDELC1	76353	25kb
ENSG00000134897	13	103426399	103518885	238	23	120000	-0.33929	0.6328	BIVM	76353	25kb
ENSG00000270181	13	103434704	103549748	305	22	120000	-0.5804	0.71918	BIVM-ERCC5	76353	25kb
ENSG00000134899	13	103472194	103553345	220	27	120000	-0.63396	0.73694	ERCC5	76353	25kb
ENSG00000125255	13	103671350	103744196	306	37	120000	-0.48184	0.68504	SLC10A2	76353	25kb
ENSG00000182346	13	106093216	106168383	305	23	120000	-0.76506	0.77788	DAOA	76353	25kb

ENSG00000125266	13	107117079	107212462	178	44	120000	-0.76963	0.77924	EFNB2	76353	25kb
ENSG00000134884	13	107169021	107245512	117	21	120000	-0.17328	0.56879	ARGLU1	76353	25kb
ENSG00000204442	13	107795883	108544083	2613	238	120000	-0.040991	0.51635	FAM155A	76353	25kb
ENSG00000174405	13	108834787	108895716	137	21	120000	0.60281	0.27332	LIG4	76353	25kb
ENSG00000139826	13	108845727	108911603	145	19	120000	0.723	0.23484	ABHD13	76353	25kb
ENSG00000102524	13	108878588	108985832	257	48	120000	0.6106	0.27073	TNFSF13B	76353	25kb
ENSG00000041515	13	109223500	109885355	2614	186	120000	-0.44285	0.67106	MYO16	76353	25kb
ENSG00000185950	13	110381184	110463915	304	32	120000	-0.12626	0.55024	IRS2	76353	25kb
ENSG00000187498	13	110776318	110984496	959	113	120000	-0.90953	0.81847	COL4A1	76353	25kb
ENSG00000134871	13	110933159	111190374	1268	135	120000	0.15247	0.43941	COL4A2	76353	25kb
ENSG00000139832	13	111150417	111239080	388	45	120000	-0.452	0.67437	RAB20	76353	25kb
ENSG00000213995	13	111242881	111317340	338	24	120000	-0.13848	0.55507	CARKD	76353	25kb
ENSG00000134905	13	111268759	111390950	468	28	120000	0.0080538	0.49679	CARS2	76353	25kb
ENSG00000153487	13	111340083	111398421	148	15	120000	0.53237	0.29724	ING1	76353	25kb
ENSG00000255874	13	111496578	111547162	159	22	120000	-0.41545	0.6611	LINC00346	76353	25kb
ENSG00000088448	13	111505887	111592416	275	24	120000	-1.254	0.89508	ANKRD10	76353	25kb
ENSG00000102606	13	111741906	111983084	800	56	120000	-0.29518	0.61607	ARHGEF7	76353	25kb
ENSG00000153495	13	111943531	112021596	337	42	120000	0.22669	0.41033	TEX29	76353	25kb
ENSG00000204398	13	112215548	112349955	541	46	120000	-0.48698	0.68686	RP11-65D24.2	76353	25kb
ENSG00000182968	13	112696913	112751020	89	23	120000	0.24366	0.40375	SOX1	76353	25kb
ENSG00000153498	13	113005633	113114003	541	39	120000	1.4662	0.071302	SPACA7	76353	25kb
ENSG00000126216	13	113114325	113267481	433	23	120000	2.3238	0.010068	TUBGCP3	76353	25kb
ENSG00000197595	13	113276358	113363811	280	37	120000	-0.12314	0.549	C13orf35	76353	25kb
ENSG00000068650	13	113319643	113566482	755	58	120000	-0.5358	0.70395	ATP11A	76353	25kb
ENSG00000126217	13	113523692	113779053	907	97	120000	-0.74304	0.77127	MCF2L	76353	25kb
ENSG00000268130	13	113730563	113781608	170	32	120000	-0.72649	0.76623	AL137002.1	76353	25kb
ENSG00000057593	13	113735105	113799995	196	39	120000	-0.91159	0.81901	F7	76353	25kb
ENSG00000126218	13	113752128	113828843	175	35	120000	-0.58147	0.71954	F10	76353	25kb
ENSG00000126231	13	113787968	113851694	130	19	120000	-0.03037	0.51211	PROZ	76353	25kb
ENSG00000126226	13	113806891	113888029	177	16	120000	-0.14071	0.55595	PCID2	76353	25kb
ENSG00000139842	13	113837552	113944399	238	19	120000	-0.95851	0.8311	CUL4A	76353	25kb
ENSG00000185896	13	113926556	114002987	121	21	120000	-1.6922	0.9547	LAMP1	76353	25kb
ENSG00000139835	13	113953505	114043446	249	42	120000	-0.71166	0.76166	GRTP1	76353	25kb
ENSG00000153531	13	114051260	114132839	286	46	120000	-0.91844	0.82081	ADPRHL1	76353	25kb
ENSG00000150401	13	114085134	114170267	371	29	120000	-0.85996	0.80509	DCUN1D2	76353	25kb
ENSG00000150403	13	114120310	114229542	505	14	120000	-0.62676	0.73459	TMCO3	76353	25kb
ENSG00000198176	13	114214013	114320785	181	19	120000	-1.0449	0.85196	TFDP1	76353	25kb
ENSG00000186009	13	114278119	114337501	86	17	120000	-1.4685	0.92902	ATP4B	76353	25kb
ENSG00000185974	13	114296470	114465708	177	23	120000	-1.3352	0.90909	GRK1	76353	25kb
ENSG00000184497	13	114437216	114539926	381	29	120000	-1.4125	0.9211	TMEM255B	76353	25kb
ENSG00000183087	13	114498522	114592046	373	35	120000	-0.60756	0.72826	GAS6	76353	25kb
ENSG00000185989	13	114722194	114923086	711	45	120000	1.1422	0.12668	RASA3	76353	25kb
ENSG00000130177	13	114975362	115063198	258	20	120000	-0.91307	0.8194	CDC16	76353	25kb
ENSG00000169062	13	115022059	115096283	174	23	120000	-1.1757	0.88015	UPF3A	76353	25kb
ENSG00000198824	13	115054988	115117796	130	14	120000	-0.98793	0.83841	CHAMP1	76353	25kb
ENSG00000165762	14	20319391	20370468	16	7	120000	1.7219	0.042546	OR4K2	76353	25kb
ENSG00000176281	14	20363741	20414818	82	17	120000	2.2165	0.013328	OR4K5	76353	25kb
ENSG00000155249	14	20378767	20429842	129	20	120000	1.7986	0.036044	OR4K1	76353	25kb
ENSG00000169488	14	20418603	20469818	235	21	120000	1.1268	0.12991	OR4K15	76353	25kb
ENSG00000169484	14	20457419	20508352	222	26	120000	3.7012	0.00010728	OR4K14	76353	25kb
ENSG00000176253	14	20477003	20527919	278	17	120000	4.2603	1.0208e-05	OR4K13	76353	25kb
ENSG00000176246	14	20503204	20554142	260	20	120000	4.1085	1.9911e-05	OR4L1	76353	25kb
ENSG00000176230	14	20560566	20611597	192	15	120000	-0.42916	0.6661	OR4K17	76353	25kb
ENSG00000184394	14	20586895	20637821	114	14	120000	-1.1587	0.8767	OR4N5	76353	25kb
ENSG00000196832	14	20640495	20691605	296	39	120000	-0.22017	0.58713	OR11G2	76353	25kb
ENSG00000176219	14	20666791	20717964	268	28	120000	-0.054589	0.52177	OR11H6	76353	25kb
ENSG00000176198	14	20685898	20736979	221	37	120000	-0.089011	0.53546	OR11H4	76353	25kb
ENSG00000136319	14	20699717	20799153	318	48	120000	-1.2075	0.88637	TTC5	76353	25kb
ENSG00000100814	14	20754527	20826471	221	25	120000	-1.0163	0.84526	CCNB1IP1	76353	25kb
ENSG00000129484	14	20786741	20851064	186	21	120000	-0.92451	0.82239	PARP2	76353	25kb
ENSG00000129566	14	20808826	20906588	365	52	120000	-1.7162	0.95694	TEP1	76353	25kb
ENSG00000185271	14	20871970	20928801	238	47	120000	-0.94644	0.82804	KLHL33	76353	25kb
ENSG00000092094	14	20889570	20948264	226	45	120000	-0.73437	0.76864	OSGEP	76353	25kb
ENSG00000100823	14	20898350	20950927	198	38	120000	-0.64064	0.73912	APEX1	76353	25kb
ENSG00000165782	14	20900878	20954771	211	40	120000	-0.53243	0.70279	TMEM55B	76353	25kb
ENSG00000198805	14	20912113	20970253	218	52	120000	0.67722	0.24913	PNP	76353	25kb
ENSG00000182545	14	20948696	21004328	198	53	120000	0.16221	0.43557	RNASE10	76353	25kb
ENSG00000188655	14	20999252	21054090	259	41	120000	0.32377	0.37305	RNASE9	76353	25kb
ENSG00000259060	14	21026051	21102954	367	64	120000	1.0671	0.14297	RNASE11	76353	25kb
ENSG00000173464	14	21026054	21103043	367	64	120000	1.0671	0.14297	RNASE11	76353	25kb
ENSG00000258436	14	21033352	21083982	243	52	120000	0.023825	0.4905	RNASE12	76353	25kb
ENSG00000181803	14	21083855	21134850	246	38	120000	3.0339	0.0012072	OR6S1	76353	25kb
ENSG00000258818	14	21127259	21193761	274	46	120000	2.0796	0.018783	RNASE4	76353	25kb
ENSG00000214274	14	21127336	21192130	267	44	120000	2.1397	0.01619	ANG	76353	25kb
ENSG00000259171	14	21127746	21193735	269	46	120000	2.1362	0.016333	AL163636.6	76353	25kb
ENSG00000181562	14	21189051	21241539	332	32	120000	0.68055	0.24808	EDDM3A	76353	25kb
ENSG00000181552	14	21211586	21264107	340	38	120000	-0.67186	0.74916	EDDM3B	76353	25kb
ENSG00000169413	14	21224210	21275626	302	38	120000	0.30262	0.38109	RNASE6	76353	25kb
ENSG00000129538	14	21244387	21296437	221	45	120000	1.0798	0.14012	RNASE1	76353	25kb
ENSG00000169397	14	21334558	21385507	115	24	120000	-0.4517	0.67426	RNASE3	76353	25kb
ENSG00000169385	14	21398611	21449595	120	26	120000	-0.42364	0.66409	RNASE2	76353	25kb
ENSG00000165792	14	21432929	21490189	200	34	120000	-0.75833	0.77587	METTL17	76353	25kb
ENSG00000165794	14	21442414	21495030	177	33	120000	-0.77813	0.78175	SLC39A2	76353	25kb
ENSG00000165795	14	21459922	21564031	291	72	120000	-0.68831	0.75437	NDRG2	76353	25kb
ENSG00000179636	14	21467268	21529435	188	53	120000	-0.45635	0.67593	TPPP2	76353	25kb
ENSG00000255472	14	21475977	21527054	162	47	120000	-0.29448	0.61581	RP11-998D10.1	76353	25kb
ENSG00000206150	14	21475979	21527944	163	47	120000	-0.29257	0.61507	RNASE13	76353	25kb
ENSG00000165799	14	21485385	21537393	169	51	120000	-0.36538	0.64258	RNASE7	76353	25kb
ENSG00000173431	14	21500981	21551614	154	46	120000	-0.14583	0.55797	RNASE8	76353	25kb
ENSG00000165801	14	21513429	21583399	180	42	120000	-0.13729	0.5546	ARHGEF40	76353	25kb
ENSG00000165804	14	21533205	21597881	178	36	120000	-0.51338	0.69616	ZNF219	76353	25kb
ENSG00000232070	14	21542096	21596883	150	32	120000	-0.6653	0.74707	TMEM253	76353	25kb
ENSG00000169327	14	21598026	21649222	203	40	120000	0.21859	0.41348	OR5AU1	76353	25kb
ENSG00000092199	14	21652295	21762653	240	34	120000	0.51429	0.30353	HNRNPC	76353	25kb
ENSG00000092200	14	21731098	21844460	298	38	120000	1.2252	0.11025	RPGRIP1	76353	25kb
ENSG00000092201	14	21794631	21877425	181	22	120000	1.6283	0.051729	SUPT16H	76353	25kb

ENSG00000100888	14	21828353	21949285	242	22	120000	0.95902	0.16877	CHD8	76353	25kb
ENSG00000129472	14	21902179	21970132	158	20	120000	-0.12004	0.54777	RAB2B	76353	25kb
ENSG00000092203	14	21919756	21992319	202	25	120000	-0.68823	0.75435	TOX4	76353	25kb
ENSG00000165819	14	21941277	22004517	175	26	120000	-0.72189	0.76482	METTL3	76353	25kb
ENSG00000165821	14	21964232	22030350	228	47	120000	-0.55367	0.7101	SALL2	76353	25kb
ENSG00000169208	14	22012934	22063875	270	33	120000	0.075327	0.46998	OR10G3	76353	25kb
ENSG00000255582	14	22076992	22128096	243	39	120000	0.20402	0.41917	OR10G2	76353	25kb
ENSG00000221977	14	22108297	22159238	222	27	120000	-1.2015	0.88521	OR4E2	76353	25kb
ENSG00000259003	14	23000534	23052939	293	32	120000	0.64651	0.25897	AE000662.92	76353	25kb
ENSG00000129562	14	23008805	23083175	382	32	120000	0.022335	0.49109	DAD1	76353	25kb
ENSG00000100439	14	23042146	23106265	278	28	120000	-1.2764	0.8991	ABHD4	76353	25kb
ENSG00000255804	14	23077673	23128716	183	20	120000	-0.74604	0.77218	OR6J1	76353	25kb
ENSG00000155463	14	23210731	23266007	213	45	120000	0.2734	0.39227	OXA1L	76353	25kb
ENSG00000155465	14	23217431	23324029	437	62	120000	-0.49665	0.69028	SLC7A7	76353	25kb
ENSG00000215306	14	23261463	23316750	240	37	120000	-0.89448	0.81447	AL135998.1	76353	25kb
ENSG00000172590	14	23274088	23329246	205	42	120000	-0.66066	0.74558	MRPL52	76353	25kb
ENSG00000157227	14	23280766	23343236	191	42	120000	-0.038923	0.51552	MMP14	76353	25kb
ENSG00000197324	14	23315822	23375789	127	25	120000	0.57082	0.28406	LRP10	76353	25kb
ENSG00000139890	14	23327374	23381895	108	22	120000	1.0722	0.14181	REM2	76353	25kb
ENSG00000100461	14	23344854	23413393	166	18	120000	1.5299	0.063026	RBM23	76353	25kb
ENSG00000100462	14	23364720	23423794	142	12	120000	1.7822	0.037358	PRMT5	76353	25kb
ENSG00000092036	14	23390437	23451370	116	11	120000	1.9238	0.02719	HAUS4	76353	25kb
ENSG00000259132	14	23390479	23476467	151	14	120000	2.0465	0.020356	RP11-298I3.5	76353	25kb
ENSG00000129474	14	23415383	23476851	90	12	120000	2.1747	0.014826	AJUBA	76353	25kb
ENSG00000100802	14	23431110	23504375	103	19	120000	1.883	0.029849	C14orf93	76353	25kb
ENSG00000100804	14	23460752	23529439	121	31	120000	1.591	0.055801	PSMB5	76353	25kb
ENSG00000222028	14	23486376	23538269	108	26	120000	0.37865	0.35247	PSMB11	76353	25kb
ENSG00000139880	14	23491271	23551747	118	28	120000	0.32923	0.37099	CDH24	76353	25kb
ENSG00000100813	14	23502773	23589823	211	36	120000	-0.55972	0.71216	ACIN1	76353	25kb
ENSG00000179933	14	23538974	23594665	139	23	120000	-0.90279	0.81668	C14orf119	76353	25kb
ENSG00000092067	14	23561513	23613825	140	35	120000	-0.60948	0.7289	CEBPE	76353	25kb
ENSG00000092068	14	23569504	23677883	288	57	120000	-0.38572	0.65015	SLC7A8	76353	25kb
ENSG00000215277	14	23629525	23767686	412	52	120000	0.94416	0.17254	C14orf164	76353	25kb
ENSG00000215271	14	23716666	23793656	190	31	120000	0.69256	0.24429	HOMEZ	76353	25kb
ENSG00000235194	14	23739852	23797057	99	27	120000	-0.57466	0.71724	PPP1R3E	76353	25kb
ENSG00000129473	14	23742999	23805968	108	26	120000	-0.81215	0.79165	BCL2L2	76353	25kb
ENSG00000258643	14	23751044	23819578	123	28	120000	-0.74004	0.77036	BCL2L2-PABPN1	76353	25kb
ENSG00000100836	14	23765498	23820394	99	24	120000	-0.61403	0.7304	PABPN1	76353	25kb
ENSG00000268702	14	23765839	23816484	95	23	120000	-0.5764	0.71783	AL049829.1	76353	25kb
ENSG00000092096	14	23790515	23847121	132	28	120000	-1.0822	0.86042	SLC22A17	76353	25kb
ENSG00000100842	14	23800611	23859961	152	32	120000	-0.67404	0.74986	EFS	76353	25kb
ENSG00000166090	14	23817018	23870612	141	30	120000	-0.55478	0.71048	IL25	76353	25kb
ENSG00000166091	14	23821017	23873981	146	32	120000	-0.56546	0.71412	CMTM5	76353	25kb
ENSG00000197616	14	23826199	23902486	205	40	120000	-0.053539	0.52135	MYH6	76353	25kb
ENSG00000092054	14	23856947	23929927	201	34	120000	-0.31882	0.62507	MYH7	76353	25kb
ENSG00000129460	14	23913897	24004071	220	25	120000	-0.30151	0.61849	NGDN	76353	25kb
ENSG00000136367	14	23965066	24050401	147	42	120000	1.4471	0.073936	ZFHX2	76353	25kb
ENSG00000259431	14	24000216	24054480	76	21	120000	1.346	0.089152	THTPA	76353	25kb
ENSG00000213983	14	24003774	24062279	83	22	120000	1.649	0.049578	AP1G2	76353	25kb
ENSG00000092051	14	24012244	24073009	86	21	120000	1.8688	0.030828	JPH4	76353	25kb
ENSG00000100867	14	24074324	24139848	158	36	120000	2.1033	0.017719	DHRS2	76353	25kb
ENSG00000197775	14	24382940	24434196	215	9	120000	0.23633	0.40659	DHRS4-AS1	76353	25kb
ENSG00000157326	14	24397795	24463488	294	22	120000	-0.32463	0.62727	DHRS4	76353	25kb
ENSG00000187630	14	24414148	24500617	315	25	120000	-0.54918	0.70856	DHRS4L2	76353	25kb
ENSG00000186648	14	24496206	24563937	156	43	120000	-0.78262	0.78307	LRRC16B	76353	25kb
ENSG00000100884	14	24515046	24572309	113	37	120000	-0.85441	0.80356	CPNE6	76353	25kb
ENSG00000129535	14	24524316	24609223	151	36	120000	-1.7576	0.96059	NRL	76353	25kb
ENSG00000100889	14	24538262	24604807	98	20	120000	-1.6913	0.95461	PCK2	76353	25kb
ENSG00000100897	14	24558404	24619451	94	18	120000	-1.1923	0.88342	DCAF11	76353	25kb
ENSG00000259371	14	24565560	24626038	87	16	120000	-1.247	0.8938	RP11-468E2.6	76353	25kb
ENSG00000139914	14	24575484	24627058	74	13	120000	-1.0475	0.85257	FITM1	76353	25kb
ENSG00000092010	14	24580367	24633176	79	16	120000	-0.78188	0.78286	PSME1	76353	25kb
ENSG00000100908	14	24583174	24635797	79	16	120000	-0.78527	0.78385	EMC9	76353	25kb
ENSG00000100911	14	24587574	24641779	83	18	120000	-0.55083	0.70912	PSME2	76353	25kb
ENSG00000092098	14	24590892	24654870	108	23	120000	-0.18802	0.57457	RNF31	76353	25kb
ENSG00000259529	14	24595427	24661611	110	23	120000	-0.15844	0.56295	RP11-468E2.4	76353	25kb
ENSG00000213928	14	24605262	24660774	92	20	120000	0.13461	0.44646	IRF9	76353	25kb
ENSG00000100918	14	24616062	24674463	117	22	120000	-0.011576	0.50462	REC8	76353	25kb
ENSG00000196497	14	24624425	24683170	129	25	120000	-0.17102	0.5679	IPO4	76353	25kb
ENSG00000259522	14	24624601	24684622	132	26	120000	-0.16949	0.5673	RP11-468E2.2	76353	25kb
ENSG00000100926	14	24633349	24707679	151	30	120000	-0.16953	0.56731	TM9SF1	76353	25kb
ENSG00000254692	14	24633363	24707679	151	30	120000	-0.16953	0.56731	TM9SF1	76353	25kb
ENSG00000139908	14	24649903	24702568	115	25	120000	-0.42069	0.66301	TSSK4	76353	25kb
ENSG00000254505	14	24653789	24708075	115	25	120000	-0.53172	0.70254	CHMP4A	76353	25kb
ENSG00000213920	14	24658143	24710276	107	23	120000	-0.48792	0.6872	MDP1	76353	25kb
ENSG00000255526	14	24658161	24726558	135	26	120000	-0.45988	0.6772	NEDD8-MDP1	76353	25kb
ENSG00000129559	14	24661058	24726660	131	25	120000	-0.4182	0.6621	NEDD8	76353	25kb
ENSG00000100938	14	24676628	24733448	102	26	120000	-0.4651	0.67907	GMPR2	76353	25kb
ENSG00000092330	14	24683849	24736880	94	25	120000	-0.32257	0.62649	TINF2	76353	25kb
ENSG00000092295	14	24693320	24758638	128	28	120000	-0.64476	0.74046	TGM1	76353	25kb
ENSG00000100949	14	24709744	24765945	130	23	120000	-0.73869	0.76995	RABGGTA	76353	25kb
ENSG00000157379	14	24734804	24794039	145	19	120000	-0.77219	0.78	DHRS1	76353	25kb
ENSG00000196943	14	24744068	24803330	162	23	120000	-1.1608	0.87713	NOP9	76353	25kb
ENSG00000136305	14	24749302	24805636	163	23	120000	-1.1954	0.88404	CIDEB	76353	25kb
ENSG00000213906	14	24749940	24806259	163	23	120000	-1.149	0.87472	LTB4R2	76353	25kb
ENSG00000272658	14	24753161	24806252	159	22	120000	-1.1075	0.86596	LTB4R2	76353	25kb
ENSG00000213903	14	24755656	24812242	183	25	120000	-0.93234	0.82442	LTB4R	76353	25kb
ENSG00000129467	14	24762555	24829299	226	29	120000	-0.64042	0.73905	ADCY4	76353	25kb
ENSG00000258973	14	24776516	24831588	189	24	120000	-0.43387	0.66781	RP11-934B9.3	76353	25kb
ENSG00000129465	14	24780227	24834251	190	23	120000	-0.33884	0.63263	RIPK3	76353	25kb
ENSG00000100968	14	24809879	24873810	204	30	120000	-0.50747	0.69409	NFATC4	76353	25kb
ENSG00000205978	14	24842992	24913494	250	53	120000	1.0631	0.14387	NYNRIN	76353	25kb
ENSG00000139899	14	24870738	24925160	258	33	120000	1.6565	0.048811	CBLN3	76353	25kb
ENSG00000100441	14	24873492	24935540	322	30	120000	1.6389	0.050615	KHNYN	76353	25kb
ENSG00000100445	14	24883972	24937111	298	21	120000	1.4368	0.075391	SDR39U1	76353	25kb
ENSG00000258744	14	24945800	25002894	216	17	120000	-0.11128	0.5443	RP11-80A15.1	76353	25kb

ENSG00000092009	14	24949559	25002471	173	19	120000	-0.038077	0.51519	CMA1	76353	25kb
ENSG00000100448	14	25017728	25070466	155	20	120000	-0.67761	0.75099	CTSG	76353	25kb
ENSG00000100450	14	25050686	25103905	173	24	120000	-0.056246	0.52243	GZMH	76353	25kb
ENSG00000100453	14	25075160	25128473	162	22	120000	0.055977	0.47768	GZMB	76353	25kb
ENSG00000168952	14	25253862	25544503	907	66	120000	0.26142	0.39689	STXBP6	76353	25kb
ENSG00000139910	14	26887299	27091960	368	22	120000	-0.44571	0.6721	NOVA1	76353	25kb
ENSG00000176165	14	29210050	29263870	104	19	120000	-0.79484	0.78665	FOXG1	76353	25kb
ENSG00000186960	14	29216910	29307493	127	35	120000	-1.2976	0.90279	C14orf23	76353	25kb
ENSG00000184304	14	30020687	30686104	1640	119	120000	-0.023608	0.50942	PRKD1	76353	25kb
ENSG00000092140	14	31003329	31114269	260	22	120000	-0.17553	0.56967	G2E3	76353	25kb
ENSG00000092108	14	31066318	31230018	399	30	120000	-0.60651	0.72791	SCFD1	76353	25kb
ENSG00000100473	14	31318720	31389271	182	38	120000	1.9236	0.027201	COCH	76353	25kb
ENSG00000196792	14	31338005	31520607	441	32	120000	0.23543	0.40694	STRN3	76353	25kb
ENSG00000100478	14	31469312	31587818	249	34	120000	-0.33624	0.63166	AP4S1	76353	25kb
ENSG00000092148	14	31544318	31702010	200	23	120000	-0.51953	0.69831	HECTD1	76353	25kb
ENSG00000129493	14	31735994	31914788	292	22	120000	0.04803	0.48085	HEATR5A	76353	25kb
ENSG00000203546	14	31778518	31951647	311	21	120000	0.11146	0.45563	RP11-176H8.1	76353	25kb
ENSG00000129480	14	31890242	31951716	121	11	120000	0.17212	0.43167	DTD2	76353	25kb
ENSG00000151413	14	31934162	32355430	1437	33	120000	-0.357	0.63945	NUBPL	76353	25kb
ENSG00000258474	14	32389059	32444588	235	40	120000	0.38011	0.35193	RP11-187E13.1	76353	25kb
ENSG00000258386	14	32451108	32512891	143	23	120000	-0.01726	0.50689	RP11-187E13.2	76353	25kb
ENSG00000100852	14	32520320	32653934	139	33	120000	-0.97495	0.83521	ARHGAP5	76353	25kb
ENSG00000151320	14	32773479	33325567	1802	156	120000	-1.1331	0.87141	AKAP6	76353	25kb
ENSG00000151322	14	33379139	34298382	3000	309	120000	0.26236	0.39652	NPAS3	76353	25kb
ENSG00000129521	14	34368437	34956980	2103	192	120000	-0.44457	0.67168	EGLN3	76353	25kb
ENSG00000165389	14	34876995	34956562	206	27	120000	-1.6657	0.95211	SPTSSA	76353	25kb
ENSG00000129518	14	34960135	35033916	203	21	120000	-0.87402	0.80895	EAPP	76353	25kb
ENSG00000129515	14	35005300	35124389	295	19	120000	-0.73565	0.76903	SNX6	76353	25kb
ENSG00000165410	14	35154593	35209029	230	25	120000	0.33436	0.36905	CFL2	76353	25kb
ENSG00000198604	14	35196937	35369853	576	32	120000	-0.55679	0.71116	BAZ1A	76353	25kb
ENSG00000100883	14	35426163	35523773	364	22	120000	-1.2883	0.90119	SRP54	76353	25kb
ENSG00000151327	14	35489113	35607336	407	27	120000	-1.1666	0.87831	FAM177A1	76353	25kb
ENSG00000092020	14	35529673	35616723	269	20	120000	-0.8869	0.81243	PPP2R3C	76353	25kb
ENSG00000100890	14	35566052	35768271	588	28	120000	-1.0076	0.84318	KIAA0391	76353	25kb
ENSG00000258790	14	35566755	35811680	739	29	120000	-0.82994	0.79671	KIAA0391	76353	25kb
ENSG00000100902	14	35722839	35811699	283	19	120000	-0.48263	0.68532	PSMA6	76353	25kb
ENSG00000100906	14	35845717	35898955	228	42	120000	0.34576	0.36476	NFKBIA	76353	25kb
ENSG00000168348	14	35978248	36031260	54	14	120000	-0.2234	0.58839	INSM2	76353	25kb
ENSG00000174373	14	35982558	36303510	403	19	120000	-0.53072	0.70219	RALGAPA1	76353	25kb
ENSG00000100916	14	36270524	36426531	240	35	120000	-0.72312	0.7652	BRMS1L	76353	25kb
ENSG00000151332	14	36742770	36814882	125	12	120000	-0.27143	0.60697	MBIP	76353	25kb
ENSG00000229415	14	36917493	37008034	191	42	120000	-0.93143	0.82418	SFTA3	76353	25kb
ENSG00000136352	14	36960602	37015354	123	25	120000	0.088003	0.46494	NKX2-1	76353	25kb
ENSG00000136327	14	37024784	37076812	170	14	120000	1.7362	0.04126	NKX2-8	76353	25kb
ENSG00000198807	14	37101773	37173920	235	27	120000	0.051748	0.47936	PAX9	76353	25kb
ENSG00000183032	14	37122636	37667071	1600	114	120000	-2.8121	0.99754	SLC25A21	76353	25kb
ENSG00000258708	14	37616093	37668016	137	14	120000	-1.0107	0.84392	SLC25A21-AS1	76353	25kb
ENSG00000151338	14	37642118	38046566	798	20	120000	-0.67499	0.75016	MIPOL1	76353	25kb
ENSG00000129514	14	38034189	38094245	149	24	120000	-2.4619	0.99309	FOXA1	76353	25kb
ENSG00000139865	14	38040052	38535647	1107	73	120000	-0.70514	0.75964	TTC6	76353	25kb
ENSG00000139874	14	38652204	38707272	144	29	120000	0.084518	0.46632	SSTR1	76353	25kb
ENSG00000176435	14	38698308	38750574	121	26	120000	-0.93444	0.82496	CLEC14A	76353	25kb
ENSG00000100934	14	39476123	39603850	308	23	120000	-0.0041646	0.50166	SEC23A	76353	25kb
ENSG00000092208	14	39558427	39631177	191	23	120000	0.28134	0.38922	GEMIN2	76353	25kb
ENSG00000182400	14	39592015	39664736	190	23	120000	0.42696	0.3347	TRAPPC6B	76353	25kb
ENSG00000100941	14	39619387	39677422	143	22	120000	0.56315	0.28667	PNN	76353	25kb
ENSG00000150526	14	39674435	39747859	186	23	120000	0.51873	0.30197	MIA2	76353	25kb
ENSG00000258941	14	39678106	39845397	402	23	120000	0.12565	0.45001	RP11-407N17.3	76353	25kb
ENSG00000150527	14	39709488	39881156	382	23	120000	0.010854	0.49567	CTAGE5	76353	25kb
ENSG00000165355	14	39841873	39926704	218	20	120000	0.055882	0.47772	FBXO33	76353	25kb
ENSG00000165379	14	42051773	42398752	1079	40	120000	0.89681	0.18491	LRFN5	76353	25kb
ENSG00000189139	14	44948545	45001482	246	23	120000	-1.4668	0.92879	FSCB	76353	25kb
ENSG00000179476	14	45341498	45401460	131	12	120000	-1.0991	0.86413	C14orf28	76353	25kb
ENSG00000179454	14	45368522	45536525	310	13	120000	-1.2538	0.89504	KLHL28	76353	25kb
ENSG00000198718	14	45406411	45568634	285	12	120000	-1.2279	0.89025	FAM179B	76353	25kb
ENSG00000185246	14	45528302	45610485	117	9	120000	-0.78591	0.78404	PRPF39	76353	25kb
ENSG00000100442	14	45559803	45629522	105	10	120000	-0.65973	0.74529	FKBP3	76353	25kb
ENSG00000187790	14	45580143	45695093	179	18	120000	-0.8683	0.80739	FANCM	76353	25kb
ENSG00000129534	14	45647393	45747743	167	16	120000	-0.73523	0.7689	MIS18BP1	76353	25kb
ENSG00000165496	14	47095222	47146028	135	15	120000	0.9201	0.17876	RPL10L	76353	25kb
ENSG00000139915	14	47283826	48169157	2600	85	120000	0.27027	0.39348	MDGA2	76353	25kb
ENSG00000272781	14	47286134	48168999	2592	84	120000	0.26327	0.39617	MDGA2	76353	25kb
ENSG00000213741	14	50018390	50090408	162	12	120000	-0.18313	0.57265	RPS29	76353	25kb
ENSG00000253459	14	50039193	50090882	122	10	120000	-0.34544	0.63512	AL139099.1	76353	25kb
ENSG00000165501	14	50040415	50106390	170	13	120000	-0.13512	0.55374	LRR1	76353	25kb
ENSG00000165502	14	50060237	50112403	149	12	120000	-0.13109	0.55215	RPL36AL	76353	25kb
ENSG00000168282	14	50062489	50115198	158	11	120000	-0.16898	0.56709	MGAT2	76353	25kb
ENSG00000165506	14	50066892	50126948	161	11	120000	-0.24383	0.59632	DNAAF2	76353	25kb
ENSG00000100479	14	50085273	50180140	196	17	120000	-0.61237	0.72985	POLE2	76353	25kb
ENSG00000197776	14	50134823	50244870	166	25	120000	-1.285	0.9006	KLHDC1	76353	25kb
ENSG00000165516	14	50209326	50274909	67	16	120000	-1.5765	0.94255	KLHDC2	76353	25kb
ENSG00000165525	14	50224997	50344921	130	21	120000	-1.6357	0.94905	NEMF	76353	25kb
ENSG00000269832	14	50266993	50318258	62	17	120000	-1.872	0.9694	AL627171.2	76353	25kb
ENSG00000197502	14	50275810	50336552	73	18	120000	-2.0615	0.98037	AL627171.1	76353	25kb
ENSG00000165527	14	50334810	50386490	98	9	120000	-1.5629	0.94096	ARF6	76353	25kb
ENSG00000214900	14	50423430	50499238	284	35	120000	-1.3999	0.91922	C14orf182	76353	25kb
ENSG00000168260	14	50525369	50584361	164	21	120000	-0.0066838	0.50267	C14orf183	76353	25kb
ENSG00000100483	14	50550350	50608318	163	19	120000	0.1918	0.42395	VCPKMT	76353	25kb
ENSG00000100485	14	50558847	50723276	475	25	120000	0.20681	0.41808	SOS2	76353	25kb
ENSG00000087299	14	50679281	50804266	297	19	120000	0.65842	0.25513	L2HGDH	76353	25kb
ENSG00000125375	14	50754044	50827276	165	11	120000	0.66209	0.25396	ATP5S	76353	25kb
ENSG00000100490	14	50771310	50908179	321	32	120000	-0.42746	0.66548	CDKL1	76353	25kb
ENSG00000012983	14	50860219	51052844	326	27	120000	-0.30452	0.61964	MAP4K5	76353	25kb
ENSG00000198513	14	50974227	51124786	302	20	120000	0.82492	0.20471	ATL1	76353	25kb
ENSG00000151748	14	51073776	51160049	231	20	120000	1.1056	0.13445	SAV1	76353	25kb
ENSG00000100503	14	51161481	51322839	456	52	120000	0.81932	0.2063	NIN	76353	25kb

ENSG00000100504	14	51299609	51436454	481	59	120000	-1.406	0.92013	PYGL	76353	25kb
ENSG00000131969	14	51313878	51396688	297	43	120000	-1.4546	0.92711	ABHD12B	76353	25kb
ENSG00000100505	14	51416980	51587779	666	52	120000	-0.6446	0.74041	TRIM9	76353	25kb
ENSG00000139921	14	51681880	51747759	200	22	120000	0.10777	0.45709	TMX1	76353	25kb
ENSG00000139926	14	51930818	52222445	979	101	120000	0.090371	0.464	FRMD6	76353	25kb
ENSG00000186469	14	52267913	52471060	794	71	120000	0.38451	0.3503	GNG2	76353	25kb
ENSG00000258928	14	52357863	52410289	182	34	120000	-0.74111	0.77069	RP11-463J10.2	76353	25kb
ENSG00000267802	14	52411014	52461247	170	24	120000	0.31669	0.37574	AL358333.1	76353	25kb
ENSG00000087302	14	52431193	52496420	196	33	120000	0.34266	0.36593	C14orf166	76353	25kb
ENSG00000087303	14	52446521	52560712	504	34	120000	-0.069365	0.52765	NID2	76353	25kb
ENSG00000168229	14	52709431	52768442	203	31	120000	-0.75393	0.77455	PTGDR	76353	25kb
ENSG00000125384	14	52756023	52820324	266	30	120000	-1.5861	0.94364	PTGER2	76353	25kb
ENSG00000087301	14	52872308	53044240	421	28	120000	-0.49957	0.69131	TXNDC16	76353	25kb
ENSG00000180998	14	52994866	53129431	285	24	120000	-0.42587	0.6649	GPR137C	76353	25kb
ENSG00000197930	14	53081634	53187618	186	26	120000	0.23383	0.40756	ERO1L	76353	25kb
ENSG00000100519	14	53148890	53220305	135	19	120000	1.4821	0.069153	PSMC6	76353	25kb
ENSG00000198252	14	53171898	53266716	148	13	120000	1.4332	0.075902	STYX	76353	25kb
ENSG00000100522	14	53216912	53283386	93	12	120000	1.4972	0.067172	GNPNAT1	76353	25kb
ENSG00000073712	14	53298986	53444153	301	23	120000	-0.3456	0.63518	FERMT2	76353	25kb
ENSG00000100523	14	53485686	53645000	299	19	120000	-0.49177	0.68856	DDHD1	76353	25kb
ENSG00000125378	14	54391454	54450479	156	34	120000	-0.23204	0.59175	BMP4	76353	25kb
ENSG00000100526	14	54838567	54911936	187	23	120000	0.17974	0.42868	CDKN3	76353	25kb
ENSG00000100528	14	54868654	54933149	144	14	120000	-0.70772	0.76044	CNIH1	76353	25kb
ENSG00000197045	14	54916202	54980914	107	10	120000	-0.61285	0.73001	GMFB	76353	25kb
ENSG00000100532	14	54951530	55030567	112	15	120000	-0.31521	0.6237	CGRRF1	76353	25kb
ENSG00000020577	14	55008815	55285033	714	117	120000	0.397	0.34568	SAMD4A	76353	25kb
ENSG00000131979	14	55283726	55394570	291	20	120000	0.36705	0.35679	GCH1	76353	25kb
ENSG00000198554	14	55380668	55518823	289	17	120000	0.40259	0.34362	WDHD1	76353	25kb
ENSG00000180008	14	55468948	55541206	136	17	120000	-0.36013	0.64062	SOCS4	76353	25kb
ENSG00000168175	14	55493349	55561910	153	16	120000	-1.101	0.86456	MAPK1IP1L	76353	25kb
ENSG00000131981	14	55565828	55637126	165	17	120000	-0.77526	0.78091	LGALS3	76353	25kb
ENSG00000126787	14	55589830	55683396	187	17	120000	-0.76216	0.77702	DLGAP5	76353	25kb
ENSG00000178974	14	55713021	55853636	354	14	120000	-1.1483	0.87459	FBXO34	76353	25kb
ENSG00000126775	14	55808110	55903576	299	17	120000	-1.1691	0.87882	ATG14	76353	25kb
ENSG00000182521	14	55855259	55948444	367	23	120000	0.12079	0.45193	TBPL2	76353	25kb
ENSG00000126777	14	56000790	56193244	577	18	120000	-0.40474	0.65716	KTN1	76353	25kb
ENSG00000139946	14	56559532	56793244	720	31	120000	1.6934	0.045187	PELI2	76353	25kb
ENSG00000070269	14	56930072	57142324	749	71	120000	-1.3888	0.91756	TMEM260	76353	25kb
ENSG00000259133	14	57090683	57222253	447	56	120000	-1.0742	0.85864	RP11-1085N6.3	76353	25kb
ENSG00000165588	14	57242425	57302197	129	29	120000	0.40154	0.34401	OTX2	76353	25kb
ENSG00000070367	14	57645518	57760726	160	17	120000	0.13403	0.44669	EXOC5	76353	25kb
ENSG00000212928	14	57646888	57698291	88	10	120000	0.13766	0.44525	AL391152.1	76353	25kb
ENSG00000053770	14	57710627	57781797	82	12	120000	-0.093177	0.53712	AP5M1	76353	25kb
ENSG00000139977	14	57832262	57907635	114	19	120000	0.73848	0.23011	NAA30	76353	25kb
ENSG00000100557	14	57911019	57985585	161	16	120000	-0.50711	0.69396	C14orf105	76353	25kb
ENSG00000151812	14	58005640	58473912	1833	102	120000	-0.036835	0.51469	SLC35F4	76353	25kb
ENSG00000139971	14	58441453	58789857	842	63	120000	1.2379	0.10787	C14orf37	76353	25kb
ENSG00000131966	14	58641798	58726750	121	19	120000	-0.92066	0.82139	ACTR10	76353	25kb
ENSG00000100567	14	58686549	58763730	118	16	120000	-0.49026	0.68802	PSMA3	76353	25kb
ENSG00000268466	14	58729751	58780865	82	9	120000	-0.060965	0.52431	AL132989.1	76353	25kb
ENSG00000032219	14	58740103	58865605	166	18	120000	-0.34359	0.63442	ARID4A	76353	25kb
ENSG00000196860	14	58837634	58900419	68	17	120000	0.23028	0.40894	TOMM20L	76353	25kb
ENSG00000100575	14	58850212	58919332	73	16	120000	1.8219	0.034236	TIMM9	76353	25kb
ENSG00000100578	14	58869103	59040216	232	30	120000	2.3506	0.0093708	KIAA0586	76353	25kb
ENSG00000165617	14	59075685	59140039	153	26	120000	-0.53847	0.70488	DACT1	76353	25kb
ENSG00000100592	14	59630364	59863123	723	37	120000	-0.27696	0.60909	DAAM1	76353	25kb
ENSG00000181619	14	59870740	59957060	320	23	120000	-0.59822	0.72515	GPR135	76353	25kb
ENSG00000126790	14	59902081	59976148	255	15	120000	-0.27469	0.60822	L3HYPDH	76353	25kb
ENSG00000050130	14	59926161	59997128	240	18	120000	-0.36259	0.64155	JKAMP	76353	25kb
ENSG00000151838	14	59946257	60068549	486	27	120000	-0.74257	0.77113	CCDC175	76353	25kb
ENSG00000139970	14	60037694	60362684	708	36	120000	-0.15348	0.56099	RTN1	76353	25kb
ENSG00000131951	14	60361431	60555277	354	15	120000	1.7405	0.040883	LRRC9	76353	25kb
ENSG00000126773	14	60533629	60660851	276	9	120000	1.8614	0.031347	PCNXL4	76353	25kb
ENSG00000100612	14	60585838	60661574	168	10	120000	1.6638	0.048072	DHRS7	76353	25kb
ENSG00000100614	14	60687470	60790805	204	16	120000	-0.83553	0.79829	PPM1A	76353	25kb
ENSG00000179008	14	60838187	61007261	360	13	120000	-1.0043	0.84239	C14orf39	76353	25kb
ENSG00000184302	14	60950669	61004568	120	10	120000	-0.93304	0.8246	SIX6	76353	25kb
ENSG00000126778	14	61085133	61149977	107	15	120000	-0.69786	0.75737	SIX1	76353	25kb
ENSG00000100625	14	61151246	61216066	87	12	120000	-0.095107	0.53789	SIX4	76353	25kb
ENSG00000020426	14	61176460	61461671	416	21	120000	0.57834	0.28152	MNAT1	76353	25kb
ENSG00000126814	14	61413169	61473076	100	14	120000	0.23816	0.40588	TRMT5	76353	25kb
ENSG00000139974	14	61422832	61575451	237	23	120000	0.20038	0.42059	SLC38A6	76353	25kb
ENSG00000027075	14	61629277	62042694	1193	113	120000	-0.084474	0.53366	PRKCH	76353	25kb
ENSG00000182107	14	61719088	61773558	134	27	120000	-0.28465	0.61205	TMEM30B	76353	25kb
ENSG00000258989	14	61970846	62149682	505	62	120000	-1.2442	0.89328	RP11-47I22.4	76353	25kb
ENSG00000232774	14	62012258	62150414	370	51	120000	-0.82858	0.79633	RP11-47I22.3	76353	25kb
ENSG00000100644	14	62137231	62239976	260	30	120000	-0.72219	0.76491	HIF1A	76353	25kb
ENSG00000023608	14	62204075	62288146	261	23	120000	-0.64581	0.7408	SNAPC1	76353	25kb
ENSG00000139973	14	62428803	62593431	608	61	120000	-0.74747	0.77261	SYT16	76353	25kb
ENSG00000140015	14	63148287	63593755	1212	77	120000	0.75213	0.22599	KCNH5	76353	25kb
ENSG00000126785	14	63645832	63784937	398	35	120000	0.13303	0.44708	RHOJ	76353	25kb
ENSG00000154001	14	63813075	64035092	562	31	120000	-1.162	0.87738	PPP2R5E	76353	25kb
ENSG00000140006	14	64038757	64133579	225	16	120000	-0.15755	0.5626	WDR89	76353	25kb
ENSG00000126821	14	64125932	64219757	152	9	120000	-0.83273	0.7975	SGPP1	76353	25kb
ENSG00000054654	14	64294683	64718165	1111	56	120000	-0.42667	0.66519	SYNE2	76353	25kb
ENSG00000140009	14	64525950	64829830	764	48	120000	-0.23522	0.59298	ESR2	76353	25kb
ENSG00000100714	14	64829749	64951722	335	33	120000	-0.44855	0.67312	MTHFD1	76353	25kb
ENSG00000089775	14	64890824	64996931	295	17	120000	0.044485	0.48226	ZBTB25	76353	25kb
ENSG00000179841	14	64907217	64966221	136	15	120000	-0.14092	0.55603	AKAP5	76353	25kb
ENSG00000126804	14	64945430	65025408	236	20	120000	0.021619	0.49138	ZBTB1	76353	25kb
ENSG00000126803	14	64977623	65034955	166	20	120000	0.41849	0.33779	HSPA2	76353	25kb
ENSG00000165807	14	64991620	65081098	221	26	120000	0.43578	0.3315	PPP1R36	76353	25kb
ENSG00000126822	14	65145820	65238610	224	32	120000	-0.51961	0.69833	PLEKHG3	76353	25kb
ENSG00000070182	14	65188002	65371601	678	50	120000	-0.77179	0.77988	SPTB	76353	25kb
ENSG00000258289	14	65356079	65436309	301	17	120000	0.25107	0.40088	CHURC1	76353	25kb
ENSG00000125954	14	65356203	65553521	560	38	120000	0.49863	0.30902	CHURC1-FNTB	76353	25kb

ENSG00000257365	14	65356203	65554368	563	39	120000	0.52823	0.29867	FNTB	76353	25kb
ENSG00000176153	14	65380870	65434531	188	19	120000	0.11464	0.45437	GPX2	76353	25kb
ENSG00000139998	14	65387532	65464494	212	25	120000	0.38404	0.35048	RAB15	76353	25kb
ENSG00000125952	14	65447892	65594413	342	32	120000	1.1464	0.12581	MAX	76353	25kb
ENSG00000033170	14	65852310	66235839	914	32	120000	-0.23907	0.59448	FUT8	76353	25kb
ENSG00000258847	14	66399015	66496241	317	44	120000	2.0199	0.021698	CTD-2014B16.3	76353	25kb
ENSG00000171723	14	66949125	67673520	2186	20	120000	-1.5716	0.94198	GPHN	76353	25kb
ENSG00000172717	14	67631110	67720267	273	18	120000	-1.8494	0.9678	FAM71D	76353	25kb
ENSG00000072415	14	67682826	67827536	480	19	120000	-1.6792	0.95344	MPP5	76353	25kb
ENSG00000100554	14	67736088	67851982	363	18	120000	-1.6316	0.94862	ATP6V1D	76353	25kb
ENSG00000134001	14	67801714	67878233	240	23	120000	-1.4852	0.93126	EIF2S1	76353	25kb
ENSG00000100558	14	67828700	67903917	228	34	120000	-1.2263	0.88996	PLEK2	76353	25kb
ENSG00000198133	14	67888801	68025456	473	66	120000	-0.68966	0.7548	TMEM229B	76353	25kb
ENSG00000054690	14	67975018	68081329	352	51	120000	-0.067122	0.52676	PLEKHH1	76353	25kb
ENSG00000100564	14	68023672	68092004	211	39	120000	0.83137	0.20288	PIGH	76353	25kb
ENSG00000081181	14	68061515	68143437	212	31	120000	2.3499	0.0093881	ARG2	76353	25kb
ENSG00000100568	14	68088792	68166548	180	22	120000	2.58	0.0049395	VTI1B	76353	25kb
ENSG00000258466	14	68101537	68182084	213	22	120000	2.5177	0.0059061	RP11-1012A1.4	76353	25kb
ENSG00000072042	14	68118518	68187531	193	19	120000	2.6192	0.0044073	RDH11	76353	25kb
ENSG00000139988	14	68143603	68226169	224	26	120000	2.8559	0.0021458	RDH12	76353	25kb
ENSG00000072121	14	68169091	68308307	380	32	120000	2.3354	0.0097603	ZFYVE26	76353	25kb
ENSG00000182185	14	68261496	69221935	2268	113	120000	-0.033631	0.51341	RAD51B	76353	25kb
ENSG00000185650	14	69229377	69288190	180	23	120000	1.258	0.1042	ZFP36L1	76353	25kb
ENSG00000072110	14	69315860	69471157	596	61	120000	-1.4325	0.924	ACTN1	76353	25kb
ENSG00000139990	14	69492598	69644867	272	23	120000	-1.7808	0.96253	DCAF5	76353	25kb
ENSG00000081177	14	69633228	69734075	201	26	120000	-1.4383	0.92482	EXD2	76353	25kb
ENSG00000100626	14	69700994	69846183	461	59	120000	0.0027182	0.49892	GALNT16	76353	25kb
ENSG00000100632	14	69821848	69890344	181	22	120000	0.59644	0.27544	ERH	76353	25kb
ENSG00000029364	14	69839732	69954105	276	20	120000	0.38706	0.34936	SLC39A9	76353	25kb
ENSG00000175985	14	69926409	70020215	224	34	120000	-0.29751	0.61696	PLEKHD1	76353	25kb
ENSG00000255994	14	70012483	70063032	84	12	120000	0.076097	0.46967	CCDC177	76353	25kb
ENSG00000267909	14	70013216	70065339	88	13	120000	0.0042278	0.49831	CCDC177	76353	25kb
ENSG00000100647	14	70053313	70206859	256	25	120000	-0.24036	0.59497	KIAA0247	76353	25kb
ENSG00000100650	14	70168617	70263722	208	26	120000	-0.33321	0.63051	SRSF5	76353	25kb
ENSG00000100652	14	70217134	70289006	160	13	120000	-0.36829	0.64367	SLC10A1	76353	25kb
ENSG00000198732	14	70295848	70524083	812	79	120000	-1.8114	0.96496	SMOC1	76353	25kb
ENSG00000100678	14	70485934	70680787	759	57	120000	-1.3357	0.90917	SLC8A3	76353	25kb
ENSG00000133983	14	70766798	70851448	311	19	120000	-1.0652	0.85661	COX16	76353	25kb
ENSG00000213463	14	70813148	70908778	348	25	120000	-1.5588	0.94048	SYNJ2BP	76353	25kb
ENSG00000139985	14	70893874	70951622	226	27	120000	-1.8317	0.9665	ADAM21	76353	25kb
ENSG00000134007	14	70964075	71026732	182	22	120000	-1.4134	0.92123	ADAM20	76353	25kb
ENSG00000133997	14	71022974	71092384	246	22	120000	-0.75611	0.77521	MED6	76353	25kb
ENSG00000133985	14	71083504	71167077	261	51	120000	-2.057	0.98016	TTC9	76353	25kb
ENSG00000006432	14	71164243	71301251	493	38	120000	-0.57754	0.71821	MAP3K9	76353	25kb
ENSG00000100731	14	71349122	71607099	667	26	120000	-1.2129	0.88741	PCNX	76353	25kb
ENSG00000267901	14	71658031	71708462	184	24	120000	-0.19173	0.57602	AC004817.1	76353	25kb
ENSG00000197555	14	71762166	72232946	682	46	120000	-1.4334	0.92413	SIPA1L1	76353	25kb
ENSG00000182732	14	72374156	73055654	2481	127	120000	1.349	0.088663	RGS6	76353	25kb
ENSG00000257040	14	72431191	72483326	183	19	120000	-0.68001	0.75175	AC005477.1	76353	25kb
ENSG00000205683	14	73061004	73385809	1007	80	120000	0.52174	0.30093	DPF3	76353	25kb
ENSG00000119599	14	73368040	73451411	331	30	120000	2.2441	0.012413	DCAF4	76353	25kb
ENSG00000165861	14	73411159	73518920	387	23	120000	1.5209	0.064148	ZFYVE1	76353	25kb
ENSG00000119707	14	73500144	73613122	231	28	120000	1.1209	0.13116	RBM25	76353	25kb
ENSG00000080815	14	73578126	73715399	285	23	120000	1.2521	0.10526	PSEN1	76353	25kb
ENSG00000100767	14	73679205	73766348	204	37	120000	0.34776	0.36401	PAPLN	76353	25kb
ENSG00000133961	14	73716815	73955348	500	33	120000	-0.16916	0.56716	NUMB	76353	25kb
ENSG00000187105	14	73920189	74050651	268	37	120000	-0.75117	0.77372	HEATR4	76353	25kb
ENSG00000184227	14	73978818	74035498	84	23	120000	-0.30159	0.61852	ACOT1	76353	25kb
ENSG00000119673	14	74009324	74067357	175	29	120000	-1.0203	0.84622	ACOT2	76353	25kb
ENSG00000177465	14	74033410	74088200	215	29	120000	-1.3002	0.90323	ACOT4	76353	25kb
ENSG00000205669	14	74052649	74111592	224	33	120000	-1.0487	0.85285	ACOT6	76353	25kb
ENSG00000119661	14	74086578	74195435	292	22	120000	-0.14608	0.55807	DNAL1	76353	25kb
ENSG00000176903	14	74153494	74206128	141	22	120000	0.41412	0.33939	PNMA1	76353	25kb
ENSG00000156030	14	74156825	74281988	311	42	120000	0.29402	0.38437	ELMSAN1	76353	25kb
ENSG00000140043	14	74293547	74378530	339	17	120000	0.097476	0.46117	PTGR2	76353	25kb
ENSG00000258653	14	74293674	74423981	497	20	120000	0.097515	0.46116	RP5-1021I20.4	76353	25kb
ENSG00000119725	14	74328320	74424214	359	17	120000	0.16973	0.43261	ZNF410	76353	25kb
ENSG00000156050	14	74373204	74442117	238	19	120000	0.053837	0.47853	FAM161B	76353	25kb
ENSG00000119723	14	74391629	74455373	213	18	120000	0.14578	0.44205	COQ6	76353	25kb
ENSG00000187097	14	74399713	74511102	399	25	120000	-0.20687	0.58194	ENTPD5	76353	25kb
ENSG00000119636	14	74461056	74574566	402	20	120000	-0.51136	0.69545	CCDC176	76353	25kb
ENSG00000119711	14	74498553	74576196	259	13	120000	-0.54248	0.70626	ALDH6A1	76353	25kb
ENSG00000205659	14	74526499	74692936	530	24	120000	-0.41627	0.66139	LIN52	76353	25kb
ENSG00000119614	14	74681175	74754441	234	29	120000	1.0277	0.15205	VSX2	76353	25kb
ENSG00000119688	14	74727126	74794759	162	20	120000	0.12197	0.45146	ABCD4	76353	25kb
ENSG00000133980	14	74744772	74851711	254	33	120000	-0.26514	0.60455	VRTN	76353	25kb
ENSG00000183379	14	74847596	74917805	178	27	120000	-0.59143	0.72288	SYNDIG1L	76353	25kb
ENSG00000119655	14	74917895	74985880	157	17	120000	0.67328	0.25038	NPC2	76353	25kb
ENSG00000165898	14	74935423	74988809	124	13	120000	1.0633	0.14382	ISCA2	76353	25kb
ENSG00000119681	14	74939873	75104306	422	42	120000	0.70482	0.24046	LTBP2	76353	25kb
ENSG00000119682	14	75095140	75204818	151	15	120000	0.9621	0.168	AREL1	76353	25kb
ENSG00000214670	14	75133879	75204800	74	8	120000	1.3698	0.085369	AC007956.1	76353	25kb
ENSG00000119616	14	75154847	75228394	94	12	120000	1.2094	0.11326	FCF1	76353	25kb
ENSG00000119596	14	75205069	75347244	246	18	120000	0.85787	0.19548	YLPM1	76353	25kb
ENSG00000119608	14	75294736	75355537	117	14	120000	1.2892	0.098659	PROX2	76353	25kb
ENSG00000119689	14	75323594	75395448	165	17	120000	1.495	0.067462	DLST	76353	25kb
ENSG00000198208	14	75345657	75415099	173	20	120000	2.0891	0.018348	RPS6KL1	76353	25kb
ENSG00000119630	14	75383537	75447487	125	17	120000	1.9862	0.023506	PGF	76353	25kb
ENSG00000119718	14	75444614	75501292	65	4	120000	0.19828	0.42141	EIF2B2	76353	25kb
ENSG00000119684	14	75455467	75543235	118	7	120000	0.078267	0.46881	MLH3	76353	25kb
ENSG00000119640	14	75494924	75561186	103	7	120000	-0.0082712	0.5033	ACYP1	76353	25kb
ENSG00000119703	14	75505873	75570126	103	7	120000	-0.0015096	0.5006	ZC2HC1C	76353	25kb
ENSG00000119638	14	75523822	75619047	154	8	120000	0.2581	0.39816	NEK9	76353	25kb
ENSG00000170348	14	75573173	75668334	152	8	120000	0.43418	0.33208	TMED10	76353	25kb
ENSG00000258740	14	75680319	75760986	213	47	120000	-0.3172	0.62445	RP11-293M10.1	76353	25kb
ENSG00000170345	14	75720477	75773933	177	40	120000	-0.23123	0.59143	FOS	76353	25kb

ENSG00000140044	14	75869419	75965814	266	46	120000	0.70906	0.23914	JDP2	76353	25kb
ENSG00000156127	14	75963768	76038358	202	32	120000	-0.056452	0.52251	BATF	76353	25kb
ENSG00000119686	14	76019960	76154557	325	43	120000	-0.91252	0.81925	FLVCR2	76353	25kb
ENSG00000119685	14	76074968	76446421	708	33	120000	-0.51072	0.69523	TTLL5	76353	25kb
ENSG00000133935	14	76091134	76152532	113	22	120000	-0.32441	0.62719	C14orf1	76353	25kb
ENSG00000119650	14	76343479	76575928	552	37	120000	-0.45694	0.67614	IFT43	76353	25kb
ENSG00000119699	14	76399442	76474334	121	16	120000	-0.70835	0.76064	TGFB3	76353	25kb
ENSG00000089916	14	76593259	76745685	535	35	120000	1.7344	0.04142	GPATCH2L	76353	25kb
ENSG00000119715	14	76751957	76993178	925	96	120000	-0.43243	0.66729	ESRRB	76353	25kb
ENSG00000071246	14	77203532	77274354	197	43	120000	1.3145	0.094331	VASH1	76353	25kb
ENSG00000013523	14	77228588	77317589	282	32	120000	0.60205	0.27357	ANGEL1	76353	25kb
ENSG00000100565	14	77267715	77361645	303	46	120000	0.10116	0.45971	C14orf166B	76353	25kb
ENSG00000119669	14	77465888	77520034	158	28	120000	0.66941	0.25162	IRF2BPL	76353	25kb
ENSG00000198894	14	77539440	77608630	159	27	120000	-0.18804	0.57458	CIPC	76353	25kb
ENSG00000259164	14	77539654	77716805	627	59	120000	-1.1731	0.87962	RP11-463C8.4	76353	25kb
ENSG00000165548	14	77557911	77750838	687	63	120000	-1.3208	0.90672	TMEM63C	76353	25kb
ENSG00000177108	14	77572613	77634077	177	36	120000	-1.203	0.88551	ZDHHC22	76353	25kb
ENSG00000269883	14	77582026	77634339	164	35	120000	-1.0968	0.86364	AC007375.1	76353	25kb
ENSG00000165553	14	77706826	77762655	181	33	120000	-1.66	0.95154	NGB	76353	25kb
ENSG00000009830	14	77716299	77812227	305	35	120000	-1.449	0.92633	POMT2	76353	25kb
ENSG00000100577	14	77762227	77822940	180	31	120000	-0.99129	0.83923	GSTZ1	76353	25kb
ENSG00000100580	14	77776364	77868452	247	33	120000	0.3225	0.37354	TMED8	76353	25kb
ENSG00000100583	14	77818032	77882840	138	17	120000	1.3435	0.089561	SAMD15	76353	25kb
ENSG00000165555	14	77835364	77914860	151	14	120000	1.8055	0.035497	NOXRED1	76353	25kb
ENSG00000268156	14	77857741	77908115	82	10	120000	1.9555	0.025263	FKSG61	76353	25kb
ENSG00000151445	14	77868018	77949295	189	17	120000	2.2246	0.013054	VIPAS39	76353	25kb
ENSG00000100591	14	77899213	77960817	171	19	120000	2.494	0.0063156	AHSA1	76353	25kb
ENSG00000100593	14	77915740	77990210	214	24	120000	2.5528	0.0053435	ISM2	76353	25kb
ENSG00000100596	14	77947340	78108116	597	51	120000	1.6542	0.04904	SPTLC2	76353	25kb
ENSG00000100601	14	78113747	78199363	389	25	120000	-0.080155	0.53194	ALKBH1	76353	25kb
ENSG00000119705	14	78149414	78252447	374	23	120000	0.023662	0.49056	SLIRP	76353	25kb
ENSG00000100603	14	78158942	78252550	329	20	120000	0.038012	0.48484	SNW1	76353	25kb
ENSG00000197734	14	78202173	78261085	202	21	120000	-0.084365	0.53362	C14orf178	76353	25kb
ENSG00000268208	14	78202711	78253033	173	18	120000	-0.0050394	0.50201	AC008372.1	76353	25kb
ENSG00000063761	14	78241426	78426355	768	45	120000	0.11353	0.4548	ADCK1	76353	25kb
ENSG00000021645	14	78683734	80355762	4324	239	120000	1.0266	0.15232	NRXN3	76353	25kb
ENSG00000211448	14	80638873	80879100	544	41	120000	1.201	0.11487	DIO2	76353	25kb
ENSG00000100629	14	80918330	81450861	1678	62	120000	-0.51244	0.69583	CEP128	76353	25kb
ENSG00000165409	14	81396333	81637646	849	56	120000	-0.23724	0.59376	TSHR	76353	25kb
ENSG00000165417	14	81616796	81712721	192	24	120000	-0.61548	0.73088	GTF2A1	76353	25kb
ENSG00000140022	14	81702000	81927809	646	50	120000	-1.5068	0.93407	STON2	76353	25kb
ENSG00000071537	14	81912893	82025205	215	35	120000	-0.21941	0.58683	SEL1L	76353	25kb
ENSG00000258945	14	85969943	86020730	178	26	120000	0.68257	0.24744	RP11-497E19.2	76353	25kb
ENSG00000185070	14	85971488	86120034	522	43	120000	0.37297	0.35458	FLRT2	76353	25kb
ENSG00000258804	14	87347122	87414099	196	19	120000	0.26884	0.39402	RP11-322L20.1	76353	25kb
ENSG00000054983	14	88279164	88485009	886	36	120000	0.46548	0.32079	GALC	76353	25kb
ENSG00000140030	14	88446468	88503419	215	18	120000	-0.079495	0.53168	GPR65	76353	25kb
ENSG00000100433	14	88624113	88818251	755	72	120000	0.2557	0.39909	KCNK10	76353	25kb
ENSG00000042317	14	88826268	88961694	195	22	120000	-1.3894	0.91765	SPATA7	76353	25kb
ENSG00000070778	14	88907122	89046077	273	15	120000	-0.96337	0.83232	PTPN21	76353	25kb
ENSG00000100722	14	89004253	89104853	191	28	120000	-0.075861	0.53024	ZC3H14	76353	25kb
ENSG00000165521	14	89053775	89284096	413	24	120000	-0.075254	0.52999	EML5	76353	25kb
ENSG00000165533	14	89265497	89369335	187	22	120000	-0.63978	0.73884	TTC8	76353	25kb
ENSG00000053254	14	89566215	90110493	1527	184	120000	0.072838	0.47097	FOXN3	76353	25kb
ENSG00000258792	14	90070265	90134015	155	23	120000	-2.346	0.99051	RP11-944C7.1	76353	25kb
ENSG00000140025	14	90236013	90446121	684	44	120000	0.23513	0.40705	EFCAB11	76353	25kb
ENSG00000042088	14	90396283	90536106	373	20	120000	0.43208	0.33284	TDP1	76353	25kb
ENSG00000152315	14	90503109	90677201	592	52	120000	-0.68972	0.75481	KCNK13	76353	25kb
ENSG00000100764	14	90697839	90763968	200	11	120000	1.3132	0.094552	PSMC1	76353	25kb
ENSG00000119720	14	90717580	90823481	317	17	120000	0.95598	0.16954	NRDE2	76353	25kb
ENSG00000198668	14	90837846	90899605	152	23	120000	0.97023	0.16597	CALM1	76353	25kb
ENSG00000165914	14	90981932	91307823	1131	89	120000	0.21441	0.41512	TTC7B	76353	25kb
ENSG00000100784	14	91311799	91551980	555	32	120000	0.018898	0.49246	RPS6KA5	76353	25kb
ENSG00000133943	14	91501677	91716976	570	62	120000	0.41756	0.33814	C14orf159	76353	25kb
ENSG00000119714	14	91673876	91745269	219	38	120000	0.0345	0.48624	GPR68	76353	25kb
ENSG00000258875	14	91684103	91742426	152	23	120000	0.022767	0.49092	CTD-2547L24.3	76353	25kb
ENSG00000015133	14	91712667	91909188	539	64	120000	-0.029526	0.51178	CCDC88C	76353	25kb
ENSG00000100796	14	91898955	92001898	230	17	120000	-0.1191	0.5474	SMEK1	76353	25kb
ENSG00000268657	14	92013788	92066383	188	15	120000	-0.24206	0.59563	AL133373.1	76353	25kb
ENSG00000133962	14	92022040	92272051	819	64	120000	-0.7074	0.76034	CATSPERB	76353	25kb
ENSG00000165929	14	92221095	92358880	311	27	120000	0.60377	0.273	TC2N	76353	25kb
ENSG00000140092	14	92310756	92439331	407	47	120000	-0.59477	0.724	FBLN5	76353	25kb
ENSG00000100815	14	92407335	92532240	328	19	120000	-1.5528	0.93977	TRIP11	76353	25kb
ENSG00000066427	14	92499896	92597965	416	22	120000	-1.3261	0.9076	ATXN3	76353	25kb
ENSG00000183648	14	92557466	92613261	208	22	120000	-0.76161	0.77685	NDUFB1	76353	25kb
ENSG00000165934	14	92563281	92655755	291	18	120000	-0.77453	0.78069	CPSF2	76353	25kb
ENSG00000140090	14	92763925	92987596	930	79	120000	-0.074729	0.52978	SLC24A4	76353	25kb
ENSG00000100599	14	92955118	93180339	809	108	120000	-0.78574	0.78399	RIN3	76353	25kb
ENSG00000100600	14	93145152	93240047	259	42	120000	0.43137	0.3331	LGMN	76353	25kb
ENSG00000066455	14	93235576	93331308	303	16	120000	0.5127	0.30408	GOLGA5	76353	25kb
ENSG00000100604	14	93364425	93426638	220	44	120000	-1.2938	0.90213	CHGA	76353	25kb
ENSG00000100605	14	93378259	93607665	702	44	120000	-1.2652	0.8971	ITPK1	76353	25kb
ENSG00000165943	14	93623541	93676273	143	27	120000	0.33508	0.36878	MOAP1	76353	25kb
ENSG00000153485	14	93626296	93678434	145	28	120000	0.28702	0.38705	TMEM251	76353	25kb
ENSG00000259066	14	93626318	93709932	241	43	120000	-0.047218	0.51883	RP11-371E8.4	76353	25kb
ENSG00000170270	14	93644239	93698439	145	25	120000	-0.053525	0.52134	C14orf142	76353	25kb
ENSG00000012963	14	93648401	93720561	188	36	120000	-0.49477	0.68962	UBR7	76353	25kb
ENSG00000011114	14	93678896	93824438	437	30	120000	-0.45587	0.67576	BTBD7	76353	25kb
ENSG00000133958	14	93774565	94199222	972	34	120000	-0.044294	0.51767	UNC79	76353	25kb
ENSG00000187581	14	93788537	93839702	171	14	120000	-0.010468	0.50418	COX8C	76353	25kb
ENSG00000175785	14	94159644	94279827	334	64	120000	-1.1961	0.88417	PRIMA1	76353	25kb
ENSG00000140067	14	94360240	94420954	231	39	120000	-0.74721	0.77253	FAM181A	76353	25kb
ENSG00000100628	14	94375499	94468137	350	60	120000	-1.0013	0.84167	ASB2	76353	25kb
ENSG00000258987	14	94380901	94435475	213	39	120000	-1.0129	0.84444	RP11-131H24.4	76353	25kb
ENSG00000089723	14	94467675	94540276	258	17	120000	0.73053	0.23253	OTUB2	76353	25kb
ENSG00000089737	14	94492266	94572591	302	23	120000	0.44424	0.32844	DDX24	76353	25kb

ENSG00000165948	14	94522628	94595192	264	27	120000	0.32706	0.37181	IFI27L1	76353	25kb
ENSG00000165949	14	94546182	94608033	236	33	120000	0.11324	0.45492	IFI27	76353	25kb
ENSG00000119632	14	94569116	94621590	151	24	120000	-0.37137	0.64482	IFI27L2	76353	25kb
ENSG00000119698	14	94587465	94771072	441	25	120000	-0.92494	0.8225	PPP4R4	76353	25kb
ENSG00000140093	14	94724650	94784608	183	31	120000	-1.3385	0.90963	SERPINA10	76353	25kb
ENSG00000170099	14	94745585	94814731	223	37	120000	-0.37346	0.6456	SERPINA6	76353	25kb
ENSG00000197249	14	94818084	94882030	267	34	120000	-0.50257	0.69237	SERPINA1	76353	25kb
ENSG00000186910	14	94883801	94944127	282	25	120000	-0.87421	0.809	SERPINA11	76353	25kb
ENSG00000170054	14	94904054	94971026	325	42	120000	-0.60139	0.72621	SERPINA9	76353	25kb
ENSG00000165953	14	94928611	95009181	314	53	120000	-0.25993	0.60254	SERPINA12	76353	25kb
ENSG00000100665	14	95002428	95061250	280	29	120000	-0.46918	0.68053	SERPINA4	76353	25kb
ENSG00000188488	14	95002779	95084457	382	41	120000	-0.7097	0.76105	SERPINA5	76353	25kb
ENSG00000273259	14	95033395	95115983	384	54	120000	0.62958	0.26448	RP11-986E7.7	76353	25kb
ENSG00000196136	14	95053714	95115392	258	43	120000	1.3718	0.085068	SERPINA3	76353	25kb
ENSG00000133937	14	95209553	95261562	151	33	120000	-0.019568	0.50781	GSC	76353	25kb
ENSG00000100697	14	95527565	95649347	208	30	120000	-0.93419	0.8249	DICER1	76353	25kb
ENSG00000165959	14	95623277	95811243	657	65	120000	-0.62769	0.7349	CLMN	76353	25kb
ENSG00000176438	14	95858831	95967173	552	87	120000	-1.5441	0.93872	SYNE3	76353	25kb
ENSG00000258572	14	95957473	96009248	201	41	120000	-2.6378	0.99583	RP11-1070N10.3	76353	25kb
ENSG00000182512	14	95974840	96036061	290	35	120000	-1.4651	0.92855	GLRX5	76353	25kb
ENSG00000213231	14	96127754	96183980	222	45	120000	0.42526	0.33533	TCL1B	76353	25kb
ENSG00000100721	14	96151304	96205533	175	25	120000	0.80613	0.21008	TCL1A	76353	25kb
ENSG00000227051	14	96480661	96585417	421	46	120000	-0.5708	0.71593	C14orf132	76353	25kb
ENSG00000168398	14	96646016	96735666	384	64	120000	-1.7311	0.95828	BDKRB2	76353	25kb
ENSG00000258691	14	96646181	96755266	446	73	120000	-1.5994	0.94513	RP11-404P21.8	76353	25kb
ENSG00000100739	14	96697161	96760304	232	38	120000	-1.1246	0.86961	BDKRB1	76353	25kb
ENSG00000258729	14	96716383	96768060	171	30	120000	-0.58065	0.71926	DKFZP434O1614	76353	25kb
ENSG00000066739	14	96722595	96855207	298	26	120000	-0.50914	0.69467	ATG2B	76353	25kb
ENSG00000100744	14	96804814	96878625	147	30	120000	-0.23641	0.59344	GSKIP	76353	25kb
ENSG00000140057	14	96833448	96980764	311	42	120000	0.18768	0.42557	AK7	76353	25kb
ENSG00000090060	14	96942770	97058448	212	23	120000	0.90385	0.18304	PAPOLA	76353	25kb
ENSG00000100749	14	97238641	97423059	508	33	120000	0.8568	0.19578	VRK1	76353	25kb
ENSG00000258869	14	98190601	98255326	229	27	120000	-2.1701	0.985	RP11-204N11.1	76353	25kb
ENSG00000246223	14	98366947	98469461	343	27	120000	-1.1962	0.8842	C14orf64	76353	25kb
ENSG00000176605	14	99152950	99209098	240	33	120000	-1.0382	0.85041	C14orf177	76353	25kb
ENSG00000127152	14	99610624	99762861	352	54	120000	-1.5386	0.93805	BCL11B	76353	25kb
ENSG00000183576	14	99839083	99972216	195	18	120000	-0.83644	0.79855	SETD3	76353	25kb
ENSG00000090061	14	99922506	100026381	229	22	120000	0.35144	0.36263	CCNK	76353	25kb
ENSG00000205476	14	99952603	100095363	469	38	120000	-0.12225	0.54865	CCDC85C	76353	25kb
ENSG00000182218	14	100086447	100171906	222	33	120000	-0.4969	0.69037	HHIPL1	76353	25kb
ENSG00000036530	14	100125641	100218638	259	30	120000	-0.84562	0.80112	CYP46A1	76353	25kb
ENSG00000066629	14	100179030	100433397	769	68	120000	-1.2194	0.88865	EML1	76353	25kb
ENSG00000196405	14	100412786	100635573	613	36	120000	0.031715	0.48735	EVL	76353	25kb
ENSG00000168350	14	100587756	100651500	160	25	120000	0.019552	0.4922	DEGS2	76353	25kb
ENSG00000100811	14	100679635	100774129	200	21	120000	-0.14361	0.5571	YY1	76353	25kb
ENSG00000197119	14	100732448	100797884	148	33	120000	-0.99817	0.8409	SLC25A29	76353	25kb
ENSG00000140107	14	100764674	100821715	140	24	120000	-1.3994	0.91916	SLC25A47	76353	25kb
ENSG00000140105	14	100775125	100868142	237	21	120000	-0.721	0.76454	WARS	76353	25kb
ENSG00000176473	14	100817755	101021640	354	33	120000	-0.84242	0.80022	WDR25	76353	25kb
ENSG00000183092	14	100978486	101078750	190	23	120000	-1.7062	0.95601	BEGAIN	76353	25kb
ENSG00000185559	14	101167042	101226539	196	32	120000	0.22194	0.41218	DLK1	76353	25kb
ENSG00000267918	14	101270066	101320537	215	37	120000	-1.2588	0.89595	AL117190.2	76353	25kb
ENSG00000254656	14	101321992	101376184	152	43	120000	-1.5768	0.94258	RTL1	76353	25kb
ENSG00000269375	14	101334265	101385646	139	36	120000	-1.6741	0.95294	AL117190.3	76353	25kb
ENSG00000197406	14	102002688	102054789	118	20	120000	-0.7759	0.7811	DIO3	76353	25kb
ENSG00000258512	14	102171774	102223859	120	4	120000	0.56231	0.28695	RP11-796G6.2	76353	25kb
ENSG00000078304	14	102203135	102419326	655	53	120000	2.9829	0.0014278	PPP2R5C	76353	25kb
ENSG00000197102	14	102405865	102542129	335	28	120000	1.323	0.092913	DYNC1H1	76353	25kb
ENSG00000080824	14	102522075	102631036	275	24	120000	1.7945	0.036365	HSP90AA1	76353	25kb
ENSG00000140153	14	102580840	102716184	332	24	120000	1.2586	0.10409	WDR20	76353	25kb
ENSG00000080823	14	102665837	102796537	335	23	120000	0.36671	0.35692	MOK	76353	25kb
ENSG00000022976	14	102758714	102834044	168	33	120000	0.71211	0.2382	ZNF839	76353	25kb
ENSG00000100865	14	102783956	102854253	164	24	120000	0.91521	0.18004	CINP	76353	25kb
ENSG00000196663	14	102804300	102993818	551	22	120000	0.77162	0.22017	TECPR2	76353	25kb
ENSG00000156381	14	102948179	103001136	187	26	120000	0.78162	0.21722	ANKRD9	76353	25kb
ENSG00000089902	14	103033998	103221913	486	26	120000	-0.68592	0.75362	RCOR1	76353	25kb
ENSG00000131323	14	103218813	103402837	429	34	120000	0.6593	0.25485	TRAF3	76353	25kb
ENSG00000166126	14	103363993	103424933	140	32	120000	1.4324	0.076008	AMN	76353	25kb
ENSG00000198752	14	103373716	103548799	409	43	120000	0.7549	0.22516	CDC42BPB	76353	25kb
ENSG00000205436	14	103541481	103601896	156	31	120000	1.3812	0.083612	EXOC3L4	76353	25kb
ENSG00000185215	14	103564779	103628776	213	36	120000	1.2257	0.11015	TNFAIP2	76353	25kb
ENSG00000100664	14	103774881	103836362	201	30	120000	-0.42027	0.66285	EIF5	76353	25kb
ENSG00000075413	14	103826729	103995168	416	17	120000	-0.78965	0.78513	MARK3	76353	25kb
ENSG00000166165	14	103960996	104014448	134	16	120000	-0.51224	0.69576	CKB	76353	25kb
ENSG00000166166	14	103970521	104028410	142	18	120000	-0.37725	0.64701	TRMT61A	76353	25kb
ENSG00000166170	14	103997881	104054168	126	15	120000	0.014339	0.49428	BAG5	76353	25kb
ENSG00000126214	14	104003233	104192888	427	26	120000	-0.4475	0.67274	KLC1	76353	25kb
ENSG00000256053	14	104004299	104098860	233	21	120000	-0.22042	0.58723	APOPT1	76353	25kb
ENSG00000256500	14	104004299	104177261	395	23	120000	-0.37301	0.64543	RP11-73M18.2	76353	25kb
ENSG00000126215	14	104138946	104206841	144	22	120000	-0.87855	0.81018	XRCC3	76353	25kb
ENSG00000224997	14	104152607	104204149	107	18	120000	-0.94802	0.82844	AL049840.1	76353	25kb
ENSG00000100711	14	104157067	104225005	138	20	120000	-0.98959	0.83881	ZFYVE21	76353	25kb
ENSG00000088808	14	104175089	104338927	347	14	120000	-0.77333	0.78034	PPP1R13B	76353	25kb
ENSG00000156411	14	104353625	104419606	260	16	120000	-1.7066	0.95605	C14orf2	76353	25kb
ENSG00000156414	14	104369799	104544004	542	19	120000	-1.6331	0.94878	TDRD9	76353	25kb
ENSG00000227729	14	104381763	104433645	207	11	120000	-1.3856	0.91707	RD3L	76353	25kb
ENSG00000166183	14	104527016	104604098	213	37	120000	0.81394	0.20784	ASPG	76353	25kb
ENSG00000066735	14	104580060	104672231	386	57	120000	2.2126	0.013462	KIF26A	76353	25kb
ENSG00000228032	14	104685541	104737473	188	17	120000	0.97588	0.16456	C14orf144	76353	25kb
ENSG00000258986	14	104916015	105096984	624	48	120000	-0.15968	0.56343	TMEM179	76353	25kb
ENSG00000184601	14	105021021	105081852	175	18	120000	-1.8488	0.96776	C14orf180	76353	25kb
ENSG00000203485	14	105130943	105210942	302	42	120000	-0.052363	0.52088	INF2	76353	25kb
ENSG00000185100	14	105165523	105238662	246	32	120000	-0.32881	0.62885	ADSSL1	76353	25kb
ENSG00000184990	14	105194437	105259831	203	28	120000	0.1011	0.45974	SIVA1	76353	25kb
ENSG00000142208	14	105210686	105287088	224	41	120000	-0.1693	0.56722	AKT1	76353	25kb
ENSG00000179627	14	105241933	105296049	148	32	120000	-0.19979	0.57918	ZBTB42	76353	25kb

ENSG00000099814	14	105306617	105388107	274	18	120000	1.0862	0.1387	CEP170B	76353	25kb
ENSG00000166428	14	105366153	105424574	205	20	120000	0.99609	0.1596	PLD4	76353	25kb
ENSG00000185567	14	105378581	105469694	285	23	120000	0.40639	0.34223	AHNAK2	76353	25kb
ENSG00000140104	14	105427112	105501819	239	19	120000	-0.11563	0.54603	C14orf79	76353	25kb
ENSG00000170779	14	105450910	105512485	208	15	120000	-0.29356	0.61545	CDCA4	76353	25kb
ENSG00000183484	14	105490728	105556782	155	18	120000	-0.56722	0.71472	GPR132	76353	25kb
ENSG00000184916	14	105582318	105660161	182	21	120000	-0.24427	0.59649	JAG2	76353	25kb
ENSG00000183828	14	105614275	105672660	122	17	120000	-1.2973	0.90274	NUDT14	76353	25kb
ENSG00000185024	14	105650623	105806926	307	20	120000	-0.93193	0.82431	BRF1	76353	25kb
ENSG00000184887	14	105689827	105742430	123	10	120000	-0.3958	0.65388	BTBD6	76353	25kb
ENSG00000179364	14	105741900	105889484	148	26	120000	-0.14084	0.556	PACS2	76353	25kb
ENSG00000226174	14	105839916	105941443	81	28	120000	1.6284	0.051719	TEX22	76353	25kb
ENSG00000182979	14	105861159	105962066	88	28	120000	1.1748	0.12003	MTA1	76353	25kb
ENSG00000182809	14	105914299	105971499	66	18	120000	-0.31571	0.62389	CRIP2	76353	25kb
ENSG00000213145	14	105927654	105980284	72	11	120000	-1.1284	0.87042	CRIP1	76353	25kb
ENSG00000257341	14	105928536	105983604	76	11	120000	-1.1366	0.87215	CRIP1	76353	25kb
ENSG00000185347	14	105931192	105990912	75	8	120000	-1.3156	0.90584	C14orf80	76353	25kb
ENSG00000184986	14	105967940	106021539	94	8	120000	-1.8888	0.97054	TMEM121	76353	25kb
ENSG00000226777	14	106358838	106416825	59	10	120000	-0.45091	0.67397	KIAA0125	76353	25kb
ENSG00000197414	15	22711246	22771002	14	5	120000	-0.93878	0.82608	GOLGA6L1	76353	25kb
ENSG00000153575	15	22808395	22898892	211	21	120000	-0.20675	0.5819	TUBGCP5	76353	25kb
ENSG00000068793	15	22867005	23031016	633	78	120000	-0.26761	0.6055	CYFIP1	76353	25kb
ENSG00000140157	15	22979684	23059427	318	34	120000	-0.46803	0.68012	NIPA2	76353	25kb
ENSG00000170113	15	23018277	23125005	335	48	120000	-1.0336	0.84934	NIPA1	76353	25kb
ENSG00000153666	15	23230242	23293823	34	10	120000	-0.40694	0.65797	GOLGA8I	76353	25kb
ENSG00000174450	15	23659645	23717381	81	6	120000	-0.38307	0.64917	GOLGA6L2	76353	25kb
ENSG00000179455	15	23785454	23898064	311	39	120000	-1.0619	0.85585	MKRN3	76353	25kb
ENSG00000254585	15	23863691	23916175	171	28	120000	0.3701	0.35565	MAGEL2	76353	25kb
ENSG00000182636	15	23905565	23957450	170	29	120000	1.7888	0.036822	NDN	76353	25kb
ENSG00000185823	15	24895541	24953593	265	20	120000	-1.3506	0.91159	NPAP1	76353	25kb
ENSG00000128739	15	25043794	25248870	781	96	120000	1.0641	0.14364	SNRPN	76353	25kb
ENSG00000273173	15	25175133	25248729	165	21	120000	0.40948	0.34109	SNURF	76353	25kb
ENSG00000214265	15	25175181	25270423	222	21	120000	0.38088	0.35165	SNURF	76353	25kb
ENSG00000114062	15	25557381	25709128	370	25	120000	1.2549	0.10476	UBE3A	76353	25kb
ENSG00000206190	15	25897420	26135317	1082	133	120000	2.5754	0.0050056	ATP10A	76353	25kb
ENSG00000166206	15	26763693	27209686	1411	131	120000	-0.92486	0.82248	GABRB3	76353	25kb
ENSG00000186297	15	27086510	27219354	336	49	120000	0.056746	0.47737	GABRA5	76353	25kb
ENSG00000182256	15	27191429	27803373	1797	176	120000	-1.1834	0.88167	GABRG3	76353	25kb
ENSG00000104044	15	27975021	28369504	1101	60	120000	0.076499	0.46951	OCA2	76353	25kb
ENSG00000128731	15	28331186	28592298	232	15	120000	-1.1351	0.87182	HERC2	76353	25kb
ENSG00000188626	15	28922100	29008530	77	5	120000	-0.041136	0.51641	GOLGA8M	76353	25kb
ENSG00000034053	15	29104629	29435518	796	47	120000	0.17181	0.43179	APBA2	76353	25kb
ENSG00000104059	15	29387457	29887927	1562	115	120000	0.44645	0.32764	FAM189A1	76353	25kb
ENSG00000185115	15	29535353	29587033	137	18	120000	0.1291	0.44864	NDNL2	76353	25kb
ENSG00000104067	15	29966571	30286068	790	61	120000	-0.46878	0.68039	TJP1	76353	25kb
ENSG00000179938	15	30350158	30410702	37	9	120000	0.032857	0.48689	GOLGA8J	76353	25kb
ENSG00000261247	15	30402352	30462770	1	1	120000	0.62758	0.26514	GOLGA8T	76353	25kb
ENSG00000261794	15	30871329	30931764	19	6	120000	1.0456	0.14786	GOLGA8H	76353	25kb
ENSG00000187951	15	30891697	31090196	333	19	120000	1.6471	0.049773	ARHGAP11B	76353	25kb
ENSG00000198690	15	31171055	31260311	231	27	120000	1.791	0.036644	FAN1	76353	25kb
ENSG00000166912	15	31206144	31308810	276	20	120000	1.3548	0.087741	MTMR10	76353	25kb
ENSG00000134160	15	31268264	31478476	697	83	120000	0.98989	0.16111	TRPM1	76353	25kb
ENSG00000169926	15	31594058	31752868	421	51	120000	-0.31781	0.62469	KLF13	76353	25kb
ENSG00000169918	15	31750329	32187992	1200	47	120000	-1.2929	0.90198	OTUD7A	76353	25kb
ENSG00000175344	15	32297691	32489722	362	45	120000	1.7129	0.043365	CHRNA7	76353	25kb
ENSG00000268759	15	32749822	32805287	2	1	120000	-0.21482	0.58505	AC135983.2	76353	25kb
ENSG00000232653	15	32860657	32921179	31	9	120000	0.58467	0.27939	GOLGA8N	76353	25kb
ENSG00000198826	15	32882345	32957150	123	29	120000	0.46636	0.32048	ARHGAP11A	76353	25kb
ENSG00000166922	15	32908877	33014299	297	54	120000	0.16664	0.43383	SCG5	76353	25kb
ENSG00000166923	15	32985175	33051870	225	36	120000	-0.72973	0.76722	GREM1	76353	25kb
ENSG00000248905	15	33032747	33511897	2105	156	120000	0.57637	0.28218	FMN1	76353	25kb
ENSG00000198838	15	33578163	34183303	2474	204	120000	-0.45886	0.67683	RYR3	76353	25kb
ENSG00000169857	15	34133428	34356377	848	47	120000	2.6306	0.0042613	AVEN	76353	25kb
ENSG00000184984	15	34235921	34382291	434	32	120000	2.0304	0.021158	CHRM5	76353	25kb
ENSG00000134153	15	34351218	34419149	161	22	120000	0.96087	0.16831	EMC7	76353	25kb
ENSG00000182405	15	34369274	34421591	136	21	120000	0.93599	0.17464	PGBD4	76353	25kb
ENSG00000134152	15	34407875	34527297	418	27	120000	-0.63099	0.73598	KATNBL1	76353	25kb
ENSG00000128463	15	34492200	34547357	206	22	120000	-1.0034	0.84218	EMC4	76353	25kb
ENSG00000140199	15	34500460	34655261	626	27	120000	-1.0531	0.85385	SLC12A6	76353	25kb
ENSG00000182117	15	34608917	34660378	231	24	120000	-1.2992	0.90307	NOP10	76353	25kb
ENSG00000184507	15	34610516	34674938	289	38	120000	-0.90497	0.81726	NUTM1	76353	25kb
ENSG00000176454	15	34626106	34684479	183	37	120000	-0.78224	0.78296	LPCAT4	76353	25kb
ENSG00000175265	15	34646269	34905704	356	43	120000	0.01275	0.49491	GOLGA8A	76353	25kb
ENSG00000215252	15	34792408	34905704	163	19	120000	-0.28906	0.61373	GOLGA8B	76353	25kb
ENSG00000159248	15	35018233	35072166	196	31	120000	-0.29046	0.61427	GJD2	76353	25kb
ENSG00000159251	15	35055297	35113340	239	53	120000	0.68982	0.24516	ACTC1	76353	25kb
ENSG00000021776	15	35122732	35287040	390	26	120000	0.81745	0.20683	AQR	76353	25kb
ENSG00000198146	15	35245542	35305488	191	21	120000	1.1306	0.12912	ZNF770	76353	25kb
ENSG00000212768	15	35245552	35297268	158	18	120000	1.1956	0.11592	AC114546.1	76353	25kb
ENSG00000134146	15	35484546	35863394	751	40	120000	-0.46924	0.68055	DPH6	76353	25kb
ENSG00000186073	15	36846812	37127449	790	50	120000	-0.32965	0.62917	C15orf41	76353	25kb
ENSG00000134138	15	37156406	37418504	539	96	120000	-0.73982	0.77029	MEIS2	76353	25kb
ENSG00000166069	15	38189140	38284925	182	22	120000	-0.53602	0.70403	TMCO5A	76353	25kb
ENSG00000166068	15	38519527	38674450	488	19	120000	0.73566	0.23097	SPRED1	76353	25kb
ENSG00000171262	15	38721328	38804911	276	16	120000	-0.48771	0.68712	FAM98B	76353	25kb
ENSG00000172575	15	38755304	38882776	304	27	120000	-0.69627	0.75687	RASGRP1	76353	25kb
ENSG00000175779	15	38963799	39017239	198	32	120000	-0.28087	0.61059	C15orf53	76353	25kb
ENSG00000175746	15	39517885	39572046	168	34	120000	1.729	0.041908	C15orf54	76353	25kb
ENSG00000137801	15	39848280	39916667	128	12	120000	1.3194	0.093515	THBS1	76353	25kb
ENSG00000150667	15	39867232	40100031	701	30	120000	0.62768	0.26511	FSIP1	76353	25kb
ENSG00000166073	15	40066233	40238093	385	47	120000	-0.1695	0.5673	GPR176	76353	25kb
ENSG00000128829	15	40201347	40352797	432	39	120000	-1.4806	0.93065	EIF2AK4	76353	25kb
ENSG00000140319	15	40302940	40356389	112	19	120000	-1.643	0.94981	SRP14	76353	25kb
ENSG00000104081	15	40355091	40426093	144	27	120000	-1.0056	0.84269	BMF	76353	25kb
ENSG00000156970	15	40428224	40538337	202	16	120000	0.47334	0.31799	BUB1B	76353	25kb
ENSG00000259288	15	40484629	40593597	242	57	120000	-0.00057849	0.50023	RP11-133K1.2	76353	25kb

ENSG00000137843	15	40484629	40594688	243	57	120000	0.010901	0.49565	PAK6	76353	25kb
ENSG00000176753	15	40517865	40570170	153	38	120000	0.046846	0.48132	C15orf56	76353	25kb
ENSG00000137841	15	40545377	40625136	204	48	120000	0.10738	0.45724	PLCB2	76353	25kb
ENSG00000230778	15	40548645	40599787	133	29	120000	0.45929	0.32301	ANKRD63	76353	25kb
ENSG00000188549	15	40598653	40658168	163	34	120000	0.10769	0.45712	C15orf52	76353	25kb
ENSG00000233041	15	40618234	40673635	158	31	120000	0.096178	0.46169	PHGR1	76353	25kb
ENSG00000140323	15	40625436	40688257	176	30	120000	-0.022399	0.50894	DISP2	76353	25kb
ENSG00000128944	15	40649922	40711447	152	20	120000	0.69512	0.24349	KNSTRN	76353	25kb
ENSG00000128928	15	40672686	40753146	171	21	120000	1.235	0.10842	IVD	76353	25kb
ENSG00000140320	15	40706920	40785441	147	23	120000	2.2097	0.013563	BAHD1	76353	25kb
ENSG00000169105	15	40738160	40790353	80	11	120000	1.8693	0.030793	CHST14	76353	25kb
ENSG00000128891	15	40795882	40882256	233	15	120000	2.2077	0.013632	C15orf57	76353	25kb
ENSG00000166133	15	40836499	40891659	187	14	120000	2.3968	0.0082687	RPUSD2	76353	25kb
ENSG00000137812	15	40861218	40981540	270	14	120000	2.4623	0.0069027	CASC5	76353	25kb
ENSG00000051180	15	40961972	41049354	178	14	120000	2.2477	0.012297	RAD51	76353	25kb
ENSG00000137824	15	41003082	41073049	132	14	120000	2.177	0.01474	RMDN3	76353	25kb
ENSG00000137880	15	41031218	41084906	92	15	120000	1.8186	0.034486	GCHFR	76353	25kb
ENSG00000104129	15	41035067	41124675	177	21	120000	1.9857	0.023533	DNAJC17	76353	25kb
ENSG00000188277	15	41037159	41089643	88	14	120000	1.8081	0.035298	C15orf62	76353	25kb
ENSG00000166140	15	41074284	41131767	114	13	120000	1.72	0.042719	ZFYVE19	76353	25kb
ENSG00000166143	15	41082650	41145907	137	16	120000	1.639	0.050605	PPP1R14D	76353	25kb
ENSG00000166145	15	41111216	41175405	137	15	120000	0.70664	0.23989	SPINT1	76353	25kb
ENSG00000104140	15	41139412	41191487	122	11	120000	0.25576	0.39907	RHOV	76353	25kb
ENSG00000104142	15	41161628	41221173	107	15	120000	0.055779	0.47776	VPS18	76353	25kb
ENSG00000128917	15	41196538	41256237	107	23	120000	-0.22415	0.58868	DLL4	76353	25kb
ENSG00000128965	15	41220160	41273710	126	20	120000	-0.24157	0.59545	CHAC1	76353	25kb
ENSG00000128908	15	41246078	41433552	293	21	120000	-0.31713	0.62443	INO80	76353	25kb
ENSG00000178997	15	41449923	41547941	340	15	120000	0.059751	0.47618	EXD1	76353	25kb
ENSG00000187446	15	41498037	41599043	282	16	120000	-0.0092377	0.50369	CHP1	76353	25kb
ENSG00000104147	15	41576466	41649819	141	13	120000	-0.7592	0.77613	OIP5	76353	25kb
ENSG00000137804	15	41599892	41698248	194	16	120000	-0.67699	0.75079	NUSAP1	76353	25kb
ENSG00000137806	15	41654551	41719717	128	13	120000	-0.73282	0.76817	NDUFAF1	76353	25kb
ENSG00000137815	15	41675606	41800761	245	22	120000	-0.78289	0.78315	RTF1	76353	25kb
ENSG00000137825	15	41760591	41820747	132	24	120000	-0.63909	0.73862	ITPKA	76353	25kb
ENSG00000062524	15	41770836	41831085	146	23	120000	-0.49415	0.6894	LTK	76353	25kb
ENSG00000103932	15	41784374	41861467	190	24	120000	-0.45079	0.67393	RPAP1	76353	25kb
ENSG00000092445	15	41824873	41896536	168	22	120000	-0.19234	0.57626	TYRO3	76353	25kb
ENSG00000174197	15	41888422	42087141	362	22	120000	-0.73862	0.76993	MGA	76353	25kb
ENSG00000137802	15	42041632	42145053	216	22	120000	-0.753	0.77427	MAPKBP1	76353	25kb
ENSG00000268632	15	42048346	42101229	79	15	120000	-0.50403	0.69288	AC073657.1	76353	25kb
ENSG00000243789	15	42095283	42154779	170	20	120000	-0.86548	0.80661	JMJD7	76353	25kb
ENSG00000243708	15	42095283	42165345	196	24	120000	-0.81904	0.79362	PLA2G4B	76353	25kb
ENSG00000168970	15	42095293	42165353	196	24	120000	-0.81904	0.79362	JMJD7-PLA2G4B	76353	25kb
ENSG00000137877	15	42115345	42211275	320	29	120000	-1.2584	0.89588	SPTBN5	76353	25kb
ENSG00000103966	15	42165950	42289776	356	44	120000	0.95096	0.17081	EHD4	76353	25kb
ENSG00000188089	15	42248780	42368388	502	53	120000	1.8216	0.034258	PLA2G4E	76353	25kb
ENSG00000159337	15	42334207	42411752	402	26	120000	1.3443	0.089427	PLA2G4D	76353	25kb
ENSG00000168907	15	42408332	42473839	182	38	120000	-0.45619	0.67587	PLA2G4F	76353	25kb
ENSG00000166887	15	42425899	42525514	214	31	120000	-1.0116	0.84415	VPS39	76353	25kb
ENSG00000103978	15	42477730	42590861	301	23	120000	-1.1206	0.86876	TMEM87A	76353	25kb
ENSG00000214013	15	42540431	42670864	469	20	120000	-1.6929	0.95476	GANC	76353	25kb
ENSG00000092529	15	42615301	42729516	308	19	120000	-1.2147	0.88777	CAPN3	76353	25kb
ENSG00000103994	15	42680021	42808321	242	29	120000	-0.22919	0.59064	ZNF106	76353	25kb
ENSG00000092531	15	42758431	42862547	185	20	120000	0.036272	0.48553	SNAP23	76353	25kb
ENSG00000180979	15	42809720	42866000	91	12	120000	0.1764	0.42999	LRRC57	76353	25kb
ENSG00000137814	15	42816008	42887192	105	14	120000	0.41126	0.34044	HAUS2	76353	25kb
ENSG00000159433	15	42842857	43038179	288	22	120000	2.02	0.021693	STARD9	76353	25kb
ENSG00000140326	15	42990757	43054324	122	11	120000	1.6872	0.045783	CDAN1	76353	25kb
ENSG00000128881	15	43005932	43238007	295	25	120000	0.91796	0.17932	TTBK2	76353	25kb
ENSG00000159459	15	43210095	43423311	221	25	120000	-0.14055	0.55589	UBR1	76353	25kb
ENSG00000166947	15	43373423	43538481	152	24	120000	0.028954	0.48845	EPB42	76353	25kb
ENSG00000137842	15	43390477	43502344	104	21	120000	0.17443	0.43076	TMEM62	76353	25kb
ENSG00000166946	15	43452316	43512396	40	11	120000	-0.3544	0.63848	CCNDBP1	76353	25kb
ENSG00000104055	15	43499793	43584055	103	11	120000	-0.87645	0.80961	TGM5	76353	25kb
ENSG00000159495	15	43543478	43619453	113	9	120000	-1.3852	0.91701	TGM7	76353	25kb
ENSG00000168806	15	43594974	43647803	104	10	120000	-1.4381	0.9248	LCMT2	76353	25kb
ENSG00000168803	15	43597872	43671096	141	11	120000	-1.6058	0.94584	ADAL	76353	25kb
ENSG00000140265	15	43625370	43688223	121	10	120000	-1.8166	0.96536	ZSCAN29	76353	25kb
ENSG00000137822	15	43636419	43724293	151	11	120000	-1.9106	0.97197	TUBGCP4	76353	25kb
ENSG00000067369	15	43674407	43827926	267	13	120000	-1.7782	0.96232	TP53BP1	76353	25kb
ENSG00000166963	15	43778156	43848818	85	10	120000	-1.2912	0.90168	MAP1A	76353	25kb
ENSG00000168781	15	43800660	43907430	78	9	120000	-0.94326	0.82723	PPIP5K1	76353	25kb
ENSG00000237289	15	43860252	43922099	43	6	120000	-1.1252	0.86974	CKMT1B	76353	25kb
ENSG00000242866	15	43866596	44035458	156	14	120000	-1.4233	0.92267	STRC	76353	25kb
ENSG00000166762	15	43895701	43985316	77	9	120000	-1.3878	0.9174	CATSPER2	76353	25kb
ENSG00000223572	15	43960084	44016420	28	8	120000	-1.8138	0.96515	CKMT1A	76353	25kb
ENSG00000167004	15	44013590	44090477	115	12	120000	-1.3536	0.91207	PDIA3	76353	25kb
ENSG00000128886	15	44039798	44094741	77	10	120000	-1.2166	0.88813	ELL3	76353	25kb
ENSG00000262560	15	44039815	44116331	107	10	120000	-1.2943	0.90221	RP11-296A16.1	76353	25kb
ENSG00000140264	15	44044285	44119787	104	10	120000	-1.2847	0.90056	SERF2	76353	25kb
ENSG00000268488	15	44050041	44100231	68	9	120000	-1.1282	0.87038	AC018512.1	76353	25kb
ENSG00000184716	15	44061360	44117419	81	10	120000	-1.3306	0.90834	SERINC4	76353	25kb
ENSG00000242028	15	44063340	44120241	79	9	120000	-1.3192	0.90645	HYPK	76353	25kb
ENSG00000140259	15	44071690	44142000	98	9	120000	-1.3199	0.90657	MFAP1	76353	25kb
ENSG00000092470	15	44094161	44185617	121	10	120000	-0.91169	0.81904	WDR76	76353	25kb
ENSG00000171877	15	44137962	44512450	550	25	120000	0.5493	0.2914	FRMD5	76353	25kb
ENSG00000166734	15	44555927	44732956	243	10	120000	-0.58203	0.71973	CASC4	76353	25kb
ENSG00000137770	15	44694432	44846236	238	13	120000	-0.57265	0.71656	CTDSPL2	76353	25kb
ENSG00000104131	15	44804255	44880227	98	12	120000	-0.66827	0.74802	EIF3J	76353	25kb
ENSG00000104133	15	44829894	44980876	120	34	120000	-0.047962	0.51913	SPG11	76353	25kb
ENSG00000229474	15	44932930	45028514	133	25	120000	0.66986	0.25147	PATL2	76353	25kb
ENSG00000166710	15	44978675	45036075	112	16	120000	0.40262	0.34361	B2M	76353	25kb
ENSG00000185880	15	44996186	45085027	272	15	120000	0.59423	0.27618	TRIM69	76353	25kb
ENSG00000167014	15	45223900	45296427	176	13	120000	-1.255	0.89527	C15orf43	76353	25kb
ENSG00000140263	15	45290302	45394383	208	29	120000	-1.4447	0.92572	SORD	76353	25kb
ENSG00000140279	15	45359848	45431542	140	20	120000	-0.73935	0.77015	DUOX2	76353	25kb

ENSG00000140274	15	45381519	45435619	109	18	120000	-1.1166	0.86792	DUOXA2	76353	25kb
ENSG00000140254	15	45384569	45447136	127	23	120000	-0.84787	0.80174	DUOXA1	76353	25kb
ENSG00000137857	15	45397131	45482774	196	29	120000	-0.29974	0.61781	DUOX1	76353	25kb
ENSG00000138606	15	45434412	45518373	221	20	120000	-0.36743	0.64335	SHF	76353	25kb
ENSG00000137860	15	45519428	45593149	231	12	120000	0.39471	0.34653	SLC28A2	76353	25kb
ENSG00000171766	15	45628322	45719525	191	7	120000	1.9186	0.027519	GATM	76353	25kb
ENSG00000171763	15	45669529	45738617	93	8	120000	2.0443	0.020462	SPATA5L1	76353	25kb
ENSG00000166920	15	45697727	45765959	74	12	120000	2.1938	0.014126	C15orf48	76353	25kb
ENSG00000104154	15	45746809	45840005	85	16	120000	1.3977	0.081107	SLC30A4	76353	25kb
ENSG00000104164	15	45854321	45933197	120	13	120000	0.69746	0.24276	BLOC1S6	76353	25kb
ENSG00000260170	15	45854549	45993512	424	40	120000	1.2637	0.10316	RP11-96O20.4	76353	25kb
ENSG00000137767	15	45898346	46008492	417	41	120000	1.2857	0.099269	SQRDL	76353	25kb
ENSG00000259752	15	47400693	47451320	137	16	120000	-0.31753	0.62458	FKSG62	76353	25kb
ENSG00000137872	15	47451298	48091420	1940	74	120000	-0.32486	0.62735	SEMA6D	76353	25kb
ENSG00000188467	15	48388169	48459869	23	10	120000	-0.61175	0.72965	SLC24A5	76353	25kb
ENSG00000104177	15	48406625	48495714	22	6	120000	-0.73025	0.76738	MYEF2	76353	25kb
ENSG00000233932	15	48458736	48520953	15	6	120000	-0.16476	0.56544	CTXN2	76353	25kb
ENSG00000074803	15	48458861	48621275	185	14	120000	0.39386	0.34684	SLC12A1	76353	25kb
ENSG00000128951	15	48598208	48660570	100	5	120000	-0.3905	0.65192	DUT	76353	25kb
ENSG00000166147	15	48675503	48963046	531	19	120000	1.7363	0.041252	FBN1	76353	25kb
ENSG00000103995	15	48980125	49128343	278	29	120000	-0.068948	0.52748	CEP152	76353	25kb
ENSG00000185634	15	49090932	49280641	468	64	120000	-0.11086	0.54414	SHC4	76353	25kb
ENSG00000255302	15	49145083	49197380	124	19	120000	-0.034497	0.51376	EID1	76353	25kb
ENSG00000138593	15	49255673	49363760	209	24	120000	-1.4682	0.92898	SECISBP2L	76353	25kb
ENSG00000166200	15	49373268	49472858	262	21	120000	-1.0158	0.84514	COPS2	76353	25kb
ENSG00000156958	15	49422853	49685066	695	21	120000	-1.1631	0.87761	GALK2	76353	25kb
ENSG00000166262	15	49594159	49938128	973	31	120000	0.30699	0.37943	FAM227B	76353	25kb
ENSG00000140285	15	49690293	49805972	350	23	120000	0.69492	0.24355	FGF7	76353	25kb
ENSG00000104047	15	49888177	49962333	200	16	120000	0.8785	0.18984	DTWD1	76353	25kb
ENSG00000104043	15	50125435	50500014	1158	66	120000	0.89546	0.18527	ATP8B4	76353	25kb
ENSG00000140284	15	50449393	50553592	389	32	120000	1.0926	0.13728	SLC27A2	76353	25kb
ENSG00000140287	15	50509144	50583223	233	26	120000	1.1127	0.13291	HDC	76353	25kb
ENSG00000104064	15	50544389	50672605	324	24	120000	0.63106	0.264	GABPB1	76353	25kb
ENSG00000138592	15	50691577	50818280	382	26	120000	-0.90093	0.81619	USP8	76353	25kb
ENSG00000170236	15	50767759	50863905	293	20	120000	-1.0517	0.85354	USP50	76353	25kb
ENSG00000092439	15	50819670	51004012	577	21	120000	-0.72056	0.76441	TRPM7	76353	25kb
ENSG00000138600	15	50974506	51083005	328	21	120000	0.18626	0.42612	SPPL2A	76353	25kb
ENSG00000081014	15	51175869	51323097	339	18	120000	0.33591	0.36847	AP4E1	76353	25kb
ENSG00000183578	15	51323795	51422473	193	26	120000	0.65779	0.25533	TNFAIP8L3	76353	25kb
ENSG00000137869	15	51475254	51655807	534	41	120000	-0.21285	0.58428	CYP19A1	76353	25kb
ENSG00000186417	15	51608826	51725210	381	34	120000	0.012632	0.49496	GLDN	76353	25kb
ENSG00000104093	15	51714908	51940030	483	22	120000	-0.30133	0.61842	DMXL2	76353	25kb
ENSG00000104112	15	51948550	52038223	211	25	120000	1.1098	0.13354	SCG3	76353	25kb
ENSG00000140280	15	51990208	52068782	216	17	120000	0.82233	0.20544	LYSMD2	76353	25kb
ENSG00000128872	15	52018758	52133565	309	12	120000	0.7282	0.23324	TMOD2	76353	25kb
ENSG00000138594	15	52096825	52264492	377	12	120000	0.80471	0.21049	TMOD3	76353	25kb
ENSG00000166477	15	52205222	52289003	162	10	120000	1.0821	0.13961	LEO1	76353	25kb
ENSG00000069956	15	52219303	52383462	304	25	120000	0.49784	0.3093	MAPK6	76353	25kb
ENSG00000137875	15	52376460	52429972	130	21	120000	0.57494	0.28266	BCL2L10	76353	25kb
ENSG00000069966	15	52388117	52508566	331	22	120000	0.23306	0.40786	GNB5	76353	25kb
ENSG00000128833	15	52459519	52612995	406	45	120000	-1.1767	0.88034	MYO5C	76353	25kb
ENSG00000197535	15	52574480	52846247	781	33	120000	-1.2041	0.88573	MYO5A	76353	25kb
ENSG00000128989	15	52814242	52887080	186	21	120000	-0.67952	0.75159	ARPP19	76353	25kb
ENSG00000047346	15	52848514	53027014	401	43	120000	-0.64793	0.74148	FAM214A	76353	25kb
ENSG00000169856	15	53024637	53108273	229	42	120000	-1.0707	0.85785	ONECUT1	76353	25kb
ENSG00000166415	15	53780938	54080075	1185	50	120000	-0.51146	0.69549	WDR72	76353	25kb
ENSG00000137766	15	54280101	54945806	2602	118	120000	0.55495	0.28947	UNC13C	76353	25kb
ENSG00000137876	15	55448004	55514265	300	14	120000	-0.3827	0.64903	RSL24D1	76353	25kb
ENSG00000069974	15	55470164	55636311	563	37	120000	-0.0070577	0.50282	RAB27A	76353	25kb
ENSG00000069943	15	55586158	55672846	269	23	120000	0.25443	0.39958	PIGB	76353	25kb
ENSG00000260916	15	55607230	55725708	400	26	120000	-0.18065	0.57168	CCPG1	76353	25kb
ENSG00000261652	15	55675746	55735962	223	18	120000	-0.17481	0.56939	C15orf65	76353	25kb
ENSG00000256061	15	55677723	55825432	530	28	120000	0.42366	0.33591	DYX1C1	76353	25kb
ENSG00000171016	15	55806088	55906145	404	36	120000	0.31797	0.37525	PYGO1	76353	25kb
ENSG00000166450	15	55878744	56060288	577	32	120000	1.4659	0.071338	PRTG	76353	25kb
ENSG00000069869	15	56094120	56310944	844	30	120000	-0.37296	0.64541	NEDD4	76353	25kb
ENSG00000181827	15	56354478	56560483	568	20	120000	-0.53069	0.70218	RFX7	76353	25kb
ENSG00000151575	15	56511207	56763195	719	23	120000	-1.1134	0.86724	TEX9	76353	25kb
ENSG00000138587	15	56688742	56782335	252	23	120000	-1.3963	0.91868	MNS1	76353	25kb
ENSG00000137871	15	56897379	57235769	656	28	120000	-1.1309	0.87095	ZNF280D	76353	25kb
ENSG00000140262	15	57185821	57616479	1110	26	120000	-0.87415	0.80898	TCF12	76353	25kb
ENSG00000128849	15	57643165	57867925	1031	98	120000	0.21093	0.41647	CGNL1	76353	25kb
ENSG00000137878	15	57859106	58031943	706	52	120000	-0.30467	0.61969	GCOM1	76353	25kb
ENSG00000263155	15	57859139	58002562	604	41	120000	-0.6684	0.74806	MYZAP	76353	25kb
ENSG00000255529	15	57859231	58099960	923	78	120000	-0.66907	0.74828	POLR2M	76353	25kb
ENSG00000128918	15	58220622	58815065	2283	144	120000	0.35949	0.35962	ALDH1A2	76353	25kb
ENSG00000103569	15	58405368	58503110	368	38	120000	-0.16378	0.56505	AQP9	76353	25kb
ENSG00000166035	15	58677768	58886151	796	99	120000	2.322	0.010118	LIPC	76353	25kb
ENSG00000137845	15	58862403	59067177	387	21	120000	0.6439	0.25982	ADAM10	76353	25kb
ENSG00000128923	15	59038391	59179099	286	22	120000	1.1625	0.12252	FAM63B	76353	25kb
ENSG00000157450	15	59132374	59414618	577	35	120000	0.66281	0.25373	RNF111	76353	25kb
ENSG00000137776	15	59146244	59250852	163	18	120000	0.91104	0.18114	SLTM	76353	25kb
ENSG00000157456	15	59372277	59442244	128	13	120000	-0.89654	0.81502	CCNB2	76353	25kb
ENSG00000157483	15	59402113	59690099	862	72	120000	-1.5332	0.93739	MYO1E	76353	25kb
ENSG00000268327	15	59414899	59465054	134	21	120000	-1.2807	0.89984	C15ORF31	76353	25kb
ENSG00000171989	15	59474042	59525705	204	29	120000	-1.7097	0.95634	LDHAL6B	76353	25kb
ENSG00000157470	15	59639892	59840748	594	55	120000	1.1464	0.12581	FAM81A	76353	25kb
ENSG00000140297	15	59862074	59957438	351	37	120000	2.1431	0.016054	GCNT3	76353	25kb
ENSG00000140307	15	59905261	59974740	249	16	120000	1.2994	0.096898	GTF2A2	76353	25kb
ENSG00000140299	15	59926345	60006733	318	12	120000	1.789	0.036806	BNIP2	76353	25kb
ENSG00000171956	15	60271421	60378929	330	34	120000	0.22533	0.41086	FOXB1	76353	25kb
ENSG00000182718	15	60614333	60720082	290	32	120000	-0.023176	0.50925	ANXA2	76353	25kb
ENSG00000128915	15	60686808	60796359	205	21	120000	0.21747	0.41392	NARG2	76353	25kb
ENSG00000069667	15	60755483	61546518	2163	231	120000	-0.65667	0.7443	RORA	76353	25kb
ENSG00000129003	15	62119588	62377672	653	24	120000	2.0902	0.018298	VPS13C	76353	25kb
ENSG00000198535	15	62334176	62388116	164	14	120000	1.4127	0.078875	C2CD4A	76353	25kb

ENSG00000205502	15	62430734	62482482	126	21	120000	0.3884	0.34886	C2CD4B	76353	25kb
ENSG00000171914	15	62657725	63161830	1486	142	120000	0.041408	0.48349	TLN2	76353	25kb
ENSG00000259458	15	62904371	62962490	207	42	120000	-0.37446	0.64597	RP11-625H11.1	76353	25kb
ENSG00000140416	15	63309831	63389114	297	31	120000	-0.3789	0.64762	TPM1	76353	25kb
ENSG00000103642	15	63388999	63459260	264	19	120000	0.4604	0.32262	LACTB	76353	25kb
ENSG00000185088	15	63393071	63475220	287	23	120000	0.25833	0.39808	RPS27L	76353	25kb
ENSG00000166128	15	63456668	63584981	282	25	120000	-0.58041	0.71918	RAB8B	76353	25kb
ENSG00000138613	15	63543217	63626325	185	31	120000	-0.55812	0.71162	APH1B	76353	25kb
ENSG00000074410	15	63588577	63699360	303	40	120000	0.33586	0.36849	CA12	76353	25kb
ENSG00000140455	15	63771793	63911839	342	26	120000	-0.44277	0.67103	USP3	76353	25kb
ENSG00000197361	15	63864552	63919627	112	16	120000	0.097745	0.46107	FBXL22	76353	25kb
ENSG00000103657	15	63875817	64151141	685	24	120000	0.66531	0.25293	HERC1	76353	25kb
ENSG00000035664	15	64174235	64389232	701	45	120000	0.73312	0.23174	DAPK2	76353	25kb
ENSG00000166797	15	64339758	64411217	158	18	120000	1.7461	0.040397	FAM96A	76353	25kb
ENSG00000028528	15	64361322	64463289	201	19	120000	1.6057	0.054173	SNX1	76353	25kb
ENSG00000157734	15	64418914	64474680	87	12	120000	0.99724	0.15933	SNX22	76353	25kb
ENSG00000166794	15	64423011	64480404	90	13	120000	0.97201	0.16552	PPIB	76353	25kb
ENSG00000169118	15	64432716	64673442	439	24	120000	0.28421	0.38812	CSNK1G1	76353	25kb
ENSG00000259316	15	64447547	64690980	438	24	120000	0.09869	0.46069	CTD-2116N17.1	76353	25kb
ENSG00000166803	15	64632193	64704886	126	17	120000	-0.10641	0.54237	KIAA0101	76353	25kb
ENSG00000103671	15	64654947	64772502	204	22	120000	-0.35972	0.64047	TRIP4	76353	25kb
ENSG00000180357	15	64727941	65003264	494	26	120000	-0.26688	0.60522	ZNF609	76353	25kb
ENSG00000180304	15	64954772	65020480	119	12	120000	-0.42936	0.66617	OAZ2	76353	25kb
ENSG00000166831	15	65007091	65092786	224	18	120000	-1.2253	0.88977	RBPMS2	76353	25kb
ENSG00000140451	15	65082831	65142867	198	23	120000	0.15807	0.4372	PIF1	76353	25kb
ENSG00000249240	15	65109082	65248118	393	29	120000	0.15378	0.43889	AC069368.3	76353	25kb
ENSG00000241839	15	65109088	65185206	248	18	120000	0.55078	0.29089	PLEKHO2	76353	25kb
ENSG00000166839	15	65179101	65276042	259	29	120000	-0.78366	0.78338	ANKDD1A	76353	25kb
ENSG00000090487	15	65230362	65307648	205	18	120000	-0.42104	0.66314	SPG21	76353	25kb
ENSG00000103707	15	65269845	65346977	182	22	120000	-0.55296	0.70985	MTFMT	76353	25kb
ENSG00000186198	15	65312708	65370734	128	22	120000	-1.0102	0.84379	SLC51B	76353	25kb
ENSG00000103710	15	65320679	65394028	139	24	120000	-1.3083	0.90461	RASL12	76353	25kb
ENSG00000234438	15	65344154	65397276	97	20	120000	-1.2868	0.90092	KBTBD13	76353	25kb
ENSG00000246922	15	65360338	65432535	87	25	120000	-0.92909	0.82358	UBAP1L	76353	25kb
ENSG00000090470	15	65384717	65451174	59	15	120000	-0.47542	0.68276	PDCD7	76353	25kb
ENSG00000166855	15	65415557	65502680	90	17	120000	-0.41043	0.65926	CLPX	76353	25kb
ENSG00000138615	15	65463337	65528826	96	17	120000	-0.51291	0.69599	CILP	76353	25kb
ENSG00000138617	15	65501798	65617956	288	19	120000	-0.17525	0.56956	PARP16	76353	25kb
ENSG00000174498	15	65594465	65695378	253	39	120000	0.066863	0.47335	IGDCC3	76353	25kb
ENSG00000103742	15	65648802	65740410	216	39	120000	-0.38353	0.64934	IGDCC4	76353	25kb
ENSG00000074603	15	65709801	65835042	266	15	120000	-0.86035	0.8052	DPP8	76353	25kb
ENSG00000074696	15	65797756	65895687	196	13	120000	-1.1504	0.87502	PTPLAD1	76353	25kb
ENSG00000138614	15	65846091	65928627	154	11	120000	-1.1846	0.88192	VWA9	76353	25kb
ENSG00000074621	15	65878704	65978333	153	12	120000	-1.3599	0.91307	SLC24A1	76353	25kb
ENSG00000174485	15	65925384	66109631	337	17	120000	-0.87118	0.80817	DENND4A	76353	25kb
ENSG00000103769	15	65993392	66209329	440	29	120000	-1.4494	0.92639	RAB11A	76353	25kb
ENSG00000157890	15	66162417	66571085	1303	125	120000	-0.834	0.79786	MEGF11	76353	25kb
ENSG00000166938	15	66560555	66651236	229	30	120000	-0.96692	0.83321	DIS3L	76353	25kb
ENSG00000075131	15	66603544	66704084	257	22	120000	-1.1121	0.86696	TIPIN	76353	25kb
ENSG00000169032	15	66654155	66809650	438	27	120000	-1.193	0.88356	MAP2K1	76353	25kb
ENSG00000174446	15	66757473	66815151	147	18	120000	-1.3455	0.91077	SNAPC5	76353	25kb
ENSG00000174444	15	66765355	66841870	212	21	120000	-1.5528	0.93976	RPL4	76353	25kb
ENSG00000174442	15	66772297	66867115	286	23	120000	-1.7158	0.9569	ZWILCH	76353	25kb
ENSG00000188501	15	66814517	66883317	262	28	120000	-2.0754	0.98103	LCTL	76353	25kb
ENSG00000259471	15	66849528	67003132	653	71	120000	-0.86187	0.80562	RP11-321F6.1	76353	25kb
ENSG00000137834	15	66969566	67099338	395	52	120000	-0.99833	0.84094	SMAD6	76353	25kb
ENSG00000166949	15	67331101	67512533	530	68	120000	-1.0187	0.84583	SMAD3	76353	25kb
ENSG00000259202	15	67410072	67464277	174	31	120000	-0.24092	0.59519	RP11-342M21.2	76353	25kb
ENSG00000103591	15	67468371	67572533	214	17	120000	0.0079735	0.49682	AAGAB	76353	25kb
ENSG00000103599	15	67522138	67819598	507	24	120000	-0.38793	0.65097	IQCH	76353	25kb
ENSG00000189227	15	67788406	67844628	127	11	120000	0.38373	0.35059	C15orf61	76353	25kb
ENSG00000137764	15	67810047	68124461	773	29	120000	-0.68268	0.7526	MAP2K5	76353	25kb
ENSG00000188779	15	68087042	68151899	219	26	120000	-1.877	0.96974	SKOR1	76353	25kb
ENSG00000033800	15	68321517	68508096	247	24	120000	0.80493	0.21043	PIAS1	76353	25kb
ENSG00000129007	15	68458043	68523417	112	12	120000	0.52748	0.29893	CALML4	76353	25kb
ENSG00000260007	15	68461139	68547056	157	14	120000	0.53101	0.29771	RP11-315D16.2	76353	25kb
ENSG00000128973	15	68474330	68574549	205	21	120000	0.97214	0.16549	CLN6	76353	25kb
ENSG00000169018	15	68545141	68613203	164	27	120000	0.55309	0.2901	FEM1B	76353	25kb
ENSG00000137809	15	68569050	68749501	664	68	120000	1.4882	0.068347	ITGA11	76353	25kb
ENSG00000103647	15	68846308	69045145	628	64	120000	0.67723	0.24913	CORO2B	76353	25kb
ENSG00000140350	15	69045874	69138236	194	33	120000	-0.0017549	0.5007	ANP32A	76353	25kb
ENSG00000258484	15	69085560	69264150	275	26	120000	-0.42461	0.66444	SPESP1	76353	25kb
ENSG00000255346	15	69197864	69380083	300	24	120000	-1.6422	0.94972	NOX5	76353	25kb
ENSG00000138604	15	69427923	69589556	293	18	120000	-0.9359	0.82534	GLCE	76353	25kb
ENSG00000137819	15	69566286	69725119	393	29	120000	0.088358	0.4648	PAQR5	76353	25kb
ENSG00000137807	15	69681585	69765764	115	14	120000	1.1236	0.13058	KIF23	76353	25kb
ENSG00000137818	15	69720123	69773255	73	14	120000	1.0425	0.1486	RPLP1	76353	25kb
ENSG00000140332	15	70315129	70415515	274	44	120000	0.82111	0.20579	TLE3	76353	25kb
ENSG00000137831	15	70921893	71080932	311	23	120000	0.6476	0.25862	UACA	76353	25kb
ENSG00000166173	15	71098863	71171498	179	14	120000	0.95551	0.16966	LARP6	76353	25kb
ENSG00000137821	15	71120578	71367414	292	25	120000	1.1614	0.12273	LRRC49	76353	25kb
ENSG00000129028	15	71148681	71210124	109	9	120000	1.244	0.10676	THAP10	76353	25kb
ENSG00000187720	15	71364291	72100722	2330	179	120000	2.3006	0.010706	THSD4	76353	25kb
ENSG00000225362	15	71377583	71432833	99	20	120000	0.4763	0.31693	CT62	76353	25kb
ENSG00000066933	15	72089632	72435918	549	20	120000	0.86747	0.19284	MYO9A	76353	25kb
ENSG00000166192	15	72381599	72458311	129	17	120000	1.2105	0.11304	SENP8	76353	25kb
ENSG00000175318	15	72427148	72515126	139	19	120000	1.1272	0.12982	GRAMD2	76353	25kb
ENSG00000067225	15	72466370	72549164	138	20	120000	0.64083	0.26082	PKM	76353	25kb
ENSG00000137817	15	72508522	72590340	168	22	120000	0.18681	0.42591	PARP6	76353	25kb
ENSG00000273025	15	72534087	72637287	236	29	120000	-0.35381	0.63826	CELF6	76353	25kb
ENSG00000140488	15	72552068	72637470	206	26	120000	-0.4704	0.68097	CELF6	76353	25kb
ENSG00000213614	15	72610775	72693817	176	21	120000	-0.050057	0.51996	HEXA	76353	25kb
ENSG00000187806	15	72665614	72725370	101	11	120000	0.2581	0.39817	TMEM202	76353	25kb
ENSG00000166233	15	72741667	72904692	225	26	120000	-0.44104	0.67041	ARIH1	76353	25kb
ENSG00000215186	15	72922079	72983735	41	17	120000	-0.073801	0.52942	GOLGA6B	76353	25kb
ENSG00000175202	15	72943124	73003490	74	17	120000	-0.42693	0.66528	HIGD2B	76353	25kb

ENSG00000140463	15	72953527	73055817	161	22	120000	-0.24396	0.59637	BBS4	76353	25kb
ENSG00000159322	15	73018710	73103187	171	18	120000	-0.35929	0.64031	ADPGK	76353	25kb
ENSG00000067141	15	73319051	73622547	672	26	120000	2.1941	0.014114	NEO1	76353	25kb
ENSG00000138622	15	73587200	73686605	252	29	120000	0.96332	0.16769	HCN4	76353	25kb
ENSG00000183324	15	73710499	73877355	328	20	120000	-0.35603	0.63909	C15orf60	76353	25kb
ENSG00000156642	15	73827355	73951475	220	22	120000	-0.42702	0.66532	NPTN	76353	25kb
ENSG00000103855	15	73951307	74031859	241	33	120000	-0.12175	0.54845	CD276	76353	25kb
ENSG00000205363	15	74007141	74070088	202	30	120000	0.2541	0.39971	C15orf59	76353	25kb
ENSG00000167139	15	74140949	74206555	202	28	120000	-1.0873	0.86155	TBC1D21	76353	25kb
ENSG00000129038	15	74193330	74269478	261	36	120000	0.40863	0.34141	LOXL1	76353	25kb
ENSG00000067221	15	74250547	74311963	164	19	120000	0.29916	0.38241	STOML1	76353	25kb
ENSG00000140464	15	74262014	74365153	239	28	120000	-0.32239	0.62642	PML	76353	25kb
ENSG00000159289	15	74337198	74399891	44	11	120000	-1.9156	0.97229	GOLGA6A	76353	25kb
ENSG00000167178	15	74367652	74455881	53	19	120000	-1.2253	0.88977	ISLR2	76353	25kb
ENSG00000248540	15	74393256	74446619	39	17	120000	-0.78727	0.78444	RP11-247C2.2	76353	25kb
ENSG00000129009	15	74441012	74494213	115	15	120000	-0.86176	0.80559	ISLR	76353	25kb
ENSG00000137868	15	74446807	74529608	194	27	120000	-1.0781	0.8595	STRA6	76353	25kb
ENSG00000140481	15	74484613	74653813	468	50	120000	-0.57546	0.71751	CCDC33	76353	25kb
ENSG00000140459	15	74605100	74685081	210	22	120000	0.055687	0.4778	CYP11A1	76353	25kb
ENSG00000138623	15	74676630	74751808	183	26	120000	-1.7187	0.95717	SEMA7A	76353	25kb
ENSG00000138629	15	74713318	74778523	145	13	120000	-1.1472	0.87436	UBL7	76353	25kb
ENSG00000179361	15	74808518	74915472	161	11	120000	-0.92154	0.82161	ARID3B	76353	25kb
ENSG00000179335	15	74865841	74957057	112	12	120000	-0.83431	0.79795	CLK3	76353	25kb
ENSG00000179151	15	74897899	75013633	109	16	120000	-0.94775	0.82837	EDC3	76353	25kb
ENSG00000140465	15	74986883	75042951	86	16	120000	-0.94597	0.82792	CYP1A1	76353	25kb
ENSG00000140505	15	75016185	75073543	150	19	120000	-0.44869	0.67317	CYP1A2	76353	25kb
ENSG00000103653	15	75049398	75120539	170	19	120000	-0.85466	0.80363	CSK	76353	25kb
ENSG00000140506	15	75080057	75143099	121	15	120000	-0.61401	0.73039	LMAN1L	76353	25kb
ENSG00000213578	15	75093888	75149141	106	15	120000	-0.2911	0.61451	CPLX3	76353	25kb
ENSG00000140474	15	75103457	75160687	97	15	120000	0.13389	0.44674	ULK3	76353	25kb
ENSG00000140497	15	75111071	75190706	116	16	120000	0.63978	0.26116	SCAMP2	76353	25kb
ENSG00000178802	15	75157346	75216798	75	10	120000	0.77264	0.21987	MPI	76353	25kb
ENSG00000178761	15	75167328	75224462	84	11	120000	0.75128	0.22624	FAM219B	76353	25kb
ENSG00000178741	15	75187132	75255509	114	14	120000	0.73298	0.23179	COX5A	76353	25kb
ENSG00000178718	15	75221757	75274805	102	15	120000	0.54782	0.29191	RPP25	76353	25kb
ENSG00000198794	15	75224560	75338837	265	22	120000	0.11038	0.45605	SCAMP5	76353	25kb
ENSG00000138621	15	75290896	75434803	377	21	120000	0.026794	0.48931	PPCDC	76353	25kb
ENSG00000167173	15	75462984	75529510	99	23	120000	0.5986	0.27472	C15orf39	76353	25kb
ENSG00000167195	15	75525940	75587540	13	7	120000	0.10481	0.45826	GOLGA6C	76353	25kb
ENSG00000140478	15	75550176	75611816	43	9	120000	-0.79826	0.78764	GOLGA6D	76353	25kb
ENSG00000140365	15	75603232	75659268	83	14	120000	-0.4099	0.65906	COMMD4	76353	25kb
ENSG00000140398	15	75614296	75672592	74	16	120000	-0.27257	0.60741	NEIL1	76353	25kb
ENSG00000140400	15	75623133	75685971	70	16	120000	-0.40614	0.65768	MAN2C1	76353	25kb
ENSG00000169375	15	75636720	75773183	149	17	120000	-0.11188	0.54454	SIN3A	76353	25kb
ENSG00000169410	15	75734462	75896630	208	20	120000	0.10496	0.4582	PTPN9	76353	25kb
ENSG00000169371	15	75865424	75943810	120	13	120000	0.67347	0.25032	SNUPN	76353	25kb
ENSG00000177971	15	75906426	75966047	107	19	120000	0.14533	0.44223	IMP3	76353	25kb
ENSG00000173548	15	75915247	75979642	119	22	120000	-0.029848	0.51191	SNX33	76353	25kb
ENSG00000173546	15	75941663	76030189	140	20	120000	0.7131	0.23789	CSPG4	76353	25kb
ENSG00000203392	15	75945672	75998093	92	15	120000	-0.022904	0.50914	AC105020.1	76353	25kb
ENSG00000182950	15	75991318	76045029	95	9	120000	1.5343	0.062481	ODF3L1	76353	25kb
ENSG00000256530	15	76005311	76056568	118	8	120000	1.5262	0.063476	AC019294.1	76353	25kb
ENSG00000140367	15	76110622	76218419	182	19	120000	-0.18656	0.574	UBE2Q2	76353	25kb
ENSG00000167196	15	76171200	76252609	162	12	120000	-0.022766	0.50908	FBXO22	76353	25kb
ENSG00000169752	15	76203310	76377136	340	13	120000	-0.17038	0.56764	NRG4	76353	25kb
ENSG00000169758	15	76327178	76546462	492	53	120000	-1.6438	0.94989	C15orf27	76353	25kb
ENSG00000140374	15	76482696	76628813	297	16	120000	-0.049994	0.51994	ETFA	76353	25kb
ENSG00000159556	15	76604065	76659817	98	18	120000	0.33833	0.36756	ISL2	76353	25kb
ENSG00000140386	15	76615526	77222785	1138	26	120000	0.58955	0.27774	SCAPER	76353	25kb
ENSG00000117906	15	77198960	77267601	116	26	120000	0.69945	0.24214	RCN2	76353	25kb
ENSG00000140368	15	77260700	77354673	168	28	120000	-0.32246	0.62645	PSTPIP1	76353	25kb
ENSG00000140391	15	77311359	77401326	132	23	120000	2.0964	0.018023	TSPAN3	76353	25kb
ENSG00000173517	15	77375471	77737486	521	23	120000	1.7883	0.036864	PEAK1	76353	25kb
ENSG00000140382	15	77687754	77802949	189	18	120000	1.3984	0.080995	HMG20A	76353	25kb
ENSG00000169783	15	77880369	78138242	813	46	120000	1.1271	0.12986	LINGO1	76353	25kb
ENSG00000167202	15	78251378	78395066	491	37	120000	0.34644	0.3645	TBC1D2B	76353	25kb
ENSG00000183476	15	78345150	78422251	238	41	120000	1.407	0.07972	SH2D7	76353	25kb
ENSG00000136425	15	78371948	78448886	210	35	120000	2.0365	0.020849	CIB2	76353	25kb
ENSG00000166411	15	78398840	78489291	235	33	120000	2.4739	0.0066825	IDH3A	76353	25kb
ENSG00000103740	15	78434810	78563030	393	51	120000	2.2513	0.012183	ACSBG1	76353	25kb
ENSG00000140403	15	78531428	78599538	228	31	120000	0.11769	0.45316	DNAJA4	76353	25kb
ENSG00000140395	15	78545177	78617136	213	33	120000	-0.38031	0.64814	WDR61	76353	25kb
ENSG00000166426	15	78607666	78665572	136	31	120000	1.5721	0.057961	CRABP1	76353	25kb
ENSG00000136381	15	78704773	78818798	258	21	120000	0.9989	0.15892	IREB2	76353	25kb
ENSG00000188266	15	78774906	78854714	190	17	120000	0.32455	0.37276	HYKK	76353	25kb
ENSG00000268838	15	78805023	78856288	138	10	120000	0.078989	0.46852	AC027228.1	76353	25kb
ENSG00000041357	15	78807747	78866604	157	11	120000	0.067195	0.47321	PSMA4	76353	25kb
ENSG00000169684	15	78832862	78912611	193	17	120000	0.079065	0.46849	CHRNA5	76353	25kb
ENSG00000080644	15	78860394	78938637	174	16	120000	0.21237	0.41591	CHRNA3	76353	25kb
ENSG00000117971	15	78891461	79045096	417	30	120000	-0.54139	0.70588	CHRNB4	76353	25kb
ENSG00000136378	15	79026545	79128773	380	21	120000	-0.2634	0.60388	ADAMTS7	76353	25kb
ENSG00000185787	15	79077829	79215475	478	27	120000	-0.53221	0.70271	MORF4L1	76353	25kb
ENSG00000103811	15	79188400	79266916	251	29	120000	-1.0079	0.84326	CTSH	76353	25kb
ENSG00000058335	15	79227289	79408115	581	79	120000	-0.15518	0.56166	RASGRF1	76353	25kb
ENSG00000235711	15	79550146	79615580	144	24	120000	1.0508	0.14667	ANKRD34C	76353	25kb
ENSG00000166557	15	79578404	79729334	472	36	120000	0.93315	0.17537	TMED3	76353	25kb
ENSG00000169330	15	79699858	79789632	231	35	120000	-0.68171	0.75229	KIAA1024	76353	25kb
ENSG00000136371	15	80100927	80214721	386	25	120000	-0.35519	0.63878	MTHFS	76353	25kb
ENSG00000259332	15	80112537	80241096	422	26	120000	-0.83773	0.79891	ST20-MTHFS	76353	25kb
ENSG00000180953	15	80166182	80241044	276	18	120000	-1.0907	0.8623	ST20	76353	25kb
ENSG00000259642	15	80190113	80242194	208	19	120000	-0.94814	0.82847	C15orf37	76353	25kb
ENSG00000257028	15	80190507	80242194	207	19	120000	-0.94322	0.82722	C15ORF37	76353	25kb
ENSG00000140379	15	80228231	80288788	125	21	120000	0.074322	0.47038	BCL2A1	76353	25kb
ENSG00000086666	15	80326910	80455735	303	28	120000	0.064602	0.47425	ZFAND6	76353	25kb
ENSG00000103876	15	80419832	80504288	259	28	120000	-1.1148	0.86754	FAH	76353	25kb
ENSG00000259495	15	80612195	80720917	447	32	120000	0.40622	0.34229	RP11-210M15.2	76353	25kb

ENSG00000172379	15	80671692	80915278	658	68	120000	0.71159	0.23836	ARNT2	76353	25kb
ENSG00000136379	15	80947025	81072962	399	38	120000	-0.77666	0.78132	ABHD17C	76353	25kb
ENSG00000103888	15	81046684	81269117	539	92	120000	-0.66144	0.74583	KIAA1199	76353	25kb
ENSG00000259649	15	81163532	81227118	129	29	120000	0.22022	0.41285	RP11-351M8.1	76353	25kb
ENSG00000117899	15	81214667	81307219	222	17	120000	-1.249	0.89417	MESDC2	76353	25kb
ENSG00000140406	15	81268295	81321342	158	12	120000	-1.268	0.89761	MESDC1	76353	25kb
ENSG00000156206	15	81274374	81466516	518	39	120000	-1.9227	0.97274	C15orf26	76353	25kb
ENSG00000172349	15	81426916	81630104	530	46	120000	0.041168	0.48358	IL16	76353	25kb
ENSG00000172345	15	81576394	81641524	179	30	120000	1.2512	0.10543	STARD5	76353	25kb
ENSG00000188869	15	81598558	81691554	245	30	120000	1.2712	0.10183	TMC3	76353	25kb
ENSG00000183496	15	82309119	82363482	150	28	120000	-0.72672	0.7663	MEX3B	76353	25kb
ENSG00000259518	15	82355935	82415035	237	49	120000	-0.60913	0.72878	RP11-597K23.2	76353	25kb
ENSG00000140598	15	82397571	82580104	519	39	120000	-0.95728	0.83079	EFTUD1	76353	25kb
ENSG00000188659	15	82530151	82602271	134	22	120000	-0.9596	0.83137	FAM154B	76353	25kb
ENSG00000259243	15	82986379	83043198	1	1	120000	1.1771	0.11959	GOLGA6L19	76353	25kb
ENSG00000182774	15	83180504	83234315	42	11	120000	0.16208	0.43562	RPS17L	76353	25kb
ENSG00000260836	15	83183783	83249682	86	17	120000	0.38933	0.34852	RP11-152F13.10	76353	25kb
ENSG00000261086	15	83184620	83256124	103	18	120000	0.42194	0.33653	RP11-379H8.1	76353	25kb
ENSG00000214575	15	83186951	83342612	313	23	120000	0.18438	0.42686	CPEB1	76353	25kb
ENSG00000103723	15	83303033	83403666	230	31	120000	0.18153	0.42798	AP3B2	76353	25kb
ENSG00000186628	15	83399114	83499822	227	31	120000	-0.73652	0.76929	FSD2	76353	25kb
ENSG00000156232	15	83453380	83528611	189	28	120000	-0.61469	0.73062	WHAMM	76353	25kb
ENSG00000103942	15	83484838	83679661	429	35	120000	-0.74439	0.77168	HOMER2	76353	25kb
ENSG00000169612	15	83629959	83684809	104	16	120000	-0.31823	0.62484	FAM103A1	76353	25kb
ENSG00000169609	15	83632193	83705393	143	23	120000	-0.41638	0.66143	C15orf40	76353	25kb
ENSG00000064726	15	83660174	83761106	225	30	120000	-0.43982	0.66997	BTBD1	76353	25kb
ENSG00000136404	15	83751159	83838606	134	19	120000	-0.068745	0.5274	TM6SF1	76353	25kb
ENSG00000166503	15	83759320	83901770	235	17	120000	0.052227	0.47917	HDGFRP3	76353	25kb
ENSG00000169594	15	83899655	83978466	116	13	120000	-0.64321	0.73996	BNC1	76353	25kb
ENSG00000140600	15	84090980	84312495	513	59	120000	-0.87427	0.80901	SH3GL3	76353	25kb
ENSG00000156218	15	84297838	84733594	1068	47	120000	-0.86723	0.80709	ADAMTSL3	76353	25kb
ENSG00000259511	15	84816242	84875986	37	8	120000	0.34929	0.36343	UBE2Q2L	76353	25kb
ENSG00000184206	15	84879525	84939120	89	18	120000	-0.29604	0.6164	GOLGA6L4	76353	25kb
ENSG00000176371	15	85119217	85196027	143	21	120000	1.0067	0.15703	ZSCAN2	76353	25kb
ENSG00000177082	15	85160999	85222574	133	18	120000	1.1781	0.11937	WDR73	76353	25kb
ENSG00000197696	15	85173360	85226794	114	17	120000	1.1884	0.11733	NMB	76353	25kb
ENSG00000140612	15	85187775	85284947	203	25	120000	0.60415	0.27287	SEC11A	76353	25kb
ENSG00000166716	15	85266866	85374659	218	25	120000	0.36361	0.35807	ZNF592	76353	25kb
ENSG00000136383	15	85334911	85441713	300	38	120000	-0.08681	0.53459	ALPK3	76353	25kb
ENSG00000156222	15	85402885	85543876	552	70	120000	-0.34834	0.63621	SLC28A1	76353	25kb
ENSG00000073417	15	85498671	85707376	707	36	120000	0.96857	0.16638	PDE8A	76353	25kb
ENSG00000170776	15	85898802	86317586	1471	45	120000	-0.48698	0.68686	AKAP13	76353	25kb
ENSG00000183655	15	86277554	86363261	396	44	120000	-0.62856	0.73518	KLHL25	76353	25kb
ENSG00000166748	15	86660227	87597283	3767	149	120000	-0.59406	0.72377	AGBL1	76353	25kb
ENSG00000140538	15	88393230	88824999	952	81	120000	-0.69863	0.75761	NTRK3	76353	25kb
ENSG00000259494	15	88977707	89035650	44	15	120000	-0.0091983	0.50367	MRPL46	76353	25kb
ENSG00000181991	15	88985684	89047222	69	20	120000	-0.18532	0.57351	MRPS11	76353	25kb
ENSG00000140543	15	89029790	89114906	214	20	120000	-0.33124	0.62977	DET1	76353	25kb
ENSG00000181026	15	89139527	89200513	240	36	120000	0.2213	0.41243	AEN	76353	25kb
ENSG00000172183	15	89154384	89224714	302	34	120000	0.13478	0.44639	ISG20	76353	25kb
ENSG00000157766	15	89321674	89443585	415	66	120000	-1.4783	0.93034	ACAN	76353	25kb
ENSG00000140511	15	89395519	89463857	227	37	120000	-1.5746	0.94233	HAPLN3	76353	25kb
ENSG00000140545	15	89416916	89481642	225	32	120000	-1.4086	0.92053	MFGE8	76353	25kb
ENSG00000140526	15	89605690	89770591	416	44	120000	0.09737	0.46122	ABHD2	76353	25kb
ENSG00000140522	15	89728100	89789982	157	16	120000	0.098805	0.46065	RLBP1	76353	25kb
ENSG00000140525	15	89762180	89885492	238	15	120000	0.052939	0.47889	FANCI	76353	25kb
ENSG00000140521	15	89834534	89903092	144	17	120000	-0.51903	0.69813	POLG	76353	25kb
ENSG00000140519	15	89973680	90064844	197	35	120000	-1.6316	0.94861	RHCG	76353	25kb
ENSG00000140534	15	90093713	90199287	297	14	120000	-0.22989	0.59091	TICRR	76353	25kb
ENSG00000166813	15	90127020	90223682	232	25	120000	0.09571	0.46188	KIF7	76353	25kb
ENSG00000166819	15	90182596	90247658	185	30	120000	-0.33918	0.63276	PLIN1	76353	25kb
ENSG00000166821	15	90195995	90259014	197	25	120000	-0.52134	0.69893	PEX11A	76353	25kb
ENSG00000140527	15	90209028	90311869	361	21	120000	-0.88715	0.8125	WDR93	76353	25kb
ENSG00000166823	15	90266892	90319541	193	12	120000	-1.1771	0.88043	MESP1	76353	25kb
ENSG00000188095	15	90278822	90346982	264	13	120000	-0.79683	0.78723	MESP2	76353	25kb
ENSG00000166825	15	90303120	90383633	298	28	120000	-0.52222	0.69924	ANPEP	76353	25kb
ENSG00000157823	15	90348831	90462574	277	22	120000	-0.42619	0.66501	AP3S2	76353	25kb
ENSG00000250021	15	90352540	90481114	304	25	120000	-0.59412	0.72378	C15orf38-AP3S2	76353	25kb
ENSG00000242498	15	90418159	90481188	159	21	120000	-1.116	0.86778	C15orf38	76353	25kb
ENSG00000140548	15	90519624	90650438	322	46	120000	1.1213	0.13108	ZNF710	76353	25kb
ENSG00000182054	15	90601277	90670736	208	25	120000	2.334	0.009799	IDH2	76353	25kb
ENSG00000185033	15	90678836	90797911	426	30	120000	-0.33283	0.63037	SEMA4B	76353	25kb
ENSG00000185043	15	90748207	90802279	210	21	120000	-1.1247	0.86964	CIB1	76353	25kb
ENSG00000183208	15	90752040	90810315	213	21	120000	-1.0488	0.85286	GDPGP1	76353	25kb
ENSG00000213471	15	90767762	90833199	188	17	120000	-0.68756	0.75413	TTLL13	76353	25kb
ENSG00000261147	15	90767788	90840440	207	19	120000	-0.57797	0.71836	RP11-697E2.6	76353	25kb
ENSG00000182768	15	90783891	90841463	151	15	120000	-0.21945	0.58685	NGRN	76353	25kb
ENSG00000238244	15	90865819	90917669	232	15	120000	1.6799	0.046492	GABARAPL3	76353	25kb
ENSG00000196391	15	90870477	90934324	277	13	120000	1.3691	0.085477	ZNF774	76353	25kb
ENSG00000140575	15	90906450	91070475	454	19	120000	0.78648	0.21579	IQGAP1	76353	25kb
ENSG00000140577	15	91048157	91213577	489	30	120000	0.24899	0.40168	CRTC3	76353	25kb
ENSG00000197299	15	91235558	91383859	455	28	120000	-0.80592	0.78986	BLM	76353	25kb
ENSG00000140564	15	91386822	91451688	163	31	120000	-0.81771	0.79324	FURIN	76353	25kb
ENSG00000182511	15	91401925	91464006	167	30	120000	-0.90311	0.81677	FES	76353	25kb
ENSG00000196547	15	91420448	91490814	216	30	120000	-0.50505	0.69324	MAN2A2	76353	25kb
ENSG00000140553	15	91448410	91522323	254	20	120000	-0.30816	0.62102	UNC45A	76353	25kb
ENSG00000184508	15	91449148	91500799	175	20	120000	-0.026267	0.51048	HDDC3	76353	25kb
ENSG00000166965	15	91473100	91531349	206	23	120000	-0.93643	0.82548	RCCD1	76353	25kb
ENSG00000198901	15	91484270	91563859	270	20	120000	-0.90737	0.81789	PRC1	76353	25kb
ENSG00000184056	15	91516646	91590833	277	31	120000	-0.18765	0.57442	VPS33B	76353	25kb
ENSG00000185518	15	91618180	91869539	741	62	120000	1.9677	0.024551	SV2B	76353	25kb
ENSG00000176463	15	92371925	92740665	1264	97	120000	-1.9057	0.97166	SLCO3A1	76353	25kb
ENSG00000140557	15	92912058	93036958	398	49	120000	-0.28534	0.61231	ST8SIA2	76353	25kb
ENSG00000183643	15	92989884	93069347	260	48	120000	0.0035744	0.49857	C15orf32	76353	25kb
ENSG00000185442	15	93135673	93378114	938	76	120000	-0.61751	0.73155	FAM174B	76353	25kb
ENSG00000173575	15	93401526	93596237	592	58	120000	-0.87963	0.81047	CHD2	76353	25kb

ENSG00000182175	15	93561636	93657433	438	81	120000	0.40723	0.34192	RGMA	76353	25kb
ENSG00000269360	15	93724295	93776277	205	34	120000	-1.4647	0.9285	AC112693.2	76353	25kb
ENSG00000140563	15	94749767	95048632	975	112	120000	-1.211	0.88706	MCTP2	76353	25kb
ENSG00000205148	15	96806280	96856660	108	19	120000	0.54325	0.29348	AC016251.1	76353	25kb
ENSG00000185551	15	96844167	96908492	132	40	120000	0.097759	0.46106	NR2F2	76353	25kb
ENSG00000268484	15	96879487	96929900	139	35	120000	-0.37372	0.64569	AC087477.1	76353	25kb
ENSG00000185594	15	97301619	97353845	213	42	120000	0.17117	0.43205	SPATA8	76353	25kb
ENSG00000251209	15	98260448	98442780	761	54	120000	-0.38375	0.64942	LINC00923	76353	25kb
ENSG00000140450	15	98437784	98542068	476	55	120000	-0.35821	0.63991	ARRDC4	76353	25kb
ENSG00000185087	15	98955391	99082611	422	39	120000	-1.599	0.94509	FAM169B	76353	25kb
ENSG00000140443	15	99167200	99532759	1070	118	120000	1.1724	0.12053	IGF1R	76353	25kb
ENSG00000183571	15	99486459	99576024	357	39	120000	-0.063891	0.52547	PGPEP1L	76353	25kb
ENSG00000182253	15	99613420	99700798	375	36	120000	1.2746	0.10123	SYNM	76353	25kb
ENSG00000103852	15	99651528	99816428	528	30	120000	-0.70537	0.75971	TTC23	76353	25kb
ENSG00000168904	15	99766567	99955934	620	41	120000	-0.010309	0.50411	LRRC28	76353	25kb
ENSG00000268730	15	99834300	99884828	151	16	120000	0.48703	0.31312	AC022819.2	76353	25kb
ENSG00000068305	15	99992370	100281671	810	63	120000	-0.0052725	0.5021	MEF2A	76353	25kb
ENSG00000269810	15	100013447	100063578	234	32	120000	0.21979	0.41302	AC015660.1	76353	25kb
ENSG00000183060	15	100230902	100298766	262	34	120000	-0.49212	0.68868	LYSMD4	76353	25kb
ENSG00000214397	15	100230908	100283029	169	25	120000	-0.53866	0.70494	DKFZP779J2370	76353	25kb
ENSG00000259363	15	100322228	100442353	579	27	120000	-1.2926	0.90192	CTD-2054N24.2	76353	25kb
ENSG00000140470	15	100486794	100907210	2188	154	120000	2.0617	0.019616	ADAMTS17	76353	25kb
ENSG00000154227	15	100915600	101110200	857	37	120000	0.69546	0.24338	CERS3	76353	25kb
ENSG00000140471	15	101074574	101168435	433	35	120000	1.7437	0.040606	LINS	76353	25kb
ENSG00000183475	15	101117739	101216910	336	46	120000	0.74206	0.22903	ASB7	76353	25kb
ENSG00000184254	15	101392919	101481831	220	40	120000	-1.9548	0.9747	ALDH1A3	76353	25kb
ENSG00000154237	15	101434420	101635317	713	61	120000	-0.055174	0.522	LRRK1	76353	25kb
ENSG00000131873	15	101690928	101817137	465	42	120000	0.16272	0.43537	CHSY1	76353	25kb
ENSG00000131871	15	101786022	101842705	185	25	120000	0.38173	0.35133	VIMP	76353	25kb
ENSG00000131876	15	101796715	101860487	200	26	120000	0.40497	0.34275	SNRPA1	76353	25kb
ENSG00000140479	15	101815818	102090405	1207	149	120000	-1.3638	0.91369	PCSK6	76353	25kb
ENSG00000184277	15	102136847	102217594	253	27	120000	-2.2401	0.98746	TM2D3	76353	25kb
ENSG00000185418	15	102168801	102289807	231	26	120000	-1.3986	0.91903	TARSL2	76353	25kb
ENSG00000259658	15	102252302	102310913	71	13	120000	-1.3766	0.91568	RP11-89K11.1	76353	25kb
ENSG00000184140	15	102320902	102371876	127	19	120000	-1.3775	0.91582	OR4F6	76353	25kb
ENSG00000182854	15	102333366	102384350	129	18	120000	-1.2409	0.89268	OR4F15	76353	25kb
ENSG00000177693	15	102437245	102488298	2	1	120000	0.22186	0.41221	OR4F4	76353	25kb
ENSG00000234769	16	39043	94452	65	9	120000	-0.72406	0.76549	WASH4P	76353	25kb
ENSG00000161980	16	71407	128628	165	22	120000	-0.46503	0.67904	POLR3K	76353	25kb
ENSG00000161981	16	78010	132669	176	23	120000	-0.39271	0.65273	SNRNP25	76353	25kb
ENSG00000007384	16	83058	151354	214	29	120000	-0.40503	0.65727	RHBDF1	76353	25kb
ENSG00000103152	16	102006	160852	146	22	120000	0.19245	0.42369	MPG	76353	25kb
ENSG00000103148	16	109273	213859	254	33	120000	0.28216	0.38891	NPRL3	76353	25kb
ENSG00000130656	16	177686	229502	110	25	120000	0.14459	0.44252	HBZ	76353	25kb
ENSG00000206177	16	178891	241767	131	28	120000	0.18175	0.42789	HBM	76353	25kb
ENSG00000188536	16	197846	248709	108	20	120000	0.44388	0.32857	HBA2	76353	25kb
ENSG00000206172	16	201679	252521	105	20	120000	0.50733	0.30596	HBA1	76353	25kb
ENSG00000086506	16	205452	256180	100	19	120000	0.53988	0.29464	HBQ1	76353	25kb
ENSG00000007392	16	213968	304462	181	25	120000	0.33845	0.36751	LUC7L	76353	25kb
ENSG00000167930	16	259545	344942	246	43	120000	-0.00073088	0.50029	ITFG3	76353	25kb
ENSG00000269881	16	274546	344942	227	41	120000	0.05511	0.47803	ITFG3	76353	25kb
ENSG00000076344	16	293300	350980	200	35	120000	0.22078	0.41263	RGS11	76353	25kb
ENSG00000242173	16	293726	358003	229	39	120000	0.027054	0.48921	ARHGDIG	76353	25kb
ENSG00000185615	16	308152	362215	201	37	120000	-0.63976	0.73884	PDIA2	76353	25kb
ENSG00000103126	16	312440	427673	459	41	120000	-1.3887	0.91754	AXIN1	76353	25kb
ENSG00000086504	16	392384	445527	256	26	120000	-0.9114	0.81896	MRPL28	76353	25kb
ENSG00000129925	16	395773	462113	290	30	120000	-1.0436	0.85165	TMEM8A	76353	25kb
ENSG00000103202	16	421725	485367	217	29	120000	-0.91931	0.82103	NME4	76353	25kb
ENSG00000242612	16	426826	487487	195	26	120000	-0.95953	0.83135	DECR2	76353	25kb
ENSG00000090565	16	450619	598011	380	36	120000	-1.6577	0.95131	RAB11FIP3	76353	25kb
ENSG00000103326	16	552717	629636	218	50	120000	-0.60058	0.72594	CAPN15	76353	25kb
ENSG00000161992	16	585422	640528	186	31	120000	0.066704	0.47341	C16orf11	76353	25kb
ENSG00000007541	16	591995	659136	222	25	120000	0.076239	0.46961	PIGQ	76353	25kb
ENSG00000257108	16	591996	644495	184	26	120000	0.11438	0.45447	NHLRC4	76353	25kb
ENSG00000197562	16	614357	704272	248	16	120000	0.10507	0.45816	RAB40C	76353	25kb
ENSG00000127578	16	654239	709116	142	16	120000	-0.028996	0.51157	WFIKKN1	76353	25kb
ENSG00000130731	16	659429	711358	138	16	120000	-0.10635	0.54235	C16orf13	76353	25kb
ENSG00000172366	16	666813	723474	171	16	120000	-0.33105	0.6297	FAM195A	76353	25kb
ENSG00000161996	16	674311	742833	183	17	120000	-0.56774	0.71489	WDR90	76353	25kb
ENSG00000140983	16	693086	749174	149	12	120000	-0.85825	0.80462	RHOT2	76353	25kb
ENSG00000103269	16	700666	753268	144	12	120000	-0.84834	0.80188	RHBDL1	76353	25kb
ENSG00000103266	16	705224	757870	145	12	120000	-0.91306	0.81939	STUB1	76353	25kb
ENSG00000161999	16	706671	759529	138	12	120000	-0.97173	0.83441	JMJD8	76353	25kb
ENSG00000127580	16	709622	765444	143	15	120000	-0.9544	0.83006	WDR24	76353	25kb
ENSG00000127585	16	717500	780829	154	21	120000	-0.98613	0.83796	FBXL16	76353	25kb
ENSG00000103260	16	740115	794655	139	18	120000	-0.64568	0.74076	METRN	76353	25kb
ENSG00000103254	16	745581	797601	139	20	120000	-0.68174	0.7523	FAM173A	76353	25kb
ENSG00000162004	16	747582	801954	152	24	120000	-0.48551	0.68634	CCDC78	76353	25kb
ENSG00000103253	16	751936	810525	188	35	120000	0.22668	0.41034	HAGHL	76353	25kb
ENSG00000103245	16	754753	816329	204	41	120000	0.90448	0.18287	NARFL	76353	25kb
ENSG00000102854	16	785762	843865	250	43	120000	3.0261	0.0012388	MSLN	76353	25kb
ENSG00000162006	16	794428	858370	303	32	120000	2.8601	0.0021177	MSLNL	76353	25kb
ENSG00000007376	16	809974	863397	256	28	120000	3.1273	0.00088218	RPUSD1	76353	25kb
ENSG00000127586	16	813046	875737	327	43	120000	3.1353	0.00085837	CHTF18	76353	25kb
ENSG00000127588	16	823041	875733	289	41	120000	3.0981	0.00097391	GNG13	76353	25kb
ENSG00000167945	16	830443	888861	336	50	120000	2.5079	0.0060734	PRR25	76353	25kb
ENSG00000103227	16	878634	1056318	965	64	120000	-1.343	0.91037	LMF1	76353	25kb
ENSG00000269047	16	1006252	1056663	271	39	120000	-0.12946	0.5515	AC009041.2	76353	25kb
ENSG00000005513	16	1006808	1061979	288	42	120000	-0.19847	0.57866	SOX8	76353	25kb
ENSG00000162009	16	1097756	1156454	318	41	120000	0.18625	0.42612	SSTR5	76353	25kb
ENSG00000184471	16	1115005	1171244	282	32	120000	-0.12707	0.55056	C1QTNF8	76353	25kb
ENSG00000196557	16	1178241	1296771	418	70	120000	1.4659	0.071331	CACNA1H	76353	25kb
ENSG00000116176	16	1246651	1300257	238	38	120000	1.7317	0.04166	TPSG1	76353	25kb
ENSG00000172236	16	1265697	1317555	177	29	120000	0.56897	0.28469	TPSAB1	76353	25kb
ENSG00000095917	16	1281060	1333532	166	26	120000	0.43847	0.33052	TPSD1	76353	25kb
ENSG00000103275	16	1330548	1402019	292	24	120000	0.98369	0.16263	UBE2I	76353	25kb

ENSG00000007516	16	1358602	1424439	260	28	120000	0.55122	0.29074	BAIAP3	76353	25kb
ENSG00000007520	16	1374241	1426912	175	31	120000	-0.14077	0.55598	TSR3	76353	25kb
ENSG00000090581	16	1376924	1438352	184	33	120000	-0.39484	0.65352	GNPTG	76353	25kb
ENSG00000059145	16	1388206	1489752	280	25	120000	-0.38137	0.64854	UNKL	76353	25kb
ENSG00000174109	16	1444745	1504345	213	28	120000	-0.45659	0.67602	C16orf91	76353	25kb
ENSG00000197599	16	1459384	1519557	279	27	120000	-0.62006	0.73239	CCDC154	76353	25kb
ENSG00000103249	16	1469935	1550581	456	44	120000	-0.92157	0.82162	CLCN7	76353	25kb
ENSG00000251692	16	1510887	1563982	295	44	120000	-0.70898	0.76083	PTX4	76353	25kb
ENSG00000100726	16	1518345	1585458	329	49	120000	-0.90771	0.81798	TELO2	76353	25kb
ENSG00000187535	16	1535428	1687111	491	38	120000	-0.34737	0.63584	IFT140	76353	25kb
ENSG00000131634	16	1553689	1630581	265	20	120000	-0.82169	0.79437	TMEM204	76353	25kb
ENSG00000007545	16	1637326	1752909	224	21	120000	0.84008	0.20043	CRAMP1L	76353	25kb
ENSG00000261732	16	1657260	1761716	185	16	120000	1.0512	0.14659	LA16c-431H6.6	76353	25kb
ENSG00000206053	16	1703257	1777281	132	10	120000	1.1717	0.12066	HN1L	76353	25kb
ENSG00000138834	16	1731184	1845318	258	24	120000	-0.24602	0.59717	MAPK8IP3	76353	25kb
ENSG00000103024	16	1795287	1846731	167	20	120000	-1.311	0.90508	NME3	76353	25kb
ENSG00000074071	16	1796891	1848156	179	20	120000	-1.1618	0.87735	MRPS34	76353	25kb
ENSG00000197774	16	1798208	1856709	256	19	120000	-1.1342	0.87164	EME2	76353	25kb
ENSG00000162032	16	1801713	1868701	359	19	120000	-1.4592	0.92774	SPSB3	76353	25kb
ENSG00000095906	16	1807902	1864192	300	18	120000	-1.4809	0.93069	NUBP2	76353	25kb
ENSG00000099769	16	1815414	1869972	343	17	120000	-1.3576	0.91271	IGFALS	76353	25kb
ENSG00000063854	16	1820621	1902195	556	14	120000	-1.6574	0.95128	HAGH	76353	25kb
ENSG00000180185	16	1851968	1915208	487	13	120000	-1.889	0.97055	FAHD1	76353	25kb
ENSG00000162039	16	1858984	1959295	636	11	120000	-1.7245	0.95769	MEIOB	76353	25kb
ENSG00000162040	16	1936464	1993441	226	19	120000	-1.259	0.89598	HS3ST6	76353	25kb
ENSG00000198736	16	1963211	2018327	252	37	120000	1.3935	0.081732	MSRB1	76353	25kb
ENSG00000140986	16	1968975	2032607	298	43	120000	0.92703	0.17696	RPL3L	76353	25kb
ENSG00000140990	16	1984509	2036976	258	31	120000	1.4779	0.069714	NDUFB10	76353	25kb
ENSG00000140988	16	1987053	2039861	267	30	120000	1.616	0.053043	RPS2	76353	25kb
ENSG00000179580	16	1991824	2043976	262	28	120000	1.6096	0.053745	RNF151	76353	25kb
ENSG00000183751	16	1997038	2057934	304	28	120000	1.4365	0.07543	TBL3	76353	25kb
ENSG00000196408	16	2003918	2059193	277	24	120000	1.4127	0.078871	NOXO1	76353	25kb
ENSG00000269205	16	2006633	2057918	269	23	120000	1.3158	0.094115	AC005606.1	76353	25kb
ENSG00000127554	16	2009208	2062750	260	23	120000	1.059	0.14481	GFER	76353	25kb
ENSG00000127561	16	2014661	2069276	237	27	120000	0.55786	0.28847	SYNGR3	76353	25kb
ENSG00000167962	16	2022655	2084824	246	28	120000	0.5287	0.29851	ZNF598	76353	25kb
ENSG00000183971	16	2034927	2095756	197	28	120000	0.55899	0.28808	NPW	76353	25kb
ENSG00000065054	16	2050357	2114027	167	35	120000	0.62233	0.26686	SLC9A3R2	76353	25kb
ENSG00000065057	16	2064816	2122867	138	31	120000	0.83944	0.20061	NTHL1	76353	25kb
ENSG00000103197	16	2072466	2163716	197	44	120000	0.75863	0.22404	TSC2	76353	25kb
ENSG00000008710	16	2113711	2210899	175	30	120000	0.95177	0.17061	PKD1	76353	25kb
ENSG00000167964	16	2165804	2229166	77	17	120000	1.1434	0.12644	RAB26	76353	25kb
ENSG00000131653	16	2180699	2253130	102	22	120000	-0.1017	0.5405	TRAF7	76353	25kb
ENSG00000167971	16	2202184	2271526	124	21	120000	-1.3023	0.9036	CASKIN1	76353	25kb
ENSG00000167965	16	2229249	2284417	110	16	120000	-1.6434	0.94985	MLST8	76353	25kb
ENSG00000182685	16	2234254	2286951	110	16	120000	-1.7141	0.95674	BRICD5	76353	25kb
ENSG00000184207	16	2236998	2289808	112	16	120000	-1.7315	0.95832	PGP	76353	25kb
ENSG00000167967	16	2248567	2310743	138	25	120000	-1.5978	0.94495	E4F1	76353	25kb
ENSG00000167968	16	2260817	2313712	110	22	120000	-1.2745	0.89876	DNASE1L2	76353	25kb
ENSG00000167969	16	2264396	2327301	114	24	120000	-1.0696	0.85761	ECI1	76353	25kb
ENSG00000205937	16	2278117	2343413	110	21	120000	-1.0514	0.85346	RNPS1	76353	25kb
ENSG00000167970	16	2278738	2337343	103	20	120000	-1.0334	0.84929	AC009065.1	76353	25kb
ENSG00000167972	16	2300882	2415747	170	33	120000	-1.4863	0.9314	ABCA3	76353	25kb
ENSG00000162063	16	2454395	2533855	154	9	120000	-0.96235	0.83206	CCNF	76353	25kb
ENSG00000162062	16	2485081	2539964	91	11	120000	-0.73906	0.77006	C16orf59	76353	25kb
ENSG00000162068	16	2496500	2549146	68	18	120000	-0.65184	0.74275	NTN3	76353	25kb
ENSG00000162065	16	2500147	2580735	75	33	120000	-0.54397	0.70677	TBC1D24	76353	25kb
ENSG00000260272	16	2521033	2595219	56	29	120000	-0.37111	0.64472	RP11-20I23.1	76353	25kb
ENSG00000185883	16	2538871	2595219	40	22	120000	-0.61961	0.73224	ATP6V0C	76353	25kb
ENSG00000259784	16	2538966	2602956	40	22	120000	-0.61961	0.73224	ATP6C	76353	25kb
ENSG00000162066	16	2545358	2606423	35	20	120000	-0.36474	0.64235	AMDHD2	76353	25kb
ENSG00000205923	16	2552081	2606420	31	18	120000	-0.27936	0.61001	CEMP1	76353	25kb
ENSG00000140992	16	2562965	2678189	41	20	120000	-0.15318	0.56087	PDPK1	76353	25kb
ENSG00000167977	16	2707476	2784031	134	19	120000	0.78162	0.21722	KCTD5	76353	25kb
ENSG00000172382	16	2737419	2795552	73	19	120000	-0.81523	0.79253	PRSS27	76353	25kb
ENSG00000167978	16	2777330	2847539	106	18	120000	0.26602	0.39511	SRRM2	76353	25kb
ENSG00000103363	16	2796415	2852298	86	14	120000	0.31293	0.37717	TCEB2	76353	25kb
ENSG00000103355	16	2808954	2862949	78	15	120000	0.63829	0.26164	PRSS33	76353	25kb
ENSG00000007038	16	2842164	2901305	98	19	120000	-0.045132	0.518	PRSS21	76353	25kb
ENSG00000162078	16	2855170	2913967	125	21	120000	-0.25921	0.60226	ZG16B	76353	25kb
ENSG00000005001	16	2877728	2933171	166	28	120000	-1.1745	0.87989	PRSS22	76353	25kb
ENSG00000162076	16	2908187	2974383	286	23	120000	-1.3771	0.91576	FLYWCH2	76353	25kb
ENSG00000059122	16	2936938	3026209	431	40	120000	-1.2284	0.89035	FLYWCH1	76353	25kb
ENSG00000131650	16	2988945	3043384	209	25	120000	-0.6723	0.7493	KREMEN2	76353	25kb
ENSG00000127564	16	2993025	3055540	218	28	120000	-0.6049	0.72738	PKMYT1	76353	25kb
ENSG00000162073	16	2994246	3048490	174	24	120000	-0.56772	0.71489	PAQR4	76353	25kb
ENSG00000213937	16	3037457	3089506	130	24	120000	0.67724	0.24913	CLDN9	76353	25kb
ENSG00000184697	16	3039713	3095072	142	25	120000	0.46099	0.3224	CLDN6	76353	25kb
ENSG00000006327	16	3043446	3097384	143	25	120000	0.397	0.34569	TNFRSF12A	76353	25kb
ENSG00000103145	16	3047621	3099287	142	24	120000	0.3077	0.37915	HCFC1R1	76353	25kb
ENSG00000131652	16	3049028	3102756	150	24	120000	0.2496	0.40145	THOC6	76353	25kb
ENSG00000162069	16	3052683	3111927	167	27	120000	0.22622	0.41051	CCDC64B	76353	25kb
ENSG00000008516	16	3071682	3135727	209	35	120000	-0.090243	0.53595	MMP25	76353	25kb
ENSG00000008517	16	3090298	3156908	222	40	120000	-0.23204	0.59175	IL32	76353	25kb
ENSG00000130182	16	3113891	3174318	173	26	120000	0.28147	0.38918	ZSCAN10	76353	25kb
ENSG00000122386	16	3137561	3195518	119	14	120000	0.41579	0.33878	ZNF205	76353	25kb
ENSG00000085644	16	3154778	3217806	134	11	120000	0.096532	0.46155	ZNF213	76353	25kb
ENSG00000228146	16	3169220	3224964	114	11	120000	-0.24718	0.59761	CASP16	76353	25kb
ENSG00000168124	16	3229247	3280188	166	22	120000	-0.43715	0.669	OR1F1	76353	25kb
ENSG00000010539	16	3247325	3311221	185	22	120000	0.45626	0.3241	ZNF200	76353	25kb
ENSG00000103313	16	3267028	3331627	151	22	120000	0.52571	0.29954	MEFV	76353	25kb
ENSG00000006194	16	3288743	3376401	174	25	120000	-0.14818	0.5589	ZNF263	76353	25kb
ENSG00000140993	16	3323832	3380729	94	16	120000	-0.61531	0.73082	TIGD7	76353	25kb
ENSG00000162086	16	3330406	3393852	134	27	120000	-0.7264	0.7662	ZNF75A	76353	25kb
ENSG00000168158	16	3380889	3431924	191	18	120000	-0.48101	0.68474	OR2C1	76353	25kb
ENSG00000262621	16	3390099	3561960	597	32	120000	-0.93979	0.82634	NAA60	76353	25kb

ENSG00000232196	16	3396053	3447283	168	14	120000	-0.55639	0.71103	MTRNR2L4	76353	25kb
ENSG00000140987	16	3407085	3476065	194	16	120000	-0.47402	0.68226	ZSCAN32	76353	25kb
ENSG00000103343	16	3426235	3484370	132	14	120000	-0.48524	0.68625	ZNF174	76353	25kb
ENSG00000167981	16	3461104	3518542	202	22	120000	-0.52296	0.6995	ZNF597	76353	25kb
ENSG00000122390	16	3468611	3561963	355	26	120000	-0.96417	0.83252	NAA60	76353	25kb
ENSG00000215131	16	3518484	3570480	186	22	120000	-0.95224	0.82951	C16orf90	76353	25kb
ENSG00000103351	16	3525924	3614048	238	32	120000	-1.1582	0.87661	CLUAP1	76353	25kb
ENSG00000188827	16	3606182	3686599	215	10	120000	-0.43383	0.66779	SLX4	76353	25kb
ENSG00000213918	16	3636729	3755144	324	26	120000	-0.63788	0.73822	DNASE1	76353	25kb
ENSG00000126602	16	3676640	3792598	289	38	120000	-0.68073	0.75198	TRAP1	76353	25kb
ENSG00000005339	16	3750055	3955727	244	56	120000	0.41632	0.33859	CREBBP	76353	25kb
ENSG00000162104	16	3978388	4191186	878	91	120000	1.9239	0.027181	ADCY9	76353	25kb
ENSG00000185739	16	4214375	4317081	360	50	120000	2.4603	0.0069407	SRL	76353	25kb
ENSG00000090447	16	4282187	4348076	121	15	120000	3.1426	0.00083727	TFAP4	76353	25kb
ENSG00000126603	16	4339762	4414598	175	38	120000	1.3408	0.089991	GLIS2	76353	25kb
ENSG00000217930	16	4356550	4430608	179	30	120000	-0.1499	0.55958	PAM16	76353	25kb
ENSG00000103426	16	4365252	4495495	317	29	120000	-0.64835	0.74162	CORO7-PAM16	76353	25kb
ENSG00000262246	16	4379543	4500706	298	27	120000	-0.6127	0.72996	CORO7	76353	25kb
ENSG00000168140	16	4396849	4458529	154	22	120000	-0.46298	0.67831	VASN	76353	25kb
ENSG00000103423	16	4450806	4531776	229	20	120000	-0.58911	0.72211	DNAJA3	76353	25kb
ENSG00000153406	16	4486681	4570764	225	15	120000	-0.49509	0.68973	NMRAL1	76353	25kb
ENSG00000103415	16	4499691	4585348	232	14	120000	-0.45464	0.67531	HMOX2	76353	25kb
ENSG00000089486	16	4535676	4613829	191	15	120000	-0.70001	0.75804	CDIP1	76353	25kb
ENSG00000205832	16	4581491	4675715	241	24	120000	-0.4417	0.67065	C16orf96	76353	25kb
ENSG00000153443	16	4633884	4690028	175	16	120000	-0.55797	0.71157	UBALD1	76353	25kb
ENSG00000102858	16	4641494	4765975	450	33	120000	-0.50719	0.69399	MGRN1	76353	25kb
ENSG00000168101	16	4718695	4770860	197	17	120000	-0.16862	0.56695	NUDT16L1	76353	25kb
ENSG00000168096	16	4721513	4809379	312	17	120000	-0.1081	0.54304	ANKS3	76353	25kb
ENSG00000166246	16	4759273	4824397	249	24	120000	-0.026443	0.51055	C16orf71	76353	25kb
ENSG00000103199	16	4773240	4842625	245	28	120000	-0.30134	0.61842	ZNF500	76353	25kb
ENSG00000140623	16	4802670	4863522	199	32	120000	0.2957	0.38373	SEPT12	76353	25kb
ENSG00000267795	16	4813398	4871492	178	27	120000	1.0424	0.14861	SMIM22	76353	25kb
ENSG00000067836	16	4821969	4877951	153	22	120000	1.44	0.074938	ROGDI	76353	25kb
ENSG00000140632	16	4828204	4922343	304	26	120000	2.1822	0.014547	GLYR1	76353	25kb
ENSG00000118900	16	4871666	4957361	319	26	120000	2.2238	0.013081	UBN1	76353	25kb
ENSG00000118898	16	4907508	5035742	595	62	120000	1.6046	0.054289	PPL	76353	25kb
ENSG00000103184	16	4983318	5094159	492	47	120000	-0.7451	0.77189	SEC14L5	76353	25kb
ENSG00000103174	16	5049845	5109142	187	31	120000	-1.3841	0.91683	NAGPA	76353	25kb
ENSG00000033011	16	5058703	5162380	395	68	120000	0.1088	0.45668	ALG1	76353	25kb
ENSG00000153446	16	5069123	5141111	247	48	120000	-1.21	0.88687	C16orf89	76353	25kb
ENSG00000118894	16	5109305	5172809	288	46	120000	0.7754	0.21905	FAM86A	76353	25kb
ENSG00000078328	16	6044095	7788340	10810	500	120000	2.1444	0.015999	RBFOX1	76353	25kb
ENSG00000232258	16	8594502	8647304	275	25	120000	-0.42206	0.66351	TMEM114	76353	25kb
ENSG00000067365	16	8690540	8765081	307	25	120000	1.8141	0.034829	METTL22	76353	25kb
ENSG00000183044	16	8743422	8903432	852	97	120000	-1.1687	0.87874	ABAT	76353	25kb
ENSG00000184857	16	8849241	8916505	349	44	120000	-0.41005	0.65912	TMEM186	76353	25kb
ENSG00000140650	16	8857680	8968188	564	37	120000	-0.31269	0.62274	PMM2	76353	25kb
ENSG00000153048	16	8921799	8987866	309	42	120000	-0.10542	0.54198	CARHSP1	76353	25kb
ENSG00000187555	16	8960951	9083371	372	32	120000	0.31196	0.37754	USP7	76353	25kb
ENSG00000182831	16	9160505	9240497	330	26	120000	-0.38979	0.65166	C16orf72	76353	25kb
ENSG00000260362	16	9735742	9795700	321	37	120000	1.0999	0.13569	RP11-297M9.1	76353	25kb
ENSG00000183454	16	9827376	10301611	2022	58	120000	-1.1882	0.88263	GRIN2A	76353	25kb
ENSG00000166669	16	10395291	10602495	740	57	120000	0.31089	0.37794	ATF7IP2	76353	25kb
ENSG00000213853	16	10597279	10699555	435	74	120000	0.21741	0.41395	EMP2	76353	25kb
ENSG00000153060	16	10696358	10813802	636	48	120000	1.2399	0.1075	TEKT5	76353	25kb
ENSG00000103274	16	10812643	10888208	232	29	120000	1.0068	0.15702	NUBP1	76353	25kb
ENSG00000166676	16	10829776	10937651	287	44	120000	-0.21144	0.58373	TVP23A	76353	25kb
ENSG00000179583	16	10946055	11051079	341	58	120000	-1.98	0.97615	CIITA	76353	25kb
ENSG00000182108	16	10997748	11061317	202	42	120000	-1.9367	0.97361	DEXI	76353	25kb
ENSG00000038532	16	11013345	11301046	824	37	120000	-1.5555	0.94009	CLEC16A	76353	25kb
ENSG00000175643	16	11318476	11470619	667	33	120000	-0.69077	0.75515	RMI2	76353	25kb
ENSG00000185338	16	11323262	11375036	200	29	120000	-0.82036	0.79399	SOCS1	76353	25kb
ENSG00000178279	16	11336605	11388390	208	27	120000	-0.56506	0.71398	TNP2	76353	25kb
ENSG00000178257	16	11342144	11392452	209	25	120000	-0.55194	0.70951	PRM3	76353	25kb
ENSG00000122304	16	11344496	11395337	212	25	120000	-0.49274	0.6889	PRM2	76353	25kb
ENSG00000175646	16	11349693	11400207	216	25	120000	-0.51756	0.69762	PRM1	76353	25kb
ENSG00000188897	16	11428702	11642444	1229	91	120000	-1.7373	0.95883	CTD-3088G3.8	76353	25kb
ENSG00000189067	16	11616853	11755237	591	58	120000	-0.27284	0.60751	LITAF	76353	25kb
ENSG00000184602	16	11737270	11798015	190	27	120000	0.56016	0.28769	SNN	76353	25kb
ENSG00000153066	16	11747936	11861734	321	28	120000	0.027694	0.48895	TXNDC11	76353	25kb
ENSG00000122299	16	11819442	11916123	289	27	120000	-1.0754	0.8589	ZC3H7A	76353	25kb
ENSG00000171490	16	11904056	11970442	192	26	120000	-0.92655	0.82292	RSL1D1	76353	25kb
ENSG00000103342	16	11936985	12034939	198	21	120000	-0.65221	0.74287	GSPT1	76353	25kb
ENSG00000234719	16	11996230	12095500	281	29	120000	1.9836	0.023652	RP11-166B2.1	76353	25kb
ENSG00000048462	16	12033964	12086925	197	25	120000	2.3834	0.0085773	TNFRSF17	76353	25kb
ENSG00000269121	16	12037000	12087333	198	25	120000	2.3718	0.0088503	AC007216.2	76353	25kb
ENSG00000048471	16	12045594	12693146	2997	135	120000	1.779	0.03762	SNX29	76353	25kb
ENSG00000175604	16	12155603	12209159	193	25	120000	0.072086	0.47127	RP11-276H1.3	76353	25kb
ENSG00000103381	16	12731919	12922874	755	68	120000	0.73222	0.23202	CPPED1	76353	25kb
ENSG00000237515	16	12970477	13359272	1603	123	120000	-0.31848	0.62494	SHISA9	76353	25kb
ENSG00000175595	16	13989014	14071202	195	16	120000	-0.5337	0.70323	ERCC4	76353	25kb
ENSG00000186260	16	14140178	14385630	592	19	120000	0.86371	0.19387	MKL2	76353	25kb
ENSG00000140694	16	14504558	14751585	354	24	120000	0.21662	0.41425	PARN	76353	25kb
ENSG00000269049	16	14539842	14590054	62	17	120000	0.49311	0.31097	AC092291.2	76353	25kb
ENSG00000103429	16	14701672	14788093	93	19	120000	0.26073	0.39715	BFAR	76353	25kb
ENSG00000069764	16	14741405	14813526	40	11	120000	0.098563	0.46074	PLA2G10	76353	25kb
ENSG00000224712	16	14777801	14845195	2	1	120000	0.066121	0.47364	NPIPA3	76353	25kb
ENSG00000254852	16	14816923	14884270	2	1	120000	-1.594	0.94453	NPIPA2	76353	25kb
ENSG00000103512	16	14902538	15015017	12	7	120000	-0.49884	0.69105	NOMO1	76353	25kb
ENSG00000183426	16	14991659	15070915	9	5	120000	0.067976	0.4729	NPIPA1	76353	25kb
ENSG00000179889	16	15043448	15258196	208	33	120000	-0.35071	0.6371	PDXDC1	76353	25kb
ENSG00000157045	16	15106710	15174921	132	21	120000	-0.36745	0.64336	NTAN1	76353	25kb
ENSG00000085721	16	15128879	15213174	148	24	120000	-0.26661	0.60511	RRN3	76353	25kb
ENSG00000183793	16	15432516	15499904	50	14	120000	-0.12514	0.5498	NPIPA5	76353	25kb
ENSG00000156968	16	15464611	15532125	138	24	120000	0.47398	0.31776	MPV17L	76353	25kb
ENSG00000261130	16	15464611	15634205	304	34	120000	0.54882	0.29156	RP11-1021N1.1	76353	25kb

ENSG00000166780	16	15503152	15743885	423	53	120000	1.421	0.077664	C16orf45	76353	25kb
ENSG00000166783	16	15663243	15762023	172	33	120000	1.9938	0.023084	KIAA0430	76353	25kb
ENSG00000072864	16	15712124	15845210	325	32	120000	2.3408	0.0096212	NDE1	76353	25kb
ENSG00000133392	16	15772029	15975890	808	70	120000	1.7349	0.041379	MYH11	76353	25kb
ENSG00000133393	16	15934577	16007482	338	12	120000	1.438	0.075222	FOPNL	76353	25kb
ENSG00000103222	16	16018434	16261931	841	78	120000	0.36588	0.35723	ABCC1	76353	25kb
ENSG00000091262	16	16217785	16342379	328	37	120000	0.54691	0.29222	ABCC6	76353	25kb
ENSG00000103226	16	16301352	16413668	31	5	120000	-0.6481	0.74154	NOMO3	76353	25kb
ENSG00000103489	16	17170626	17589738	1274	121	120000	0.9047	0.18281	XYLT1	76353	25kb
ENSG00000185164	16	18486182	18598533	13	7	120000	0.40959	0.34105	NOMO2	76353	25kb
ENSG00000134419	16	18767617	18826705	104	12	120000	1.9008	0.028665	RPS15A	76353	25kb
ENSG00000260342	16	18774385	18837841	113	13	120000	1.9052	0.028378	RP11-1035H13.3	76353	25kb
ENSG00000170540	16	18777991	18838000	112	13	120000	1.9163	0.027662	ARL6IP1	76353	25kb
ENSG00000157106	16	18791175	18962776	355	17	120000	2.5009	0.0061947	SMG1	76353	25kb
ENSG00000170537	16	18970256	19100264	330	21	120000	1.6798	0.046496	TMC7	76353	25kb
ENSG00000167186	16	19053921	19116417	155	22	120000	1.291	0.098343	COQ7	76353	25kb
ENSG00000205730	16	19100254	19157946	138	26	120000	1.5264	0.063457	ITPRIPL2	76353	25kb
ENSG00000261427	16	19100324	19209900	258	52	120000	1.5072	0.065882	CTD-2349B8.1	76353	25kb
ENSG00000103528	16	19154293	19304652	395	70	120000	1.0194	0.154	SYT17	76353	25kb
ENSG00000261210	16	19272061	19358467	314	31	120000	-0.88166	0.81102	CLEC19A	76353	25kb
ENSG00000103534	16	19396818	19535435	550	31	120000	-1.2816	0.90001	TMC5	76353	25kb
ENSG00000006007	16	19488015	19558467	247	20	120000	-1.31	0.9049	GDE1	76353	25kb
ENSG00000103540	16	19510133	19589730	260	14	120000	-1.174	0.87981	CCP110	76353	25kb
ENSG00000103544	16	19541562	19743115	584	39	120000	-1.3502	0.91153	C16orf62	76353	25kb
ENSG00000103550	16	19689902	19754557	136	15	120000	0.29945	0.3823	KNOP1	76353	25kb
ENSG00000174628	16	19702778	19893907	381	23	120000	0.013013	0.49481	IQCK	76353	25kb
ENSG00000167191	16	19843616	19922489	178	23	120000	-0.61574	0.73097	GPRC5B	76353	25kb
ENSG00000180269	16	20017807	20110239	286	28	120000	-1.3649	0.91386	GPR139	76353	25kb
ENSG00000169347	16	20295894	20364130	222	38	120000	-0.93937	0.82623	GP2	76353	25kb
ENSG00000169344	16	20319374	20392623	226	40	120000	0.02517	0.48996	UMOD	76353	25kb
ENSG00000169340	16	20345492	20441059	355	39	120000	-0.094052	0.53747	PDILT	76353	25kb
ENSG00000183549	16	20395856	20477658	290	23	120000	-0.44202	0.67076	ACSM5	76353	25kb
ENSG00000183747	16	20437783	20523991	218	17	120000	-1.0206	0.84627	ACSM2A	76353	25kb
ENSG00000267824	16	20474024	20525981	125	14	120000	-1.5875	0.9438	AC137056.1	76353	25kb
ENSG00000066813	16	20522548	20612749	223	25	120000	-1.25	0.89435	ACSM2B	76353	25kb
ENSG00000005187	16	20596565	20833903	575	22	120000	-0.94752	0.82831	ACSM3	76353	25kb
ENSG00000166743	16	20609559	20735212	337	20	120000	-0.72844	0.76683	ACSM1	76353	25kb
ENSG00000066654	16	20719986	20778406	128	12	120000	-0.97868	0.83613	THUMPD1	76353	25kb
ENSG00000196678	16	20766515	20936671	253	16	120000	-0.97709	0.83574	ERI2	76353	25kb
ENSG00000005189	16	20792751	20885987	122	17	120000	-0.90727	0.81787	AC004381.6	76353	25kb
ENSG00000188215	16	20841247	20936706	140	17	120000	-0.97577	0.83541	DCUN1D3	76353	25kb
ENSG00000102897	16	20886190	20961328	138	21	120000	-0.57075	0.71591	LYRM1	76353	25kb
ENSG00000158486	16	20919433	21195762	638	43	120000	-0.9021	0.8165	DNAH3	76353	25kb
ENSG00000011638	16	21144698	21216937	197	20	120000	-0.93186	0.82429	TMEM159	76353	25kb
ENSG00000103310	16	21183773	21250831	173	36	120000	0.28107	0.38933	ZP2	76353	25kb
ENSG00000175311	16	21219986	21288750	185	32	120000	0.60063	0.27404	ANKS4B	76353	25kb
ENSG00000103316	16	21225195	21339404	248	40	120000	0.69924	0.2422	CRYM	76353	25kb
ENSG00000169246	16	21388558	21484888	4	2	120000	0.56252	0.28688	NPIPB3	76353	25kb
ENSG00000197006	16	21583539	21693794	135	27	120000	0.93315	0.17537	METTL9	76353	25kb
ENSG00000140749	16	21627609	21688981	90	20	120000	0.8934	0.18582	IGSF6	76353	25kb
ENSG00000155719	16	21664835	21797050	150	27	120000	1.4204	0.077752	OTOA	76353	25kb
ENSG00000185864	16	21820890	21917148	26	10	120000	2.3246	0.010048	NPIPB4	76353	25kb
ENSG00000140740	16	21938981	22019981	77	17	120000	0.10445	0.45841	UQCRC2	76353	25kb
ENSG00000155714	16	21970186	22037433	70	17	120000	0.27218	0.39274	PDZD9	76353	25kb
ENSG00000185716	16	21993959	22123855	121	20	120000	0.36232	0.35856	C16orf52	76353	25kb
ENSG00000175267	16	22078859	22193287	143	28	120000	-0.079311	0.53161	VWA3A	76353	25kb
ENSG00000183921	16	22152618	22228075	114	17	120000	-0.81205	0.79162	SDR42E2	76353	25kb
ENSG00000103319	16	22192603	22323554	234	31	120000	-1.1363	0.87207	EEF2K	76353	25kb
ENSG00000058600	16	22283730	22371424	102	18	120000	0.44767	0.32719	POLR3E	76353	25kb
ENSG00000140743	16	22332257	22473486	89	23	120000	0.2396	0.40532	CDR2	76353	25kb
ENSG00000243716	16	22465442	22572842	2	1	120000	0.45416	0.32486	NPIPB5	76353	25kb
ENSG00000122254	16	22800498	22952659	493	81	120000	-1.1438	0.87365	HS3ST2	76353	25kb
ENSG00000103404	16	23047727	23185591	323	13	120000	-0.5911	0.72277	USP31	76353	25kb
ENSG00000166828	16	23169036	23253204	274	17	120000	-1.5737	0.94222	SCNN1G	76353	25kb
ENSG00000168447	16	23264552	23417620	406	63	120000	1.1159	0.13223	SCNN1B	76353	25kb
ENSG00000168434	16	23374814	23489501	214	28	120000	1.9141	0.027802	COG7	76353	25kb
ENSG00000103365	16	23449863	23558316	229	18	120000	1.7583	0.039352	GGA2	76353	25kb
ENSG00000103356	16	23508335	23594052	225	18	120000	1.5097	0.065558	EARS2	76353	25kb
ENSG00000103353	16	23543392	23610710	198	19	120000	1.0419	0.14873	UBFD1	76353	25kb
ENSG00000004779	16	23567323	23632677	202	23	120000	0.4454	0.32801	NDUFAB1	76353	25kb
ENSG00000083093	16	23589488	23677631	260	19	120000	-0.24681	0.59747	PALB2	76353	25kb
ENSG00000166847	16	23627713	23706195	218	19	120000	-0.73012	0.76734	DCTN5	76353	25kb
ENSG00000166851	16	23663977	23726688	163	20	120000	-1.0379	0.85035	PLK1	76353	25kb
ENSG00000134398	16	23676647	23749821	199	35	120000	-1.3176	0.90618	ERN2	76353	25kb
ENSG00000166869	16	23740948	23795272	161	24	120000	-0.85587	0.80397	CHP2	76353	25kb
ENSG00000166501	16	23822322	24256932	1355	114	120000	-1.7188	0.95718	PRKCB	76353	25kb
ENSG00000006116	16	24241874	24399122	516	77	120000	-0.10718	0.54268	CACNG3	76353	25kb
ENSG00000122257	16	24524014	24609184	274	21	120000	0.69357	0.24397	RBBP6	76353	25kb
ENSG00000090905	16	24716016	24863953	328	27	120000	0.9801	0.16352	TNRC6A	76353	25kb
ENSG00000158865	16	24832162	24947949	292	39	120000	0.50498	0.30679	SLC5A11	76353	25kb
ENSG00000140750	16	24905706	25051987	305	22	120000	0.46785	0.31994	ARHGAP17	76353	25kb
ENSG00000205629	16	25098050	25214552	293	23	120000	-0.88311	0.81141	LCMT1	76353	25kb
ENSG00000103375	16	25202052	25265261	190	30	120000	-0.12159	0.54839	AQP8	76353	25kb
ENSG00000155592	16	25222322	25294252	263	20	120000	-0.42427	0.66431	ZKSCAN2	76353	25kb
ENSG00000182601	16	25678347	26174009	1740	169	120000	-0.64808	0.74153	HS3ST4	76353	25kb
ENSG00000155666	16	27189807	27258089	166	27	120000	-0.8403	0.79963	KDM8	76353	25kb
ENSG00000169189	16	27211312	27305115	194	22	120000	-0.92199	0.82173	NSMCE1	76353	25kb
ENSG00000245888	16	27254526	27326813	148	19	120000	-0.93592	0.82534	CTD-3203P2.2	76353	25kb
ENSG00000077238	16	27299989	27401099	312	36	120000	0.15335	0.43906	IL4R	76353	25kb
ENSG00000103522	16	27388483	27487115	295	42	120000	-1.0353	0.84974	IL21R	76353	25kb
ENSG00000077235	16	27445876	27586234	176	17	120000	-0.62596	0.73433	GTF3C1	76353	25kb
ENSG00000047578	16	27536454	27816690	408	32	120000	-1.068	0.85724	KIAA0556	76353	25kb
ENSG00000169181	16	27773850	28099830	999	111	120000	0.37496	0.35384	GSG1L	76353	25kb
ENSG00000169180	16	28084300	28248241	221	14	120000	-0.81474	0.79239	XPO6	76353	25kb
ENSG00000188322	16	28278840	28360170	130	31	120000	-1.1642	0.87784	SBK1	76353	25kb
ENSG00000198156	16	28328876	28399829	37	12	120000	-0.45197	0.67435	NPIPB6	76353	25kb

ENSG00000205609	16	28365900	28440200	3	1	120000	0.44398	0.32853	EIF3CL	76353	25kb
ENSG00000233232	16	28442693	28506868	28	5	120000	0.54103	0.29424	NPIPB7	76353	25kb
ENSG00000261832	16	28452974	28528333	74	10	120000	0.11663	0.45358	CLN3	76353	25kb
ENSG00000188603	16	28452983	28531896	84	11	120000	0.083193	0.46685	CLN3	76353	25kb
ENSG00000184730	16	28480970	28535291	92	11	120000	0.12061	0.452	APOBR	76353	25kb
ENSG00000197272	16	28485683	28548372	121	14	120000	0.016359	0.49347	IL27	76353	25kb
ENSG00000176046	16	28523606	28575495	143	16	120000	-0.37303	0.64544	NUPR1	76353	25kb
ENSG00000176476	16	28540236	28628111	274	18	120000	-0.24357	0.59622	CCDC101	76353	25kb
ENSG00000197165	16	28578264	28633430	179	17	120000	0.057227	0.47718	SULT1A2	76353	25kb
ENSG00000196502	16	28591903	28659946	157	18	120000	-0.10813	0.54305	SULT1A1	76353	25kb
ENSG00000255524	16	28623975	28695003	43	10	120000	-0.51049	0.69515	NPIPB8	76353	25kb
ENSG00000184110	16	28674879	28772051	4	2	120000	-0.19735	0.57822	EIF3C	76353	25kb
ENSG00000196993	16	28738108	28809128	4	2	120000	-0.19735	0.57822	NPIPB9	76353	25kb
ENSG00000168488	16	28809356	28873558	96	5	120000	0.17024	0.43241	ATXN2L	76353	25kb
ENSG00000178952	16	28828732	28882729	91	5	120000	0.18368	0.42713	TUFM	76353	25kb
ENSG00000178188	16	28832921	28910526	115	6	120000	0.25461	0.39951	SH2B1	76353	25kb
ENSG00000196296	16	28864726	28940830	96	9	120000	0.45986	0.32281	ATP2A1	76353	25kb
ENSG00000177548	16	28890742	28972847	99	18	120000	0.83792	0.20104	RABEP2	76353	25kb
ENSG00000177455	16	28918260	28975667	76	16	120000	0.85961	0.195	CD19	76353	25kb
ENSG00000176953	16	28937128	29003418	110	18	120000	0.95384	0.17008	NFATC2IP	76353	25kb
ENSG00000169682	16	28960542	29020869	113	22	120000	1.5896	0.055965	SPNS1	76353	25kb
ENSG00000213658	16	28971147	29027104	104	22	120000	2.0947	0.0181	LAT	76353	25kb
ENSG00000196796	16	29024923	29089047	26	11	120000	2.4413	0.0073177	CTB-134H23.2	76353	25kb
ENSG00000254206	16	29367675	29440350	21	10	120000	1.3956	0.081416	NPIPB11	76353	25kb
ENSG00000261740	16	29429551	29490972	12	7	120000	1.6887	0.045643	RP11-345J4.5	76353	25kb
ENSG00000183336	16	29439914	29491285	2	1	120000	1.4166	0.078301	BOLA2	76353	25kb
ENSG00000181625	16	29440822	29494540	2	1	120000	1.4166	0.078301	SLX1B	76353	25kb
ENSG00000213648	16	29442127	29501300	1	1	120000	1.3195	0.093493	SULT1A4	76353	25kb
ENSG00000197471	16	29649300	29707187	96	15	120000	0.73781	0.23032	SPN	76353	25kb
ENSG00000103485	16	29649600	29735020	132	19	120000	0.63285	0.26342	QPRT	76353	25kb
ENSG00000185905	16	29728784	29782327	99	9	120000	-0.48703	0.68688	C16orf54	76353	25kb
ENSG00000174992	16	29764561	29818096	64	8	120000	-0.64953	0.742	ZG16	76353	25kb
ENSG00000079616	16	29777040	29841706	74	14	120000	-0.18126	0.57192	KIF22	76353	25kb
ENSG00000103495	16	29792427	29848649	52	9	120000	0.28052	0.38954	MAZ	76353	25kb
ENSG00000167371	16	29798177	29852201	50	7	120000	0.37614	0.3534	PRRT2	76353	25kb
ENSG00000185928	16	29802285	29866948	64	7	120000	0.60671	0.27202	PAGR1	76353	25kb
ENSG00000263136	16	29802832	29856438	50	6	120000	0.47435	0.31763	PAGR1	76353	25kb
ENSG00000013364	16	29806715	29884355	77	9	120000	1.2904	0.098454	MVP	76353	25kb
ENSG00000103502	16	29844678	29900057	49	8	120000	1.867	0.030948	CDIPT	76353	25kb
ENSG00000174938	16	29857480	29935868	88	17	120000	1.9634	0.024798	SEZ6L2	76353	25kb
ENSG00000174939	16	29886696	29956185	81	13	120000	1.4807	0.069348	ASPHD1	76353	25kb
ENSG00000174943	16	29891333	29963356	87	13	120000	1.7246	0.042302	KCTD13	76353	25kb
ENSG00000149932	16	29927206	30009373	113	10	120000	2.1957	0.014057	TMEM219	76353	25kb
ENSG00000149930	16	29959962	30028582	105	10	120000	2.2398	0.012552	TAOK2	76353	25kb
ENSG00000149929	16	29978645	30032757	87	10	120000	2.0953	0.018072	HIRIP3	76353	25kb
ENSG00000169592	16	29981615	30042114	99	10	120000	1.8999	0.02872	INO80E	76353	25kb
ENSG00000149927	16	29991830	30059591	108	11	120000	1.6771	0.046763	DOC2A	76353	25kb
ENSG00000167194	16	30009655	30064057	97	10	120000	1.4504	0.073478	C16orf92	76353	25kb
ENSG00000149926	16	30010748	30089299	110	11	120000	1.4602	0.072124	FAM57B	76353	25kb
ENSG00000149925	16	30039411	30106778	75	10	120000	1.0498	0.14691	ALDOA	76353	25kb
ENSG00000149923	16	30062299	30121698	65	12	120000	0.65374	0.25664	PPP4C	76353	25kb
ENSG00000149922	16	30072114	30128208	63	13	120000	0.64655	0.25896	TBX6	76353	25kb
ENSG00000090238	16	30078635	30133236	65	13	120000	0.69881	0.24234	YPEL3	76353	25kb
ENSG00000102886	16	30091131	30150177	75	16	120000	-0.053526	0.52134	GDPD3	76353	25kb
ENSG00000102882	16	30100426	30159827	78	17	120000	-0.49885	0.69106	MAPK3	76353	25kb
ENSG00000102879	16	30169148	30225397	39	8	120000	-1.161	0.87719	CORO1A	76353	25kb
ENSG00000169627	16	30179255	30230627	21	7	120000	-1.1306	0.87089	BOLA2B	76353	25kb
ENSG00000132207	16	30180208	30233882	21	7	120000	-1.1306	0.87089	SLX1A	76353	25kb
ENSG00000261052	16	30180754	30240631	21	7	120000	-1.1306	0.87089	SULT1A3	76353	25kb
ENSG00000258130	16	30190637	30245392	11	5	120000	-1.1093	0.86635	RP11-347C12.3	76353	25kb
ENSG00000198064	16	30209258	30290831	1	1	120000	0.49877	0.30897	RP11-347C12.1	76353	25kb
ENSG00000169217	16	30337087	30391682	53	12	120000	0.63622	0.26232	CD2BP2	76353	25kb
ENSG00000169221	16	30343423	30406585	80	15	120000	0.55267	0.29024	TBC1D10B	76353	25kb
ENSG00000180209	16	30357255	30414312	78	13	120000	0.42622	0.33497	MYLPF	76353	25kb
ENSG00000180035	16	30364427	30436429	88	15	120000	0.39054	0.34807	ZNF48	76353	25kb
ENSG00000180096	16	30364454	30432312	81	14	120000	0.35933	0.35968	SEPT1	76353	25kb
ENSG00000270466	16	30364755	30418863	70	12	120000	0.42502	0.33541	SEPT1	76353	25kb
ENSG00000179965	16	30393618	30465920	92	22	120000	0.23077	0.40875	ZNF771	76353	25kb
ENSG00000179958	16	30409940	30466396	59	19	120000	0.30051	0.3819	DCTPP1	76353	25kb
ENSG00000179918	16	30429952	30482502	74	21	120000	0.41523	0.33899	SEPHS2	76353	25kb
ENSG00000005844	16	30458979	30559506	155	32	120000	-0.29653	0.61659	ITGAL	76353	25kb
ENSG00000169957	16	30510325	30563142	70	21	120000	-0.75155	0.77384	ZNF768	76353	25kb
ENSG00000261459	16	30512244	30571173	75	22	120000	-0.91866	0.82086	ZNF747	76353	25kb
ENSG00000169955	16	30512244	30571668	75	22	120000	-0.91866	0.82086	ZNF747	76353	25kb
ENSG00000260869	16	30520548	30594600	66	20	120000	-1.2689	0.89777	AC002310.13	76353	25kb
ENSG00000169951	16	30540085	30594819	49	16	120000	-1.4154	0.92152	ZNF764	76353	25kb
ENSG00000229809	16	30555667	30609055	46	14	120000	-1.8628	0.96875	ZNF688	76353	25kb
ENSG00000197162	16	30560061	30622092	40	14	120000	-1.9522	0.97454	ZNF785	76353	25kb
ENSG00000156853	16	30588879	30660333	61	9	120000	-0.45436	0.67522	ZNF689	76353	25kb
ENSG00000156858	16	30637038	30692761	43	7	120000	0.19673	0.42202	PRR14	76353	25kb
ENSG00000156860	16	30644752	30707135	48	10	120000	-0.1916	0.57597	FBRS	76353	25kb
ENSG00000080603	16	30684530	30780602	64	15	120000	-0.48237	0.68523	SRCAP	76353	25kb
ENSG00000156873	16	30734591	30797490	41	13	120000	-0.40269	0.65641	PHKG2	76353	25kb
ENSG00000196118	16	30743744	30799031	34	11	120000	-0.29272	0.61513	C16orf93	76353	25kb
ENSG00000103549	16	30748066	30812628	40	10	120000	-0.10067	0.54009	RNF40	76353	25kb
ENSG00000102870	16	30764778	30823523	45	10	120000	0.099063	0.46054	ZNF629	76353	25kb
ENSG00000099385	16	30819947	30931281	123	14	120000	0.58759	0.2784	BCL7C	76353	25kb
ENSG00000150281	16	30882928	30939881	67	12	120000	0.29735	0.3831	CTF1	76353	25kb
ENSG00000099364	16	30909376	30985104	86	9	120000	0.53684	0.29569	FBXL19	76353	25kb
ENSG00000175938	16	30935387	30992782	60	7	120000	0.66802	0.25206	ORAI3	76353	25kb
ENSG00000099381	16	30943615	31021437	94	11	120000	0.88655	0.18766	SETD1A	76353	25kb
ENSG00000099377	16	30971519	31025473	71	11	120000	1.0309	0.1513	HSD3B7	76353	25kb
ENSG00000268863	16	30971808	31022533	67	10	120000	1.0256	0.15255	AC135048.1	76353	25kb
ENSG00000099365	16	30975577	31046949	91	12	120000	1.0845	0.13908	STX1B	76353	25kb
ENSG00000103496	16	31019210	31079296	86	10	120000	1.0463	0.1477	STX4	76353	25kb
ENSG00000167394	16	31047164	31110641	105	12	120000	1.0452	0.14797	ZNF668	76353	25kb

ENSG00000232748	16	31047813	31098451	83	10	120000	1.1196	0.13143	ZNF668	76353	25kb
ENSG00000167395	16	31060743	31120517	85	11	120000	0.89689	0.18489	ZNF646	76353	25kb
ENSG00000151006	16	31069746	31125949	76	10	120000	0.75064	0.22644	PRSS53	76353	25kb
ENSG00000255439	16	31069760	31131277	85	10	120000	0.72633	0.23382	RP11-196G11.1	76353	25kb
ENSG00000167397	16	31077163	31132301	80	10	120000	0.78569	0.21602	VKORC1	76353	25kb
ENSG00000103507	16	31092428	31149110	75	9	120000	0.41373	0.33954	BCKDK	76353	25kb
ENSG00000103510	16	31102075	31167714	84	12	120000	0.25329	0.40002	KAT8	76353	25kb
ENSG00000052344	16	31117756	31172083	69	12	120000	0.19085	0.42432	PRSS8	76353	25kb
ENSG00000178226	16	31125246	31186415	73	14	120000	0.10972	0.45632	PRSS36	76353	25kb
ENSG00000089280	16	31166431	31228127	45	11	120000	-0.16787	0.56666	FUS	76353	25kb
ENSG00000103490	16	31187806	31239771	39	12	120000	-0.033152	0.51322	PYCARD	76353	25kb
ENSG00000261359	16	31188206	31239773	39	12	120000	-0.033152	0.51322	C16orf98	76353	25kb
ENSG00000177238	16	31200342	31261510	45	11	120000	0.23151	0.40846	TRIM72	76353	25kb
ENSG00000169900	16	31202283	31253680	31	9	120000	0.13675	0.44562	PYDC1	76353	25kb
ENSG00000169896	16	31246311	31369213	254	15	120000	0.043493	0.48265	ITGAM	76353	25kb
ENSG00000140678	16	31341455	31419318	162	23	120000	-0.71903	0.76394	ITGAX	76353	25kb
ENSG00000156886	16	31379633	31462826	121	21	120000	-1.2727	0.89843	ITGAD	76353	25kb
ENSG00000156885	16	31414052	31464967	75	17	120000	-1.2414	0.89276	COX6A2	76353	25kb
ENSG00000176723	16	31420569	31479346	88	21	120000	-1.0108	0.84395	ZNF843	76353	25kb
ENSG00000140691	16	31444401	31503487	72	18	120000	0.020058	0.492	ARMC5	76353	25kb
ENSG00000140682	16	31457906	31514281	61	16	120000	0.048087	0.48082	TGFB1I1	76353	25kb
ENSG00000140675	16	31469323	31527181	78	15	120000	-0.17648	0.57004	SLC5A2	76353	25kb
ENSG00000140688	16	31475792	31545630	144	15	120000	0.0077288	0.49692	C16orf58	76353	25kb
ENSG00000169877	16	31514185	31565124	178	17	120000	0.13353	0.44689	AHSP	76353	25kb
ENSG00000197302	16	31699550	31831190	255	11	120000	1.465	0.071464	ZNF720	76353	25kb
ENSG00000185947	16	31860079	31953668	109	8	120000	-0.18017	0.57149	ZNF267	76353	25kb
ENSG00000223931	16	32038311	32088601	1	1	120000	-0.61585	0.731	AC142381.1	76353	25kb
ENSG00000179755	16	34231507	34282278	15	6	120000	-0.50576	0.69349	CTD-2144E22.5	76353	25kb
ENSG00000171241	16	46589466	46680538	48	17	120000	-0.66163	0.7459	SHCBP1	76353	25kb
ENSG00000069329	16	46665054	46748430	42	6	120000	-0.69873	0.75764	VPS35	76353	25kb
ENSG00000091651	16	46698555	46757306	29	5	120000	-0.75899	0.77607	ORC6	76353	25kb
ENSG00000140795	16	46715891	46849319	87	9	120000	-0.60348	0.7269	MYLK3	76353	25kb
ENSG00000155330	16	46805519	46890323	57	7	120000	-0.69488	0.75643	C16orf87	76353	25kb
ENSG00000166123	16	46893290	46990209	85	15	120000	-0.87296	0.80866	GPT2	76353	25kb
ENSG00000069345	16	46964299	47032699	62	10	120000	-1.6984	0.95529	DNAJA2	76353	25kb
ENSG00000171208	16	47086614	47202908	95	11	120000	-0.72765	0.76658	NETO2	76353	25kb
ENSG00000129636	16	47163298	47523060	300	17	120000	-0.71929	0.76402	ITFG1	76353	25kb
ENSG00000102893	16	47470034	47760434	225	16	120000	-0.96862	0.83363	PHKB	76353	25kb
ENSG00000140798	16	48091884	48214929	123	20	120000	-1.6446	0.94997	ABCC12	76353	25kb
ENSG00000121270	16	48175821	48306479	296	16	120000	-1.4766	0.93011	ABCC11	76353	25kb
ENSG00000102910	16	48253207	48422033	280	13	120000	-1.178	0.88061	LONP2	76353	25kb
ENSG00000196470	16	48365275	48507313	213	34	120000	-1.7435	0.95938	SIAH1	76353	25kb
ENSG00000102921	16	48547637	48679059	248	10	120000	-0.4327	0.66738	N4BP1	76353	25kb
ENSG00000102924	16	49286828	49340742	121	23	120000	-1.2473	0.89386	CBLN1	76353	25kb
ENSG00000166152	16	49382734	49458342	185	22	120000	-0.6183	0.73181	C16orf78	76353	25kb
ENSG00000102935	16	49496435	49916830	1258	128	120000	0.63135	0.26391	ZNF423	76353	25kb
ENSG00000205423	16	50033321	50095999	173	17	120000	-0.65707	0.74443	CNEP1R1	76353	25kb
ENSG00000155393	16	50074852	50165298	257	14	120000	-0.73276	0.76815	HEATR3	76353	25kb
ENSG00000121274	16	50161829	50294221	283	30	120000	-0.67995	0.75173	PAPD5	76353	25kb
ENSG00000121281	16	50255048	50377046	281	45	120000	-0.91628	0.82024	ADCY7	76353	25kb
ENSG00000166164	16	50322398	50427845	227	32	120000	-0.64115	0.73929	BRD7	76353	25kb
ENSG00000140807	16	50557241	50695647	189	31	120000	1.9045	0.028421	NKD1	76353	25kb
ENSG00000167208	16	50675211	50740264	104	19	120000	1.0516	0.1465	SNX20	76353	25kb
ENSG00000167207	16	50702514	50791988	148	20	120000	-0.61362	0.73027	NOD2	76353	25kb
ENSG00000083799	16	50750961	50860846	207	29	120000	-0.31713	0.62443	CYLD	76353	25kb
ENSG00000103449	16	51144886	51210278	124	26	120000	-1.4233	0.92268	SALL1	76353	25kb
ENSG00000261190	16	52014399	52137386	289	43	120000	-0.9084	0.81817	C16orf97	76353	25kb
ENSG00000103460	16	52446917	52606714	357	50	120000	-0.59233	0.72318	TOX3	76353	25kb
ENSG00000177200	16	53063945	53388062	779	26	120000	-0.97169	0.8344	CHD9	76353	25kb
ENSG00000103479	16	53442889	53550561	204	18	120000	0.92133	0.17844	RBL2	76353	25kb
ENSG00000166971	16	53499952	53563323	155	24	120000	1.0409	0.14896	AKTIP	76353	25kb
ENSG00000103494	16	53606595	53762850	328	23	120000	-0.89966	0.81585	RPGRIP1L	76353	25kb
ENSG00000140718	16	53712875	54180853	1424	131	120000	0.58816	0.27821	FTO	76353	25kb
ENSG00000259517	16	54254456	54329658	259	19	120000	-0.31245	0.62265	RP11-324D17.1	76353	25kb
ENSG00000177508	16	54292216	54345675	152	25	120000	-1.1271	0.87015	IRX3	76353	25kb
ENSG00000176842	16	54939774	54993397	108	11	120000	-0.78389	0.78345	IRX5	76353	25kb
ENSG00000159387	16	55332672	55389672	161	16	120000	1.5378	0.062051	IRX6	76353	25kb
ENSG00000087245	16	55398612	55565603	748	65	120000	-0.0047757	0.50191	MMP2	76353	25kb
ENSG00000087253	16	55517910	55645582	322	23	120000	0.042144	0.48319	LPCAT2	76353	25kb
ENSG00000256812	16	55575584	55626599	119	13	120000	-0.35942	0.64036	CAPNS2	76353	25kb
ENSG00000103546	16	55664516	55765104	374	49	120000	-0.69242	0.75566	SLC6A2	76353	25kb
ENSG00000198848	16	55811763	55892249	215	41	120000	0.83444	0.20202	CES1	76353	25kb
ENSG00000159398	16	55855066	56014943	523	53	120000	0.13673	0.44562	CES5A	76353	25kb
ENSG00000087258	16	56200302	56416356	593	49	120000	0.74812	0.22719	GNAO1	76353	25kb
ENSG00000159461	16	56370364	56484450	273	23	120000	-0.58864	0.72195	AMFR	76353	25kb
ENSG00000167005	16	56438045	56511111	163	18	120000	-0.81537	0.79257	NUDT21	76353	25kb
ENSG00000087263	16	56460402	56538012	161	18	120000	-1.1146	0.86749	OGFOD1	76353	25kb
ENSG00000125124	16	56475748	56579195	279	25	120000	-1.3346	0.90899	BBS2	76353	25kb
ENSG00000102891	16	56573961	56627869	258	38	120000	-1.2209	0.88893	MT4	76353	25kb
ENSG00000087250	16	56597986	56650000	227	42	120000	-0.29399	0.61562	MT3	76353	25kb
ENSG00000125148	16	56617111	56668409	230	42	120000	0.14945	0.4406	MT2A	76353	25kb
ENSG00000169715	16	56634387	56686024	256	36	120000	-0.25103	0.59911	MT1E	76353	25kb
ENSG00000268317	16	56637971	56691150	277	35	120000	-0.36192	0.64129	AC026461.1	76353	25kb
ENSG00000205364	16	56641145	56692898	270	33	120000	-0.50056	0.69166	MT1M	76353	25kb
ENSG00000205362	16	56647578	56698999	286	28	120000	-0.55358	0.71007	MT1A	76353	25kb
ENSG00000169688	16	56660811	56712116	275	28	120000	-0.89992	0.81592	MT1B	76353	25kb
ENSG00000198417	16	56666606	56719610	281	31	120000	-1.0332	0.84925	MT1F	76353	25kb
ENSG00000125144	16	56675643	56726977	246	39	120000	-0.61725	0.73147	MT1G	76353	25kb
ENSG00000205358	16	56678726	56730041	249	40	120000	-0.34905	0.63647	MT1H	76353	25kb
ENSG00000187193	16	56691336	56743108	226	43	120000	-0.63115	0.73603	MT1X	76353	25kb
ENSG00000102900	16	56739017	56903797	370	30	120000	-0.61358	0.73025	NUP93	76353	25kb
ENSG00000070915	16	56874119	56974762	367	41	120000	0.28623	0.38735	SLC12A3	76353	25kb
ENSG00000051108	16	56940960	57002798	212	27	120000	0.95107	0.17078	HERPUD1	76353	25kb
ENSG00000087237	16	56970762	57042757	253	46	120000	0.23193	0.4083	CETP	76353	25kb
ENSG00000140853	16	56998397	57142443	565	93	120000	-0.42965	0.66627	NLRC5	76353	25kb
ENSG00000140848	16	57101449	57206878	356	60	120000	-1.3618	0.91337	CPNE2	76353	25kb

ENSG00000172775	16	57161378	57245028	177	25	120000	-1.4467	0.92601	FAM192A	76353	25kb
ENSG00000159579	16	57195049	57299387	187	21	120000	-1.7099	0.95636	RSPRY1	76353	25kb
ENSG00000102931	16	57254010	57312516	130	19	120000	-1.6235	0.94775	ARL2BP	76353	25kb
ENSG00000102934	16	57265004	57343599	260	30	120000	-0.58518	0.72079	PLLP	76353	25kb
ENSG00000102962	16	57367684	57425102	208	34	120000	-1.4859	0.93134	CCL22	76353	25kb
ENSG00000006210	16	57381370	57443960	242	38	120000	-0.92282	0.82195	CX3CL1	76353	25kb
ENSG00000102970	16	57413679	57474974	206	30	120000	-0.10003	0.53984	CCL17	76353	25kb
ENSG00000005194	16	57437081	57506440	212	30	120000	0.17735	0.42962	CIAPIN1	76353	25kb
ENSG00000088682	16	57456337	57520187	192	27	120000	0.21882	0.41339	COQ9	76353	25kb
ENSG00000102978	16	57471299	57530922	170	27	120000	0.30605	0.37978	POLR2C	76353	25kb
ENSG00000125170	16	57480863	57546239	203	36	120000	0.4712	0.31875	DOK4	76353	25kb
ENSG00000135736	16	57521090	57595511	240	37	120000	0.61617	0.26889	CCDC102A	76353	25kb
ENSG00000159618	16	57551333	57650593	356	33	120000	0.044942	0.48208	GPR114	76353	25kb
ENSG00000205336	16	57619564	57723944	462	79	120000	0.18914	0.42499	GPR56	76353	25kb
ENSG00000182885	16	57677099	57748975	232	54	120000	0.63881	0.26147	GPR97	76353	25kb
ENSG00000159625	16	57703705	57790717	247	51	120000	1.5689	0.058332	CCDC135	76353	25kb
ENSG00000140854	16	57744642	57816162	212	35	120000	2.7824	0.0026976	KATNB1	76353	25kb
ENSG00000140859	16	57767129	57921957	562	51	120000	2.3259	0.010011	KIFC3	76353	25kb
ENSG00000187185	16	57807098	57875850	240	40	120000	2.3957	0.0082948	CTD-2600O9.1	76353	25kb
ENSG00000070729	16	57892503	58030020	481	82	120000	-1.0021	0.84186	CNGB1	76353	25kb
ENSG00000159648	16	57985339	58047020	165	24	120000	0.40568	0.34249	TEPP	76353	25kb
ENSG00000166188	16	58003572	58059357	131	18	120000	0.64004	0.26107	ZNF319	76353	25kb
ENSG00000103005	16	58008450	58080522	151	23	120000	0.87294	0.19135	USB1	76353	25kb
ENSG00000102996	16	58034470	58105805	160	27	120000	0.36489	0.3576	MMP15	76353	25kb
ENSG00000070761	16	58122496	58188354	213	23	120000	1.3779	0.084114	C16orf80	76353	25kb
ENSG00000070770	16	58166811	58256824	318	24	120000	1.2604	0.10376	CSNK2A2	76353	25kb
ENSG00000103021	16	58240061	58342740	417	23	120000	0.43119	0.33316	CCDC113	76353	25kb
ENSG00000103023	16	58288901	58353951	258	11	120000	0.33161	0.37009	PRSS54	76353	25kb
ENSG00000181938	16	58303984	58465048	555	51	120000	-0.09138	0.5364	GINS3	76353	25kb
ENSG00000103034	16	58471750	58572532	269	53	120000	-0.75358	0.77445	NDRG4	76353	25kb
ENSG00000103037	16	58524383	58579431	144	21	120000	-0.36325	0.64179	SETD6	76353	25kb
ENSG00000125107	16	58528855	58688790	475	24	120000	-0.78744	0.78449	CNOT1	76353	25kb
ENSG00000103042	16	58674013	58744008	237	31	120000	-0.1755	0.56966	SLC38A7	76353	25kb
ENSG00000125166	16	58716035	58793261	337	24	120000	-0.311	0.6221	GOT2	76353	25kb
ENSG00000260240	16	59718897	59813752	308	29	120000	1.7182	0.04288	RP11-105C20.2	76353	25kb
ENSG00000150394	16	61656146	62095939	1003	62	120000	-0.96667	0.83314	CDH8	76353	25kb
ENSG00000140937	16	64952656	65185015	778	40	120000	-0.33812	0.63236	CDH11	76353	25kb
ENSG00000179776	16	66375533	66463686	283	36	120000	-1.1981	0.88456	CDH5	76353	25kb
ENSG00000166546	16	66436200	66552432	240	35	120000	0.72448	0.23438	BEAN1	76353	25kb
ENSG00000260851	16	66478715	66576215	161	17	120000	1.2668	0.10261	RP11-403P17.5	76353	25kb
ENSG00000247270	16	66491775	66544747	77	13	120000	1.4964	0.067272	AC132186.1	76353	25kb
ENSG00000166548	16	66516906	66611447	168	8	120000	0.787	0.21564	TK2	76353	25kb
ENSG00000217555	16	66561466	66625154	143	8	120000	0.6828	0.24737	CKLF	76353	25kb
ENSG00000254788	16	66561490	66638038	166	10	120000	0.71528	0.23722	CKLF-CMTM1	76353	25kb
ENSG00000089505	16	66575294	66638040	141	9	120000	0.71712	0.23665	CMTM1	76353	25kb
ENSG00000140932	16	66588351	66647178	132	11	120000	0.61985	0.26768	CMTM2	76353	25kb
ENSG00000140931	16	66612777	66672795	137	15	120000	-0.20722	0.58208	CMTM3	76353	25kb
ENSG00000183723	16	66623653	66755610	260	16	120000	-0.36009	0.64061	CMTM4	76353	25kb
ENSG00000135720	16	66729796	66810701	142	9	120000	1.3679	0.085669	DYNC1LI2	76353	25kb
ENSG00000249961	16	66763879	66860523	166	12	120000	1.3871	0.082698	CCDC79	76353	25kb
ENSG00000159593	16	66811778	66932159	208	19	120000	0.79839	0.21232	NAE1	76353	25kb
ENSG00000168748	16	66853282	66913056	100	11	120000	0.60488	0.27263	CA7	76353	25kb
ENSG00000172840	16	66887492	66954657	122	15	120000	-0.37352	0.64562	PDP2	76353	25kb
ENSG00000166589	16	66917025	66977887	94	16	120000	-0.32782	0.62848	CDH16	76353	25kb
ENSG00000166592	16	66930582	66984547	75	14	120000	-0.36147	0.64113	RRAD	76353	25kb
ENSG00000166595	16	66940959	66993326	64	15	120000	-0.39156	0.65231	FAM96B	76353	25kb
ENSG00000172831	16	66943347	67003999	72	17	120000	-0.26975	0.60632	CES2	76353	25kb
ENSG00000172828	16	66970140	67034051	73	19	120000	-0.02139	0.50853	CES3	76353	25kb
ENSG00000172824	16	66997492	67068661	78	17	120000	0.33392	0.36922	CES4A	76353	25kb
ENSG00000067955	16	67038019	67159961	129	12	120000	0.46603	0.3206	CBFB	76353	25kb
ENSG00000125149	16	67118861	67207442	79	14	120000	0.40905	0.34125	C16orf70	76353	25kb
ENSG00000237172	16	67157008	67210117	41	13	120000	0.43439	0.332	B3GNT9	76353	25kb
ENSG00000102871	16	67163083	67219201	53	13	120000	0.67229	0.2507	TRADD	76353	25kb
ENSG00000135722	16	67168834	67223473	55	14	120000	0.62589	0.26569	FBXL8	76353	25kb
ENSG00000265690	16	67172288	67224718	50	13	120000	0.63798	0.26174	RP11-5A19.5	76353	25kb
ENSG00000102878	16	67172288	67228848	54	13	120000	0.62357	0.26646	HSF4	76353	25kb
ENSG00000140939	16	67179057	67234643	60	12	120000	0.27445	0.39187	NOL3	76353	25kb
ENSG00000196123	16	67184505	67242943	72	12	120000	0.20678	0.41809	KIAA0895L	76353	25kb
ENSG00000179044	16	67193269	67249107	75	11	120000	0.14249	0.44335	EXOC3L1	76353	25kb
ENSG00000205250	16	67201072	67257821	77	11	120000	0.13212	0.44744	E2F4	76353	25kb
ENSG00000102890	16	67208014	67262932	79	11	120000	0.066174	0.47362	ELMO3	76353	25kb
ENSG00000125122	16	67216042	67285951	97	14	120000	0.20794	0.41764	LRRC29	76353	25kb
ENSG00000237102	16	67219251	67285930	92	13	120000	0.065838	0.47375	AC040160.1	76353	25kb
ENSG00000168701	16	67236006	67288181	70	12	120000	0.21158	0.41622	TMEM208	76353	25kb
ENSG00000135723	16	67238290	67306561	83	14	120000	0.15494	0.43843	FHOD1	76353	25kb
ENSG00000135740	16	67246586	67331093	104	18	120000	0.10483	0.45826	SLC9A5	76353	25kb
ENSG00000196155	16	67286413	67348402	68	12	120000	-0.070983	0.52829	PLEKHG4	76353	25kb
ENSG00000168676	16	67298331	67385666	121	15	120000	-0.067728	0.527	KCTD19	76353	25kb
ENSG00000159708	16	67335701	67444106	168	15	120000	0.21826	0.41361	LRRC36	76353	25kb
ENSG00000159713	16	67398712	67452438	87	12	120000	0.40988	0.34095	TPPP3	76353	25kb
ENSG00000159714	16	67403322	67475736	114	13	120000	0.43511	0.33174	ZDHHC1	76353	25kb
ENSG00000176387	16	67439555	67496456	82	11	120000	0.64554	0.25929	HSD11B2	76353	25kb
ENSG00000159720	16	67446917	67540140	119	14	120000	0.71698	0.23669	ATP6V0D1	76353	25kb
ENSG00000159723	16	67491474	67542716	53	9	120000	0.77924	0.21792	AGRP	76353	25kb
ENSG00000039523	16	67527321	67605691	97	17	120000	1.0285	0.15186	FAM65A	76353	25kb
ENSG00000102974	16	67571310	67698086	160	24	120000	0.94715	0.17178	CTCF	76353	25kb
ENSG00000159753	16	67653822	67716472	74	16	120000	0.23892	0.40559	RLTPR	76353	25kb
ENSG00000102977	16	67666415	67719713	62	12	120000	0.04599	0.48166	ACD	76353	25kb
ENSG00000102981	16	67669849	67721681	59	11	120000	-0.12782	0.55085	PARD6A	76353	25kb
ENSG00000124074	16	67671848	67726168	65	11	120000	-0.018663	0.50744	ENKD1	76353	25kb
ENSG00000159761	16	67675719	67727661	67	12	120000	-0.0037879	0.50151	C16orf86	76353	25kb
ENSG00000141098	16	67683434	67778324	95	12	120000	0.051312	0.47954	GFOD2	76353	25kb
ENSG00000141084	16	67732005	67865555	157	18	120000	0.18336	0.42726	RANBP10	76353	25kb
ENSG00000102904	16	67815668	67891051	106	15	120000	0.19015	0.42459	TSNAXIP1	76353	25kb
ENSG00000102901	16	67837060	67906714	89	12	120000	0.2646	0.39566	CENPT	76353	25kb
ENSG00000168286	16	67851213	67903097	71	11	120000	0.34861	0.36369	THAP11	76353	25kb

ENSG00000102898	16	67855635	67931470	96	13	120000	0.11967	0.45237	NUTF2	76353	25kb
ENSG00000038358	16	67881926	67943406	85	13	120000	0.087874	0.46499	EDC4	76353	25kb
ENSG00000188038	16	67893708	67947758	64	11	120000	-0.001646	0.50066	NRN1L	76353	25kb
ENSG00000159792	16	67902175	67988581	85	12	120000	0.034651	0.48618	PSKH1	76353	25kb
ENSG00000141086	16	67936543	67991317	50	9	120000	0.15782	0.4373	CTRL	76353	25kb
ENSG00000261884	16	67938517	67994920	49	9	120000	0.091483	0.46355	CTC-479C5.12	76353	25kb
ENSG00000205220	16	67943405	67995990	42	8	120000	0.068478	0.4727	PSMB10	76353	25kb
ENSG00000213398	16	67948653	68003034	49	9	120000	0.10176	0.45947	LCAT	76353	25kb
ENSG00000124067	16	67952377	68028504	84	13	120000	0.024991	0.49003	SLC12A4	76353	25kb
ENSG00000141096	16	67984566	68039732	75	12	120000	-0.17773	0.57053	DPEP3	76353	25kb
ENSG00000167261	16	67996297	68059489	87	13	120000	-0.32188	0.62623	DPEP2	76353	25kb
ENSG00000167264	16	67996649	68138223	205	18	120000	-0.36242	0.64148	DUS2	76353	25kb
ENSG00000182810	16	68030179	68082770	69	7	120000	-0.42935	0.66616	DDX28	76353	25kb
ENSG00000072736	16	68093654	68288162	307	14	120000	-0.21479	0.58503	NFATC3	76353	25kb
ENSG00000103067	16	68238014	68297005	107	10	120000	-0.12	0.54776	ESRP2	76353	25kb
ENSG00000103066	16	68254207	68319961	107	13	120000	-0.044922	0.51792	PLA2G15	76353	25kb
ENSG00000103064	16	68273433	68360722	179	21	120000	0.28821	0.38659	SLC7A6	76353	25kb
ENSG00000103061	16	68293406	68369849	154	18	120000	0.44146	0.32944	SLC7A6OS	76353	25kb
ENSG00000132600	16	68319877	68417466	259	17	120000	0.57515	0.28259	PRMT7	76353	25kb
ENSG00000103056	16	68367231	68507591	314	25	120000	-0.17328	0.56878	SMPD3	76353	25kb
ENSG00000184939	16	68538993	68634975	294	13	120000	-0.80051	0.78829	ZFP90	76353	25kb
ENSG00000062038	16	68645092	68781519	376	25	120000	0.14259	0.44331	CDH3	76353	25kb
ENSG00000039068	16	68746128	68894451	461	29	120000	-0.18529	0.5735	CDH1	76353	25kb
ENSG00000103047	16	68852507	69144083	596	33	120000	-0.45382	0.67502	TANGO6	76353	25kb
ENSG00000103044	16	69114467	69177622	102	22	120000	-0.12437	0.54949	HAS3	76353	25kb
ENSG00000168802	16	69126913	69191487	101	22	120000	-0.4597	0.67713	CHTF8	76353	25kb
ENSG00000263203	16	69127757	69179331	79	19	120000	-0.51113	0.69537	CHTF8	76353	25kb
ENSG00000141076	16	69140194	69290033	283	26	120000	-0.56224	0.71302	CIRH1A	76353	25kb
ENSG00000168807	16	69196032	69367955	353	23	120000	-0.47052	0.68101	SNTB2	76353	25kb
ENSG00000260914	16	69308585	69379614	151	17	120000	-0.6519	0.74277	RP11-343C2.11	76353	25kb
ENSG00000132612	16	69320259	69383949	138	16	120000	-0.60502	0.72742	VPS4A	76353	25kb
ENSG00000213380	16	69329043	69398570	140	18	120000	-0.72585	0.76604	COG8	76353	25kb
ENSG00000258429	16	69337524	69389498	109	16	120000	-0.5708	0.71593	PDF	76353	25kb
ENSG00000272617	16	69337547	69393774	113	16	120000	-0.63627	0.7377	RP11-343C2.12	76353	25kb
ENSG00000260371	16	69343994	69415209	131	17	120000	-0.8034	0.78913	RP11-343C2.9	76353	25kb
ENSG00000259900	16	69344179	69410633	129	17	120000	-0.79281	0.78606	RP11-343C2.7	76353	25kb
ENSG00000132603	16	69348333	69402014	113	17	120000	-0.72163	0.76474	NIP7	76353	25kb
ENSG00000157315	16	69352151	69410712	111	16	120000	-0.81091	0.79129	TMED6	76353	25kb
ENSG00000132604	16	69364464	69467474	209	34	120000	-0.91359	0.81953	TERF2	76353	25kb
ENSG00000103018	16	69433428	69525169	275	26	120000	0.27049	0.39339	CYB5B	76353	25kb
ENSG00000102908	16	69573997	69763569	406	26	120000	-0.75834	0.77588	NFAT5	76353	25kb
ENSG00000181019	16	69715899	69785854	158	22	120000	-0.86692	0.80701	NQO1	76353	25kb
ENSG00000141101	16	69750770	69813843	144	20	120000	-0.84481	0.80089	NOB1	76353	25kb
ENSG00000198373	16	69771209	70000644	521	27	120000	-1.103	0.86499	WWP2	76353	25kb
ENSG00000157322	16	69959810	70023141	102	12	120000	-1.5264	0.93654	CLEC18A	76353	25kb
ENSG00000090857	16	70122529	70220203	55	11	120000	-1.3217	0.90686	PDPR	76353	25kb
ENSG00000157335	16	70182225	70246264	24	10	120000	-1.046	0.85221	CLEC18C	76353	25kb
ENSG00000269746	16	70214303	70264683	16	7	120000	-1.1325	0.87129	AC009060.1	76353	25kb
ENSG00000269866	16	70233261	70283641	19	8	120000	-1.2451	0.89345	FKSG63	76353	25kb
ENSG00000223496	16	70259134	70310833	34	16	120000	-1.1834	0.88167	EXOSC6	76353	25kb
ENSG00000090861	16	70261198	70348446	66	23	120000	-1.1716	0.87931	AARS	76353	25kb
ENSG00000157349	16	70298566	70394186	86	28	120000	-0.92926	0.82362	DDX19B	76353	25kb
ENSG00000260537	16	70308097	70425163	110	30	120000	-0.56035	0.71238	RP11-529K1.3	76353	25kb
ENSG00000168872	16	70355732	70432286	79	24	120000	-0.28546	0.61235	DDX19A	76353	25kb
ENSG00000157350	16	70388338	70498140	109	29	120000	-0.62772	0.73491	ST3GAL2	76353	25kb
ENSG00000157353	16	70463324	70539177	76	18	120000	-0.45606	0.67583	FUK	76353	25kb
ENSG00000103051	16	70489471	70582468	111	15	120000	-0.042485	0.51694	COG4	76353	25kb
ENSG00000189091	16	70532691	70633820	137	14	120000	0.078512	0.46871	SF3B3	76353	25kb
ENSG00000157368	16	70588798	70719585	349	30	120000	0.75227	0.22595	IL34	76353	25kb
ENSG00000132613	16	70670107	70744969	250	38	120000	0.51135	0.30455	MTSS1L	76353	25kb
ENSG00000268927	16	70670570	70724739	187	29	120000	0.76007	0.22361	FLJ00418	76353	25kb
ENSG00000103043	16	70696342	70860064	331	35	120000	1.8374	0.033074	VAC14	76353	25kb
ENSG00000157423	16	70816281	71289625	383	45	120000	1.2522	0.10524	HYDIN	76353	25kb
ENSG00000180917	16	71290292	71348618	190	20	120000	0.93844	0.17401	CMTR2	76353	25kb
ENSG00000172137	16	71367616	71449341	231	28	120000	1.1424	0.12665	CALB2	76353	25kb
ENSG00000167377	16	71456500	71521998	136	20	120000	1.6967	0.044879	ZNF23	76353	25kb
ENSG00000261611	16	71456503	71548214	188	24	120000	1.9973	0.022894	AC010547.9	76353	25kb
ENSG00000157429	16	71473453	71623992	264	26	120000	2.2313	0.012831	ZNF19	76353	25kb
ENSG00000140835	16	71534136	71597649	107	21	120000	2.0527	0.02005	CHST4	76353	25kb
ENSG00000198650	16	71574563	71636033	113	20	120000	2.4735	0.0066897	TAT	76353	25kb
ENSG00000140832	16	71635064	71701017	150	19	120000	2.0816	0.01869	MARVELD3	76353	25kb
ENSG00000040199	16	71646738	71783604	292	18	120000	1.7632	0.038935	PHLPP2	76353	25kb
ENSG00000166747	16	71737913	71868104	280	19	120000	1.6322	0.051319	AP1G1	76353	25kb
ENSG00000224470	16	71854894	71944171	185	18	120000	1.1601	0.12301	ATXN1L	76353	25kb
ENSG00000182149	16	71854899	71987913	283	17	120000	1.1175	0.1319	IST1	76353	25kb
ENSG00000102984	16	71868583	71954239	175	18	120000	1.2545	0.10483	ZNF821	76353	25kb
ENSG00000102967	16	72017487	72083954	226	29	120000	-0.34942	0.63661	DHODH	76353	25kb
ENSG00000140830	16	72053188	72153330	264	20	120000	0.22171	0.41227	TXNL4B	76353	25kb
ENSG00000257017	16	72063491	72119954	133	19	120000	-0.028139	0.51122	HP	76353	25kb
ENSG00000261701	16	72063522	72136145	187	20	120000	0.39036	0.34814	HPR	76353	25kb
ENSG00000140829	16	72102461	72171811	200	23	120000	0.64735	0.2587	DHX38	76353	25kb
ENSG00000118557	16	72121056	72235777	321	25	120000	0.46184	0.3221	PMFBP1	76353	25kb
ENSG00000140836	16	72791784	73118597	794	106	120000	1.3579	0.087254	ZFHX3	76353	25kb
ENSG00000197445	16	73135536	73203346	241	39	120000	-0.44052	0.67022	C16orf47	76353	25kb
ENSG00000103035	16	74305673	74365186	122	25	120000	-0.61117	0.72946	PSMD7	76353	25kb
ENSG00000196436	16	74386776	74450978	23	10	120000	0.69373	0.24393	NPIPB15	76353	25kb
ENSG00000140839	16	74417529	74480368	72	13	120000	-0.47078	0.6811	CLEC18B	76353	25kb
ENSG00000090863	16	74460856	74666012	606	27	120000	0.78644	0.2158	GLG1	76353	25kb
ENSG00000168411	16	74630292	74725779	322	23	120000	1.564	0.058907	RFWD3	76353	25kb
ENSG00000168404	16	74680753	74759858	304	42	120000	1.551	0.06045	MLKL	76353	25kb
ENSG00000103089	16	74721853	74833729	406	52	120000	1.6943	0.045105	FA2H	76353	25kb
ENSG00000103091	16	74882468	75059071	602	67	120000	2.2297	0.012885	WDR59	76353	25kb
ENSG00000186187	16	75007928	75169892	426	36	120000	0.37463	0.35397	ZNRF1	76353	25kb
ENSG00000166816	16	75120758	75175669	163	21	120000	-0.47919	0.6841	LDHD	76353	25kb
ENSG00000184517	16	75157390	75231134	331	28	120000	-0.099835	0.53976	ZFP1	76353	25kb
ENSG00000168928	16	75212994	75266083	303	26	120000	0.01117	0.49554	CTRB2	76353	25kb

ENSG00000168925	16	75227898	75283822	318	23	120000	0.078684	0.46864	CTRB1	76353	25kb
ENSG00000050820	16	75237928	75326951	418	29	120000	0.2019	0.42	BCAR1	76353	25kb
ENSG00000153774	16	75302596	75492383	663	20	120000	-1.0247	0.84725	CFDP1	76353	25kb
ENSG00000261717	16	75421582	75523604	392	18	120000	-1.1179	0.86819	RP11-77K12.1	76353	25kb
ENSG00000166822	16	75451952	75524395	253	16	120000	-1.1956	0.88408	TMEM170A	76353	25kb
ENSG00000183196	16	75485949	75554282	222	36	120000	-0.32014	0.62557	CHST6	76353	25kb
ENSG00000260092	16	75537430	75604326	198	20	120000	0.30627	0.3797	RP11-77K12.7	76353	25kb
ENSG00000135702	16	75537433	75594145	178	18	120000	0.13907	0.4447	CHST5	76353	25kb
ENSG00000205084	16	75547015	75615184	200	19	120000	0.66742	0.25225	TMEM231	76353	25kb
ENSG00000034713	16	75575249	75636779	165	20	120000	0.8615	0.19448	GABARAPL2	76353	25kb
ENSG00000065457	16	75605879	75682198	243	25	120000	0.75732	0.22443	ADAT1	76353	25kb
ENSG00000065427	16	75636622	75707541	235	25	120000	0.5287	0.29851	KARS	76353	25kb
ENSG00000166848	16	75656684	75820770	436	32	120000	-0.026621	0.51062	TERF2IP	76353	25kb
ENSG00000214325	16	75703367	75759089	153	14	120000	-0.24093	0.59519	AC025287.1	76353	25kb
ENSG00000152910	16	76286176	76618135	1391	65	120000	-0.61176	0.72965	CNTNAP4	76353	25kb
ENSG00000261833	16	76562314	76694520	384	54	120000	-0.47242	0.68169	RP11-58C22.1	76353	25kb
ENSG00000103111	16	77199732	77261302	306	22	120000	0.60116	0.27387	MON1B	76353	25kb
ENSG00000205078	16	77208294	77272112	346	24	120000	0.52806	0.29873	SYCE1L	76353	25kb
ENSG00000140873	16	77256710	77494011	1260	65	120000	2.3951	0.0083087	ADAMTS18	76353	25kb
ENSG00000140876	16	77731411	77801157	473	47	120000	0.16324	0.43517	NUDT7	76353	25kb
ENSG00000171724	16	77797427	78039004	1191	116	120000	0.99753	0.15925	VAT1L	76353	25kb
ENSG00000166509	16	78031412	78125658	697	25	120000	-1.1532	0.87559	CLEC3A	76353	25kb
ENSG00000186153	16	78108310	79271564	7111	393	120000	-1.171	0.87919	WWOX	76353	25kb
ENSG00000268977	16	78990647	79040739	344	58	120000	-1.9312	0.97327	PIH1	76353	25kb
ENSG00000178573	16	79594740	79659611	286	53	120000	1.2156	0.11207	MAF	76353	25kb
ENSG00000168589	16	80549631	80609657	250	31	120000	0.30241	0.38117	DYNLRB2	76353	25kb
ENSG00000166446	16	80606803	80863226	901	65	120000	-1.0852	0.86109	CDYL2	76353	25kb
ENSG00000103121	16	80984698	81078875	466	20	120000	0.94375	0.17265	CMC2	76353	25kb
ENSG00000166451	16	81015103	81091719	298	13	120000	1.1193	0.1315	CENPN	76353	25kb
ENSG00000166454	16	81044452	81105963	168	15	120000	0.44939	0.32658	ATMIN	76353	25kb
ENSG00000166455	16	81062102	81135872	236	11	120000	-0.53155	0.70248	C16orf46	76353	25kb
ENSG00000140905	16	81090566	81155008	288	27	120000	-0.44805	0.67294	GCSH	76353	25kb
ENSG00000135697	16	81247053	81349747	406	51	120000	-1.0226	0.84674	BCMO1	76353	25kb
ENSG00000261609	16	81323557	81449489	640	46	120000	0.14898	0.44078	GAN	76353	25kb
ENSG00000153815	16	81453775	81770367	1356	132	120000	-0.36522	0.64253	CMIP	76353	25kb
ENSG00000197943	16	81747702	82016899	1560	144	120000	0.80921	0.2092	PLCG2	76353	25kb
ENSG00000184860	16	82006221	82070093	115	24	120000	-0.19699	0.57808	SDR42E1	76353	25kb
ENSG00000086696	16	82043609	82157139	274	35	120000	-0.79315	0.78616	HSD17B2	76353	25kb
ENSG00000135698	16	82156403	82228831	506	14	120000	-0.53559	0.70388	MPHOSPH6	76353	25kb
ENSG00000140945	16	82635408	83855204	7033	367	120000	-1.9386	0.97372	CDH13	76353	25kb
ENSG00000230989	16	83816448	83878342	357	34	120000	-1.5106	0.93455	HSBP1	76353	25kb
ENSG00000103150	16	83907731	83974787	339	34	120000	-0.447	0.67256	MLYCD	76353	25kb
ENSG00000260300	16	83916738	84010058	506	48	120000	-0.56923	0.7154	RP11-505K9.4	76353	25kb
ENSG00000140961	16	83956887	84024937	430	50	120000	-0.23917	0.59451	OSGIN1	76353	25kb
ENSG00000103154	16	83977237	84061381	664	71	120000	-0.20368	0.5807	NECAB2	76353	25kb
ENSG00000166558	16	84018272	84101241	676	79	120000	1.2566	0.10445	SLC38A8	76353	25kb
ENSG00000140943	16	84062368	84175511	565	42	120000	1.1948	0.11609	MBTPS1	76353	25kb
ENSG00000103160	16	84130886	84203797	253	29	120000	-0.2736	0.6078	HSDL1	76353	25kb
ENSG00000154099	16	84153865	84237373	390	45	120000	-0.59793	0.72506	DNAAF1	76353	25kb
ENSG00000103168	16	84186458	84245669	357	41	120000	-0.55537	0.71068	TAF1C	76353	25kb
ENSG00000140955	16	84199744	84255774	341	42	120000	-0.52537	0.70034	ADAD2	76353	25kb
ENSG00000168418	16	84230823	84298356	427	71	120000	-1.5373	0.93789	KCNG4	76353	25kb
ENSG00000103175	16	84303252	84388450	562	89	120000	-0.018545	0.5074	WFDC1	76353	25kb
ENSG00000064270	16	84377133	84522793	1047	94	120000	-0.72272	0.76507	ATP2C2	76353	25kb
ENSG00000140950	16	84486681	84612639	856	71	120000	-0.4535	0.6749	TLDC1	76353	25kb
ENSG00000103187	16	84574200	84676683	550	72	120000	-0.85501	0.80373	COTL1	76353	25kb
ENSG00000135686	16	84657131	84726292	311	36	120000	-2.2686	0.98835	KLHL36	76353	25kb
ENSG00000103194	16	84708584	84838528	550	47	120000	-1.1498	0.87489	USP10	76353	25kb
ENSG00000103196	16	84828590	84979374	814	89	120000	-1.6794	0.95346	CRISPLD2	76353	25kb
ENSG00000153786	16	84982787	85070141	400	36	120000	-1.2594	0.89606	ZDHHC7	76353	25kb
ENSG00000135709	16	85036375	85152836	611	45	120000	-0.67448	0.75	KIAA0513	76353	25kb
ENSG00000153789	16	85106965	85171114	351	51	120000	-0.16363	0.56499	FAM92B	76353	25kb
ENSG00000262601	16	85179882	85243969	289	38	120000	-0.72461	0.76565	CTC-786C10.1	76353	25kb
ENSG00000261567	16	85366069	85418055	214	48	120000	-0.87727	0.80983	RP11-680G10.1	76353	25kb
ENSG00000131149	16	85620015	85734810	312	49	120000	-0.92807	0.82331	GSE1	76353	25kb
ENSG00000131153	16	85684804	85748679	197	24	120000	-0.12978	0.55163	GINS2	76353	25kb
ENSG00000154102	16	85698690	85809735	378	34	120000	0.95583	0.16958	C16orf74	76353	25kb
ENSG00000131148	16	85780364	85858214	212	27	120000	-0.07419	0.52957	EMC8	76353	25kb
ENSG00000131143	16	85807239	85865650	153	17	120000	-0.067548	0.52693	COX4I1	76353	25kb
ENSG00000140968	16	85907409	85981215	341	40	120000	-0.29327	0.61534	IRF8	76353	25kb
ENSG00000103241	16	86519133	86573076	304	40	120000	-2.0211	0.97837	FOXF1	76353	25kb
ENSG00000103248	16	86538782	86613841	336	30	120000	-1.1953	0.88402	MTHFSD	76353	25kb
ENSG00000176692	16	86575857	86627539	185	30	120000	-0.56797	0.71497	FOXC2	76353	25kb
ENSG00000176678	16	86584974	86640303	237	30	120000	-0.38473	0.64978	FOXL1	76353	25kb
ENSG00000260456	16	87092168	87376022	1265	87	120000	0.32048	0.3743	C16orf95	76353	25kb
ENSG00000131152	16	87311482	87392879	358	37	120000	-1.6449	0.95	RP11-178L8.4	76353	25kb
ENSG00000103264	16	87335593	87450748	391	45	120000	-1.0917	0.86251	FBXO31	76353	25kb
ENSG00000140941	16	87392601	87463385	209	28	120000	-0.52726	0.70099	MAP1LC3B	76353	25kb
ENSG00000140948	16	87414852	87550651	463	25	120000	-1.0261	0.84758	ZCCHC14	76353	25kb
ENSG00000154118	16	87610441	87756762	704	79	120000	1.1227	0.13077	JPH3	76353	25kb
ENSG00000226180	16	87702144	87754753	223	36	120000	-0.050694	0.52022	AC010536.1	76353	25kb
ENSG00000104731	16	87705091	87824598	514	37	120000	0.75547	0.22498	KLHDC4	76353	25kb
ENSG00000205047	16	87706749	87764290	262	31	120000	-0.13064	0.55197	FLJ00104	76353	25kb
ENSG00000103257	16	87838629	87928094	552	44	120000	1.7071	0.043906	SLC7A5	76353	25kb
ENSG00000174990	16	87896625	87995135	541	51	120000	-0.29653	0.61659	CA5A	76353	25kb
ENSG00000172530	16	87957850	88135924	749	29	120000	-1.2811	0.89991	BANP	76353	25kb
ENSG00000225614	16	88468879	88532165	211	36	120000	-0.7502	0.77343	ZNF469	76353	25kb
ENSG00000179588	16	88494725	88628424	551	64	120000	-0.61829	0.73181	ZFPM1	76353	25kb
ENSG00000158545	16	88611789	88723374	470	55	120000	-1.2823	0.90012	ZC3H18	76353	25kb
ENSG00000124391	16	88679999	88731881	197	45	120000	-1.0887	0.86186	IL17C	76353	25kb
ENSG00000051523	16	88684691	88742560	210	46	120000	-0.88607	0.81221	CYBA	76353	25kb
ENSG00000167508	16	88693343	88754569	203	43	120000	-0.6824	0.75251	MVD	76353	25kb
ENSG00000185669	16	88719090	88777901	221	36	120000	-0.46042	0.67739	SNAI3	76353	25kb
ENSG00000158717	16	88737903	88797829	258	31	120000	-0.80276	0.78894	RNF166	76353	25kb
ENSG00000174177	16	88747871	88806794	285	32	120000	-0.40412	0.65694	CTU2	76353	25kb
ENSG00000103335	16	88756751	88876619	600	63	120000	-0.53322	0.70306	PIEZO1	76353	25kb

ENSG00000167513	16	88844621	88900666	284	33	120000	-0.69821	0.75748	CDT1	76353	25kb
ENSG00000198931	16	88850747	88903352	282	26	120000	-0.41629	0.6614	APRT	76353	25kb
ENSG00000141012	16	88855142	88948378	471	35	120000	-0.21778	0.5862	GALNS	76353	25kb
ENSG00000269422	16	88869366	88919545	317	22	120000	-0.72124	0.76462	AC092384.1	76353	25kb
ENSG00000167515	16	88897628	88954094	260	26	120000	0.24432	0.40349	TRAPPC2L	76353	25kb
ENSG00000205022	16	88903034	88958068	243	31	120000	0.1496	0.44054	PABPN1L	76353	25kb
ENSG00000129993	16	88916266	89068612	633	56	120000	1.1971	0.11563	CBFA2T3	76353	25kb
ENSG00000205018	16	88981197	89042932	240	36	120000	0.65454	0.25638	RP11-830F9.6	76353	25kb
ENSG00000176715	16	89129783	89247254	599	31	120000	2.0424	0.020555	ACSF3	76353	25kb
ENSG00000129910	16	89213175	89286900	316	27	120000	0.052257	0.47916	CDH15	76353	25kb
ENSG00000259803	16	89237406	89293072	223	27	120000	-0.16919	0.56717	SLC22A31	76353	25kb
ENSG00000170100	16	89259118	89320363	298	21	120000	0.44653	0.32761	ZNF778	76353	25kb
ENSG00000167522	16	89309038	89581969	648	41	120000	-1.025	0.84732	ANKRD11	76353	25kb
ENSG00000268218	16	89309512	89364452	176	26	120000	0.32987	0.37075	AC137932.1	76353	25kb
ENSG00000197912	16	89532325	89649176	351	18	120000	-1.1485	0.87462	SPG7	76353	25kb
ENSG00000167526	16	89602065	89655950	166	22	120000	-0.63984	0.73886	RPL13	76353	25kb
ENSG00000178773	16	89617176	89688654	244	43	120000	-0.65489	0.74373	CPNE7	76353	25kb
ENSG00000015413	16	89654716	89729839	299	35	120000	-0.19658	0.57792	DPEP1	76353	25kb
ENSG00000131165	16	89685839	89749253	249	29	120000	0.23581	0.40679	CHMP1A	76353	25kb
ENSG00000167523	16	89699210	89762680	261	23	120000	0.27934	0.38999	SPATA33	76353	25kb
ENSG00000185324	16	89722145	89787772	254	28	120000	0.17261	0.43148	CDK10	76353	25kb
ENSG00000260259	16	89724028	89777977	211	29	120000	0.28697	0.38707	RP11-368I7.4	76353	25kb
ENSG00000158792	16	89737751	89793113	214	35	120000	-0.28689	0.6129	SPATA2L	76353	25kb
ENSG00000075399	16	89748542	89812394	302	36	120000	0.4318	0.33294	VPS9D1	76353	25kb
ENSG00000158805	16	89761808	89832311	395	34	120000	0.63826	0.26165	ZNF276	76353	25kb
ENSG00000187741	16	89778957	89908065	733	28	120000	0.61812	0.26825	FANCA	76353	25kb
ENSG00000204991	16	89859587	89962727	371	25	120000	0.65389	0.25659	SPIRE2	76353	25kb
ENSG00000141002	16	89915000	90002792	280	20	120000	0.92572	0.1773	TCF25	76353	25kb
ENSG00000258839	16	89953527	90012385	217	24	120000	0.66806	0.25205	MC1R	76353	25kb
ENSG00000198211	16	89960573	90027500	271	23	120000	0.51728	0.30248	TUBB3	76353	25kb
ENSG00000258947	16	89962800	90030169	270	24	120000	0.41166	0.34029	TUBB3	76353	25kb
ENSG00000140995	16	89989333	90059468	329	32	120000	0.63629	0.26229	DEF8	76353	25kb
ENSG00000177946	16	90011206	90063942	241	30	120000	0.9046	0.18284	CENPBD1	76353	25kb
ENSG00000003249	16	90046273	90111536	280	31	120000	0.81361	0.20793	DBNDD1	76353	25kb
ENSG00000141013	16	90061037	90136383	287	28	120000	0.75394	0.22544	GAS8	76353	25kb
ENSG00000221819	16	90070316	90121309	199	27	120000	0.89032	0.18665	C16orf3	76353	25kb
ENSG00000126856	16	90097974	90183480	233	30	120000	0.027046	0.48921	PRDM7	76353	25kb
ENSG00000272636	17	0	56427	204	25	120000	-0.96418	0.83252	DOC2B	76353	25kb
ENSG00000181031	17	37293	261045	1012	155	120000	-0.34597	0.63532	RPH3AL	76353	25kb
ENSG00000187624	17	235118	298510	193	34	120000	0.30325	0.38085	C17orf97	76353	25kb
ENSG00000183688	17	264769	320730	109	22	120000	0.88765	0.18736	FAM101B	76353	25kb
ENSG00000141252	17	386908	649957	806	39	120000	1.6483	0.049649	VPS53	76353	25kb
ENSG00000167695	17	610652	671206	236	38	120000	1.9657	0.024668	FAM57A	76353	25kb
ENSG00000179409	17	622654	682239	213	34	120000	1.8586	0.031541	GEMIN4	76353	25kb
ENSG00000167699	17	635337	710581	292	34	120000	1.8831	0.029842	GLOD4	76353	25kb
ENSG00000171861	17	660513	720749	251	46	120000	1.6821	0.046277	RNMTL1	76353	25kb
ENSG00000167693	17	677553	908010	1093	149	120000	0.48206	0.31488	NXN	76353	25kb
ENSG00000262003	17	787872	839452	331	57	120000	-0.18856	0.57478	RP11-676J12.7	76353	25kb
ENSG00000177370	17	875357	931911	174	34	120000	-0.25005	0.59872	TIMM22	76353	25kb
ENSG00000159842	17	881758	1157315	1085	145	120000	-0.055831	0.52226	ABR	76353	25kb
ENSG00000205899	17	1148853	1199754	221	50	120000	1.5945	0.055409	BHLHA9	76353	25kb
ENSG00000184811	17	1157957	1229281	341	49	120000	1.3572	0.087365	TUSC5	76353	25kb
ENSG00000108953	17	1222566	1328672	435	35	120000	0.5107	0.30478	YWHAE	76353	25kb
ENSG00000167193	17	1298983	1391456	294	30	120000	0.33997	0.36694	CRK	76353	25kb
ENSG00000197879	17	1342392	1421106	269	43	120000	-0.24783	0.59787	MYO1C	76353	25kb
ENSG00000132376	17	1372865	1445182	252	33	120000	-1.5872	0.94376	INPP5K	76353	25kb
ENSG00000174238	17	1396012	1491110	339	42	120000	-1.7061	0.95601	PITPNA	76353	25kb
ENSG00000167703	17	1447561	1557180	350	76	120000	-0.92388	0.82223	SLC43A2	76353	25kb
ENSG00000074660	17	1512152	1574041	181	29	120000	-0.65871	0.74496	SCARF1	76353	25kb
ENSG00000167705	17	1524444	1578371	155	24	120000	-0.51012	0.69502	RILP	76353	25kb
ENSG00000174231	17	1528923	1613176	250	37	120000	-0.71461	0.76258	PRPF8	76353	25kb
ENSG00000185561	17	1586064	1638732	147	37	120000	-1.4779	0.93028	TLCD2	76353	25kb
ENSG00000167716	17	1594817	1666893	221	38	120000	-0.50419	0.69293	WDR81	76353	25kb
ENSG00000167711	17	1621130	1683562	212	34	120000	-0.36318	0.64176	SERPINF2	76353	25kb
ENSG00000132386	17	1640253	1705868	242	36	120000	-0.64921	0.7419	SERPINF1	76353	25kb
ENSG00000186532	17	1657779	1758928	352	25	120000	-0.65012	0.74219	SMYD4	76353	25kb
ENSG00000132383	17	1707996	1828376	346	31	120000	0.024597	0.49019	RPA1	76353	25kb
ENSG00000185924	17	1812971	1953639	462	66	120000	0.16132	0.43592	RTN4RL1	76353	25kb
ENSG00000108963	17	1908404	1971724	178	30	120000	-0.54608	0.70749	DPH1	76353	25kb
ENSG00000262664	17	1920327	1971724	130	27	120000	-0.40209	0.65619	OVCA2	76353	25kb
ENSG00000177374	17	1932448	1987981	124	22	120000	-0.94668	0.8281	HIC1	76353	25kb
ENSG00000070366	17	1938133	2232065	693	33	120000	-0.60194	0.72639	SMG6	76353	25kb
ENSG00000167720	17	2181677	2253554	163	21	120000	-0.45697	0.67615	SRR	76353	25kb
ENSG00000167721	17	2200797	2265801	151	28	120000	0.1871	0.42579	TSR1	76353	25kb
ENSG00000141258	17	2215792	2309352	249	39	120000	0.45433	0.3248	SGSM2	76353	25kb
ENSG00000070444	17	2262354	2329412	196	33	120000	0.1038	0.45866	MNT	76353	25kb
ENSG00000127804	17	2283856	2440185	417	50	120000	-1.2161	0.88803	METTL16	76353	25kb
ENSG00000205821	17	2285279	2343731	127	25	120000	-0.69208	0.75556	AC006435.1	76353	25kb
ENSG00000007168	17	2471504	2613909	291	22	120000	-0.10022	0.53991	PAFAH1B1	76353	25kb
ENSG00000132361	17	2567680	2640957	142	31	120000	-0.30585	0.62014	CLUH	76353	25kb
ENSG00000132359	17	2655350	2966033	1144	171	120000	0.81559	0.20737	RAP1GAP2	76353	25kb
ENSG00000262628	17	2940963	2991901	87	19	120000	0.72462	0.23434	OR1D5	76353	25kb
ENSG00000184166	17	2970352	3021290	113	13	120000	-1.0132	0.84452	OR1D2	76353	25kb
ENSG00000183024	17	3004878	3055875	152	18	120000	-0.6758	0.75042	OR1G1	76353	25kb
ENSG00000172150	17	3075813	3126742	176	19	120000	-0.1566	0.56222	OR1A2	76353	25kb
ENSG00000172146	17	3093915	3144844	179	17	120000	0.099851	0.46023	OR1A1	76353	25kb
ENSG00000221882	17	3156193	3207268	164	14	120000	0.34908	0.36351	OR3A2	76353	25kb
ENSG00000180090	17	3169929	3220876	210	12	120000	0.52551	0.29962	OR3A1	76353	25kb
ENSG00000180016	17	3275398	3326704	138	14	120000	0.65072	0.25761	OR1E1	76353	25kb
ENSG00000159961	17	3298862	3349827	126	18	120000	-0.00069468	0.50028	OR3A3	76353	25kb
ENSG00000127780	17	3311164	3362135	151	16	120000	-0.39899	0.65505	OR1E2	76353	25kb
ENSG00000141255	17	3318313	3442146	465	60	120000	-0.76507	0.77789	SPATA22	76353	25kb
ENSG00000108381	17	3350668	3431713	349	48	120000	-1.6832	0.95383	ASPA	76353	25kb
ENSG00000167723	17	3388796	3486289	396	69	120000	-0.040831	0.51628	TRPV3	76353	25kb
ENSG00000196689	17	3443738	3525392	300	42	120000	-0.3748	0.6461	TRPV1	76353	25kb
ENSG00000262304	17	3443738	3564543	454	55	120000	0.29468	0.38412	SHPK	76353	25kb

ENSG00000197417	17	3486556	3564616	303	39	120000	0.39633	0.34593	SHPK	76353	25kb
ENSG00000040531	17	3514762	3589836	279	29	120000	1.0291	0.15171	CTNS	76353	25kb
ENSG00000213977	17	3541196	3596976	191	27	120000	1.4413	0.074749	TAX1BP3	76353	25kb
ENSG00000257950	17	3541357	3624488	313	30	120000	1.0593	0.14474	P2RX5-TAX1BP3	76353	25kb
ENSG00000127774	17	3547109	3597962	164	27	120000	1.3749	0.084577	EMC6	76353	25kb
ENSG00000083454	17	3550493	3624698	274	30	120000	0.96232	0.16794	P2RX5	76353	25kb
ENSG00000083457	17	3592922	3729537	474	56	120000	-0.44199	0.67075	ITGAE	76353	25kb
ENSG00000177602	17	3602211	3655067	213	20	120000	-0.25152	0.59929	GSG2	76353	25kb
ENSG00000074356	17	3689460	3774545	269	44	120000	-1.066	0.85679	C17orf85	76353	25kb
ENSG00000004660	17	3738609	3823185	277	56	120000	-0.39508	0.65361	CAMKK1	76353	25kb
ENSG00000108405	17	3774886	3844794	254	46	120000	0.29692	0.38326	P2RX1	76353	25kb
ENSG00000074370	17	3802169	3892736	312	36	120000	-0.57041	0.7158	ATP2A3	76353	25kb
ENSG00000074755	17	3882739	4071314	639	21	120000	0.2981	0.38281	ZZEF1	76353	25kb
ENSG00000167740	17	4021462	4115605	301	20	120000	0.99057	0.16095	CYB5D2	76353	25kb
ENSG00000185722	17	4042201	4192274	324	19	120000	1.118	0.13178	ANKFY1	76353	25kb
ENSG00000132388	17	4147554	4294923	227	16	120000	0.70513	0.24037	UBE2G1	76353	25kb
ENSG00000182557	17	4311983	4416503	538	59	120000	-0.52475	0.70012	SPNS3	76353	25kb
ENSG00000183018	17	4377133	4467330	352	71	120000	-0.31164	0.62234	SPNS2	76353	25kb
ENSG00000132382	17	4417192	4483926	265	49	120000	0.20801	0.41761	MYBBP1A	76353	25kb
ENSG00000167741	17	4435222	4489113	214	43	120000	0.4407	0.32971	GGT6	76353	25kb
ENSG00000188176	17	4462294	4536614	261	41	120000	0.24628	0.40273	SMTNL2	76353	25kb
ENSG00000161905	17	4509197	4570589	171	26	120000	0.5676	0.28515	ALOX15	76353	25kb
ENSG00000141456	17	4549679	4632632	283	15	120000	0.66614	0.25266	PELP1	76353	25kb
ENSG00000141480	17	4588784	4649794	193	23	120000	0.58926	0.27784	ARRB2	76353	25kb
ENSG00000161920	17	4609723	4661905	177	35	120000	0.42515	0.33536	MED11	76353	25kb
ENSG00000161921	17	4611821	4668217	181	38	120000	0.2964	0.38346	CXCL16	76353	25kb
ENSG00000141497	17	4618319	4674411	202	44	120000	0.39915	0.34489	ZMYND15	76353	25kb
ENSG00000142484	17	4650179	4711506	192	39	120000	-0.39442	0.65337	TM4SF5	76353	25kb
ENSG00000182853	17	4663580	4714728	155	31	120000	-0.58455	0.72057	VMO1	76353	25kb
ENSG00000182327	17	4667254	4718685	154	31	120000	-0.65029	0.74225	GLTPD2	76353	25kb
ENSG00000142507	17	4674439	4726790	148	34	120000	-1.0636	0.85625	PSMB6	76353	25kb
ENSG00000129219	17	4685391	4751729	182	38	120000	-0.59817	0.72514	PLD2	76353	25kb
ENSG00000141503	17	4711683	4826356	333	28	120000	-0.097999	0.53903	MINK1	76353	25kb
ENSG00000108556	17	4776069	4831369	157	20	120000	0.19243	0.4237	CHRNE	76353	25kb
ENSG00000205710	17	4777713	4831227	154	20	120000	0.17888	0.42901	C17orf107	76353	25kb
ENSG00000185245	17	4810592	4863325	135	21	120000	0.40031	0.34447	GP1BA	76353	25kb
ENSG00000108528	17	4815425	4868546	123	21	120000	0.28231	0.38885	SLC25A11	76353	25kb
ENSG00000108523	17	4818303	4873517	120	21	120000	0.19007	0.42463	RNF167	76353	25kb
ENSG00000108518	17	4823947	4877356	121	21	120000	0.12403	0.45065	PFN1	76353	25kb
ENSG00000108515	17	4826387	4885426	133	23	120000	-0.00025503	0.5001	ENO3	76353	25kb
ENSG00000091640	17	4837521	4896167	138	22	120000	0.060201	0.476	SPAG7	76353	25kb
ENSG00000108509	17	4846287	4915960	170	25	120000	0.11418	0.45455	CAMTA2	76353	25kb
ENSG00000196388	17	4866425	4925905	152	23	120000	-0.13619	0.55417	INCA1	76353	25kb
ENSG00000129250	17	4876243	4956696	238	29	120000	-0.16448	0.56533	KIF1C	76353	25kb
ENSG00000132517	17	4910895	4980304	221	32	120000	0.22478	0.41107	SLC52A1	76353	25kb
ENSG00000180787	17	4956543	5024669	210	29	120000	1.2956	0.097553	ZFP3	76353	25kb
ENSG00000167840	17	4983836	5051411	194	29	120000	1.5366	0.062193	ZNF232	76353	25kb
ENSG00000129204	17	4994733	5103329	252	25	120000	1.248	0.10602	USP6	76353	25kb
ENSG00000180626	17	5057831	5120178	123	19	120000	1.0187	0.15416	ZNF594	76353	25kb
ENSG00000161929	17	5087256	5163155	233	30	120000	1.4206	0.077716	SCIMP	76353	25kb
ENSG00000029725	17	5160558	5314129	538	26	120000	-0.82352	0.79489	RABEP1	76353	25kb
ENSG00000108559	17	5239258	5348480	410	21	120000	-0.31369	0.62312	NUP88	76353	25kb
ENSG00000129197	17	5297961	5361196	251	17	120000	0.22591	0.41064	RPAIN	76353	25kb
ENSG00000108561	17	5311097	5377150	230	23	120000	0.64284	0.26016	C1QBP	76353	25kb
ENSG00000005100	17	5319232	5397380	281	26	120000	0.55611	0.28907	DHX33	76353	25kb
ENSG00000072849	17	5349571	5415131	264	23	120000	-0.14794	0.5588	DERL2	76353	25kb
ENSG00000167842	17	5364605	5419134	226	18	120000	-0.7476	0.77265	MIS12	76353	25kb
ENSG00000091592	17	5377747	5547744	699	33	120000	-0.81248	0.79174	NLRP1	76353	25kb
ENSG00000179314	17	5650554	6052747	1567	175	120000	-0.68361	0.75289	WSCD1	76353	25kb
ENSG00000129221	17	6272013	6363519	438	69	120000	-0.25581	0.60095	AIPL1	76353	25kb
ENSG00000129195	17	6322735	6379789	283	42	120000	-0.68119	0.75212	FAM64A	76353	25kb
ENSG00000091622	17	6329584	6484814	574	58	120000	0.48278	0.31463	PITPNM3	76353	25kb
ENSG00000198920	17	6456468	6569247	353	22	120000	2.0988	0.017918	KIAA0753	76353	25kb
ENSG00000129235	17	6519078	6572861	191	19	120000	2.1071	0.017553	TXNDC17	76353	25kb
ENSG00000108590	17	6521635	6579954	223	25	120000	1.984	0.023627	MED31	76353	25kb
ENSG00000212734	17	6529971	6581791	207	24	120000	1.9005	0.028686	C17orf100	76353	25kb
ENSG00000141485	17	6563032	6641886	302	58	120000	-0.88683	0.81241	SLC13A5	76353	25kb
ENSG00000132530	17	6633766	6703966	252	30	120000	-1.2465	0.8937	XAF1	76353	25kb
ENSG00000177294	17	6654542	6725420	276	29	120000	-1.2136	0.88756	FBXO39	76353	25kb
ENSG00000167858	17	6667452	6760080	339	36	120000	-1.0316	0.84887	TEKT1	76353	25kb
ENSG00000215067	17	6754954	6940668	450	64	120000	-1.0731	0.85839	AC027763.2	76353	25kb
ENSG00000108839	17	6874384	6939055	178	20	120000	-0.18829	0.57468	ALOX12	76353	25kb
ENSG00000219200	17	6890736	6942851	136	16	120000	0.088312	0.46481	RNASEK	76353	25kb
ENSG00000269871	17	6890928	6942755	136	16	120000	0.088312	0.46481	AC040977.1	76353	25kb
ENSG00000161939	17	6890954	6945839	139	17	120000	0.063775	0.47457	RNASEK-C17orf49	76353	25kb
ENSG00000258315	17	6892814	6945844	135	17	120000	0.033938	0.48646	C17orf49	76353	25kb
ENSG00000161940	17	6901339	6958609	153	24	120000	-0.89929	0.81575	BCL6B	76353	25kb
ENSG00000174327	17	6914394	6968440	153	28	120000	-2.1034	0.98229	SLC16A13	76353	25kb
ENSG00000174326	17	6919949	6972242	156	30	120000	-2.2968	0.98919	SLC16A11	76353	25kb
ENSG00000132514	17	6952856	7008626	218	33	120000	0.58703	0.27859	CLEC10A	76353	25kb
ENSG00000161944	17	6979641	7044019	240	34	120000	1.5145	0.064948	ASGR2	76353	25kb
ENSG00000141505	17	7051750	7107883	111	31	120000	-0.58176	0.71964	ASGR1	76353	25kb
ENSG00000132535	17	7068209	7148021	127	30	120000	-0.45362	0.67495	DLG4	76353	25kb
ENSG00000072778	17	7095444	7153592	68	12	120000	-0.18092	0.57178	ACADVL	76353	25kb
ENSG00000004975	17	7103660	7162864	73	10	120000	-0.15214	0.56046	DVL2	76353	25kb
ENSG00000040633	17	7113347	7168041	65	9	120000	-0.44628	0.6723	PHF23	76353	25kb
ENSG00000170296	17	7118333	7171089	59	8	120000	-0.38201	0.64877	GABARAP	76353	25kb
ENSG00000262526	17	7118746	7172954	62	8	120000	-0.38148	0.64858	CTD-2545G14.7	76353	25kb
ENSG00000175826	17	7121910	7180810	72	9	120000	-0.36803	0.64358	CTDNEP1	76353	25kb
ENSG00000262302	17	7125148	7190408	84	11	120000	-0.33967	0.63295	RP1-4G17.5	76353	25kb
ENSG00000170291	17	7129735	7188259	82	10	120000	-0.38327	0.64924	ELP5	76353	25kb
ENSG00000181885	17	7138222	7192302	71	9	120000	-0.49598	0.69005	CLDN7	76353	25kb
ENSG00000181856	17	7159986	7216576	109	22	120000	-0.43686	0.66889	SLC2A4	76353	25kb
ENSG00000006047	17	7166571	7222934	115	23	120000	-0.20814	0.58244	YBX2	76353	25kb
ENSG00000132507	17	7185318	7240774	127	21	120000	0.3852	0.35005	EIF5A	76353	25kb
ENSG00000132522	17	7189643	7243883	124	21	120000	0.37093	0.35534	GPS2	76353	25kb

ENSG00000261915	17	7190980	7247493	130	22	120000	0.45543	0.3244	RP11-542C16.2	76353	25kb
ENSG00000215041	17	7193947	7257712	147	27	120000	0.73763	0.23037	NEURL4	76353	25kb
ENSG00000072818	17	7214848	7279797	177	40	120000	0.91182	0.18093	ACAP1	76353	25kb
ENSG00000213859	17	7230208	7283258	155	36	120000	0.94711	0.17179	KCTD11	76353	25kb
ENSG00000182896	17	7233442	7285538	154	35	120000	0.9721	0.1655	TMEM95	76353	25kb
ENSG00000174292	17	7258853	7318093	183	35	120000	0.28956	0.38608	TNK1	76353	25kb
ENSG00000187838	17	7268046	7332416	188	37	120000	-0.081423	0.53245	TMEM256-PLSCR3	76353	25kb
ENSG00000262481	17	7268053	7332416	188	37	120000	-0.081423	0.53245	C17orf61-PLSCR3	76353	25kb
ENSG00000205544	17	7281294	7332456	132	33	120000	-0.029339	0.5117	TMEM256	76353	25kb
ENSG00000169992	17	7283193	7348179	157	38	120000	-0.16851	0.56691	NLGN2	76353	25kb
ENSG00000181323	17	7298679	7349951	113	30	120000	-0.91859	0.82084	SPEM1	76353	25kb
ENSG00000184560	17	7303934	7355887	108	26	120000	-0.93648	0.82549	C17orf74	76353	25kb
ENSG00000181284	17	7313762	7365998	105	21	120000	-0.64588	0.74082	TMEM102	76353	25kb
ENSG00000161958	17	7316592	7373256	105	18	120000	-0.61874	0.73196	FGF11	76353	25kb
ENSG00000170175	17	7323380	7386026	125	18	120000	-0.59872	0.72532	CHRNB1	76353	25kb
ENSG00000174282	17	7337685	7412582	214	16	120000	-0.74838	0.77289	ZBTB4	76353	25kb
ENSG00000259224	17	7359721	7411383	162	11	120000	-0.81599	0.79275	SLC35G6	76353	25kb
ENSG00000181222	17	7362685	7442933	271	17	120000	-0.878	0.81003	POLR2A	76353	25kb
ENSG00000239697	17	7427208	7489925	183	28	120000	-0.16338	0.56489	TNFSF12	76353	25kb
ENSG00000248871	17	7427416	7489918	182	27	120000	-0.16576	0.56583	TNFSF12-TNFSF13	76353	25kb
ENSG00000161955	17	7436609	7489925	152	22	120000	0.076936	0.46934	TNFSF13	76353	25kb
ENSG00000161956	17	7440192	7500287	147	22	120000	0.026127	0.48958	SENP3	76353	25kb
ENSG00000161960	17	7451024	7507323	122	21	120000	-0.12282	0.54888	EIF4A1	76353	25kb
ENSG00000129226	17	7457785	7510429	109	21	120000	-0.15297	0.56079	CD68	76353	25kb
ENSG00000129255	17	7461847	7521107	108	21	120000	-0.20548	0.5814	MPDU1	76353	25kb
ENSG00000129194	17	7466496	7518488	99	18	120000	-0.13714	0.55454	SOX15	76353	25kb
ENSG00000129245	17	7469548	7543189	145	24	120000	-0.36328	0.6418	FXR2	76353	25kb
ENSG00000268503	17	7492264	7542427	88	18	120000	-0.51892	0.69809	AC007421.1	76353	25kb
ENSG00000129214	17	7492382	7561700	183	19	120000	-0.82238	0.79457	SHBG	76353	25kb
ENSG00000141504	17	7504552	7556194	154	15	120000	-0.9391	0.82616	SAT2	76353	25kb
ENSG00000129244	17	7524945	7586086	212	25	120000	-0.8034	0.78913	ATP1B2	76353	25kb
ENSG00000141510	17	7540097	7615856	249	31	120000	-0.82506	0.79533	TP53	76353	25kb
ENSG00000141499	17	7564389	7631820	196	37	120000	-1.0173	0.84549	WRAP53	76353	25kb
ENSG00000108947	17	7583520	7639696	143	28	120000	-1.4687	0.92904	EFNB3	76353	25kb
ENSG00000183914	17	7595672	7762062	374	45	120000	-0.017751	0.50708	DNAH2	76353	25kb
ENSG00000132510	17	7718222	7783114	132	25	120000	-0.052885	0.52109	KDM6B	76353	25kb
ENSG00000167874	17	7733383	7784417	106	19	120000	-0.013798	0.5055	TMEM88	76353	25kb
ENSG00000183011	17	7735003	7813556	146	27	120000	-0.59151	0.72291	LSMD1	76353	25kb
ENSG00000182224	17	7736064	7790600	112	20	120000	-0.17944	0.5712	CYB5D1	76353	25kb
ENSG00000170004	17	7763124	7841078	155	29	120000	-1.4504	0.92653	CHD3	76353	25kb
ENSG00000170049	17	7800177	7858121	127	14	120000	-1.7687	0.96153	KCNAB3	76353	25kb
ENSG00000262730	17	7804249	7858940	126	14	120000	-1.7525	0.96015	RP11-1099M24.7	76353	25kb
ENSG00000170043	17	7808663	7860441	121	13	120000	-1.7285	0.95805	TRAPPC1	76353	25kb
ENSG00000170037	17	7810419	7878236	188	17	120000	-1.5295	0.93693	CNTROB	76353	25kb
ENSG00000132518	17	7880912	7948657	225	36	120000	-0.37686	0.64686	GUCY2D	76353	25kb
ENSG00000179593	17	7917335	7977452	250	44	120000	-0.53653	0.7042	ALOX15B	76353	25kb
ENSG00000179477	17	7950954	8016021	255	46	120000	-0.38687	0.65057	ALOX12B	76353	25kb
ENSG00000214999	17	7957800	8009883	197	44	120000	-0.12711	0.55057	AC129492.6	76353	25kb
ENSG00000179148	17	7974218	8047365	251	60	120000	0.49863	0.30902	ALOXE3	76353	25kb
ENSG00000179111	17	7998908	8052410	188	40	120000	0.35933	0.35967	HES7	76353	25kb
ENSG00000179094	17	8018790	8084824	182	26	120000	0.83339	0.20231	PER1	76353	25kb
ENSG00000263620	17	8029134	8091293	161	21	120000	0.69993	0.24199	RP11-599B13.6	76353	25kb
ENSG00000220205	17	8037467	8091864	135	17	120000	0.69403	0.24383	VAMP2	76353	25kb
ENSG00000179029	17	8051555	8104717	125	16	120000	0.43456	0.33194	TMEM107	76353	25kb
ENSG00000196544	17	8066652	8118564	123	19	120000	0.58156	0.28043	C17orf59	76353	25kb
ENSG00000178999	17	8083056	8138918	139	23	120000	0.68537	0.24656	AURKB	76353	25kb
ENSG00000178971	17	8105191	8176362	188	27	120000	1.0267	0.15228	CTC1	76353	25kb
ENSG00000178921	17	8125936	8198809	204	27	120000	1.3967	0.081257	PFAS	76353	25kb
ENSG00000125434	17	8166081	8223661	194	24	120000	1.6913	0.045393	SLC25A35	76353	25kb
ENSG00000108961	17	8166815	8218410	174	22	120000	1.7051	0.044084	RANGRF	76353	25kb
ENSG00000198844	17	8188559	8250829	204	30	120000	1.2135	0.11247	ARHGEF15	76353	25kb
ENSG00000184650	17	8218182	8274364	177	16	120000	-0.12638	0.55028	ODF4	76353	25kb
ENSG00000198150	17	8236731	8288538	162	14	120000	-0.26286	0.60367	AC135178.1	76353	25kb
ENSG00000184619	17	8246955	8305029	163	18	120000	-0.39226	0.65257	KRBA2	76353	25kb
ENSG00000263809	17	8246956	8311505	173	21	120000	-0.4951	0.68973	RP11-849F2.7	76353	25kb
ENSG00000161970	17	8255838	8311531	130	20	120000	-0.35211	0.63762	RPL26	76353	25kb
ENSG00000189051	17	8269022	8326144	146	23	120000	-1.1147	0.86752	RNF222	76353	25kb
ENSG00000166579	17	8291449	8418729	300	31	120000	-2.1834	0.9855	NDEL1	76353	25kb
ENSG00000133026	17	8352523	8559079	467	24	120000	-1.8083	0.96472	MYH10	76353	25kb
ENSG00000161973	17	8608252	8673537	253	21	120000	-0.78081	0.78254	CCDC42	76353	25kb
ENSG00000183318	17	8631424	8686877	166	26	120000	-0.79193	0.7858	SPDYE4	76353	25kb
ENSG00000185156	17	8675436	8727667	137	20	120000	0.88832	0.18718	MFSD6L	76353	25kb
ENSG00000174083	17	8681041	8795994	274	37	120000	0.68378	0.24706	PIK3R6	76353	25kb
ENSG00000141506	17	8757233	8894029	327	47	120000	1.7747	0.037973	PIK3R5	76353	25kb
ENSG00000065320	17	8899859	9172317	994	109	120000	1.175	0.12	NTN1	76353	25kb
ENSG00000170310	17	9128788	9504908	1091	126	120000	1.5814	0.056893	STX8	76353	25kb
ENSG00000166596	17	9454944	9571776	218	34	120000	-0.04953	0.51975	WDR16	76353	25kb
ENSG00000154914	17	9523015	9658008	345	35	120000	0.34035	0.3668	USP43	76353	25kb
ENSG00000184544	17	9649751	9719614	300	39	120000	-0.092896	0.53701	DHRS7C	76353	25kb
ENSG00000214978	17	9669544	9750388	341	48	120000	-0.668	0.74793	RP11-477N12.3	76353	25kb
ENSG00000065325	17	9700523	9820419	540	46	120000	-0.89529	0.81468	GLP2R	76353	25kb
ENSG00000109047	17	9774637	9833938	267	29	120000	-0.91659	0.82032	RCVRN	76353	25kb
ENSG00000007237	17	9788926	10126868	1417	183	120000	-0.98043	0.83656	GAS7	76353	25kb
ENSG00000264194	17	10023831	10075277	227	50	120000	-1.7215	0.95742	AC000003.2	76353	25kb
ENSG00000006788	17	10176401	10301447	401	62	120000	-1.738	0.9589	MYH13	76353	25kb
ENSG00000133020	17	10268639	10350267	138	16	120000	-0.551	0.70918	MYH8	76353	25kb
ENSG00000264424	17	10321607	10397876	118	11	120000	-0.32031	0.62563	MYH4	76353	25kb
ENSG00000109061	17	10370624	10446860	131	13	120000	-0.17023	0.56759	MYH1	76353	25kb
ENSG00000125414	17	10399465	10478274	148	13	120000	-0.27284	0.60751	MYH2	76353	25kb
ENSG00000109063	17	10506843	10585626	238	29	120000	-1.3283	0.90796	MYH3	76353	25kb
ENSG00000133028	17	10558654	10626692	203	14	120000	-0.46453	0.67887	SCO1	76353	25kb
ENSG00000170222	17	10575911	10639550	152	4	120000	0.077319	0.46918	ADPRM	76353	25kb
ENSG00000187824	17	10577332	10658633	175	7	120000	0.098854	0.46063	TMEM220	76353	25kb
ENSG00000233670	17	10700792	10766762	195	38	120000	-0.79972	0.78806	PIRT	76353	25kb
ENSG00000188803	17	11119580	11492380	1241	131	120000	-0.068163	0.52717	SHISA6	76353	25kb
ENSG00000007174	17	11476748	11898065	1045	98	120000	-0.055994	0.52233	DNAH9	76353	25kb

ENSG00000154957	17	11855756	11925827	146	15	120000	0.75597	0.22483	ZNF18	76353	25kb
ENSG00000065559	17	11899141	12072147	358	33	120000	0.82502	0.20468	MAP2K4	76353	25kb
ENSG00000141052	17	12544207	12697266	433	61	120000	1.1006	0.13554	MYOCD	76353	25kb
ENSG00000273452	17	12601199	12686542	231	47	120000	1.4322	0.076044	AC005358.1	76353	25kb
ENSG00000006740	17	12667856	12919960	974	64	120000	0.97675	0.16435	ARHGAP44	76353	25kb
ENSG00000006744	17	12870708	12946504	355	22	120000	0.80156	0.2114	ELAC2	76353	25kb
ENSG00000153976	17	13374006	13530244	637	60	120000	1.7862	0.037032	HS3ST3A1	76353	25kb
ENSG00000006695	17	13947813	14136994	523	46	120000	-0.34846	0.63625	COX10	76353	25kb
ENSG00000223510	17	14113990	14165179	133	19	120000	-0.29418	0.61569	CDRT15	76353	25kb
ENSG00000125430	17	14179400	14277721	379	81	120000	0.50788	0.30577	HS3ST3B1	76353	25kb
ENSG00000109099	17	15108095	15193643	203	26	120000	-0.58844	0.72188	PMP22	76353	25kb
ENSG00000125409	17	15182128	15269958	267	25	120000	0.053123	0.47882	TEKT3	76353	25kb
ENSG00000239704	17	15314332	15431922	348	37	120000	-0.34705	0.63572	CDRT4	76353	25kb
ENSG00000259024	17	15314338	15491875	421	40	120000	-0.42023	0.66284	TVP23C-CDRT4	76353	25kb
ENSG00000175106	17	15316205	15491909	416	41	120000	-0.44031	0.67014	TVP23C	76353	25kb
ENSG00000181464	17	15443797	15494590	58	10	120000	-0.47866	0.68391	CDRT1	76353	25kb
ENSG00000241322	17	15443798	15547826	120	22	120000	-1.275	0.89885	CDRT1	76353	25kb
ENSG00000251537	17	15449805	15579967	180	32	120000	-1.3538	0.9121	RP11-385D13.1	76353	25kb
ENSG00000221926	17	15506274	15612625	169	28	120000	-0.78374	0.7834	TRIM16	76353	25kb
ENSG00000187607	17	15577891	15665874	132	22	120000	-0.19392	0.57688	ZNF286A	76353	25kb
ENSG00000255104	17	15578006	15673092	142	23	120000	-0.34435	0.63471	ZNF286A	76353	25kb
ENSG00000214946	17	15610561	15674476	86	15	120000	-0.68565	0.75353	TBC1D26	76353	25kb
ENSG00000170425	17	15823231	15904060	171	21	120000	0.56508	0.28601	ADORA2B	76353	25kb
ENSG00000214941	17	15854874	15928031	163	14	120000	0.71416	0.23756	ZSWIM7	76353	25kb
ENSG00000011295	17	15877694	15973329	200	12	120000	0.38439	0.35034	TTC19	76353	25kb
ENSG00000141027	17	15907471	16146499	415	14	120000	0.20315	0.41951	NCOR1	76353	25kb
ENSG00000227782	17	15918505	15968994	105	9	120000	0.3454	0.3649	AC002553.1	76353	25kb
ENSG00000108474	17	16095505	16277115	332	18	120000	0.3375	0.36787	PIGL	76353	25kb
ENSG00000166582	17	16220848	16281970	128	16	120000	0.61	0.27093	CENPV	76353	25kb
ENSG00000170315	17	16259112	16311059	101	14	120000	0.26037	0.39729	UBB	76353	25kb
ENSG00000187688	17	16293856	16365317	228	23	120000	0.069463	0.47231	TRPV2	76353	25kb
ENSG00000181350	17	16319891	16420467	313	29	120000	-0.11869	0.54724	FAM211A	76353	25kb
ENSG00000141040	17	16429701	16497520	151	15	120000	-0.41315	0.66025	ZNF287	76353	25kb
ENSG00000197566	17	16499051	16582170	239	14	120000	-0.18621	0.57386	ZNF624	76353	25kb
ENSG00000170160	17	16567851	16732767	101	17	120000	0.37138	0.35518	CCDC144A	76353	25kb
ENSG00000266302	17	16568575	16732767	97	16	120000	0.41057	0.34069	RP11-219A15.1	76353	25kb
ENSG00000240505	17	16807849	16900432	397	39	120000	0.407	0.342	TNFRSF13B	76353	25kb
ENSG00000133030	17	16920859	17145993	551	32	120000	2.6802	0.0036788	MPRIP	76353	25kb
ENSG00000179598	17	17079309	17134629	163	27	120000	2.1083	0.017504	PLD6	76353	25kb
ENSG00000264187	17	17080963	17165499	236	30	120000	1.9188	0.027503	RP11-45M22.4	76353	25kb
ENSG00000154803	17	17090526	17165502	214	27	120000	1.7708	0.0383	FLCN	76353	25kb
ENSG00000141030	17	17125141	17209607	252	18	120000	1.4496	0.073579	COPS3	76353	25kb
ENSG00000205309	17	17181649	17275977	287	33	120000	0.28283	0.38865	NT5M	76353	25kb
ENSG00000141026	17	17355300	17421540	162	20	120000	0.18917	0.42498	MED9	76353	25kb
ENSG00000108551	17	17372751	17424709	129	18	120000	0.04498	0.48206	RASD1	76353	25kb
ENSG00000133027	17	17383877	17520022	350	27	120000	-1.4303	0.92369	PEMT	76353	25kb
ENSG00000108557	17	17559787	17739767	268	26	120000	-1.4447	0.92573	RAI1	76353	25kb
ENSG00000072310	17	17688713	17765325	110	11	120000	-0.72722	0.76645	SREBF1	76353	25kb
ENSG00000175662	17	17721828	17900736	272	10	120000	-0.29201	0.61486	TOM1L2	76353	25kb
ENSG00000171962	17	17851127	17945203	143	10	120000	-0.23014	0.59101	LRRC48	76353	25kb
ENSG00000171953	17	17855723	17967523	180	11	120000	-0.29595	0.61637	ATPAF2	76353	25kb
ENSG00000141034	17	17917606	17996718	128	10	120000	-0.63799	0.73826	GID4	76353	25kb
ENSG00000108591	17	17966200	18036285	136	11	120000	-1.1789	0.88078	DRG2	76353	25kb
ENSG00000091536	17	17987020	18108116	237	25	120000	-1.0514	0.85346	MYO15A	76353	25kb
ENSG00000091542	17	18061392	18138268	182	14	120000	-0.3617	0.64121	ALKBH5	76353	25kb
ENSG00000131899	17	18103901	18173189	158	17	120000	-0.54912	0.70854	LLGL1	76353	25kb
ENSG00000177731	17	18123150	18187230	131	18	120000	-0.43186	0.66708	FLII	76353	25kb
ENSG00000177427	17	18138848	18194866	108	16	120000	-0.12664	0.55039	MIEF2	76353	25kb
ENSG00000177302	17	18149742	18243321	198	16	120000	0.38476	0.35021	TOP3A	76353	25kb
ENSG00000176994	17	18193624	18251517	127	11	120000	0.47553	0.3172	SMCR8	76353	25kb
ENSG00000176974	17	18206187	18291856	199	17	120000	0.15036	0.44024	SHMT1	76353	25kb
ENSG00000214860	17	18255976	18317961	78	11	120000	-0.065555	0.52613	EVPLL	76353	25kb
ENSG00000171916	17	18355051	18423259	6	3	120000	-1.0055	0.84268	LGALS9C	76353	25kb
ENSG00000213077	17	18402880	18455160	1	1	120000	-1.1703	0.87906	FAM106A	76353	25kb
ENSG00000189375	17	18513319	18590263	68	18	120000	-1.3597	0.91303	TBC1D28	76353	25kb
ENSG00000249459	17	18536742	18610575	93	20	120000	-1.4615	0.92807	ZNF286B	76353	25kb
ENSG00000108448	17	18576311	18664431	140	24	120000	-1.4388	0.9249	TRIM16L	76353	25kb
ENSG00000171931	17	18622326	18707662	143	22	120000	-1.4579	0.92756	FBXW10	76353	25kb
ENSG00000171928	17	18659308	18735027	111	16	120000	-1.1877	0.88253	TVP23B	76353	25kb
ENSG00000141127	17	18718398	18859581	345	22	120000	-0.68109	0.75209	PRPSAP2	76353	25kb
ENSG00000154025	17	18828658	18950908	227	53	120000	-0.41355	0.6604	SLC5A10	76353	25kb
ENSG00000188522	17	18847102	18933117	170	42	120000	-0.031723	0.51265	FAM83G	76353	25kb
ENSG00000154016	17	18898986	18975950	67	12	120000	0.67426	0.25007	GRAP	76353	25kb
ENSG00000072134	17	19093928	19265028	213	12	120000	0.86564	0.19335	EPN2	76353	25kb
ENSG00000230493	17	19097674	19150839	16	4	120000	1.15	0.12508	AC106017.1	76353	25kb
ENSG00000108641	17	19215867	19306495	144	12	120000	0.85435	0.19646	B9D1	76353	25kb
ENSG00000166484	17	19256034	19311857	79	12	120000	0.87907	0.18968	MAPK7	76353	25kb
ENSG00000166482	17	19261755	19315553	76	13	120000	1.0718	0.14191	MFAP4	76353	25kb
ENSG00000128482	17	19289438	19345589	83	9	120000	0.90184	0.18357	RNF112	76353	25kb
ENSG00000142494	17	19373698	19507347	273	36	120000	-0.53577	0.70394	SLC47A1	76353	25kb
ENSG00000072210	17	19526449	19605911	207	18	120000	0.79871	0.21223	ALDH3A2	76353	25kb
ENSG00000180638	17	19556601	19647292	223	27	120000	0.3488	0.36362	SLC47A2	76353	25kb
ENSG00000108602	17	19616297	19677256	128	21	120000	-0.211	0.58356	ALDH3A1	76353	25kb
ENSG00000083290	17	19649142	19796249	262	15	120000	-0.4937	0.68924	ULK2	76353	25kb
ENSG00000108599	17	19782615	19906656	199	18	120000	-1.5113	0.93465	AKAP10	76353	25kb
ENSG00000128487	17	19887657	20247339	750	21	120000	-0.092928	0.53702	SPECC1	76353	25kb
ENSG00000170298	17	20327708	20395852	27	5	120000	-0.46636	0.67952	LGALS9B	76353	25kb
ENSG00000214819	17	20458037	20509224	23	10	120000	-1.4008	0.91937	CDRT15L2	76353	25kb
ENSG00000205212	17	20714760	20824453	213	28	120000	2.1598	0.015393	CCDC144NL	76353	25kb
ENSG00000124422	17	20877910	20972073	288	16	120000	1.7415	0.040796	USP22	76353	25kb
ENSG00000109016	17	21001677	21121578	207	26	120000	-1.0808	0.86012	DHRS7B	76353	25kb
ENSG00000178307	17	21075593	21142937	153	24	120000	-0.69013	0.75494	TMEM11	76353	25kb
ENSG00000154035	17	21117183	21181722	181	35	120000	-0.67827	0.7512	C17orf103	76353	25kb
ENSG00000034152	17	21162984	21243552	97	20	120000	-0.11572	0.54606	MAP2K3	76353	25kb
ENSG00000184185	17	21254509	21348179	118	26	120000	-1.0038	0.84225	KCNJ12	76353	25kb
ENSG00000212719	17	21403051	21502722	122	21	120000	-1.3196	0.90651	C17orf51	76353	25kb

ENSG00000263563	17	21704601	21756762	131	11	120000	0.39511	0.34638	UBBP4	76353	25kb
ENSG00000256618	17	21997437	22048991	162	22	120000	0.46348	0.32151	MTRNR2L1	76353	25kb
ENSG00000109046	17	25596102	25665657	136	20	120000	-1.6374	0.94922	WSB1	76353	25kb
ENSG00000141068	17	25758670	25978461	475	44	120000	0.25504	0.39935	KSR1	76353	25kb
ENSG00000266728	17	25925390	25992803	154	13	120000	0.62692	0.26536	AC015688.3	76353	25kb
ENSG00000168961	17	25931824	26001586	165	14	120000	0.54822	0.29177	LGALS9	76353	25kb
ENSG00000007171	17	26058792	26152525	271	27	120000	-0.65096	0.74246	NOS2	76353	25kb
ENSG00000266202	17	26100832	26245391	269	27	120000	0.64211	0.2604	RP1-66C13.4	76353	25kb
ENSG00000232859	17	26180340	26246778	101	18	120000	0.85376	0.19662	LYRM9	76353	25kb
ENSG00000087095	17	26343763	26548407	113	38	120000	-0.91505	0.81992	NLK	76353	25kb
ENSG00000268206	17	26613667	26667323	64	14	120000	-0.98382	0.8374	AC061975.10	76353	25kb
ENSG00000109084	17	26621121	26680707	77	15	120000	-1.0847	0.86097	TMEM97	76353	25kb
ENSG00000109083	17	26630352	26687515	75	15	120000	-1.1624	0.87746	IFT20	76353	25kb
ENSG00000109079	17	26637628	26699035	90	14	120000	-0.8523	0.80298	TNFAIP1	76353	25kb
ENSG00000004142	17	26648659	26709545	92	12	120000	-0.76601	0.77817	POLDIP2	76353	25kb
ENSG00000244045	17	26659604	26733716	126	15	120000	-0.6311	0.73601	TMEM199	76353	25kb
ENSG00000109072	17	26666290	26725110	109	13	120000	-0.61245	0.72988	VTN	76353	25kb
ENSG00000273171	17	26666310	26720033	98	12	120000	-0.69668	0.757	CTB-96E2.2	76353	25kb
ENSG00000004139	17	26666378	26753065	153	21	120000	-0.75818	0.77583	SARM1	76353	25kb
ENSG00000076351	17	26696661	26759215	109	18	120000	-0.84186	0.80007	SLC46A1	76353	25kb
ENSG00000258472	17	26757770	26966215	285	37	120000	-2.2874	0.98892	RP11-192H23.4	76353	25kb
ENSG00000007216	17	26775311	26849799	154	17	120000	-1.7916	0.9634	SLC13A2	76353	25kb
ENSG00000109101	17	26808261	26890914	138	22	120000	-1.2939	0.90215	FOXN1	76353	25kb
ENSG00000109103	17	26848725	26904686	70	18	120000	-0.97137	0.83432	UNC119	76353	25kb
ENSG00000087111	17	26855401	26923890	67	19	120000	-1.0567	0.85467	PIGS	76353	25kb
ENSG00000109107	17	26875133	26929282	36	13	120000	-2.1168	0.98286	ALDOC	76353	25kb
ENSG00000076382	17	26879588	26951297	49	15	120000	-2.167	0.98488	SPAG5	76353	25kb
ENSG00000167524	17	26909982	26966218	40	17	120000	-2.304	0.98939	SGK494	76353	25kb
ENSG00000007202	17	26916458	26997472	62	21	120000	-1.8462	0.96757	KIAA0100	76353	25kb
ENSG00000132581	17	26950374	27014207	53	15	120000	-1.4407	0.92516	SDF2	76353	25kb
ENSG00000109111	17	26964109	27054697	81	16	120000	-1.2671	0.89743	SUPT6H	76353	25kb
ENSG00000167525	17	27005215	27063872	64	10	120000	-1.1133	0.86722	PROCA1	76353	25kb
ENSG00000109113	17	27016299	27070447	55	11	120000	-1.2285	0.89037	RAB34	76353	25kb
ENSG00000198242	17	27021411	27076377	57	13	120000	-1.2563	0.8955	RPL23A	76353	25kb
ENSG00000160606	17	27026366	27079953	64	14	120000	-1.4972	0.93283	TLCD1	76353	25kb
ENSG00000160602	17	27027915	27095473	80	15	120000	-1.5288	0.93685	NEK8	76353	25kb
ENSG00000076604	17	27046002	27102974	72	12	120000	-1.3049	0.90404	TRAF4	76353	25kb
ENSG00000173065	17	27057996	27207250	218	14	120000	-0.99453	0.84002	FAM222B	76353	25kb
ENSG00000132591	17	27156956	27213085	88	12	120000	-0.95391	0.82993	ERAL1	76353	25kb
ENSG00000132589	17	27181353	27249697	93	18	120000	-0.96428	0.83255	FLOT2	76353	25kb
ENSG00000167536	17	27199799	27255089	64	15	120000	-0.6601	0.74541	DHRS13	76353	25kb
ENSG00000109118	17	27207268	27303789	103	17	120000	-0.61185	0.72968	PHF12	76353	25kb
ENSG00000179761	17	27252531	27409234	251	47	120000	-0.89618	0.81492	PIPOX	76353	25kb
ENSG00000063015	17	27256919	27358458	146	29	120000	-0.54549	0.70729	SEZ6	76353	25kb
ENSG00000196535	17	27375528	27532430	306	23	120000	-0.70427	0.75937	MYO18A	76353	25kb
ENSG00000221995	17	27375537	27443537	144	19	120000	-0.88734	0.81255	TIAF1	76353	25kb
ENSG00000108255	17	27548881	27606512	134	18	120000	-0.24026	0.59494	CRYBA1	76353	25kb
ENSG00000108256	17	27557854	27646136	189	21	120000	-0.11709	0.5466	NUFIP2	76353	25kb
ENSG00000160551	17	27692482	27903922	373	20	120000	-1.0917	0.86251	TAOK1	76353	25kb
ENSG00000168792	17	27862565	27919155	87	23	120000	0.070316	0.47197	ABHD15	76353	25kb
ENSG00000167543	17	27868070	27925175	85	24	120000	0.52976	0.29814	TP53I13	76353	25kb
ENSG00000108262	17	27875487	27946072	102	27	120000	0.54812	0.29181	GIT1	76353	25kb
ENSG00000198720	17	27891787	27966779	102	23	120000	0.40841	0.34149	ANKRD13B	76353	25kb
ENSG00000167549	17	27916774	27974925	68	17	120000	0.026048	0.48961	CORO6	76353	25kb
ENSG00000141298	17	27927956	28282294	425	18	120000	-0.63744	0.73808	SSH2	76353	25kb
ENSG00000176927	17	28231218	28460470	288	14	120000	-0.21197	0.58394	EFCAB5	76353	25kb
ENSG00000126653	17	28417539	28538493	178	12	120000	-0.66751	0.74778	NSRP1	76353	25kb
ENSG00000108576	17	28496337	28588020	140	21	120000	0.005773	0.4977	SLC6A4	76353	25kb
ENSG00000108578	17	28550218	28644074	148	21	120000	0.51348	0.30381	BLMH	76353	25kb
ENSG00000182271	17	28618351	28686077	135	16	120000	0.19935	0.42099	TMIGD1	76353	25kb
ENSG00000108582	17	28680923	28822007	193	15	120000	1.1119	0.13309	CPD	76353	25kb
ENSG00000108587	17	28779380	28879610	156	16	120000	0.50031	0.30843	GOSR1	76353	25kb
ENSG00000266733	17	28859130	28915511	121	17	120000	-0.53834	0.70483	TBC1D29	76353	25kb
ENSG00000176390	17	29071406	29176794	342	7	120000	-0.43367	0.66774	CRLF3	76353	25kb
ENSG00000176208	17	29133988	29247887	259	8	120000	-0.63512	0.73732	ATAD5	76353	25kb
ENSG00000172171	17	29199354	29258838	113	7	120000	-0.93652	0.8255	TEFM	76353	25kb
ENSG00000184060	17	29208362	29311340	144	15	120000	-0.75574	0.7751	ADAP2	76353	25kb
ENSG00000181481	17	29270803	29351929	74	17	120000	-0.28326	0.61151	RNF135	76353	25kb
ENSG00000196712	17	29396945	29734134	455	13	120000	-0.14069	0.55594	NF1	76353	25kb
ENSG00000126861	17	29574031	29649557	104	9	120000	-0.25882	0.60211	OMG	76353	25kb
ENSG00000185862	17	29605784	29666130	73	7	120000	-0.35026	0.63693	EVI2B	76353	25kb
ENSG00000265118	17	29607231	29670849	77	7	120000	-0.35596	0.63907	CTD-2370N5.3	76353	25kb
ENSG00000126860	17	29619578	29673902	67	6	120000	-0.36686	0.64314	EVI2A	76353	25kb
ENSG00000131242	17	29693642	29890236	566	60	120000	0.34406	0.3654	RAB11FIP4	76353	25kb
ENSG00000228768	17	29873161	29927678	197	49	120000	1.8932	0.029162	AC003101.1	76353	25kb
ENSG00000172301	17	30153883	30211356	121	19	120000	-1.1602	0.87702	COPRS	76353	25kb
ENSG00000108651	17	30162923	30253784	209	21	120000	-1.1154	0.86765	UTP6	76353	25kb
ENSG00000178691	17	30239037	30353064	233	9	120000	-0.73598	0.76913	SUZ12	76353	25kb
ENSG00000185158	17	30309891	30405523	124	8	120000	-0.88027	0.81064	LRRC37B	76353	25kb
ENSG00000214708	17	30443245	30495154	81	6	120000	-1.1421	0.87329	AC090616.2	76353	25kb
ENSG00000126858	17	30444473	30605393	272	20	120000	-1.7005	0.95548	RHOT1	76353	25kb
ENSG00000269402	17	30481379	30531885	68	9	120000	-1.507	0.9341	AC116407.2	76353	25kb
ENSG00000141314	17	30568195	30676680	262	31	120000	-1.6187	0.94725	RHBDL3	76353	25kb
ENSG00000108666	17	30626584	30702042	146	17	120000	-1.1654	0.87806	C17orf75	76353	25kb
ENSG00000010244	17	30652136	30733905	105	15	120000	-1.8009	0.96414	ZNF207	76353	25kb
ENSG00000108671	17	30746279	30835336	104	17	120000	-1.6511	0.95064	PSMD11	76353	25kb
ENSG00000176749	17	30788637	30843274	75	16	120000	-1.5807	0.94303	CDK5R1	76353	25kb
ENSG00000176658	17	30794540	31229195	789	64	120000	-0.40899	0.65873	MYO1D	76353	25kb
ENSG00000006042	17	31229928	31297124	208	26	120000	1.087	0.13851	TMEM98	76353	25kb
ENSG00000141316	17	31272394	31349895	302	40	120000	0.35992	0.35945	SPACA3	76353	25kb
ENSG00000108684	17	31315105	32526983	3960	309	120000	-0.21216	0.58401	ASIC2	76353	25kb
ENSG00000268024	17	32556900	32607306	138	20	120000	2.0721	0.019128	AC005549.3	76353	25kb
ENSG00000108691	17	32557304	32609222	141	20	120000	2.0329	0.021029	CCL2	76353	25kb
ENSG00000108688	17	32572240	32624261	140	19	120000	2.2149	0.013383	CCL7	76353	25kb
ENSG00000172156	17	32587687	32640353	161	23	120000	2.218	0.013279	CCL11	76353	25kb
ENSG00000108700	17	32621055	32673421	162	18	120000	0.97938	0.1637	CCL8	76353	25kb

ENSG00000181374	17	32658471	32710629	216	34	120000	-0.31989	0.62547	CCL13	76353	25kb
ENSG00000108702	17	32662347	32715250	241	35	120000	-0.55085	0.70913	CCL1	76353	25kb
ENSG00000197322	17	32876142	32931388	179	34	120000	0.16526	0.43437	C17orf102	76353	25kb
ENSG00000181291	17	32882768	32991337	333	67	120000	-0.029981	0.51196	TMEM132E	76353	25kb
ENSG00000132141	17	33229878	33333097	135	27	120000	0.57475	0.28273	CCT6B	76353	25kb
ENSG00000198783	17	33263549	33315205	66	19	120000	0.55068	0.29093	ZNF830	76353	25kb
ENSG00000005156	17	33282513	33357083	84	18	120000	1.1331	0.12858	LIG3	76353	25kb
ENSG00000092871	17	33308009	33441338	133	21	120000	0.98993	0.1611	RFFL	76353	25kb
ENSG00000267618	17	33313368	33473512	166	29	120000	0.7263	0.23383	RAD51L3-RFFL	76353	25kb
ENSG00000185379	17	33401811	33473541	88	26	120000	0.44899	0.32672	RAD51D	76353	25kb
ENSG00000073598	17	33423598	33482751	84	28	120000	-0.11932	0.54749	FNDC8	76353	25kb
ENSG00000073536	17	33430772	33494334	105	27	120000	-0.33398	0.6308	NLE1	76353	25kb
ENSG00000141161	17	33449836	33541364	194	29	120000	-0.9669	0.8332	UNC45B	76353	25kb
ENSG00000164729	17	33494539	33546412	126	18	120000	-0.81461	0.79235	SLC35G3	76353	25kb
ENSG00000166750	17	33545055	33625674	231	33	120000	-1.6364	0.94912	SLFN5	76353	25kb
ENSG00000172716	17	33652324	33725720	228	31	120000	-0.30762	0.62081	SLFN11	76353	25kb
ENSG00000172123	17	33713079	33785302	216	35	120000	-0.078078	0.53112	SLFN12	76353	25kb
ENSG00000154760	17	33737115	33800856	199	35	120000	-0.40757	0.6582	SLFN13	76353	25kb
ENSG00000205045	17	33766268	33889880	399	60	120000	0.51911	0.30184	SLFN12L	76353	25kb
ENSG00000236320	17	33850144	33910117	199	32	120000	0.80073	0.21164	SLFN14	76353	25kb
ENSG00000108733	17	33876814	33930882	195	24	120000	0.62406	0.26629	PEX12	76353	25kb
ENSG00000006125	17	33880065	34078436	594	29	120000	0.81905	0.20638	AP2B1	76353	25kb
ENSG00000141150	17	34033668	34095540	175	31	120000	0.37131	0.35521	RASL10B	76353	25kb
ENSG00000132139	17	34046530	34104897	163	25	120000	0.29784	0.38291	GAS2L2	76353	25kb
ENSG00000129270	17	34058268	34147711	217	24	120000	-0.23636	0.59342	MMP28	76353	25kb
ENSG00000154768	17	34062916	34117098	143	18	120000	-0.00646	0.50258	C17orf50	76353	25kb
ENSG00000172660	17	34111459	34216619	236	21	120000	-0.84046	0.79968	TAF15	76353	25kb
ENSG00000172653	17	34156955	34220895	151	16	120000	-0.74937	0.77318	C17orf66	76353	25kb
ENSG00000161570	17	34173495	34232797	142	13	120000	-0.47933	0.68415	CCL5	76353	25kb
ENSG00000187456	17	34220070	34282777	237	15	120000	-0.94801	0.82844	RDM1	76353	25kb
ENSG00000161572	17	34236522	34295703	251	24	120000	-0.7097	0.76106	LYZL6	76353	25kb
ENSG00000161573	17	34278529	34333532	251	34	120000	0.55086	0.29086	CCL16	76353	25kb
ENSG00000213494	17	34285327	34354100	320	29	120000	0.56983	0.2844	CCL14	76353	25kb
ENSG00000266956	17	34285692	34342306	274	25	120000	0.74196	0.22906	CTB-186H2.3	76353	25kb
ENSG00000161574	17	34285692	34353660	317	29	120000	0.56992	0.28437	CCL15-CCL14	76353	25kb
ENSG00000267596	17	34298476	34354084	237	24	120000	0.41915	0.33755	CCL15	76353	25kb
ENSG00000167236	17	34315096	34370005	229	25	120000	0.19711	0.42187	CCL23	76353	25kb
ENSG00000006074	17	34366640	34424392	149	21	120000	0.22817	0.40976	CCL18	76353	25kb
ENSG00000006075	17	34390602	34442515	161	15	120000	0.31181	0.37759	CCL3	76353	25kb
ENSG00000129277	17	34405983	34458014	197	24	120000	0.88979	0.18679	CCL4	76353	25kb
ENSG00000224226	17	34468061	34528996	59	19	120000	-1.7834	0.96274	TBC1D3B	76353	25kb
ENSG00000256515	17	34497268	34549157	1	1	120000	1.6448	0.050008	CCL3L3	76353	25kb
ENSG00000205020	17	34513310	34565280	1	1	120000	1.6448	0.050008	CCL4L1	76353	25kb
ENSG00000242384	17	34556086	34833061	50	13	120000	-0.5176	0.69763	TBC1D3H	76353	25kb
ENSG00000161583	17	34720936	34833104	50	13	120000	-0.5176	0.69763	TBC1D3G	76353	25kb
ENSG00000108278	17	34817473	34880154	119	22	120000	-0.55775	0.71149	ZNHIT3	76353	25kb
ENSG00000141140	17	34826477	34924284	174	18	120000	-0.23069	0.59122	MYO19	76353	25kb
ENSG00000184886	17	34865847	34920159	106	12	120000	-0.14624	0.55813	PIGW	76353	25kb
ENSG00000005955	17	34875737	34971278	193	19	120000	0.22094	0.41257	GGNBP2	76353	25kb
ENSG00000108272	17	34923228	34982235	142	18	120000	0.39654	0.34585	DHRS11	76353	25kb
ENSG00000129282	17	34933001	34990407	138	18	120000	0.35752	0.36035	MRM1	76353	25kb
ENSG00000132130	17	35269084	35326917	119	30	120000	0.83164	0.20281	LHX1	76353	25kb
ENSG00000108270	17	35281175	35439171	241	32	120000	0.87345	0.19121	AATF	76353	25kb
ENSG00000132142	17	35416923	35791909	591	40	120000	-0.3541	0.63837	ACACA	76353	25kb
ENSG00000167230	17	35707928	35774662	124	27	120000	-0.66333	0.74644	C17orf78	76353	25kb
ENSG00000108264	17	35741965	35864835	294	19	120000	-0.50153	0.692	TADA2A	76353	25kb
ENSG00000161326	17	35824937	35898603	177	24	120000	-0.21306	0.58436	DUSP14	76353	25kb
ENSG00000006114	17	35849900	35994544	232	33	120000	-0.39864	0.65492	SYNRG	76353	25kb
ENSG00000141141	17	35944787	36028493	115	21	120000	-0.040681	0.51622	DDX52	76353	25kb
ENSG00000108753	17	36021435	36130237	318	76	120000	-0.91537	0.82	HNF1B	76353	25kb
ENSG00000185128	17	36258971	36319915	3	2	120000	-0.80655	0.79004	TBC1D3F	76353	25kb
ENSG00000174093	17	36312717	36446858	57	8	120000	0.90101	0.18379	RP11-1407O15.2	76353	25kb
ENSG00000174100	17	36427989	36504101	119	16	120000	0.66378	0.25342	MRPL45	76353	25kb
ENSG00000188888	17	36456413	36524730	111	20	120000	0.063692	0.47461	GPR179	76353	25kb
ENSG00000174111	17	36483111	36581019	208	24	120000	-0.70815	0.76057	SOCS7	76353	25kb
ENSG00000225485	17	36559662	36693628	405	55	120000	-0.50334	0.69264	ARHGAP23	76353	25kb
ENSG00000017373	17	36661251	36787183	274	33	120000	-1.3701	0.91466	SRCIN1	76353	25kb
ENSG00000179294	17	36802961	36856187	84	16	120000	-1.4379	0.92477	C17orf96	76353	25kb
ENSG00000108292	17	36836795	36911056	113	25	120000	-1.4141	0.92134	MLLT6	76353	25kb
ENSG00000269094	17	36859086	36909451	86	22	120000	-1.2368	0.89192	AC006449.1	76353	25kb
ENSG00000230055	17	36861488	36916297	100	26	120000	-1.3037	0.90384	CISD3	76353	25kb
ENSG00000056661	17	36865150	36931070	128	30	120000	-1.6864	0.95414	PCGF2	76353	25kb
ENSG00000227450	17	36880613	36931969	115	26	120000	-1.7656	0.96127	CTB-58E17.5	76353	25kb
ENSG00000108294	17	36883989	36945484	138	30	120000	-1.7784	0.96233	PSMB3	76353	25kb
ENSG00000141720	17	36896942	36981379	196	32	120000	-1.5437	0.93867	PIP4K2B	76353	25kb
ENSG00000108296	17	36931687	37006734	194	28	120000	-1.0331	0.84921	CWC25	76353	25kb
ENSG00000214556	17	36966341	37022708	149	26	120000	-0.36564	0.64268	C17orf98	76353	25kb
ENSG00000125691	17	36979118	37035096	153	25	120000	-0.20432	0.58095	RPL23	76353	25kb
ENSG00000002834	17	37001112	37103023	253	41	120000	-0.45438	0.67522	LASP1	76353	25kb
ENSG00000204952	17	37067685	37148655	116	19	120000	0.45198	0.32564	FBXO47	76353	25kb
ENSG00000161381	17	37194556	37335647	487	43	120000	-0.69622	0.75685	PLXDC1	76353	25kb
ENSG00000141748	17	37288147	37348737	190	28	120000	-0.34837	0.63622	ARL5C	76353	25kb
ENSG00000067191	17	37304709	37378956	204	39	120000	0.068403	0.47273	CACNB1	76353	25kb
ENSG00000108298	17	37331536	37385980	104	29	120000	1.0224	0.15329	RPL19	76353	25kb
ENSG00000141750	17	37341789	37407125	118	27	120000	0.63617	0.26233	STAC2	76353	25kb
ENSG00000108306	17	37390384	37583776	373	12	120000	0.035069	0.48601	FBXL20	76353	25kb
ENSG00000125686	17	37535538	37632539	181	13	120000	-0.097668	0.5389	MED1	76353	25kb
ENSG00000167258	17	37592764	37746160	307	16	120000	-0.019642	0.50784	CDK12	76353	25kb
ENSG00000171532	17	37734789	37791030	129	18	120000	0.40596	0.34239	NEUROD2	76353	25kb
ENSG00000131771	17	37757993	37817879	143	19	120000	0.13715	0.44546	PPP1R1B	76353	25kb
ENSG00000131748	17	37768318	37844737	208	21	120000	-0.5921	0.72311	STARD3	76353	25kb
ENSG00000173991	17	37795440	37847808	140	13	120000	-0.97584	0.83543	TCAP	76353	25kb
ENSG00000141744	17	37799234	37851728	137	13	120000	-1.0568	0.8547	PNMT	76353	25kb
ENSG00000161395	17	37802375	37878050	164	15	120000	-1.1893	0.88284	PGAP3	76353	25kb
ENSG00000141736	17	37819167	37911679	157	16	120000	-0.20941	0.58294	ERBB2	76353	25kb
ENSG00000141741	17	37859749	37912040	67	15	120000	1.1427	0.12658	MIEN1	76353	25kb

ENSG00000141738	17	37869180	37928544	71	12	120000	1.644	0.05009	GRB7	76353	25kb
ENSG00000161405	17	37896198	38045441	316	9	120000	1.79	0.036727	IKZF3	76353	25kb
ENSG00000186075	17	37999417	38059149	140	9	120000	1.3741	0.084699	ZPBP2	76353	25kb
ENSG00000073605	17	38035848	38101107	149	13	120000	-0.022242	0.50887	GSDMB	76353	25kb
ENSG00000172057	17	38052294	38108854	148	15	120000	-0.37703	0.64692	ORMDL3	76353	25kb
ENSG00000204913	17	38072727	38126000	170	15	120000	-0.86611	0.80679	LRRC3C	76353	25kb
ENSG00000167914	17	38094226	38159019	223	21	120000	-1.194	0.88376	GSDMA	76353	25kb
ENSG00000108344	17	38112050	38179213	245	20	120000	-0.9196	0.82111	PSMD3	76353	25kb
ENSG00000108342	17	38146614	38199066	161	13	120000	-0.36902	0.64394	CSF3	76353	25kb
ENSG00000008838	17	38150350	38242468	210	19	120000	-0.22408	0.58865	MED24	76353	25kb
ENSG00000126351	17	38189543	38275120	142	29	120000	-0.028383	0.51132	THRA	76353	25kb
ENSG00000126368	17	38224040	38281978	93	24	120000	-0.26533	0.60462	NR1D1	76353	25kb
ENSG00000188895	17	38253551	38318042	113	15	120000	-0.86302	0.80594	MSL1	76353	25kb
ENSG00000108349	17	38271571	38353436	131	14	120000	-1.1761	0.88023	CASC3	76353	25kb
ENSG00000108352	17	38308263	38376908	124	18	120000	-1.4709	0.92934	RAPGEFL1	76353	25kb
ENSG00000171475	17	38350556	38465388	189	17	120000	-1.0214	0.84646	WIPF2	76353	25kb
ENSG00000094804	17	38418885	38484171	80	10	120000	-0.76571	0.77808	CDC6	76353	25kb
ENSG00000131759	17	38440444	38538094	160	15	120000	-0.76044	0.7765	RARA	76353	25kb
ENSG00000266208	17	38491907	38545945	116	12	120000	-0.86171	0.80558	CTD-2267D19.3	76353	25kb
ENSG00000183153	17	38492235	38545067	112	12	120000	-0.83755	0.79886	GJD3	76353	25kb
ENSG00000131747	17	38519768	38599202	163	15	120000	-1.1731	0.87962	TOP2A	76353	25kb
ENSG00000141753	17	38574702	38638983	152	27	120000	-1.2767	0.89915	IGFBP4	76353	25kb
ENSG00000131746	17	38607080	38682849	205	36	120000	-1.3694	0.91457	TNS4	76353	25kb
ENSG00000126353	17	38685021	38746724	106	29	120000	-1.0709	0.85789	CCR7	76353	25kb
ENSG00000073584	17	38756214	38829760	156	19	120000	-1.4689	0.92907	SMARCE1	76353	25kb
ENSG00000264058	17	38760049	38846393	207	20	120000	-1.4085	0.92051	KRT222	76353	25kb
ENSG00000213424	17	38785917	38846433	139	14	120000	-1.2705	0.89804	KRT222	76353	25kb
ENSG00000167916	17	38829243	38885002	178	20	120000	-1.3836	0.91676	KRT24	76353	25kb
ENSG00000204897	17	38879273	38936584	160	25	120000	-1.9839	0.97636	KRT25	76353	25kb
ENSG00000186393	17	38897490	38953414	146	24	120000	-2.1005	0.98216	KRT26	76353	25kb
ENSG00000171446	17	38908060	38963786	157	26	120000	-1.9336	0.97342	KRT27	76353	25kb
ENSG00000173908	17	38923455	38981211	163	28	120000	-1.7859	0.96294	KRT28	76353	25kb
ENSG00000186395	17	38949369	39003847	156	22	120000	-1.3244	0.90732	KRT10	76353	25kb
ENSG00000167920	17	38950358	39017522	193	24	120000	-1.314	0.90557	TMEM99	76353	25kb
ENSG00000187242	17	38992555	39048462	159	23	120000	-1.6662	0.95217	KRT12	76353	25kb
ENSG00000171431	17	39007193	39066479	171	30	120000	-0.72895	0.76698	KRT20	76353	25kb
ENSG00000108244	17	39053948	39118886	252	24	120000	0.030885	0.48768	KRT23	76353	25kb
ENSG00000196859	17	39089669	39148144	294	17	120000	-0.36312	0.64174	KRT39	76353	25kb
ENSG00000204889	17	39108968	39168387	341	19	120000	-0.87623	0.80955	KRT40	76353	25kb
ENSG00000212899	17	39124686	39175385	312	17	120000	-0.96327	0.83229	KRTAP3-3	76353	25kb
ENSG00000212900	17	39130447	39181138	302	17	120000	-1.0077	0.84321	KRTAP3-2	76353	25kb
ENSG00000212901	17	39139773	39200576	311	19	120000	-0.99302	0.83965	KRTAP3-1	76353	25kb
ENSG00000221852	17	39157278	39208454	226	21	120000	-0.80149	0.78858	KRTAP1-5	76353	25kb
ENSG00000204887	17	39160949	39211356	206	20	120000	-0.78457	0.78365	KRTAP1-4	76353	25kb
ENSG00000221880	17	39165142	39216107	184	20	120000	-0.7232	0.76522	KRTAP1-3	76353	25kb
ENSG00000188581	17	39171811	39222713	210	22	120000	-0.4323	0.66724	KRTAP1-1	76353	25kb
ENSG00000212725	17	39177793	39228568	205	22	120000	-0.3489	0.63642	KRTAP2-1	76353	25kb
ENSG00000214518	17	39185750	39236482	199	22	120000	-0.40093	0.65576	KRTAP2-2	76353	25kb
ENSG00000212724	17	39190495	39241344	194	19	120000	-0.34339	0.63435	KRTAP2-3	76353	25kb
ENSG00000213417	17	39196369	39247131	216	17	120000	-0.2064	0.58176	KRTAP2-4	76353	25kb
ENSG00000240871	17	39215459	39266396	292	11	120000	0.84087	0.20021	KRTAP4-7	76353	25kb
ENSG00000204880	17	39228233	39279393	328	9	120000	1.2421	0.1071	KRTAP4-8	76353	25kb
ENSG00000241241	17	39232754	39283461	339	10	120000	1.2824	0.099847	KRTAP4-16P	76353	25kb
ENSG00000212722	17	39236584	39287740	325	10	120000	1.3361	0.09076	KRTAP4-9	76353	25kb
ENSG00000212721	17	39248433	39299606	282	9	120000	1.6111	0.053578	KRTAP4-11	76353	25kb
ENSG00000213416	17	39254343	39305419	249	9	120000	1.683	0.046189	KRTAP4-12	76353	25kb
ENSG00000198090	17	39270685	39321739	159	12	120000	1.7097	0.043658	KRTAP4-6	76353	25kb
ENSG00000198271	17	39280178	39331054	123	17	120000	1.2536	0.10499	KRTAP4-5	76353	25kb
ENSG00000171396	17	39290910	39341983	151	12	120000	0.36934	0.35594	KRTAP4-4	76353	25kb
ENSG00000196156	17	39298484	39349424	139	10	120000	0.16858	0.43306	KRTAP4-3	76353	25kb
ENSG00000244537	17	39308698	39359460	132	8	120000	0.029071	0.4884	KRTAP4-2	76353	25kb
ENSG00000198443	17	39315354	39366594	131	8	120000	-0.05928	0.52364	KRTAP4-1	76353	25kb
ENSG00000240542	17	39319697	39371891	143	8	120000	-0.073717	0.52938	KRTAP9-1	76353	25kb
ENSG00000239886	17	39357900	39408904	109	11	120000	-1.0565	0.85463	KRTAP9-2	76353	25kb
ENSG00000204873	17	39363715	39414705	125	11	120000	-1.0833	0.86065	KRTAP9-3	76353	25kb
ENSG00000187272	17	39369297	39420256	118	10	120000	-1.0885	0.86182	KRTAP9-8	76353	25kb
ENSG00000241595	17	39380939	39431904	91	7	120000	-0.74124	0.77073	KRTAP9-4	76353	25kb
ENSG00000198083	17	39386636	39437616	89	6	120000	-0.68877	0.75452	KRTAP9-9	76353	25kb
ENSG00000212659	17	39396591	39447893	107	7	120000	-0.5956	0.72428	KRTAP9-6	76353	25kb
ENSG00000180386	17	39406911	39457949	119	8	120000	-0.39665	0.65419	KRTAP9-7	76353	25kb
ENSG00000212658	17	39433078	39484103	171	10	120000	-0.34853	0.63628	KRTAP29-1	76353	25kb
ENSG00000212657	17	39438952	39490505	179	11	120000	-0.30218	0.61874	KRTAP16-1	76353	25kb
ENSG00000186860	17	39446173	39496947	163	12	120000	-0.27269	0.60745	KRTAP17-1	76353	25kb
ENSG00000006059	17	39477344	39532064	108	16	120000	-0.52893	0.70157	KRT33A	76353	25kb
ENSG00000131738	17	39494746	39551052	108	15	120000	-0.61261	0.72993	KRT33B	76353	25kb
ENSG00000131737	17	39508902	39563655	114	15	120000	-0.5537	0.71011	KRT34	76353	25kb
ENSG00000094796	17	39524976	39578844	127	18	120000	-0.77385	0.78049	KRT31	76353	25kb
ENSG00000108417	17	39552005	39605775	198	19	120000	-0.83237	0.7974	KRT37	76353	25kb
ENSG00000171360	17	39567698	39622173	269	15	120000	-0.6317	0.73621	KRT38	76353	25kb
ENSG00000108759	17	39591063	39648681	315	15	120000	-0.78115	0.78264	KRT32	76353	25kb
ENSG00000197079	17	39607941	39662392	270	19	120000	-0.53421	0.7034	KRT35	76353	25kb
ENSG00000126337	17	39617388	39673798	231	23	120000	-0.80666	0.79007	KRT36	76353	25kb
ENSG00000171401	17	39632233	39686957	167	29	120000	-0.95811	0.831	KRT13	76353	25kb
ENSG00000171346	17	39644995	39703781	150	23	120000	-0.62064	0.73258	KRT15	76353	25kb
ENSG00000171345	17	39654869	39709560	126	17	120000	-1.1381	0.87246	KRT19	76353	25kb
ENSG00000171403	17	39697096	39753310	126	10	120000	-0.60146	0.72623	KRT9	76353	25kb
ENSG00000186847	17	39713531	39768173	148	7	120000	-0.56428	0.71372	KRT14	76353	25kb
ENSG00000186832	17	39741030	39797151	146	10	120000	-1.0931	0.86283	KRT16	76353	25kb
ENSG00000128422	17	39750689	39806094	125	15	120000	-1.2647	0.89701	KRT17	76353	25kb
ENSG00000173801	17	39750692	39968183	661	50	120000	-0.74475	0.77179	JUP	76353	25kb
ENSG00000173812	17	39820137	39873920	203	11	120000	-0.1355	0.55389	EIF1	76353	25kb
ENSG00000184502	17	39843578	39897221	193	19	120000	-0.43585	0.66853	GAST	76353	25kb
ENSG00000173805	17	39848994	39915896	239	27	120000	-0.78396	0.78347	HAP1	76353	25kb
ENSG00000141696	17	39933199	39993856	179	21	120000	-0.2947	0.61589	LEPREL4	76353	25kb
ENSG00000141756	17	39943932	40004465	173	16	120000	-0.4869	0.68684	FKBP10	76353	25kb
ENSG00000141698	17	39956335	40017523	176	14	120000	-0.84156	0.79998	NT5C3B	76353	25kb

ENSG00000161594	17	39966937	40029636	173	11	120000	-0.99631	0.84045	KLHL10	76353	25kb
ENSG00000178502	17	39984797	40046684	145	10	120000	-1.0396	0.85074	KLHL11	76353	25kb
ENSG00000131473	17	39998161	40111795	219	17	120000	-0.94941	0.82879	ACLY	76353	25kb
ENSG00000173786	17	40093759	40154749	102	18	120000	-0.27455	0.60817	CNP	76353	25kb
ENSG00000168259	17	40103451	40198394	138	23	120000	-0.31957	0.62535	DNAJC7	76353	25kb
ENSG00000168256	17	40138400	40202659	83	13	120000	0.17072	0.43222	NKIRAS2	76353	25kb
ENSG00000187595	17	40152594	40242383	140	20	120000	0.24626	0.40274	ZNF385C	76353	25kb
ENSG00000108771	17	40228422	40289751	112	15	120000	0.60527	0.2725	DHX58	76353	25kb
ENSG00000108773	17	40240126	40298376	115	11	120000	0.65147	0.25737	KAT2A	76353	25kb
ENSG00000267261	17	40246692	40331934	154	17	120000	0.77844	0.21815	CTD-2132N18.3	76353	25kb
ENSG00000197723	17	40249756	40300371	102	9	120000	0.53515	0.29627	HSPB9	76353	25kb
ENSG00000108774	17	40251994	40332035	146	16	120000	0.76136	0.22322	RAB5C	76353	25kb
ENSG00000089558	17	40283909	40358296	101	18	120000	1.0319	0.15105	KCNH4	76353	25kb
ENSG00000161610	17	40311078	40362470	42	15	120000	0.67926	0.24849	HCRT	76353	25kb
ENSG00000167925	17	40315817	40371531	43	21	120000	0.68202	0.24761	GHDC	76353	25kb
ENSG00000173757	17	40326186	40453725	131	18	120000	0.85972	0.19497	STAT5B	76353	25kb
ENSG00000126561	17	40414565	40488961	110	11	120000	0.52437	0.30001	STAT5A	76353	25kb
ENSG00000168610	17	40440342	40565586	263	21	120000	2.0667	0.019381	STAT3	76353	25kb
ENSG00000177469	17	40529470	40600535	189	22	120000	2.7996	0.0025585	PTRF	76353	25kb
ENSG00000033627	17	40585862	40699629	177	23	120000	2.1509	0.015742	ATP6V0A1	76353	25kb
ENSG00000108784	17	40663190	40721467	98	16	120000	2.4446	0.0072514	NAGLU	76353	25kb
ENSG00000108786	17	40676232	40732231	113	16	120000	2.6667	0.0038303	HSD17B1	76353	25kb
ENSG00000068120	17	40688485	40743295	105	13	120000	2.7213	0.003251	COASY	76353	25kb
ENSG00000108788	17	40694086	40750257	108	12	120000	2.6636	0.0038659	MLX	76353	25kb
ENSG00000131470	17	40699333	40754849	101	11	120000	2.754	0.0029432	PSMC3IP	76353	25kb
ENSG00000141699	17	40706531	40787641	143	12	120000	2.7389	0.0030819	FAM134C	76353	25kb
ENSG00000131462	17	40736694	40792252	93	9	120000	2.6073	0.0045631	TUBG1	76353	25kb
ENSG00000037042	17	40786323	40844024	94	11	120000	2.7648	0.0028475	TUBG2	76353	25kb
ENSG00000068137	17	40794932	40854048	83	10	120000	2.9381	0.0016509	PLEKHH3	76353	25kb
ENSG00000184451	17	40805907	40860935	58	8	120000	2.9964	0.0013661	CCR10	76353	25kb
ENSG00000108797	17	40809631	40876832	65	10	120000	3.2505	0.00057609	CNTNAP1	76353	25kb
ENSG00000108799	17	40827293	40922071	88	12	120000	3.2704	0.00053702	EZH1	76353	25kb
ENSG00000131477	17	40885465	40940059	41	12	120000	2.889	0.0019321	RAMP2	76353	25kb
ENSG00000131475	17	40900454	40956617	38	15	120000	2.418	0.0078035	VPS25	76353	25kb
ENSG00000126562	17	40907696	40973954	38	17	120000	1.0265	0.15232	WNK4	76353	25kb
ENSG00000183978	17	40922165	40975722	24	13	120000	0.16051	0.43624	COA3	76353	25kb
ENSG00000176563	17	40925810	40988605	24	14	120000	0.31193	0.37755	CNTD1	76353	25kb
ENSG00000126581	17	40937152	41010367	35	18	120000	0.92952	0.17631	BECN1	76353	25kb
ENSG00000131467	17	40951402	41020774	29	17	120000	0.55526	0.28936	PSME3	76353	25kb
ENSG00000131480	17	40971617	41027724	29	15	120000	1.2691	0.1022	AOC2	76353	25kb
ENSG00000131471	17	40978201	41035147	32	16	120000	1.2728	0.10155	AOC3	76353	25kb
ENSG00000131482	17	41027814	41090386	89	20	120000	-0.76574	0.77808	G6PC	76353	25kb
ENSG00000266967	17	41077543	41141515	61	10	120000	-0.45193	0.67434	AARSD1	76353	25kb
ENSG00000108825	17	41077543	41157545	86	12	120000	-0.49891	0.69108	PTGES3L-AARSD1	76353	25kb
ENSG00000267060	17	41095105	41157545	68	8	120000	-0.18339	0.57275	PTGES3L	76353	25kb
ENSG00000198863	17	41107582	41170707	77	10	120000	-0.25288	0.59982	RUNDC1	76353	25kb
ENSG00000131469	17	41125290	41179976	73	10	120000	-0.22907	0.59059	RPL27	76353	25kb
ENSG00000068079	17	41133742	41191473	87	11	120000	-0.20402	0.58083	IFI35	76353	25kb
ENSG00000108828	17	41141622	41202140	93	10	120000	-0.1405	0.55587	VAT1	76353	25kb
ENSG00000108830	17	41152258	41209057	94	9	120000	-0.060276	0.52403	RND2	76353	25kb
ENSG00000012048	17	41171312	41302500	257	7	120000	0.0040183	0.4984	BRCA1	76353	25kb
ENSG00000188554	17	41297498	41388708	171	7	120000	0.09928	0.46046	NBR1	76353	25kb
ENSG00000184988	17	41338854	41397061	101	6	120000	-0.023132	0.50923	TMEM106A	76353	25kb
ENSG00000175906	17	41451327	41503505	117	16	120000	0.44013	0.32992	ARL4D	76353	25kb
ENSG00000067596	17	41536233	41646831	183	11	120000	1.3889	0.082425	DHX8	76353	25kb
ENSG00000175832	17	41580212	41681988	198	15	120000	1.0014	0.15831	ETV4	76353	25kb
ENSG00000005102	17	41692756	41764322	198	28	120000	-0.86877	0.80751	MEOX1	76353	25kb
ENSG00000167941	17	41806099	41861156	138	18	120000	-0.77387	0.7805	SOST	76353	25kb
ENSG00000108861	17	41818489	41881356	136	16	120000	-0.53515	0.70373	DUSP3	76353	25kb
ENSG00000231256	17	41832803	41889980	113	12	120000	-0.45109	0.67404	C17orf105	76353	25kb
ENSG00000161647	17	41853167	41935538	132	12	120000	-0.74338	0.77137	MPP3	76353	25kb
ENSG00000161649	17	41899516	41965997	124	13	120000	0.41532	0.33895	CD300LG	76353	25kb
ENSG00000108852	17	41927725	42012068	244	24	120000	1.7631	0.038939	MPP2	76353	25kb
ENSG00000267496	17	41969576	42020326	176	19	120000	1.7621	0.039027	FAM215A	76353	25kb
ENSG00000261514	17	41990731	42041327	157	20	120000	1.2479	0.10603	RP11-527L4.2	76353	25kb
ENSG00000108849	17	41993172	42044836	149	20	120000	1.2532	0.10507	PPY	76353	25kb
ENSG00000131096	17	42005106	42106837	252	27	120000	0.24039	0.40501	PYY	76353	25kb
ENSG00000161653	17	42056914	42111431	134	19	120000	0.11652	0.45362	NAGS	76353	25kb
ENSG00000091947	17	42063556	42126314	136	18	120000	0.15249	0.4394	TMEM101	76353	25kb
ENSG00000161654	17	42087003	42169987	145	20	120000	0.17662	0.4299	LSM12	76353	25kb
ENSG00000141349	17	42123103	42178709	88	16	120000	0.25638	0.39883	G6PC3	76353	25kb
ENSG00000108840	17	42129114	42226070	161	20	120000	-0.063419	0.52528	HDAC5	76353	25kb
ENSG00000125319	17	42194274	42264844	122	13	120000	-0.6626	0.74621	C17orf53	76353	25kb
ENSG00000161664	17	42222815	42281451	106	13	120000	-0.77714	0.78146	ASB16	76353	25kb
ENSG00000168591	17	42239338	42294099	102	15	120000	-0.73583	0.76908	TMUB2	76353	25kb
ENSG00000087152	17	42244173	42302481	105	16	120000	-0.67236	0.74932	ATXN7L3	76353	25kb
ENSG00000108312	17	42257401	42323994	115	17	120000	-0.54426	0.70687	UBTF	76353	25kb
ENSG00000004939	17	42300753	42370509	153	32	120000	-0.73478	0.76876	SLC4A1	76353	25kb
ENSG00000214921	17	42300758	42352236	106	22	120000	-0.61425	0.73048	AC003102.1	76353	25kb
ENSG00000268413	17	42310297	42380825	173	39	120000	-1.2101	0.88688	AC003043.1	76353	25kb
ENSG00000108309	17	42360781	42421039	153	24	120000	-1.308	0.90457	RUNDC3A	76353	25kb
ENSG00000013306	17	42371993	42427238	131	20	120000	-1.2272	0.89012	SLC25A39	76353	25kb
ENSG00000030582	17	42397614	42455470	109	23	120000	-0.77347	0.78038	GRN	76353	25kb
ENSG00000161682	17	42405583	42466243	94	21	120000	-0.3889	0.65132	FAM171A2	76353	25kb
ENSG00000005961	17	42424548	42491873	84	17	120000	-0.58227	0.71981	ITGA2B	76353	25kb
ENSG00000186566	17	42447652	42605798	230	17	120000	-0.56367	0.71351	GPATCH8	76353	25kb
ENSG00000180340	17	42609925	42661907	140	17	120000	2.6948	0.0035214	FZD2	76353	25kb
ENSG00000180336	17	42708762	42792676	157	20	120000	0.2241	0.41134	C17orf104	76353	25kb
ENSG00000180329	17	42725437	42792147	103	21	120000	0.53704	0.29562	CCDC43	76353	25kb
ENSG00000161692	17	42760976	42854632	167	25	120000	0.49068	0.31183	DBF4B	76353	25kb
ENSG00000073670	17	42811399	42884214	132	20	120000	0.75989	0.22366	ADAM11	76353	25kb
ENSG00000182963	17	42850816	42933184	172	23	120000	-0.39342	0.653	GJC1	76353	25kb
ENSG00000131097	17	42898721	42952848	125	18	120000	-0.63591	0.73758	HIGD1B	76353	25kb
ENSG00000108883	17	42902311	43002030	276	22	120000	-0.36725	0.64328	EFTUD2	76353	25kb
ENSG00000167131	17	42951510	43007758	173	25	120000	-0.16366	0.565	CCDC103	76353	25kb
ENSG00000214447	17	42952135	43007758	173	25	120000	-0.16366	0.565	FAM187A	76353	25kb

ENSG00000131095	17	42957376	43019305	184	28	120000	-0.19745	0.57826	GFAP	76353	25kb
ENSG00000186185	17	42977077	43050082	230	34	120000	-0.1333	0.55302	KIF18B	76353	25kb
ENSG00000131094	17	43012061	43070439	176	31	120000	-0.16874	0.567	C1QL1	76353	25kb
ENSG00000172992	17	43075708	43163473	234	26	120000	-0.2434	0.59615	DCAKD	76353	25kb
ENSG00000136448	17	43103978	43211384	299	25	120000	-0.38878	0.65128	NMT1	76353	25kb
ENSG00000161714	17	43161335	43235721	176	32	120000	-0.3165	0.62419	PLCD3	76353	25kb
ENSG00000181513	17	43184967	43246548	128	28	120000	-0.47203	0.68155	ACBD4	76353	25kb
ENSG00000186834	17	43199684	43254468	101	21	120000	-0.74495	0.77185	HEXIM1	76353	25kb
ENSG00000168517	17	43213067	43272407	96	19	120000	-1.1252	0.86974	HEXIM2	76353	25kb
ENSG00000184922	17	43273811	43349687	166	31	120000	0.44759	0.32723	FMNL1	76353	25kb
ENSG00000184361	17	43306760	43364479	120	19	120000	0.29595	0.38364	SPATA32	76353	25kb
ENSG00000159314	17	43446275	43536787	200	14	120000	-0.75402	0.77458	ARHGAP27	76353	25kb
ENSG00000225190	17	43488266	43593115	176	9	120000	-0.34678	0.63562	PLEKHM1	76353	25kb
ENSG00000120088	17	43674267	43938194	1165	11	120000	0.10257	0.45915	CRHR1	76353	25kb
ENSG00000185294	17	43897256	43949438	336	6	120000	0.060066	0.47605	SPPL2C	76353	25kb
ENSG00000186868	17	43946748	44130700	932	6	120000	-0.087795	0.53498	MAPT	76353	25kb
ENSG00000256762	17	44051616	44102060	261	5	120000	-0.08257	0.5329	STH	76353	25kb
ENSG00000120071	17	44082282	44327733	877	6	120000	0.074165	0.47044	KANSL1	76353	25kb
ENSG00000228696	17	44327150	44464130	56	5	120000	-0.19312	0.57657	ARL17B	76353	25kb
ENSG00000176681	17	44345099	44440160	40	4	120000	-0.14845	0.55901	LRRC37A	76353	25kb
ENSG00000238083	17	44563877	44658016	6	2	120000	-0.0092491	0.50369	LRRC37A2	76353	25kb
ENSG00000185829	17	44569068	44682088	5	2	120000	-0.44757	0.67277	ARL17A	76353	25kb
ENSG00000073969	17	44643035	44859830	131	12	120000	-0.61978	0.7323	NSF	76353	25kb
ENSG00000108379	17	44814872	44935520	244	39	120000	-1.6046	0.94571	WNT3	76353	25kb
ENSG00000158955	17	44885567	44989096	226	37	120000	-1.591	0.9442	WNT9B	76353	25kb
ENSG00000108433	17	44975483	45130003	428	27	120000	0.55545	0.28929	GOSR2	76353	25kb
ENSG00000262633	17	44975499	45149520	493	28	120000	0.62993	0.26437	RP11-156P1.2	76353	25kb
ENSG00000179673	17	45030523	45081614	199	17	120000	0.72865	0.23311	RPRML	76353	25kb
ENSG00000004897	17	45170069	45291788	275	25	120000	-0.081015	0.53228	CDC27	76353	25kb
ENSG00000198336	17	45252812	45326045	215	39	120000	-1.1442	0.87372	MYL4	76353	25kb
ENSG00000259207	17	45306212	45446658	496	39	120000	-1.0692	0.85752	ITGB3	76353	25kb
ENSG00000259753	17	45306263	45446658	496	39	120000	-1.0692	0.85752	ITGB3	76353	25kb
ENSG00000178852	17	45375656	45543678	445	24	120000	-0.38431	0.64962	EFCAB13	76353	25kb
ENSG00000141279	17	45575308	45725642	208	19	120000	0.29447	0.3842	NPEPPS	76353	25kb
ENSG00000108424	17	45701842	45787871	151	19	120000	0.34803	0.36391	KPNB1	76353	25kb
ENSG00000198933	17	45746447	45814416	127	24	120000	-0.090902	0.53621	TBKBP1	76353	25kb
ENSG00000073861	17	45785610	45848485	171	23	120000	-1.7163	0.95694	TBX21	76353	25kb
ENSG00000006025	17	45859738	45924200	207	33	120000	-1.907	0.97174	OSBPL7	76353	25kb
ENSG00000159111	17	45875638	45933900	196	25	120000	-1.0633	0.85617	MRPL10	76353	25kb
ENSG00000141294	17	45883993	45940079	187	21	120000	-0.86154	0.80553	LRRC46	76353	25kb
ENSG00000141295	17	45890058	45943699	169	20	120000	-0.73731	0.76953	SCRN2	76353	25kb
ENSG00000189120	17	45897279	45958240	184	26	120000	-0.52118	0.69888	SP6	76353	25kb
ENSG00000167182	17	45948516	46031323	207	25	120000	-0.1839	0.57295	SP2	76353	25kb
ENSG00000108439	17	45993872	46050654	147	21	120000	-0.33264	0.6303	PNPO	76353	25kb
ENSG00000167183	17	46004333	46060244	150	23	120000	-0.30233	0.6188	PRR15L	76353	25kb
ENSG00000108465	17	46020176	46084140	183	22	120000	0.014011	0.49441	CDK5RAP3	76353	25kb
ENSG00000005243	17	46078533	46140392	142	24	120000	-0.12974	0.55161	COPZ2	76353	25kb
ENSG00000082641	17	46100691	46163849	156	21	120000	0.11567	0.45396	NFE2L1	76353	25kb
ENSG00000108468	17	46122414	46203883	229	20	120000	0.3597	0.35954	CBX1	76353	25kb
ENSG00000002919	17	46155719	46225436	195	19	120000	0.15137	0.43984	SNX11	76353	25kb
ENSG00000141293	17	46185802	46532637	802	28	120000	-0.15866	0.56303	SKAP1	76353	25kb
ENSG00000120094	17	46580888	46633359	147	19	120000	-0.12094	0.54813	HOXB1	76353	25kb
ENSG00000173917	17	46593256	46648441	137	21	120000	-0.080302	0.532	HOXB2	76353	25kb
ENSG00000120093	17	46601232	46707274	263	29	120000	-0.41558	0.66114	HOXB3	76353	25kb
ENSG00000182742	17	46627875	46682473	125	24	120000	0.2441	0.40358	HOXB4	76353	25kb
ENSG00000120075	17	46643619	46696323	130	25	120000	-0.038644	0.51541	HOXB5	76353	25kb
ENSG00000108511	17	46646639	46707354	157	28	120000	-0.46203	0.67797	HOXB6	76353	25kb
ENSG00000260027	17	46659594	46735934	220	34	120000	-0.37486	0.64612	HOXB7	76353	25kb
ENSG00000120068	17	46663739	46717478	154	27	120000	-0.55367	0.7101	HOXB8	76353	25kb
ENSG00000170689	17	46673518	46728839	166	27	120000	-0.79718	0.78733	HOXB9	76353	25kb
ENSG00000159182	17	46774084	46824884	120	23	120000	0.23585	0.40677	PRAC1	76353	25kb
ENSG00000159184	17	46777125	46831540	131	25	120000	-0.04173	0.51664	HOXB13	76353	25kb
ENSG00000170703	17	46814597	46919576	288	26	120000	-1.4523	0.92679	TTLL6	76353	25kb
ENSG00000136436	17	46883350	46968884	209	20	120000	-1.7437	0.9594	CALCOCO2	76353	25kb
ENSG00000159199	17	46945127	46998233	128	12	120000	-1.807	0.96462	ATP5G1	76353	25kb
ENSG00000159202	17	46960731	47031418	199	12	120000	-1.622	0.9476	UBE2Z	76353	25kb
ENSG00000159210	17	46981678	47047479	200	11	120000	-1.3493	0.91138	SNF8	76353	25kb
ENSG00000159224	17	47010916	47070958	171	19	120000	-1.3006	0.90329	GIP	76353	25kb
ENSG00000159217	17	47049774	47158012	201	28	120000	-1.5719	0.94201	IGF2BP1	76353	25kb
ENSG00000167080	17	47184822	47272351	289	29	120000	-1.025	0.84731	B4GALNT2	76353	25kb
ENSG00000167083	17	47255153	47312936	159	23	120000	-0.090009	0.53586	GNGT2	76353	25kb
ENSG00000108798	17	47262589	47325587	167	28	120000	0.023605	0.49058	ABI3	76353	25kb
ENSG00000173868	17	47275724	47333128	150	28	120000	0.23562	0.40686	PHOSPHO1	76353	25kb
ENSG00000198740	17	47341568	47464835	278	17	120000	1.2354	0.10833	ZNF652	76353	25kb
ENSG00000262039	17	47423102	47579350	427	67	120000	-0.40702	0.658	RP11-81K2.1	76353	25kb
ENSG00000167085	17	47456414	47517246	143	25	120000	-0.97749	0.83584	PHB	76353	25kb
ENSG00000064300	17	47547655	47617379	204	50	120000	0.29352	0.38456	NGFR	76353	25kb
ENSG00000182575	17	47628220	47686189	90	24	120000	-0.79202	0.78583	NXPH3	76353	25kb
ENSG00000121067	17	47651246	47780596	154	31	120000	-0.85938	0.80493	SPOP	76353	25kb
ENSG00000121073	17	47753305	47811376	68	18	120000	-0.094419	0.53761	SLC35B1	76353	25kb
ENSG00000121104	17	47762694	47891542	125	29	120000	-0.42294	0.66383	FAM117A	76353	25kb
ENSG00000136504	17	47840917	47931458	86	21	120000	-0.93516	0.82515	KAT7	76353	25kb
ENSG00000176358	17	47890671	47950379	76	14	120000	-0.31568	0.62388	TAC4	76353	25kb
ENSG00000108813	17	48021334	48077321	180	43	120000	-0.46298	0.67831	DLX4	76353	25kb
ENSG00000064195	17	48042369	48097588	155	35	120000	-0.54741	0.70795	DLX3	76353	25kb
ENSG00000005884	17	48108332	48192845	266	32	120000	1.1563	0.12378	ITGA3	76353	25kb
ENSG00000005882	17	48147101	48214516	160	29	120000	1.2118	0.11279	PDK2	76353	25kb
ENSG00000167100	17	48162404	48232246	151	30	120000	0.33903	0.36729	SAMD14	76353	25kb
ENSG00000108819	17	48186104	48252991	118	31	120000	-0.25439	0.6004	PPP1R9B	76353	25kb
ENSG00000108823	17	48216575	48278292	135	32	120000	-0.55352	0.71004	SGCA	76353	25kb
ENSG00000108821	17	48235650	48303993	167	41	120000	-0.71736	0.76342	COL1A1	76353	25kb
ENSG00000167105	17	48323767	48383844	204	49	120000	-1.1075	0.86595	TMEM92	76353	25kb
ENSG00000015532	17	48398453	48465499	244	18	120000	-0.13452	0.5535	XYLT2	76353	25kb
ENSG00000108826	17	48420218	48475575	193	15	120000	-0.42218	0.66355	MRPL27	76353	25kb
ENSG00000154920	17	48425581	48483844	194	17	120000	-0.74496	0.77185	EME1	76353	25kb
ENSG00000108829	17	48427420	48499914	245	22	120000	-1.1048	0.86538	LRRC59	76353	25kb

ENSG00000167107	17	48478519	48577206	258	26	120000	-2.0467	0.97965	ACSF2	76353	25kb
ENSG00000136457	17	48516857	48571327	119	17	120000	-1.7799	0.96246	CHAD	76353	25kb
ENSG00000136444	17	48531161	48588336	119	19	120000	-1.5935	0.94448	RSAD1	76353	25kb
ENSG00000136449	17	48560745	48633862	213	33	120000	-1.6356	0.94904	MYCBPAP	76353	25kb
ENSG00000049283	17	48584904	48646111	187	28	120000	-1.4156	0.92156	EPN3	76353	25kb
ENSG00000006282	17	48595419	48658213	189	28	120000	-1.3939	0.91832	SPATA20	76353	25kb
ENSG00000006283	17	48613429	48729835	303	44	120000	-1.4481	0.92621	CACNA1G	76353	25kb
ENSG00000108846	17	48687138	48794613	233	54	120000	-0.12875	0.55122	ABCC3	76353	25kb
ENSG00000154945	17	48745551	48810285	120	21	120000	0.27901	0.39012	ANKRD40	76353	25kb
ENSG00000108848	17	48771905	48858574	195	35	120000	-0.095902	0.5382	LUC7L3	76353	25kb
ENSG00000173714	17	48887011	48944709	167	20	120000	0.70175	0.24142	WFIKKN2	76353	25kb
ENSG00000141232	17	48914584	48970339	118	14	120000	0.22115	0.41249	TOB1	76353	25kb
ENSG00000008294	17	49014535	49223226	317	23	120000	-1.4414	0.92526	SPAG9	76353	25kb
ENSG00000239672	17	49205897	49264789	177	24	120000	0.95403	0.17003	NME1	76353	25kb
ENSG00000243678	17	49205920	49274108	192	25	120000	0.87217	0.19156	NME1-NME2	76353	25kb
ENSG00000011052	17	49205951	49274105	192	25	120000	0.87217	0.19156	NME2	76353	25kb
ENSG00000011258	17	49229786	49362524	338	21	120000	0.13787	0.44517	MBTD1	76353	25kb
ENSG00000011260	17	49312889	49400297	211	20	120000	0.22997	0.40906	UTP18	76353	25kb
ENSG00000154975	17	49682674	50262377	1408	84	120000	-2.1967	0.98598	CA10	76353	25kb
ENSG00000226364	17	50914481	51001948	408	30	120000	0.066383	0.47354	AC102948.2	76353	25kb
ENSG00000227011	17	51037880	51090012	140	18	120000	-1.2265	0.88999	C17orf112	76353	25kb
ENSG00000141200	17	51875239	51927573	196	24	120000	-0.44368	0.67136	KIF2B	76353	25kb
ENSG00000141198	17	52951748	53064310	381	40	120000	-0.4095	0.65891	TOM1L1	76353	25kb
ENSG00000166260	17	53004263	53071146	212	15	120000	-0.5371	0.7044	COX11	76353	25kb
ENSG00000166263	17	53021088	53266646	616	20	120000	-1.4209	0.92233	STXBP4	76353	25kb
ENSG00000108924	17	53317373	53427426	266	43	120000	0.35945	0.35963	HLF	76353	25kb
ENSG00000108960	17	53444974	53524353	184	32	120000	1.7668	0.038631	MMD	76353	25kb
ENSG00000166292	17	53771988	53834482	151	30	120000	-1.2513	0.89458	TMEM100	76353	25kb
ENSG00000141179	17	53803340	53945191	410	23	120000	-1.6635	0.95189	PCTP	76353	25kb
ENSG00000153930	17	54163319	54614020	999	65	120000	-0.65384	0.74339	ANKFN1	76353	25kb
ENSG00000183691	17	54646060	54697951	90	16	120000	1.1233	0.13067	NOG	76353	25kb
ENSG00000214226	17	54844274	54941134	250	20	120000	1.049	0.14709	C17orf67	76353	25kb
ENSG00000153933	17	54886460	54971036	193	22	120000	0.0041378	0.49835	DGKE	76353	25kb
ENSG00000121060	17	54940270	55016399	132	36	120000	-0.93068	0.82399	TRIM25	76353	25kb
ENSG00000121058	17	54990548	55063415	144	25	120000	-1.6668	0.95222	COIL	76353	25kb
ENSG00000121064	17	55030466	55109129	213	38	120000	-0.82334	0.79484	SCPEP1	76353	25kb
ENSG00000121057	17	55137453	55223710	275	39	120000	0.8114	0.20857	AKAP1	76353	25kb
ENSG00000153944	17	55308212	55787046	1311	167	120000	-1.1728	0.87957	MSI2	76353	25kb
ENSG00000166329	17	55796842	55847673	199	30	120000	-0.76705	0.77847	CCDC182	76353	25kb
ENSG00000181610	17	55891842	55952417	178	27	120000	0.35373	0.36177	MRPS23	76353	25kb
ENSG00000180891	17	55913604	56057684	370	64	120000	-1.2468	0.89377	CUEDC1	76353	25kb
ENSG00000136451	17	56023910	56090620	121	20	120000	-0.73056	0.76747	VEZF1	76353	25kb
ENSG00000266086	17	56041399	56107614	117	16	120000	-0.55272	0.70977	RP11-159D12.5	76353	25kb
ENSG00000136450	17	56055721	56109707	92	14	120000	-0.45607	0.67583	SRSF1	76353	25kb
ENSG00000264364	17	56135776	56197897	114	20	120000	0.22467	0.41112	DYNLL2	76353	25kb
ENSG00000141194	17	56207494	56258517	135	24	120000	-0.13835	0.55502	OR4D1	76353	25kb
ENSG00000255713	17	56222017	56272940	153	24	120000	-0.42978	0.66632	OR4D2	76353	25kb
ENSG00000121053	17	56245098	56307535	142	21	120000	-1.3356	0.90916	EPX	76353	25kb
ENSG00000011143	17	56257803	56321966	113	23	120000	-0.89794	0.81539	MKS1	76353	25kb
ENSG00000167419	17	56270909	56370879	166	29	120000	-0.34844	0.63625	LPO	76353	25kb
ENSG00000005381	17	56322217	56383296	129	19	120000	-0.26751	0.60546	MPO	76353	25kb
ENSG00000005379	17	56353592	56431152	210	29	120000	-0.49512	0.68974	BZRAP1	76353	25kb
ENSG00000213246	17	56397539	56455454	126	25	120000	-0.31304	0.62287	SUPT4H1	76353	25kb
ENSG00000108375	17	56404861	56519956	192	27	120000	-0.082371	0.53282	RNF43	76353	25kb
ENSG00000176160	17	56472528	56590745	154	19	120000	0.51818	0.30216	HSF5	76353	25kb
ENSG00000108389	17	56541898	56620266	115	16	120000	0.62362	0.26644	MTMR4	76353	25kb
ENSG00000108387	17	56572611	56643179	122	18	120000	0.55202	0.29047	SEPT4	76353	25kb
ENSG00000181013	17	56593948	56646729	99	17	120000	0.46111	0.32236	C17orf47	76353	25kb
ENSG00000121101	17	56609039	56794416	372	25	120000	0.4543	0.32481	TEX14	76353	25kb
ENSG00000108384	17	56744934	56836703	157	18	120000	0.13964	0.44447	RAD51C	76353	25kb
ENSG00000175175	17	56808230	57083983	395	23	120000	0.45997	0.32277	PPM1E	76353	25kb
ENSG00000108395	17	57034999	57209282	249	24	120000	0.46147	0.32223	TRIM37	76353	25kb
ENSG00000182628	17	57162312	57257630	127	22	120000	0.074578	0.47028	SKA2	76353	25kb
ENSG00000068489	17	57207860	57307066	156	17	120000	-1.1147	0.86751	PRR11	76353	25kb
ENSG00000265303	17	57249927	57317587	119	14	120000	-1.0805	0.86003	CTD-2510F5.6	76353	25kb
ENSG00000167447	17	57261761	57317608	94	11	120000	-1.1345	0.87171	SMG8	76353	25kb
ENSG00000153982	17	57272828	57378328	200	19	120000	-1.1828	0.88157	GDPD1	76353	25kb
ENSG00000175155	17	57384050	57504090	249	35	120000	-0.27372	0.60785	YPEL2	76353	25kb
ENSG00000108406	17	57617886	57710706	92	10	120000	-0.41193	0.6598	DHX40	76353	25kb
ENSG00000141367	17	57672219	57798671	176	14	120000	-0.40534	0.65739	CLTC	76353	25kb
ENSG00000141378	17	57726997	57809987	129	16	120000	-0.13446	0.55348	PTRH2	76353	25kb
ENSG00000062716	17	57759553	57944616	278	18	120000	0.62843	0.26486	VMP1	76353	25kb
ENSG00000108423	17	57911851	57995304	142	16	120000	0.75137	0.22622	TUBD1	76353	25kb
ENSG00000108443	17	57945447	58052925	172	17	120000	0.61647	0.26879	RPS6KB1	76353	25kb
ENSG00000267318	17	57993269	58075462	94	13	120000	0.45093	0.32602	RP11-178C3.1	76353	25kb
ENSG00000189050	17	58004601	58067122	74	12	120000	0.427	0.33469	RNFT1	76353	25kb
ENSG00000068097	17	58095555	58181292	98	7	120000	0.88443	0.18823	HEATR6	76353	25kb
ENSG00000167434	17	58202297	58273260	103	19	120000	-0.10656	0.54243	CA4	76353	25kb
ENSG00000170832	17	58231455	58524831	242	14	120000	-0.79795	0.78755	USP32	76353	25kb
ENSG00000141371	17	58444790	58533782	70	6	120000	-0.87782	0.80998	C17orf64	76353	25kb
ENSG00000062725	17	58495520	58628580	69	18	120000	-0.98779	0.83837	APPBP2	76353	25kb
ENSG00000267772	17	58616908	58688984	45	10	120000	1.0912	0.13759	RP11-15E18.4	76353	25kb
ENSG00000170836	17	58652544	58766849	34	10	120000	2.1187	0.017058	PPM1D	76353	25kb
ENSG00000141376	17	58729814	59495199	1010	65	120000	0.16728	0.43357	BCAS3	76353	25kb
ENSG00000121068	17	59452257	59511827	140	24	120000	-1.2018	0.88528	TBX2	76353	25kb
ENSG00000187013	17	59464112	59515641	117	19	120000	-0.91972	0.82114	C17orf82	76353	25kb
ENSG00000121075	17	59504765	59587471	157	31	120000	0.73436	0.23137	TBX4	76353	25kb
ENSG00000253506	17	59642794	59693563	129	17	120000	0.75195	0.22604	NACA2	76353	25kb
ENSG00000136492	17	59733627	59965882	357	23	120000	-0.66484	0.74692	BRIP1	76353	25kb
ENSG00000108506	17	59917731	60030377	164	20	120000	-1.0918	0.86253	INTS2	76353	25kb
ENSG00000108510	17	59994966	60167643	211	20	120000	-0.98088	0.83667	MED13	76353	25kb
ENSG00000270033	17	60324848	60385276	106	18	120000	-0.66748	0.74777	RP11-51L5.7	76353	25kb
ENSG00000172421	17	60422579	60518837	183	27	120000	-1.1913	0.88323	EFCAB3	76353	25kb
ENSG00000087995	17	60476228	60552454	76	16	120000	-0.94709	0.8282	METTL2A	76353	25kb
ENSG00000146872	17	60511019	60717842	236	21	120000	-0.97698	0.83571	TLK2	76353	25kb
ENSG00000011028	17	60679762	60795958	208	33	120000	-1.4808	0.93067	MRC2	76353	25kb

ENSG00000173838	17	60753675	60910705	388	34	120000	-0.33607	0.63159	MARCH10	76353	25kb
ENSG00000170921	17	61061917	61530060	752	32	120000	0.68708	0.24601	TANC2	76353	25kb
ENSG00000008283	17	61484665	61548739	85	17	120000	0.39472	0.34652	CYB561	76353	25kb
ENSG00000159640	17	61529422	61624205	190	27	120000	-0.09616	0.5383	ACE	76353	25kb
ENSG00000264813	17	61537184	61624209	180	25	120000	-0.22013	0.58712	ACE	76353	25kb
ENSG00000173826	17	61575695	61651338	136	20	120000	0.4466	0.32758	KCNH6	76353	25kb
ENSG00000136485	17	61602822	61696639	99	20	120000	1.0587	0.14486	DCAF7	76353	25kb
ENSG00000136463	17	61653231	61710725	48	9	120000	0.11041	0.45604	TACO1	76353	25kb
ENSG00000198909	17	61674775	61798663	146	12	120000	0.045415	0.48189	MAP3K3	76353	25kb
ENSG00000136490	17	61748262	61803532	81	10	120000	-0.17511	0.56951	LIMD2	76353	25kb
ENSG00000266173	17	61755192	61844330	158	12	120000	-0.27716	0.60917	STRADA	76353	25kb
ENSG00000125695	17	61755205	61854457	183	13	120000	-0.29947	0.61771	RP11-51F16.8	76353	25kb
ENSG00000108588	17	61797610	61878711	172	11	120000	-0.36912	0.64398	CCDC47	76353	25kb
ENSG00000198231	17	61825963	61921677	180	10	120000	-0.41208	0.65986	DDX42	76353	25kb
ENSG00000108592	17	61871793	61932372	115	7	120000	-0.55927	0.71201	FTSJ3	76353	25kb
ENSG00000087191	17	61879512	61934379	102	7	120000	-0.51392	0.69635	PSMC5	76353	25kb
ENSG00000108604	17	61884444	61945425	130	8	120000	-0.52733	0.70102	SMARCD2	76353	25kb
ENSG00000213218	17	61924372	61976126	136	9	120000	-0.68943	0.75472	CSH2	76353	25kb
ENSG00000136487	17	61932578	61984295	136	10	120000	-0.64358	0.74007	GH2	76353	25kb
ENSG00000136488	17	61947275	61999021	135	13	120000	-0.72002	0.76424	CSH1	76353	25kb
ENSG00000204414	17	61961957	62021198	138	15	120000	-0.50166	0.69205	CSHL1	76353	25kb
ENSG00000259384	17	61969560	62021179	117	14	120000	-0.45074	0.67391	GH1	76353	25kb
ENSG00000007312	17	61981100	62034714	119	16	120000	-0.25057	0.59892	CD79B	76353	25kb
ENSG00000007314	17	61990914	62075278	182	35	120000	-0.19206	0.57615	SCN4A	76353	25kb
ENSG00000224383	17	62050711	62106664	88	20	120000	0.022585	0.49099	C17orf72	76353	25kb
ENSG00000108622	17	62054954	62122994	135	21	120000	-0.045359	0.51809	ICAM2	76353	25kb
ENSG00000178607	17	62091502	62233179	309	31	120000	-0.70752	0.76038	ERN1	76353	25kb
ENSG00000136478	17	62199587	62365661	404	24	120000	-0.68787	0.75423	TEX2	76353	25kb
ENSG00000271605	17	62436569	62489760	51	5	120000	-1.3777	0.91586	MILR1	76353	25kb
ENSG00000256525	17	62448902	62518154	112	11	120000	-1.2521	0.89474	POLG2	76353	25kb
ENSG00000108654	17	62470734	62529317	117	13	120000	-1.1717	0.87933	DDX5	76353	25kb
ENSG00000258890	17	62477706	62563579	162	15	120000	-0.94491	0.82765	CEP95	76353	25kb
ENSG00000108854	17	62513413	62683186	282	14	120000	-0.63681	0.73788	SMURF2	76353	25kb
ENSG00000176809	17	62825430	62940598	29	19	120000	0.16315	0.4352	LRRC37A3	76353	25kb
ENSG00000120063	17	62981833	63077957	196	21	120000	0.47371	0.31785	GNA13	76353	25kb
ENSG00000108370	17	63108549	63248821	546	33	120000	-0.77702	0.78143	RGS9	76353	25kb
ENSG00000168646	17	63499681	63582765	238	56	120000	-1.0422	0.85134	AXIN2	76353	25kb
ENSG00000266076	17	63509220	63662151	401	56	120000	0.30843	0.37888	CTD-2535L24.2	76353	25kb
ENSG00000154240	17	63606656	64213202	2119	36	120000	-0.62223	0.73311	CEP112	76353	25kb
ENSG00000091583	17	64183151	64277643	407	30	120000	0.55735	0.28864	APOH	76353	25kb
ENSG00000154229	17	64273754	64831861	1965	141	120000	-0.095383	0.53799	PRKCA	76353	25kb
ENSG00000075429	17	64806235	64906603	379	60	120000	1.8406	0.032839	CACNG5	76353	25kb
ENSG00000075461	17	64936026	65054514	399	69	120000	-0.10009	0.53987	CACNG4	76353	25kb
ENSG00000214167	17	65002509	65053198	148	34	120000	0.22699	0.41022	AC005544.1	76353	25kb
ENSG00000108878	17	65015706	65077909	156	29	120000	0.4427	0.32899	CACNG1	76353	25kb
ENSG00000198265	17	65041554	65267105	437	22	120000	-0.092219	0.53674	HELZ	76353	25kb
ENSG00000197170	17	65309032	65387743	160	28	120000	0.72521	0.23416	PSMD12	76353	25kb
ENSG00000154217	17	65348575	65718372	979	135	120000	-0.32902	0.62893	PITPNC1	76353	25kb
ENSG00000130935	17	65688949	65765647	205	19	120000	0.17653	0.42994	NOL11	76353	25kb
ENSG00000171634	17	65796640	66005494	419	23	120000	0.42244	0.33635	BPTF	76353	25kb
ENSG00000186665	17	65962217	66014765	115	16	120000	0.23445	0.40732	C17orf58	76353	25kb
ENSG00000182481	17	66006635	66067958	169	13	120000	0.24019	0.40509	KPNA2	76353	25kb
ENSG00000196704	17	66218715	66278297	242	28	120000	0.95758	0.16914	AMZ2	76353	25kb
ENSG00000141337	17	66230323	66443872	591	85	120000	0.96759	0.16663	ARSG	76353	25kb
ENSG00000108932	17	66238167	66312408	186	31	120000	0.59172	0.27702	SLC16A6	76353	25kb
ENSG00000070540	17	66392089	66478654	209	34	120000	0.94328	0.17277	WIPI1	76353	25kb
ENSG00000108946	17	66482921	66572460	209	31	120000	2.3171	0.01025	PRKAR1A	76353	25kb
ENSG00000108950	17	66506254	66622530	254	39	120000	1.5518	0.060353	FAM20A	76353	25kb
ENSG00000269657	17	66514369	66567970	119	20	120000	1.8758	0.030339	AC079210.1	76353	25kb
ENSG00000141338	17	66838433	66976533	346	29	120000	1.2983	0.097098	ABCA8	76353	25kb
ENSG00000154258	17	66945629	67082205	287	26	120000	2.3056	0.010565	ABCA9	76353	25kb
ENSG00000154262	17	67049843	67163029	215	18	120000	1.4804	0.069387	ABCA6	76353	25kb
ENSG00000154263	17	67118355	67265987	306	21	120000	1.2881	0.098847	ABCA10	76353	25kb
ENSG00000154265	17	67215452	67348385	299	28	120000	0.74836	0.22712	ABCA5	76353	25kb
ENSG00000108984	17	67385839	67564472	428	57	120000	1.1804	0.11892	MAP2K6	76353	25kb
ENSG00000153822	17	68024570	68156749	385	25	120000	-0.47629	0.68307	KCNJ16	76353	25kb
ENSG00000123700	17	68139814	68201189	141	19	120000	1.0893	0.13801	KCNJ2	76353	25kb
ENSG00000267893	17	70011164	70061872	160	19	120000	-1.0525	0.85372	AC007461.1	76353	25kb
ENSG00000125398	17	70092161	70147561	135	18	120000	0.35518	0.36123	SOX9	76353	25kb
ENSG00000133195	17	70617088	71113851	1729	188	120000	-0.30738	0.62072	SLC39A11	76353	25kb
ENSG00000180616	17	71136151	71192185	186	35	120000	0.08098	0.46773	SSTR2	76353	25kb
ENSG00000166685	17	71164129	71229646	202	15	120000	-0.3798	0.64795	COG1	76353	25kb
ENSG00000133193	17	71178492	71257892	260	11	120000	-0.4738	0.68218	FAM104A	76353	25kb
ENSG00000141219	17	71203372	71270091	222	13	120000	-0.38228	0.64887	C17orf80	76353	25kb
ENSG00000187959	17	71219588	71283491	203	17	120000	-0.35382	0.63826	CPSF4L	76353	25kb
ENSG00000179604	17	71254763	71333314	222	44	120000	0.27152	0.393	CDC42EP4	76353	25kb
ENSG00000069188	17	71305523	71665228	1173	151	120000	1.1347	0.12826	SDK2	76353	25kb
ENSG00000172809	17	72174721	72231794	177	39	120000	1.3321	0.09142	RPL38	76353	25kb
ENSG00000141540	17	72184653	72283155	327	43	120000	0.69779	0.24265	TTYH2	76353	25kb
ENSG00000171595	17	72245386	72336023	350	38	120000	-0.88262	0.81128	DNAI2	76353	25kb
ENSG00000196169	17	72297349	72376959	262	40	120000	-0.22239	0.58799	KIF19	76353	25kb
ENSG00000268626	17	72321906	72373271	158	29	120000	0.38916	0.34858	AC103809.2	76353	25kb
ENSG00000204347	17	72327555	72383085	182	38	120000	0.2108	0.41652	BTBD17	76353	25kb
ENSG00000257008	17	72338546	72393761	169	38	120000	0.60144	0.27377	GPR142	76353	25kb
ENSG00000170412	17	72395990	72472792	257	32	120000	2.1995	0.01392	GPRC5C	76353	25kb
ENSG00000167851	17	72437555	72505933	276	36	120000	1.7087	0.043752	CD300A	76353	25kb
ENSG00000178789	17	72492313	72552613	231	27	120000	0.48146	0.31509	CD300LB	76353	25kb
ENSG00000167850	17	72512247	72567282	223	25	120000	1.19	0.11703	CD300C	76353	25kb
ENSG00000204345	17	72550504	72613422	250	28	120000	0.5389	0.29498	CD300LD	76353	25kb
ENSG00000182352	17	72555818	72615348	230	26	120000	0.17335	0.43119	C17orf77	76353	25kb
ENSG00000186407	17	72581026	72644897	230	28	120000	-1.4739	0.92975	CD300E	76353	25kb
ENSG00000172794	17	72641717	72768474	240	51	120000	0.80513	0.21037	RAB37	76353	25kb
ENSG00000186074	17	72665452	72734117	122	29	120000	1.1052	0.13453	CD300LF	76353	25kb
ENSG00000109062	17	72719791	72790492	138	19	120000	-0.56655	0.71449	SLC9A3R1	76353	25kb
ENSG00000109065	17	72741686	72797506	124	15	120000	-0.76521	0.77793	NAT9	76353	25kb
ENSG00000109066	17	72747622	72860918	243	24	120000	-0.90521	0.81732	TMEM104	76353	25kb

ENSG00000161509	17	72813162	72882627	142	12	120000	-0.44562	0.67206	GRIN2C	76353	25kb
ENSG00000161513	17	72833619	72894156	142	16	120000	-0.72436	0.76558	FDXR	76353	25kb
ENSG00000172782	17	72848428	72914708	162	21	120000	-0.27112	0.60685	FADS6	76353	25kb
ENSG00000182040	17	72887176	72944351	146	24	120000	0.32965	0.37083	USH1G	76353	25kb
ENSG00000183034	17	72895370	72955007	134	34	120000	0.33246	0.36977	OTOP2	76353	25kb
ENSG00000182938	17	72906814	72971087	129	33	120000	-0.6638	0.74659	OTOP3	76353	25kb
ENSG00000167861	17	72921838	72994261	152	36	120000	-0.37173	0.64495	HID1	76353	25kb
ENSG00000109089	17	72958727	73026895	156	26	120000	0.78776	0.21542	CDR2L	76353	25kb
ENSG00000167862	17	72983765	73042356	178	26	120000	0.77539	0.21905	ICT1	76353	25kb
ENSG00000180901	17	73003670	73086984	292	26	120000	0.84611	0.19874	KCTD2	76353	25kb
ENSG00000167863	17	73009958	73068080	207	26	120000	0.044771	0.48214	ATP5H	76353	25kb
ENSG00000170190	17	73058822	73127257	244	31	120000	2.224	0.013076	SLC16A5	76353	25kb
ENSG00000125449	17	73081047	73151360	208	27	120000	2.6379	0.0041713	ARMC7	76353	25kb
ENSG00000125458	17	73101320	73152890	113	17	120000	2.7968	0.0025802	NT5C	76353	25kb
ENSG00000189159	17	73106343	73189376	162	21	120000	2.9617	0.0015297	HN1	76353	25kb
ENSG00000188612	17	73138408	73204078	128	20	120000	2.6602	0.0039046	SUMO2	76353	25kb
ENSG00000125450	17	73176754	73256853	167	18	120000	1.5214	0.064074	NUP85	76353	25kb
ENSG00000125447	17	73207694	73283444	172	18	120000	0.79716	0.21268	GGA3	76353	25kb
ENSG00000125445	17	73232755	73287454	126	14	120000	0.19144	0.42409	MRPS7	76353	25kb
ENSG00000125457	17	73237309	73292308	138	15	120000	0.075944	0.46973	MIF4GD	76353	25kb
ENSG00000125454	17	73244073	73310591	177	17	120000	-0.069952	0.52788	SLC25A19	76353	25kb
ENSG00000177885	17	73289157	73426790	334	17	120000	-0.47212	0.68158	GRB2	76353	25kb
ENSG00000177728	17	73412240	73521171	329	24	120000	0.53525	0.29624	KIAA0195	76353	25kb
ENSG00000177303	17	73471342	73536664	221	27	120000	0.73508	0.23115	CASKIN2	76353	25kb
ENSG00000182173	17	73487141	73545820	232	28	120000	0.81016	0.20892	TSEN54	76353	25kb
ENSG00000073350	17	73496161	73596289	505	44	120000	0.082144	0.46727	LLGL2	76353	25kb
ENSG00000266714	17	73559139	73647929	378	42	120000	0.77516	0.21912	MYO15B	76353	25kb
ENSG00000108469	17	73597925	73688269	204	25	120000	1.1329	0.12864	RECQL5	76353	25kb
ENSG00000204323	17	73604514	73662484	126	17	120000	0.99957	0.15876	SMIM5	76353	25kb
ENSG00000259120	17	73617323	73669057	84	13	120000	0.54226	0.29382	SMIM6	76353	25kb
ENSG00000161526	17	73638196	73729142	133	17	120000	1.0767	0.14082	SAP30BP	76353	25kb
ENSG00000132470	17	73692408	73778899	163	40	120000	0.69548	0.24338	ITGB4	76353	25kb
ENSG00000108479	17	73722675	73786792	143	37	120000	0.16264	0.4354	GALK1	76353	25kb
ENSG00000132475	17	73747515	73806974	130	24	120000	-0.35899	0.6402	H3F3B	76353	25kb
ENSG00000132478	17	73755681	73846886	221	27	120000	-0.98781	0.83838	UNK	76353	25kb
ENSG00000092929	17	73798306	73865798	175	21	120000	-0.83149	0.79715	UNC13D	76353	25kb
ENSG00000132471	17	73816780	73877588	167	19	120000	-0.47592	0.68294	WBP2	76353	25kb
ENSG00000132481	17	73845242	73899656	132	14	120000	0.092539	0.46313	TRIM47	76353	25kb
ENSG00000141569	17	73851416	73918084	152	17	120000	-0.10797	0.54299	TRIM65	76353	25kb
ENSG00000204316	17	73869724	73930899	124	17	120000	0.025804	0.48971	MRPL38	76353	25kb
ENSG00000267426	17	73869726	73951210	169	19	120000	-0.32255	0.62648	RP11-552F3.12	76353	25kb
ENSG00000188878	17	73880655	73962221	180	24	120000	-0.70889	0.7608	FBF1	76353	25kb
ENSG00000161533	17	73912588	74000515	232	33	120000	-0.72917	0.76705	ACOX1	76353	25kb
ENSG00000257949	17	73950301	74021667	227	32	120000	-0.652	0.7428	TEN1	76353	25kb
ENSG00000250506	17	73971987	74027080	154	27	120000	-0.59271	0.72331	CDK3	76353	25kb
ENSG00000167880	17	73975583	74048533	184	34	120000	-0.47574	0.68287	EVPL	76353	25kb
ENSG00000167881	17	74010184	74093734	173	20	120000	-0.289	0.61371	SRP68	76353	25kb
ENSG00000186919	17	74043042	74104747	111	9	120000	-0.076101	0.53033	ZACN	76353	25kb
ENSG00000182687	17	74045875	74098622	96	7	120000	0.051027	0.47965	GALR2	76353	25kb
ENSG00000182473	17	74052087	74142657	180	20	120000	-0.48602	0.68652	EXOC7	76353	25kb
ENSG00000129654	17	74107414	74162380	120	18	120000	-0.17993	0.5714	FOXJ1	76353	25kb
ENSG00000141576	17	74113534	74261454	345	35	120000	-0.28427	0.6119	RNF157	76353	25kb
ENSG00000185262	17	74236283	74292375	165	19	120000	-0.43072	0.66666	UBALD2	76353	25kb
ENSG00000129646	17	74245130	74328761	281	23	120000	-0.4258	0.66487	QRICH2	76353	25kb
ENSG00000161542	17	74280567	74405602	381	42	120000	0.46049	0.32258	PRPSAP1	76353	25kb
ENSG00000176170	17	74347665	74408941	161	24	120000	1.0052	0.1574	SPHK1	76353	25kb
ENSG00000175931	17	74360532	74474288	331	32	120000	0.74551	0.22798	UBE2O	76353	25kb
ENSG00000129673	17	74424433	74491199	234	37	120000	0.16144	0.43587	AANAT	76353	25kb
ENSG00000129667	17	74441973	74522872	301	42	120000	-0.17109	0.56792	RHBDF2	76353	25kb
ENSG00000161544	17	74498438	74572257	244	38	120000	0.33535	0.36868	CYGB	76353	25kb
ENSG00000214140	17	74498668	74574660	255	32	120000	0.15264	0.43934	PRCD	76353	25kb
ENSG00000070731	17	74534792	74608038	278	35	120000	0.070125	0.47205	ST6GALNAC2	76353	25kb
ENSG00000070526	17	74595843	74664920	265	20	120000	0.68785	0.24577	ST6GALNAC1	76353	25kb
ENSG00000182534	17	74643633	74732098	257	19	120000	0.5021	0.3078	MXRA7	76353	25kb
ENSG00000070495	17	74683919	74747866	146	17	120000	-0.052714	0.52102	JMJD6	76353	25kb
ENSG00000181038	17	74697912	74755018	111	17	120000	0.0010525	0.49958	METTL23	76353	25kb
ENSG00000267168	17	74704060	74759604	88	19	120000	0.056388	0.47752	RP11-318A15.7	76353	25kb
ENSG00000161547	17	74705197	74758456	81	18	120000	0.22496	0.411	SRSF2	76353	25kb
ENSG00000092931	17	74706947	74802531	118	26	120000	0.71764	0.23649	MFSD11	76353	25kb
ENSG00000167889	17	74839538	74971475	676	75	120000	0.87405	0.19105	MGAT5B	76353	25kb
ENSG00000129657	17	75057798	75238179	664	63	120000	0.53637	0.29585	SEC14L1	76353	25kb
ENSG00000184640	17	75251651	75521678	893	92	120000	-0.68852	0.75444	SEPT9	76353	25kb
ENSG00000204283	17	75850109	75903659	196	19	120000	-0.81068	0.79122	FLJ45079	76353	25kb
ENSG00000078687	17	75975249	76129916	375	43	120000	-0.51827	0.69787	TNRC6C	76353	25kb
ENSG00000141524	17	76081539	76153488	260	50	120000	0.43473	0.33188	TMC6	76353	25kb
ENSG00000167895	17	76101851	76164049	250	58	120000	1.2128	0.11261	TMC8	76353	25kb
ENSG00000187997	17	76117434	76187258	306	49	120000	0.69068	0.24488	C17orf99	76353	25kb
ENSG00000108639	17	76139639	76194608	253	36	120000	0.36467	0.35768	SYNGR2	76353	25kb
ENSG00000167900	17	76145160	76208314	302	43	120000	-0.10121	0.54031	TK1	76353	25kb
ENSG00000183077	17	76158398	76228782	339	46	120000	-0.93205	0.82434	AFMID	76353	25kb
ENSG00000089685	17	76185267	76246717	305	37	120000	-1.6622	0.95176	BIRC5	76353	25kb
ENSG00000268310	17	76185462	76245740	302	39	120000	-1.7332	0.95847	AC087645.1	76353	25kb
ENSG00000204278	17	76202391	76262068	271	29	120000	-1.8194	0.96557	TMEM235	76353	25kb
ENSG00000184557	17	76327864	76381158	185	40	120000	-1.7707	0.96169	SOCS3	76353	25kb
ENSG00000087157	17	76349721	76446195	402	27	120000	-0.82648	0.79574	PGS1	76353	25kb
ENSG00000187775	17	76394778	76598476	1009	106	120000	-0.34055	0.63328	DNAH17	76353	25kb
ENSG00000268965	17	76397409	76447834	215	15	120000	-0.25801	0.6018	AC061992.1	76353	25kb
ENSG00000268470	17	76469911	76524138	332	46	120000	-0.2981	0.61718	DNAH17-AS1	76353	25kb
ENSG00000108669	17	76645130	76803379	371	24	120000	-0.26966	0.60629	CYTH1	76353	25kb
ENSG00000055483	17	76758463	76862523	182	23	120000	0.26191	0.39669	USP36	76353	25kb
ENSG00000035862	17	76824059	76946469	411	38	120000	1.3236	0.092823	TIMP2	76353	25kb
ENSG00000178404	17	76841992	76924299	292	28	120000	1.4643	0.071554	DDC8	76353	25kb
ENSG00000108679	17	76942320	77001191	187	39	120000	-0.53471	0.70357	LGALS3BP	76353	25kb
ENSG00000171302	17	76962799	77030949	194	37	120000	-0.66509	0.747	CANT1	76353	25kb
ENSG00000265096	17	76990291	77048737	163	26	120000	-1.6049	0.94574	C1QTNF1-AS1	76353	25kb
ENSG00000173918	17	76993896	77070870	191	28	120000	-1.5928	0.9444	C1QTNF1	76353	25kb

ENSG00000167280	17	77046021	77109681	199	34	120000	-0.57021	0.71573	ENGASE	76353	25kb
ENSG00000167281	17	77060427	77638550	2158	267	120000	0.085281	0.46602	RBFOX3	76353	25kb
ENSG00000182156	17	77679681	77741021	216	33	120000	0.25951	0.39762	ENPP7	76353	25kb
ENSG00000173894	17	77726931	77786782	99	27	120000	0.042035	0.48324	CBX2	76353	25kb
ENSG00000141570	17	77740931	77800482	110	31	120000	0.19311	0.42344	CBX8	76353	25kb
ENSG00000141582	17	77781955	77838228	144	28	120000	-0.12468	0.54961	CBX4	76353	25kb
ENSG00000167291	17	77881142	78034647	649	105	120000	0.23844	0.40577	TBC1D16	76353	25kb
ENSG00000141519	17	77985435	78099412	530	36	120000	-0.51322	0.6961	CCDC40	76353	25kb
ENSG00000171298	17	78050355	78118678	335	26	120000	0.12868	0.44881	GAA	76353	25kb
ENSG00000141543	17	78084013	78145982	245	30	120000	0.24814	0.40201	EIF4A3	76353	25kb
ENSG00000141527	17	78118791	78208130	326	45	120000	-0.31721	0.62446	CARD14	76353	25kb
ENSG00000181523	17	78155515	78219722	260	31	120000	-0.65893	0.74503	SGSH	76353	25kb
ENSG00000181045	17	78168498	78252299	337	40	120000	-0.34054	0.63327	SLC26A11	76353	25kb
ENSG00000173821	17	78209665	78397586	614	61	120000	0.32477	0.37268	RNF213	76353	25kb
ENSG00000173818	17	78363965	78436886	251	40	120000	0.5653	0.28594	ENDOV	76353	25kb
ENSG00000171246	17	78415948	78476643	174	43	120000	0.046981	0.48126	NPTX1	76353	25kb
ENSG00000141564	17	78493619	78965171	1862	80	120000	0.89646	0.185	RPTOR	76353	25kb
ENSG00000176108	17	78940398	79008317	289	40	120000	0.048178	0.48079	CHMP6	76353	25kb
ENSG00000175911	17	78952135	79003932	220	38	120000	-0.20731	0.58212	AC127496.1	76353	25kb
ENSG00000175866	17	78983948	79116232	585	71	120000	1.0783	0.14046	BAIAP2	76353	25kb
ENSG00000181409	17	79066095	79164877	405	56	120000	1.1203	0.13129	AATK	76353	25kb
ENSG00000141577	17	79138393	79221799	312	35	120000	0.74013	0.22961	AZI1	76353	25kb
ENSG00000167302	17	79177077	79237891	209	33	120000	0.60163	0.27371	ENTHD2	76353	25kb
ENSG00000224877	17	79188039	79240081	181	31	120000	0.18065	0.42832	C17orf89	76353	25kb
ENSG00000157637	17	79193800	79294347	339	39	120000	-0.71045	0.76129	SLC38A10	76353	25kb
ENSG00000185332	17	79260074	79329474	255	28	120000	-0.38918	0.65143	TMEM105	76353	25kb
ENSG00000263053	17	79344275	79400658	148	23	120000	-0.28433	0.61192	RP11-1055B8.6	76353	25kb
ENSG00000171282	17	79348540	79458357	327	48	120000	-1.1671	0.87842	RP11-1055B8.7	76353	25kb
ENSG00000184009	17	79451997	79515873	287	28	120000	-0.86104	0.80539	ACTG1	76353	25kb
ENSG00000186765	17	79470422	79529156	238	37	120000	-0.75289	0.77424	FSCN2	76353	25kb
ENSG00000185504	17	79481911	79545987	225	38	120000	-0.37443	0.64596	C17orf70	76353	25kb
ENSG00000182446	17	79498913	79640495	647	32	120000	-0.084286	0.53359	NPLOC4	76353	25kb
ENSG00000185527	17	79592489	79655142	362	22	120000	-0.22474	0.58891	PDE6G	76353	25kb
ENSG00000204237	17	79607066	79658665	311	21	120000	-0.45487	0.6754	OXLD1	76353	25kb
ENSG00000185298	17	79608394	79665934	328	21	120000	-0.53435	0.70345	CCDC137	76353	25kb
ENSG00000214087	17	79623204	79675954	282	15	120000	-1.0008	0.84154	ARL16	76353	25kb
ENSG00000185359	17	79625356	79695168	361	22	120000	-1.1172	0.86805	HGS	76353	25kb
ENSG00000262814	17	79645387	79699556	240	26	120000	-1.2055	0.88599	MRPL12	76353	25kb
ENSG00000262660	17	79645401	79712569	253	25	120000	-1.0059	0.84278	SLC25A10	76353	25kb
ENSG00000183048	17	79645404	79713042	253	25	120000	-1.0059	0.84278	SLC25A10	76353	25kb
ENSG00000215644	17	79737008	79796889	87	8	120000	0.82599	0.20441	GCGR	76353	25kb
ENSG00000225663	17	79755287	79816178	127	10	120000	0.94123	0.17329	FAM195B	76353	25kb
ENSG00000215621	17	79755293	79809533	113	9	120000	0.86709	0.19295	AC174470.1	76353	25kb
ENSG00000182676	17	79766368	79817926	118	9	120000	0.97225	0.16546	PPP1R27	76353	25kb
ENSG00000185624	17	79776035	79843570	169	11	120000	1.0359	0.15013	P4HB	76353	25kb
ENSG00000141522	17	79800597	79854282	109	10	120000	1.2352	0.10837	ARHGDIA	76353	25kb
ENSG00000183684	17	79820713	79874462	76	17	120000	1.5082	0.06575	ALYREF	76353	25kb
ENSG00000141552	17	79823666	79883867	76	19	120000	1.6001	0.054786	ANAPC11	76353	25kb
ENSG00000183979	17	79833621	79885781	52	23	120000	2.0096	0.022235	NPB	76353	25kb
ENSG00000185813	17	79833841	79894340	63	29	120000	1.7749	0.037961	PCYT2	76353	25kb
ENSG00000187531	17	79844815	79904199	60	21	120000	0.68025	0.24817	SIRT7	76353	25kb
ENSG00000197063	17	79851146	79910590	75	18	120000	-0.098002	0.53903	MAFG	76353	25kb
ENSG00000183010	17	79865260	79925288	104	14	120000	-0.92268	0.82191	PYCR1	76353	25kb
ENSG00000185105	17	79872521	79930109	105	13	120000	-1.0862	0.8613	MYADML2	76353	25kb
ENSG00000185269	17	79885388	79944716	125	15	120000	-1.4216	0.92243	NOTUM	76353	25kb
ENSG00000169696	17	79909683	80000282	207	27	120000	-1.1847	0.88192	ASPSCR1	76353	25kb
ENSG00000169689	17	79951578	80006983	134	21	120000	-0.18393	0.57297	STRA13	76353	25kb
ENSG00000169683	17	79956178	80014027	148	24	120000	0.14289	0.44319	LRRC45	76353	25kb
ENSG00000169750	17	79964500	80017080	130	20	120000	0.34391	0.36546	RAC3	76353	25kb
ENSG00000169738	17	79968012	80020608	134	21	120000	0.33874	0.3674	DCXR	76353	25kb
ENSG00000169733	17	79980778	80034707	139	22	120000	0.38341	0.35071	RFNG	76353	25kb
ENSG00000169727	17	79983567	80040346	145	25	120000	0.32308	0.37332	GPS1	76353	25kb
ENSG00000169718	17	79990382	80048763	157	28	120000	0.17621	0.43006	DUS1L	76353	25kb
ENSG00000169710	17	80011214	80081208	200	23	120000	-0.34298	0.63419	FASN	76353	25kb
ENSG00000176155	17	80034336	80195706	623	16	120000	-0.28319	0.61149	CCDC57	76353	25kb
ENSG00000141526	17	80161273	80244005	155	23	120000	0.34051	0.36673	SLC16A3	76353	25kb
ENSG00000141551	17	80171899	80256607	154	26	120000	0.029814	0.48811	CSNK1D	76353	25kb
ENSG00000268852	17	80189131	80241080	60	23	120000	0.84461	0.19916	AC132872.2	76353	25kb
ENSG00000173762	17	80247744	80300478	163	16	120000	-0.70117	0.7584	CD7	76353	25kb
ENSG00000141574	17	80253900	80316950	223	19	120000	-0.49309	0.68903	SECTM1	76353	25kb
ENSG00000182459	17	80292123	80346652	205	17	120000	0.098561	0.46074	TEX19	76353	25kb
ENSG00000181408	17	80307153	80358462	196	19	120000	0.24264	0.40414	UTS2R	76353	25kb
ENSG00000181396	17	80322099	80401513	248	30	120000	-0.44399	0.67148	OGFOD3	76353	25kb
ENSG00000169660	17	80351194	80425521	225	31	120000	-1.3603	0.91313	HEXDC	76353	25kb
ENSG00000178927	17	80375465	80433705	170	19	120000	-1.2737	0.89862	C17orf62	76353	25kb
ENSG00000141562	17	80391056	80473413	278	27	120000	-1.8334	0.96663	NARF	76353	25kb
ENSG00000141568	17	80452589	80627538	727	68	120000	-1.1168	0.86795	FOXK2	76353	25kb
ENSG00000141580	17	80547438	80631429	334	36	120000	-0.31742	0.62454	WDR45B	76353	25kb
ENSG00000141542	17	80587849	80681604	373	44	120000	0.37422	0.35412	RAB40B	76353	25kb
ENSG00000141560	17	80649559	80713204	250	26	120000	1.0084	0.15662	FN3KRP	76353	25kb
ENSG00000167363	17	80668451	80734073	209	23	120000	1.2563	0.1045	FN3K	76353	25kb
ENSG00000141556	17	80684940	80925724	871	31	120000	1.4069	0.079732	TBCD	76353	25kb
ENSG00000141579	17	80762311	80823454	207	21	120000	1.8612	0.031359	ZNF750	76353	25kb
ENSG00000175711	17	80875031	81034686	719	57	120000	1.9644	0.02474	B3GNTL1	76353	25kb
ENSG00000176845	17	81012567	81077864	254	40	120000	2.8541	0.0021581	METRNL	76353	25kb
ENSG00000173213	18	22225	98545	107	13	120000	1.4133	0.078785	RP11-683L23.1	76353	25kb
ENSG00000101557	18	133383	239629	216	27	120000	-0.4158	0.66122	USP14	76353	25kb
ENSG00000079134	18	189520	293050	221	23	120000	-0.57658	0.71789	THOC1	76353	25kb
ENSG00000158270	18	294361	525722	1095	108	120000	-2.4566	0.99299	COLEC12	76353	25kb
ENSG00000177143	18	555367	606524	229	45	120000	-0.64729	0.74128	CETN1	76353	25kb
ENSG00000079101	18	571988	675334	384	69	120000	-0.56736	0.71477	CLUL1	76353	25kb
ENSG00000176912	18	616320	683340	242	34	120000	0.065619	0.47384	C18orf56	76353	25kb
ENSG00000176890	18	632604	698578	281	23	120000	0.07854	0.4687	TYMS	76353	25kb
ENSG00000132199	18	645324	737676	375	24	120000	-0.18013	0.57147	ENOSF1	76353	25kb
ENSG00000176105	18	696588	837547	491	36	120000	0.75442	0.2253	YES1	76353	25kb
ENSG00000141433	18	879944	937173	258	36	120000	-0.99064	0.83907	ADCYAP1	76353	25kb

ENSG00000101574	18	2512524	2596508	304	24	120000	-1.3874	0.91734	METTL4	76353	25kb
ENSG00000080986	18	2546510	2641634	382	33	120000	-0.61813	0.73175	NDC80	76353	25kb
ENSG00000101596	18	2630737	2830015	792	37	120000	-0.091048	0.53627	SMCHD1	76353	25kb
ENSG00000132205	18	2822028	2940991	374	88	120000	1.8131	0.034908	EMILIN2	76353	25kb
ENSG00000101577	18	2891992	3038313	467	52	120000	1.4027	0.080356	LPIN2	76353	25kb
ENSG00000101605	18	3041805	3245106	772	82	120000	-1.383	0.91667	MYOM1	76353	25kb
ENSG00000101608	18	3222479	3281234	235	24	120000	-0.66572	0.7472	MYL12A	76353	25kb
ENSG00000118680	18	3236907	3303282	241	22	120000	-0.44244	0.67091	MYL12B	76353	25kb
ENSG00000177426	18	3386606	3483409	290	51	120000	-1.0437	0.85169	TGIF1	76353	25kb
ENSG00000170579	18	3471030	4480335	3523	327	120000	1.7259	0.042185	DLGAP1	76353	25kb
ENSG00000231824	18	5120284	5222502	413	56	120000	0.020555	0.4918	C18orf42	76353	25kb
ENSG00000198081	18	5264018	5322052	220	33	120000	-0.23545	0.59307	ZBTB14	76353	25kb
ENSG00000082397	18	5367383	5655699	836	85	120000	2.3657	0.0089973	EPB41L3	76353	25kb
ENSG00000206432	18	5857071	5920954	172	38	120000	-0.21306	0.58436	TMEM200C	76353	25kb
ENSG00000154655	18	5929705	6440236	1324	103	120000	-1.0431	0.85154	L3MBTL4	76353	25kb
ENSG00000265944	18	6486415	6615652	482	75	120000	0.10276	0.45908	C18orf64	76353	25kb
ENSG00000088756	18	6704717	6940715	709	72	120000	-2.4432	0.99272	ARHGAP28	76353	25kb
ENSG00000101680	18	6916743	7142813	792	125	120000	-0.4084	0.65851	LAMA1	76353	25kb
ENSG00000206422	18	7206123	7257045	125	19	120000	-0.97921	0.83626	LRRC30	76353	25kb
ENSG00000173482	18	7541780	8431859	2656	180	120000	0.87934	0.18961	PTPRM	76353	25kb
ENSG00000268046	18	8311437	8362038	189	20	120000	-1.3636	0.91365	AP001094.1	76353	25kb
ENSG00000206418	18	8584443	8664379	279	28	120000	-1.6563	0.95117	RAB12	76353	25kb
ENSG00000168502	18	8680659	8857776	628	80	120000	0.17797	0.42937	SOGA2	76353	25kb
ENSG00000178127	18	9077628	9159343	180	25	120000	-0.5401	0.70543	NDUFV2	76353	25kb
ENSG00000101745	18	9111226	9310983	471	26	120000	-0.28543	0.61234	ANKRD12	76353	25kb
ENSG00000128791	18	9309765	9427418	389	30	120000	0.31406	0.37674	TWSG1	76353	25kb
ENSG00000017797	18	9450007	9563114	377	32	120000	-1.034	0.84943	RALBP1	76353	25kb
ENSG00000154845	18	9521789	9640238	381	33	120000	-2.0284	0.97874	PPP4R1	76353	25kb
ENSG00000168461	18	9683162	9887548	913	129	120000	0.47554	0.3172	RAB31	76353	25kb
ENSG00000168454	18	9860763	9914272	232	37	120000	0.66718	0.25233	TXNDC2	76353	25kb
ENSG00000101558	18	9888999	9985018	269	30	120000	0.2843	0.38809	VAPA	76353	25kb
ENSG00000154856	18	10429625	10514945	280	29	120000	0.081249	0.46762	APCDD1	76353	25kb
ENSG00000134265	18	10500902	10577758	200	17	120000	0.34677	0.36438	NAPG	76353	25kb
ENSG00000154864	18	10641480	11173587	1993	162	120000	0.33939	0.36716	PIEZO2	76353	25kb
ENSG00000141404	18	11663955	11910684	869	67	120000	0.72531	0.23413	GNAL	76353	25kb
ENSG00000255112	18	11826395	11879448	200	33	120000	-0.78445	0.78361	CHMP1B	76353	25kb
ENSG00000154889	18	11857621	11934222	351	62	120000	-1.2041	0.88573	MPPE1	76353	25kb
ENSG00000141401	18	11956024	12055876	473	46	120000	-0.91746	0.82055	IMPA2	76353	25kb
ENSG00000181626	18	12068852	12154748	211	14	120000	-0.31194	0.62246	ANKRD62	76353	25kb
ENSG00000176194	18	12229318	12302594	251	42	120000	0.86772	0.19277	CIDEA	76353	25kb
ENSG00000176014	18	12282668	12369319	252	14	120000	0.20893	0.41725	TUBB6	76353	25kb
ENSG00000141385	18	12303943	12402313	268	21	120000	-0.091858	0.53659	AFG3L2	76353	25kb
ENSG00000141391	18	12382895	12457237	223	36	120000	-0.99177	0.83935	SLMO1	76353	25kb
ENSG00000134278	18	12421511	12683133	739	38	120000	0.099606	0.46033	SPIRE1	76353	25kb
ENSG00000215527	18	12633042	12688470	147	15	120000	0.28395	0.38823	AP005482.1	76353	25kb
ENSG00000128789	18	12633737	12750739	318	23	120000	0.065277	0.47398	PSMG2	76353	25kb
ENSG00000101624	18	12636832	12727776	229	17	120000	0.2355	0.40691	CEP76	76353	25kb
ENSG00000175354	18	12760477	12954642	536	48	120000	-1.5635	0.94104	PTPN2	76353	25kb
ENSG00000085415	18	12922132	13012535	249	27	120000	0.66851	0.2519	SEH1L	76353	25kb
ENSG00000101639	18	12966361	13150051	526	23	120000	0.6356	0.26252	CEP192	76353	25kb
ENSG00000168675	18	13192497	13677754	1384	166	120000	0.15228	0.43948	LDLRAD4	76353	25kb
ENSG00000177150	18	13638346	13751662	331	23	120000	0.1593	0.43672	FAM210A	76353	25kb
ENSG00000101654	18	13701659	13789557	246	28	120000	-0.28968	0.61397	RNMT	76353	25kb
ENSG00000176136	18	13799154	13851861	303	24	120000	-0.26333	0.60385	MC5R	76353	25kb
ENSG00000185231	18	13857043	13940706	314	26	120000	-0.97223	0.83453	MC2R	76353	25kb
ENSG00000175322	18	14032456	14157489	287	22	120000	1.13	0.12924	ZNF519	76353	25kb
ENSG00000183206	18	14482338	14568584	99	15	120000	-0.54362	0.70665	POTEC	76353	25kb
ENSG00000180777	18	14703271	14877737	344	20	120000	-0.56467	0.71385	ANKRD30B	76353	25kb
ENSG00000067900	18	18501867	18716812	159	17	120000	-1.0636	0.85623	ROCK1	76353	25kb
ENSG00000141449	18	18797203	19130378	353	24	120000	-0.26248	0.60353	GREB1L	76353	25kb
ENSG00000141446	18	19084242	19205845	139	15	120000	-0.13831	0.555	ESCO1	76353	25kb
ENSG00000167088	18	19167228	19235417	89	10	120000	-0.31277	0.62277	SNRPD1	76353	25kb
ENSG00000158201	18	19205858	19309766	131	15	120000	-0.43006	0.66642	ABHD3	76353	25kb
ENSG00000101752	18	19259918	19475918	310	22	120000	-0.14764	0.55869	MIB1	76353	25kb
ENSG00000263588	18	19643021	19703741	213	23	120000	-0.14793	0.5588	RP11-595B24.2	76353	25kb
ENSG00000141448	18	19724404	19807491	111	27	120000	0.19348	0.42329	GATA6	76353	25kb
ENSG00000212710	18	19968564	20022878	106	29	120000	0.17883	0.42904	CTAGE1	76353	25kb
ENSG00000101773	18	20353224	20631451	611	20	120000	0.10123	0.45968	RBBP8	76353	25kb
ENSG00000134508	18	20689528	20865431	384	53	120000	1.2069	0.11374	CABLES1	76353	25kb
ENSG00000134490	18	20752108	21042925	602	53	120000	0.18313	0.42735	TMEM241	76353	25kb
ENSG00000101782	18	21007787	21091567	221	28	120000	-0.69217	0.75558	RIOK3	76353	25kb
ENSG00000141452	18	21058473	21136746	166	19	120000	-0.26875	0.60594	C18orf8	76353	25kb
ENSG00000141458	18	21061148	21191862	278	21	120000	0.0041051	0.49836	NPC1	76353	25kb
ENSG00000154065	18	21153890	21267849	204	34	120000	-0.20906	0.5828	ANKRD29	76353	25kb
ENSG00000053747	18	21244407	21560030	558	23	120000	-1.0663	0.85685	LAMA3	76353	25kb
ENSG00000168234	18	21547737	21740574	467	49	120000	0.98962	0.16118	TTC39C	76353	25kb
ENSG00000154040	18	21693942	21766567	154	17	120000	2.0747	0.019005	CABYR	76353	25kb
ENSG00000141447	18	21717008	22002844	697	88	120000	1.971	0.02436	OSBPL1A	76353	25kb
ENSG00000154059	18	21981580	22058499	234	24	120000	0.80089	0.2116	IMPACT	76353	25kb
ENSG00000134489	18	22015593	22084921	196	35	120000	0.76183	0.22308	HRH4	76353	25kb
ENSG00000198795	18	22616890	22957154	874	92	120000	-0.52833	0.70136	ZNF521	76353	25kb
ENSG00000141380	18	23571578	23696181	272	15	120000	1.9106	0.028028	SS18	76353	25kb
ENSG00000154611	18	23688816	23798319	233	18	120000	1.6174	0.052891	PSMA8	76353	25kb
ENSG00000141384	18	23780900	23996649	526	30	120000	0.076963	0.46933	TAF4B	76353	25kb
ENSG00000134504	18	24009874	24262365	751	108	120000	-0.83541	0.79826	KCTD1	76353	25kb
ENSG00000171885	18	24407002	24470782	189	40	120000	2.7769	0.0027437	AQP4	76353	25kb
ENSG00000154080	18	24470595	24790281	858	53	120000	-0.80498	0.78958	CHST9	76353	25kb
ENSG00000170558	18	25505930	25782410	572	50	120000	-0.66576	0.74722	CDH2	76353	25kb
ENSG00000134762	18	28544974	28647781	301	34	120000	-0.84163	0.8	DSC3	76353	25kb
ENSG00000134755	18	28620940	28707378	199	29	120000	-0.51682	0.69736	DSC2	76353	25kb
ENSG00000134765	18	28684199	28767819	289	19	120000	-1.1386	0.87256	DSC1	76353	25kb
ENSG00000134760	18	28873052	28961992	344	20	120000	-0.75745	0.77561	DSG1	76353	25kb
ENSG00000175065	18	28931740	29019875	192	21	120000	-0.34996	0.63681	DSG4	76353	25kb
ENSG00000134757	18	29002758	29083665	294	27	120000	-0.39292	0.65281	DSG3	76353	25kb
ENSG00000046604	18	29053006	29153971	297	30	120000	-0.6482	0.74157	DSG2	76353	25kb
ENSG00000118271	18	29146689	29203974	127	14	120000	-1.0493	0.85298	TTR	76353	25kb

ENSG00000118276	18	29177210	29290799	240	29	120000	-1.3836	0.91676	B4GALT6	76353	25kb
ENSG00000141437	18	29314525	29365843	152	20	120000	0.82252	0.20539	SLC25A52	76353	25kb
ENSG00000153339	18	29384136	29558099	467	34	120000	2.9374	0.0016547	TRAPPC8	76353	25kb
ENSG00000101695	18	29573335	29678176	241	43	120000	0.52677	0.29918	RNF125	76353	25kb
ENSG00000134758	18	29646818	29736524	198	22	120000	-0.56126	0.71269	RNF138	76353	25kb
ENSG00000141441	18	29679840	30075447	1020	59	120000	0.15824	0.43713	GAREM	76353	25kb
ENSG00000141434	18	29740032	29825367	206	29	120000	0.55677	0.28884	MEP1B	76353	25kb
ENSG00000197705	18	30227634	30378025	352	19	120000	1.0736	0.14151	KLHL14	76353	25kb
ENSG00000228835	18	30324758	30379376	86	16	120000	0.90517	0.18269	AC012123.1	76353	25kb
ENSG00000166960	18	30492366	31046065	1156	30	120000	0.11785	0.45309	CCDC178	76353	25kb
ENSG00000141431	18	31133579	31356156	501	36	120000	-0.71978	0.76417	ASXL3	76353	25kb
ENSG00000101746	18	31406064	31829916	877	56	120000	0.70497	0.24041	NOL4	76353	25kb
ENSG00000134769	18	32048254	32496808	1018	38	120000	0.30773	0.37914	DTNA	76353	25kb
ENSG00000166974	18	32531892	32748434	498	44	120000	-0.89889	0.81565	MAPRE2	76353	25kb
ENSG00000186812	18	32795994	32872097	169	27	120000	-0.6025	0.72658	ZNF397	76353	25kb
ENSG00000186814	18	32806032	32895196	177	26	120000	-0.56753	0.71482	ZSCAN30	76353	25kb
ENSG00000172466	18	32887176	32950446	123	16	120000	0.02853	0.48862	ZNF24	76353	25kb
ENSG00000186496	18	32921661	32982301	132	17	120000	0.20168	0.42008	ZNF396	76353	25kb
ENSG00000267140	18	32998835	33102904	205	35	120000	-0.46954	0.68066	RP11-322E11.6	76353	25kb
ENSG00000153391	18	33007194	33102955	192	32	120000	-0.48485	0.68611	INO80C	76353	25kb
ENSG00000141429	18	33136081	33316798	503	23	120000	-0.81801	0.79332	GALNT1	76353	25kb
ENSG00000141428	18	33527046	33584241	104	10	120000	-0.93226	0.8244	C18orf21	76353	25kb
ENSG00000141425	18	33539350	33672539	269	21	120000	-0.5952	0.72415	RPRD1A	76353	25kb
ENSG00000141424	18	33663495	33734348	214	26	120000	-0.40063	0.65565	SLC39A6	76353	25kb
ENSG00000134759	18	33684407	33782909	263	19	120000	-0.52694	0.70088	ELP2	76353	25kb
ENSG00000075643	18	33742482	33877120	377	54	120000	0.073474	0.47071	MOCOS	76353	25kb
ENSG00000134775	18	33852677	34385018	1292	97	120000	1.7549	0.039635	FHOD3	76353	25kb
ENSG00000134779	18	34334987	34434158	170	12	120000	-0.25896	0.60217	TPGS2	76353	25kb
ENSG00000150477	18	34384069	34837135	858	20	120000	-1.361	0.91324	KIAA1328	76353	25kb
ENSG00000101489	18	34798010	35171000	968	107	120000	-1.7851	0.96287	CELF4	76353	25kb
ENSG00000078142	18	39510171	39692794	298	19	120000	1.004	0.15769	PIK3C3	76353	25kb
ENSG00000152214	18	40298192	40720657	1094	70	120000	-0.4294	0.66618	RIT2	76353	25kb
ENSG00000132872	18	40822843	40882615	87	14	120000	-0.40807	0.65839	SYT4	76353	25kb
ENSG00000152217	18	42235138	42673475	836	91	120000	-0.058887	0.52348	SETBP1	76353	25kb
ENSG00000132874	18	42767960	43288072	1636	109	120000	0.2787	0.39024	SLC14A2	76353	25kb
ENSG00000141469	18	43279092	43357485	366	26	120000	-0.085143	0.53393	SLC14A1	76353	25kb
ENSG00000197046	18	43380477	43449045	148	20	120000	0.55221	0.2904	SIGLEC15	76353	25kb
ENSG00000152223	18	43402574	43572240	450	33	120000	1.8896	0.029409	EPG5	76353	25kb
ENSG00000152229	18	43538502	43677238	415	26	120000	-0.071111	0.52835	PSTPIP2	76353	25kb
ENSG00000152234	18	43639110	43709300	224	17	120000	-1.6387	0.94936	ATP5A1	76353	25kb
ENSG00000152240	18	43659298	43733299	229	20	120000	-1.9508	0.97446	HAUS1	76353	25kb
ENSG00000152242	18	43728519	43871949	238	17	120000	-1.5851	0.94353	C18orf25	76353	25kb
ENSG00000141622	18	43881772	44068103	504	76	120000	0.77535	0.21907	RNF165	76353	25kb
ENSG00000167210	18	44031935	44261996	734	87	120000	1.5203	0.064224	LOXHD1	76353	25kb
ENSG00000101638	18	44234081	44364220	467	74	120000	0.70539	0.24028	ST8SIA5	76353	25kb
ENSG00000078043	18	44363353	44525123	414	19	120000	1.9361	0.026428	PIAS2	76353	25kb
ENSG00000167216	18	44472455	44652658	454	27	120000	2.3197	0.010178	KATNAL2	76353	25kb
ENSG00000266996	18	44517731	44569607	130	17	120000	2.6601	0.0039062	TCEB3CL2	76353	25kb
ENSG00000234298	18	44523658	44575534	120	17	120000	2.5066	0.0060955	TCEB3CL	76353	25kb
ENSG00000183791	18	44529573	44581449	119	16	120000	2.5899	0.0048001	TCEB3C	76353	25kb
ENSG00000206181	18	44533943	44586988	121	16	120000	2.6951	0.0035183	TCEB3B	76353	25kb
ENSG00000167220	18	44608774	44701891	267	17	120000	0.68469	0.24677	HDHD2	76353	25kb
ENSG00000267228	18	44636027	44727652	285	18	120000	0.68011	0.24822	IER3IP1	76353	25kb
ENSG00000134049	18	44656413	44727745	231	20	120000	0.55526	0.28936	IER3IP1	76353	25kb
ENSG00000215474	18	44721293	44800554	205	22	120000	0.83528	0.20178	SKOR2	76353	25kb
ENSG00000175387	18	45332922	45482515	265	14	120000	-0.58831	0.72184	SMAD2	76353	25kb
ENSG00000184828	18	45528044	45962123	1555	166	120000	-1.5464	0.93899	ZBTB7C	76353	25kb
ENSG00000268960	18	45753672	45804208	215	35	120000	-0.68009	0.75178	AC091150.1	76353	25kb
ENSG00000134030	18	46040417	46414588	1186	145	120000	-1.7519	0.9601	CTIF	76353	25kb
ENSG00000101665	18	46421223	46502081	241	48	120000	2.0409	0.020632	SMAD7	76353	25kb
ENSG00000141627	18	46545039	47012717	1159	34	120000	-0.96972	0.83391	DYM	76353	25kb
ENSG00000177576	18	46983028	47038622	220	23	120000	-1.1524	0.87542	C18orf32	76353	25kb
ENSG00000215472	18	46983051	47042956	238	24	120000	-1.0761	0.85906	RPL17-C18orf32	76353	25kb
ENSG00000265681	18	46989851	47043906	217	21	120000	-0.94878	0.82863	RPL17	76353	25kb
ENSG00000101670	18	47062069	47144272	208	37	120000	0.72773	0.23339	LIPG	76353	25kb
ENSG00000167315	18	47284869	47365330	212	24	120000	1.0686	0.14261	ACAA2	76353	25kb
ENSG00000266997	18	47297163	47388993	210	22	120000	0.99389	0.16014	RP11-886H22.1	76353	25kb
ENSG00000167306	18	47324183	47746463	1508	72	120000	-0.088681	0.53533	MYO5B	76353	25kb
ENSG00000172361	18	47728563	47817892	185	23	120000	0.16812	0.43325	CCDC11	76353	25kb
ENSG00000141644	18	47768252	47833144	153	22	120000	0.21748	0.41392	MBD1	76353	25kb
ENSG00000154832	18	47783713	47839674	134	24	120000	0.077431	0.46914	CXXC1	76353	25kb
ENSG00000154839	18	47876365	47945543	196	15	120000	1.5402	0.061752	SKA1	76353	25kb
ENSG00000141639	18	48061448	48283194	548	57	120000	-0.82165	0.79436	MAPK4	76353	25kb
ENSG00000134042	18	48299574	48376772	287	48	120000	0.17673	0.42986	MRO	76353	25kb
ENSG00000082212	18	48380419	48499691	237	22	120000	-0.73963	0.77024	ME2	76353	25kb
ENSG00000141642	18	48469361	48539491	102	14	120000	-1.2571	0.89564	ELAC1	76353	25kb
ENSG00000267699	18	48469389	48609514	172	16	120000	-1.1022	0.8648	RP11-729L2.2	76353	25kb
ENSG00000141646	18	48469410	48636415	201	17	120000	-1.1083	0.86613	SMAD4	76353	25kb
ENSG00000176624	18	48675920	48769674	205	17	120000	0.68614	0.24631	MEX3C	76353	25kb
ENSG00000187323	18	49841542	51082784	4657	127	120000	-0.18744	0.57434	DCC	76353	25kb
ENSG00000134046	18	51654079	51776158	237	29	120000	-0.64591	0.74083	MBD2	76353	25kb
ENSG00000101751	18	51770774	51872636	292	15	120000	-1.2759	0.89901	POLI	76353	25kb
ENSG00000174448	18	51825728	51909334	251	17	120000	-1.6507	0.9506	STARD6	76353	25kb
ENSG00000166845	18	51859287	51936588	248	13	120000	-1.5866	0.94369	C18orf54	76353	25kb
ENSG00000178690	18	52229988	52291724	129	17	120000	0.33409	0.36916	DYNAP	76353	25kb
ENSG00000041353	18	52360091	52587747	716	39	120000	0.83868	0.20082	RAB27B	76353	25kb
ENSG00000166510	18	52543740	52651739	272	23	120000	1.1645	0.1221	CCDC68	76353	25kb
ENSG00000196628	18	52864562	53357018	828	54	120000	-1.3406	0.90997	TCF4	76353	25kb
ENSG00000091164	18	54239439	54343831	292	20	120000	0.60528	0.27249	TXNL1	76353	25kb
ENSG00000091157	18	54293574	54723828	1211	60	120000	-0.87932	0.81039	WDR7	76353	25kb
ENSG00000228075	18	54789293	54842531	158	23	120000	-1.2424	0.89295	BOD1L2	76353	25kb
ENSG00000177511	18	54993044	55063962	151	14	120000	-0.25029	0.59882	ST8SIA3	76353	25kb
ENSG00000119547	18	55077917	55183529	426	45	120000	0.79691	0.21275	ONECUT2	76353	25kb
ENSG00000066926	18	55190515	55279004	278	47	120000	-0.26508	0.60452	FECH	76353	25kb
ENSG00000134440	18	55242888	55314445	233	35	120000	0.89459	0.1855	NARS	76353	25kb
ENSG00000081923	18	55288658	55495333	644	76	120000	0.67049	0.25127	ATP8B1	76353	25kb

ENSG00000049759	18	55686599	56093772	1245	107	120000	-1.0178	0.84561	NEDD4L	76353	25kb
ENSG00000198796	18	56123479	56321189	714	77	120000	1.2499	0.10567	ALPK2	76353	25kb
ENSG00000172175	18	56313618	56442371	336	26	120000	1.4949	0.067469	MALT1	76353	25kb
ENSG00000074657	18	56504832	56678712	435	39	120000	-1.2344	0.89146	ZNF532	76353	25kb
ENSG00000166562	18	56781709	56851068	239	26	120000	0.48261	0.31469	SEC11C	76353	25kb
ENSG00000134443	18	56862400	56923006	235	34	120000	-1.3602	0.91311	GRP	76353	25kb
ENSG00000134438	18	56909267	56966318	207	24	120000	-0.55443	0.71036	RAX	76353	25kb
ENSG00000166569	18	56917388	57010881	296	28	120000	-0.43962	0.66989	CPLX4	76353	25kb
ENSG00000074695	18	56970055	57052194	246	21	120000	-0.36329	0.6418	LMAN1	76353	25kb
ENSG00000183287	18	57073172	57389612	1225	116	120000	-0.72096	0.76453	CCBE1	76353	25kb
ENSG00000141682	18	57542180	57596538	143	22	120000	-0.25982	0.6025	PMAIP1	76353	25kb
ENSG00000166603	18	58013564	58065001	157	26	120000	0.049455	0.48028	MC4R	76353	25kb
ENSG00000101542	18	58975815	59248006	706	123	120000	-1.2117	0.88718	CDH20	76353	25kb
ENSG00000176641	18	59450296	59586480	498	43	120000	-0.61373	0.7303	RNF152	76353	25kb
ENSG00000197563	18	59685800	59879351	718	23	120000	0.91243	0.18077	PIGN	76353	25kb
ENSG00000134444	18	59829491	59999355	534	22	120000	1.0576	0.14511	KIAA1468	76353	25kb
ENSG00000141655	18	59967520	60083516	462	51	120000	-0.049656	0.5198	TNFRSF11A	76353	25kb
ENSG00000141664	18	60165240	60279942	275	30	120000	-0.67763	0.751	ZCCHC2	76353	25kb
ENSG00000081913	18	60357672	60672666	570	30	120000	0.035123	0.48599	PHLPP1	76353	25kb
ENSG00000215431	18	60466423	60517724	74	8	120000	0.9925	0.16048	AC015989.1	76353	25kb
ENSG00000269630	18	60480640	60530948	79	13	120000	0.68369	0.24708	AC015989.2	76353	25kb
ENSG00000171791	18	60765579	61012361	720	121	120000	-0.39929	0.65516	BCL2	76353	25kb
ENSG00000119537	18	60969959	61059743	190	31	120000	-1.6011	0.94532	KDSR	76353	25kb
ENSG00000119541	18	61031423	61114716	202	21	120000	0.82575	0.20447	VPS4B	76353	25kb
ENSG00000206075	18	61118994	61197318	314	25	120000	-0.051788	0.52065	SERPINB5	76353	25kb
ENSG00000166634	18	61198393	61259244	192	11	120000	-0.15811	0.56281	SERPINB12	76353	25kb
ENSG00000197641	18	61229223	61296873	161	11	120000	-0.47914	0.68408	SERPINB13	76353	25kb
ENSG00000206073	18	61279493	61336532	67	12	120000	-0.33669	0.63182	SERPINB4	76353	25kb
ENSG00000057149	18	61297431	61354197	68	13	120000	-0.25877	0.6021	SERPINB3	76353	25kb
ENSG00000166396	18	61395169	61497604	461	25	120000	-0.44543	0.672	SERPINB7	76353	25kb
ENSG00000197632	18	61513926	61596124	282	7	120000	0.53959	0.29474	SERPINB2	76353	25kb
ENSG00000242550	18	61539408	61628345	330	12	120000	0.80675	0.2099	SERPINB10	76353	25kb
ENSG00000268706	18	61577042	61634601	221	13	120000	0.96999	0.16603	AC009802.1	76353	25kb
ENSG00000221887	18	61591535	61674008	238	29	120000	0.72646	0.23378	HMSD	76353	25kb
ENSG00000166401	18	61612159	61697278	230	36	120000	0.10451	0.45838	SERPINB8	76353	25kb
ENSG00000081138	18	63392488	63573638	579	27	120000	-0.42326	0.66395	CDH7	76353	25kb
ENSG00000071991	18	64143320	64296375	317	27	120000	-1.3185	0.90633	CDH19	76353	25kb
ENSG00000171451	18	65148819	65209217	122	22	120000	0.73005	0.23268	DSEL	76353	25kb
ENSG00000166479	18	66315925	66407535	196	17	120000	-0.65272	0.74303	TMX3	76353	25kb
ENSG00000150636	18	66357446	66747426	1189	66	120000	-0.32251	0.62647	CCDC102B	76353	25kb
ENSG00000264472	18	66542018	66594492	174	21	120000	0.26407	0.39586	RP11-861L17.3	76353	25kb
ENSG00000206052	18	67043291	67541323	1829	144	120000	-1.5204	0.93579	DOK6	76353	25kb
ENSG00000150637	18	67473394	67654039	478	52	120000	-0.75905	0.77609	CD226	76353	25kb
ENSG00000176225	18	67646029	67898181	625	35	120000	-1.2563	0.89549	RTTN	76353	25kb
ENSG00000170677	18	67931137	68022436	314	35	120000	-0.3635	0.64188	SOCS6	76353	25kb
ENSG00000266258	18	67977675	68044695	215	28	120000	0.36397	0.35794	RP11-41O4.1	76353	25kb
ENSG00000263417	18	68272755	68342980	223	27	120000	0.18193	0.42782	GTSCR1	76353	25kb
ENSG00000265352	18	69374851	69480364	488	27	120000	-0.2788	0.6098	RP11-723G8.2	76353	25kb
ENSG00000141668	18	70178915	70330756	453	45	120000	1.5825	0.056763	CBLN2	76353	25kb
ENSG00000166342	18	70384549	70560381	688	57	120000	-1.0892	0.86196	NETO1	76353	25kb
ENSG00000263711	18	70793476	70956715	515	52	120000	0.095403	0.462	RP11-169F17.1	76353	25kb
ENSG00000141665	18	71715588	71840100	380	15	120000	-0.28936	0.61385	FBXO15	76353	25kb
ENSG00000075336	18	71790746	71851197	225	17	120000	-0.20417	0.58089	TIMM21	76353	25kb
ENSG00000166347	18	71895530	71984251	394	23	120000	3.411	0.00032363	CYB5A	76353	25kb
ENSG00000206043	18	71958048	72051422	387	23	120000	3.6398	0.00013641	C18orf63	76353	25kb
ENSG00000187773	18	72077963	72150179	245	46	120000	1.333	0.091272	FAM69C	76353	25kb
ENSG00000133313	18	72138051	72213366	244	38	120000	0.23077	0.40875	CNDP2	76353	25kb
ENSG00000150656	18	72176675	72279448	332	66	120000	1.4148	0.078565	CNDP1	76353	25kb
ENSG00000215421	18	72240106	72802627	1107	55	120000	-0.19493	0.57728	ZNF407	76353	25kb
ENSG00000180011	18	72882063	72946303	221	26	120000	0.29685	0.38329	ZADH2	76353	25kb
ENSG00000179981	18	72897710	73026905	469	60	120000	-0.63973	0.73883	TSHZ1	76353	25kb
ENSG00000264116	18	73093967	73155995	196	27	120000	1.0743	0.14133	RP11-321M21.3	76353	25kb
ENSG00000206026	18	73096431	73164658	216	27	120000	0.92714	0.17693	SMIM21	76353	25kb
ENSG00000266614	18	73946121	73997069	161	17	120000	0.49226	0.31127	RP11-94B19.4	76353	25kb
ENSG00000101493	18	74044644	74232146	557	71	120000	-0.51641	0.69721	ZNF516	76353	25kb
ENSG00000220032	18	74182477	74235045	147	32	120000	-0.69856	0.75759	RP11-17M16.1	76353	25kb
ENSG00000263812	18	74215612	74347925	628	79	120000	-0.021745	0.50867	LINC00908	76353	25kb
ENSG00000264278	18	74481688	74559251	282	29	120000	-1.0721	0.85817	RP11-162A12.2	76353	25kb
ENSG00000130856	18	74509563	74707683	550	25	120000	-0.38697	0.65061	ZNF236	76353	25kb
ENSG00000197971	18	74665783	74870639	909	126	120000	-1.9227	0.97274	MBP	76353	25kb
ENSG00000166573	18	74937505	75005858	395	32	120000	-1.8265	0.96611	GALR1	76353	25kb
ENSG00000256463	18	76715275	76787677	194	40	120000	-0.60352	0.72692	SALL3	76353	25kb
ENSG00000166377	18	76804285	77163283	1139	35	120000	0.93333	0.17532	ATP9B	76353	25kb
ENSG00000131196	18	77130856	77314325	844	74	120000	-0.25876	0.60209	NFATC1	76353	25kb
ENSG00000269679	18	77250812	77301057	247	31	120000	-0.53471	0.70357	AC018445.1	76353	25kb
ENSG00000060069	18	77414801	77539510	514	23	120000	-0.72084	0.7645	CTDP1	76353	25kb
ENSG00000178342	18	77598668	77685184	253	23	120000	-0.56399	0.71362	KCNG2	76353	25kb
ENSG00000122490	18	77637420	77736664	273	27	120000	-0.76313	0.77731	PQLC1	76353	25kb
ENSG00000226742	18	77699561	77755822	177	23	120000	-0.47443	0.6824	HSBP1L1	76353	25kb
ENSG00000141759	18	77707867	77818949	358	21	120000	-0.90221	0.81653	TXNL4A	76353	25kb
ENSG00000267127	18	77769346	77879968	415	21	120000	-1.1547	0.8759	RP11-795F19.5	76353	25kb
ENSG00000101546	18	77769358	77831397	236	20	120000	-1.641	0.9496	RBFA	76353	25kb
ENSG00000101544	18	77841915	77930406	300	20	120000	-0.67225	0.74929	ADNP2	76353	25kb
ENSG00000267270	18	77880807	77961315	232	23	120000	-0.52796	0.70124	AC139100.2	76353	25kb
ENSG00000178184	18	77890115	78030429	318	21	120000	-0.41852	0.66222	PARD6G	76353	25kb
ENSG00000141934	19	256040	316393	187	30	120000	1.0889	0.1381	PPAP2C	76353	25kb
ENSG00000105556	19	280575	369798	353	33	120000	1.2749	0.10117	MIER2	76353	25kb
ENSG00000105549	19	336750	401013	249	56	120000	-0.15595	0.56196	THEG	76353	25kb
ENSG00000183186	19	380438	434139	218	45	120000	-1.1708	0.87916	C2CD4C	76353	25kb
ENSG00000129946	19	391583	485996	398	57	120000	-1.9029	0.97148	SHC2	76353	25kb
ENSG00000181781	19	438346	499983	295	39	120000	-2.2859	0.98887	ODF3L2	76353	25kb
ENSG00000099866	19	464176	530347	308	62	120000	-2.5726	0.99495	MADCAM1	76353	25kb
ENSG00000141933	19	482299	544654	320	64	120000	-2.3989	0.99178	TPGS1	76353	25kb
ENSG00000099804	19	506714	567097	308	54	120000	-1.5288	0.93684	CDC34	76353	25kb
ENSG00000197540	19	519034	574919	293	54	120000	-1.4414	0.92527	GZMM	76353	25kb
ENSG00000172270	19	546297	608493	362	50	120000	-0.94143	0.82676	BSG	76353	25kb

ENSG00000099822	19	564893	642159	422	67	120000	0.044919	0.48209	HCN2	76353	25kb
ENSG00000099821	19	592223	658597	334	59	120000	0.29939	0.38232	POLRMT	76353	25kb
ENSG00000070388	19	614879	668703	231	46	120000	0.39217	0.34747	FGF22	76353	25kb
ENSG00000070423	19	622526	688277	274	50	120000	0.53841	0.29515	RNF126	76353	25kb
ENSG00000070404	19	651392	708385	253	34	120000	1.6184	0.052785	FSTL3	76353	25kb
ENSG00000185198	19	660521	720460	276	40	120000	2.2053	0.013717	PRSS57	76353	25kb
ENSG00000099864	19	683953	773329	471	83	120000	1.8553	0.031779	PALM	76353	25kb
ENSG00000099812	19	726126	789319	331	71	120000	-0.01215	0.50485	MISP	76353	25kb
ENSG00000011304	19	772075	837327	300	42	120000	-0.72563	0.76597	PTBP1	76353	25kb
ENSG00000129951	19	787518	846967	265	37	120000	-0.55783	0.71152	LPPR3	76353	25kb
ENSG00000172232	19	800097	857017	236	47	120000	-0.45228	0.67446	AZU1	76353	25kb
ENSG00000196415	19	815963	873175	234	52	120000	0.85988	0.19493	PRTN3	76353	25kb
ENSG00000197561	19	826014	881242	216	48	120000	1.2756	0.10105	ELANE	76353	25kb
ENSG00000197766	19	834453	888453	212	42	120000	1.5424	0.061487	CFD	76353	25kb
ENSG00000175221	19	842962	918218	250	51	120000	1.5414	0.061611	MED16	76353	25kb
ENSG00000198858	19	871503	938240	226	52	120000	1.4499	0.073545	R3HDM4	76353	25kb
ENSG00000116014	19	892287	946015	194	44	120000	0.49882	0.30895	KISS1R	76353	25kb
ENSG00000116017	19	900781	1000939	419	55	120000	-0.74729	0.77256	ARID3A	76353	25kb
ENSG00000065268	19	958174	1019569	372	38	120000	-1.4643	0.92844	WDR18	76353	25kb
ENSG00000269169	19	974601	1024952	351	39	120000	-1.4029	0.91967	AC004528.1	76353	25kb
ENSG00000116032	19	975418	1034731	393	42	120000	-1.2671	0.89745	GRIN3B	76353	25kb
ENSG00000182087	19	984647	1046117	425	47	120000	-0.39138	0.65224	TMEM259	76353	25kb
ENSG00000064666	19	1001298	1064068	381	49	120000	0.11052	0.456	CNN2	76353	25kb
ENSG00000064687	19	1015102	1090571	372	53	120000	0.098473	0.46078	ABCA7	76353	25kb
ENSG00000180448	19	1040922	1111627	295	47	120000	-0.18803	0.57457	HMHA1	76353	25kb
ENSG00000099817	19	1061594	1120598	252	42	120000	-0.90645	0.81765	POLR2E	76353	25kb
ENSG00000167468	19	1078936	1131787	227	41	120000	-0.86521	0.80654	GPX4	76353	25kb
ENSG00000064932	19	1082636	1199282	451	59	120000	-0.85547	0.80385	SBNO2	76353	25kb
ENSG00000118046	19	1164406	1253428	254	40	120000	-0.31565	0.62386	STK11	76353	25kb
ENSG00000099625	19	1203286	1263026	144	28	120000	-0.59509	0.72411	C19orf26	76353	25kb
ENSG00000099624	19	1216749	1269824	126	28	120000	-0.67067	0.74878	ATP5D	76353	25kb
ENSG00000167470	19	1223552	1284142	127	32	120000	-0.93538	0.8252	MIDN	76353	25kb
ENSG00000099622	19	1234384	1299879	122	34	120000	-0.97985	0.83642	CIRBP	76353	25kb
ENSG00000228300	19	1250026	1304248	103	28	120000	-1.0672	0.85706	C19orf24	76353	25kb
ENSG00000160953	19	1260890	1403430	373	67	120000	0.0016893	0.49933	MUM1	76353	25kb
ENSG00000099617	19	1261153	1326430	143	35	120000	0.39511	0.34638	EFNA2	76353	25kb
ENSG00000115286	19	1358526	1420583	159	27	120000	-0.53613	0.70406	NDUFS7	76353	25kb
ENSG00000130005	19	1372091	1426569	119	25	120000	-0.17128	0.568	GAMT	76353	25kb
ENSG00000071626	19	1382568	1460719	176	36	120000	-0.63348	0.73679	DAZAP1	76353	25kb
ENSG00000115268	19	1413358	1465583	112	24	120000	-0.1925	0.57632	RPS15	76353	25kb
ENSG00000268798	19	1415838	1466937	106	23	120000	-0.21874	0.58658	AC027307.3	76353	25kb
ENSG00000115266	19	1421300	1498243	191	39	120000	0.089683	0.46427	APC2	76353	25kb
ENSG00000119559	19	1436142	1504555	176	33	120000	-0.00025522	0.5001	C19orf25	76353	25kb
ENSG00000115257	19	1456427	1515751	158	31	120000	0.25408	0.39972	PCSK4	76353	25kb
ENSG00000115255	19	1466165	1522926	162	32	120000	0.30931	0.37854	REEP6	76353	25kb
ENSG00000185761	19	1480017	1538603	222	39	120000	-0.4759	0.68293	ADAMTSL5	76353	25kb
ENSG00000185988	19	1499073	1560455	210	39	120000	-0.76411	0.7776	PLK5	76353	25kb
ENSG00000181588	19	1529668	1593057	160	34	120000	-1.713	0.95664	MEX3D	76353	25kb
ENSG00000071655	19	1551639	1617882	99	23	120000	-0.647	0.74118	MBD3	76353	25kb
ENSG00000267059	19	1553338	1630444	123	29	120000	-0.69739	0.75722	UQCR11	76353	25kb
ENSG00000127540	19	1572171	1630480	100	21	120000	-0.71	0.76115	UQCR11	76353	25kb
ENSG00000071564	19	1584291	1677604	188	43	120000	-0.8996	0.81583	TCF3	76353	25kb
ENSG00000205922	19	1727372	1805987	247	61	120000	-0.71518	0.76275	ONECUT3	76353	25kb
ENSG00000130270	19	1757074	1837275	226	52	120000	-1.4519	0.92674	ATP8B3	76353	25kb
ENSG00000079313	19	1790248	1873452	205	25	120000	-0.45335	0.67485	REXO1	76353	25kb
ENSG00000129911	19	1827399	1888567	146	16	120000	-0.45551	0.67563	KLF16	76353	25kb
ENSG00000129968	19	1851809	1910546	144	20	120000	-1.4615	0.92806	ABHD17A	76353	25kb
ENSG00000227500	19	1880213	1951016	230	37	120000	-1.4565	0.92737	SCAMP4	76353	25kb
ENSG00000213638	19	1880377	1938444	183	31	120000	-1.8215	0.96573	ADAT3	76353	25kb
ENSG00000133275	19	1916188	2006337	327	28	120000	0.34556	0.36484	CSNK1G2	76353	25kb
ENSG00000133243	19	1960447	2059880	370	57	120000	1.01	0.15625	BTBD2	76353	25kb
ENSG00000099875	19	2012470	2076243	221	47	120000	0.46983	0.31924	MKNK2	76353	25kb
ENSG00000172081	19	2046037	2121672	234	46	120000	-1.0919	0.86256	MOB3A	76353	25kb
ENSG00000099840	19	2071380	2124592	174	27	120000	-0.9499	0.82892	IZUMO4	76353	25kb
ENSG00000065000	19	2075988	2189464	413	39	120000	0.023971	0.49044	AP3D1	76353	25kb
ENSG00000104885	19	2139148	2257577	396	39	120000	0.14913	0.44072	DOT1L	76353	25kb
ENSG00000104886	19	2205002	2262703	165	39	120000	-0.71003	0.76116	PLEKHJ1	76353	25kb
ENSG00000104897	19	2211520	2273678	193	42	120000	-1.0075	0.84316	SF3A2	76353	25kb
ENSG00000104899	19	2224308	2277072	167	35	120000	-1.2059	0.88606	AMH	76353	25kb
ENSG00000167476	19	2227251	2294758	225	44	120000	-1.1341	0.87162	JSRP1	76353	25kb
ENSG00000104904	19	2244485	2298487	190	36	120000	-1.0331	0.84922	OAZ1	76353	25kb
ENSG00000188305	19	2249631	2307181	204	39	120000	-1.2165	0.8881	C19orf35	76353	25kb
ENSG00000220008	19	2264774	2333156	256	42	120000	-0.49313	0.68904	LINGO3	76353	25kb
ENSG00000130332	19	2296516	2353619	215	34	120000	0.01573	0.49372	LSM7	76353	25kb
ENSG00000178297	19	2364769	2451237	418	32	120000	1.0697	0.14238	TMPRSS9	76353	25kb
ENSG00000099800	19	2400622	2452892	240	21	120000	1.1978	0.1155	TIMM13	76353	25kb
ENSG00000176619	19	2402636	2481994	330	29	120000	0.75104	0.22632	LMNB2	76353	25kb
ENSG00000099860	19	2451120	2503257	221	48	120000	0.66555	0.25285	GADD45B	76353	25kb
ENSG00000176533	19	2486217	2727707	1022	124	120000	1.7442	0.040562	GNG7	76353	25kb
ENSG00000176490	19	2689565	2746416	185	27	120000	1.542	0.061535	DIRAS1	76353	25kb
ENSG00000267001	19	2692767	2765046	240	30	120000	1.7232	0.042425	AC006538.4	76353	25kb
ENSG00000141873	19	2707202	2765150	204	25	120000	1.7644	0.038829	SLC39A3	76353	25kb
ENSG00000104969	19	2729712	2808369	206	18	120000	2.2494	0.012242	SGTA	76353	25kb
ENSG00000172009	19	2760458	2838599	215	28	120000	3.0372	0.001194	THOP1	76353	25kb
ENSG00000172006	19	2794872	2860771	210	30	120000	3.0869	0.0010114	ZNF554	76353	25kb
ENSG00000186300	19	2816433	2885482	274	27	120000	2.5474	0.0054267	ZNF555	76353	25kb
ENSG00000172000	19	2842333	2903515	248	37	120000	1.3697	0.085385	ZNF556	76353	25kb
ENSG00000171970	19	2875896	2943474	305	26	120000	-0.36719	0.64326	ZNF57	76353	25kb
ENSG00000175691	19	2908216	2969969	247	46	120000	0.00098209	0.49961	ZNF77	76353	25kb
ENSG00000104953	19	2952444	3020177	260	41	120000	-0.82967	0.79664	TLE6	76353	25kb
ENSG00000065717	19	2972636	3072633	392	77	120000	1.5133	0.065104	TLE2	76353	25kb
ENSG00000104964	19	3027908	3088105	208	48	120000	1.0959	0.13657	AES	76353	25kb
ENSG00000088256	19	3069408	3149002	383	57	120000	1.3638	0.086318	GNA11	76353	25kb
ENSG00000060558	19	3111191	3188767	417	65	120000	0.43496	0.33179	GNA15	76353	25kb
ENSG00000125910	19	3147344	3205329	266	37	120000	-0.27228	0.6073	S1PR4	76353	25kb
ENSG00000125912	19	3160561	3234573	293	29	120000	-0.31193	0.62245	NCLN	76353	25kb

ENSG00000161082	19	3199701	3322074	425	52	120000	-0.39381	0.65314	CELF5	76353	25kb
ENSG00000141905	19	3334561	3494215	533	75	120000	0.32153	0.3739	NFIC	76353	25kb
ENSG00000095932	19	3449405	3505540	141	28	120000	1.2584	0.10412	C19orf77	76353	25kb
ENSG00000129932	19	3465819	3525938	121	21	120000	1.2057	0.11397	DOHH	76353	25kb
ENSG00000105325	19	3481271	3563328	200	31	120000	0.97103	0.16577	FZR1	76353	25kb
ENSG00000161091	19	3513259	3599288	246	53	120000	0.43414	0.33209	MFSD12	76353	25kb
ENSG00000183397	19	3514152	3569028	152	29	120000	0.47796	0.31634	C19orf71	76353	25kb
ENSG00000064961	19	3547775	3604086	181	45	120000	0.16874	0.433	HMG20B	76353	25kb
ENSG00000179855	19	3560551	3618539	202	34	120000	0.045095	0.48202	GIPC3	76353	25kb
ENSG00000006638	19	3569504	3631838	221	30	120000	-0.028903	0.51153	TBXA2R	76353	25kb
ENSG00000105298	19	3585639	3651813	246	29	120000	0.11257	0.45519	CACTIN	76353	25kb
ENSG00000186111	19	3605181	3725477	354	37	120000	-0.15981	0.56349	PIP5K1C	76353	25kb
ENSG00000105289	19	3683107	3775811	319	53	120000	-0.029146	0.51163	TJP3	76353	25kb
ENSG00000011132	19	3725817	3786697	240	44	120000	0.27318	0.39236	APBA3	76353	25kb
ENSG00000183617	19	3737662	3793573	196	41	120000	0.12207	0.45142	MRPL54	76353	25kb
ENSG00000173976	19	3744087	3797233	172	38	120000	-0.22918	0.59064	RAX2	76353	25kb
ENSG00000007264	19	3752971	3827127	225	54	120000	-0.89125	0.8136	MATK	76353	25kb
ENSG00000105278	19	3779022	3894030	335	37	120000	-1.8519	0.96798	ZFR2	76353	25kb
ENSG00000167654	19	3854862	3953077	263	57	120000	-1.5705	0.94185	ATCAY	76353	25kb
ENSG00000077009	19	3908101	3967414	146	36	120000	-0.81365	0.79208	NMRK2	76353	25kb
ENSG00000167657	19	3933451	3996121	140	36	120000	-0.83287	0.79754	DAPK3	76353	25kb
ENSG00000167658	19	3951054	4010467	122	26	120000	-0.6851	0.75336	EEF2	76353	25kb
ENSG00000105229	19	3982644	4064384	208	25	120000	-0.39761	0.65454	PIAS4	76353	25kb
ENSG00000205147	19	4016127	4068154	146	18	120000	-0.30958	0.62156	AC016586.1	76353	25kb
ENSG00000178951	19	4019362	4091943	200	26	120000	-0.22189	0.5878	ZBTB7A	76353	25kb
ENSG00000126934	19	4065319	4149126	240	40	120000	-0.17962	0.57127	MAP2K2	76353	25kb
ENSG00000060566	19	4128598	4198051	236	39	120000	-0.25679	0.60133	CREB3L3	76353	25kb
ENSG00000077463	19	4149106	4207601	175	33	120000	0.52507	0.29977	SIRT6	76353	25kb
ENSG00000089847	19	4158351	4249811	302	39	120000	0.73314	0.23174	ANKRD24	76353	25kb
ENSG00000105246	19	4204495	4262528	212	23	120000	0.10118	0.4597	EBI3	76353	25kb
ENSG00000105248	19	4222076	4294087	229	23	120000	-0.4072	0.65807	CCDC94	76353	25kb
ENSG00000105251	19	4253598	4315721	191	32	120000	-0.018987	0.50757	SHD	76353	25kb
ENSG00000167664	19	4267229	4327428	191	39	120000	0.75487	0.22516	TMIGD2	76353	25kb
ENSG00000105255	19	4279597	4348840	230	40	120000	1.0188	0.15415	FSD1	76353	25kb
ENSG00000178078	19	4299040	4367783	221	43	120000	0.60778	0.27167	STAP2	76353	25kb
ENSG00000008382	19	4318524	4385083	202	41	120000	0.042164	0.48318	MPND	76353	25kb
ENSG00000141985	19	4335367	4425544	258	27	120000	-0.85971	0.80503	SH3GL1	76353	25kb
ENSG00000167670	19	4377659	4470015	271	21	120000	-0.83412	0.79789	CHAF1A	76353	25kb
ENSG00000167671	19	4419996	4482819	208	32	120000	-1.148	0.87452	UBXN6	76353	25kb
ENSG00000167674	19	4447284	4527223	283	29	120000	-1.5039	0.93369	HDGFRP2	76353	25kb
ENSG00000167676	19	4477204	4542716	218	35	120000	-1.7249	0.95773	PLIN4	76353	25kb
ENSG00000214456	19	4497543	4560236	194	46	120000	-0.52659	0.70076	PLIN5	76353	25kb
ENSG00000267385	19	4503439	4565079	189	43	120000	0.2474	0.4023	CTB-50L17.14	76353	25kb
ENSG00000171236	19	4511421	4565486	159	35	120000	0.45142	0.32584	LRG1	76353	25kb
ENSG00000167680	19	4517600	4584820	213	48	120000	0.81735	0.20686	SEMA6B	76353	25kb
ENSG00000185361	19	4614530	4680580	208	30	120000	2.1708	0.014973	TNFAIP8L1	76353	25kb
ENSG00000074842	19	4616386	4695382	223	32	120000	2.2017	0.013845	C19orf10	76353	25kb
ENSG00000142002	19	4650236	4749685	291	36	120000	1.6426	0.050229	DPP9	76353	25kb
ENSG00000141965	19	4766728	4820571	212	31	120000	0.70559	0.24022	FEM1A	76353	25kb
ENSG00000127666	19	4790944	4856716	251	40	120000	1.2896	0.09859	TICAM1	76353	25kb
ENSG00000105355	19	4813353	4892780	299	48	120000	1.1509	0.12488	PLIN3	76353	25kb
ENSG00000205784	19	4865449	4927879	203	49	120000	0.23567	0.40685	ARRDC5	76353	25kb
ENSG00000127663	19	4944125	5178606	704	43	120000	-0.4288	0.66596	KDM4B	76353	25kb
ENSG00000105426	19	5133506	5365814	812	105	120000	0.087159	0.46527	PTPRS	76353	25kb
ENSG00000105428	19	5430426	5481867	173	41	120000	-1.4784	0.93035	ZNRF4	76353	25kb
ENSG00000130254	19	5562010	5649057	212	24	120000	-0.38268	0.64902	SAFB2	76353	25kb
ENSG00000160633	19	5598046	5693489	192	23	120000	-0.41139	0.65961	SAFB	76353	25kb
ENSG00000130255	19	5649958	5716887	156	19	120000	-1.2159	0.888	RPL36	76353	25kb
ENSG00000174917	19	5653432	5705907	128	18	120000	-1.2969	0.90266	C19orf70	76353	25kb
ENSG00000167733	19	5655615	5713533	143	18	120000	-1.2546	0.89518	HSD11B1L	76353	25kb
ENSG00000196365	19	5666845	5745583	184	26	120000	-1.5578	0.94036	LONP1	76353	25kb
ENSG00000174898	19	5695688	5803745	304	49	120000	-1.153	0.87555	CATSPERD	76353	25kb
ENSG00000212123	19	5757971	5809776	160	22	120000	-0.23811	0.5941	PRR22	76353	25kb
ENSG00000267157	19	5759016	5810630	163	22	120000	-0.25621	0.60111	CTB-54O9.9	76353	25kb
ENSG00000141994	19	5759843	5816249	176	26	120000	-0.035331	0.51409	DUS3L	76353	25kb
ENSG00000171119	19	5798813	5853335	192	36	120000	1.4844	0.068847	NRTN	76353	25kb
ENSG00000156413	19	5805621	5864742	213	44	120000	1.8323	0.033457	FUT6	76353	25kb
ENSG00000171124	19	5817899	5876485	227	50	120000	1.7438	0.040597	FUT3	76353	25kb
ENSG00000130383	19	5840837	5928798	321	40	120000	0.99277	0.16041	FUT5	76353	25kb
ENSG00000267740	19	5841182	5928798	320	40	120000	0.97568	0.16461	AC024592.12	76353	25kb
ENSG00000174886	19	5866287	5929017	228	26	120000	0.95826	0.16897	NDUFA11	76353	25kb
ENSG00000267314	19	5879852	5939718	179	23	120000	1.1029	0.13503	AC104532.2	76353	25kb
ENSG00000187650	19	5879869	5935864	172	22	120000	1.1407	0.127	VMAC	76353	25kb
ENSG00000105519	19	5886718	5940888	154	23	120000	1.1012	0.13541	CAPS	76353	25kb
ENSG00000031823	19	5891150	6003153	243	33	120000	1.0272	0.15215	RANBP3	76353	25kb
ENSG00000087903	19	5968175	6224583	701	51	120000	-0.23486	0.59284	RFX2	76353	25kb
ENSG00000130377	19	6110258	6218112	422	28	120000	-0.20586	0.58155	ACSBG2	76353	25kb
ENSG00000130382	19	6187966	6304959	519	37	120000	1.7017	0.044407	MLLT1	76353	25kb
ENSG00000167769	19	6281153	6358640	458	31	120000	0.46365	0.32145	ACER1	76353	25kb
ENSG00000125656	19	6336463	6393919	280	15	120000	-1.3701	0.91468	CLPP	76353	25kb
ENSG00000125652	19	6347444	6400042	221	20	120000	-1.2231	0.88935	ALKBH7	76353	25kb
ENSG00000125650	19	6350159	6404069	218	23	120000	-1.3157	0.90586	PSPN	76353	25kb
ENSG00000125651	19	6354580	6418992	244	30	120000	-1.1599	0.87696	GTF2F1	76353	25kb
ENSG00000088247	19	6388359	6449805	221	23	120000	0.047241	0.48116	KHSRP	76353	25kb
ENSG00000181240	19	6401048	6458790	216	22	120000	0.34042	0.36677	SLC25A41	76353	25kb
ENSG00000125648	19	6411090	6490214	266	30	120000	0.11396	0.45463	SLC25A23	76353	25kb
ENSG00000130545	19	6438788	6492232	163	30	120000	0.091354	0.46361	CRB3	76353	25kb
ENSG00000205744	19	6442218	6507568	180	39	120000	0.51119	0.30461	DENND1C	76353	25kb
ENSG00000104833	19	6469330	6527859	157	44	120000	0.69272	0.24424	TUBB4A	76353	25kb
ENSG00000125657	19	6506010	6560931	164	38	120000	-0.70754	0.76038	TNFSF9	76353	25kb
ENSG00000125726	19	6558194	6629114	304	38	120000	-0.69258	0.75571	CD70	76353	25kb
ENSG00000125735	19	6638148	6695599	296	39	120000	-0.50769	0.69416	TNFSF14	76353	25kb
ENSG00000125730	19	6652715	6755573	373	56	120000	1.5289	0.06314	C3	76353	25kb
ENSG00000125734	19	6704925	6762614	153	38	120000	1.4213	0.07761	GPR108	76353	25kb
ENSG00000125733	19	6712936	6776537	172	33	120000	0.91271	0.1807	TRIP10	76353	25kb
ENSG00000125731	19	6727171	6792599	224	31	120000	-0.11197	0.54457	SH2D3A	76353	25kb

ENSG00000141968	19	6747725	6882377	517	63	120000	-1.7929	0.9635	VAV1	76353	25kb
ENSG00000174837	19	6862577	6965470	509	31	120000	-0.86806	0.80732	EMR1	76353	25kb
ENSG00000204752	19	6986514	7038338	171	29	120000	0.97586	0.16457	AC025278.1	76353	25kb
ENSG00000237247	19	7005589	7058022	67	10	120000	0.39327	0.34706	MBD3L5	76353	25kb
ENSG00000205718	19	7012759	7065190	42	9	120000	0.61414	0.26956	MBD3L4	76353	25kb
ENSG00000230522	19	7024332	7076746	41	10	120000	-0.48486	0.68611	MBD3L2	76353	25kb
ENSG00000182315	19	7031220	7083651	62	14	120000	-0.48117	0.6848	MBD3L3	76353	25kb
ENSG00000130544	19	7044455	7112979	169	33	120000	-0.43104	0.66678	ZNF557	76353	25kb
ENSG00000171105	19	7087266	7319045	857	103	120000	-0.13129	0.55223	INSR	76353	25kb
ENSG00000263264	19	7388848	7473271	228	21	120000	0.0267	0.48935	CTB-133G6.1	76353	25kb
ENSG00000268861	19	7420850	7560131	594	30	120000	-0.22275	0.58813	CTD-2207O23.3	76353	25kb
ENSG00000104880	19	7434999	7562363	553	31	120000	-0.11051	0.544	ARHGEF18	76353	25kb
ENSG00000104883	19	7516761	7587335	243	40	120000	-0.18094	0.57179	PEX11G	76353	25kb
ENSG00000198723	19	7532862	7598336	215	38	120000	-0.059049	0.52354	C19orf45	76353	25kb
ENSG00000267952	19	7546938	7609508	183	35	120000	-0.17052	0.5677	CTD-2207O23.12	76353	25kb
ENSG00000198816	19	7555178	7610912	158	32	120000	-0.49891	0.69108	ZNF358	76353	25kb
ENSG00000090674	19	7562512	7623895	199	34	120000	-0.73454	0.76869	MCOLN1	76353	25kb
ENSG00000268614	19	7573481	7629945	195	30	120000	-0.38159	0.64862	CTD-2207O23.10	76353	25kb
ENSG00000032444	19	7573890	7651650	305	34	120000	0.19543	0.42253	PNPLA6	76353	25kb
ENSG00000076826	19	7635788	7708190	204	22	120000	0.59709	0.27523	CAMSAP3	76353	25kb
ENSG00000076924	19	7659411	7719451	151	16	120000	0.16778	0.43338	XAB2	76353	25kb
ENSG00000229833	19	7669623	7721842	128	13	120000	0.15419	0.43873	PET100	76353	25kb
ENSG00000268400	19	7669682	7732934	160	17	120000	0.30955	0.37845	CTD-3214H19.4	76353	25kb
ENSG00000174788	19	7671497	7723634	136	14	120000	0.11158	0.45558	PCP2	76353	25kb
ENSG00000076944	19	7676767	7737759	167	19	120000	0.15364	0.43895	STXBP2	76353	25kb
ENSG00000104918	19	7708930	7760334	231	35	120000	1.6652	0.047936	RETN	76353	25kb
ENSG00000183019	19	7716514	7769712	257	32	120000	1.7458	0.040422	C19orf59	76353	25kb
ENSG00000269711	19	7718387	7772740	267	34	120000	1.8274	0.03382	CTD-3214H19.16	76353	25kb
ENSG00000181029	19	7720729	7772744	265	33	120000	1.8637	0.031179	TRAPPC5	76353	25kb
ENSG00000104921	19	7728644	7792032	313	39	120000	1.1921	0.11662	FCER2	76353	25kb
ENSG00000182566	19	7768843	7823792	233	43	120000	-0.57011	0.7157	CLEC4G	76353	25kb
ENSG00000090659	19	7779879	7837464	257	44	120000	-0.94919	0.82874	CD209	76353	25kb
ENSG00000104938	19	7803035	7859491	298	41	120000	-1.7569	0.96053	CLEC4M	76353	25kb
ENSG00000142459	19	7870119	7954862	227	30	120000	0.67073	0.2512	EVI5L	76353	25kb
ENSG00000183248	19	7908605	7964326	154	24	120000	0.67463	0.24996	CTD-3193O13.9	76353	25kb
ENSG00000171017	19	7928390	7991901	161	30	120000	-0.15958	0.56339	LRRC8E	76353	25kb
ENSG00000214248	19	7938689	7993427	140	29	120000	-0.33654	0.63177	AC010336.1	76353	25kb
ENSG00000076984	19	7943728	8004363	182	35	120000	-0.92889	0.82353	MAP2K7	76353	25kb
ENSG00000260001	19	7956030	8008982	158	28	120000	-1.0175	0.84553	TGFBR3L	76353	25kb
ENSG00000104976	19	7960201	8013135	159	26	120000	-1.267	0.89743	SNAPC2	76353	25kb
ENSG00000178531	19	7964372	8016051	153	24	120000	-1.3582	0.91281	CTXN1	76353	25kb
ENSG00000104980	19	7966603	8033805	186	25	120000	-1.1526	0.87546	TIMM44	76353	25kb
ENSG00000066044	19	7998463	8095543	258	17	120000	-0.79814	0.78761	ELAVL1	76353	25kb
ENSG00000131142	19	8092651	8152534	260	33	120000	-0.97724	0.83578	CCL25	76353	25kb
ENSG00000142449	19	8105286	8239730	633	80	120000	-0.80088	0.7884	FBN3	76353	25kb
ENSG00000090661	19	8246620	8352305	428	66	120000	2.5349	0.0056245	CERS4	76353	25kb
ENSG00000167775	19	8342011	8398240	150	18	120000	-0.56382	0.71356	CD320	76353	25kb
ENSG00000167774	19	8348167	8411263	213	23	120000	-0.70188	0.75862	NDUFA7	76353	25kb
ENSG00000267855	19	8348490	8411280	213	23	120000	-0.70188	0.75862	NDUFA7	76353	25kb
ENSG00000233927	19	8361042	8413224	218	23	120000	-0.70438	0.7594	RPS28	76353	25kb
ENSG00000186994	19	8362468	8433146	267	25	120000	-0.59288	0.72337	KANK3	76353	25kb
ENSG00000167772	19	8403173	8464257	197	31	120000	-0.26727	0.60537	ANGPTL4	76353	25kb
ENSG00000185236	19	8429865	8494318	209	29	120000	-0.090326	0.53599	RAB11B	76353	25kb
ENSG00000099785	19	8453154	8528901	249	38	120000	-0.41504	0.66094	MARCH2	76353	25kb
ENSG00000099783	19	8484651	8578998	296	25	120000	-1.3954	0.91855	HNRNPM	76353	25kb
ENSG00000133246	19	8529940	8592996	203	22	120000	-1.418	0.9219	PRAM1	76353	25kb
ENSG00000133250	19	8550462	8604048	157	14	120000	-1.0664	0.85688	ZNF414	76353	25kb
ENSG00000142347	19	8560674	8667461	297	43	120000	-0.076297	0.53041	MYO1F	76353	25kb
ENSG00000142303	19	8620126	8700620	170	45	120000	-0.31977	0.62543	ADAMTS10	76353	25kb
ENSG00000181786	19	8782751	8834172	148	22	120000	-1.1766	0.88033	ACTL9	76353	25kb
ENSG00000181733	19	8816316	8867371	65	15	120000	-1.4809	0.93068	OR2Z1	76353	25kb
ENSG00000167785	19	8895380	8968004	196	22	120000	-0.29172	0.61475	ZNF558	76353	25kb
ENSG00000170948	19	8918074	8979016	167	18	120000	-0.12712	0.55058	MBD3L1	76353	25kb
ENSG00000181143	19	8934520	9117018	661	53	120000	-0.38702	0.65063	MUC16	76353	25kb
ENSG00000170929	19	9178855	9229889	224	22	120000	-0.90039	0.81604	OR1M1	76353	25kb
ENSG00000170923	19	9187945	9238982	240	23	120000	-0.71519	0.76275	OR7G2	76353	25kb
ENSG00000161807	19	9200274	9251439	237	26	120000	-0.46675	0.67966	OR7G1	76353	25kb
ENSG00000170920	19	9211688	9262626	247	29	120000	-0.39919	0.65512	OR7G3	76353	25kb
ENSG00000130803	19	9226056	9299100	307	30	120000	-0.021241	0.50847	ZNF317	76353	25kb
ENSG00000188000	19	9271279	9324493	189	25	120000	-0.019057	0.5076	OR7D2	76353	25kb
ENSG00000174667	19	9299526	9350542	152	17	120000	-0.51997	0.69846	OR7D4	76353	25kb
ENSG00000237521	19	9336606	9387820	114	15	120000	1.0863	0.13867	OR7E24	76353	25kb
ENSG00000196110	19	9379951	9445514	127	11	120000	0.90092	0.18382	ZNF699	76353	25kb
ENSG00000188321	19	9409448	9486838	164	11	120000	0.57372	0.28308	ZNF559	76353	25kb
ENSG00000270011	19	9409850	9518293	231	11	120000	0.44906	0.3267	ZNF559-ZNF177	76353	25kb
ENSG00000188629	19	9410021	9518293	231	11	120000	0.44906	0.3267	ZNF177	76353	25kb
ENSG00000174652	19	9498272	9571254	221	9	120000	0.1926	0.42364	ZNF266	76353	25kb
ENSG00000198028	19	9552183	9634283	247	11	120000	-0.53954	0.70524	ZNF560	76353	25kb
ENSG00000130818	19	9613667	9674303	256	5	120000	-1.0725	0.85826	ZNF426	76353	25kb
ENSG00000197961	19	9646029	9720209	289	9	120000	-1.0231	0.84687	ZNF121	76353	25kb
ENSG00000171469	19	9690356	9757075	138	17	120000	-1.3084	0.90464	ZNF561	76353	25kb
ENSG00000267106	19	9706967	9781572	159	14	120000	-1.5714	0.94196	C19orf82	76353	25kb
ENSG00000171466	19	9734330	9810776	183	14	120000	-1.8119	0.965	ZNF562	76353	25kb
ENSG00000224689	19	9775600	9836452	232	10	120000	-1.8706	0.9693	ZNF812	76353	25kb
ENSG00000196605	19	9837669	9928856	170	12	120000	-1.7802	0.96248	ZNF846	76353	25kb
ENSG00000127452	19	9895943	9963492	111	18	120000	-0.32602	0.62779	FBXL12	76353	25kb
ENSG00000198258	19	9913568	9965791	92	18	120000	0.079832	0.46819	UBL5	76353	25kb
ENSG00000127445	19	9920933	9985358	121	20	120000	0.28514	0.38777	PIN1	76353	25kb
ENSG00000105088	19	9939394	10072228	372	43	120000	0.015962	0.49363	OLFM2	76353	25kb
ENSG00000080573	19	10045237	10146147	471	63	120000	0.34058	0.36671	COL5A3	76353	25kb
ENSG00000080511	19	10098925	10157955	286	50	120000	1.0147	0.15512	RDH8	76353	25kb
ENSG00000130813	19	10171798	10228928	145	34	120000	-0.34697	0.63569	C19orf66	76353	25kb
ENSG00000130812	19	10178014	10238472	146	37	120000	-0.18852	0.57476	ANGPTL6	76353	25kb
ENSG00000243207	19	10191899	10250456	118	31	120000	0.17196	0.43174	PPAN-P2RY11	76353	25kb
ENSG00000130810	19	10191965	10250414	118	31	120000	0.17196	0.43174	PPAN	76353	25kb
ENSG00000244165	19	10197214	10251048	111	28	120000	0.21687	0.41416	P2RY11	76353	25kb

ENSG00000130811	19	10200693	10255596	119	28	120000	0.29154	0.38532	EIF3G	76353	25kb
ENSG00000130816	19	10219021	10366962	315	32	120000	0.034813	0.48611	DNMT1	76353	25kb
ENSG00000267534	19	10309520	10366948	132	26	120000	0.44551	0.32797	S1PR2	76353	25kb
ENSG00000105364	19	10337577	10395721	120	28	120000	-0.33049	0.62948	MRPL4	76353	25kb
ENSG00000090339	19	10356511	10422291	159	37	120000	0.13547	0.44612	ICAM1	76353	25kb
ENSG00000105371	19	10372643	10424198	130	29	120000	0.26662	0.39488	ICAM4	76353	25kb
ENSG00000105376	19	10375657	10432454	152	32	120000	0.036305	0.48552	ICAM5	76353	25kb
ENSG00000220201	19	10390479	10445556	169	30	120000	0.11005	0.45619	ZGLP1	76353	25kb
ENSG00000167807	19	10391103	10451685	192	32	120000	0.048349	0.48072	CTD-2369P2.10	76353	25kb
ENSG00000267673	19	10391103	10451691	192	32	120000	0.048349	0.48072	FDX1L	76353	25kb
ENSG00000267303	19	10401147	10456354	175	30	120000	0.032635	0.48698	CTD-2369P2.12	76353	25kb
ENSG00000161847	19	10401888	10469316	203	35	120000	0.50443	0.30698	RAVER1	76353	25kb
ENSG00000076662	19	10419452	10475499	170	30	120000	0.65679	0.25566	ICAM3	76353	25kb
ENSG00000105397	19	10436209	10516352	213	34	120000	1.2879	0.098887	TYK2	76353	25kb
ENSG00000105401	19	10476810	10555797	173	23	120000	0.93441	0.17505	CDC37	76353	25kb
ENSG00000065989	19	10502449	10605305	215	24	120000	0.65882	0.255	PDE4A	76353	25kb
ENSG00000079999	19	10571796	10639417	178	26	120000	1.218	0.11162	KEAP1	76353	25kb
ENSG00000180739	19	10598623	10653607	166	26	120000	1.2127	0.11262	S1PR5	76353	25kb
ENSG00000130734	19	10629571	10689094	207	24	120000	0.36012	0.35938	ATG4D	76353	25kb
ENSG00000129347	19	10638761	10701713	228	23	120000	0.77466	0.21927	KRI1	76353	25kb
ENSG00000129355	19	10652138	10704735	196	21	120000	1.0326	0.15088	CDKN2D	76353	25kb
ENSG00000129354	19	10658347	10722991	229	26	120000	1.2132	0.11254	AP1M2	76353	25kb
ENSG00000129353	19	10688133	10780235	247	28	120000	0.3195	0.37468	SLC44A2	76353	25kb
ENSG00000226104	19	10726715	10777093	108	11	120000	-1.135	0.87182	AC011475.1	76353	25kb
ENSG00000129351	19	10739937	10828093	151	16	120000	-1.2716	0.89825	ILF3	76353	25kb
ENSG00000213339	19	10787106	10849113	157	16	120000	-1.2405	0.8926	QTRT1	76353	25kb
ENSG00000079805	19	10803755	10969164	372	32	120000	-1.1685	0.8787	DNM2	76353	25kb
ENSG00000099203	19	10918114	10971994	82	17	120000	-1.2316	0.89094	TMED1	76353	25kb
ENSG00000214212	19	10922251	11005466	125	19	120000	-1.1121	0.86696	C19orf38	76353	25kb
ENSG00000142453	19	10957189	11058453	153	16	120000	-0.59357	0.7236	CARM1	76353	25kb
ENSG00000130733	19	11008444	11064357	98	15	120000	-0.72748	0.76653	YIPF2	76353	25kb
ENSG00000142444	19	11014409	11069211	109	15	120000	-0.79419	0.78646	C19orf52	76353	25kb
ENSG00000127616	19	11046598	11201071	425	30	120000	-1.9454	0.97414	SMARCA4	76353	25kb
ENSG00000130164	19	11175038	11269492	335	34	120000	-0.15779	0.56269	LDLR	76353	25kb
ENSG00000161888	19	11217196	11291484	289	32	120000	0.43506	0.33176	SPC24	76353	25kb
ENSG00000197256	19	11249943	11333467	273	34	120000	-1.6902	0.95451	KANK2	76353	25kb
ENSG00000130158	19	11284971	11398157	325	29	120000	-0.46108	0.67763	DOCK6	76353	25kb
ENSG00000130173	19	11323178	11377619	122	19	120000	-0.55904	0.71193	C19orf80	76353	25kb
ENSG00000130167	19	11381824	11462672	239	27	120000	-0.42338	0.66399	TSPAN16	76353	25kb
ENSG00000105514	19	11407722	11481946	162	23	120000	-0.3501	0.63687	RAB3D	76353	25kb
ENSG00000105518	19	11428452	11482194	124	16	120000	-0.24099	0.59522	TMEM205	76353	25kb
ENSG00000183401	19	11430360	11490620	124	16	120000	-0.083113	0.53312	CCDC159	76353	25kb
ENSG00000105520	19	11441062	11501374	108	17	120000	0.8366	0.20141	DKFZP761J1410	76353	25kb
ENSG00000173928	19	11460361	11512627	109	13	120000	1.4067	0.079752	SWSAP1	76353	25kb
ENSG00000187266	19	11463236	11520018	111	12	120000	1.4064	0.079795	EPOR	76353	25kb
ENSG00000205517	19	11470017	11555018	157	22	120000	1.724	0.04235	RGL3	76353	25kb
ENSG00000198003	19	11506272	11571603	144	27	120000	1.4369	0.075368	CCDC151	76353	25kb
ENSG00000130175	19	11521109	11586783	148	28	120000	0.97187	0.16556	PRKCSH	76353	25kb
ENSG00000196361	19	11537141	11616861	208	28	120000	0.34112	0.36651	ELAVL3	76353	25kb
ENSG00000267477	19	11552055	11641513	237	26	120000	0.2091	0.41718	CTC-398G3.6	76353	25kb
ENSG00000161914	19	11569242	11641738	196	20	120000	0.035587	0.48581	ZNF653	76353	25kb
ENSG00000130159	19	11591731	11664989	193	17	120000	-0.13404	0.55332	ECSIT	76353	25kb
ENSG00000130176	19	11624532	11686138	141	22	120000	-0.33911	0.63274	CNN1	76353	25kb
ENSG00000130165	19	11636962	11695051	144	27	120000	-0.91041	0.8187	ELOF1	76353	25kb
ENSG00000198551	19	11645189	11754950	294	33	120000	-1.4929	0.93227	ZNF627	76353	25kb
ENSG00000102575	19	11660475	11714823	147	23	120000	-1.5202	0.93577	ACP5	76353	25kb
ENSG00000197933	19	11807080	11874824	199	18	120000	0.18378	0.42709	ZNF823	76353	25kb
ENSG00000197044	19	11852815	11919893	244	23	120000	-0.90901	0.81833	ZNF441	76353	25kb
ENSG00000177599	19	11883482	11945437	170	12	120000	-1.0191	0.84591	ZNF491	76353	25kb
ENSG00000171295	19	11900099	11971016	196	14	120000	-1.0969	0.86365	ZNF440	76353	25kb
ENSG00000171291	19	11934541	12019565	263	18	120000	-0.92776	0.82323	ZNF439	76353	25kb
ENSG00000198429	19	11973599	12050144	197	24	120000	-0.66503	0.74698	ZNF69	76353	25kb
ENSG00000196757	19	12010883	12086588	203	15	120000	-0.27281	0.6075	ZNF700	76353	25kb
ENSG00000267179	19	12010890	12115105	294	16	120000	-0.27177	0.6071	ZNF763	76353	25kb
ENSG00000197054	19	12010890	12116196	296	16	120000	-0.23739	0.59382	ZNF763	76353	25kb
ENSG00000197647	19	12100547	12171556	128	11	120000	0.0021891	0.49913	ZNF433	76353	25kb
ENSG00000257355	19	12103703	12188782	161	15	120000	0.65843	0.25513	CTD-2006C1.10	76353	25kb
ENSG00000257446	19	12129620	12192127	136	14	120000	0.98671	0.16189	ZNF878	76353	25kb
ENSG00000223547	19	12150514	12217380	143	15	120000	1.3112	0.094894	ZNF844	76353	25kb
ENSG00000188474	19	12178078	12250491	147	18	120000	1.2532	0.10506	ZNF788	76353	25kb
ENSG00000214189	19	12178078	12273050	179	21	120000	0.92021	0.17873	ZNF788	76353	25kb
ENSG00000132010	19	12178658	12276222	183	22	120000	0.90432	0.18291	ZNF20	76353	25kb
ENSG00000213297	19	12217932	12292546	129	17	120000	-0.41558	0.66114	ZNF625-ZNF20	76353	25kb
ENSG00000257591	19	12226032	12292546	113	15	120000	-0.70073	0.75826	ZNF625	76353	25kb
ENSG00000196646	19	12248879	12325064	124	13	120000	-1.2754	0.89891	ZNF136	76353	25kb
ENSG00000197857	19	12310501	12430702	273	14	120000	-0.55461	0.71042	ZNF44	76353	25kb
ENSG00000188868	19	12403291	12469534	128	9	120000	-0.18579	0.5737	ZNF563	76353	25kb
ENSG00000198342	19	12435185	12501719	101	20	120000	-0.12075	0.54805	ZNF442	76353	25kb
ENSG00000268744	19	12465003	12537088	212	17	120000	-1.1144	0.86744	CTD-3105H18.14	76353	25kb
ENSG00000196466	19	12475830	12537085	201	16	120000	-1.2083	0.88654	ZNF799	76353	25kb
ENSG00000268870	19	12477763	12576896	381	15	120000	-1.3978	0.91892	CTD-3105H18.16	76353	25kb
ENSG00000180855	19	12515521	12576926	321	10	120000	-1.3789	0.91603	ZNF443	76353	25kb
ENSG00000269755	19	12518917	12620630	459	14	120000	-1.5324	0.93729	CTD-3105H18.18	76353	25kb
ENSG00000242852	19	12546998	12649668	332	16	120000	-1.2402	0.89254	ZNF709	76353	25kb
ENSG00000196826	19	12547373	12687288	409	28	120000	-0.57943	0.71885	ZNF709	76353	25kb
ENSG00000249709	19	12611184	12687356	196	28	120000	0.019993	0.49202	ZNF564	76353	25kb
ENSG00000269693	19	12611534	12716789	247	33	120000	1.0595	0.14468	CTD-2192J16.20	76353	25kb
ENSG00000188033	19	12663775	12775912	209	18	120000	2.2223	0.013131	ZNF490	76353	25kb
ENSG00000173875	19	12696732	12767735	143	12	120000	2.2482	0.01228	ZNF791	76353	25kb
ENSG00000269242	19	12729645	12784211	92	15	120000	1.7456	0.040436	CTD-2192J16.22	76353	25kb
ENSG00000104774	19	12732325	12802556	119	18	120000	1.0498	0.1469	MAN2B1	76353	25kb
ENSG00000269590	19	12750642	12805211	87	15	120000	0.62734	0.26522	CTD-2192J16.24	76353	25kb
ENSG00000123154	19	12752614	12811646	98	13	120000	0.26949	0.39378	WDR83	76353	25kb
ENSG00000105583	19	12753885	12807170	88	13	120000	0.26649	0.39493	WDR83OS	76353	25kb
ENSG00000095059	19	12761531	12817716	94	10	120000	-0.054422	0.5217	DHPS	76353	25kb
ENSG00000132004	19	12773867	12832457	119	7	120000	-0.14341	0.55702	FBXW9	76353	25kb

ENSG00000105576	19	12785008	12859825	122	7	120000	-0.2391	0.59449	TNPO2	76353	25kb
ENSG00000123144	19	12816454	12870589	74	8	120000	-0.051851	0.52068	C19orf43	76353	25kb
ENSG00000198356	19	12822953	12884137	74	15	120000	-0.12309	0.54898	ASNA1	76353	25kb
ENSG00000039987	19	12837516	12894272	61	20	120000	0.15696	0.43764	BEST2	76353	25kb
ENSG00000095066	19	12848817	13008554	307	27	120000	-0.31119	0.62217	HOOK2	76353	25kb
ENSG00000171223	19	12877310	12929124	127	15	120000	0.015556	0.49379	JUNB	76353	25kb
ENSG00000167815	19	12882634	12937694	142	17	120000	0.070862	0.47175	PRDX2	76353	25kb
ENSG00000104889	19	12892394	12949452	147	17	120000	0.067764	0.47299	RNASEH2A	76353	25kb
ENSG00000132026	19	12911292	12971242	157	18	120000	-0.16698	0.56631	RTBDN	76353	25kb
ENSG00000105613	19	12919765	13010765	201	22	120000	-0.52277	0.69943	MAST1	76353	25kb
ENSG00000105612	19	12961025	13017282	101	16	120000	-0.82738	0.79599	DNASE2	76353	25kb
ENSG00000105610	19	12970237	13022995	94	16	120000	-0.81094	0.7913	KLF1	76353	25kb
ENSG00000105607	19	12976840	13050021	122	18	120000	-0.46013	0.67729	GCDH	76353	25kb
ENSG00000161860	19	12984600	13055090	115	16	120000	-0.3699	0.64427	SYCE2	76353	25kb
ENSG00000179115	19	13008293	13069851	93	14	120000	-0.20541	0.58137	FARSA	76353	25kb
ENSG00000179218	19	13024392	13080303	84	16	120000	-0.034298	0.51368	CALR	76353	25kb
ENSG00000179262	19	13031669	13089456	83	19	120000	-0.12846	0.55111	RAD23A	76353	25kb
ENSG00000179271	19	13039972	13093050	69	22	120000	-0.20943	0.58294	GADD45GIP1	76353	25kb
ENSG00000179284	19	13050973	13110567	103	24	120000	0.27212	0.39276	DAND5	76353	25kb
ENSG00000008441	19	13081422	13234610	243	31	120000	0.91889	0.17908	NFIX	76353	25kb
ENSG00000104903	19	13184847	13238975	80	24	120000	0.57493	0.28267	LYL1	76353	25kb
ENSG00000104907	19	13190716	13253381	97	26	120000	0.70992	0.23888	TRMT1	76353	25kb
ENSG00000160877	19	13203917	13276955	129	26	120000	0.28018	0.38967	NACC1	76353	25kb
ENSG00000104915	19	13229872	13286197	107	22	120000	-0.25602	0.60103	STX10	76353	25kb
ENSG00000160888	19	13236229	13290722	100	23	120000	-0.31827	0.62486	IER2	76353	25kb
ENSG00000141837	19	13292256	13759804	1645	181	120000	-0.2452	0.59685	CACNA1A	76353	25kb
ENSG00000104957	19	13817574	13899110	204	27	120000	-0.46569	0.67928	CCDC130	76353	25kb
ENSG00000037757	19	13850346	13910096	163	23	120000	-1.228	0.89027	MRI1	76353	25kb
ENSG00000104979	19	13859982	13914276	149	21	120000	-1.177	0.8804	C19orf53	76353	25kb
ENSG00000214109	19	13865884	13925972	150	20	120000	-0.77614	0.78117	AC008686.1	76353	25kb
ENSG00000132003	19	13881274	13968044	194	23	120000	-0.38362	0.64937	ZSWIM4	76353	25kb
ENSG00000187556	19	13947877	14016571	147	15	120000	-0.04314	0.51721	NANOS3	76353	25kb
ENSG00000132016	19	13968161	14042265	136	11	120000	0.24989	0.40134	C19orf57	76353	25kb
ENSG00000132024	19	13992014	14066692	127	11	120000	0.31254	0.37731	CC2D1A	76353	25kb
ENSG00000132000	19	14017000	14089204	69	20	120000	-0.3584	0.63998	PODNL1	76353	25kb
ENSG00000132017	19	14038304	14097254	52	17	120000	-1.0139	0.84469	DCAF15	76353	25kb
ENSG00000132005	19	14047350	14142851	116	26	120000	-1.4887	0.93172	RFX1	76353	25kb
ENSG00000171136	19	14113960	14166854	128	19	120000	-1.5437	0.93868	RLN3	76353	25kb
ENSG00000104998	19	14117560	14188743	173	27	120000	-1.5558	0.94012	IL27RA	76353	25kb
ENSG00000187867	19	14139177	14194971	127	21	120000	-1.3606	0.91318	PALM3	76353	25kb
ENSG00000141854	19	14158348	14210874	87	19	120000	-0.8401	0.79958	hsa-mir-1199	76353	25kb
ENSG00000188032	19	14167431	14221687	84	17	120000	-0.3455	0.63514	C19orf67	76353	25kb
ENSG00000141858	19	14173652	14226848	72	15	120000	-0.43709	0.66898	SAMD1	76353	25kb
ENSG00000072062	19	14177500	14253896	108	17	120000	-0.53715	0.70442	PRKACA	76353	25kb
ENSG00000105011	19	14205321	14272768	93	11	120000	-0.44105	0.67041	ASF1B	76353	25kb
ENSG00000072071	19	14235750	14341999	236	39	120000	-0.82037	0.794	LPHN1	76353	25kb
ENSG00000123146	19	14466313	14544537	174	50	120000	1.5526	0.06026	CD97	76353	25kb
ENSG00000123136	19	14494631	14555192	127	36	120000	-0.78643	0.78419	DDX39A	76353	25kb
ENSG00000123143	19	14518865	14607679	278	48	120000	-1.1222	0.8691	PKN1	76353	25kb
ENSG00000160951	19	14558278	14611174	216	38	120000	-0.80713	0.7902	PTGER1	76353	25kb
ENSG00000123159	19	14563572	14631944	268	36	120000	-0.83892	0.79924	GIPC1	76353	25kb
ENSG00000132002	19	14600582	14665582	199	27	120000	-0.75421	0.77464	DNAJB1	76353	25kb
ENSG00000099797	19	14602897	14701792	281	36	120000	-0.37661	0.64677	TECR	76353	25kb
ENSG00000099795	19	14651890	14707874	160	26	120000	0.26079	0.39713	NDUFB7	76353	25kb
ENSG00000187912	19	14668896	14746969	267	34	120000	0.33847	0.3675	CLEC17A	76353	25kb
ENSG00000131355	19	14704929	14825839	425	46	120000	1.2727	0.10156	EMR3	76353	25kb
ENSG00000160961	19	14775613	14869558	322	28	120000	1.6871	0.04579	ZNF333	76353	25kb
ENSG00000268550	19	14809747	14860121	184	26	120000	1.5661	0.058657	AC090427.1	76353	25kb
ENSG00000127507	19	14818205	14914353	373	40	120000	1.7987	0.036034	EMR2	76353	25kb
ENSG00000188269	19	14878302	14971188	386	54	120000	1.1238	0.13056	OR7A5	76353	25kb
ENSG00000127530	19	14884958	14936023	202	31	120000	1.4267	0.076838	OR7C1	76353	25kb
ENSG00000127515	19	14926760	14977689	232	38	120000	0.21878	0.41341	OR7A10	76353	25kb
ENSG00000185385	19	14966138	15017264	254	25	120000	-0.059699	0.5238	OR7A17	76353	25kb
ENSG00000127529	19	15027301	15078260	250	27	120000	-0.62797	0.73499	OR7C2	76353	25kb
ENSG00000105143	19	15035846	15158802	690	58	120000	-0.76604	0.77817	SLC1A6	76353	25kb
ENSG00000160994	19	15096556	15159081	389	52	120000	-0.27658	0.60895	CCDC105	76353	25kb
ENSG00000105141	19	15135195	15194104	306	26	120000	0.61162	0.27039	CASP14	76353	25kb
ENSG00000094661	19	15172791	15224032	240	30	120000	0.15828	0.43712	OR1I1	76353	25kb
ENSG00000105137	19	15193214	15250799	212	31	120000	-1.1715	0.87931	SYDE1	76353	25kb
ENSG00000105135	19	15200795	15261596	210	28	120000	-0.64726	0.74127	ILVBL	76353	25kb
ENSG00000074181	19	15245444	15336792	245	25	120000	0.74871	0.22702	NOTCH3	76353	25kb
ENSG00000105131	19	15312730	15369246	65	22	120000	3.1083	0.00094098	EPHX3	76353	25kb
ENSG00000141867	19	15322647	15468356	168	28	120000	3.6872	0.00011336	BRD4	76353	25kb
ENSG00000105127	19	15439196	15515609	106	14	120000	3.3986	0.00033866	AKAP8	76353	25kb
ENSG00000011243	19	15465859	15554952	119	12	120000	3.3419	0.00041602	AKAP8L	76353	25kb
ENSG00000011451	19	15507319	15585762	106	24	120000	3.5256	0.00021123	WIZ	76353	25kb
ENSG00000105122	19	15537435	15600382	108	25	120000	1.7082	0.043796	RASAL3	76353	25kb
ENSG00000161031	19	15554456	15634767	179	33	120000	1.3721	0.085009	PGLYRP2	76353	25kb
ENSG00000171954	19	15594304	15688128	324	53	120000	0.64596	0.25915	CYP4F22	76353	25kb
ENSG00000186529	19	15726707	15798635	445	32	120000	0.37342	0.35442	CYP4F3	76353	25kb
ENSG00000186204	19	15758567	15832984	492	33	120000	0.017507	0.49302	CYP4F12	76353	25kb
ENSG00000171942	19	15813834	15864862	222	17	120000	0.37984	0.35203	OR10H2	76353	25kb
ENSG00000171936	19	15827203	15878153	222	18	120000	0.13413	0.44665	OR10H3	76353	25kb
ENSG00000172519	19	15879761	15930892	185	21	120000	-0.85777	0.80449	OR10H5	76353	25kb
ENSG00000186723	19	15892817	15943936	192	19	120000	-1.0578	0.85492	OR10H1	76353	25kb
ENSG00000186115	19	15963833	16033930	372	24	120000	-0.84777	0.80172	CYP4F2	76353	25kb
ENSG00000171903	19	15998177	16070677	357	20	120000	0.10653	0.45758	CYP4F11	76353	25kb
ENSG00000176231	19	16034818	16085768	258	14	120000	0.73629	0.23078	OR10H4	76353	25kb
ENSG00000167460	19	16152831	16238813	267	34	120000	0.588	0.27827	TPM4	76353	25kb
ENSG00000167461	19	16197439	16270044	184	26	120000	-0.93347	0.82471	RAB8A	76353	25kb
ENSG00000196684	19	16219838	16294386	216	29	120000	-0.82782	0.79611	HSH2D	76353	25kb
ENSG00000141977	19	16247179	16309336	162	22	120000	-0.18422	0.57308	CIB3	76353	25kb
ENSG00000105058	19	16271191	16327857	160	17	120000	0.64899	0.25817	FAM32A	76353	25kb
ENSG00000072958	19	16283389	16371160	294	14	120000	0.85415	0.19651	AP1M1	76353	25kb
ENSG00000127528	19	16410628	16463685	186	30	120000	0.98008	0.16352	KLF2	76353	25kb
ENSG00000127527	19	16441050	16607896	348	31	120000	1.3745	0.084644	EPS15L1	76353	25kb

ENSG00000269058	19	16564868	16632003	187	23	120000	-0.65728	0.7445	CALR3	76353	25kb
ENSG00000141979	19	16564878	16764015	436	35	120000	-0.92207	0.82176	CTD-3222D19.2	76353	25kb
ENSG00000105072	19	16582122	16657163	192	24	120000	-0.89263	0.81397	C19orf44	76353	25kb
ENSG00000085872	19	16603700	16678341	157	24	120000	-0.62563	0.73422	CHERP	76353	25kb
ENSG00000127526	19	16635642	16708193	123	19	120000	-0.26199	0.60333	SLC35E1	76353	25kb
ENSG00000105085	19	16660718	16764873	194	26	120000	-0.7899	0.78521	MED26	76353	25kb
ENSG00000268790	19	16663453	16795926	263	29	120000	-1.1918	0.88333	CTC-429P9.4	76353	25kb
ENSG00000214046	19	16716562	16796253	182	27	120000	-1.6278	0.94822	SMIM7	76353	25kb
ENSG00000072954	19	16746938	16825840	220	24	120000	-1.0878	0.86165	TMEM38A	76353	25kb
ENSG00000188039	19	16805787	16953774	384	55	120000	1.0637	0.14372	NWD1	76353	25kb
ENSG00000127511	19	16915211	17016164	223	33	120000	1.1581	0.12342	SIN3B	76353	25kb
ENSG00000127533	19	16974671	17028417	147	29	120000	0.044944	0.48208	F2RL3	76353	25kb
ENSG00000160111	19	16978758	17162625	606	92	120000	0.80682	0.20989	CPAMD8	76353	25kb
ENSG00000131351	19	17135539	17211435	246	30	120000	0.04979	0.48015	HAUS8	76353	25kb
ENSG00000099331	19	17161591	17350346	720	73	120000	0.81395	0.20784	MYO9B	76353	25kb
ENSG00000053501	19	17301155	17355638	222	30	120000	-0.08064	0.53214	USE1	76353	25kb
ENSG00000099330	19	17312013	17365028	209	33	120000	-0.91783	0.82065	OCEL1	76353	25kb
ENSG00000160113	19	17317692	17381749	234	39	120000	-1.0102	0.84381	NR2F6	76353	25kb
ENSG00000269095	19	17321749	17391257	258	34	120000	-1.1766	0.88031	AC010646.3	76353	25kb
ENSG00000130307	19	17334985	17418595	308	35	120000	-1.4256	0.92301	USHBP1	76353	25kb
ENSG00000105393	19	17353159	17417058	220	28	120000	-1.0033	0.84215	BABAM1	76353	25kb
ENSG00000269307	19	17353227	17418775	228	29	120000	-1.0609	0.85564	CTD-2278I10.6	76353	25kb
ENSG00000160117	19	17367454	17423455	195	24	120000	-1.0975	0.8638	ANKLE1	76353	25kb
ENSG00000127220	19	17377940	17446045	240	28	120000	-1.2815	0.9	ABHD8	76353	25kb
ENSG00000130312	19	17378418	17442652	234	27	120000	-1.2651	0.89708	MRPL34	76353	25kb
ENSG00000130311	19	17395327	17459110	222	25	120000	-1.5895	0.94402	DDA1	76353	25kb
ENSG00000074855	19	17409032	17470638	218	26	120000	-1.6983	0.95527	ANO8	76353	25kb
ENSG00000130299	19	17420729	17478544	202	28	120000	-1.4217	0.92245	GTPBP3	76353	25kb
ENSG00000130300	19	17437257	17513159	268	38	120000	-1.3114	0.90514	PLVAP	76353	25kb
ENSG00000130303	19	17488748	17541457	191	21	120000	-0.28681	0.61287	BST2	76353	25kb
ENSG00000141971	19	17491531	17569533	244	32	120000	-0.45685	0.67611	MVB12A	76353	25kb
ENSG00000188051	19	17521318	17584376	181	30	120000	-0.3135	0.62305	TMEM221	76353	25kb
ENSG00000269035	19	17522420	17596613	226	34	120000	0.13598	0.44592	CTD-2521M24.10	76353	25kb
ENSG00000171773	19	17541234	17596763	158	22	120000	0.24238	0.40424	NXNL1	76353	25kb
ENSG00000130304	19	17554578	17641977	246	34	120000	-0.015949	0.50636	SLC27A1	76353	25kb
ENSG00000130313	19	17597438	17657097	171	34	120000	-0.34713	0.63575	PGLS	76353	25kb
ENSG00000167483	19	17609110	17689648	233	44	120000	-0.34088	0.6334	FAM129C	76353	25kb
ENSG00000130309	19	17641403	17718971	259	36	120000	-0.18095	0.5718	COLGALT1	76353	25kb
ENSG00000130477	19	17687137	17824401	541	89	120000	-0.30956	0.62155	UNC13A	76353	25kb
ENSG00000130479	19	17805051	17870325	230	29	120000	-0.50773	0.69418	MAP1S	76353	25kb
ENSG00000130475	19	17833527	17924377	334	39	120000	-1.396	0.91864	FCHO1	76353	25kb
ENSG00000179913	19	17880637	17948891	256	54	120000	-1.4929	0.93227	B3GNT3	76353	25kb
ENSG00000248099	19	17902321	17957383	193	48	120000	-1.5468	0.93905	INSL3	76353	25kb
ENSG00000105639	19	17910589	17983880	258	53	120000	-1.1028	0.86494	JAK3	76353	25kb
ENSG00000105640	19	17945685	17999962	167	24	120000	0.46686	0.3203	RPL18A	76353	25kb
ENSG00000105641	19	17957782	18030983	206	29	120000	0.49103	0.3117	SLC5A5	76353	25kb
ENSG00000007080	19	18018825	18079800	141	32	120000	0.050955	0.47968	CCDC124	76353	25kb
ENSG00000105642	19	18037102	18135889	270	39	120000	0.14986	0.44044	KCNN1	76353	25kb
ENSG00000105643	19	18086941	18149911	178	34	120000	0.29533	0.38387	ARRDC2	76353	25kb
ENSG00000096996	19	18144805	18234754	355	37	120000	2.1322	0.016494	IL12RB1	76353	25kb
ENSG00000099308	19	18183603	18287502	333	27	120000	2.5985	0.0046817	MAST3	76353	25kb
ENSG00000105647	19	18238928	18306350	177	25	120000	2.4934	0.006326	PIK3R2	76353	25kb
ENSG00000268173	19	18238968	18313927	197	26	120000	2.8424	0.0022391	PIK3R2	76353	25kb
ENSG00000216490	19	18258972	18313927	138	21	120000	2.9478	0.0016004	IFI30	76353	25kb
ENSG00000254858	19	18278992	18332758	134	17	120000	3.0631	0.0010952	MPV17L2	76353	25kb
ENSG00000105649	19	18282594	18339884	145	17	120000	3.0935	0.00098897	RAB3A	76353	25kb
ENSG00000105650	19	18293771	18391229	319	25	120000	2.9046	0.0018386	PDE4C	76353	25kb
ENSG00000130518	19	18342908	18410319	256	19	120000	1.9356	0.02646	KIAA1683	76353	25kb
ENSG00000130522	19	18365563	18417432	214	26	120000	1.6745	0.047015	JUND	76353	25kb
ENSG00000130520	19	18392040	18459084	201	37	120000	0.82688	0.20415	LSM4	76353	25kb
ENSG00000130517	19	18426397	18505760	242	41	120000	0.038833	0.48451	PGPEP1	76353	25kb
ENSG00000130513	19	18460541	18524986	187	36	120000	-0.19734	0.57822	GDF15	76353	25kb
ENSG00000175489	19	18476954	18533427	152	26	120000	-0.65448	0.7436	LRRC25	76353	25kb
ENSG00000130511	19	18504674	18570372	120	13	120000	-0.093441	0.53722	SSBP4	76353	25kb
ENSG00000105655	19	18520198	18574111	96	9	120000	-0.061234	0.52441	ISYNA1	76353	25kb
ENSG00000105656	19	18528473	18657937	265	14	120000	-0.19637	0.57784	ELL	76353	25kb
ENSG00000105701	19	18617561	18679887	137	21	120000	-0.057789	0.52304	FKBP8	76353	25kb
ENSG00000105700	19	18643572	18705197	146	18	120000	-0.12885	0.55126	KXD1	76353	25kb
ENSG00000221983	19	18657540	18713360	148	17	120000	-0.5873	0.7215	UBA52	76353	25kb
ENSG00000006016	19	18658030	18743551	244	29	120000	-0.4791	0.68407	CRLF1	76353	25kb
ENSG00000006015	19	18674495	18728146	166	21	120000	-0.87067	0.80803	C19orf60	76353	25kb
ENSG00000105696	19	18693240	18756849	205	29	120000	-0.44458	0.67169	TMEM59L	76353	25kb
ENSG00000167487	19	18722775	18806309	250	33	120000	0.18129	0.42807	KLHL26	76353	25kb
ENSG00000105662	19	18769487	18918004	382	42	120000	-0.16919	0.56718	CRTC1	76353	25kb
ENSG00000105664	19	18868583	18927123	130	31	120000	-0.22522	0.5891	COMP	76353	25kb
ENSG00000005007	19	18917747	19004045	193	31	120000	0.78834	0.21525	UPF1	76353	25kb
ENSG00000130283	19	18954361	19031905	159	28	120000	0.6949	0.24356	GDF1	76353	25kb
ENSG00000223802	19	18954361	19032536	160	28	120000	0.67571	0.24961	CERS1	76353	25kb
ENSG00000105669	19	18985323	19055206	158	30	120000	0.59106	0.27724	COPE	76353	25kb
ENSG00000268193	19	18992822	19070206	174	37	120000	0.53626	0.29589	AC002985.3	76353	25kb
ENSG00000105671	19	19005484	19064436	126	27	120000	0.67975	0.24833	DDX49	76353	25kb
ENSG00000051128	19	19015010	19077070	134	36	120000	0.40116	0.34415	HOMER3	76353	25kb
ENSG00000064607	19	19076697	19169832	195	23	120000	0.85086	0.19742	SUGP2	76353	25kb
ENSG00000105676	19	19119384	19195563	150	16	120000	1.216	0.112	ARMC6	76353	25kb
ENSG00000181035	19	19149808	19248697	196	20	120000	0.81349	0.20797	SLC25A42	76353	25kb
ENSG00000064545	19	19204978	19274322	171	27	120000	0.74838	0.22712	TMEM161A	76353	25kb
ENSG00000064489	19	19231376	19327967	281	24	120000	0.84346	0.19948	MEF2BNB-MEF2B	76353	25kb
ENSG00000213999	19	19231377	19328400	281	24	120000	0.84346	0.19948	MEF2B	76353	25kb
ENSG00000254901	19	19262712	19328400	204	20	120000	0.99109	0.16082	MEF2BNB	76353	25kb
ENSG00000064490	19	19278008	19337678	161	21	120000	0.81873	0.20647	RFXANK	76353	25kb
ENSG00000184162	19	19287225	19339233	118	21	120000	0.79646	0.21288	NR2C2AP	76353	25kb
ENSG00000130287	19	19297782	19388042	212	30	120000	0.27363	0.39218	NCAN	76353	25kb
ENSG00000187664	19	19341450	19398605	159	20	120000	-0.06862	0.52735	HAPLN4	76353	25kb
ENSG00000213996	19	19350173	19409200	157	20	120000	-0.092123	0.5367	TM6SF2	76353	25kb
ENSG00000105705	19	19361827	19456653	223	20	120000	-0.30411	0.61948	SUGP1	76353	25kb
ENSG00000129933	19	19406490	19494563	194	17	120000	-0.64962	0.74203	MAU2	76353	25kb

ENSG00000167491	19	19471635	19644740	309	15	120000	-0.36667	0.64307	GATAD2A	76353	25kb
ENSG00000178093	19	19598227	19651838	105	10	120000	-0.5089	0.69459	TSSK6	76353	25kb
ENSG00000186010	19	19601545	19669285	137	13	120000	-0.57567	0.71758	NDUFA13	76353	25kb
ENSG00000258674	19	19602036	19671885	143	13	120000	-0.5725	0.71651	CTC-260F20.3	76353	25kb
ENSG00000250067	19	19602036	19673390	145	14	120000	-0.59763	0.72496	YJEFN3	76353	25kb
ENSG00000160161	19	19624057	19682468	105	12	120000	-0.54346	0.70659	CILP2	76353	25kb
ENSG00000105717	19	19647522	19754725	167	21	120000	-0.29613	0.61644	PBX4	76353	25kb
ENSG00000064547	19	19709477	19764739	104	20	120000	-0.12927	0.55143	LPAR2	76353	25kb
ENSG00000089639	19	19715285	19779476	139	26	120000	-0.41374	0.66047	GMIP	76353	25kb
ENSG00000105726	19	19731007	19799502	149	26	120000	-0.85613	0.80404	ATP13A1	76353	25kb
ENSG00000181896	19	19754605	19819318	192	31	120000	-1.181	0.8812	ZNF101	76353	25kb
ENSG00000105708	19	19796280	19868906	241	34	120000	-0.60851	0.72858	ZNF14	76353	25kb
ENSG00000081665	19	19871648	19957560	212	27	120000	1.2033	0.11442	ZNF506	76353	25kb
ENSG00000256771	19	19951695	20030483	213	25	120000	1.2551	0.10472	ZNF253	76353	25kb
ENSG00000184635	19	19986722	20071384	302	24	120000	0.34339	0.36565	ZNF93	76353	25kb
ENSG00000268461	19	20022769	20073639	215	25	120000	0.12161	0.45161	AC007204.1	76353	25kb
ENSG00000197124	19	20082867	20175315	388	24	120000	0.058503	0.47667	ZNF682	76353	25kb
ENSG00000213988	19	20163803	20262885	349	27	120000	-1.0722	0.85819	ZNF90	76353	25kb
ENSG00000256229	19	20253037	20336299	236	15	120000	-0.28257	0.61125	ZNF486	76353	25kb
ENSG00000237440	19	20693631	20773615	373	21	120000	0.17768	0.42949	ZNF737	76353	25kb
ENSG00000188171	19	20777867	20869402	419	24	120000	-0.36828	0.64367	ZNF626	76353	25kb
ENSG00000160229	19	20934110	21016922	481	18	120000	-0.8944	0.81445	ZNF66	76353	25kb
ENSG00000105750	19	21081028	21158503	444	14	120000	-0.39305	0.65286	ZNF85	76353	25kb
ENSG00000118620	19	21178426	21267856	236	16	120000	-0.25844	0.60196	ZNF430	76353	25kb
ENSG00000160352	19	21239965	21333073	316	17	120000	-0.35162	0.63744	ZNF714	76353	25kb
ENSG00000196705	19	21299827	21398034	305	22	120000	0.92235	0.17817	ZNF431	76353	25kb
ENSG00000182141	19	21448962	21537227	324	16	120000	0.10667	0.45753	ZNF708	76353	25kb
ENSG00000172687	19	21516732	21587104	324	11	120000	0.60886	0.27131	ZNF738	76353	25kb
ENSG00000196268	19	21554921	21635375	227	19	120000	0.33249	0.36976	ZNF493	76353	25kb
ENSG00000269237	19	21562961	21699068	378	20	120000	0.79234	0.21408	CTD-2561J22.3	76353	25kb
ENSG00000197013	19	21654484	21764072	304	15	120000	1.3434	0.089574	ZNF429	76353	25kb
ENSG00000197020	19	21880568	21975430	336	24	120000	0.5242	0.30007	ZNF100	76353	25kb
ENSG00000198521	19	21962752	22059927	265	26	120000	0.31894	0.37488	ZNF43	76353	25kb
ENSG00000160321	19	22090760	22218751	426	18	120000	-0.25468	0.60052	ZNF208	76353	25kb
ENSG00000197134	19	22210254	22299282	369	13	120000	-1.0243	0.84715	ZNF257	76353	25kb
ENSG00000196109	19	22336893	22404753	188	9	120000	0.98675	0.16188	ZNF676	76353	25kb
ENSG00000196350	19	22444210	22524978	145	21	120000	0.38042	0.35182	ZNF729	76353	25kb
ENSG00000197360	19	22548821	22740287	581	41	120000	-0.053912	0.5215	ZNF98	76353	25kb
ENSG00000229676	19	22792126	22875472	401	12	120000	0.47534	0.31727	ZNF492	76353	25kb
ENSG00000213973	19	22914007	22991909	340	13	120000	1.8345	0.033287	ZNF99	76353	25kb
ENSG00000269067	19	23133270	23210978	223	8	120000	1.6464	0.049843	ZNF728	76353	25kb
ENSG00000183850	19	23233012	23355021	305	18	120000	0.74526	0.22806	ZNF730	76353	25kb
ENSG00000196081	19	23379401	23458192	181	12	120000	-0.066321	0.52644	ZNF724P	76353	25kb
ENSG00000167232	19	23462793	23603362	468	25	120000	0.80068	0.21166	ZNF91	76353	25kb
ENSG00000197372	19	23683433	23895022	506	25	120000	1.0723	0.14178	ZNF675	76353	25kb
ENSG00000196172	19	23896997	23966693	143	22	120000	-0.18284	0.57254	ZNF681	76353	25kb
ENSG00000205246	19	23920807	24035937	239	34	120000	-0.35047	0.63701	RPSAP58	76353	25kb
ENSG00000213967	19	24072678	24152961	225	25	120000	-1.8553	0.96822	ZNF726	76353	25kb
ENSG00000213096	19	24191276	24337654	434	22	120000	-0.31496	0.62361	ZNF254	76353	25kb
ENSG00000169021	19	29673173	29729448	154	20	120000	0.18302	0.42739	UQCRFS1	76353	25kb
ENSG00000187135	19	29992406	30080386	403	42	120000	1.4737	0.070283	VSTM2B	76353	25kb
ENSG00000105171	19	30069924	30133144	255	32	120000	-0.096684	0.53851	POP4	76353	25kb
ENSG00000166289	19	30130963	30191364	163	27	120000	-0.18208	0.57224	PLEKHF1	76353	25kb
ENSG00000131943	19	30166721	30231364	231	32	120000	-0.55905	0.71194	C19orf12	76353	25kb
ENSG00000105173	19	30277805	30340215	144	21	120000	-0.76109	0.7767	CCNE1	76353	25kb
ENSG00000105176	19	30389551	30531611	288	19	120000	0.16234	0.43552	URI1	76353	25kb
ENSG00000198597	19	30694197	31229445	1309	145	120000	-0.39403	0.65322	ZNF536	76353	25kb
ENSG00000268953	19	31615362	31665757	120	17	120000	-0.86177	0.80559	AC020952.1	76353	25kb
ENSG00000121297	19	31740851	31865453	274	44	120000	-0.44014	0.67008	TSHZ3	76353	25kb
ENSG00000168813	19	32811500	32903573	229	28	120000	-0.77894	0.78199	ZNF507	76353	25kb
ENSG00000269093	19	32870915	32921466	101	17	120000	-0.78842	0.78478	AC007773.3	76353	25kb
ENSG00000178904	19	32871449	33001801	264	23	120000	-0.45929	0.67699	DPY19L3	76353	25kb
ENSG00000105185	19	33046974	33103358	187	21	120000	0.62711	0.26529	PDCD5	76353	25kb
ENSG00000105186	19	33062913	33192503	523	27	120000	0.13839	0.44497	ANKRD27	76353	25kb
ENSG00000186326	19	33141313	33194206	213	30	120000	-0.071381	0.52845	RGS9BP	76353	25kb
ENSG00000213965	19	33157867	33229702	222	30	120000	-0.24687	0.5975	NUDT19	76353	25kb
ENSG00000173809	19	33185659	33345483	405	23	120000	-0.43288	0.66745	TDRD12	76353	25kb
ENSG00000021488	19	33296415	33385672	347	27	120000	0.71591	0.23702	SLC7A9	76353	25kb
ENSG00000121289	19	33344902	33487897	581	31	120000	0.81844	0.20655	CEP89	76353	25kb
ENSG00000131944	19	33438115	33494128	214	34	120000	0.52924	0.29832	C19orf40	76353	25kb
ENSG00000131941	19	33444499	33580794	533	44	120000	0.088698	0.46466	RHPN2	76353	25kb
ENSG00000076650	19	33546786	33646448	307	19	120000	-0.88583	0.81215	GPATCH1	76353	25kb
ENSG00000166359	19	33597996	33691701	285	30	120000	-0.29436	0.61576	WDR88	76353	25kb
ENSG00000130881	19	33643509	33723694	259	35	120000	0.21952	0.41312	LRP3	76353	25kb
ENSG00000130876	19	33674570	33741756	213	44	120000	0.30163	0.38147	SLC7A10	76353	25kb
ENSG00000245848	19	33765840	33818470	172	28	120000	1.5668	0.058575	CEBPA	76353	25kb
ENSG00000153879	19	33839236	33898592	256	23	120000	1.2144	0.1123	CEBPG	76353	25kb
ENSG00000124299	19	33852856	34037700	779	44	120000	-0.022804	0.5091	PEPD	76353	25kb
ENSG00000124302	19	34087861	34289414	661	82	120000	1.51	0.065519	CHST8	76353	25kb
ENSG00000153885	19	34261838	34331668	212	37	120000	2.1223	0.016905	KCTD15	76353	25kb
ENSG00000257103	19	34638409	34745420	289	20	120000	0.90636	0.18237	LSM14A	76353	25kb
ENSG00000166398	19	34720442	34871491	342	26	120000	0.84222	0.19983	KIAA0355	76353	25kb
ENSG00000105220	19	34825385	34918061	206	27	120000	0.015027	0.49401	GPI	76353	25kb
ENSG00000266953	19	34862220	34925269	132	19	120000	-1.1006	0.86447	RP11-618P17.4	76353	25kb
ENSG00000126249	19	34870289	34942073	147	20	120000	-0.91115	0.81889	PDCD2L	76353	25kb
ENSG00000126261	19	34894257	34985853	202	22	120000	-0.10244	0.5408	UBA2	76353	25kb
ENSG00000142279	19	34946874	35022258	216	41	120000	1.1143	0.13258	WTIP	76353	25kb
ENSG00000205209	19	35059234	35191604	527	35	120000	0.65274	0.25696	SCGB2B2	76353	25kb
ENSG00000089335	19	35143544	35202302	235	24	120000	1.3123	0.094703	ZNF302	76353	25kb
ENSG00000197841	19	35200060	35258777	164	27	120000	1.9052	0.028377	ZNF181	76353	25kb
ENSG00000153896	19	35223981	35289134	149	25	120000	1.1753	0.11994	ZNF599	76353	25kb
ENSG00000168661	19	35392807	35461074	198	17	120000	1.2312	0.10913	ZNF30	76353	25kb
ENSG00000180884	19	35422258	35479953	202	25	120000	0.79322	0.21382	ZNF792	76353	25kb
ENSG00000089351	19	35460688	35542375	243	32	120000	0.22058	0.41271	GRAMD1A	76353	25kb
ENSG00000105711	19	35496588	35556352	179	23	120000	0.43627	0.33132	SCN1B	76353	25kb
ENSG00000105707	19	35506410	35582475	238	34	120000	0.32621	0.37213	HPN	76353	25kb

ENSG00000179066	19	35571873	35623141	153	27	120000	0.84673	0.19857	AC020907.1	76353	25kb
ENSG00000089356	19	35581732	35640228	163	23	120000	0.45894	0.32314	FXYD3	76353	25kb
ENSG00000153902	19	35590417	35658355	214	35	120000	-0.48315	0.68551	LGI4	76353	25kb
ENSG00000266964	19	35604712	35659013	175	31	120000	-1.586	0.94363	FXYD1	76353	25kb
ENSG00000221946	19	35609154	35670204	217	35	120000	-1.599	0.94509	FXYD7	76353	25kb
ENSG00000089327	19	35620633	35685786	276	31	120000	-0.66243	0.74615	FXYD5	76353	25kb
ENSG00000177558	19	35690703	35744632	219	29	120000	0.30331	0.38083	FAM187B	76353	25kb
ENSG00000105699	19	35714233	35783867	228	25	120000	-0.32655	0.628	LSR	76353	25kb
ENSG00000105698	19	35734881	35795724	166	23	120000	-0.77105	0.77966	USF2	76353	25kb
ENSG00000105697	19	35746619	35801046	141	21	120000	-0.68426	0.75309	HAMP	76353	25kb
ENSG00000105695	19	35758028	35829707	183	36	120000	-0.22673	0.58968	MAG	76353	25kb
ENSG00000012124	19	35785164	35863258	230	58	120000	1.4615	0.071944	CD22	76353	25kb
ENSG00000126266	19	35817445	35868367	178	50	120000	2.0378	0.020786	FFAR1	76353	25kb
ENSG00000185897	19	35824362	35876387	200	50	120000	1.8206	0.034331	FFAR3	76353	25kb
ENSG00000126251	19	35836831	35888855	202	38	120000	1.5101	0.065511	GPR42	76353	25kb
ENSG00000126262	19	35909809	35967669	218	51	120000	0.4237	0.33589	FFAR2	76353	25kb
ENSG00000188508	19	35953226	36011460	180	34	120000	-0.49362	0.68921	KRTDAP	76353	25kb
ENSG00000161249	19	35963122	36029560	230	45	120000	-0.44515	0.6719	DMKN	76353	25kb
ENSG00000189001	19	35989269	36044253	184	38	120000	-0.51797	0.69776	SBSN	76353	25kb
ENSG00000105679	19	35999314	36061218	184	36	120000	-0.51081	0.69526	GAPDHS	76353	25kb
ENSG00000105677	19	36011497	36063428	152	29	120000	-0.36419	0.64214	TMEM147	76353	25kb
ENSG00000105675	19	36015945	36079560	172	37	120000	-0.5034	0.69266	ATP4A	76353	25kb
ENSG00000249115	19	36078646	36141251	135	24	120000	0.82715	0.20408	HAUS5	76353	25kb
ENSG00000126254	19	36094932	36153588	124	23	120000	0.38533	0.35	RBM42	76353	25kb
ENSG00000105672	19	36107647	36160773	130	23	120000	0.27709	0.39086	ETV2	76353	25kb
ENSG00000126267	19	36114125	36174763	168	23	120000	0.25968	0.39756	COX6B1	76353	25kb
ENSG00000105668	19	36132715	36194367	184	20	120000	0.18455	0.42679	UPK1A	76353	25kb
ENSG00000011590	19	36170429	36232940	158	22	120000	-0.20547	0.5814	ZBTB32	76353	25kb
ENSG00000272333	19	36183921	36254779	160	23	120000	-0.35549	0.63889	KMT2B	76353	25kb
ENSG00000126246	19	36205058	36258354	116	20	120000	-0.29424	0.61571	IGFLR1	76353	25kb
ENSG00000267120	19	36205153	36261333	128	22	120000	-0.26105	0.60297	AD000671.6	76353	25kb
ENSG00000161265	19	36208365	36261346	119	21	120000	-0.20231	0.58016	U2AF1L4	76353	25kb
ENSG00000205155	19	36211015	36262911	120	21	120000	-0.26009	0.6026	PSENEN	76353	25kb
ENSG00000188223	19	36211579	36270420	155	23	120000	-0.38218	0.64884	AC002398.9	76353	25kb
ENSG00000267796	19	36214262	36270420	150	22	120000	-0.46135	0.67773	LIN37	76353	25kb
ENSG00000004776	19	36220469	36273980	145	22	120000	-0.39738	0.65446	HSPB6	76353	25kb
ENSG00000167595	19	36224044	36286930	166	23	120000	-0.35804	0.63984	C19orf55	76353	25kb
ENSG00000004777	19	36240434	36304724	163	25	120000	-0.36334	0.64183	ARHGAP33	76353	25kb
ENSG00000250799	19	36265890	36329201	169	34	120000	0.90751	0.18207	PRODH2	76353	25kb
ENSG00000161270	19	36291866	36385189	206	21	120000	0.54362	0.29335	NPHS1	76353	25kb
ENSG00000126259	19	36321763	36383011	129	8	120000	0.034482	0.48625	KIRREL2	76353	25kb
ENSG00000105290	19	36333801	36395693	133	8	120000	-0.26204	0.60335	APLP1	76353	25kb
ENSG00000167604	19	36353555	36418205	147	9	120000	-0.43086	0.66672	NFKBID	76353	25kb
ENSG00000126264	19	36368382	36420279	136	10	120000	-0.37734	0.64704	HCST	76353	25kb
ENSG00000011600	19	36370303	36424197	142	10	120000	-0.32639	0.62794	TYROBP	76353	25kb
ENSG00000126243	19	36401260	36461669	126	14	120000	-0.46561	0.67925	LRFN3	76353	25kb
ENSG00000205138	19	36461090	36512220	89	27	120000	-1.6452	0.95004	SDHAF1	76353	25kb
ENSG00000181392	19	36469209	36524695	102	26	120000	-1.9013	0.97137	SYNE4	76353	25kb
ENSG00000239382	19	36475022	36530141	100	25	120000	-2.0626	0.98042	ALKBH6	76353	25kb
ENSG00000105270	19	36480562	36549245	138	28	120000	-2.2561	0.98797	CLIP3	76353	25kb
ENSG00000161277	19	36500887	36570664	142	24	120000	-2.0269	0.97866	THAP8	76353	25kb
ENSG00000075702	19	36520783	36621008	253	21	120000	-1.6019	0.94541	WDR62	76353	25kb
ENSG00000105261	19	36577104	36629613	153	23	120000	-1.2769	0.89919	OVOL3	76353	25kb
ENSG00000105258	19	36579612	36631248	144	23	120000	-1.2237	0.88947	POLR2I	76353	25kb
ENSG00000105254	19	36580191	36641849	168	30	120000	-0.85932	0.80492	TBCB	76353	25kb
ENSG00000126247	19	36605477	36666255	170	43	120000	0.36868	0.35618	CAPNS1	76353	25kb
ENSG00000161281	19	36616824	36668771	149	38	120000	0.81671	0.20705	COX7A1	76353	25kb
ENSG00000196357	19	36648188	36762159	326	22	120000	-0.031043	0.51238	ZNF565	76353	25kb
ENSG00000167635	19	36680504	36754676	221	19	120000	-0.45263	0.67459	ZNF146	76353	25kb
ENSG00000142065	19	36802162	36895101	200	15	120000	-0.6583	0.74483	ZFP14	76353	25kb
ENSG00000181007	19	36849022	36934558	185	20	120000	-0.50484	0.69316	ZFP82	76353	25kb
ENSG00000186017	19	36911021	37005804	225	24	120000	0.47614	0.31699	ZNF566	76353	25kb
ENSG00000254004	19	36976597	37044562	190	20	120000	0.56101	0.2874	ZNF260	76353	25kb
ENSG00000186020	19	37000676	37121178	262	20	120000	0.24816	0.402	ZNF529	76353	25kb
ENSG00000161298	19	37070719	37144499	130	15	120000	-0.021491	0.50857	ZNF382	76353	25kb
ENSG00000197808	19	37103094	37182755	161	15	120000	-0.21361	0.58458	ZNF461	76353	25kb
ENSG00000189042	19	37153514	37243603	187	17	120000	-0.34919	0.63653	ZNF567	76353	25kb
ENSG00000267041	19	37180285	37288727	230	23	120000	-0.59136	0.72286	ZNF850	76353	25kb
ENSG00000267260	19	37239055	37292980	117	17	120000	-0.77201	0.77995	CTD-2162K18.4	76353	25kb
ENSG00000197863	19	37283330	37366689	133	19	120000	-0.26718	0.60534	ZNF790	76353	25kb
ENSG00000251247	19	37316263	37428931	226	22	120000	-0.69378	0.75609	ZNF345	76353	25kb
ENSG00000185869	19	37354026	37432193	177	16	120000	-0.70934	0.76094	ZNF829	76353	25kb
ENSG00000198453	19	37382231	37514602	445	16	120000	-0.49435	0.68947	ZNF568	76353	25kb
ENSG00000197050	19	37473759	37646216	438	14	120000	-0.17879	0.57095	ZNF420	76353	25kb
ENSG00000267360	19	37558921	37726414	284	21	120000	0.24243	0.40422	CTC-454I21.3	76353	25kb
ENSG00000196967	19	37572636	37688643	210	15	120000	0.26455	0.39568	ZNF585A	76353	25kb
ENSG00000245680	19	37647481	37734055	129	20	120000	0.44976	0.32644	ZNF585B	76353	25kb
ENSG00000188283	19	37683828	37759828	112	19	120000	0.45173	0.32573	ZNF383	76353	25kb
ENSG00000181666	19	37778739	37885267	175	21	120000	0.65308	0.25685	HKR1	76353	25kb
ENSG00000189164	19	37836963	37908968	120	17	120000	0.68009	0.24822	ZNF527	76353	25kb
ENSG00000196437	19	37877057	37985177	174	21	120000	0.51963	0.30166	ZNF569	76353	25kb
ENSG00000171827	19	37933487	38001260	114	15	120000	0.39203	0.34752	ZNF570	76353	25kb
ENSG00000188227	19	37972841	38064664	162	24	120000	0.74819	0.22717	ZNF793	76353	25kb
ENSG00000171817	19	38017308	38129998	207	22	120000	0.77112	0.22032	ZNF540	76353	25kb
ENSG00000180479	19	38020684	38110673	169	19	120000	0.81421	0.20776	ZNF571	76353	25kb
ENSG00000120784	19	38079650	38208238	244	25	120000	0.77947	0.21785	ZFP30	76353	25kb
ENSG00000196381	19	38133652	38208223	161	19	120000	0.81743	0.20684	ZNF781	76353	25kb
ENSG00000267552	19	38155766	38235089	167	20	120000	0.55742	0.28862	CTD-2528L19.4	76353	25kb
ENSG00000198182	19	38162264	38235691	156	19	120000	0.57118	0.28394	ZNF607	76353	25kb
ENSG00000189144	19	38201734	38332940	270	23	120000	0.6928	0.24422	ZNF573	76353	25kb
ENSG00000171804	19	38350463	38422317	151	19	120000	0.41337	0.33967	WDR87	76353	25kb
ENSG00000105738	19	38372868	38724012	935	56	120000	-0.94646	0.82804	SIPA1L3	76353	25kb
ENSG00000011332	19	38676646	38745354	173	38	120000	-0.56605	0.71432	DPF1	76353	25kb
ENSG00000167642	19	38709675	38808254	283	33	120000	0.02938	0.48828	SPINT2	76353	25kb
ENSG00000167641	19	38716877	38772231	159	26	120000	-0.77133	0.77974	PPP1R14A	76353	25kb
ENSG00000267748	19	38754791	38820550	197	27	120000	0.66867	0.25185	CTB-102L5.4	76353	25kb

ENSG00000167644	19	38769801	38820649	150	20	120000	0.86098	0.19462	C19orf33	76353	25kb
ENSG00000167645	19	38770753	38832913	194	23	120000	0.90082	0.18384	YIF1B	76353	25kb
ENSG00000099337	19	38785484	38844660	186	21	120000	1.0767	0.14081	KCNK6	76353	25kb
ENSG00000099338	19	38801415	38886589	255	20	120000	0.90474	0.1828	CATSPERG	76353	25kb
ENSG00000099341	19	38840176	38899464	194	17	120000	0.7898	0.21482	PSMD8	76353	25kb
ENSG00000179168	19	38849905	38903722	185	17	120000	0.58808	0.27824	GGN	76353	25kb
ENSG00000188766	19	38854061	38911881	193	19	120000	0.48977	0.31215	SPRED3	76353	25kb
ENSG00000130244	19	38868775	38924728	176	18	120000	0.48157	0.31506	FAM98C	76353	25kb
ENSG00000171777	19	38874695	38941945	203	25	120000	0.28956	0.38607	RASGRP4	76353	25kb
ENSG00000196218	19	38899339	39103204	622	49	120000	-0.42926	0.66613	RYR1	76353	25kb
ENSG00000104814	19	39053281	39134522	144	20	120000	-0.51022	0.69505	MAP4K1	76353	25kb
ENSG00000178982	19	39084735	39152595	137	24	120000	-0.83783	0.79894	EIF3K	76353	25kb
ENSG00000130402	19	39113289	39247223	367	21	120000	0.72887	0.23304	ACTN4	76353	25kb
ENSG00000182472	19	39195827	39285544	324	23	120000	0.30721	0.37934	CAPN12	76353	25kb
ENSG00000205076	19	39236611	39289132	200	22	120000	-0.85897	0.80482	LGALS7	76353	25kb
ENSG00000178934	19	39254851	39307389	176	22	120000	-1.2326	0.89114	LGALS7B	76353	25kb
ENSG00000171747	19	39267311	39329004	184	27	120000	-1.7076	0.95615	LGALS4	76353	25kb
ENSG00000104823	19	39281062	39347645	183	24	120000	-1.6273	0.94817	ECH1	76353	25kb
ENSG00000268083	19	39283111	39355818	195	25	120000	-1.6364	0.94912	AC104534.3	76353	25kb
ENSG00000104824	19	39302028	39367987	140	16	120000	-1.2182	0.88842	HNRNPL	76353	25kb
ENSG00000187994	19	39333470	39393919	115	24	120000	-1.3039	0.90387	RINL	76353	25kb
ENSG00000068903	19	39344197	39415502	147	32	120000	-1.0558	0.85447	SIRT2	76353	25kb
ENSG00000104825	19	39365340	39424533	133	26	120000	-0.64205	0.73958	NFKBIB	76353	25kb
ENSG00000262484	19	39374620	39427798	123	21	120000	-0.45756	0.67637	CCER2	76353	25kb
ENSG00000104835	19	39380906	39465495	218	27	120000	-0.26208	0.60337	SARS2	76353	25kb
ENSG00000269547	19	39381044	39465495	218	27	120000	-0.26208	0.60337	CTC-360G5.8	76353	25kb
ENSG00000128626	19	39396188	39448802	136	17	120000	-0.27354	0.60778	MRPS12	76353	25kb
ENSG00000269190	19	39407044	39491550	275	24	120000	-0.36048	0.64076	FBXO17	76353	25kb
ENSG00000161243	19	39456354	39548425	463	21	120000	-0.13859	0.55511	FBXO27	76353	25kb
ENSG00000183760	19	39549553	39627133	289	22	120000	-1.3983	0.91899	PAPL	76353	25kb
ENSG00000130669	19	39591410	39698456	317	27	120000	-0.82991	0.7967	PAK4	76353	25kb
ENSG00000188505	19	39662601	39717524	107	14	120000	-0.40356	0.65673	NCCRP1	76353	25kb
ENSG00000179751	19	39668471	39719906	102	12	120000	-0.44894	0.67326	SYCN	76353	25kb
ENSG00000197110	19	39709246	39760646	110	27	120000	-0.13413	0.55335	IFNL3	76353	25kb
ENSG00000183709	19	39734154	39785732	190	20	120000	-0.091329	0.53638	IFNL2	76353	25kb
ENSG00000182393	19	39761965	39814313	171	15	120000	-0.42015	0.66281	IFNL1	76353	25kb
ENSG00000128011	19	39772208	39830976	170	18	120000	-0.69563	0.75667	LRFN1	76353	25kb
ENSG00000130755	19	39793993	39858012	140	16	120000	-0.34563	0.63519	GMFG	76353	25kb
ENSG00000179134	19	39808036	39901350	205	24	120000	-0.068376	0.52726	SAMD4B	76353	25kb
ENSG00000006712	19	39851492	39906835	113	21	120000	0.25431	0.39963	PAF1	76353	25kb
ENSG00000063322	19	39856943	39916277	125	27	120000	0.28787	0.38672	MED29	76353	25kb
ENSG00000128016	19	39872453	39925052	105	24	120000	0.34045	0.36676	ZFP36	76353	25kb
ENSG00000090924	19	39878225	39944054	158	25	120000	-0.14156	0.55628	PLEKHG2	76353	25kb
ENSG00000105193	19	39898847	39951588	139	17	120000	-0.69167	0.75543	RPS16	76353	25kb
ENSG00000196235	19	39901796	39992310	240	21	120000	-0.62485	0.73397	SUPT5H	76353	25kb
ENSG00000213922	19	39905212	39957082	161	17	120000	-0.63655	0.73779	AC011500.1	76353	25kb
ENSG00000105197	19	39946052	40009422	161	17	120000	-0.52752	0.70108	TIMM50	76353	25kb
ENSG00000090932	19	39964535	40024121	139	21	120000	-0.81376	0.79211	DLL3	76353	25kb
ENSG00000186838	19	39980753	40036326	136	24	120000	-0.98681	0.83813	SELV	76353	25kb
ENSG00000176401	19	39996630	40048494	137	22	120000	-1.2665	0.89733	EID2B	76353	25kb
ENSG00000176396	19	40003890	40055870	141	20	120000	-1.3253	0.90746	EID2	76353	25kb
ENSG00000105198	19	40068164	40123116	182	18	120000	-1.1829	0.88157	LGALS13	76353	25kb
ENSG00000249861	19	40121534	40176287	135	17	120000	-0.54414	0.70683	LGALS16	76353	25kb
ENSG00000006659	19	40169946	40225084	174	15	120000	-0.24524	0.59687	LGALS14	76353	25kb
ENSG00000105205	19	40196890	40253668	213	17	120000	-0.72687	0.76635	CLC	76353	25kb
ENSG00000213921	19	40242235	40301775	191	24	120000	-0.28925	0.6138	LEUTX	76353	25kb
ENSG00000105204	19	40290990	40349841	181	22	120000	-0.36881	0.64387	DYRK1B	76353	25kb
ENSG00000105202	19	40300098	40362054	217	30	120000	-0.5582	0.71164	FBL	76353	25kb
ENSG00000090920	19	40328963	40465533	348	51	120000	-0.42104	0.66314	FCGBP	76353	25kb
ENSG00000013275	19	40451912	40512348	171	16	120000	-0.81017	0.79108	PSMC4	76353	25kb
ENSG00000187187	19	40465041	40548514	207	15	120000	-1.0093	0.84359	ZNF546	76353	25kb
ENSG00000128000	19	40509167	40587116	173	12	120000	-1.1344	0.87169	ZNF780B	76353	25kb
ENSG00000197782	19	40550059	40621845	226	15	120000	-0.4769	0.68328	ZNF780A	76353	25kb
ENSG00000130758	19	40672651	40746482	177	24	120000	1.2099	0.11317	MAP3K10	76353	25kb
ENSG00000174521	19	40696965	40749306	95	20	120000	0.6202	0.26756	TTC9B	76353	25kb
ENSG00000105219	19	40703115	40757597	91	20	120000	0.74853	0.22707	CNTD2	76353	25kb
ENSG00000105221	19	40711224	40816443	200	25	120000	0.45133	0.32588	AKT2	76353	25kb
ENSG00000160392	19	40800443	40879434	174	22	120000	-0.13325	0.553	C19orf47	76353	25kb
ENSG00000105223	19	40829363	40911346	187	34	120000	-0.068482	0.5273	PLD3	76353	25kb
ENSG00000160396	19	40860178	40921094	124	30	120000	0.51219	0.30426	HIPK4	76353	25kb
ENSG00000105227	19	40874675	40944273	131	29	120000	0.99809	0.15912	PRX	76353	25kb
ENSG00000197019	19	40902499	40956932	116	20	120000	1.2822	0.09989	SERTAD1	76353	25kb
ENSG00000167565	19	40921751	40975612	138	21	120000	1.3011	0.09662	SERTAD3	76353	25kb
ENSG00000090013	19	40928696	40996747	191	35	120000	1.1171	0.13197	BLVRB	76353	25kb
ENSG00000160460	19	40947148	41107370	404	64	120000	0.15977	0.43653	SPTBN4	76353	25kb
ENSG00000160410	19	41057757	41122305	159	26	120000	-1.9222	0.97271	SHKBP1	76353	25kb
ENSG00000090006	19	41073789	41160725	249	35	120000	-2.068	0.98068	LTBP4	76353	25kb
ENSG00000105245	19	41147596	41221877	220	32	120000	-0.34137	0.63359	NUMBL	76353	25kb
ENSG00000123815	19	41172434	41249112	195	27	120000	0.57003	0.28433	ADCK4	76353	25kb
ENSG00000086544	19	41198008	41271765	189	24	120000	0.73483	0.23122	ITPKC	76353	25kb
ENSG00000188493	19	41221761	41282458	162	21	120000	0.5736	0.28312	C19orf54	76353	25kb
ENSG00000077312	19	41231542	41296294	172	22	120000	0.053877	0.47852	SNRPA	76353	25kb
ENSG00000261857	19	41252553	41308395	163	22	120000	-0.23278	0.59203	MIA	76353	25kb
ENSG00000268975	19	41252553	41317590	189	24	120000	0.12655	0.44965	MIA-RAB4B	76353	25kb
ENSG00000167578	19	41259121	41327847	195	25	120000	0.073442	0.47073	RAB4B	76353	25kb
ENSG00000171570	19	41259147	41339103	235	33	120000	0.057249	0.47717	RAB4B-EGLN2	76353	25kb
ENSG00000269858	19	41279901	41339338	183	31	120000	0.30658	0.37958	EGLN2	76353	25kb
ENSG00000268797	19	41282202	41429187	503	48	120000	-0.45766	0.6764	CTC-490E21.12	76353	25kb
ENSG00000255974	19	41324443	41381352	166	29	120000	0.11337	0.45487	CYP2A6	76353	25kb
ENSG00000198077	19	41356344	41413657	209	28	120000	-1.1524	0.87543	CYP2A7	76353	25kb
ENSG00000197408	19	41472204	41549303	336	28	120000	-1.5165	0.9353	CYP2B6	76353	25kb
ENSG00000197838	19	41569377	41627100	193	16	120000	-0.7341	0.76856	CYP2A13	76353	25kb
ENSG00000197446	19	41595337	41659271	270	26	120000	-0.79973	0.78807	CYP2F1	76353	25kb
ENSG00000167600	19	41673954	41738444	191	40	120000	0.79161	0.21429	CYP2S1	76353	25kb
ENSG00000167601	19	41700108	41792671	209	36	120000	0.41071	0.34064	AXL	76353	25kb
ENSG00000105323	19	41743401	41838503	175	15	120000	0.052227	0.47917	HNRNPUL1	76353	25kb

ENSG00000105329	19	41782492	41884816	195	25	120000	0.055823	0.47774	TGFB1	76353	25kb
ENSG00000142039	19	41791094	41855788	122	15	120000	0.058592	0.47664	CCDC97	76353	25kb
ENSG00000255730	19	41831758	41955906	274	21	120000	0.92063	0.17862	CTC-435M10.3	76353	25kb
ENSG00000142046	19	41831816	41914988	179	21	120000	0.94075	0.17342	TMEM91	76353	25kb
ENSG00000123810	19	41835326	41895078	116	18	120000	0.61141	0.27046	B9D2	76353	25kb
ENSG00000248098	19	41859215	41955910	224	15	120000	1.0832	0.13936	BCKDHA	76353	25kb
ENSG00000077348	19	41867279	41928384	140	9	120000	1.1176	0.13187	EXOSC5	76353	25kb
ENSG00000177191	19	41906264	41959635	123	8	120000	0.99952	0.15877	B3GNT8	76353	25kb
ENSG00000105341	19	41912223	41971622	138	13	120000	1.0808	0.13989	ATP5SL	76353	25kb
ENSG00000204978	19	41924063	41975670	112	16	120000	1.1581	0.12342	C19orf69	76353	25kb
ENSG00000007129	19	42030886	42118197	220	27	120000	-1.7075	0.95613	CEACAM21	76353	25kb
ENSG00000105352	19	42100344	42158442	194	30	120000	-1.9554	0.97473	CEACAM4	76353	25kb
ENSG00000007306	19	42152235	42235895	246	26	120000	-0.51979	0.69839	CEACAM7	76353	25kb
ENSG00000105388	19	42187504	42258718	199	22	120000	-0.11721	0.54665	CEACAM5	76353	25kb
ENSG00000267881	19	42187531	42290190	251	30	120000	-0.2637	0.60399	CEA	76353	25kb
ENSG00000086548	19	42229885	42301113	118	20	120000	0.035956	0.48566	CEACAM6	76353	25kb
ENSG00000170956	19	42275369	42340591	109	7	120000	-0.9972	0.84067	CEACAM3	76353	25kb
ENSG00000183103	19	42316148	42373760	98	7	120000	-0.87859	0.81019	LYPD4	76353	25kb
ENSG00000142025	19	42323806	42381401	90	7	120000	-0.85147	0.80274	DMRTC2	76353	25kb
ENSG00000105372	19	42338988	42401994	77	8	120000	-0.74144	0.77079	RPS19	76353	25kb
ENSG00000105369	19	42356190	42410439	57	6	120000	-0.35103	0.63722	CD79A	76353	25kb
ENSG00000076928	19	42362228	42459302	115	17	120000	0.3574	0.3604	ARHGEF1	76353	25kb
ENSG00000105404	19	42435833	42488542	61	12	120000	0.11116	0.45575	RABAC1	76353	25kb
ENSG00000105409	19	42445734	42526649	70	21	120000	0.763	0.22273	ATP1A3	76353	25kb
ENSG00000105737	19	42477473	42598650	80	19	120000	2.2957	0.010848	GRIK5	76353	25kb
ENSG00000105732	19	42547629	42610701	49	13	120000	2.5611	0.0052169	ZNF574	76353	25kb
ENSG00000028277	19	42565263	42725737	158	12	120000	2.6664	0.0038335	POU2F2	76353	25kb
ENSG00000160570	19	42677750	42749292	78	8	120000	2.1777	0.014715	DEDD2	76353	25kb
ENSG00000167625	19	42699423	42757353	61	7	120000	2.1782	0.014696	ZNF526	76353	25kb
ENSG00000105723	19	42709338	42771777	77	8	120000	2.1842	0.014474	GSK3A	76353	25kb
ENSG00000268643	19	42713735	42784284	83	8	120000	1.9671	0.024585	AC006486.9	76353	25kb
ENSG00000204957	19	42721927	42774125	69	7	120000	2.0749	0.018998	AC006486.1	76353	25kb
ENSG00000105722	19	42726724	42784309	76	7	120000	1.8896	0.029404	ERF	76353	25kb
ENSG00000079432	19	42747689	42824949	90	8	120000	1.5705	0.058153	CIC	76353	25kb
ENSG00000079462	19	42776185	42832698	53	5	120000	0.90703	0.1822	PAFAH1B3	76353	25kb
ENSG00000188368	19	42781250	42839973	51	6	120000	0.91309	0.1806	PRR19	76353	25kb
ENSG00000167619	19	42792477	42854214	61	5	120000	0.94198	0.1731	TMEM145	76353	25kb
ENSG00000105429	19	42804761	42907921	117	8	120000	0.70542	0.24027	MEGF8	76353	25kb
ENSG00000105427	19	42866171	42919439	58	6	120000	0.3019	0.38136	CNFN	76353	25kb
ENSG00000079435	19	42880659	42956578	76	8	120000	0.68093	0.24796	LIPE	76353	25kb
ENSG00000189377	19	42907440	42972200	49	7	120000	1.1911	0.1168	CXCL17	76353	25kb
ENSG00000079385	19	42986456	43090386	85	9	120000	2.0384	0.020756	CEACAM1	76353	25kb
ENSG00000124469	19	43059393	43124207	60	5	120000	1.8962	0.028967	CEACAM8	76353	25kb
ENSG00000221826	19	43200790	43269721	177	10	120000	0.94116	0.17331	PSG3	76353	25kb
ENSG00000124467	19	43231838	43384843	508	19	120000	1.9979	0.022863	PSG8	76353	25kb
ENSG00000231924	19	43345616	43408974	197	9	120000	1.7612	0.039102	PSG1	76353	25kb
ENSG00000170848	19	43381231	43448715	246	11	120000	1.2452	0.10652	PSG6	76353	25kb
ENSG00000243130	19	43486808	43555664	205	15	120000	-0.94445	0.82753	PSG11	76353	25kb
ENSG00000242221	19	43543363	43612197	327	17	120000	-2.3114	0.98959	PSG2	76353	25kb
ENSG00000204941	19	43645408	43715688	151	19	120000	-0.50905	0.69464	PSG5	76353	25kb
ENSG00000243137	19	43671854	43736451	109	18	120000	-0.31984	0.62546	PSG4	76353	25kb
ENSG00000183668	19	43690943	43798682	285	28	120000	-0.29363	0.61548	PSG9	76353	25kb
ENSG00000131126	19	43880648	43947763	245	23	120000	-0.52858	0.70145	TEX101	76353	25kb
ENSG00000124466	19	43939939	43994812	183	32	120000	-1.0502	0.8532	LYPD3	76353	25kb
ENSG00000176531	19	43954106	44034087	205	27	120000	-0.045172	0.51801	PHLDB3	76353	25kb
ENSG00000105755	19	43985871	44056396	198	27	120000	-0.30065	0.61816	ETHE1	76353	25kb
ENSG00000176472	19	44004649	44065282	184	26	120000	-0.1676	0.56655	ZNF575	76353	25kb
ENSG00000073050	19	44022192	44109625	248	35	120000	-0.39048	0.65191	XRCC1	76353	25kb
ENSG00000268361	19	44033844	44122188	236	35	120000	-0.27473	0.60824	L34079.2	76353	25kb
ENSG00000234465	19	44055952	44113116	152	28	120000	-0.17247	0.56847	PINLYP	76353	25kb
ENSG00000167378	19	44063521	44125287	140	26	120000	0.26331	0.39616	IRGQ	76353	25kb
ENSG00000124444	19	44075544	44130309	106	21	120000	0.78305	0.2168	ZNF576	76353	25kb
ENSG00000226763	19	44075769	44143649	131	22	120000	0.86599	0.19325	SRRM5	76353	25kb
ENSG00000131116	19	44086371	44149026	102	17	120000	1.0631	0.14386	ZNF428	76353	25kb
ENSG00000105767	19	44101520	44168991	166	28	120000	0.7241	0.2345	CADM4	76353	25kb
ENSG00000011422	19	44125247	44199699	242	49	120000	0.028732	0.48854	PLAUR	76353	25kb
ENSG00000124449	19	44195162	44249173	174	32	120000	-0.60902	0.72874	IRGC	76353	25kb
ENSG00000105771	19	44210301	44284142	213	30	120000	-0.73459	0.7687	SMG9	76353	25kb
ENSG00000104783	19	44245685	44310409	231	28	120000	-1.037	0.85013	KCNN4	76353	25kb
ENSG00000159871	19	44275081	44356358	344	22	120000	-1.2891	0.90133	LYPD5	76353	25kb
ENSG00000167637	19	44306444	44378307	249	21	120000	-1.0491	0.85294	ZNF283	76353	25kb
ENSG00000176222	19	44351515	44430537	177	9	120000	-0.67381	0.74978	ZNF404	76353	25kb
ENSG00000124459	19	44391781	44464430	224	18	120000	-1.1972	0.88439	ZNF45	76353	25kb
ENSG00000159905	19	44430375	44496861	255	17	120000	-1.3147	0.90569	ZNF221	76353	25kb
ENSG00000204920	19	44447014	44527477	250	25	120000	-0.83367	0.79777	ZNF155	76353	25kb
ENSG00000159882	19	44482100	44543078	163	17	120000	0.057092	0.47724	ZNF230	76353	25kb
ENSG00000159885	19	44504494	44562265	123	13	120000	1.1324	0.12873	ZNF222	76353	25kb
ENSG00000267022	19	44504506	44616471	250	20	120000	0.81364	0.20792	ZNF223	76353	25kb
ENSG00000178386	19	44530520	44597144	141	12	120000	1.1424	0.12664	ZNF223	76353	25kb
ENSG00000186026	19	44551297	44618766	157	16	120000	0.57294	0.28334	ZNF284	76353	25kb
ENSG00000267680	19	44573492	44637479	138	13	120000	0.1304	0.44812	ZNF224	76353	25kb
ENSG00000256294	19	44591334	44662027	158	17	120000	-0.02008	0.50801	ZNF225	76353	25kb
ENSG00000263002	19	44620710	44688156	120	19	120000	-0.015295	0.5061	ZNF234	76353	25kb
ENSG00000167380	19	44644226	44707534	102	13	120000	0.087854	0.465	ZNF226	76353	25kb
ENSG00000131115	19	44686700	44766421	148	17	120000	0.24565	0.40298	ZNF227	76353	25kb
ENSG00000159917	19	44707882	44834199	244	23	120000	0.27669	0.39101	ZNF235	76353	25kb
ENSG00000159915	19	44729318	44804470	133	16	120000	0.094219	0.46247	ZNF233	76353	25kb
ENSG00000062370	19	44805708	44896377	292	26	120000	0.55287	0.29018	ZNF112	76353	25kb
ENSG00000267173	19	44808848	44930774	464	43	120000	0.58864	0.27805	CTC-512J12.6	76353	25kb
ENSG00000267508	19	44861459	44930774	313	30	120000	0.29047	0.38573	ZNF285	76353	25kb
ENSG00000167383	19	44896685	44977766	372	32	120000	-0.2122	0.58402	ZNF229	76353	25kb
ENSG00000167384	19	44954854	45029576	313	28	120000	0.24015	0.40511	ZNF180	76353	25kb
ENSG00000216588	19	45091940	45165081	203	32	120000	-0.014545	0.5058	IGSF23	76353	25kb
ENSG00000073008	19	45122098	45191850	168	26	120000	-0.29994	0.61789	PVR	76353	25kb
ENSG00000186567	19	45140545	45212631	186	39	120000	-0.52996	0.70193	CEACAM19	76353	25kb
ENSG00000213892	19	45177421	45238986	236	46	120000	1.0779	0.14054	CEACAM16	76353	25kb

ENSG00000069399	19	45225962	45288301	190	26	120000	0.62179	0.26704	BCL3	76353	25kb
ENSG00000142273	19	45256126	45328891	198	24	120000	0.095321	0.46203	CBLC	76353	25kb
ENSG00000187244	19	45287328	45349673	195	32	120000	0.12266	0.45119	BCAM	76353	25kb
ENSG00000130202	19	45324432	45417485	334	41	120000	-0.87883	0.81025	PVRL2	76353	25kb
ENSG00000130204	19	45368826	45431946	197	42	120000	-1.3665	0.91411	TOMM40	76353	25kb
ENSG00000130203	19	45384011	45437650	144	30	120000	-2.1258	0.98324	APOE	76353	25kb
ENSG00000130208	19	45392504	45447606	145	34	120000	-2.059	0.98025	APOC1	76353	25kb
ENSG00000267467	19	45420495	45477820	170	22	120000	-1.0166	0.84534	APOC4	76353	25kb
ENSG00000224916	19	45420495	45477822	170	22	120000	-1.0166	0.84534	APOC4-APOC2	76353	25kb
ENSG00000234906	19	45424243	45477822	160	20	120000	-0.84906	0.80207	APOC2	76353	25kb
ENSG00000267114	19	45428301	45482264	163	19	120000	-0.7947	0.7866	CTB-129P6.11	76353	25kb
ENSG00000104853	19	45432842	45521599	250	25	120000	-0.91562	0.82007	CLPTM1	76353	25kb
ENSG00000104856	19	45479688	45566452	201	39	120000	-0.82729	0.79596	RELB	76353	25kb
ENSG00000104859	19	45517298	45599214	200	33	120000	0.68843	0.24559	CLASRP	76353	25kb
ENSG00000170684	19	45549758	45604846	152	21	120000	1.1356	0.12807	ZNF296	76353	25kb
ENSG00000142252	19	45557453	45619782	186	21	120000	1.2771	0.10078	GEMIN7	76353	25kb
ENSG00000007047	19	45557546	45833541	825	73	120000	0.18226	0.42769	MARK4	76353	25kb
ENSG00000104866	19	45569654	45676335	287	22	120000	0.92076	0.17859	PPP1R37	76353	25kb
ENSG00000179846	19	45628008	45688408	127	20	120000	0.088062	0.46491	NKPD1	76353	25kb
ENSG00000007255	19	45641186	45706495	168	32	120000	-0.13597	0.55408	TRAPPC6A	76353	25kb
ENSG00000189114	19	45657003	45710059	151	32	120000	-0.50806	0.69429	BLOC1S3	76353	25kb
ENSG00000267545	19	45658080	45730702	265	41	120000	-0.53219	0.7027	AC005779.2	76353	25kb
ENSG00000266958	19	45665646	45745191	297	43	120000	-0.24899	0.59831	AC006126.3	76353	25kb
ENSG00000130201	19	45690879	45762469	286	38	120000	0.026377	0.48948	EXOC3L2	76353	25kb
ENSG00000104879	19	45784672	45851235	206	34	120000	-1.0519	0.85359	CKM	76353	25kb
ENSG00000104892	19	45811692	45879778	211	33	120000	-1.8174	0.96542	KLC3	76353	25kb
ENSG00000268341	19	45817445	45867639	168	28	120000	-1.9399	0.97381	L47234.1	76353	25kb
ENSG00000104884	19	45828095	45899176	186	29	120000	-1.8324	0.96655	ERCC2	76353	25kb
ENSG00000104881	19	45857892	45934607	179	31	120000	-1.6817	0.95368	PPP1R13L	76353	25kb
ENSG00000117877	19	45884467	45939024	128	22	120000	-1.6177	0.94713	CD3EAP	76353	25kb
ENSG00000012061	19	45885591	46007086	328	40	120000	-2.4174	0.99218	ERCC1	76353	25kb
ENSG00000125740	19	45946253	46003437	188	32	120000	-2.0793	0.9812	FOSB	76353	25kb
ENSG00000125744	19	45963547	46025319	189	30	120000	-1.6477	0.95029	RTN2	76353	25kb
ENSG00000213889	19	45967035	46030768	195	31	120000	-1.4924	0.9322	PPM1N	76353	25kb
ENSG00000125753	19	45984837	46055241	194	31	120000	-0.42311	0.66389	VASP	76353	25kb
ENSG00000125741	19	46005685	46130470	318	44	120000	-0.37711	0.64695	OPA3	76353	25kb
ENSG00000177464	19	46068022	46130466	158	31	120000	0.0051733	0.49794	GPR4	76353	25kb
ENSG00000125746	19	46085252	46173887	279	32	120000	0.56589	0.28573	EML2	76353	25kb
ENSG00000267757	19	46119752	46171098	195	22	120000	0.61649	0.26878	C19orf83	76353	25kb
ENSG00000010310	19	46146502	46211982	211	24	120000	0.59235	0.27681	GIPR	76353	25kb
ENSG00000125743	19	46165712	46220827	123	23	120000	0.80514	0.21037	SNRPD2	76353	25kb
ENSG00000011478	19	46170741	46232247	114	23	120000	0.56655	0.28551	QPCTL	76353	25kb
ENSG00000177051	19	46188887	46259162	116	26	120000	-0.32676	0.62807	FBXO46	76353	25kb
ENSG00000237452	19	46211509	46292792	123	19	120000	-0.13631	0.55421	AC074212.3	76353	25kb
ENSG00000177045	19	46243043	46297484	82	12	120000	0.047833	0.48092	SIX5	76353	25kb
ENSG00000104936	19	46247975	46310810	115	17	120000	0.13826	0.44502	DMPK	76353	25kb
ENSG00000268434	19	46257695	46314231	116	17	120000	0.41523	0.33899	AC011530.4	76353	25kb
ENSG00000185800	19	46261205	46321060	132	20	120000	0.68229	0.24753	DMWD	76353	25kb
ENSG00000104941	19	46273968	46343577	202	21	120000	1.5689	0.058333	RSPH6A	76353	25kb
ENSG00000125755	19	46293668	46391548	322	22	120000	1.9619	0.024889	SYMPK	76353	25kb
ENSG00000170608	19	46342247	46402055	191	28	120000	2.5464	0.0054428	FOXA3	76353	25kb
ENSG00000170604	19	46361866	46414376	174	33	120000	2.7826	0.0026962	IRF2BP1	76353	25kb
ENSG00000176182	19	46368278	46430862	226	37	120000	2.2429	0.012452	MYPOP	76353	25kb
ENSG00000188425	19	46391475	46443036	189	29	120000	2.2332	0.012769	NANOS2	76353	25kb
ENSG00000104967	19	46411992	46501804	256	28	120000	1.4365	0.075425	NOVA2	76353	25kb
ENSG00000104983	19	46473339	46549576	225	34	120000	1.7458	0.040424	CCDC61	76353	25kb
ENSG00000008438	19	46497411	46551323	170	32	120000	1.8048	0.035556	PGLYRP1	76353	25kb
ENSG00000204869	19	46518006	46605376	355	28	120000	0.5031	0.30745	IGFL4	76353	25kb
ENSG00000188624	19	46598328	46652931	158	16	120000	0.64171	0.26053	IGFL3	76353	25kb
ENSG00000204866	19	46621363	46689556	144	18	120000	0.25268	0.40026	IGFL2	76353	25kb
ENSG00000188293	19	46708009	46759500	201	24	120000	-1.5356	0.93768	IGFL1	76353	25kb
ENSG00000124440	19	46775303	46871690	415	42	120000	-0.59891	0.72538	HIF3A	76353	25kb
ENSG00000011485	19	46825251	46921238	261	33	120000	-0.17986	0.57137	PPP5C	76353	25kb
ENSG00000169515	19	46888629	46941841	227	38	120000	0.52652	0.29927	CCDC8	76353	25kb
ENSG00000182013	19	46944748	46999820	290	21	120000	0.01217	0.49515	PNMAL1	76353	25kb
ENSG00000230510	19	46959053	47129457	629	66	120000	-0.1742	0.56915	PPP5D1	76353	25kb
ENSG00000204851	19	46965163	47024755	266	26	120000	0.36563	0.35732	PNMAL2	76353	25kb
ENSG00000204850	19	46972765	47024755	237	28	120000	0.4661	0.32057	AC011484.1	76353	25kb
ENSG00000268423	19	47025313	47129118	327	47	120000	-0.64559	0.74073	AC011551.3	76353	25kb
ENSG00000160014	19	47079331	47139050	155	25	120000	0.24076	0.40487	CALM3	76353	25kb
ENSG00000160013	19	47098725	47153375	162	24	120000	0.58222	0.28021	PTGIR	76353	25kb
ENSG00000167414	19	47112333	47162942	148	22	120000	0.88269	0.1887	GNG8	76353	25kb
ENSG00000197380	19	47125869	47189395	147	29	120000	1.4231	0.077348	DACT3	76353	25kb
ENSG00000105287	19	47152532	47245384	175	24	120000	0.68572	0.24645	PRKD2	76353	25kb
ENSG00000090372	19	47197764	47275251	185	25	120000	0.60562	0.27238	STRN4	76353	25kb
ENSG00000181027	19	47224303	47305245	216	30	120000	0.82864	0.20365	FKRP	76353	25kb
ENSG00000105281	19	47253140	47316851	193	30	120000	0.90434	0.18291	SLC1A5	76353	25kb
ENSG00000042753	19	47316393	47379249	122	44	120000	0.75854	0.22406	AP2S1	76353	25kb
ENSG00000160007	19	47396933	47533334	209	32	120000	0.90035	0.18397	ARHGAP35	76353	25kb
ENSG00000130751	19	47498077	47574033	178	46	120000	-0.071329	0.52843	NPAS1	76353	25kb
ENSG00000130748	19	47524165	47576888	119	30	120000	-1.0662	0.85684	TMEM160	76353	25kb
ENSG00000130749	19	47542444	47642009	150	24	120000	-1.359	0.91293	ZC3H4	76353	25kb
ENSG00000142230	19	47591531	47738886	157	29	120000	-0.22361	0.58847	SAE1	76353	25kb
ENSG00000105327	19	47699081	47761023	93	24	120000	-0.073534	0.52931	BBC3	76353	25kb
ENSG00000105321	19	47734237	47800210	187	24	120000	-0.47866	0.68391	CCDC9	76353	25kb
ENSG00000257704	19	47753142	47803979	164	17	120000	-0.42817	0.66574	PRR24	76353	25kb
ENSG00000197405	19	47768280	47850323	272	20	120000	-0.43536	0.66835	C5AR1	76353	25kb
ENSG00000134830	19	47810404	47871606	200	21	120000	0.097566	0.46114	C5AR2	76353	25kb
ENSG00000134815	19	47827538	47910961	304	22	120000	0.30231	0.38121	DHX34	76353	25kb
ENSG00000105419	19	47881381	47947780	231	33	120000	0.018324	0.49269	MEIS3	76353	25kb
ENSG00000118160	19	47906274	48000425	216	39	120000	-1.3853	0.91703	SLC8A2	76353	25kb
ENSG00000118162	19	47953401	48012525	79	18	120000	-1.7922	0.96345	KPTN	76353	25kb
ENSG00000105402	19	47965894	48043497	96	14	120000	-1.4043	0.91989	NAPA	76353	25kb
ENSG00000118156	19	47998942	48084113	121	16	120000	-0.90318	0.81679	ZNF541	76353	25kb
ENSG00000063169	19	48086453	48231533	440	42	120000	1.2111	0.11292	GLTSCR1	76353	25kb
ENSG00000024422	19	48191600	48271391	308	29	120000	0.56559	0.28584	EHD2	76353	25kb

ENSG00000105373	19	48223779	48285315	252	16	120000	0.62999	0.26435	GLTSCR2	76353	25kb
ENSG00000178980	19	48256829	48312943	218	27	120000	0.76682	0.2216	SEPW1	76353	25kb
ENSG00000178928	19	48279500	48347308	315	38	120000	-0.26139	0.6031	TPRX1	76353	25kb
ENSG00000105392	19	48297703	48371587	382	35	120000	-0.89396	0.81433	CRX	76353	25kb
ENSG00000259009	19	48312701	48389769	397	30	120000	-1.1332	0.87144	TPRX2P	76353	25kb
ENSG00000105398	19	48348723	48414654	249	28	120000	-0.52182	0.6991	SULT2A1	76353	25kb
ENSG00000188334	19	48446303	48520427	274	31	120000	2.3796	0.0086663	BSPH1	76353	25kb
ENSG00000169393	19	48472908	48553410	406	46	120000	2.774	0.0027688	ELSPBP1	76353	25kb
ENSG00000105507	19	48508210	48572310	354	37	120000	2.9956	0.0013695	CABP5	76353	25kb
ENSG00000105499	19	48526100	48639074	523	58	120000	1.1768	0.11963	PLA2G4C	76353	25kb
ENSG00000105486	19	48593702	48698860	428	27	120000	-0.31772	0.62465	LIG1	76353	25kb
ENSG00000185453	19	48648949	48725877	288	27	120000	-1.1058	0.8656	C19orf68	76353	25kb
ENSG00000178150	19	48650575	48815865	665	52	120000	0.61165	0.27038	ZNF114	76353	25kb
ENSG00000105483	19	48659027	48784203	520	40	120000	-0.4308	0.66669	CARD8	76353	25kb
ENSG00000105479	19	48774714	48850151	274	24	120000	1.495	0.067455	CCDC114	76353	25kb
ENSG00000142227	19	48799766	48858810	195	22	120000	1.4219	0.077533	EMP3	76353	25kb
ENSG00000161558	19	48810613	48892494	230	30	120000	1.0928	0.13725	TMEM143	76353	25kb
ENSG00000105467	19	48842652	48904633	152	25	120000	1.33	0.091766	SYNGR4	76353	25kb
ENSG00000105438	19	48860827	48919810	131	29	120000	1.0117	0.15585	KDELR1	76353	25kb
ENSG00000105464	19	48873132	48973188	236	43	120000	1.014	0.15529	GRIN2D	76353	25kb
ENSG00000105447	19	48924030	48985279	157	25	120000	0.49987	0.30858	GRWD1	76353	25kb
ENSG00000182324	19	48933766	48994367	144	21	120000	0.45653	0.32401	KCNJ14	76353	25kb
ENSG00000268465	19	48944094	49000688	140	20	120000	-0.0016022	0.50064	CTC-273B12.7	76353	25kb
ENSG00000105443	19	48947289	49010571	158	22	120000	-0.083199	0.53315	CYTH2	76353	25kb
ENSG00000142235	19	48963528	49041446	161	26	120000	-0.4549	0.67541	LMTK3	76353	25kb
ENSG00000088002	19	49030332	49127682	393	43	120000	-0.346	0.63533	SULT2B1	76353	25kb
ENSG00000105523	19	49079067	49143111	205	36	120000	-0.12541	0.5499	FAM83E	76353	25kb
ENSG00000177202	19	49085000	49135970	166	31	120000	-0.016082	0.50642	SPACA4	76353	25kb
ENSG00000063177	19	49093585	49147793	132	23	120000	0.37723	0.353	RPL18	76353	25kb
ENSG00000063176	19	49097548	49158974	137	23	120000	0.19885	0.42119	SPHK2	76353	25kb
ENSG00000105516	19	49108287	49165695	100	17	120000	0.33177	0.37003	DBP	76353	25kb
ENSG00000063180	19	49116199	49174569	103	20	120000	0.20117	0.42028	CA11	76353	25kb
ENSG00000142233	19	49139664	49201338	127	21	120000	0.15678	0.43771	NTN5	76353	25kb
ENSG00000176920	19	49174228	49234207	160	17	120000	0.32961	0.37085	FUT2	76353	25kb
ENSG00000176909	19	49190999	49247978	147	13	120000	0.36267	0.35843	MAMSTR	76353	25kb
ENSG00000105538	19	49198844	49268978	210	18	120000	0.46467	0.32108	RASIP1	76353	25kb
ENSG00000182264	19	49219109	49275166	159	20	120000	0.39295	0.34718	IZUMO1	76353	25kb
ENSG00000174951	19	49226268	49283647	162	23	120000	0.5527	0.29023	FUT1	76353	25kb
ENSG00000105550	19	49233816	49286587	154	25	120000	0.72384	0.23458	FGF21	76353	25kb
ENSG00000105552	19	49273319	49339286	196	33	120000	-0.13022	0.55181	BCAT2	76353	25kb
ENSG00000087076	19	49291274	49364935	222	32	120000	-0.17442	0.56923	HSD17B14	76353	25kb
ENSG00000105559	19	49315354	49396889	279	23	120000	-0.40564	0.6575	PLEKHA4	76353	25kb
ENSG00000087074	19	49350649	49404314	235	20	120000	-0.017777	0.50709	PPP1R15A	76353	25kb
ENSG00000104804	19	49359222	49426990	295	21	120000	-0.099816	0.53975	TULP2	76353	25kb
ENSG00000104805	19	49378307	49451629	284	37	120000	-0.44871	0.67318	NUCB1	76353	25kb
ENSG00000104808	19	49411939	49473226	186	38	120000	-1.2054	0.88598	DHDH	76353	25kb
ENSG00000087088	19	49433072	49490055	149	25	120000	-1.4719	0.92948	BAX	76353	25kb
ENSG00000087086	19	49443558	49495135	125	22	120000	-1.4801	0.93057	FTL	76353	25kb
ENSG00000104812	19	49446382	49521567	198	29	120000	-1.3031	0.90372	GYS1	76353	25kb
ENSG00000183207	19	49471705	49544252	185	26	120000	-1.2371	0.89198	RUVBL2	76353	25kb
ENSG00000104826	19	49494237	49545338	146	18	120000	-0.94989	0.82892	LHB	76353	25kb
ENSG00000104827	19	49501126	49552590	148	20	120000	-1.1791	0.88083	CGB	76353	25kb
ENSG00000267335	19	49501210	49565152	192	29	120000	-1.5115	0.93467	CTB-60B18.6	76353	25kb
ENSG00000104818	19	49510169	49561495	165	27	120000	-1.6246	0.94787	CGB2	76353	25kb
ENSG00000267631	19	49513831	49577006	193	35	120000	-2.0793	0.98121	CGB1	76353	25kb
ENSG00000189052	19	49522141	49573568	145	31	120000	-2.2573	0.988	CGB5	76353	25kb
ENSG00000213030	19	49525895	49577400	150	31	120000	-2.2405	0.98747	CGB8	76353	25kb
ENSG00000196337	19	49532531	49587117	162	30	120000	-2.0975	0.98202	CGB7	76353	25kb
ENSG00000225950	19	49534050	49593333	165	31	120000	-2.086	0.98151	NTF4	76353	25kb
ENSG00000104848	19	49545675	49601198	160	29	120000	-2.0605	0.98032	KCNA7	76353	25kb
ENSG00000104852	19	49563676	49636862	186	32	120000	-1.3363	0.90927	SNRNP70	76353	25kb
ENSG00000104863	19	49592581	49646717	141	28	120000	-0.82118	0.79423	LIN7B	76353	25kb
ENSG00000221916	19	49596655	49647397	138	27	120000	-0.76096	0.77666	C19orf73	76353	25kb
ENSG00000177380	19	49597646	49679283	233	41	120000	-0.86477	0.80642	PPFIA3	76353	25kb
ENSG00000130528	19	49629455	49683681	186	28	120000	-0.093218	0.53713	HRC	76353	25kb
ENSG00000130529	19	49635998	49740093	339	39	120000	0.21244	0.41588	TRPM4	76353	25kb
ENSG00000063127	19	49767895	49853482	167	19	120000	-0.51274	0.69593	SLC6A16	76353	25kb
ENSG00000104894	19	49813428	49871592	113	9	120000	-0.31518	0.62369	CD37	76353	25kb
ENSG00000074219	19	49818852	49890714	185	9	120000	-0.24423	0.59647	TEAD2	76353	25kb
ENSG00000104901	19	49840040	49903373	189	9	120000	-0.46592	0.67936	DKKL1	76353	25kb
ENSG00000161609	19	49866475	49946251	225	16	120000	-0.34968	0.63671	CCDC155	76353	25kb
ENSG00000142538	19	49900671	49951698	112	23	120000	0.9746	0.16488	PTH2	76353	25kb
ENSG00000261949	19	49902006	49957075	122	23	120000	1.519	0.064387	GFY	76353	25kb
ENSG00000104888	19	49907658	49970617	132	21	120000	2.2367	0.012652	SLC17A7	76353	25kb
ENSG00000104872	19	49924555	49981754	130	19	120000	2.957	0.0015533	PIH1D1	76353	25kb
ENSG00000161618	19	49931426	49999305	165	22	120000	3.6108	0.00015261	ALDH16A1	76353	25kb
ENSG00000269469	19	49941009	50014488	187	21	120000	3.7218	9.8886e-05	CTD-3148I10.9	76353	25kb
ENSG00000090554	19	49952464	50014488	152	18	120000	3.786	7.6551e-05	FLT3LG	76353	25kb
ENSG00000142541	19	49965811	50020565	131	15	120000	3.6723	0.00012018	RPL13A	76353	25kb
ENSG00000142534	19	49974622	50027946	131	15	120000	3.5053	0.00022801	RPS11	76353	25kb
[truncated: 11,596,989 more chars]
